# A Stem-like Patient-Derived Ovarian Cancer Model of Platinum Resistance Reveals Dissociation of Stemness and Resistance

**DOI:** 10.3390/ijms25073843

**Published:** 2024-03-29

**Authors:** Tise Suzuki, Ashlyn Conant, Yeonkyu Jung, Ryan Bax, Ashley Antonissen, Wanqiu Chen, Gary Yu, Yevgeniya J. Ioffe, Charles Wang, Juli J. Unternaehrer

**Affiliations:** 1 Division of Biochemistry, Department of Basic Sciences, Loma Linda University, Loma Linda, CA 92354, USA; tsuzuki@students.llu.edu (T.S.); abartlett@students.llu.edu (A.C.); yeonkyujung@llu.edu (Y.J.); rbax@students.llu.edu (R.B.); ashleyantonissen@llu.edu (A.A.); wchen@llu.edu (W.C.); chwang@llu.edu (C.W.); 2 Department of Biology, California State University San Bernardino, San Bernardino, CA 92407, USA; 3 Center for Genomics, Loma Linda University, Loma Linda, CA 92354, USA; 4 Department of Population and Family Health, Mailman School of Public Health, Columbia University, New York, NY 10032, USA; gy2153@columbia.edu; 5 Division of Gynecologic Oncology, Department of Gynecology and Obstetrics, Loma Linda University Medical Center, Loma Linda, CA 92354, USA; yioffe@llu.edu

**Keywords:** high-grade serous ovarian cancer, cisplatin resistance, epithelial–mesenchymal transition, cancer stemness, RNA sequencing, patient-derived model

## Abstract

To understand chemoresistance in the context of cancer stem cells (CSC), a cisplatin resistance model was developed using a high-grade serous ovarian cancer patient-derived, cisplatin-sensitive sample, PDX4. As a molecular subtype-specific stem-like cell line, PDX4 was selected for its representative features, including its histopathological and *BRCA2* mutation status, and exposed to cisplatin in vitro. In the cisplatin-resistant cells, transcriptomics were carried out, and cell morphology, protein expression, and functional status were characterized. Additionally, potential signaling pathways involved in cisplatin resistance were explored. Our findings reveal the presence of distinct molecular signatures and phenotypic changes in cisplatin-resistant PDX4 compared to their sensitive counterparts. Surprisingly, we observed that chemoresistance was not inherently linked with increased stemness. In fact, although resistant cells expressed a combination of EMT and stemness markers, functional assays revealed that they were less proliferative, migratory, and clonogenic–features indicative of an underlying complex mechanism for cell survival. Furthermore, DNA damage tolerance and cellular stress management pathways were enriched. This novel, syngeneic model provides a valuable platform for investigating the underlying mechanisms of cisplatin resistance in a clinically relevant context, contributing to the development of targeted therapies tailored to combat resistance in stem-like ovarian cancer.

## 1. Introduction

In the United States, ovarian cancer ranks fifth among the leading causes of cancer death in women, making it the most lethal among gynecologic cancers [1]. Its lethality can often be attributed to the heterogeneity of the disease from various subtypes with their respective molecular profiles, response to therapy, cell-of-origin, precursor lesions, and immune phenotype [2]. Histopathologically, the World Health Organization (WHO) has classified ovarian cancer into five major groups: high-grade serous ovarian carcinoma (HGSOC; 70%), endometrioid ovarian carcinoma (EOVC; 10%), ovarian clear cell carcinoma (OCCC; 6–10%), low-grade serous ovarian carcinoma (LGSOC; 5%), and mucinous ovarian carcinoma (MOC; 3–4%) [3]. Furthermore, molecular heterogeneity has led to the subclassification of these subtypes into several clusters with differing gene signatures. For example, thirty-two percent of HGSOC has a mesenchymal or C1 gene signature, meaning that they express fibroblastic/mesenchymal and inflammatory genes [4,5]. With such complexity, effective targeting and eradication of the disease through a more personalized therapeutic approach remains a priority.

In addition to the complex heterogeneity noted in ovarian cancer, high mortality in advanced disease is observed due to a lack of effective screening for early detection [6]. Specifically, in HGSOC, 80% of cases are diagnosed at an advanced stage (FIGO III-IV) [7]. The difficulty in diagnosis lies in the asymptomatic nature of early disease and the poor sensitivity and specificity of available markers, such as CA125 and HE-4 [6]. Once diagnosed, treatment options include cytoreductive surgery and a combination of platinum- and taxane-based therapies, with poly (ADP-ribose) polymerase inhibitors (PARPi) as maintenance therapy with particular efficacy for *BRCA1/2* mutant patients [8]. Although initial response is favorable, most patients experience relapse, and recurrent disease is often resistant to therapy [9,10].

To persist through initial treatment, cancer cells adopt multiple mechanisms of cisplatin resistance that have been identified and extensively studied. ABC transporters, such as the multidrug resistance protein 1, confer resistance by effluxing drugs from cancer cells; expression of this and related proteins are a common cause of resistance [11]. DNA damage repair pathways are another important way for cells to achieve chemoresistance. For example, patients with *BRCA* and other homologous recombination mutations can be treated successfully with PARP inhibitors and respond better to platinum agents; however, *BRCA* reversion mutations can render the patients resistant to both drug classes [10]. Autophagy induction in cisplatin-resistant cells provides a survival response by activating ERK signaling, protecting against cisplatin-induced cell death [12,13]. Other signaling pathways that enhance survival, including those associated with apoptosis and cell cycle regulation, can contribute to cisplatin resistance [14]. Defects affecting metabolic pathways and oxidative stress are also mediators of resistance by allowing cells to deal with toxicity caused by cisplatin [11]. Particularly in ovarian cancer, epithelial–mesenchymal transition (EMT), a dynamic and reversible biological process characterized by molecular and functional changes occurring in epithelial cells that result in a decrease in cell adhesion and an increase in motility, has been linked to therapy resistance [15,16,17,18]. In this context, as cells transition to a more mesenchymal phenotype, chemoresistance has been correlated with the generation of cancer stem cells (CSC), which are a small subpopulation within tumors that are defined by their capacity for self-renewal and differentiation [15,19,20,21]. 

Considering the heterogeneity of ovarian cancer, few in vitro models have been adequately characterized and derived for the study of complex mechanisms of chemoresistance [22,23]. Models like A2780/A2780-cis [24], SKOV3/SKOV3-CDDP [25], HeyA8/HeyA8-MDR [26], and IGROV-1/IGROV1-pt [27], among others, have very limited applicability and lack HGSOC subtype specificity. For instance, although both A2780 and SKOV3 have been classified as having a Proliferative/C5/Stem-A and Mesenchymal/C1 molecular subtype, respectively, several studies have highlighted their poor representation of HGSOC and possible non-ovarian origin [5,28,29]. Still, these cell lines account for most of the publications available for HGSOC research [28]. Likewise, HeyA8 and IGROV-1 have been classified as OCCC and LGSOC, respectively, with IGROV-1 having fewer copy number alternations and a heavier mutational burden compared to HGSOC tumor samples [30,31]. One of the only models that would be applicable to the study of chemoresistance in HGSOC would be that of PEO1 and its resistant counterpart PEO4 [32], both of which are classified as having an Epithelial-A/C3/Differentiated molecular subtype [5] that only contributes to 7% of all HGSOC cases [4]. Therefore, additional models that represent other molecular subtypes of HGSOC are needed.

Previously, our lab has focused on characterizing a variety of patient-derived high-grade serous ovarian carcinoma (HGSOC) samples [33,34]. As the most aggressive and common subtype of ovarian cancer, the HGSOC samples indicated a large distribution of growth rate, morphology, gene and protein expression, and response to commonly used therapies, like cisplatin, a platinum-based agent that forms adducts with DNA, and olaparib, a PARPi [33]. To better understand the development and mechanisms of resistance specific to stem-like cancer cells, we sought to create a syngeneic model of cisplatin resistance from one of the cisplatin-sensitive patient-derived samples that was previously characterized, PDX4. PDX4 was classified histopathologically as a HGSOC subtype sample and as a Proliferative/C5/Stem-A molecular subtype, which does not currently have adequate representative in vitro models of cisplatin resistance. Furthermore, PDX4 was determined to have a *BRCA2* mutation, which is the case for 25% of HGSOC patients [35]. Thus, with a defined molecular and mutational profile, PDX4 provides a rare model that aids in integrating both clinical and experimental data for aggressive disease. Therefore, our goal was to establish a methodology for developing chemoresistance in vitro that parallel clinically relevant treatment concentrations. In this study, we characterize the cisplatin-resistant PDX4 and its potential mechanisms for thwarting cell death.

## 2. Results

### 2.1. Generation of Cisplati-Resistant Cell Line

**Figure 1:**
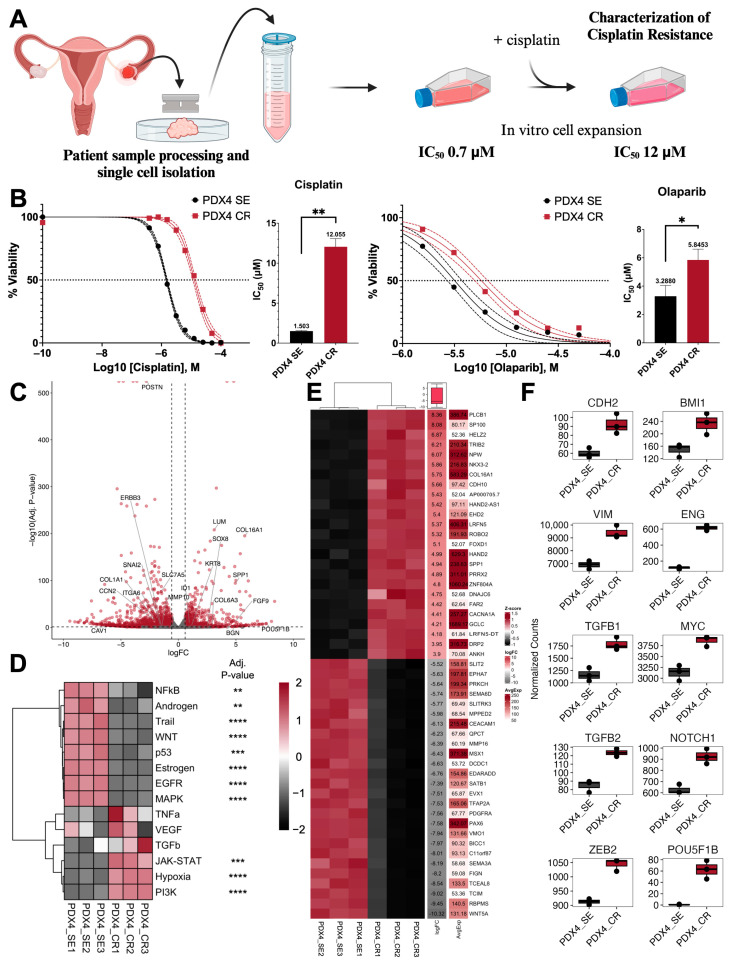
Cisplatin and olaparib-resistant patient-derived cell lines express markers of epithelial–mesenchymal transition (EMT) and stemness. (**A**) PDX4 was obtained from a patient diagnosed with stage IVB high-grade serous ovarian cancer (HGSOC). After sample dissociation and processing, cells were grown in vitro in increasing concentrations of cisplatin; (**B**) while growing at 10 µM of cisplatin, PDX4 CR (cisplatin-resistant) had cisplatin and olaparib IC50 of 12 µM (*n* = 4) and 5.8 µM (*n* = 3), respectively, as measured by thiazolyl blue tetrazolium bromide (methylthiazolyldiphenyl-tetrazolium bromide; MTT) viability assay; (**C**) RNA sequencing revealed a total of 7506 differentially expressed genes (DEGs), with 3890 genes downregulated and 3616 genes upregulated in PDX4 CR (*n* = 3) relative to PDX4 SE (cisplatin-sensitive; *n* = 3). Each dot represents a single gene. The highlighted genes are associated with cisplatin resistance, cancer stemness, and/or EMT. Adjusted *p*-value < 0.1 and absolute log2(FC) > 0.58; (**D**) PROGENy pathway signatures identified in cisplatin sensitive and resistant samples; (**E**) clustering heatmap displaying the top 50 differentially expressed genes (DEG) within the RNA-seq data. The logFC column indicates the expression relative to the cisplatin-sensitive sample, and the “AvgExp” column displays the average normalized count values across all samples. Above the logFC column, the box and whiskers plot summarize the spread of the logFC expression values; (**F**) gene expression profiles of putative EMT (*CDH2*, *VIM*, *TGFB1*, *TGFB2*, and *ZEB2*) and stemness genes (*BMI1*, *ENG*, *MYC*, *NOTCH1*, and *POU5F1B*) in the cisplatin-sensitive and cisplatin-resistant samples as identified using RNA-seq. * *p*  <  0.05, ** *p*  <  0.01, *** *p*  <  0.001, **** *p*  <  0.0001. Created with Biorender.com, accessed on 12 October 2023.

PDX4, a high-grade serous ovarian cancer (HGSOC) sample, was obtained as previously described from a chemotherapy naïve patient [33]. In vitro, the cells were described as epithelial-like, with a low migration rate but high spheroid forming capacity compared to additional patient-derived samples; this and other characteristics (including tumorigenicity) made this a sample with high stemness attributes [33]. PDX4 was also determined to be *BRCA2* mutant and homologous recombination deficient via clinical testing [33]. Furthermore, in vivo, PDX4 demonstrated high tumorigenicity when orthotopically injected into the ovarian bursae of nude mice [33]. To further characterize this cell line, cisplatin-resistant PDX4 (referred to as PDX4 CR throughout the remainder of the study) was generated through continual and repeated exposure to increasing concentrations of cisplatin (Figure 1A). A final cisplatin concentration of 8.5–10 µM was determined to be the maximal clinically relevant concentration that is observed in the plasma of patients; therefore, it was designated as the endpoint for further characterization [36,37,38]. Compared to vehicle-treated control cells (cisplatin-sensitive PDX4; PDX4 SE), PDX4 CR had an 8- and 1.8-fold increase in cisplatin and olaparib IC_50_, respectively (Figure 1B). At the transcript level, RNA sequencing revealed over 7500 genes that were differentially expressed, with 3890 genes that were downregulated and 3616 genes that were upregulated in the chemoresistant cell line (adjusted *p*-value < 0.1; Figure 1C and Table S1). PROGENy algorithms were then utilized to identify signaling pathways that could be contributing to the differences observed between the two groups (Figure 1D). PDX4 CR displayed a significant activation of genes within the hypoxia, PI3K, and JAK-STAT signaling pathways, while NF-κB, androgen, TRAIL, WNT, p53, estrogen, EGFR, and MAPK pathways were significantly downregulated. Database searches revealed that out of the top 50 differentially expressed genes, 2 genes have previously been associated with cisplatin resistance (*XAF1* and *TRIB2*) [39], 7 with EMT (*CCAT2*, *EVPL*, *SATB1*, *PDGFRA*, *BICC1*, *SFRP1*, and *WNT5A*) [40], and 23 with stemness [41] (Figure 1E and Table S2). Furthermore, markers and promoters of EMT, *CDH2*, *VIM*, *TGFB1*, *TGFB2*, and *ZEB2* were upregulated in the resistant sample, while genes associated with pluripotency and stemness, *BMI1*, *ENG*, *MYC*, *NOTCH1*, and *POU5F1B*, were upregulated in PDX4 CR (Figure 1F).

### 2.2. Growth and Proliferation Characteristics after the Acquisition of Cisplatin Resistance

**Figure 2:**
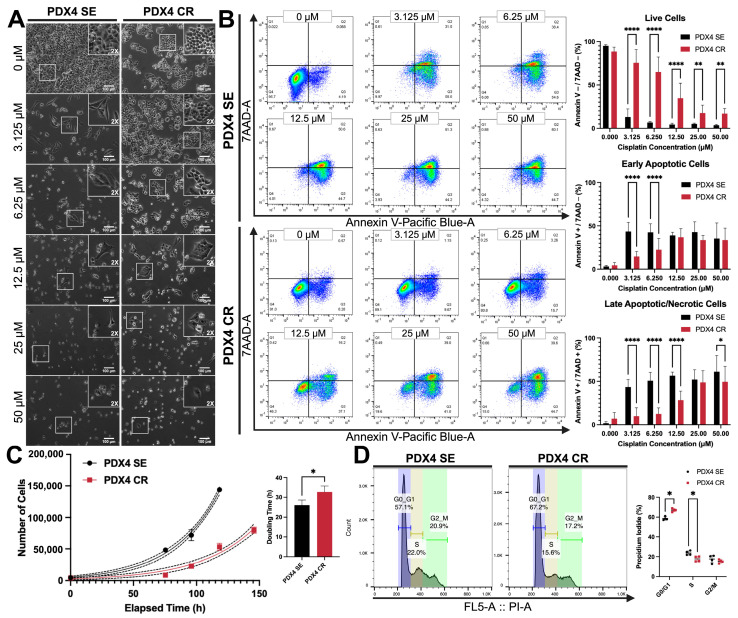
Growth characteristics of PDX4 CR after cisplatin treatment. (**A**) After three days of cisplatin treatment (0–50 µM), PDX4 SE and CR were imaged at 10× magnification. Cell morphologies are highlighted in each panel with an inlaid panel (white box) of 2× magnification (total magnification 200×). Scale bar: 100 µm. (**B**) Representative FACS profiles of PDX4 SE and CR cells stained with Annexin V-Pacific Blue (PB) and 7-aminoactinomycin D (7AAD) after 72 h of cisplatin treatment (0–50 µM). Quantification of live Annexin V^−^/7AAD^−^ (**top**), early apoptotic Annexin V^+^/7AAD^−^ (**middle**), and late apoptotic/necrotic Annexin V^+^/7AAD^+^ and Annexin V^−^/7AAD^+^ (**bottom**) cells. Percent positive cells were gated from single, intact cells. (**C**) Analysis of cell proliferation using MTT assay. PDX4 SE and CR were seeded at 1000 cells per well on day zero and allowed to grow for 96–144 h (4–6 days). Doubling time was determined from the exponential (Malthusian) growth curve (*n* = 3). Data are presented as mean ± SD. Curve fitting and doubling time were calculated using GraphPad Prism software. Black bars in (**B**,**C**): PDX4 SE; red bars: PDX4 CR. (**D**) Cell cycle analysis using flow cytometry (**left**). Untreated PDX4 SE and CR cells were stained with FxCycle™ propidium iodide/RNase staining solution. Percent distribution of cells through the different cell cycle stages (**right**). All flow cytometry data were collected with a MACSQuant Analyzer 10 and analyzed with FlowJo software. * *p*  <  0.05, ** *p*  <  0.01, **** *p*  <  0.0001. Created with Biorender.com, accessed on 31 July 2023.

To verify cell viability, PDX4 SE and CR were treated with cisplatin for 72 h, imaged, labeled with Annexin V and 7AAD, and quantified with flow cytometry (Figure 2A,B). After cisplatin treatment, PDX4 SE displayed mild morphological changes under phase-contrast micrographs, acquiring a more elongated shape (Figure 2A; left). In contrast, PDX4 CR maintained its epithelial-like cobblestone pattern even at high concentrations of cisplatin (Figure 2A; right). Furthermore, PDX4 SE greatly decreased cell confluency with the first concentration of cisplatin (3.125 μM), while PDX4 CR gradually decreased in confluency (Figure 2A). More specifically, at 3.125 μM cisplatin, PDX4 SE had around 2–4% live cells (Annexin V^−^/7AAD^−^; Figure 2B, top), 30–59% cells undergoing early apoptosis (Annexin V^+^/7AAD^−^; Figure 2B, middle), and 32–54% cells in late apoptosis and necrosis (Annexin V^+^/7AAD^+^; Figure 2B bottom). In comparison, the percentage of live cells for PDX4 CR only decreased considerably from 6.25 μM (42–84%) to 12.5 μM (12–51%) of cisplatin (Figure 2B). At 12.5 μM and 50 μM of cisplatin, PDX4 CR had the highest percentage of early apoptotic (51%) and late apoptotic/necrotic cells (75%), respectively (Figure 2B).

As cisplatin-sensitive and resistant cells were grown in parallel in vitro, we observed that PDX4 CR seemed to proliferate more slowly. In fact, quantification of doubling time revealed that PDX4 SE had an average doubling rate of 26 h, while PDX4 CR doubled every 33 h (Figure 2C). Additionally, both cell lines were stained with propidium iodide (PI) for cell cycle analysis through flow cytometry (Figure 2D). As expected, PDX4 CR had a higher percentage (67%) of cells in the G0/G1 phase than PDX4 SE (59%; Figure 2D), indicating that the cells were likely less proliferative due to a portion of them being in cell cycle arrest. At the S phase, a slightly higher proportion of sensitive cells (24%) than resistant cells (18%) was found.

### 2.3. Defining Epithelial–Mesenchymal Transition Status

The previous literature indicates that platinum-based therapies may induce the epithelial–mesenchymal transition (EMT) process [42,43,44,45,46]. Since the RNA-seq data highlighted pathways activated in EMT (Figure 1D) along with the differential expression of several EMT markers (Figure 1F), we set out to validate not only the EMT-associated phenotypical and functional changes observed in PDX4 SE and CR but also the mechanism of cisplatin resistance.

**Figure 3:**
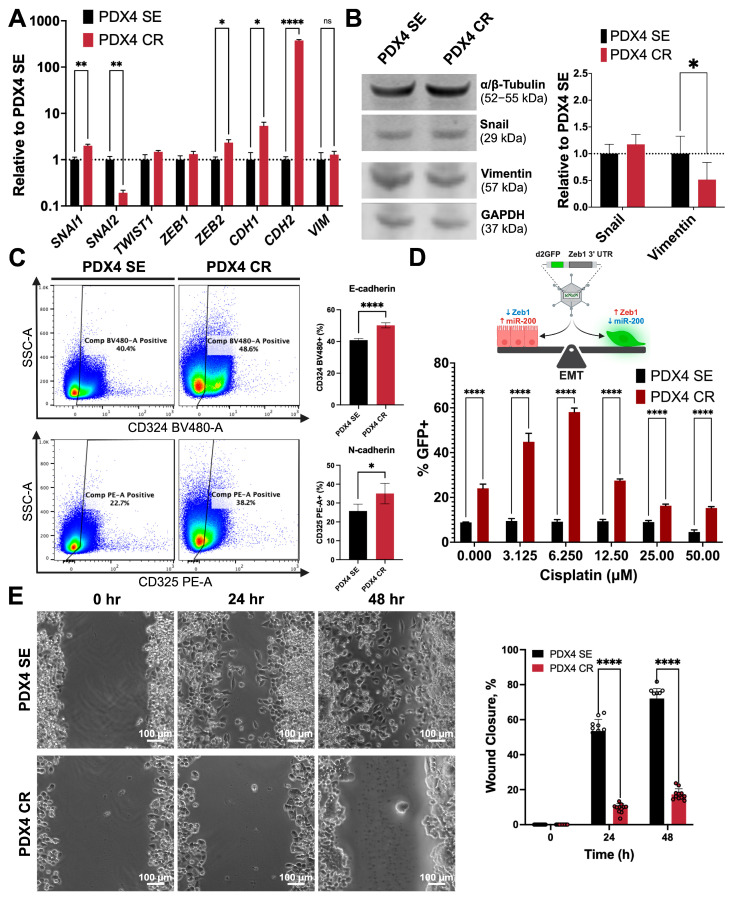
PDX4 SE and CR cells exhibit hybrid/partial EMT phenotypes. (**A**) RT-qPCR validation of EMT transcription factors and markers. Expression levels are relative to the sensitive cell line. Results are presented as the means ± SD. (**B**) Protein quantification of Snail and Vimentin through Western blot, normalized to either α/β-tubulin or GAPDH. Expression levels are relative to PDX4 SE. (**C**) Representative pseudocolor flow cytometry dot plots (**left**) for the expression of E-cadherin (CD324) and N-cadherin (CD325). Colors on dot plot represent cell density (cool colors: low density; warm colors: high density). Quantification of single and double positive populations (**right**). Percent positive cells were obtained from single, intact, live cells. (**D**) The Zeb1 3′UTR GFP reporter (Toneff et al., 2016 [47]) detects the presence of miR-200 within cells. In the presence of *miR-200*, its binding to the Zeb1 3′UTR (arrow pointing to left) silences GFP expression; in the absence of *miR-200* GFP is expressed (arrow pointing to right). PDX4 SE and CR cells were transduced with the lentiviral reporter system and treated with cisplatin (0–50 µM) for 72 h. Quantification of GFP expression was measured through flow cytometry. GFP-positive populations were obtained from single, intact cells that were Annexin V^−^/7AAD^−^. All flow cytometry data were collected with a MACSQuant Analyzer 10 and analyzed with FlowJo software. (**E**) Migration capacity was determined through 24- and 48-h scratch wound healing assays (*n* = 12). Black bars: PDX4 SE; red bars: PDX4 CR. Results are presented as the means ± SD. Statistical significance was determined by two-way ANOVA with Tukey’s test for multiple comparison correction. *p*-values: *p* ≤ 0.05 (*); *p* ≤ 0.01 (**); *p* ≤ 0.0001 (****); ns, not significant. Created with Biorender.com, accessed on 1 August 2023.

We used RT-qPCR to validate the mRNA-seq results. In mRNA expression, PDX4 CR had a significant increase in the expression of Snail (*SNAI1*), *ZEB2*, E-cadherin (*CDH1*), and N-cadherin (*CDH2*), while PDX4 SE had a higher expression of Slug (*SNAI2*) (Figure 3A). Interestingly, at the protein level, Snail expression only slightly increased, and vimentin decreased significantly in PDX4 CR (Figure 3B and Figures S1–S3). Additionally, flow cytometry identification of E-cadherin (CD324) and N-cadherin (CD325) revealed that PDX4 CR had more CD324^+^ (50%) and CD325^+^ (35%) cells than the cisplatin-sensitive cells (41% and 26%, respectively; Figure 3C).

Since EMT is a transitional process, we aimed to describe the effect of different concentrations of cisplatin on the expression of EMT markers on a per-cell basis. For this purpose, we transduced PDX4 SE and CR with the *ZEB1* 3′UTR destabilized GFP (dGFP) reporter [47] that senses EMT changes as a function of *miR-200* levels. More specifically, as Zeb1 levels increase with EMT, *miR-200* repression allows dGFP expression, detected through flow cytometry (Figure 3D). For all concentrations tested, GFP-positive populations were greater in the chemoresistant cells, with the highest enrichment achieved at 6.25 μM.

Functionally, migratory capacity was measured through a wound-healing assay. Because gene expression changes are consistent with a more mesenchymal phenotype overall, it was expected that PDX4 CR would be more migratory. However, after scratching the monolayer of cells, PDX4 SE displayed a higher efficiency in gap closure compared to PDX4 CR (Figure 3E). By 24 h, PDX4 SE cells at the leading edge spread towards the center of the gap, while PDX4 CR cells appeared stationary, with their leading edge mainly intact (Figure 3E).

### 2.4. Stemness and Self-Renewal Characteristics

Stemness, within the context of cancer, refers to the ability of cancer cells to self-renew and differentiate into heterogeneous populations within a given tumor. In many cancer types, activation of EMT has often been associated with the generation of stemness characteristics [19,20,48]. Given that both the cisplatin-sensitive and resistant cells expressed EMT markers, we sought to characterize their stemness potential.

**Figure 4:**
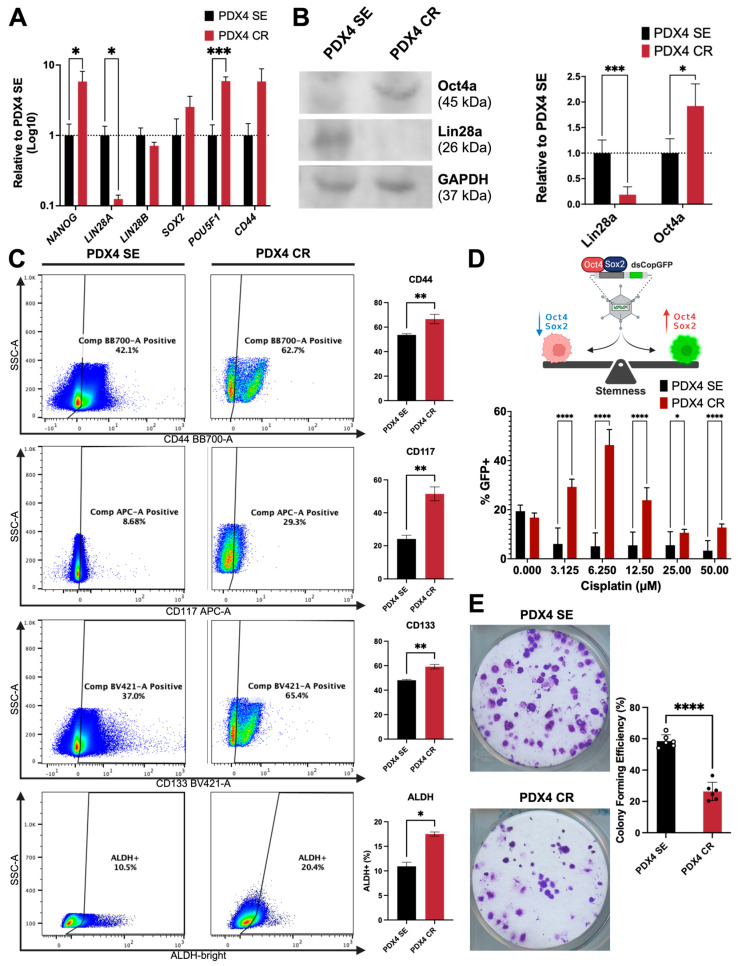
Cisplatin-resistant cells express more stemness markers but are less clonogenic. (**A**) RT-qPCR validation of pluripotency and stemness markers. Expression levels are relative to the sensitive cell line. Results are presented as the means ± SD. (**B**) Protein quantification of Lin28a and Oct4a through Western blot, normalized to GAPDH, relative to PDX4 SE. (**C**) Representative flow cytometry pseudocolor dot plots (**left**) for the expression of cell surface markers CD44 (**first row**), CD117 (c-Kit; **second row**), CD133 (PROM1; **third row**), and ALDH (**last row**). Comparison of single positive populations between PDX4 SE and CR (**right**). Percent positive cells were obtained from single, intact cells. ALDH gates were drawn based on DEAB-treated cells. Colors on dot plot represent cell density (cool colors: low density; warm colors: high density). (**D**) The SORE6 GFP reporter (Tang et al., 2015 [51]) detects the presence of SOX2 and Oct4 within cells. In the presence of OCT4 and SOX2, they bind to SORE6 elements (arrow pointing to right) and activate GFP expression; in their absence GFP is not expressed (arrow pointing to left). PDX4 SE and CR cells were transduced with the reporter system and treated with cisplatin in increasing concentrations (0–50 µM) for 72 h. Quantification of GFP expression was measured through flow cytometry. GFP-positive populations were obtained from single, intact cells that were Annexin V^−^/7AAD^−^. All flow cytometry data were collected with a MACSQuant Analyzer 10 and analyzed with FlowJo software. (**E**) Representative images of PDX4 SE and CR colonies that were grown for 10–11 days in a 6-well plate (well diameter = 35-mm; **left**). Images were acquired with an HP scanner. Colony-forming efficiency was calculated relative to the number of plated cells (*n* = 6; **right**). Results are presented as the means ± SD. Statistical significance was determined using the unpaired *t*-test. *p*-values: *p* ≤ 0.05 (*); *p* ≤ 0.01 (**); *p* ≤ 0.001 (***); *p* ≤ 0.0001 (****). Created with Biorender.com, accessed on 19 September 2023.

PDX4 SE had a higher expression of *LIN28A*/Lin28a at both the mRNA and protein level than its resistant pair, while PDX4 CR expressed more *NANOG* and Oct4 at mRNA and protein level, respectively, relative to PDX4 SE (Figure 4A,B and Figure S2). Corroborating the LIN28 expression patterns, we observed an overall enrichment in the expression of *let-7* family members in the resistant cells, microRNAs known to promote a differentiated state, acting in a negative feedback loop in the regulation of LIN28 expression (Figure S4 and Table S3). Furthermore, flow cytometry was used to quantify cells positive for CD44, CD117, CD133, and aldehyde dehydrogenase (ALDH) activity, all markers of stemness that have been validated in ovarian cancer [49,50]. All tested markers were expressed abundantly in both sensitive and resistant cells (Figure 4C), but PDX4 CR had a significantly higher proportion of all markers. Specifically, more than half of the live population of cells were CD44^+^ in both PDX4 SE (54%) and PDX4 CR (67%; Figure 4C). Likewise, CD117^+^, CD133^+^, and ALDH^+^ populations were 2.5-, 1.2-, and 1.6-fold greater, respectively, in the resistant cells compared to their sensitive counterpart (Figure 4C).

Taking into consideration that stem cells are a rare population, we aimed to describe the effect of different concentrations of cisplatin on the expression of pluripotency markers on a per-cell basis. Thus, we transduced PDX4 cells with the SORE6-GFP reporter [51], with GFP expression under the control of the SOX2/Oct4 response element (SORE). Notably, pluripotent cells would express more SOX2 and Oct4, resulting in an increase in the translation of GFP that can be detected through flow cytometry (Figure 4D). For all concentrations tested (including in the untreated cells), GFP expression was greater in the chemoresistant cells, with the highest enrichment achieved at 6.25 μM (Figure 4D).

To test self-renewal capacity, sensitive and resistant PDX4 cells were subjected to colony-forming assays. As a result, PDX4 SE demonstrated higher efficiency at colony formation, with an average of almost 60% of cells plated being able to form colonies. In comparison, around 25% of PDX4 CR cells demonstrated colony-forming ability (Figure 4E). Together, these results indicate that, while PDX4 SE and CR had different stem-like expression patterns, PDX4 SE had higher self-renewal capacity.

## 3. Discussion

Many mechanisms of cisplatin resistance have been described in the literature [10]. In accordance with previous findings, we hypothesized that resistance would correlate with a more mesenchymal-like status and increased stemness [42,43,44,45,46]. In agreement with the hypothesis, cisplatin-resistant cells retained expression of most EMT and stemness markers, increased frequency of stem cell marker-expressing cells (including CD44, CD117, CD133, and ALDH1), and increased expression of SORE6 and Zeb-1-3′UTR even at low doses of cisplatin. Furthermore, RNA-seq results revealed that PDX4 CR had an enrichment in the expression of genes associated with the hypoxia, autophagy, PI3K/Akt, and JAK-STAT pathways, all of which have been associated with EMT to different extents [52,53,54,55,56]. However, we observed that functional findings were contradictory: resistant cells were less migratory and clonogenic, consistent with their epithelial morphology and reduced proliferation. To support our current hypothesis, PDX4 SE and CR were classified as Stem-A, with the sensitive cell line showing greater enrichment of Stem-A signature than the resistant cells (Figure S5). The Stem-A ovarian cancer subtype has been described as corresponding to the Tothill et al. C5 and the TCGA proliferative subtypes [4]. In terms of gene expression profile, this subtype is known for demonstrating an enrichment in the expression of genes associated with development, proliferation, and stemness [4]. Moreover, its aggressiveness in ovarian cancer has been attributed to the activation of non-canonical Wnt/PCP pathway through *FZD7* [57]. Gene set enrichment analysis confirmed that the non-canonical Wnt pathway was activated in PDX4 SE; however, PDX4 CR did not exhibit such patterns (Figure 1 and Figure S6 and Tables S4 and S5). In fact, the cisplatin-resistant cells demonstrated enrichment in pathways associated with immune signaling, protein localization, and mitochondrial function (Table S5). These results indicated that a more complex mechanism of cisplatin resistance was adopted via PDX4 CR: stem-like, persistent HGSOC cells dissociate stemness features from resistance mechanisms to survive cytotoxic stress. Our initial findings led us to a new hypothesis that stem-like resistant cells undergo reprogramming to obtain a distinctive hybrid EMT phenotype characterized via cell cycle regulation and DNA damage repair. In fact, the shift away from the Stem-A subtype could potentially be explained by PDX4 CR cells utilizing the enriched signaling cascades to hijack pathways aiding DNA damage tolerance. Further studies are underway to elucidate whether such mechanisms contribute to resistance and/or compensate for cytotoxic stress. Additionally, our current focus has been to target these pathways in a Stem-A/C5/Proliferative subtype-specific manner to uncover potential personalized therapeutic strategies.

Another candidate mechanism of cisplatin resistance, autophagy, could play a role in the PDX4-CR cells. Cisplatin-induced autophagy contributes to cisplatin resistance via ERK activation in ovarian cancer cell lines and protects against cisplatin-induced cell death [12,13]. A correlation between autophagy score and prognosis has been reported in ovarian cancer [58]. Because ovarian cancer stem cells have been shown to have increased autophagic flux, this pathway is an important potential therapeutic target in this disease [59]. Our RNA-seq results showing activation of autophagy-associated pathways in CR cells (Table S6) provide a rationale for exploring this pathway in future studies to reverse cisplatin resistance. 

In addition to analyzing pathways, we initially assessed multiple cisplatin and copper transporters through qPCR and flow cytometry; however, no significant difference was detected between the cisplatin-sensitive and resistant cells (Figure S7). Furthermore, while testing DNA damage repair capabilities, Western blot quantification of PARP1 indicated that PDX4 CR had lower expression of the protein (Figure S2C). Considering that PDX4 samples are *BRCA2* mutants, treatment with olaparib should have induced synthetic lethality; however, the resistant cells had increased resistance to the PARPi, suggesting the potential for higher DNA damage repair proficiency. A decrease in PARP1 expression, or function, has been associated with acquiring PARPi resistance, potentially via preventing PARP trap lesions [60,61]. Our data agree with findings elsewhere that PARPi-resistant cells have lower PARP1 expression [60,61]. PARP1 has mainly been studied for its role in DNA damage repair, but this protein has other functions that may explain this apparent contradiction between PARP1 level and PARPi resistance. PARP1 increases Snail activity, and thus EMT, via post-transcriptional stabilization of Snail protein and transcriptional regulation [62,63,64]. Thus, PARP1 reduction would correlate with (or play a causal role in) a more epithelial phenotype, as observed in the PDX4-CR cells. Another notable PARP1 role is in the establishment of stemness: PARP1 is recruited to stem cell-related loci and establishes epigenetic marks consistent with an activated chromatin state (allowing expression of pluripotency genes), which increases the expression of stemness genes [65,66]. Similar effects have been seen in cancer cells [67,68]. We see some evidence of reduced stemness in the PDX4-CR cells, which the reduction of PARP1 could explain. These diverse activities of PARP proteins illustrate how levels of protein and resistance to PARPi may not be directly related in all cases.

As a response to cisplatin treatment (presumably, the DNA damage caused by that drug), we observed that cells proliferated at a slower pace (Figure 2C), remaining in the earlier stages of the cell cycle, G0/G1 (Figure 2D). Previously, our characterization of HGSOC patient-derived sample heterogeneity highlighted similar findings in the cancer stemness context; samples with the highest cisplatin resistance were less proliferative [33]. The reduced proliferation rate and reprogramming associated with stemness in resistant cells could help explain why cisplatin-resistant cells appear less migratory and clonogenic. Future studies could focus on determining whether cisplatin withdrawal would restore or improve these cellular functions. Alternatively, the accumulation of cells in G0 could indicate that the resistant cells are transitioning into a state of dormancy while facing genomic instability or biological stress. This interpretation would agree with the assessment that PDX4 CR cells may, in response to the presence of DNA fragments, be activating the cGAS-STING signaling pathway, which has been associated with cell cycle arrest [69,70,71,72,73]. Studies relating to this and associated pathways are ongoing.

It is important to recognize that the PDX4 cisplatin resistance model has limitations. Although the cells were carefully treated with cisplatin concentrations that mimicked clinical patient data, this model was designed to study molecular mechanisms of resistance in vitro. To remedy the shortcomings of in vitro culture, which display limitations in 3D growth and microenvironmental context, future studies would include the development of animal models/xenografts that would provide such an environment. Furthermore, with the observed complexity of the mechanism of resistance for PDX4, it is possible that our findings are not universal to all ovarian cancer molecular subtypes. In other words, the patterns observed might be specific only to the Proliferative/C5/Stem-A ovarian cancer subtype. If that is the case, our goal is to continue characterizing patient-derived samples and developing subtype-specific resistant models.

Taking together the complex nature of these results and the mechanism of action of cisplatin, we reason that PDX4 CR cells maintain and lose some EMT- and stem-like characteristics to undergo functional changes towards dormancy to promote DNA damage repair and, thereby, survival. With this study, we have developed a novel syngeneic model of cisplatin resistance to study the effect of treatment on stem-like ovarian cancer cells. To our knowledge, this model is the first to demonstrate the aforementioned mechanism of cisplatin resistance within ovarian cancer. Additionally, with this model, there is the potential to better understand the interface between DNA damage and immune response and determine if this pathway is targetable for enhancing patient response to immunotherapies.

## 4. Materials and Methods

### 4.1. Cell Culture

The collection and use of the cells in this study were approved by the Loma Linda University (LLU) Institutional Review Board (IRB, 58328). PDX4, a chemotherapy naïve, cisplatin-sensitive HGSOC sample, was collected, preserved, and processed as previously described [33]. With informed consent, tumor tissue was collected by the Loma Linda University Cancer Center Biospecimen Laboratory (LLUCCBL), stripped of patient identifiers, and immediately transported to the laboratory for processing. The same tumor samples were used to clinically diagnose HGSOC. To obtain a single cell suspension, the tissue section was washed in 1× phosphate-buffered saline (PBS; 138 mM NaCl, 2.7 mM NaCl, 8.1 mM Na_2_HPO_4_, 1.2 mM KH_2_PO_4_ in ddH_2_O) containing 20 μg/mL gentamicin sulfate (G020-1GM; Caisson Labs, Smithfieeld, UT, USA) minced with a razor blade (SKU 71964; Electron Microscopy Sciences, Hatfield, PA, USA), and passed through a 100 μm cell strainer (15-1100; Biologix, Camarillo, CA, USA) using a cell pestle (229480; CELLTREAT Scientific Products, Pepperell, MA, USA). Erythrocytes were removed with Cytiva Ficoll-Paque^TM^ PLUS media (17144002; Cytiva, Marlborough, MA, USA) centrifugation. Cells were cultured long-term in a 3:1 mixture of Hyclone^TM^ Ham’s Nutrient Mixture F12 with L-glutamine (SH30026.01; Cytiva) and Dulbecco’s Modified Eagle’s Medium with high glucose and L-glutamine (DMEM; 25-501; Genesee Scientific, El Cajon, CA, USA), supplemented with 5% FBS (Omega Scientific, Tarzana, CA, USA), 0.4 μg/mL hydrocortisone (H0888-1G; Sigma-Aldrich, St. Louis, MO, USA), 5 μg/mL insulin (91077C-100MG; Sigma-Aldrich), 2 μg/mL isoprenaline hydrochloride (I5627-5G; Sigma-Aldrich), 24 μg/mL adenine (A8626; Sigma-Aldrich), 100 U penicillin, and 100 μg/mL streptomycin (25-512; Genesee Scientific). PDX4 CR and SE were cultured to approximately 80 and 50 passages, respectively.

Cells were maintained at 37 °C with 5% CO_2_ and tested for mycoplasma contamination routinely using the PlasmoTestTM Mycoplasma Detection Kit (rep-pt1; InvivoGen, San Diego, CA, USA). The purity and identity of PDX4 were confirmed before and after the generation of cisplatin resistance utilizing Short Tandem Repeat validation (Laragen Inc., Culver City, CA, USA). 

### 4.2. Generation of Cisplatin Resistant Cell Line

PDX4 cells were plated at 25% confluence in three 25 cm^2^ tissue culture flasks (12-556-009; Thermo Fisher Scientific, Waltham, WA, USA). After 24 h, one flask received vehicle treatment (PBS), and a second flask received 0.7 μM cis-Diammineplatinum(II) Dichloride (cisplatin; IC-50 determined previously [33]; D3371-100MG; TCI Chemicals, Portland, OR, USA), and the last flask was used as a plating, health assessment control. Once cells achieved 70–80% confluence, they were passaged three more times within the same concentration of cisplatin before treatment concentration was increased by 0.05, 0.1, and then finally 0.5 μM increments until they were able to withstand and grow healthily at 10 μM cisplatin, which was determined to be relevant resistance criteria as the maximal plasma concentration observed in patients [36,37,38]. Increasing cisplatin resistance was periodically confirmed using a cell viability assay, detailed below.

One millimolar cisplatin aliquots were prepared in large batches and frozen at −70 °C to prevent batch variation and degradation through multiple freeze-thaw cycles.

### 4.3. Cell Viability Assays

Cell viability was measured using thiazolyl blue tetrazolium bromide (MTT; 00697; Chem-Impex, Wood Dale, IL, USA) assays. One thousand PDX4 cells were seeded in triplicates in flat-bottomed 96-well plates (229195; CELLTREAT Scientific Products) and allowed to adhere for 24 h prior to the addition of cisplatin or Olaparib (AZD2281; S1060; Selleck Chemicals, Houston, TX, USA). Throughout the experiments, a growth medium without penicillin–streptomycin was used. Cells were then treated with vehicle (PBS for cisplatin and DMSO for olaparib) or increasing concentrations of cisplatin or olaparib (0–100 μM) followed by an incubation of 72 h. At the endpoint, plates were treated with 5 mg/mL MTT (solubilized in PBS) and incubated for 3 h at 37 °C. After confirmation of formazan crystal formation, wells were aspirated before the addition of 100 μL dimethyl sulfoxide (DMSO; 0219605591; MP Biomedicals, Santa Ana, CA, USA) per well. Absorbance was measured at 560 nm using a SpectraMax i3x microplate reader (Molecular Devices LLC, San Jose, CA, USA). Absorbance readings were normalized to values from vehicle-treated wells and values from 100 μM cisplatin-treated wells. The drug’s half-maximal inhibitory concentration (IC-50) was determined in cisplatin-sensitive and resistant cells using GraphPad Prism v 9.5.0 (GraphPad Software, La Jolla, CA, USA). Initial PDX4 IC-50 values were compared to previously published PDX4 IC-50 values to validate cisplatin sensitivity [6]. Results were validated through multiple independent experiments (*n* = 3). 

### 4.4. Library Preparation and RNA Sequencing

Total RNA was extracted from PDX4 SE and PDX4 CR cells using the miRNeasy Mini Kit (217004, Qiagen, Germantown, MD, USA). Before sequencing, RNA quality was checked through agarose gel electrophoresis using 2X RNA loading dye (R0641; Thermo Fisher Scientific) according to the manufacturer’s protocol. All RNA samples were derived from three independent experiments. Subsequently, RNA-seq library construction and generation of raw data was performed at the Loma Linda University Center for Genomics. RNA-seq libraries were constructed using the Ovation Universal RNA-seq System (0364; Tecan; Männedorf, Switzerland). Briefly, 100 ng of total RNA was reverse transcribed and then made into double-stranded cDNA by adding a DNA polymerase. cDNA was concentrated using Agencourt beads, followed by end repair and adaptor ligation. Unique barcodes were used for each sample for multiplexing. Targeted rRNA-depletion was performed before the final library construction. Libraries were amplified using 13 cycles in the Eppendorf^TM^ Mastercycler^TM^ pro PCR system (Hamburg, Germany) and purified using Agencourt beads. 

Before library construction, RNA quality was assessed using RNA integrity (RIN) via a TapeStation 2200 (Agilent Technologies, Santa Clara, CA, USA). All libraries were quantified using Qubit 4.0 (Life Technologies, Carlsbad, CA, USA). Following RNA seq library construction, library quality, and size distribution were evaluated with D1000 ScreenTape using TapeStation 2200 (Agilent Technologies, Santa Clara, CA, USA).

RNA-seq libraries were sequenced on Illumina NextSeq 550 (Illumina, San Diego, CA, USA) with single 76 bp reads. Illumina RTA v2.4.11 software was used for basecalling, and bcl2fastq v2.17.14 was used for generating FASTQ files.

Specific analysis pipelines and codes used to generate graphs can be found at https://github.com/tsuzukiPhD/RNAseq (accessed on 8 January 2024). 

### 4.5. Bioinformatics Analysis

Sample quality was determined with FASTQC (v 0.11.9) [74], and MultiQC (v 1.11) before and after reads were trimmed with Trimmomatic (v 0.39) [75]. Subsequently, reads were aligned to the human genome with STAR (v2.7.10a) [76], using GRCh38 [77] (accessed on 4 May 2023), release 109, as a reference. BAM (binary alignment map) files that were generated from STAR alignment were sorted and indexed with SAMtools (v 1.12) [78]. Further processing of reads involved (1) utilizing HTSeq (v 2.0.2) for the assembly of RNA-seq reads into transcripts, (2) counting transcripts, and (3) producing a count matrix from the HTSeq output for the differential gene expression analysis between samples with DESeq2 (v 1.43.1) [79,80]. Genes with less than 10 counts in all samples were excluded from the matrix to enhance the robustness of gene expression analysis. Downstream analysis required the conversion of Ensembl identifiers (ID) to HGNC symbols, which was done with the BioMart R package (v 2.58.0) [81].

### 4.6. Pathway Analysis

The PROGENy (v 1.24.0) [82] R package was used for inference of pathway activity. DEGs were selected based on whether their expression had an adjusted *p*-value < 0.1 and absolute FC greater than 1.5. Genes that did not fit these criteria were filtered out from the normalized count matrix, which was used as input for PROGENy. Cisplatin-resistant samples were compared to sensitive samples with a linear regression model. The *p*-values obtained were adjusted using the Banjamini-Hoechberg false discovery rate (FDR) method. Activity was considered significant if FDR was <0.1.

Ingenuity Pathway Analysis (IPA; QIAGEN Inc., Hilden, Germany, https://digitalinsights.qiagen.com/IPA; accessed on 5 May 2023) and Gene Set Enrichment Analysis (GSEA; v 4.3.2; accessed on 27 October 2023) were used for the identification of canonical/hallmark pathways activated in the DEGs of PDX4 CR [83,84,85].

### 4.7. Microscope Imaging

Cell imaging acquisitions were performed with a Nikon Eclipse Ti microscope (Nikon Instruments, Melville, NY, USA) and μManager v 1.4.22 software. Unless otherwise noted, images were taken at 10× magnification. Magnification insets were generated and added to image panels with the QuickFigures (https://github.com/grishkam/QuickFigures; accessed on 28 November 2023) plugin in ImageJ.

### 4.8. Flow Cytometry

The apoptosis assay was conducted using Annexin V-Pacific Blue (640918; Biolegend, San Diego, CA, USA) and 7AAD (13-6993-T500; Tonbo Biosciences, San Diego, CA, USA). One hundred thousand cells were plated per well in a 12-well plate. Cells were treated with cisplatin for 72 h and then harvested in PBS. After centrifugation, cells were resuspended in 5 μL of Annexin V and 10 μL Annexin V binding buffer (20 mM HEPES, 140 mM NaCl, and 2.5 mM CaCl_2_ in ddH_2_O) and incubated for 15 min at 4 °C. Next, cells are stained with 5 μL of 7AAD for 5 min at 4 °C. Once the incubation period ends, cell suspension is diluted with 180 μL of Annexin V binding buffer. Single stain controls were also plated for both Annexin V-PB and 7AAD. 

For cell cycle analysis, PDX4 CR cells were cultured without Cisplatin (10 uM) for 1 week and collected before the assay was performed. PDX4 SE and CR were collected via trypsinization, counted to 1 × 10^6^, rinsed with room temperature 1× sterile PBS twice, fixed with chilled 70% EtOH, and rinsed with 1× sterile PBS twice. Cells were resuspended in 500 μL of FxCycle™PI/RNase Staining Solution (F10797, lot# 2561624, Invitrogen, Carlsbad, CA, USA) and left to incubate in the dark for 30 min. 

Cells were resuspended in FACS stain [1% FBS, 0.1% sodium azide (NaN_3_; s2002-5g; Sigma), and 2 mM EDTA in PBS] or BD Horizon Brilliant Stain Buffer (563794; BD, Franklin Lakes, NJ, USA), and labeled with conjugated fluorescent dye antibodies against CD44 BB700 (745949), CD117 APC (567128), CD133 BV421 (566598), CD324 BV480 (E-Cadherin; 752951), and CD325 PE (N-Cadherin; 561554) obtained from BD. After incubation at 4 °C for 30 min, cells were washed and resuspended in FACS stain. UltraComp eBeads (01-2222; Thermo Fisher Scientific) and BD Horizon^TM^ fixable viability stain 780 (565388) were used for compensation and live cell gating, respectively. ALDH-positive cells were identified with the ALDEFLUOR^TM^ assay kit (01700; STEMCELL Technologies, Vancouver, BC, Canada) using the manufacturer’s instructions. Briefly, one million cells were resuspended in 1 mL of ALDEFLUOR^TM^ Assay Buffer. Subsequently, five microliters of activated ALDEFLUOR Reagent were added to the cell suspension and mixed. Half of the cell suspension was immediately transferred to the DEAB Reagent control tube before incubation for 30 min at 37 °C. 

Flow cytometry was performed on MACSQuant Analyzer 10 (Miltenyi Biotec, Bergisch Gladbach, Germany), and data analysis was performed using FlowJo 10 (FlowJo LLC, Ashland, OR, USA).

### 4.9. Proliferation Assay

Cells were seeded in two flat-bottomed 96-well plates: the test plate was seeded with 1000 cells/well, and the standard growth curve plate with a two-fold serial dilution of cell suspension, ranging from 0 to 50,000 cells/well in triplicates. After incubation for 24 h, the standard growth curve plate and quadruplicates of the test plate were treated with 5 mg/mL MTT (solubilized in PBS) and incubated for 3 h at 37 °C. After confirmation of formazan crystal formation, wells were aspirated before the addition of 100 μL DMSO per well. Absorbance was measured at 560 nm using a SpectraMax spectrophotometer. Absorbance readings from the standard growth curve were normalized to blank wells before being calculated into relative viable cell count. The remaining wells from the test plate were harvested in quadruplicates in the following days (up to 7 days) for the calculation of doubling time. After each reading, wells were washed at least three times once DMSO was removed. 

### 4.10. Quantitative Reverse Transcription PCR (RT-qPCR)

Total RNA from cell culture samples was isolated using the IBI Isolate DNA/RNA Reagent Kit (#IB47602, IBI Scientific, Dubuque, IA, USA) according to the manufacturer’s instructions. After RNA purification, cDNA was synthesized from 1 μg of total RNA using Maxima First Strand cDNA Synthesis Kit (K1672; Thermo Fisher Scientific). RT-qPCR was performed using Applied Biosystems^TM^ PowerUP^TM^ SYBR^TM^ Green Master Mix (A25778; Thermo Fisher Scientific) and specific primers on a Stratagene Mx3005P Instrument (Agilent Technology, Santa Clara, CA, USA). The sequence and amplicon size of all human primers is shown in Table S7. The results were analyzed using the ΔΔ cycles to threshold (ΔΔ*C*_t_) method [86].

### 4.11. Protein Extraction and Quantification

To quantify protein expression, cisplatin sensitive and resistant cells were plated in triplicates in 6-well (100,000 cells/well) or 12-well plates (50,000 cells/well) and allowed to expand for 72 h before trypsinization, PBS washing, and addition of Laemmli sample buffer [0.5 M Tris HCl at pH 6.8 (50-213-711; Fisher Scientific), 10% sodium dodecyl sulfate (SDS), 100 mM PMSF (329-98-6; Sigma-Aldrich) in isopropanol (190764-4x4l; Sigma), glycerol (G5516-1L; Sigma-Aldrich), and protease inhibitor (11836170001; Sigma-Aldrich) dissolved in ddH_2_O]. Lysates of cells were sonicated with a Sonic Dismembrator Model F60 (remote, 10 s at 50% power, then 2 min on ice, repeated three times; Fisher Scientific) and passed through a Hamilton syringe (14-824-663; Thermo Fisher Scientific) to dissociate proteins attached to chromatin. Subsequently, samples were centrifuged at 10,000 rpm for 10 min, and the supernatant was transferred to a new tube. The membranes were stained with one antibody at a time to ensure specificity. Quantification of proteins was carryout out with a BCA protein assay (23227; Thermo Fisher Scientific). Absorbance was measured at 562 nm using a SpectraMax i3x microplate reader (Molecular Devices LLC). Values were normalized to both the internal control (GAPDH or alpha-beta tubulin) and the average value of the control sample (PDX4 SE). 

### 4.12. Electrophoresis and Immunoblotting

Loading buffer [8% SDS, 10% 2-mercaptoethanol (21985023; Life Technologies, Carlsbad, CA, USA), 30% glycerol, 0.008% bromophenol blue (BP115-25; FisherScientific, Fair Lawn, NJ, USA), and 0.2 M Tris HCl in ddH_2_O] was added to 50 μg of proteins from whole cell lysates, then samples were heated to 100 °C for 5 min before being loaded into individual lanes of 4–12% SurePAGE^TM^ Bis-Tris polyacrylamide gels (GenScipt, Piscataway, NJ, USA) and separated with electrophoresis within MES SDS running buffer (M00677; GenScript), using a Mini PREOTEAN 3 cell (525BR058974; Bio-Rad, Hercules, CA, USA) and PowerPac^TM^ Power Supply (043BR09142; Bio-Rad) at 150 V for 1 h and 45 min in a cold room. Proteins were then transferred to a 0.45 μm Immobilon-FL PVDF membranes (IPFL00010; EMD Millipore, Burlington, MA, USA) using the eBlot^TM^ L1 Fast Wet Transfer System (GenScript) according to the manufacturer’s instructions. Membranes were dried at room temperature for 1 h, re-activated with methanol (MX0485-7; EMD Millipore), and blocked with 5% milk in TBS [20 mM Tris–HCl, pH 7.6, and 140 mM NaCl (SB0476-1; Bio Basic, Markham, ON, Canada) in ddH_2_O] for 1 h. Membranes were probed with corresponding primary antibodies at a 1:1000 dilution (5% milk in TBST) overnight at 4 °C. Primary antibodies (Cell Signaling Technologies, Danvers, MA, USA) included mouse anti-Snail (3895S), rabbit anti-Vimentin (5741), rabbit anti-LIN28A (3978), rabbit anti-OCT4A (2840), rabbit anti-α/β-tubulin (2148), and rabbit anti-GAPDH (2118). Secondary antibody immunoblotting was done with goat anti-rabbit IgG (IRDye 680RD; 926-68071; LI-COR Biosciences, Lincoln, NE, USA, or DyLight 680; PI35569; Thermo Fisher Scientific) and goat anti-mouse DyLight 800 (SA5-10176; Invitrogen, Waltham, MA, USA) for an hour at a 1:30,000 working concentration (5% milk in TBST with 0.01% SDS) at room temperature. Membranes were imaged with LI-COR Odyssey CLx Infrared Imaging System (LI-COR Biosciences) and analyzed with ImageJ software v1.54f (National Institutes of Health, Bethesda, MD, USA) and ImageStudio (LI-COR Biosciences). Full blots are shown in Figures S1 and S2.

### 4.13. GFP Reporters

FUGW-eGFP-ZEB1 3’UTR was a gift from Dr. Jeffrey Rosen (Addgene plasmid #81018; RRID: Addgene_81018). SORE6-GFP lentiviral reporters were a gift from Dr. Lalage M. Wakefield. Both plasmids were selected with ampicillin in Stbl3 *E. coli* (C737303; Invitrogen) cultures and verified with DNA digestion and gel electrophoresis before clonal expansion and purification with QIAGEN Plasmid Maxi Kit was performed (12163; Qiagen, Redwood City, CA, USA). Lentivirus particles were produced in HEK293T cells after co-transfection of lentivirus plasmid vectors with packaging plasmids (VSVG and lenti gag/pol) using polyethylenimine (PEI; NC1014320; Polysciences, Warrington, PA, USA). After 48 h and 72 h, a medium containing lentivirus was collected and filtered through a 0.22 μM filter. Filtered virus-containing medium was used for cell transduction or stored at −70 °C. Cells were transduced with lentivirus in the presence of 6 μg/mL protamine sulfate (p3369-10g, Sigma-Aldrich), and flow cytometry for the identification of GFP expression was performed within two weeks of transduction.

### 4.14. Scratch Migration Assay

Cisplatin-sensitive and resistant PDX4 cells were seeded in 48-well tissue culture plates, with 1.0 × 10^6^ and 1.2 × 10^6^ cells per well, respectively, according to their growth rate and phenotypes to achieve overnight confluence. Once the plates reached confluency, growth media was replaced with FBS-free media, and serum starvation was conducted for 24 h before wells were scratched with a 10-μL pipet tip. Scratches were imaged at regular time intervals using a Nikon Eclipse Ti microscope. Images were analyzed with ImageJ software.

### 4.15. Colony Formation Assay

PDX4 SE and PDX4 CR cells were seeded at low density in 48-well plates (20 cells/well; *n* = 24 per plating; 25-108MP; Genesee Scientific) for 10 days. On day 5, additional media was added to replenish nutrients. On the final day, surviving colonies were washed with PBS, fixed with methanol mixed with acetic acid (A6283-2.5L; Sigma-Aldrich) solution (3:1 ratio) for 5 to 10 min, and washed with PBS again before staining. Colonies were stained with 0.1% crystal violet dissolved in methanol and diluted in PBS (1:10) for 20 min. The crystal violet solution was removed, and the residual solution was washed gently with tap water. Colonies were airdried overnight and imaged with an EPSON Perfection V500 Scanner. Colonies were quantified manually and with the ImageJ software’s automated colony counting feature. Identical parameters were utilized for all the wells.

### 4.16. Statistical Analysis

For all experiments, samples in the same treatment group were harvested from at least three biological replicates and processed individually. All values in the figures and text are the means ± SD. Graphs were generated, and statistical analyses were performed using Prism v 10.1.1. Statistically significant differences were determined by unpaired *t*-tests, selected as parental cells were split into two groups and subsequently treated as separate populations or two-way ANOVAs unless otherwise noted. *p*-values less than 0.05 were considered significant. Outliers were not removed. 

## Data Availability

The data is contained within the article and Supplementary Materials.

## Supplementary Materials

The following supporting information can be downloaded at https://www.mdpi.com/article/10.3390/ijms25073843/s1.
