# Supplementary material for: A Stem-like Patient-Derived Ovarian Cancer Model of Platinum Resistance Reveals Dissociation of Stemness and Resistance
Source: Int J Mol Sci. 2024 Mar 29;25(7):3843. doi: 10.3390/ijms25073843 (PMC11011340; doi:10.3390/ijms25073843)
Supplement: Supplementary file 1 [file ijms-25-03843-s001.zip › Figure S1.pdf]

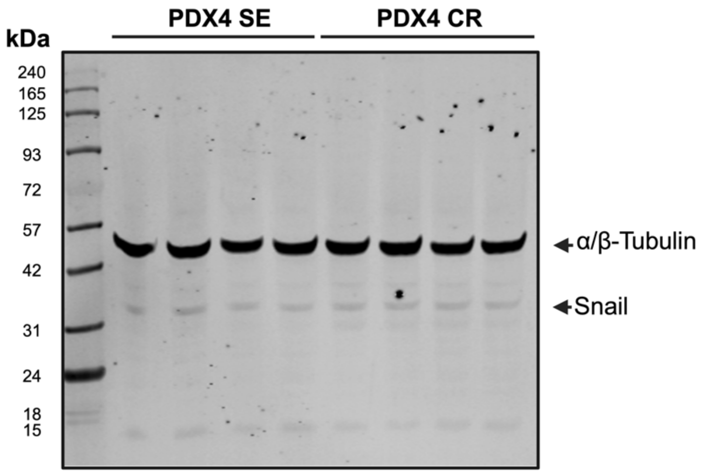

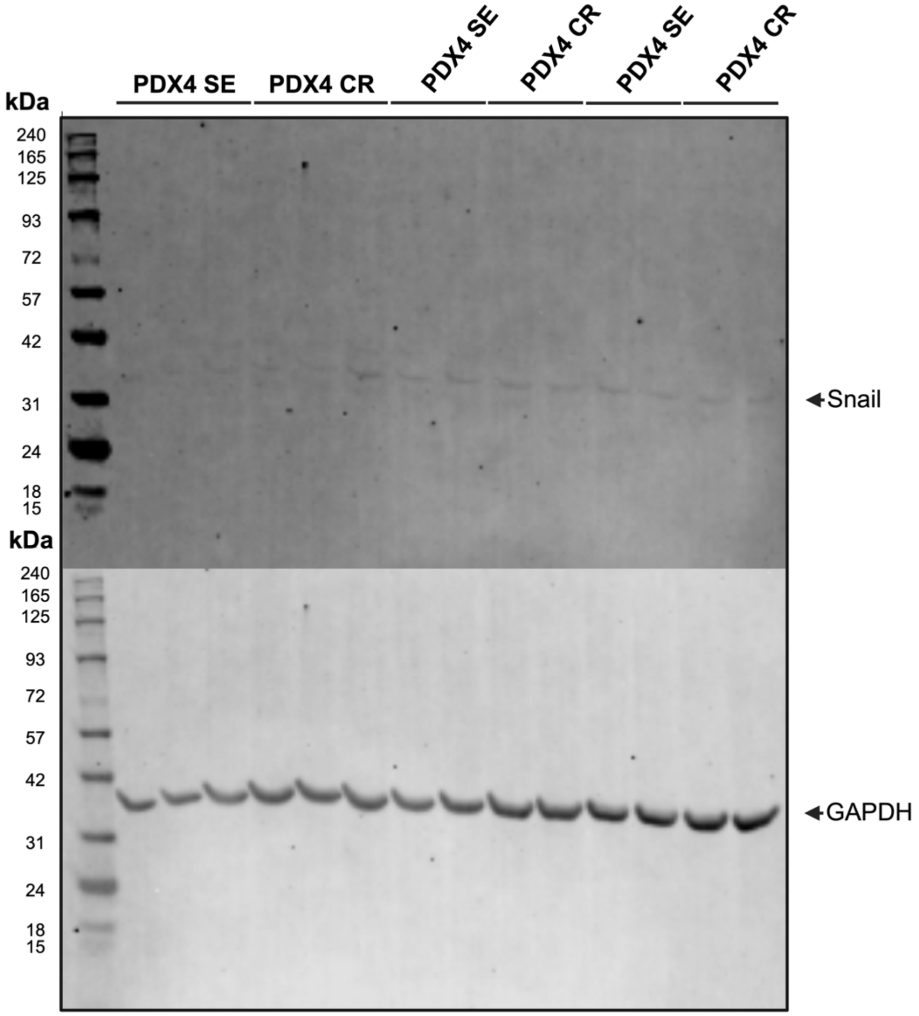

Figure S1. Snail Western blots. Whole protein was extracted from PDX4 SE and CR. After protein quantification with BCA protein assay, fifty micrograms of protein was added per lane and electrophoresed for 1 hr and 50 min. Protein transfer to PVDF membranes was done using Genscript's eBlot^{TM} L1 Fast Transfer System with the manufacturer's recommended settings. Membranes were then probed with anti-Snail and anti-Tubulin/Gapdh (internal controls), washed, and scanned with Licor Odyssey DLx Imager.
