# Supplementary material for: A Stem-like Patient-Derived Ovarian Cancer Model of Platinum Resistance Reveals Dissociation of Stemness and Resistance
Source: Int J Mol Sci. 2024 Mar 29;25(7):3843. doi: 10.3390/ijms25073843 (PMC11011340; doi:10.3390/ijms25073843)
Supplement: Supplementary file 1 [file ijms-25-03843-s001.zip › Figure S3.pdf]

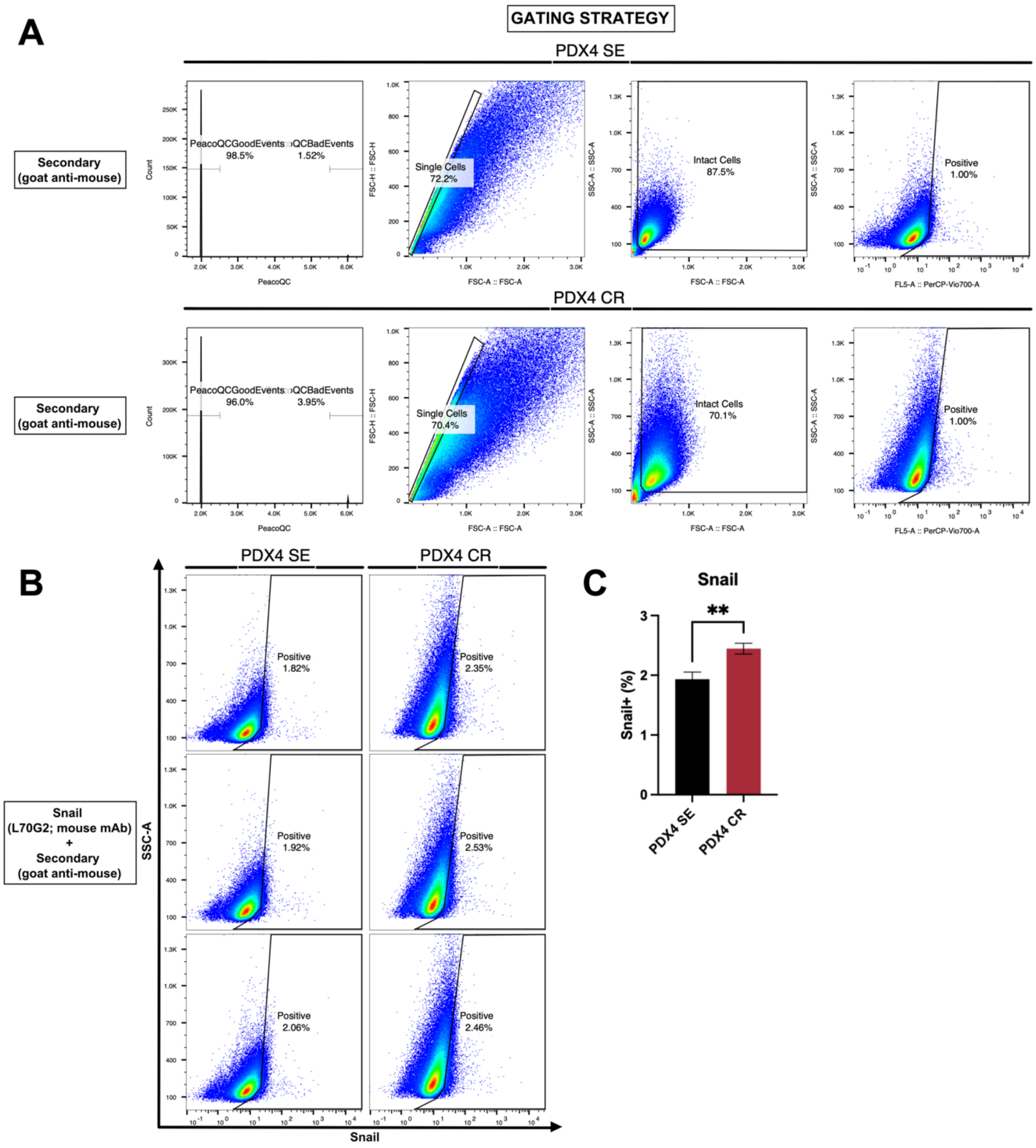

Figure S3. Quantification of Snail expression with flow cytometry. A. Gating strategy for the identification of positive population relative to populations stained with only secondary antibody. **B.** Dot plots demonstrating proportion of Snail positive cells. **C.** Bar chart depicting PDX4 CR with a significantly higher expression of Snail when compared to PDX4 SE.
