# Supplementary material for: A Stem-like Patient-Derived Ovarian Cancer Model of Platinum Resistance Reveals Dissociation of Stemness and Resistance
Source: Int J Mol Sci. 2024 Mar 29;25(7):3843. doi: 10.3390/ijms25073843 (PMC11011340; doi:10.3390/ijms25073843)
Supplement: Supplementary file 1 [file ijms-25-03843-s001.zip › Figure S6.pdf]

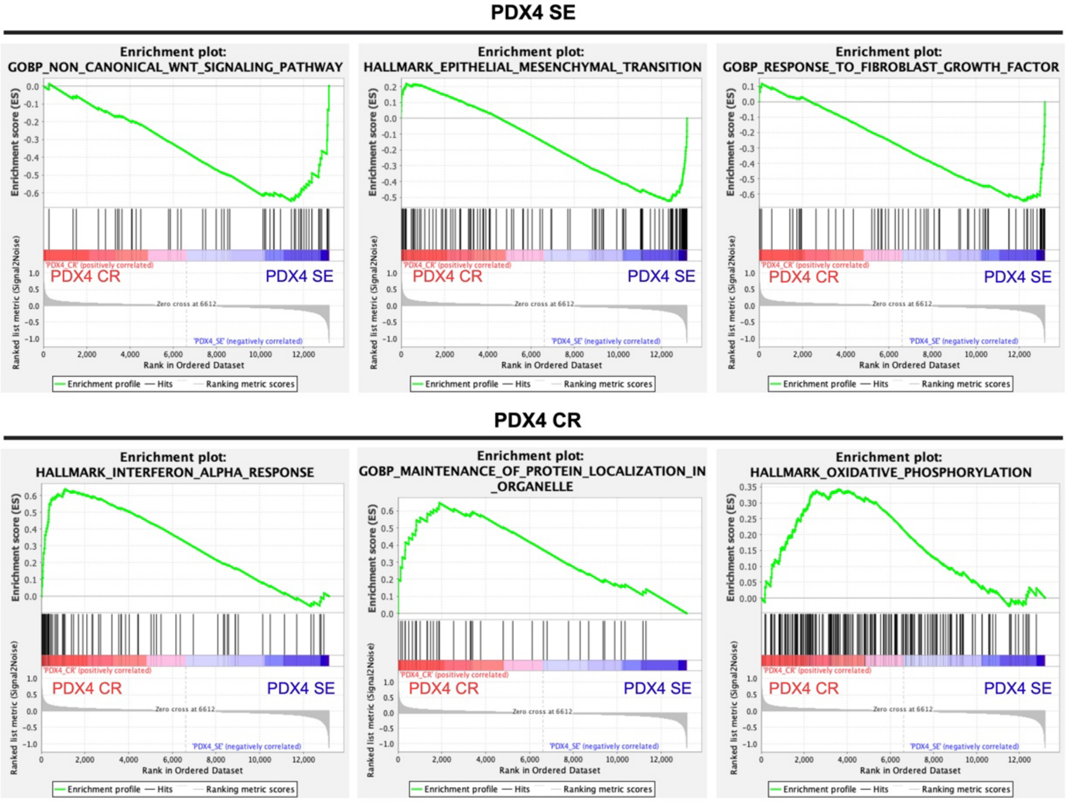

Figure S6. Gene set enrichment analysis for cisplatin sensitive and resistant PDX4. PDX4 SE had an enrichment in genes associated with non-canonical Wnt signaling, epithelial-mesenchymal transition, and response to fibroblast growth factor. PDX4 CR had an enrichment in genes associated with interferon alpha response, maintenance of protein localization in organelle, and oxidative phosphorylation.
