# Supplementary material for: A Stem-like Patient-Derived Ovarian Cancer Model of Platinum Resistance Reveals Dissociation of Stemness and Resistance
Source: Int J Mol Sci. 2024 Mar 29;25(7):3843. doi: 10.3390/ijms25073843 (PMC11011340; doi:10.3390/ijms25073843)
Supplement: Supplementary file 1 [file ijms-25-03843-s001.zip › Figure S7.pdf]

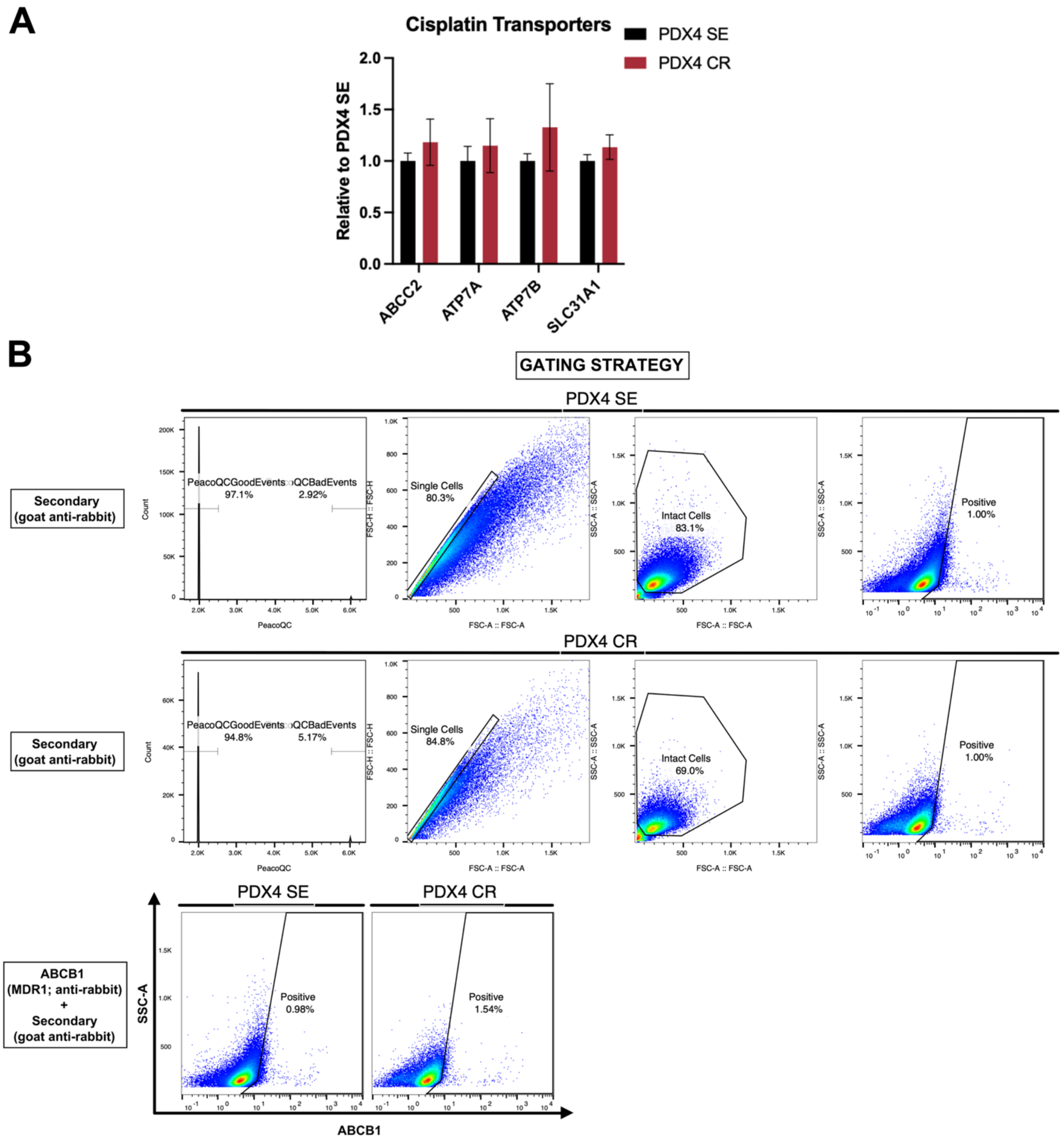

Figure S7. Quantification of cisplatin/copper transporter expression. A. RT-qPCR quantification of ABCC2, ATP7A, ATP7B, SLC31A1. **B.** Gating strategy and quantification of ABCB1/MDR1 through flow cytometry.
