# Supplementary material for: A Stem-like Patient-Derived Ovarian Cancer Model of Platinum Resistance Reveals Dissociation of Stemness and Resistance
Source: Int J Mol Sci. 2024 Mar 29;25(7):3843. doi: 10.3390/ijms25073843 (PMC11011340; doi:10.3390/ijms25073843)
Supplement: Supplementary file 1 [file ijms-25-03843-s001.zip › Table S1.pdf]

Table S1. List of differentially expressed genes in PDX4 CR relative to PDX4 SE generated by DESeq2 analysis

ENSEMBL_ID	baseMean	log2FoldChange	lfcSE	stat	pvalue	padj
ENSG00000000003	40.59986718	-0.35429728	0.274947356	-1.288600429	0.197537034	0.349075698
ENSG00000000419	428.4565816	-0.142497551	0.098644362	-1.444558498	0.148581937	0.28288567
ENSG00000000457	105.8455314	0.004146622	0.172980401	0.02397163	0.980875238	0.990179984
ENSG00000000460	119.7737167	0.013348296	0.169473231	0.078763447	0.937220778	0.965610993
ENSG00000000971	1583.423364	-0.339207603	0.078016243	-4.347909973	1.37E-05	9.57E-05
ENSG00000001084	1689.17011	4.21014563	0.086992924	48.39641492	0	0
ENSG00000001167	446.3020695	-0.024597817	0.09720158	-0.253059851	0.800221963	0.880924836
ENSG00000001460	81.57840761	-0.060706794	0.199694523	-0.303998295	0.76112919	0.855668806
ENSG00000001461	187.1915532	-0.321289092	0.146285546	-2.196314683	0.028069428	0.076324283
ENSG00000001497	613.2490046	0.181135977	0.090717717	1.996699027	0.045857888	0.113951132
ENSG00000001617	78.36764284	-0.371397593	0.202671616	-1.832509164	0.066875598	0.15349698
ENSG00000001629	348.7071257	0.011520765	0.108583337	0.106100671	0.915502479	0.954364625
ENSG00000001630	55.68681438	0.475792767	0.262448412	1.812900152	0.069847223	0.158761155
ENSG00000001631	137.7896528	-0.214258157	0.160495673	-1.334977784	0.181883618	0.328691519
ENSG00000002016	43.25408199	0.114692183	0.270740742	0.423623657	0.6718403	0.790794427
ENSG00000002330	112.5164289	0.298876406	0.178351016	1.675776303	0.093782017	0.199578607
ENSG00000002549	352.0957271	0.338560778	0.10914106	3.102047747	0.00192187	0.007909183
ENSG00000002586	1029.585846	0.541452485	0.081101661	6.67621939	2.45E-11	4.15E-10
ENSG00000002834	798.4529909	-0.252243008	0.084674072	-2.978987582	0.002892025	0.011185098
ENSG00000002919	77.58454902	-0.280325493	0.203279608	-1.379014331	0.167890335	0.310105923
ENSG00000003056	564.2283616	0.258856746	0.088821442	2.914349721	0.003564304	0.013412208
ENSG00000003096	78.00559189	0.26846853	0.201576501	1.331844376	0.182911332	0.329996851
ENSG00000003249	359.5801931	0.274171907	0.104781899	2.616596077	0.008881137	0.029209395
ENSG00000003393	431.0186537	-0.416354915	0.100006822	-4.163265132	3.14E-05	0.000202246
ENSG00000003402	367.999499	-0.167125622	0.120413974	-1.387925472	0.16515974	0.306432291
ENSG00000003436	443.8603386	-4.977707669	0.186195939	-26.73370691	1.91E-157	9.94E-155
ENSG00000003509	220.4103281	-0.423783348	0.128139098	-3.307213442	0.000942291	0.00426429
ENSG00000003756	1072.219378	-0.0180742	0.071125065	-0.254118571	0.799403957	0.880792798
ENSG00000003989	217.7315921	-0.451706019	0.12772439	-3.536568221	0.000405362	0.002006446
ENSG00000004059	220.275701	0.572998082	0.12384087	4.626889974	3.71E-06	2.93E-05
ENSG00000004139	420.0521618	0.226776257	0.097408271	2.328100639	0.01990676	0.057564343
ENSG00000004142	612.4889898	0.029011239	0.085667142	0.338650718	0.734872871	0.836663901
ENSG00000004399	973.8801386	1.640534889	0.086237055	19.02354961	1.09E-80	2.04E-78
ENSG00000004455	512.4103474	-0.236978149	0.090960151	-2.605296336	0.009179483	0.030021932
ENSG00000004478	1015.360089	0.208325886	0.080173748	2.598430169	0.009365109	0.030550787
ENSG00000004487	1328.641659	0.139084172	0.068306045	2.036191258	0.041731154	0.105773641
ENSG00000004534	1001.500619	-0.011602143	0.078415643	-0.147956989	0.882376712	0.935240751
ENSG00000004660	165.5858285	1.027568845	0.143930951	7.139318119	9.38E-13	1.86E-11
ENSG00000004700	200.7401961	-0.129573836	0.131264058	-0.987123497	0.3235821	0.495352851
ENSG00000004766	67.88462671	0.157716382	0.228817217	0.689267986	0.49065464	0.650209975
ENSG00000004777	55.83094121	-0.120043489	0.240427492	-0.499291857	0.617573791	0.751514768
ENSG00000004779	838.0940494	0.362367294	0.098016562	3.697000662	0.000218162	0.001156859
ENSG00000004864	169.8582811	-0.775799983	0.147268512	-5.267928461	1.38E-07	1.42E-06
ENSG00000004866	191.1246944	0.444809025	0.138148751	3.219783183	0.001282876	0.005573943
ENSG00000004897	845.0165815	-0.016766417	0.079737507	-0.210270139	0.83345684	0.902386945
ENSG00000004961	131.1328716	0.250889553	0.171418583	1.46360767	0.143301176	0.275595785
ENSG00000004975	528.6556523	0.268272481	0.094780246	2.830468296	0.004647992	0.016831745
ENSG00000005007	591.0822676	0.023734346	0.090657118	0.26180345	0.793472979	0.876940593

ENSG00000005022	2268.238954	0.223072817	0.064675483	3.449109428	0.000562439	0.002687622
ENSG00000005059	150.2419536	0.205193629	0.158263328	1.296533011	0.194791915	0.346015632
ENSG00000005073	104.0362267	-0.36972443	0.178756313	-2.068315366	0.038610378	0.099025009
ENSG00000005075	245.1768107	0.484555919	0.121905622	3.974844719	7.04E-05	0.000419891
ENSG00000005100	617.3667726	0.078865577	0.085653178	0.920754827	0.357178444	0.528469097
ENSG00000005102	125.6211748	-2.977893496	0.211960061	-14.04931419	7.78E-45	6.87E-43
ENSG00000005108	184.9935407	-0.824914714	0.143385449	-5.753127113	8.76E-09	1.08E-07
ENSG00000005156	363.4329032	0.174268001	0.106895642	1.630262917	0.103045941	0.214677312
ENSG00000005175	409.5213454	-0.092685343	0.098902359	-0.937139862	0.348686619	0.520620848
ENSG00000005187	7.263415843	-1.865889024	0.720032893	-2.591394147	0.009558794	0.03108157
ENSG00000005189	78.83300503	-0.236014451	0.205036089	-1.151087364	0.249696296	0.413789489
ENSG00000005194	226.8440016	0.181146784	0.136432582	1.32773844	0.18426453	0.331713937
ENSG00000005206	101.5063749	-0.248276737	0.204875621	-1.211841292	0.225573139	0.383961822
ENSG00000005238	82.18132588	-0.087861125	0.201566009	-0.435892564	0.662914681	0.784604829
ENSG00000005249	352.7246415	1.250506681	0.109019387	11.47049822	1.86E-30	1.03E-28
ENSG00000005302	138.3234565	0.389766254	0.159377776	2.445549581	0.014463156	0.044127705
ENSG00000005339	2147.615077	0.03539113	0.060550068	0.584493653	0.558888234	0.705699959
ENSG00000005436	196.0365982	0.049359488	0.133647795	0.369325121	0.711885403	0.819996249
ENSG00000005448	127.2504419	0.139072182	0.179188551	0.776122034	0.437676935	0.603314879
ENSG00000005469	25.98250733	-0.205808169	0.338122371	-0.608679538	0.542736872	0.691942716
ENSG00000005483	986.3050266	0.639336279	0.074237072	8.612089113	7.17E-18	2.11E-16
ENSG00000005486	303.7184015	0.126357271	0.114895317	1.099759968	0.271436719	0.439119151
ENSG00000005513	16.70758001	2.510385323	0.503554543	4.98532951	6.19E-07	5.74E-06
ENSG00000005700	614.962276	-0.154215666	0.083977506	-1.836392545	0.066299621	0.152424023
ENSG00000005801	247.5180544	-0.282172016	0.122887991	-2.296172427	0.021666024	0.061620297
ENSG00000005810	1146.758245	0.066152343	0.07223454	0.915799328	0.359772156	0.531076027
ENSG00000005812	143.2115773	0.218756135	0.159733487	1.369507041	0.170840835	0.313921637
ENSG00000005882	261.6879307	0.096061988	0.118165127	0.812947017	0.416248436	0.584061711
ENSG00000005884	51.84807895	-0.983643227	0.258991652	-3.797972707	0.000145884	0.000808059
ENSG00000005889	541.335623	-0.172844805	0.093904415	-1.840646202	0.065673423	0.151355958
ENSG00000005893	312.4608514	0.31028105	0.109008594	2.846390731	0.00442179	0.016137409
ENSG00000005961	19.62805734	-0.561662866	0.413646832	-1.357831905	0.174517028	0.318907337
ENSG00000006007	415.4254562	0.195323236	0.09879492	1.977057497	0.048035131	0.118273355
ENSG00000006015	126.4113084	-0.004129637	0.162559944	-0.025403782	0.979732895	0.989786143
ENSG00000006016	14.2940776	0.322804406	0.506489837	0.637336394	0.52390575	0.677354147
ENSG00000006025	8.661726004	-2.66600567	0.746813299	-3.569842256	0.000357196	0.001795825
ENSG00000006062	137.5301489	-0.153068293	0.158465995	-0.965937787	0.334075313	0.505990311
ENSG00000006071	14.91212167	-1.057270646	0.465044586	-2.273482324	0.02299713	0.064659865
ENSG00000006118	491.9679166	0.119524488	0.103872054	1.150689558	0.249859974	0.413838003
ENSG00000006125	1141.309134	-0.540204054	0.070201038	-7.695100664	1.41E-14	3.30E-13
ENSG00000006194	142.5398974	0.033197074	0.159278561	0.208421483	0.834899884	0.903322704
ENSG00000006282	80.58569165	-0.350663885	0.200905302	-1.74541877	0.080912001	0.178145905
ENSG00000006283	86.3886801	-0.93523704	0.194627746	-4.805260607	1.55E-06	1.31E-05
ENSG00000006327	101.3714916	0.137479711	0.189310978	0.726210983	0.46770944	0.629756504
ENSG00000006432	114.7213582	-0.901787957	0.174247772	-5.175319878	2.28E-07	2.27E-06
ENSG00000006451	224.5132658	-0.170744675	0.127909777	-1.334883693	0.181914416	0.328704888
ENSG00000006453	387.1915411	-0.285213787	0.101533299	-2.809066488	0.004968538	0.01781344
ENSG00000006459	168.39475	0.970455191	0.145446173	6.672263507	2.52E-11	4.26E-10
ENSG00000006468	570.6057082	1.012277183	0.090142438	11.2297516	2.91E-29	1.53E-27
ENSG00000006530	382.5529226	0.25385802	0.1043113	2.4336579	0.014947114	0.045377592

ENSG00000006534	61.7497913	1.422196614	0.250169932	5.684922249	1.31E-08	1.59E-07
ENSG00000006576	380.7560598	-0.064152699	0.100146669	-0.640587446	0.521790758	0.675986973
ENSG00000006607	566.384809	0.201590815	0.092270961	2.184769861	0.028905725	0.07814448
ENSG00000006625	125.6035039	-0.037333469	0.160067095	-0.233236375	0.815577861	0.891619754
ENSG00000006634	317.4248212	0.192660541	0.112054287	1.719350027	0.085550656	0.186063904
ENSG00000006638	79.56738501	0.52070808	0.238908991	2.179524833	0.029292702	0.078917258
ENSG00000006652	70.0587087	1.061794049	0.212098602	5.006134119	5.55E-07	5.20E-06
ENSG00000006695	169.9235794	-0.093292342	0.145826744	-0.639747826	0.522336557	0.676245311
ENSG00000006704	221.0371716	-0.391122049	0.140894465	-2.775993	0.005503339	0.019398448
ENSG00000006712	497.3251118	0.098360487	0.093347204	1.053705764	0.292017649	0.462535239
ENSG00000006715	274.4737161	-0.095848179	0.121666229	-0.787796092	0.430815988	0.597477385
ENSG00000006740	87.56532235	-0.441762017	0.193034355	-2.288514998	0.022107548	0.062572074
ENSG00000006744	768.5847744	0.240583407	0.079834912	3.013511255	0.002582435	0.010169739
ENSG00000006747	70.86239637	-1.86159818	0.236287599	-7.878526784	3.31E-15	8.09E-14
ENSG00000006756	9.924340433	0.905145505	0.569273228	1.590001885	0.11183438	0.228799525
ENSG00000006757	61.41195571	0.211843269	0.240618734	0.880410539	0.378636949	0.547981993
ENSG00000006831	588.3872784	0.035445157	0.085808358	0.41307348	0.679552778	0.796741918
ENSG00000006837	11.10948798	-0.501402767	0.562132797	-0.891964976	0.372411706	0.542944186
ENSG00000007047	409.9143626	-0.117193941	0.103492523	-1.132390412	0.257470334	0.423283469
ENSG00000007080	607.7962113	0.494317828	0.094163968	5.249543288	1.52E-07	1.57E-06
ENSG00000007168	574.2496258	-0.169885133	0.0869871	-1.952992273	0.050820511	0.1233372
ENSG00000007171	12.56730469	-0.894138329	0.531963489	-1.6808265	0.09279662	0.198072347
ENSG00000007202	1637.706047	-0.03364065	0.066673022	-0.504561651	0.613866751	0.748716754
ENSG00000007255	29.83514937	-0.760515468	0.322070271	-2.36133396	0.018209324	0.053534193
ENSG00000007264	200.9114436	-0.28202104	0.140615447	-2.005619196	0.044896892	0.11205875
ENSG00000007314	7.98281781	-0.987196545	0.660446772	-1.49474051	0.134982177	0.263822825
ENSG00000007341	153.5616188	-0.267098609	0.157596591	-1.694824788	0.090108664	0.193511145
ENSG00000007372	342.0672948	-7.57531094	0.465183276	-16.28457284	1.27E-59	1.65E-57
ENSG00000007376	172.1665231	-0.065680434	0.139483087	-0.470884572	0.637723166	0.766193178
ENSG00000007384	145.6710171	0.005079542	0.1490158	0.034087274	0.972807556	0.986075028
ENSG00000007392	493.1557255	-0.078532585	0.092362998	-0.850260241	0.395180416	0.563217718
ENSG00000007402	27.49103176	-2.399684213	0.405690916	-5.915055322	3.32E-09	4.32E-08
ENSG00000007520	184.4155838	0.551622882	0.141022051	3.911607287	9.17E-05	0.000532403
ENSG00000007541	60.56284626	-0.224669662	0.237024878	-0.947873757	0.343193726	0.515047251
ENSG00000007545	108.3727561	0.286853111	0.181037956	1.584491553	0.113081902	0.230948166
ENSG00000007866	150.8241128	-0.152853592	0.151867016	-1.006496319	0.314176882	0.485451838
ENSG00000007923	1542.937083	0.348613754	0.072631548	4.799756646	1.59E-06	1.35E-05
ENSG00000007944	90.46040906	0.212359292	0.193754581	1.096022045	0.273069127	0.44109959
ENSG00000007968	261.5453501	-0.049089715	0.120560077	-0.40718052	0.683875408	0.800149899
ENSG00000008018	888.8022528	0.471602238	0.081918746	5.756951398	8.56E-09	1.06E-07
ENSG00000008056	11.33625345	-0.029369182	0.525771816	-0.055859179	0.955453991	0.976091797
ENSG00000008083	435.4000377	-0.654045066	0.096307325	-6.791228651	1.11E-11	1.95E-10
ENSG00000008128	44.44026085	0.23969115	0.259412332	0.923977472	0.355498045	0.527037577
ENSG00000008130	299.2193881	0.186009592	0.114352622	1.626631638	0.103815356	0.215867235
ENSG00000008256	269.1740653	-0.499758558	0.118342531	-4.22298352	2.41E-05	0.000159144
ENSG00000008277	241.1494775	0.488692333	0.120005803	4.072239177	4.66E-05	0.00028903
ENSG00000008282	248.6124156	0.750259322	0.120105957	6.246645397	4.19E-10	6.15E-09
ENSG00000008283	126.0482941	0.202844173	0.163960551	1.237152299	0.216030547	0.372341032
ENSG00000008294	1216.076097	-0.300256254	0.06801484	-4.414569748	1.01E-05	7.25E-05
ENSG00000008300	548.7970077	-0.578363218	0.103933402	-5.564748232	2.63E-08	3.04E-07

ENSG00000008311	86.07932538	-1.973091817	0.224413007	-8.792234662	1.47E-18	4.65E-17
ENSG00000008324	49.77502805	0.368526749	0.249602839	1.476452555	0.139822443	0.27068438
ENSG00000008382	141.6893908	0.219236635	0.160579905	1.365280631	0.172164864	0.315429744
ENSG00000008394	455.4112282	0.642533772	0.094146061	6.824860886	8.80E-12	1.56E-10
ENSG00000008405	136.7161582	-0.678338812	0.161727214	-4.194339316	2.74E-05	0.000178469
ENSG00000008441	1356.638715	0.916815042	0.079571779	11.52186182	1.02E-30	5.75E-29
ENSG00000008513	79.04283644	1.130798923	0.211765162	5.339872293	9.30E-08	9.85E-07
ENSG00000008516	26.40744705	0.867449504	0.344204495	2.5201574	0.011730237	0.036944986
ENSG00000008710	575.7625296	-0.073254853	0.097332163	-0.752627396	0.45167385	0.616629899
ENSG00000008735	23.31362353	-1.281886287	0.375395171	-3.414764993	0.000638371	0.003009125
ENSG00000008838	543.3908649	-0.152417859	0.088669303	-1.718947301	0.085623968	0.186128115
ENSG00000008853	184.2307646	0.316884185	0.135610364	2.336725411	0.019453466	0.056460977
ENSG00000008869	347.3623585	0.476730449	0.106374995	4.481602543	7.41E-06	5.46E-05
ENSG00000008952	969.939035	-0.03070669	0.076173479	-0.403115233	0.686863455	0.802559553
ENSG00000008988	4133.190393	0.1070902	0.066923125	1.600197233	0.109554836	0.225317244
ENSG00000009307	3986.748535	0.014088185	0.054812156	0.257026648	0.797158201	0.878938874
ENSG00000009335	955.7516357	0.644990895	0.074833527	8.619009713	6.75E-18	1.99E-16
ENSG00000009413	652.2136487	-0.162779726	0.087299742	-1.864607173	0.062236487	0.14500513
ENSG00000009694	22.88194529	1.935370557	0.419753391	4.610732394	4.01E-06	3.13E-05
ENSG00000009724	26.95927912	0.02459697	0.342946603	0.07172245	0.94282279	0.968520046
ENSG00000009780	64.26038004	0.006581761	0.220740965	0.029816672	0.976213262	0.987958767
ENSG00000009830	170.7802644	-0.001943325	0.15206882	-0.012779248	0.989803912	0.994759318
ENSG00000009844	225.6909385	-0.142435941	0.129642556	-1.098681981	0.271906805	0.439626483
ENSG00000009950	92.9274492	0.280776672	0.182634747	1.537367212	0.124203411	0.248283096
ENSG00000009954	2564.001896	0.004166376	0.059075674	0.070526079	0.943774944	0.968739981
ENSG00000010017	374.9782866	0.159899383	0.101538621	1.574764176	0.115310888	0.234240354
ENSG00000010072	117.5147244	0.106024484	0.170072665	0.623406962	0.533017124	0.684458501
ENSG00000010165	294.9477642	0.096637417	0.11116781	0.86929316	0.384686803	0.553785012
ENSG00000010219	52.23770439	-0.051986437	0.240933271	-0.215771099	0.829166177	0.900163426
ENSG00000010244	1065.83522	0.081172425	0.079245168	1.024320187	0.305684066	0.47668118
ENSG00000010256	1950.918642	0.127470944	0.066496228	1.916965028	0.055242375	0.131971027
ENSG00000010270	137.7412861	-0.134842306	0.158196926	-0.852369949	0.394008794	0.562063728
ENSG00000010278	126.2001025	-0.897378205	0.163146177	-5.500455012	3.79E-08	4.29E-07
ENSG00000010292	1511.55355	-0.084155534	0.06874775	-1.224120552	0.220906754	0.378654933
ENSG00000010295	139.0709562	-0.138295901	0.158654992	-0.871676958	0.383384634	0.552575821
ENSG00000010310	42.78747789	-1.924305066	0.32006476	-6.012236598	1.83E-09	2.47E-08
ENSG00000010318	60.3827314	-0.233989989	0.223274573	-1.047992105	0.294642244	0.464881456
ENSG00000010319	46.69377142	0.688597701	0.260281485	2.645588488	0.008154896	0.02718363
ENSG00000010322	878.7853733	0.159621181	0.078519117	2.032895776	0.042063054	0.106557208
ENSG00000010327	144.4753506	0.530384638	0.157172391	3.37454075	0.00073939	0.003428923
ENSG00000010361	134.2947107	-0.172712956	0.166607118	-1.036648124	0.299899877	0.470406446
ENSG00000010404	111.6665453	0.082806969	0.181296135	0.456749777	0.647850908	0.773379936
ENSG00000010539	77.73873605	0.55184797	0.208603485	2.645439844	0.008158479	0.027189124
ENSG00000010626	34.68778596	-0.371130791	0.295970761	-1.253944107	0.209862322	0.364933899
ENSG00000010704	86.81085813	1.264746423	0.19726155	6.411520268	1.44E-10	2.23E-09
ENSG00000010803	349.0164516	-0.206323591	0.110697761	-1.863846119	0.062343317	0.145205865
ENSG00000010810	49.66794999	-0.056268172	0.257677912	-0.2183663	0.827143721	0.898668717
ENSG00000010818	477.8005895	-0.461326414	0.094224559	-4.896031569	9.78E-07	8.66E-06
ENSG00000011007	1213.752981	0.189117527	0.067672316	2.794606969	0.005196285	0.018494733
ENSG00000011009	123.8797274	0.177298033	0.161125545	1.100371969	0.271170087	0.438811526

ENSG00000011021	472.1454831	0.404704156	0.093316496	4.336898343	1.45E-05	0.000100143
ENSG00000011028	1412.661988	-0.135468717	0.075884628	-1.785193129	0.074229996	0.166142249
ENSG00000011105	197.9133626	0.37997059	0.133095328	2.854875495	0.00430537	0.015765781
ENSG00000011114	339.4857298	-0.182831339	0.108284017	-1.68844253	0.091326312	0.19567735
ENSG00000011132	82.16692019	0.802678534	0.196591089	4.082985341	4.45E-05	0.000276836
ENSG00000011143	112.3206929	-0.121743632	0.168424735	-0.722837011	0.469780026	0.631664595
ENSG00000011198	187.7010564	0.175356171	0.135756988	1.291691673	0.196463942	0.347660494
ENSG00000011243	897.653957	0.063000679	0.077334496	0.814651708	0.415271703	0.583209876
ENSG00000011258	275.6236016	-0.238828258	0.114704283	-2.082121531	0.03733137	0.096342781
ENSG00000011260	275.1105536	0.286665108	0.112206554	2.554798251	0.010624936	0.033927819
ENSG00000011275	691.4949026	0.150128983	0.082873832	1.811536646	0.070057822	0.159111111
ENSG00000011295	392.0450054	-0.095057899	0.098942134	-0.96074235	0.336681732	0.508534225
ENSG00000011304	3677.345438	0.043784789	0.058765968	0.745070504	0.456229099	0.61991199
ENSG00000011332	27.32548334	-1.025319048	0.360248843	-2.846141131	0.004425258	0.016145869
ENSG00000011376	924.3847832	-0.22320348	0.074292997	-3.004367707	0.002661335	0.010427829
ENSG00000011405	1000.65128	-0.651403634	0.078439016	-8.304587019	1.00E-16	2.72E-15
ENSG00000011422	88.64048269	-1.005145781	0.196186977	-5.123407255	3.00E-07	2.92E-06
ENSG00000011426	1014.137147	0.136758299	0.074599037	1.833244821	0.066766171	0.153270862
ENSG00000011451	598.3158388	0.104453015	0.096724012	1.079907801	0.280183239	0.449028404
ENSG00000011454	488.7605788	-0.376965327	0.099756205	-3.778865939	0.000157544	0.000866154
ENSG00000011465	1155.83733	3.288926307	0.089483012	36.75475625	9.76E-296	1.52E-292
ENSG00000011478	113.9699336	-0.077977615	0.192914378	-0.404208418	0.686059464	0.801843308
ENSG00000011485	597.7893333	0.139089445	0.086524562	1.607514006	0.107941659	0.222651721
ENSG00000011523	143.3015963	-0.630103274	0.159094959	-3.960548331	7.48E-05	0.000442835
ENSG00000011566	274.6537452	-0.248344257	0.116117866	-2.138725632	0.032457895	0.085847497
ENSG00000011638	13.25280898	-0.027045336	0.490394308	-0.055150182	0.956018817	0.97639816
ENSG00000012048	709.947829	-0.335212085	0.087511916	-3.830473606	0.000127897	0.000719202
ENSG00000012061	977.0357453	0.221450499	0.076629726	2.889877213	0.003853923	0.014325499
ENSG00000012171	45.12962224	-0.943618465	0.27316178	-3.454430798	0.000551456	0.002643788
ENSG00000012174	158.2962331	-0.134079034	0.143648482	-0.933382876	0.350622319	0.522500564
ENSG00000012211	307.2782876	0.000359944	0.109939701	0.003274012	0.997387721	0.998813347
ENSG00000012232	480.3286964	-0.338860743	0.094031135	-3.603707895	0.00031371	0.00159952
ENSG00000012660	2681.951502	-0.082468531	0.064788487	-1.272888665	0.203057552	0.356009665
ENSG00000012822	138.7326321	-0.160559696	0.160402809	-1.000978082	0.316837405	0.488488094
ENSG00000012963	201.1632461	-0.123111939	0.136766739	-0.900159934	0.368035145	0.539393505
ENSG00000012983	435.5287986	0.113595806	0.09578027	1.186004237	0.235620626	0.396663823
ENSG00000013016	561.0683228	0.051073889	0.087783613	0.581815762	0.560690786	0.707146494
ENSG00000013275	921.4946714	0.540736003	0.075234672	7.187324511	6.61E-13	1.34E-11
ENSG00000013288	342.8922584	0.210040117	0.106592514	1.970495957	0.048781558	0.11967072
ENSG00000013297	9.024080541	-2.254972147	0.67606484	-3.335437686	0.000851652	0.00388793
ENSG00000013306	878.2288829	0.234078312	0.084150576	2.781660248	0.005408162	0.019098375
ENSG00000013364	106.1470848	-0.961250153	0.179570815	-5.353042208	8.65E-08	9.21E-07
ENSG00000013374	368.9596975	0.826596343	0.104495869	7.910325549	2.57E-15	6.36E-14
ENSG00000013375	266.281609	0.007430522	0.114570003	0.064855739	0.948288862	0.971358017
ENSG00000013392	21.47729069	0.011627091	0.383643777	0.030306997	0.975822217	0.98780011
ENSG00000013441	235.6831269	0.161127904	0.131570554	1.224650185	0.220707051	0.378451165
ENSG00000013503	190.2493943	0.168279923	0.135944985	1.237853111	0.215770531	0.372121381
ENSG00000013523	251.335904	-0.127925312	0.120654489	-1.060261514	0.289025643	0.459217645
ENSG00000013561	90.75713258	-0.048794221	0.1930088	-0.252808271	0.800416376	0.88094091
ENSG00000013563	100.5125969	-0.641169618	0.180342757	-3.555283448	0.000377572	0.001887898

ENSG00000013573	253.4002976	-0.111035379	0.123907579	-0.896114509	0.370191609	0.541449555
ENSG00000013583	172.5198353	-0.042774718	0.137181043	-0.311812166	0.755183278	0.852042366
ENSG00000013588	8.993080515	-0.412436071	0.574995844	-0.717285309	0.47319807	0.634438608
ENSG00000013810	810.0043188	0.120140028	0.077406888	1.552058632	0.120648195	0.242834962
ENSG00000014123	207.0362771	0.220594502	0.133553864	1.651726843	0.098590248	0.20744416
ENSG00000014138	88.12249405	-0.238137034	0.192833754	-1.234934388	0.216854924	0.373487141
ENSG00000014164	215.7912229	0.156289274	0.139018407	1.124234389	0.260913604	0.427162401
ENSG00000014216	301.1180238	-0.549680765	0.112363466	-4.89198832	9.98E-07	8.84E-06
ENSG00000014641	660.5611598	0.089867677	0.093575583	0.960375286	0.336866372	0.508758405
ENSG00000014824	433.9445708	-0.238511837	0.108415208	-2.19998506	0.027807955	0.075774655
ENSG00000014914	312.5838754	-0.269912039	0.112423563	-2.400849352	0.016357069	0.048990991
ENSG00000014919	101.2804871	-0.529133221	0.186019381	-2.844505873	0.004448037	0.016216343
ENSG00000015133	522.1625761	0.201222188	0.096206905	2.091556608	0.036478198	0.094645224
ENSG00000015153	151.1019568	0.223855549	0.147724799	1.515355246	0.129682465	0.25624164
ENSG00000015171	619.291925	0.041476388	0.083005329	0.499683437	0.617297997	0.75140251
ENSG00000015413	11.02800538	-0.045393264	0.539960585	-0.084067737	0.933002576	0.963270344
ENSG00000015475	259.3846923	0.460594448	0.123469137	3.730441948	0.000191144	0.001026241
ENSG00000015479	266.4806426	-0.115329738	0.116185566	-0.992633954	0.320888372	0.492590252
ENSG00000015532	375.7172947	-0.066012776	0.117510786	-0.56175929	0.574280033	0.718527801
ENSG00000015676	347.1249723	0.083781733	0.107285958	0.780919834	0.434849646	0.601106126
ENSG00000016082	670.1502057	-0.116215271	0.085260091	-1.363067633	0.172861196	0.316581646
ENSG00000016391	64.64398317	-1.847516098	0.249990554	-7.390343631	1.46E-13	3.18E-12
ENSG00000016402	32.49106632	1.800495262	0.333198164	5.40367702	6.53E-08	7.09E-07
ENSG00000016864	345.1359722	0.109774616	0.117717596	0.93252513	0.351065206	0.522671567
ENSG00000017260	364.642131	0.219228168	0.102081723	2.147575109	0.031747525	0.084254199
ENSG00000017483	10.95075555	6.09397569	1.25367031	4.86090772	1.17E-06	1.02E-05
ENSG00000017797	441.4801101	0.136622562	0.095548082	1.429882826	0.152750652	0.289055498
ENSG00000018189	327.0490917	-0.228228554	0.109027103	-2.093319444	0.036320648	0.094323561
ENSG00000018408	686.9165609	-0.191586886	0.095802687	-1.999807011	0.045521107	0.113293164
ENSG00000018510	426.0808716	-0.090056506	0.097754435	-0.921252377	0.356918678	0.528196025
ENSG00000018610	122.5387934	0.385615363	0.160756272	2.398757802	0.016450792	0.049229713
ENSG00000018699	354.1750845	0.138125159	0.101535741	1.360359977	0.173716037	0.317691599
ENSG00000019144	526.1159707	0.282672785	0.095773772	2.951463425	0.00316272	0.012085617
ENSG00000019485	86.27703858	-0.616179181	0.206002447	-2.991125535	0.002779512	0.010812463
ENSG00000019549	142.4984735	-1.586114186	0.303437369	-5.227155084	1.72E-07	1.75E-06
ENSG00000019582	305.2092625	0.355606963	0.113633466	3.129421065	0.001751511	0.00730724
ENSG00000019991	7470.910417	-0.547315488	0.056796326	-9.636459357	5.61E-22	2.11E-20
ENSG00000019995	257.7516755	-0.406204064	0.124974079	-3.250306536	0.001152807	0.005084294
ENSG00000020129	381.5566479	0.18505888	0.107454322	1.722209743	0.085031531	0.185155466
ENSG00000020181	244.8355139	-0.092742369	0.122786604	-0.755313413	0.450060952	0.615051564
ENSG00000020256	815.8667596	-0.634447276	0.084655363	-7.494472371	6.66E-14	1.47E-12
ENSG00000020426	207.7276098	-0.8651479	0.133664913	-6.472513096	9.64E-11	1.52E-09
ENSG00000020577	250.8038837	-0.207613202	0.119610397	-1.735745438	0.082608856	0.181055399
ENSG00000020633	8.971664446	0.951310362	0.591789988	1.607513445	0.107941782	0.222651721
ENSG00000020922	150.6004521	-0.317987232	0.150921647	-2.106968998	0.035120267	0.091697842
ENSG00000021300	58.35025002	0.032615728	0.230701218	0.141376487	0.887572528	0.938340581
ENSG00000021574	187.079658	-0.275053499	0.166673251	-1.650255801	0.098890628	0.207889353
ENSG00000021762	121.6493891	-0.203403416	0.1687067	-1.205662941	0.22794748	0.386682938
ENSG00000021776	1097.703748	-0.172501675	0.076137076	-2.265672442	0.023471446	0.065769679
ENSG00000021826	174.8846605	-1.669576543	0.16229502	-10.28729373	8.04E-25	3.54E-23

ENSG00000022267	183.8885133	0.850253205	0.137123559	6.200635469	5.62E-10	8.11E-09
ENSG00000022277	171.3414726	0.166538711	0.140164609	1.188165203	0.23476833	0.395703231
ENSG00000022556	196.8520182	0.262652995	0.134442909	1.953639627	0.050743851	0.123236319
ENSG00000022567	166.2824263	0.182364935	0.146523415	1.244612922	0.213274068	0.368721334
ENSG00000022840	702.1155493	0.008562554	0.082379815	0.103939953	0.917217001	0.955230368
ENSG00000022976	177.907935	0.088721224	0.145756215	0.608695992	0.542725964	0.691942716
ENSG00000023041	131.3248863	0.008622412	0.165666052	0.052046945	0.958491288	0.977701483
ENSG00000023171	25.53552776	-0.943279654	0.370805081	-2.543869282	0.01096321	0.034873237
ENSG00000023191	514.6637684	0.686095293	0.10519115	6.522367063	6.92E-11	1.12E-09
ENSG00000023228	902.1339607	-0.138824742	0.075818507	-1.831013919	0.067098466	0.153907927
ENSG00000023287	412.8710449	0.031738683	0.103245314	0.307410394	0.758531017	0.854526041
ENSG00000023318	349.1623867	0.075806972	0.108073394	0.701439729	0.483028636	0.643570018
ENSG00000023330	568.9388611	0.227780879	0.086499818	2.633310506	0.0084557	0.028066452
ENSG00000023445	7.416287071	-0.495089163	0.706421137	-0.700841378	0.483402012	0.644001144
ENSG00000023516	745.2362401	0.256613383	0.078337193	3.275754104	0.001053803	0.004709757
ENSG00000023572	201.1557822	0.214551122	0.139656146	1.536281271	0.124469411	0.248708651
ENSG00000023608	77.32393252	-0.744762815	0.206865059	-3.600234942	0.00031793	0.001617581
ENSG00000023697	75.95467602	-0.510385501	0.206185127	-2.475374955	0.013309636	0.041162584
ENSG00000023734	1126.303883	0.164459592	0.074797709	2.198724975	0.027897484	0.075951239
ENSG00000023839	18.27116365	0.190824757	0.404729178	0.471487521	0.637292629	0.765956258
ENSG00000023892	71.93424178	-0.116195601	0.21261307	-0.546512029	0.584714021	0.726667865
ENSG00000023902	273.8384294	0.631119796	0.114612348	5.506560232	3.66E-08	4.15E-07
ENSG00000023909	134.4286566	-0.019204462	0.156736107	-0.122527364	0.902481376	0.947258155
ENSG00000024048	456.6038773	-0.093930977	0.09698782	-0.968482198	0.332803606	0.505081442
ENSG00000024422	121.0890944	5.404936271	0.359639454	15.02876341	4.76E-51	5.26E-49
ENSG00000024526	508.4987592	-0.138533407	0.09061005	-1.528896705	0.126290053	0.251238688
ENSG00000024862	16.88729005	0.090080435	0.430360843	0.209313734	0.834203331	0.902777673
ENSG00000025039	171.1578567	0.035561298	0.140033742	0.253948065	0.799535681	0.880801115
ENSG00000025156	168.1593976	-1.189796553	0.149779076	-7.943676673	1.96E-15	4.88E-14
ENSG00000025293	282.7214482	-0.225013873	0.122091981	-1.842986511	0.06533098	0.150717261
ENSG00000025434	55.26393178	-0.993120386	0.253224463	-3.921897492	8.79E-05	0.000512709
ENSG00000025770	186.885862	0.236456622	0.135881088	1.740173153	0.081828618	0.179784224
ENSG00000025772	428.9482652	0.47239599	0.097111251	4.86448261	1.15E-06	1.00E-05
ENSG00000025796	925.5993192	-0.08473143	0.07977483	-1.062132385	0.28817559	0.458177986
ENSG00000025800	630.8607211	-0.774457753	0.086810487	-8.921246525	4.61E-19	1.49E-17
ENSG00000026025	8157.032736	0.449217633	0.059860255	7.50443898	6.17E-14	1.37E-12
ENSG00000026103	51.40981426	0.251045404	0.260727865	0.962863726	0.335615915	0.507360822
ENSG00000026297	388.1210023	0.268298948	0.123162474	2.178414735	0.029375174	0.079078779
ENSG00000026559	163.4903361	-0.125189822	0.144321867	-0.86743488	0.385703777	0.554545339
ENSG00000026652	161.8305421	0.21709114	0.143877957	1.508856143	0.13133555	0.258711326
ENSG00000026950	50.81114745	0.805873616	0.264270273	3.049429689	0.002292763	0.009183473
ENSG00000027001	85.3714524	-0.282503374	0.194338262	-1.453668313	0.146038287	0.279482264
ENSG00000027075	199.34413	-5.64414014	0.327695932	-17.22371134	1.76E-66	2.72E-64
ENSG00000027697	54.98904433	0.159531167	0.2376058	0.671411081	0.501958687	0.660269097
ENSG00000027847	110.8029497	0.277066059	0.173388592	1.597948608	0.110054415	0.226179132
ENSG00000027869	107.0955911	0.190637558	0.172879462	1.102719525	0.270148985	0.437539812
ENSG00000028116	195.8153102	-0.170025791	0.137217284	-1.239098931	0.215308863	0.371553267
ENSG00000028203	234.9015979	0.001506132	0.122238171	0.012321291	0.99016928	0.994914727
ENSG00000028310	362.6272365	0.063000481	0.106395332	0.592135767	0.553759678	0.700924384
ENSG00000028528	269.8321472	-0.848375888	0.120818113	-7.021926322	2.19E-12	4.21E-11

ENSG00000028839	32.8330013	-0.027981311	0.311607295	-0.089796715	0.928448759	0.960943097
ENSG00000029153	152.7173468	1.029383761	0.148615695	6.926480827	4.31E-12	8.02E-11
ENSG00000029363	1628.884082	-0.001837044	0.065503818	-0.028044837	0.977626391	0.988629033
ENSG00000029364	330.1395229	0.013178963	0.108932493	0.120982849	0.90370462	0.948177575
ENSG00000029534	50.30681294	-2.830265638	0.322904659	-8.765019522	1.87E-18	5.82E-17
ENSG00000029639	129.2642655	0.142740589	0.163248462	0.874376315	0.381913352	0.551414026
ENSG00000029725	1216.37935	0.252292033	0.074760959	3.374649486	0.000739098	0.0034287
ENSG00000029993	477.0002648	-0.049438797	0.093984834	-0.526029518	0.598867698	0.737617016
ENSG00000030066	537.7477702	-0.108230767	0.092499674	-1.170066466	0.241974222	0.404692451
ENSG00000030110	61.25755684	0.081731005	0.237847856	0.343627253	0.731126627	0.834021092
ENSG00000030419	14.31647066	-4.246223633	0.810210418	-5.240889944	1.60E-07	1.63E-06
ENSG00000030582	664.5048739	0.032063469	0.097288384	0.329571404	0.741723833	0.842008184
ENSG00000031003	158.6318507	-0.442035332	0.145783695	-3.032131495	0.002428334	0.009635821
ENSG00000031081	52.39027822	-0.113307074	0.242260067	-0.467708425	0.639993116	0.767565619
ENSG00000031691	36.17106353	0.04319255	0.289765092	0.149060569	0.881505839	0.934740887
ENSG00000031698	848.8954802	-0.121734056	0.077286456	-1.575102069	0.115232887	0.234115759
ENSG00000031823	547.0452694	0.175647474	0.087664443	2.003634171	0.045109263	0.112428994
ENSG00000032219	554.9177941	0.126080514	0.087667318	1.43817008	0.150385802	0.285700482
ENSG00000032389	190.2692568	0.025039194	0.157122583	0.159360884	0.873384555	0.929849389
ENSG00000032444	314.2791828	-0.267081501	0.118136852	-2.260780587	0.023772847	0.066468389
ENSG00000032742	186.423583	-0.223239894	0.140610147	-1.587651379	0.112365193	0.229751655
ENSG00000033011	85.55334109	-0.18060556	0.190254884	-0.949282121	0.342477144	0.514411511
ENSG00000033030	344.0341958	-0.145672019	0.107271579	-1.357974033	0.174471924	0.318907337
ENSG00000033050	276.8293263	0.97861319	0.121876873	8.029523279	9.79E-16	2.51E-14
ENSG00000033100	346.2357965	0.657030233	0.106703282	6.157544722	7.39E-10	1.05E-08
ENSG00000033170	108.4832188	-0.445835587	0.17498399	-2.547865017	0.01083844	0.034523258
ENSG00000033178	537.2629334	0.273520613	0.0999014	2.737905717	0.00618318	0.021387621
ENSG00000033327	129.3625106	-0.175127032	0.165357227	-1.059083027	0.289561969	0.459809743
ENSG00000033627	464.6741444	0.283652607	0.095483956	2.970683429	0.002971379	0.011441601
ENSG00000033800	401.6009717	-0.098013465	0.105715669	-0.92714227	0.353852672	0.525319011
ENSG00000033867	798.4688805	-0.163722716	0.080636841	-2.030371149	0.042318827	0.107089275
ENSG00000034053	363.7136211	0.169191172	0.105217059	1.608020347	0.107830722	0.222520664
ENSG00000034152	251.7317957	0.729908351	0.119604959	6.102659587	1.04E-09	1.45E-08
ENSG00000034510	435.1271166	-0.259784813	0.095120768	-2.731105088	0.006312234	0.021764348
ENSG00000034533	36.33474166	-0.057332702	0.288990368	-0.198389662	0.842740205	0.908748678
ENSG00000034677	187.6637028	0.269894737	0.136427159	1.97830652	0.047894138	0.117946872
ENSG00000034693	70.297797	0.411266537	0.214484427	1.917465725	0.055178791	0.131841551
ENSG00000034713	237.1423514	-0.28826497	0.143926927	-2.002856424	0.045192701	0.112596999
ENSG00000034971	18.82163026	-0.207449396	0.412094258	-0.503402782	0.614681116	0.749255956
ENSG00000035115	126.4588686	-0.111935123	0.179288663	-0.624329061	0.532411502	0.683993449
ENSG00000035141	479.3187957	-0.025531565	0.091912955	-0.27777983	0.781181375	0.868878442
ENSG00000035403	2317.239163	-0.814842563	0.061073253	-13.34205265	1.32E-40	1.05E-38
ENSG00000035499	147.4575977	-0.124479087	0.155055252	-0.802804712	0.422087618	0.589721979
ENSG00000035681	518.6240534	0.242531789	0.090539234	2.678747955	0.0073898	0.024973678
ENSG00000035687	330.7796203	0.05341643	0.108536088	0.492153633	0.62261074	0.75505595
ENSG00000035862	1152.911162	0.762887655	0.078583081	9.708039467	2.79E-22	1.06E-20
ENSG00000035928	664.3017961	-0.079863524	0.084908141	-0.940587367	0.346916358	0.518915353
ENSG00000036054	183.2078762	-0.268326338	0.138935249	-1.931304983	0.053445348	0.128376675
ENSG00000036257	657.2712949	0.01991011	0.08251214	0.241299157	0.809323264	0.887406439
ENSG00000036530	18.31691143	-0.86688607	0.417619305	-2.075780646	0.037914243	0.097513442

ENSG00000036549	377.1336591	-0.074130735	0.100116443	-0.740445152	0.45902993	0.62242959
ENSG00000036672	29.39660885	-1.00926583	0.338834384	-2.978640534	0.002895302	0.01119161
ENSG00000037042	79.34600914	-0.098064807	0.205809637	-0.476483066	0.633730269	0.76313837
ENSG00000037241	77.90705972	0.587187086	0.201369947	2.915961863	0.003545938	0.013358132
ENSG00000037280	125.0817133	-0.099789045	0.159448155	-0.625840073	0.531419845	0.683156819
ENSG00000037474	885.6521446	0.071111427	0.074137837	0.959178607	0.337468776	0.509394358
ENSG00000037637	196.6927664	-0.156089374	0.129766676	-1.202846366	0.229035778	0.387861666
ENSG00000037749	118.2410829	-0.337901411	0.168257102	-2.008244566	0.044617309	0.111519456
ENSG00000037757	271.6438687	0.420302421	0.130137976	3.229667739	0.001239342	0.005416624
ENSG00000037897	82.41368696	0.142389375	0.199298002	0.714454606	0.474946116	0.635988668
ENSG00000037965	143.4011124	-0.15881021	0.151834258	-1.045944524	0.295586646	0.46600512
ENSG00000038002	36.70397947	0.476107982	0.293549946	1.621897698	0.104825255	0.217597215
ENSG00000038210	268.2539158	-0.973268434	0.121868507	-7.986217779	1.39E-15	3.53E-14
ENSG00000038219	1001.006198	-0.045541481	0.076717665	-0.593624446	0.552763327	0.700024022
ENSG00000038274	198.806654	0.014321571	0.130371207	0.109852255	0.912526548	0.953030514
ENSG00000038358	364.0494972	-0.595609567	0.108776667	-5.475526904	4.36E-08	4.87E-07
ENSG00000038382	812.9256999	-0.717003305	0.084115759	-8.524006812	1.54E-17	4.44E-16
ENSG00000038427	153.141501	-2.254090994	0.176549731	-12.76745639	2.49E-37	1.77E-35
ENSG00000038532	219.9269534	-0.974586483	0.129589462	-7.520568964	5.45E-14	1.22E-12
ENSG00000039123	820.427178	0.080456764	0.077664586	1.035951756	0.30022465	0.47062333
ENSG00000039319	458.6461872	-0.672173623	0.099111358	-6.782003963	1.19E-11	2.07E-10
ENSG00000039523	600.3230206	1.148784401	0.090700529	12.66568574	9.16E-37	6.31E-35
ENSG00000039560	514.9951623	0.349777199	0.089023498	3.929043528	8.53E-05	0.000499996
ENSG00000039650	288.9400197	-0.089964418	0.116219954	-0.774087534	0.438879029	0.604227552
ENSG00000040199	322.3673854	0.549138134	0.113443264	4.840641158	1.29E-06	1.12E-05
ENSG00000040275	192.1514827	-0.203989892	0.137855544	-1.479736582	0.138943559	0.269577372
ENSG00000040341	280.7844524	-0.831992108	0.114460626	-7.26880619	3.63E-13	7.54E-12
ENSG00000040487	203.0092657	0.276342763	0.130846718	2.111957935	0.034690057	0.090768141
ENSG00000040531	153.2922758	0.350123943	0.147810899	2.368728864	0.017849333	0.052641105
ENSG00000040608	40.74502006	0.176475751	0.27549671	0.64057299	0.521800153	0.675986973
ENSG00000040633	283.1551272	0.408042415	0.113109951	3.60748466	0.00030918	0.00158044
ENSG00000040731	97.4167871	5.65704701	0.4336341	13.04566918	6.73E-39	5.05E-37
ENSG00000040933	238.9094173	-0.025894288	0.123247336	-0.210100183	0.833589482	0.902460999
ENSG00000041357	1098.254315	0.188628151	0.079646783	2.368308474	0.01786963	0.052666568
ENSG00000041802	219.9291308	0.336330128	0.126076187	2.667673703	0.00763784	0.025716379
ENSG00000041880	98.54590989	0.094777344	0.183224746	0.517273707	0.604965106	0.742113775
ENSG00000041988	43.28383503	-0.388124411	0.268500654	-1.445525013	0.148310472	0.282483689
ENSG00000042062	6.506801409	-1.389859012	0.721146996	-1.92728947	0.053943565	0.12941849
ENSG00000042088	251.5171827	-0.002380805	0.116952186	-0.02035708	0.983758522	0.991961125
ENSG00000042286	17.2289231	-1.296374399	0.445638697	-2.909025648	0.003625571	0.01360469
ENSG00000042317	20.2245769	-0.656299729	0.396427779	-1.655534156	0.097816194	0.206114597
ENSG00000042429	157.5955535	0.505411751	0.158240772	3.193941389	0.001403446	0.006032499
ENSG00000042445	105.3305476	-0.371412229	0.175396563	-2.117557057	0.034212598	0.089795286
ENSG00000042493	10.19181321	-2.549324842	0.671649021	-3.795620575	0.000147275	0.000814475
ENSG00000042753	548.3107494	-0.013399662	0.102758518	-0.130399523	0.896250344	0.943750269
ENSG00000043093	114.247659	-0.359250026	0.168785981	-2.128435223	0.033301015	0.08774702
ENSG00000043143	608.7358888	0.436562507	0.084477623	5.167788719	2.37E-07	2.36E-06
ENSG00000043355	47.92042091	-1.168856613	0.267575044	-4.368331945	1.25E-05	8.78E-05
ENSG00000043514	236.7820417	0.003949208	0.122034007	0.032361536	0.974183736	0.986686779
ENSG00000043591	7.644623827	-3.785428885	0.992092168	-3.815602024	0.000135851	0.000759071

ENSG00000044090	408.1332099	0.19303999	0.098608032	1.957649763	0.050271124	0.122342078
ENSG00000044115	1540.408935	0.290166988	0.063985655	4.534875603	5.76E-06	4.36E-05
ENSG00000044446	351.1182005	0.127449515	0.105187135	1.211645468	0.225648122	0.384017345
ENSG00000044459	344.6221021	-0.249092155	0.106348713	-2.342220671	0.019169377	0.055773041
ENSG00000044524	84.79274632	3.74428343	0.280285109	13.35883821	1.05E-40	8.44E-39
ENSG00000044574	3212.98136	0.418109095	0.063403966	6.594368218	4.27E-11	7.09E-10
ENSG00000046604	51.15290069	0.188563974	0.260281326	0.724462169	0.468782045	0.630926644
ENSG00000046647	50.05185264	-0.070997977	0.248179472	-0.286075137	0.774820574	0.865106478
ENSG00000046651	237.2201978	0.265708965	0.125666369	2.114399956	0.034481121	0.090331278
ENSG00000046653	11.67546023	-1.98919069	0.583515089	-3.408979011	0.000652065	0.003066474
ENSG00000047056	190.4711265	0.181602226	0.133145139	1.363941835	0.172585873	0.316136399
ENSG00000047188	620.6425972	-0.098167233	0.088595497	-1.108038635	0.267845131	0.434710636
ENSG00000047230	181.9366356	0.106284913	0.137487758	0.773050015	0.439492781	0.604795228
ENSG00000047249	166.8917322	-0.187225216	0.152142354	-1.230592344	0.21847537	0.375499801
ENSG00000047315	771.0800038	0.052940598	0.086282062	0.613575949	0.539495581	0.689580557
ENSG00000047346	155.4172723	0.075839569	0.146271677	0.518484307	0.604120407	0.741660492
ENSG00000047365	163.3329182	-0.069835012	0.148988642	-0.468727084	0.639264725	0.767106748
ENSG00000047410	4382.835611	0.185538251	0.059223821	3.132831476	0.001731288	0.007233612
ENSG00000047578	147.6012041	-0.084211871	0.155422045	-0.541827072	0.58793764	0.729572441
ENSG00000047579	193.0253668	0.549542075	0.136759715	4.018303755	5.86E-05	0.000354462
ENSG00000047597	40.25912264	0.02950048	0.280788839	0.105062865	0.916325926	0.954797884
ENSG00000047621	84.67903576	-0.142594773	0.196063237	-0.727289699	0.467048507	0.629196354
ENSG00000047644	258.3739712	0.367973127	0.119622328	3.076124098	0.002097105	0.008494245
ENSG00000047849	2410.089734	0.117647667	0.064828635	1.814748467	0.069562573	0.158264883
ENSG00000047932	351.5460238	0.091630705	0.109500158	0.836808888	0.402700001	0.570982831
ENSG00000048028	236.0887733	0.066329056	0.125067578	0.530345736	0.595872242	0.735455354
ENSG00000048052	715.7346445	0.262360462	0.088584497	2.961697246	0.003059485	0.011742384
ENSG00000048140	175.2482555	0.247605491	0.143461554	1.725936213	0.084358889	0.184090308
ENSG00000048162	279.9025441	0.303146147	0.117158149	2.587495182	0.009667655	0.031370189
ENSG00000048342	351.920247	-0.238940736	0.111511266	-2.142749743	0.032133199	0.085052769
ENSG00000048392	299.7334769	-0.404962183	0.10996444	-3.682664886	0.000230808	0.001215661
ENSG00000048405	358.82254	0.769962134	0.104668054	7.356228614	1.89E-13	4.05E-12
ENSG00000048471	95.7292828	-0.695460729	0.183197148	-3.796242121	0.000146906	0.000813178
ENSG00000048540	36.63177442	1.801798431	0.31232747	5.768939987	7.98E-09	9.94E-08
ENSG00000048544	157.4821553	0.527614337	0.15101527	3.493781377	0.000476231	0.002319561
ENSG00000048649	1240.093449	-0.117674669	0.071081718	-1.6554843	0.097826299	0.206114597
ENSG00000048707	618.2155169	-0.120955047	0.08663194	-1.396194602	0.162655902	0.302786569
ENSG00000048828	1592.166103	0.376772	0.063176492	5.963800625	2.46E-09	3.27E-08
ENSG00000048991	1243.601663	0.04099927	0.077937734	0.526051596	0.598852359	0.737617016
ENSG00000049089	19.29316452	0.22773434	0.412763182	0.551731236	0.581132506	0.723664022
ENSG00000049167	180.9145206	-0.40567815	0.138434259	-2.930475113	0.003384441	0.012817806
ENSG00000049192	91.32416512	-0.889013803	0.200118805	-4.442430106	8.89E-06	6.45E-05
ENSG00000049239	466.3053441	-0.462018088	0.097717794	-4.72808551	2.27E-06	1.87E-05
ENSG00000049245	231.0977095	0.121144144	0.123046083	0.984542867	0.324848665	0.49685858
ENSG00000049246	235.6287237	0.317069812	0.124746295	2.541717275	0.011030936	0.035048984
ENSG00000049323	88.15576878	-0.853215036	0.208984741	-4.08266666	4.45E-05	0.000277093
ENSG00000049449	495.9705985	0.060139346	0.102853277	0.58471006	0.558742689	0.705687696
ENSG00000049541	492.1600129	-0.045954232	0.09166347	-0.501336374	0.61613441	0.750506422
ENSG00000049618	735.0264107	-0.280382084	0.088678928	-3.16176674	0.001568151	0.006628896
ENSG00000049656	714.855092	0.452908704	0.081539609	5.554462565	2.78E-08	3.20E-07

ENSG00000049759	246.8657851	0.170451075	0.121023706	1.408410633	0.159009512	0.297536515
ENSG00000049768	12.42916949	0.176670882	0.48634211	0.363264621	0.716407219	0.823449162
ENSG00000049769	30.95634287	-0.868346804	0.33104084	-2.623080598	0.008713866	0.028733256
ENSG00000049860	1061.520516	0.574508989	0.07873062	7.297148078	2.94E-13	6.17E-12
ENSG00000049883	150.5806404	-0.035200237	0.147256553	-0.23904021	0.811074404	0.888702196
ENSG00000050030	21.02645142	-3.411294982	0.552487385	-6.174430535	6.64E-10	9.50E-09
ENSG00000050130	112.929225	-0.059423412	0.168821127	-0.351990375	0.724845481	0.829602619
ENSG00000050165	46.31577795	-0.190549675	0.268165091	-0.710568531	0.47735165	0.637876581
ENSG00000050344	69.81048535	0.266040857	0.209991559	1.266912151	0.205186703	0.358682061
ENSG00000050393	220.3704235	-0.306692986	0.127790644	-2.39996432	0.01639667	0.049078207
ENSG00000050405	382.504054	-0.467500558	0.104428881	-4.47673626	7.58E-06	5.58E-05
ENSG00000050426	147.5146972	0.459689545	0.149173599	3.081574411	0.00205909	0.008366803
ENSG00000050438	83.18278482	-0.465242947	0.19848078	-2.344020145	0.019077141	0.055527683
ENSG00000050555	238.5135708	0.626766699	0.147547311	4.24790323	2.16E-05	0.000143925
ENSG00000050748	233.3568602	-0.205233907	0.128177074	-1.601174856	0.109338196	0.224970506
ENSG00000050767	73.00383508	-1.468686334	0.225046023	-6.526159924	6.75E-11	1.10E-09
ENSG00000050820	360.4126948	0.365969766	0.114690907	3.190922229	0.001418194	0.006086581
ENSG00000051009	123.845509	-0.177521551	0.181423	-0.978495289	0.32782942	0.500219407
ENSG00000051108	342.6000865	0.136443618	0.11169435	1.221580303	0.221866381	0.379790038
ENSG00000051128	73.34470989	0.28500968	0.222426457	1.281365909	0.200065173	0.352125733
ENSG00000051180	110.3550442	-0.217104661	0.176480805	-1.23018852	0.218626518	0.375663204
ENSG00000051341	433.1319945	-0.600559819	0.102316971	-5.869601219	4.37E-09	5.60E-08
ENSG00000051382	226.1811777	-0.283177011	0.123473274	-2.293427568	0.021823401	0.061967519
ENSG00000051523	19.19312325	0.861682221	0.442414054	1.94768275	0.051452935	0.124635176
ENSG00000051596	139.8619056	0.590860863	0.163038616	3.624054695	0.00029002	0.001494466
ENSG00000051620	104.2135269	0.214191611	0.175898208	1.217702065	0.223337212	0.381703105
ENSG00000051825	285.6512615	0.113433706	0.116044052	0.97750556	0.328318927	0.500640031
ENSG00000052126	1033.438003	-0.179744639	0.075376682	-2.384618603	0.017096836	0.050859648
ENSG00000052723	343.705215	-0.161679413	0.11333216	-1.426597818	0.153695853	0.290346682
ENSG00000052749	462.0341324	0.277610454	0.103556741	2.680756954	0.007345585	0.024839534
ENSG00000052795	217.6122787	0.243861938	0.125874733	1.937338275	0.052704006	0.127074009
ENSG00000052802	403.7771541	0.370709216	0.110003767	3.36996837	0.000751768	0.003480582
ENSG00000052841	768.0311554	-0.245543126	0.080164808	-3.06297904	0.002191454	0.008818281
ENSG00000053254	598.7526773	-0.089648319	0.089825874	-0.998023339	0.318268042	0.48997802
ENSG00000053328	28.91558005	-1.978864455	0.371570283	-5.325680075	1.01E-07	1.06E-06
ENSG00000053371	412.4293952	0.324427201	0.121803379	2.66353202	0.007732505	0.025985287
ENSG00000053372	521.295838	0.21373377	0.092698979	2.305675566	0.021128767	0.060422597
ENSG00000053501	151.5340225	0.762552144	0.159684247	4.775374886	1.79E-06	1.50E-05
ENSG00000053524	19.36725296	0.484400296	0.39726848	1.219327282	0.222719998	0.380926313
ENSG00000053770	174.6147247	-0.290394011	0.144525549	-2.009291866	0.04450619	0.111281319
ENSG00000053900	172.2433309	-0.20578977	0.141560727	-1.453720776	0.146023736	0.279482264
ENSG00000054116	290.6964425	-0.328858464	0.113049437	-2.908979236	0.003626109	0.01360469
ENSG00000054118	2654.734731	0.129281403	0.05929013	2.180487757	0.029221325	0.078781864
ENSG00000054148	307.4261893	-0.030329424	0.117442614	-0.258248888	0.79621483	0.878366283
ENSG00000054179	192.648462	0.891031578	0.145225678	6.135496062	8.49E-10	1.19E-08
ENSG00000054267	788.967232	0.037201734	0.081642457	0.455666518	0.648629802	0.7738494
ENSG00000054277	45.59194943	0.141748719	0.259541845	0.54614977	0.584962991	0.726908628
ENSG00000054282	175.7897303	0.052881903	0.138646838	0.381414417	0.702895765	0.814114183
ENSG00000054523	934.7275507	0.085494525	0.077075802	1.109226541	0.267332466	0.434229781
ENSG00000054598	366.9324241	-1.092362438	0.107015032	-10.20756073	1.83E-24	7.86E-23

ENSG00000054611	147.2284964	-0.079570151	0.15224129	-0.522658152	0.601212165	0.739437208
ENSG00000054654	443.1293895	-0.599460166	0.097852566	-6.126156825	9.00E-10	1.26E-08
ENSG00000054690	154.1764632	-0.48158622	0.149520653	-3.22086755	0.001278032	0.005559713
ENSG00000054793	407.4455755	-0.783806757	0.113356176	-6.914548344	4.69E-12	8.63E-11
ENSG00000054965	669.1645444	0.820288574	0.082685248	9.920615729	3.39E-23	1.35E-21
ENSG00000054967	73.62481613	0.744836703	0.223272111	3.336004204	0.000849919	0.003881277
ENSG00000054983	183.5888881	-0.161244202	0.137447654	-1.173131711	0.240742992	0.403257429
ENSG00000055044	1290.73409	0.20852118	0.067400356	3.093769708	0.001976308	0.008096005
ENSG00000055070	1112.024845	0.102416817	0.076746499	1.334481949	0.182045959	0.328815685
ENSG00000055118	234.3104211	0.226149212	0.132189781	1.710791942	0.087119528	0.188470422
ENSG00000055130	820.6290098	0.523111443	0.086348999	6.058106656	1.38E-09	1.89E-08
ENSG00000055147	102.4482684	0.336360421	0.189092942	1.778810024	0.075270913	0.16793997
ENSG00000055163	400.4994489	0.155832992	0.103618504	1.503910847	0.132604324	0.260735293
ENSG00000055208	555.4079661	0.010447816	0.094354003	0.110729967	0.911830482	0.952735327
ENSG00000055211	136.9144869	0.213525485	0.155183107	1.375958309	0.168834541	0.311358645
ENSG00000055332	1881.592466	0.28479885	0.070241664	4.054557292	5.02E-05	0.000308919
ENSG00000055483	1142.840482	0.244544188	0.070115545	3.487731412	0.000487137	0.002366119
ENSG00000055609	2399.454419	0.442403762	0.069743463	6.343300783	2.25E-10	3.41E-09
ENSG00000055732	109.9972771	-1.830091751	0.198312139	-9.228339532	2.75E-20	9.46E-19
ENSG00000055917	1348.694872	-0.070979745	0.068850779	-1.030921449	0.302577658	0.47320289
ENSG00000055950	202.6883072	0.267146716	0.128398683	2.080603245	0.037470237	0.096576957
ENSG00000056050	111.5146646	0.133887154	0.173835279	0.770195522	0.44118392	0.606449801
ENSG00000056097	1110.235307	0.06820799	0.072372265	0.942460344	0.345957005	0.517756025
ENSG00000056277	255.4564318	0.150208549	0.125412113	1.19771963	0.231026185	0.390477983
ENSG00000056487	114.721223	-0.653952611	0.177738473	-3.67929689	0.000233878	0.001229062
ENSG00000056558	29.49249024	-0.261572373	0.323693082	-0.808087622	0.419040143	0.58692361
ENSG00000056586	628.9177548	0.000430055	0.092387796	0.004654885	0.996285953	0.998279095
ENSG00000056736	23.57921787	-1.870427016	0.412007924	-4.539784083	5.63E-06	4.27E-05
ENSG00000056972	215.6861363	0.500676913	0.128230468	3.904508193	9.44E-05	0.00054661
ENSG00000056998	133.6881001	-1.713097888	0.171189878	-10.00700454	1.42E-23	5.76E-22
ENSG00000057019	354.7059406	-0.566358714	0.106489797	-5.318431716	1.05E-07	1.10E-06
ENSG00000057252	419.3269918	-0.03330666	0.097841495	-0.340414463	0.733544429	0.835693316
ENSG00000057294	37.42005864	-0.034843067	0.288376876	-0.120824761	0.903829838	0.948177575
ENSG00000057468	14.32581345	0.544231713	0.456414511	1.192406682	0.233101822	0.393279993
ENSG00000057608	999.5671154	0.036624351	0.075233737	0.486807548	0.626394721	0.757486798
ENSG00000057663	102.2011341	0.051875434	0.179032376	0.289754489	0.772004068	0.86337203
ENSG00000057757	209.8903916	-0.101123374	0.128724093	-0.785582336	0.432112216	0.598430475
ENSG00000057935	846.0031726	-0.028302808	0.075499218	-0.374875513	0.707753051	0.816776792
ENSG00000058056	151.8901348	-0.213245394	0.148822567	-1.43288346	0.151891145	0.288053856
ENSG00000058063	212.6726392	-0.349952571	0.128495103	-2.723470089	0.006460006	0.022224764
ENSG00000058262	1275.732974	0.248341257	0.06955086	3.570642526	0.000356107	0.00179163
ENSG00000058272	1028.909948	-0.04875121	0.079631545	-0.612209777	0.540398974	0.690340523
ENSG00000058453	473.4794059	0.492950124	0.104042683	4.7379605	2.16E-06	1.79E-05
ENSG00000058600	514.60134	0.31088948	0.094238162	3.298976479	0.00097038	0.004369655
ENSG00000058668	350.5410477	-0.669507823	0.104183043	-6.426264832	1.31E-10	2.03E-09
ENSG00000058673	15.65320191	0.26373482	0.443120823	0.595175866	0.551725921	0.699340733
ENSG00000058729	169.2686108	0.322047849	0.142125132	2.26594582	0.023454701	0.065738606
ENSG00000058799	49.35810811	0.17327076	0.249855418	0.693484101	0.4880058	0.647922265
ENSG00000058804	384.7476881	0.030660806	0.098528005	0.311188744	0.755657139	0.852102835
ENSG00000059122	151.338631	-0.022355682	0.148100038	-0.150949876	0.880015256	0.934004754

ENSG00000059145	75.37474712	0.669790274	0.212562443	3.151028309	0.001626967	0.006854832
ENSG00000059573	762.8443225	0.051629205	0.078916533	0.65422545	0.512966568	0.668923262
ENSG00000059588	291.6548378	-0.242421845	0.120685754	-2.008703069	0.044568633	0.111417617
ENSG00000059691	182.3725414	0.052723734	0.136480853	0.386308648	0.699268096	0.811250326
ENSG00000059728	150.2446423	0.382344205	0.152734138	2.503331676	0.012303019	0.038481519
ENSG00000059758	153.7173258	-0.222905453	0.148786179	-1.498159675	0.13409177	0.262667283
ENSG00000059769	44.91865893	0.150385077	0.2675229	0.562139081	0.574021263	0.718419883
ENSG00000059804	353.1228511	-0.621857822	0.109929201	-5.65689387	1.54E-08	1.84E-07
ENSG00000059915	60.36631195	0.693278568	0.226953189	3.054720547	0.002252703	0.009051408
ENSG00000060069	201.2018825	0.383349772	0.134846117	2.842868446	0.004470952	0.016282983
ENSG00000060138	1317.347145	-0.447596039	0.087353724	-5.12394911	2.99E-07	2.92E-06
ENSG00000060237	2992.952798	-0.246846522	0.060356791	-4.089788705	4.32E-05	0.000269317
ENSG00000060339	1394.769231	0.109948858	0.073826967	1.48927774	0.136414247	0.265918808
ENSG00000060491	397.111256	0.412078394	0.102035288	4.038587073	5.38E-05	0.000327565
ENSG00000060642	59.15288115	0.024657756	0.27168939	0.090757154	0.927685556	0.960604431
ENSG00000060656	84.32696016	-1.710048038	0.219543737	-7.789099623	6.75E-15	1.60E-13
ENSG00000060688	736.1809845	0.157898976	0.07909772	1.996251938	0.045906507	0.113991285
ENSG00000060718	2424.576655	-4.804218168	0.092226	-52.09179826	0	0
ENSG00000060749	1287.552576	0.034757627	0.076040823	0.457091678	0.647605152	0.773169561
ENSG00000060762	51.19700059	-0.228490703	0.252131567	-0.906236002	0.364810951	0.535979441
ENSG00000060971	184.4684222	0.536164501	0.135791771	3.948431472	7.87E-05	0.000463704
ENSG00000060982	902.0989815	0.008271893	0.076829745	0.10766524	0.914261244	0.953636867
ENSG00000061273	220.7970281	-0.170746562	0.125994251	-1.355193276	0.175355986	0.31987728
ENSG00000061656	45.79176028	-0.122664157	0.255262378	-0.480541462	0.630842428	0.760980193
ENSG00000061676	980.6880364	0.20844851	0.0715669	2.912638523	0.003583892	0.013482306
ENSG00000061794	299.4354287	0.271413481	0.113927583	2.382333363	0.017203316	0.051111459
ENSG00000061918	11.54305344	-3.523694791	0.782206339	-4.504814929	6.64E-06	4.94E-05
ENSG00000061936	503.1795859	0.239053123	0.090931094	2.628948049	0.008564944	0.02836204
ENSG00000061938	230.9043142	0.02555489	0.139498022	0.183191772	0.85464755	0.916518175
ENSG00000061987	465.8107261	-0.020205451	0.092440904	-0.218576947	0.826979612	0.898668717
ENSG00000062038	14.34085566	-0.9048292	0.502744941	-1.799777831	0.071895726	0.162219309
ENSG00000062194	343.275341	-0.175086738	0.108790973	-1.609386638	0.107531825	0.222066973
ENSG00000062282	36.38462424	0.139363128	0.290480119	0.479768213	0.631392218	0.761420933
ENSG00000062370	33.33815569	-0.206900271	0.323484162	-0.639599386	0.522433082	0.676307944
ENSG00000062485	883.1131339	-0.044955612	0.095969499	-0.468436457	0.639472502	0.767224995
ENSG00000062582	10.94251307	1.298821655	0.552644987	2.350191689	0.018763745	0.054809174
ENSG00000062598	198.9880445	-0.57488939	0.133595452	-4.303210785	1.68E-05	0.000114861
ENSG00000062650	894.2533169	-0.13492244	0.074601293	-1.808580444	0.070516209	0.159945291
ENSG00000062716	1164.16772	-0.516609549	0.079604076	-6.489737372	8.60E-11	1.38E-09
ENSG00000062725	297.5099771	-0.033410085	0.109429499	-0.305311509	0.760128911	0.85522874
ENSG00000062822	393.0136603	0.088974241	0.102699073	0.866358754	0.386293456	0.555102336
ENSG00000063046	3134.501946	-0.139344016	0.065408047	-2.130380312	0.033140228	0.087388921
ENSG00000063127	82.61148694	-0.227445038	0.200594015	-1.133857544	0.256854309	0.422535821
ENSG00000063169	291.8804619	-0.008485165	0.114181269	-0.074313106	0.940761249	0.967262249
ENSG00000063176	108.282197	0.675127651	0.17915369	3.768427265	0.000164279	0.000897562
ENSG00000063177	3839.311627	0.169281948	0.063063043	2.684328902	0.007267557	0.024640931
ENSG00000063180	852.9986671	0.282548582	0.091580536	3.085247093	0.002033831	0.008285738
ENSG00000063241	183.6510986	-0.767590348	0.139463782	-5.503868718	3.72E-08	4.21E-07
ENSG00000063244	1816.121334	0.189623627	0.075364127	2.516099306	0.011866174	0.037281179
ENSG00000063245	629.5178245	-0.019134335	0.093073807	-0.205582384	0.83711714	0.905024587

ENSG00000063322	195.6356042	-0.276785973	0.132917163	-2.082394521	0.037306449	0.096296156
ENSG00000063587	175.4717477	0.289975152	0.139508966	2.078541335	0.037659528	0.097011441
ENSG00000063601	249.4632634	0.314295525	0.119397622	2.632343256	0.008479814	0.028137
ENSG00000063660	375.2477478	0.162119497	0.120828258	1.341734947	0.179681952	0.325676436
ENSG00000063761	51.07291866	-0.064685467	0.242461237	-0.266786839	0.789633288	0.874348005
ENSG00000063854	146.6762519	0.157179903	0.15369991	1.022641477	0.306477391	0.47752037
ENSG00000063978	545.7196095	0.089761921	0.089424653	1.003771536	0.315488747	0.486941084
ENSG00000064012	192.1040522	-0.246630085	0.133296383	-1.850238397	0.064279197	0.148826418
ENSG00000064102	409.3850729	0.438940855	0.097286534	4.51183566	6.43E-06	4.80E-05
ENSG00000064115	456.6005567	0.123949629	0.09306328	1.331885455	0.182897831	0.329996851
ENSG00000064199	90.78082356	-0.653398875	0.204290025	-3.198388546	0.001381979	0.00596006
ENSG00000064225	21.09956696	0.113187455	0.376142494	0.300916427	0.763478224	0.857280464
ENSG00000064309	146.460546	0.044578316	0.153803023	0.289840308	0.771938409	0.86337203
ENSG00000064313	734.3171821	0.089373256	0.085784071	1.041839769	0.297485973	0.468106358
ENSG00000064393	407.8514772	-0.224190343	0.09615709	-2.331500904	0.019726962	0.057111426
ENSG00000064419	913.5607733	0.479052502	0.074861893	6.399150269	1.56E-10	2.41E-09
ENSG00000064490	182.8072677	-0.435940865	0.139439548	-3.126378925	0.001769734	0.007359221
ENSG00000064545	245.1418441	0.318302645	0.132817557	2.396540428	0.016550668	0.049465368
ENSG00000064547	23.47962526	-0.325225474	0.375193024	-0.866821751	0.386039682	0.554865386
ENSG00000064601	805.5643457	0.922138273	0.080722237	11.42359661	3.19E-30	1.74E-28
ENSG00000064607	1136.867417	-0.057831751	0.078995778	-0.732086609	0.464115707	0.627026563
ENSG00000064651	361.7272958	-0.059106857	0.116078134	-0.509198892	0.61061282	0.746810014
ENSG00000064652	55.23354016	-0.337256182	0.256601795	-1.314317316	0.188739442	0.337692389
ENSG00000064666	707.8552805	-1.013609957	0.084975555	-11.92825351	8.43E-33	5.02E-31
ENSG00000064687	475.6663363	-0.058014878	0.108087847	-0.536738212	0.591448457	0.732133279
ENSG00000064703	216.8701116	-0.10082464	0.127269225	-0.792215397	0.428235092	0.59540912
ENSG00000064726	822.9916469	0.190881955	0.079793455	2.392200645	0.016747684	0.049969141
ENSG00000064763	62.64352669	4.4161414	0.38032421	11.61151797	3.60E-31	2.06E-29
ENSG00000064835	18.87243745	-7.518352965	1.220803559	-6.158528054	7.34E-10	1.04E-08
ENSG00000064932	504.3694507	0.992034564	0.101265866	9.79633713	1.17E-22	4.52E-21
ENSG00000064933	254.8190681	-0.21089617	0.127316685	-1.65646922	0.097626833	0.205786934
ENSG00000064961	597.4425077	0.219467764	0.086094841	2.549139543	0.010798908	0.034412945
ENSG00000064989	72.3933081	-2.200386175	0.265368239	-8.291821906	1.12E-16	3.01E-15
ENSG00000064995	423.4919285	-0.452164589	0.095896808	-4.715116147	2.42E-06	1.98E-05
ENSG00000064999	345.3174327	0.107273505	0.108836467	0.985639358	0.324310117	0.496196952
ENSG00000065000	2088.037161	0.091508161	0.061111102	1.497406491	0.13428752	0.262904084
ENSG00000065029	238.8591571	0.113738293	0.120090988	0.947100983	0.343587324	0.515472727
ENSG00000065054	315.4523343	1.292979363	0.114742062	11.26857352	1.88E-29	9.98E-28
ENSG00000065057	132.4881479	0.28905137	0.168891756	1.711459322	0.086996354	0.188353543
ENSG00000065060	522.0819188	-0.466681041	0.097023153	-4.809996643	1.51E-06	1.29E-05
ENSG00000065135	217.792421	0.219265879	0.132075295	1.660158165	0.096882638	0.204501687
ENSG00000065150	1634.245399	-0.255944707	0.087394616	-2.928609556	0.003404818	0.012884559
ENSG00000065154	212.8454146	0.030226283	0.128662653	0.234926624	0.814265685	0.890878579
ENSG00000065183	653.8280332	0.377695024	0.089132372	4.237461814	2.26E-05	0.000150354
ENSG00000065243	679.120782	-0.117637288	0.086655867	-1.357522488	0.174615253	0.319003831
ENSG00000065268	309.3565329	0.16645404	0.112264289	1.482698034	0.138154657	0.268269072
ENSG00000065308	468.6961381	-0.022019615	0.092100283	-0.239083028	0.811041202	0.888702196
ENSG00000065328	445.8760464	-0.10253096	0.094124068	-1.08931714	0.276014057	0.444476608
ENSG00000065357	104.5180204	-0.422493733	0.17573942	-2.404091997	0.016212693	0.048631779
ENSG00000065361	42.24597241	-1.512452185	0.295062607	-5.125868717	2.96E-07	2.89E-06

ENSG00000065413	14.45380806	0.873883384	0.465889962	1.875729154	0.06069248	0.14220972
ENSG00000065427	719.0719655	0.382268583	0.08331598	4.588178448	4.47E-06	3.46E-05
ENSG00000065457	156.6912424	-0.072816157	0.147551088	-0.493497934	0.621660799	0.754639706
ENSG00000065485	234.8048489	0.631039714	0.128884372	4.896169356	9.77E-07	8.66E-06
ENSG00000065491	138.8617631	-0.014435247	0.156838528	-0.09203891	0.926667125	0.959877081
ENSG00000065518	578.3692933	0.147905738	0.085958263	1.72066923	0.085310863	0.185619841
ENSG00000065526	2844.465213	-0.015264463	0.059158076	-0.258028391	0.796384996	0.878387645
ENSG00000065534	34.21113174	-1.591699334	0.322642598	-4.933320475	8.08E-07	7.32E-06
ENSG00000065548	1214.917654	0.230030979	0.072676768	3.165123949	0.001550169	0.006568695
ENSG00000065559	199.8918951	0.119838159	0.131188181	0.913482891	0.360988628	0.532109326
ENSG00000065600	160.656579	0.142680418	0.144321426	0.988629494	0.32284445	0.494547
ENSG00000065613	1110.32937	0.322448491	0.071448921	4.512993173	6.39E-06	4.78E-05
ENSG00000065615	73.59128286	-0.195664693	0.209987174	-0.931793544	0.351443231	0.523068772
ENSG00000065621	35.22770653	-2.265514925	0.347338117	-6.522505915	6.91E-11	1.12E-09
ENSG00000065665	62.9123764	0.006440035	0.231058481	0.027871883	0.977764334	0.988629033
ENSG00000065717	125.1395551	-1.319215974	0.171071852	-7.711473013	1.24E-14	2.92E-13
ENSG00000065802	266.2984837	-0.063497778	0.126311267	-0.502708738	0.615169065	0.74963078
ENSG00000065809	93.28094206	0.029997305	0.198736651	0.150939975	0.880023066	0.934004754
ENSG00000065833	147.7780858	0.597411051	0.154267544	3.872564729	0.000107696	0.000615979
ENSG00000065882	412.5187774	0.167872254	0.096695964	1.736083359	0.082549096	0.180984416
ENSG00000065883	729.6437424	-0.053156187	0.079362072	-0.669793343	0.502989538	0.660882428
ENSG00000065911	678.3649024	-0.599437813	0.083904019	-7.144327738	9.04E-13	1.80E-11
ENSG00000065923	163.6517237	-1.830091249	0.160820324	-11.37972618	5.28E-30	2.87E-28
ENSG00000065970	366.7394643	0.146307601	0.104873274	1.395089476	0.162988859	0.30328508
ENSG00000065978	3950.896223	-0.848874451	0.060521303	-14.02604378	1.08E-44	9.48E-43
ENSG00000065989	548.2005234	-0.498913436	0.092108509	-5.416583535	6.07E-08	6.64E-07
ENSG00000066027	256.5264885	0.217065429	0.118894922	1.825691338	0.067896769	0.15548515
ENSG00000066044	854.9662029	-0.104688371	0.080368545	-1.302603778	0.192710067	0.343266335
ENSG00000066084	494.1723007	0.078548517	0.090103812	0.871755759	0.383341635	0.552575821
ENSG00000066117	637.4146605	0.104995153	0.083681785	1.254695421	0.209589349	0.364504343
ENSG00000066135	354.9094086	-0.236554823	0.111243415	-2.12646135	0.033464864	0.088112642
ENSG00000066136	469.3124566	0.230920252	0.094987356	2.431063055	0.015054594	0.045674274
ENSG00000066230	94.00568166	-3.130521875	0.250770138	-12.48363101	9.17E-36	6.08E-34
ENSG00000066248	31.49992703	-1.198360424	0.328934705	-3.643155942	0.000269316	0.001395454
ENSG00000066279	2098.692272	-0.154045676	0.06409192	-2.403511636	0.01623845	0.048687622
ENSG00000066322	255.0662374	0.506648837	0.11895677	4.25910047	2.05E-05	0.000137687
ENSG00000066379	73.20312199	0.353318881	0.208117086	1.697692814	0.089565755	0.19261025
ENSG00000066382	68.53692671	-5.981018495	0.618137434	-9.675871684	3.82E-22	1.44E-20
ENSG00000066422	441.547766	0.050388685	0.096576045	0.521751385	0.601843442	0.739964537
ENSG00000066427	183.0257404	0.062356086	0.13642195	0.457082501	0.647611748	0.773169561
ENSG00000066455	309.1766324	0.361758306	0.11069215	3.268147805	0.001082538	0.00482064
ENSG00000066557	111.1356951	0.062247893	0.167728603	0.371122707	0.710546139	0.818722141
ENSG00000066583	113.1872013	-0.293955236	0.172971307	-1.699445078	0.089235355	0.192046733
ENSG00000066629	195.6353262	0.010467224	0.130424317	0.080255156	0.936034324	0.964881635
ENSG00000066651	226.3491001	0.032545175	0.122764546	0.265102392	0.790930584	0.875273298
ENSG00000066654	340.4052037	-0.10054975	0.103238059	-0.973960102	0.33007635	0.502392728
ENSG00000066697	284.5782181	0.459886902	0.115900074	3.967960389	7.25E-05	0.000430195
ENSG00000066735	32.15985886	0.084492078	0.306444676	0.275717233	0.782765258	0.870139877
ENSG00000066739	181.725811	0.067568625	0.142775564	0.473250623	0.636034385	0.765060371
ENSG00000066777	620.4641134	-0.049692679	0.085532578	-0.580979556	0.561254232	0.707650857

ENSG00000066827	139.3805972	0.388177307	0.156252033	2.484302439	0.012980551	0.040304554
ENSG00000066855	178.2925032	0.265925828	0.145763255	1.824368063	0.068096447	0.155790031
ENSG00000066926	199.2280592	-0.081639503	0.136101785	-0.599841536	0.548611849	0.696965781
ENSG00000066933	706.019085	-0.07741378	0.080246082	-0.964704798	0.334692692	0.506346606
ENSG00000067057	1260.433092	0.997099748	0.070010342	14.242178	5.01E-46	4.70E-44
ENSG00000067064	1018.280288	0.095196324	0.081952964	1.161597085	0.245399167	0.408959793
ENSG00000067066	80.16762293	8.082933832	1.033548258	7.820567416	5.26E-15	1.26E-13
ENSG00000067082	197.2221349	0.548592	0.134106994	4.09070387	4.30E-05	0.000268614
ENSG00000067113	60.77192761	-0.040153196	0.225754177	-0.177862472	0.858830984	0.919492083
ENSG00000067141	452.5832617	-0.184977867	0.094435675	-1.958771054	0.050139605	0.122106628
ENSG00000067167	464.2085011	0.089103334	0.09297904	0.958316348	0.337903263	0.509885824
ENSG00000067177	377.3572788	-0.104414302	0.110059545	-0.948707374	0.342769463	0.514590639
ENSG00000067182	327.6761936	0.041195696	0.109352657	0.376723322	0.706379229	0.815678152
ENSG00000067191	73.66901686	0.15393281	0.209238717	0.73568034	0.461925257	0.624920929
ENSG00000067208	315.4023705	-0.221090478	0.109885658	-2.012004865	0.044219424	0.11072197
ENSG00000067221	22.11890221	0.357983803	0.370172615	0.967072628	0.333507728	0.505752246
ENSG00000067225	7992.947106	0.389355421	0.062296217	6.250065283	4.10E-10	6.02E-09
ENSG00000067248	375.4577284	0.155321418	0.105913921	1.466487281	0.142515584	0.27457364
ENSG00000067334	508.8592159	0.34855656	0.100571299	3.465765724	0.000528724	0.002546103
ENSG00000067365	126.7485073	0.124085655	0.161193707	0.769792179	0.44142318	0.60654127
ENSG00000067369	815.3059563	-0.291387708	0.081779306	-3.563098312	0.000366503	0.001838669
ENSG00000067533	443.0472808	0.253889371	0.09809642	2.588161423	0.009648975	0.031338533
ENSG00000067560	1017.548936	0.261552792	0.078122959	3.347963215	0.000814078	0.003738266
ENSG00000067596	424.36822	-0.264513058	0.096163804	-2.750650939	0.005947698	0.020700525
ENSG00000067606	173.5675878	0.727686494	0.138686664	5.246982479	1.55E-07	1.58E-06
ENSG00000067704	1378.217218	0.463472376	0.069025436	6.714515764	1.89E-11	3.23E-10
ENSG00000067798	112.8380838	-4.193741286	0.290388512	-14.44182916	2.82E-47	2.73E-45
ENSG00000067829	97.58431511	0.369963288	0.180206922	2.052991544	0.040073398	0.101958163
ENSG00000067836	94.67735371	0.401975598	0.187045248	2.149082116	0.031627891	0.084016072
ENSG00000067840	33.14301975	-1.264589159	0.322665681	-3.919193246	8.88E-05	0.00051742
ENSG00000067900	1215.285625	-0.205961402	0.068357648	-3.012997203	0.002586813	0.010184126
ENSG00000067955	502.2495508	0.167292938	0.092390124	1.810723181	0.070183713	0.159294007
ENSG00000067992	210.5815039	-0.29493113	0.128878205	-2.288448458	0.022111418	0.062572074
ENSG00000068001	317.075774	0.063940891	0.117973033	0.541995824	0.587821383	0.729570925
ENSG00000068024	200.5712447	-0.254005108	0.134059005	-1.894726182	0.058128687	0.137186436
ENSG00000068028	112.2448534	0.525562903	0.180068869	2.918677204	0.0035152	0.013252286
ENSG00000068078	148.4171819	1.460192831	0.162337916	8.99477378	2.37E-19	7.75E-18
ENSG00000068079	76.51622624	-0.343067025	0.208521265	-1.645237601	0.099920822	0.209615859
ENSG00000068097	624.3010036	0.111082897	0.083983566	1.322674222	0.18594374	0.334008612
ENSG00000068120	184.8302177	-0.207985683	0.134622247	-1.544957747	0.122356484	0.245710011
ENSG00000068137	223.5650919	0.003391768	0.134134	0.025286414	0.97982651	0.989786143
ENSG00000068305	519.9499195	0.067464092	0.089940448	0.750097354	0.453196073	0.617396281
ENSG00000068308	390.4782759	0.050193886	0.100306239	0.500406423	0.616788931	0.750912994
ENSG00000068323	391.5787065	0.224700471	0.106161601	2.116588941	0.03429475	0.089926889
ENSG00000068354	106.4446647	0.528411763	0.171287985	3.084931869	0.002035988	0.00829212
ENSG00000068366	497.1511053	0.097902511	0.091358019	1.071635656	0.283883712	0.453613298
ENSG00000068383	204.9836381	-0.38026142	0.132901759	-2.861221879	0.004220116	0.015498387
ENSG00000068394	150.5948072	0.470604901	0.151926453	3.097583689	0.001951053	0.008008896
ENSG00000068400	392.2979769	-0.136190757	0.115038306	-1.183873112	0.236463295	0.397701138
ENSG00000068438	250.6545403	0.54388357	0.127146934	4.277598772	1.89E-05	0.00012734

ENSG00000068489	1002.44717	0.162504773	0.080475922	2.01929682	0.043456378	0.109318006
ENSG00000068650	709.3698832	0.124513089	0.085114355	1.462891767	0.143496997	0.275859083
ENSG00000068654	1063.131079	0.105717007	0.073080991	1.446573259	0.14801648	0.282191572
ENSG00000068697	507.1012145	-0.249542817	0.092320376	-2.703009112	0.006871486	0.023428099
ENSG00000068724	209.5058833	-0.185637892	0.134616171	-1.379016288	0.167889732	0.310105923
ENSG00000068745	570.7212329	-0.083608285	0.09626073	-0.86856068	0.385087469	0.554120434
ENSG00000068784	434.1073705	-0.108548564	0.097764383	-1.110307875	0.266866381	0.43356648
ENSG00000068796	409.4296399	-0.392357478	0.101268686	-3.874420546	0.000106879	0.000612536
ENSG00000068831	106.1392975	-1.866632581	0.207235852	-9.007285982	2.11E-19	6.95E-18
ENSG00000068878	797.9466777	0.071876172	0.085661352	0.839073518	0.401428056	0.569754461
ENSG00000068885	62.60507542	-0.974532431	0.227754375	-4.278874684	1.88E-05	0.000126673
ENSG00000068903	182.9949479	0.386987536	0.137610982	2.81218498	0.00492062	0.017659669
ENSG00000068912	368.7363681	0.075673142	0.106594741	0.709914405	0.477757217	0.638134946
ENSG00000068971	55.31845796	0.076406785	0.23610842	0.323608895	0.746234153	0.845214574
ENSG00000068976	153.4631871	-2.619491323	0.180006828	-14.55217756	5.66E-48	5.72E-46
ENSG00000069011	429.2311506	0.349041929	0.10540737	3.31136172	0.000928431	0.004209707
ENSG00000069020	10.33314668	-5.666572511	1.256066565	-4.511363227	6.44E-06	4.81E-05
ENSG00000069188	169.4767025	-2.670924213	0.177937819	-15.01043579	6.27E-51	6.78E-49
ENSG00000069248	435.9427419	-0.24190295	0.10415256	-2.322582857	0.020201573	0.058257337
ENSG00000069275	4120.006085	-0.565787585	0.05647878	-10.01770195	1.27E-23	5.22E-22
ENSG00000069329	425.1835861	0.076295157	0.095077542	0.802451932	0.422291583	0.589889605
ENSG00000069345	536.7973634	-0.003449829	0.093133834	-0.037041626	0.970451816	0.984539698
ENSG00000069399	92.27016348	-0.156973562	0.186737894	-0.840609037	0.400566994	0.568747917
ENSG00000069424	124.757538	-0.4207116	0.168153456	-2.501950357	0.012351124	0.038623384
ENSG00000069493	25.19832479	-0.956230588	0.364287224	-2.624935834	0.00866653	0.028617476
ENSG00000069509	78.99547723	0.064606066	0.200013079	0.323009209	0.746688268	0.845576459
ENSG00000069696	12.84802453	-0.489087635	0.484599561	-1.009261408	0.312849283	0.484076461
ENSG00000069702	25.12130346	0.876812101	0.352414251	2.488015452	0.012845815	0.039921497
ENSG00000069849	347.5998857	0.03898315	0.103413438	0.376964069	0.706200308	0.815661201
ENSG00000069869	350.5559413	0.02514087	0.103816804	0.242165707	0.808651766	0.886947088
ENSG00000069943	60.28500992	0.006082053	0.237646212	0.02559289	0.979582057	0.989728056
ENSG00000069956	409.6816264	-0.118062642	0.105041105	-1.123966109	0.261027404	0.427271747
ENSG00000069966	65.09863959	-0.377284994	0.233033304	-1.619017484	0.105443501	0.218501439
ENSG00000069974	59.97077541	-0.18693551	0.228029829	-0.819785333	0.412338494	0.580607352
ENSG00000069998	301.628096	0.118067874	0.123317937	0.957426609	0.338351973	0.51028883
ENSG00000070010	441.4281623	0.540924004	0.107096565	5.050806267	4.40E-07	4.19E-06
ENSG00000070018	722.4506878	-0.20745675	0.079289208	-2.616456325	0.008884773	0.029214515
ENSG00000070047	814.5490999	0.313488304	0.08460474	3.705327914	0.000211117	0.001124172
ENSG00000070061	410.5029135	0.347499001	0.097228993	3.574026539	0.000351533	0.001769888
ENSG00000070081	456.6123932	0.076153347	0.093280799	0.816388234	0.414278124	0.582467547
ENSG00000070087	644.8681519	0.159038624	0.088034002	1.806559061	0.070831058	0.160500295
ENSG00000070159	22.86553563	1.622133366	0.387497195	4.186180927	2.84E-05	0.000184403
ENSG00000070193	7.191538272	-5.136924214	1.29071879	-3.979894191	6.89E-05	0.000412121
ENSG00000070214	1196.796337	-1.496492762	0.073793508	-20.27946355	1.95E-91	4.42E-89
ENSG00000070269	93.23070166	-0.348133643	0.190211838	-1.830241728	0.067213801	0.154147307
ENSG00000070366	423.0360506	-0.354699743	0.108552263	-3.267548133	0.001084834	0.004826945
ENSG00000070367	452.8594801	-0.066449112	0.096789552	-0.68653187	0.492377774	0.651755616
ENSG00000070371	53.8558628	-0.22135041	0.242321776	-0.913456536	0.361002483	0.532109326
ENSG00000070404	43.39795926	-0.36657952	0.270229619	-1.356548259	0.174924792	0.319373027
ENSG00000070413	305.2071212	-0.28525967	0.109446859	-2.606376035	0.009150594	0.029941392

ENSG00000070423	450.8036325	0.261258338	0.101438802	2.575526663	0.010008756	0.032297953
ENSG00000070444	615.7734955	0.079502497	0.086633743	0.917685124	0.358783738	0.530006853
ENSG00000070476	313.9418196	-0.050165949	0.108989541	-0.460282226	0.645313657	0.771662632
ENSG00000070495	296.547748	0.356789692	0.112835574	3.162031955	0.001566724	0.00662685
ENSG00000070501	43.81290231	0.433607254	0.270255929	1.604431975	0.108618868	0.223719829
ENSG00000070540	104.1237608	-0.000917872	0.175489889	-0.005230343	0.995826809	0.998100549
ENSG00000070610	224.3096159	-0.293160494	0.13364013	-2.193656167	0.028260137	0.076738865
ENSG00000070614	553.9946443	-0.300497335	0.090366462	-3.325319256	0.000883173	0.004021373
ENSG00000070669	42.96121192	-0.891098223	0.285458543	-3.12163796	0.00179848	0.007461088
ENSG00000070718	77.71248238	-0.105618442	0.19948676	-0.529450888	0.596492705	0.73589786
ENSG00000070756	17137.16041	0.117114722	0.0604506	1.937362421	0.052701057	0.127074009
ENSG00000070759	26.4288314	-0.729078504	0.346186577	-2.10602765	0.035201951	0.091842831
ENSG00000070761	444.1523034	-0.001456699	0.095126216	-0.015313325	0.987782212	0.99372461
ENSG00000070770	553.816745	-0.047832293	0.089991098	-0.531522501	0.595056753	0.734903558
ENSG00000070778	152.4827861	-2.1856448	0.180643977	-12.09918445	1.07E-33	6.60E-32
ENSG00000070785	201.3555548	0.17180821	0.136580514	1.257926221	0.20841844	0.362827342
ENSG00000070814	2025.680799	0.184867898	0.065584654	2.818767586	0.004820841	0.017350334
ENSG00000070831	100.8269571	0.158019109	0.18055895	0.875166307	0.381483423	0.550886667
ENSG00000070882	205.9132252	-0.124279801	0.131955059	-0.941834302	0.34627748	0.518180437
ENSG00000070886	81.40738376	0.35227953	0.194275789	1.813296098	0.069786165	0.15867872
ENSG00000070950	249.3077073	0.02312796	0.117367365	0.197056139	0.843783606	0.909432081
ENSG00000070961	262.5321153	-1.220387813	0.1219288	-10.00902009	1.39E-23	5.66E-22
ENSG00000071051	230.707288	-0.392515312	0.12405956	-3.163926358	0.001556562	0.006591807
ENSG00000071054	715.0753053	-0.402437574	0.083850641	-4.799457357	1.59E-06	1.35E-05
ENSG00000071073	11.91060311	0.122422279	0.526397087	0.232566407	0.816098115	0.891910855
ENSG00000071082	11687.62023	0.379645057	0.056323834	6.740397927	1.58E-11	2.73E-10
ENSG00000071127	2189.553558	0.059592402	0.05973752	0.99757074	0.318487555	0.490117735
ENSG00000071189	393.5628042	0.142886115	0.102409506	1.3952427	0.162942665	0.303277645
ENSG00000071205	224.3362933	0.065052337	0.126322143	0.514971764	0.606572751	0.743501522
ENSG00000071242	63.65603884	-1.113433472	0.252526452	-4.409175603	1.04E-05	7.41E-05
ENSG00000071243	119.3397083	-0.602263411	0.180176619	-3.342627993	0.000829891	0.003804659
ENSG00000071246	321.2456052	0.015075435	0.1162049	0.129731495	0.896778863	0.944094484
ENSG00000071282	28.560712	-0.845418393	0.342532517	-2.46814054	0.013581698	0.041810677
ENSG00000071462	1110.491864	0.773807337	0.075063813	10.30866012	6.44E-25	2.85E-23
ENSG00000071537	397.6116448	-0.024127513	0.103178384	-0.233842711	0.81510709	0.891521397
ENSG00000071539	340.3183637	0.2647414	0.114307567	2.316044398	0.020555845	0.059126532
ENSG00000071553	897.8161101	0.497928021	0.077069104	6.460799381	1.04E-10	1.64E-09
ENSG00000071564	1070.963863	0.329755939	0.078865487	4.181245228	2.90E-05	0.000188018
ENSG00000071575	210.3385976	6.20964948	0.350224679	17.73047377	2.44E-70	3.98E-68
ENSG00000071626	1306.551348	0.310786841	0.074271809	4.184452308	2.86E-05	0.000185554
ENSG00000071655	332.2528684	0.14866937	0.118596625	1.253571674	0.209997732	0.365122354
ENSG00000071794	1000.753539	-0.086840778	0.071800396	-1.209474918	0.226480442	0.384874276
ENSG00000071859	483.0671984	0.107894027	0.106957039	1.008760415	0.31308955	0.484283494
ENSG00000071889	161.0525671	0.006726949	0.158755159	0.042373107	0.966201267	0.982498132
ENSG00000071894	1153.213413	0.306230543	0.071944533	4.256481093	2.08E-05	0.000139176
ENSG00000071967	332.5964754	0.255024601	0.11078614	2.301954039	0.021337764	0.060883927
ENSG00000071994	498.115839	0.31197008	0.098034173	3.18225848	0.001461313	0.006254439
ENSG00000072041	298.3050225	0.566336896	0.109979268	5.149487762	2.61E-07	2.58E-06
ENSG00000072042	929.0523792	0.039313245	0.082005209	0.479399365	0.631654545	0.761618146
ENSG00000072062	962.6774889	-0.131060086	0.080866209	-1.620702736	0.105081407	0.218032278

ENSG00000072071	2282.889635	-0.043025536	0.069679068	-0.617481512	0.536917172	0.687643647
ENSG00000072110	1101.771002	0.031028419	0.070012395	0.443184654	0.657632178	0.780564116
ENSG00000072121	341.8067069	0.034823613	0.103597904	0.336142064	0.736763739	0.838085161
ENSG00000072133	39.69264905	-1.656998153	0.305108089	-5.430856186	5.61E-08	6.16E-07
ENSG00000072134	319.3991366	-0.084831551	0.112699045	-0.752726441	0.451614317	0.616629899
ENSG00000072135	346.6519038	0.273362769	0.102571944	2.665083231	0.007696927	0.025886468
ENSG00000072163	12.38938787	-0.630964542	0.498730125	-1.265142228	0.205820348	0.359373328
ENSG00000072195	63.29992916	-0.541500611	0.226228893	-2.393596164	0.016684107	0.049807584
ENSG00000072210	320.706368	-0.178004223	0.108837461	-1.635505094	0.101943208	0.212872936
ENSG00000072274	761.565734	0.013081878	0.085690781	0.152663768	0.878663435	0.933347343
ENSG00000072310	1382.740357	0.303356259	0.079497076	3.815942368	0.000135664	0.000758595
ENSG00000072364	1813.151633	-0.00764623	0.064280934	-0.118950197	0.905314812	0.94931003
ENSG00000072401	101.6769633	-0.018356663	0.179602232	-0.102207319	0.918592118	0.955355281
ENSG00000072415	283.0866043	-0.079346316	0.118905784	-0.667304086	0.504577917	0.662287983
ENSG00000072422	183.7529929	-1.195607472	0.147728424	-8.093279826	5.81E-16	1.51E-14
ENSG00000072501	4170.417183	0.017757429	0.053803853	0.330040101	0.741369662	0.842008184
ENSG00000072506	320.7457672	0.739028591	0.11553066	6.396817868	1.59E-10	2.44E-09
ENSG00000072518	535.9668316	0.062713333	0.092412173	0.67862632	0.497374662	0.655493567
ENSG00000072571	552.1619043	-0.400813285	0.090635331	-4.422263176	9.77E-06	7.02E-05
ENSG00000072609	80.99891647	0.007122474	0.19802645	0.035967289	0.971308442	0.985053042
ENSG00000072657	12.38465636	-4.027776654	0.822478637	-4.897120089	9.73E-07	8.63E-06
ENSG00000072682	439.9000936	0.612754144	0.105134054	5.828312705	5.60E-09	7.12E-08
ENSG00000072736	499.8162848	-0.050431525	0.093065527	-0.541892651	0.587892459	0.729572441
ENSG00000072756	130.5437323	-0.199048835	0.159280943	-1.249671374	0.211419619	0.366375183
ENSG00000072778	491.2706704	0.292582652	0.094494688	3.096286776	0.001959607	0.008034619
ENSG00000072786	355.7817059	0.24116006	0.101535901	2.375121096	0.017543194	0.051924981
ENSG00000072803	408.2058422	-0.015600347	0.109558153	-0.142393302	0.886769353	0.938065731
ENSG00000072818	9.595767673	-0.161649539	0.577912926	-0.279712621	0.779697994	0.868262525
ENSG00000072840	771.3061324	0.062932562	0.082878199	0.759337956	0.447650418	0.612760224
ENSG00000072849	142.3023741	-0.085451719	0.160732273	-0.531640082	0.594975298	0.734903558
ENSG00000072864	302.6433511	-0.205744964	0.108445042	-1.897227941	0.057797861	0.136497402
ENSG00000072954	107.3751798	-0.602432615	0.179534239	-3.355530502	0.000792129	0.003644133
ENSG00000072958	383.5786737	0.166023308	0.108984484	1.523366455	0.12766705	0.25299064
ENSG00000073008	727.3086747	0.235092992	0.083369282	2.81989943	0.00480387	0.01729812
ENSG00000073050	379.8991353	0.079290254	0.111157745	0.713312907	0.475652152	0.636590614
ENSG00000073060	3002.972274	0.884089518	0.060784391	14.54468	6.31E-48	6.33E-46
ENSG00000073111	1123.067287	-0.048851827	0.073425591	-0.665324258	0.505843118	0.663390746
ENSG00000073150	18.74363352	0.992818184	0.405695539	2.447200152	0.014397086	0.043935666
ENSG00000073169	205.0757986	-0.005034735	0.142312101	-0.035378122	0.97177823	0.985302874
ENSG00000073331	35.39731817	-1.074456582	0.307442747	-3.494818438	0.000474384	0.002311369
ENSG00000073350	150.5986722	-0.562272087	0.149550382	-3.759750259	0.000170083	0.000924954
ENSG00000073417	539.4089132	-0.151278078	0.096384511	-1.569526858	0.116525226	0.235990463
ENSG00000073464	61.64807758	2.929276003	0.286117238	10.23802699	1.34E-24	5.77E-23
ENSG00000073536	204.7310327	0.255145554	0.139892898	1.823863525	0.068172707	0.155862959
ENSG00000073578	105.7166091	0.091102621	0.172491851	0.528156087	0.597391005	0.736682596
ENSG00000073584	106.0522449	-0.503533107	0.175928481	-2.862146616	0.004207822	0.01546906
ENSG00000073605	43.18799994	0.350876817	0.292955736	1.197712738	0.231028869	0.390477983
ENSG00000073614	262.8859014	-0.213727889	0.11948136	-1.788796927	0.073647526	0.165154342
ENSG00000073670	221.0067525	0.325877453	0.125568192	2.595222939	0.009452958	0.030765918
ENSG00000073711	187.4276517	-0.285164075	0.13555525	-2.103674151	0.035406881	0.092274667

ENSG00000073712	408.8733431	-0.011771407	0.103880333	-0.113316997	0.909779242	0.951381168
ENSG00000073756	23.81289941	-0.866858064	0.364781542	-2.376375899	0.017483642	0.051812809
ENSG00000073792	762.5853629	-0.322919565	0.082367044	-3.920494793	8.84E-05	0.000515274
ENSG00000073803	10.19023986	-0.673347972	0.569822506	-1.181680199	0.237332618	0.398733559
ENSG00000073849	58.81343109	0.016755977	0.228874604	0.073210294	0.941638775	0.96767868
ENSG00000073910	69.19855858	-2.263584084	0.258214698	-8.766286734	1.85E-18	5.78E-17
ENSG00000073921	754.2940011	0.059479417	0.08124405	0.732107977	0.464102665	0.627026563
ENSG00000073969	102.8225822	0.191510196	0.184775874	1.036445896	0.299994168	0.470406446
ENSG00000074047	693.1202785	0.071628503	0.091654757	0.78150339	0.434506484	0.600798561
ENSG00000074054	700.1738738	-0.110280186	0.081844271	-1.347439289	0.177838794	0.323295139
ENSG00000074071	1290.390365	0.317817868	0.070778552	4.490313191	7.11E-06	5.27E-05
ENSG00000074181	922.575806	0.241902303	0.097850758	2.472155642	0.013430103	0.041441742
ENSG00000074201	820.1087377	0.241155527	0.082953787	2.907106914	0.003647885	0.013664491
ENSG00000074211	159.2528639	-0.338817958	0.148119477	-2.287463907	0.02216876	0.062662318
ENSG00000074219	596.1782385	-0.204617089	0.087750933	-2.331793892	0.019711537	0.057102073
ENSG00000074266	140.3253338	0.107824423	0.162566426	0.663263784	0.507161627	0.66425169
ENSG00000074317	67.34066547	-0.089976539	0.213373305	-0.421686016	0.673254213	0.791795205
ENSG00000074319	201.1361731	0.178809023	0.134482128	1.32961179	0.183646212	0.330989821
ENSG00000074356	639.0604546	-0.076006301	0.085346169	-0.890564881	0.373162647	0.54340148
ENSG00000074370	39.80481623	0.871968603	0.289586261	3.01108416	0.002603167	0.010231305
ENSG00000074410	644.699462	0.445003683	0.089534063	4.970216576	6.69E-07	6.16E-06
ENSG00000074582	186.5962952	0.636410522	0.143290012	4.441415805	8.94E-06	6.47E-05
ENSG00000074603	328.1408102	-0.29642084	0.110660101	-2.678660493	0.00739173	0.024973678
ENSG00000074621	94.95718014	-0.367219716	0.188172883	-1.951501778	0.050997385	0.123702343
ENSG00000074657	380.8177993	0.029866522	0.103305138	0.289109748	0.772497394	0.863610895
ENSG00000074660	8.993035369	0.762094815	0.577670342	1.319255568	0.187083687	0.335389465
ENSG00000074695	2067.98913	-0.215115594	0.064768395	-3.32130499	0.000895976	0.004077023
ENSG00000074696	1162.423093	0.005520157	0.07153662	0.077165465	0.938491915	0.966102042
ENSG00000074755	494.3171779	0.04020899	0.093074965	0.432006502	0.665736688	0.786165328
ENSG00000074800	5368.654847	0.346237155	0.05803767	5.965731515	2.44E-09	3.23E-08
ENSG00000074842	1502.252965	1.003745358	0.066053469	15.1959522	3.76E-52	4.30E-50
ENSG00000074855	190.3318478	-0.254476326	0.139568111	-1.823312816	0.068256026	0.156002669
ENSG00000074935	141.2677901	-0.551576196	0.158154597	-3.487576132	0.00048742	0.002366675
ENSG00000074964	46.88222392	0.573312215	0.254392823	2.253649329	0.024218237	0.067498726
ENSG00000075043	430.7240686	0.484432699	0.095584147	5.068128103	4.02E-07	3.86E-06
ENSG00000075089	263.6677303	0.588438682	0.133821237	4.397199548	1.10E-05	7.80E-05
ENSG00000075131	168.7872722	0.118356599	0.143410833	0.825297481	0.409202721	0.577637398
ENSG00000075142	162.7815299	0.495744655	0.14478698	3.423958803	0.00061716	0.002922874
ENSG00000075151	1247.001372	0.108697485	0.074861508	1.451980975	0.146506895	0.280143847
ENSG00000075188	55.36357258	0.189860611	0.234427801	0.809889487	0.418003695	0.58598722
ENSG00000075213	58.67968505	-8.186816235	1.194278461	-6.855031305	7.13E-12	1.27E-10
ENSG00000075218	404.31921	0.064983841	0.098054074	0.662734739	0.507500457	0.664432322
ENSG00000075223	50.27791956	-1.08248164	0.276431291	-3.91591573	9.01E-05	0.000523633
ENSG00000075234	220.9309423	-0.061737136	0.126684057	-0.487331541	0.626023399	0.757363766
ENSG00000075239	493.5540439	-0.301620951	0.093038868	-3.241881145	0.001187435	0.005210842
ENSG00000075240	214.2815625	0.017692597	0.127748952	0.138495046	0.889849184	0.939631412
ENSG00000075275	808.1497327	-0.04429487	0.090369185	-0.490154584	0.624024515	0.755922074
ENSG00000075292	1346.038263	-0.153847881	0.072887065	-2.110770709	0.034792025	0.090993032
ENSG00000075303	101.3860419	-0.218126431	0.192723062	-1.131812812	0.25771314	0.423403097
ENSG00000075336	299.7960742	0.245759669	0.115119311	2.134825741	0.032775247	0.086572751

ENSG00000075340	302.3433146	0.073070669	0.116537311	0.627015232	0.530649251	0.682791062
ENSG00000075391	476.589983	-0.19871264	0.096988715	-2.048822275	0.040479497	0.102823778
ENSG00000075399	69.54528156	1.133512637	0.222851948	5.086393227	3.65E-07	3.52E-06
ENSG00000075407	1050.363896	0.212049992	0.070563781	3.005082639	0.002655087	0.010409839
ENSG00000075413	763.3446449	0.027484714	0.080844244	0.339971187	0.733878226	0.836005299
ENSG00000075415	1662.518875	-0.09541216	0.073607304	-1.296232234	0.194895487	0.346105804
ENSG00000075420	590.8843412	0.053879357	0.092892292	0.580019669	0.561901354	0.708212501
ENSG00000075426	165.5218126	0.709794197	0.143465925	4.947475842	7.52E-07	6.84E-06
ENSG00000075539	563.2595593	-0.206614835	0.092315262	-2.23814383	0.025211677	0.069769194
ENSG00000075568	805.5341052	-0.010070666	0.077809188	-0.129427727	0.89701921	0.944134611
ENSG00000075618	574.015935	-0.234891453	0.093600806	-2.509502449	0.012090138	0.037900059
ENSG00000075624	10280.26189	-0.130846478	0.061745395	-2.119129342	0.034079538	0.089496217
ENSG00000075643	133.1585485	-0.12477026	0.159988452	-0.779870409	0.435467158	0.60165311
ENSG00000075651	32.60142387	-0.117103235	0.314714541	-0.372093498	0.709823237	0.818224767
ENSG00000075702	216.3527724	-0.063287521	0.136040526	-0.465210793	0.64178051	0.768812112
ENSG00000075711	1162.924776	0.019255332	0.075950552	0.253524575	0.799862873	0.880907053
ENSG00000075785	1941.470703	0.166482477	0.083510346	1.993555117	0.046200695	0.114580006
ENSG00000075790	8.976598053	0.871597574	0.615376539	1.416364647	0.156668769	0.294450923
ENSG00000075826	74.07375248	0.155214277	0.203543964	0.762558977	0.445726458	0.610751217
ENSG00000075856	992.6713268	0.087389543	0.076022387	1.149523799	0.250340059	0.414440402
ENSG00000075884	28.77959898	-1.35607326	0.341210622	-3.974299657	7.06E-05	0.000420318
ENSG00000075914	281.0186327	0.23658969	0.112357899	2.10567919	0.035232229	0.091904757
ENSG00000075945	94.74641776	-0.573063708	0.184968913	-3.098162279	0.001947248	0.007995612
ENSG00000075975	300.8633417	0.47045546	0.115981005	4.056314742	4.99E-05	0.000307278
ENSG00000076003	947.0974972	0.112108829	0.077897368	1.439186352	0.150097731	0.285230361
ENSG00000076043	120.5551107	0.420728395	0.164228312	2.561850578	0.01041161	0.033390842
ENSG00000076053	85.78159746	-0.131373288	0.189645177	-0.692732026	0.488477737	0.648303739
ENSG00000076067	279.2839027	-0.23400053	0.115485957	-2.026224974	0.042741735	0.10788736
ENSG00000076108	2702.346249	0.010572422	0.062269261	0.169785565	0.86517878	0.924195157
ENSG00000076201	1025.000503	0.373634993	0.080741512	4.627545183	3.70E-06	2.92E-05
ENSG00000076248	430.3679746	-0.229732326	0.096043896	-2.391951348	0.016759064	0.049992476
ENSG00000076321	119.5116931	-0.02536764	0.169329694	-0.149812119	0.880912848	0.934253325
ENSG00000076351	143.8073081	0.1821791	0.149859851	1.215663162	0.224113263	0.382378113
ENSG00000076382	732.0819173	-0.055249871	0.080314403	-0.687919834	0.491503266	0.651150276
ENSG00000076513	147.7844414	-0.656441688	0.149988667	-4.376608601	1.21E-05	8.49E-05
ENSG00000076554	190.371307	0.29294205	0.13323466	2.198692523	0.027899793	0.075951239
ENSG00000076555	277.1851703	-0.442241191	0.114801907	-3.852211185	0.000117056	0.000664895
ENSG00000076604	180.8169144	0.11408908	0.137179221	0.831679021	0.405590145	0.573801237
ENSG00000076641	38.91152076	-0.036955287	0.279632103	-0.13215681	0.894860262	0.943064154
ENSG00000076650	114.4864231	0.127593802	0.170147114	0.749902829	0.453313231	0.617396281
ENSG00000076662	39.27167865	-0.296441814	0.284811641	-1.040834613	0.297952313	0.468661042
ENSG00000076685	423.0753387	0.191110897	0.094899063	2.013833341	0.044027034	0.110437097
ENSG00000076706	12.12966137	-0.487033742	0.55223029	-0.881939565	0.37780949	0.547235502
ENSG00000076716	47.8347298	-1.226714534	0.265067453	-4.627933464	3.69E-06	2.91E-05
ENSG00000076770	109.8797338	0.415746111	0.17473255	2.379328362	0.017344218	0.051443039
ENSG00000076826	27.45175861	-0.139384102	0.335942624	-0.414904487	0.678211825	0.795757928
ENSG00000076864	27.58613402	-0.862855665	0.341307944	-2.528085505	0.011468642	0.036251127
ENSG00000076924	348.830281	0.428784228	0.103967083	4.124230646	3.72E-05	0.000235685
ENSG00000076928	293.6485681	0.08350071	0.120914378	0.690577183	0.489831291	0.649486515
ENSG00000076944	11.92160944	-1.056171252	0.551246706	-1.915968369	0.055369122	0.132161437

ENSG00000076984	417.7994246	0.186902811	0.112537367	1.66080668	0.096752279	0.204311375
ENSG00000077044	216.653276	-0.035594118	0.124878521	-0.285029948	0.775621198	0.86552026
ENSG00000077092	14.60862246	0.183468329	0.448228718	0.40931855	0.682305904	0.799028849
ENSG00000077097	3345.129228	0.271906343	0.058951313	4.612388219	3.98E-06	3.11E-05
ENSG00000077147	1185.158272	-0.28459329	0.069487838	-4.095584155	4.21E-05	0.000263369
ENSG00000077150	360.9328045	0.429843145	0.109291248	3.93300609	8.39E-05	0.000493263
ENSG00000077152	272.5490883	-0.301177849	0.115576593	-2.605872365	0.00916406	0.029978471
ENSG00000077157	300.3594058	-0.227755026	0.113920208	-1.999250444	0.045581263	0.113384098
ENSG00000077232	471.4820497	0.038316681	0.092384469	0.414752411	0.67832316	0.795757928
ENSG00000077235	2134.652219	-0.107695193	0.06131328	-1.756474168	0.079007478	0.174719466
ENSG00000077238	31.79368081	-1.358919523	0.347135552	-3.914665371	9.05E-05	0.000526136
ENSG00000077254	262.4515241	-0.150137509	0.12304701	-1.220163815	0.222402781	0.380615481
ENSG00000077312	2136.356064	0.349670572	0.061910184	5.648029953	1.62E-08	1.93E-07
ENSG00000077327	9.563976542	-2.758407758	0.731746243	-3.769623398	0.000163494	0.000893619
ENSG00000077348	108.0028039	0.536718626	0.171705344	3.125812006	0.001773149	0.00737124
ENSG00000077380	1089.907853	0.322110152	0.069477526	4.636177628	3.55E-06	2.81E-05
ENSG00000077454	62.71879508	-0.331924992	0.241297862	-1.375581986	0.168951087	0.311407528
ENSG00000077458	169.0554461	-0.117390633	0.139830057	-0.839523596	0.401175554	0.569453618
ENSG00000077463	55.07106147	-0.004701537	0.236250111	-0.019900677	0.984122605	0.991961125
ENSG00000077514	234.9089275	-0.247606345	0.122516313	-2.02100715	0.043279025	0.109047617
ENSG00000077522	135.1628257	0.164612152	0.157858619	1.042782161	0.297049195	0.467576204
ENSG00000077549	1055.871675	-0.014227625	0.076398106	-0.186230075	0.85226434	0.915251049
ENSG00000077585	217.5608194	0.950946995	0.136032834	6.990569623	2.74E-12	5.19E-11
ENSG00000077684	559.1326098	-0.180464921	0.089611733	-2.013853706	0.044024895	0.110437097
ENSG00000077713	205.3601452	0.342727785	0.136540663	2.510078513	0.012070432	0.037855182
ENSG00000077721	260.4735902	0.423386221	0.119635378	3.538971741	0.000401689	0.00199101
ENSG00000077782	870.4389173	0.429809134	0.085169123	5.046537019	4.50E-07	4.27E-06
ENSG00000077942	1237.300202	-0.403207272	0.068780018	-5.862273422	4.57E-09	5.84E-08
ENSG00000078018	37.78979858	-3.194450873	0.399524638	-7.995629225	1.29E-15	3.27E-14
ENSG00000078043	392.0117605	-0.124414914	0.106816188	-1.164757113	0.244117323	0.407293897
ENSG00000078061	356.8464228	-0.090908444	0.110849781	-0.820104861	0.412156331	0.580465208
ENSG00000078070	115.8579798	-0.11636409	0.171349087	-0.679105396	0.497071084	0.655435794
ENSG00000078081	10.08344312	-0.30475489	0.568631787	-0.535944167	0.591997138	0.732410058
ENSG00000078114	40.94751669	-2.225708558	0.327919659	-6.78735933	1.14E-11	2.00E-10
ENSG00000078124	106.9158817	-0.320016933	0.174086505	-1.838263876	0.066023531	0.151963386
ENSG00000078140	653.7236087	-0.220058716	0.08361044	-2.631952617	0.00848957	0.028158912
ENSG00000078142	198.4885559	-0.151456468	0.13528335	-1.11954995	0.262905594	0.429372733
ENSG00000078177	248.1997966	-0.426738885	0.120305781	-3.547118704	0.000389469	0.001938651
ENSG00000078237	154.8097069	-0.77911236	0.149219414	-5.221253302	1.78E-07	1.80E-06
ENSG00000078246	370.6076046	-0.137776795	0.107498521	-1.281662234	0.199961158	0.35203088
ENSG00000078269	119.7238385	-0.199123477	0.163939457	-1.214615936	0.224512608	0.382778461
ENSG00000078304	1166.23014	1.721074487	0.074004624	23.25631013	1.23E-119	4.01E-117
ENSG00000078369	2197.735499	0.041417474	0.079083314	0.523719501	0.60047365	0.738900556
ENSG00000078399	96.75377837	-0.602168217	0.190181629	-3.166279625	0.001544023	0.006546601
ENSG00000078403	861.1100353	0.091602993	0.07438198	1.231521299	0.218127956	0.375127741
ENSG00000078487	17.09348452	-1.301757571	0.443582727	-2.934644412	0.003339301	0.012667341
ENSG00000078596	18.27254078	4.098672878	0.653403452	6.27280567	3.55E-10	5.24E-09
ENSG00000078618	1105.663298	-0.023207986	0.072789742	-0.318835939	0.749850924	0.848168256
ENSG00000078668	438.5675629	0.156974491	0.106526791	1.473568186	0.140597893	0.27166143
ENSG00000078674	1637.773355	-0.270007043	0.063347213	-4.26233502	2.02E-05	0.000135838

ENSG00000078687	535.9761467	-0.345285485	0.090433839	-3.818100497	0.000134483	0.000752623
ENSG00000078699	220.6288088	-0.135491488	0.127461909	-1.062995909	0.287783807	0.457814171
ENSG00000078747	175.5037802	-0.194893995	0.139000901	-1.402105986	0.1608836	0.300163624
ENSG00000078804	317.5149011	-0.910479698	0.114908859	-7.923494383	2.31E-15	5.73E-14
ENSG00000078808	795.1341978	0.666461951	0.095585528	6.97241479	3.12E-12	5.86E-11
ENSG00000078902	202.8467749	0.620043922	0.132570297	4.677095347	2.91E-06	2.34E-05
ENSG00000078967	170.6171887	-0.015604997	0.147601974	-0.105723501	0.915801734	0.954435272
ENSG00000079134	310.9477761	0.006655928	0.108268565	0.061476087	0.950980058	0.973078152
ENSG00000079150	30.18511506	0.148019338	0.327071274	0.452559885	0.65086569	0.775528913
ENSG00000079156	184.0245697	-0.282870704	0.144712313	-1.954710689	0.050617228	0.123039961
ENSG00000079215	51.41927818	-0.472068606	0.261656381	-1.804154766	0.071207048	0.161147963
ENSG00000079246	2350.37134	0.22580771	0.069532848	3.247496917	0.001164249	0.005126711
ENSG00000079277	92.94882876	-0.049586253	0.189629244	-0.261490539	0.793714245	0.877138226
ENSG00000079308	36.66389475	-0.48657686	0.296270803	-1.642338207	0.100519933	0.210495453
ENSG00000079313	651.835086	0.250716399	0.108504169	2.310661426	0.020851563	0.059751512
ENSG00000079332	686.1133459	-0.210699865	0.081801106	-2.57575815	0.010002058	0.032297953
ENSG00000079335	248.5286565	-0.643305146	0.122508997	-5.2510849	1.51E-07	1.55E-06
ENSG00000079385	215.4766033	-6.129294961	0.370026451	-16.56447789	1.26E-61	1.72E-59
ENSG00000079387	334.8291897	-0.197355894	0.112964164	-1.747066388	0.080625823	0.177601808
ENSG00000079432	1291.277629	0.18166455	0.093638978	1.940052688	0.052373287	0.126512271
ENSG00000079435	92.13334612	-0.541383544	0.196099754	-2.760755851	0.005766777	0.020155739
ENSG00000079459	1386.720611	0.112619509	0.070313383	1.601679569	0.109226485	0.224806512
ENSG00000079462	533.5910854	0.318959068	0.092299287	3.455704587	0.000548856	0.002634924
ENSG00000079482	111.1369855	-0.170508533	0.17699578	-0.963348011	0.335372907	0.507124904
ENSG00000079616	1315.905233	0.11521355	0.066050388	1.744328139	0.081101888	0.178452101
ENSG00000079691	223.8215168	-0.608548733	0.125839206	-4.835923198	1.33E-06	1.14E-05
ENSG00000079739	354.9166822	0.033344384	0.106329579	0.313594619	0.753828947	0.851208619
ENSG00000079785	1241.36233	0.209923089	0.072536223	2.894044923	0.003803137	0.014155451
ENSG00000079805	934.9493574	0.01570953	0.076590156	0.205111601	0.837484932	0.905074151
ENSG00000079819	1780.330726	-0.072516837	0.064524691	-1.123861831	0.261071646	0.427271747
ENSG00000079841	203.9996824	0.425040457	0.137021953	3.10198802	0.001922257	0.007909183
ENSG00000079950	114.5480901	-0.107314272	0.166803992	-0.643355537	0.519993428	0.674538277
ENSG00000079974	50.37943156	0.128661084	0.247593507	0.519646439	0.603310024	0.741253138
ENSG00000079999	732.2800993	-0.099972686	0.081496332	-1.226713942	0.219930126	0.377487595
ENSG00000080031	103.5757346	0.594719033	0.189076878	3.145382127	0.0016587	0.006971802
ENSG00000080189	322.8274577	0.198879703	0.107176457	1.855628647	0.063506507	0.147330714
ENSG00000080200	211.914028	0.943752863	0.129357687	7.295684444	2.97E-13	6.23E-12
ENSG00000080224	89.12399783	-4.445662447	0.346292445	-12.8378846	1.01E-37	7.25E-36
ENSG00000080298	120.4511549	-0.154558114	0.168087564	-0.919509514	0.357829132	0.529153154
ENSG00000080345	1679.938364	-0.071129509	0.067277781	-1.057251102	0.290397003	0.460719076
ENSG00000080371	383.0583832	-0.24804027	0.104575503	-2.371877378	0.017697963	0.052271507
ENSG00000080493	6.042188483	-5.875395725	1.306278874	-4.497811181	6.87E-06	5.10E-05
ENSG00000080503	42.1430517	1.191278688	0.292389064	4.074292903	4.62E-05	0.000286745
ENSG00000080546	110.0060817	-0.307344511	0.169562544	-1.812573133	0.069897685	0.15885015
ENSG00000080561	102.3844099	-0.220315539	0.176201337	-1.250362469	0.211167168	0.366275621
ENSG00000080603	151.2272561	-0.138357921	0.148391884	-0.932381997	0.351139145	0.522671567
ENSG00000080608	884.954894	0.238079957	0.081053815	2.937307248	0.003310759	0.012569252
ENSG00000080644	25.8327322	-0.662329469	0.342153824	-1.935765211	0.052896464	0.127406828
ENSG00000080802	682.6927883	0.44734873	0.089771906	4.983170695	6.26E-07	5.80E-06
ENSG00000080815	367.8984915	-0.192067493	0.105110317	-1.827294398	0.067655517	0.15500849

ENSG00000080819	191.0585707	0.091770481	0.132094253	0.694734854	0.487221481	0.647676578
ENSG00000080822	194.6866474	0.096896303	0.142460975	0.680160327	0.496402949	0.65504671
ENSG00000080823	210.6137175	0.1302694	0.130198361	1.000545623	0.31704653	0.48874351
ENSG00000080824	4209.558897	0.651681025	0.055309814	11.78237593	4.81E-32	2.82E-30
ENSG00000080839	271.215575	-0.005926981	0.116986528	-0.05066379	0.959593431	0.978612525
ENSG00000080845	466.4804169	-0.022033158	0.100183973	-0.219926972	0.82592803	0.898324082
ENSG00000080986	220.670379	0.028078298	0.129927931	0.216106716	0.828904565	0.89997137
ENSG00000081014	224.0159043	-0.026532025	0.128441906	-0.206568293	0.836347024	0.904331203
ENSG00000081019	165.1608271	-0.152215234	0.14223421	-1.070173162	0.284541372	0.454166006
ENSG00000081026	102.2100092	0.292693641	0.177334141	1.650520536	0.098836517	0.207806698
ENSG00000081059	138.4137415	0.378055267	0.155040862	2.438423405	0.014751485	0.044870962
ENSG00000081087	158.9562451	0.231078783	0.155130017	1.489581367	0.136334344	0.265873738
ENSG00000081138	7.491597126	-6.186768487	1.291588567	-4.790045875	1.67E-06	1.41E-05
ENSG00000081154	514.6261581	-0.042769634	0.088038871	-0.485803977	0.627106155	0.758020838
ENSG00000081177	110.8665485	0.183801512	0.170715986	1.076650853	0.281636276	0.450739976
ENSG00000081181	8.686718568	-0.69714095	0.643269098	-1.083746992	0.278476994	0.447353079
ENSG00000081307	275.2646278	0.230404266	0.123766314	1.861607235	0.062658473	0.145777036
ENSG00000081320	98.16010739	-0.29372382	0.187804302	-1.563988775	0.117820205	0.238269304
ENSG00000081377	62.85066174	-0.909864599	0.233032996	-3.904445359	9.44E-05	0.00054661
ENSG00000081386	133.5231617	-0.202911046	0.156944141	-1.292887036	0.196050132	0.347103126
ENSG00000081692	187.7350829	0.006667425	0.138973756	0.047976145	0.961735254	0.979729866
ENSG00000081721	172.8933819	-0.122069587	0.142909096	-0.854176471	0.393007218	0.561159532
ENSG00000081760	151.9153238	-0.134555952	0.151922941	-0.885685539	0.375787003	0.545716162
ENSG00000081791	609.1461615	-0.042433941	0.089173218	-0.475859703	0.634174332	0.763476761
ENSG00000081870	120.8380221	0.06473437	0.172827526	0.37456053	0.707987332	0.81689601
ENSG00000081913	170.7596406	-0.1670997	0.14507867	-1.151786818	0.249408686	0.41362973
ENSG00000082014	578.8071335	0.526303538	0.086283994	6.099665918	1.06E-09	1.48E-08
ENSG00000082068	206.5271054	-0.067457214	0.136337693	-0.49478037	0.620755162	0.753970589
ENSG00000082126	8.693898657	-3.481393758	0.866650205	-4.017069098	5.89E-05	0.000356017
ENSG00000082146	40.92954537	-0.370302363	0.296803505	-1.247634737	0.212164851	0.367255658
ENSG00000082153	530.084704	0.140582597	0.089834527	1.564906083	0.117604932	0.237902719
ENSG00000082196	9.692986068	-1.172200908	0.587539359	-1.99510193	0.046031764	0.114241729
ENSG00000082212	646.8819329	0.061367549	0.083298451	0.736718962	0.461293273	0.624585702
ENSG00000082213	158.527074	-0.020633787	0.157222836	-0.131239121	0.89558615	0.943333732
ENSG00000082258	323.4594971	0.080178969	0.106724329	0.751271713	0.45248915	0.61715847
ENSG00000082269	215.830937	-0.1664617	0.129552765	-1.284894997	0.198828979	0.350564914
ENSG00000082458	215.2575235	-2.235108057	0.147697059	-15.13305726	9.80E-52	1.11E-49
ENSG00000082512	239.1632578	-0.059347721	0.119892674	-0.495007066	0.620595133	0.753848135
ENSG00000082515	168.6715775	0.272318986	0.147301222	1.848721841	0.064497988	0.14918545
ENSG00000082516	772.8358032	0.203556061	0.077392289	2.630185316	0.008533834	0.028290788
ENSG00000082641	1221.253517	-0.106855007	0.069154666	-1.545159753	0.122307627	0.245647016
ENSG00000082701	857.8609071	-0.273779612	0.075722819	-3.615549644	0.000299711	0.001537634
ENSG00000082781	258.693583	0.056510883	0.117944066	0.479132907	0.631844081	0.761725204
ENSG00000082805	735.7338112	-0.423176301	0.085640365	-4.941318296	7.76E-07	7.05E-06
ENSG00000082898	2044.640933	0.058800409	0.064394833	0.913123098	0.361177803	0.532159624
ENSG00000082996	106.774403	-0.126728071	0.173223431	-0.731587349	0.464420472	0.627219918
ENSG00000083093	230.2589033	0.024585188	0.131126725	0.187491818	0.851275038	0.914699033
ENSG00000083097	89.55429662	-0.077617643	0.185370259	-0.418716808	0.675423111	0.793548715
ENSG00000083099	202.7401303	-0.039770119	0.134791469	-0.29504923	0.767956272	0.860382976
ENSG00000083123	177.9509037	0.468858577	0.15237354	3.0770341	0.002090714	0.008473242

ENSG00000083168	1297.254751	-0.226139482	0.068744431	-3.289568037	0.001003413	0.004508938
ENSG00000083223	333.6016319	-0.242450155	0.10856734	-2.233177635	0.025537233	0.070572793
ENSG00000083290	172.1122874	-0.249903587	0.142246632	-1.756833072	0.078946266	0.174639087
ENSG00000083312	1738.881399	0.221659214	0.070800175	3.130772123	0.001743474	0.00727587
ENSG00000083444	2886.61218	0.393023711	0.062430811	6.29534847	3.07E-10	4.55E-09
ENSG00000083454	30.15321989	-0.122279983	0.316096961	-0.386843274	0.698872238	0.810992014
ENSG00000083457	36.52310001	0.299936229	0.295825472	1.013895886	0.310632435	0.482043064
ENSG00000083520	672.7685766	-0.095095918	0.082425199	-1.153723848	0.248613404	0.412749377
ENSG00000083535	130.9416854	0.039262279	0.16932215	0.231879163	0.816631869	0.892216525
ENSG00000083544	297.0446912	-0.410766037	0.115886236	-3.544562772	0.000393265	0.001954083
ENSG00000083635	106.5917194	0.264415011	0.177746207	1.48759861	0.13685678	0.26648561
ENSG00000083642	683.1622372	-0.132420999	0.080612517	-1.642685331	0.100448055	0.21040767
ENSG00000083720	504.6944607	0.565598227	0.093135794	6.072834101	1.26E-09	1.73E-08
ENSG00000083750	57.05569293	-0.292079133	0.231036713	-1.264210909	0.206154336	0.359643562
ENSG00000083799	120.2661225	0.474617216	0.168426001	2.817956927	0.004833029	0.01738084
ENSG00000083807	168.7186976	0.06514646	0.141992561	0.458801921	0.646376415	0.772319358
ENSG00000083812	71.06420216	0.247795733	0.206967277	1.197270102	0.231201294	0.390628407
ENSG00000083817	22.15184171	0.195249066	0.367871566	0.530753351	0.59558971	0.735203051
ENSG00000083828	123.4240381	0.516366905	0.166267637	3.105636868	0.001898697	0.007832893
ENSG00000083838	33.83552692	-0.297520141	0.302614687	-0.983164909	0.325526282	0.497624082
ENSG00000083844	283.231422	0.146169531	0.117018428	1.249115492	0.211622835	0.366543276
ENSG00000083845	8466.580351	0.224370123	0.162846788	1.377798885	0.168265391	0.310553665
ENSG00000083857	3167.552226	-0.50424524	0.066981345	-7.528144422	5.15E-14	1.15E-12
ENSG00000083896	603.9542476	-0.126580355	0.085327907	-1.483457868	0.137952802	0.267988247
ENSG00000083937	144.6710802	-0.00959992	0.149151115	-0.064363713	0.948680623	0.971432914
ENSG00000084070	281.5195465	0.515488456	0.1154389	4.465465781	7.99E-06	5.87E-05
ENSG00000084072	208.2470803	0.077672374	0.1296495	0.599095049	0.549109503	0.69718384
ENSG00000084073	190.1527758	-0.21029509	0.140007393	-1.502028463	0.133089758	0.261397068
ENSG00000084090	501.8725348	0.0211962	0.095157371	0.222748907	0.823730935	0.896764198
ENSG00000084092	295.4228512	0.294591794	0.111559651	2.640666151	0.008274321	0.027542509
ENSG00000084093	1000.286807	-0.091278229	0.074415865	-1.226596364	0.219974337	0.37751735
ENSG00000084112	528.423774	-0.02737843	0.087823234	-0.311744726	0.755234534	0.852042366
ENSG00000084207	2845.322106	0.34232938	0.057880489	5.914417568	3.33E-09	4.33E-08
ENSG00000084234	2526.872826	-0.093964221	0.061673591	-1.523573046	0.127615401	0.252943916
ENSG00000084444	128.4051162	-0.110010319	0.164760799	-0.667697169	0.504326918	0.662082263
ENSG00000084463	1140.761213	-0.071453936	0.070560091	-1.012667845	0.311218842	0.482646216
ENSG00000084623	525.8099539	0.146867936	0.099038097	1.482943843	0.138089332	0.268179295
ENSG00000084628	31.99031549	-1.619082259	0.334508748	-4.840179117	1.30E-06	1.12E-05
ENSG00000084636	583.2914231	5.754984029	0.191377917	30.07130673	1.15E-198	9.50E-196
ENSG00000084652	1314.610585	0.188173476	0.068040556	2.765607563	0.005681686	0.019909584
ENSG00000084676	519.3229129	-0.11191279	0.094950443	-1.178644209	0.23853988	0.400139682
ENSG00000084693	361.4190897	-0.501374289	0.103324191	-4.852438541	1.22E-06	1.06E-05
ENSG00000084710	237.5253322	-0.062731123	0.120742481	-0.519544752	0.603380914	0.741253138
ENSG00000084731	377.5841914	0.214852972	0.102159392	2.103115219	0.035455699	0.092384753
ENSG00000084733	545.315364	-0.218147909	0.088679454	-2.459959979	0.013895251	0.042598557
ENSG00000084754	3098.408732	0.125616768	0.055110223	2.279373242	0.022644888	0.063797179
ENSG00000084764	150.4292113	0.359434917	0.148120201	2.426643453	0.015239224	0.04612484
ENSG00000084774	1790.088304	0.104326987	0.072032689	1.448328357	0.147525241	0.281752149
ENSG00000085063	182.4260589	0.443490139	0.142558466	3.110935131	0.001864959	0.007711828
ENSG00000085185	937.4234663	-0.420165258	0.078987997	-5.319355765	1.04E-07	1.09E-06

ENSG00000085224	1891.738605	-0.201384217	0.071720294	-2.807911192	0.004986398	0.017863792
ENSG00000085231	77.80818007	-0.174145549	0.202531175	-0.859845644	0.389874135	0.558214797
ENSG00000085274	136.9110046	-0.082272584	0.168958092	-0.486940774	0.626300303	0.757437827
ENSG00000085365	298.3561636	-0.121794372	0.110382789	-1.103381904	0.269861352	0.437174767
ENSG00000085377	360.7199525	-0.054203904	0.103845008	-0.521969277	0.601691722	0.739842754
ENSG00000085382	263.3926036	-1.673348211	0.13121096	-12.75311312	3.00E-37	2.11E-35
ENSG00000085415	473.6888443	0.017717298	0.093962736	0.18855664	0.850440319	0.914361666
ENSG00000085433	75.77451079	0.187192769	0.206037004	0.908539558	0.363593224	0.534581748
ENSG00000085449	276.1719792	-0.237909648	0.114291888	-2.081596972	0.037379298	0.096446587
ENSG00000085465	21.21155054	-1.114158107	0.400324685	-2.783136163	0.005383621	0.019038255
ENSG00000085511	472.5437666	-0.273472411	0.098793381	-2.768124829	0.005637986	0.019789301
ENSG00000085514	9.498151457	0.344356036	0.578899166	0.594846317	0.551946204	0.699430612
ENSG00000085552	11.04373034	-1.948682408	0.586319357	-3.323585322	0.000888682	0.004045146
ENSG00000085644	107.0807444	0.550765547	0.170794779	3.224721221	0.001260954	0.005495693
ENSG00000085662	490.5439521	0.177596467	0.091718832	1.936314085	0.052829245	0.127288577
ENSG00000085719	327.6967231	-0.13613522	0.108680852	-1.252614585	0.210346005	0.365458296
ENSG00000085721	301.3795425	0.110243617	0.119968107	0.918941042	0.358126411	0.529369851
ENSG00000085733	3394.054491	1.01307397	0.057272838	17.68855888	5.14E-70	8.20E-68
ENSG00000085741	15.22155003	-1.459208174	0.470895907	-3.098791371	0.001943118	0.007983322
ENSG00000085760	603.0495147	0.007983119	0.093091658	0.085755468	0.931660808	0.963061253
ENSG00000085788	379.9095451	-0.516079422	0.108961114	-4.736363313	2.18E-06	1.80E-05
ENSG00000085831	215.7024031	0.942519325	0.13685079	6.887204098	5.69E-12	1.03E-10
ENSG00000085832	479.634397	-0.026645145	0.097390716	-0.273590201	0.784399566	0.871011269
ENSG00000085840	163.2061827	0.102778633	0.158284614	0.649328008	0.516126386	0.671234308
ENSG00000085872	1374.5629	0.346221124	0.068740296	5.036654533	4.74E-07	4.48E-06
ENSG00000085978	277.9807929	0.234149946	0.113505636	2.062892692	0.039122825	0.100047027
ENSG00000085982	183.7552086	-0.009343469	0.13647562	-0.068462551	0.94541743	0.969665557
ENSG00000085998	343.6879382	0.011930746	0.110795874	0.107682223	0.914247772	0.953636867
ENSG00000085999	221.9948666	-0.274920286	0.1276649	-2.153452404	0.031283141	0.083273504
ENSG00000086015	613.9979249	0.015681494	0.083276893	0.188305463	0.850637203	0.914433366
ENSG00000086061	2130.778526	0.266501409	0.065986846	4.038705087	5.37E-05	0.000327542
ENSG00000086062	398.8841461	-0.164762703	0.10012086	-1.645638104	0.099838289	0.209474003
ENSG00000086065	261.379769	0.183847376	0.116067589	1.583968252	0.113200944	0.23109049
ENSG00000086102	500.0653567	0.425817935	0.097886675	4.350111352	1.36E-05	9.49E-05
ENSG00000086159	78.93495889	-0.485966772	0.205230376	-2.367908601	0.017888955	0.05269158
ENSG00000086189	323.0775583	0.266787818	0.107895055	2.472660289	0.013411156	0.041403627
ENSG00000086200	153.5330706	-0.25386588	0.148585833	-1.708547012	0.087534891	0.189082364
ENSG00000086232	941.1424255	0.005217228	0.077368911	0.067433127	0.946236898	0.970132815
ENSG00000086289	104.7709185	0.576968692	0.181127855	3.185422211	0.00144543	0.006192116
ENSG00000086475	521.5541652	0.06762689	0.094419871	0.716235778	0.473845775	0.634943394
ENSG00000086504	284.8295506	0.244633752	0.114102877	2.143975321	0.032034864	0.084904479
ENSG00000086506	76.23890607	0.187976762	0.200364741	0.938172863	0.348155582	0.520159695
ENSG00000086544	147.8544145	0.252872132	0.15990694	1.581370585	0.113793329	0.231839783
ENSG00000086589	280.4157503	0.179716177	0.121919311	1.47405834	0.140465884	0.271518409
ENSG00000086598	1551.426083	0.768155801	0.070908261	10.83309316	2.40E-27	1.17E-25
ENSG00000086619	424.5170416	-3.062410988	0.127939974	-23.93631079	1.28E-126	4.87E-124
ENSG00000086666	436.4634741	-0.006389812	0.098868698	-0.06462927	0.948469179	0.971358017
ENSG00000086712	759.2259003	0.438643308	0.081297755	5.395515612	6.83E-08	7.38E-07
ENSG00000086758	5223.303127	-0.011650926	0.055579345	-0.209626896	0.833958886	0.902721285
ENSG00000086827	132.9356527	0.004479159	0.164466531	0.027234469	0.978272724	0.988831902

ENSG00000086848	53.8115997	0.054962901	0.243275583	0.225928554	0.821256989	0.895180952
ENSG00000087008	88.64085872	0.155497027	0.190890816	0.814586214	0.415309204	0.583209876
ENSG00000087053	237.9254749	0.166912544	0.127140117	1.312823586	0.1892424	0.338478825
ENSG00000087074	427.4024247	-0.082964417	0.103407521	-0.80230544	0.422376296	0.589928798
ENSG00000087076	135.0897805	-2.089450921	0.177336135	-11.78243182	4.81E-32	2.82E-30
ENSG00000087077	372.8478054	0.683287149	0.101339205	6.742574562	1.56E-11	2.70E-10
ENSG00000087085	67.12677378	-1.754612979	0.234868536	-7.470617444	7.98E-14	1.75E-12
ENSG00000087086	20043.46747	0.229577996	0.068110208	3.370684117	0.000749818	0.003473843
ENSG00000087087	1752.351474	0.501795412	0.071679031	7.000588674	2.55E-12	4.86E-11
ENSG00000087088	216.9691877	0.141962945	0.140670011	1.009191254	0.31288292	0.484076461
ENSG00000087095	81.57750754	-0.29230548	0.205104956	-1.425150744	0.154113631	0.29108971
ENSG00000087111	599.2785194	0.300257023	0.087448853	3.433515859	0.000595807	0.002834614
ENSG00000087152	262.32291	0.012596175	0.132662247	0.094949209	0.92435517	0.958686031
ENSG00000087157	161.7016956	-0.259982177	0.147873951	-1.758133706	0.078724762	0.174286325
ENSG00000087191	1025.417442	0.311133916	0.073450458	4.235969727	2.28E-05	0.000151141
ENSG00000087206	214.6207354	0.011971114	0.128938925	0.092843293	0.926028057	0.959498127
ENSG00000087263	293.4526765	0.000400905	0.110501892	0.003628035	0.997105253	0.998813347
ENSG00000087266	191.8837231	0.353826265	0.132979133	2.660765319	0.007796328	0.026160233
ENSG00000087269	877.0737529	0.149073176	0.078222453	1.905759413	0.056681427	0.134614313
ENSG00000087274	1086.713018	-0.219690396	0.072812278	-3.017216332	0.002551077	0.010057529
ENSG00000087299	171.4798697	-0.149624179	0.142973291	-1.046518394	0.295321757	0.465796623
ENSG00000087301	110.0913791	-0.515579915	0.17059815	-3.022189362	0.002509535	0.009921598
ENSG00000087302	864.8060117	0.022256103	0.079720363	0.279177137	0.780108888	0.868456931
ENSG00000087303	354.5349607	-2.124431615	0.128925683	-16.47795507	5.28E-61	7.07E-59
ENSG00000087338	246.0943146	0.256291886	0.119008203	2.15356488	0.031274311	0.083272862
ENSG00000087365	2087.913587	0.072967612	0.061208599	1.192113726	0.233216656	0.393371081
ENSG00000087448	427.0631273	-0.069541837	0.095108991	-0.731180469	0.464668928	0.627409989
ENSG00000087460	7521.126522	-0.634769725	0.073249023	-8.665913927	4.48E-18	1.35E-16
ENSG00000087470	934.8850566	-0.079050771	0.075595433	-1.045708286	0.295695736	0.46612479
ENSG00000087502	419.8060299	0.060261954	0.100030993	0.602432831	0.546886062	0.69527636
ENSG00000087586	629.1755877	-0.327013573	0.088281549	-3.704212006	0.000212049	0.001128277
ENSG00000087842	117.9814677	0.444924579	0.173081819	2.57060263	0.010152174	0.032708163
ENSG00000087884	37.60034996	-0.74681292	0.303428434	-2.46124897	0.013845425	0.042473615
ENSG00000087903	163.1408698	0.311402732	0.147102128	2.116915208	0.034267045	0.089887802
ENSG00000087995	474.7937197	0.137907448	0.095724112	1.440676181	0.149676187	0.284583297
ENSG00000088035	47.55726972	-0.284511128	0.253191631	-1.123698785	0.261140832	0.427285413
ENSG00000088038	915.994461	0.219379809	0.076668527	2.861406356	0.004217661	0.015497118
ENSG00000088179	331.4459292	-0.285421398	0.106222832	-2.687006105	0.007209564	0.024473837
ENSG00000088205	608.7530961	0.151423011	0.089749739	1.68716938	0.091570787	0.196051646
ENSG00000088247	4529.395908	0.150299565	0.070113285	2.143667428	0.032059544	0.08492182
ENSG00000088256	599.6578719	0.213475502	0.093441511	2.284589581	0.022336904	0.06303128
ENSG00000088280	160.1598275	-0.349595806	0.143049839	-2.44387417	0.014530493	0.044286565
ENSG00000088298	77.71767642	-0.469118407	0.206620575	-2.270434139	0.023181255	0.065086366
ENSG00000088305	225.4134378	-0.380743289	0.128666025	-2.959159491	0.003084794	0.011816772
ENSG00000088325	1294.06408	-0.166123229	0.070367773	-2.360785644	0.018236268	0.053602188
ENSG00000088340	163.4583104	-0.203480705	0.162057501	-1.255608066	0.209258106	0.363973776
ENSG00000088356	85.49424337	0.5606057	0.192528331	2.911808862	0.003593424	0.01351093
ENSG00000088367	378.4960211	0.597837308	0.10618395	5.63020409	1.80E-08	2.14E-07
ENSG00000088387	217.0627575	-0.551811528	0.126208677	-4.372215459	1.23E-05	8.64E-05
ENSG00000088448	405.5960153	-0.093570773	0.098795037	-0.947120181	0.343577542	0.515472727

ENSG00000088451	103.4627348	0.110288316	0.174959199	0.63036592	0.528455203	0.681090948
ENSG00000088538	289.0539303	0.209023199	0.116616541	1.792397524	0.073069311	0.164179315
ENSG00000088543	155.6240431	-0.110373987	0.151225327	-0.729864426	0.465473059	0.628097989
ENSG00000088682	356.9105401	-0.054049604	0.106456422	-0.507715763	0.611652692	0.747517508
ENSG00000088727	36.52057637	0.414134107	0.287149737	1.442223533	0.149239324	0.283957615
ENSG00000088756	8.537864255	-6.374616706	1.274279981	-5.002524408	5.66E-07	5.28E-06
ENSG00000088766	16.24987749	0.69036541	0.433953062	1.590875764	0.111637538	0.228563255
ENSG00000088808	176.9643468	-0.044338031	0.140105632	-0.316461443	0.751652286	0.849638663
ENSG00000088812	495.8134247	-0.111476426	0.100211346	-1.112413226	0.265960521	0.432751933
ENSG00000088826	16.05101005	-1.053644408	0.46535482	-2.264174265	0.023563399	0.065961551
ENSG00000088832	621.964021	0.407113175	0.111463699	3.652428345	0.000259772	0.001351488
ENSG00000088833	483.2090522	0.47349182	0.093075263	5.08719294	3.63E-07	3.51E-06
ENSG00000088836	191.0979135	0.100597662	0.132714252	0.758001946	0.448449818	0.613495772
ENSG00000088854	158.597536	0.063863854	0.145719203	0.438266564	0.661193061	0.783316094
ENSG00000088876	113.331895	0.531815684	0.173039006	3.073386146	0.002116444	0.008552846
ENSG00000088881	5.014363428	-5.606114238	1.333042561	-4.205502812	2.61E-05	0.000170914
ENSG00000088888	909.996617	0.047703514	0.076721818	0.621772464	0.534091495	0.685336917
ENSG00000088899	40.96204952	-0.44716399	0.291657305	-1.533182887	0.1252308	0.249768145
ENSG00000088930	942.8634791	-0.037421298	0.076020569	-0.492252276	0.622541013	0.75505595
ENSG00000088970	35.18942952	2.0935277	0.325912398	6.423590249	1.33E-10	2.07E-09
ENSG00000088986	765.2278564	0.349908184	0.102047844	3.428864056	0.000606113	0.002876375
ENSG00000088992	23.58785002	-1.876039795	0.413217294	-4.540080537	5.62E-06	4.27E-05
ENSG00000089006	451.2944949	-0.116726572	0.096134374	-1.214202238	0.224670506	0.382809194
ENSG00000089009	2734.08473	0.217927074	0.062895757	3.464893079	0.000530442	0.002553504
ENSG00000089022	383.3489196	-0.050529581	0.105639262	-0.478321984	0.632421052	0.762019087
ENSG00000089048	520.4737614	0.216194791	0.089486387	2.415951729	0.015694142	0.047328383
ENSG00000089050	131.5019918	0.035763088	0.155067612	0.230628999	0.817603037	0.892507031
ENSG00000089053	745.024483	0.067543174	0.082917096	0.814586819	0.415308858	0.583209876
ENSG00000089057	224.5238242	0.119992578	0.125741071	0.954283096	0.339940362	0.511717865
ENSG00000089060	79.70990795	-0.197505987	0.20311359	-0.972391787	0.330855676	0.503197237
ENSG00000089063	233.7065958	0.040532856	0.121194647	0.334444279	0.738044331	0.839322273
ENSG00000089091	34.71662997	0.027516338	0.302289848	0.091026339	0.927471662	0.960497968
ENSG00000089094	747.4231374	0.026720847	0.080745589	0.330926398	0.740700082	0.841524915
ENSG00000089123	72.43791558	-0.182233499	0.224511356	-0.811689451	0.416969849	0.584957103
ENSG00000089127	10.40961116	6.970020605	1.277961504	5.454014523	4.92E-08	5.45E-07
ENSG00000089154	985.4885405	-0.017559495	0.080051786	-0.219351691	0.826376099	0.898460144
ENSG00000089157	3072.142225	-0.005217341	0.072125233	-0.072337253	0.942333518	0.968180144
ENSG00000089159	316.6613493	-0.697840428	0.112404233	-6.20831095	5.36E-10	7.73E-09
ENSG00000089177	267.1335794	0.04208937	0.11478565	0.366677977	0.713859236	0.821528813
ENSG00000089195	214.4035999	0.277049701	0.126019994	2.198458304	0.027916463	0.075981894
ENSG00000089199	16.80941507	-0.699717786	0.437930783	-1.59778169	0.110091571	0.226222395
ENSG00000089220	1291.068055	0.143985277	0.070041629	2.055709987	0.039810479	0.101473017
ENSG00000089234	187.1339511	-0.050064428	0.138315433	-0.361958368	0.717383141	0.823964104
ENSG00000089248	1023.585939	0.417439691	0.086849161	4.806490763	1.54E-06	1.31E-05
ENSG00000089280	4210.619446	-0.13000703	0.05585927	-2.327402938	0.01994383	0.057632375
ENSG00000089289	139.0496195	-0.744780024	0.161294023	-4.617530211	3.88E-06	3.05E-05
ENSG00000089327	1042.256893	0.730624735	0.073713214	9.911719922	3.70E-23	1.46E-21
ENSG00000089335	72.18079078	-0.207214231	0.214131732	-0.967695117	0.333196657	0.505498806
ENSG00000089351	467.7437394	0.094416761	0.101505913	0.930160207	0.352288142	0.523715899
ENSG00000089486	310.9583097	0.492232272	0.110486906	4.455118628	8.38E-06	6.12E-05

ENSG00000089505	24.06410033	-0.980020353	0.371300577	-2.639425884	0.008304658	0.027630396
ENSG00000089597	2951.45157	0.244335135	0.066511341	3.673586041	0.00023917	0.001254995
ENSG00000089639	59.1044628	0.331135251	0.226126157	1.464382786	0.143089389	0.275339267
ENSG00000089682	248.1091784	-0.030304849	0.123034161	-0.246312476	0.805440353	0.88501413
ENSG00000089685	667.7637712	-0.067557421	0.081741037	-0.82648108	0.408531246	0.577095577
ENSG00000089692	16.70273818	-0.21402549	0.428976587	-0.498921146	0.617834937	0.751600186
ENSG00000089693	1775.549548	0.010035964	0.071529106	0.140306014	0.888418213	0.93888133
ENSG00000089723	39.34495711	0.657785815	0.28110482	2.340001909	0.019283641	0.056035852
ENSG00000089737	1098.091535	0.23896974	0.070750338	3.377648016	0.000731086	0.00339602
ENSG00000089775	148.2468704	0.046368165	0.151433639	0.306194615	0.759456472	0.85495152
ENSG00000089818	162.3620803	0.303752006	0.143672549	2.114196546	0.034498484	0.090343065
ENSG00000089820	327.3497104	-0.563834436	0.118826472	-4.745023788	2.08E-06	1.73E-05
ENSG00000089847	23.88785764	-1.488524934	0.384639842	-3.869918738	0.000108872	0.000621873
ENSG00000089876	179.6605841	-0.123255526	0.141226669	-0.872749647	0.38279955	0.552299228
ENSG00000089902	812.4204194	0.240829098	0.084728888	2.842349318	0.004478239	0.016305295
ENSG00000089916	703.1695691	-0.005907505	0.083356894	-0.070870019	0.943501204	0.968637121
ENSG00000090006	1413.533545	-0.750933324	0.093852518	-8.001205972	1.23E-15	3.14E-14
ENSG00000090013	371.0965386	0.26810957	0.103165938	2.598818717	0.009354516	0.030523319
ENSG00000090020	215.1617747	0.232368221	0.140883167	1.649368243	0.099072217	0.208177625
ENSG00000090054	341.0253766	-0.186345868	0.105911038	-1.759456529	0.078499998	0.173980667
ENSG00000090060	1852.625609	0.270257011	0.066389701	4.070767078	4.69E-05	0.000290377
ENSG00000090061	656.8928056	-0.093812797	0.087609794	-1.070802624	0.284258187	0.453798699
ENSG00000090097	266.2467841	0.04642189	0.116081908	0.399906327	0.689225512	0.804480669
ENSG00000090263	260.0559854	0.425411297	0.11540466	3.686257545	0.000227576	0.001199985
ENSG00000090266	400.2112534	1.034154827	0.11567957	8.939822545	3.90E-19	1.27E-17
ENSG00000090273	1949.879963	0.377268137	0.075352405	5.006716593	5.54E-07	5.19E-06
ENSG00000090316	352.9924604	-0.462465473	0.106512119	-4.341904718	1.41E-05	9.81E-05
ENSG00000090339	16.79982446	-2.227229258	0.50213528	-4.435516381	9.19E-06	6.63E-05
ENSG00000090372	640.434278	0.098827677	0.0845584	1.168750566	0.242504142	0.405240981
ENSG00000090432	232.4161425	-0.215393985	0.127678731	-1.686999737	0.091603402	0.196083439
ENSG00000090447	321.7350062	0.046254682	0.11134739	0.415408762	0.677842693	0.79559294
ENSG00000090470	277.2036864	0.265529311	0.123636274	2.147665097	0.03174037	0.08425113
ENSG00000090487	336.2263022	0.307565267	0.106900095	2.877128099	0.004013126	0.014843682
ENSG00000090520	155.6338679	0.283161095	0.1443803	1.961216969	0.049853718	0.121584607
ENSG00000090565	316.0008601	0.329909447	0.120328024	2.741750726	0.00611127	0.021178285
ENSG00000090581	139.6353182	0.445096585	0.157879534	2.819216494	0.004814104	0.017330526
ENSG00000090612	130.0927027	-0.189718005	0.164531062	-1.153083207	0.248876234	0.413038861
ENSG00000090615	1431.638997	0.381566333	0.075384362	5.061611197	4.16E-07	3.98E-06
ENSG00000090621	1559.639079	0.055506551	0.065196795	0.851369318	0.39456423	0.562684643
ENSG00000090661	162.2431836	-0.242269606	0.16678212	-1.452611383	0.146331684	0.279929343
ENSG00000090674	83.99324055	0.665566095	0.195644052	3.401923477	0.000669134	0.003139386
ENSG00000090686	911.7924918	0.158093026	0.074734985	2.115381779	0.034397422	0.090162452
ENSG00000090776	102.5968395	-0.117772091	0.178261476	-0.660670457	0.508823674	0.665497779
ENSG00000090857	567.8904518	-0.124160221	0.088055045	-1.410029607	0.158530941	0.2968783
ENSG00000090861	1616.527752	-0.324248836	0.066409861	-4.882540465	1.05E-06	9.23E-06
ENSG00000090863	1203.659438	-1.017901984	0.070922196	-14.35237552	1.03E-46	9.77E-45
ENSG00000090889	354.7561053	0.11227226	0.102330137	1.097157332	0.272572621	0.44055087
ENSG00000090905	1153.311497	0.013356549	0.069427854	0.192380265	0.84744435	0.912257704
ENSG00000090924	1292.188694	-0.162272144	0.070821116	-2.29129608	0.021946295	0.06226613
ENSG00000090932	94.51259889	0.348714338	0.183709942	1.898178906	0.057672519	0.136293047

ENSG00000090971	250.4215727	0.074719149	0.119632261	0.624573572	0.532250969	0.683849875
ENSG00000090975	122.5912535	0.429326665	0.163619256	2.623937268	0.008691979	0.028667817
ENSG00000090989	112.9250125	-0.025068764	0.175765357	-0.142626307	0.88658532	0.938065731
ENSG00000091009	172.8280704	-0.083450534	0.148411586	-0.562291232	0.573917611	0.718394287
ENSG00000091039	617.6458647	-0.05493358	0.084318154	-0.651503585	0.514721464	0.670589167
ENSG00000091073	96.99592365	0.380060983	0.184283433	2.062371953	0.039172337	0.100105442
ENSG00000091127	411.5137381	0.777067697	0.106301153	7.310059002	2.67E-13	5.62E-12
ENSG00000091136	1722.309782	0.769097266	0.075673684	10.16333846	2.89E-24	1.22E-22
ENSG00000091140	506.5721049	0.522318006	0.093952982	5.55935526	2.71E-08	3.12E-07
ENSG00000091157	207.1821637	-0.013063428	0.128659588	-0.101534819	0.919125919	0.955581501
ENSG00000091164	685.4038197	0.329629886	0.093460973	3.526925466	0.000420415	0.002074314
ENSG00000091262	108.5403068	-0.576244359	0.18863094	-3.0548772	0.002251527	0.00904927
ENSG00000091317	485.8650787	0.173391553	0.090531921	1.915253226	0.055460218	0.132288958
ENSG00000091409	171.7753956	-2.231248481	0.174175452	-12.81034991	1.43E-37	1.02E-35
ENSG00000091428	10.69187833	0.302961084	0.545405502	0.555478599	0.578567344	0.722026962
ENSG00000091436	623.8508804	0.084825533	0.085631515	0.990587789	0.321886903	0.493780814
ENSG00000091483	556.6680847	0.310738749	0.093115389	3.337136365	0.000846464	0.003866758
ENSG00000091490	384.6437527	1.171576706	0.103288944	11.34271163	8.06E-30	4.37E-28
ENSG00000091527	1356.498749	0.103558086	0.067843177	1.52643333	0.126901982	0.252063369
ENSG00000091536	46.25823888	0.283924607	0.265832169	1.068059627	0.285493616	0.455148865
ENSG00000091542	838.4485734	0.100684777	0.082383944	1.222140782	0.221654393	0.379473402
ENSG00000091622	10.83849958	-0.356886666	0.537192536	-0.66435522	0.506462987	0.663949991
ENSG00000091640	876.0535482	0.046740768	0.08113503	0.576086168	0.56455694	0.710622166
ENSG00000091651	350.479231	-0.060176954	0.103107008	-0.583635925	0.559465285	0.706155886
ENSG00000091732	174.9718962	0.632478988	0.142305072	4.444528782	8.81E-06	6.39E-05
ENSG00000091947	126.9659352	0.213121642	0.163390929	1.304366426	0.192108677	0.342412205
ENSG00000091972	53.66472965	-0.903227297	0.248836353	-3.62980443	0.000283636	0.001463716
ENSG00000091986	549.3288166	-1.141293099	0.094275198	-12.10597398	9.82E-34	6.10E-32
ENSG00000092010	549.9224138	0.182695305	0.101683356	1.796708066	0.072381978	0.163097472
ENSG00000092020	136.8775279	-0.125495235	0.157755902	-0.795502629	0.426321182	0.593579721
ENSG00000092036	73.12316688	-0.650309054	0.210813916	-3.084753915	0.002037206	0.008293941
ENSG00000092051	23.68662675	0.71670653	0.362756361	1.975724224	0.04818602	0.118561748
ENSG00000092068	125.3753591	1.239882654	0.168483674	7.359067053	1.85E-13	3.98E-12
ENSG00000092094	219.2490535	0.058955394	0.12498611	0.471695567	0.637144102	0.765870546
ENSG00000092096	90.88681234	-0.146606932	0.195027177	-0.751725655	0.452216059	0.616965713
ENSG00000092098	11.81708529	0.180040228	0.499561541	0.360396495	0.718550647	0.824765561
ENSG00000092108	403.3592418	-0.214679278	0.099940239	-2.148076491	0.031707679	0.084196177
ENSG00000092140	241.7186741	-0.403298333	0.129271132	-3.119786509	0.001809822	0.007501494
ENSG00000092148	1211.963903	0.123997754	0.073255255	1.692680675	0.090516266	0.194238006
ENSG00000092199	2893.929041	0.193962795	0.063020074	3.077793825	0.002085391	0.008459075
ENSG00000092201	2732.858084	-0.141618522	0.07329438	-1.93218802	0.053336303	0.128202439
ENSG00000092203	351.1899549	0.186594975	0.106392192	1.753840871	0.079457778	0.175494246
ENSG00000092208	25.63544866	-0.303831486	0.354763772	-0.856433238	0.391758187	0.560088725
ENSG00000092330	141.4890704	0.1316034	0.15390771	0.85507997	0.392506878	0.560718408
ENSG00000092421	16.79359873	-1.924646537	0.475973124	-4.04360339	5.26E-05	0.000321608
ENSG00000092439	538.7350805	-0.150975728	0.095899364	-1.574314174	0.115414834	0.234315968
ENSG00000092445	86.06120133	0.946617034	0.201273889	4.703128847	2.56E-06	2.09E-05
ENSG00000092470	361.8570565	-0.066345559	0.105651684	-0.627964997	0.53002687	0.682240213
ENSG00000092529	14.14030922	-0.076173004	0.464717375	-0.163912538	0.869799997	0.927577954
ENSG00000092531	213.0206526	-0.302861391	0.135541766	-2.234450676	0.025453435	0.070382755

ENSG00000092607	36.71868677	1.254575668	0.303544112	4.133091757	3.58E-05	0.000227598
ENSG00000092621	1380.619109	-0.402745454	0.077339968	-5.207468557	1.91E-07	1.93E-06
ENSG00000092758	70.04285199	1.026523696	0.219154217	4.684024384	2.81E-06	2.27E-05
ENSG00000092820	1330.599035	-0.078349377	0.069346572	-1.129823354	0.258550666	0.424332422
ENSG00000092841	353.9917141	-0.10403151	0.13441067	-0.773982527	0.438941124	0.604253819
ENSG00000092847	566.7776173	-0.658148281	0.091894906	-7.161966943	7.95E-13	1.60E-11
ENSG00000092853	786.2866616	-0.325476347	0.077920244	-4.177044744	2.95E-05	0.000190818
ENSG00000092871	89.93850837	-0.138220872	0.185058327	-0.746904366	0.45512129	0.61888565
ENSG00000092929	12.59894382	-0.403073846	0.498604371	-0.808404155	0.418857961	0.586785457
ENSG00000092931	99.15823193	-0.013962191	0.178616952	-0.078168342	0.937694144	0.965886166
ENSG00000092964	1274.939617	-0.01586008	0.070663505	-0.224445134	0.822410955	0.896299688
ENSG00000092969	103.644109	0.541330437	0.175116372	3.091261149	0.001993083	0.008145718
ENSG00000092978	438.0683036	-0.568312086	0.103639545	-5.483544751	4.17E-08	4.68E-07
ENSG00000093000	590.4203229	0.126191265	0.08921991	1.414384588	0.157249013	0.295273461
ENSG00000093009	317.9762282	0.044767458	0.111396234	0.401875866	0.687775385	0.803224213
ENSG00000093010	251.8598771	0.383377178	0.117141567	3.272768048	0.001064998	0.004753744
ENSG00000093144	313.9732168	0.146915925	0.10853272	1.353655602	0.175846279	0.320627295
ENSG00000093167	304.7363846	0.154857547	0.108759453	1.423853686	0.154488831	0.291719937
ENSG00000093183	415.7425173	0.03913007	0.097712421	0.400461572	0.688816582	0.804194845
ENSG00000093217	543.7596018	0.486229213	0.090632494	5.364844248	8.10E-08	8.64E-07
ENSG00000094631	252.982866	-1.93752318	0.137742648	-14.06625482	6.12E-45	5.51E-43
ENSG00000094804	368.3527667	-0.0353277	0.107619839	-0.328263828	0.742712191	0.842720368
ENSG00000094841	51.99940396	0.219527506	0.244466606	0.897985658	0.369193195	0.540608445
ENSG00000094880	328.8023686	-0.170051339	0.105249481	-1.615697653	0.106159699	0.219782651
ENSG00000094914	461.3002122	-0.062252754	0.108587907	-0.573293614	0.566445908	0.712087495
ENSG00000094916	2596.499366	-0.648416413	0.070533016	-9.193090742	3.82E-20	1.30E-18
ENSG00000094975	473.0652505	-0.267303636	0.101595633	-2.631054385	0.008512041	0.028226781
ENSG00000095002	559.3461926	0.018759199	0.09271591	0.20232988	0.839658842	0.906725688
ENSG00000095015	120.6870839	-0.138660761	0.165805011	-0.836288121	0.402992834	0.571289494
ENSG00000095059	23.5512201	-0.008853984	0.38906191	-0.022757262	0.981843899	0.990825814
ENSG00000095066	111.7921112	-0.020835782	0.174535556	-0.119378436	0.904975544	0.949095974
ENSG00000095139	1261.752508	0.059352249	0.078635262	0.754779053	0.450381564	0.615349942
ENSG00000095203	17.26017895	-0.647425514	0.420805473	-1.538538719	0.123916949	0.248028125
ENSG00000095209	111.240466	0.581868459	0.173254076	3.358469082	0.000783755	0.003612852
ENSG00000095261	485.6628112	0.262995195	0.096478063	2.725958478	0.006411506	0.022074125
ENSG00000095319	1771.509544	0.1961913	0.067296701	2.91531822	0.00355326	0.013380933
ENSG00000095321	352.5333315	-0.091376242	0.111866757	-0.81683106	0.414024979	0.582431606
ENSG00000095370	10.07744505	-1.687547754	0.603252493	-2.797415299	0.005151327	0.018351685
ENSG00000095380	376.0585037	0.019116289	0.104116146	0.183605423	0.854323008	0.916431867
ENSG00000095383	18.35430705	-0.000327219	0.418247771	-0.000782358	0.999375769	0.999854605
ENSG00000095397	217.0365624	-0.23734247	0.142689467	-1.663349612	0.096242473	0.203602111
ENSG00000095485	104.8232359	-0.072272237	0.179522788	-0.402579741	0.687257416	0.802819536
ENSG00000095539	244.2941113	0.337851304	0.122180173	2.765189279	0.005688977	0.019928446
ENSG00000095564	741.0115025	-0.240319528	0.084176484	-2.854948514	0.004304381	0.015765781
ENSG00000095574	90.96114474	0.03875204	0.188928267	0.205115101	0.837482198	0.905074151
ENSG00000095587	10.50004	-0.091434601	0.525391584	-0.17403134	0.861840833	0.921610701
ENSG00000095596	11.17441191	-2.259249877	0.628188818	-3.596450325	0.000322589	0.001639439
ENSG00000095637	10.16179693	-0.168328953	0.540294601	-0.311550314	0.7553823	0.852072204
ENSG00000095739	10.02283495	-6.605470097	1.260051265	-5.242223298	1.59E-07	1.62E-06
ENSG00000095752	43.72839563	0.495564828	0.275250564	1.800413487	0.071795373	0.162035604

ENSG00000095787	1751.832887	0.011815545	0.065739401	0.179733085	0.857362118	0.918346431
ENSG00000095794	448.1691574	-0.429775673	0.102684437	-4.18540224	2.85E-05	0.000184866
ENSG00000095906	149.2452848	0.196329739	0.17058983	1.15088771	0.249778434	0.413789489
ENSG00000095951	360.0507492	-0.131363837	0.105369251	-1.246699928	0.212507545	0.36771292
ENSG00000096060	2166.630632	0.213505173	0.062523964	3.414773447	0.000638351	0.003009125
ENSG00000096063	1026.143311	-0.013247615	0.073527628	-0.180171931	0.857017594	0.918060409
ENSG00000096070	505.436196	-0.170052625	0.090142183	-1.886493312	0.059228509	0.139337274
ENSG00000096080	141.0994451	0.09814964	0.152564857	0.643330591	0.520009611	0.674538277
ENSG00000096092	11.05829146	-0.279140353	0.539310377	-0.51758758	0.604746049	0.741997252
ENSG00000096093	92.26652748	-0.332366349	0.202244941	-1.643385229	0.100303254	0.210198391
ENSG00000096384	9935.797542	0.326694701	0.051584595	6.333183409	2.40E-10	3.62E-09
ENSG00000096401	520.1632151	-0.113647148	0.092724884	-1.225638066	0.220334907	0.377905303
ENSG00000096433	636.6562925	0.610134099	0.090157713	6.76740875	1.31E-11	2.28E-10
ENSG00000096654	73.91836858	-0.173420717	0.206068572	-0.841568004	0.400029805	0.568171757
ENSG00000096696	440.8979657	-2.246838162	0.114713816	-19.58646525	2.02E-85	4.05E-83
ENSG00000096717	287.1289451	-0.331280301	0.112384339	-2.947744349	0.003201017	0.01220871
ENSG00000096746	3907.395815	0.284931206	0.056220931	5.068062797	4.02E-07	3.86E-06
ENSG00000096872	127.7470882	-0.115206986	0.168246033	-0.68475306	0.493499761	0.652748695
ENSG00000096968	76.25992854	-0.053389812	0.205234798	-0.260140153	0.794755666	0.877736681
ENSG00000097007	961.294854	0.158086358	0.075630783	2.090238278	0.036596401	0.094875041
ENSG00000097021	433.0130787	0.402635721	0.095260935	4.226661447	2.37E-05	0.000156935
ENSG00000097033	347.3603396	-0.36802586	0.106071886	-3.469589102	0.000521255	0.002513584
ENSG00000097046	303.3450814	-0.337810364	0.119268541	-2.83235094	0.00462071	0.016758874
ENSG00000097096	262.2385543	-0.690388356	0.123929786	-5.570802438	2.54E-08	2.94E-07
ENSG00000099194	7212.728582	-0.061326689	0.062949971	-0.974213146	0.329950719	0.502255933
ENSG00000099203	233.7798627	0.483498061	0.126262296	3.829314659	0.000128501	0.000721731
ENSG00000099204	519.2289364	0.298521641	0.098066173	3.04408373	0.002333902	0.009319021
ENSG00000099219	224.3603173	-0.749569409	0.12730269	-5.888087736	3.91E-09	5.04E-08
ENSG00000099246	348.3895572	0.168924832	0.108167832	1.561691944	0.118360577	0.239155787
ENSG00000099250	31.32137358	0.374159003	0.309639016	1.208371631	0.226904347	0.385361548
ENSG00000099256	72.02292985	-0.759325989	0.217371828	-3.49321251	0.000477247	0.002323703
ENSG00000099282	6.488996693	-3.528303913	1.00511098	-3.510362521	0.000447496	0.00219482
ENSG00000099284	360.8487002	-0.74713049	0.108761146	-6.869461391	6.44E-12	1.16E-10
ENSG00000099290	200.4849452	0.148618561	0.130728925	1.136845279	0.255602971	0.420970211
ENSG00000099308	534.5156306	-0.061315856	0.092693733	-0.66148869	0.508298966	0.665159127
ENSG00000099326	255.2521357	0.288872012	0.125618181	2.299603532	0.021470692	0.061163822
ENSG00000099330	137.2190764	-0.329648854	0.162627417	-2.027018943	0.042660475	0.107701599
ENSG00000099331	1089.135981	-0.04338	0.073729262	-0.588368838	0.556284749	0.703356436
ENSG00000099337	60.278902	0.297597108	0.225695359	1.318578765	0.187309977	0.335645005
ENSG00000099338	42.48259066	-0.299249603	0.291840359	-1.025388003	0.305180147	0.476159671
ENSG00000099341	1402.219639	0.166807579	0.070593395	2.362934629	0.018130867	0.053348198
ENSG00000099364	410.7488942	0.091675866	0.231690059	0.395683211	0.692338742	0.806274442
ENSG00000099365	76.88041977	-0.622128333	0.207034751	-3.004946421	0.002656276	0.010410914
ENSG00000099377	529.5694368	1.107757071	0.105451381	10.50490818	8.20E-26	3.81E-24
ENSG00000099381	825.3114344	0.055700294	0.079398754	0.701526042	0.482974789	0.643570018
ENSG00000099385	221.8065219	0.038517296	0.135768775	0.283697752	0.776642018	0.866107528
ENSG00000099622	828.3992133	-0.564154061	0.076544718	-7.370254588	1.70E-13	3.67E-12
ENSG00000099624	842.5227435	0.421968781	0.090426899	4.666407759	3.07E-06	2.45E-05
ENSG00000099625	214.3088719	0.521569007	0.132081586	3.948839689	7.85E-05	0.000463108
ENSG00000099783	2803.354425	0.283545484	0.061613375	4.602011876	4.18E-06	3.24E-05

ENSG00000099785	22.64617032	0.039823469	0.379935632	0.104816357	0.916521531	0.954800179
ENSG00000099795	284.2626955	0.073702431	0.114529101	0.643525801	0.519882979	0.674538277
ENSG00000099797	506.7038872	-0.068567992	0.099674273	-0.687920661	0.491502745	0.651150276
ENSG00000099800	588.8659653	0.20974769	0.08492442	2.469815975	0.013518257	0.041643871
ENSG00000099804	408.7428751	0.191459595	0.104770096	1.827425968	0.067635748	0.154988475
ENSG00000099810	349.3001239	-0.048217176	0.102402076	-0.470861313	0.637739776	0.766193178
ENSG00000099814	261.294735	0.503191482	0.123576751	4.071894402	4.66E-05	0.00028933
ENSG00000099817	603.4806159	-0.009282516	0.087842127	-0.105672711	0.915842033	0.954435272
ENSG00000099821	350.1070502	0.223703194	0.110553445	2.023484603	0.043023209	0.108519846
ENSG00000099822	32.2007709	-1.24488647	0.319312242	-3.898649366	9.67E-05	0.000558248
ENSG00000099849	38.87816801	-0.135403774	0.295206422	-0.458674895	0.646467645	0.772319358
ENSG00000099860	58.79708086	0.815397577	0.249193779	3.272142588	0.001067357	0.00476276
ENSG00000099864	349.3465639	-0.032217309	0.115671257	-0.27852476	0.780609561	0.868601276
ENSG00000099875	1392.33195	0.242988757	0.078551317	3.093375996	0.001978932	0.008104391
ENSG00000099889	56.85499847	-0.660295594	0.23466778	-2.813746287	0.004896786	0.017587612
ENSG00000099899	352.4218815	0.375825164	0.105839473	3.550897911	0.000383919	0.00191594
ENSG00000099901	1188.601013	-0.014276082	0.070924992	-0.201284224	0.840476337	0.907329474
ENSG00000099904	391.3275849	0.418076374	0.116323403	3.594086523	0.000325532	0.001652887
ENSG00000099910	161.4537948	0.012354992	0.168555726	0.073299156	0.941568063	0.967676806
ENSG00000099917	454.0320804	0.254115431	0.093928952	2.705400473	0.006822209	0.023279898
ENSG00000099940	278.4594675	-0.113932327	0.116215162	-0.980356828	0.326910012	0.499087593
ENSG00000099942	740.0733047	-0.064208203	0.078802257	-0.814801573	0.415185901	0.583209876
ENSG00000099949	125.6538281	0.431469952	0.162630405	2.653070632	0.007976318	0.026696053
ENSG00000099953	74.68025757	-0.153417312	0.225810606	-0.679407023	0.496880001	0.655429935
ENSG00000099954	601.3498956	-0.667877046	0.090863172	-7.350360236	1.98E-13	4.21E-12
ENSG00000099956	573.6023819	0.160573716	0.090155296	1.78107912	0.074899526	0.167294267
ENSG00000099958	36.79394979	1.097644907	0.306651492	3.579453989	0.000344313	0.001738992
ENSG00000099968	339.4104836	-0.390791132	0.105571996	-3.701655244	0.000214198	0.001138416
ENSG00000099977	64.17806885	0.39730484	0.222047001	1.78928262	0.073569312	0.16502283
ENSG00000099991	1047.825029	-0.388976566	0.07157044	-5.434877386	5.48E-08	6.04E-07
ENSG00000099992	44.35745932	0.326325909	0.275532246	1.184347438	0.236275558	0.39748057
ENSG00000099994	17.71600859	-0.54249526	0.411404055	-1.318643444	0.187288342	0.335645005
ENSG00000099995	1765.266755	0.157949653	0.069986224	2.256867755	0.024016339	0.067042432
ENSG00000099999	59.60015686	0.169629557	0.229127619	0.740327847	0.459101088	0.62242959
ENSG00000100003	33.01289562	0.184215528	0.309801096	0.594625165	0.552094055	0.699491764
ENSG00000100014	141.8783455	-0.181086359	0.157125433	-1.152495528	0.249117506	0.413244404
ENSG00000100023	399.7056145	0.202278723	0.099337127	2.036285208	0.041721724	0.105768825
ENSG00000100028	214.2288621	-0.07442184	0.125656396	-0.592264641	0.553673389	0.700923765
ENSG00000100029	1040.271463	0.410316818	0.071456499	5.742190321	9.35E-09	1.15E-07
ENSG00000100030	693.9673949	-0.0160373	0.084666711	-0.189416827	0.849766137	0.914037896
ENSG00000100031	23.41237938	0.677739543	0.376592161	1.799664501	0.07191363	0.162224306
ENSG00000100034	355.131094	0.344558753	0.108660428	3.170968129	0.001519318	0.006459401
ENSG00000100036	63.68014163	1.449957132	0.229020222	6.331131453	2.43E-10	3.66E-09
ENSG00000100038	141.0952925	-0.220202371	0.15290626	-1.440113508	0.149835288	0.2848087
ENSG00000100056	105.9176932	0.054874135	0.17632772	0.31120538	0.755644493	0.852102835
ENSG00000100065	39.42377394	-0.414992311	0.281922118	-1.472010473	0.14101805	0.272302901
ENSG00000100075	766.4892674	0.447568949	0.099008327	4.520518252	6.17E-06	4.64E-05
ENSG00000100077	203.6370207	-0.473010961	0.130599973	-3.621830481	0.000292526	0.001504067
ENSG00000100078	232.1357231	0.77289293	0.148024941	5.22136961	1.78E-07	1.80E-06
ENSG00000100083	444.3644024	0.05874983	0.112113816	0.524019538	0.600264951	0.738799769

ENSG00000100084	152.7615723	0.349214599	0.158100346	2.208816155	0.027187431	0.074357835
ENSG00000100092	207.4275797	0.289225247	0.132442	2.183787974	0.028977832	0.078309273
ENSG00000100097	2735.347088	0.199996601	0.06037011	3.31284139	0.000923533	0.004191558
ENSG00000100099	313.7802626	0.01348277	0.109717955	0.122885722	0.902197592	0.947258155
ENSG00000100100	64.46025679	0.454911253	0.21689456	2.097384333	0.035959567	0.093530499
ENSG00000100104	162.0920596	0.426943168	0.14320245	2.981395684	0.002869378	0.011118938
ENSG00000100105	392.6428288	0.18798459	0.116002808	1.620517588	0.105121139	0.218082505
ENSG00000100106	721.5258983	-0.192709785	0.08610057	-2.238194076	0.025208402	0.069769194
ENSG00000100109	146.9842758	0.09878564	0.151287559	0.652966049	0.513778166	0.669670771
ENSG00000100116	85.91860316	-0.785861561	0.201706849	-3.896057901	9.78E-05	0.000563326
ENSG00000100124	241.6969963	-0.228230486	0.124180168	-1.837898029	0.066077433	0.152062532
ENSG00000100129	1063.052375	0.017882198	0.075636351	0.236423333	0.813104193	0.890231847
ENSG00000100138	849.0572154	0.566012441	0.081400468	6.953429808	3.57E-12	6.68E-11
ENSG00000100139	254.3395206	0.324156044	0.120837678	2.682574252	0.007305793	0.024740713
ENSG00000100142	759.4047994	0.445315319	0.082003845	5.430420009	5.62E-08	6.17E-07
ENSG00000100147	288.3251786	-0.025361266	0.110395184	-0.229731631	0.818300314	0.892848047
ENSG00000100150	201.2530498	0.162684681	0.129986385	1.2515517	0.210733264	0.36590484
ENSG00000100151	111.1339846	0.089567999	0.17409867	0.514466877	0.606925613	0.743700634
ENSG00000100154	845.0358439	-0.074196814	0.075384092	-0.984250294	0.324992463	0.496998182
ENSG00000100162	122.783739	-0.043630656	0.176960977	-0.24655524	0.805252448	0.884946107
ENSG00000100167	35.28840203	-0.160877995	0.309366696	-0.520023639	0.603047099	0.741060465
ENSG00000100196	189.9481942	0.555992852	0.13619704	4.082268241	4.46E-05	0.000277446
ENSG00000100201	2986.431079	0.02026701	0.068017632	0.297966997	0.765728352	0.858773078
ENSG00000100206	62.64843699	-0.773545546	0.228165149	-3.390287906	0.000698193	0.003264817
ENSG00000100207	1242.479287	0.042615566	0.072274379	0.589635871	0.555434798	0.702516337
ENSG00000100209	41.32334162	-0.440509385	0.276459834	-1.593393798	0.111071876	0.22770383
ENSG00000100211	89.87912504	0.245655866	0.187193069	1.312312826	0.189414606	0.338727814
ENSG00000100216	638.799726	0.311548288	0.086711425	3.592932405	0.000326977	0.001657543
ENSG00000100219	384.3753896	-0.259122943	0.107364213	-2.413494557	0.015800364	0.047618046
ENSG00000100220	316.7744112	0.117347442	0.110938289	1.057772232	0.290159295	0.460445958
ENSG00000100221	506.0832744	0.226306627	0.103267734	2.191455344	0.028418858	0.077064333
ENSG00000100225	552.512128	0.119708761	0.088996868	1.345089589	0.178596305	0.324336431
ENSG00000100226	627.3803805	-0.010279846	0.094570645	-0.108700181	0.913440298	0.953417734
ENSG00000100228	80.47226203	-0.180717511	0.195711097	-0.923389189	0.355804424	0.527227053
ENSG00000100239	400.8980186	0.275566411	0.100098735	2.752945995	0.005906164	0.020586571
ENSG00000100241	613.3350597	-0.096667178	0.092046153	-1.050203344	0.293624632	0.463987641
ENSG00000100242	253.307469	0.395409077	0.132727991	2.97909336	0.002891027	0.011184316
ENSG00000100243	582.1196243	-0.051403939	0.107306238	-0.479039615	0.631910447	0.761730545
ENSG00000100246	40.09908053	-0.137490502	0.283481202	-0.485007475	0.627671043	0.758494295
ENSG00000100258	300.0526141	0.260394094	0.12558078	2.073518684	0.038124034	0.097974443
ENSG00000100263	106.3190884	0.350941781	0.173835982	2.018809787	0.043506993	0.109366989
ENSG00000100266	275.0738845	-0.143704768	0.113855342	-1.262169745	0.206887713	0.360788542
ENSG00000100271	33.16584478	-0.248991572	0.31625259	-0.787318681	0.431095337	0.597552486
ENSG00000100276	29.6308986	-0.111162463	0.321670034	-0.345579169	0.729659003	0.832888023
ENSG00000100280	900.4882364	0.098233878	0.075947815	1.293439163	0.195859213	0.347068876
ENSG00000100281	413.6053565	-0.100737815	0.097006379	-1.038465884	0.29905321	0.469468087
ENSG00000100284	119.3129875	0.267702193	0.1643713	1.628643153	0.103388583	0.21521924
ENSG00000100285	3454.278705	0.790044743	0.060516248	13.05508473	5.94E-39	4.49E-37
ENSG00000100292	172.8016613	1.396433524	0.148739177	9.38847151	6.09E-21	2.18E-19
ENSG00000100294	143.757103	0.251330574	0.164700452	1.525985935	0.127013367	0.252210352

ENSG00000100296	709.0063314	0.461313587	0.083656778	5.514359983	3.50E-08	3.98E-07
ENSG00000100297	1165.410571	0.18102572	0.070621354	2.563328383	0.010367394	0.033309877
ENSG00000100299	48.56030119	0.005376012	0.263642166	0.020391322	0.983731206	0.991961125
ENSG00000100300	377.1186835	0.830298005	0.215824342	3.847100834	0.000119524	0.000677543
ENSG00000100304	749.7997661	0.439788122	0.08378873	5.24877415	1.53E-07	1.57E-06
ENSG00000100307	62.40284755	0.406392186	0.227761854	1.78428555	0.074377277	0.166365862
ENSG00000100316	4780.545424	0.270227123	0.05739441	4.7082481	2.50E-06	2.04E-05
ENSG00000100319	64.6711839	0.2656577	0.223989652	1.186026664	0.23561177	0.396663823
ENSG00000100320	1590.7094	-0.471495551	0.069146386	-6.818802491	9.18E-12	1.63E-10
ENSG00000100321	105.2538413	0.150626048	0.187876268	0.801730044	0.422709132	0.590003496
ENSG00000100324	129.8664534	-0.086190398	0.167543375	-0.514436324	0.606946969	0.743700634
ENSG00000100325	391.0411702	0.279266183	0.11006527	2.537277955	0.011171821	0.035440509
ENSG00000100330	311.5781761	0.024567557	0.112429458	0.218515301	0.827027638	0.898668717
ENSG00000100335	451.2537878	0.134600354	0.094138073	1.429818447	0.152769133	0.289055498
ENSG00000100344	135.7502977	0.696340047	0.157367843	4.424919573	9.65E-06	6.94E-05
ENSG00000100345	5042.172343	0.158490248	0.054295427	2.919034957	0.003511168	0.01324064
ENSG00000100347	295.9178491	-0.063657742	0.12321188	-0.516652634	0.605398666	0.742455469
ENSG00000100348	630.9609435	0.443091729	0.090808502	4.879407988	1.06E-06	9.37E-06
ENSG00000100350	375.3124148	0.241755375	0.106608772	2.267687452	0.023348263	0.065489827
ENSG00000100353	1470.980345	0.330160916	0.081054211	4.073334516	4.63E-05	0.000287801
ENSG00000100354	1979.150756	-0.382326806	0.062989769	-6.06966519	1.28E-09	1.76E-08
ENSG00000100359	10.00807819	0.094107153	0.551215966	0.170726464	0.864438853	0.923615469
ENSG00000100360	127.716778	-0.798840081	0.16610261	-4.809316849	1.51E-06	1.29E-05
ENSG00000100364	335.1459401	0.307818292	0.107991499	2.850393724	0.004366514	0.015960557
ENSG00000100372	97.02359133	0.224974302	0.184783901	1.217499473	0.223414237	0.381788295
ENSG00000100376	136.3787036	0.171606209	0.165159229	1.039034937	0.298788488	0.469262287
ENSG00000100379	83.39358931	0.039315302	0.196654986	0.19992019	0.841543	0.907853046
ENSG00000100380	1541.39313	0.178238207	0.071005893	2.51018893	0.012066658	0.037851797
ENSG00000100387	229.8207891	0.117262303	0.121536309	0.964833512	0.334628208	0.50630358
ENSG00000100393	2154.296334	-0.093358018	0.063634303	-1.467102075	0.14234829	0.274326578
ENSG00000100395	277.725581	-0.107138822	0.112115874	-0.955607957	0.33927034	0.511070291
ENSG00000100401	584.5747675	0.103312031	0.091861755	1.124646826	0.260738723	0.427025398
ENSG00000100403	984.0958831	0.180415788	0.081255347	2.220355885	0.026394619	0.072585203
ENSG00000100410	280.9178488	0.1334325	0.11311933	1.179572933	0.238170114	0.399662595
ENSG00000100412	1842.478689	-0.056558095	0.061699341	-0.916672597	0.35931423	0.530567328
ENSG00000100413	260.6130393	-0.011104117	0.120256148	-0.092337209	0.926430126	0.959773138
ENSG00000100416	251.8810492	0.084042212	0.11958107	0.702805319	0.482177082	0.642735004
ENSG00000100417	97.79940832	-0.183553236	0.179390069	-1.023207347	0.30620982	0.477289097
ENSG00000100418	642.7511938	0.33282259	0.083337308	3.993680625	6.51E-05	0.000390694
ENSG00000100422	354.6153693	-0.420229656	0.10370096	-4.052321758	5.07E-05	0.000311614
ENSG00000100425	775.960049	0.018061479	0.080615841	0.224043801	0.822723221	0.896348169
ENSG00000100426	565.9523548	0.043313661	0.08591448	0.504148554	0.61415699	0.748942029
ENSG00000100427	151.4116218	-3.893627937	0.233887588	-16.64743292	3.16E-62	4.44E-60
ENSG00000100429	35.32131176	0.036376573	0.296611984	0.122640268	0.902391966	0.947258155
ENSG00000100439	103.85817	0.148050563	0.188892079	0.783783864	0.433166944	0.599418388
ENSG00000100441	413.587523	-0.13992377	0.099658563	-1.404031579	0.160309446	0.299330957
ENSG00000100442	347.7255564	-0.172774087	0.10373579	-1.665520526	0.095808952	0.203021323
ENSG00000100445	174.2755192	0.285122075	0.14798616	1.92668067	0.054019438	0.129556266
ENSG00000100461	370.1987124	-0.223490196	0.105132557	-2.125794356	0.033520386	0.08820431
ENSG00000100462	1137.534298	0.42748366	0.082677849	5.170473901	2.34E-07	2.33E-06

ENSG00000100478	37.09092326	-0.265622794	0.283156583	-0.938077411	0.34820463	0.520177649
ENSG00000100479	169.3325083	-0.195316368	0.14210255	-1.37447476	0.169294341	0.31187903
ENSG00000100483	41.75161475	0.098769168	0.274613792	0.359665722	0.719097128	0.825140781
ENSG00000100485	194.5135219	-0.056932106	0.135142743	-0.421273867	0.673555111	0.792016461
ENSG00000100490	53.39290836	-0.058259709	0.247686482	-0.235215537	0.814041447	0.8908414
ENSG00000100503	1570.34504	0.406784098	0.070538276	5.766856275	8.08E-09	1.01E-07
ENSG00000100504	1352.288186	0.335769081	0.068881475	4.874591934	1.09E-06	9.56E-06
ENSG00000100505	314.7297138	0.383476804	0.112667045	3.403628855	0.00066497	0.003122982
ENSG00000100519	286.4259065	0.533919086	0.113653289	4.697788247	2.63E-06	2.14E-05
ENSG00000100522	393.1671314	0.176430998	0.104027486	1.696003666	0.089885186	0.193119793
ENSG00000100523	403.3996888	0.177331049	0.100644219	1.761959608	0.078076122	0.173274477
ENSG00000100526	200.9659171	0.007270259	0.136127071	0.053407886	0.957406922	0.977076206
ENSG00000100528	470.9073633	-0.172916007	0.09241231	-1.871136073	0.061326223	0.143312169
ENSG00000100532	11.37147304	-0.337708168	0.52189747	-0.647077611	0.517581715	0.67256895
ENSG00000100554	113.4310073	-0.021397978	0.166217288	-0.128734975	0.897567359	0.944486149
ENSG00000100564	44.69543899	0.405793863	0.260037171	1.560522526	0.118636451	0.23960981
ENSG00000100567	207.4093549	0.238619241	0.140533852	1.69794849	0.089517484	0.192542585
ENSG00000100568	293.7606823	-0.397904581	0.120517896	-3.301622376	0.000961274	0.004336228
ENSG00000100575	134.5746014	-0.039249347	0.16060716	-0.244381057	0.806935711	0.8858602
ENSG00000100577	113.4786793	-0.344733015	0.178115878	-1.935442356	0.052936036	0.127458433
ENSG00000100578	287.8032218	-0.314390263	0.112831016	-2.786381559	0.005330009	0.018880375
ENSG00000100580	452.9964496	0.197859372	0.096399728	2.052488898	0.040122173	0.102026822
ENSG00000100583	8.094160665	-0.205494457	0.610151504	-0.336792511	0.736273319	0.837783014
ENSG00000100591	1047.746525	0.278636557	0.076225558	3.655421683	0.00025676	0.001337301
ENSG00000100592	62.8281723	-0.53789499	0.222731445	-2.414993489	0.015735491	0.047442894
ENSG00000100593	25.75705616	0.627171606	0.340952554	1.839468865	0.065846253	0.15167962
ENSG00000100596	349.16138	-0.572824876	0.104665142	-5.472928867	4.43E-08	4.93E-07
ENSG00000100600	406.1056872	0.160545504	0.106353607	1.509544513	0.131159689	0.25843739
ENSG00000100601	70.09987811	-0.021726604	0.220440569	-0.098559916	0.921487697	0.956727767
ENSG00000100603	496.5931004	0.066481946	0.092268315	0.720528451	0.471199689	0.633139543
ENSG00000100605	437.6687624	-0.134075494	0.099602388	-1.346107226	0.178267939	0.323966351
ENSG00000100612	140.0237176	-0.743485849	0.15912926	-4.672213328	2.98E-06	2.39E-05
ENSG00000100614	272.936981	-0.470010448	0.119898839	-3.920058366	8.85E-05	0.000515994
ENSG00000100625	460.4462157	-2.115980325	0.108610431	-19.48229371	1.55E-84	3.07E-82
ENSG00000100626	50.68860658	-3.155840858	0.345586168	-9.131849439	6.73E-20	2.26E-18
ENSG00000100629	375.6571046	-0.497205933	0.114457222	-4.344032857	1.40E-05	9.73E-05
ENSG00000100632	557.0253707	0.189358098	0.100331997	1.887315154	0.059117949	0.139100475
ENSG00000100644	1039.795212	-0.45951616	0.083224457	-5.521407715	3.36E-08	3.83E-07
ENSG00000100647	69.07206786	-0.63328875	0.220777133	-2.868452648	0.004124849	0.015215798
ENSG00000100650	2064.555924	0.032068885	0.059605759	0.538016562	0.590565615	0.731670065
ENSG00000100664	887.7305848	0.127719809	0.080784946	1.580985256	0.113881409	0.23194284
ENSG00000100697	1003.878378	-0.139448567	0.075427436	-1.848777763	0.06448991	0.14918545
ENSG00000100711	183.4927747	0.43313765	0.138153261	3.135196721	0.001717389	0.007181948
ENSG00000100714	1164.055163	-0.127017272	0.068913855	-1.843131136	0.065309866	0.150715902
ENSG00000100722	561.8897244	-0.20026911	0.086383929	-2.318360741	0.020429724	0.058806625
ENSG00000100726	263.2323451	0.665378883	0.135154309	4.923105228	8.52E-07	7.66E-06
ENSG00000100731	925.0338117	-0.115216562	0.078401863	-1.469564089	0.141679852	0.273300863
ENSG00000100744	43.7404952	0.001808502	0.271049418	0.006672221	0.994676377	0.99758811
ENSG00000100749	309.6087516	-0.022274497	0.109247036	-0.203891092	0.838438606	0.905825804
ENSG00000100764	184.0670517	0.555132311	0.136765275	4.059015067	4.93E-05	0.000304013

ENSG00000100784	69.70827901	0.260979035	0.226765254	1.15087753	0.249782623	0.413789489
ENSG00000100796	857.9898966	0.010968895	0.082780055	0.132506499	0.894583683	0.942843412
ENSG00000100802	88.69370735	-0.22982493	0.189913432	-1.210156265	0.226218936	0.384569454
ENSG00000100804	997.2836652	0.197827017	0.07312411	2.70535967	0.006823047	0.023279898
ENSG00000100811	1074.75679	0.132244376	0.073416241	1.801295918	0.071656251	0.161877671
ENSG00000100813	2170.770249	0.173495035	0.061440708	2.823779895	0.004746097	0.017125204
ENSG00000100814	274.5257092	-0.366165525	0.118404619	-3.092493592	0.001984825	0.008121421
ENSG00000100815	772.6526934	-0.043129238	0.080664902	-0.534671678	0.592876909	0.733089931
ENSG00000100823	1549.340129	0.343261748	0.064839172	5.29404893	1.20E-07	1.25E-06
ENSG00000100836	252.2637458	0.1940055	0.136951782	1.416597122	0.15660075	0.29436247
ENSG00000100852	764.3111157	-0.210151479	0.079766074	-2.634597236	0.008423717	0.027980127
ENSG00000100865	153.0474825	0.130261443	0.157160567	0.828843049	0.407193225	0.57561067
ENSG00000100867	195.5816881	-0.697438262	0.13598996	-5.128601117	2.92E-07	2.85E-06
ENSG00000100883	336.7993479	-0.01645669	0.107308871	-0.153358152	0.878115845	0.932836215
ENSG00000100888	1772.252687	-0.148290538	0.067344899	-2.201956488	0.027668382	0.075438231
ENSG00000100889	523.0796959	-0.837208711	0.09278841	-9.022772508	1.83E-19	6.05E-18
ENSG00000100890	65.36510658	-0.38102075	0.218374605	-1.744803385	0.081019099	0.178297868
ENSG00000100897	160.5173401	-0.27259193	0.143791871	-1.895739508	0.057994498	0.136938766
ENSG00000100902	100.8788806	0.308932004	0.2092346	1.476486219	0.139813412	0.27068438
ENSG00000100906	250.8471193	0.367770638	0.119100846	3.087892746	0.002015812	0.008231428
ENSG00000100908	66.14753012	0.571643239	0.236886553	2.413151917	0.015815226	0.047652613
ENSG00000100911	514.4657819	0.287705703	0.105742655	2.720810273	0.006512213	0.022371497
ENSG00000100916	119.8347415	0.176555222	0.166548528	1.060082754	0.289106953	0.459294885
ENSG00000100918	28.00358117	0.734682547	0.35768591	2.053987941	0.039976859	0.101767839
ENSG00000100926	36.19574501	-0.023135335	0.286881575	-0.080644198	0.935724916	0.964731953
ENSG00000100934	717.8434612	-0.471755744	0.090519991	-5.211619419	1.87E-07	1.89E-06
ENSG00000100938	220.8680367	-0.223639628	0.124772598	-1.79237775	0.073072476	0.164179315
ENSG00000100941	2724.119729	0.076719458	0.059434943	1.290814018	0.196768176	0.347995448
ENSG00000100949	203.46094	0.330115302	0.14442856	2.285664991	0.022273865	0.062890649
ENSG00000100968	321.4991799	0.239986011	0.107411476	2.234267888	0.025465452	0.070402126
ENSG00000100979	970.6198218	0.068304671	0.07448415	0.91703632	0.359123608	0.530453136
ENSG00000100982	224.2285294	-0.234788949	0.132199171	-1.776024362	0.075728902	0.168797824
ENSG00000100983	339.9187865	0.209108605	0.10715736	1.951416177	0.051007559	0.123705659
ENSG00000100991	425.8565891	0.096062742	0.100675519	0.954181735	0.339991659	0.511717865
ENSG00000100994	254.9892398	-0.075116395	0.131886491	-0.569553371	0.568980662	0.71437892
ENSG00000100997	273.0140869	0.320981927	0.120187714	2.670671698	0.007569965	0.025524554
ENSG00000101000	384.2248305	0.844848688	0.10313101	8.191994735	2.57E-16	6.82E-15
ENSG00000101003	208.8161641	-0.58370819	0.141267571	-4.131933365	3.60E-05	0.000228645
ENSG00000101004	411.2047777	0.010171912	0.111743891	0.091028796	0.92746971	0.960497968
ENSG00000101017	63.731405	-1.083369838	0.234844473	-4.613137462	3.97E-06	3.10E-05
ENSG00000101019	159.724212	-0.032011581	0.143132554	-0.223649897	0.823029735	0.896348169
ENSG00000101040	1143.651383	-0.57297482	0.071792948	-7.980934589	1.45E-15	3.67E-14
ENSG00000101052	61.16942561	-0.267577054	0.228765265	-1.169657698	0.242138748	0.404832326
ENSG00000101057	709.3222984	-0.11378193	0.086260453	-1.319050923	0.187152089	0.335450478
ENSG00000101079	238.1481027	0.525479438	0.127734163	4.113851971	3.89E-05	0.000245325
ENSG00000101084	66.05556113	0.274535855	0.215345356	1.27486313	0.202357691	0.355004182
ENSG00000101096	229.119004	-1.319382001	0.135840034	-9.712762571	2.66E-22	1.02E-20
ENSG00000101098	66.85806433	-0.943298976	0.229838263	-4.104185982	4.06E-05	0.000254895
ENSG00000101104	502.0671507	0.389280921	0.102842764	3.785204774	0.000153582	0.00084669
ENSG00000101109	595.1329706	0.129301579	0.090045764	1.435954047	0.151015418	0.28658654

ENSG00000101115	929.5383262	-2.050805457	0.082755625	-24.78146283	1.42E-135	6.24E-133
ENSG00000101126	1023.025747	0.021251196	0.07793104	0.272692319	0.78508974	0.871377653
ENSG00000101132	132.6779163	0.134001722	0.160028779	0.837360148	0.402390159	0.570658715
ENSG00000101138	270.0806948	-0.001227246	0.114929477	-0.010678254	0.991480148	0.995624671
ENSG00000101144	609.0159259	-0.104269843	0.086689583	-1.202795538	0.229055452	0.387861666
ENSG00000101146	258.7077105	0.071532034	0.122727669	0.582851727	0.55999312	0.706631635
ENSG00000101150	526.0330351	0.453788493	0.087911487	5.161879391	2.44E-07	2.43E-06
ENSG00000101152	218.2350806	0.062682042	0.126755593	0.494511052	0.620945303	0.753988302
ENSG00000101158	928.9389686	0.067416891	0.097685528	0.690142054	0.490104859	0.649726591
ENSG00000101160	1113.643945	-0.086380256	0.070987419	-1.21683895	0.223665497	0.382038284
ENSG00000101161	756.9710459	0.317466421	0.078976443	4.019760945	5.83E-05	0.000352429
ENSG00000101166	723.6203969	-0.348517659	0.079978218	-4.357657227	1.31E-05	9.18E-05
ENSG00000101181	174.5885916	0.114042284	0.14398372	0.792049852	0.428331609	0.595484374
ENSG00000101182	521.0789189	0.353621955	0.093034387	3.800981214	0.000144124	0.000799571
ENSG00000101187	213.8277946	0.369814988	0.135345182	2.732383843	0.006287784	0.021696021
ENSG00000101189	265.4334806	0.357480514	0.123777655	2.888086021	0.003875939	0.014392099
ENSG00000101190	95.70138249	0.278650763	0.181207862	1.537741022	0.124111949	0.248159578
ENSG00000101191	902.8472072	-0.222913857	0.080939787	-2.754070217	0.005885914	0.020551686
ENSG00000101193	305.2306013	-0.02017598	0.111186864	-0.181460104	0.856006446	0.917537012
ENSG00000101199	437.1188694	0.199000229	0.118429945	1.680320202	0.092895033	0.198222167
ENSG00000101204	15.24596622	-2.471932399	0.577363285	-4.281415987	1.86E-05	0.000125476
ENSG00000101210	2481.83688	0.592573902	0.065433696	9.056097059	1.35E-19	4.48E-18
ENSG00000101216	114.5665565	0.319751179	0.167789917	1.905663843	0.056693833	0.134614313
ENSG00000101220	112.1744457	0.241689124	0.18109867	1.334571501	0.182016631	0.328804991
ENSG00000101224	686.2989809	0.225166548	0.081354663	2.767715319	0.005645074	0.019804285
ENSG00000101236	99.50382945	0.623194157	0.181725202	3.429321575	0.000605092	0.002873472
ENSG00000101246	121.5663628	-0.38436672	0.173523656	-2.215068129	0.026755389	0.073433557
ENSG00000101247	197.438892	0.058558071	0.133059638	0.440088907	0.659872716	0.782521221
ENSG00000101255	169.8439223	-0.215742265	0.151799501	-1.421231711	0.155249409	0.292709201
ENSG00000101265	11.83550395	0.796475913	0.505513039	1.575579366	0.115122774	0.233988755
ENSG00000101266	1554.796634	-0.085759216	0.067432725	-1.271774444	0.203453272	0.356436531
ENSG00000101290	638.3317056	-0.470683148	0.085731284	-5.490214612	4.01E-08	4.52E-07
ENSG00000101294	778.4176549	0.458865312	0.077644886	5.909794404	3.43E-09	4.44E-08
ENSG00000101298	68.91474938	-0.754540755	0.214985025	-3.509736335	0.000448551	0.002198465
ENSG00000101310	498.4314531	0.299516447	0.100761034	2.972542414	0.002953444	0.011378779
ENSG00000101311	244.9221655	0.539128087	0.129731086	4.155735548	3.24E-05	0.00020826
ENSG00000101335	2800.851227	-0.173190285	0.073720926	-2.349268984	0.018810313	0.054898912
ENSG00000101337	702.8515043	0.060776449	0.080188381	0.757920891	0.448498343	0.613502407
ENSG00000101343	233.3704214	0.316335558	0.123940042	2.552327342	0.010700593	0.034130616
ENSG00000101346	475.0356601	0.438490456	0.091495423	4.792485159	1.65E-06	1.39E-05
ENSG00000101347	252.2224434	0.786885211	0.119996531	6.557566299	5.47E-11	8.99E-10
ENSG00000101350	224.7029821	-0.033025107	0.125592383	-0.262954699	0.792585496	0.876311364
ENSG00000101353	12.09308813	0.029013432	0.493972303	0.058734937	0.953163232	0.974299858
ENSG00000101361	1207.82474	0.17363579	0.07264758	2.39011115	0.016843275	0.050158465
ENSG00000101363	288.5518704	0.318480739	0.116706125	2.728911957	0.006354367	0.021890896
ENSG00000101365	928.882202	-0.172647349	0.090417094	-1.909454737	0.056203455	0.133653282
ENSG00000101367	850.6064833	-0.348288859	0.079699499	-4.370025728	1.24E-05	8.72E-05
ENSG00000101384	8.810961642	-3.989728563	0.973975687	-4.096332812	4.20E-05	0.000262753
ENSG00000101391	189.7165311	0.071569714	0.135166758	0.52949198	0.596464206	0.73589786
ENSG00000101400	98.20540556	0.014166918	0.189416398	0.074792457	0.940379844	0.967234068

ENSG00000101407	383.8760941	-0.055141847	0.105374973	-0.523291682	0.600771289	0.739163904
ENSG00000101412	175.2261135	-0.426708802	0.148132631	-2.880586123	0.003969365	0.014715669
ENSG00000101413	283.4975868	-0.034178358	0.111520257	-0.306476679	0.759241734	0.854860493
ENSG00000101417	38.57418238	-0.33077989	0.284533954	-1.162532221	0.245019344	0.408472197
ENSG00000101421	280.709625	0.261580619	0.112965282	2.31558417	0.020580984	0.059157168
ENSG00000101439	1205.803477	0.523801667	0.070570269	7.422412805	1.15E-13	2.51E-12
ENSG00000101442	29.4053182	0.646497198	0.322451873	2.004941672	0.044969283	0.112174629
ENSG00000101443	28.12976051	-5.703544478	0.87941954	-6.48557852	8.84E-11	1.41E-09
ENSG00000101444	1245.164553	-0.372539863	0.073149196	-5.092877075	3.53E-07	3.41E-06
ENSG00000101447	166.9130984	-0.087323875	0.151726365	-0.575535275	0.564929341	0.710817131
ENSG00000101452	109.6890528	0.107012524	0.173871359	0.615469532	0.538244684	0.688933648
ENSG00000101457	162.5412345	0.180200156	0.158101569	1.139774622	0.254380209	0.41939892
ENSG00000101460	19.94018689	0.578679359	0.386320317	1.497926292	0.134152402	0.262749413
ENSG00000101464	139.4177723	0.215834585	0.153217631	1.408679816	0.158929866	0.297506373
ENSG00000101473	34.39388045	-0.125237801	0.296101152	-0.422956142	0.67232726	0.791168721
ENSG00000101474	525.8284375	0.563431094	0.088110331	6.394608786	1.61E-10	2.47E-09
ENSG00000101493	1251.028976	-0.424907693	0.077525572	-5.480871433	4.23E-08	4.74E-07
ENSG00000101544	575.0421719	-0.104858191	0.091517299	-1.145774538	0.251888453	0.416463877
ENSG00000101546	93.51103873	0.56314377	0.18984271	2.966370262	0.003013375	0.011584257
ENSG00000101557	325.5450026	0.170758279	0.104744578	1.630235025	0.103051834	0.214677312
ENSG00000101558	526.0795647	0.119948083	0.089545528	1.339520631	0.180401243	0.326642983
ENSG00000101574	60.9634249	-0.405557629	0.230456888	-1.759798258	0.078442019	0.173879601
ENSG00000101577	329.5329016	0.047437415	0.116639868	0.406699837	0.684228461	0.800412824
ENSG00000101596	1356.049514	-0.002359168	0.06538326	-0.036082147	0.971216858	0.985031278
ENSG00000101605	19.66693734	-1.157320945	0.414463094	-2.792337756	0.00523287	0.018604436
ENSG00000101608	465.3557463	-0.451360286	0.092375998	-4.886120786	1.03E-06	9.09E-06
ENSG00000101624	98.49652138	0.077680937	0.178607733	0.434924823	0.663616996	0.784929938
ENSG00000101639	640.168336	-0.276613802	0.083647653	-3.306892578	0.000943371	0.004266534
ENSG00000101654	322.4106096	0.325612781	0.106454411	3.058706319	0.00222295	0.008939528
ENSG00000101665	63.22432317	-0.299468541	0.230507735	-1.299169159	0.193885883	0.34476587
ENSG00000101670	563.9427189	0.20245169	0.088031855	2.299754898	0.02146211	0.061163822
ENSG00000101695	36.86680795	0.335210122	0.286646725	1.169418986	0.242234864	0.404918311
ENSG00000101745	607.0740451	-0.021761035	0.09527621	-0.228399465	0.819335706	0.893395096
ENSG00000101751	117.4768721	-0.234779948	0.165383057	-1.41961306	0.155720361	0.293217683
ENSG00000101752	1087.49427	-0.175638146	0.078156069	-2.247274577	0.024622485	0.068421769
ENSG00000101773	325.2361792	0.138723032	0.109514777	1.266706064	0.205260411	0.358682061
ENSG00000101782	115.7812073	-0.394323383	0.181952205	-2.167181121	0.030221046	0.08101432
ENSG00000101811	213.6644985	0.128167729	0.132326189	0.968574175	0.332757693	0.505081442
ENSG00000101843	86.48399765	0.081763978	0.193407899	0.422754076	0.672474697	0.791275931
ENSG00000101844	23.83301539	-0.516422723	0.364070706	-1.41846821	0.156054113	0.293649314
ENSG00000101846	72.92392096	0.980345144	0.209825344	4.672196062	2.98E-06	2.39E-05
ENSG00000101849	587.6365915	0.11664028	0.087902644	1.326925725	0.184533254	0.33202749
ENSG00000101856	1302.324937	-2.119978822	0.074678837	-28.38794647	2.85E-177	2.11E-174
ENSG00000101868	409.8730341	-0.11873011	0.097709315	-1.215136035	0.224314212	0.382628019
ENSG00000101871	139.7396145	-1.974661144	0.190890279	-10.34448246	4.43E-25	1.99E-23
ENSG00000101882	157.5430231	0.171691715	0.151659367	1.132087768	0.257597537	0.423361718
ENSG00000101888	20.77565634	-0.243484012	0.395390008	-0.615807194	0.538021779	0.688744366
ENSG00000101901	246.3856674	-0.076683344	0.125588987	-0.610589717	0.541471232	0.691142874
ENSG00000101911	342.9709793	0.196552277	0.106212563	1.850555812	0.064233482	0.14874509
ENSG00000101928	48.55322115	-0.533445253	0.256349594	-2.080928799	0.037440424	0.096517826

ENSG00000101935	150.8974326	0.288015012	0.15529202	1.854667171	0.06364377	0.147574122
ENSG00000101938	13.44489444	-1.507303418	0.503719089	-2.992349211	0.002768394	0.010780579
ENSG00000101940	367.0803919	-0.265931389	0.106043119	-2.507766571	0.01214969	0.038052775
ENSG00000101945	145.6051107	-0.163119016	0.15549264	-1.049046537	0.2941567	0.464428366
ENSG00000101955	43.83734744	0.493577632	0.271630865	1.817089645	0.069203388	0.157706034
ENSG00000101966	372.3892931	0.004518083	0.106162065	0.042558358	0.966053591	0.982419083
ENSG00000101972	1386.751524	0.382037182	0.066466793	5.74779018	9.04E-09	1.11E-07
ENSG00000101974	402.3065368	0.137144327	0.098365908	1.394226204	0.163249307	0.30356937
ENSG00000101986	324.6192835	1.975367281	0.119386692	16.54595881	1.71E-61	2.31E-59
ENSG00000101997	57.96961184	0.767534251	0.239079837	3.210367978	0.001325651	0.00573673
ENSG00000102003	63.83604683	0.288915805	0.217307024	1.32952815	0.183673785	0.330989821
ENSG00000102007	870.7115775	0.204636669	0.085319551	2.398473337	0.016463575	0.049257474
ENSG00000102024	337.9588604	0.118999292	0.112824669	1.054727599	0.291549925	0.462078506
ENSG00000102030	242.0893515	0.340300008	0.125998684	2.700821926	0.006916836	0.023542718
ENSG00000102032	20.46337354	1.49649529	0.409289464	3.656324977	0.000255857	0.001333094
ENSG00000102034	96.0374641	-0.802319964	0.185182011	-4.332602076	1.47E-05	0.000101867
ENSG00000102038	1156.083016	-0.063585574	0.073504181	-0.865060635	0.387005509	0.555798629
ENSG00000102053	104.9958477	0.196683642	0.173155007	1.135881919	0.256005987	0.421535118
ENSG00000102054	899.7426582	0.460853725	0.079502614	5.79671163	6.76E-09	8.54E-08
ENSG00000102057	31.72535128	0.084579169	0.311874753	0.271195948	0.786240329	0.872090054
ENSG00000102078	21.08984104	-0.338175199	0.383677176	-0.881405565	0.378098346	0.547434005
ENSG00000102081	362.5970583	0.235030395	0.107321417	2.189967303	0.028526608	0.077298084
ENSG00000102096	164.4525233	0.373895936	0.141252692	2.647000437	0.008120925	0.02710255
ENSG00000102098	271.79768	0.132658999	0.122560206	1.082398631	0.279075436	0.447809167
ENSG00000102100	69.36716477	0.379795733	0.211052771	1.799529718	0.071934927	0.162246295
ENSG00000102103	276.3491664	0.288281885	0.123590882	2.332549785	0.019671787	0.057022202
ENSG00000102109	151.8741145	0.46586582	0.146550589	3.178873752	0.001478485	0.006316385
ENSG00000102119	240.0890255	0.279453272	0.132967663	2.101663405	0.035582771	0.092681474
ENSG00000102125	69.50743948	-0.053512046	0.21330198	-0.250874587	0.801911073	0.88203311
ENSG00000102144	2160.330139	0.39756979	0.070743005	5.619916645	1.91E-08	2.25E-07
ENSG00000102158	226.9044529	0.1123741	0.137649306	0.81637971	0.414282997	0.582467547
ENSG00000102172	276.7602139	-0.225787937	0.113181001	-1.994927904	0.046050744	0.114268644
ENSG00000102178	248.3704666	0.262621906	0.126344452	2.078618419	0.037652437	0.097010964
ENSG00000102181	179.5070034	-0.472596656	0.138034061	-3.423768389	0.000617592	0.002923936
ENSG00000102189	781.9042044	0.041297381	0.080883795	0.510576703	0.609647492	0.745903521
ENSG00000102218	45.01390432	0.139121085	0.26405735	0.526859353	0.598291262	0.737311593
ENSG00000102221	116.484435	0.202766225	0.175929377	1.152543305	0.249097885	0.413244404
ENSG00000102225	1140.909825	-0.012605209	0.06911217	-0.182387688	0.855278487	0.916966639
ENSG00000102226	544.7645459	-0.300031012	0.088779641	-3.379502407	0.000726172	0.00337766
ENSG00000102230	175.2920891	0.223830331	0.145078438	1.54282286	0.122873761	0.246466903
ENSG00000102241	948.8441641	0.106512115	0.07310826	1.456909454	0.145141376	0.27825862
ENSG00000102265	5508.241773	0.263411503	0.080600871	3.268097467	0.001082731	0.00482064
ENSG00000102290	58.23498276	0.317402811	0.238600958	1.330266293	0.183430549	0.330678763
ENSG00000102302	597.6710883	0.233989313	0.085445131	2.738474514	0.006172494	0.021355919
ENSG00000102309	148.5736838	0.034825384	0.151691214	0.22958076	0.818417559	0.892848047
ENSG00000102312	111.8411151	0.266773398	0.166356865	1.603621216	0.108797571	0.223989372
ENSG00000102316	518.7825368	-0.234411192	0.089788502	-2.610703904	0.009035609	0.029624164
ENSG00000102317	692.2174643	0.050067533	0.082151089	0.609456714	0.542221756	0.691581745
ENSG00000102349	127.1462229	-0.168476958	0.162027501	-1.039804705	0.298430643	0.469014907
ENSG00000102362	24.72955569	-0.421772696	0.355691538	-1.185782205	0.235708321	0.396763904

ENSG00000102383	18.65259632	-5.104978766	0.909268478	-5.614380008	1.97E-08	2.32E-07
ENSG00000102384	128.1849304	0.21524142	0.167553608	1.284612266	0.19892781	0.350695149
ENSG00000102385	316.7298459	3.953406694	0.161688619	24.45074185	4.94E-132	1.98E-129
ENSG00000102390	156.2569707	-0.574521304	0.15399113	-3.730872699	0.000190818	0.001025015
ENSG00000102393	204.7125961	0.581240277	0.128506825	4.523030415	6.10E-06	4.59E-05
ENSG00000102401	163.8952965	0.059693101	0.1622383	0.367934707	0.71292192	0.820852564
ENSG00000102409	245.216285	-1.364326035	0.128198008	-10.64233414	1.89E-26	8.98E-25
ENSG00000102471	187.9147094	-0.257400124	0.137503479	-1.871953535	0.061213032	0.143160932
ENSG00000102531	635.785593	0.219733067	0.087969161	2.497842008	0.012495187	0.038987092
ENSG00000102543	28.64565637	0.146713495	0.318841969	0.460144866	0.645412242	0.771714825
ENSG00000102547	47.51467376	0.196645449	0.252407403	0.779079563	0.435932848	0.602000464
ENSG00000102572	1079.729955	-0.075884442	0.072255571	-1.05022271	0.293615731	0.463987641
ENSG00000102580	237.3662557	-0.103852829	0.120775431	-0.859883735	0.389853135	0.558214797
ENSG00000102595	229.8631201	-0.048826047	0.133761282	-0.365023766	0.715093666	0.822477747
ENSG00000102606	418.0892636	-0.042070026	0.0967066	-0.435027456	0.663542499	0.784907914
ENSG00000102678	27.69385247	4.927403811	0.648602104	7.596959337	3.03E-14	6.95E-13
ENSG00000102699	780.8188794	0.072069297	0.079323716	0.908546654	0.363589477	0.534581748
ENSG00000102710	503.0695209	-0.07049252	0.093280715	-0.755703039	0.449827262	0.614901085
ENSG00000102738	126.5485584	0.152781103	0.16810416	0.90884784	0.36343045	0.534510264
ENSG00000102743	142.401569	-0.481145217	0.157844062	-3.048231335	0.002301926	0.009209673
ENSG00000102753	270.3132845	-0.16406283	0.11985104	-1.368889498	0.171033819	0.314052555
ENSG00000102763	693.475181	-0.070279236	0.089224475	-0.787667691	0.430891109	0.597477385
ENSG00000102780	712.4977967	-0.138122265	0.083644806	-1.651295178	0.098678316	0.207534086
ENSG00000102781	84.27649581	-0.616221621	0.199692705	-3.085849439	0.002029716	0.008273771
ENSG00000102786	560.4642561	0.228498984	0.089960239	2.539999751	0.011085255	0.035205646
ENSG00000102796	16.94784907	-0.112212415	0.432029575	-0.259733179	0.795069597	0.87776105
ENSG00000102804	753.7678413	-0.107551428	0.0830734	-1.294655428	0.195439122	0.346545487
ENSG00000102805	51.68739109	0.067164854	0.245653066	0.273413458	0.784535408	0.871011269
ENSG00000102858	715.9215559	-0.074961138	0.080950108	-0.926016534	0.354437388	0.525826821
ENSG00000102870	587.098876	-0.138544238	0.09702566	-1.427913375	0.153316794	0.28985747
ENSG00000102871	33.37352202	0.341016552	0.315286192	1.081609536	0.279426065	0.448121697
ENSG00000102878	14.30893349	0.434225872	0.459759421	0.94446324	0.344932978	0.516663846
ENSG00000102879	26.01081971	-0.046886343	0.372554132	-0.125851088	0.899849801	0.945967201
ENSG00000102882	370.9551384	0.920435803	0.104252287	8.828926692	1.06E-18	3.37E-17
ENSG00000102886	13.89389844	1.311740149	0.50276195	2.609068067	0.009078918	0.029741503
ENSG00000102890	19.51502501	0.216928288	0.394959917	0.549241275	0.582839882	0.724975811
ENSG00000102893	299.6381365	-0.050011964	0.113493094	-0.440660853	0.659458541	0.782184761
ENSG00000102897	124.2541579	-0.148807991	0.171971909	-0.86530406	0.386871923	0.555720411
ENSG00000102898	1104.293353	0.287435191	0.088438986	3.250095953	0.001153661	0.005086463
ENSG00000102900	425.501054	0.069587183	0.096740086	0.719321079	0.471943111	0.633818202
ENSG00000102901	154.3042293	0.022987383	0.149858525	0.153393897	0.878087657	0.932836215
ENSG00000102908	1912.783077	-0.235129669	0.070551023	-3.332760573	0.000859889	0.003921708
ENSG00000102910	799.6281451	0.208175286	0.076665022	2.715388055	0.006619815	0.022687934
ENSG00000102921	368.2896936	-0.021139688	0.10562519	-0.200138698	0.841372109	0.907808128
ENSG00000102931	335.6057467	0.271012019	0.104726791	2.587800277	0.009659097	0.031356906
ENSG00000102934	34.07722246	-0.156061687	0.300522858	-0.519300553	0.603551167	0.741285586
ENSG00000102935	220.2474972	-0.302944057	0.136882007	-2.213176611	0.026885472	0.073732961
ENSG00000102967	142.6616766	0.792661524	0.158260434	5.008589334	5.48E-07	5.15E-06
ENSG00000102974	855.4651849	0.110138913	0.076191122	1.44556099	0.148300375	0.282483689
ENSG00000102977	323.5991043	0.599954702	0.111216037	5.394498126	6.87E-08	7.42E-07

ENSG00000102978	350.3619381	0.433680673	0.107762615	4.024407486	5.71E-05	0.000346288
ENSG00000102981	86.87555726	0.399872095	0.190403256	2.100132653	0.035717174	0.092979825
ENSG00000102984	25.3821319	0.471847845	0.351118797	1.343841028	0.178999799	0.324901173
ENSG00000102996	47.26776378	-1.36391414	0.275128183	-4.957377043	7.15E-07	6.53E-06
ENSG00000103005	170.9101153	-0.215536974	0.143129978	-1.505882815	0.132097259	0.259927795
ENSG00000103018	757.4735875	-0.014964504	0.081734355	-0.183087072	0.8547297	0.916518175
ENSG00000103021	84.63071545	-0.183394732	0.205565274	-0.892148407	0.372313392	0.54287242
ENSG00000103024	166.2561053	0.104679792	0.143354791	0.73021481	0.465258891	0.62786931
ENSG00000103034	62.95534767	0.789117609	0.227901188	3.462542755	0.000535097	0.002573265
ENSG00000103035	743.8961429	-0.262396804	0.101565332	-2.583527253	0.009779574	0.031645629
ENSG00000103037	64.26632438	0.473396812	0.233543266	2.027019744	0.042660394	0.107701599
ENSG00000103042	133.6056943	0.31858814	0.154151844	2.066716367	0.038760888	0.099356605
ENSG00000103043	179.773398	-0.810524246	0.141864581	-5.713365792	1.11E-08	1.35E-07
ENSG00000103044	9.402133589	-0.280842706	0.584718149	-0.480304411	0.631010953	0.761038109
ENSG00000103047	154.0214775	0.15015229	0.149000801	1.007728072	0.313585025	0.484803967
ENSG00000103051	224.6955861	-0.239192768	0.12449771	-1.9212624	0.054698635	0.130894672
ENSG00000103061	199.2247229	0.109865128	0.131188977	0.837457011	0.402335731	0.570658715
ENSG00000103064	464.8160888	0.104943606	0.096882275	1.08320749	0.278716336	0.447443242
ENSG00000103066	96.43093759	0.779208963	0.184521444	4.222863999	2.41E-05	0.000159154
ENSG00000103091	468.5430717	0.416957299	0.094861675	4.395424152	1.11E-05	7.86E-05
ENSG00000103111	251.1022061	-0.148943532	0.120288398	-1.238220271	0.215634397	0.371932266
ENSG00000103121	74.25836421	-0.178800421	0.211985199	-0.843457096	0.398972851	0.567071906
ENSG00000103126	452.9676912	0.374670081	0.092394725	4.055102519	5.01E-05	0.00030847
ENSG00000103145	437.7811324	-0.321566279	0.096301646	-3.339156626	0.000840332	0.003846249
ENSG00000103148	562.1939622	0.134224039	0.088278093	1.520468261	0.12839333	0.254091449
ENSG00000103150	43.63083208	-0.110710161	0.274793139	-0.402885463	0.687032486	0.802690288
ENSG00000103152	393.9906309	0.413937802	0.100230333	4.12986557	3.63E-05	0.000230398
ENSG00000103160	142.7888066	-0.012843859	0.159375788	-0.080588522	0.935769195	0.964731953
ENSG00000103168	194.2628438	0.093986444	0.141524697	0.664099242	0.506626796	0.663984568
ENSG00000103174	45.40780409	-0.173879801	0.260812125	-0.666686034	0.504972702	0.662682319
ENSG00000103175	66.57384075	0.673951742	0.223046815	3.021570794	0.002514668	0.0099363
ENSG00000103184	64.60533078	-0.854315439	0.231158903	-3.695792931	0.000219202	0.001161058
ENSG00000103187	769.9719647	0.693524746	0.091502745	7.579278081	3.47E-14	7.95E-13
ENSG00000103194	532.1590604	-0.049503972	0.092290564	-0.536392559	0.591687273	0.732260534
ENSG00000103197	745.2621552	-0.003701217	0.08135677	-0.045493657	0.96371383	0.981000042
ENSG00000103199	86.06679746	0.090279376	0.20913052	0.431689146	0.665967357	0.786253443
ENSG00000103202	3065.237015	-0.136626358	0.062004745	-2.203482318	0.027560772	0.075159418
ENSG00000103222	695.6502633	0.095759769	0.080289494	1.192681179	0.232994259	0.39318481
ENSG00000103227	99.76388204	0.0339529	0.18820691	0.180401985	0.856836996	0.918006963
ENSG00000103245	73.94989993	-0.10035903	0.207847845	-0.482848546	0.629203278	0.759640606
ENSG00000103248	76.05361807	-0.421747167	0.208635431	-2.021455157	0.04323267	0.108969911
ENSG00000103249	496.674037	0.429405503	0.104600108	4.105210908	4.04E-05	0.000253881
ENSG00000103253	106.147244	0.417858343	0.173499658	2.408410184	0.016022168	0.048172816
ENSG00000103254	41.93436796	0.484795136	0.272141072	1.781411139	0.07484531	0.167199756
ENSG00000103257	1049.650094	-1.515171244	0.080521989	-18.81686312	5.49E-79	9.77E-77
ENSG00000103260	334.4694291	0.35480357	0.136942203	2.590900115	0.009572527	0.031111821
ENSG00000103264	202.7347999	-0.894913969	0.135239441	-6.617255739	3.66E-11	6.11E-10
ENSG00000103266	424.6266053	0.663606796	0.096388939	6.884677888	5.79E-12	1.05E-10
ENSG00000103269	29.37332022	-0.073738542	0.315616574	-0.233633303	0.815269671	0.891560379
ENSG00000103274	90.4661196	-0.3521187	0.186171184	-1.891370572	0.058574894	0.137984494

ENSG00000103275	1000.194603	-0.058853123	0.085621359	-0.687364974	0.491852764	0.651295106
ENSG00000103319	547.5472369	-0.031656093	0.087022447	-0.363769278	0.716030306	0.823285398
ENSG00000103326	462.2382257	0.050937643	0.103636133	0.491504666	0.623069553	0.755351515
ENSG00000103335	715.3529369	-0.63858798	0.082967649	-7.696831067	1.39E-14	3.27E-13
ENSG00000103342	1550.395493	-0.024315991	0.064453152	-0.377266128	0.705975841	0.815529823
ENSG00000103343	103.2875441	0.116048229	0.176638999	0.656979659	0.511193996	0.667447979
ENSG00000103351	181.345661	0.123690097	0.138096017	0.895681858	0.370422702	0.541536387
ENSG00000103353	466.8306462	0.126037123	0.095394968	1.321213533	0.186430175	0.334626204
ENSG00000103356	398.9900681	-0.167420481	0.097916799	-1.709823872	0.087298447	0.188774829
ENSG00000103363	927.8293365	0.326153173	0.086883808	3.753900519	0.000174104	0.000944627
ENSG00000103365	715.0541996	0.116647189	0.079441822	1.468334763	0.142013313	0.273777723
ENSG00000103404	247.850698	-0.161119551	0.122447074	-1.315830146	0.188231059	0.336954464
ENSG00000103415	480.5940919	0.183579985	0.094629369	1.939989536	0.052380962	0.126512271
ENSG00000103423	862.3784568	0.313887518	0.074678287	4.203196529	2.63E-05	0.000172424
ENSG00000103429	292.6713765	-0.191391671	0.118652551	-1.613043034	0.106735159	0.220746286
ENSG00000103479	267.092125	-0.691569016	0.120238222	-5.751657041	8.84E-09	1.09E-07
ENSG00000103485	2392.669451	0.047352619	0.065104526	0.72733221	0.467022471	0.629196354
ENSG00000103494	242.6002626	-0.038059226	0.122314318	-0.311159206	0.755679593	0.852102835
ENSG00000103495	1483.109728	0.017080734	0.069672524	0.245157387	0.80633457	0.885511821
ENSG00000103496	298.9416707	0.533023861	0.123233264	4.325324543	1.52E-05	0.000104927
ENSG00000103502	368.2311072	0.140897827	0.106667318	1.32090907	0.186531685	0.334722864
ENSG00000103507	777.917067	0.806329459	0.080949281	9.960921853	2.26E-23	9.09E-22
ENSG00000103510	193.0309768	0.087673992	0.131904502	0.664677785	0.506256606	0.663809068
ENSG00000103512	169.568038	0.462487672	0.144473459	3.201194697	0.00136859	0.005906171
ENSG00000103522	165.1718435	-0.800051677	0.160198822	-4.994117112	5.91E-07	5.50E-06
ENSG00000103540	348.3937739	0.00712185	0.104060933	0.068439225	0.945435998	0.969665557
ENSG00000103544	371.0010333	-0.161896299	0.100672078	-1.608154932	0.107801251	0.222492532
ENSG00000103549	634.5245118	-0.086069875	0.085107316	-1.011309953	0.311868104	0.483319865
ENSG00000103550	703.9805378	-0.142361841	0.083982487	-1.695137242	0.090049389	0.19341342
ENSG00000103591	231.231836	-0.548784284	0.127957611	-4.288797534	1.80E-05	0.00012167
ENSG00000103599	24.6468073	-1.19222593	0.372849225	-3.197608713	0.001385722	0.005972762
ENSG00000103642	61.84084112	0.35223729	0.229208877	1.536752396	0.124353955	0.248513303
ENSG00000103653	460.3217877	0.40705454	0.096477697	4.219156901	2.45E-05	0.000161641
ENSG00000103657	582.5576482	-0.121306172	0.086876285	-1.396309385	0.162621349	0.302762371
ENSG00000103671	84.68418936	-0.412874364	0.195184711	-2.115300744	0.034404323	0.090163718
ENSG00000103707	97.91944435	0.120085728	0.190396995	0.630712307	0.528228651	0.680934946
ENSG00000103723	39.37886197	-1.050616169	0.2899937	-3.622893082	0.000291326	0.001499989
ENSG00000103740	73.58210912	0.817357353	0.222784879	3.6688188	0.000243674	0.001275292
ENSG00000103742	121.0024555	-0.174396944	0.162861904	-1.070827122	0.284247169	0.453798699
ENSG00000103769	585.4085504	-0.556823607	0.088189374	-6.313953504	2.72E-10	4.06E-09
ENSG00000103811	59.3474016	-0.184980895	0.238876072	-0.774380179	0.438706001	0.604166981
ENSG00000103832	9.724385765	-0.543467654	0.556221874	-0.977069905	0.328534546	0.500914443
ENSG00000103852	213.5541493	-0.420023258	0.132837149	-3.16194123	0.001567212	0.006626919
ENSG00000103855	934.2294132	-0.342670451	0.073940497	-4.634408268	3.58E-06	2.83E-05
ENSG00000103876	146.8981839	-0.027397915	0.151602682	-0.180721834	0.85658592	0.917807964
ENSG00000103932	300.4094782	0.255812704	0.111457872	2.295151513	0.021724443	0.061761435
ENSG00000103942	447.2848749	0.054185291	0.097812672	0.553970046	0.579599345	0.722800027
ENSG00000103966	144.3148049	-0.080936312	0.150924582	-0.536269913	0.591772021	0.732260534
ENSG00000103978	133.0195606	0.012728906	0.158973161	0.080069531	0.936181956	0.964894558
ENSG00000103994	951.4898104	-0.213792763	0.072796772	-2.936844011	0.003315708	0.012584641

ENSG00000103995	278.3819303	-0.412998927	0.11442143	-3.609454325	0.000306842	0.001570775
ENSG00000104044	45.77924584	0.41966112	0.260721483	1.609614654	0.107482007	0.221998702
ENSG00000104047	122.9189094	-0.132228977	0.167331637	-0.790221024	0.4293987	0.596200805
ENSG00000104064	108.1047805	0.198263664	0.172673289	1.148201116	0.250885549	0.415196666
ENSG00000104067	1635.296014	0.017140765	0.070497749	0.24313918	0.80789758	0.886465967
ENSG00000104081	13.73145308	-0.907425714	0.475078182	-1.910055542	0.056126062	0.133537059
ENSG00000104093	854.7859638	0.053594526	0.078211006	0.685255548	0.493182677	0.652452163
ENSG00000104129	139.6373991	0.501334893	0.154226145	3.250647903	0.001151424	0.005082982
ENSG00000104131	775.9876506	0.058018235	0.082092763	0.706739942	0.479728103	0.640321234
ENSG00000104133	393.7488986	-0.130374837	0.101449225	-1.285124035	0.198748944	0.350564914
ENSG00000104140	48.96904543	0.286168938	0.248365651	1.152208194	0.249235531	0.41339137
ENSG00000104142	230.2542231	0.410330464	0.125479417	3.270101766	0.001075088	0.004792688
ENSG00000104147	9.321641574	-0.06048467	0.565263078	-0.10700269	0.914786845	0.953830969
ENSG00000104154	56.71997182	-0.299025891	0.232712271	-1.284959701	0.198806367	0.350564914
ENSG00000104164	172.306256	0.073338704	0.145197932	0.50509469	0.613492331	0.748612122
ENSG00000104177	707.444263	-0.661675417	0.08195641	-8.073504132	6.83E-16	1.77E-14
ENSG00000104205	14.33160084	0.598751759	0.469818368	1.274432417	0.20251021	0.355138691
ENSG00000104213	37.09676917	-0.789164971	0.290529086	-2.716302805	0.006601551	0.022634119
ENSG00000104218	364.8557573	-0.15665768	0.102315907	-1.531117533	0.125740347	0.250570954
ENSG00000104219	50.00045796	-0.15260263	0.24913469	-0.612530633	0.540186737	0.690194933
ENSG00000104221	59.77737085	0.419636865	0.228618772	1.835531095	0.066427036	0.152631687
ENSG00000104228	120.8163534	-0.059337799	0.169096121	-0.350911652	0.72565463	0.83027373
ENSG00000104231	265.8874352	-0.000543841	0.119218242	-0.004561723	0.996360284	0.998279095
ENSG00000104290	288.3952684	-0.079086141	0.110430472	-0.7161623	0.473891139	0.634943611
ENSG00000104299	92.48590275	-0.275009156	0.18757688	-1.466114354	0.142617136	0.27469394
ENSG00000104312	93.1531736	0.448282535	0.184727824	2.426719082	0.015236048	0.04612484
ENSG00000104313	17.98323518	-5.017689642	0.894922203	-5.60684451	2.06E-08	2.42E-07
ENSG00000104320	569.6426951	0.299203672	0.093584214	3.197159629	0.001387881	0.005978401
ENSG00000104324	39.63530007	0.405720617	0.29022887	1.397933352	0.162133082	0.301973403
ENSG00000104325	361.8465146	0.134788943	0.1043833	1.291288382	0.196603698	0.34786398
ENSG00000104331	1054.383557	-0.197071901	0.078152154	-2.521643882	0.011680789	0.036814012
ENSG00000104332	45.00279403	-8.771805494	1.19714712	-7.327257735	2.35E-13	4.96E-12
ENSG00000104341	881.8546278	0.130716718	0.096718502	1.35151719	0.176529817	0.321581422
ENSG00000104343	97.12095069	-0.357282403	0.181105683	-1.972784055	0.048520171	0.119154342
ENSG00000104356	314.6588302	0.948693894	0.116906928	8.114950183	4.86E-16	1.27E-14
ENSG00000104361	82.55250998	-0.258353996	0.218189334	-1.184081692	0.236380727	0.397609875
ENSG00000104365	187.8018526	0.202467614	0.148582462	1.362661584	0.17298919	0.316650919
ENSG00000104368	206.4100456	0.78931718	0.132254427	5.96817208	2.40E-09	3.19E-08
ENSG00000104369	15.60534656	-3.774731599	0.689191872	-5.477040212	4.32E-08	4.84E-07
ENSG00000104375	238.6020683	-0.081851689	0.119975392	-0.682237308	0.495088908	0.654123943
ENSG00000104381	43.19332569	-1.073105862	0.290164677	-3.698264969	0.000217078	0.001152418
ENSG00000104388	483.4705076	0.136590123	0.094353267	1.447645933	0.147716098	0.281855101
ENSG00000104408	3692.067185	0.026141381	0.054296531	0.481455821	0.630192572	0.760442836
ENSG00000104412	268.3712841	-0.216083107	0.117706208	-1.835783439	0.066389692	0.15260612
ENSG00000104419	43.77809211	4.150646686	0.592885662	7.000754033	2.55E-12	4.86E-11
ENSG00000104427	53.81126715	-0.272390291	0.24507793	-1.111443576	0.266377464	0.433229621
ENSG00000104442	122.9078118	-0.014339591	0.161141325	-0.088987667	0.929091711	0.961351227
ENSG00000104447	6740.423195	0.781063946	0.052339533	14.92302102	2.33E-50	2.47E-48
ENSG00000104450	61.6156313	-0.106663661	0.230856786	-0.4620339	0.64405701	0.770422279
ENSG00000104472	499.0993832	0.093740954	0.095290947	0.983734092	0.325246273	0.497304277

ENSG00000104490	11.12431207	-1.705864474	0.582448763	-2.928780317	0.003402948	0.012880953
ENSG00000104497	181.4763378	0.416464299	0.142823093	2.915945095	0.003546129	0.013358132
ENSG00000104517	2853.069268	0.352825966	0.062149653	5.677038351	1.37E-08	1.65E-07
ENSG00000104522	374.3570147	0.80848215	0.107561783	7.516444268	5.63E-14	1.26E-12
ENSG00000104524	128.5116039	0.190650568	0.165599602	1.151274314	0.249619401	0.413789489
ENSG00000104529	1548.865471	0.196773781	0.064164674	3.066699629	0.002164362	0.008726406
ENSG00000104549	1082.860953	0.178213918	0.082252827	2.16666008	0.030260783	0.081074426
ENSG00000104611	178.2487372	-0.355092746	0.137443412	-2.583555955	0.00977876	0.031645629
ENSG00000104613	479.8887698	0.313843666	0.092227038	3.40294638	0.000666634	0.003128701
ENSG00000104626	151.0854393	-0.01765892	0.171197578	-0.103149354	0.917844435	0.955355281
ENSG00000104635	552.1990914	-0.147648154	0.090225478	-1.636435272	0.101748522	0.212572927
ENSG00000104643	201.7639292	-0.317790529	0.137453835	-2.311980089	0.020778781	0.059603743
ENSG00000104660	164.849017	0.016112561	0.141950483	0.11350832	0.909627567	0.951381168
ENSG00000104671	97.99549234	-0.121858196	0.178985063	-0.680828859	0.495979787	0.654672813
ENSG00000104679	77.09489047	0.293799141	0.211245484	1.390794897	0.164287631	0.305257719
ENSG00000104687	872.6296348	0.220172762	0.078453297	2.80641822	0.005009563	0.017937846
ENSG00000104691	44.22126965	0.28805067	0.263750297	1.092134015	0.2747742	0.443140435
ENSG00000104695	151.5111819	0.175714489	0.153884709	1.141858017	0.253513042	0.418411197
ENSG00000104714	320.7217983	-0.212294789	0.116106294	-1.828452024	0.06748174	0.154686031
ENSG00000104722	97.15576228	-1.372738431	0.193781946	-7.083933571	1.40E-12	2.76E-11
ENSG00000104723	361.2311731	0.261070132	0.101189625	2.580008877	0.009879778	0.031940445
ENSG00000104728	25.7127954	-0.188394668	0.341158746	-0.552219955	0.580797664	0.723524724
ENSG00000104731	192.4196348	-0.895517547	0.137846269	-6.496494633	8.22E-11	1.32E-09
ENSG00000104738	1738.285167	0.008049415	0.062413318	0.128969504	0.897381779	0.944375326
ENSG00000104756	214.2991606	0.062405173	0.131494675	0.474583273	0.635084031	0.764244443
ENSG00000104760	6.365924096	-3.48701152	1.015854768	-3.432588623	0.000597848	0.002842917
ENSG00000104763	337.9410169	0.244196665	0.109930013	2.221383038	0.026325029	0.072408005
ENSG00000104765	448.3452404	0.809567103	0.101762848	7.955428926	1.79E-15	4.45E-14
ENSG00000104774	149.083839	-0.120511811	0.149396744	-0.806656208	0.419864581	0.587668171
ENSG00000104804	16.73194926	3.502444285	0.604348424	5.795405675	6.82E-09	8.59E-08
ENSG00000104805	870.9785633	0.348505292	0.087617399	3.977580911	6.96E-05	0.000415796
ENSG00000104812	732.8282676	0.378285282	0.08200446	4.612984244	3.97E-06	3.10E-05
ENSG00000104823	1289.607481	0.40023951	0.082614003	4.844693308	1.27E-06	1.10E-05
ENSG00000104824	1509.691207	0.310720237	0.063401627	4.900824338	9.54E-07	8.49E-06
ENSG00000104825	134.4290993	0.238851748	0.16199147	1.474471146	0.140354781	0.271453065
ENSG00000104833	158.3049477	-0.364903091	0.158631944	-2.300312802	0.021430505	0.061123716
ENSG00000104835	68.27110392	0.182383924	0.244905072	0.744712728	0.456445403	0.620145911
ENSG00000104852	1799.883622	0.268925292	0.081909801	3.28318821	0.001026401	0.004601933
ENSG00000104853	838.375381	0.202074934	0.079666735	2.536503267	0.01119657	0.035486962
ENSG00000104856	105.6624173	0.428136188	0.184041067	2.326307902	0.020002131	0.057753326
ENSG00000104859	263.1430283	0.362222505	0.117568912	3.0809378	0.002063497	0.008379864
ENSG00000104863	54.343386	0.176556436	0.252486875	0.699269757	0.484383463	0.644880533
ENSG00000104866	130.6166695	-0.083008218	0.164764339	-0.503799661	0.614402166	0.749042347
ENSG00000104870	163.8126968	-0.831866216	0.146012948	-5.697208544	1.22E-08	1.48E-07
ENSG00000104872	183.7765658	-0.733831846	0.141318942	-5.192735222	2.07E-07	2.08E-06
ENSG00000104880	150.4645806	-0.272167047	0.153286573	-1.775543952	0.075808115	0.168893988
ENSG00000104881	199.8409365	-0.100380316	0.138849786	-0.722941811	0.469715634	0.631654585
ENSG00000104884	408.1423833	-0.08829845	0.097242107	-0.908026912	0.363864003	0.534850783
ENSG00000104885	1164.690975	-0.288966966	0.069956485	-4.130667299	3.62E-05	0.000229804
ENSG00000104886	356.2113293	0.370657788	0.101998954	3.633937153	0.000279129	0.001444171

ENSG00000104888	223.3767071	-0.281917107	0.129192295	-2.18215108	0.029098384	0.078574588
ENSG00000104897	607.9590927	0.369989526	0.092457957	4.001705606	6.29E-05	0.000378808
ENSG00000104899	19.14608431	-0.983461505	0.440036793	-2.234952897	0.025420441	0.070305363
ENSG00000104901	17.18985355	0.047318837	0.447071533	0.105841757	0.915707905	0.954435272
ENSG00000104904	2371.68875	0.28434712	0.068147127	4.172547459	3.01E-05	0.000194535
ENSG00000104907	311.1944227	0.117056709	0.106916295	1.094844414	0.273584805	0.441780176
ENSG00000104915	239.9420951	0.29065539	0.123578683	2.35198647	0.018673454	0.054578862
ENSG00000104936	258.5574404	-0.216625003	0.123693444	-1.751305455	0.079893309	0.176331292
ENSG00000104946	348.6943628	0.543500458	0.105326296	5.160159236	2.47E-07	2.45E-06
ENSG00000104953	42.39833049	-2.325406283	0.323304466	-7.192620362	6.36E-13	1.29E-11
ENSG00000104957	86.96145785	0.044583351	0.209376365	0.212934017	0.831378429	0.901477895
ENSG00000104960	620.8568902	0.250007888	0.082952505	3.013867846	0.002579401	0.010160642
ENSG00000104964	838.3518925	-0.286768342	0.078513411	-3.652475894	0.000259724	0.001351488
ENSG00000104967	11.78129869	-2.111700987	0.588983803	-3.585329474	0.000336653	0.001701678
ENSG00000104969	727.1764407	0.124306467	0.086923015	1.430075419	0.152695374	0.289017785
ENSG00000104973	573.420799	0.259962663	0.085488163	3.04091997	0.002358565	0.009393469
ENSG00000104976	169.298805	0.186493995	0.149066208	1.251081633	0.210904696	0.366157244
ENSG00000104979	652.6286194	0.458932503	0.085118224	5.391706776	6.98E-08	7.51E-07
ENSG00000104980	409.6099425	0.008340814	0.107728658	0.077424281	0.938286025	0.966102042
ENSG00000104983	18.53095104	-0.201374098	0.442612811	-0.454966719	0.649133183	0.774121217
ENSG00000104998	356.6986913	0.525096024	0.105939909	4.956545924	7.18E-07	6.55E-06
ENSG00000105011	142.1335086	-0.919682143	0.154756102	-5.942784356	2.80E-09	3.68E-08
ENSG00000105048	803.6321027	-0.758783498	0.080059453	-9.477750221	2.60E-21	9.48E-20
ENSG00000105053	92.28035732	0.202689133	0.19232999	1.053861297	0.291946425	0.462535239
ENSG00000105058	409.3856078	-0.019855837	0.107134088	-0.185336314	0.852965257	0.915744647
ENSG00000105063	1055.423619	0.474798398	0.079220445	5.993382153	2.06E-09	2.76E-08
ENSG00000105072	73.02675885	0.455574867	0.20787287	2.191603298	0.028408164	0.077051453
ENSG00000105085	160.2814035	0.148186898	0.151747738	0.976534481	0.328799669	0.501209871
ENSG00000105088	195.7734614	0.427500657	0.134768568	3.172109504	0.001513359	0.006436015
ENSG00000105127	523.7606464	0.137613493	0.093655665	1.469355789	0.141736312	0.273334703
ENSG00000105131	7.524775332	-0.044061246	0.622213476	-0.070813713	0.943546018	0.968637121
ENSG00000105135	231.3669397	0.051714788	0.13374853	0.386656868	0.699010252	0.8110439
ENSG00000105136	38.88624508	0.030466838	0.282048322	0.108019922	0.913979889	0.953626644
ENSG00000105137	335.8698667	0.199864966	0.104954189	1.9043067	0.056870252	0.134850849
ENSG00000105171	117.6099928	-0.073855732	0.173528071	-0.425612593	0.670390163	0.789616855
ENSG00000105173	162.3937363	-0.160524839	0.147765138	-1.086351225	0.277323632	0.445920908
ENSG00000105176	469.7235277	-5.05E-05	0.095117125	-0.000530834	0.999576456	0.999854605
ENSG00000105185	476.2322759	0.410891512	0.094889256	4.330221687	1.49E-05	0.000102772
ENSG00000105186	327.8918848	0.093525503	0.108975565	0.858224524	0.390768497	0.559133568
ENSG00000105193	3529.945599	0.155925819	0.065702135	2.373223017	0.017633613	0.052136259
ENSG00000105197	530.2529301	0.213376053	0.090682542	2.35300034	0.018622617	0.054452935
ENSG00000105202	1304.451833	0.492511378	0.072073853	6.833426562	8.29E-12	1.47E-10
ENSG00000105204	102.5380878	-0.005071432	0.187537333	-0.027042252	0.978426034	0.988843871
ENSG00000105220	2369.774084	0.217266052	0.062682035	3.466161458	0.000527946	0.00254323
ENSG00000105221	749.4127867	0.023077464	0.093870148	0.245844547	0.805802571	0.885189466
ENSG00000105223	1758.437765	0.340044021	0.077537497	4.385542935	1.16E-05	8.18E-05
ENSG00000105227	46.03835844	0.205139669	0.273312205	0.750569005	0.452912081	0.617396281
ENSG00000105229	268.2437894	-0.112327343	0.124825008	-0.899878517	0.368184904	0.539469422
ENSG00000105245	348.6190604	0.267356536	0.106993304	2.498815578	0.012460914	0.038897435
ENSG00000105246	9.135116325	-2.161965616	0.665998072	-3.246204015	0.00116955	0.005143603

ENSG00000105248	224.9771373	0.142964093	0.123388835	1.158646918	0.246600136	0.410522886
ENSG00000105254	754.5982812	0.252873635	0.092035024	2.747580467	0.006003678	0.020848865
ENSG00000105255	63.83583322	0.150019368	0.222987958	0.672768923	0.501094311	0.659379091
ENSG00000105258	337.5192116	0.523955262	0.106534504	4.918174314	8.74E-07	7.83E-06
ENSG00000105270	82.61935381	0.141306554	0.196774023	0.718115896	0.47268583	0.633981332
ENSG00000105281	586.6064457	-0.1465447	0.099842958	-1.467751995	0.142171602	0.274023667
ENSG00000105287	247.1524092	-0.07414897	0.126864981	-0.58447153	0.558903115	0.705699959
ENSG00000105289	20.16227563	-1.638977095	0.423432615	-3.870691665	0.000108527	0.000620211
ENSG00000105290	1396.804888	0.273214601	0.06731631	4.058668715	4.94E-05	0.000304331
ENSG00000105298	244.9539136	0.000690094	0.124542198	0.005541047	0.995578907	0.997940364
ENSG00000105321	217.1119551	0.079288472	0.126163136	0.628459904	0.529702705	0.682186526
ENSG00000105323	3739.682811	0.124671736	0.063725898	1.956374721	0.050421028	0.122682436
ENSG00000105325	212.0760379	0.18762681	0.139060655	1.349244401	0.177258478	0.322541668
ENSG00000105327	56.09915435	-0.100328189	0.241071311	-0.416176394	0.677280932	0.795199369
ENSG00000105329	1474.27798	0.61810274	0.08553007	7.226730165	4.95E-13	1.01E-11
ENSG00000105339	47.05159539	-0.356056866	0.284692117	-1.250673428	0.211053649	0.366274791
ENSG00000105341	374.6322502	-0.066529212	0.100994225	-0.658742741	0.510060982	0.666495499
ENSG00000105355	410.2087918	0.177575378	0.098847484	1.796458247	0.072421667	0.163146311
ENSG00000105357	457.5518513	0.124046957	0.095224759	1.302675473	0.192685579	0.343266243
ENSG00000105364	622.0364948	0.37400889	0.093155881	4.014871466	5.95E-05	0.000359042
ENSG00000105372	5386.553815	0.121314858	0.057879946	2.095973916	0.036084505	0.093762309
ENSG00000105373	1343.92752	0.046753476	0.075371912	0.620303705	0.535057856	0.685971818
ENSG00000105376	163.9695322	0.728626207	0.150630035	4.83719073	1.32E-06	1.14E-05
ENSG00000105379	130.9954036	0.168138508	0.161608452	1.040406647	0.298151015	0.468750272
ENSG00000105393	109.8190819	0.288053475	0.17351901	1.660068685	0.096900636	0.204501687
ENSG00000105397	569.0846576	0.019054216	0.098034005	0.194363334	0.845891398	0.910865194
ENSG00000105401	731.8318728	0.161958954	0.080650414	2.008160229	0.044626268	0.111522004
ENSG00000105402	195.9803363	0.518620011	0.137628187	3.768268875	0.000164384	0.000897782
ENSG00000105404	75.55744382	-0.032766231	0.216071804	-0.151645102	0.879466858	0.933918276
ENSG00000105409	7.655561235	-1.775200699	0.707063496	-2.510666594	0.012050345	0.037809067
ENSG00000105419	111.9047023	-0.968980415	0.181114404	-5.350101323	8.79E-08	9.33E-07
ENSG00000105426	2112.580437	-0.913316127	0.07012495	-13.02412515	8.92E-39	6.67E-37
ENSG00000105427	72.54249969	-2.860221684	0.278482199	-10.27075228	9.55E-25	4.19E-23
ENSG00000105429	382.2227011	-1.122105617	0.114144401	-9.830579547	8.31E-23	3.24E-21
ENSG00000105438	1891.213083	0.19772105	0.0676401	2.923133617	0.003465278	0.01308515
ENSG00000105443	313.7693586	-0.179920043	0.113561529	-1.584339743	0.113116426	0.230985091
ENSG00000105447	438.4423361	0.091903045	0.099566924	0.923027869	0.355992682	0.527308874
ENSG00000105464	447.4461437	0.706109038	0.101017108	6.989994602	2.75E-12	5.20E-11
ENSG00000105472	1209.014151	0.343996299	0.097224165	3.538176959	0.0004029	0.001996309
ENSG00000105479	26.13753811	-0.042601508	0.339693773	-0.125411506	0.90019778	0.946063011
ENSG00000105483	182.8212185	0.022398468	0.137432743	0.162977666	0.87053602	0.927945136
ENSG00000105486	764.4263042	-0.550502661	0.081114109	-6.786768281	1.15E-11	2.01E-10
ENSG00000105497	53.9575994	-0.174452718	0.242693664	-0.718818595	0.472252699	0.633897148
ENSG00000105499	124.4929834	0.350998841	0.172232692	2.037933891	0.041556545	0.105369095
ENSG00000105514	172.0136009	-0.105473339	0.138239144	-0.762977374	0.44547689	0.610498914
ENSG00000105516	245.9672277	0.218799373	0.132126889	1.655979142	0.097726043	0.20596515
ENSG00000105518	315.4632809	-0.218726236	0.107647519	-2.031874389	0.042166374	0.106799685
ENSG00000105520	175.1711375	0.373215146	0.143593257	2.599113311	0.009346492	0.030513582
ENSG00000105538	31.54697741	-1.866987353	0.350146069	-5.332024312	9.71E-08	1.02E-06
ENSG00000105552	232.2688432	0.247524475	0.129898819	1.905517508	0.056712834	0.13463667

ENSG00000105556	347.6281493	0.462323348	0.105310305	4.390105495	1.13E-05	8.04E-05
ENSG00000105559	634.0838926	0.397040881	0.086238315	4.603996258	4.14E-06	3.22E-05
ENSG00000105568	872.1054756	-0.077214291	0.081383396	-0.948772047	0.342736562	0.514590639
ENSG00000105576	920.9774907	0.01651285	0.073063612	0.226006476	0.821196383	0.895180952
ENSG00000105605	409.4759094	-0.341078107	0.105232753	-3.241178231	0.001190367	0.005220446
ENSG00000105607	111.0175748	0.012239501	0.172161764	0.071093026	0.943323719	0.968637121
ENSG00000105612	406.8494329	0.208087627	0.103981581	2.001196987	0.045371166	0.112981521
ENSG00000105613	187.4929934	0.168249453	0.14167919	1.18753822	0.23501539	0.396024614
ENSG00000105617	121.1980415	0.382354745	0.162399317	2.354411051	0.018552083	0.05430734
ENSG00000105618	435.3911949	0.285787872	0.0956992	2.986314121	0.002823624	0.010962811
ENSG00000105619	135.6173957	0.051240469	0.154911401	0.330772743	0.740816151	0.841588716
ENSG00000105639	124.5787054	-0.498065623	0.169509839	-2.938269685	0.003300498	0.012537072
ENSG00000105640	815.2225607	0.224303731	0.083539203	2.685011627	0.007252728	0.02459659
ENSG00000105641	29.76054243	-4.116412895	0.547699702	-7.515820945	5.66E-14	1.26E-12
ENSG00000105642	29.50449356	-1.05135242	0.339027577	-3.101082303	0.001928147	0.007931092
ENSG00000105643	115.3650001	0.149701619	0.172005025	0.870332827	0.384118544	0.553241226
ENSG00000105649	248.6993574	0.173523209	0.141002026	1.230643373	0.218456276	0.375499801
ENSG00000105650	11.33206517	-2.274169968	0.609063214	-3.733881665	0.000188551	0.001016338
ENSG00000105655	1455.653218	-0.35272453	0.083144232	-4.242321094	2.21E-05	0.000147344
ENSG00000105656	108.4500864	-0.011765801	0.175974316	-0.066860899	0.946692441	0.970529027
ENSG00000105662	333.295081	-0.011708311	0.106830556	-0.109597023	0.912728972	0.953043412
ENSG00000105668	9.446225045	0.56740809	0.558172113	1.016546827	0.30936906	0.480613492
ENSG00000105669	798.8735725	0.231256645	0.101953186	2.268263056	0.023313178	0.065404477
ENSG00000105671	263.49712	0.347865977	0.117008429	2.972999286	0.002949051	0.011371212
ENSG00000105676	184.2010987	0.383503954	0.137545229	2.788202531	0.00530014	0.018791283
ENSG00000105677	254.9549664	0.958174159	0.127019287	7.543532826	4.57E-14	1.04E-12
ENSG00000105698	557.5019279	0.466755229	0.099938707	4.670414948	3.01E-06	2.41E-05
ENSG00000105699	296.1101263	-1.518875197	0.124215155	-12.22777685	2.21E-34	1.40E-32
ENSG00000105700	715.6674116	0.11054086	0.081514413	1.356089758	0.175070611	0.319564395
ENSG00000105701	1376.074605	0.24824659	0.091696758	2.707255922	0.006784194	0.023164212
ENSG00000105705	226.6329329	-0.075964771	0.125875912	-0.603489338	0.546183206	0.694729455
ENSG00000105708	42.85732558	0.314060344	0.271211427	1.157990825	0.246867781	0.410919743
ENSG00000105711	28.35091995	-0.555426874	0.336287839	-1.651641268	0.098607702	0.207449811
ENSG00000105722	458.9876906	0.109537156	0.112272946	0.97563269	0.329246519	0.501782125
ENSG00000105723	453.4351702	0.250424773	0.102070012	2.453460794	0.014148896	0.043272272
ENSG00000105726	830.6801517	0.461904173	0.076318482	6.05232389	1.43E-09	1.95E-08
ENSG00000105732	265.127139	0.098350226	0.123140223	0.798684815	0.424473194	0.591642683
ENSG00000105737	10.17681812	0.760469795	0.543412085	1.399434822	0.161682631	0.301334207
ENSG00000105738	487.6439973	-0.196411084	0.090491177	-2.170499817	0.029969002	0.08040131
ENSG00000105755	90.67689827	-0.173927461	0.190915731	-0.911016917	0.36228646	0.533106842
ENSG00000105767	41.33699435	0.293047933	0.290566805	1.008538923	0.313195812	0.484308847
ENSG00000105771	249.4221385	0.060004523	0.121671243	0.49316931	0.621892961	0.754771939
ENSG00000105778	442.8895344	0.002454507	0.100825686	0.024344064	0.980578166	0.990159622
ENSG00000105793	107.6051067	0.094669105	0.173647536	0.545179663	0.585629958	0.727480015
ENSG00000105819	622.5197844	0.364831019	0.092413652	3.947804363	7.89E-05	0.000464725
ENSG00000105821	822.7833576	1.0276988	0.078164218	13.14794456	1.75E-39	1.33E-37
ENSG00000105829	31.55940993	0.427441835	0.312536157	1.367655632	0.171419894	0.314504514
ENSG00000105835	556.0822398	0.732848367	0.09209594	7.957444912	1.76E-15	4.41E-14
ENSG00000105849	99.25122682	0.404385599	0.182884422	2.211153882	0.027025183	0.074029396
ENSG00000105854	417.4699495	0.535963456	0.098095333	5.46369983	4.66E-08	5.19E-07

ENSG00000105856	188.1179459	0.1428923	0.137479742	1.039369862	0.298632755	0.469182736
ENSG00000105865	15.49605228	0.301502407	0.435917274	0.691650514	0.489156831	0.648959766
ENSG00000105866	119.6567858	-0.132648045	0.168047583	-0.789348127	0.429908565	0.59673178
ENSG00000105875	110.7019064	0.705814203	0.170607092	4.137074217	3.52E-05	0.000223888
ENSG00000105879	433.1408575	0.484899632	0.096881834	5.00506249	5.58E-07	5.23E-06
ENSG00000105887	712.1644698	0.552245928	0.08447141	6.537666719	6.25E-11	1.02E-09
ENSG00000105926	286.9010548	0.288203878	0.112584111	2.559898318	0.010470279	0.033517779
ENSG00000105928	186.452948	-0.106827452	0.144645083	-0.738548797	0.460181026	0.62341237
ENSG00000105939	1123.248254	0.400031077	0.070261591	5.693453194	1.24E-08	1.51E-07
ENSG00000105948	48.8757091	0.674559982	0.26121837	2.582360432	0.009812704	0.031738213
ENSG00000105953	685.6472844	-0.552091824	0.087089104	-6.3393903	2.31E-10	3.48E-09
ENSG00000105967	22.43206079	-4.619360021	0.717830862	-6.435164979	1.23E-10	1.93E-09
ENSG00000105968	467.4813006	-0.207812028	0.093304012	-2.227257141	0.025930093	0.071531818
ENSG00000105974	8.683549523	-6.399131177	1.269697058	-5.039888165	4.66E-07	4.41E-06
ENSG00000105983	637.7084533	0.483570872	0.084021491	5.755323643	8.65E-09	1.07E-07
ENSG00000105993	371.0455454	0.750692954	0.10631111	7.061284157	1.65E-12	3.22E-11
ENSG00000106009	297.3205653	0.013238279	0.118949166	0.111293583	0.911383544	0.952474119
ENSG00000106012	458.1246692	0.045373337	0.094833228	0.478453998	0.632327109	0.761995244
ENSG00000106025	128.7594252	-3.115587985	0.21305244	-14.62357333	1.99E-48	2.05E-46
ENSG00000106028	1162.07654	0.870923044	0.070531455	12.34800904	4.99E-35	3.25E-33
ENSG00000106031	902.4765508	-2.282623594	0.095980675	-23.78211638	5.11E-125	1.84E-122
ENSG00000106034	740.8985714	-3.793235616	0.113946136	-33.2897258	5.44E-243	5.09E-240
ENSG00000106038	65.8726786	-7.511445662	1.010867844	-7.430690078	1.08E-13	2.36E-12
ENSG00000106049	495.2543024	-0.723538656	0.094652772	-7.644135967	2.10E-14	4.87E-13
ENSG00000106052	821.6042917	-1.14799087	0.078947684	-14.54115949	6.65E-48	6.62E-46
ENSG00000106070	64.40921421	-3.334051593	0.316477009	-10.53489354	5.97E-26	2.80E-24
ENSG00000106077	178.4019843	-0.007959306	0.136895807	-0.058141343	0.953636043	0.974659888
ENSG00000106080	263.0030396	0.364022344	0.119602189	3.043609372	0.002337585	0.009331074
ENSG00000106086	211.8783787	-0.078483797	0.126180169	-0.621997877	0.533943264	0.685234734
ENSG00000106089	20.23397666	0.362255825	0.388470375	0.932518536	0.351068612	0.522671567
ENSG00000106100	70.60216194	0.194306972	0.218563556	0.889018166	0.373993317	0.544085443
ENSG00000106105	1539.800768	-0.219533611	0.0713324	-3.07761426	0.002086648	0.008461648
ENSG00000106125	23.92782261	0.052191172	0.368250255	0.141727456	0.887295287	0.93824263
ENSG00000106144	511.4343816	0.78745693	0.100969091	7.798989963	6.24E-15	1.49E-13
ENSG00000106153	1616.014366	0.25214938	0.15716893	1.604320782	0.108643362	0.223737474
ENSG00000106211	2206.636676	1.885490929	0.075303704	25.03848856	2.33E-138	1.06E-135
ENSG00000106244	1011.333868	0.334722296	0.078224102	4.279017442	1.88E-05	0.000126652
ENSG00000106245	512.4692095	-0.123349515	0.089908112	-1.371950899	0.170078724	0.313030658
ENSG00000106246	122.8479299	0.012232193	0.164438095	0.074387828	0.940701794	0.967262249
ENSG00000106261	930.92386	-0.003710477	0.074026732	-0.050123475	0.960023993	0.978814678
ENSG00000106263	2294.906892	0.398150823	0.060694088	6.55996056	5.38E-11	8.86E-10
ENSG00000106266	275.3800908	0.3932215	0.115192823	3.413593732	0.000641121	0.00301905
ENSG00000106268	201.2242515	-0.003525021	0.134107072	-0.026285122	0.979029921	0.989312517
ENSG00000106290	587.7393891	-0.105881468	0.087184498	-1.214452915	0.224574819	0.382778461
ENSG00000106299	762.2783031	0.776707233	0.080832745	9.608819158	7.34E-22	2.74E-20
ENSG00000106305	161.4043846	0.257453037	0.156257375	1.64762167	0.099430331	0.208805181
ENSG00000106327	249.6005251	0.763117821	0.119692644	6.375645119	1.82E-10	2.78E-09
ENSG00000106330	46.9651392	-0.076312753	0.261926408	-0.291351886	0.770782208	0.862998381
ENSG00000106333	1563.79412	0.004843145	0.072970031	0.066371703	0.947081898	0.970644931
ENSG00000106344	1162.595125	0.66897149	0.069789518	9.585558205	9.20E-22	3.42E-20

ENSG00000106346	404.5133644	0.155988872	0.100467071	1.552636813	0.120509926	0.2426502
ENSG00000106348	964.1941735	1.136822921	0.075378352	15.08155705	2.14E-51	2.41E-49
ENSG00000106351	62.97008357	-0.941334031	0.234075792	-4.021492453	5.78E-05	0.000350149
ENSG00000106355	146.4130107	-0.031758037	0.149158822	-0.212914239	0.831393856	0.901477895
ENSG00000106366	62.8436046	-0.463063407	0.22146284	-2.090930497	0.036534295	0.094755768
ENSG00000106367	379.6395023	0.240938564	0.105149525	2.29138993	0.021940871	0.062263317
ENSG00000106392	154.8866826	-0.195879179	0.146176006	-1.340022793	0.180237935	0.326473536
ENSG00000106397	595.0602041	0.490385457	0.085281282	5.750212067	8.91E-09	1.10E-07
ENSG00000106399	140.6817687	-0.228925621	0.152532351	-1.50083323	0.133398702	0.261750019
ENSG00000106400	339.1635855	0.349300008	0.117131049	2.982129936	0.002862505	0.011098428
ENSG00000106404	42.64532107	0.377058364	0.274864409	1.371797699	0.170126424	0.313042425
ENSG00000106415	42.93339182	0.41395868	0.269103243	1.538289454	0.123977857	0.248044005
ENSG00000106436	17.3816961	0.949394126	0.451017209	2.105006433	0.035290748	0.09202323
ENSG00000106443	952.7655835	-0.017631475	0.076207088	-0.231362666	0.817033066	0.892446616
ENSG00000106459	141.9922011	0.259044093	0.151688176	1.707740841	0.087684441	0.189347169
ENSG00000106460	273.7505045	0.05075166	0.120855989	0.419935	0.674532939	0.792834423
ENSG00000106462	462.0157113	0.432389344	0.09372977	4.613148464	3.97E-06	3.10E-05
ENSG00000106477	124.2152076	0.280016304	0.165805883	1.688820074	0.091253916	0.19555206
ENSG00000106479	86.06685056	0.334289956	0.200611911	1.666351487	0.095643427	0.202792939
ENSG00000106484	462.3610238	-1.593444529	0.109971673	-14.48959075	1.41E-47	1.39E-45
ENSG00000106511	64.79630428	2.189682127	0.253349579	8.642927828	5.48E-18	1.64E-16
ENSG00000106524	72.262358	-0.083361418	0.206230336	-0.404215112	0.686054542	0.801843308
ENSG00000106537	57.31930503	0.831893881	0.240765789	3.455199691	0.000549885	0.00263806
ENSG00000106546	9.571683561	-3.286340573	0.786489021	-4.17849517	2.93E-05	0.000189867
ENSG00000106554	707.417999	0.564440985	0.082089519	6.875920236	6.16E-12	1.11E-10
ENSG00000106571	318.2577553	-0.818494431	0.116803396	-7.007454046	2.43E-12	4.65E-11
ENSG00000106588	26.69655652	0.302364197	0.339607027	0.890335516	0.373285756	0.54348518
ENSG00000106591	296.7994107	-0.278136567	0.120175395	-2.314421906	0.02064459	0.059303591
ENSG00000106603	299.1983321	-0.259223589	0.123633481	-2.096710274	0.03601923	0.093640149
ENSG00000106605	165.7355645	-0.168690686	0.142547471	-1.183400058	0.236650634	0.397778085
ENSG00000106608	283.8563598	0.164096417	0.117449214	1.397169145	0.162362713	0.302361001
ENSG00000106609	598.086414	0.114817331	0.088885138	1.291749481	0.196443916	0.347660494
ENSG00000106615	440.2927462	0.781672766	0.115719836	6.754872759	1.43E-11	2.48E-10
ENSG00000106617	176.1426564	0.500116436	0.137646768	3.633332199	0.000279784	0.001446497
ENSG00000106628	1224.946119	0.317660866	0.068473249	4.639196631	3.50E-06	2.78E-05
ENSG00000106635	329.8352052	-0.015519222	0.116639947	-0.133052373	0.894151961	0.942712147
ENSG00000106636	332.7900609	0.168048207	0.108983111	1.541965593	0.123081955	0.246814021
ENSG00000106638	242.9923935	-0.046936157	0.118731161	-0.395314565	0.692610751	0.806457495
ENSG00000106665	142.3835489	-0.17835919	0.154366939	-1.155423506	0.247917041	0.412129074
ENSG00000106682	998.0735439	0.237726536	0.072431408	3.282091906	0.0010304	0.004616916
ENSG00000106683	454.6226334	0.459213396	0.09532038	4.817578329	1.45E-06	1.24E-05
ENSG00000106686	15.78776853	0.309179859	0.445032	0.694736241	0.487220612	0.647676578
ENSG00000106688	16.63392945	1.976165708	0.482847879	4.092729394	4.26E-05	0.000266396
ENSG00000106692	269.3973461	-0.058072546	0.118651882	-0.489436367	0.624532795	0.756146541
ENSG00000106701	42.7008077	-0.210279119	0.269378886	-0.780607279	0.43503351	0.601231376
ENSG00000106714	29.53562906	-0.789933766	0.32656659	-2.418905638	0.015567278	0.047006353
ENSG00000106723	546.3939155	-0.085537265	0.09064118	-0.943690984	0.345327583	0.517089495
ENSG00000106733	24.47662419	0.417094912	0.357986423	1.165113772	0.243972943	0.407162638
ENSG00000106771	446.6751441	-0.236341499	0.096137183	-2.458377608	0.013956634	0.042768071
ENSG00000106780	86.29908559	0.069709587	0.191744457	0.363554641	0.716190604	0.823334922

ENSG00000106785	583.3493649	0.2887108	0.091105659	3.168966698	0.001529819	0.006494219
ENSG00000106789	20.84017038	-1.384211905	0.405388897	-3.41452841	0.000638925	0.003010729
ENSG00000106799	223.0670905	-0.48335114	0.125087711	-3.864097715	0.000111501	0.000635653
ENSG00000106803	197.0760163	0.229640722	0.142746565	1.608730275	0.107675333	0.222330648
ENSG00000106804	57.36319664	-2.391774247	0.281206066	-8.505414828	1.81E-17	5.17E-16
ENSG00000106829	1054.79245	0.553866653	0.070933441	7.80825863	5.80E-15	1.39E-13
ENSG00000106852	124.7343863	0.417256874	0.162864313	2.561990822	0.010407407	0.033384984
ENSG00000106853	392.1972176	-0.160334928	0.098814688	-1.622581948	0.104678802	0.217357448
ENSG00000106948	211.3459942	-0.285635141	0.130121854	-2.195135811	0.028153857	0.076509427
ENSG00000106976	419.4609526	0.048961223	0.105119967	0.465765209	0.641383571	0.768467675
ENSG00000106991	366.3405602	2.397377588	0.119539142	20.05516811	1.82E-89	3.99E-87
ENSG00000106992	20.81059902	0.623207892	0.405958157	1.535153024	0.124746244	0.249049246
ENSG00000106993	56.00429795	0.056359675	0.23974168	0.23508501	0.814142753	0.890857104
ENSG00000107020	57.16187372	-0.7022001	0.250505646	-2.803130828	0.005060914	0.018087784
ENSG00000107021	130.5831061	0.251083945	0.159483453	1.574357342	0.11540486	0.234315968
ENSG00000107036	373.2075984	-0.452737737	0.105020378	-4.310951309	1.63E-05	0.000111331
ENSG00000107077	336.7066217	0.079627995	0.112239989	0.709444076	0.478048944	0.638443955
ENSG00000107099	61.32741192	-2.599990706	0.280073721	-9.283236919	1.64E-20	5.67E-19
ENSG00000107104	668.3670499	0.170016267	0.087112286	1.951691038	0.050974897	0.123690514
ENSG00000107130	409.6333608	0.425428309	0.106714031	3.986620164	6.70E-05	0.00040147
ENSG00000107140	169.4401846	-0.038138404	0.157733226	-0.241790551	0.808942461	0.887127391
ENSG00000107159	32.20131314	4.737570351	0.573323706	8.263342857	1.42E-16	3.80E-15
ENSG00000107164	756.1249057	-0.107860519	0.078310723	-1.377340353	0.168407045	0.310733451
ENSG00000107165	677.0682028	-0.028954633	0.086142762	-0.336123805	0.736777508	0.838085161
ENSG00000107175	168.7234125	0.297294988	0.138553778	2.145701065	0.031896836	0.084585513
ENSG00000107185	341.1712748	0.053202523	0.106755347	0.498359327	0.618230799	0.75188641
ENSG00000107186	961.0932255	0.09118859	0.073363358	1.242971862	0.213878202	0.369492942
ENSG00000107201	22.00406235	1.019275145	0.389184943	2.618999435	0.008818809	0.029024793
ENSG00000107223	1542.977035	0.342555701	0.069567143	4.924101926	8.47E-07	7.63E-06
ENSG00000107262	303.2281884	0.271164518	0.108254699	2.504875277	0.012249458	0.038339603
ENSG00000107263	443.6593826	0.088738071	0.096176491	0.922658648	0.356185122	0.527443855
ENSG00000107281	505.1842144	0.925214348	0.09662772	9.575040698	1.02E-21	3.76E-20
ENSG00000107290	1382.008633	0.002539197	0.071485129	0.035520627	0.971664599	0.985302874
ENSG00000107331	1030.697207	-0.015942188	0.088163157	-0.180825971	0.856504178	0.917790387
ENSG00000107338	25.10654184	0.025645101	0.357004	0.071834212	0.942733846	0.968520046
ENSG00000107341	697.2989301	0.07402655	0.079573822	0.930287717	0.352222136	0.523715899
ENSG00000107362	67.98927524	-0.750102205	0.22137906	-3.388315979	0.000703232	0.003285101
ENSG00000107371	152.6515676	0.072074686	0.145366875	0.495812309	0.62002684	0.753353258
ENSG00000107372	611.8141277	0.319713248	0.085921912	3.720974535	0.000198455	0.001063197
ENSG00000107404	393.8830026	0.019995308	0.099963497	0.200026095	0.841460173	0.907833414
ENSG00000107438	30.19361891	-0.961407372	0.328207697	-2.92926516	0.003397644	0.012864343
ENSG00000107443	183.4895765	-0.47152741	0.144791339	-3.256599547	0.001127554	0.0049933
ENSG00000107518	279.2524563	0.243268365	0.116727894	2.084063691	0.037154374	0.095956517
ENSG00000107521	111.9524406	-0.33378799	0.174797285	-1.909571936	0.056188351	0.133653282
ENSG00000107537	103.7296956	-0.742614207	0.178097956	-4.169695278	3.05E-05	0.000196805
ENSG00000107551	17.86333603	-1.435365021	0.440770322	-3.256491985	0.001127981	0.004993618
ENSG00000107554	174.762841	-0.061970247	0.137930519	-0.449285969	0.653225378	0.77746328
ENSG00000107560	229.0697293	0.269949084	0.127507891	2.117116683	0.034249946	0.089876517
ENSG00000107566	356.5721098	0.323717286	0.106442817	3.041231866	0.002356123	0.009388963
ENSG00000107581	8819.25433	0.196714635	0.050787183	3.873312559	0.000107366	0.000614827

ENSG00000107611	53.96068089	0.164492518	0.247059734	0.665800598	0.505538563	0.663115154
ENSG00000107614	76.88289885	-0.373135458	0.221130221	-1.687401464	0.091526182	0.195986019
ENSG00000107625	278.3276244	0.113686835	0.117124303	0.970651113	0.331722044	0.504187357
ENSG00000107643	253.8928927	-0.112368699	0.126051825	-0.89144841	0.372688657	0.543151359
ENSG00000107651	524.6183557	0.183103531	0.094737041	1.932755434	0.053266333	0.128100013
ENSG00000107669	316.5533361	0.032538835	0.108981069	0.298573275	0.765265659	0.858571124
ENSG00000107672	192.1225086	0.020919729	0.133884186	0.156252428	0.875834053	0.931201069
ENSG00000107679	166.3753496	-0.207661935	0.145417326	-1.428041221	0.153279994	0.28985747
ENSG00000107719	303.2232429	0.154868862	0.111015581	1.395019156	0.163010063	0.30328508
ENSG00000107731	97.01345388	-0.455297776	0.183599696	-2.479839486	0.013144154	0.040713546
ENSG00000107742	141.2403652	-2.992391993	0.211071923	-14.17712008	1.27E-45	1.17E-43
ENSG00000107745	434.2438311	0.427625894	0.098311539	4.34970198	1.36E-05	9.50E-05
ENSG00000107758	235.6069157	0.295442956	0.122148685	2.418715817	0.015575403	0.04702078
ENSG00000107771	316.7272368	0.044776466	0.11022744	0.406218871	0.68458179	0.800759464
ENSG00000107779	221.4344497	0.312229028	0.131410584	2.375980824	0.017502372	0.051857377
ENSG00000107789	125.5736662	-0.723542739	0.171242644	-4.225248581	2.39E-05	0.000157848
ENSG00000107796	196.3146979	-0.15320834	0.145301678	-1.054415486	0.291692735	0.462252691
ENSG00000107798	620.0694092	0.310400993	0.085789662	3.618163159	0.000296701	0.00152442
ENSG00000107815	307.1816114	0.154344176	0.109047777	1.415381224	0.156956752	0.29483438
ENSG00000107816	439.4947586	-0.032600628	0.116708189	-0.279334534	0.779988106	0.868394424
ENSG00000107819	55.28877738	-0.218583692	0.251854211	-0.867897706	0.385450335	0.554301889
ENSG00000107821	295.5891091	-0.39046225	0.120376632	-3.243671509	0.001179997	0.00518306
ENSG00000107829	178.4885685	-0.237095779	0.150678074	-1.573525411	0.115597209	0.234516753
ENSG00000107833	451.2193646	0.814525381	0.117146167	6.95306897	3.57E-12	6.69E-11
ENSG00000107854	609.2376527	0.058342107	0.08914212	0.654484172	0.512799921	0.668768034
ENSG00000107862	1075.403198	0.073995889	0.070604951	1.048026909	0.294626209	0.464881456
ENSG00000107863	318.8359213	-0.181108031	0.110588759	-1.63767125	0.101490291	0.212147934
ENSG00000107864	108.1328697	-0.273606954	0.173766736	-1.574564613	0.115356976	0.234266221
ENSG00000107872	133.8712704	0.717578368	0.156550473	4.583687008	4.57E-06	3.53E-05
ENSG00000107874	296.1850399	-0.337262614	0.11229292	-3.003418336	0.002669652	0.010451672
ENSG00000107882	137.0070083	-0.1040166	0.158750417	-0.65522096	0.512325502	0.668273407
ENSG00000107890	372.6887842	-0.286207129	0.104000779	-2.751970997	0.005923777	0.020637721
ENSG00000107897	249.9966129	0.249617697	0.125613036	1.987195798	0.046900703	0.116049706
ENSG00000107902	159.1573291	-0.463254203	0.14739789	-3.142882181	0.001672932	0.007021115
ENSG00000107929	1061.88298	0.019636806	0.071678613	0.273956274	0.784118225	0.871011269
ENSG00000107937	1112.734695	-0.022179413	0.073654483	-0.301127811	0.763317034	0.857280464
ENSG00000107938	278.9890929	-0.405112827	0.118504674	-3.418538816	0.000629583	0.002974691
ENSG00000107949	573.7734287	0.258601195	0.088202865	2.931891102	0.003369049	0.01276984
ENSG00000107951	204.6478084	-0.036364697	0.128279491	-0.283480208	0.776808751	0.866224699
ENSG00000107957	1277.473828	-0.232020763	0.073003692	-3.178205866	0.001481895	0.006327106
ENSG00000107959	384.7279638	0.029104078	0.103125668	0.282219536	0.77777518	0.86696492
ENSG00000107960	22.36719564	0.272442728	0.375214338	0.726098926	0.467778128	0.629756504
ENSG00000107968	11.86118267	-1.701782562	0.593772155	-2.866053161	0.004156244	0.015311554
ENSG00000107984	811.7001509	-2.298466365	0.091068541	-25.23886234	1.50E-140	7.03E-138
ENSG00000108010	321.8150009	0.352918416	0.113836725	3.100215819	0.001933797	0.007952003
ENSG00000108021	1194.873782	0.070392506	0.070734343	0.995167311	0.319654898	0.491322064
ENSG00000108039	473.6874442	0.270676644	0.097442681	2.777803758	0.005472766	0.019301015
ENSG00000108055	1516.535822	0.129495119	0.070686814	1.831955796	0.066958007	0.153661023
ENSG00000108061	240.1846321	-0.244622984	0.124322723	-1.967644987	0.0491089	0.120234286
ENSG00000108064	413.5847455	-0.166807476	0.108862826	-1.53227214	0.125455293	0.250109353

ENSG00000108091	811.5693887	0.149978286	0.07738214	1.938151181	0.052604781	0.126921909
ENSG00000108094	283.2396032	-0.0534321	0.118314703	-0.451609973	0.651549984	0.776080949
ENSG00000108100	340.4263517	-0.091171188	0.105725078	-0.862342117	0.388499283	0.55708957
ENSG00000108106	678.4493886	0.099598772	0.169322808	0.588218286	0.556385785	0.703406635
ENSG00000108107	9677.684438	0.058313645	0.076545875	0.761813037	0.446171598	0.611152846
ENSG00000108175	1877.008738	0.26139129	0.070785517	3.692722767	0.000221866	0.0011734
ENSG00000108179	341.5858566	-0.044459446	0.106439458	-0.417697039	0.676168637	0.794177656
ENSG00000108187	18.30432371	-0.874490101	0.41909525	-2.086614202	0.036923022	0.095517071
ENSG00000108219	226.8959704	-0.048641071	0.126158456	-0.385555376	0.699825987	0.811562424
ENSG00000108239	136.1489407	-0.229934758	0.154979423	-1.483647008	0.137902591	0.26792776
ENSG00000108256	1871.247094	-0.075963559	0.062572911	-1.214000719	0.22474745	0.382809194
ENSG00000108262	645.1054568	0.348355084	0.082173789	4.239248171	2.24E-05	0.000149233
ENSG00000108298	7046.821611	0.300881075	0.05578411	5.393669884	6.90E-08	7.45E-07
ENSG00000108306	366.9124487	-0.149705062	0.104184897	-1.436917117	0.150741546	0.286182794
ENSG00000108309	63.10715118	-0.329517337	0.224164995	-1.469976777	0.141568044	0.273170733
ENSG00000108312	1834.021382	0.150221972	0.062872612	2.389306998	0.01688019	0.050257744
ENSG00000108344	872.0453113	0.381475049	0.077900142	4.89697504	9.73E-07	8.63E-06
ENSG00000108349	1198.818788	0.151967137	0.069149998	2.197644832	0.027974426	0.076110161
ENSG00000108352	76.31025818	0.673096505	0.20395796	3.300172768	0.000966253	0.004354494
ENSG00000108370	34.20129022	-0.458912869	0.314515645	-1.459109828	0.144534882	0.277398754
ENSG00000108379	51.42932803	-0.573492442	0.249015892	-2.303035505	0.021276846	0.060752394
ENSG00000108384	264.6416704	-0.118243226	0.118500392	-0.997829826	0.318361885	0.48997802
ENSG00000108389	520.2953362	0.49635124	0.092030661	5.393324762	6.92E-08	7.46E-07
ENSG00000108395	487.5856397	0.009462665	0.092703088	0.102074968	0.91869717	0.955355281
ENSG00000108406	216.5628444	0.112239271	0.127368971	0.881213606	0.378202216	0.547522058
ENSG00000108423	107.2966845	-0.405266568	0.175973119	-2.303002698	0.021278691	0.060752394
ENSG00000108424	3551.163539	0.155751039	0.055319936	2.81545951	0.004870753	0.017507542
ENSG00000108433	193.2296046	-0.039032128	0.135340949	-0.288398514	0.773041705	0.864018874
ENSG00000108439	208.5956204	0.225528032	0.14789521	1.524917759	0.127279612	0.252485707
ENSG00000108443	798.4365092	-0.15065484	0.077530672	-1.943164385	0.0519963	0.125743187
ENSG00000108465	35.38531291	-0.34718382	0.311461741	-1.114691707	0.264982556	0.431693045
ENSG00000108468	896.2341931	-0.302151023	0.086070791	-3.510494327	0.000447274	0.002194503
ENSG00000108469	190.8959388	-0.172240012	0.136216818	-1.264454815	0.206066829	0.359535554
ENSG00000108474	74.30923366	-0.150258199	0.213511575	-0.70374732	0.481590146	0.642074486
ENSG00000108479	256.7819141	0.004320979	0.135952185	0.031783078	0.974645041	0.987011672
ENSG00000108506	300.861491	-0.276163411	0.11242419	-2.456441193	0.014032077	0.042943046
ENSG00000108509	332.8035454	0.573376192	0.107895735	5.314169198	1.07E-07	1.12E-06
ENSG00000108510	1136.660937	-0.180636605	0.077531079	-2.329860583	0.019813521	0.057326576
ENSG00000108511	451.6210196	-0.053777749	0.095598473	-0.562537743	0.573749696	0.718312119
ENSG00000108515	127.2341036	-2.019748926	0.186663852	-10.82024666	2.76E-27	1.35E-25
ENSG00000108518	4674.96535	-0.135522702	0.074965764	-1.807794581	0.070638478	0.160170895
ENSG00000108523	406.8610736	0.041863447	0.100880333	0.414981252	0.678155628	0.795757928
ENSG00000108528	363.8811547	-0.00596702	0.103615262	-0.057588233	0.954076623	0.974968305
ENSG00000108557	1151.101522	-0.146463414	0.075770541	-1.932986242	0.053237893	0.128053541
ENSG00000108559	545.5559914	0.285678809	0.088512366	3.227558159	0.001248517	0.005451636
ENSG00000108561	1391.673282	0.229018838	0.068468144	3.344896232	0.000823134	0.003776146
ENSG00000108578	418.5140099	-0.085345775	0.097680314	-0.873725435	0.382267796	0.551702017
ENSG00000108582	1039.001557	-0.013984025	0.074948762	-0.186581138	0.851989056	0.915116247
ENSG00000108587	283.3175056	0.007448336	0.117838371	0.063208066	0.949600822	0.972060789
ENSG00000108588	774.7318795	0.314699734	0.080415324	3.91342991	9.10E-05	0.000528618

ENSG00000108590	83.35416674	0.553123491	0.194379612	2.845583885	0.004433008	0.016169947
ENSG00000108591	209.1734878	0.417498745	0.130609165	3.196550137	0.001390817	0.005987374
ENSG00000108592	936.7459106	0.038198787	0.072706192	0.525385613	0.599315153	0.737889382
ENSG00000108599	285.528215	-0.008941509	0.120109829	-0.074444438	0.940656751	0.967262249
ENSG00000108604	779.1526689	0.319202439	0.084511067	3.777048974	0.000158698	0.000871812
ENSG00000108639	216.2060272	0.211192852	0.131305916	1.608403179	0.107746906	0.222413047
ENSG00000108641	88.80560831	0.250934636	0.186444917	1.345891537	0.178337499	0.324033998
ENSG00000108651	239.1976402	-0.001947067	0.121613307	-0.016010311	0.987226166	0.993349947
ENSG00000108654	6267.959903	-0.031579972	0.05116577	-0.617208968	0.537096901	0.68781112
ENSG00000108666	80.13648584	0.077075967	0.196458002	0.392327961	0.694815906	0.808422019
ENSG00000108669	442.2479017	0.351190918	0.095456498	3.67906768	0.000234088	0.001229707
ENSG00000108671	614.0293279	0.423360353	0.104409079	4.054823176	5.02E-05	0.000308703
ENSG00000108679	181.3523655	2.212080054	0.185185738	11.9451966	6.88E-33	4.13E-31
ENSG00000108733	20.52376878	0.313775425	0.381640128	0.822176186	0.41097663	0.579326515
ENSG00000108771	13.15370416	2.145537482	0.543844178	3.945132762	7.98E-05	0.000469543
ENSG00000108773	474.8415725	0.577552039	0.092873475	6.218697396	5.01E-10	7.25E-09
ENSG00000108774	284.586813	0.277685505	0.129279448	2.147947795	0.031717903	0.084207405
ENSG00000108784	47.34164176	0.482201774	0.268057893	1.798871762	0.072038968	0.162402726
ENSG00000108786	120.3142475	1.438024154	0.176636827	8.141134437	3.92E-16	1.03E-14
ENSG00000108788	347.2979796	0.25546267	0.116411695	2.194475989	0.028201208	0.076623283
ENSG00000108797	645.4809635	0.41496791	0.085702289	4.841969963	1.29E-06	1.11E-05
ENSG00000108799	189.0134602	0.597218356	0.137669445	4.338060299	1.44E-05	9.97E-05
ENSG00000108819	2523.009511	0.22280387	0.067376901	3.306828686	0.000943586	0.004266534
ENSG00000108821	83.24892244	-2.012840454	0.230075068	-8.748624848	2.16E-18	6.70E-17
ENSG00000108823	102.8505563	0.038822339	0.175725942	0.220925483	0.825150455	0.897892048
ENSG00000108826	438.1948393	0.191686462	0.097232804	1.971417618	0.048676127	0.119495554
ENSG00000108828	601.4711553	-0.078040038	0.087147065	-0.89549818	0.370520838	0.541536387
ENSG00000108829	1530.595374	0.249605607	0.072804869	3.428419171	0.000607107	0.002879148
ENSG00000108830	162.982506	-0.641166932	0.152400095	-4.20712949	2.59E-05	0.000169768
ENSG00000108840	403.8458369	0.491424636	0.100723617	4.878941491	1.07E-06	9.38E-06
ENSG00000108848	2666.775811	0.191254977	0.067308634	2.841462768	0.00449071	0.016342227
ENSG00000108852	181.5055785	-0.34464155	0.138589091	-2.486786995	0.012890255	0.040041887
ENSG00000108854	306.1860077	-0.34162913	0.116088669	-2.942829247	0.003252277	0.012367575
ENSG00000108861	317.3450719	0.116450117	0.106952876	1.088798362	0.276242814	0.444743015
ENSG00000108883	1022.088936	0.010766657	0.070987865	0.151668985	0.879448021	0.933918276
ENSG00000108932	19.21477326	0.13342366	0.401623062	0.332211151	0.739729824	0.840830623
ENSG00000108946	1341.600062	-0.159685251	0.068490245	-2.331503594	0.019726821	0.057111426
ENSG00000108947	73.01634094	-0.917357636	0.219130145	-4.186359834	2.83E-05	0.000184343
ENSG00000108953	2707.716366	-0.065465807	0.068506863	-0.955609472	0.339269574	0.511070291
ENSG00000108960	48.73938062	-0.114498641	0.257567716	-0.444538013	0.656653652	0.779779681
ENSG00000108961	119.8379186	0.05710094	0.167084922	0.341748015	0.732540534	0.83529482
ENSG00000108963	117.1028473	0.17519884	0.166033704	1.055200451	0.291333658	0.461787846
ENSG00000108984	728.3759254	-1.354473527	0.086297147	-15.69546123	1.62E-55	1.97E-53
ENSG00000109016	64.00350383	-0.402948801	0.225847072	-1.78416659	0.074396599	0.166382587
ENSG00000109046	431.6994461	0.127043172	0.10163484	1.249996279	0.211300907	0.366298879
ENSG00000109062	524.2339527	-0.167800267	0.092434511	-1.815342179	0.069471341	0.158111459
ENSG00000109063	18.96960973	0.02331505	0.421055193	0.055372908	0.955841379	0.976345539
ENSG00000109065	209.379058	0.357227585	0.132730417	2.691376958	0.007115774	0.024167137
ENSG00000109066	169.7713885	0.184504703	0.141010208	1.308449271	0.19072097	0.340500441
ENSG00000109079	164.250705	-0.317997895	0.146283947	-2.173839995	0.029717154	0.079898384

ENSG00000109083	46.27210327	0.302324071	0.264025554	1.145056099	0.25218592	0.416857568
ENSG00000109084	373.0972884	-0.510298581	0.102090778	-4.998478702	5.78E-07	5.38E-06
ENSG00000109089	411.3503479	0.233101705	0.098143498	2.37511103	0.017543672	0.051924981
ENSG00000109099	21.15814659	0.187845738	0.373348383	0.503137945	0.61486729	0.749417859
ENSG00000109103	164.6384898	0.038270154	0.144938177	0.264044679	0.791745491	0.875713229
ENSG00000109107	387.379874	0.622113578	0.109772364	5.667305985	1.45E-08	1.74E-07
ENSG00000109111	2072.604581	-1.31E-05	0.059188864	-0.000222009	0.999822862	0.999954923
ENSG00000109113	841.417919	0.088643145	0.076706093	1.155620654	0.247836356	0.412129074
ENSG00000109118	580.7139698	-0.214052484	0.086763199	-2.467088419	0.013621672	0.041897005
ENSG00000109133	662.7723332	0.141961386	0.0823574	1.723723495	0.084757772	0.184720248
ENSG00000109171	604.7105646	-0.063155554	0.084190937	-0.750146708	0.453166351	0.617396281
ENSG00000109180	814.1771143	0.081779765	0.076650987	1.066910784	0.286012125	0.455666589
ENSG00000109184	219.6796736	-0.067416465	0.126878437	-0.531346904	0.595178408	0.734921862
ENSG00000109189	174.5933988	0.022276469	0.13942061	0.159778883	0.873055259	0.929780684
ENSG00000109220	22.77973965	-0.035212835	0.359939772	-0.097829797	0.922067446	0.956947278
ENSG00000109265	111.4416801	0.816759656	0.187139438	4.364444321	1.27E-05	8.92E-05
ENSG00000109270	95.56218757	0.007192902	0.195667449	0.036760849	0.970675691	0.984695705
ENSG00000109320	545.1059708	0.540466674	0.091863895	5.883341576	4.02E-09	5.18E-08
ENSG00000109323	176.1739883	-0.055014841	0.136113514	-0.404183536	0.686077759	0.801843308
ENSG00000109332	1240.815227	0.201354677	0.071779378	2.805188394	0.005028718	0.017987879
ENSG00000109339	70.65340495	-0.919985982	0.223719312	-4.112233198	3.92E-05	0.00024683
ENSG00000109381	239.5238046	-0.143958708	0.121523016	-1.184620932	0.236167357	0.397355584
ENSG00000109390	492.6476541	0.703181374	0.095817719	7.338740503	2.16E-13	4.56E-12
ENSG00000109436	282.6621185	0.341183311	0.112515118	3.032333038	0.002426713	0.009634833
ENSG00000109445	224.9671738	0.464711886	0.123670721	3.757654871	0.000171513	0.00093165
ENSG00000109452	298.340583	-0.362359251	0.120466502	-3.007966907	0.002630018	0.010319514
ENSG00000109458	160.719637	-0.553042398	0.153797874	-3.595904047	0.000323267	0.00164229
ENSG00000109466	69.13415989	0.374278178	0.209836145	1.783668763	0.074477505	0.166484009
ENSG00000109472	214.136994	0.671204385	0.133943629	5.011096005	5.41E-07	5.10E-06
ENSG00000109475	1838.219373	0.445609666	0.072551954	6.141938836	8.15E-10	1.15E-08
ENSG00000109501	155.2470555	0.18825332	0.146897304	1.281530127	0.200007525	0.352068384
ENSG00000109519	455.2001422	0.214215957	0.093641851	2.287609173	0.022160291	0.062658134
ENSG00000109534	276.9365661	0.584097919	0.146591238	3.984534997	6.76E-05	0.000404839
ENSG00000109536	250.2575413	-0.031523662	0.121399568	-0.259668648	0.795119379	0.87776105
ENSG00000109572	636.6802983	0.077535198	0.083441547	0.929215732	0.352777296	0.524165716
ENSG00000109576	157.9333868	0.184615635	0.143025494	1.290788305	0.196777094	0.347995448
ENSG00000109586	206.6615757	-0.888356228	0.139361738	-6.374462917	1.84E-10	2.80E-09
ENSG00000109606	1180.220141	0.053079348	0.072862269	0.728488817	0.466314412	0.628880558
ENSG00000109618	120.3400013	-0.799344391	0.167891115	-4.761088112	1.93E-06	1.61E-05
ENSG00000109654	235.9187501	-0.291675511	0.128368322	-2.272176709	0.023075839	0.064855205
ENSG00000109670	275.1001612	-0.216849815	0.112767594	-1.922979882	0.054482576	0.130481271
ENSG00000109674	68.955627	-0.106421751	0.211645029	-0.502831328	0.615082866	0.749615566
ENSG00000109680	13.9091691	0.285987198	0.470410687	0.60795217	0.543219197	0.692180702
ENSG00000109685	1760.432975	-0.082709526	0.062434462	-1.324741554	0.185256886	0.333158811
ENSG00000109686	200.898007	-0.754468092	0.132771752	-5.682444354	1.33E-08	1.61E-07
ENSG00000109689	328.6739968	0.132855676	0.105593859	1.258176163	0.208328054	0.362714944
ENSG00000109705	216.8329993	5.860206859	0.310001371	18.90380946	1.06E-79	1.94E-77
ENSG00000109736	199.4819175	0.982687299	0.141065091	6.966197581	3.26E-12	6.11E-11
ENSG00000109738	14.47177806	1.856482648	0.494296592	3.755807095	0.000172784	0.000937826
ENSG00000109756	308.6579473	-0.316822888	0.108269941	-2.926231288	0.003430958	0.012974608

ENSG00000109762	142.8761656	0.043457098	0.151384381	0.287064606	0.774062852	0.864738597
ENSG00000109771	11.11617052	-0.31924364	0.520318012	-0.613554851	0.539509526	0.689580557
ENSG00000109775	151.8158813	0.28389218	0.15304225	1.854992201	0.06359734	0.147490811
ENSG00000109787	310.5217596	0.332271008	0.109535024	3.03346818	0.002417602	0.009606805
ENSG00000109790	541.547219	-0.734594789	0.090450688	-8.121494798	4.60E-16	1.20E-14
ENSG00000109794	95.60972588	0.052956116	0.194306575	0.272538981	0.785207623	0.871396457
ENSG00000109805	394.342157	0.085992401	0.101527491	0.846986368	0.39700272	0.564900446
ENSG00000109814	449.5461904	0.103918371	0.092882492	1.118815491	0.263218861	0.429734466
ENSG00000109861	335.6194722	0.435451216	0.116929459	3.724050539	0.000196052	0.00105072
ENSG00000109881	206.0360645	-0.051607106	0.136331211	-0.378542124	0.705027907	0.815025133
ENSG00000109911	165.5041935	-0.206914509	0.146411211	-1.41324224	0.157584511	0.295736756
ENSG00000109917	296.9820312	0.114303259	0.114707564	0.996475348	0.319019239	0.490506048
ENSG00000109919	488.3601969	0.093417326	0.101066596	0.924314554	0.355322567	0.526906758
ENSG00000109920	1044.971919	0.154854924	0.071818456	2.156199561	0.031068086	0.082842331
ENSG00000109927	11.52487112	-0.391426205	0.506711075	-0.772484013	0.439827812	0.605118636
ENSG00000109929	560.3300933	1.257921376	0.091586277	13.73482374	6.28E-43	5.38E-41
ENSG00000109971	9665.767498	0.486174987	0.060895198	7.983798472	1.42E-15	3.59E-14
ENSG00000110011	88.45727384	-0.105012502	0.191226154	-0.549153449	0.582900147	0.724986574
ENSG00000110013	112.2751626	-0.584710853	0.169726099	-3.445026165	0.000571004	0.002724489
ENSG00000110031	17.71618756	-0.775600721	0.420666705	-1.843741644	0.065220801	0.150559834
ENSG00000110042	58.77838143	-1.168978863	0.242146008	-4.827578498	1.38E-06	1.19E-05
ENSG00000110046	166.9719324	-0.467766008	0.171965363	-2.720117594	0.00652587	0.022412934
ENSG00000110047	172.895951	0.186849284	0.14241744	1.311983162	0.189525816	0.338829423
ENSG00000110048	525.3969622	-0.168430775	0.092482177	-1.821224154	0.06857279	0.156497479
ENSG00000110057	66.74240488	1.081931361	0.225167989	4.804996333	1.55E-06	1.32E-05
ENSG00000110060	78.60517702	0.22632969	0.196753224	1.150322652	0.250011004	0.413944434
ENSG00000110063	182.2632172	0.094872054	0.145361477	0.652662977	0.513973576	0.669862756
ENSG00000110066	427.0195359	0.015969997	0.102237148	0.156205428	0.875871099	0.931201069
ENSG00000110074	159.8148368	-0.363880642	0.151125719	-2.407800895	0.016048931	0.048242956
ENSG00000110075	1335.779432	-0.052440649	0.067292807	-0.779290554	0.435808578	0.601888024
ENSG00000110080	49.33074707	0.114934182	0.246302287	0.466638713	0.640758385	0.768195995
ENSG00000110090	988.7502804	0.53534615	0.073395603	7.293981192	3.01E-13	6.29E-12
ENSG00000110092	3451.695048	0.424397743	0.057252555	7.412730165	1.24E-13	2.69E-12
ENSG00000110104	431.4940919	0.321385961	0.107414514	2.992016142	0.002771416	0.010783956
ENSG00000110107	617.6933056	0.34795388	0.097795026	3.557991584	0.000373701	0.001872105
ENSG00000110108	787.0828522	0.636746145	0.090686942	7.021365292	2.20E-12	4.22E-11
ENSG00000110171	59.23290214	0.399774178	0.227617788	1.756339792	0.079030407	0.174742661
ENSG00000110172	483.5825115	0.338926515	0.093802826	3.613180205	0.000302464	0.001550061
ENSG00000110200	133.8069978	0.656011687	0.156086156	4.202881932	2.64E-05	0.000172476
ENSG00000110218	104.9560961	0.003195585	0.179076568	0.017844794	0.98576267	0.992622086
ENSG00000110237	320.3399663	0.391105377	0.112080709	3.489497717	0.000483929	0.002352164
ENSG00000110274	240.5820823	-0.214168559	0.124494615	-1.720303799	0.085377233	0.18573548
ENSG00000110315	72.0525787	-0.174362865	0.216227618	-0.806385728	0.420020473	0.587769236
ENSG00000110318	72.07634212	1.419172664	0.220640679	6.432053553	1.26E-10	1.96E-09
ENSG00000110321	5571.696312	0.088018527	0.068575765	1.283522357	0.199309132	0.35123515
ENSG00000110328	94.01795819	0.532991807	0.192962396	2.762153757	0.005742143	0.020079632
ENSG00000110330	208.5311494	-0.000589592	0.129206617	-0.00456317	0.99635913	0.998279095
ENSG00000110344	360.6755797	-0.060631818	0.1157814	-0.523674945	0.600504645	0.738900556
ENSG00000110367	1949.044261	-0.069553918	0.062861886	-1.106456107	0.268529152	0.435669785
ENSG00000110395	701.2656436	-0.093509135	0.083424574	-1.120882387	0.262337929	0.428598579

ENSG00000110400	304.7350628	-0.069242261	0.109037296	-0.635032815	0.525407013	0.678660352
ENSG00000110422	600.9836917	0.248244213	0.086946197	2.855147462	0.004301685	0.015760503
ENSG00000110427	17.5360771	-1.923152251	0.463481715	-4.149359483	3.33E-05	0.000213852
ENSG00000110429	72.93402371	0.349008001	0.218044509	1.600627336	0.109459484	0.225154103
ENSG00000110435	333.2718395	-0.136011821	0.104157057	-1.305833945	0.191609034	0.34183689
ENSG00000110442	67.77218521	0.305333297	0.213271291	1.431666191	0.152239376	0.288402768
ENSG00000110446	22.25560937	3.527635415	0.528850577	6.670382087	2.55E-11	4.31E-10
ENSG00000110492	5559.884918	0.584932383	0.137464422	4.255154712	2.09E-05	0.00013987
ENSG00000110497	419.1951609	-0.375684459	0.104543843	-3.593558924	0.000326192	0.001654755
ENSG00000110514	490.4372739	0.04622064	0.090363807	0.511495052	0.609004452	0.745246585
ENSG00000110536	70.66761583	-0.043760086	0.21832512	-0.200435415	0.841140064	0.907697202
ENSG00000110583	222.5631441	-0.327766076	0.130921183	-2.503537393	0.012295869	0.038467721
ENSG00000110619	500.6289666	-0.487109229	0.114303788	-4.261531811	2.03E-05	0.000136262
ENSG00000110628	81.8789556	-0.423252332	0.198990412	-2.126998617	0.033420198	0.088028037
ENSG00000110651	545.3998684	0.547719519	0.092673839	5.910184851	3.42E-09	4.43E-08
ENSG00000110660	229.6993669	0.192207933	0.123241542	1.559603437	0.118853624	0.239979424
ENSG00000110696	924.5498226	0.008881812	0.07686814	0.115546083	0.908012296	0.950719195
ENSG00000110697	182.7843377	0.004806446	0.14345107	0.033505825	0.973271221	0.986330341
ENSG00000110700	2352.917919	0.214110081	0.078228988	2.736966016	0.00620087	0.021432977
ENSG00000110711	343.9342656	0.261658377	0.105299026	2.484907858	0.012958497	0.040244972
ENSG00000110713	1790.992447	0.152910511	0.068322891	2.238056795	0.025217352	0.069771152
ENSG00000110717	702.4723567	0.170968961	0.101812946	1.679245783	0.093104153	0.19854775
ENSG00000110719	114.4659277	1.285026514	0.18529114	6.935175185	4.06E-12	7.56E-11
ENSG00000110721	221.0570244	-0.463698164	0.133903522	-3.462927311	0.000534333	0.00257047
ENSG00000110723	81.34567158	-1.800467674	0.219858354	-8.189216556	2.63E-16	6.97E-15
ENSG00000110756	119.7838506	-0.202506219	0.166496074	-1.216282246	0.223877422	0.382164648
ENSG00000110768	190.8957001	0.125433727	0.138537039	0.905416542	0.365244755	0.536504557
ENSG00000110801	44.25202057	0.128452159	0.261638932	0.490952008	0.623460392	0.755694894
ENSG00000110811	328.5482066	0.825303102	0.137079225	6.020628592	1.74E-09	2.35E-08
ENSG00000110841	346.8282512	-0.000335506	0.105933759	-0.003167132	0.997472999	0.99882401
ENSG00000110844	118.8267531	0.077167218	0.169964594	0.454019369	0.649814886	0.774539644
ENSG00000110851	198.179977	-0.058040664	0.14210845	-0.408425143	0.682961578	0.799479082
ENSG00000110852	15.49307453	3.810422036	0.661124033	5.763550933	8.24E-09	1.02E-07
ENSG00000110871	113.9645528	-0.420233955	0.181861618	-2.310734722	0.020847512	0.059751512
ENSG00000110880	560.1415604	-0.070150262	0.090221934	-0.777530025	0.436846127	0.602891221
ENSG00000110881	257.3084863	0.386166329	0.123705953	3.1216471	0.001798424	0.007461088
ENSG00000110888	173.8467175	0.172068393	0.139826451	1.230585428	0.218477958	0.375499801
ENSG00000110906	228.0862175	0.080774256	0.124095819	0.650902316	0.515109546	0.670845893
ENSG00000110911	461.9734461	0.058539884	0.094075476	0.622265083	0.533767577	0.68517163
ENSG00000110917	1194.035553	-0.166985344	0.067962007	-2.457039624	0.014008723	0.042890266
ENSG00000110921	220.2940605	0.921395981	0.136918881	6.729502703	1.70E-11	2.93E-10
ENSG00000110925	312.0519559	0.513921625	0.113748096	4.518067928	6.24E-06	4.69E-05
ENSG00000110931	463.8004173	0.241406842	0.092145708	2.619838152	0.008797151	0.028973875
ENSG00000110955	2988.267084	0.075067381	0.061288799	1.224814019	0.220645302	0.378391473
ENSG00000110958	1659.057634	0.200684191	0.072624564	2.763310105	0.005721838	0.020018593
ENSG00000110987	203.1268984	-0.12358565	0.134267576	-0.920442994	0.35734131	0.528654385
ENSG00000111011	1242.366865	-0.011103726	0.068452105	-0.162211603	0.871139223	0.928186766
ENSG00000111012	28.93396534	-0.120542865	0.330327364	-0.364919404	0.715171569	0.822500003
ENSG00000111057	191.6759621	0.26705212	0.136605246	1.954918475	0.050592694	0.123018101
ENSG00000111077	166.5739953	-1.160088265	0.153648725	-7.55026288	4.34E-14	9.89E-13

ENSG00000111087	190.6704582	0.113277536	0.134197764	0.844108962	0.39860852	0.566726101
ENSG00000111110	87.47350626	-0.533368327	0.198868178	-2.682019483	0.00731792	0.024763869
ENSG00000111142	1589.943209	0.094349952	0.067853406	1.39049692	0.164378034	0.305344914
ENSG00000111144	615.8256983	-0.04087493	0.097862948	-0.41767524	0.676184578	0.794177656
ENSG00000111145	415.2809531	0.334758272	0.101446146	3.299861889	0.000967324	0.004357923
ENSG00000111196	110.5855633	0.253871529	0.175299262	1.448217897	0.147556121	0.281757964
ENSG00000111199	28.06464257	-1.51716107	0.372619001	-4.071614882	4.67E-05	0.00028955
ENSG00000111203	167.9721288	-0.080821191	0.150522547	-0.536937437	0.59131083	0.732133279
ENSG00000111206	1178.192109	-0.066568501	0.068933074	-0.965697556	0.334195543	0.505990311
ENSG00000111224	65.58531854	-0.37749407	0.227592679	-1.658638901	0.097188578	0.204955405
ENSG00000111229	421.6714708	0.280473946	0.096392145	2.909717849	0.003617552	0.013583467
ENSG00000111231	89.20919376	-0.030428179	0.196197044	-0.155089896	0.876750447	0.931879051
ENSG00000111237	432.27079	0.216869795	0.111386825	1.946996834	0.051535114	0.124748191
ENSG00000111247	139.3718873	0.032158468	0.158138861	0.203355885	0.838856878	0.906138309
ENSG00000111252	344.2153277	-0.350554855	0.107640299	-3.256725011	0.001127056	0.004992668
ENSG00000111261	311.0527272	0.318056771	0.114576971	2.775922319	0.005504536	0.019398448
ENSG00000111266	402.0261049	0.334288666	0.104160743	3.209353689	0.001330337	0.005755235
ENSG00000111269	126.8822592	-0.18572166	0.162844888	-1.140481976	0.254085558	0.418964434
ENSG00000111271	212.9145802	-0.586888019	0.127474952	-4.603947766	4.15E-06	3.22E-05
ENSG00000111275	107.7443985	-0.961438387	0.180391922	-5.329719743	9.84E-08	1.04E-06
ENSG00000111276	320.2851986	-0.032986248	0.113515238	-0.290588722	0.771365887	0.863328623
ENSG00000111300	597.6594387	0.095135836	0.088112461	1.07970921	0.280271691	0.449067691
ENSG00000111319	13.72686559	-1.892633155	0.544137044	-3.478228832	0.000504739	0.002442324
ENSG00000111328	481.8060528	1.042622821	0.095438956	10.92449944	8.80E-28	4.38E-26
ENSG00000111331	257.5761022	1.535104932	0.137143749	11.19340067	4.39E-29	2.29E-27
ENSG00000111335	48.42808365	4.587930525	0.614936592	7.460818863	8.60E-14	1.89E-12
ENSG00000111341	12.93925693	-2.267104234	0.589090887	-3.848479553	0.000118853	0.000674558
ENSG00000111344	11.67884185	-0.375169687	0.498866742	-0.752043892	0.452024664	0.616884334
ENSG00000111358	194.3479884	0.538268121	0.136025019	3.957125865	7.59E-05	0.000448658
ENSG00000111361	580.4659516	1.22923143	0.092710586	13.25880341	4.01E-40	3.15E-38
ENSG00000111364	408.1393904	0.604147861	0.108638165	5.561101491	2.68E-08	3.10E-07
ENSG00000111371	2172.524685	-0.210973062	0.066408937	-3.176877571	0.001488699	0.006352293
ENSG00000111404	268.7293325	-0.375771689	0.127110855	-2.95625176	0.003114028	0.011919005
ENSG00000111412	253.2002793	-0.116192918	0.118494698	-0.980574827	0.326802453	0.498977615
ENSG00000111424	126.9188127	1.021145635	0.161590872	6.319327454	2.63E-10	3.93E-09
ENSG00000111445	352.9858216	-0.265941742	0.109217248	-2.434979328	0.01489264	0.045261125
ENSG00000111450	104.8556326	0.002122607	0.176046433	0.012057087	0.99038007	0.994984182
ENSG00000111481	772.1748449	0.166534395	0.079768314	2.087726162	0.036822543	0.095327362
ENSG00000111530	810.1581323	-0.022873726	0.081274145	-0.281439145	0.778373598	0.867349986
ENSG00000111540	522.3216852	-0.034203699	0.092040295	-0.371616571	0.710178349	0.818499776
ENSG00000111554	43.25332357	-0.440132959	0.278328138	-1.581345534	0.113799054	0.231839783
ENSG00000111581	358.1320265	0.032574243	0.107811548	0.30214057	0.762544906	0.856780379
ENSG00000111596	539.2631409	-0.168598051	0.091284632	-1.846948903	0.064754546	0.149704923
ENSG00000111602	1073.283257	0.003322649	0.070613668	0.047053913	0.962470258	0.980123222
ENSG00000111605	804.6789616	-0.355382234	0.083020925	-4.280634455	1.86E-05	0.000125857
ENSG00000111615	383.5649454	-0.205238622	0.100526237	-2.041642335	0.041187023	0.10456427
ENSG00000111639	412.7480761	0.070053322	0.097618812	0.717621127	0.472990927	0.634281962
ENSG00000111640	12455.22028	0.477844441	0.068875788	6.937770993	3.98E-12	7.43E-11
ENSG00000111641	84.2547093	0.067938936	0.199431053	0.34066378	0.733356709	0.835693316
ENSG00000111642	159.2954125	-0.059378652	0.145567271	-0.407912104	0.683338206	0.799704389

ENSG00000111647	252.3202749	0.107514466	0.119858583	0.897010993	0.369713051	0.541059478
ENSG00000111652	380.4509274	0.787378474	0.102119793	7.710341455	1.25E-14	2.94E-13
ENSG00000111653	38.05962627	-0.072947342	0.284219913	-0.256658098	0.797442721	0.879045586
ENSG00000111664	62.25968257	0.162178839	0.227150544	0.713970728	0.47524528	0.636211803
ENSG00000111665	254.7398604	0.154118382	0.116404968	1.323984574	0.185508168	0.333439953
ENSG00000111666	137.4091229	0.24820009	0.159634774	1.554799641	0.119993795	0.241899086
ENSG00000111667	540.6281277	0.071111424	0.093464801	0.760836416	0.446754781	0.611772705
ENSG00000111669	1288.48108	0.466254944	0.075043526	6.213126815	5.19E-10	7.51E-09
ENSG00000111670	178.8464603	-0.164244495	0.13928306	-1.179213712	0.238313087	0.399840264
ENSG00000111671	20.05024458	-1.021934255	0.398387405	-2.565177116	0.010312315	0.033163253
ENSG00000111676	2968.115928	-0.398101217	0.063357287	-6.283432181	3.31E-10	4.91E-09
ENSG00000111678	178.5210222	0.505180947	0.138214574	3.655048325	0.000257134	0.001338753
ENSG00000111679	53.89798465	-1.191597578	0.251900866	-4.730422706	2.24E-06	1.85E-05
ENSG00000111684	352.873834	-0.094677523	0.110700757	-0.855256324	0.392409261	0.560693001
ENSG00000111696	165.2879063	-0.276209277	0.147324188	-1.874839979	0.060814741	0.142401179
ENSG00000111707	344.9571689	-0.061764598	0.103332491	-0.597726782	0.550022252	0.698126192
ENSG00000111711	97.29007454	-0.074933936	0.180226007	-0.415777597	0.677572754	0.795342565
ENSG00000111716	237.1488144	0.296601945	0.127977722	2.317606066	0.02047074	0.058912618
ENSG00000111725	116.6636186	-0.225906625	0.176102299	-1.282814738	0.199556989	0.351583724
ENSG00000111726	106.1192179	0.115071599	0.17439306	0.659840471	0.509356209	0.665836024
ENSG00000111727	47.59035773	0.358192242	0.253289804	1.414159735	0.157315007	0.29530989
ENSG00000111731	321.0098255	0.040357121	0.105917656	0.381023547	0.703185776	0.814114448
ENSG00000111737	314.0163693	0.045405261	0.110369868	0.411391819	0.680785252	0.797846691
ENSG00000111752	96.24232773	-0.323731234	0.180532778	-1.793199203	0.072941078	0.163962765
ENSG00000111775	157.4840087	0.373803945	0.163338941	2.288517008	0.022107431	0.062572074
ENSG00000111785	155.464319	-0.165508881	0.155364528	-1.065293887	0.286742958	0.456572937
ENSG00000111786	819.126676	0.086013703	0.078864419	1.090652837	0.275425671	0.443783482
ENSG00000111788	38.04043691	0.033158754	0.287847147	0.115195702	0.908290005	0.950898041
ENSG00000111790	129.5179026	-0.191752606	0.173317073	-1.106368822	0.268566915	0.435680731
ENSG00000111799	14.90930452	-1.231737169	0.471462376	-2.612588475	0.008985943	0.02948506
ENSG00000111801	31.47694208	0.867514748	0.325124382	2.668254966	0.007624637	0.02569053
ENSG00000111802	133.5121128	0.119878913	0.157067819	0.763230262	0.445326084	0.61035179
ENSG00000111817	490.2161664	-0.780795803	0.094065793	-8.30052859	1.04E-16	2.81E-15
ENSG00000111832	321.9290861	-0.065381898	0.106100293	-0.616227304	0.53774451	0.688558843
ENSG00000111834	16.21297724	-0.960517776	0.450022787	-2.134375866	0.032812026	0.086637316
ENSG00000111843	178.8776084	0.08997674	0.141660857	0.635155982	0.525326689	0.678660352
ENSG00000111845	195.7565002	0.206223911	0.143463719	1.437463862	0.150586234	0.285965233
ENSG00000111846	340.6625151	-0.57931001	0.109795138	-5.276281089	1.32E-07	1.36E-06
ENSG00000111850	71.61726913	-0.685547279	0.21948487	-3.123437529	0.001787518	0.007422189
ENSG00000111860	178.9972177	-0.498875336	0.140321857	-3.555221875	0.00037766	0.001887898
ENSG00000111875	59.77747087	-0.124217442	0.229367583	-0.541564943	0.588118248	0.72968478
ENSG00000111877	238.1069624	0.188244567	0.123784469	1.520744635	0.128323933	0.253993248
ENSG00000111879	129.7056318	0.211570086	0.157432151	1.343881058	0.178986852	0.324901173
ENSG00000111880	202.7508551	-0.296575004	0.130558372	-2.271589331	0.023111326	0.064941947
ENSG00000111897	338.080823	-0.342472065	0.106121005	-3.227184521	0.001250148	0.005457064
ENSG00000111906	146.4139746	0.310641151	0.151127893	2.055485221	0.039832162	0.101509865
ENSG00000111911	226.2825383	0.295109663	0.130237774	2.26592988	0.023455677	0.065738606
ENSG00000111912	187.4801465	0.178163698	0.13524864	1.317304912	0.18773644	0.336283316
ENSG00000111961	246.3295153	0.337690974	0.123088785	2.743474764	0.006079272	0.02107491
ENSG00000111962	82.6635787	0.288456751	0.196342154	1.469153438	0.141791176	0.273402972

ENSG00000112029	258.2318521	-0.155676223	0.128629714	-1.210266417	0.22617668	0.384569454
ENSG00000112031	56.72980122	-0.677847343	0.241913006	-2.802029344	0.005078225	0.018137257
ENSG00000112033	812.0196955	0.335812617	0.083786515	4.007955411	6.12E-05	0.000369241
ENSG00000112039	100.118895	-0.115678934	0.186167876	-0.621368928	0.534356911	0.685427041
ENSG00000112062	198.514768	0.187691233	0.130578871	1.437378283	0.150610536	0.285972722
ENSG00000112078	676.9966431	0.038149624	0.085180675	0.447867129	0.654249093	0.778174175
ENSG00000112079	240.7785322	-0.412937807	0.129552774	-3.187409999	0.001435531	0.006153466
ENSG00000112081	1313.74638	-0.059661283	0.074236077	-0.803669656	0.421587783	0.589199442
ENSG00000112110	294.6932829	0.245713188	0.114174608	2.152082598	0.031390849	0.083512739
ENSG00000112118	1597.557756	-0.214076258	0.06675729	-3.206784743	0.001342274	0.005801514
ENSG00000112130	210.8015648	0.244297957	0.127692071	1.913180318	0.055724972	0.132852798
ENSG00000112139	27.83164189	-0.844183492	0.341404626	-2.472677368	0.013410515	0.041403627
ENSG00000112144	157.7539372	-0.304651337	0.147420062	-2.066552773	0.038776315	0.099378014
ENSG00000112146	259.0210457	0.093503486	0.122792121	0.761477899	0.446371674	0.611307684
ENSG00000112149	22.65975022	0.608629683	0.395927911	1.537223484	0.124238591	0.248318084
ENSG00000112159	2101.49072	-0.548914512	0.062933157	-8.722183009	2.73E-18	8.37E-17
ENSG00000112167	114.1122953	0.004813268	0.167053561	0.028812723	0.977013953	0.988555428
ENSG00000112175	289.6437583	-1.244684256	0.118780933	-10.478822	1.08E-25	4.99E-24
ENSG00000112183	21.17284902	-0.557816706	0.400229148	-1.39374333	0.163395127	0.30380031
ENSG00000112186	177.4886235	0.089863598	0.137960041	0.651374102	0.514805024	0.670635832
ENSG00000112200	509.5226855	-0.058215691	0.091252715	-0.637961197	0.523498939	0.677001436
ENSG00000112208	385.44393	0.490944407	0.100787361	4.871090975	1.11E-06	9.71E-06
ENSG00000112210	75.41979662	-0.114495055	0.20630599	-0.55497688	0.578910475	0.722326829
ENSG00000112218	97.27402906	-0.28227783	0.188811728	-1.495022753	0.134908504	0.263752228
ENSG00000112234	165.9391838	-0.122479722	0.155609773	-0.787095307	0.431226076	0.597556742
ENSG00000112237	143.0179267	-0.352378173	0.154783804	-2.27658298	0.02281114	0.064201179
ENSG00000112242	291.0502857	0.142501542	0.113812609	1.252071652	0.210543756	0.365654348
ENSG00000112249	947.9455709	0.491183775	0.078689989	6.242010992	4.32E-10	6.31E-09
ENSG00000112276	126.9921356	-3.828780532	0.256255989	-14.94123337	1.78E-50	1.90E-48
ENSG00000112282	202.4149605	0.005752116	0.138623419	0.041494548	0.966901639	0.982787985
ENSG00000112290	266.1073432	-0.056613675	0.126975476	-0.445863065	0.655696163	0.779180728
ENSG00000112293	110.0167872	0.345365238	0.171427808	2.014639531	0.043942432	0.110323386
ENSG00000112294	220.5808182	0.159888511	0.126246318	1.266480589	0.205341075	0.358759462
ENSG00000112304	395.8294533	0.079404374	0.100345658	0.791308518	0.428763979	0.595731712
ENSG00000112305	178.3569769	0.376878467	0.135428571	2.782857886	0.00538824	0.019045951
ENSG00000112306	3136.634122	0.022338436	0.06184231	0.361216068	0.717937931	0.824196969
ENSG00000112308	203.7694204	0.218200513	0.129873947	1.680094569	0.092938917	0.198255577
ENSG00000112312	182.5659516	-0.259326255	0.139386527	-1.860482929	0.062817232	0.145970827
ENSG00000112319	309.8332361	-1.580331625	0.115657908	-13.66384416	1.67E-42	1.41E-40
ENSG00000112320	327.7583052	0.150678084	0.112729803	1.336630429	0.181343301	0.327968244
ENSG00000112335	479.4613666	0.100331179	0.093388948	1.074336748	0.282671781	0.452173323
ENSG00000112339	321.8883944	-0.070455916	0.109561213	-0.643073528	0.520176391	0.674660147
ENSG00000112343	33.24079736	1.255644285	0.310826133	4.039699857	5.35E-05	0.000326298
ENSG00000112357	35.83286372	0.520166407	0.29381797	1.770369616	0.076665588	0.170587916
ENSG00000112365	363.7782929	0.027511969	0.108950911	0.252517106	0.800641394	0.880981486
ENSG00000112367	83.94625623	-0.066672678	0.193465591	-0.344622928	0.730377867	0.83331836
ENSG00000112378	56.12933588	0.177102975	0.23612694	0.750032904	0.453234888	0.617396281
ENSG00000112379	47.36494064	1.309036784	0.266818778	4.90608942	9.29E-07	8.28E-06
ENSG00000112394	87.96774692	0.010088304	0.187236959	0.053879875	0.957030871	0.976921201
ENSG00000112406	104.0264943	-0.50971485	0.179167953	-2.844899672	0.004442541	0.016200514

ENSG00000112414	179.5196438	-1.22700828	0.144842709	-8.471315449	2.43E-17	6.84E-16
ENSG00000112419	393.1093791	-0.420568079	0.100843279	-4.170511748	3.04E-05	0.000196191
ENSG00000112425	49.74005084	-0.813025806	0.253700907	-3.204662594	0.001352209	0.005840861
ENSG00000112473	726.7431134	0.024052925	0.082405693	0.291884267	0.770375113	0.862680103
ENSG00000112511	151.6407118	0.087460441	0.146463328	0.59714908	0.550407852	0.698263542
ENSG00000112514	400.710101	0.040159683	0.100451336	0.39979242	0.689309414	0.804480669
ENSG00000112531	967.5800983	-0.012655789	0.077830885	-0.162606261	0.870828457	0.928112848
ENSG00000112541	269.9178323	0.047272991	0.124061197	0.381045741	0.703169308	0.814114448
ENSG00000112559	23.64431915	-0.587191619	0.365517795	-1.606465205	0.108171734	0.223027792
ENSG00000112561	38.82960918	-0.616288268	0.286646107	-2.149996998	0.031555452	0.083855361
ENSG00000112576	321.3386423	0.222150896	0.113649962	1.954693971	0.050619202	0.123039961
ENSG00000112578	205.77495	0.115802655	0.135043736	0.857519637	0.391157767	0.559473898
ENSG00000112584	283.4485048	-0.282829634	0.113544525	-2.490913884	0.0127415	0.03968511
ENSG00000112592	178.311393	0.005825196	0.138714414	0.041994167	0.966503348	0.98273197
ENSG00000112624	239.0284199	0.205222483	0.125213523	1.638980181	0.101217387	0.21170349
ENSG00000112640	412.6664258	-0.049733131	0.097170906	-0.511810919	0.608783348	0.745198811
ENSG00000112651	26.53381619	-0.276425757	0.344837862	-0.801610809	0.422778123	0.590041162
ENSG00000112655	918.4519025	0.048819589	0.077688792	0.628399387	0.529742339	0.682186526
ENSG00000112658	699.2991548	0.001481669	0.083059893	0.017838559	0.985767644	0.992622086
ENSG00000112659	9.426301703	-0.569985304	0.563200563	-1.012046759	0.311515697	0.482986865
ENSG00000112667	642.4029331	0.276289926	0.092020102	3.002495334	0.002677761	0.010480498
ENSG00000112679	186.6207768	0.064395812	0.133859081	0.481071671	0.630465561	0.760706901
ENSG00000112685	260.5826007	-0.107675376	0.120891349	-0.890678919	0.373101447	0.54340148
ENSG00000112695	1025.7854	0.117778253	0.077823046	1.513410986	0.130175296	0.256930221
ENSG00000112697	286.2013492	-0.217306128	0.115996548	-1.873384429	0.061015319	0.142775642
ENSG00000112699	139.5268064	0.071224574	0.151742183	0.469378865	0.638798853	0.766678698
ENSG00000112701	834.4820569	-0.066869031	0.077190273	-0.866288298	0.386332082	0.555102336
ENSG00000112715	834.3089617	0.265026003	0.080783591	3.280691043	0.001035531	0.00463547
ENSG00000112739	1531.362062	0.075609765	0.070098255	1.078625499	0.280754709	0.449518669
ENSG00000112742	245.1961976	-0.387690437	0.122286789	-3.170337865	0.001522618	0.006469514
ENSG00000112759	540.0933313	-0.246025817	0.095448644	-2.577572673	0.009949696	0.032159083
ENSG00000112763	191.3756803	0.151242977	0.136724833	1.106185125	0.2686464	0.435759351
ENSG00000112773	1994.727078	1.734487981	0.064379703	26.94153436	7.17E-160	3.87E-157
ENSG00000112787	1180.522942	0.616344053	0.090710166	6.79465245	1.09E-11	1.91E-10
ENSG00000112812	12.52515806	-2.131640398	0.567550836	-3.755858088	0.000172749	0.000937826
ENSG00000112851	826.2086786	-0.224771501	0.077419528	-2.903292062	0.003692621	0.01379161
ENSG00000112852	68.04431226	-0.382910519	0.214852199	-1.782204329	0.074715917	0.16693725
ENSG00000112855	83.35559605	0.013415469	0.192215048	0.069794063	0.944357572	0.969125571
ENSG00000112874	52.85032761	-0.191049713	0.242083752	-0.789188499	0.430001843	0.596802281
ENSG00000112877	168.4852738	-0.383806305	0.142199417	-2.699070884	0.006953336	0.02365549
ENSG00000112893	239.4102349	-0.904107705	0.125154121	-7.223954731	5.05E-13	1.03E-11
ENSG00000112941	477.8599329	0.086824994	0.091450489	0.949420776	0.342406648	0.514366687
ENSG00000112964	11.50602412	0.255640658	0.504495683	0.506725165	0.612347671	0.747774297
ENSG00000112972	981.6883191	0.143762148	0.090846451	1.582474015	0.1135414	0.231516337
ENSG00000112977	244.6507381	-0.314702179	0.121214562	-2.596240703	0.009425001	0.030710507
ENSG00000112983	418.0639972	-0.018120406	0.098189917	-0.184544465	0.853586348	0.916061383
ENSG00000112984	601.139238	0.254021406	0.0894698	2.839186014	0.004522878	0.016438
ENSG00000112992	386.8433462	-0.473405278	0.104178896	-4.544157182	5.52E-06	4.19E-05
ENSG00000112996	722.8296394	-0.812683618	0.08170473	-9.946592098	2.61E-23	1.05E-21
ENSG00000113013	4139.887813	0.174972951	0.060965283	2.870042464	0.004104167	0.015143481

ENSG00000113048	884.0032005	0.134636733	0.078613643	1.71263827	0.086779108	0.187999095
ENSG00000113068	205.2237788	-0.10896574	0.129947121	-0.83853909	0.401728003	0.570064983
ENSG00000113070	11.99184878	0.697836144	0.520830892	1.339851676	0.180293572	0.326532207
ENSG00000113083	20.8289775	-0.315494619	0.392076066	-0.804677066	0.421006057	0.588503542
ENSG00000113108	27.93386721	0.408564728	0.332073529	1.230344161	0.218568254	0.375609036
ENSG00000113119	96.71945834	0.048237203	0.189508706	0.254538192	0.799079802	0.880504705
ENSG00000113140	503.3691113	-1.38213834	0.100163678	-13.79879775	2.59E-43	2.23E-41
ENSG00000113141	419.741784	0.115559083	0.100729193	1.147225339	0.251288502	0.415585498
ENSG00000113161	1261.517184	-0.101211401	0.07565096	-1.33787331	0.180937739	0.327507088
ENSG00000113163	191.7623113	-0.568730287	0.138668926	-4.101353516	4.11E-05	0.00025746
ENSG00000113194	840.1189379	-0.111686676	0.075649996	-1.476360639	0.139847103	0.270694814
ENSG00000113240	80.39233141	-0.023821812	0.199934831	-0.119147881	0.905158197	0.949216662
ENSG00000113269	187.4458123	0.316770063	0.140246541	2.258665778	0.023904183	0.066755876
ENSG00000113272	168.7525345	0.143429454	0.142130438	1.009139606	0.312907686	0.484076461
ENSG00000113273	305.0383235	0.136666213	0.113188618	1.207420101	0.227270398	0.385773426
ENSG00000113282	559.1614931	0.03233751	0.089806687	0.360079085	0.718787993	0.824903181
ENSG00000113300	505.8820836	-0.06911989	0.092524791	-0.747041843	0.455038303	0.618832709
ENSG00000113312	240.2288397	0.325066035	0.123250682	2.637438018	0.008353489	0.027779702
ENSG00000113318	199.6127845	-0.403935464	0.144819581	-2.789232381	0.005283314	0.018752768
ENSG00000113328	322.1587313	-1.047197655	0.112518259	-9.306913092	1.32E-20	4.60E-19
ENSG00000113356	171.199751	-0.231964976	0.141710358	-1.636895	0.101652411	0.212455202
ENSG00000113360	1226.770049	0.115517303	0.073637309	1.568733358	0.116710082	0.236330766
ENSG00000113361	16.72290379	1.361508125	0.435461933	3.126583569	0.001768502	0.007358457
ENSG00000113368	1115.505927	-0.408846057	0.077179342	-5.297350928	1.17E-07	1.22E-06
ENSG00000113369	159.6554415	0.192980776	0.148292452	1.301352652	0.193137773	0.343853776
ENSG00000113384	413.8612722	0.130778358	0.095651468	1.367238385	0.171550598	0.314673706
ENSG00000113387	921.1347905	-0.06109701	0.077338564	-0.789994103	0.429531212	0.596325849
ENSG00000113391	185.5133105	-0.031191458	0.139531288	-0.223544546	0.823111718	0.896367959
ENSG00000113407	1283.118122	-1.075106506	0.0788032	-13.64292948	2.22E-42	1.87E-40
ENSG00000113441	488.512272	-0.158079544	0.09418362	-1.678418646	0.0932654	0.198740758
ENSG00000113448	1806.199087	-0.964408724	0.068274725	-14.12541357	2.65E-45	2.42E-43
ENSG00000113456	131.0571201	-0.254677883	0.165196197	-1.541669165	0.123154009	0.24688802
ENSG00000113460	426.9981558	0.223714142	0.104537194	2.140043493	0.032351252	0.085597671
ENSG00000113504	237.6044699	0.53656652	0.121159768	4.428586554	9.49E-06	6.83E-05
ENSG00000113522	114.9625584	0.062438534	0.165431693	0.377427882	0.70585565	0.81545804
ENSG00000113552	224.4476092	0.402758754	0.126606234	3.18119212	0.001466703	0.006273685
ENSG00000113558	522.773887	0.339454578	0.095609032	3.55044467	0.000384581	0.001918398
ENSG00000113569	1235.699996	-0.193647493	0.070403811	-2.750525706	0.005949972	0.02070331
ENSG00000113575	222.9672565	0.059824437	0.143391199	0.417211358	0.676523818	0.794509662
ENSG00000113583	340.9759709	-0.389014054	0.110093547	-3.533486416	0.000410117	0.002026351
ENSG00000113593	248.2494679	-0.111780665	0.118883249	-0.9402558	0.347086366	0.518998461
ENSG00000113595	60.00828388	-0.117045938	0.238546463	-0.490663064	0.623664774	0.755746988
ENSG00000113597	89.23578146	-0.283208082	0.188375937	-1.503419632	0.132730867	0.260911068
ENSG00000113615	418.9791397	0.150503494	0.101723415	1.479536392	0.138997013	0.269606605
ENSG00000113621	182.5974208	0.093846032	0.138294731	0.678594414	0.497394883	0.655493567
ENSG00000113638	86.5611585	-0.208215959	0.188905805	-1.102221073	0.270365573	0.437789648
ENSG00000113643	692.273438	0.324205686	0.080691011	4.017866182	5.87E-05	0.000354968
ENSG00000113645	293.2471018	0.330561417	0.111083977	2.975779455	0.002922449	0.011287228
ENSG00000113648	1430.621774	-0.10625421	0.077990542	-1.362398651	0.173072109	0.316761423
ENSG00000113649	1591.340624	0.08685059	0.065424542	1.327492514	0.184345815	0.331817742

ENSG00000113658	509.9662221	-0.033946385	0.089868326	-0.3777347	0.705627687	0.815261731
ENSG00000113712	895.6150235	-0.119040242	0.074365241	-1.600751097	0.109432059	0.225130658
ENSG00000113716	445.0380414	0.188928925	0.095089993	1.986843395	0.046939754	0.116091213
ENSG00000113719	648.4571825	-0.084803269	0.084419484	-1.004546164	0.315115432	0.486580902
ENSG00000113721	46.95845638	0.2161256	0.258437008	0.836279609	0.402997622	0.571289494
ENSG00000113732	186.5289645	0.289175178	0.159901816	1.808454614	0.070535775	0.15996384
ENSG00000113734	74.23642905	0.343027356	0.20372948	1.683739415	0.09223204	0.197035205
ENSG00000113739	527.7735874	-0.22562109	0.103150338	-2.187303435	0.02872038	0.077763142
ENSG00000113742	122.7614461	0.256253934	0.164397152	1.558749228	0.119055745	0.240318445
ENSG00000113758	1428.435975	0.089855528	0.076527509	1.174159842	0.240331008	0.4027114
ENSG00000113761	139.0380896	-0.048855396	0.151601011	-0.322262999	0.747253461	0.846027998
ENSG00000113810	2551.961984	0.191720548	0.058212876	3.293438891	0.000989699	0.004451583
ENSG00000113811	153.6688253	0.230195007	0.145400394	1.583180082	0.113380425	0.231322415
ENSG00000113812	272.1008683	-0.122661968	0.117150321	-1.047047642	0.295077606	0.465516074
ENSG00000113838	34.65297119	0.194438342	0.295344442	0.65834434	0.510316892	0.666767871
ENSG00000113845	339.3502219	0.099784332	0.110869904	0.900012793	0.368113443	0.539420982
ENSG00000113851	46.3393871	-0.196925155	0.26283828	-0.749225549	0.453721273	0.617606294
ENSG00000113916	119.6604492	0.235650096	0.175377463	1.343673769	0.179053903	0.324908693
ENSG00000113966	38.95570352	-0.286255487	0.278716637	-1.02704844	0.304397655	0.475361185
ENSG00000113971	39.24258673	0.592970876	0.281483952	2.106588574	0.035153258	0.091753856
ENSG00000114019	185.5183969	-0.713745547	0.138948883	-5.136749085	2.80E-07	2.74E-06
ENSG00000114021	245.0144139	-0.047259096	0.13016089	-0.363082154	0.716543515	0.823538435
ENSG00000114023	482.0387125	0.903481363	0.095885024	9.422549231	4.40E-21	1.59E-19
ENSG00000114026	379.4264918	0.332580595	0.103715818	3.206652576	0.001342891	0.005802395
ENSG00000114030	488.1317453	-0.093634016	0.090142163	-1.038737175	0.298926987	0.469374847
ENSG00000114054	707.4284269	0.175488919	0.082519617	2.126632751	0.033450609	0.088091621
ENSG00000114062	1091.991573	-0.034376046	0.072066664	-0.477003436	0.633359675	0.7628883
ENSG00000114098	163.5262026	-0.153482557	0.148615146	-1.032751783	0.301720073	0.47217935
ENSG00000114107	157.3742965	-0.375845613	0.146477209	-2.56589824	0.010290901	0.033101967
ENSG00000114115	16.22440791	-3.186903706	0.595561599	-5.351089986	8.74E-08	9.30E-07
ENSG00000114120	590.3819463	-0.00163431	0.090400767	-0.018078496	0.985576233	0.992571648
ENSG00000114125	275.0248907	0.065219499	0.133439515	0.488757016	0.625013733	0.75653321
ENSG00000114126	509.0686045	0.115020775	0.09337248	1.231848768	0.218005582	0.375025739
ENSG00000114127	397.7880391	-0.000830572	0.099952256	-0.008309687	0.993369905	0.99670479
ENSG00000114166	94.05457699	1.03883923	0.187595219	5.537663685	3.07E-08	3.51E-07
ENSG00000114200	13.58193568	-6.050042674	1.238845524	-4.883613458	1.04E-06	9.19E-06
ENSG00000114209	218.5628854	-0.082254437	0.129133388	-0.636972656	0.524142655	0.677522028
ENSG00000114251	131.1842083	-10.31533138	1.185807459	-8.698993501	3.35E-18	1.02E-16
ENSG00000114268	350.4245711	0.896152622	0.113683882	7.882846744	3.20E-15	7.84E-14
ENSG00000114270	105.2524981	-0.176337662	0.181327734	-0.972480368	0.330811627	0.503184724
ENSG00000114302	1232.339367	0.113780317	0.074267428	1.53203525	0.125513737	0.250190359
ENSG00000114315	1702.727038	-0.990777838	0.065015007	-15.23921747	1.94E-52	2.24E-50
ENSG00000114316	372.0995515	0.124276314	0.107026135	1.161177264	0.245569819	0.40905007
ENSG00000114331	485.4375956	0.029779219	0.099435433	0.299482967	0.764571566	0.858233722
ENSG00000114346	571.1501285	-0.281176606	0.091788516	-3.063309205	0.002189037	0.008818213
ENSG00000114353	1458.021056	0.137439037	0.06595784	2.083740703	0.03718376	0.096014756
ENSG00000114354	1310.345439	0.085988364	0.079745756	1.078281385	0.280908202	0.449677209
ENSG00000114378	37.48328041	-1.950214902	0.329633649	-5.916310141	3.29E-09	4.29E-08
ENSG00000114383	104.0356823	0.085744278	0.17509724	0.4896952	0.624349599	0.756055071
ENSG00000114388	141.9914434	0.299543524	0.15429657	1.941349213	0.052215934	0.126200486

ENSG00000114391	1610.173249	0.455744279	0.067699096	6.731910876	1.67E-11	2.89E-10
ENSG00000114395	42.06886403	0.515493574	0.287728332	1.791598243	0.073197345	0.164380992
ENSG00000114416	622.7334861	-0.102204723	0.084526868	-1.209138887	0.226609492	0.385000427
ENSG00000114423	250.4091499	-2.107812531	0.13631515	-15.46278995	6.19E-54	7.18E-52
ENSG00000114439	1075.377801	0.469827175	0.074916449	6.271348727	3.58E-10	5.29E-09
ENSG00000114446	145.2296372	-0.300963291	0.154355612	-1.949804661	0.051199406	0.124065662
ENSG00000114450	860.4311632	-0.053956703	0.08181775	-0.659474298	0.509591246	0.665943644
ENSG00000114473	39.86706537	-0.038380243	0.277160569	-0.138476563	0.889863791	0.939631412
ENSG00000114480	196.1138782	0.093911723	0.130311186	0.720672765	0.471110873	0.633103807
ENSG00000114491	220.0908418	-0.136006294	0.137656459	-0.988012437	0.323146557	0.494901842
ENSG00000114503	772.1171444	0.253734771	0.079354442	3.197486664	0.001386308	0.005973458
ENSG00000114520	157.5012213	0.034793807	0.148037574	0.235033623	0.814182637	0.890857104
ENSG00000114529	14.35135827	-0.769977283	0.47891198	-1.607763672	0.107886948	0.222603989
ENSG00000114544	108.4374339	0.706692148	0.178007745	3.970007858	7.19E-05	0.000426876
ENSG00000114554	625.2602026	0.084008787	0.084974595	0.988634151	0.322842171	0.494547
ENSG00000114573	482.6532918	-0.263291505	0.091925854	-2.864172524	0.004181002	0.015394636
ENSG00000114626	19.66644016	0.628540017	0.390154421	1.611003191	0.107179027	0.22150122
ENSG00000114631	139.8438376	0.939190201	0.162727102	5.771565965	7.85E-09	9.80E-08
ENSG00000114646	59.97488416	0.826849468	0.240321385	3.440598789	0.000580429	0.002766637
ENSG00000114648	326.2626828	0.188482045	0.106708098	1.766333102	0.077339981	0.17181634
ENSG00000114650	757.9260376	0.235244499	0.092418535	2.54542554	0.010914464	0.03474178
ENSG00000114654	11.1915581	0.412381754	0.555507016	0.742352017	0.457874083	0.621666078
ENSG00000114656	11.63505118	-0.747139891	0.51675243	-1.445837209	0.148222867	0.28246607
ENSG00000114670	16.39190468	-1.183734707	0.462034819	-2.562003245	0.010407035	0.033384984
ENSG00000114686	551.5004587	0.220837688	0.08802133	2.508911053	0.012110397	0.037946632
ENSG00000114735	197.6814499	0.441508209	0.139101357	3.174003617	0.001503518	0.006401916
ENSG00000114737	29.90422937	-0.808170512	0.329584878	-2.452086141	0.014203066	0.043419034
ENSG00000114738	738.8638089	0.151867913	0.079386438	1.913020877	0.05574538	0.132856354
ENSG00000114739	410.6800622	-0.635022864	0.101512346	-6.255621995	3.96E-10	5.82E-09
ENSG00000114742	295.1261057	-0.262580701	0.109905403	-2.38915189	0.016887319	0.050268313
ENSG00000114744	81.7093847	-0.725444402	0.219745429	-3.301294623	0.000962398	0.004339904
ENSG00000114745	187.9656175	0.114153943	0.135143256	0.844688417	0.398284828	0.566485979
ENSG00000114767	219.5073977	0.236030676	0.134372544	1.75653946	0.07899634	0.174719466
ENSG00000114770	152.1938148	-0.032827612	0.162408189	-0.202130278	0.839814878	0.906824475
ENSG00000114779	709.5715526	0.369685752	0.086857216	4.256246868	2.08E-05	0.000139256
ENSG00000114784	318.1014161	-0.136307066	0.108049064	-1.261529354	0.207118191	0.361055998
ENSG00000114790	88.74477855	0.537638831	0.192174334	2.797662001	0.005147395	0.018342327
ENSG00000114796	111.1474199	0.044094344	0.167693136	0.262946624	0.792591719	0.876311364
ENSG00000114805	68.23374844	-1.810330612	0.239246403	-7.566803892	3.83E-14	8.74E-13
ENSG00000114841	203.4214339	0.065094908	0.14653664	0.444222742	0.656881552	0.779984375
ENSG00000114850	497.5188121	0.076429137	0.090176665	0.847548946	0.396689217	0.564511542
ENSG00000114853	267.3212331	0.480273538	0.139395797	3.445394693	0.000570226	0.002721701
ENSG00000114854	191.2124219	0.76421681	0.139328256	5.48500951	4.13E-08	4.64E-07
ENSG00000114857	1723.650446	0.023551273	0.071589481	0.328976722	0.742173283	0.842381069
ENSG00000114859	97.95055945	0.152812124	0.184036932	0.830334009	0.406349965	0.574534268
ENSG00000114867	4364.957189	0.148460923	0.063893406	2.323571922	0.020148449	0.0581388
ENSG00000114902	717.590679	0.55625196	0.081072819	6.861139965	6.83E-12	1.22E-10
ENSG00000114904	380.5410744	-0.29226668	0.106345879	-2.748265222	0.005991152	0.020810514
ENSG00000114923	30.29228542	0.079417292	0.32021626	0.248011429	0.804125564	0.88388651
ENSG00000114933	674.4221413	-0.209429709	0.087934234	-2.381662967	0.017234663	0.051172123

ENSG00000114942	1798.471007	0.1503224	0.086869073	1.730447839	0.083550293	0.182780091
ENSG00000114956	211.0299019	0.118445827	0.132767612	0.892128923	0.372323834	0.54287242
ENSG00000114978	1097.990552	-0.188961091	0.084027141	-2.248810191	0.024524575	0.068203663
ENSG00000114982	443.227295	-0.25555058	0.096045998	-2.660710338	0.007797601	0.026160233
ENSG00000114988	71.87456958	-0.477157432	0.210322915	-2.268689705	0.023287202	0.065344652
ENSG00000114993	593.2091373	0.004017184	0.088987886	0.045143046	0.96399329	0.981105184
ENSG00000114999	776.4343709	-0.055460999	0.088716686	-0.625147331	0.531874366	0.683490917
ENSG00000115020	583.3636521	-0.338099874	0.090432863	-3.738683719	0.000184986	0.000998656
ENSG00000115041	194.7459472	0.642598753	0.137223121	4.682875219	2.83E-06	2.28E-05
ENSG00000115042	183.7599959	0.534884453	0.138695565	3.856536084	0.000115005	0.000654039
ENSG00000115053	18313.82462	0.069475537	0.050673421	1.371044947	0.170360945	0.313317044
ENSG00000115073	173.9483697	0.08856042	0.153054094	0.578621697	0.562844461	0.70902994
ENSG00000115084	303.8144377	-0.275137223	0.126459055	-2.175702039	0.029577548	0.079577823
ENSG00000115091	830.7553826	0.040984145	0.080172488	0.511199615	0.609211288	0.745434753
ENSG00000115107	117.7602887	0.121649455	0.169377397	0.718215403	0.472624482	0.633981332
ENSG00000115109	175.4203219	-0.689593972	0.147253801	-4.683030018	2.83E-06	2.28E-05
ENSG00000115112	18.18874133	-1.429167136	0.440496535	-3.244445809	0.001176794	0.005170606
ENSG00000115128	940.2729155	0.17339564	0.085813175	2.020617935	0.043319331	0.10908362
ENSG00000115129	160.7640769	-0.958547443	0.158666346	-6.041277594	1.53E-09	2.09E-08
ENSG00000115137	67.02022711	0.008083066	0.215130989	0.037572764	0.970028323	0.984394442
ENSG00000115145	151.6206188	-0.006806884	0.15512475	-0.043880062	0.965000008	0.981631915
ENSG00000115159	374.2436708	-0.376450569	0.105109236	-3.581517511	0.000341604	0.001726084
ENSG00000115163	195.3513432	-0.316112376	0.146464843	-2.158281603	0.030905944	0.082503952
ENSG00000115170	111.0106393	-0.344256929	0.168748708	-2.040056687	0.04134468	0.104926598
ENSG00000115183	507.0423355	-0.416346001	0.091982125	-4.526379462	6.00E-06	4.53E-05
ENSG00000115204	528.1048682	0.358430488	0.09018211	3.974518747	7.05E-05	0.00042011
ENSG00000115207	671.6228654	0.088133059	0.082220696	1.071908456	0.283761152	0.453469044
ENSG00000115211	535.2950199	0.128711231	0.098119158	1.311784907	0.189592719	0.338830651
ENSG00000115216	853.1821082	0.018684112	0.08134558	0.22968811	0.818334135	0.892848047
ENSG00000115226	40.1999799	0.971835011	0.292659405	3.320703161	0.00089791	0.004083179
ENSG00000115232	13.30924724	-7.013372925	1.241180934	-5.650564501	1.60E-08	1.91E-07
ENSG00000115233	353.6512384	0.499282274	0.107096696	4.661976431	3.13E-06	2.50E-05
ENSG00000115234	501.5052838	0.340769469	0.092291961	3.692298486	0.000222236	0.001174917
ENSG00000115239	163.2946114	-0.326958883	0.147650677	-2.214408286	0.026800705	0.073529201
ENSG00000115241	2699.754875	0.140266587	0.059349395	2.36340382	0.018107926	0.053298107
ENSG00000115252	14.04593045	-5.26903675	1.06879441	-4.929888015	8.23E-07	7.44E-06
ENSG00000115255	141.3676055	-1.088782761	0.158009031	-6.890636268	5.55E-12	1.01E-10
ENSG00000115257	19.37271353	-0.479296433	0.412662054	-1.16147445	0.245449008	0.408994331
ENSG00000115263	40.55303252	1.956167023	0.308716603	6.336449045	2.35E-10	3.55E-09
ENSG00000115266	197.630569	-0.119290961	0.152464855	-0.782416122	0.433970062	0.600174999
ENSG00000115267	20.54393287	2.535304966	0.470816059	5.384916076	7.25E-08	7.77E-07
ENSG00000115268	6145.167496	0.413276038	0.064866571	6.37117139	1.88E-10	2.86E-09
ENSG00000115275	328.4168366	0.472874486	0.11195787	4.223682422	2.40E-05	0.0001588
ENSG00000115282	209.5474242	-0.126002724	0.130787266	-0.963417368	0.335338114	0.507124904
ENSG00000115286	562.6496985	0.300147352	0.089991498	3.33528564	0.000852118	0.003888793
ENSG00000115289	141.6821583	0.333328272	0.162593189	2.050075243	0.040357089	0.102549933
ENSG00000115295	317.6562757	0.467462094	0.108928855	4.291444116	1.78E-05	0.000120461
ENSG00000115306	6510.619251	0.098848225	0.051701625	1.911897835	0.055889299	0.133063896
ENSG00000115307	801.5342466	0.309728735	0.076098172	4.070120573	4.70E-05	0.000291028
ENSG00000115310	1016.144951	0.047616798	0.078751088	0.604649407	0.545411971	0.694030433

ENSG00000115317	99.34933561	0.571741569	0.181759025	3.145602085	0.001657453	0.006968646
ENSG00000115318	63.25228199	-1.015042634	0.234094939	-4.336029811	1.45E-05	0.00010044
ENSG00000115325	36.35836166	0.041102601	0.300671413	0.136702724	0.891265776	0.94027635
ENSG00000115350	81.19873835	0.33795548	0.205728257	1.64272757	0.100439312	0.21040767
ENSG00000115355	1231.58494	-0.064004355	0.068889084	-0.929092848	0.352840971	0.524204877
ENSG00000115364	534.7262956	0.133035484	0.09052281	1.469634936	0.141660653	0.273300863
ENSG00000115365	396.6785963	-0.28070309	0.102093829	-2.749461872	0.005969321	0.020750074
ENSG00000115368	714.9461683	-0.482218477	0.086411199	-5.58050902	2.40E-08	2.79E-07
ENSG00000115392	937.3476451	-0.412456521	0.092999898	-4.435021223	9.21E-06	6.64E-05
ENSG00000115415	628.6940798	0.747632144	0.086142466	8.679019535	3.99E-18	1.21E-16
ENSG00000115419	342.638021	-0.042047732	0.109824632	-0.382862487	0.701821727	0.813272546
ENSG00000115421	156.8231292	-0.324344504	0.143773265	-2.255944481	0.024074108	0.067163654
ENSG00000115423	18.43954287	-2.263425646	0.477910352	-4.736088343	2.18E-06	1.80E-05
ENSG00000115425	88.26642219	-0.649778043	0.189484268	-3.429192574	0.00060538	0.002873866
ENSG00000115446	65.49707361	0.185668703	0.217458904	0.853810533	0.393209977	0.561290936
ENSG00000115457	1332.847403	-0.920653538	0.172591725	-5.334285523	9.59E-08	1.01E-06
ENSG00000115459	53.28663564	0.130051281	0.24017857	0.541477457	0.588178532	0.72968478
ENSG00000115461	94.17917206	-5.399902116	0.442727074	-12.19690965	3.23E-34	2.02E-32
ENSG00000115464	1215.085286	-0.090061958	0.074792233	-1.204161903	0.228527008	0.387368031
ENSG00000115468	154.4001695	-1.704347924	0.163037351	-10.45372677	1.41E-25	6.49E-24
ENSG00000115484	2198.794301	0.366920043	0.065216656	5.626170782	1.84E-08	2.18E-07
ENSG00000115486	255.9646494	-0.141857344	0.116537752	-1.217265148	0.22350335	0.38189412
ENSG00000115504	308.47679	-0.08840047	0.114988917	-0.768773827	0.442027589	0.607074847
ENSG00000115507	57.20818535	0.634227054	0.232173849	2.731690305	0.006301034	0.021736401
ENSG00000115514	84.39049887	-0.07623758	0.202395326	-0.376676584	0.706413967	0.815678152
ENSG00000115520	219.9611815	0.069473312	0.127122468	0.546506951	0.584717511	0.726667865
ENSG00000115524	2743.541097	0.044516634	0.057091249	0.779745325	0.435540794	0.601695667
ENSG00000115525	100.1259949	0.445208403	0.181369545	2.454703199	0.014100095	0.043141807
ENSG00000115526	267.4756038	-0.014666338	0.117354454	-0.124974702	0.900543578	0.94621405
ENSG00000115539	131.1357009	-0.069781507	0.159017711	-0.438828523	0.660785791	0.783076162
ENSG00000115540	78.84362533	-0.070200257	0.21373172	-0.328450344	0.742571183	0.842628436
ENSG00000115541	390.2115893	0.644861413	0.11143728	5.786765561	7.18E-09	9.00E-08
ENSG00000115548	300.4293555	0.125018384	0.11109037	1.125375525	0.260429938	0.426569419
ENSG00000115556	35.84298052	0.294246902	0.288614272	1.019516117	0.307958006	0.479110212
ENSG00000115561	208.5065165	-0.228136822	0.127836095	-1.784604117	0.074325553	0.166292761
ENSG00000115568	863.537458	0.235185727	0.075736874	3.105300176	0.00190086	0.007834912
ENSG00000115594	18.91528996	-0.693278892	0.419856619	-1.651227729	0.098692083	0.207534086
ENSG00000115616	33.07147897	2.256758981	0.345954564	6.523281421	6.88E-11	1.12E-09
ENSG00000115641	79.64638271	-0.158908062	0.203882022	-0.779411838	0.435737153	0.601848553
ENSG00000115649	137.5749751	0.189471794	0.153694749	1.232779881	0.2176579	0.374640426
ENSG00000115652	498.3773148	0.146510828	0.092126207	1.590327372	0.111761032	0.228749412
ENSG00000115657	57.68587991	0.190628539	0.231673019	0.822834439	0.410602149	0.57903106
ENSG00000115661	98.93217256	-0.023355315	0.190030175	-0.12290319	0.902183759	0.947258155
ENSG00000115677	5625.467074	0.303466895	0.051999815	5.835922567	5.35E-09	6.81E-08
ENSG00000115685	301.6442046	0.403449074	0.111793871	3.608865774	0.000307539	0.001573196
ENSG00000115687	223.0716081	-0.215481113	0.124533723	-1.730303313	0.083576098	0.182808074
ENSG00000115694	546.0852582	0.083372117	0.088114678	0.946177406	0.344058108	0.515848463
ENSG00000115738	218.3277805	1.789572333	0.139489778	12.82941558	1.12E-37	8.04E-36
ENSG00000115750	91.52608655	-0.118205923	0.207172685	-0.570567121	0.568293111	0.713722708
ENSG00000115756	80.35731709	0.346688854	0.204616244	1.694336911	0.09020128	0.193680432

ENSG00000115758	811.4139323	0.305251804	0.091230031	3.345957454	0.00081999	0.003762952
ENSG00000115760	2571.579016	-0.45130655	0.063046544	-7.158307568	8.17E-13	1.64E-11
ENSG00000115761	452.7049493	0.115568991	0.094763487	1.219551905	0.222634788	0.380873325
ENSG00000115762	299.8024899	0.270443386	0.11639472	2.323502187	0.02015219	0.0581388
ENSG00000115806	782.1026333	0.03760541	0.080903931	0.464815601	0.642063514	0.768923285
ENSG00000115808	795.0694171	0.093926608	0.08141111	1.153732056	0.248610037	0.412749377
ENSG00000115816	494.5215192	-0.58145582	0.093429263	-6.223487158	4.86E-10	7.05E-09
ENSG00000115825	607.8808922	-0.763635599	0.087369632	-8.74028635	2.33E-18	7.19E-17
ENSG00000115827	194.1886927	-0.042319289	0.131791415	-0.321108086	0.748128483	0.846813924
ENSG00000115828	67.66378275	-6.228380664	0.6691834	-9.307434501	1.31E-20	4.59E-19
ENSG00000115839	506.5163624	-0.067784947	0.090469193	-0.749259989	0.453700518	0.617606294
ENSG00000115840	357.9519825	0.409419907	0.102805017	3.982489569	6.82E-05	0.000407992
ENSG00000115841	71.71027315	-0.979626989	0.218785877	-4.477560452	7.55E-06	5.56E-05
ENSG00000115844	2564.148982	-0.388398544	0.150385977	-2.58267794	0.009803679	0.031716325
ENSG00000115866	459.485637	0.237967661	0.099553609	2.390346916	0.016832465	0.050157301
ENSG00000115875	987.5755796	0.331444482	0.073329136	4.519956183	6.19E-06	4.65E-05
ENSG00000115884	624.8345102	0.284944551	0.096263824	2.960037732	0.003076014	0.011786851
ENSG00000115902	196.0203247	-0.367580258	0.155583096	-2.362597658	0.018147359	0.053385541
ENSG00000115904	1475.968318	-0.592610195	0.069263503	-8.555879597	1.17E-17	3.37E-16
ENSG00000115935	141.2177695	-0.181170232	0.154981376	-1.168980668	0.24241142	0.405134232
ENSG00000115942	353.939287	-0.046179511	0.113418318	-0.407160955	0.683889777	0.800149899
ENSG00000115944	683.9362531	0.092071036	0.101698683	0.905331646	0.365289716	0.536514496
ENSG00000115946	226.224915	-0.146344319	0.1406203	-1.040705501	0.29801225	0.468671974
ENSG00000115947	258.0283164	-0.262437521	0.118934757	-2.206567085	0.027344319	0.074714189
ENSG00000115963	55.92020283	-4.432792696	0.435676614	-10.17450226	2.58E-24	1.09E-22
ENSG00000115966	405.801881	-0.122046104	0.105447896	-1.157406725	0.247106228	0.4112472
ENSG00000115970	479.4538263	-0.083461248	0.097674128	-0.854486755	0.392835345	0.561073523
ENSG00000115977	613.0640801	-0.234321401	0.087826999	-2.667988263	0.007630692	0.025698474
ENSG00000115993	374.334307	-0.262343041	0.105646221	-2.483222201	0.013019984	0.040400203
ENSG00000115998	90.17882141	0.201125097	0.186086568	1.080814696	0.27977955	0.448534962
ENSG00000116001	283.2769438	-0.442254011	0.118699344	-3.725833657	0.000194671	0.001044116
ENSG00000116005	745.7252623	-0.17703721	0.088345639	-2.003915651	0.045079097	0.112393694
ENSG00000116014	15.92792629	0.356662259	0.44548082	0.800623154	0.423349843	0.590574257
ENSG00000116017	552.305417	-0.599954925	0.097012062	-6.184333322	6.24E-10	8.94E-09
ENSG00000116030	1443.469175	0.183509656	0.070378479	2.607468331	0.009121451	0.029859944
ENSG00000116035	56.41516243	-0.234046694	0.231730477	-1.009995305	0.312497539	0.484076461
ENSG00000116044	281.0362134	-0.137980371	0.11471113	-1.20285077	0.229034074	0.387861666
ENSG00000116062	1975.657481	-0.088222281	0.061020841	-1.445772948	0.148240896	0.28246607
ENSG00000116095	210.8801477	-0.174295792	0.135891049	-1.282614221	0.199627265	0.351641386
ENSG00000116096	84.56961888	0.638724147	0.194000399	3.292385743	0.000993413	0.004466858
ENSG00000116120	245.8022594	0.408257763	0.121750594	3.353230155	0.000798743	0.003672647
ENSG00000116127	769.1628168	-0.447600977	0.08218527	-5.446243292	5.14E-08	5.68E-07
ENSG00000116128	1079.947394	-0.207450183	0.074830959	-2.772250773	0.005567013	0.019589137
ENSG00000116132	30.91068841	-4.518723141	0.606930077	-7.445212075	9.68E-14	2.12E-12
ENSG00000116133	2223.617619	-0.116273367	0.06657651	-1.746462326	0.080730648	0.177802354
ENSG00000116138	247.7933195	-0.366134298	0.120561627	-3.036905739	0.002390202	0.009505993
ENSG00000116151	49.12752089	-0.505644019	0.254855194	-1.984044393	0.047250891	0.116568828
ENSG00000116161	1160.148234	0.311656626	0.071584413	4.353693967	1.34E-05	9.35E-05
ENSG00000116171	234.6835537	-0.125659485	0.135704429	-0.92597925	0.354456764	0.525826821
ENSG00000116191	118.7349973	0.089996475	0.173285597	0.519353467	0.603514275	0.741285586

ENSG00000116194	730.9884256	2.762797935	0.094754657	29.15738421	6.73E-187	5.26E-184
ENSG00000116198	187.9829076	0.282134715	0.138368839	2.039004722	0.041449556	0.105154762
ENSG00000116199	1016.519002	0.574989127	0.073326262	7.841516946	4.45E-15	1.07E-13
ENSG00000116205	75.67177066	0.161848242	0.202062796	0.800979916	0.423143273	0.590374806
ENSG00000116209	194.6263697	-0.251920102	0.13306228	-1.893249552	0.058324691	0.137556569
ENSG00000116212	125.7339939	0.269922935	0.163725371	1.648632297	0.099222988	0.208463253
ENSG00000116213	69.38313816	0.312749738	0.235417075	1.328492157	0.184015573	0.331393173
ENSG00000116221	539.5057204	0.362016522	0.088407575	4.094858657	4.22E-05	0.000264077
ENSG00000116237	576.3084222	-0.065424698	0.098000882	-0.66759295	0.504393459	0.66210774
ENSG00000116251	505.828168	-0.008989971	0.092250252	-0.097451998	0.922367452	0.957117205
ENSG00000116254	35.4547931	-1.484411398	0.347096506	-4.276653245	1.90E-05	0.00012782
ENSG00000116260	515.3920416	0.271708737	0.101335121	2.681288916	0.007333917	0.024812025
ENSG00000116266	95.79564991	-0.380388293	0.185390822	-2.051818363	0.040187318	0.102173983
ENSG00000116273	204.8621316	-0.581415004	0.137085921	-4.24124519	2.22E-05	0.000147982
ENSG00000116285	90.14517892	-2.064672124	0.21310851	-9.688360759	3.38E-22	1.28E-20
ENSG00000116288	1090.8106	0.39696647	0.074578518	5.322799149	1.02E-07	1.07E-06
ENSG00000116299	70.45120136	-0.0280064	0.218150857	-0.128380887	0.897847556	0.944497571
ENSG00000116337	518.2096638	-0.137783129	0.090725522	-1.51868103	0.128842804	0.254765606
ENSG00000116350	1242.819679	-0.012743573	0.071863019	-0.177331448	0.85924805	0.919748332
ENSG00000116353	166.295995	-0.038320087	0.148262873	-0.258460436	0.796051579	0.878339214
ENSG00000116396	102.5273132	0.133565048	0.1785985	0.747850897	0.454550098	0.618431859
ENSG00000116406	430.6981997	0.245714002	0.094986603	2.586827974	0.009686394	0.031423735
ENSG00000116455	123.9206315	0.238625653	0.163001281	1.463949558	0.143207731	0.275453786
ENSG00000116459	629.8663097	-0.75786728	0.092727082	-8.17309535	3.01E-16	7.94E-15
ENSG00000116473	122.8523298	-0.174740557	0.165860646	-1.05353838	0.292094314	0.462575967
ENSG00000116478	1642.970501	0.036928142	0.079416686	0.464992234	0.641937018	0.768868459
ENSG00000116489	858.8753549	0.284206414	0.082279469	3.454159559	0.000552011	0.002645546
ENSG00000116497	297.2246026	-0.193621443	0.111251786	-1.740389531	0.081790643	0.179770483
ENSG00000116514	108.821536	0.027842102	0.185655012	0.149966876	0.880790749	0.934253325
ENSG00000116521	574.4046277	0.210277216	0.096054956	2.189134473	0.028587068	0.077432036
ENSG00000116525	97.83608214	-0.360696361	0.178974572	-2.015349761	0.043868014	0.110215346
ENSG00000116539	1719.579079	0.056498477	0.070447701	0.801991775	0.422557716	0.590003496
ENSG00000116560	4814.981671	-0.216274972	0.052962274	-4.083566605	4.43E-05	0.000276266
ENSG00000116580	566.3884537	-0.082360179	0.091279456	-0.902286036	0.366904941	0.53821154
ENSG00000116584	551.7000904	-0.280163895	0.090476492	-3.096537991	0.001957948	0.008030158
ENSG00000116586	544.853704	0.221040106	0.091943461	2.404087297	0.016212901	0.048631779
ENSG00000116604	1243.793793	0.309007744	0.069366707	4.454698213	8.40E-06	6.13E-05
ENSG00000116641	549.6980901	0.077675734	0.088221345	0.880464181	0.378607901	0.547981993
ENSG00000116649	1697.716184	0.81507469	0.066256808	12.30175	8.86E-35	5.71E-33
ENSG00000116661	292.362274	-1.237311368	0.116715053	-10.60112929	2.94E-26	1.39E-24
ENSG00000116667	99.51949321	0.29687928	0.181778971	1.633188255	0.102429409	0.21372934
ENSG00000116668	21.72538781	0.571853279	0.368057741	1.553705343	0.120254718	0.242286005
ENSG00000116670	163.0019726	0.455813252	0.147174495	3.097094035	0.001954279	0.008019793
ENSG00000116675	52.6788247	4.752496569	0.456371212	10.41366423	2.15E-25	9.83E-24
ENSG00000116679	361.2324646	-0.111695466	0.108798508	-1.026626821	0.30459622	0.475495347
ENSG00000116685	639.5956578	0.220436859	0.085262242	2.585398336	0.009726655	0.031532501
ENSG00000116688	944.5629288	0.124993928	0.073321381	1.704740518	0.08824283	0.190289688
ENSG00000116691	77.4655987	-0.022309593	0.212800426	-0.104838101	0.916504276	0.954800179
ENSG00000116698	1505.957303	-0.021283129	0.069318065	-0.307035817	0.75881611	0.85471012
ENSG00000116704	118.5839797	-0.312007482	0.162887265	-1.915481123	0.055431174	0.132264601

ENSG00000116711	25.37042338	3.318608582	0.46867009	7.080905429	1.43E-12	2.82E-11
ENSG00000116717	120.4438517	-0.408590911	0.173091683	-2.360546179	0.018248046	0.053617588
ENSG00000116731	627.5784008	-0.109409549	0.087499122	-1.250407393	0.211150765	0.366275621
ENSG00000116741	74.15599848	-0.069562839	0.203657779	-0.341567306	0.732676545	0.835382096
ENSG00000116747	1011.354869	-0.042339501	0.081771942	-0.517775413	0.604614975	0.741943439
ENSG00000116750	708.7340861	0.079382834	0.079097651	1.003605449	0.315568827	0.486959828
ENSG00000116752	140.7177416	0.068679115	0.151932832	0.452036036	0.651243022	0.7759127
ENSG00000116754	1320.333371	0.180956433	0.070572362	2.564126056	0.010343597	0.033256239
ENSG00000116761	25.70927987	-0.916850372	0.354805526	-2.584092703	0.009763555	0.031608355
ENSG00000116786	392.4578008	0.202153576	0.106170564	1.904045422	0.056904269	0.134885971
ENSG00000116791	106.500173	0.036048886	0.170833125	0.211018127	0.832873126	0.902241753
ENSG00000116793	152.8589173	-0.200816763	0.148317512	-1.353965288	0.175747452	0.320488701
ENSG00000116809	179.8348499	0.066552407	0.137677452	0.483393661	0.628816248	0.759303863
ENSG00000116815	36.78532301	0.357052636	0.285468812	1.250758826	0.211022481	0.366274791
ENSG00000116830	529.6777653	-0.047762632	0.089178777	-0.535582949	0.592246816	0.73258991
ENSG00000116852	20.30861011	-0.628949982	0.405201377	-1.552191125	0.120616499	0.242805956
ENSG00000116857	397.3355979	0.049014062	0.105935906	0.462676573	0.643596213	0.770067802
ENSG00000116863	88.52389403	0.60953096	0.195763086	3.113615403	0.001848102	0.007648878
ENSG00000116871	1208.285267	0.208072543	0.078173704	2.661669242	0.007775424	0.026117021
ENSG00000116874	96.42088355	0.086196435	0.183627447	0.469409319	0.638777089	0.766678698
ENSG00000116885	15.06214805	-1.224885382	0.472462788	-2.592554194	0.009526617	0.03099129
ENSG00000116898	482.742331	0.278678347	0.093672643	2.975023858	0.002929658	0.011302637
ENSG00000116903	168.2018398	0.05821911	0.139690826	0.416771178	0.676845786	0.794803163
ENSG00000116906	256.7073947	-0.050171094	0.134789322	-0.372218607	0.709730093	0.818195874
ENSG00000116918	151.212285	0.178386716	0.159149175	1.120877421	0.262340043	0.428598579
ENSG00000116922	144.2844112	0.354718764	0.16329803	2.172217041	0.029839296	0.080097953
ENSG00000116954	130.1046631	0.9177024	0.160845445	5.705491997	1.16E-08	1.41E-07
ENSG00000116962	5027.106647	0.043038608	0.059594727	0.722188189	0.470178784	0.63201927
ENSG00000116977	38.46955098	-4.905732539	0.593337105	-8.268035996	1.36E-16	3.67E-15
ENSG00000116981	10.2641163	-1.037495407	0.597042203	-1.737725409	0.082259209	0.180489708
ENSG00000116983	64.43797666	-2.659427197	0.273168824	-9.735471104	2.13E-22	8.15E-21
ENSG00000116984	1206.396293	-0.049629257	0.073238658	-0.677637439	0.498001602	0.6560741
ENSG00000116985	148.8901265	0.258893382	0.157759193	1.641066855	0.100783538	0.210921687
ENSG00000116990	10.48376789	-0.554115847	0.549094238	-1.009145258	0.312904975	0.484076461
ENSG00000117000	236.7417397	-0.082769926	0.124931861	-0.662520557	0.507637665	0.664441509
ENSG00000117010	25.47155057	-0.389253158	0.35122512	-1.108272544	0.267744129	0.434647149
ENSG00000117013	69.71923131	0.562282397	0.216683351	2.594949698	0.009460476	0.030783255
ENSG00000117016	17.98292109	-1.217295308	0.430320673	-2.828809735	0.004672147	0.016901138
ENSG00000117036	341.5307695	0.315502812	0.110407815	2.857613036	0.004268406	0.015650821
ENSG00000117054	446.1860293	-0.171047172	0.093262466	-1.834040854	0.06664793	0.153074472
ENSG00000117114	272.0108738	-1.213518979	0.123667726	-9.812737883	9.92E-23	3.85E-21
ENSG00000117115	52.14153616	-0.648264448	0.24584967	-2.636832691	0.008368409	0.02781615
ENSG00000117118	661.9031811	0.41158087	0.090314228	4.557209646	5.18E-06	3.96E-05
ENSG00000117122	821.5368162	0.094122822	0.087189431	1.079521007	0.280355534	0.449150797
ENSG00000117133	416.4631315	0.020190234	0.100649608	0.200599227	0.841011962	0.907697202
ENSG00000117139	1060.49206	-0.278754081	0.074166973	-3.758466473	0.000170958	0.000928993
ENSG00000117143	276.4956884	0.447615309	0.114282441	3.916746129	8.98E-05	0.000522049
ENSG00000117151	44.51429987	-0.614503577	0.279150197	-2.201336711	0.027712196	0.075528369
ENSG00000117153	296.7647283	-0.094835936	0.116130852	-0.816629978	0.414139918	0.582431606
ENSG00000117155	255.9562912	-0.293207969	0.11771692	-2.49078865	0.012745991	0.0396903

ENSG00000117174	349.5704733	0.449758422	0.1132946	3.969813406	7.19E-05	0.000427044
ENSG00000117222	309.1958976	-0.686157545	0.110835539	-6.190771925	5.99E-10	8.60E-09
ENSG00000117228	45.60668172	0.629879497	0.260490829	2.41804865	0.01560399	0.047086843
ENSG00000117242	128.1923648	0.256304908	0.158234107	1.619782949	0.10527891	0.21834532
ENSG00000117245	50.93845373	-0.894855557	0.253216377	-3.533956095	0.000409389	0.002023465
ENSG00000117262	74.06876431	-0.875792025	0.216265155	-4.049621509	5.13E-05	0.000314681
ENSG00000117266	165.3697	-1.716653369	0.156389958	-10.97674932	4.94E-28	2.50E-26
ENSG00000117280	161.7433594	0.204658641	0.146939277	1.392810999	0.163676951	0.304203511
ENSG00000117298	405.9097179	-0.083847647	0.098615068	-0.850251875	0.395185066	0.563217718
ENSG00000117305	57.28635349	0.073884949	0.233584596	0.316309167	0.751767854	0.849700921
ENSG00000117308	188.0352176	0.124922253	0.146470103	0.852885676	0.393722706	0.561769716
ENSG00000117318	525.5939678	0.776096884	0.089052502	8.715048608	2.91E-18	8.89E-17
ENSG00000117335	724.230257	-0.084280279	0.080669642	-1.044758319	0.296134684	0.466579334
ENSG00000117360	687.9476005	0.042741632	0.082148029	0.52030015	0.602854392	0.740948083
ENSG00000117362	419.3714292	0.049131421	0.103091812	0.476579276	0.633661744	0.763121272
ENSG00000117385	359.1554603	-0.806833263	0.107131803	-7.531220834	5.03E-14	1.13E-12
ENSG00000117394	270.7720109	-0.374276317	0.114738299	-3.261999889	0.001106292	0.004913083
ENSG00000117395	736.173199	0.08774477	0.096156223	0.912523052	0.36149344	0.532441895
ENSG00000117399	533.511097	0.151936476	0.09963971	1.524858667	0.127294353	0.252485707
ENSG00000117407	10.88456434	-1.674936592	0.575458056	-2.910614551	0.003607187	0.013555419
ENSG00000117408	104.1360085	-0.068006192	0.174280057	-0.390212129	0.696379693	0.809436951
ENSG00000117410	187.3212181	0.644722136	0.144510243	4.461428648	8.14E-06	5.96E-05
ENSG00000117411	266.7116869	0.176890865	0.116683506	1.515988594	0.129522237	0.256012817
ENSG00000117419	368.8150149	0.330055209	0.100406116	3.287202232	0.001011881	0.004541181
ENSG00000117425	22.41502853	-0.457774337	0.365299499	-1.253148001	0.21015185	0.365252634
ENSG00000117448	329.2216678	0.182652387	0.112743698	1.620067368	0.105217806	0.218250816
ENSG00000117450	1106.937357	0.370168808	0.083317078	4.442892356	8.88E-06	6.44E-05
ENSG00000117461	255.8159189	0.028174287	0.116875067	0.241063284	0.809506069	0.887468333
ENSG00000117475	106.5753388	0.370953839	0.182835342	2.028895706	0.042468914	0.10739169
ENSG00000117477	91.04398444	1.220037833	0.197725982	6.170346563	6.81E-10	9.72E-09
ENSG00000117479	196.656728	0.169366011	0.136122959	1.244213407	0.213421031	0.368930004
ENSG00000117481	242.459236	0.033416512	0.121851261	0.274240185	0.783900048	0.871011269
ENSG00000117500	405.503037	-0.09788707	0.096978866	-1.009364977	0.312799629	0.484076461
ENSG00000117505	476.9205891	0.091821004	0.104524305	0.878465576	0.379691111	0.549218745
ENSG00000117519	716.8083875	0.27522086	0.090247206	3.049633024	0.002291211	0.009181829
ENSG00000117523	10482.37327	-0.068586252	0.055006763	-1.246869432	0.212445377	0.367650635
ENSG00000117528	265.9812463	-0.301423866	0.119363893	-2.52525164	0.011561547	0.036487318
ENSG00000117533	112.5829176	-0.410854433	0.168204253	-2.442592423	0.014582195	0.044423357
ENSG00000117543	97.51803202	0.134083131	0.178586212	0.750803376	0.452770997	0.617363056
ENSG00000117569	303.3013368	-0.148029181	0.111833186	-1.323660588	0.185615793	0.333590717
ENSG00000117586	9.029415508	-0.266811406	0.570260229	-0.467876581	0.639872852	0.767565619
ENSG00000117592	1586.767055	0.350767363	0.07863287	4.460823603	8.16E-06	5.97E-05
ENSG00000117593	360.1604465	-0.566721406	0.112322305	-5.045493024	4.52E-07	4.29E-06
ENSG00000117597	250.3033685	0.120427991	0.12456943	0.966753976	0.333667037	0.50583001
ENSG00000117602	63.54213023	-1.072953001	0.231603451	-4.632715949	3.61E-06	2.86E-05
ENSG00000117614	594.3847308	0.283804614	0.088034727	3.223780228	0.001265105	0.005508656
ENSG00000117616	222.3478824	0.399303914	0.132189678	3.020689061	0.002522002	0.009962475
ENSG00000117620	125.8786433	-0.453156784	0.16056423	-2.822277321	0.004768392	0.017192404
ENSG00000117625	413.1043737	-0.183898298	0.100002657	-1.838934118	0.065924877	0.151835833
ENSG00000117632	840.6317773	-0.235309149	0.096919818	-2.427874427	0.015187601	0.046002712

ENSG00000117640	182.7510702	-0.171109844	0.134833189	-1.269048413	0.204423798	0.357690718
ENSG00000117643	55.19244787	0.411188859	0.23468317	1.75210203	0.079756265	0.176070447
ENSG00000117650	230.33163	-0.080542527	0.13245889	-0.608056781	0.543149815	0.692169604
ENSG00000117676	144.4211416	-0.548651325	0.157835216	-3.47610209	0.000508759	0.002458133
ENSG00000117682	161.8484083	0.133522367	0.143850462	0.928202556	0.353302509	0.52460864
ENSG00000117691	431.9298577	0.630535967	0.09936332	6.345761847	2.21E-10	3.36E-09
ENSG00000117697	430.9802087	-0.179153886	0.094042959	-1.905021782	0.05677724	0.134740606
ENSG00000117713	2303.805255	-0.133580824	0.062961431	-2.121629401	0.033868871	0.088976255
ENSG00000117724	3885.123433	-0.123778405	0.059261603	-2.088677986	0.03673672	0.095140248
ENSG00000117748	445.6499501	0.200237145	0.097994016	2.043360931	0.041016722	0.10415074
ENSG00000117751	269.1628651	0.012841611	0.116286331	0.110430959	0.912067601	0.952834567
ENSG00000117758	94.47197495	0.142945131	0.184284673	0.775675633	0.437940531	0.603467788
ENSG00000117859	682.4455671	-0.322433585	0.081555771	-3.953534868	7.70E-05	0.000454874
ENSG00000117862	137.8726016	0.549580949	0.17287503	3.179064943	0.00147751	0.006314141
ENSG00000117868	603.9424227	0.348680375	0.086457059	4.032989083	5.51E-05	0.000334744
ENSG00000117877	338.7828045	-0.231178195	0.104783043	-2.206255779	0.027366096	0.074730083
ENSG00000117899	815.3199681	0.225728465	0.079167393	2.851280777	0.00435435	0.015920238
ENSG00000117906	753.0774802	0.081973697	0.083917936	0.976831668	0.328652495	0.501039896
ENSG00000117971	11.47796477	0.096571293	0.512580519	0.188402192	0.850561381	0.914421837
ENSG00000117984	183.1036683	0.531679875	0.305799053	1.738657692	0.082094992	0.180213839
ENSG00000118004	30.13323208	1.180546948	0.323999556	3.643668412	0.00026878	0.001393191
ENSG00000118007	399.9285037	0.363512223	0.100930455	3.601610854	0.000316252	0.001610727
ENSG00000118046	339.9666986	0.346594093	0.1253138	2.765809457	0.00567817	0.01990548
ENSG00000118058	1570.512688	-0.209549533	0.069378116	-3.020398165	0.002524426	0.009969246
ENSG00000118096	24.08831602	-0.956770419	0.366588356	-2.609931281	0.009056041	0.029673481
ENSG00000118160	59.30064592	0.056331614	0.229073019	0.245911171	0.805750996	0.885189466
ENSG00000118162	41.63302205	0.301570214	0.267092601	1.129084869	0.258862034	0.424744187
ENSG00000118181	4523.703086	0.273819332	0.146440454	1.869833953	0.06150688	0.14363876
ENSG00000118193	611.7101786	-0.20603321	0.091476643	-2.252304008	0.024303067	0.06768144
ENSG00000118197	241.3972121	-0.096857814	0.122844057	-0.78846154	0.430426786	0.597156056
ENSG00000118200	852.2637234	0.115410087	0.076822557	1.502294262	0.13302113	0.261298813
ENSG00000118217	576.3675576	0.168832482	0.087078547	1.938852772	0.052519268	0.126759134
ENSG00000118242	10.69822618	-0.047650678	0.537907511	-0.088585262	0.929411521	0.961585302
ENSG00000118246	80.68607557	-0.184784713	0.200944268	-0.919581907	0.357791285	0.529152893
ENSG00000118260	316.8993265	-0.217591097	0.112191508	-1.939461375	0.052445183	0.126645606
ENSG00000118276	135.0647747	-1.470826301	0.169138794	-8.695972508	3.44E-18	1.05E-16
ENSG00000118292	85.84045164	-1.109933898	0.205934416	-5.389744562	7.06E-08	7.58E-07
ENSG00000118298	157.5240462	-1.442587487	0.154801615	-9.318943401	1.18E-20	4.13E-19
ENSG00000118363	99.72813243	0.070879243	0.203472776	0.348347549	0.727579188	0.831595187
ENSG00000118369	70.42125956	0.030300752	0.212813129	0.142381968	0.886778305	0.938065731
ENSG00000118402	206.2948303	-0.018754253	0.133579687	-0.140397491	0.88834594	0.938875587
ENSG00000118412	423.0096301	-0.299912866	0.101305463	-2.960480698	0.003071594	0.011779054
ENSG00000118418	2715.697758	-1.187163618	0.06120932	-19.39514463	8.48E-84	1.65E-81
ENSG00000118420	40.97420748	0.116046377	0.279754404	0.414815192	0.678277197	0.795757928
ENSG00000118454	190.6927346	-0.390668544	0.13264491	-2.945220772	0.003227243	0.012288145
ENSG00000118482	1526.617348	-0.356397467	0.068381181	-5.21192324	1.87E-07	1.89E-06
ENSG00000118495	351.1001372	0.261129112	0.1052429	2.481204073	0.013093938	0.04059381
ENSG00000118496	209.3978316	-0.341461311	0.131345734	-2.599713761	0.009330155	0.030472143
ENSG00000118503	25.88219673	1.018027422	0.343032598	2.967727932	0.003000098	0.011536373
ENSG00000118507	49.48413506	-0.285289148	0.254236241	-1.122141936	0.261802096	0.428099137

ENSG00000118508	310.9596822	0.33851791	0.114768204	2.94957922	0.00318207	0.012149643
ENSG00000118513	87.3472728	-1.586827584	0.204017506	-7.777899122	7.37E-15	1.75E-13
ENSG00000118514	10.30181253	-1.4209431	0.577170406	-2.461912608	0.013819834	0.042404369
ENSG00000118515	11.50925958	-1.033610689	0.557621317	-1.853606842	0.063795428	0.14787696
ENSG00000118518	77.34987446	-0.104242417	0.202826322	-0.513949157	0.607287538	0.743988143
ENSG00000118523	72.31450942	-2.13736151	0.241264573	-8.858994445	8.07E-19	2.58E-17
ENSG00000118557	36.94016232	-0.559835115	0.293978009	-1.904343514	0.056865461	0.134850849
ENSG00000118564	190.5811726	-0.151420128	0.136286929	-1.111039259	0.266551451	0.433380991
ENSG00000118579	266.2766549	-0.021745039	0.115234235	-0.18870294	0.850325647	0.914361666
ENSG00000118596	222.9206124	0.675308815	0.137279406	4.919228864	8.69E-07	7.79E-06
ENSG00000118600	84.12790874	0.333073052	0.191745726	1.737055937	0.082377298	0.180692365
ENSG00000118620	44.55819701	-0.135988613	0.264738134	-0.513672175	0.607481209	0.744127919
ENSG00000118640	92.98835214	-0.730282952	0.198827823	-3.672941439	0.000239774	0.001257697
ENSG00000118655	147.0655828	0.202506748	0.147425571	1.373620245	0.169559609	0.312171358
ENSG00000118680	847.7855405	-0.098713161	0.085183443	-1.158830375	0.246525333	0.410447002
ENSG00000118689	203.3844207	-0.20331762	0.136436304	-1.490201753	0.136171196	0.265703534
ENSG00000118690	11.34800313	0.510372314	0.515904651	0.989276436	0.32252791	0.494364363
ENSG00000118705	1560.356929	0.466307133	0.070095011	6.652501014	2.88E-11	4.85E-10
ENSG00000118707	351.7905407	0.089831307	0.107509753	0.835564255	0.403400085	0.571686944
ENSG00000118733	13.45737804	-1.297295443	0.496966399	-2.610428884	0.009042877	0.02963726
ENSG00000118762	248.2455454	0.147469302	0.125646958	1.173679843	0.240523287	0.402985521
ENSG00000118785	238.6251959	4.939013763	0.235300807	20.99021178	8.06E-98	1.95E-95
ENSG00000118816	1772.4872	0.035878269	0.071840171	0.49941792	0.617484997	0.751499892
ENSG00000118855	134.3026313	0.675752197	0.15814619	4.272959067	1.93E-05	0.000129895
ENSG00000118873	713.9491795	0.111630604	0.081025782	1.377717078	0.168290657	0.310559492
ENSG00000118894	75.162927	0.216626201	0.203912268	1.062350015	0.288076817	0.458124652
ENSG00000118898	10.37662288	-0.695979714	0.54391304	-1.279579018	0.200693233	0.353009998
ENSG00000118900	523.645126	0.041816712	0.095259831	0.438975289	0.660679442	0.783076162
ENSG00000118922	48.0270056	-4.198501534	0.443026726	-9.47685836	2.62E-21	9.54E-20
ENSG00000118946	31.26785654	-0.662651014	0.330201895	-2.006805605	0.044770366	0.111782675
ENSG00000118960	267.1541271	0.14262724	0.132833042	1.073733141	0.282942303	0.452333908
ENSG00000118965	401.2132202	-0.29070888	0.101140355	-2.874311443	0.004049095	0.014959924
ENSG00000118971	2800.100463	-1.067377534	0.066650943	-16.01444021	1.01E-57	1.27E-55
ENSG00000118985	57.46959916	-2.437930725	0.281528209	-8.659632129	4.73E-18	1.42E-16
ENSG00000119004	207.9636739	-0.136822366	0.130093662	-1.051721994	0.292927117	0.463541004
ENSG00000119013	716.8635167	0.29330447	0.083729665	3.502993463	0.000460061	0.002247817
ENSG00000119041	552.4877595	0.332111807	0.08850412	3.752501091	0.000175079	0.00094955
ENSG00000119048	191.1330753	-0.121044436	0.134756854	-0.898243258	0.369055876	0.540463708
ENSG00000119121	101.7212273	-0.168135027	0.184022429	-0.91366595	0.360892401	0.532058728
ENSG00000119125	111.3446862	1.265182937	0.178354735	7.093632441	1.31E-12	2.58E-11
ENSG00000119138	336.5634792	0.042652888	0.108015074	0.394879038	0.692932161	0.806697999
ENSG00000119139	23.30126502	1.015287881	0.378289706	2.683889793	0.007277109	0.024661411
ENSG00000119185	281.0953208	0.337456093	0.123261032	2.737735417	0.006186382	0.021393431
ENSG00000119203	363.4426804	-0.340485223	0.105985923	-3.212551366	0.001315616	0.005700327
ENSG00000119231	474.8977562	-0.028612326	0.093887044	-0.304752659	0.76055454	0.855365062
ENSG00000119242	395.5749291	0.316559212	0.102693355	3.08256764	0.002052231	0.008343759
ENSG00000119280	165.5752842	0.022202045	0.149740214	0.148270426	0.882129354	0.935107024
ENSG00000119285	1496.435719	0.05152762	0.076635229	0.672375101	0.50134493	0.65964708
ENSG00000119314	590.2381339	0.021726751	0.084895298	0.255924082	0.798009459	0.879601293
ENSG00000119318	1588.610182	0.143549242	0.065486419	2.19204601	0.028376185	0.077024206

ENSG00000119321	392.1778989	0.259652141	0.097979742	2.650059452	0.008047761	0.026871142
ENSG00000119326	297.0830231	0.140905478	0.112721593	1.250030938	0.211288246	0.366298879
ENSG00000119328	52.04853224	0.608443272	0.246361284	2.469719525	0.013521902	0.041644851
ENSG00000119333	336.9317361	0.036390145	0.122715989	0.29653956	0.766818061	0.859519095
ENSG00000119335	1577.176565	0.11957221	0.065141229	1.835584184	0.066419178	0.152631687
ENSG00000119383	613.5523339	0.220852766	0.094091655	2.347208851	0.018914649	0.055146135
ENSG00000119392	273.9561703	0.017901416	0.125476506	0.14266747	0.886552809	0.938065731
ENSG00000119396	298.4033679	0.336618329	0.11363345	2.962317256	0.00305333	0.011725021
ENSG00000119397	329.0137791	-0.076302285	0.118322681	-0.644866096	0.519013964	0.673862508
ENSG00000119401	78.07100997	0.089412475	0.202423708	0.4417095	0.658699434	0.781482344
ENSG00000119402	635.8794093	0.061942103	0.082769493	0.748368759	0.454237761	0.61822283
ENSG00000119403	537.5680227	0.041018297	0.090988234	0.450808809	0.652127352	0.776439474
ENSG00000119408	308.385983	-0.331692106	0.110219189	-3.009386206	0.002617761	0.010280037
ENSG00000119414	223.2269089	-0.143143351	0.130947551	-1.093134999	0.274334531	0.442634638
ENSG00000119421	193.5317179	0.165583158	0.134716241	1.229125433	0.219024781	0.376163479
ENSG00000119431	41.83328793	0.211274101	0.290969417	0.726104149	0.467774926	0.629756504
ENSG00000119446	152.3142389	0.340863508	0.149454513	2.280717394	0.022565175	0.063619539
ENSG00000119471	287.900329	-0.037167688	0.112749635	-0.329647971	0.741665971	0.842008184
ENSG00000119487	410.703435	0.104056786	0.100467889	1.035721841	0.30033193	0.470693473
ENSG00000119508	35.19008669	-0.622190871	0.302119309	-2.059421067	0.039453919	0.100692079
ENSG00000119509	339.8459291	-0.213155026	0.112209225	-1.899621229	0.057482844	0.135936282
ENSG00000119514	65.95240113	-0.345597022	0.222086992	-1.55613356	0.119676336	0.241397687
ENSG00000119522	249.7630514	0.032869144	0.118322653	0.277792488	0.781171658	0.868878442
ENSG00000119523	154.4736675	-0.226414582	0.156701193	-1.444881033	0.148491305	0.282751438
ENSG00000119537	194.6937672	-0.233855771	0.135355136	-1.727719973	0.084038441	0.183533579
ENSG00000119541	383.7709405	-0.025879025	0.099666209	-0.259656964	0.795128392	0.87776105
ENSG00000119547	7.688563868	-2.246395093	0.736903338	-3.048425724	0.002300438	0.009206339
ENSG00000119559	101.0567898	-0.326635278	0.17813692	-1.833619212	0.066710538	0.153193221
ENSG00000119574	151.0718025	0.125768519	0.171658813	0.732665666	0.463762369	0.62681324
ENSG00000119596	1463.527417	-0.091415687	0.067546005	-1.353384073	0.175932963	0.320743716
ENSG00000119599	55.51668917	0.060119782	0.23315548	0.257852751	0.796520551	0.878442662
ENSG00000119616	131.2566586	0.234222996	0.164031449	1.427915181	0.153316273	0.28985747
ENSG00000119630	70.8028333	-0.174503281	0.212865257	-0.819782821	0.412339926	0.580607352
ENSG00000119632	117.6548864	-0.180133088	0.166110228	-1.084419003	0.278179062	0.446989394
ENSG00000119636	11.80857153	0.064874904	0.522212736	0.124230797	0.901132539	0.946691255
ENSG00000119638	570.4516593	-0.234676178	0.098008475	-2.394447805	0.016645412	0.04970623
ENSG00000119640	53.27817121	0.176292605	0.248006138	0.710839686	0.477183585	0.637712665
ENSG00000119650	48.86562237	-0.398358432	0.248196055	-1.605015165	0.108490467	0.223520914
ENSG00000119655	421.6826005	0.14264477	0.103356395	1.380125248	0.167548085	0.309718114
ENSG00000119661	68.25618417	-0.208999786	0.224137487	-0.932462428	0.351097595	0.522671567
ENSG00000119669	436.230247	-0.833945773	0.11515571	-7.241896835	4.42E-13	9.14E-12
ENSG00000119673	67.6569537	0.060719012	0.252220411	0.240737899	0.809758263	0.887675536
ENSG00000119681	65.67951471	-0.600206037	0.230551012	-2.603354597	0.009231642	0.030185491
ENSG00000119682	289.1553818	-0.213674149	0.117382227	-1.820327961	0.068709076	0.156757575
ENSG00000119684	10.32761157	-1.95373428	0.621235552	-3.144917049	0.001661339	0.006977344
ENSG00000119685	411.4903728	-0.116587014	0.101326768	-1.150604286	0.249895069	0.413838003
ENSG00000119686	28.55190875	0.641780279	0.33768467	1.900531282	0.057363434	0.135722445
ENSG00000119688	126.1514611	-0.131162976	0.163590065	-0.80177837	0.422681173	0.590003496
ENSG00000119689	512.4078884	-0.141923171	0.091233015	-1.555611972	0.11980039	0.241543861
ENSG00000119699	151.4611834	0.170003534	0.148086742	1.147999688	0.250968693	0.415285337

ENSG00000119703	12.58884651	-0.318414269	0.496940748	-0.640748962	0.521685798	0.675963509
ENSG00000119705	696.4848968	0.39275203	0.102549202	3.829888703	0.000128201	0.000720337
ENSG00000119707	3440.612468	-0.050168019	0.05804222	-0.864336666	0.387402973	0.55608518
ENSG00000119711	67.35426491	-1.442524136	0.23148737	-6.231545759	4.62E-10	6.72E-09
ENSG00000119718	183.492903	0.105615054	0.143845769	0.734224266	0.462812067	0.625890161
ENSG00000119720	267.4044497	-0.092938082	0.116306774	-0.799077118	0.424245697	0.591443436
ENSG00000119723	72.82656138	-0.507037523	0.211162913	-2.401167494	0.016342854	0.048969299
ENSG00000119725	34.39037875	-0.104246649	0.315964784	-0.329931229	0.741451926	0.842008184
ENSG00000119729	399.7122951	0.070294634	0.1022279	0.687626703	0.491687888	0.651295106
ENSG00000119737	38.2422855	-0.566397594	0.291634461	-1.942149059	0.052119058	0.126009709
ENSG00000119760	606.5459218	-0.17241528	0.092228482	-1.869436387	0.061562126	0.143743881
ENSG00000119771	7.673283079	-2.643431125	0.774217392	-3.414326716	0.000639399	0.003011949
ENSG00000119772	815.1573502	-0.689473194	0.083619647	-8.245349249	1.65E-16	4.41E-15
ENSG00000119777	924.7680861	0.123799884	0.076116534	1.626451951	0.103853547	0.215867235
ENSG00000119778	360.179808	-0.190530669	0.10280798	-1.853267303	0.063844055	0.14796526
ENSG00000119782	12.0814526	-0.453105446	0.505718506	-0.895963745	0.370272127	0.541510939
ENSG00000119787	462.4457068	-0.204344976	0.09307709	-2.195437947	0.028132198	0.07646536
ENSG00000119801	100.3340785	-8.72E-05	0.18903769	-0.000461225	0.999631996	0.999854605
ENSG00000119812	585.2184421	0.666589214	0.086845846	7.675545176	1.65E-14	3.83E-13
ENSG00000119820	376.4981269	-0.125265706	0.106042899	-1.181273871	0.237493944	0.398813799
ENSG00000119844	181.3617331	0.376922558	0.135580987	2.780054685	0.005434975	0.019182183
ENSG00000119862	153.483406	0.308383237	0.155332479	1.985310726	0.047109911	0.116341935
ENSG00000119865	209.0331832	0.324897286	0.130075952	2.497750595	0.012498409	0.038988486
ENSG00000119878	72.82061891	-0.073324067	0.211465048	-0.346743197	0.728784255	0.832566072
ENSG00000119888	10.18190308	-0.846796701	0.551311661	-1.535967332	0.124546393	0.248799261
ENSG00000119899	136.7231716	0.663014439	0.164330249	4.034646356	5.47E-05	0.000332678
ENSG00000119900	82.75588554	-0.398404202	0.200672631	-1.985343985	0.047106214	0.116341935
ENSG00000119906	534.2917076	-0.110238894	0.089678315	-1.229270357	0.218970458	0.376116167
ENSG00000119912	329.2171915	-0.047703271	0.10722353	-0.444895549	0.65639524	0.779657734
ENSG00000119915	17.61198009	-1.141184645	0.46375261	-2.460761663	0.013864243	0.042522058
ENSG00000119917	55.69136048	1.062281443	0.243305065	4.366047388	1.27E-05	8.85E-05
ENSG00000119919	11.0888692	6.087390051	1.25444579	4.852652941	1.22E-06	1.06E-05
ENSG00000119922	49.41378229	-0.194479172	0.250320017	-0.776922174	0.437204688	0.603138409
ENSG00000119927	76.92284313	-0.223632622	0.206529998	-1.082809391	0.278893036	0.447675749
ENSG00000119929	162.5313306	0.08752533	0.145224753	0.602688785	0.546715744	0.695122742
ENSG00000119938	7.988036678	-1.052492677	0.626907753	-1.678863711	0.093178608	0.198616071
ENSG00000119946	479.0574235	-0.188437927	0.093256645	-2.020638073	0.043317245	0.10908362
ENSG00000119950	183.006034	0.452713053	0.135988079	3.329064258	0.000871383	0.00397155
ENSG00000119953	547.4740035	0.150696397	0.087371582	1.724775886	0.084567869	0.184403114
ENSG00000119965	68.82903126	0.233799735	0.228414071	1.023578512	0.306034399	0.477121554
ENSG00000119969	891.6983578	-0.2913424	0.085583102	-3.404204722	0.00066357	0.00311849
ENSG00000119977	248.6734116	0.276649481	0.122625055	2.256059998	0.024066874	0.067156809
ENSG00000119979	110.452966	0.584608989	0.172003061	3.398828995	0.00067675	0.003173
ENSG00000119986	59.46105498	-0.204613635	0.231305266	-0.884604307	0.376370083	0.546164313
ENSG00000120008	371.9885926	-0.287396642	0.11406557	-2.519573979	0.011749695	0.036990298
ENSG00000120029	135.1539888	-0.220605867	0.155033811	-1.422953257	0.154749706	0.292054868
ENSG00000120049	31.51280053	0.077790706	0.30845141	0.252197602	0.800888331	0.881184177
ENSG00000120053	335.5944531	-0.186602147	0.104401428	-1.787352436	0.073880544	0.165465562
ENSG00000120063	431.8802226	-0.127931431	0.10633022	-1.203152132	0.228917455	0.387795886
ENSG00000120068	278.83639	-0.162798223	0.117717755	-1.382953853	0.166679016	0.308528087

ENSG00000120071	733.9035335	-0.181569704	0.0823923	-2.203721754	0.027543918	0.075128043
ENSG00000120075	400.3803097	-0.160258905	0.098273879	-1.630737557	0.102945708	0.214551686
ENSG00000120093	672.9671661	-0.345576679	0.092610422	-3.731509618	0.000190336	0.001023993
ENSG00000120129	69.51105185	0.411661535	0.2102834	1.957651129	0.050270963	0.122342078
ENSG00000120137	896.4707717	0.027688414	0.075577446	0.366358153	0.714097841	0.821736084
ENSG00000120156	40.76395007	-6.823967058	1.021652816	-6.679340525	2.40E-11	4.07E-10
ENSG00000120158	135.0767229	-0.197124199	0.156643548	-1.258425271	0.208237997	0.362603093
ENSG00000120159	364.8614734	0.048750889	0.108584624	0.448966779	0.653455622	0.777559757
ENSG00000120162	15.08638368	-0.296646637	0.449789532	-0.659523213	0.509559845	0.665943644
ENSG00000120253	367.6745477	0.146017789	0.108878742	1.341104671	0.179886471	0.325920967
ENSG00000120254	699.252567	-0.135137042	0.089648521	-1.507409599	0.1317057	0.25929502
ENSG00000120256	35.75648848	-0.416933586	0.295851879	-1.409264621	0.158756936	0.297222269
ENSG00000120265	241.0341246	0.235686673	0.122647328	1.921661704	0.054648339	0.130796595
ENSG00000120278	7.071418502	-1.333728182	0.683722464	-1.950686502	0.05109435	0.123851999
ENSG00000120306	184.3751927	0.187485105	0.142907868	1.311929896	0.189543789	0.338829423
ENSG00000120314	571.0721438	0.15579117	0.084739203	1.838478102	0.065991986	0.151940244
ENSG00000120327	10.58978952	-0.405758072	0.541392481	-0.749471199	0.453573251	0.617557765
ENSG00000120333	156.7713355	0.181540846	0.150316175	1.20772662	0.227152435	0.385691383
ENSG00000120334	216.2689	-0.147183183	0.132296067	-1.112528789	0.265910859	0.432748217
ENSG00000120370	121.3745651	-0.509269694	0.170498796	-2.986940125	0.002817849	0.010943413
ENSG00000120437	542.7014145	0.211514759	0.092936479	2.275906734	0.022851592	0.064276395
ENSG00000120438	1459.823135	0.38316761	0.071085107	5.390265669	7.04E-08	7.56E-07
ENSG00000120458	97.43774808	-0.17996337	0.180817318	-0.99527729	0.31960142	0.491293625
ENSG00000120498	23.71219901	-4.34081551	0.653937353	-6.637968436	3.18E-11	5.34E-10
ENSG00000120509	122.6907201	-0.062943324	0.16467139	-0.382235941	0.702286365	0.813676721
ENSG00000120519	60.70478664	-0.112601993	0.228202186	-0.493430825	0.621708206	0.754639706
ENSG00000120526	310.9822628	-0.498022808	0.115603706	-4.308017674	1.65E-05	0.000112598
ENSG00000120533	308.1178985	0.151526496	0.110645482	1.369477483	0.170850069	0.313921637
ENSG00000120539	255.4178137	0.07879391	0.118774493	0.663390831	0.507080277	0.664207073
ENSG00000120549	73.19163625	0.972882645	0.226668119	4.292101819	1.77E-05	0.000120218
ENSG00000120616	338.4472706	0.003966084	0.109292635	0.036288667	0.971052186	0.984973605
ENSG00000120647	45.66251204	-0.599409728	0.275796672	-2.173375495	0.029752068	0.07995548
ENSG00000120656	247.2292428	-0.401197653	0.122223602	-3.282489184	0.001028949	0.004611886
ENSG00000120662	52.65231412	0.403916939	0.246549043	1.638282327	0.101362813	0.211912997
ENSG00000120675	157.5642724	0.074149518	0.143720047	0.515930239	0.605903134	0.742940059
ENSG00000120685	943.9541141	-0.076894388	0.076384917	-1.006669784	0.314093487	0.485376371
ENSG00000120686	281.1628926	-0.018872324	0.118810546	-0.158843845	0.873791904	0.930071597
ENSG00000120688	159.1067433	0.121223912	0.142254749	0.852160742	0.394124883	0.562172241
ENSG00000120690	272.4139402	-0.000306197	0.123418115	-0.00248097	0.998020474	0.999084016
ENSG00000120693	282.3115388	0.670029953	0.122079442	5.488474906	4.05E-08	4.56E-07
ENSG00000120694	1136.453225	0.134759522	0.074738762	1.803074043	0.071376587	0.16145361
ENSG00000120696	118.3247042	0.034903017	0.165944016	0.210330071	0.833410067	0.902386945
ENSG00000120697	208.1696666	-0.090916707	0.128613725	-0.706897393	0.479630244	0.640312302
ENSG00000120699	239.5838869	-0.074908762	0.122001285	-0.613999777	0.539215475	0.689580557
ENSG00000120705	782.6254147	0.000193171	0.079784545	0.002421161	0.998068195	0.999084016
ENSG00000120709	446.1147872	-0.235435566	0.096930537	-2.428910145	0.015144285	0.045906727
ENSG00000120725	256.9258926	0.281837786	0.118273277	2.382937154	0.017175126	0.051060105
ENSG00000120727	506.5262525	0.288003921	0.091929934	3.132863352	0.0017311	0.007233612
ENSG00000120733	1035.333704	-0.121209278	0.071795248	-1.688263236	0.091360709	0.195721196
ENSG00000120738	11013.63782	0.381940339	0.056836656	6.719964985	1.82E-11	3.12E-10

ENSG00000120742	429.790525	0.078264893	0.101503252	0.771057983	0.440672564	0.60586547
ENSG00000120756	112.7270545	-0.125857876	0.171272653	-0.734839296	0.462437372	0.625443652
ENSG00000120784	125.0448561	-0.208222026	0.162012315	-1.28522345	0.198714211	0.350564914
ENSG00000120798	152.4717668	-0.376866018	0.147173733	-2.560688041	0.010446511	0.033462203
ENSG00000120800	342.6353719	0.226560308	0.109858865	2.062285163	0.039180595	0.100105442
ENSG00000120802	1917.80397	0.215449218	0.081121815	2.655872731	0.007910346	0.02649419
ENSG00000120805	525.089357	0.157838534	0.090819811	1.737930665	0.082223032	0.180438514
ENSG00000120820	258.7679388	-0.152623056	0.119412369	-1.278117654	0.201207945	0.353605406
ENSG00000120832	46.6299562	-0.727488388	0.259386677	-2.804648243	0.005037152	0.01801346
ENSG00000120833	118.2295235	-1.787787426	0.182629568	-9.789145558	1.25E-22	4.84E-21
ENSG00000120837	253.7486207	-0.087996051	0.12118489	-0.72613055	0.467758742	0.629756504
ENSG00000120860	39.46764168	0.176359917	0.276796274	0.637147004	0.524029094	0.677437574
ENSG00000120868	44.25141339	-1.411504959	0.286802907	-4.921515523	8.59E-07	7.70E-06
ENSG00000120885	825.5162125	-0.016662781	0.076297922	-0.218391024	0.827124459	0.898668717
ENSG00000120889	916.0840995	0.036481401	0.077220683	0.472430437	0.636619584	0.765502079
ENSG00000120896	496.0187538	0.079181531	0.105511042	0.750457299	0.452979332	0.617396281
ENSG00000120899	26.60478493	-0.483174045	0.350421099	-1.378838334	0.167944605	0.310165377
ENSG00000120910	70.2533804	0.288211006	0.209354327	1.376666105	0.168615503	0.311077222
ENSG00000120913	70.90592625	0.823064747	0.21397409	3.846562679	0.000119786	0.000678758
ENSG00000120925	154.5229433	-0.225585344	0.148086528	-1.523334682	0.127674994	0.25299064
ENSG00000120942	153.828573	-0.098314586	0.14684488	-0.669513199	0.503168164	0.660933822
ENSG00000120948	572.4299303	0.136630287	0.090412869	1.51118186	0.130742123	0.257830759
ENSG00000120963	472.2990355	0.242776151	0.093314468	2.601698934	0.009276325	0.030310429
ENSG00000120992	407.8305645	-0.232651599	0.098234034	-2.368340067	0.017868104	0.052666568
ENSG00000121005	17.30165192	-2.706711357	0.532896555	-5.079243492	3.79E-07	3.66E-06
ENSG00000121022	216.2921896	0.183659201	0.126932081	1.446909243	0.147922344	0.282050382
ENSG00000121039	1031.504009	-3.950921032	0.104934868	-37.65117453	0	0
ENSG00000121057	487.2076213	-0.177247623	0.096567028	-1.835488026	0.066433412	0.152631687
ENSG00000121058	401.391119	0.17018343	0.10094384	1.685921891	0.091810845	0.196356111
ENSG00000121060	1305.842103	0.202347858	0.07028699	2.878880696	0.003990893	0.014779876
ENSG00000121064	247.9250423	0.750047028	0.12703858	5.904088585	3.55E-09	4.58E-08
ENSG00000121067	413.517611	-0.074792302	0.099548811	-0.751312861	0.452464391	0.61715847
ENSG00000121068	6035.27733	0.082072531	0.054761128	1.498737046	0.133941862	0.262446831
ENSG00000121073	300.3199424	0.439348939	0.129919609	3.381698445	0.000720392	0.00335522
ENSG00000121075	13.73022156	-1.871422508	0.527560751	-3.54731186	0.000389184	0.001937916
ENSG00000121104	303.7433609	0.34356992	0.114707106	2.995192982	0.002742713	0.010687069
ENSG00000121152	473.3766238	-0.078934413	0.092308618	-0.855114235	0.39248791	0.560718408
ENSG00000121207	24.74616336	-1.342110422	0.397502026	-3.376361208	0.000734514	0.00340969
ENSG00000121210	184.2436348	0.18032514	0.132977647	1.356056035	0.17508134	0.319564395
ENSG00000121211	15.20058879	-0.075826831	0.475201193	-0.159567846	0.87322151	0.929849389
ENSG00000121236	50.55215177	-4.068116781	0.413565178	-9.83670048	7.82E-23	3.05E-21
ENSG00000121274	474.4374826	-0.812125952	0.092999104	-8.732621203	2.49E-18	7.68E-17
ENSG00000121281	258.4487354	0.129581859	0.116162215	1.11552504	0.264625494	0.431329115
ENSG00000121289	94.88658841	-0.29580185	0.186367206	-1.587199035	0.112467573	0.229860614
ENSG00000121310	251.1898728	-0.128663983	0.124277272	-1.035297774	0.300529869	0.4708514
ENSG00000121316	178.7195065	0.105250854	0.143521716	0.733344451	0.46334837	0.62643451
ENSG00000121350	86.67789518	-0.112062549	0.207856354	-0.539134585	0.589793993	0.731165495
ENSG00000121390	695.0244277	-0.329888837	0.081607239	-4.042396738	5.29E-05	0.000323128
ENSG00000121406	47.02277105	0.252365626	0.258489775	0.976307964	0.328911875	0.501326509
ENSG00000121413	32.97275479	-0.835025408	0.313788453	-2.661109423	0.007788364	0.026147981

ENSG00000121417	60.39996158	0.124267905	0.237290381	0.523695499	0.600490347	0.738900556
ENSG00000121481	352.4728908	-0.096582233	0.103772259	-0.930713403	0.352001835	0.523622765
ENSG00000121486	230.2880446	0.057473668	0.125453623	0.458126806	0.646861342	0.772538913
ENSG00000121542	21.41897662	-0.501879815	0.379256888	-1.323324193	0.18572759	0.333706249
ENSG00000121552	12.42156079	1.973379342	0.537662488	3.670293883	0.000242272	0.001269374
ENSG00000121578	100.7749555	-0.772081315	0.182585613	-4.228598852	2.35E-05	0.000155663
ENSG00000121579	852.8056484	-0.041389988	0.083990431	-0.492794091	0.622158087	0.754883986
ENSG00000121621	158.0899636	-0.175222347	0.145425822	-1.204891571	0.228245163	0.38696065
ENSG00000121644	422.5074239	-0.176632678	0.099062238	-1.783047512	0.07457857	0.166666196
ENSG00000121653	80.70338063	-1.35771325	0.209661514	-6.475739024	9.43E-11	1.50E-09
ENSG00000121671	93.96583204	0.173863933	0.184368986	0.943021583	0.345669864	0.517436549
ENSG00000121680	97.26022464	0.415729677	0.190214108	2.185588029	0.028845759	0.078012395
ENSG00000121690	33.15228312	0.570183185	0.304406208	1.873099725	0.061054615	0.142843801
ENSG00000121691	197.5407802	0.145986639	0.138661457	1.052827815	0.292419914	0.462987211
ENSG00000121716	32.19311311	0.145269588	0.311376183	0.46654046	0.640828694	0.768195995
ENSG00000121741	1029.262352	-0.079472273	0.070874613	-1.12130803	0.262156769	0.428498502
ENSG00000121743	67.99991447	0.212100342	0.214162131	0.990372768	0.321991951	0.493846379
ENSG00000121749	201.4872651	-0.243130827	0.130202913	-1.867322485	0.061856567	0.144351407
ENSG00000121753	186.6971847	-0.057004227	0.137347007	-0.415038001	0.678114085	0.795757928
ENSG00000121766	139.8932388	-0.169873156	0.155327918	-1.093642138	0.274111961	0.442344827
ENSG00000121769	125.5676017	-0.535941884	0.165108186	-3.246004321	0.001170371	0.005145601
ENSG00000121774	1588.027668	0.054169406	0.066126735	0.819175563	0.412686255	0.581036767
ENSG00000121775	225.7741236	0.264800518	0.122651911	2.158959572	0.030853304	0.082410412
ENSG00000121851	34.21548549	-0.79060005	0.303271465	-2.6069055	0.009136457	0.0299021
ENSG00000121858	98.20910019	-3.38867035	0.26344751	-12.86279132	7.29E-38	5.31E-36
ENSG00000121864	218.7980191	0.028458225	0.129449246	0.219840793	0.825995148	0.898324082
ENSG00000121871	69.48584797	-5.768765476	0.573202332	-10.0640998	7.96E-24	3.29E-22
ENSG00000121879	200.1261851	-0.350646428	0.129420026	-2.70936763	0.00674116	0.023050894
ENSG00000121892	1162.390982	0.089392765	0.069123707	1.293228746	0.195931956	0.347068876
ENSG00000121895	6.635454998	-3.39909632	0.985536006	-3.448982382	0.000562703	0.002687622
ENSG00000121897	87.71560325	-0.004600323	0.188045762	-0.024463849	0.980482619	0.990139421
ENSG00000121900	37.66197364	-0.673063075	0.324069519	-2.07690954	0.037809909	0.097291774
ENSG00000121903	100.9833774	-0.100054311	0.179566424	-0.55719944	0.577391172	0.721135751
ENSG00000121931	255.1059444	-0.301930139	0.127430827	-2.369364971	0.01781866	0.05256168
ENSG00000121940	437.681548	0.113446859	0.097308392	1.165848675	0.243675633	0.406763116
ENSG00000121957	217.4635894	-0.188490727	0.128905871	-1.462235398	0.143676714	0.276053455
ENSG00000121964	160.1432572	0.318815942	0.14646227	2.176778644	0.029497087	0.079391758
ENSG00000121988	116.8600233	-0.57912644	0.166545855	-3.477279211	0.00050653	0.002450147
ENSG00000121989	45.31163084	-0.174048206	0.26483862	-0.657185899	0.511061392	0.667402909
ENSG00000122008	287.9571374	0.140072077	0.115164595	1.216277247	0.223879326	0.382164648
ENSG00000122026	184.7316482	0.415213407	0.138399365	3.000110648	0.002698815	0.010536482
ENSG00000122033	267.8105597	0.337816094	0.122849416	2.749838836	0.005962458	0.020731349
ENSG00000122034	923.86842	0.310785964	0.074334026	4.180938108	2.90E-05	0.000188185
ENSG00000122035	18.1140042	0.150751848	0.414358626	0.36381974	0.715992621	0.823285398
ENSG00000122042	218.041223	-0.070439568	0.133753968	-0.526635354	0.598446836	0.737337313
ENSG00000122068	661.554975	0.11125736	0.086021482	1.293367158	0.195884103	0.347068876
ENSG00000122085	173.8846175	0.031886008	0.14373981	0.221831432	0.82444511	0.89742775
ENSG00000122126	337.3230846	0.079265109	0.104203432	0.760676565	0.446850276	0.611843827
ENSG00000122140	675.8832139	0.12936445	0.086999377	1.486958351	0.137025811	0.266735479
ENSG00000122203	800.4424705	-0.065575958	0.086466203	-0.758399879	0.44821163	0.61328938

ENSG00000122218	1557.815563	0.048110781	0.064216793	0.749193153	0.453740795	0.617606294
ENSG00000122257	1219.935559	-0.01817056	0.069885266	-0.260005593	0.794859459	0.87776105
ENSG00000122299	228.1658417	-0.114811158	0.12391945	-0.926498284	0.354187088	0.525593284
ENSG00000122335	40.34337085	0.161815376	0.289989702	0.558003871	0.576841741	0.720834083
ENSG00000122359	579.3942147	0.380944411	0.085597437	4.450418417	8.57E-06	6.24E-05
ENSG00000122367	44.39031745	1.851191668	0.283071535	6.539660265	6.17E-11	1.01E-09
ENSG00000122376	176.0057409	0.044340906	0.136847667	0.324016528	0.745925521	0.845069425
ENSG00000122378	491.1457786	0.35769449	0.099297297	3.602258088	0.000315465	0.001607304
ENSG00000122386	66.67893448	-0.001642209	0.221548277	-0.00741242	0.994085799	0.997280618
ENSG00000122390	257.1283027	-0.065193412	0.122671142	-0.531448641	0.595107922	0.734903558
ENSG00000122406	6509.704072	0.169734605	0.060416668	2.809400308	0.004963389	0.017799521
ENSG00000122417	167.4743855	-0.580576765	0.143161991	-4.055383422	5.01E-05	0.000308369
ENSG00000122435	78.62498576	-0.024113696	0.203587861	-0.11844368	0.905716118	0.949518196
ENSG00000122481	16.09832244	-0.171632929	0.445975355	-0.384848461	0.700349692	0.811968647
ENSG00000122482	663.704932	-0.209660853	0.091617456	-2.288437835	0.022112036	0.062572074
ENSG00000122483	258.712908	-0.547556718	0.124820642	-4.386748126	1.15E-05	8.14E-05
ENSG00000122484	270.1419551	-0.440144313	0.114720395	-3.836670148	0.000124714	0.000704122
ENSG00000122490	101.9201718	0.244722991	0.176968257	1.382863771	0.166706641	0.308528087
ENSG00000122507	34.94681133	-0.036728791	0.318937727	-0.115159756	0.908318496	0.950898041
ENSG00000122512	140.3695934	-0.169954051	0.151236706	-1.123761918	0.261114041	0.427285413
ENSG00000122515	526.0005866	0.218623514	0.101984409	2.143695456	0.032057297	0.08492182
ENSG00000122545	645.8460783	0.10240833	0.083738953	1.222947352	0.221349582	0.379136396
ENSG00000122547	127.3121912	0.504066079	0.162296309	3.105838223	0.001897405	0.007829861
ENSG00000122550	38.28008388	-0.550329076	0.292365277	-1.882333914	0.059790693	0.14040118
ENSG00000122557	190.7581684	-0.440271164	0.13977595	-3.14983488	0.001633628	0.006876706
ENSG00000122565	950.3903132	0.135092185	0.07628327	1.770928065	0.076572665	0.170408146
ENSG00000122566	14367.29351	0.188546721	0.047411556	3.97680943	6.98E-05	0.000416792
ENSG00000122574	130.1050559	1.492474806	0.174353154	8.56006773	1.13E-17	3.26E-16
ENSG00000122591	267.7583353	-0.408795441	0.125686582	-3.252498666	0.001143951	0.005054761
ENSG00000122642	296.5512008	-0.980022075	0.117058134	-8.372097188	5.66E-17	1.57E-15
ENSG00000122643	106.9715225	0.128880884	0.18450189	0.698534224	0.484843162	0.645370217
ENSG00000122644	64.69358999	0.175830031	0.21624338	0.813111742	0.416153995	0.583987529
ENSG00000122674	28.26287301	-0.313721081	0.337966743	-0.928260215	0.353272606	0.52460864
ENSG00000122678	183.0244924	0.112355824	0.144792467	0.775978386	0.437761748	0.603314879
ENSG00000122691	932.8784162	-0.513685258	0.07950767	-6.460826473	1.04E-10	1.64E-09
ENSG00000122692	399.4093656	-0.129893078	0.099472819	-1.305814779	0.191615553	0.34183689
ENSG00000122694	36.33138211	0.528434922	0.290526656	1.81888619	0.068928795	0.157182271
ENSG00000122696	68.94791195	0.01928539	0.213778102	0.090212188	0.928118599	0.960813764
ENSG00000122705	764.3444647	0.169073769	0.081225678	2.081531046	0.037385325	0.096446587
ENSG00000122707	44.59487813	-0.268133165	0.276413645	-0.970043158	0.33202498	0.504440134
ENSG00000122729	549.0148068	0.395258924	0.092854967	4.256734328	2.07E-05	0.000139085
ENSG00000122741	271.0293116	-0.177982652	0.120666271	-1.474999195	0.14021276	0.271215731
ENSG00000122756	11.63660131	-3.503287538	0.766850574	-4.568409619	4.91E-06	3.77E-05
ENSG00000122778	605.4655522	0.480626022	0.092105557	5.218208729	1.81E-07	1.83E-06
ENSG00000122779	799.8916705	0.489667715	0.080684963	6.068884389	1.29E-09	1.77E-08
ENSG00000122783	239.507409	0.46728578	0.120265135	3.885463393	0.000102135	0.000586785
ENSG00000122786	2107.225902	-0.707753564	0.066222162	-10.68756359	1.16E-26	5.56E-25
ENSG00000122862	16.14077967	2.868107415	0.53854788	5.325631241	1.01E-07	1.06E-06
ENSG00000122863	287.4502402	0.555568824	0.115267118	4.819837884	1.44E-06	1.23E-05
ENSG00000122870	90.31934501	-7.969495544	1.007510081	-7.910090125	2.57E-15	6.36E-14

ENSG00000122873	291.1118287	0.277252135	0.110157092	2.516879588	0.011839929	0.037219882
ENSG00000122877	77.39247445	-1.807261251	0.231598737	-7.803415828	6.03E-15	1.44E-13
ENSG00000122882	238.778172	0.042690181	0.121939613	0.350092799	0.726269055	0.830706084
ENSG00000122884	640.9573766	0.485171529	0.084590065	5.735561604	9.72E-09	1.19E-07
ENSG00000122912	68.39509277	-0.271638462	0.217382801	-1.249585802	0.211450892	0.366375183
ENSG00000122952	628.4286978	0.112617231	0.083005871	1.356738134	0.174864431	0.31933446
ENSG00000122958	281.9420947	0.400027267	0.118396541	3.378707378	0.000728275	0.003384081
ENSG00000122965	532.8438961	0.54355379	0.091033699	5.970907406	2.36E-09	3.14E-08
ENSG00000122966	978.2513486	-0.549113543	0.076193488	-7.206830407	5.73E-13	1.16E-11
ENSG00000122970	161.9331493	-1.182564793	0.149865302	-7.890851148	3.00E-15	7.40E-14
ENSG00000122971	48.44362207	-0.122196783	0.24996638	-0.488852876	0.62494586	0.756516245
ENSG00000123009	9.050631355	0.356572805	0.576833821	0.618155163	0.536473063	0.687200175
ENSG00000123064	953.370701	0.349787132	0.07773765	4.49958458	6.81E-06	5.06E-05
ENSG00000123066	995.2346905	-0.477013939	0.073660027	-6.475886015	9.43E-11	1.50E-09
ENSG00000123080	206.0767386	0.335076188	0.145689882	2.299927652	0.021452319	0.061161098
ENSG00000123091	236.7863463	0.41970215	0.131962747	3.180459329	0.001470418	0.006287659
ENSG00000123094	244.1022275	-0.40043403	0.119233408	-3.358404647	0.000783938	0.003612852
ENSG00000123095	14.65981927	0.103638679	0.448419342	0.231120001	0.817221577	0.892507031
ENSG00000123104	471.1529118	-0.70809162	0.100715603	-7.030604948	2.06E-12	3.97E-11
ENSG00000123106	105.3757126	0.242997239	0.185942142	1.306843282	0.191265942	0.341430003
ENSG00000123124	123.159326	-0.438871138	0.16404938	-2.675238022	0.007467621	0.025215787
ENSG00000123130	194.1466652	0.061180048	0.140412972	0.435715075	0.663043467	0.784647984
ENSG00000123131	375.1137119	0.683662745	0.102600066	6.663375315	2.68E-11	4.51E-10
ENSG00000123136	478.1360329	0.587851236	0.101717699	5.77924237	7.50E-09	9.39E-08
ENSG00000123143	720.0127352	0.240837308	0.089890933	2.679216898	0.007379458	0.024948072
ENSG00000123144	610.9694678	0.029345094	0.086187462	0.34047985	0.733495195	0.835693316
ENSG00000123146	1140.33868	0.090012956	0.076606145	1.175009609	0.239990871	0.402189426
ENSG00000123154	62.40694895	0.012325877	0.227167572	0.054258964	0.956728843	0.97684684
ENSG00000123159	490.4517329	-0.097365417	0.09027226	-1.078575154	0.280777162	0.449518669
ENSG00000123178	84.23798679	0.263860634	0.206020757	1.280747815	0.200282259	0.352419503
ENSG00000123179	89.7197158	0.206007472	0.211598893	0.973575376	0.330267418	0.502520193
ENSG00000123191	109.0500239	0.055433473	0.169118656	0.327778577	0.743079086	0.842922019
ENSG00000123200	1376.907538	0.010907508	0.073814112	0.147769954	0.882524323	0.935256066
ENSG00000123213	440.8129356	-0.152484916	0.109008771	-1.398831632	0.161863478	0.301587696
ENSG00000123219	141.8649413	-0.47098506	0.158632411	-2.969034244	0.002987373	0.011493736
ENSG00000123240	253.7039995	0.153216098	0.123482746	1.240789531	0.21468351	0.370701814
ENSG00000123268	348.7127058	0.853242433	0.105806936	8.064144591	7.38E-16	1.90E-14
ENSG00000123297	15.18308505	0.531956421	0.44078731	1.206832432	0.227496683	0.386083074
ENSG00000123349	11.2106627	0.426775824	0.574034788	0.743466829	0.457199097	0.620809639
ENSG00000123352	339.06077	-0.062577554	0.104050257	-0.601416622	0.54756253	0.695834778
ENSG00000123353	97.80904876	0.051484632	0.187081674	0.275198691	0.783163593	0.870477844
ENSG00000123358	243.7894808	-0.804686922	0.12687029	-6.34259542	2.26E-10	3.42E-09
ENSG00000123374	258.4591253	-0.058338779	0.123369829	-0.472877196	0.636300795	0.765247362
ENSG00000123384	1703.907409	-0.284052349	0.064746162	-4.387168886	1.15E-05	8.13E-05
ENSG00000123388	48.53600705	-0.034020836	0.255789217	-0.1330034	0.894190692	0.942712147
ENSG00000123395	200.794722	0.181074547	0.135566152	1.335691426	0.181650153	0.328354098
ENSG00000123411	133.3820282	-0.046916713	0.161300517	-0.290865234	0.771154392	0.863277474
ENSG00000123415	91.12244694	0.117031005	0.185307744	0.631549457	0.527681322	0.680530622
ENSG00000123416	2773.148437	0.174501703	0.077769293	2.243838107	0.02484282	0.068870552
ENSG00000123427	37.6867783	0.394107692	0.290117424	1.358441997	0.174323476	0.318719493

ENSG00000123444	156.6567417	0.145564593	0.151815979	0.958822612	0.337648116	0.509610303
ENSG00000123453	141.936408	0.4490348	0.156243718	2.873938258	0.004053882	0.014973674
ENSG00000123472	335.3538659	-0.430726449	0.111540583	-3.861611944	0.000112641	0.000641896
ENSG00000123473	319.2098136	-0.309256959	0.112497162	-2.749020085	0.005977372	0.020772922
ENSG00000123485	542.6904171	-0.009739394	0.088149268	-0.110487515	0.912022751	0.952834567
ENSG00000123496	8.382892828	-1.313294321	0.635895856	-2.065266363	0.038897804	0.099616674
ENSG00000123505	811.9513635	-0.217207059	0.077412368	-2.805844403	0.005018492	0.017955873
ENSG00000123545	119.1804002	0.377633179	0.170104766	2.220003523	0.026418528	0.072622518
ENSG00000123552	128.139812	0.088062291	0.16101974	0.546903696	0.584444896	0.726457611
ENSG00000123562	892.2835749	0.083604455	0.074822212	1.11737481	0.263834101	0.430338785
ENSG00000123575	211.3457984	-0.222391337	0.131278068	-1.694047907	0.09025618	0.193709482
ENSG00000123595	47.31443534	0.095612584	0.251876875	0.379600487	0.704241999	0.814598762
ENSG00000123600	398.4177348	-0.204231953	0.102718323	-1.988271885	0.046781628	0.115795865
ENSG00000123607	386.9988383	-0.404771452	0.10386536	-3.897078406	9.74E-05	0.000561189
ENSG00000123636	681.0666877	-0.318669044	0.094260518	-3.380726643	0.000722944	0.003365992
ENSG00000123643	124.2752042	-0.419758952	0.168702743	-2.488157235	0.012840695	0.039914415
ENSG00000123684	487.4141315	-0.171909558	0.090349053	-1.902726728	0.057076212	0.135235532
ENSG00000123685	16.39110476	-0.885704428	0.449700904	-1.969541135	0.048890984	0.119834522
ENSG00000123728	180.7155803	-0.29162706	0.138341768	-2.108018883	0.035029356	0.091528528
ENSG00000123737	397.0020467	-0.03498953	0.09735022	-0.359419115	0.719281577	0.82519996
ENSG00000123739	69.43238329	-0.008914027	0.214588151	-0.041540163	0.966865275	0.982787985
ENSG00000123810	30.67279708	0.175677776	0.322089057	0.545432303	0.585456229	0.727328526
ENSG00000123815	302.2982756	0.402336129	0.111156827	3.619535912	0.000295132	0.001516911
ENSG00000123836	186.0093129	-0.007899476	0.140436995	-0.056249255	0.955143244	0.975916289
ENSG00000123908	1714.020191	-0.042509364	0.067691292	-0.627988661	0.530011368	0.682240213
ENSG00000123933	479.9164063	0.203863594	0.09907848	2.057597096	0.039628826	0.101101728
ENSG00000123965	9.798905073	-0.161710869	0.569021393	-0.284191194	0.776263863	0.865892042
ENSG00000123975	688.1385097	-0.015473818	0.1006208	-0.153783495	0.877780448	0.932691525
ENSG00000123983	1498.124745	-0.236205989	0.069029311	-3.421821637	0.000622031	0.002942967
ENSG00000123989	899.1065777	0.109432937	0.24737545	0.442375898	0.658217217	0.781108156
ENSG00000123992	375.9395618	0.34870817	0.102441402	3.403976941	0.000664124	0.003120048
ENSG00000124006	423.6442759	-0.687966564	0.101929407	-6.749441469	1.48E-11	2.57E-10
ENSG00000124067	272.8455773	-0.234491901	0.118221964	-1.983488463	0.047312894	0.116699601
ENSG00000124074	120.342475	-0.238315205	0.167428623	-1.423383886	0.154624901	0.291898399
ENSG00000124098	155.2871055	-0.135696441	0.1508995	-0.899250431	0.368519284	0.539846739
ENSG00000124104	166.4990935	-0.181242399	0.139748971	-1.296914012	0.194660775	0.345863889
ENSG00000124120	147.3764938	-0.343050554	0.157858318	-2.173154748	0.029768672	0.079968486
ENSG00000124126	78.35840498	-3.277323325	0.279110968	-11.74200839	7.76E-32	4.47E-30
ENSG00000124140	30.25516532	-0.189394508	0.317181133	-0.597117825	0.550428718	0.698263542
ENSG00000124143	23.64860199	1.61427451	0.383634803	4.207841674	2.58E-05	0.000169472
ENSG00000124145	38.13351011	-0.530634231	0.307644531	-1.72482907	0.084558281	0.184403114
ENSG00000124151	526.6022759	-0.36603459	0.099031668	-3.696136763	0.000218905	0.001160362
ENSG00000124155	393.3732772	0.145818308	0.099693468	1.46266662	0.143558624	0.275939791
ENSG00000124160	313.5125232	-0.037947162	0.109897986	-0.345294423	0.729873039	0.833064644
ENSG00000124164	471.6510247	0.135516537	0.095639835	1.416946586	0.156498545	0.294209724
ENSG00000124171	128.3709096	-0.488714203	0.168320927	-2.903466675	0.003690562	0.013787588
ENSG00000124172	1099.212568	-0.174014395	0.081057867	-2.146792191	0.031809829	0.084387662
ENSG00000124177	609.0958175	-1.246680955	0.086951732	-14.33762074	1.27E-46	1.20E-44
ENSG00000124181	635.0294429	-0.384991305	0.086936533	-4.428417966	9.49E-06	6.83E-05
ENSG00000124193	526.8356036	-0.050831116	0.089526844	-0.567775143	0.57018766	0.715255051

ENSG00000124198	611.8678409	0.068462395	0.089333248	0.766370826	0.443455687	0.608381876
ENSG00000124201	380.576191	0.548152373	0.10108833	5.422508929	5.88E-08	6.43E-07
ENSG00000124207	1405.878383	0.125853091	0.07322358	1.718750853	0.085659748	0.18614828
ENSG00000124209	311.6278358	0.186767853	0.109141459	1.711245698	0.087035766	0.188409833
ENSG00000124212	39.14400316	-0.002795864	0.291138674	-0.009603204	0.99233787	0.996167373
ENSG00000124214	987.0296324	0.139611909	0.072281066	1.931514251	0.053419489	0.128348228
ENSG00000124216	34.10727711	0.452486594	0.301415949	1.501203222	0.133303007	0.261706127
ENSG00000124217	78.66482565	-0.085898069	0.205115701	-0.418778613	0.675377937	0.793548715
ENSG00000124222	229.7391259	0.126608263	0.125800385	1.006421905	0.314212661	0.485453723
ENSG00000124226	245.7496751	0.160878828	0.117954051	1.363911011	0.172595575	0.316136399
ENSG00000124228	966.886035	0.52042235	0.074108399	7.022447619	2.18E-12	4.20E-11
ENSG00000124243	49.73060877	-0.698599827	0.250096835	-2.793317349	0.005217048	0.018557578
ENSG00000124257	19.40229987	0.090124526	0.40677705	0.221557548	0.824658333	0.897495592
ENSG00000124275	220.0092491	0.162096742	0.13672141	1.185598813	0.235780771	0.396838305
ENSG00000124279	40.02232637	-0.459476955	0.275665418	-1.66679215	0.095555742	0.202637606
ENSG00000124299	163.5740017	0.310162416	0.152794359	2.029933679	0.042363281	0.107182459
ENSG00000124313	308.7601785	-0.161968825	0.110473837	-1.466128346	0.142613325	0.27469394
ENSG00000124333	116.788904	-0.321655276	0.165255829	-1.946408053	0.051605743	0.124854611
ENSG00000124356	128.7507821	-1.880894065	0.178971552	-10.50945829	7.81E-26	3.65E-24
ENSG00000124357	82.01592265	-0.216910365	0.199638146	-1.086517628	0.277250046	0.445853623
ENSG00000124370	43.62517071	0.190684781	0.273773404	0.696505865	0.486112086	0.646507903
ENSG00000124374	20.73609477	-1.015258253	0.410135608	-2.475420896	0.013307924	0.041162584
ENSG00000124380	187.9482834	0.154948207	0.139971387	1.106999154	0.268294294	0.435339026
ENSG00000124383	530.7490606	0.395750255	0.089535696	4.420027667	9.87E-06	7.09E-05
ENSG00000124406	272.3183025	0.165017207	0.122006989	1.352522578	0.176208201	0.321120472
ENSG00000124422	2180.51017	-0.145067087	0.064124325	-2.262278588	0.023680196	0.066235707
ENSG00000124444	53.52360403	0.22908705	0.248137212	0.923227305	0.355888762	0.527227319
ENSG00000124459	101.6372148	0.352899218	0.177575038	1.987324466	0.046886452	0.116034883
ENSG00000124486	1560.773248	0.130416462	0.065921095	1.978372214	0.047886732	0.117946872
ENSG00000124496	699.4170066	-0.068296214	0.084402354	-0.809174278	0.418414908	0.586398705
ENSG00000124507	11.38677546	-0.541208993	0.529207826	-1.022677606	0.306460303	0.47752037
ENSG00000124508	39.80812212	-0.445233773	0.282595271	-1.575517425	0.11513706	0.233988755
ENSG00000124523	224.5104182	-0.262311739	0.125393141	-2.091914578	0.036446158	0.094579564
ENSG00000124532	242.0862311	0.078332656	0.119329681	0.656438995	0.511541707	0.667685036
ENSG00000124535	645.2801092	0.301374812	0.086233942	3.494851379	0.000474326	0.002311369
ENSG00000124541	338.0524576	0.368599647	0.10684847	3.449741946	0.000561123	0.002682808
ENSG00000124562	930.0421784	0.330368792	0.0873247	3.783222757	0.000154811	0.00085246
ENSG00000124570	569.3045896	-0.064904657	0.087581717	-0.741075409	0.458647719	0.622312879
ENSG00000124571	680.9444583	-0.097811349	0.092307767	-1.059622091	0.289316559	0.459478891
ENSG00000124574	167.9000626	-0.038503463	0.142459165	-0.270277191	0.786947011	0.872399068
ENSG00000124575	1689.342822	-0.042310518	0.073545668	-0.575295851	0.565091227	0.71091414
ENSG00000124587	110.2948314	-0.317543066	0.179521612	-1.768829177	0.076922386	0.171078017
ENSG00000124588	344.6686205	0.486733106	0.123033209	3.956111624	7.62E-05	0.000450376
ENSG00000124593	8.820203793	-1.086658399	0.635800365	-1.709118866	0.087428934	0.188922499
ENSG00000124596	176.518015	-0.25077551	0.138320318	-1.813005593	0.069830959	0.158749875
ENSG00000124608	255.7334806	-0.185445872	0.123894772	-1.496801428	0.134444935	0.263138915
ENSG00000124613	26.25713095	-0.030343454	0.336293093	-0.090229193	0.928105086	0.960813764
ENSG00000124614	17.27938067	0.722800849	0.428797372	1.68564664	0.09186388	0.196439629
ENSG00000124635	481.5962282	-0.511549068	0.098281876	-5.204917626	1.94E-07	1.95E-06
ENSG00000124641	28.20255203	0.0094917	0.328940399	0.028855381	0.976979932	0.988555428

ENSG00000124659	217.2544478	0.059051448	0.132303229	0.446334136	0.655355902	0.779028886
ENSG00000124688	64.12723633	0.234058405	0.218750663	1.069978036	0.284629195	0.454184517
ENSG00000124702	271.1667266	-0.350060898	0.116626119	-3.001565191	0.002685955	0.01050379
ENSG00000124733	433.8813688	0.181688774	0.107448099	1.690944518	0.0908474	0.194770059
ENSG00000124762	363.0607642	0.426694296	0.111375769	3.831123237	0.00012756	0.00071788
ENSG00000124766	343.2333685	-0.179049792	0.116034004	-1.543080354	0.122811281	0.246376758
ENSG00000124767	768.3932887	0.008849041	0.081600164	0.108443908	0.913643573	0.953559051
ENSG00000124782	1188.307141	-0.136657931	0.069376363	-1.969805354	0.048860683	0.119781154
ENSG00000124783	1006.694982	0.180081464	0.072872491	2.471185794	0.013466583	0.041525905
ENSG00000124784	287.496156	0.301523765	0.111754867	2.698081724	0.006974032	0.023720151
ENSG00000124786	50.05741884	-0.021414466	0.252516579	-0.084804198	0.932417056	0.963270344
ENSG00000124787	52.03011931	0.387586227	0.243091993	1.594401455	0.110846147	0.227340608
ENSG00000124789	1037.484653	-0.107578029	0.085247552	-1.261948613	0.206967278	0.3608377
ENSG00000124795	2057.336944	0.014422442	0.06608412	0.218243692	0.827239244	0.898686295
ENSG00000124802	31.43356364	-0.189319184	0.315303965	-0.600433884	0.548217112	0.696527295
ENSG00000124831	678.5591107	-0.499682588	0.081668734	-6.118407455	9.45E-10	1.32E-08
ENSG00000124920	50.46558788	1.057418002	0.255168094	4.144005569	3.41E-05	0.000218312
ENSG00000124942	2069.457106	-0.427120568	0.063675733	-6.707744854	1.98E-11	3.38E-10
ENSG00000125037	223.076857	0.29005543	0.125488166	2.311416605	0.020809855	0.05966851
ENSG00000125089	9.460125208	1.654802657	0.612349509	2.702382597	0.006884449	0.023460907
ENSG00000125107	3347.429431	-0.095011203	0.077515314	-1.225708799	0.220308278	0.377905303
ENSG00000125124	162.9013783	0.02392977	0.145859574	0.164060332	0.869683649	0.927526097
ENSG00000125148	178.3588123	0.090470157	0.13912056	0.650300411	0.515498192	0.671096242
ENSG00000125149	150.2803625	0.103340279	0.159898132	0.646288216	0.518092718	0.673067613
ENSG00000125166	1370.291822	-0.024317686	0.078087214	-0.311417005	0.755483628	0.852102835
ENSG00000125170	192.1568619	0.644999111	0.138934751	4.642460631	3.44E-06	2.74E-05
ENSG00000125246	46.86019058	-0.471667221	0.264018371	-1.786493942	0.074019317	0.165749936
ENSG00000125247	130.2629868	-0.444667333	0.159504067	-2.787811878	0.005306535	0.018804464
ENSG00000125249	187.9994945	0.192754636	0.137263663	1.404265573	0.160239782	0.299280444
ENSG00000125257	325.228752	-0.105213476	0.109604249	-0.959939759	0.337085534	0.508979953
ENSG00000125266	37.60898796	0.176304456	0.284133253	0.6204992	0.534929181	0.685910562
ENSG00000125304	580.4977871	0.014910714	0.094768516	0.157338262	0.874978267	0.930840633
ENSG00000125319	103.7421226	-0.0340609	0.17278417	-0.197129748	0.843726004	0.909432081
ENSG00000125337	10.45219906	-0.2120399	0.54086633	-0.39203753	0.695030484	0.808537739
ENSG00000125347	76.95391344	0.641298319	0.210779745	3.042504488	0.002346183	0.009360078
ENSG00000125351	470.5270665	-0.106509678	0.097612487	-1.091148089	0.275207725	0.443584959
ENSG00000125352	129.7241913	0.026314408	0.156630695	0.168002883	0.866581011	0.925200537
ENSG00000125354	685.4326849	-0.082221415	0.089448116	-0.9192079	0.35798684	0.529316947
ENSG00000125356	1536.115819	0.479808631	0.078705952	6.096217879	1.09E-09	1.50E-08
ENSG00000125375	45.42041213	-0.472965862	0.266881676	-1.772193088	0.076362511	0.170048222
ENSG00000125384	41.06249797	-3.46102056	0.400856265	-8.634068782	5.92E-18	1.76E-16
ENSG00000125386	582.7477491	-0.339737921	0.087161935	-3.897778554	9.71E-05	0.000560029
ENSG00000125388	40.63752258	0.075212629	0.270824936	0.277716778	0.78122978	0.868878442
ENSG00000125434	296.7822982	-1.066606676	0.114881479	-9.284409359	1.63E-20	5.63E-19
ENSG00000125445	787.1956348	0.293654142	0.080502976	3.647742671	0.000264554	0.001373825
ENSG00000125447	472.9103444	-0.033235512	0.093435982	-0.355703566	0.722062574	0.827735087
ENSG00000125449	60.74610687	0.158611537	0.243661841	0.650949429	0.515079132	0.670845893
ENSG00000125450	536.3628116	0.149968436	0.088273555	1.698905582	0.089336975	0.192235982
ENSG00000125454	266.4227484	0.564861209	0.115567142	4.887731938	1.02E-06	9.02E-06
ENSG00000125457	141.7889895	0.266714976	0.155685556	1.713164557	0.08668227	0.187847246

ENSG00000125458	469.071808	0.088546005	0.096802635	0.914706557	0.360345701	0.531671417
ENSG00000125459	56.0222347	0.368368769	0.237571325	1.55056074	0.121006987	0.24345247
ENSG00000125482	179.2314325	0.094961423	0.138632772	0.684985387	0.493353143	0.652616216
ENSG00000125484	733.8341262	-0.141488149	0.08532659	-1.658195278	0.097278058	0.205082526
ENSG00000125485	182.5609724	0.11146988	0.143286357	0.777951801	0.436597429	0.602647464
ENSG00000125503	320.8617446	0.171176539	0.113318073	1.51058462	0.130894315	0.257997422
ENSG00000125505	484.277767	0.392183113	0.098178593	3.994588846	6.48E-05	0.000389533
ENSG00000125510	14.014978	-1.777459228	0.50572228	-3.514694327	0.00044026	0.002164627
ENSG00000125520	459.1125442	-0.246851497	0.106601451	-2.315648557	0.020577465	0.059157168
ENSG00000125534	596.5643255	0.266467844	0.186718782	1.427107872	0.153548804	0.290139904
ENSG00000125611	110.7052712	0.064775735	0.167986175	0.385601583	0.69979176	0.811562424
ENSG00000125629	23.70317033	0.738101211	0.358351235	2.059714435	0.039425849	0.100657015
ENSG00000125630	696.0942753	0.262996971	0.080522881	3.266114795	0.00109034	0.004849909
ENSG00000125633	186.4300231	0.046715666	0.13949839	0.33488319	0.737713203	0.839013631
ENSG00000125648	1190.68541	0.311913894	0.071049268	4.390107055	1.13E-05	8.04E-05
ENSG00000125650	38.8118746	-0.524009737	0.296215733	-1.769013857	0.076891562	0.171043492
ENSG00000125651	1137.893701	-0.067214731	0.072369739	-0.92876845	0.353009098	0.524343746
ENSG00000125652	108.4070509	-0.604412627	0.172457269	-3.504709478	0.000457106	0.002234934
ENSG00000125656	424.9238825	0.242359262	0.100679066	2.407245845	0.016073346	0.048295676
ENSG00000125657	15.75711276	0.38193867	0.459872884	0.830530964	0.406238649	0.57446427
ENSG00000125676	961.3820573	-0.025745322	0.073482816	-0.350358408	0.726069736	0.830545732
ENSG00000125686	1228.790996	0.00400982	0.069408504	0.057771313	0.953930789	0.974890199
ENSG00000125691	6657.487192	0.148116342	0.063621742	2.328077422	0.019907993	0.057564343
ENSG00000125703	49.25112175	-0.224021609	0.257109953	-0.871306639	0.383586747	0.552753696
ENSG00000125726	65.79955174	2.274877718	0.246521031	9.227925526	2.76E-20	9.48E-19
ENSG00000125730	19.06057994	-0.955323957	0.408572937	-2.33819686	0.019377038	0.056283966
ENSG00000125733	206.3730768	-0.474241478	0.130534488	-3.633074192	0.000280064	0.001447412
ENSG00000125734	214.8200159	0.119115595	0.132776931	0.897110621	0.369659892	0.541059478
ENSG00000125740	765.2739523	0.342935509	0.220331261	1.556454166	0.119600134	0.241313283
ENSG00000125741	214.5026039	-0.077687539	0.125326628	-0.619880549	0.535336433	0.686182213
ENSG00000125743	541.79708	0.244469768	0.098570981	2.480139339	0.013133105	0.040688294
ENSG00000125744	322.1259345	-1.148996017	0.113559632	-10.11799704	4.60E-24	1.93E-22
ENSG00000125746	362.4032549	-0.573006346	0.111540589	-5.137200281	2.79E-07	2.74E-06
ENSG00000125753	476.9170161	-0.11333363	0.10059229	-1.126663192	0.25988491	0.425974404
ENSG00000125755	1009.796768	0.120841666	0.073079865	1.653556228	0.098217713	0.206815201
ENSG00000125772	81.00627363	-0.35520373	0.197805158	-1.795725319	0.072538213	0.163302323
ENSG00000125775	26.18024691	0.552417337	0.342760416	1.61167192	0.107033351	0.221259462
ENSG00000125779	268.2261047	-0.299521756	0.12105403	-2.474281572	0.013350443	0.041270615
ENSG00000125812	155.0408534	-0.024480586	0.145440688	-0.168320064	0.86633149	0.925004442
ENSG00000125814	46.11887852	0.804765696	0.269495073	2.986198183	0.002824695	0.010963938
ENSG00000125817	527.7236257	0.328977418	0.116400866	2.826245466	0.004709716	0.017020164
ENSG00000125818	762.5720163	0.233712221	0.082265574	2.840948052	0.004497964	0.016360121
ENSG00000125821	159.0589427	0.141114432	0.150066566	0.940345581	0.347040326	0.518998461
ENSG00000125826	301.4281742	0.258272541	0.111921158	2.307629275	0.021019765	0.060159869
ENSG00000125827	119.2688405	-0.163031523	0.171335512	-0.951533757	0.341333491	0.513297457
ENSG00000125834	418.1049718	-0.256031996	0.101727689	-2.516836843	0.011841365	0.037219882
ENSG00000125835	759.0985267	0.477327133	0.078507756	6.079999715	1.20E-09	1.66E-08
ENSG00000125841	474.5034179	-0.243736105	0.096150764	-2.534936746	0.011246763	0.035621935
ENSG00000125843	50.06150505	0.252308742	0.259073831	0.973887409	0.330112446	0.502393232
ENSG00000125844	1719.874144	-0.227377683	0.067273945	-3.379877372	0.000725182	0.003374173

ENSG00000125846	159.9539067	0.053243051	0.147320595	0.36140942	0.717793407	0.824098405
ENSG00000125848	32.54727878	-5.131323439	0.697153376	-7.360393878	1.83E-13	3.95E-12
ENSG00000125863	130.5676623	0.487071731	0.161713177	3.01194832	0.002595768	0.010210796
ENSG00000125868	490.6814298	0.153850764	0.094898239	1.62121832	0.104970825	0.21783501
ENSG00000125870	413.4822328	0.204012015	0.104193235	1.958015934	0.050228143	0.122279848
ENSG00000125871	299.6916324	0.060441086	0.11202526	0.539530874	0.5895206	0.730955589
ENSG00000125875	369.5354429	0.045554246	0.104476187	0.436025155	0.662818479	0.784604829
ENSG00000125877	123.2588631	0.664401312	0.195935873	3.39091204	0.000696605	0.003258476
ENSG00000125885	198.6856808	-0.358262293	0.140823082	-2.544059459	0.010957243	0.03486215
ENSG00000125898	17.17767674	-0.253692313	0.427426798	-0.593533944	0.552823874	0.700037587
ENSG00000125901	695.5731905	0.187143255	0.090431144	2.069455789	0.038503336	0.09878655
ENSG00000125912	830.0043084	0.462831544	0.101314033	4.568286665	4.92E-06	3.77E-05
ENSG00000125931	5.89589968	-5.839983863	1.312350692	-4.450017742	8.59E-06	6.25E-05
ENSG00000125944	2568.803065	-0.098250558	0.058819149	-1.670383879	0.094843437	0.201339846
ENSG00000125945	71.41246406	-0.054574852	0.21547099	-0.253281668	0.800050561	0.880924836
ENSG00000125952	416.4618361	0.437061221	0.097361794	4.489042395	7.15E-06	5.29E-05
ENSG00000125962	82.10206589	-0.031989686	0.196668286	-0.162658081	0.870787654	0.928112848
ENSG00000125967	283.2704539	0.420657759	0.114490786	3.674162556	0.000238631	0.001252633
ENSG00000125968	175.4013106	0.951124374	0.141081616	6.741660616	1.57E-11	2.71E-10
ENSG00000125970	1324.961421	-0.054819814	0.068568103	-0.799494397	0.424003794	0.591223079
ENSG00000125971	30.42765107	0.169410668	0.31254274	0.542040003	0.587790948	0.729570925
ENSG00000125977	849.7439832	-0.009570797	0.076173538	-0.125644637	0.900013228	0.945967201
ENSG00000125991	761.3092044	0.373740264	0.077329497	4.833088025	1.34E-06	1.16E-05
ENSG00000125995	944.5781508	0.554111301	0.087551624	6.328966603	2.47E-10	3.71E-09
ENSG00000126001	1438.4375	0.031306265	0.075697715	0.41356948	0.679189428	0.796574593
ENSG00000126003	639.4044942	-0.070579962	0.088033731	-0.801737706	0.4227047	0.590003496
ENSG00000126005	111.2582049	-0.173198196	0.168377584	-1.02862977	0.303653683	0.474463102
ENSG00000126012	1591.012388	0.356134795	0.068126092	5.227582942	1.72E-07	1.75E-06
ENSG00000126016	52.43104382	0.937887089	0.256138333	3.661642825	0.000250603	0.001308633
ENSG00000126062	293.7498789	0.19736359	0.117776018	1.675753638	0.093786459	0.199578607
ENSG00000126067	813.1559801	0.515079325	0.08220993	6.265414988	3.72E-10	5.49E-09
ENSG00000126070	478.0031535	-0.350441496	0.094072531	-3.725226602	0.00019514	0.001046233
ENSG00000126088	347.4636623	-0.179221542	0.103657685	-1.728974964	0.083813576	0.183241914
ENSG00000126091	84.37061707	-0.667190703	0.216165207	-3.086485155	0.002025381	0.008260895
ENSG00000126106	113.2495164	0.008892127	0.166094688	0.053536492	0.957304456	0.977058468
ENSG00000126107	165.3751539	0.144080394	0.140675816	1.024201586	0.30574007	0.476715591
ENSG00000126214	254.0160231	0.064712424	0.120668854	0.536281085	0.591764301	0.732260534
ENSG00000126215	242.2555013	-0.178792107	0.125577033	-1.423764383	0.154514689	0.291729548
ENSG00000126216	417.2572338	-0.004585741	0.096347441	-0.047595877	0.962038318	0.979825424
ENSG00000126217	83.90973663	0.598031283	0.196103533	3.049569146	0.002291699	0.009181829
ENSG00000126226	274.3373127	0.034131623	0.117649037	0.290113916	0.77172909	0.86337203
ENSG00000126243	143.293378	0.097133934	0.151054798	0.643037728	0.520199619	0.674660147
ENSG00000126247	1398.516566	0.32960555	0.069500862	4.742467071	2.11E-06	1.75E-05
ENSG00000126249	47.55635052	-0.436110499	0.28960065	-1.50590304	0.132092066	0.259927795
ENSG00000126254	348.3299729	0.309233441	0.112992855	2.736752173	0.006204902	0.021441637
ENSG00000126259	59.86253991	-0.893772752	0.240674078	-3.713622841	0.000204313	0.001090108
ENSG00000126261	874.1759198	0.10738216	0.077573484	1.384263725	0.166277717	0.308058967
ENSG00000126267	1440.255496	0.054893687	0.067345034	0.815111132	0.4150087	0.583079337
ENSG00000126351	242.8358097	0.1073688	0.122232089	0.878401087	0.379726095	0.549218745
ENSG00000126368	203.1232277	0.49925971	0.133836189	3.730379021	0.000191192	0.001026241

ENSG00000126391	101.8914398	0.403594234	0.1773122	2.2761786	0.022835322	0.064256364
ENSG00000126432	505.0204701	0.02068647	0.098168422	0.210724277	0.83310243	0.902386945
ENSG00000126453	178.0288894	0.017775243	0.143721704	0.123678211	0.901570064	0.946938439
ENSG00000126456	422.3592577	0.131388252	0.111790554	1.175307274	0.239871805	0.402037853
ENSG00000126457	834.2211685	0.218841093	0.079739763	2.744441213	0.006061401	0.021018144
ENSG00000126458	377.5295274	-0.668279876	0.105067815	-6.360462292	2.01E-10	3.06E-09
ENSG00000126461	535.7601418	0.152649393	0.105635168	1.445062241	0.148440404	0.282692835
ENSG00000126464	1012.393849	-0.385044052	0.087627974	-4.394076834	1.11E-05	7.90E-05
ENSG00000126522	77.28620648	0.487967626	0.207759568	2.348713132	0.018838414	0.054958091
ENSG00000126524	390.8698879	0.189868932	0.101944108	1.862480683	0.062535366	0.145629026
ENSG00000126562	27.62813288	-0.107358986	0.328110995	-0.327203258	0.743514157	0.843153577
ENSG00000126581	330.8843349	-0.022490049	0.105736682	-0.21269864	0.831562027	0.901477895
ENSG00000126583	43.66753869	-1.077265669	0.278137531	-3.873140261	0.000107442	0.000615011
ENSG00000126602	2818.64278	0.40029948	0.057395958	6.974349668	3.07E-12	5.79E-11
ENSG00000126603	425.2489628	-0.123611302	0.104642347	-1.181274177	0.237493822	0.398813799
ENSG00000126653	931.165385	0.230239793	0.074806833	3.07779093	0.002085412	0.008459075
ENSG00000126698	517.2617005	0.325098737	0.094685089	3.433473414	0.000595901	0.002834614
ENSG00000126705	231.2971678	0.123080511	0.123856609	0.993733895	0.320352436	0.492017349
ENSG00000126709	242.1776347	2.795971916	0.43707536	6.397001913	1.58E-10	2.44E-09
ENSG00000126733	29.16691346	1.21673797	0.328440685	3.70458967	0.000211733	0.001127024
ENSG00000126746	584.1468761	0.242364219	0.085875162	2.822285428	0.004768272	0.017192404
ENSG00000126749	135.8598618	-0.030040248	0.168590883	-0.1781843	0.858578239	0.919311573
ENSG00000126756	71.88008058	0.016646402	0.206568252	0.08058548	0.935771614	0.964731953
ENSG00000126767	265.3857593	0.355263115	0.122333007	2.904065915	0.003683506	0.013768031
ENSG00000126768	435.0856359	0.086765142	0.116577793	0.744268183	0.456714246	0.620271199
ENSG00000126773	562.0353751	-0.210867077	0.093207202	-2.262347459	0.023675944	0.066235707
ENSG00000126775	118.4903541	-0.203918895	0.174702923	-1.167232303	0.243116569	0.40603539
ENSG00000126777	1593.384563	0.001913539	0.063199477	0.030277768	0.975845527	0.98780011
ENSG00000126778	1292.648588	-2.796715005	0.080834293	-34.59812545	2.70E-262	2.70E-259
ENSG00000126787	455.3309184	0.013659	0.092663847	0.147403761	0.882813337	0.935491773
ENSG00000126790	61.65375157	-0.002180653	0.232606509	-0.009374857	0.992520056	0.996207876
ENSG00000126804	265.4306692	-0.301793965	0.118278137	-2.551561696	0.010724134	0.034190173
ENSG00000126814	127.6389681	0.225079966	0.16412762	1.371371659	0.170259128	0.313204553
ENSG00000126821	117.1110527	0.265751725	0.173263752	1.533798741	0.125079173	0.24952252
ENSG00000126822	235.740009	0.215687164	0.128370479	1.680192871	0.092919796	0.198244893
ENSG00000126858	138.761909	-0.244920203	0.16338355	-1.499050565	0.133860515	0.262360632
ENSG00000126870	559.6743995	0.306301106	0.090572966	3.381816021	0.000720084	0.003354897
ENSG00000126878	180.3794393	-0.442947266	0.140202323	-3.159343272	0.001581251	0.006678243
ENSG00000126882	463.710591	0.475722128	0.103109206	4.613769662	3.95E-06	3.10E-05
ENSG00000126883	1550.458064	0.01244982	0.067770636	0.183705221	0.854244713	0.916431867
ENSG00000126903	105.8355127	0.119097614	0.172173831	0.691728896	0.489107597	0.64895574
ENSG00000126934	1031.838632	0.119452927	0.075774674	1.576422849	0.114928386	0.233699919
ENSG00000126945	362.9994857	-0.013551239	0.105675202	-0.128234801	0.89796316	0.944497571
ENSG00000126950	152.2118278	2.073837387	0.170587031	12.15706358	5.26E-34	3.28E-32
ENSG00000126953	81.96423109	0.504295049	0.196387534	2.567856718	0.010232944	0.032930623
ENSG00000126970	82.93471714	0.141259416	0.196135714	0.720212614	0.471394099	0.633289298
ENSG00000127022	3562.83627	0.60102245	0.055655107	10.79905291	3.48E-27	1.68E-25
ENSG00000127054	431.5365736	0.020122154	0.095603642	0.210474763	0.833297147	0.902386945
ENSG00000127080	140.707056	0.215752586	0.164571148	1.310998855	0.189858151	0.339261855
ENSG00000127081	97.37458425	-0.349797523	0.183250369	-1.908850308	0.056281405	0.133773595

ENSG00000127084	23.92508253	0.228060586	0.347714993	0.655883671	0.511898975	0.667965156
ENSG00000127124	183.9576163	0.219618746	0.140890212	1.5587935	0.119045263	0.240318445
ENSG00000127125	170.3810627	-0.12834114	0.139735586	-0.918457092	0.358379613	0.529688386
ENSG00000127184	1695.341089	0.318927541	0.077002187	4.14179847	3.45E-05	0.000219923
ENSG00000127191	122.4245302	-0.117398161	0.173268754	-0.677549519	0.498057362	0.6560741
ENSG00000127220	59.69516983	-0.407765085	0.23204587	-1.757260684	0.078873386	0.174560336
ENSG00000127311	62.04344015	0.330856666	0.222804663	1.484962932	0.137553644	0.267397735
ENSG00000127314	372.3453369	0.042238628	0.108830269	0.388114701	0.697931153	0.810339634
ENSG00000127328	195.6089495	0.180129715	0.139522942	1.291040121	0.196689766	0.347928616
ENSG00000127334	371.2763034	0.091954711	0.102868857	0.893902324	0.371374154	0.54217406
ENSG00000127337	172.0287903	0.133438174	0.154995425	0.860916857	0.389283837	0.557731961
ENSG00000127364	12.99984524	0.85912781	0.523683979	1.64054629	0.100891632	0.211116454
ENSG00000127415	55.03146834	0.403294383	0.23482133	1.717452089	0.085896601	0.186547551
ENSG00000127418	717.7243706	0.661429356	0.108382645	6.102723904	1.04E-09	1.45E-08
ENSG00000127419	91.32488057	0.283484345	0.190890125	1.485065528	0.137526467	0.267381907
ENSG00000127423	117.6026115	-0.008951449	0.171934564	-0.052063117	0.958478401	0.977701483
ENSG00000127445	333.7327343	0.027409551	0.112215079	0.244259068	0.807030183	0.8858602
ENSG00000127452	218.9018535	0.232228116	0.124445346	1.866105269	0.062026639	0.144636155
ENSG00000127463	849.3788688	0.508341058	0.079361138	6.405415446	1.50E-10	2.32E-09
ENSG00000127481	2776.127272	-0.252239449	0.063640453	-3.963508073	7.39E-05	0.000438117
ENSG00000127483	1546.221724	-0.12065644	0.064354497	-1.874871935	0.060810344	0.142401179
ENSG00000127507	44.11022662	0.712751596	0.273198395	2.608915752	0.00908296	0.029747806
ENSG00000127511	403.1998922	0.237731723	0.098795062	2.406311794	0.016114505	0.048377955
ENSG00000127526	364.3487437	0.326559625	0.106600384	3.063400071	0.002188373	0.00881815
ENSG00000127527	385.0286369	0.113523615	0.105659736	1.074426444	0.282631596	0.452173323
ENSG00000127528	19.23305968	-0.018601372	0.403353193	-0.046116833	0.96321713	0.980741593
ENSG00000127540	87.02076497	-0.069229409	0.192493651	-0.359645155	0.719112511	0.825140781
ENSG00000127554	161.6827184	0.160714968	0.145116736	1.107487476	0.268083224	0.435046794
ENSG00000127561	44.63146558	-0.003371321	0.260232245	-0.012955049	0.989663656	0.994744093
ENSG00000127564	204.9642268	-0.106561574	0.130122627	-0.818931931	0.412825251	0.581174213
ENSG00000127580	35.41570824	0.774205065	0.29464578	2.627579003	0.008599487	0.028447185
ENSG00000127585	11.96233567	-0.500487572	0.500941002	-0.999094843	0.317748749	0.489570766
ENSG00000127586	189.4932667	0.044196555	0.152371218	0.290058421	0.771771544	0.86337203
ENSG00000127603	2770.563378	-0.33888384	0.065051973	-5.209432148	1.89E-07	1.91E-06
ENSG00000127616	4781.426127	0.198979861	0.052368881	3.799582052	0.00014494	0.000803781
ENSG00000127663	381.0460029	-0.061817766	0.101274594	-0.610397568	0.541598479	0.691142874
ENSG00000127666	124.9643155	0.659568082	0.163858197	4.025237032	5.69E-05	0.000345368
ENSG00000127720	25.2237291	0.059486149	0.350196724	0.169864951	0.865116346	0.924195157
ENSG00000127774	498.5509973	-0.165961373	0.092102463	-1.801921119	0.071557817	0.161765358
ENSG00000127804	542.207118	0.17779588	0.090633746	1.961696259	0.049797858	0.121484979
ENSG00000127824	28.1735343	-0.04458635	0.346816624	-0.128558861	0.89770672	0.944497571
ENSG00000127837	634.0976566	0.372138132	0.083839831	4.438679427	9.05E-06	6.55E-05
ENSG00000127838	355.9042245	-0.216898176	0.102089026	-2.124598342	0.033620142	0.088388946
ENSG00000127870	489.5972526	-0.01114336	0.095226693	-0.117019285	0.906844771	0.950209129
ENSG00000127884	336.4308728	0.351102189	0.107941605	3.252704902	0.001143121	0.005054274
ENSG00000127903	10.47451245	-1.065716024	0.55966113	-1.904216619	0.056881978	0.13485589
ENSG00000127914	1663.154105	-0.006821759	0.067015112	-0.101794343	0.918919916	0.955445452
ENSG00000127922	411.8396679	0.168785436	0.112092209	1.505773126	0.132125424	0.259939193
ENSG00000127946	672.5051613	-0.037644075	0.084463153	-0.445686358	0.65582382	0.779255389
ENSG00000127947	456.857807	0.088840992	0.099807418	0.890124144	0.37339923	0.54353703

ENSG00000127948	356.3289249	0.038092073	0.102243689	0.372561604	0.709474754	0.818158761
ENSG00000127952	47.65732289	-0.911626184	0.261923922	-3.480499901	0.000500479	0.002425053
ENSG00000127955	152.450153	-0.202907068	0.147438782	-1.376212319	0.168755909	0.311295372
ENSG00000127980	218.2922492	-0.191969864	0.128821461	-1.490200951	0.136171406	0.265703534
ENSG00000127989	63.80593936	-0.036140366	0.219241816	-0.164842486	0.869067963	0.927010226
ENSG00000127993	88.31252446	-0.175292029	0.190223996	-0.921503242	0.356787749	0.528096127
ENSG00000127995	72.28109054	0.174866056	0.210678275	0.830014652	0.4065305	0.574731675
ENSG00000128000	67.96181908	-0.399823456	0.234155094	-1.707515517	0.087726276	0.189408392
ENSG00000128011	118.9073829	0.17182908	0.166277909	1.033384897	0.301423812	0.471851946
ENSG00000128016	759.1295129	0.014147563	0.100443382	0.14085112	0.887987558	0.938604531
ENSG00000128039	28.39629822	-0.509993449	0.333568945	-1.528899665	0.126289319	0.251238688
ENSG00000128050	1518.827436	0.135388255	0.072723581	1.861683011	0.062647785	0.145777036
ENSG00000128059	300.3448643	-0.055342925	0.115943262	-0.477327657	0.63312882	0.762675631
ENSG00000128159	173.1967462	0.040730937	0.145335114	0.280255304	0.77928164	0.868088632
ENSG00000128185	54.43442463	0.052998958	0.239493251	0.221296248	0.824861771	0.897647452
ENSG00000128191	316.0796305	0.052063265	0.115480983	0.450838432	0.652106	0.776439474
ENSG00000128203	38.43711971	0.25099992	0.283512038	0.885323678	0.375982083	0.545883237
ENSG00000128228	132.5972751	0.964590541	0.298914478	3.226978323	0.001251049	0.005459301
ENSG00000128245	558.0034643	-0.128982176	0.096157782	-1.341359717	0.17980369	0.325817945
ENSG00000128266	97.80397663	-0.459247326	0.193465034	-2.37380014	0.017606077	0.052076768
ENSG00000128268	187.1585023	-0.324453536	0.141255386	-2.296928602	0.021622843	0.061534861
ENSG00000128271	9.749966144	-0.857801039	0.568412023	-1.509118393	0.13126853	0.258615574
ENSG00000128272	1498.114836	-0.258674848	0.074482587	-3.472957371	0.000514757	0.002484809
ENSG00000128283	690.8791622	0.440664239	0.097294438	4.529182214	5.92E-06	4.48E-05
ENSG00000128294	59.80538564	-1.86E-05	0.225560355	-8.25E-05	0.999934187	0.999954923
ENSG00000128309	156.2518551	-0.343367463	0.155525772	-2.207784978	0.027259266	0.074525283
ENSG00000128311	87.39421328	-0.373988826	0.19699292	-1.898488662	0.05763174	0.136219595
ENSG00000128335	87.43060012	0.873519199	0.191609503	4.558851123	5.14E-06	3.93E-05
ENSG00000128342	71.00644703	-1.509605534	0.227696873	-6.629891386	3.36E-11	5.63E-10
ENSG00000128394	171.5013147	-0.082191212	0.140301887	-0.585816866	0.557998589	0.705122829
ENSG00000128463	347.4978153	0.365886067	0.103940997	3.520132348	0.000431332	0.002122955
ENSG00000128487	414.8461012	0.012882805	0.099118082	0.129974312	0.896586751	0.943962981
ENSG00000128512	42.74064466	0.532687661	0.270367112	1.970238378	0.048811057	0.119690719
ENSG00000128513	115.6450945	0.554673521	0.167460782	3.312259225	0.000925457	0.004198935
ENSG00000128524	1177.027901	0.761267527	0.088631789	8.589102523	8.77E-18	2.57E-16
ENSG00000128534	759.2184598	0.428949484	0.084205202	5.094097201	3.50E-07	3.39E-06
ENSG00000128536	62.17418688	0.499807993	0.224240826	2.22888937	0.025821267	0.071259594
ENSG00000128563	191.4934397	0.145660181	0.138695128	1.050218439	0.293617694	0.463987641
ENSG00000128564	1565.891337	-1.193720024	0.07587673	-15.73235984	9.08E-56	1.11E-53
ENSG00000128567	32.79836204	0.441147889	0.30907551	1.427314278	0.153489327	0.290105567
ENSG00000128578	48.87046513	-0.144720128	0.265174454	-0.545754411	0.585234765	0.72718202
ENSG00000128581	76.89356069	-0.672294653	0.218105021	-3.082435466	0.002053142	0.00834505
ENSG00000128585	721.6309158	0.28046225	0.087825397	3.193407156	0.001406045	0.006041823
ENSG00000128590	52.0128493	0.440894302	0.250664068	1.758905078	0.078593633	0.174105781
ENSG00000128591	1994.858432	0.498724034	0.061275256	8.139077103	3.98E-16	1.05E-14
ENSG00000128595	1861.442191	0.450885063	0.061290436	7.356532192	1.89E-13	4.05E-12
ENSG00000128596	46.85123005	1.091513925	0.273197706	3.995326102	6.46E-05	0.000388489
ENSG00000128602	345.8142912	0.709611636	0.111148843	6.384336671	1.72E-10	2.64E-09
ENSG00000128606	8.309716679	-0.188191812	0.609086157	-0.308974042	0.757341273	0.853459515
ENSG00000128607	167.932974	-0.229868406	0.145574301	-1.579045235	0.114325682	0.232642743

ENSG00000128609	302.2882158	0.692070229	0.110553359	6.260056079	3.85E-10	5.67E-09
ENSG00000128626	218.597372	0.20423118	0.132259952	1.544164927	0.122548383	0.246025031
ENSG00000128641	68.29795907	-0.935587688	0.2226907	-4.201287654	2.65E-05	0.0001734
ENSG00000128654	149.5575485	-0.012798522	0.150217863	-0.085199735	0.932102601	0.96324653
ENSG00000128656	88.58961128	-0.30513672	0.187584128	-1.626665986	0.103808057	0.215867235
ENSG00000128694	71.49906168	0.043420077	0.209355044	0.207399239	0.835698077	0.903907822
ENSG00000128699	186.0701497	0.20952509	0.14162917	1.479392205	0.139035522	0.269644068
ENSG00000128708	527.0990785	0.026772027	0.088111766	0.303841683	0.761248509	0.855734459
ENSG00000128714	10.50211109	-6.671558265	1.258483166	-5.301269374	1.15E-07	1.20E-06
ENSG00000128731	1056.17204	-0.16358064	0.079312227	-2.06248956	0.03916115	0.100105442
ENSG00000128739	43.13690949	0.372485423	0.283292065	1.314845942	0.188561684	0.33746031
ENSG00000128789	221.1122006	-0.008313358	0.124501176	-0.066773333	0.946762153	0.970529663
ENSG00000128791	288.2000924	0.003818338	0.111797634	0.034154012	0.972754338	0.986075028
ENSG00000128805	91.35865578	0.804450808	0.191381094	4.203397468	2.63E-05	0.000172352
ENSG00000128829	1275.185702	0.228922188	0.072067361	3.176502976	0.001490623	0.006358571
ENSG00000128833	38.99223126	-0.688339917	0.29160472	-2.360523922	0.018249141	0.053617588
ENSG00000128872	148.2194316	0.274513987	0.152139915	1.804352179	0.071176115	0.161111762
ENSG00000128881	260.8552451	-0.4525009	0.119213638	-3.7957142	0.000147219	0.000814475
ENSG00000128891	75.32750922	-0.303923344	0.21923106	-1.386315169	0.165650687	0.307221442
ENSG00000128908	676.87503	-0.038771982	0.088889761	-0.436180516	0.662705763	0.784604829
ENSG00000128915	286.3559225	0.164534606	0.11065687	1.486890107	0.137043838	0.266735479
ENSG00000128918	16.8128748	-0.559724034	0.440042246	-1.271977949	0.203380954	0.35635428
ENSG00000128923	436.1916449	0.031688854	0.103780028	0.305346361	0.76010237	0.85522874
ENSG00000128928	367.4056881	-0.182356348	0.102964645	-1.771057895	0.076551075	0.170387093
ENSG00000128944	308.5886262	0.066075185	0.120170464	0.54984547	0.582425368	0.724652715
ENSG00000128951	348.187623	0.170600778	0.11560116	1.475770463	0.140005523	0.270926791
ENSG00000128965	167.0005035	-1.459466954	0.317403383	-4.598145551	4.26E-06	3.30E-05
ENSG00000128973	157.4278695	0.113673333	0.143450849	0.792420081	0.428115774	0.595302146
ENSG00000128989	1181.428113	-0.199336467	0.070191193	-2.839907089	0.004512668	0.016405135
ENSG00000129003	299.1065682	1.5298745	0.114252865	13.3902506	6.89E-41	5.57E-39
ENSG00000129007	20.81794399	0.068791894	0.372362651	0.184744344	0.853429563	0.916032577
ENSG00000129028	21.29057298	-0.21087164	0.388688594	-0.542520782	0.587459795	0.729379663
ENSG00000129055	205.1171489	0.256455602	0.133172489	1.925740104	0.054136834	0.129771349
ENSG00000129071	120.503584	-0.163770496	0.166944056	-0.980990282	0.326597534	0.498773164
ENSG00000129083	509.4139602	0.238068409	0.090539663	2.62943775	0.008552619	0.028341268
ENSG00000129084	105.2608324	0.36318837	0.176920619	2.052832349	0.04008884	0.101978981
ENSG00000129103	598.0311417	0.23950602	0.089764062	2.668172702	0.007626504	0.02569053
ENSG00000129116	199.3139591	-0.172012947	0.134335216	-1.280475454	0.200377972	0.352543759
ENSG00000129128	445.9263869	0.354919281	0.098752145	3.594041245	0.000325588	0.001652887
ENSG00000129158	100.3629665	0.593278402	0.184739478	3.211432704	0.001320749	0.005719039
ENSG00000129173	196.5132843	0.668467742	0.137657999	4.85600364	1.20E-06	1.04E-05
ENSG00000129187	410.4474449	0.335500731	0.097619129	3.436833903	0.000588556	0.002803476
ENSG00000129195	174.6605837	0.125352581	0.138877062	0.902615441	0.366730029	0.538011145
ENSG00000129197	350.0575162	-0.0672487	0.103512296	-0.649668713	0.515906238	0.671096242
ENSG00000129219	54.62807644	-0.729493985	0.246669526	-2.957373761	0.003102718	0.011882191
ENSG00000129235	588.6307675	0.168299916	0.093213743	1.805526854	0.070992277	0.160817371
ENSG00000129244	113.4555181	-2.769379371	0.217549007	-12.7299104	4.03E-37	2.82E-35
ENSG00000129245	640.2843687	0.315183879	0.084958158	3.709871829	0.000207364	0.001105864
ENSG00000129250	1414.17499	0.390731672	0.073053575	5.34856334	8.87E-08	9.41E-07
ENSG00000129255	361.0600318	0.068514831	0.108070244	0.633984233	0.526091112	0.679290546

ENSG00000129292	442.0165191	0.235341891	0.098417593	2.391258342	0.016790733	0.050065683
ENSG00000129315	463.8854883	-0.105181242	0.096986558	-1.084492991	0.278146273	0.446987839
ENSG00000129317	285.0927472	-0.067369571	0.113287447	-0.594678167	0.552058619	0.699491764
ENSG00000129347	693.434998	-0.250874132	0.084140653	-2.981604312	0.002867423	0.011114431
ENSG00000129351	4880.763582	0.054980106	0.057458681	0.956863346	0.338636234	0.510520298
ENSG00000129353	61.45304374	-2.460773808	0.270614127	-9.093293982	9.61E-20	3.21E-18
ENSG00000129354	18.36122905	-0.402823349	0.41466788	-0.971436103	0.331331157	0.503811298
ENSG00000129355	28.50633291	-0.025073133	0.323241354	-0.077567837	0.938171827	0.966102042
ENSG00000129422	341.6233434	-1.965548355	0.117810707	-16.68395347	1.71E-62	2.43E-60
ENSG00000129460	207.5664443	0.051794936	0.129865342	0.39883571	0.690014263	0.804902039
ENSG00000129472	110.1051256	0.199952054	0.173417122	1.153012178	0.248905386	0.413038861
ENSG00000129473	179.1503661	0.439661469	0.136326591	3.225060246	0.001259462	0.005490893
ENSG00000129474	121.4222126	-0.236234185	0.176003318	-1.342214383	0.179526494	0.325436658
ENSG00000129480	66.39158059	-0.203072965	0.228021877	-0.890585447	0.373151609	0.54340148
ENSG00000129484	252.5765494	0.261851879	0.120457603	2.173809483	0.029719446	0.079898384
ENSG00000129493	105.5376175	-0.031425835	0.179219005	-0.175348785	0.860805581	0.920854161
ENSG00000129514	28.23925962	0.943830036	0.330887714	2.852417897	0.004338802	0.015867522
ENSG00000129515	265.1626427	0.510633674	0.117716377	4.337830375	1.44E-05	9.98E-05
ENSG00000129518	100.3235869	0.25675845	0.192146015	1.336267367	0.181461899	0.328098248
ENSG00000129534	238.1903902	-0.239934412	0.121374706	-1.976807359	0.04806341	0.118322242
ENSG00000129559	166.3197019	0.232226192	0.146813509	1.581776726	0.11370055	0.231768071
ENSG00000129562	344.5583267	0.317179523	0.115338021	2.749999694	0.005959532	0.020726305
ENSG00000129566	106.2341509	-0.545759443	0.179329	-3.043341805	0.002339664	0.009336722
ENSG00000129596	34.98079471	-0.126676306	0.292112299	-0.433656188	0.664538126	0.785491334
ENSG00000129625	536.4191679	0.32946136	0.094671836	3.480035585	0.000501347	0.002428175
ENSG00000129636	307.7062212	0.176288493	0.121150214	1.455123243	0.145635146	0.278900735
ENSG00000129646	114.8529765	-0.115692765	0.188325139	-0.614324593	0.539000855	0.689580557
ENSG00000129657	899.4614373	0.497298866	0.075629494	6.575462001	4.85E-11	8.03E-10
ENSG00000129667	154.9680936	0.062821708	0.144300867	0.435352259	0.663306758	0.784755231
ENSG00000129675	83.31870112	1.004847138	0.198463227	5.06314018	4.12E-07	3.95E-06
ENSG00000129680	583.0341979	0.179371217	0.091811006	1.953700593	0.050736636	0.123236319
ENSG00000129691	289.9414755	-0.278778293	0.12062611	-2.311094109	0.020827657	0.059707368
ENSG00000129696	84.56903576	0.116409617	0.192092607	0.606007796	0.544509576	0.693258952
ENSG00000129757	40.14701445	-0.109738928	0.27527533	-0.398651517	0.690149996	0.804993523
ENSG00000129810	224.3500211	-0.096248506	0.131602444	-0.731358048	0.464560483	0.627348693
ENSG00000129911	343.1761706	0.033017079	0.130706608	0.252604514	0.800573842	0.880976166
ENSG00000129925	441.5672216	-0.034728154	0.094460429	-0.367647647	0.713135982	0.820898233
ENSG00000129932	184.3165756	0.119110162	0.138192084	0.861917405	0.38873297	0.557259172
ENSG00000129933	421.4361917	0.228360256	0.09543158	2.392921252	0.016714828	0.049881706
ENSG00000129951	126.2770269	-0.454785848	0.162370704	-2.800910741	0.005095861	0.018181753
ENSG00000129968	83.46117092	0.041240007	0.191428571	0.215432872	0.829429843	0.9003169
ENSG00000129990	256.7372329	0.103259641	0.118561063	0.870940581	0.383786598	0.552984956
ENSG00000129991	45.93033689	0.417497169	0.261631639	1.59574419	0.110545918	0.226791078
ENSG00000129993	14.37303235	-2.174434049	0.571943479	-3.801833798	0.000143629	0.000797769
ENSG00000130005	263.1755203	-0.327610878	0.116111253	-2.821525641	0.004779582	0.017228325
ENSG00000130021	119.5287654	-0.277751011	0.163127989	-1.702656992	0.088632278	0.190982912
ENSG00000130023	46.98601091	0.169100539	0.267720514	0.631630859	0.527628117	0.680524484
ENSG00000130024	480.1962439	0.187587915	0.090961518	2.062277751	0.0391813	0.100105442
ENSG00000130052	28.42247314	-0.482934193	0.333600674	-1.447641535	0.147717329	0.281855101
ENSG00000130054	80.04626347	-1.268312716	0.209275668	-6.060488187	1.36E-09	1.86E-08

ENSG00000130066	66.8581703	-0.154688996	0.230590844	-0.670837544	0.502324027	0.660436748
ENSG00000130119	1144.531312	0.099282996	0.073938785	1.342772893	0.179345523	0.325192535
ENSG00000130147	129.4166919	0.018381174	0.161523431	0.113798811	0.909397281	0.951381168
ENSG00000130150	67.0934186	0.503787564	0.212698724	2.368550006	0.017857966	0.05265551
ENSG00000130158	226.802369	-0.182553965	0.123413648	-1.479204024	0.139085794	0.269667102
ENSG00000130159	369.3743706	0.160732742	0.105278892	1.526732838	0.126827458	0.252037117
ENSG00000130164	1812.586625	0.338642387	0.067583987	5.010689692	5.42E-07	5.10E-06
ENSG00000130165	282.5218381	0.145140156	0.127935474	1.134479364	0.256593525	0.422354024
ENSG00000130175	3791.871261	0.225009691	0.074202694	3.032365539	0.002426452	0.009634833
ENSG00000130176	48.10832829	-1.522954929	0.273920275	-5.559847406	2.70E-08	3.11E-07
ENSG00000130177	270.4036896	0.370972626	0.117806921	3.148988385	0.001638367	0.006892525
ENSG00000130193	281.0518858	0.385472248	0.12200491	3.15948144	0.001580502	0.006677085
ENSG00000130202	548.3806266	-0.057421097	0.086911608	-0.660683862	0.508815075	0.665497779
ENSG00000130203	1438.474186	-3.624005968	0.087058062	-41.62745985	0	0
ENSG00000130204	1262.05669	0.014486717	0.069088881	0.209682315	0.83391563	0.902721285
ENSG00000130208	81.71880056	-1.165498347	0.226255206	-5.151255385	2.59E-07	2.56E-06
ENSG00000130227	856.1348396	0.179119588	0.083174837	2.153530983	0.031276972	0.083272862
ENSG00000130244	79.50339875	0.078739157	0.202899149	0.388070418	0.697963923	0.810339634
ENSG00000130254	1083.682647	0.027494956	0.074940164	0.366892123	0.713699487	0.821479572
ENSG00000130255	7866.175012	0.373977494	0.060391333	6.192568915	5.92E-10	8.52E-09
ENSG00000130270	63.83422302	-0.439410046	0.227306848	-1.933113983	0.053222158	0.128037618
ENSG00000130294	90.68730083	0.654307272	0.190830407	3.428736981	0.000606397	0.00287675
ENSG00000130299	252.9208722	0.297847373	0.151438922	1.966782177	0.049208328	0.120339378
ENSG00000130303	1257.666451	1.714188046	0.158626918	10.80641334	3.21E-27	1.56E-25
ENSG00000130304	82.18421197	-0.416200378	0.198161356	-2.1003105	0.035701536	0.092956345
ENSG00000130305	136.331716	0.09898284	0.168237431	0.588352064	0.556296006	0.703356436
ENSG00000130309	664.014738	0.193239907	0.082580262	2.34002536	0.019282431	0.056035852
ENSG00000130311	341.2391415	0.119147218	0.105221711	1.132344427	0.257489659	0.423283469
ENSG00000130312	450.5156142	0.072312338	0.097736712	0.739868737	0.459379646	0.622670717
ENSG00000130313	221.760654	0.088479844	0.126902578	0.697226528	0.48566104	0.646030363
ENSG00000130332	350.3956495	0.537537412	0.109177442	4.923520849	8.50E-07	7.64E-06
ENSG00000130338	759.203503	-0.081618515	0.080248898	-1.017067105	0.309121508	0.480282028
ENSG00000130340	526.2840901	0.24757945	0.091428045	2.70791582	0.006770719	0.023129449
ENSG00000130347	35.30398081	0.070863453	0.304921794	0.232398781	0.816228296	0.89198373
ENSG00000130348	163.7065998	0.195911186	0.146742399	1.335068716	0.181853857	0.328680022
ENSG00000130349	144.5006896	0.15577	0.15841401	0.983309495	0.325455139	0.497569474
ENSG00000130363	173.6331449	0.168446379	0.13912184	1.210783152	0.225978524	0.384346812
ENSG00000130382	342.2476254	-0.927851317	0.112599182	-8.240302484	1.72E-16	4.60E-15
ENSG00000130396	700.4414473	0.101736766	0.088401964	1.150842824	0.249796903	0.413789489
ENSG00000130402	2265.242136	0.323697961	0.061177463	5.291130836	1.22E-07	1.26E-06
ENSG00000130413	47.47119924	-0.110846688	0.251902336	-0.44003835	0.659909332	0.782521221
ENSG00000130414	835.1514226	-0.003889089	0.078165132	-0.04975478	0.960317802	0.978924831
ENSG00000130429	95.13180988	0.083325007	0.188815618	0.441303575	0.658993238	0.781764886
ENSG00000130449	194.0830642	0.011773577	0.136209111	0.086437513	0.931118628	0.962642479
ENSG00000130475	245.9643393	0.05073822	0.123621173	0.41043309	0.681488277	0.798400454
ENSG00000130477	1492.516695	-0.032967031	0.066929275	-0.492565193	0.622319847	0.754963891
ENSG00000130479	331.2565428	0.175276891	0.12829572	1.36619437	0.171877964	0.315099944
ENSG00000130511	585.4602472	0.253391373	0.110715079	2.288679875	0.022097959	0.062572074
ENSG00000130513	79.38792976	-1.360427955	0.215088286	-6.324974648	2.53E-10	3.80E-09
ENSG00000130517	174.214394	-0.395770521	0.145403789	-2.721872131	0.006491325	0.022305198

ENSG00000130518	71.39645606	-1.165643938	0.223920325	-5.205619171	1.93E-07	1.94E-06
ENSG00000130520	980.3216973	-0.078048943	0.072158345	-1.081634325	0.279415046	0.448121697
ENSG00000130522	643.1390144	0.19252199	0.095724219	2.011215058	0.044302746	0.110871312
ENSG00000130528	93.78400354	-2.129638789	0.215766747	-9.870097297	5.61E-23	2.20E-21
ENSG00000130529	122.0044839	-0.236609607	0.191917519	-1.232871329	0.217623774	0.374627592
ENSG00000130540	98.33859655	0.131222525	0.177089109	0.74099715	0.458695168	0.622312879
ENSG00000130544	158.0500744	0.297714292	0.147761657	2.014827789	0.043922696	0.110304062
ENSG00000130559	653.140056	0.133559469	0.081617441	1.636408425	0.101754137	0.212572927
ENSG00000130560	250.2806801	0.020689508	0.120634727	0.171505407	0.863826379	0.923031275
ENSG00000130584	104.9885223	0.639533416	0.175109719	3.652186867	0.000260017	0.001352259
ENSG00000130589	52.35722231	6.869777194	0.859035174	7.997084872	1.27E-15	3.24E-14
ENSG00000130590	37.93047926	0.429295201	0.281841614	1.523178906	0.127713952	0.253032141
ENSG00000130635	21.85550016	-2.949668343	0.494907597	-5.960038529	2.52E-09	3.34E-08
ENSG00000130638	315.5379774	0.046832671	0.109027993	0.429547218	0.667525039	0.787430654
ENSG00000130640	407.5433006	0.153358859	0.096899367	1.582661098	0.11349873	0.231462929
ENSG00000130643	66.88357326	-1.759795808	0.249331486	-7.058056879	1.69E-12	3.29E-11
ENSG00000130649	12.10252011	-2.377050591	0.595435872	-3.992118554	6.55E-05	0.000392774
ENSG00000130653	18.55065215	-0.285465047	0.417220414	-0.684206806	0.493844585	0.653081801
ENSG00000130669	381.2311916	0.503724968	0.101337935	4.970744334	6.67E-07	6.15E-06
ENSG00000130684	193.9763468	-0.068079984	0.146876256	-0.463519327	0.642992165	0.769607271
ENSG00000130695	355.3640183	-0.265636723	0.104664334	-2.537987023	0.011149212	0.035376774
ENSG00000130699	322.5207325	-0.003587208	0.107908368	-0.033243095	0.973480732	0.986330341
ENSG00000130700	56.87756439	-0.025105695	0.23529106	-0.106700589	0.915026516	0.954010055
ENSG00000130702	1060.921607	-0.235005768	0.093322293	-2.518216814	0.011795069	0.037099269
ENSG00000130703	320.9556864	-0.160248399	0.108084728	-1.482618328	0.138175845	0.26827313
ENSG00000130706	912.8037304	0.804437572	0.078562951	10.2394011	1.32E-24	5.71E-23
ENSG00000130707	38.31619786	0.216823643	0.286274669	0.757397236	0.448811912	0.613811793
ENSG00000130713	440.9571091	0.129307101	0.096739333	1.336654878	0.181335317	0.327968244
ENSG00000130714	292.4101537	0.394220903	0.124467989	3.167247315	0.001538894	0.006527844
ENSG00000130717	134.0478958	0.11274988	0.154993428	0.727449421	0.466950689	0.629185259
ENSG00000130724	461.9200101	0.170077553	0.09313387	1.826162188	0.067825835	0.155348036
ENSG00000130725	544.9493633	0.32736253	0.093358032	3.506527738	0.000453994	0.002221907
ENSG00000130726	1688.178384	0.280348963	0.075155363	3.73025892	0.000191283	0.001026338
ENSG00000130731	440.1056338	0.369680863	0.097435551	3.794106551	0.000148176	0.000819138
ENSG00000130733	186.9586998	0.717214722	0.143683924	4.991614241	5.99E-07	5.57E-06
ENSG00000130734	71.72130839	0.037484832	0.230812364	0.162403919	0.870987785	0.928186766
ENSG00000130741	2241.633249	0.273944437	0.060639094	4.517620893	6.25E-06	4.69E-05
ENSG00000130748	137.9041546	-0.161650306	0.168520491	-0.959232346	0.337441709	0.509394358
ENSG00000130749	910.3294395	0.003345893	0.081016658	0.041298822	0.967057672	0.982799821
ENSG00000130751	118.910716	0.755894745	0.18177161	4.158486281	3.20E-05	0.000206051
ENSG00000130758	92.3864225	0.092772535	0.194113433	0.477929493	0.632700389	0.762290279
ENSG00000130762	34.52132282	0.65808516	0.308578235	2.132636347	0.032954569	0.086948316
ENSG00000130764	330.6125242	0.413353262	0.108542664	3.808210031	0.000139976	0.000779021
ENSG00000130766	330.5994622	-0.320561177	0.116073755	-2.761702493	0.005750085	0.020102399
ENSG00000130768	38.08349555	0.489171898	0.282664	1.730577285	0.083527186	0.182769687
ENSG00000130770	2208.226994	-0.119292453	0.061955891	-1.925441663	0.054174128	0.129838589
ENSG00000130772	42.58616825	-0.103634921	0.273291029	-0.379210839	0.704531303	0.814832908
ENSG00000130775	58.12050897	-0.170021406	0.229815942	-0.739815545	0.459411925	0.622670717
ENSG00000130779	822.2092027	-0.459153746	0.079080011	-5.806192273	6.39E-09	8.09E-08
ENSG00000130783	16.36962773	0.282972582	0.430824733	0.656816009	0.511299229	0.66749259

ENSG00000130787	222.0357949	0.101151581	0.123771859	0.817242155	0.413790056	0.582182384
ENSG00000130803	388.6500218	-0.010528573	0.101818774	-0.103405029	0.917641521	0.955355281
ENSG00000130810	143.0962809	0.295818609	0.151642374	1.950764824	0.051085028	0.123851999
ENSG00000130811	605.2098042	0.268690859	0.087974206	3.054200458	0.002256612	0.009061931
ENSG00000130813	52.37126873	0.815401163	0.245770345	3.317736169	0.000907502	0.004124126
ENSG00000130816	2403.718992	0.113143991	0.059759551	1.89332063	0.058315243	0.137556569
ENSG00000130821	867.2303162	1.451141568	0.078569131	18.46961465	3.63E-76	6.21E-74
ENSG00000130826	833.940083	0.119299543	0.080843524	1.475684595	0.140028583	0.270934092
ENSG00000130827	336.428093	0.132449192	0.1090902	1.214125478	0.224699812	0.382809194
ENSG00000130829	105.2202109	0.608833402	0.176692711	3.445718829	0.000569543	0.002719363
ENSG00000130830	79.96259579	-0.092164226	0.202051303	-0.456142697	0.648287368	0.77370371
ENSG00000130844	165.4155935	0.237821292	0.149872341	1.586825768	0.112552111	0.229999928
ENSG00000130856	400.5084902	0.369972108	0.101422404	3.647834148	0.00026446	0.001373825
ENSG00000130876	9.308862544	-2.770175565	0.721115576	-3.841513976	0.000122278	0.000690924
ENSG00000130881	184.7830841	0.096758643	0.141867576	0.682034934	0.495216861	0.654135261
ENSG00000130921	122.3379164	-0.453246723	0.167611412	-2.704151928	0.006847897	0.023359012
ENSG00000130935	555.4395694	0.052975799	0.093508974	0.566531708	0.571032384	0.716186777
ENSG00000130939	959.6960286	-0.148090129	0.080059246	-1.849756728	0.06434862	0.14891352
ENSG00000130940	13.3055514	-0.41040544	0.483256761	-0.849249246	0.395742616	0.563477628
ENSG00000130956	342.936213	0.432437055	0.104224922	4.149075369	3.34E-05	0.00021402
ENSG00000130958	36.76796323	0.178335819	0.294876493	0.604781403	0.545324252	0.693981678
ENSG00000130962	89.19627879	-0.196947401	0.203071449	-0.969842893	0.332124809	0.50452657
ENSG00000130985	3924.869056	-0.044184857	0.069587904	-0.634950248	0.525460864	0.678663916
ENSG00000131013	248.0321578	0.139713731	0.118271342	1.181298261	0.237484258	0.398813799
ENSG00000131016	3857.234793	0.703573399	0.058838925	11.95761823	5.92E-33	3.57E-31
ENSG00000131018	816.3343112	1.460596318	0.085962925	16.99100309	9.57E-65	1.40E-62
ENSG00000131019	22.35144812	0.579019864	0.369427729	1.567342727	0.117034603	0.236885457
ENSG00000131023	592.5045186	-0.213744817	0.085895323	-2.488433704	0.012830716	0.039892223
ENSG00000131037	61.16295146	-0.617263181	0.244298162	-2.5266796	0.01151465	0.036372
ENSG00000131043	169.8691615	0.024245499	0.138915475	0.174534181	0.86144567	0.921258267
ENSG00000131051	1553.473415	-0.002461881	0.069644042	-0.035349484	0.971801065	0.985302874
ENSG00000131061	128.4656647	0.745538946	0.159777381	4.666110702	3.07E-06	2.45E-05
ENSG00000131067	225.4925237	0.476601286	0.131346344	3.628584333	0.00028498	0.00146957
ENSG00000131069	638.5915778	-0.381140045	0.098420733	-3.872558495	0.000107699	0.000615979
ENSG00000131080	108.4238742	-0.260807183	0.170553546	-1.529180658	0.126219664	0.251169801
ENSG00000131089	219.0604307	-0.211402293	0.125467015	-1.68492327	0.092003376	0.196678044
ENSG00000131094	80.89631039	-0.825562227	0.210362102	-3.924481732	8.69E-05	0.000507447
ENSG00000131100	608.1369588	-0.131684025	0.083677483	-1.573709197	0.115554695	0.234516753
ENSG00000131115	159.1373445	0.597524168	0.148949497	4.011589017	6.03E-05	0.000363759
ENSG00000131116	143.987386	0.269929421	0.157428949	1.714611081	0.086416556	0.187453484
ENSG00000131143	1986.207602	0.226275142	0.07173298	3.154408772	0.001608237	0.006784053
ENSG00000131148	200.6301357	0.053019541	0.131042208	0.404598956	0.685772326	0.801843308
ENSG00000131149	1288.441341	-0.244783687	0.068995363	-3.547828074	0.000388422	0.001934808
ENSG00000131153	275.9675425	-0.371333012	0.11779279	-3.152425636	0.001619201	0.006828253
ENSG00000131165	268.7657652	-0.058480583	0.12739901	-0.459034831	0.646209154	0.772273267
ENSG00000131171	685.5156369	0.448715964	0.091049628	4.928256947	8.30E-07	7.49E-06
ENSG00000131174	742.3986286	0.394366743	0.082254535	4.794468087	1.63E-06	1.38E-05
ENSG00000131187	160.7253798	0.482302057	0.145897974	3.305748834	0.00094723	0.004279747
ENSG00000131188	112.3745574	0.527799119	0.181723073	2.90441445	0.003679407	0.01375689
ENSG00000131236	854.4135352	-0.010484215	0.07897424	-0.132754874	0.894387244	0.942843412

ENSG00000131238	541.7948378	0.549728984	0.095109439	5.779962398	7.47E-09	9.36E-08
ENSG00000131242	178.8529097	-1.001453043	0.144477904	-6.931530815	4.16E-12	7.75E-11
ENSG00000131263	797.4355951	-0.124937143	0.079982776	-1.562050611	0.118276067	0.239019408
ENSG00000131269	240.7368414	-0.318011838	0.124622844	-2.551794096	0.010716983	0.034175134
ENSG00000131323	493.6502802	0.061350269	0.092433324	0.663724575	0.506866608	0.664065744
ENSG00000131351	187.0207888	0.163900071	0.135078197	1.213371773	0.224987713	0.383162156
ENSG00000131368	984.3997644	0.193741031	0.072935703	2.656326376	0.007899712	0.026464882
ENSG00000131370	155.5705242	0.657696432	0.149048258	4.412640854	1.02E-05	7.31E-05
ENSG00000131373	157.6682214	0.056445153	0.148693089	0.37960845	0.704236088	0.814598762
ENSG00000131374	1703.159428	0.051773919	0.061803382	0.837719832	0.402188073	0.570602551
ENSG00000131375	478.3312417	0.173740141	0.096478308	1.800820772	0.071731134	0.161994732
ENSG00000131378	62.31862283	0.764020039	0.227510625	3.358173008	0.000784595	0.003613851
ENSG00000131381	195.5322007	0.502989268	0.135432109	3.713958767	0.000204042	0.001089388
ENSG00000131389	622.0104232	0.233852782	0.088728664	2.635594527	0.008399003	0.027904634
ENSG00000131398	786.837071	-0.251981696	0.080727785	-3.121375059	0.001800086	0.007465548
ENSG00000131408	322.9252879	0.581222831	0.115869654	5.016178208	5.27E-07	4.97E-06
ENSG00000131409	222.0487252	0.27778468	0.141844267	1.958377912	0.050185685	0.122197662
ENSG00000131437	225.2093108	-0.183592895	0.122632389	-1.497099553	0.134367356	0.263023725
ENSG00000131446	468.400658	-0.479119181	0.099411472	-4.819556249	1.44E-06	1.23E-05
ENSG00000131459	109.639943	-0.020982047	0.182408786	-0.115027614	0.908423235	0.950936819
ENSG00000131462	676.5759756	0.263567018	0.094170919	2.798815409	0.005129045	0.018281577
ENSG00000131467	706.8232669	0.041959948	0.098564698	0.425709702	0.670319391	0.789599706
ENSG00000131469	2878.911618	0.404897793	0.06247528	6.480928012	9.12E-11	1.45E-09
ENSG00000131470	148.8734007	-0.356667485	0.150352703	-2.372205343	0.017682261	0.052236112
ENSG00000131471	19.45814076	-0.589303688	0.405210134	-1.454316264	0.145858643	0.279252605
ENSG00000131473	2304.634886	0.085579629	0.059771157	1.431788068	0.152204483	0.288402768
ENSG00000131475	341.3572509	-0.10713311	0.104341369	-1.026755834	0.304535451	0.475480291
ENSG00000131480	26.56928776	-0.457306348	0.344554236	-1.327240532	0.184429128	0.331925172
ENSG00000131495	165.4308081	-0.090498153	0.148720055	-0.608513445	0.542846992	0.6920203
ENSG00000131503	29.34245187	-0.328472814	0.321695648	-1.021067011	0.307222691	0.478284068
ENSG00000131504	516.1656964	-0.708822613	0.089990506	-7.876637706	3.36E-15	8.19E-14
ENSG00000131507	349.1332686	0.223207393	0.10236082	2.18059402	0.029213458	0.078781864
ENSG00000131508	382.1294532	0.246586358	0.113608428	2.170493533	0.029969478	0.08040131
ENSG00000131558	419.0178528	0.348211104	0.098233027	3.544745747	0.000392992	0.001953418
ENSG00000131584	187.2692754	-0.067440583	0.148268679	-0.454853872	0.64921437	0.774150659
ENSG00000131591	134.1189792	0.364791464	0.164928309	2.211818377	0.026979217	0.073932317
ENSG00000131626	753.8448402	0.666918022	0.078812851	8.462046638	2.63E-17	7.38E-16
ENSG00000131650	271.9334349	-0.091276302	0.13364982	-0.682951176	0.494637695	0.653699849
ENSG00000131652	126.9401258	-0.114016065	0.171150183	-0.666175537	0.505298907	0.662868093
ENSG00000131653	644.4227271	0.062355351	0.092851265	0.671561678	0.501862782	0.660204766
ENSG00000131668	336.610161	3.033394099	0.14533388	20.87189929	9.64E-97	2.30E-94
ENSG00000131669	334.7970821	0.283711567	0.122475915	2.316468247	0.020532716	0.059078874
ENSG00000131697	160.4292724	-0.206414656	0.155389779	-1.328367011	0.184056892	0.331425095
ENSG00000131711	1116.069603	-1.459426952	0.079101378	-18.45008253	5.21E-76	8.81E-74
ENSG00000131724	483.4699906	0.022657361	0.100728715	0.224934484	0.822030243	0.895954284
ENSG00000131725	128.4556608	-0.418326287	0.164905144	-2.536769177	0.011188069	0.03547603
ENSG00000131732	182.4081274	0.245144499	0.136590559	1.794739709	0.072695181	0.163567068
ENSG00000131747	2068.885036	-0.12999356	0.069648152	-1.866432286	0.06198091	0.144558977
ENSG00000131748	134.2748317	-0.016119938	0.162807516	-0.09901225	0.921128544	0.956727767
ENSG00000131759	248.1996407	-0.061605642	0.123333823	-0.499503224	0.617424916	0.751491881

ENSG00000131771	62.62824093	0.398173808	0.226407434	1.758660488	0.078635192	0.174142921
ENSG00000131778	76.83471653	0.278122319	0.206403062	1.347471863	0.17782831	0.323295139
ENSG00000131779	42.27299163	0.195660975	0.281867505	0.694159388	0.487582258	0.64779673
ENSG00000131781	14.26594087	-0.406401415	0.499791505	-0.813141902	0.416136705	0.583987529
ENSG00000131788	324.2592575	-0.083570527	0.115262088	-0.725047827	0.46842269	0.630503404
ENSG00000131791	195.2660768	-0.378807947	0.133811057	-2.830916637	0.004641482	0.016829864
ENSG00000131828	759.4973908	-0.02383067	0.077770674	-0.306422315	0.75928312	0.854860493
ENSG00000131844	710.6186975	-0.254064778	0.08207669	-3.09545596	0.001965106	0.008054811
ENSG00000131845	59.32522478	0.266663417	0.245868212	1.084578664	0.278108309	0.446980132
ENSG00000131848	20.21995064	-0.417978628	0.389152445	-1.074074269	0.282789396	0.452173323
ENSG00000131871	307.0247459	0.175990054	0.109910126	1.601217836	0.10932868	0.224970506
ENSG00000131873	655.9319191	-0.09102944	0.084576151	-1.076301512	0.281792433	0.450938517
ENSG00000131876	578.7920205	0.22237286	0.085933882	2.58772041	0.009661336	0.03135693
ENSG00000131899	287.0394269	0.010829901	0.11446564	0.094612683	0.924622476	0.958810774
ENSG00000131914	530.0650647	-2.352799806	0.105585383	-22.28338558	5.36E-110	1.57E-107
ENSG00000131931	124.6671976	0.280042591	0.166083263	1.686157807	0.091765408	0.196288821
ENSG00000131941	148.7401919	0.128340001	0.147788951	0.868400518	0.385175111	0.554189777
ENSG00000131943	102.1872864	0.293049253	0.178031153	1.646056032	0.099752224	0.209324691
ENSG00000131944	31.75491934	0.128612354	0.306195535	0.420033409	0.674461048	0.792816263
ENSG00000131966	159.4719657	0.162006625	0.144443893	1.121588603	0.2620374	0.428353235
ENSG00000131979	16.85308952	-0.093582335	0.424876978	-0.220257487	0.825670628	0.898318899
ENSG00000131981	187.5980335	3.713776827	0.193171582	19.22527521	2.27E-82	4.38E-80
ENSG00000132000	23.80226119	-0.331608009	0.353706451	-0.937523213	0.348489489	0.520494777
ENSG00000132002	794.937355	0.103939363	0.076981792	1.350181141	0.176957886	0.32211059
ENSG00000132003	158.7573272	0.145245761	0.151574929	0.958243961	0.337939755	0.509886115
ENSG00000132004	45.96939764	-0.090675725	0.258491743	-0.350787705	0.725747622	0.830312498
ENSG00000132005	257.0094323	0.250733521	0.126289874	1.985381039	0.047102094	0.116341935
ENSG00000132016	17.19440155	-0.279074134	0.449377103	-0.621024374	0.534583586	0.685592589
ENSG00000132017	283.3511179	0.001374349	0.119445472	0.011506082	0.990819678	0.995236126
ENSG00000132024	325.5131331	0.305071128	0.109376079	2.789194229	0.005283937	0.018752768
ENSG00000132026	39.66469974	-2.405590736	0.332639388	-7.23182768	4.77E-13	9.80E-12
ENSG00000132109	29.70611106	0.774878295	0.318947005	2.42948917	0.015120117	0.045853255
ENSG00000132122	207.8696611	-0.117757956	0.133259604	-0.883673314	0.37687259	0.546505362
ENSG00000132128	668.993451	0.265701807	0.083709873	3.174079703	0.001503124	0.006401916
ENSG00000132153	2007.447423	0.226262033	0.063032045	3.589634958	0.000331141	0.001676232
ENSG00000132155	1045.02236	0.201229361	0.070263954	2.863906007	0.004184522	0.015399522
ENSG00000132170	87.62469653	0.838355738	0.190384652	4.403483828	1.07E-05	7.59E-05
ENSG00000132182	3362.575377	0.070153316	0.065480738	1.071358054	0.284008466	0.453633078
ENSG00000132196	84.88408855	0.425493738	0.201162924	2.115169778	0.03441548	0.090176132
ENSG00000132199	561.022187	-0.16591244	0.105231971	-1.576635298	0.114879466	0.233634264
ENSG00000132204	60.16879532	1.538195667	0.237045698	6.489025871	8.64E-11	1.38E-09
ENSG00000132254	365.5650397	0.334257115	0.106498651	3.138604213	0.001697546	0.00711379
ENSG00000132256	28.95282362	-3.102431618	0.441150833	-7.032587015	2.03E-12	3.92E-11
ENSG00000132275	164.2058392	0.172241779	0.144202275	1.194445641	0.232303692	0.392207928
ENSG00000132286	381.3256947	-0.122717658	0.117464096	-1.044724836	0.296150163	0.466579334
ENSG00000132294	474.2852167	-0.001892394	0.097637877	-0.019381763	0.984536558	0.992018958
ENSG00000132300	717.8914714	-0.16211193	0.082557053	-1.963635129	0.04957242	0.121040116
ENSG00000132305	1024.776706	-0.166335006	0.080887917	-2.056364073	0.039747437	0.101360727
ENSG00000132313	181.7383218	0.072380694	0.13665103	0.529675441	0.596336978	0.73589786
ENSG00000132323	106.6620055	0.467268126	0.176553648	2.646607028	0.008130377	0.027108334

ENSG00000132326	198.3014955	0.186885923	0.132261029	1.413008236	0.157653302	0.295786822
ENSG00000132330	116.191687	-0.273693694	0.166886964	-1.639994444	0.101006322	0.211324964
ENSG00000132334	27.06546864	4.663793193	0.611060357	7.632295461	2.31E-14	5.32E-13
ENSG00000132341	1836.614289	0.155673136	0.073018038	2.131981898	0.033008335	0.087057469
ENSG00000132356	404.6059111	-0.215118386	0.098373893	-2.186742637	0.028761317	0.077843973
ENSG00000132359	235.1157069	-0.260431631	0.122524832	-2.125541627	0.033541444	0.088231586
ENSG00000132361	1206.50715	0.212100372	0.07282971	2.912278149	0.00358803	0.013494258
ENSG00000132376	52.78468262	0.263130447	0.246042649	1.069450553	0.284866698	0.454408643
ENSG00000132382	993.7881761	0.201206449	0.071583018	2.810812586	0.004941656	0.017730637
ENSG00000132383	756.9694736	0.178463853	0.084648114	2.10830277	0.035004809	0.091498427
ENSG00000132386	1624.864992	-0.433674072	0.072994783	-5.941165312	2.83E-09	3.71E-08
ENSG00000132388	405.9339956	-0.003483609	0.103793408	-0.03356291	0.973225699	0.986330341
ENSG00000132394	143.5475824	-0.543015547	0.154802458	-3.50779668	0.000451834	0.00221224
ENSG00000132405	603.4225444	-0.058732485	0.085980047	-0.683094353	0.494547224	0.653641781
ENSG00000132406	36.16410951	0.100099987	0.289520745	0.345743745	0.729535306	0.832882188
ENSG00000132423	46.19969749	0.172568974	0.282250622	0.611403345	0.540932589	0.690770916
ENSG00000132424	1695.350676	0.04528886	0.068469741	0.661443422	0.508327987	0.665159127
ENSG00000132429	103.3403127	-0.158918247	0.178380392	-0.890895268	0.372985359	0.543385744
ENSG00000132432	427.8358443	0.600518095	0.124990951	4.80449258	1.55E-06	1.32E-05
ENSG00000132434	172.3306297	0.233883607	0.142467382	1.641664246	0.100659605	0.210693708
ENSG00000132436	145.372253	-0.058087288	0.148908122	-0.390088109	0.696471395	0.809476559
ENSG00000132463	831.643622	0.356983688	0.080169421	4.452865998	8.47E-06	6.18E-05
ENSG00000132464	11.07314878	-2.949654186	0.685809093	-4.300984361	1.70E-05	0.00011595
ENSG00000132466	2295.19861	-0.003037838	0.063171727	-0.048088565	0.96164566	0.979709645
ENSG00000132467	549.9871359	-0.260553434	0.091340054	-2.85256492	0.004336795	0.015866487
ENSG00000132470	30.05756816	0.62439991	0.335409974	1.861602092	0.062659198	0.145777036
ENSG00000132471	1387.949361	0.414334115	0.076317182	5.429106583	5.66E-08	6.21E-07
ENSG00000132475	1639.280787	-0.54450093	0.072636534	-7.496240547	6.57E-14	1.45E-12
ENSG00000132478	382.0412254	-0.477666005	0.109789858	-4.3507298	1.36E-05	9.47E-05
ENSG00000132485	506.3816184	0.222232954	0.094280837	2.357138116	0.018416396	0.053984789
ENSG00000132507	2853.398399	0.418907693	0.082654061	5.068204631	4.02E-07	3.86E-06
ENSG00000132510	1062.412469	-0.228715901	0.07626489	-2.998967179	0.002708965	0.010567295
ENSG00000132522	27.29003187	-0.158391454	0.345205668	-0.458832135	0.646354716	0.772319358
ENSG00000132530	21.35413825	7.059906775	1.218990204	5.79160255	6.97E-09	8.78E-08
ENSG00000132535	527.8769221	0.080416593	0.092167162	0.872508082	0.382931261	0.55240667
ENSG00000132541	81.07718944	-0.144406569	0.198328134	-0.728119435	0.466540478	0.628880558
ENSG00000132549	1088.24278	0.011415828	0.079045164	0.144421586	0.885167572	0.937067517
ENSG00000132563	345.9134416	0.479665459	0.114414528	4.192347492	2.76E-05	0.000179813
ENSG00000132570	118.1378702	-0.002713203	0.165958785	-0.016348653	0.986956243	0.993178189
ENSG00000132581	75.17394789	-0.18593975	0.207978216	-0.894034738	0.371303305	0.54217406
ENSG00000132589	512.7618184	-0.384140145	0.090536186	-4.242945968	2.21E-05	0.000147003
ENSG00000132591	321.1148582	-0.030950598	0.108962344	-0.284048572	0.776373158	0.865943204
ENSG00000132600	246.6980411	0.012794536	0.123914722	0.103252748	0.917762376	0.955355281
ENSG00000132603	300.0457231	0.300577213	0.113410222	2.650353802	0.008040752	0.026870075
ENSG00000132604	305.6290551	0.181300123	0.115211892	1.573623353	0.115574551	0.234516753
ENSG00000132612	295.9325397	0.222597494	0.113788582	1.956237525	0.05043718	0.122682436
ENSG00000132613	500.9895813	-0.834187423	0.09139723	-9.127053653	7.04E-20	2.36E-18
ENSG00000132622	19.39216866	0.183333044	0.398568798	0.459978416	0.645531713	0.771726296
ENSG00000132635	185.6017224	-0.139136148	0.136112712	-1.022212735	0.306680224	0.47759835
ENSG00000132640	282.9670666	-0.047027907	0.113851099	-0.413065022	0.679558975	0.796741918

ENSG00000132646	534.7227193	-0.304375957	0.090335399	-3.369398485	0.000753324	0.003486638
ENSG00000132661	124.1382817	0.302760041	0.164451491	1.841029471	0.06561724	0.151251292
ENSG00000132664	92.96256933	0.149999763	0.18397509	0.815326482	0.414885454	0.582964486
ENSG00000132669	174.7568068	-0.827710131	0.141348065	-5.855829216	4.75E-09	6.06E-08
ENSG00000132670	582.2215197	-0.165684865	0.087545476	-1.892557702	0.058416713	0.137704241
ENSG00000132676	848.0130404	0.216878722	0.082577604	2.626362497	0.008630285	0.028517906
ENSG00000132680	426.1906181	0.202079093	0.099120486	2.03872176	0.041477805	0.105207426
ENSG00000132688	6208.733138	0.055783968	0.063261903	0.881794016	0.377888208	0.547293088
ENSG00000132692	12.5420267	-1.591419321	0.544948421	-2.920311832	0.003496813	0.013190046
ENSG00000132694	1084.315932	-0.043735858	0.071171152	-0.614516651	0.538873973	0.689521106
ENSG00000132716	623.5404123	-0.080368047	0.083435908	-0.963230925	0.335431649	0.507136839
ENSG00000132718	68.91464018	2.659840222	0.263628339	10.08935622	6.16E-24	2.57E-22
ENSG00000132740	204.2946196	0.882068145	0.134491311	6.558551165	5.43E-11	8.94E-10
ENSG00000132749	69.5343837	0.706478255	0.222180694	3.17974637	0.00147404	0.00630123
ENSG00000132763	166.4008028	0.276321286	0.147439229	1.87413681	0.060911571	0.142604139
ENSG00000132768	134.7207763	0.595147433	0.191654749	3.10531012	0.001900796	0.007834912
ENSG00000132773	208.7826345	0.122966053	0.126203316	0.97434883	0.329883367	0.502255933
ENSG00000132780	3009.602182	-0.123779614	0.058831815	-2.103957081	0.035382191	0.092227433
ENSG00000132781	26.01386759	0.134144578	0.336219215	0.398979512	0.689908301	0.804902039
ENSG00000132792	325.6170684	0.139678671	0.109114921	1.280106055	0.200507839	0.352728068
ENSG00000132793	110.3843952	-0.781912262	0.177815677	-4.397319046	1.10E-05	7.80E-05
ENSG00000132801	11.97128022	-0.269981028	0.499549726	-0.540448756	0.588887591	0.730235169
ENSG00000132819	368.5692502	0.845658595	0.128640152	6.573830832	4.90E-11	8.09E-10
ENSG00000132821	13.01623314	-1.780246744	0.573609281	-3.103587758	0.001911895	0.007872914
ENSG00000132823	63.6244702	-0.019001951	0.217789896	-0.087249	0.930473592	0.962258893
ENSG00000132824	623.1435371	0.176345377	0.086927807	2.028641744	0.042494794	0.107437791
ENSG00000132825	69.15059426	-0.945709835	0.219756982	-4.303434755	1.68E-05	0.000114841
ENSG00000132842	777.7933112	-0.218708822	0.078939325	-2.770594016	0.005595414	0.019674289
ENSG00000132846	344.5555266	-0.10759513	0.111504167	-0.964942677	0.334573524	0.506275374
ENSG00000132849	196.2027136	-0.561815385	0.133670338	-4.202992163	2.63E-05	0.000172476
ENSG00000132879	141.7103403	-0.137403542	0.15379673	-0.893410034	0.371637631	0.542378577
ENSG00000132881	17.31881767	0.167434214	0.410654871	0.407724894	0.683475659	0.799798615
ENSG00000132906	136.1154174	-0.033706107	0.153936312	-0.218961376	0.826680135	0.898668717
ENSG00000132912	465.9111198	0.255224122	0.103281464	2.471151266	0.013467884	0.041525905
ENSG00000132932	17.35851455	-1.801081134	0.467401933	-3.853388287	0.000116494	0.000662241
ENSG00000132950	73.476739	0.351588009	0.211738753	1.660480209	0.096817886	0.204419186
ENSG00000132952	141.6599685	0.246027207	0.153769157	1.599977603	0.109603552	0.225351448
ENSG00000132953	593.9065232	-0.124553933	0.096302355	-1.293363318	0.195885431	0.347068876
ENSG00000132963	557.7552101	0.387230453	0.097061514	3.989536512	6.62E-05	0.000396735
ENSG00000132964	234.7448009	0.03767592	0.126549577	0.297716679	0.765919412	0.858785918
ENSG00000132967	467.8944843	-0.050923874	0.094495651	-0.538901773	0.589954634	0.731235572
ENSG00000132970	595.2388107	0.261903687	0.085126318	3.076647645	0.002093426	0.008481787
ENSG00000133019	19.92603224	-5.781248475	1.04568459	-5.528673301	3.23E-08	3.68E-07
ENSG00000133026	3936.37207	-0.08514942	0.056733389	-1.500869612	0.133389289	0.261750019
ENSG00000133027	238.6069894	0.396886653	0.126480111	3.137937276	0.001701413	0.007127869
ENSG00000133028	506.2805455	0.18826111	0.094604207	1.989986666	0.046592404	0.115408835
ENSG00000133030	1068.632814	-0.070487121	0.069593851	-1.012835468	0.311138756	0.482641219
ENSG00000133056	171.5025412	-0.089958313	0.14632979	-0.614764176	0.538710471	0.689374691
ENSG00000133059	354.7263591	0.084123606	0.103107247	0.815884509	0.414566193	0.582690746
ENSG00000133065	676.0100555	0.001205299	0.080508891	0.014971	0.988055316	0.993928174

ENSG00000133069	50.23419694	-0.975867215	0.258253379	-3.778720029	0.000157637	0.000866323
ENSG00000133083	14.56637654	2.261782096	0.523338756	4.321831832	1.55E-05	0.000106342
ENSG00000133103	86.71190716	0.407402022	0.192706408	2.114107293	0.034506104	0.090346179
ENSG00000133104	15.08269452	-3.060665387	0.606118037	-5.049619383	4.43E-07	4.21E-06
ENSG00000133107	56.48599115	-4.554296804	0.444470499	-10.24656714	1.23E-24	5.33E-23
ENSG00000133110	2821.380381	-2.806201309	0.070271596	-39.93364997	0	0
ENSG00000133111	31.36	-0.353319692	0.316032998	-1.11798355	0.263574019	0.430136762
ENSG00000133112	9819.420527	-0.074931313	0.050673764	-1.478700356	0.139220417	0.269816391
ENSG00000133114	233.4140669	-0.22491163	0.126942618	-1.771758237	0.076434698	0.170176474
ENSG00000133119	247.1285651	-0.111179984	0.132078999	-0.841768828	0.399917364	0.568069492
ENSG00000133121	49.77859032	0.456421233	0.247215357	1.846249517	0.064855984	0.14986544
ENSG00000133131	142.1507804	-0.14301645	0.153174099	-0.933685598	0.350466096	0.522448749
ENSG00000133134	70.03490875	-0.669566222	0.216463445	-3.093206905	0.00198006	0.008106647
ENSG00000133138	68.72649924	0.334652117	0.219062083	1.527658788	0.126597277	0.251742916
ENSG00000133142	1378.141595	0.082182457	0.070165979	1.17125789	0.241495132	0.404089479
ENSG00000133193	508.7996082	0.077136484	0.090354143	0.853712756	0.393264165	0.561290936
ENSG00000133195	63.72047751	-0.23006279	0.246503307	-0.933305085	0.35066247	0.522500564
ENSG00000133216	41.74073485	-0.565432487	0.271502556	-2.082604657	0.037287274	0.096264353
ENSG00000133226	2717.67229	0.025495354	0.060249067	0.423165948	0.672174189	0.791054862
ENSG00000133243	505.6520033	-0.344731667	0.092749282	-3.716812244	0.000201752	0.001078392
ENSG00000133247	118.1085736	0.259071136	0.18853143	1.374153562	0.169394014	0.311985595
ENSG00000133250	65.74970339	0.391470679	0.234342217	1.670508557	0.094818788	0.201317943
ENSG00000133265	116.9446537	0.467898644	0.16606456	2.817570728	0.004838846	0.017397303
ENSG00000133275	950.0278087	0.15240271	0.086256643	1.766851859	0.077253041	0.171681177
ENSG00000133302	123.9257321	-0.22114821	0.160998325	-1.373605658	0.169564139	0.312171358
ENSG00000133313	381.842584	0.395404228	0.102615484	3.853260861	0.000116555	0.000662318
ENSG00000133315	172.0589153	0.067506232	0.147974561	0.456201603	0.648245013	0.77370371
ENSG00000133316	377.1898545	0.434222366	0.113300068	3.832498721	0.000126848	0.000715023
ENSG00000133318	490.8306543	0.272247042	0.094225377	2.889317618	0.003860789	0.014347223
ENSG00000133393	372.5488246	-0.234534842	0.112987884	-2.075752143	0.037916881	0.097513442
ENSG00000133398	74.65414798	0.566691876	0.204828055	2.766671173	0.005663185	0.019857901
ENSG00000133401	63.45203458	1.75064368	0.261180015	6.702824038	2.04E-11	3.49E-10
ENSG00000133422	365.8980145	0.133566969	0.099906071	1.336925447	0.181246973	0.32792069
ENSG00000133433	25.25897651	0.878936773	0.35306999	2.489412294	0.012795448	0.03979138
ENSG00000133460	80.42599941	-0.675792035	0.209682002	-3.222937734	0.001268831	0.005523171
ENSG00000133466	22.40930023	-0.060294646	0.38655822	-0.155978176	0.876050224	0.93127573
ENSG00000133597	95.27409611	0.991649308	0.207504509	4.778928953	1.76E-06	1.48E-05
ENSG00000133606	490.0168143	0.594219027	0.092176834	6.446511554	1.14E-10	1.80E-09
ENSG00000133612	587.7221425	0.814543674	0.092893827	8.768544705	1.81E-18	5.69E-17
ENSG00000133619	136.2835575	0.370660507	0.172414337	2.149824155	0.031569127	0.083875832
ENSG00000133627	102.6570326	0.356034598	0.175640896	2.027059793	0.042656298	0.107701599
ENSG00000133636	23.89596336	1.217299465	0.368696393	3.301631064	0.000961244	0.004336228
ENSG00000133639	356.827149	0.233938446	0.10131045	2.309124535	0.020936672	0.059958701
ENSG00000133640	9.612281573	-0.576689723	0.560463334	-1.02895174	0.303502354	0.474358887
ENSG00000133641	157.7069985	-0.257890952	0.155523873	-1.65820814	0.097275462	0.205082526
ENSG00000133657	707.7786517	-0.119596627	0.085200146	-1.403713879	0.160404068	0.299467828
ENSG00000133678	37.61215424	-0.751694314	0.292478919	-2.570080317	0.010167494	0.0327425
ENSG00000133687	40.48886898	3.821211517	0.41096987	9.298033248	1.43E-20	4.96E-19
ENSG00000133703	184.9031416	0.020211721	0.137503841	0.146990226	0.883139735	0.935555344
ENSG00000133704	223.7040172	0.147423513	0.127714434	1.15432147	0.248368397	0.412635231

ENSG00000133706	1417.608041	0.0745627	0.06474609	1.15161702	0.249478484	0.413686171
ENSG00000133731	188.8656342	-0.251194536	0.132858396	-1.890693722	0.058665241	0.138127831
ENSG00000133739	99.41158011	-0.900152977	0.186328286	-4.831005517	1.36E-06	1.17E-05
ENSG00000133740	124.3584158	-0.136322219	0.168655387	-0.808288557	0.418924489	0.586820133
ENSG00000133773	453.5558513	-0.121104682	0.092320314	-1.311788022	0.189591668	0.338830651
ENSG00000133789	261.1590037	-0.02573704	0.123786886	-0.207914106	0.835296034	0.903681716
ENSG00000133794	12.83000959	0.442572397	0.485569396	0.911450352	0.362058134	0.532826674
ENSG00000133812	461.4841652	-0.091702526	0.110270639	-0.831613266	0.405627272	0.573801237
ENSG00000133816	32.49248546	-0.669678779	0.311126926	-2.152429514	0.031363541	0.083468174
ENSG00000133818	89.22321552	0.731661813	0.190429471	3.842166907	0.000121953	0.000689365
ENSG00000133835	522.6644949	0.034515113	0.08885257	0.388453735	0.697680285	0.81024793
ENSG00000133858	704.4069999	0.290826652	0.083261391	3.492935292	0.000477742	0.00232531
ENSG00000133863	1546.515697	0.07474955	0.06332709	1.180372405	0.237852132	0.399224388
ENSG00000133872	397.0057641	-0.271662343	0.109934243	-2.471134893	0.0134685	0.041525905
ENSG00000133874	24.25113305	-0.280190987	0.353959778	-0.791589905	0.428599835	0.595621477
ENSG00000133884	342.3036034	0.141201849	0.109845349	1.285460433	0.198631435	0.350564914
ENSG00000133895	345.2587742	0.312363741	0.108750415	2.872299312	0.004074968	0.015047602
ENSG00000133935	373.0472249	0.215765888	0.117479	1.836633686	0.066263991	0.152391991
ENSG00000133943	166.5594586	-0.017528675	0.148882916	-0.117734629	0.906277928	0.949823626
ENSG00000133961	307.4351665	-0.003265581	0.107829018	-0.030284807	0.975839914	0.98780011
ENSG00000133983	13.94126712	-0.293176873	0.481186111	-0.609279583	0.542339137	0.691623955
ENSG00000133997	74.2655207	-0.231926902	0.215925548	-1.074105886	0.282775227	0.452173323
ENSG00000134001	576.5732009	0.191774673	0.095637105	2.005232939	0.04493815	0.112141801
ENSG00000134013	298.0903954	-1.050622702	0.115807735	-9.07212889	1.17E-19	3.88E-18
ENSG00000134014	329.340441	-0.037766469	0.112429248	-0.335913204	0.73693632	0.838197934
ENSG00000134030	303.5853786	0.153794882	0.112280515	1.36973795	0.170768718	0.313854269
ENSG00000134046	323.5033347	-0.004680456	0.118826581	-0.039388967	0.968580276	0.983424201
ENSG00000134049	127.692533	0.147557858	0.181237629	0.814167887	0.415548779	0.583340116
ENSG00000134056	87.39526551	-0.188451224	0.204805692	-0.920146422	0.357496248	0.528772223
ENSG00000134057	566.6752431	-0.035169453	0.092532024	-0.380078717	0.703886983	0.814364144
ENSG00000134058	177.276368	0.045360101	0.144819624	0.313217919	0.754115105	0.851326439
ENSG00000134070	76.78702397	0.154872508	0.206439474	0.750207822	0.453129548	0.617396281
ENSG00000134072	323.1370024	0.65626262	0.114330605	5.740043267	9.47E-09	1.16E-07
ENSG00000134077	410.5939956	0.099026483	0.098827453	1.002013921	0.316336869	0.487930603
ENSG00000134086	990.0665979	-0.089196914	0.076967197	-1.158895183	0.246498913	0.410447002
ENSG00000134107	389.2761521	0.032773006	0.104291073	0.314245549	0.75333455	0.850923965
ENSG00000134108	60.87335428	-0.449393119	0.234998935	-1.912319806	0.055835187	0.132980141
ENSG00000134109	210.6269879	0.009785322	0.126873248	0.077126757	0.938522707	0.966102042
ENSG00000134138	1254.930701	-0.464426534	0.068300335	-6.799769531	1.05E-11	1.85E-10
ENSG00000134146	161.0237011	-0.348799325	0.145104578	-2.403778919	0.016226583	0.048662428
ENSG00000134152	95.56930937	-0.28526027	0.182484812	-1.56320006	0.118005546	0.238541359
ENSG00000134153	378.3253617	0.302733307	0.10278008	2.945447276	0.003224881	0.012283054
ENSG00000134184	193.3739822	0.311710595	0.136628946	2.28143893	0.022522487	0.063516035
ENSG00000134186	1040.471638	0.048868357	0.080349593	0.608196691	0.543057029	0.692162425
ENSG00000134202	41.63871712	0.229687195	0.272403504	0.843187373	0.399123659	0.567228859
ENSG00000134222	188.455688	-0.097973722	0.144121268	-0.679800584	0.496630737	0.655162656
ENSG00000134243	163.1994624	-1.610618791	0.154895608	-10.39809207	2.53E-25	1.15E-23
ENSG00000134245	9.462804049	-0.625272617	0.562904384	-1.110797206	0.266655649	0.433380991
ENSG00000134247	803.2165912	0.100626787	0.078153853	1.287547361	0.197903573	0.349415723
ENSG00000134248	448.0147732	0.400216979	0.105064778	3.809240237	0.000139394	0.000776581

ENSG00000134250	1286.84619	0.034784933	0.06969577	0.49909676	0.61771122	0.751514768
ENSG00000134253	36.1856181	-0.128432135	0.288719449	-0.44483368	0.656439953	0.779657734
ENSG00000134255	214.5153347	-0.053502725	0.128010838	-0.417954654	0.675980272	0.794117173
ENSG00000134262	71.06544174	0.367732024	0.217319402	1.692126983	0.090621765	0.194434692
ENSG00000134265	234.1666298	-0.334776791	0.140823305	-2.377282595	0.017440721	0.05170743
ENSG00000134278	420.0912315	0.010875739	0.099249265	0.109580044	0.912742438	0.953043412
ENSG00000134283	507.1404038	0.248851714	0.096288289	2.58444424	0.009753607	0.031597846
ENSG00000134285	55.6938941	-0.586502732	0.271370723	-2.161260161	0.030675249	0.082012793
ENSG00000134287	487.802766	-0.38550625	0.095575817	-4.033512477	5.49E-05	0.000334144
ENSG00000134291	194.5400094	0.253450744	0.133801452	1.894230152	0.058194468	0.137314109
ENSG00000134294	1462.40445	-0.389528041	0.072075631	-5.404434657	6.50E-08	7.07E-07
ENSG00000134297	35.28518534	-0.563119661	0.302889205	-1.859160552	0.063004385	0.146357299
ENSG00000134308	1662.785661	0.115964424	0.072831726	1.592224042	0.111334373	0.228142077
ENSG00000134313	517.3300878	-0.189854255	0.090882179	-2.089015211	0.036706354	0.095096672
ENSG00000134318	672.0976596	-0.317437863	0.083114674	-3.819275804	0.000133844	0.000749345
ENSG00000134324	508.2800085	-0.195397958	0.099076286	-1.972197044	0.048587117	0.119297891
ENSG00000134333	3743.220809	0.239908043	0.079437678	3.020078751	0.00252709	0.009976961
ENSG00000134343	413.9638773	1.002923394	0.105940901	9.466819547	2.88E-21	1.05E-19
ENSG00000134352	339.9724324	0.095934868	0.103848568	0.923795772	0.355592657	0.527122209
ENSG00000134369	432.1568263	-1.925581537	0.117425248	-16.39836045	1.96E-60	2.58E-58
ENSG00000134371	580.03807	-0.042612094	0.086845519	-0.490665435	0.623663097	0.755746988
ENSG00000134375	489.4209803	0.14016984	0.101692961	1.378363256	0.168091162	0.310367177
ENSG00000134419	484.1984431	0.103738959	0.221826838	0.467657384	0.640029621	0.767565619
ENSG00000134440	1493.947949	0.10246906	0.070574821	1.451920943	0.146523588	0.280143847
ENSG00000134444	184.3758931	-0.008839493	0.134042755	-0.065945327	0.947421353	0.970914655
ENSG00000134452	479.5086287	-0.008102437	0.100392794	-0.080707354	0.935674688	0.964731953
ENSG00000134453	895.0596665	0.148621262	0.076052704	1.954187743	0.050679018	0.123142738
ENSG00000134461	132.8173973	0.412867299	0.15885085	2.59908775	0.009347188	0.030513582
ENSG00000134470	57.60667614	0.927532351	0.242806046	3.820054589	0.000133422	0.000747281
ENSG00000134480	159.9750668	-0.131401384	0.155573111	-0.844627866	0.398318646	0.566485979
ENSG00000134490	84.84375109	-0.208691876	0.191317832	-1.090812463	0.275355411	0.443721173
ENSG00000134504	106.510227	0.466734741	0.177820511	2.624751994	0.00867121	0.028626202
ENSG00000134508	23.78148542	-4.706662127	0.712064625	-6.609880565	3.85E-11	6.41E-10
ENSG00000134531	2281.332158	-1.50368911	0.078638515	-19.12153484	1.67E-81	3.17E-79
ENSG00000134533	9.439823461	-5.53358099	1.260579065	-4.389713539	1.14E-05	8.05E-05
ENSG00000134569	445.5600752	0.031770534	0.096213835	0.330207547	0.741243145	0.841937613
ENSG00000134574	205.4232059	-0.216790609	0.130198746	-1.665074473	0.095897899	0.203097048
ENSG00000134575	155.033214	-0.324330763	0.148746256	-2.180429752	0.029225621	0.078781864
ENSG00000134588	5.743718977	-5.802563716	1.313225533	-4.418558403	9.94E-06	7.13E-05
ENSG00000134590	222.7121235	0.215670229	0.125835449	1.713906784	0.086545847	0.187638449
ENSG00000134597	165.9613234	0.001940276	0.146885975	0.013209401	0.989460729	0.994744093
ENSG00000134602	394.08983	0.343518641	0.105792134	3.247109482	0.001165835	0.005132087
ENSG00000134644	1040.164009	-0.181609031	0.073759359	-2.462182863	0.013809424	0.042381686
ENSG00000134668	49.85208225	-0.607055205	0.257926225	-2.353600168	0.018592598	0.054376477
ENSG00000134684	1621.551232	0.62205199	0.066323971	9.378991908	6.66E-21	2.37E-19
ENSG00000134686	425.7987797	0.271714095	0.096572608	2.813573128	0.004899424	0.017588094
ENSG00000134690	350.3413675	-0.120389762	0.107286105	-1.122137501	0.261803981	0.428099137
ENSG00000134697	713.548732	0.047275325	0.08605009	0.54939309	0.582735715	0.724910431
ENSG00000134698	122.0013555	-0.159369781	0.164996262	-0.965899346	0.33409455	0.505990311
ENSG00000134717	225.4249324	-0.137965194	0.12913296	-1.068396437	0.285341724	0.454959727

ENSG00000134744	922.2854733	-0.284184305	0.078394353	-3.625060903	0.000288893	0.001489206
ENSG00000134748	414.8753841	-0.158070194	0.10636831	-1.486064725	0.137262002	0.266941622
ENSG00000134758	129.320019	-0.25970618	0.16089265	-1.614158139	0.106493129	0.220343053
ENSG00000134759	219.425331	-0.174293127	0.127290038	-1.369259763	0.170918091	0.313964485
ENSG00000134769	6.496527107	-5.979302076	1.304270718	-4.584402604	4.55E-06	3.52E-05
ENSG00000134775	31.46923985	-0.609043152	0.324346897	-1.87775236	0.06041505	0.141630542
ENSG00000134779	509.7283632	-0.064843994	0.093824343	-0.691121214	0.48948937	0.6491557
ENSG00000134780	47.46138397	0.489478281	0.254430039	1.92382269	0.054376812	0.130279905
ENSG00000134809	307.9189593	0.636710952	0.121170316	5.25467764	1.48E-07	1.53E-06
ENSG00000134815	361.3818431	0.625173246	0.103416389	6.045204732	1.49E-09	2.04E-08
ENSG00000134824	2242.315739	-0.230617658	0.075117905	-3.070075731	0.002140045	0.008633318
ENSG00000134825	302.0595045	0.56496354	0.123151786	4.587538323	4.49E-06	3.47E-05
ENSG00000134851	353.7833102	0.011185156	0.10263649	0.108978357	0.913219657	0.953329111
ENSG00000134852	622.9167163	-0.057029005	0.100310982	-0.568522051	0.569680537	0.714810407
ENSG00000134853	67.76565906	-7.560090284	1.011504605	-7.47410367	7.77E-14	1.71E-12
ENSG00000134864	33.96902668	-0.870039021	0.309050927	-2.815196285	0.004874745	0.017517407
ENSG00000134871	7.172319222	-6.125063402	1.305807044	-4.690634373	2.72E-06	2.21E-05
ENSG00000134874	1062.677943	-0.161240933	0.074696588	-2.158611766	0.030880299	0.082451162
ENSG00000134882	166.6623703	-0.012893972	0.148928895	-0.086578044	0.931006919	0.962597835
ENSG00000134884	773.3675047	0.009661169	0.079688235	0.12123708	0.903503255	0.948047376
ENSG00000134897	109.069408	0.216983234	0.179805291	1.206767791	0.227521583	0.386083074
ENSG00000134899	53.86670189	0.49619567	0.240548403	2.062768512	0.039134627	0.100058993
ENSG00000134900	734.2254068	0.010832822	0.079199246	0.136779364	0.891205195	0.94027635
ENSG00000134901	115.1330492	-0.311267589	0.170837984	-1.822004577	0.068454291	0.156303223
ENSG00000134905	694.2444398	0.259041744	0.088286075	2.934117793	0.003344972	0.012685428
ENSG00000134909	169.4126324	-0.237440716	0.144203607	-1.646565716	0.099647342	0.209167098
ENSG00000134910	1562.392279	0.49693177	0.06507019	7.636857536	2.23E-14	5.14E-13
ENSG00000134970	235.6681995	0.150635415	0.125975612	1.195750616	0.231793893	0.391488375
ENSG00000134982	542.0519265	-0.257189402	0.08791913	-2.925295115	0.003441297	0.013005084
ENSG00000134986	213.7395715	-0.331816865	0.14294931	-2.321220469	0.020274948	0.05844494
ENSG00000134987	428.9778561	0.139379261	0.100365627	1.38871509	0.164919404	0.30606723
ENSG00000134996	77.27067555	0.463695157	0.199300323	2.326615178	0.019985756	0.057736276
ENSG00000135002	67.78348117	0.049401133	0.230903824	0.213946792	0.83058855	0.90129605
ENSG00000135018	1013.026053	0.301539331	0.073729605	4.089799897	4.32E-05	0.000269317
ENSG00000135040	318.96272	-0.021525691	0.108264997	-0.198824108	0.842400337	0.908568607
ENSG00000135045	89.80649158	-0.090773485	0.188905063	-0.480524362	0.630854584	0.760980193
ENSG00000135047	539.7442051	0.596095215	0.09786674	6.090886633	1.12E-09	1.55E-08
ENSG00000135048	21.45840605	-0.250786118	0.373878989	-0.670768152	0.502368239	0.660436748
ENSG00000135049	135.2875754	-0.266258558	0.15552804	-1.711964981	0.086903122	0.188192695
ENSG00000135052	2461.864035	0.666168398	0.065280429	10.20471851	1.89E-24	8.06E-23
ENSG00000135069	864.4424726	-0.186302499	0.078938271	-2.360103608	0.018269832	0.053660482
ENSG00000135070	176.3587363	0.38671866	0.147093689	2.629063575	0.008562035	0.028359092
ENSG00000135074	59.71092364	-0.967151836	0.236627203	-4.087238599	4.37E-05	0.000272173
ENSG00000135090	303.4252859	0.029414961	0.113713691	0.258675629	0.795885525	0.878339214
ENSG00000135093	66.19897992	-0.038649358	0.254178013	-0.15205626	0.879142562	0.933715074
ENSG00000135097	27.88706786	-3.091187659	0.79248369	-3.900632527	9.59E-05	0.000554606
ENSG00000135108	257.6599132	-0.018900343	0.118806427	-0.159085186	0.87360176	0.930010149
ENSG00000135119	219.7363837	0.300421765	0.133215233	2.255160758	0.02412324	0.06726952
ENSG00000135124	86.17861352	0.292214214	0.190668776	1.532575077	0.125380586	0.249995897
ENSG00000135127	75.22718377	0.305795544	0.224499717	1.362119953	0.173160032	0.316881055

ENSG00000135148	271.1558592	-0.110856367	0.11676308	-0.949412836	0.342410685	0.514366687
ENSG00000135164	369.2704989	-0.118350653	0.106751026	-1.10866057	0.267576638	0.434425455
ENSG00000135185	72.59821211	-0.126913995	0.218080337	-0.581959827	0.560593741	0.707146494
ENSG00000135205	11.83080202	-0.856073663	0.507592109	-1.686538555	0.091692115	0.196187994
ENSG00000135211	16.98762891	-0.088895494	0.440427027	-0.201839325	0.840042337	0.906960118
ENSG00000135218	10.96509728	-0.266949388	0.533143556	-0.500708271	0.61657645	0.750779831
ENSG00000135241	91.98254931	0.599761725	0.185229995	3.237929831	0.001204004	0.005270379
ENSG00000135245	48.95870293	1.137822818	0.259663256	4.38191693	1.18E-05	8.31E-05
ENSG00000135249	164.6603586	0.725662412	0.142218061	5.102463141	3.35E-07	3.26E-06
ENSG00000135250	680.5522861	0.631805401	0.084065566	7.515626551	5.66E-14	1.26E-12
ENSG00000135253	22.87150777	1.503089111	0.384526668	3.908933335	9.27E-05	0.000537663
ENSG00000135269	41.9543358	-7.703376077	1.199379785	-6.422799663	1.34E-10	2.08E-09
ENSG00000135272	87.61600191	0.476010272	0.194800399	2.443579548	0.014542363	0.044311622
ENSG00000135297	219.3153311	0.059406852	0.124872723	0.475739224	0.634260173	0.763514667
ENSG00000135299	36.17406854	-0.804747091	0.292600148	-2.750330431	0.00595352	0.020710523
ENSG00000135315	37.79636072	0.022454532	0.289436777	0.077580094	0.938162077	0.966102042
ENSG00000135316	696.4911404	-0.136042455	0.08527725	-1.595295986	0.110646063	0.226963382
ENSG00000135317	206.6976597	-0.267068721	0.129282923	-2.065769519	0.038850248	0.099513025
ENSG00000135324	19.81327247	1.301765994	0.413930904	3.144887182	0.001661509	0.006977344
ENSG00000135333	197.8117712	-5.633873415	0.328510408	-17.14975622	6.31E-66	9.64E-64
ENSG00000135334	238.0969875	0.0193613	0.119684877	0.161768978	0.871487783	0.928463321
ENSG00000135336	238.8811935	0.210091778	0.122592056	1.71374708	0.086575186	0.187644135
ENSG00000135338	429.0441718	-1.809406996	0.106133238	-17.04844806	3.59E-65	5.31E-63
ENSG00000135341	272.3661416	-0.028809546	0.114373065	-0.251891004	0.801125313	0.881375883
ENSG00000135362	10.91384549	-0.495538083	0.521055122	-0.951028139	0.341590092	0.513573373
ENSG00000135365	1156.260997	0.019970425	0.069369522	0.28788472	0.773434983	0.864315132
ENSG00000135372	999.5687606	0.099727122	0.07670485	1.300141015	0.193552642	0.344374157
ENSG00000135378	19.13943949	2.547632066	0.471691663	5.401053833	6.63E-08	7.19E-07
ENSG00000135387	2640.586235	0.031027865	0.062361233	0.497550539	0.618800873	0.752449434
ENSG00000135390	1948.395016	0.335691668	0.062173534	5.399269551	6.69E-08	7.25E-07
ENSG00000135392	274.3533173	0.475166417	0.121025755	3.926159497	8.63E-05	0.000504342
ENSG00000135404	1676.38471	0.378720206	0.076831196	4.929250445	8.25E-07	7.46E-06
ENSG00000135407	42.30588627	-0.494016891	0.294848995	-1.675491181	0.0938379	0.199616702
ENSG00000135414	285.733906	-0.467470124	0.115275316	-4.055249111	5.01E-05	0.000308411
ENSG00000135423	50.51136646	-0.262953574	0.270642343	-0.971590665	0.331254228	0.503748852
ENSG00000135424	161.9624013	0.302310855	0.146786981	2.059520899	0.039444365	0.100685989
ENSG00000135436	8.248599253	-1.267329821	0.633747971	-1.999737875	0.045528576	0.113293164
ENSG00000135439	499.4274044	-0.31220837	0.102389058	-3.049235683	0.002294244	0.009186787
ENSG00000135441	9.688977013	-0.530316381	0.590099957	-0.898689069	0.368818301	0.540172107
ENSG00000135446	767.9421331	0.253259371	0.0785285	3.225063143	0.001259449	0.005490893
ENSG00000135451	379.5542787	-0.311052618	0.106976814	-2.907663883	0.003641395	0.013651101
ENSG00000135452	71.22772425	-0.351003048	0.213357311	-1.645141883	0.099940555	0.209625949
ENSG00000135454	320.0552234	0.917917402	0.126162665	7.275665916	3.45E-13	7.17E-12
ENSG00000135457	402.417652	0.466061948	0.098963501	4.70943268	2.48E-06	2.03E-05
ENSG00000135469	96.86284464	-0.971703608	0.18948131	-5.128229306	2.92E-07	2.86E-06
ENSG00000135473	88.03096688	-0.574591627	0.195593768	-2.937678604	0.003306796	0.012557602
ENSG00000135476	477.8998522	-0.187885304	0.098994403	-1.897938647	0.057704165	0.136321964
ENSG00000135482	73.13198142	0.630254977	0.230857854	2.730056464	0.006332348	0.021826914
ENSG00000135486	2330.226522	0.049612651	0.060835133	0.815526295	0.41477112	0.582882566
ENSG00000135503	88.55238663	-0.163544433	0.187611805	-0.871717175	0.383362688	0.552575821

ENSG00000135506	885.6362911	0.214138194	0.075176415	2.848475716	0.004392921	0.016044554
ENSG00000135519	38.83109485	0.162921406	0.284246803	0.573168827	0.566530389	0.712129967
ENSG00000135521	305.1245388	0.239015584	0.114388961	2.0894987	0.036662855	0.095001498
ENSG00000135525	34.63358151	1.662700252	0.323270198	5.143376227	2.70E-07	2.65E-06
ENSG00000135535	723.376791	0.134319851	0.082938815	1.619505316	0.105338584	0.218402811
ENSG00000135537	40.7697878	-0.302707515	0.270510299	-1.119023993	0.263129904	0.429689114
ENSG00000135540	60.9224287	-0.441270578	0.230786814	-1.912026816	0.055872755	0.133047056
ENSG00000135541	414.9068608	-0.013322352	0.096452592	-0.138123317	0.890142958	0.939855542
ENSG00000135587	81.22973159	0.233986354	0.210460343	1.111783586	0.266231211	0.433092046
ENSG00000135596	37.23831345	-0.548718597	0.302610761	-1.81328184	0.069788363	0.15867872
ENSG00000135597	319.8456079	-0.018324642	0.112207834	-0.163309827	0.870274497	0.927874449
ENSG00000135605	59.49383565	-0.587554206	0.234490932	-2.505658534	0.012222359	0.038263312
ENSG00000135617	54.70343112	0.456210586	0.241959719	1.885481551	0.059364853	0.139587896
ENSG00000135622	41.22337836	0.080842518	0.284056818	0.284599814	0.775950754	0.865699624
ENSG00000135624	1799.111234	0.314757818	0.063464323	4.959602565	7.06E-07	6.47E-06
ENSG00000135625	14.21549547	0.009921121	0.459039084	0.021612802	0.982756822	0.991509378
ENSG00000135631	273.3309859	0.613667714	0.114019508	5.382129117	7.36E-08	7.89E-07
ENSG00000135632	146.7086147	0.147332735	0.158353753	0.930402547	0.3521627	0.523715899
ENSG00000135637	54.84767556	0.153764834	0.234339066	0.656163892	0.511718679	0.667791925
ENSG00000135643	12.15089856	-0.070385746	0.526206477	-0.133760699	0.893591805	0.94236367
ENSG00000135655	211.94954	-0.290408877	0.129055406	-2.250265105	0.024432122	0.067986928
ENSG00000135677	228.8493647	0.008566692	0.124686623	0.068705785	0.945223814	0.969665557
ENSG00000135679	928.0625035	0.044627156	0.076668472	0.582079641	0.560513038	0.707146494
ENSG00000135686	232.8142948	0.096998703	0.125008595	0.775936273	0.437786614	0.603314879
ENSG00000135698	94.38896528	0.090679885	0.185873987	0.487856782	0.625651288	0.757101486
ENSG00000135709	40.40362881	0.933762176	0.282025551	3.310913403	0.00092992	0.004213737
ENSG00000135720	784.2831364	0.046062662	0.079181088	0.581738171	0.560743056	0.707146494
ENSG00000135722	14.55638523	0.80862851	0.473981647	1.706033379	0.088001865	0.189886667
ENSG00000135723	583.44364	0.228909901	0.08592793	2.663975511	0.007722318	0.025957264
ENSG00000135736	137.5466998	-0.254317053	0.154642627	-1.644546898	0.100063285	0.209789398
ENSG00000135740	55.97054384	0.384439795	0.242283696	1.586734072	0.112572886	0.230008921
ENSG00000135747	13.96763711	-0.34914161	0.473339978	-0.737612765	0.460749796	0.624062519
ENSG00000135763	233.8500555	0.0100935	0.120057769	0.084072028	0.932999164	0.963270344
ENSG00000135766	213.5449709	0.592425601	0.128248669	4.619350863	3.85E-06	3.02E-05
ENSG00000135775	321.2193941	-0.23923212	0.121425199	-1.970201584	0.048815272	0.119690719
ENSG00000135776	194.8920051	0.126363818	0.143041346	0.883407639	0.377016065	0.546593525
ENSG00000135778	162.337052	0.029755535	0.150701436	0.197446924	0.843477813	0.909242025
ENSG00000135801	374.1601257	0.030457074	0.101655856	0.299609639	0.764474931	0.858193827
ENSG00000135821	711.3187743	-0.427551433	0.080394774	-5.31814959	1.05E-07	1.10E-06
ENSG00000135823	539.2886992	-0.004134621	0.088892037	-0.046512834	0.962901505	0.980491297
ENSG00000135828	37.144588	-0.611876737	0.304180776	-2.011556234	0.044266737	0.110800936
ENSG00000135829	2941.97509	0.092109457	0.060786646	1.515290986	0.12969873	0.25624164
ENSG00000135835	43.47163222	1.011981674	0.295400672	3.425793408	0.000613006	0.002906143
ENSG00000135837	1203.587732	-0.635972612	0.074196672	-8.571443922	1.02E-17	2.97E-16
ENSG00000135838	60.24453292	-0.13063667	0.251703144	-0.51901088	0.603753154	0.741439589
ENSG00000135842	106.1243595	0.546560017	0.174479156	3.132523275	0.001733107	0.007239058
ENSG00000135845	145.6381697	-0.677941586	0.158243562	-4.284165346	1.83E-05	0.000124054
ENSG00000135862	2923.658254	0.015959589	0.057135071	0.279330868	0.77999092	0.868394424
ENSG00000135870	626.3566924	-0.207651607	0.087206219	-2.381155961	0.017258403	0.051231782
ENSG00000135899	48.07737212	1.662588634	0.275199568	6.041392592	1.53E-09	2.09E-08

ENSG00000135900	162.4933937	-0.217691152	0.145541967	-1.49572771	0.134724626	0.263466076
ENSG00000135912	627.4455891	0.060005046	0.090549164	0.662679183	0.507536045	0.664432322
ENSG00000135913	223.5594958	-0.277447159	0.12478716	-2.223363038	0.02619133	0.072153287
ENSG00000135914	44.2901685	-6.361501741	0.866162898	-7.344463441	2.07E-13	4.38E-12
ENSG00000135916	548.005018	0.17099077	0.10192996	1.677532004	0.093438497	0.198988866
ENSG00000135917	29.95388091	0.296229514	0.317621974	0.932648047	0.351001717	0.522671567
ENSG00000135924	199.4764863	0.3293209	0.141485578	2.327593422	0.019933703	0.057614964
ENSG00000135926	233.1071581	0.613042744	0.135775698	4.515113917	6.33E-06	4.74E-05
ENSG00000135930	400.8355555	-0.197080357	0.102860569	-1.915995199	0.055365707	0.132161437
ENSG00000135931	724.5011523	-2.135630563	0.09409248	-22.69714387	4.78E-114	1.49E-111
ENSG00000135932	262.3505988	-0.078969748	0.121263091	-0.651226582	0.514900233	0.670697661
ENSG00000135940	1065.237209	0.230680664	0.077892752	2.961516424	0.003061282	0.011742715
ENSG00000135945	232.0278844	-0.030432131	0.125628845	-0.242238407	0.808595437	0.886947088
ENSG00000135951	49.12890892	-0.143811771	0.254781984	-0.564450313	0.572447706	0.717385398
ENSG00000135953	97.22775001	0.255961191	0.179355679	1.427115059	0.153546732	0.290139904
ENSG00000135956	197.0338847	-0.0396274	0.142020001	-0.279026895	0.780224185	0.868470711
ENSG00000135966	354.8267155	0.241246944	0.10292492	2.343911902	0.019082678	0.055532294
ENSG00000135968	336.4037664	-0.183191122	0.108302276	-1.691479888	0.090745186	0.194610324
ENSG00000135972	215.5477222	-0.030634156	0.13898321	-0.220416235	0.825547002	0.898253969
ENSG00000135974	147.7785844	-0.074969476	0.15072438	-0.49739449	0.61891089	0.752518071
ENSG00000135976	383.3310503	-0.319254597	0.103920594	-3.07210136	0.002125575	0.008581483
ENSG00000135999	263.3532704	-0.095046434	0.115310295	-0.824266686	0.409788043	0.578173226
ENSG00000136002	115.1570145	-0.214252993	0.165657253	-1.293351115	0.195889649	0.347068876
ENSG00000136003	234.7127807	-0.035626337	0.122946109	-0.289771978	0.771990687	0.86337203
ENSG00000136010	674.7900497	-0.449246129	0.094690511	-4.744362687	2.09E-06	1.74E-05
ENSG00000136021	286.9972085	0.002889248	0.116021955	0.024902597	0.980132656	0.989928345
ENSG00000136026	1609.321487	0.761189135	0.066400132	11.46366909	2.01E-30	1.11E-28
ENSG00000136044	92.26965698	-0.574971396	0.193244258	-2.975360835	0.002926441	0.011297601
ENSG00000136045	350.7976344	0.157268711	0.102209969	1.538682703	0.123881777	0.247993063
ENSG00000136048	61.40611503	0.057439135	0.223670202	0.256802801	0.797331007	0.879045586
ENSG00000136051	311.4157787	0.039374942	0.113817123	0.345949192	0.729380899	0.832773591
ENSG00000136052	57.9089206	0.512166789	0.234349851	2.185479476	0.028853709	0.078018875
ENSG00000136068	969.9213411	-0.16922399	0.074694355	-2.26555259	0.023478791	0.065777138
ENSG00000136098	117.965407	-0.046055522	0.175401434	-0.262572095	0.792880411	0.876423602
ENSG00000136100	367.7284604	-0.295417508	0.105454382	-2.80137727	0.005088499	0.018164714
ENSG00000136104	232.6939466	-0.353847895	0.129911237	-2.723766654	0.006454209	0.022210258
ENSG00000136108	862.651338	-0.200153162	0.076386542	-2.62026734	0.008786086	0.028944219
ENSG00000136111	411.4947548	-0.114168107	0.101845535	-1.120992759	0.262290944	0.428598579
ENSG00000136114	18.60813586	-0.192682908	0.396253884	-0.486261247	0.626781952	0.757759366
ENSG00000136122	88.87096079	-0.227485295	0.188803632	-1.204877748	0.2282505	0.38696065
ENSG00000136141	189.2316936	0.265634969	0.134819515	1.970300582	0.048803932	0.119690719
ENSG00000136143	178.3656648	-0.17276215	0.135650175	-1.273585896	0.202810213	0.355620405
ENSG00000136144	157.7673298	0.064964999	0.148033187	0.438854286	0.660767123	0.783076162
ENSG00000136146	477.0557142	0.000328843	0.091599643	0.003590007	0.997135595	0.998813347
ENSG00000136147	61.54458304	0.240976065	0.221396498	1.088436661	0.276402384	0.444897936
ENSG00000136152	141.7172634	0.194700936	0.154457909	1.26054365	0.207473312	0.361480964
ENSG00000136153	119.8603244	-0.677134935	0.172308399	-3.929784837	8.50E-05	0.000498873
ENSG00000136156	1231.830148	0.245890517	0.079448706	3.094959368	0.001968399	0.008065955
ENSG00000136158	2174.216414	-0.04600536	0.06591057	-0.69799669	0.485179264	0.645572902
ENSG00000136159	163.0695569	-0.101240539	0.146655011	-0.690331263	0.489985892	0.649630187

ENSG00000136161	48.68746994	-0.181960254	0.250508426	-0.726363806	0.467615773	0.629756504
ENSG00000136169	114.4504394	-0.056789744	0.173375944	-0.327552614	0.743249955	0.842922019
ENSG00000136193	1284.294682	-0.077492478	0.066794888	-1.16015582	0.245985371	0.409693681
ENSG00000136205	33.75978315	-2.226455212	0.3534746	-6.29877002	3.00E-10	4.46E-09
ENSG00000136213	174.3752317	-0.255885212	0.140468035	-1.8216615	0.068506363	0.156396698
ENSG00000136231	590.8197223	-0.049651973	0.084779753	-0.585658385	0.558105105	0.705122829
ENSG00000136238	711.2095453	0.187046918	0.093775517	1.994624226	0.046083879	0.11433067
ENSG00000136240	877.5346706	-0.079471769	0.086687754	-0.916758891	0.359268998	0.530567328
ENSG00000136243	147.5254717	-0.705785847	0.165528663	-4.263828594	2.01E-05	0.000135062
ENSG00000136247	163.0083303	-0.292164754	0.160838626	-1.816508643	0.069292383	0.157798757
ENSG00000136261	349.3924174	0.124797568	0.107595728	1.159874757	0.246099801	0.409835674
ENSG00000136267	122.3004938	-4.457757543	0.296896198	-15.01453227	5.90E-51	6.42E-49
ENSG00000136270	478.6815471	0.067935035	0.09887611	0.687072285	0.492037178	0.651427544
ENSG00000136271	809.2737928	0.192746127	0.084826499	2.27223957	0.023072044	0.064855205
ENSG00000136273	87.07234264	-0.099318905	0.193601142	-0.513007846	0.607945832	0.744534883
ENSG00000136279	525.3569367	0.060197702	0.089297204	0.674127508	0.500230252	0.658427132
ENSG00000136280	202.4432557	0.1476566	0.132409516	1.115150962	0.264785736	0.431540229
ENSG00000136295	687.0285883	-0.042516513	0.098198258	-0.432966061	0.665039426	0.785752277
ENSG00000136319	82.10778911	0.111143844	0.194263981	0.572127904	0.567235326	0.712697194
ENSG00000136367	209.8685264	-0.363136979	0.127092136	-2.857273393	0.004272976	0.015663491
ENSG00000136371	39.40274399	-0.826701067	0.301135954	-2.745275203	0.006046017	0.020975156
ENSG00000136378	43.05672715	-1.979716862	0.307144921	-6.445546463	1.15E-10	1.81E-09
ENSG00000136379	191.4672637	0.30832411	0.131169309	2.350581186	0.018744118	0.054774002
ENSG00000136381	307.4780099	-0.393724411	0.110520344	-3.562460964	0.000367395	0.001842482
ENSG00000136383	13.11688081	-0.126346161	0.4846042	-0.260720317	0.794308197	0.877449453
ENSG00000136425	189.8619699	-0.412412572	0.136001629	-3.032409066	0.002426102	0.009634833
ENSG00000136436	352.4605079	-0.157877218	0.108441668	-1.455872269	0.145427933	0.278617863
ENSG00000136444	320.1334227	0.409618781	0.111018372	3.689648602	0.000224564	0.001186776
ENSG00000136448	825.5034002	0.202008071	0.076852829	2.628505324	0.008576101	0.028392292
ENSG00000136450	983.8523115	0.120655109	0.076858958	1.569824949	0.116455842	0.235986037
ENSG00000136451	299.2955273	-0.407408801	0.113873323	-3.577736995	0.000346582	0.00174872
ENSG00000136463	70.92226388	-0.103818347	0.21958026	-0.472803645	0.636353274	0.765247362
ENSG00000136478	627.1492642	-0.219811989	0.086729001	-2.534469271	0.01126178	0.035661458
ENSG00000136485	1121.828077	-0.15867034	0.0723936	-2.191773035	0.028395899	0.077033062
ENSG00000136490	126.7251919	-0.179642724	0.165282633	-1.086882028	0.27708895	0.445696608
ENSG00000136492	566.5447352	-0.652273783	0.089260241	-7.307551202	2.72E-13	5.72E-12
ENSG00000136504	613.2881421	-0.027244933	0.086642356	-0.314452819	0.753177145	0.850814585
ENSG00000136518	341.748716	0.150910712	0.10727291	1.406792377	0.159488964	0.298195324
ENSG00000136521	74.02630301	-0.097887331	0.219136456	-0.446695785	0.655094725	0.778784714
ENSG00000136522	274.7317617	0.363371968	0.116985211	3.10613594	0.001895496	0.00782428
ENSG00000136527	1273.509442	-0.023267534	0.067155312	-0.346473464	0.728986923	0.832662165
ENSG00000136535	5.77349964	-3.941544468	1.15811483	-3.403414207	0.000665493	0.003124392
ENSG00000136536	611.8913922	-0.119940676	0.091606688	-1.309300434	0.190432603	0.340115292
ENSG00000136560	191.9089964	-0.605315178	0.136991879	-4.418620885	9.93E-06	7.13E-05
ENSG00000136603	468.4548776	-0.672025644	0.097063883	-6.923539645	4.40E-12	8.14E-11
ENSG00000136628	1532.543179	0.334981955	0.071026462	4.716297948	2.40E-06	1.97E-05
ENSG00000136631	269.9913653	-0.077877258	0.119055857	-0.6541237	0.513032114	0.668946635
ENSG00000136636	560.99649	0.0642552	0.105439444	0.609403822	0.542256805	0.691581745
ENSG00000136643	185.6820255	0.17393532	0.135967663	1.279240342	0.200812434	0.353175442
ENSG00000136682	36.52100725	-0.162196526	0.318779446	-0.508804843	0.610889024	0.746967107

ENSG00000136699	721.4116446	0.085850376	0.081806665	1.049430089	0.293980216	0.464358495
ENSG00000136709	776.8435166	-0.115831487	0.082341898	-1.406713836	0.159512261	0.298199193
ENSG00000136710	142.432389	0.220605371	0.159285943	1.384964466	0.166063333	0.307783565
ENSG00000136715	509.8408133	-0.085840198	0.089814188	-0.955753213	0.339196931	0.511070291
ENSG00000136717	278.3510525	0.626885509	0.115289391	5.437495178	5.40E-08	5.96E-07
ENSG00000136718	620.5711946	0.364266485	0.083422325	4.366534815	1.26E-05	8.84E-05
ENSG00000136720	69.73926051	-0.151776556	0.213778439	-0.709971299	0.477721934	0.638134946
ENSG00000136731	1024.305807	-0.041684901	0.073363276	-0.568198458	0.569900219	0.715022184
ENSG00000136738	203.3907732	-0.225785002	0.132915406	-1.698712055	0.089373451	0.192285015
ENSG00000136754	365.5842847	0.116896816	0.102418873	1.141360116	0.253720095	0.418654548
ENSG00000136758	844.4185104	0.123624598	0.076111553	1.624255348	0.104321323	0.216679228
ENSG00000136770	418.5216192	-0.343787217	0.097532856	-3.524834908	0.000423747	0.002090018
ENSG00000136783	14.03619393	-0.554730828	0.463787723	-1.196087782	0.231662305	0.39131318
ENSG00000136802	284.1111674	0.08097371	0.112714816	0.718394557	0.472514042	0.633981332
ENSG00000136807	263.3689978	0.021723625	0.116283383	0.186816242	0.851804711	0.914988207
ENSG00000136810	487.2646323	0.493805818	0.107184884	4.607047161	4.08E-06	3.18E-05
ENSG00000136811	1034.987343	-0.119444245	0.073423212	-1.626791329	0.103781424	0.215867235
ENSG00000136813	1683.986454	0.27137419	0.066981192	4.051498357	5.09E-05	0.000312439
ENSG00000136816	164.5577747	0.23883426	0.142354207	1.677746415	0.093396614	0.198929832
ENSG00000136819	966.2093428	0.440779204	0.081762978	5.390938718	7.01E-08	7.54E-07
ENSG00000136824	682.4398295	0.201574741	0.080209578	2.513100619	0.01196752	0.037574374
ENSG00000136827	280.0736072	0.01387406	0.117609884	0.117966787	0.906093974	0.949742267
ENSG00000136828	49.02452038	-0.770060445	0.258324917	-2.980976256	0.002873311	0.011128038
ENSG00000136830	232.5203034	0.241461302	0.124007272	1.947154376	0.05151623	0.124730741
ENSG00000136840	226.2972221	0.483529512	0.131128128	3.687458355	0.000226505	0.001195236
ENSG00000136848	345.3740828	-0.944191113	0.11165109	-8.456622422	2.75E-17	7.72E-16
ENSG00000136854	922.8731349	0.079034816	0.075146532	1.051742693	0.292917618	0.463541004
ENSG00000136856	41.19949176	-0.385074482	0.277641532	-1.386948411	0.165457494	0.306903661
ENSG00000136859	145.1278191	-0.474546903	0.152731298	-3.107070438	0.001889514	0.007804175
ENSG00000136861	1155.629744	-0.168941626	0.071198068	-2.372840039	0.017651907	0.052179363
ENSG00000136866	52.93284208	0.034278701	0.241189515	0.142123511	0.886982448	0.938194798
ENSG00000136867	14.97958778	0.338577595	0.48269386	0.701433401	0.483032584	0.643570018
ENSG00000136868	321.6190225	-0.254675045	0.118378755	-2.151357696	0.031447978	0.083617214
ENSG00000136870	96.56729159	0.025582517	0.178584939	0.143251259	0.886091748	0.937763187
ENSG00000136874	333.6604166	-0.567652114	0.10639356	-5.335399215	9.53E-08	1.01E-06
ENSG00000136875	520.58677	0.166218512	0.089958894	1.847716273	0.064643399	0.149497172
ENSG00000136877	111.2335593	0.265543279	0.179446486	1.479790908	0.138929056	0.269577372
ENSG00000136878	163.1813692	0.261631613	0.146225484	1.789234034	0.073577133	0.16502283
ENSG00000136888	1131.643636	0.553370101	0.077193906	7.168572381	7.58E-13	1.53E-11
ENSG00000136891	358.4143584	0.328358813	0.103597469	3.169564043	0.001526678	0.006482844
ENSG00000136895	97.90099266	-0.321879569	0.186265082	-1.72807252	0.083975224	0.183532304
ENSG00000136897	141.802572	-0.086090162	0.154600323	-0.55685629	0.577625621	0.721236186
ENSG00000136908	118.3389667	0.275503258	0.165271509	1.666973694	0.095519636	0.202591624
ENSG00000136925	135.0016875	0.114340794	0.159122068	0.718572824	0.472404163	0.633981332
ENSG00000136930	992.625073	0.539691433	0.093468768	5.774029585	7.74E-09	9.67E-08
ENSG00000136932	53.77537699	-0.277820665	0.245157742	-1.13323227	0.257116726	0.422868358
ENSG00000136933	164.1161428	0.155465953	0.143519713	1.083237626	0.278702963	0.447443242
ENSG00000136935	206.204895	0.347711831	0.131378564	2.646640511	0.008129572	0.027108334
ENSG00000136936	153.7261179	0.256188027	0.148809565	1.721583067	0.085145073	0.185316522
ENSG00000136937	333.7393673	0.080986774	0.108602939	0.74571439	0.455839965	0.61958531

ENSG00000136938	2164.938116	0.124826334	0.061543214	2.028271293	0.042532567	0.107513941
ENSG00000136940	201.7405936	-0.145868915	0.132488156	-1.10099589	0.270898446	0.438500861
ENSG00000136942	2160.574754	0.383241546	0.061076489	6.274780211	3.50E-10	5.19E-09
ENSG00000136943	130.9048704	-0.988265853	0.161479388	-6.120074319	9.35E-10	1.31E-08
ENSG00000136944	50.95853958	-1.468472767	0.2638407	-5.565755272	2.61E-08	3.02E-07
ENSG00000136950	306.9246485	0.215474554	0.118624694	1.816439287	0.069303013	0.157798757
ENSG00000136982	194.5602184	-0.470884805	0.134921972	-3.490052796	0.000482925	0.002348096
ENSG00000136986	393.4132745	0.335863528	0.102525375	3.275906362	0.001053235	0.004708717
ENSG00000136997	3498.557789	0.304390561	0.061997652	4.90971111	9.12E-07	8.15E-06
ENSG00000137038	196.8115091	0.18431475	0.145730902	1.264760925	0.205957043	0.359509655
ENSG00000137040	99.23127099	0.199616911	0.179181936	1.114045957	0.265259472	0.431911417
ENSG00000137054	316.8777107	0.310141215	0.109803332	2.824515507	0.004735216	0.017090333
ENSG00000137055	338.312492	-0.117839737	0.105774766	-1.114062847	0.265252226	0.431911417
ENSG00000137070	12.95733696	-0.744778542	0.486971576	-1.529408651	0.126163169	0.25112853
ENSG00000137073	1007.764245	-0.059718921	0.07468663	-0.799593196	0.423946531	0.591201918
ENSG00000137074	201.9151024	0.100183719	0.139834776	0.716443518	0.473717532	0.634892679
ENSG00000137075	346.4929906	-0.07758921	0.111392089	-0.696541469	0.486089797	0.646507903
ENSG00000137076	1965.526488	-0.032924835	0.065224275	-0.504794185	0.6137034	0.748716754
ENSG00000137094	51.61477261	-0.576342194	0.26069729	-2.21077171	0.02705165	0.074073007
ENSG00000137100	158.1446949	0.391256324	0.152681012	2.562573559	0.010389958	0.033351859
ENSG00000137103	48.6326369	-0.806561835	0.261224119	-3.087623912	0.002017636	0.008236483
ENSG00000137106	549.6596029	0.190238653	0.0985943	1.929509656	0.053667623	0.12879951
ENSG00000137124	562.5509647	-0.128921298	0.088226833	-1.461248164	0.14394735	0.276497801
ENSG00000137133	28.70256069	-0.406812465	0.361434252	-1.125550396	0.260355874	0.426544224
ENSG00000137135	174.4395775	0.016800679	0.148258063	0.113320505	0.909776462	0.951381168
ENSG00000137142	11.6337763	-2.831108537	0.657910591	-4.303181275	1.68E-05	0.000114861
ENSG00000137145	402.2235944	0.018765519	0.098145248	0.191201501	0.848367728	0.912928344
ENSG00000137154	8312.264511	-0.041111018	0.057238599	-0.718239421	0.472609676	0.633981332
ENSG00000137161	348.116735	0.142120973	0.104295603	1.36267464	0.172985074	0.316650919
ENSG00000137166	503.6072607	0.022371228	0.091021088	0.245780712	0.805851989	0.885189466
ENSG00000137168	213.3198659	0.001850458	0.128532733	0.014396786	0.988513423	0.994175418
ENSG00000137171	57.11040585	-0.40483707	0.234398729	-1.727129969	0.084144324	0.183654196
ENSG00000137177	540.8757201	0.087947743	0.09128365	0.963455588	0.335318941	0.507124904
ENSG00000137185	75.09476654	0.067309251	0.209915785	0.320648833	0.748476527	0.847003124
ENSG00000137193	64.43099666	-1.154976547	0.263942342	-4.375866861	1.21E-05	8.51E-05
ENSG00000137200	215.9881078	-0.090531132	0.126773545	-0.71411691	0.475154891	0.636151425
ENSG00000137203	165.0648644	-7.528844143	0.65808252	-11.4405776	2.62E-30	1.44E-28
ENSG00000137207	600.9210487	0.359841052	0.094346119	3.814052495	0.000136706	0.000762938
ENSG00000137210	122.1181252	-0.271580369	0.161289601	-1.683805817	0.092219203	0.197035205
ENSG00000137216	253.8056057	-0.041200265	0.123944896	-0.332407921	0.739581258	0.840729783
ENSG00000137218	32.27772658	-0.13936862	0.317451907	-0.439022783	0.660645028	0.783076162
ENSG00000137221	173.8587477	0.350403027	0.148589078	2.358201774	0.018363708	0.053864066
ENSG00000137266	21.47196658	-1.941216402	0.434910492	-4.463484877	8.06E-06	5.91E-05
ENSG00000137267	24.8683477	0.803474184	0.350558949	2.29198024	0.021906783	0.062191711
ENSG00000137269	92.19639333	-0.176427451	0.183732769	-0.960239441	0.336934721	0.508806926
ENSG00000137274	99.63758682	-0.232322236	0.177409042	-1.30952872	0.190355316	0.340020488
ENSG00000137275	345.8340084	-0.18097692	0.104023118	-1.73977596	0.081898365	0.179866532
ENSG00000137285	9.116412886	-1.931827318	0.654745285	-2.950502071	0.003172579	0.012116699
ENSG00000137288	291.5676859	-0.236474365	0.113845231	-2.077156525	0.037787115	0.097277032
ENSG00000137309	6631.481681	0.123648352	0.066649307	1.855208359	0.063566478	0.14744358

ENSG00000137310	131.6367021	0.119092531	0.160968125	0.739851636	0.459390023	0.622670717
ENSG00000137312	413.6993029	0.1418788	0.097223556	1.459304778	0.144481241	0.277371599
ENSG00000137331	112.1359998	-0.373636583	0.170466204	-2.191851367	0.028390241	0.077032589
ENSG00000137337	1389.028813	0.077902182	0.065466875	1.189948082	0.234066799	0.394662864
ENSG00000137338	37.7876712	-0.599419587	0.292406262	-2.049954685	0.040368853	0.102561273
ENSG00000137343	99.38479889	0.2139236	0.184850138	1.157281257	0.247157469	0.411255741
ENSG00000137364	89.64714729	0.107084091	0.192706449	0.555685042	0.578426184	0.721948544
ENSG00000137404	162.7304365	-0.259391531	0.147882636	-1.754036434	0.079424264	0.175467193
ENSG00000137409	624.6315951	0.20088305	0.089489403	2.244769132	0.024782957	0.068745301
ENSG00000137413	281.1999153	-0.018682427	0.117231952	-0.15936293	0.873382943	0.929849389
ENSG00000137414	308.1835808	0.504929504	0.117841464	4.284820345	1.83E-05	0.000123748
ENSG00000137449	27.16153794	0.883131065	0.343700907	2.569475515	0.010185259	0.032784677
ENSG00000137474	158.4978839	0.478098609	0.149673566	3.194275534	0.001401823	0.006027754
ENSG00000137478	334.0795731	0.665973014	0.111377258	5.979434485	2.24E-09	3.00E-08
ENSG00000137486	121.4826527	0.516260187	0.162630011	3.174445996	0.001501228	0.006396444
ENSG00000137492	218.2586347	0.223369384	0.130548964	1.711000823	0.087080961	0.188441124
ENSG00000137494	176.1365563	-0.150737909	0.144210428	-1.045263583	0.295901164	0.46639628
ENSG00000137496	63.0230092	0.490288409	0.229356731	2.137667413	0.032543746	0.086042161
ENSG00000137497	3730.074957	0.430953482	0.058644973	7.348515309	2.00E-13	4.26E-12
ENSG00000137500	249.4266531	-0.334758913	0.123650849	-2.707291671	0.006783463	0.023164212
ENSG00000137501	6.481590555	-1.75573387	0.750552702	-2.339254612	0.01932226	0.056136461
ENSG00000137502	31.81062661	-0.094326776	0.309060939	-0.305204457	0.760210438	0.855251964
ENSG00000137504	637.6072754	0.008315562	0.086106324	0.09657319	0.923065348	0.957558448
ENSG00000137509	297.9814213	-0.465194319	0.111786884	-4.161439169	3.16E-05	0.000203777
ENSG00000137513	88.26186277	-0.391758982	0.189626014	-2.065955898	0.038832644	0.099486075
ENSG00000137522	136.6873234	0.413313217	0.156307596	2.644229894	0.008187703	0.027273573
ENSG00000137547	193.7672669	0.076266987	0.131711804	0.579044433	0.562559193	0.708853276
ENSG00000137558	15.78682028	-0.196195101	0.447389925	-0.438532676	0.661000188	0.783153647
ENSG00000137561	13.35652655	0.938735402	0.485920204	1.931871518	0.053375366	0.128274383
ENSG00000137563	231.5957456	0.316387383	0.126474304	2.501594194	0.012363555	0.03864505
ENSG00000137574	533.7347817	-0.187892954	0.093287784	-2.014121744	0.043996753	0.110423076
ENSG00000137575	147.4634652	0.563339332	0.156263815	3.605052988	0.000312089	0.001592415
ENSG00000137601	110.3876999	-0.16761352	0.171831393	-0.97545342	0.329335396	0.501837061
ENSG00000137628	54.01890796	0.176116017	0.241857374	0.728181297	0.466502613	0.628880558
ENSG00000137642	451.431051	0.860203063	0.09409168	9.142179897	6.12E-20	2.06E-18
ENSG00000137656	174.9424914	0.203727557	0.137770652	1.478744227	0.139208687	0.269816391
ENSG00000137691	31.74036797	-0.212621764	0.309980499	-0.685919805	0.49276368	0.652082093
ENSG00000137692	540.5768991	0.223335884	0.087425244	2.554592613	0.010631214	0.033940152
ENSG00000137693	1023.161562	0.306632239	0.075325831	4.070744879	4.69E-05	0.000290377
ENSG00000137700	272.5089836	0.172335382	0.11690889	1.474099883	0.1404547	0.271518409
ENSG00000137709	23.77917917	0.208702527	0.349952179	0.596374419	0.550925126	0.698621617
ENSG00000137710	730.5934901	-0.150591132	0.079233784	-1.900592466	0.057355413	0.135722445
ENSG00000137713	225.9975733	-0.240754015	0.124389429	-1.935486133	0.052930669	0.127458433
ENSG00000137714	88.53104313	-0.30334516	0.198953827	-1.524701307	0.127333616	0.252492278
ENSG00000137720	49.8432932	-1.007524337	0.258697001	-3.894611587	9.84E-05	0.000566233
ENSG00000137726	40.72564975	0.867853896	0.281341962	3.084694119	0.002037616	0.008293941
ENSG00000137760	70.07603023	0.073926547	0.218099498	0.338957896	0.734641449	0.83646822
ENSG00000137764	147.7053088	0.11442895	0.152608866	0.749818494	0.453364029	0.617396281
ENSG00000137770	673.3869588	-0.277804662	0.087098956	-3.189529181	0.001425048	0.006114124
ENSG00000137776	1593.779143	0.131012649	0.065150735	2.010915912	0.044334339	0.110930614

ENSG00000137801	36.2378604	-3.475072103	0.437316831	-7.946348856	1.92E-15	4.78E-14
ENSG00000137802	243.2292294	0.041435761	0.12039874	0.34415444	0.730730143	0.833636508
ENSG00000137806	113.39208	0.253513958	0.173858297	1.45816428	0.144795267	0.277783072
ENSG00000137807	524.0623475	0.03935391	0.088078489	0.446805011	0.655015853	0.778784714
ENSG00000137812	652.803653	-0.126490187	0.087045397	-1.453151932	0.146181575	0.279712991
ENSG00000137814	97.77272673	0.42082667	0.18250833	2.305794314	0.021122128	0.06041591
ENSG00000137815	1058.325156	-0.064570691	0.074815112	-0.863070168	0.388098889	0.556629068
ENSG00000137817	143.1070445	0.298135746	0.152577292	1.953998145	0.050701437	0.123175904
ENSG00000137818	2516.106229	0.078544744	0.06460607	1.215748673	0.224080676	0.382368958
ENSG00000137819	24.29197914	0.703556754	0.354111853	1.986820685	0.046942272	0.116091213
ENSG00000137821	25.88580378	-0.005479635	0.343390918	-0.015957425	0.987268357	0.993349947
ENSG00000137822	490.3066874	0.23425224	0.094532677	2.478002825	0.01321201	0.040887663
ENSG00000137824	134.1488491	0.302462854	0.159270353	1.899053073	0.057557498	0.136067011
ENSG00000137825	415.6126486	0.431547911	0.102768805	4.199211151	2.68E-05	0.000174835
ENSG00000137831	1604.251873	-0.17463185	0.069543512	-2.511116342	0.012035003	0.03777553
ENSG00000137834	204.1007428	0.202722643	0.142713679	1.420485022	0.155466526	0.292975354
ENSG00000137842	54.95427394	0.457192651	0.24327073	1.879357419	0.060195706	0.141187024
ENSG00000137845	944.1609141	0.212811936	0.073203539	2.907126322	0.003647659	0.013664491
ENSG00000137868	122.7833324	-2.414649946	0.195394546	-12.3578165	4.42E-35	2.89E-33
ENSG00000137871	71.57073585	-0.380530259	0.216951629	-1.753986641	0.079432796	0.175467193
ENSG00000137872	173.9108418	-5.736345708	0.360152134	-15.92756272	4.08E-57	5.03E-55
ENSG00000137876	480.6712172	0.175920387	0.096451023	1.823934901	0.068161915	0.155862959
ENSG00000137880	147.6703238	-0.128359176	0.163048386	-0.787245911	0.431137926	0.597552486
ENSG00000137936	20.9794324	-1.291493004	0.401705104	-3.215027616	0.00130432	0.005656616
ENSG00000137941	62.22644826	0.533875761	0.229476347	2.326495814	0.019992116	0.057736276
ENSG00000137942	297.8022107	-0.25499299	0.109308319	-2.332786686	0.019659344	0.057009662
ENSG00000137944	48.44260301	0.014994444	0.248760326	0.060276669	0.951935284	0.973488274
ENSG00000137947	145.212439	0.362118013	0.152451248	2.375303696	0.017534517	0.051919763
ENSG00000137955	674.8154833	0.13352668	0.083786223	1.593659142	0.1110124	0.22761512
ENSG00000137959	13.01257994	-3.485124665	0.710146795	-4.90761162	9.22E-07	8.23E-06
ENSG00000137992	259.9790137	-0.438739064	0.121387833	-3.614357827	0.000301093	0.001543596
ENSG00000137996	175.0577984	0.447652446	0.137158264	3.263765755	0.00109942	0.004885655
ENSG00000138002	191.5771956	0.080824081	0.138256219	0.584596353	0.558819161	0.705699959
ENSG00000138018	721.7187766	-0.141719401	0.081599667	-1.736764464	0.082428754	0.180777003
ENSG00000138029	766.1014087	0.186840374	0.078789298	2.371392798	0.017721187	0.052329096
ENSG00000138030	86.69686222	-1.040156773	0.199806031	-5.205832703	1.93E-07	1.94E-06
ENSG00000138031	871.5500464	0.511232494	0.077030254	6.636775373	3.21E-11	5.38E-10
ENSG00000138032	44.51313884	-0.40253038	0.267242597	-1.50623585	0.13200664	0.259851074
ENSG00000138035	410.7903941	0.134634096	0.096693065	1.392386265	0.163805461	0.304402079
ENSG00000138036	78.4747689	-0.033208645	0.199880943	-0.166142127	0.868045102	0.926341226
ENSG00000138050	146.853289	0.071457396	0.150274246	0.475513259	0.634421185	0.763643049
ENSG00000138061	258.6546369	-4.541516116	0.212103792	-21.411763	1.04E-101	2.75E-99
ENSG00000138069	1016.577164	0.02933431	0.077077285	0.380583075	0.703512642	0.814282078
ENSG00000138071	1136.721368	-0.080095219	0.085865385	-0.932799862	0.350923312	0.522671567
ENSG00000138073	582.2326807	0.223702569	0.088762017	2.520251076	0.011727115	0.036943437
ENSG00000138074	469.5453732	0.07155774	0.093843391	0.762522949	0.445747951	0.610751217
ENSG00000138078	521.3310583	0.031167752	0.096509977	0.322948497	0.746734247	0.845576459
ENSG00000138080	499.6166114	-1.247573958	0.186420613	-6.692253259	2.20E-11	3.73E-10
ENSG00000138081	896.0356928	0.089795747	0.082106822	1.093645391	0.274110534	0.442344827
ENSG00000138085	661.7892621	0.436862196	0.099133579	4.406803454	1.05E-05	7.48E-05

ENSG00000138092	341.8201272	-0.185326296	0.104098772	-1.780292817	0.075028052	0.167474821
ENSG00000138095	2464.028203	-0.65829553	0.063940276	-10.2954753	7.39E-25	3.26E-23
ENSG00000138101	282.9403039	-0.095147267	0.120588302	-0.78902568	0.430096998	0.596875373
ENSG00000138107	389.8887036	-0.008503225	0.101187083	-0.084034687	0.933028853	0.963270344
ENSG00000138111	43.28753889	-0.26510761	0.262629829	-1.009434502	0.312766299	0.484076461
ENSG00000138119	296.3803158	-0.127410664	0.118608427	-1.074212574	0.282727418	0.452173323
ENSG00000138131	19.80560753	-1.505392118	0.425613273	-3.536995236	0.000404707	0.002004555
ENSG00000138134	73.23015909	0.745476465	0.208716858	3.571711797	0.000354656	0.001784968
ENSG00000138136	15.52278519	-5.384221741	1.060131629	-5.078823792	3.80E-07	3.66E-06
ENSG00000138138	249.5218085	-0.456225439	0.121705896	-3.74858947	0.000177832	0.000964108
ENSG00000138160	847.9821918	-0.169839254	0.076346114	-2.224595943	0.026108375	0.071938868
ENSG00000138162	452.4907574	-0.574569094	0.097002226	-5.923256791	3.16E-09	4.12E-08
ENSG00000138166	52.06581893	-0.436666446	0.24350578	-1.793248795	0.072933151	0.163962765
ENSG00000138175	311.4437383	0.082930711	0.114815075	0.722298101	0.47011122	0.631988928
ENSG00000138180	236.3908302	0.082865243	0.128565964	0.644534838	0.519228674	0.67396093
ENSG00000138182	770.7229185	-0.212601997	0.091116881	-2.333288804	0.019632993	0.056945003
ENSG00000138185	11.80430262	-1.45470356	0.560284281	-2.596366896	0.00942154	0.030706351
ENSG00000138190	212.4643166	0.440107151	0.135203119	3.255155319	0.001133304	0.005015601
ENSG00000138193	39.44104374	-0.211905951	0.283532575	-0.747377797	0.454835544	0.618676759
ENSG00000138231	103.733349	-0.26798964	0.176046875	-1.522262977	0.127943201	0.253414854
ENSG00000138246	402.1451279	-0.063976765	0.10286187	-0.621967739	0.533963081	0.685234734
ENSG00000138279	322.0238112	0.432078069	0.106032291	4.074966835	4.60E-05	0.000286042
ENSG00000138286	98.34473324	-0.141229372	0.180004144	-0.784589557	0.432694255	0.599012271
ENSG00000138303	141.3150972	-0.095196016	0.15145244	-0.628553865	0.529641172	0.682186526
ENSG00000138316	20.23313638	0.034733775	0.379176959	0.091603073	0.92701341	0.960164973
ENSG00000138326	7154.46349	0.182041627	0.059252749	3.072289979	0.002124233	0.008579383
ENSG00000138336	672.9472046	-0.448845059	0.09232663	-4.861490734	1.17E-06	1.02E-05
ENSG00000138346	105.6415703	-0.167556179	0.1773342	-0.944861055	0.344729816	0.516469681
ENSG00000138347	6.193688473	-4.879230761	1.302775857	-3.74525728	0.000180209	0.000975866
ENSG00000138363	818.8225925	0.071829648	0.083981088	0.855307425	0.392380978	0.560693001
ENSG00000138375	182.1485008	-0.232620761	0.134633531	-1.727807024	0.084022828	0.183533579
ENSG00000138376	161.9458497	-0.27546996	0.143848298	-1.915003269	0.055492087	0.132342503
ENSG00000138380	32.45792279	-0.721094588	0.318226618	-2.265978229	0.023452716	0.065738606
ENSG00000138381	49.33549847	-0.290354362	0.29112875	-0.997340052	0.318599479	0.490203497
ENSG00000138382	324.7402032	0.13686437	0.109805151	1.246429419	0.212606786	0.36779403
ENSG00000138385	1464.168499	0.063243878	0.066075537	0.957145123	0.338494011	0.510448242
ENSG00000138386	347.1716176	0.399988304	0.104382084	3.831963182	0.000127125	0.000716084
ENSG00000138398	1494.100592	-0.168580457	0.073306552	-2.299664249	0.021467249	0.061163822
ENSG00000138399	135.9093314	-0.232907436	0.162615463	-1.432258851	0.152069755	0.288353651
ENSG00000138411	148.5449512	0.280061834	0.147097262	1.903922819	0.056920237	0.134901057
ENSG00000138413	431.9484549	0.049315368	0.095025589	0.518969341	0.603782121	0.741439589
ENSG00000138430	670.2545006	0.20281617	0.088176909	2.300105229	0.021442259	0.061144826
ENSG00000138433	197.0874902	0.109736222	0.138832005	0.790424527	0.429279883	0.596126245
ENSG00000138434	12.40198498	-1.952664866	0.556618235	-3.50808641	0.000451342	0.002210602
ENSG00000138439	182.0892823	0.054709135	0.160485249	0.340898216	0.733180208	0.835693316
ENSG00000138442	456.8157857	-0.028625128	0.093343264	-0.306665168	0.759098245	0.854860493
ENSG00000138443	1334.118481	-0.054203222	0.071641981	-0.756584641	0.449298746	0.614298178
ENSG00000138448	1826.267879	-1.727116737	0.073125293	-23.6185959	2.48E-123	8.72E-121
ENSG00000138449	19.15366345	-2.404761522	0.487945464	-4.928340766	8.29E-07	7.49E-06
ENSG00000138459	94.22221972	-0.193921753	0.187235167	-1.035712233	0.300336413	0.470693473

ENSG00000138463	58.91099151	-0.126730709	0.231066728	-0.548459357	0.583376529	0.725450611
ENSG00000138468	152.542824	-0.308459837	0.147574873	-2.090192127	0.036600544	0.094875041
ENSG00000138495	399.2271795	0.054122308	0.118161629	0.458036238	0.646926407	0.772538913
ENSG00000138496	217.1447842	0.805086465	0.130004926	6.192738132	5.91E-10	8.52E-09
ENSG00000138592	573.1208736	-0.132524797	0.087138415	-1.520853875	0.128296511	0.253993248
ENSG00000138593	447.0918674	0.059604056	0.099505882	0.599000326	0.549172667	0.69718384
ENSG00000138594	328.2453201	-0.242043339	0.10685382	-2.265181903	0.023501519	0.065814562
ENSG00000138600	277.8953981	-0.358104755	0.1198043	-2.989080977	0.00279818	0.010876046
ENSG00000138604	84.52464713	-1.025318588	0.209211624	-4.90086816	9.54E-07	8.49E-06
ENSG00000138606	34.45604685	0.458459779	0.317889241	1.442199732	0.149246036	0.283957615
ENSG00000138613	55.57049787	-0.449444447	0.235813757	-1.905929716	0.056659325	0.134600632
ENSG00000138614	228.7601222	0.198673023	0.12146891	1.635587445	0.10192596	0.212868563
ENSG00000138617	44.44878239	0.228330541	0.25807712	0.884737637	0.376298151	0.546116348
ENSG00000138621	46.78933691	-0.043691042	0.251573228	-0.173671271	0.862123817	0.921632668
ENSG00000138623	15.65159672	-0.172787171	0.442796179	-0.390218296	0.696375134	0.809436951
ENSG00000138629	202.1328972	0.320717878	0.132054437	2.428679301	0.01515393	0.045916146
ENSG00000138640	401.9584394	0.509725179	0.102316269	4.981858566	6.30E-07	5.83E-06
ENSG00000138641	10.78146837	0.109674265	0.517916482	0.211760523	0.832293868	0.90196204
ENSG00000138642	69.34693122	1.445872485	0.226656096	6.379146703	1.78E-10	2.73E-09
ENSG00000138646	49.10746158	2.701548252	0.307481581	8.786049054	1.55E-18	4.90E-17
ENSG00000138650	520.3034565	-0.809102661	0.093444757	-8.658620213	4.78E-18	1.43E-16
ENSG00000138653	9.482782367	-0.504921912	0.576401836	-0.875989423	0.381035784	0.550538001
ENSG00000138658	186.3916034	-0.224873178	0.151067356	-1.488562345	0.136602653	0.266138346
ENSG00000138660	116.0648954	0.039079857	0.164494358	0.23757567	0.812210222	0.889524775
ENSG00000138663	174.1097182	0.187641408	0.138657127	1.353276335	0.175967367	0.320764806
ENSG00000138668	4230.390886	0.269597734	0.065422091	4.120897528	3.77E-05	0.000238799
ENSG00000138674	1569.290824	0.169986854	0.076296831	2.227967418	0.025882688	0.071415069
ENSG00000138685	165.6895812	-0.235016032	0.14288038	-1.644844668	0.100001848	0.209691888
ENSG00000138686	171.6612564	-0.054562015	0.14315228	-0.381146676	0.703094414	0.814114448
ENSG00000138688	599.7158275	-0.307614437	0.098830775	-3.112536915	0.001854868	0.007674619
ENSG00000138698	170.0258832	0.199782974	0.137897642	1.448777312	0.147399782	0.281627412
ENSG00000138709	302.4987671	-0.125951535	0.111798718	-1.126591952	0.259915044	0.425974404
ENSG00000138722	865.7444833	-3.604490815	0.103292837	-34.89584489	8.60E-267	1.01E-263
ENSG00000138735	26.91303458	-0.791035973	0.3427828	-2.307688639	0.02101646	0.060159869
ENSG00000138738	123.2679366	-0.530495969	0.162166557	-3.271303149	0.001070531	0.004773888
ENSG00000138744	216.4763659	0.266655013	0.131883943	2.021891432	0.043187569	0.108875769
ENSG00000138750	309.1991701	0.002659353	0.110323421	0.024105064	0.980768804	0.990179984
ENSG00000138756	191.6051958	-0.272360942	0.13530993	-2.012867358	0.044128585	0.11057883
ENSG00000138757	890.8113001	-0.064993651	0.073324061	-0.886389138	0.37540787	0.545387771
ENSG00000138758	851.0727724	-0.130722916	0.080432164	-1.625256739	0.104107865	0.216267847
ENSG00000138759	319.0567648	-0.969005382	0.111279699	-8.707836108	3.10E-18	9.46E-17
ENSG00000138760	977.9667363	0.592745139	0.077820243	7.616850295	2.60E-14	5.98E-13
ENSG00000138764	140.412852	-0.261407235	0.158801886	-1.646121732	0.099738699	0.209324691
ENSG00000138767	301.2488156	-0.007396629	0.111046733	-0.066608257	0.946893572	0.97059355
ENSG00000138768	786.0208182	0.175159334	0.083877389	2.088278335	0.036772735	0.095215964
ENSG00000138769	8.783817103	0.234315736	0.625306667	0.374721313	0.70786774	0.816842039
ENSG00000138771	640.6853181	-1.793344435	0.094975183	-18.88224252	1.60E-79	2.88E-77
ENSG00000138777	246.1368919	-0.257684943	0.119845061	-2.150150711	0.031543295	0.083838916
ENSG00000138778	1353.869681	-0.786303488	0.072510673	-10.84396901	2.13E-27	1.05E-25
ENSG00000138780	232.9822673	-0.48578463	0.123733575	-3.926053467	8.64E-05	0.000504354

ENSG00000138785	123.4998197	0.039040856	0.165455593	0.235959727	0.813463921	0.890417494
ENSG00000138794	75.61242219	-0.254827898	0.213723896	-1.192322909	0.233134655	0.393279993
ENSG00000138796	306.7103551	-0.125566959	0.11547112	-1.087431722	0.276846058	0.445411754
ENSG00000138801	411.4215495	0.327240357	0.099609708	3.285225561	0.001019008	0.004570243
ENSG00000138802	614.2436626	0.206453917	0.095304391	2.16625819	0.030291463	0.081141147
ENSG00000138814	222.4440841	0.279089962	0.127626085	2.186778372	0.028758707	0.077843973
ENSG00000138821	180.4431511	-0.253988641	0.139065686	-1.826393324	0.067791037	0.155293655
ENSG00000138829	67.75648623	-5.053766693	0.470011234	-10.75243809	5.77E-27	2.78E-25
ENSG00000138834	358.3672113	0.142932335	0.114513422	1.248171016	0.211968436	0.367051358
ENSG00000138835	901.4567078	0.576911695	0.096864226	5.955879904	2.59E-09	3.41E-08
ENSG00000138867	496.1478474	0.016910976	0.103222199	0.163830805	0.86986434	0.927577954
ENSG00000138942	80.55959547	0.056359525	0.217080074	0.259625511	0.795152656	0.87776105
ENSG00000139044	77.53466963	1.331531011	0.213449697	6.238148984	4.43E-10	6.46E-09
ENSG00000139083	233.8519851	-0.426973489	0.122664284	-3.48082976	0.000499863	0.002422904
ENSG00000139112	109.690993	0.213545246	0.170845822	1.249929578	0.211325273	0.366298879
ENSG00000139116	226.2593461	0.346320585	0.125787059	2.753229045	0.00590106	0.020575238
ENSG00000139117	171.9239006	-0.091715568	0.141139152	-0.649823714	0.515806099	0.671096242
ENSG00000139131	162.9304125	0.054007047	0.146356751	0.369009604	0.712120567	0.820132623
ENSG00000139133	25.56561679	-0.753407103	0.347718863	-2.166713352	0.030256718	0.081074426
ENSG00000139144	426.1231672	1.325321255	0.105040218	12.61727443	1.70E-36	1.16E-34
ENSG00000139146	518.3927713	-0.056926862	0.093381503	-0.609616036	0.542116186	0.691527975
ENSG00000139154	1094.622972	0.445274074	0.074299396	5.992970309	2.06E-09	2.76E-08
ENSG00000139160	10.66814858	-0.57681478	0.543004031	-1.062266111	0.288114894	0.45813334
ENSG00000139163	386.7831176	-0.141737856	0.1006237	-1.40859316	0.158955502	0.297514716
ENSG00000139168	646.4586084	-0.04325495	0.082769659	-0.522594277	0.601256624	0.739437208
ENSG00000139173	148.7342405	0.263016002	0.157718999	1.667624093	0.095390372	0.202348016
ENSG00000139174	262.9318795	-0.786341018	0.119037032	-6.605852014	3.95E-11	6.57E-10
ENSG00000139178	120.458314	-0.215575488	0.170299588	-1.265860302	0.205563101	0.359102709
ENSG00000139180	50.27782006	-0.594117368	0.259731302	-2.287430756	0.022170693	0.062662318
ENSG00000139182	137.7148577	0.280599789	0.157819916	1.777974518	0.07540804	0.168189384
ENSG00000139190	96.04124149	0.273605403	0.185357026	1.476099443	0.139917198	0.270793178
ENSG00000139197	284.1249439	-0.112940997	0.113107642	-0.998526668	0.31802404	0.48988389
ENSG00000139200	249.2620025	0.488062835	0.125101619	3.901331089	9.57E-05	0.000553236
ENSG00000139211	25.27206732	-0.761133044	0.353759374	-2.151555833	0.031432354	0.083601154
ENSG00000139218	1056.575473	-0.214057769	0.072219657	-2.96398206	0.00303686	0.011668154
ENSG00000139219	107.9827082	-1.151576283	0.184486253	-6.242070947	4.32E-10	6.31E-09
ENSG00000139233	150.4592621	0.205303195	0.146744013	1.399056702	0.16179598	0.301505457
ENSG00000139239	73.92246292	0.372435821	0.204692646	1.819488035	0.068837007	0.156998447
ENSG00000139263	114.1988948	-1.71332335	0.182499813	-9.388082783	6.11E-21	2.18E-19
ENSG00000139266	172.5516727	0.204018462	0.140932408	1.44763341	0.147719602	0.281855101
ENSG00000139269	10.31680001	-1.866642786	0.604325676	-3.088802711	0.002009648	0.008211033
ENSG00000139289	35.54521255	-1.654196805	0.320826276	-5.156051514	2.52E-07	2.50E-06
ENSG00000139291	62.37732469	-0.288473775	0.226778457	-1.272051051	0.203354981	0.356353222
ENSG00000139292	17.86341798	-6.452938219	1.224256143	-5.27090532	1.36E-07	1.40E-06
ENSG00000139318	1302.424404	-2.431943339	0.085891756	-28.31404841	2.32E-176	1.55E-173
ENSG00000139323	153.4491136	-1.561581711	0.160192497	-9.748157603	1.88E-22	7.21E-21
ENSG00000139324	161.0105832	-0.335632081	0.15315974	-2.191385812	0.028423885	0.077064333
ENSG00000139329	729.2032144	3.103550688	0.09981556	31.09285444	3.01E-212	2.64E-209
ENSG00000139330	14.92766382	-2.488980483	0.561207359	-4.43504605	9.21E-06	6.64E-05
ENSG00000139343	1749.539752	0.287947047	0.061831702	4.656948385	3.21E-06	2.56E-05

ENSG00000139350	235.2126023	-0.379093682	0.127205824	-2.980159799	0.002880981	0.011154667
ENSG00000139354	235.026175	-0.103249264	0.122372853	-0.843726868	0.39882205	0.56691493
ENSG00000139370	99.91537317	0.577330214	0.184076069	3.136367582	0.001710547	0.007159728
ENSG00000139372	80.06198585	-0.311160772	0.204313676	-1.522956163	0.127769673	0.253106839
ENSG00000139405	326.5818963	-0.112768182	0.109395171	-1.030833273	0.302619012	0.47321488
ENSG00000139410	41.19344415	-0.619380989	0.279649474	-2.214847682	0.026770521	0.073460737
ENSG00000139428	161.4639323	0.087240422	0.148274036	0.588372885	0.556282034	0.703356436
ENSG00000139433	159.8240239	0.395017803	0.146572537	2.695032852	0.007038169	0.023926709
ENSG00000139436	298.9702652	-0.026190703	0.114683907	-0.228372958	0.81935631	0.893395096
ENSG00000139437	378.1347653	-0.029826005	0.111611354	-0.267230923	0.789291369	0.87424506
ENSG00000139438	72.92485991	-0.438571497	0.213074857	-2.058297736	0.039561561	0.100948456
ENSG00000139445	90.96663316	-2.039054447	0.21235026	-9.602316677	7.82E-22	2.91E-20
ENSG00000139496	1151.180551	0.101144887	0.068658151	1.473166482	0.140706151	0.271833214
ENSG00000139505	184.8245222	-0.200363652	0.139737351	-1.433858954	0.151612517	0.28760311
ENSG00000139514	1464.159157	-0.14409774	0.071372381	-2.018956599	0.043491731	0.109361608
ENSG00000139517	104.7453738	-0.012971518	0.178343203	-0.072733461	0.942018221	0.967997802
ENSG00000139531	180.5129855	-0.326512343	0.145720599	-2.240673891	0.025047207	0.069396077
ENSG00000139546	104.7423334	-0.121740467	0.174945056	-0.695878293	0.486505052	0.646908034
ENSG00000139579	249.7020222	0.553040782	0.119700703	4.620196627	3.83E-06	3.01E-05
ENSG00000139613	1344.380778	0.230008969	0.069565277	3.30637612	0.000945111	0.00427155
ENSG00000139618	578.6534869	-0.638066832	0.092696003	-6.883434116	5.84E-12	1.06E-10
ENSG00000139620	160.5359771	0.140616684	0.155570536	0.903877353	0.366060439	0.537374197
ENSG00000139624	421.3619351	0.009746397	0.09928772	0.098163168	0.921802729	0.956832011
ENSG00000139625	443.9216535	-0.146780821	0.096634386	-1.518929516	0.128780239	0.254677743
ENSG00000139629	103.2080101	-0.459348961	0.176914675	-2.596443509	0.009419439	0.030706351
ENSG00000139631	79.4680149	-0.066721473	0.200998506	-0.331950096	0.739926942	0.840986629
ENSG00000139636	38.83484786	-0.044028404	0.291757888	-0.150907329	0.880048819	0.934004754
ENSG00000139641	631.4020712	0.048713295	0.091724443	0.53108303	0.595361243	0.735083008
ENSG00000139644	1336.876146	0.169471013	0.072267335	2.3450569	0.019024176	0.055419453
ENSG00000139645	709.1583562	-0.187111878	0.080645885	-2.320166459	0.020331874	0.058573535
ENSG00000139651	332.0667893	-0.047409979	0.111846649	-0.423883765	0.671650585	0.790703634
ENSG00000139668	211.9230448	-0.450388754	0.132527588	-3.39845282	0.000677682	0.003176307
ENSG00000139684	559.6549428	0.028360117	0.099051266	0.286317564	0.774634907	0.865083113
ENSG00000139687	339.2031645	0.245646882	0.103527841	2.37276155	0.017655658	0.052179471
ENSG00000139697	1354.713444	0.428817902	0.070454404	6.086459864	1.15E-09	1.59E-08
ENSG00000139718	782.2121241	-0.090531536	0.07890221	-1.147389098	0.251220845	0.415555724
ENSG00000139719	89.19094297	-0.285263333	0.200898639	-1.419936609	0.155626137	0.293122149
ENSG00000139722	157.9922486	0.00097882	0.159299778	0.006144514	0.995097418	0.997939133
ENSG00000139725	239.6991276	-0.26821691	0.124227555	-2.159077424	0.030844161	0.08240166
ENSG00000139726	510.2865441	0.320860572	0.088566608	3.622816556	0.000291412	0.001499989
ENSG00000139734	364.6817551	-0.169827332	0.102625545	-1.654825134	0.097959974	0.20636529
ENSG00000139746	1531.995459	0.152876894	0.066174836	2.310196811	0.020877261	0.059812947
ENSG00000139793	166.2298954	-0.01144431	0.14584109	-0.078471092	0.937453324	0.965779747
ENSG00000139800	9.895909037	-3.050986146	0.750234073	-4.066712316	4.77E-05	0.000295185
ENSG00000139826	88.69019199	0.032034828	0.193639574	0.165435334	0.868601344	0.926794006
ENSG00000139832	15.38158372	0.409114381	0.46206457	0.885405218	0.375938119	0.545875828
ENSG00000139835	95.79832192	0.202811139	0.186722829	1.08616145	0.277407569	0.446004822
ENSG00000139842	858.5383973	-0.047578982	0.079710905	-0.596894265	0.550577976	0.698389817
ENSG00000139865	9.468608941	-0.58184444	0.55920228	-1.040490107	0.298112258	0.468750272
ENSG00000139880	103.5861126	0.235555422	0.173545997	1.3573083	0.174683271	0.319086594

ENSG00000139899	29.00524668	-0.263958124	0.325687811	-0.810463626	0.417673763	0.58565216
ENSG00000139910	211.6282692	1.083230521	0.137686694	7.867358041	3.62E-15	8.79E-14
ENSG00000139921	203.8916571	0.119501801	0.139946868	0.853908361	0.393155767	0.561290936
ENSG00000139926	125.2186086	-0.393220151	0.168290661	-2.336553606	0.019462407	0.056473546
ENSG00000139971	30.14498385	0.054202023	0.33258098	0.16297391	0.870538977	0.927945136
ENSG00000139973	11.50137932	-2.404126485	0.613743992	-3.917148707	8.96E-05	0.000521394
ENSG00000139974	45.47880233	-0.653636678	0.260303222	-2.511058733	0.012036967	0.03777553
ENSG00000139977	132.86478	0.047659599	0.159459823	0.298881549	0.765030426	0.858474388
ENSG00000139990	768.7960714	-0.651488844	0.082809321	-7.867337144	3.62E-15	8.79E-14
ENSG00000139998	134.0537676	0.578267971	0.156770614	3.688624778	0.000225469	0.001190665
ENSG00000140006	72.77162774	9.30E-05	0.206808467	0.000449855	0.999641068	0.999854605
ENSG00000140025	29.52940612	-0.81371977	0.356035652	-2.285500804	0.022283479	0.062905151
ENSG00000140043	56.7217275	-0.617384401	0.256142599	-2.410315207	0.015938743	0.047963052
ENSG00000140044	43.62335703	-2.947488846	0.363263478	-8.113914629	4.90E-16	1.28E-14
ENSG00000140057	34.71486644	-0.874362339	0.335227233	-2.608267622	0.009100178	0.029797248
ENSG00000140090	34.25757442	-0.57961558	0.306352673	-1.891987995	0.05849258	0.137859946
ENSG00000140092	17.47103942	-1.611832806	0.483913944	-3.330825299	0.000865889	0.003947792
ENSG00000140104	38.10346012	-0.046910931	0.289247757	-0.162182524	0.871162122	0.928186766
ENSG00000140105	709.3448729	-0.783612161	0.085875146	-9.12501694	7.17E-20	2.40E-18
ENSG00000140153	172.7513145	-0.029999813	0.148045701	-0.202638865	0.839417311	0.906604257
ENSG00000140157	371.3744619	-0.036940064	0.103949684	-0.355364856	0.722316273	0.827735087
ENSG00000140199	166.2701774	-0.184355044	0.14361539	-1.283671923	0.199256772	0.351186936
ENSG00000140259	369.9080698	0.006705828	0.105910559	0.06331595	0.949514915	0.972060789
ENSG00000140262	758.4576492	-0.015187397	0.081160348	-0.187128292	0.851560046	0.914865313
ENSG00000140263	178.3151872	0.497366724	0.137511782	3.616902622	0.000298149	0.001530181
ENSG00000140264	542.235303	0.123261555	0.179313338	0.687408734	0.491825195	0.651295106
ENSG00000140265	153.1922274	-0.461067983	0.152265579	-3.028051287	0.002461363	0.009761369
ENSG00000140280	31.86584134	0.328951633	0.319729512	1.02884351	0.303553217	0.474358887
ENSG00000140284	110.1059816	0.605787025	0.174254344	3.476452924	0.000508093	0.002456018
ENSG00000140299	380.2057627	-0.253094111	0.10471577	-2.41696271	0.01565062	0.047207272
ENSG00000140307	206.0402101	-0.079720663	0.129379839	-0.616175315	0.537778819	0.688558843
ENSG00000140319	1517.316435	0.229531783	0.083347943	2.753898584	0.005889001	0.020553444
ENSG00000140320	818.8011031	0.25353072	0.082438186	3.075403935	0.002102176	0.00851233
ENSG00000140323	18.3272997	-0.133021399	0.405475117	-0.328063039	0.742864	0.842824538
ENSG00000140326	114.02828	0.293095643	0.169865997	1.725452109	0.084446027	0.184194619
ENSG00000140332	466.5736827	-0.272794642	0.094985543	-2.871959613	0.004079351	0.015059827
ENSG00000140350	1424.656119	0.236695358	0.070386562	3.362791877	0.000771585	0.003564109
ENSG00000140365	588.846952	0.339092351	0.10077519	3.364839615	0.000765882	0.003538927
ENSG00000140367	170.553359	-0.111555545	0.142473294	-0.782992666	0.433631419	0.59982477
ENSG00000140368	12.27702876	3.209224512	0.662366315	4.845090154	1.27E-06	1.10E-05
ENSG00000140374	355.6010231	-0.218664053	0.109705424	-1.993192721	0.046240348	0.11461764
ENSG00000140382	440.2013689	-0.070655766	0.093817344	-0.753120516	0.451377497	0.616540227
ENSG00000140386	301.5567457	-0.292670808	0.109986318	-2.660974681	0.007791482	0.026152196
ENSG00000140391	865.0900007	-0.10520967	0.084473608	-1.245473856	0.21295762	0.36831023
ENSG00000140395	608.4510982	0.495800513	0.085483593	5.799949423	6.63E-09	8.39E-08
ENSG00000140396	645.0326415	-0.297339993	0.083876444	-3.544976148	0.000392649	0.001952402
ENSG00000140398	90.48727849	0.254909059	0.187724304	1.357890556	0.174498414	0.318907337
ENSG00000140400	324.3231453	0.290008381	0.117782046	2.462246087	0.01380699	0.042381686
ENSG00000140406	378.9169109	0.188324679	0.10209318	1.844635247	0.065090616	0.15030871
ENSG00000140416	361.6118454	-1.320251525	0.110336903	-11.96563877	5.38E-33	3.26E-31

ENSG00000140443	1490.974084	-0.672041025	0.064968584	-10.34409227	4.45E-25	1.99E-23
ENSG00000140450	152.2632168	-1.611988879	0.162633373	-9.911796385	3.70E-23	1.46E-21
ENSG00000140451	110.3811374	-0.233967893	0.184649157	-1.267094288	0.205121578	0.358643615
ENSG00000140455	286.5315932	-0.206296079	0.119351608	-1.728473399	0.083903386	0.183409721
ENSG00000140463	141.1623359	-0.241267846	0.163283986	-1.477596498	0.139515812	0.270314292
ENSG00000140464	535.0831203	0.617499959	0.089177279	6.924409029	4.38E-12	8.10E-11
ENSG00000140465	13.22134795	-3.080781409	0.656197645	-4.69489861	2.67E-06	2.17E-05
ENSG00000140471	90.80595023	0.097464825	0.186689856	0.522068139	0.601622889	0.739822877
ENSG00000140474	191.5330592	0.636525634	0.14719469	4.324379047	1.53E-05	0.000105275
ENSG00000140479	368.1171581	0.144957136	0.113085249	1.281839466	0.199898967	0.351965503
ENSG00000140481	31.45549488	-2.013019295	0.355444924	-5.663378929	1.48E-08	1.78E-07
ENSG00000140497	379.6163008	0.200483244	0.103291302	1.940949912	0.052264353	0.126274057
ENSG00000140511	105.7190759	-3.006462847	0.243021272	-12.37119212	3.74E-35	2.46E-33
ENSG00000140521	789.4237046	0.154273291	0.085918132	1.795584784	0.072560578	0.163316525
ENSG00000140525	1118.45841	-0.063426558	0.073588304	-0.861910861	0.388736572	0.557259172
ENSG00000140526	373.3574332	-0.580444478	0.106225979	-5.464242196	4.65E-08	5.17E-07
ENSG00000140534	791.7885912	-0.082152547	0.081450747	-1.008616243	0.313158714	0.484304796
ENSG00000140543	22.52271218	-0.415306151	0.372939478	-1.113602006	0.265449966	0.432171496
ENSG00000140545	1456.949161	-1.756517561	0.085569075	-20.527481	1.22E-93	2.82E-91
ENSG00000140548	204.37095	0.027054043	0.136360876	0.198400334	0.842731856	0.908748678
ENSG00000140553	760.955124	0.373023483	0.077165453	4.834073634	1.34E-06	1.15E-05
ENSG00000140564	542.0528529	-0.091536767	0.089299125	-1.025057824	0.305335904	0.476296885
ENSG00000140575	632.9149485	0.022337374	0.090628438	0.246472014	0.805316866	0.884947666
ENSG00000140577	563.9413403	0.256840464	0.085512184	3.003554039	0.002668462	0.010449925
ENSG00000140598	183.5667428	0.103937855	0.134565536	0.772395798	0.439880042	0.605131226
ENSG00000140600	18.64062728	-0.224666977	0.40292986	-0.557583339	0.577128936	0.721000548
ENSG00000140612	1102.55462	0.156986615	0.071077749	2.208660466	0.027198266	0.074372989
ENSG00000140632	890.8982207	-0.026974843	0.074906115	-0.36011537	0.71876086	0.824903181
ENSG00000140650	415.5523841	0.316680709	0.101527576	3.119159569	0.001813677	0.007515257
ENSG00000140682	166.7885161	-0.413192933	0.142112197	-2.907512109	0.003643162	0.013654083
ENSG00000140688	303.1674846	0.312999246	0.119860902	2.611353993	0.009018449	0.029577901
ENSG00000140691	53.75956756	-0.094309325	0.245387892	-0.384327539	0.700735699	0.812215064
ENSG00000140694	374.061667	0.033806281	0.115811124	0.291908754	0.77035639	0.862680103
ENSG00000140718	394.4537614	-0.026183933	0.10419851	-0.251288942	0.801590724	0.881749796
ENSG00000140740	1366.009516	0.027208885	0.076176495	0.357182156	0.720955444	0.826715193
ENSG00000140743	148.7731703	0.274126529	0.15016496	1.825502628	0.067925215	0.155524938
ENSG00000140750	1038.585904	0.516366607	0.073878161	6.98943504	2.76E-12	5.22E-11
ENSG00000140795	6.025220016	-3.329482871	1.005857492	-3.310094023	0.000932646	0.004224729
ENSG00000140807	124.9345751	-0.316560996	0.162576263	-1.947153847	0.051516293	0.124730741
ENSG00000140829	1016.264164	0.676362456	0.080301867	8.422748825	3.68E-17	1.02E-15
ENSG00000140830	120.0581901	0.866776895	0.167846971	5.164090172	2.42E-07	2.40E-06
ENSG00000140836	1857.583958	-0.357771156	0.066227475	-5.402156093	6.58E-08	7.15E-07
ENSG00000140848	83.38479381	-0.571124901	0.196790395	-2.90219907	0.00370553	0.013832468
ENSG00000140853	112.4260304	1.637174279	0.186069706	8.798714828	1.38E-18	4.40E-17
ENSG00000140854	249.0292984	-0.225376536	0.12813389	-1.758914339	0.078592059	0.174105781
ENSG00000140859	148.6936659	0.28717974	0.148213607	1.937607121	0.052671173	0.127038456
ENSG00000140905	19.50993605	0.116558401	0.394492042	0.295464517	0.767639055	0.860096179
ENSG00000140931	182.9994992	0.234861698	0.139942973	1.678267176	0.093294953	0.198773579
ENSG00000140939	174.512576	1.203473065	0.153516745	7.839360217	4.53E-15	1.09E-13
ENSG00000140941	340.0826775	-0.650709341	0.109307041	-5.953041378	2.63E-09	3.47E-08

ENSG00000140943	643.3308193	0.105643716	0.083305415	1.268149444	0.204744587	0.358155148
ENSG00000140948	483.8530165	-1.018155193	0.095916257	-10.61504302	2.54E-26	1.20E-24
ENSG00000140950	85.75646564	0.124875698	0.197995645	0.630699217	0.528237211	0.680934946
ENSG00000140961	42.9168572	0.495339347	0.275847881	1.795697485	0.072542642	0.163302323
ENSG00000140983	247.0876134	0.117778597	0.12027381	0.979253895	0.327454545	0.499810278
ENSG00000140987	165.9116121	0.229579596	0.141761459	1.619478225	0.105344408	0.218402811
ENSG00000140988	7514.667729	0.235537827	0.065205787	3.612222749	0.000303584	0.001555229
ENSG00000140990	1537.834894	0.405270137	0.07523243	5.386907454	7.17E-08	7.69E-07
ENSG00000140992	223.3245234	-0.013743251	0.124755888	-0.110161141	0.91228158	0.952987235
ENSG00000140993	17.28592883	0.008470135	0.422844101	0.020031343	0.98401837	0.991961125
ENSG00000140995	203.7282009	0.236389489	0.130637326	1.809509547	0.070371878	0.159695279
ENSG00000141002	648.2777001	0.400722107	0.085264584	4.69974857	2.60E-06	2.12E-05
ENSG00000141012	176.2691106	-1.54244838	0.153590267	-10.04261797	9.90E-24	4.07E-22
ENSG00000141013	273.2195503	-0.172681257	0.12487042	-1.382883601	0.166700559	0.308528087
ENSG00000141026	98.17606194	0.09454837	0.190276401	0.49690014	0.619259473	0.75281158
ENSG00000141027	2400.626771	-0.101303133	0.060862009	-1.664472383	0.096018066	0.203249814
ENSG00000141030	376.1565869	0.03958302	0.101098214	0.391530355	0.695405256	0.808571942
ENSG00000141034	135.4074519	0.155006394	0.166520132	0.930856778	0.351927655	0.523567863
ENSG00000141068	148.1879481	-0.252771582	0.150135529	-1.683622681	0.092254613	0.197035205
ENSG00000141076	317.2317297	0.250193424	0.119066494	2.101291593	0.035615377	0.092749204
ENSG00000141084	269.9622918	-0.16382374	0.121092186	-1.352884489	0.176092535	0.320951323
ENSG00000141098	163.9245634	0.041572322	0.144351149	0.287994395	0.773351029	0.864290072
ENSG00000141101	724.1858132	0.336153565	0.089063001	3.774334579	0.000160435	0.00087861
ENSG00000141127	195.5254878	0.397807261	0.13052447	3.047760011	0.00230554	0.009221502
ENSG00000141179	30.54523859	-0.902618996	0.322179051	-2.801606721	0.005084882	0.018156415
ENSG00000141198	246.8179311	0.464380936	0.12310331	3.772286362	0.000161758	0.000885166
ENSG00000141219	265.5475905	0.245997761	0.114204472	2.154011629	0.03123926	0.08322857
ENSG00000141232	987.5828484	-0.138484816	0.085719041	-1.615566545	0.106188062	0.219808975
ENSG00000141252	656.3856359	-0.090689401	0.083075863	-1.091645614	0.274988899	0.443384878
ENSG00000141258	293.9693743	0.068088245	0.123688129	0.550483266	0.581987952	0.724300944
ENSG00000141279	707.659398	0.186112038	0.087574436	2.125186831	0.033571026	0.088292868
ENSG00000141293	23.72778639	-4.680828757	0.706944106	-6.621214774	3.56E-11	5.96E-10
ENSG00000141295	81.27881689	-0.044271025	0.208749962	-0.212076806	0.832047114	0.901764202
ENSG00000141298	420.3790054	0.0967394	0.096051885	1.007157746	0.313858975	0.485067336
ENSG00000141314	27.91729832	0.782887851	0.331469413	2.361870567	0.018182989	0.053467961
ENSG00000141337	86.25272771	-0.144552163	0.201309909	-0.718057861	0.472721611	0.633981332
ENSG00000141338	9.562038739	-5.531761444	1.263152165	-4.379331008	1.19E-05	8.39E-05
ENSG00000141349	253.7328647	0.081819109	0.117246511	0.697838331	0.485278304	0.645643525
ENSG00000141367	4126.029341	0.094359844	0.058378797	1.616337584	0.106021345	0.219528572
ENSG00000141376	806.4717416	0.259822579	0.07808935	3.327247283	0.000877085	0.003996242
ENSG00000141378	325.2173939	0.211380893	0.115804344	1.825327832	0.067951573	0.155542916
ENSG00000141380	1387.611015	-0.261728356	0.085449297	-3.062966763	0.002191544	0.008818281
ENSG00000141384	94.7885279	0.794889354	0.191202576	4.157315084	3.22E-05	0.000207015
ENSG00000141385	584.1379609	-0.109353437	0.08734168	-1.252018923	0.210562969	0.365654348
ENSG00000141391	30.64425871	-0.178927359	0.323289062	-0.553459365	0.579948898	0.723108661
ENSG00000141401	366.4120523	-0.343150072	0.103900461	-3.302680948	0.000957653	0.004324059
ENSG00000141404	261.4027351	-0.460635963	0.117926471	-3.906128625	9.38E-05	0.00054349
ENSG00000141424	536.546691	0.332477274	0.090700742	3.665651093	0.00024671	0.001290703
ENSG00000141425	592.2956192	0.058837622	0.090569163	0.649642996	0.515922853	0.671096242
ENSG00000141428	37.45138379	-0.07803316	0.285293966	-0.273518438	0.784454721	0.871011269

ENSG00000141429	464.2313322	0.07099029	0.102362358	0.693519488	0.4879836	0.647922265
ENSG00000141446	298.7596874	-0.15910466	0.111926278	-1.421513017	0.155167672	0.292709201
ENSG00000141447	370.3120249	0.152018887	0.104149506	1.459621782	0.144394049	0.277272848
ENSG00000141449	243.0255968	-0.662980154	0.136749408	-4.848139129	1.25E-06	1.08E-05
ENSG00000141452	125.173381	0.327894357	0.159672958	2.053537192	0.040020507	0.101860492
ENSG00000141456	1019.275312	0.290139555	0.080357956	3.610589037	0.000305502	0.001564489
ENSG00000141458	491.1287179	0.131092887	0.089365502	1.46692946	0.142395245	0.274379425
ENSG00000141480	837.435715	0.550815748	0.076001813	7.247402763	4.25E-13	8.79E-12
ENSG00000141497	16.08333493	0.691294412	0.439766475	1.571957964	0.1159603	0.235114293
ENSG00000141499	153.8643268	-0.069600344	0.16547303	-0.42061443	0.674036655	0.792450025
ENSG00000141503	698.1733162	-0.217361933	0.080572288	-2.697725713	0.006981494	0.023739783
ENSG00000141504	359.7011041	-0.141458748	0.102226727	-1.3837746	0.166427483	0.308255123
ENSG00000141505	21.84607978	-0.814900633	0.373788713	-2.180110326	0.029249285	0.078830526
ENSG00000141506	21.71631151	1.037010641	0.375122671	2.764457393	0.005701755	0.019968227
ENSG00000141510	723.3538281	-0.241269804	0.087167208	-2.767896435	0.005641938	0.019798228
ENSG00000141519	47.92251451	-0.61266286	0.259233787	-2.363360377	0.018110049	0.053298107
ENSG00000141522	2113.931246	0.566240387	0.083105735	6.813493543	9.53E-12	1.69E-10
ENSG00000141524	73.84829593	-0.749258342	0.208890803	-3.586842181	0.000334707	0.001693059
ENSG00000141526	452.2149554	0.588823457	0.101721074	5.788608309	7.10E-09	8.91E-08
ENSG00000141540	126.5725162	0.096589997	0.161717062	0.597277712	0.550321982	0.698254257
ENSG00000141542	196.7880298	0.02441278	0.138133619	0.176733084	0.859718053	0.920041101
ENSG00000141543	515.2564547	0.22193452	0.102132376	2.173008482	0.029779679	0.079968486
ENSG00000141551	854.4764682	0.042903709	0.076965681	0.557439478	0.577227199	0.721059179
ENSG00000141552	225.0506972	0.127837332	0.126240365	1.012650211	0.311227268	0.482646216
ENSG00000141556	1173.059396	-0.537219756	0.08725884	-6.156622723	7.43E-10	1.05E-08
ENSG00000141560	155.9768906	-0.560036987	0.155928372	-3.591629785	0.000328616	0.001665251
ENSG00000141562	278.6721136	-0.217135303	0.118022286	-1.83978222	0.065800217	0.151598433
ENSG00000141564	865.9558719	-0.040933522	0.075354187	-0.543214963	0.586981806	0.728901469
ENSG00000141568	2060.909652	-0.488408407	0.066442326	-7.350862619	1.97E-13	4.20E-12
ENSG00000141569	388.0809205	0.050873829	0.120639645	0.421700751	0.673243456	0.791795205
ENSG00000141570	326.9854714	0.338558729	0.124065527	2.72887027	0.00635517	0.021890896
ENSG00000141576	60.44373642	-3.023499979	0.305417046	-9.899578372	4.18E-23	1.64E-21
ENSG00000141577	335.5423123	0.009290868	0.10926041	0.085034169	0.932234226	0.96324653
ENSG00000141579	9.56952399	0.261558832	0.564179183	0.463609505	0.642927543	0.769595501
ENSG00000141580	589.6126109	-0.086337532	0.086791341	-0.994771267	0.319847524	0.491564351
ENSG00000141582	410.8849973	0.221637441	0.104059681	2.12990699	0.033179293	0.087475512
ENSG00000141627	325.986677	-0.23244785	0.116076562	-2.002539065	0.045226786	0.112641961
ENSG00000141642	21.28625686	-0.233190114	0.371676635	-0.627400519	0.530396727	0.68262236
ENSG00000141644	381.0816145	0.050307666	0.098735226	0.509520948	0.610387119	0.746613363
ENSG00000141646	344.6034205	-0.106393922	0.104025718	-1.022765564	0.306418703	0.47752037
ENSG00000141655	8.850907137	0.570334596	0.590131034	0.966454164	0.333816972	0.505948102
ENSG00000141664	392.0020827	0.171718854	0.099053636	1.733594654	0.08299003	0.181752564
ENSG00000141668	9.174366599	-2.745507839	0.767815044	-3.575741139	0.000349237	0.00176022
ENSG00000141696	599.2090248	0.103632806	0.085986561	1.205220962	0.228118012	0.3869082
ENSG00000141698	293.138999	0.381635582	0.114198386	3.341864953	0.000832175	0.003812644
ENSG00000141699	174.8408632	-0.028476376	0.144979173	-0.196417011	0.844283783	0.90976923
ENSG00000141736	462.476883	-0.089609052	0.097968692	-0.914670293	0.360364744	0.531671417
ENSG00000141738	21.97264638	-0.356740647	0.368783429	-0.967344569	0.33337181	0.505655309
ENSG00000141741	155.6655836	0.096530836	0.148987358	0.647912934	0.517041264	0.671928822
ENSG00000141750	63.07024693	-1.008007843	0.238222749	-4.231366841	2.32E-05	0.000153904

ENSG00000141753	121.9228008	-0.399419188	0.171357468	-2.330912057	0.019757997	0.057189489
ENSG00000141756	2323.566066	0.320420725	0.06444174	4.972254375	6.62E-07	6.11E-06
ENSG00000141759	731.7006223	0.216436566	0.084232051	2.569527431	0.010183733	0.032784677
ENSG00000141837	257.2694586	4.409561452	0.195959233	22.50244294	3.93E-112	1.20E-109
ENSG00000141854	29.99646983	0.547651732	0.315071183	1.738184137	0.082178373	0.180368689
ENSG00000141858	678.2723052	0.51558125	0.086871183	5.935008972	2.94E-09	3.84E-08
ENSG00000141867	2921.477623	0.074427998	0.063773695	1.16706422	0.243184436	0.406039673
ENSG00000141873	198.2296755	-0.271419149	0.144306411	-1.88085302	0.059991916	0.140850148
ENSG00000141905	3172.966751	0.095336544	0.055982195	1.702979753	0.088571857	0.190911291
ENSG00000141933	45.87793935	0.495766298	0.260560324	1.90269297	0.05708062	0.135235532
ENSG00000141956	269.396896	0.28500252	0.118053716	2.414176618	0.015770816	0.047539195
ENSG00000141959	374.2466122	0.062144363	0.111137015	0.559168899	0.576046456	0.720288817
ENSG00000141965	553.5622034	0.212616292	0.097878196	2.172253876	0.029836519	0.080097953
ENSG00000141971	190.9057025	0.147543793	0.138232394	1.067360475	0.285809089	0.455498103
ENSG00000141985	856.2336532	0.223322949	0.079681119	2.802708503	0.005067545	0.018103714
ENSG00000141994	191.6072901	0.20925105	0.14109457	1.483055301	0.138059719	0.268158859
ENSG00000142002	510.6678687	0.347481866	0.090760333	3.828565333	0.000128892	0.000723642
ENSG00000142039	261.4117662	-0.123071793	0.131823789	-0.933608372	0.350505945	0.522448749
ENSG00000142046	18.30999691	0.106360895	0.426013114	0.249665778	0.802845826	0.882853868
ENSG00000142065	56.6186731	-0.348437768	0.236977432	-1.47034156	0.14146927	0.273044633
ENSG00000142082	108.9076417	-0.240682539	0.170412579	-1.412351952	0.157846356	0.29603041
ENSG00000142089	1837.944558	0.762831333	0.067894233	11.23558355	2.73E-29	1.43E-27
ENSG00000142102	412.7183205	0.80724367	0.105203871	7.673136535	1.68E-14	3.90E-13
ENSG00000142156	1482.790918	0.495251699	0.088620529	5.588453404	2.29E-08	2.67E-07
ENSG00000142166	249.5979642	0.016722203	0.120262591	0.139047418	0.889412679	0.939574317
ENSG00000142168	961.7478244	0.217028418	0.075877104	2.860262273	0.004232908	0.015528769
ENSG00000142173	268.1236466	-0.498755707	0.141902523	-3.51477689	0.000440124	0.002164627
ENSG00000142178	8.722800256	0.671401432	0.589210097	1.139494104	0.254497126	0.41954244
ENSG00000142186	829.7022231	0.383494282	0.080781028	4.747331056	2.06E-06	1.71E-05
ENSG00000142188	112.5866346	-0.095837818	0.1685591	-0.568571013	0.569647301	0.714810407
ENSG00000142192	4518.0418	0.28702864	0.060387961	4.753077185	2.00E-06	1.67E-05
ENSG00000142197	105.8943063	0.233924883	0.181270847	1.29047162	0.196886959	0.348145911
ENSG00000142207	649.7854371	0.212559641	0.084090146	2.527759221	0.011479305	0.036276669
ENSG00000142208	952.0230642	-0.133333394	0.080900893	-1.648107801	0.099330552	0.208626833
ENSG00000142227	591.7433954	0.216055641	0.1073158	2.013269635	0.044086271	0.110526477
ENSG00000142230	848.4429283	-0.054188901	0.082461162	-0.657144517	0.511087997	0.667402909
ENSG00000142235	70.04818309	-0.075558946	0.207955648	-0.363341641	0.716349691	0.823449162
ENSG00000142252	79.21850062	0.353595808	0.198783169	1.778801544	0.075272304	0.16793997
ENSG00000142279	157.8070708	-0.068080851	0.149339001	-0.455881257	0.648475369	0.773796594
ENSG00000142303	21.0444628	-0.661040042	0.426726698	-1.549094645	0.12135897	0.244084244
ENSG00000142327	209.4167963	0.057082203	0.141148851	0.404411389	0.685910227	0.801843308
ENSG00000142330	108.9191897	-0.169512477	0.185059449	-0.915989312	0.359672501	0.530984721
ENSG00000142396	101.8678008	0.268958608	0.174910592	1.537691942	0.124123955	0.248159578
ENSG00000142408	692.1408058	0.31240277	0.097573753	3.20170906	0.001366149	0.005899261
ENSG00000142409	210.6483741	0.24032812	0.133933856	1.794379167	0.07275267	0.163664987
ENSG00000142444	68.49879237	-0.003096735	0.21141531	-0.014647637	0.988313294	0.994116494
ENSG00000142453	1270.956415	0.397674474	0.072841031	5.459484424	4.78E-08	5.30E-07
ENSG00000142459	84.25835557	-0.466609622	0.192385197	-2.425392535	0.015291842	0.046264163
ENSG00000142494	143.5193139	0.275579533	0.151163416	1.823057062	0.068294749	0.156065778
ENSG00000142507	491.6701327	0.626989088	0.090985905	6.89105735	5.54E-12	1.01E-10

ENSG00000142528	136.5587067	0.172982172	0.15396319	1.12352941	0.261212717	0.427353254
ENSG00000142530	11.65005691	0.282606917	0.509513778	0.554660009	0.579127235	0.722533108
ENSG00000142534	12954.5295	0.112869528	0.079427652	1.42103569	0.155306384	0.292752117
ENSG00000142538	97.96698833	-1.678891241	0.197260802	-8.5110231	1.72E-17	4.94E-16
ENSG00000142541	4574.884096	0.16570662	0.05713782	2.900121506	0.00373018	0.013906009
ENSG00000142544	30.06890993	0.767497395	0.323034947	2.375895867	0.017506403	0.05185838
ENSG00000142546	214.0476459	0.082395515	0.128244976	0.642485325	0.520558114	0.674960491
ENSG00000142552	97.05454523	0.480713957	0.185079278	2.597340789	0.009394866	0.030633632
ENSG00000142556	55.30897999	0.170260925	0.239818626	0.709957052	0.477730769	0.638134946
ENSG00000142599	1683.521684	0.096056584	0.067508742	1.42287623	0.154772038	0.292054868
ENSG00000142611	20.46312361	0.963934464	0.392254113	2.457423473	0.013993762	0.042853799
ENSG00000142627	392.4506948	0.148178912	0.100840907	1.469432554	0.141715503	0.273332098
ENSG00000142634	366.3535498	0.516672599	0.112769754	4.581659339	4.61E-06	3.55E-05
ENSG00000142655	222.1278669	-0.027942596	0.13275785	-0.210477921	0.833294682	0.902386945
ENSG00000142657	1904.465501	0.000208481	0.061343719	0.003398579	0.997288332	0.998813347
ENSG00000142669	369.9438187	0.139305671	0.107194002	1.299565906	0.193749791	0.344593988
ENSG00000142675	10.16927022	0.179972481	0.555693611	0.323869985	0.746036469	0.845126958
ENSG00000142676	7605.15253	0.273868278	0.057311469	4.77859461	1.77E-06	1.48E-05
ENSG00000142684	144.1116004	0.29254119	0.171525833	1.705522632	0.088096995	0.190033552
ENSG00000142686	65.09572116	0.150085211	0.232014157	0.646879541	0.517709908	0.672673303
ENSG00000142687	302.7708784	-0.129838848	0.109698092	-1.18360169	0.236570771	0.39772109
ENSG00000142694	102.2330269	0.123496522	0.210916036	0.585524572	0.558195049	0.705122829
ENSG00000142731	214.7444478	-0.093503265	0.132453541	-0.705932538	0.480230092	0.640808597
ENSG00000142733	138.1715588	0.536440652	0.158682171	3.380598129	0.000723282	0.003366451
ENSG00000142751	145.3165575	-0.17741849	0.150884368	-1.175857332	0.239651892	0.401813096
ENSG00000142765	121.5889338	0.256193579	0.163661014	1.565391612	0.117491114	0.23774095
ENSG00000142784	215.5324351	-0.309723971	0.126530514	-2.447820387	0.014372328	0.043879177
ENSG00000142794	88.21662319	-0.058756843	0.193830834	-0.303134653	0.761787247	0.856271541
ENSG00000142798	1161.081625	-0.544781298	0.077876035	-6.995493505	2.64E-12	5.03E-11
ENSG00000142856	209.5291692	-0.068573417	0.128335283	-0.534330197	0.593113104	0.73320952
ENSG00000142864	3844.423753	0.112311875	0.055588513	2.020415155	0.043340343	0.109104264
ENSG00000142867	78.13357554	-0.209968691	0.204421198	-1.027137564	0.304355693	0.475348501
ENSG00000142871	519.9009492	-0.832896587	0.096527942	-8.628554292	6.21E-18	1.84E-16
ENSG00000142875	81.36097978	-0.272198072	0.202289394	-1.345587462	0.178435597	0.32412839
ENSG00000142892	149.6529445	-0.08943358	0.147036181	-0.608241995	0.543026986	0.692162425
ENSG00000142937	5935.391203	0.113193445	0.071614002	1.580604937	0.113968396	0.232077311
ENSG00000142945	484.8479437	-0.217560406	0.099004095	-2.197488973	0.027985543	0.076125663
ENSG00000142949	553.8291021	0.087646298	0.096098209	0.912049237	0.361742799	0.532534336
ENSG00000142961	65.16313288	0.135667516	0.226311522	0.59947242	0.548857897	0.697089229
ENSG00000143013	911.4614072	-2.082150615	0.084358215	-24.68225066	1.66E-134	7.06E-132
ENSG00000143028	267.9294212	-0.241243165	0.117298543	-2.056659514	0.039718989	0.101313354
ENSG00000143033	305.0991106	-0.324447441	0.108428135	-2.992280921	0.002769013	0.010780579
ENSG00000143061	35.71390706	-1.36865449	0.307426182	-4.451977649	8.51E-06	6.20E-05
ENSG00000143067	83.34842412	-0.129085477	0.195298188	-0.660966076	0.508634069	0.66549765
ENSG00000143079	158.4743915	-0.110620194	0.144906602	-0.763389604	0.445231078	0.610325858
ENSG00000143093	259.0104956	0.167525839	0.120614254	1.388938982	0.164851305	0.305981275
ENSG00000143106	302.6535586	0.252789107	0.11370888	2.223125472	0.02620734	0.072168405
ENSG00000143126	417.4566726	-0.478403358	0.108093125	-4.425844446	9.61E-06	6.91E-05
ENSG00000143147	48.24681221	-0.92804746	0.260872945	-3.557469177	0.000374445	0.00187469
ENSG00000143149	214.1656207	-0.132957169	0.12811455	-1.037799139	0.299363578	0.469797808

ENSG00000143153	301.2698091	1.185222388	0.116682512	10.15766948	3.06E-24	1.29E-22
ENSG00000143155	440.0398	-0.056639371	0.097872722	-0.578704364	0.562788671	0.70902994
ENSG00000143156	51.32387713	0.136188588	0.246261606	0.553024038	0.58024695	0.723159339
ENSG00000143157	769.0104901	0.264014821	0.079840082	3.306795454	0.000943698	0.004266534
ENSG00000143158	322.5218205	0.14190514	0.108647958	1.306100392	0.19151842	0.341750412
ENSG00000143162	304.5678673	0.414810307	0.111661694	3.714884592	0.000203296	0.00108582
ENSG00000143164	965.2079543	-0.020761688	0.076736986	-0.270556465	0.786732183	0.8723046
ENSG00000143179	1388.885117	-0.081471125	0.074538374	-1.093009162	0.274389777	0.442672929
ENSG00000143183	169.4614551	-0.009026863	0.142922556	-0.063159122	0.949639795	0.972060789
ENSG00000143190	1077.13302	-0.276553958	0.071210763	-3.883597722	0.000102922	0.000591066
ENSG00000143198	632.3546132	0.223616829	0.109706709	2.038314991	0.041518442	0.105291487
ENSG00000143207	239.1315832	-0.228447959	0.125862433	-1.815060731	0.069514577	0.158184232
ENSG00000143222	361.6862965	0.350127394	0.106133712	3.298927255	0.000970551	0.004369655
ENSG00000143224	83.5039533	0.272727657	0.19718082	1.383134818	0.166623531	0.30849621
ENSG00000143228	256.2258648	0.083497764	0.118140036	0.706769413	0.479709786	0.640321234
ENSG00000143248	13.88041884	-1.287029588	0.509810348	-2.524526212	0.011585436	0.036546289
ENSG00000143252	163.2726771	-0.143912926	0.144085074	-0.998805233	0.317889051	0.489733219
ENSG00000143256	722.4940344	0.394329419	0.086880183	4.538772911	5.66E-06	4.29E-05
ENSG00000143258	262.1580669	0.115345825	0.120698242	0.955654553	0.33924679	0.511070291
ENSG00000143294	1004.199888	0.308997706	0.074401	4.153139124	3.28E-05	0.000210445
ENSG00000143303	147.1138388	-0.564067141	0.15471193	-3.645918834	0.000266438	0.001382074
ENSG00000143314	448.7024479	0.467766227	0.101592439	4.604340936	4.14E-06	3.22E-05
ENSG00000143315	75.20434854	0.128711053	0.206212075	0.624168363	0.532517022	0.684003896
ENSG00000143319	595.0583476	-0.013227722	0.084556738	-0.156436047	0.875689326	0.931173956
ENSG00000143320	723.7078863	-1.956400836	0.089137689	-21.94807668	9.03E-107	2.59E-104
ENSG00000143321	2777.946845	0.202745939	0.057264588	3.540511638	0.000399352	0.001982225
ENSG00000143322	943.2699228	-0.247709211	0.073733634	-3.359514466	0.000780796	0.003601917
ENSG00000143324	538.1406708	-0.561268228	0.099041353	-5.667008869	1.45E-08	1.75E-07
ENSG00000143333	170.5987753	-0.389746782	0.146442201	-2.66143762	0.007780776	0.026128749
ENSG00000143337	692.7654139	0.236611057	0.093909371	2.51956812	0.01174989	0.036990298
ENSG00000143340	10.00292387	-4.07119107	0.937550071	-4.342371888	1.41E-05	9.79E-05
ENSG00000143344	104.1600456	-0.054296052	0.173519804	-0.312909825	0.754349173	0.851522246
ENSG00000143363	206.3535807	-0.108466228	0.134527866	-0.806273315	0.420085273	0.587797573
ENSG00000143367	125.6855564	-0.247480303	0.15823044	-1.564049897	0.117805851	0.238269304
ENSG00000143368	1001.674741	0.224842806	0.088640917	2.536557748	0.011194828	0.035486962
ENSG00000143369	76.45854567	-0.438840923	0.212621956	-2.063949234	0.03902253	0.099845078
ENSG00000143373	366.8083226	-0.271209305	0.114411182	-2.370478999	0.017765053	0.052436584
ENSG00000143374	557.2270548	-0.042405096	0.086226596	-0.491786738	0.622870113	0.755174907
ENSG00000143375	85.97035567	-1.765436898	0.211205477	-8.358859448	6.33E-17	1.75E-15
ENSG00000143376	894.4113147	-0.028998412	0.076628213	-0.378429972	0.705111206	0.815025133
ENSG00000143379	581.9120716	-0.0513769	0.084845142	-0.605537319	0.544822036	0.693593904
ENSG00000143382	374.0487224	-0.73716785	0.131861919	-5.590452934	2.26E-08	2.65E-07
ENSG00000143384	1920.430632	0.320094851	0.06453407	4.96009083	7.05E-07	6.46E-06
ENSG00000143387	71.19126444	-0.626216543	0.218492218	-2.866081672	0.00415587	0.015311554
ENSG00000143390	717.0151325	0.044781361	0.084263586	0.531443812	0.595111268	0.734903558
ENSG00000143393	833.8470547	0.043898065	0.082130964	0.534488616	0.593003523	0.733138575
ENSG00000143398	1830.624828	-0.075724681	0.065593234	-1.154458732	0.248312148	0.412635231
ENSG00000143401	1532.491853	-0.002286755	0.064930498	-0.035218507	0.971905505	0.985302874
ENSG00000143409	167.1741505	-0.059638652	0.140350395	-0.424926854	0.670889998	0.79003143
ENSG00000143418	1144.767021	0.242129733	0.077314604	3.131746413	0.001737699	0.007253925

ENSG00000143420	838.445903	-0.096722844	0.080001563	-1.209011927	0.226658263	0.385036718
ENSG00000143434	46.2982216	-1.11843963	0.27149516	-4.119556425	3.80E-05	0.000239887
ENSG00000143436	221.3602817	0.19361064	0.140039134	1.38254668	0.166803908	0.308626778
ENSG00000143437	739.6144775	0.080143404	0.084473964	0.948734969	0.342755424	0.514590639
ENSG00000143442	1992.963211	-0.228037444	0.076650432	-2.975031415	0.002929585	0.011302637
ENSG00000143443	124.6830502	0.514277547	0.166668594	3.085629594	0.002031217	0.008277489
ENSG00000143457	166.7081132	-0.002806724	0.139644194	-0.020099109	0.983964311	0.991961125
ENSG00000143458	207.0388691	0.43372372	0.134319193	3.229052451	0.001242011	0.005424916
ENSG00000143476	628.7214018	-0.417794208	0.088994524	-4.694605809	2.67E-06	2.17E-05
ENSG00000143479	297.9015948	0.459785502	0.11789908	3.899822647	9.63E-05	0.000556007
ENSG00000143486	360.5865532	0.304145735	0.105681339	2.877951183	0.004002671	0.014812872
ENSG00000143493	268.4506294	-0.328983543	0.137302283	-2.396052975	0.016572695	0.049520665
ENSG00000143494	190.37286	0.463109031	0.138721239	3.338414759	0.000842579	0.003852768
ENSG00000143498	71.77153361	-0.057726762	0.211093598	-0.273465243	0.784495607	0.871011269
ENSG00000143499	59.99171789	0.226760087	0.226416147	1.001519062	0.316575929	0.488192126
ENSG00000143514	1153.325341	-0.146966591	0.07444042	-1.974284826	0.048349365	0.118901179
ENSG00000143515	935.6364048	0.322229312	0.078639558	4.097547353	4.18E-05	0.000261495
ENSG00000143537	1120.063538	0.694803511	0.086692031	8.014617966	1.10E-15	2.83E-14
ENSG00000143543	45.35468048	0.793409234	0.287085321	2.763670504	0.005715522	0.020011451
ENSG00000143545	496.0440008	0.34953198	0.095012205	3.678811386	0.000234323	0.001230483
ENSG00000143549	2199.131896	0.08692335	0.06748009	1.28813329	0.197699569	0.34921957
ENSG00000143553	115.6284033	0.074740383	0.174927884	0.427263973	0.66918707	0.788596542
ENSG00000143554	45.43524735	-0.198034916	0.278225609	-0.711778174	0.47660215	0.63753861
ENSG00000143569	5869.841035	0.094222495	0.052707494	1.787648925	0.073832667	0.165384703
ENSG00000143570	16.6870271	0.481885805	0.429393949	1.122246381	0.261757697	0.428099137
ENSG00000143575	1072.656402	0.045474506	0.074991613	0.606394561	0.544252779	0.692994814
ENSG00000143578	53.53290088	-0.091129948	0.247088619	-0.368814835	0.712265746	0.820165336
ENSG00000143590	52.46409629	0.757531276	0.240370562	3.151514356	0.001624262	0.006845486
ENSG00000143603	16.39204254	-3.033980805	0.574171921	-5.284098188	1.26E-07	1.31E-06
ENSG00000143612	843.0461763	0.169025882	0.078485548	2.153592422	0.031272149	0.083272862
ENSG00000143614	1190.225064	0.044930349	0.070753273	0.635028556	0.525409791	0.678660352
ENSG00000143621	1383.086838	-0.027590348	0.087909417	-0.313849742	0.753635163	0.851126639
ENSG00000143622	163.0335858	-0.172133725	0.142774146	-1.205636521	0.227957671	0.386682938
ENSG00000143624	761.3691332	0.19146125	0.08147685	2.349885268	0.018779199	0.054830888
ENSG00000143630	98.34780817	0.056305149	0.180546209	0.311860045	0.755146888	0.852042366
ENSG00000143632	8.725428223	0.090407165	0.576284935	0.156879278	0.875339991	0.931034312
ENSG00000143633	184.9282836	-0.122489685	0.136652356	-0.896359848	0.370060604	0.541314309
ENSG00000143641	1105.776926	0.071827311	0.073702932	0.974551615	0.329782724	0.502217902
ENSG00000143643	306.73239	-0.082550991	0.125003286	-0.660390569	0.509003223	0.665670633
ENSG00000143653	661.6385767	0.180906618	0.085815335	2.108091956	0.035023036	0.091528528
ENSG00000143669	98.85375096	-0.144884244	0.181710358	-0.797336185	0.42525581	0.592557124
ENSG00000143702	689.5305905	0.245849226	0.081241648	3.026147696	0.002476913	0.009817493
ENSG00000143727	356.1616718	0.308622933	0.109873778	2.808886151	0.004971322	0.017818482
ENSG00000143740	184.6657546	-0.148664043	0.13501729	-1.101074115	0.270864402	0.438500861
ENSG00000143742	272.5780772	-0.122156046	0.115497283	-1.057652987	0.290213676	0.460480233
ENSG00000143748	241.0471218	-0.071044526	0.128617135	-0.552372168	0.580693395	0.723518357
ENSG00000143751	463.0984527	0.141658759	0.095310067	1.486293767	0.137201435	0.266871768
ENSG00000143753	442.8169125	0.168759527	0.098328493	1.716283061	0.086110247	0.18692484
ENSG00000143756	396.2876877	-0.289440642	0.11370754	-2.545483278	0.010912659	0.03474178
ENSG00000143761	2079.613798	0.100196454	0.061757146	1.622426887	0.104711976	0.217394196

ENSG00000143771	378.1422807	0.296137068	0.117094433	2.529044804	0.011437343	0.036168472
ENSG00000143772	441.9637855	0.469891845	0.100716344	4.665497438	3.08E-06	2.46E-05
ENSG00000143774	862.3553827	0.8069227	0.080132473	10.06985897	7.51E-24	3.11E-22
ENSG00000143776	1484.535686	0.017794836	0.071536481	0.248751913	0.80355269	0.883419725
ENSG00000143793	511.4006236	0.455911642	0.093061034	4.899060579	9.63E-07	8.56E-06
ENSG00000143797	168.9942933	-0.206189012	0.140159339	-1.471104344	0.141262901	0.272683793
ENSG00000143799	4069.216157	-0.016304379	0.053580669	-0.304295928	0.760902447	0.855550842
ENSG00000143801	37.50547819	-0.054069334	0.282833574	-0.191170139	0.848392297	0.912928344
ENSG00000143811	245.8960641	0.152226588	0.12510106	1.216828922	0.223669313	0.382038284
ENSG00000143815	632.8988276	-0.080562555	0.086497472	-0.931386247	0.351653802	0.523326725
ENSG00000143819	384.2659758	-0.17288774	0.106289449	-1.626574801	0.103827435	0.215867235
ENSG00000143842	87.77308987	-0.549514952	0.202155395	-2.718279926	0.006562229	0.022515788
ENSG00000143845	13.38953893	-1.906363229	0.537142903	-3.54908018	0.000386579	0.001926998
ENSG00000143847	146.2455075	0.932589082	0.154443244	6.038393508	1.56E-09	2.12E-08
ENSG00000143850	7.977421879	-2.457475283	0.735669611	-3.340460507	0.000836396	0.003830731
ENSG00000143862	290.0376918	0.453261929	0.115326835	3.930238167	8.49E-05	0.000498142
ENSG00000143870	3461.52226	0.747870258	0.064764096	11.54760585	7.59E-31	4.28E-29
ENSG00000143878	396.4853769	0.641680212	0.10468008	6.129917096	8.79E-10	1.23E-08
ENSG00000143889	257.5197354	-0.252727933	0.119711998	-2.111132873	0.034760893	0.09092854
ENSG00000143891	89.36923907	-0.305885768	0.189658226	-1.612826263	0.106782259	0.220811186
ENSG00000143919	103.6511183	-0.190490336	0.181318502	-1.050584106	0.293449644	0.463987641
ENSG00000143924	1269.233946	-0.144375703	0.076859025	-1.878448283	0.060319866	0.141454617
ENSG00000143933	1218.352499	0.022298327	0.075158141	0.296685445	0.76670667	0.85946282
ENSG00000143942	67.28887296	0.352566643	0.222901318	1.581716278	0.113714355	0.231768071
ENSG00000143947	2662.428748	0.233045875	0.068152253	3.419488915	0.000627389	0.00296532
ENSG00000143951	91.95002561	0.204763961	0.187895941	1.089773197	0.275813063	0.444254799
ENSG00000143952	180.4265478	-0.105978888	0.138536058	-0.764991368	0.444276685	0.609210718
ENSG00000143970	1910.438886	-0.034956407	0.063110037	-0.553896163	0.579649911	0.722800027
ENSG00000143971	147.5582671	-0.634308477	0.152459034	-4.160517493	3.18E-05	0.000204414
ENSG00000143977	484.2452633	0.490459166	0.114510382	4.283097826	1.84E-05	0.00012459
ENSG00000143995	1086.625283	0.229661685	0.073110054	3.141314659	0.001681912	0.007052856
ENSG00000144021	735.1539778	0.255468042	0.084622488	3.018914333	0.002536823	0.010006949
ENSG00000144026	46.14097474	0.070350825	0.264611731	0.265864348	0.790343683	0.874789829
ENSG00000144028	4059.977303	0.074692728	0.058967518	1.266675794	0.205271239	0.358682061
ENSG00000144029	134.9514553	0.511106404	0.164245459	3.111844956	0.001859221	0.007690365
ENSG00000144034	98.5977544	-0.430606702	0.183906755	-2.341440386	0.019209493	0.055878185
ENSG00000144036	164.7314414	-0.206642155	0.142450011	-1.450629261	0.146883123	0.280754828
ENSG00000144040	190.0456365	-0.055257054	0.138896014	-0.39783038	0.690755222	0.80536509
ENSG00000144043	220.3597614	0.300437734	0.129220074	2.325008229	0.02007152	0.057941768
ENSG00000144048	85.15147821	-0.514008619	0.193260294	-2.659670071	0.007821723	0.026228633
ENSG00000144061	14.1453273	-1.206954267	0.487163634	-2.477513062	0.013230157	0.040934804
ENSG00000144115	58.52302985	-0.877645779	0.237635301	-3.693246641	0.000221409	0.001171425
ENSG00000144118	111.8250759	-0.389656679	0.172177119	-2.263115336	0.02362858	0.066130837
ENSG00000144120	113.2904079	0.307005845	0.174689539	1.757436913	0.078843366	0.174521394
ENSG00000144134	36.74406404	0.036656068	0.285866524	0.128227915	0.89796861	0.944497571
ENSG00000144136	432.0821943	-0.009062441	0.095862525	-0.094535804	0.924683543	0.958810774
ENSG00000144152	88.36765026	0.158243806	0.185891158	0.851271291	0.394618669	0.562705151
ENSG00000144161	151.5341659	-0.29125199	0.154034187	-1.890826937	0.05864745	0.138109092
ENSG00000144182	34.61312129	0.383045545	0.319426009	1.199168303	0.230462509	0.389708303
ENSG00000144199	44.78384096	0.163114782	0.257808001	0.632698679	0.526930433	0.680061722

ENSG00000144214	11.4923326	-0.716014782	0.516773613	-1.385548263	0.165884885	0.307537077
ENSG00000144224	602.2430022	0.271275989	0.084407353	3.213890481	0.001309496	0.005677312
ENSG00000144228	178.6187041	-0.075810334	0.138278915	-0.548242179	0.583525624	0.725571784
ENSG00000144231	583.0494397	-0.058776665	0.086202932	-0.681840677	0.4953397	0.654135261
ENSG00000144233	318.9584951	0.031561208	0.107290359	0.294166305	0.768630827	0.860932719
ENSG00000144278	22.59126256	-3.546285616	0.545110767	-6.50562387	7.74E-11	1.24E-09
ENSG00000144283	888.3160825	-0.16132284	0.078186098	-2.063318727	0.039082356	0.099961736
ENSG00000144306	63.22552727	0.294592667	0.232023387	1.269667989	0.20420292	0.357437809
ENSG00000144320	197.1255034	0.390494797	0.130122316	3.000982524	0.0026911	0.010515127
ENSG00000144354	371.4875499	-0.312332096	0.10737502	-2.908796637	0.003628228	0.013609003
ENSG00000144355	1348.121228	-0.797773366	0.070768633	-11.27297973	1.78E-29	9.53E-28
ENSG00000144357	496.837091	0.097800119	0.094568779	1.034169205	0.301057068	0.471454697
ENSG00000144366	136.9914592	-0.713594953	0.17031872	-4.189762315	2.79E-05	0.000181769
ENSG00000144369	1319.399836	-0.587029438	0.06697354	-8.765094955	1.87E-18	5.82E-17
ENSG00000144381	4073.18556	-0.107508719	0.062018832	-1.733485052	0.083009492	0.181766849
ENSG00000144395	84.53400489	-0.27430656	0.193783643	-1.415529999	0.156913159	0.294791915
ENSG00000144401	93.83966774	-0.300613981	0.188223745	-1.597109761	0.110241243	0.226395741
ENSG00000144426	208.949203	0.04775911	0.132104404	0.361525497	0.717706648	0.824088015
ENSG00000144451	61.38832702	-0.671762147	0.23171044	-2.899144932	0.003741819	0.013945696
ENSG00000144455	55.60912884	0.159086513	0.244791946	0.649884588	0.515766774	0.671096242
ENSG00000144468	164.104135	-0.165529266	0.15262123	-1.084575621	0.278109657	0.446980132
ENSG00000144485	226.3279458	0.565100812	0.140848233	4.012125678	6.02E-05	0.000363089
ENSG00000144488	236.8447301	0.689797188	0.127431029	5.413102218	6.19E-08	6.76E-07
ENSG00000144504	264.4458601	-0.322829309	0.130239956	-2.478727094	0.013185215	0.040831726
ENSG00000144524	256.219533	0.042800582	0.119758435	0.357390958	0.720799145	0.826670933
ENSG00000144535	278.3605943	-0.117881797	0.117949372	-0.99942708	0.317587847	0.489376534
ENSG00000144554	392.0641434	-0.320520619	0.099176928	-3.231806285	0.001230104	0.005377925
ENSG00000144559	208.2860721	-0.015645324	0.142369656	-0.109892265	0.912494817	0.953030514
ENSG00000144560	411.2612787	-0.070648097	0.103231981	-0.68436251	0.493746283	0.65301328
ENSG00000144566	559.1832665	0.109542361	0.093401599	1.172810338	0.240871871	0.40337701
ENSG00000144567	222.6936981	0.503336818	0.128799557	3.907908005	9.31E-05	0.000539727
ENSG00000144579	480.0459606	0.152056549	0.098478621	1.544056445	0.122574659	0.246029645
ENSG00000144580	480.4217648	-0.015246853	0.103849782	-0.146816419	0.883276925	0.935630116
ENSG00000144583	51.52105846	-3.009214827	0.326198382	-9.225106555	2.83E-20	9.71E-19
ENSG00000144589	146.1906065	0.016180639	0.147900353	0.109402298	0.912883411	0.953048924
ENSG00000144591	181.7871381	-0.047230776	0.141840591	-0.332984909	0.739145675	0.840370643
ENSG00000144596	22.57839577	-2.034739238	0.426082923	-4.775453618	1.79E-06	1.50E-05
ENSG00000144597	275.6700691	0.165917648	0.12297224	1.349228473	0.177263592	0.322541668
ENSG00000144635	404.8363719	0.402263419	0.102405473	3.928143759	8.56E-05	0.000501033
ENSG00000144642	259.9854706	-1.49983066	0.127637806	-11.75067718	7.01E-32	4.07E-30
ENSG00000144645	76.65816146	-4.086715251	0.339173193	-12.04905145	1.96E-33	1.20E-31
ENSG00000144647	295.3973388	0.613764526	0.114263489	5.371484179	7.81E-08	8.34E-07
ENSG00000144649	12.53392964	-2.045336248	0.553177089	-3.697434853	0.000217789	0.00115536
ENSG00000144655	245.2337638	0.432899105	0.127844735	3.386131664	0.000708854	0.003308062
ENSG00000144659	217.9661068	0.099766531	0.135518592	0.736183347	0.461619126	0.624677335
ENSG00000144674	4082.22679	0.260121295	0.062830263	4.140063788	3.47E-05	0.000221391
ENSG00000144677	503.7132521	0.377155783	0.092837533	4.062535607	4.85E-05	0.00029999
ENSG00000144711	956.8057661	0.031056411	0.079840777	0.388979312	0.69729145	0.810027539
ENSG00000144712	278.2368349	0.289679183	0.11869985	2.440434268	0.014669615	0.04465094
ENSG00000144713	7114.406163	0.339477672	0.075188669	4.515011083	6.33E-06	4.74E-05

ENSG00000144724	432.5946659	0.006745031	0.098403608	0.068544555	0.945352154	0.969665557
ENSG00000144730	89.17453568	-0.887261066	0.195331587	-4.542332745	5.56E-06	4.23E-05
ENSG00000144736	124.260854	0.015527681	0.16470945	0.094273166	0.924892165	0.958816254
ENSG00000144741	176.3451276	-0.128770245	0.139169909	-0.925273619	0.354823598	0.526259854
ENSG00000144744	220.2393584	-0.112347168	0.126288904	-0.889604421	0.373678331	0.543774943
ENSG00000144746	332.2219512	0.143764241	0.109571944	1.31205339	0.189502121	0.338829423
ENSG00000144747	332.82721	0.110060011	0.107402092	1.024747366	0.305482407	0.476472504
ENSG00000144749	12.82586794	-3.273867483	0.681670021	-4.802715949	1.57E-06	1.33E-05
ENSG00000144791	1046.910978	0.282792454	0.072590786	3.8957073	9.79E-05	0.000563911
ENSG00000144802	125.6856749	-3.687018916	0.245377089	-15.02592981	4.97E-51	5.45E-49
ENSG00000144815	218.0181474	0.095764984	0.13002028	0.736538823	0.46140285	0.624585702
ENSG00000144821	87.29052298	-1.236373703	0.201383075	-6.139412165	8.28E-10	1.17E-08
ENSG00000144827	80.734076	-0.137209946	0.206599147	-0.664136072	0.506603225	0.663984568
ENSG00000144840	57.58058337	0.0084658	0.233594701	0.036241402	0.971089873	0.984973605
ENSG00000144848	310.5514915	0.240098177	0.108297666	2.217020802	0.02662167	0.073109404
ENSG00000144857	291.2935791	-4.155042163	0.185739809	-22.37022949	7.67E-111	2.29E-108
ENSG00000144867	351.4775117	0.195037001	0.104787778	1.861257149	0.062707871	0.145813188
ENSG00000144893	84.26011427	-1.374672001	0.210886157	-6.518550211	7.10E-11	1.15E-09
ENSG00000144895	529.2255267	0.17778673	0.093859923	1.894170855	0.058202335	0.137314109
ENSG00000144909	212.4772021	-0.147564237	0.128907813	-1.144726865	0.252322319	0.416935845
ENSG00000144935	86.50432829	-0.682623878	0.19569119	-3.488270867	0.000486155	0.002362167
ENSG00000144959	43.11127811	-0.770778546	0.270080463	-2.853884863	0.004318818	0.015806784
ENSG00000145012	667.6356574	-0.308563962	0.091602052	-3.368526748	0.000755711	0.003495379
ENSG00000145014	91.277717	0.412676027	0.190441267	2.166946451	0.030238937	0.081046815
ENSG00000145016	499.9066049	0.061501499	0.090148747	0.682222458	0.495098296	0.654123943
ENSG00000145022	57.06984097	0.177746793	0.233409761	0.761522537	0.446345023	0.611307684
ENSG00000145041	892.8313614	0.242112845	0.077412279	3.127576762	0.001762538	0.007340163
ENSG00000145050	1648.047528	0.719985695	0.065730206	10.95365041	6.38E-28	3.20E-26
ENSG00000145088	14.69114058	0.014146831	0.469565028	0.030127523	0.97596535	0.987850214
ENSG00000145147	158.8137797	-5.515043392	0.354897904	-15.53980268	1.87E-54	2.20E-52
ENSG00000145191	207.5858467	-0.183651697	0.132256446	-1.388603	0.164953505	0.306090076
ENSG00000145214	78.63077997	0.234685564	0.204988195	1.144873554	0.25226154	0.416884454
ENSG00000145216	340.911721	-0.06608992	0.105597106	-0.625868666	0.531401089	0.683156819
ENSG00000145220	634.8166616	0.059334835	0.084246301	0.704301969	0.48124474	0.64167488
ENSG00000145241	215.7497912	-0.214798127	0.128073728	-1.677144334	0.093514261	0.199120028
ENSG00000145242	175.4444137	0.382550122	0.15067579	2.538895744	0.011120296	0.03530097
ENSG00000145246	94.3467817	0.712036948	0.194564189	3.65965059	0.000252559	0.001317868
ENSG00000145248	33.7246094	-0.023027174	0.310467184	-0.074169429	0.940875571	0.967262249
ENSG00000145284	34.64051648	-2.702748003	0.372281113	-7.259965418	3.87E-13	8.02E-12
ENSG00000145287	55.35426326	-2.910621634	0.308800063	-9.425586266	4.28E-21	1.54E-19
ENSG00000145293	130.6289114	0.098753365	0.158113299	0.624573429	0.532251063	0.683849875
ENSG00000145331	37.84279922	0.40536604	0.280076403	1.447340922	0.147801463	0.281934703
ENSG00000145332	240.1828294	0.059462019	0.122815421	0.484157599	0.628274026	0.758910074
ENSG00000145335	523.6392547	-2.182759764	0.102331898	-21.3301992	5.96E-101	1.52E-98
ENSG00000145348	184.3475517	-0.185291685	0.142642007	-1.298998022	0.193944608	0.34476587
ENSG00000145349	318.9684424	0.327782486	0.111383731	2.942821915	0.003252354	0.012367575
ENSG00000145354	113.9473337	0.003545105	0.17542515	0.020208646	0.983876931	0.991961125
ENSG00000145362	548.5376219	-0.114152344	0.087086859	-1.310787244	0.189929653	0.339346457
ENSG00000145365	34.7803053	0.674633006	0.301130713	2.240332774	0.025069327	0.069443667
ENSG00000145375	230.129935	-0.432792271	0.122142381	-3.543342347	0.000395089	0.001961761

ENSG00000145386	584.307419	-0.025575087	0.093532837	-0.27343431	0.784519381	0.871011269
ENSG00000145388	186.0300293	-0.203527826	0.136608802	-1.489858797	0.136261367	0.265805225
ENSG00000145390	208.1038301	-0.720316299	0.130284155	-5.528809698	3.22E-08	3.68E-07
ENSG00000145391	635.7292116	-0.301684758	0.08384082	-3.598303987	0.000320299	0.00162898
ENSG00000145414	180.2840797	0.067605003	0.146064628	0.462843085	0.643476846	0.769990566
ENSG00000145416	10.92106687	-0.806561704	0.537769747	-1.499827219	0.133659165	0.262075696
ENSG00000145425	180.2236898	0.04392167	0.139891069	0.313970512	0.753543435	0.851091472
ENSG00000145428	42.48985877	0.127015253	0.265241642	0.478866182	0.63203383	0.761813902
ENSG00000145431	53.41056719	-1.041281735	0.249234787	-4.177914912	2.94E-05	0.000190264
ENSG00000145439	254.9650956	0.010344528	0.127857891	0.080906453	0.935516348	0.964731953
ENSG00000145451	14.71071709	-6.18366422	1.235051737	-5.006805818	5.53E-07	5.19E-06
ENSG00000145476	147.4716441	-0.25859805	0.149267367	-1.732448667	0.083193709	0.182113454
ENSG00000145494	1017.392551	0.121121954	0.076611957	1.58097977	0.113882663	0.23194284
ENSG00000145495	862.6588721	-0.084718871	0.078745182	-1.075861013	0.28198942	0.451202345
ENSG00000145506	36.92056972	-0.699041614	0.289894682	-2.411364049	0.015892976	0.047866345
ENSG00000145545	80.40226728	-0.255333738	0.196948204	-1.296451213	0.194820078	0.346015632
ENSG00000145592	6461.173477	0.228678503	0.06470877	3.533964602	0.000409376	0.002023465
ENSG00000145604	431.4661976	0.101897128	0.100132705	1.017620844	0.308858178	0.480085296
ENSG00000145623	5.435065621	-5.723881768	1.322766292	-4.327205647	1.51E-05	0.000104137
ENSG00000145632	56.38991838	-1.781249408	0.2593546	-6.86800776	6.51E-12	1.17E-10
ENSG00000145642	25.12734079	0.387822747	0.34842664	1.113068585	0.265678975	0.432478778
ENSG00000145675	281.346225	-0.524203632	0.115429389	-4.541335941	5.59E-06	4.25E-05
ENSG00000145685	209.5204685	0.170763345	0.128365514	1.330289879	0.18342278	0.330678763
ENSG00000145687	486.9901199	-0.151396633	0.106764377	-1.41804445	0.156177788	0.293803319
ENSG00000145700	21.98558555	-1.485870445	0.423781116	-3.506221462	0.000454517	0.00222305
ENSG00000145703	21.91039456	-4.0537954	0.636361277	-6.370273537	1.89E-10	2.87E-09
ENSG00000145715	303.3358324	0.359706744	0.109392189	3.288230624	0.001008192	0.004528965
ENSG00000145723	28.29290941	-0.210127402	0.331578427	-0.633718556	0.526264513	0.679389542
ENSG00000145725	376.8673726	-0.269174817	0.100356929	-2.682174724	0.007314525	0.024763869
ENSG00000145730	440.8203218	0.447883643	0.107136887	4.180480285	2.91E-05	0.000188477
ENSG00000145734	1443.058325	0.085600826	0.068289283	1.253503071	0.210022682	0.365122354
ENSG00000145736	11.19288188	0.073257058	0.520266867	0.140806695	0.888022654	0.938604531
ENSG00000145740	358.8356832	0.045055766	0.113132091	0.398258046	0.690439982	0.805254219
ENSG00000145741	2199.382687	0.263931686	0.058994442	4.47383989	7.68E-06	5.65E-05
ENSG00000145743	134.0397174	-0.127966629	0.164718518	-0.776880649	0.437229189	0.603138409
ENSG00000145779	19.21339396	-0.723616233	0.42216166	-1.714073784	0.086515176	0.187600907
ENSG00000145780	165.4664453	-0.061921697	0.14888702	-0.415897213	0.677485219	0.795306274
ENSG00000145781	82.92905076	-0.071413792	0.194175717	-0.367779211	0.713037872	0.820852564
ENSG00000145782	329.7616529	0.156756175	0.107000943	1.46499807	0.142921444	0.275091467
ENSG00000145808	51.7412408	-0.229346024	0.254794889	-0.90012019	0.368056293	0.539393505
ENSG00000145817	166.5319622	0.099428396	0.144655702	0.687345159	0.491865247	0.651295106
ENSG00000145819	39.29993418	-0.164419566	0.281590738	-0.583895503	0.559290619	0.70606231
ENSG00000145833	2792.459861	0.120369974	0.061439936	1.959148739	0.05009537	0.122047589
ENSG00000145860	567.2588166	-0.411104952	0.088705353	-4.634499911	3.58E-06	2.83E-05
ENSG00000145868	179.6442467	-0.089397123	0.143460203	-0.623149281	0.533186427	0.684613322
ENSG00000145882	180.3203028	-0.548703523	0.144423752	-3.799260964	0.000145128	0.000804188
ENSG00000145901	251.4055293	0.313892481	0.120059844	2.614466844	0.008936683	0.029343991
ENSG00000145907	1771.730531	-0.37206492	0.062845751	-5.920287629	3.21E-09	4.19E-08
ENSG00000145911	16.00432928	-0.646615841	0.454977563	-1.421203798	0.155257521	0.292709201
ENSG00000145912	702.4878462	0.445586871	0.085243283	5.22723731	1.72E-07	1.75E-06

ENSG00000145916	228.3617358	0.012366258	0.125623231	0.098439262	0.921583499	0.956727767
ENSG00000145919	230.8682482	0.111064618	0.127962047	0.867949689	0.385421876	0.554301889
ENSG00000145979	28.02054357	-0.185721901	0.337048991	-0.551023461	0.581617592	0.723904157
ENSG00000145982	70.08602912	-0.041882652	0.219670184	-0.190661524	0.848790785	0.91321723
ENSG00000145990	184.6826512	0.108859626	0.135039314	0.806132839	0.420166258	0.587797573
ENSG00000145996	182.3938569	-0.239041834	0.142358814	-1.679150226	0.09312277	0.198548295
ENSG00000146007	155.9218928	-0.08280292	0.145200867	-0.570264639	0.56849822	0.713900814
ENSG00000146021	112.9022989	0.184086389	0.17417667	1.056894636	0.290559677	0.460873055
ENSG00000146054	13.84373951	-2.436804117	0.567368508	-4.294923109	1.75E-05	0.000118875
ENSG00000146063	357.851791	0.015790522	0.104359017	0.151309609	0.879731489	0.934004754
ENSG00000146066	727.3839374	0.321983791	0.101391881	3.175636836	0.00149508	0.006373714
ENSG00000146067	167.0786963	0.203567205	0.142351579	1.430031245	0.152708052	0.289017785
ENSG00000146072	25.16962566	-0.685506404	0.378537611	-1.810933403	0.070151161	0.159245857
ENSG00000146083	663.253339	0.163513244	0.082854564	1.973497128	0.048438951	0.11903813
ENSG00000146085	99.93591407	0.17482253	0.181681548	0.962247026	0.335925532	0.507774233
ENSG00000146090	24.10366609	-0.051568281	0.356901068	-0.144489008	0.885114336	0.937067517
ENSG00000146094	61.63713518	0.089825303	0.225669855	0.398038557	0.690601764	0.805319856
ENSG00000146109	159.0534915	0.047021893	0.144493903	0.325424752	0.744859625	0.844210781
ENSG00000146112	563.9013078	0.14309111	0.09073214	1.577071926	0.114778976	0.233497506
ENSG00000146143	102.7521586	-0.056054627	0.175641252	-0.31914272	0.74961829	0.848021916
ENSG00000146147	10.45223607	-6.666484157	1.254056795	-5.3159348	1.06E-07	1.11E-06
ENSG00000146197	39.40245162	-1.32545507	0.295564344	-4.484489073	7.31E-06	5.40E-05
ENSG00000146205	68.23078291	0.325154165	0.242624823	1.340152093	0.180195903	0.326439495
ENSG00000146221	12.58651952	-0.556000118	0.490856562	-1.132714038	0.257334361	0.423176746
ENSG00000146223	511.3705533	-0.108340844	0.090633804	-1.195369042	0.231942876	0.391692904
ENSG00000146232	50.28897708	0.139914612	0.252287986	0.554582936	0.579179963	0.72253472
ENSG00000146242	155.1898259	-0.027778503	0.146962483	-0.189017646	0.850078987	0.914253084
ENSG00000146243	69.400541	-0.520473011	0.221825791	-2.346314229	0.018960115	0.055267221
ENSG00000146247	3377.658871	-0.588400617	0.065656212	-8.961842249	3.19E-19	1.04E-17
ENSG00000146263	295.5142324	-0.333536099	0.11291905	-2.953762883	0.003139251	0.012002466
ENSG00000146267	97.60089695	-0.366091795	0.184501419	-1.984222107	0.047231085	0.116559215
ENSG00000146276	93.38626513	-3.848845167	0.290670463	-13.24126684	5.07E-40	3.96E-38
ENSG00000146278	898.9377541	-0.347661941	0.076452621	-4.547416888	5.43E-06	4.13E-05
ENSG00000146281	435.0997954	0.092705941	0.097029808	0.955437752	0.33935637	0.511145071
ENSG00000146282	325.856083	-0.012581442	0.108467886	-0.115992327	0.907658623	0.950649754
ENSG00000146350	136.5798964	-0.961007568	0.161533623	-5.949272678	2.69E-09	3.54E-08
ENSG00000146373	81.69663911	0.278915564	0.19638854	1.420223212	0.155542708	0.2930796
ENSG00000146376	17.43091577	0.763503061	0.426661113	1.789483593	0.073536967	0.164985431
ENSG00000146386	160.7678445	0.284104334	0.153560712	1.850110815	0.064297579	0.148844446
ENSG00000146409	128.0619093	0.084942687	0.159672457	0.531980835	0.594739269	0.734702534
ENSG00000146410	44.89854898	-0.366283509	0.264498947	-1.384820291	0.166107425	0.30782467
ENSG00000146411	36.01488473	0.250827111	0.312679666	0.802185555	0.422445631	0.589928798
ENSG00000146414	552.0194732	-0.178410643	0.092977522	-1.918857796	0.055002333	0.131554192
ENSG00000146416	96.54703736	0.043852028	0.191877606	0.228541666	0.819225168	0.893395096
ENSG00000146425	175.4537011	-0.295060155	0.145975714	-2.021296197	0.043249113	0.108991799
ENSG00000146426	37.57784511	-2.822761578	0.370389858	-7.621055274	2.52E-14	5.79E-13
ENSG00000146433	299.18969	-0.155543328	0.110968174	-1.401693134	0.161006902	0.300274024
ENSG00000146457	385.6261866	-0.125813894	0.106900807	-1.176921838	0.239226705	0.401148087
ENSG00000146463	767.60535	-0.073439194	0.078764445	-0.932390165	0.351134926	0.522671567
ENSG00000146476	111.9715401	0.033203946	0.176577146	0.188042149	0.850843611	0.914488597

ENSG00000146530	159.4258844	-0.491625008	0.14550268	-3.378803795	0.000728019	0.003384014
ENSG00000146535	333.2901241	0.159199199	0.105548462	1.508304295	0.131476664	0.258882943
ENSG00000146540	378.9171679	0.141839917	0.105461701	1.344942441	0.178643823	0.324380789
ENSG00000146576	190.957008	-0.015499669	0.136936569	-0.113188676	0.909880973	0.951381168
ENSG00000146587	336.9308533	-0.183500924	0.103795453	-1.767909086	0.077076104	0.171392754
ENSG00000146648	126.6776617	0.206239559	0.16082404	1.282392606	0.199704957	0.351712077
ENSG00000146670	328.3893383	0.252441495	0.11346401	2.224859621	0.026090663	0.071904169
ENSG00000146676	238.6439926	-0.379146499	0.120632194	-3.142995961	0.001672281	0.007020484
ENSG00000146677	27.82350612	0.470387689	0.345473488	1.361573914	0.173332392	0.317113847
ENSG00000146700	8.428626917	0.450496534	0.594257282	0.758083322	0.448401104	0.613488877
ENSG00000146701	2893.424756	0.144829986	0.062591447	2.313894211	0.020673526	0.059374569
ENSG00000146707	36.93614829	0.25144361	0.283152876	0.888013616	0.374533427	0.54453973
ENSG00000146729	523.6700394	-0.12847835	0.096082262	-1.33717033	0.181167044	0.3278575
ENSG00000146731	2078.135118	0.202166877	0.065966533	3.064688556	0.002178968	0.008782772
ENSG00000146733	217.1992768	-0.649110838	0.127930542	-5.073931745	3.90E-07	3.75E-06
ENSG00000146757	103.1641694	-0.310469118	0.186879884	-1.661329787	0.096647231	0.204150925
ENSG00000146776	303.7802784	0.784685355	0.113768775	6.897194365	5.30E-12	9.66E-11
ENSG00000146802	205.1391195	0.287022425	0.129174081	2.221981554	0.026284552	0.072324996
ENSG00000146826	45.63543184	-0.289159117	0.279563333	-1.034324186	0.300984634	0.471393818
ENSG00000146828	80.03239829	-0.128175756	0.202508347	-0.632940607	0.526772429	0.679920271
ENSG00000146830	605.6024315	0.223490366	0.096998331	2.304064042	0.021219049	0.060631403
ENSG00000146833	80.18988211	-0.256244402	0.207190945	-1.236754827	0.216178117	0.372458483
ENSG00000146834	75.31201982	-0.003607041	0.400218555	-0.009012678	0.992809021	0.996284349
ENSG00000146842	210.9432433	0.406789706	0.134746887	3.018917272	0.002536798	0.010006949
ENSG00000146856	16.49034918	-0.51503284	0.442575807	-1.163716661	0.244538855	0.40776796
ENSG00000146858	298.4601958	0.709342482	0.129456114	5.479405032	4.27E-08	4.78E-07
ENSG00000146872	381.5901221	-0.206269	0.101535828	-2.031489812	0.042205332	0.106859823
ENSG00000146909	926.8344827	0.673423172	0.084134485	8.004127856	1.20E-15	3.07E-14
ENSG00000146918	463.388199	0.565567772	0.097068704	5.826468738	5.66E-09	7.19E-08
ENSG00000146950	17.2397206	-0.535981277	0.424638546	-1.262205899	0.206874707	0.360788542
ENSG00000146963	485.289894	0.45584145	0.09166094	4.973126478	6.59E-07	6.08E-06
ENSG00000147010	286.1603985	-0.004609663	0.112900284	-0.040829506	0.967431817	0.982880628
ENSG00000147027	21.71667563	-3.025571037	0.521750623	-5.798883424	6.68E-09	8.43E-08
ENSG00000147044	318.8105747	-0.397789535	0.108770871	-3.657132958	0.000255052	0.001329394
ENSG00000147050	491.5376283	-0.215324346	0.101710244	-2.117036973	0.03425671	0.089877476
ENSG00000147065	922.7307421	0.130567916	0.0753511	1.732793775	0.08313233	0.182007456
ENSG00000147082	21.93346268	-1.060444241	0.393845483	-2.69253879	0.007091029	0.024094746
ENSG00000147099	240.1588414	0.072863422	0.132598237	0.549505205	0.582658793	0.724878936
ENSG00000147100	427.3189646	0.202430095	0.097879666	2.068152699	0.038625667	0.099046137
ENSG00000147117	12.69144334	-0.161538949	0.494641001	-0.326578162	0.743986963	0.843553545
ENSG00000147118	47.69438866	0.337380274	0.263181063	1.281932177	0.199866439	0.351952347
ENSG00000147119	39.35866692	0.31616107	0.300498967	1.052120321	0.29274435	0.463396426
ENSG00000147121	46.79536009	0.007182518	0.25628093	0.028025956	0.977641449	0.988629033
ENSG00000147123	669.0846232	0.352051404	0.089619353	3.928296626	8.55E-05	0.000500924
ENSG00000147124	130.3303332	-0.576583628	0.165559943	-3.482627616	0.000496519	0.002407523
ENSG00000147130	964.7362978	-0.068119527	0.077752892	-0.876102817	0.380974142	0.550538001
ENSG00000147133	594.9466568	-0.14655721	0.091187352	-1.607209849	0.108008342	0.222756303
ENSG00000147140	2510.118944	0.246050066	0.070022424	3.513875283	0.00044162	0.002169034
ENSG00000147144	34.01144187	0.220585136	0.310066347	0.711412697	0.476828533	0.63753861
ENSG00000147155	352.0293292	-0.023790616	0.265330823	-0.089663974	0.928554246	0.96098147

ENSG00000147162	1387.067682	0.009083817	0.077035081	0.117917929	0.906132687	0.949742267
ENSG00000147164	405.9667128	0.31552632	0.098942867	3.188974891	0.001427783	0.006122814
ENSG00000147166	12.1332492	-0.418614015	0.495094103	-0.84552414	0.397818261	0.565946233
ENSG00000147174	11.34506407	-0.358261033	0.516360425	-0.693819696	0.487795289	0.647887711
ENSG00000147180	280.2667652	-0.174376433	0.11732468	-1.486272401	0.137207084	0.266871768
ENSG00000147202	365.9782241	0.223576399	0.104732315	2.134741304	0.032782147	0.086574698
ENSG00000147224	401.304466	0.082835922	0.100419683	0.824897267	0.409429918	0.577842063
ENSG00000147251	37.31877704	1.081214731	0.291470226	3.709520331	0.000207652	0.00110698
ENSG00000147257	32.60510255	-0.15700188	0.316951065	-0.495350537	0.620352703	0.75361882
ENSG00000147274	1540.872445	-0.132649218	0.066424614	-1.996988926	0.045826386	0.113913156
ENSG00000147316	440.2769627	0.071817372	0.100108121	0.717398055	0.473128519	0.63440591
ENSG00000147324	303.1197442	0.035921858	0.111792696	0.321325623	0.747963641	0.846695565
ENSG00000147364	20.65539299	1.035173324	0.385378702	2.686119703	0.007228719	0.024521084
ENSG00000147383	137.8606874	0.144744357	0.156779618	0.923234535	0.355884995	0.527227319
ENSG00000147394	21.76460082	1.666233277	0.402700041	4.137653609	3.51E-05	0.000223627
ENSG00000147400	46.58867429	-0.0926378	0.279585083	-0.33134028	0.740387469	0.841305838
ENSG00000147403	2266.838324	0.163302784	0.076678198	2.129715978	0.033195069	0.087489073
ENSG00000147416	308.3056613	0.114574123	0.11965599	0.957529356	0.338300136	0.510265437
ENSG00000147419	324.5798248	0.086656982	0.112545813	0.769970726	0.441317258	0.60654127
ENSG00000147421	290.6807853	-0.099904994	0.119616521	-0.835210664	0.403599107	0.57179593
ENSG00000147439	89.74964628	-0.286969331	0.187498023	-1.530519238	0.125888255	0.250830117
ENSG00000147454	572.9755472	0.279575003	0.086330259	3.238435828	0.001201871	0.00526432
ENSG00000147457	249.3870562	0.160112094	0.12088443	1.324505515	0.185335213	0.333257006
ENSG00000147459	121.5743484	2.355788664	0.188404045	12.50391768	7.11E-36	4.78E-34
ENSG00000147471	209.2665558	0.218940698	0.135431307	1.61661807	0.105960749	0.219435447
ENSG00000147475	227.9950458	0.029970461	0.125940628	0.237972936	0.811902086	0.889400967
ENSG00000147509	22.23631873	-1.188467392	0.393274642	-3.02197819	0.002511286	0.009925729
ENSG00000147526	166.1921482	-0.315807179	0.146595208	-2.154280364	0.031218192	0.083210994
ENSG00000147533	232.6332031	-0.152037405	0.122098863	-1.245199191	0.21305854	0.368439408
ENSG00000147535	138.2723123	0.778592776	0.156842356	4.964174192	6.90E-07	6.33E-06
ENSG00000147536	133.1140569	-0.286726614	0.163194285	-1.756964796	0.07892381	0.174617969
ENSG00000147548	1128.282515	0.160882468	0.069110345	2.327907158	0.019917034	0.057578633
ENSG00000147576	8.664196928	-1.391763207	0.665297492	-2.09194116	0.03644378	0.094579564
ENSG00000147586	40.84768837	-0.589259923	0.282273935	-2.087546353	0.036838776	0.095344332
ENSG00000147592	159.2376443	0.000498621	0.152502275	0.003269596	0.997391245	0.998813347
ENSG00000147601	269.906828	-0.774933711	0.119214893	-6.500309559	8.02E-11	1.29E-09
ENSG00000147604	482.1977749	-0.126339936	0.120743927	-1.046346095	0.295401271	0.465817428
ENSG00000147649	1539.01156	0.162384183	0.067182984	2.417043328	0.015647154	0.047206954
ENSG00000147650	281.7255481	0.061572152	0.12127484	0.507707555	0.61165845	0.747517508
ENSG00000147654	119.8686812	0.122679617	0.175504318	0.699011958	0.484544558	0.645033871
ENSG00000147669	376.7496627	0.13627619	0.10519027	1.295520872	0.195140606	0.346334948
ENSG00000147677	2395.904797	0.1681091	0.063004208	2.668220171	0.007625427	0.02569053
ENSG00000147679	294.8752574	0.033262822	0.116062283	0.286594587	0.77442276	0.864944408
ENSG00000147684	1452.435142	0.368931898	0.06632456	5.562523118	2.66E-08	3.07E-07
ENSG00000147687	356.6668644	0.451916516	0.104565681	4.321843562	1.55E-05	0.000106342
ENSG00000147789	262.1461259	0.223395196	0.119382073	1.871262495	0.061308706	0.143295246
ENSG00000147799	203.7298511	-0.094285871	0.131823423	-0.715243685	0.47445848	0.635643135
ENSG00000147804	362.5181488	0.173105412	0.104698052	1.653377586	0.098254042	0.206838489
ENSG00000147813	16.32665534	0.235351656	0.444070741	0.529986855	0.596121045	0.735697796
ENSG00000147852	33.51397511	0.19370991	0.296832624	0.652589691	0.514020834	0.669862756

ENSG00000147853	248.2045805	0.002742813	0.128564137	0.021334202	0.982979061	0.991662372
ENSG00000147854	313.7931343	-0.195191673	0.11434215	-1.707084161	0.087806411	0.189552275
ENSG00000147862	821.7260313	-0.541630198	0.078404485	-6.908153268	4.91E-12	8.98E-11
ENSG00000147869	24.9110404	-1.041805429	0.36816745	-2.829705424	0.004659088	0.016863233
ENSG00000147872	168.779406	0.204521871	0.142840449	1.431820415	0.152195223	0.288402768
ENSG00000147874	342.1777469	-0.186570762	0.105980494	-1.760425471	0.078335694	0.173726159
ENSG00000147889	46.84857	0.65808018	0.264256094	2.490312224	0.012763092	0.039729654
ENSG00000147894	34.36813572	0.162081691	0.302687909	0.535474614	0.592321707	0.732618035
ENSG00000147905	177.5479842	0.222660501	0.139512161	1.595993493	0.110490246	0.226776226
ENSG00000147912	61.64620355	0.050908949	0.23936356	0.212684624	0.83157296	0.901477895
ENSG00000147955	2342.170801	0.417609257	0.07013947	5.953983639	2.62E-09	3.45E-08
ENSG00000147996	187.6020227	0.210677953	0.133847313	1.574017053	0.115483507	0.234421507
ENSG00000148019	435.4437989	-0.315931127	0.09817532	-3.218030029	0.001290743	0.005601195
ENSG00000148057	19.50000748	-0.146289601	0.387126129	-0.377886147	0.705515174	0.815198787
ENSG00000148082	9.44098588	-5.534626174	1.265399242	-4.373818153	1.22E-05	8.58E-05
ENSG00000148090	89.98569154	0.176578018	0.188839388	0.935069852	0.349752302	0.521768329
ENSG00000148110	392.628815	0.002941387	0.10391984	0.028304379	0.977419388	0.988629033
ENSG00000148120	77.06099585	-0.863457188	0.20381745	-4.236424242	2.27E-05	0.000150907
ENSG00000148143	772.8583502	-0.250020319	0.08264041	-3.025400283	0.002483043	0.009839013
ENSG00000148153	174.8997951	0.263493962	0.138545533	1.901858228	0.057189692	0.135388028
ENSG00000148154	136.928874	-0.44878504	0.154470913	-2.9053045	0.00366896	0.01372148
ENSG00000148158	266.6144798	0.199703359	0.120140047	1.662254714	0.096461714	0.203881687
ENSG00000148175	623.8075656	0.404700154	0.091723169	4.412191157	1.02E-05	7.32E-05
ENSG00000148180	1410.121094	-0.272647191	0.072228152	-3.774805049	0.000160133	0.000877638
ENSG00000148187	283.1754653	0.022173975	0.115427343	0.192103315	0.847661279	0.912351389
ENSG00000148200	178.4704312	-0.831218892	0.139193729	-5.971669127	2.35E-09	3.13E-08
ENSG00000148218	189.0787609	0.257148073	0.13521075	1.901831574	0.057193177	0.135388028
ENSG00000148219	68.70738052	-0.628776743	0.218391692	-2.879123921	0.003987816	0.014776273
ENSG00000148225	49.38401703	-0.947201007	0.256894545	-3.687119967	0.000226806	0.001196376
ENSG00000148229	545.4399638	-0.036780925	0.092584633	-0.397268142	0.691169738	0.805563918
ENSG00000148248	1133.940543	0.002460091	0.074002885	0.03324318	0.973480665	0.986330341
ENSG00000148288	12.5331195	-0.6941916	0.497698181	-1.394804376	0.163074839	0.303365417
ENSG00000148290	42.53303572	-0.258096772	0.282600597	-0.913291673	0.361089161	0.532125413
ENSG00000148291	190.4074686	0.573242344	0.139584553	4.106774944	4.01E-05	0.000252507
ENSG00000148296	540.0981645	0.195016875	0.095072871	2.051235782	0.040243991	0.102281046
ENSG00000148297	286.4889911	0.110231404	0.113131856	0.974362193	0.329876735	0.502255933
ENSG00000148300	443.1414628	0.111733871	0.095465727	1.170408219	0.24183673	0.404510662
ENSG00000148303	2985.124652	0.067062275	0.158178665	0.423965363	0.671591074	0.790699842
ENSG00000148308	412.7187465	-0.062527027	0.100014929	-0.625176943	0.531854933	0.683490917
ENSG00000148331	268.1902542	-0.191120756	0.117109645	-1.63198135	0.102683415	0.214132116
ENSG00000148334	467.0923485	0.238432425	0.096007682	2.483472363	0.013010843	0.040380758
ENSG00000148335	178.0841066	0.08903735	0.140015749	0.635909539	0.524835385	0.678167846
ENSG00000148337	1215.629295	0.026112269	0.069476289	0.375844318	0.707032638	0.816145642
ENSG00000148339	266.485403	-0.043231303	0.122006261	-0.354336754	0.723086527	0.828279902
ENSG00000148341	260.2020329	0.157985253	0.125623655	1.25760751	0.208533736	0.362983072
ENSG00000148343	134.9600717	-0.08741746	0.153730143	-0.56864229	0.569598919	0.714810407
ENSG00000148344	69.5266453	0.203635373	0.215808567	0.943592629	0.34537786	0.517109657
ENSG00000148356	139.068295	0.516245512	0.15484291	3.333995161	0.000856081	0.003905611
ENSG00000148358	489.5551337	-0.00905595	0.095879187	-0.09445168	0.924750364	0.958810774
ENSG00000148362	50.70731676	0.250723651	0.265502492	0.944336337	0.344997803	0.516705846

ENSG00000148384	184.6990202	0.736998668	0.144842613	5.088272385	3.61E-07	3.50E-06
ENSG00000148396	2120.832557	0.029188707	0.06792442	0.42972331	0.667396925	0.787413177
ENSG00000148399	149.6723054	-0.07174318	0.154360951	-0.464775449	0.642092271	0.768923285
ENSG00000148400	775.3647133	0.560965009	0.086391115	6.49331831	8.40E-11	1.34E-09
ENSG00000148408	108.2392328	-0.382942521	0.177537346	-2.156968835	0.031008093	0.08269806
ENSG00000148411	308.0152976	0.033088152	0.119832811	0.2761193	0.782456437	0.869898344
ENSG00000148426	36.8545094	0.1354344	0.286955656	0.471969788	0.636948353	0.765710468
ENSG00000148429	282.3611214	0.178271039	0.112411385	1.585880647	0.112766387	0.230337274
ENSG00000148444	20.6586838	-0.227828355	0.382081796	-0.596281627	0.550987103	0.698621617
ENSG00000148450	133.8914456	-0.207616701	0.157843902	-1.315329248	0.188399273	0.337212612
ENSG00000148459	109.7036213	0.202624303	0.169715518	1.193905574	0.232514907	0.392470187
ENSG00000148468	249.2511055	0.198382098	0.126728315	1.565412578	0.117486201	0.23774095
ENSG00000148481	70.67170871	0.017231295	0.216677622	0.079525031	0.936615023	0.965199268
ENSG00000148484	298.3986048	-0.201918581	0.118001856	-1.711147501	0.087053887	0.188420024
ENSG00000148498	376.5631358	-0.03318596	0.100409481	-0.330506243	0.741017476	0.841749352
ENSG00000148516	357.3834036	-0.341735071	0.103084583	-3.315093879	0.000916124	0.004159272
ENSG00000148572	85.10330116	-0.197769235	0.197672058	-1.000491609	0.317072656	0.48874351
ENSG00000148600	187.8492291	-0.338851487	0.15592823	-2.173124689	0.029770934	0.079968486
ENSG00000148606	444.1855928	-0.247806501	0.096891203	-2.557574817	0.010540488	0.033681128
ENSG00000148634	326.5075045	0.179166884	0.105214979	1.702864794	0.088593374	0.190928371
ENSG00000148660	140.693405	-0.459786458	0.154312889	-2.979572609	0.002886508	0.011169912
ENSG00000148672	558.0458765	-0.101213276	0.092925179	-1.089191077	0.276069633	0.444515146
ENSG00000148688	136.8807478	-0.042693985	0.153395168	-0.278326791	0.780761512	0.868632739
ENSG00000148690	325.8231903	-0.038947166	0.109593159	-0.355379532	0.722305281	0.827735087
ENSG00000148700	406.8757205	-0.120795205	0.109912812	-1.099009326	0.271763998	0.439474839
ENSG00000148719	461.194874	0.290349833	0.093901208	3.092077723	0.001987608	0.00813044
ENSG00000148730	788.5768689	-0.217291884	0.086265177	-2.518882943	0.011772779	0.03705405
ENSG00000148737	820.2314977	-0.031596019	0.086419025	-0.365614158	0.714653009	0.822240217
ENSG00000148773	9167.563667	-0.024644462	0.054104132	-0.455500557	0.648749167	0.773893979
ENSG00000148798	469.779697	-0.415724973	0.098890064	-4.203910425	2.62E-05	0.000172042
ENSG00000148803	210.7533629	0.343659629	0.135722136	2.532082379	0.011338735	0.035880876
ENSG00000148814	72.48807041	0.215072987	0.235838906	0.911948713	0.361795717	0.532552073
ENSG00000148824	89.95971425	0.445250762	0.214548074	2.075296011	0.037959109	0.097604174
ENSG00000148834	104.7531925	0.341893049	0.17502412	1.953405334	0.050771585	0.123282359
ENSG00000148835	230.5039203	0.13806327	0.124043951	1.113018972	0.265700281	0.432478778
ENSG00000148840	1004.930233	0.094191054	0.072859947	1.292768627	0.196091094	0.347131896
ENSG00000148841	163.4264512	-0.414067062	0.144758269	-2.860403525	0.004231023	0.015525908
ENSG00000148842	350.9046343	-0.112296037	0.101993766	-1.101008828	0.270892815	0.438500861
ENSG00000148843	1489.201768	0.21060623	0.066641082	3.16030628	0.001576034	0.006660212
ENSG00000148908	304.9727876	0.211995865	0.112492297	1.884536721	0.059492413	0.139836798
ENSG00000148925	93.07158649	0.122367511	0.186187188	0.657228419	0.511034055	0.667402909
ENSG00000148926	107.6386541	0.044403729	0.17540441	0.253150586	0.80015185	0.880924836
ENSG00000148943	397.4040436	-0.225577624	0.100261198	-2.249899538	0.024455323	0.06802454
ENSG00000148950	32.90678325	-0.084500134	0.302167818	-0.279646373	0.779748826	0.868262525
ENSG00000148985	72.58298541	0.054878775	0.207047068	0.265054585	0.790967412	0.875273298
ENSG00000149016	44.20803142	0.196669231	0.280805369	0.700375609	0.483692764	0.644164813
ENSG00000149050	26.79463076	-0.240275996	0.3450353	-0.69638091	0.486190315	0.64655073
ENSG00000149054	129.0315918	-0.188531632	0.160737249	-1.172918119	0.240828643	0.403352802
ENSG00000149084	455.2578603	0.034801416	0.101654983	0.342348349	0.732088754	0.834847437
ENSG00000149089	105.5517882	0.150943748	0.174212608	0.866434121	0.386252139	0.555102336

ENSG00000149091	246.0485527	0.31965457	0.130798949	2.443861911	0.014530987	0.044286565
ENSG00000149100	687.3628793	0.121922222	0.084168139	1.448555517	0.147461751	0.281707497
ENSG00000149115	567.8610052	-0.009388156	0.09412455	-0.099741839	0.920549284	0.956523119
ENSG00000149131	919.6970489	-0.413193993	0.086344814	-4.785394424	1.71E-06	1.44E-05
ENSG00000149136	1868.599666	0.000706865	0.061373396	0.01151745	0.990810608	0.995236126
ENSG00000149150	116.2232235	0.366672125	0.165823437	2.211220157	0.027020595	0.074029396
ENSG00000149177	161.1245866	0.515589093	0.148159041	3.479970512	0.000501469	0.002428175
ENSG00000149179	136.6291009	-0.067327929	0.156062394	-0.431416739	0.666165381	0.786348438
ENSG00000149182	267.6767299	0.302238222	0.115175307	2.624158161	0.008686344	0.028667817
ENSG00000149187	1012.712478	0.036055426	0.074023154	0.487083086	0.626199452	0.757381067
ENSG00000149196	94.67091331	0.167022574	0.188484152	0.886135901	0.375544299	0.545529553
ENSG00000149212	475.7448512	-0.571516671	0.101331435	-5.640072802	1.70E-08	2.02E-07
ENSG00000149218	138.8387934	-0.420983442	0.160917539	-2.61614393	0.008892906	0.029224957
ENSG00000149231	159.2049478	0.50699233	0.152408788	3.326529518	0.000879347	0.004005249
ENSG00000149257	2830.409605	0.173861768	0.062424226	2.78516498	0.005350049	0.018934788
ENSG00000149260	47.50533219	-1.028756699	0.268469122	-3.831936769	0.000127138	0.000716084
ENSG00000149262	245.8486324	0.352088786	0.127090013	2.770389089	0.005598936	0.019681009
ENSG00000149269	199.0504991	-0.06494235	0.13313596	-0.4877897	0.625698807	0.757101486
ENSG00000149273	5508.327967	0.065204698	0.057632649	1.131384718	0.257893201	0.423550309
ENSG00000149289	25.41283388	-2.720145361	0.43738933	-6.219048284	5.00E-10	7.24E-09
ENSG00000149292	79.1770203	-0.183317453	0.199204213	-0.920248878	0.357442717	0.52874872
ENSG00000149308	193.5011405	-0.15883605	0.1408608	-1.127610026	0.259484651	0.425616638
ENSG00000149311	729.7419838	-0.3043047	0.089952634	-3.382943756	0.000717133	0.003343116
ENSG00000149313	227.2117702	0.0260456	0.123758313	0.210455357	0.833312291	0.902386945
ENSG00000149328	94.99684447	0.476773632	0.180980556	2.63439146	0.008428825	0.027990473
ENSG00000149346	124.4162553	-0.241693615	0.162784533	-1.484745572	0.137611235	0.267472674
ENSG00000149357	393.2213057	0.877438868	0.105044574	8.353014669	6.65E-17	1.83E-15
ENSG00000149403	23.3470758	2.352008612	0.414084753	5.680017422	1.35E-08	1.63E-07
ENSG00000149428	1500.51344	0.430569521	0.072108973	5.971094901	2.36E-09	3.13E-08
ENSG00000149474	109.9834096	-0.375278437	0.175663217	-2.136351847	0.032650746	0.086292573
ENSG00000149476	141.668649	-0.072069789	0.152987949	-0.471081476	0.637582553	0.76614774
ENSG00000149480	1091.357773	0.10451118	0.069637035	1.500798817	0.133407605	0.261750019
ENSG00000149483	280.8999598	0.317819454	0.11891274	2.67271156	0.007524091	0.02539426
ENSG00000149485	2787.260451	-0.11482248	0.061397191	-1.870158511	0.061461809	0.143581247
ENSG00000149489	23.13511131	-0.326507108	0.365119352	-0.894247612	0.371189423	0.54217406
ENSG00000149499	215.3185623	-0.184249891	0.130547534	-1.411362478	0.158137761	0.296339531
ENSG00000149503	1094.722648	-0.18955573	0.070675958	-2.682039774	0.007317476	0.024763869
ENSG00000149531	13.13560204	-0.592727476	0.500698937	-1.183800148	0.236492184	0.397702107
ENSG00000149532	959.4493256	0.016262043	0.074081761	0.219514796	0.826249056	0.898391554
ENSG00000149541	77.19374836	-0.186225603	0.212538716	-0.876196143	0.380923413	0.550538001
ENSG00000149547	730.0620554	0.039186949	0.094828482	0.413240288	0.679430573	0.796724373
ENSG00000149548	35.49769654	-0.277680756	0.314465428	-0.883024749	0.3772229	0.546670343
ENSG00000149554	158.5674785	-0.44417851	0.146846275	-3.02478568	0.002488094	0.009849697
ENSG00000149557	16.07781053	-1.219495035	0.4552679	-2.678631712	0.007392365	0.024973678
ENSG00000149564	8.688341821	-0.598964243	0.598188935	-1.001296092	0.316683682	0.488304685
ENSG00000149577	623.6813289	-0.428120038	0.090472976	-4.732021172	2.22E-06	1.83E-05
ENSG00000149582	165.6983431	0.594512969	0.149483172	3.97712305	6.98E-05	0.00041642
ENSG00000149591	249.8596885	-0.262467974	0.121524329	-2.159797763	0.030788329	0.082283801
ENSG00000149596	49.3979252	-5.056476186	0.548336238	-9.221488268	2.93E-20	1.00E-18
ENSG00000149600	64.32139013	0.407828665	0.236464846	1.724690462	0.084583271	0.184408072

ENSG00000149636	119.557073	-0.08400225	0.163826301	-0.512751917	0.608124868	0.744689219
ENSG00000149639	2316.046233	-0.103629324	0.063710456	-1.626566985	0.103829096	0.215867235
ENSG00000149646	19.82152622	0.417575817	0.383640453	1.088456165	0.276393778	0.444897936
ENSG00000149657	500.3044796	0.176006223	0.094546283	1.861587968	0.062661191	0.145777036
ENSG00000149658	573.6790347	0.04635908	0.087001638	0.532852958	0.594135372	0.734279392
ENSG00000149679	129.3899456	-0.431878141	0.161844047	-2.668483323	0.007619456	0.025685265
ENSG00000149716	71.04004366	0.422639909	0.212756935	1.986491812	0.046978741	0.116160953
ENSG00000149743	100.3651605	0.150171646	0.176036246	0.853072305	0.393619209	0.561679097
ENSG00000149761	95.43311449	0.519211398	0.185400775	2.80048127	0.005102647	0.018200117
ENSG00000149781	37.94522048	-0.198410331	0.278923628	-0.711342857	0.4768718	0.63753861
ENSG00000149782	580.2352814	0.115875429	0.086137668	1.345235262	0.178549272	0.324292944
ENSG00000149792	484.8823217	0.228000866	0.095814148	2.379615865	0.017330694	0.051413781
ENSG00000149806	965.410663	0.290296701	0.09742478	2.979700877	0.0028853	0.011168313
ENSG00000149809	283.8505253	-0.615274988	0.113268054	-5.432025772	5.57E-08	6.13E-07
ENSG00000149823	355.8601709	-0.090255718	0.113485483	-0.795306286	0.426435358	0.593670711
ENSG00000149922	17.58596446	-0.361302882	0.415942105	-0.868637432	0.385045474	0.554120434
ENSG00000149923	358.2903477	0.098192074	0.108124206	0.908141458	0.363803489	0.534834915
ENSG00000149925	701.633579	0.383230075	0.087434839	4.383036342	1.17E-05	8.27E-05
ENSG00000149926	90.2647982	-0.820680384	0.190443589	-4.309309592	1.64E-05	0.000112051
ENSG00000149927	78.53900444	0.118164485	0.19956345	0.592114863	0.553773675	0.700924384
ENSG00000149929	437.2505028	0.241702976	0.099742471	2.423270385	0.015381474	0.046525314
ENSG00000149930	424.9090358	0.045892517	0.103363297	0.443992393	0.657048084	0.780116172
ENSG00000149932	350.520981	0.214555987	0.1086989	1.973856108	0.048398107	0.118979385
ENSG00000149948	3691.05738	-1.668289743	0.059509312	-28.03409543	6.24E-173	3.99E-170
ENSG00000149968	11.85688291	2.666585044	0.609081477	4.378043243	1.20E-05	8.44E-05
ENSG00000149970	12.5897364	4.34289494	0.824138444	5.269618193	1.37E-07	1.41E-06
ENSG00000149972	19.76134132	-1.118701579	0.411513619	-2.718504388	0.006557779	0.022511514
ENSG00000150048	9.590139404	-3.012631296	0.753442799	-3.998487075	6.37E-05	0.000383502
ENSG00000150093	2731.985248	-0.161766791	0.05622552	-2.877106182	0.004013405	0.014843682
ENSG00000150281	67.1741357	-0.591704318	0.230915594	-2.56242685	0.010394348	0.03335833
ENSG00000150316	285.932674	0.207580657	0.112470075	1.845652332	0.064942703	0.150016468
ENSG00000150347	2581.311992	0.243660204	0.059489399	4.095859237	4.21E-05	0.000263173
ENSG00000150401	78.2978062	-0.324644885	0.216337052	-1.500643935	0.133447681	0.261770644
ENSG00000150403	266.2495531	-0.279721922	0.121505085	-2.302141697	0.021327183	0.060866098
ENSG00000150433	49.53410097	0.223603411	0.247916538	0.901930193	0.367093951	0.538376356
ENSG00000150455	69.1602031	-0.060517271	0.215925394	-0.280269353	0.779270861	0.868088632
ENSG00000150456	28.83462268	0.542418427	0.32411543	1.673534726	0.094222076	0.2002629
ENSG00000150457	569.5940173	-0.065830626	0.089478368	-0.735715536	0.461903833	0.624920929
ENSG00000150459	494.9495202	0.019736316	0.092377524	0.213648462	0.830821205	0.901344809
ENSG00000150471	53.78297664	-1.936957293	0.270490282	-7.160912681	8.01E-13	1.61E-11
ENSG00000150477	72.17241471	-0.169107592	0.213787682	-0.791007182	0.428939801	0.595858127
ENSG00000150510	17.10618482	-0.02223394	0.422620984	-0.052609644	0.958042933	0.977492516
ENSG00000150527	267.3250733	-0.037341188	0.115544203	-0.323176646	0.746561466	0.845517126
ENSG00000150556	22.96296711	-6.821737386	1.2137976	-5.620160548	1.91E-08	2.25E-07
ENSG00000150593	702.5971119	0.06074404	0.081235852	0.747749158	0.454611474	0.618431859
ENSG00000150625	49.03255197	1.371485986	0.279512028	4.906715449	9.26E-07	8.26E-06
ENSG00000150627	80.32233752	-0.214366449	0.196016194	-1.093616014	0.274123423	0.442344827
ENSG00000150636	21.52566465	-0.92574467	0.387948134	-2.386258858	0.017020766	0.050644079
ENSG00000150667	9.138962425	-0.240505998	0.586351422	-0.410173811	0.68167845	0.798493762
ENSG00000150672	16.66728047	-0.836605959	0.42907307	-1.949798339	0.05120016	0.124065662

ENSG00000150687	11.56839434	1.061435299	0.527322023	2.012878758	0.044127386	0.11057883
ENSG00000150712	290.6179565	-0.04977208	0.118384906	-0.420425893	0.674174356	0.792545583
ENSG00000150753	2014.120079	0.221048085	0.061755919	3.579382986	0.000344406	0.001738992
ENSG00000150756	88.46703663	0.317163727	0.190834817	1.661980414	0.096516702	0.203942456
ENSG00000150760	1391.079006	-0.071664122	0.068194093	-1.050884597	0.293311596	0.463928384
ENSG00000150764	420.5825673	0.161497831	0.097147574	1.662396947	0.096433211	0.203852116
ENSG00000150768	808.1905481	-0.12960563	0.077391068	-1.674684595	0.093996132	0.199903658
ENSG00000150776	264.4315784	-0.070027108	0.115849332	-0.604467082	0.545533148	0.694121751
ENSG00000150779	179.5896528	0.188037223	0.145146751	1.295497291	0.195148735	0.346334948
ENSG00000150787	89.03781873	0.160676178	0.188957584	0.850329338	0.39514201	0.563217718
ENSG00000150867	257.0204453	-0.219511984	0.126483283	-1.735501945	0.082651937	0.181096828
ENSG00000150893	72.37166346	-1.346420062	0.220218617	-6.114015624	9.72E-10	1.35E-08
ENSG00000150907	23.00477323	-1.437244083	0.388238952	-3.701957458	0.000213942	0.001137491
ENSG00000150938	69.0106442	-5.367075961	0.511357595	-10.49573922	9.04E-26	4.19E-24
ENSG00000150961	399.8381699	0.639377464	0.100229472	6.379136281	1.78E-10	2.73E-09
ENSG00000150967	116.8340404	0.030165086	0.168152047	0.179391727	0.857630127	0.91843641
ENSG00000150977	149.658455	0.198725014	0.146643224	1.355159887	0.175366622	0.31987728
ENSG00000150990	386.889426	0.33494561	0.116106368	2.884816886	0.003916414	0.014527031
ENSG00000150991	2602.184561	0.396674197	0.075016124	5.287852456	1.24E-07	1.28E-06
ENSG00000150995	105.1746204	-0.006926877	0.180724761	-0.038328325	0.969425907	0.984067475
ENSG00000151012	270.9927537	0.058465107	0.136747676	0.42754004	0.668986028	0.788491923
ENSG00000151014	170.4435784	-0.18521008	0.143578286	-1.289958839	0.19706495	0.348416784
ENSG00000151065	174.5715661	-0.124875345	0.152031755	-0.821376724	0.411431717	0.579793472
ENSG00000151067	34.52636669	-1.42565974	0.329550894	-4.326068498	1.52E-05	0.000104625
ENSG00000151092	174.3222267	0.180011045	0.149525158	1.203884668	0.228634159	0.387502899
ENSG00000151093	97.78190672	0.160492447	0.212403191	0.755602807	0.449887373	0.614923414
ENSG00000151116	150.9324062	-0.076494435	0.148369566	-0.515566886	0.606156943	0.743151644
ENSG00000151131	204.3373705	-0.041702466	0.1281517	-0.325414848	0.744867119	0.844210781
ENSG00000151135	310.5161543	0.205636098	0.112894728	1.821485395	0.068533105	0.156432324
ENSG00000151136	8.644226469	-2.398436994	0.749907339	-3.198311137	0.00138235	0.00596006
ENSG00000151148	318.8688954	-0.089523703	0.109619142	-0.816679475	0.414111624	0.582431606
ENSG00000151150	272.6639039	-1.933706538	0.13131943	-14.72521269	4.44E-49	4.62E-47
ENSG00000151151	88.93813845	-0.123270501	0.200911573	-0.613556	0.539508767	0.689580557
ENSG00000151176	2156.258	0.51409484	0.068510422	7.503892445	6.20E-14	1.37E-12
ENSG00000151208	762.6123597	0.157179451	0.078896567	1.992221675	0.046346741	0.114861092
ENSG00000151224	9.028087986	0.274219218	0.58102402	0.471958489	0.636956419	0.765710468
ENSG00000151229	114.4029454	-0.371520529	0.167466132	-2.218481577	0.026522014	0.072864216
ENSG00000151233	354.759307	-0.169934507	0.102420339	-1.659187116	0.097078093	0.204783897
ENSG00000151239	269.646311	0.508446192	0.117979327	4.309621077	1.64E-05	0.000111948
ENSG00000151240	287.923319	-0.106484819	0.116947495	-0.910535269	0.36254029	0.533424475
ENSG00000151247	400.9222043	0.029209515	0.104382135	0.279832513	0.779606006	0.868244866
ENSG00000151276	478.235265	-0.290229049	0.091776717	-3.162338525	0.001565075	0.00662187
ENSG00000151287	63.12020191	0.004438699	0.239221494	0.018554767	0.985196287	0.992402456
ENSG00000151292	283.9830123	-0.014217653	0.112262866	-0.126646093	0.899220514	0.945499634
ENSG00000151304	98.02512909	-0.142628627	0.183715363	-0.776356558	0.437538487	0.603314879
ENSG00000151320	47.07919439	0.376246786	0.255228062	1.474159162	0.140438742	0.271518409
ENSG00000151327	250.2051667	0.151954924	0.122184788	1.243648467	0.21362897	0.369153173
ENSG00000151332	89.80818704	-0.019213171	0.184723476	-0.104010447	0.917161058	0.955230368
ENSG00000151338	398.3088243	-0.266005279	0.105292668	-2.526341903	0.011525725	0.0363988
ENSG00000151348	275.4668506	-0.051344185	0.11585839	-0.443163284	0.657647634	0.780564116

ENSG00000151353	169.8138256	-0.358369865	0.141253685	-2.537065598	0.011178601	0.035454008
ENSG00000151366	20.15714608	0.180886285	0.418322673	0.432408514	0.665444531	0.786025079
ENSG00000151376	39.44081251	1.38390941	0.292699923	4.728082584	2.27E-06	1.87E-05
ENSG00000151413	73.98536306	0.296076166	0.210664845	1.405436992	0.159891373	0.298709153
ENSG00000151414	202.9626522	0.212473848	0.138474654	1.534388005	0.124934227	0.249318239
ENSG00000151422	254.2295524	-0.251171745	0.120421185	-2.085777056	0.036998823	0.09566031
ENSG00000151445	112.1987844	-0.148172107	0.17216207	-0.860654771	0.38942821	0.557852139
ENSG00000151458	368.9620391	-0.280441123	0.110302825	-2.542465471	0.011007348	0.034981948
ENSG00000151461	864.5928709	0.133468362	0.077419667	1.723959375	0.084715177	0.184666992
ENSG00000151465	662.1397208	0.162183868	0.084354129	1.922654779	0.05452342	0.130542096
ENSG00000151466	231.8660132	-0.496127698	0.130850785	-3.791553086	0.000149708	0.000826631
ENSG00000151468	67.47861053	0.678968214	0.234703707	2.892873838	0.003817346	0.014204572
ENSG00000151470	44.90003184	-0.487716594	0.264094231	-1.846752169	0.064783067	0.149721576
ENSG00000151474	7.951975029	-0.991064679	0.626121846	-1.582862323	0.113452848	0.231402956
ENSG00000151491	70.72884144	1.421575039	0.218288464	6.512369054	7.40E-11	1.19E-09
ENSG00000151498	89.51832149	-0.100559153	0.188791787	-0.532645802	0.59427879	0.734327425
ENSG00000151500	118.6411234	-0.038857074	0.162865592	-0.238583692	0.811428412	0.88902074
ENSG00000151502	221.0005	0.226467675	0.126654099	1.788080113	0.073763084	0.16528155
ENSG00000151503	698.1503961	-0.171472209	0.084246142	-2.035371635	0.041813493	0.105963222
ENSG00000151532	152.9084018	-0.482169139	0.14647046	-3.291920704	0.000995057	0.004472819
ENSG00000151552	144.2224423	-0.010078526	0.153111755	-0.065824639	0.94751744	0.970914655
ENSG00000151553	173.7002865	0.420630862	0.13803252	3.047331615	0.002308828	0.009232028
ENSG00000151575	64.59033909	-0.8953924	0.225990857	-3.962073568	7.43E-05	0.000440572
ENSG00000151576	230.0027246	0.034059792	0.125321244	0.271779878	0.785791278	0.87181187
ENSG00000151611	77.78429933	0.431892826	0.205193986	2.104802554	0.035308499	0.092052428
ENSG00000151612	581.4407106	-0.169562609	0.094662811	-1.791227285	0.073256829	0.164435711
ENSG00000151617	17.44990052	-0.256590945	0.411321273	-0.623821235	0.532744993	0.684234151
ENSG00000151640	221.4016217	0.561410498	0.126377527	4.44232857	8.90E-06	6.45E-05
ENSG00000151651	45.69215212	-0.07776725	0.260621126	-0.298391967	0.765404019	0.858571124
ENSG00000151657	54.80853908	0.041843284	0.233513988	0.179189626	0.857788809	0.918536317
ENSG00000151665	38.16391678	-0.065115199	0.291733679	-0.223200829	0.823379207	0.896520248
ENSG00000151687	19.67005733	-0.762393993	0.406120978	-1.877258339	0.060482695	0.141765463
ENSG00000151689	104.9229298	-0.549703527	0.177334373	-3.09981375	0.001936424	0.007960475
ENSG00000151693	137.4562917	-0.315917318	0.16025759	-1.971309552	0.048688479	0.119495927
ENSG00000151694	410.1997154	0.037371741	0.10206471	0.366157321	0.714247687	0.821841191
ENSG00000151718	678.1349819	-0.048236938	0.084171213	-0.573081185	0.566589726	0.712140827
ENSG00000151725	123.9765921	-0.398424554	0.1682366	-2.368239448	0.017872964	0.052666568
ENSG00000151726	442.5420945	0.897873868	0.098745522	9.092805947	9.65E-20	3.21E-18
ENSG00000151729	443.2087947	-0.817573947	0.096530823	-8.4695636	2.46E-17	6.93E-16
ENSG00000151743	53.29054788	0.27221441	0.24741927	1.100215072	0.271238425	0.438848901
ENSG00000151746	331.4618916	0.077006023	0.115878396	0.664541674	0.506343686	0.66386128
ENSG00000151748	274.1830707	0.407994678	0.113168876	3.605184505	0.000311931	0.001592187
ENSG00000151773	31.15788407	0.10863229	0.308676014	0.351929807	0.724890905	0.829602619
ENSG00000151779	712.7642242	0.228635448	0.087414251	2.615539762	0.008908655	0.029265639
ENSG00000151789	35.98026077	-1.786789705	0.325237388	-5.493801677	3.93E-08	4.43E-07
ENSG00000151806	764.3119001	0.593944778	0.093861706	6.327871121	2.49E-10	3.73E-09
ENSG00000151834	10.66928141	-1.094451803	0.555202089	-1.971267444	0.048693293	0.119495927
ENSG00000151835	1465.094284	-0.027636905	0.071042345	-0.389020176	0.697261221	0.810027539
ENSG00000151849	432.7580658	-0.195678832	0.098021104	-1.996292876	0.045902053	0.113991285
ENSG00000151876	18.03167693	-0.417479496	0.411227763	-1.015202603	0.31000926	0.481341889

ENSG00000151881	53.77022078	-0.028351121	0.243449189	-0.116456008	0.907291149	0.950460047
ENSG00000151883	199.7091738	-0.001252147	0.132465288	-0.009452644	0.992457994	0.996207876
ENSG00000151892	82.12375927	1.468863174	0.203153008	7.23032944	4.82E-13	9.88E-12
ENSG00000151893	446.8173723	0.043299442	0.097855145	0.442485084	0.658138222	0.781080399
ENSG00000151914	2719.523025	0.34717623	0.064883424	5.350769242	8.76E-08	9.31E-07
ENSG00000151923	607.5189128	0.201255907	0.085509022	2.353621904	0.018591511	0.054376477
ENSG00000151929	560.4397857	0.556984088	0.09540834	5.837897254	5.29E-09	6.74E-08
ENSG00000151948	85.89543296	0.415485134	0.1903523	2.182716652	0.029056683	0.078492159
ENSG00000151967	22.25074896	-6.784478548	1.215116946	-5.58339555	2.36E-08	2.75E-07
ENSG00000152056	132.6855537	0.143466942	0.164464548	0.872327465	0.383029758	0.552444955
ENSG00000152061	300.6611348	-0.280012359	0.110431554	-2.535619121	0.011224874	0.035568647
ENSG00000152076	31.87741773	0.450226793	0.325050336	1.385098685	0.166022294	0.307748108
ENSG00000152078	78.20132192	-0.381747341	0.211857728	-1.801904253	0.071560471	0.161765358
ENSG00000152082	1102.373087	0.43802388	0.078126374	5.606607041	2.06E-08	2.42E-07
ENSG00000152102	1126.796291	0.007010678	0.077811827	0.090097847	0.92820946	0.960837015
ENSG00000152104	890.176805	0.301349142	0.083539445	3.607267718	0.000309438	0.001581186
ENSG00000152117	69.30167861	0.140771835	0.215463511	0.6533442	0.513534403	0.669454701
ENSG00000152127	524.2285324	-0.070376609	0.090519399	-0.77747544	0.436878319	0.602891221
ENSG00000152133	327.3399046	0.520885177	0.106357248	4.897505208	9.71E-07	8.62E-06
ENSG00000152147	177.9146378	0.131463761	0.137617247	0.955285503	0.339433337	0.511206184
ENSG00000152193	195.4772887	-0.037628792	0.141041301	-0.266792719	0.789628761	0.874348005
ENSG00000152207	488.7625398	2.677271054	0.111996411	23.90497186	2.72E-126	1.01E-123
ENSG00000152219	148.5419626	-0.406050068	0.152850045	-2.656525665	0.007895044	0.026455555
ENSG00000152223	360.4141217	0.30931362	0.10290549	3.005802887	0.002648806	0.010390333
ENSG00000152229	88.3477028	-0.41285419	0.190878275	-2.162918696	0.030547434	0.081780026
ENSG00000152234	1817.96185	0.057933015	0.069999086	0.827625318	0.407882725	0.576353349
ENSG00000152240	139.5147547	0.349407517	0.153640866	2.274183464	0.022954958	0.064554213
ENSG00000152242	251.2177182	0.054689044	0.119989164	0.455783187	0.648545895	0.773815017
ENSG00000152253	109.3432014	-0.840346697	0.175639301	-4.784502625	1.71E-06	1.45E-05
ENSG00000152256	269.5155353	0.407815021	0.115454109	3.532269446	0.000412009	0.002034269
ENSG00000152270	361.892165	0.144642033	0.108553207	1.332452876	0.182711417	0.329805612
ENSG00000152284	84.62103844	-4.448812664	0.355937621	-12.49885485	7.57E-36	5.07E-34
ENSG00000152291	708.3465104	0.213142387	0.085465263	2.493906637	0.012634577	0.039369544
ENSG00000152332	775.3111236	0.236124672	0.077447157	3.048848806	0.002297201	0.009196004
ENSG00000152348	81.2335843	-0.191560454	0.204844658	-0.935149867	0.34971107	0.521762231
ENSG00000152359	63.06240758	-0.411169105	0.230240343	-1.785825631	0.074127495	0.16596572
ENSG00000152380	32.18049655	0.509655019	0.306144659	1.664752284	0.095962187	0.203174872
ENSG00000152382	127.1392089	-0.196136422	0.159609628	-1.228850824	0.219127742	0.376294302
ENSG00000152404	208.2104664	-0.18170394	0.131707072	-1.379606559	0.167707817	0.309972593
ENSG00000152409	348.4680864	0.2310385	0.10698616	2.159517637	0.030810031	0.082326136
ENSG00000152413	258.9348087	-0.433832372	0.136208539	-3.185060023	0.00144724	0.006196092
ENSG00000152422	72.74665837	0.161512443	0.205322412	0.786628416	0.43149942	0.597805177
ENSG00000152439	29.90153545	0.365107634	0.322580266	1.131834996	0.257703812	0.423403097
ENSG00000152443	67.41062715	-0.191724094	0.248451169	-0.771677163	0.44030566	0.605657423
ENSG00000152454	77.46478706	-0.020540259	0.198735477	-0.103354767	0.917681411	0.955355281
ENSG00000152455	112.0567615	-0.086782951	0.186469502	-0.465400239	0.641644863	0.768715172
ENSG00000152457	272.8509281	-0.075996163	0.121245074	-0.626797946	0.53079169	0.682911785
ENSG00000152464	226.8717901	0.06301145	0.146810555	0.429202447	0.667775902	0.787594299
ENSG00000152465	73.3042428	-0.516495554	0.215389724	-2.397958195	0.016486747	0.04930642
ENSG00000152475	18.62988475	-0.877829776	0.420783268	-2.086180328	0.036962292	0.095583452

ENSG00000152484	287.6149336	-0.070731756	0.11438063	-0.618389283	0.53631876	0.687065174
ENSG00000152492	483.6587743	-0.78165221	0.099088786	-7.888402343	3.06E-15	7.53E-14
ENSG00000152495	17.44987354	1.63094773	0.43988359	3.707680323	0.000209166	0.001114629
ENSG00000152518	1053.405404	0.193645639	0.086950074	2.227089994	0.02594126	0.071548572
ENSG00000152520	213.7064	-0.196314727	0.127503973	-1.539675367	0.123639504	0.247613931
ENSG00000152527	23.72055132	-2.875853086	0.466942237	-6.158905442	7.32E-10	1.04E-08
ENSG00000152556	533.987955	0.169326343	0.08829499	1.917734446	0.055144692	0.131782491
ENSG00000152558	896.7282236	0.244233358	0.08596777	2.840987462	0.004497408	0.016360121
ENSG00000152578	43.33159246	-1.715617093	0.29799216	-5.757255797	8.55E-09	1.06E-07
ENSG00000152580	20.98501885	-2.804244554	0.489516104	-5.728605313	1.01E-08	1.24E-07
ENSG00000152582	23.49295609	-2.784776701	0.458237333	-6.077149328	1.22E-09	1.69E-08
ENSG00000152601	862.8900233	0.543156302	0.075357407	7.207736125	5.69E-13	1.16E-11
ENSG00000152620	315.6770877	-0.58269766	0.108960142	-5.347805616	8.90E-08	9.44E-07
ENSG00000152642	187.0214205	0.313793779	0.1371911	2.287275042	0.022179774	0.062675375
ENSG00000152661	1049.197621	-5.191579219	0.13557128	-38.29409298	0	0
ENSG00000152669	62.11081584	1.661659958	0.237279061	7.002977635	2.51E-12	4.80E-11
ENSG00000152683	277.17297	-0.237364047	0.125417305	-1.89259406	0.058411874	0.137704241
ENSG00000152684	154.1019376	0.437154933	0.145704849	3.000277181	0.00269734	0.01053365
ENSG00000152700	166.2846989	-0.065428597	0.146248987	-0.447378119	0.654602072	0.778462183
ENSG00000152749	203.7638569	-0.124377589	0.128798121	-0.965678595	0.334205033	0.505990311
ENSG00000152763	14.75154547	-1.325911053	0.479433357	-2.765579474	0.005682176	0.019909584
ENSG00000152767	676.5908682	-0.40159982	0.085638085	-4.689500233	2.74E-06	2.22E-05
ENSG00000152778	116.3447204	0.297984931	0.167643105	1.777495898	0.075486685	0.168338064
ENSG00000152782	114.4559387	-0.22802481	0.168261989	-1.35517719	0.17536111	0.31987728
ENSG00000152795	2568.740612	0.257260143	0.06022574	4.27159784	1.94E-05	0.000130628
ENSG00000152804	87.97201543	0.58685397	0.197296256	2.974481025	0.002934846	0.011319545
ENSG00000152818	740.8795496	-1.013188747	0.093181408	-10.873293	1.55E-27	7.67E-26
ENSG00000152894	55.43794226	0.361248493	0.239507179	1.508299228	0.131477961	0.258882943
ENSG00000152904	77.61107355	0.009890196	0.217375657	0.045498177	0.963710228	0.981000042
ENSG00000152926	20.87237852	-1.170391832	0.414634181	-2.822709477	0.00476197	0.017178068
ENSG00000152942	234.2519948	0.093242672	0.123546443	0.754717574	0.45041846	0.615349942
ENSG00000152944	130.4552521	0.111448084	0.167293681	0.666182268	0.505294605	0.662868093
ENSG00000152952	242.5592504	-0.262586521	0.1233869	-2.128155578	0.033324187	0.087791607
ENSG00000152977	33.58267305	-0.053871571	0.305346945	-0.176427411	0.859958171	0.920195816
ENSG00000152990	538.2312073	-0.144656425	0.088430736	-1.635816147	0.101878072	0.212800189
ENSG00000153006	350.3849158	-0.065763538	0.102754898	-0.640003929	0.522170045	0.676154371
ENSG00000153012	127.1535826	-2.718403565	0.19966126	-13.61507769	3.26E-42	2.72E-40
ENSG00000153015	653.9760064	-0.030786777	0.086984487	-0.353934111	0.723388263	0.828557969
ENSG00000153029	99.85365173	-0.730368571	0.180450683	-4.047469143	5.18E-05	0.000317172
ENSG00000153037	193.1861907	-0.039559137	0.131107881	-0.301729663	0.762858153	0.856873889
ENSG00000153044	55.60322834	-0.177046648	0.243697319	-0.726502239	0.467530936	0.629756504
ENSG00000153046	422.7511986	-0.070382866	0.106606124	-0.660214099	0.509116446	0.665756722
ENSG00000153048	193.2224985	0.288460924	0.136135078	2.118931634	0.034096245	0.089523356
ENSG00000153066	157.8269637	0.031831028	0.145683848	0.218493875	0.82704433	0.898668717
ENSG00000153071	8.792110627	-5.408121454	1.276524102	-4.236599563	2.27E-05	0.000150861
ENSG00000153094	101.1496481	-0.852062654	0.181843897	-4.685681877	2.79E-06	2.26E-05
ENSG00000153107	347.5270647	0.068300148	0.103361778	0.660787281	0.508748741	0.665497779
ENSG00000153113	502.0885284	-0.406156431	0.098499212	-4.123448542	3.73E-05	0.000236381
ENSG00000153130	137.2992015	-0.193410473	0.15292868	-1.264710276	0.205975205	0.359509655
ENSG00000153132	69.23771748	-0.458391812	0.21747438	-2.107796841	0.035048566	0.091544665

ENSG00000153140	189.1297361	-0.005789429	0.13560674	-0.04269278	0.965946435	0.982381232
ENSG00000153147	1435.961008	-0.878810552	0.06970632	-12.60732953	1.92E-36	1.31E-34
ENSG00000153157	9.580793476	-0.711713111	0.580614456	-1.225792956	0.220276598	0.377897579
ENSG00000153179	288.2279619	-0.393736318	0.122252038	-3.220693301	0.001278809	0.005559713
ENSG00000153187	10902.7186	0.110070175	0.050510001	2.179175857	0.029318607	0.078971902
ENSG00000153201	963.1006137	-0.038348901	0.092786738	-0.413301528	0.67938571	0.796724373
ENSG00000153207	1255.674134	0.140240345	0.067504183	2.077505989	0.037754883	0.097221419
ENSG00000153208	284.2624848	-0.46859208	0.114190396	-4.103603254	4.07E-05	0.000255424
ENSG00000153214	255.8560662	-0.391879447	0.120563272	-3.250404879	0.001152408	0.005084132
ENSG00000153233	6.491075903	-5.978368539	1.300823952	-4.595832151	4.31E-06	3.34E-05
ENSG00000153234	93.95445533	-0.104976484	0.184620199	-0.568607792	0.569622336	0.714810407
ENSG00000153250	644.8154937	1.255867517	0.084297652	14.89801297	3.40E-50	3.56E-48
ENSG00000153310	365.6518568	-0.200851587	0.103169414	-1.946813303	0.051557121	0.12477996
ENSG00000153317	2493.389246	0.195004744	0.067691559	2.880783767	0.003966877	0.014710327
ENSG00000153339	290.1593015	0.048499791	0.113163243	0.428582547	0.668227049	0.78799407
ENSG00000153363	115.8997561	-0.468300375	0.171365245	-2.732761684	0.006280576	0.021676474
ENSG00000153391	16.27340842	0.788023849	0.490710786	1.605882469	0.108299736	0.223226176
ENSG00000153395	827.2183754	-0.155299342	0.078952479	-1.966997667	0.04918348	0.120320505
ENSG00000153406	454.3141733	-0.062183971	0.108298437	-0.574190848	0.565838662	0.711478939
ENSG00000153443	360.2963454	-0.086610253	0.105539739	-0.820641149	0.411850702	0.580267483
ENSG00000153485	16.28359987	-0.699759369	0.454198043	-1.540648136	0.123402446	0.247209663
ENSG00000153487	273.117904	-0.203677869	0.122415322	-1.663826595	0.096147088	0.203461606
ENSG00000153531	44.22164912	-1.102991048	0.272381709	-4.049431416	5.13E-05	0.000314799
ENSG00000153551	346.0579323	0.215914223	0.102970659	2.096851915	0.036006686	0.093629382
ENSG00000153558	35.8397655	-0.298869145	0.311502445	-0.959443978	0.337335129	0.509302081
ENSG00000153560	1519.525932	0.101990692	0.072610592	1.404625532	0.16013266	0.299120143
ENSG00000153561	514.7350736	-0.183441824	0.094916637	-1.932662479	0.05327779	0.128105635
ENSG00000153574	228.134717	-0.123441046	0.130960126	-0.942584963	0.345893234	0.517715745
ENSG00000153707	511.9889262	-0.998208776	0.097250906	-10.26426198	1.02E-24	4.47E-23
ENSG00000153714	287.058316	0.233843741	0.113264756	2.064576383	0.0389631	0.099747524
ENSG00000153721	164.2750595	-0.280732923	0.142280515	-1.973094646	0.04848478	0.119099371
ENSG00000153767	249.5017579	-0.178539054	0.118016543	-1.512830732	0.13032266	0.257112698
ENSG00000153774	479.4296073	-0.127991048	0.100183647	-1.277564277	0.201403103	0.353805345
ENSG00000153786	386.321217	-0.035783896	0.101637666	-0.352073175	0.724783385	0.829602619
ENSG00000153790	13.13884985	0.418000726	0.475559363	0.878966452	0.379419466	0.548944818
ENSG00000153814	29.14284259	-1.361358274	0.350332984	-3.885898095	0.000101952	0.000585975
ENSG00000153815	493.9340707	0.082527654	0.090316774	0.913757773	0.360844139	0.532058728
ENSG00000153827	1376.076067	-0.071592483	0.07413484	-0.965706304	0.334191164	0.505990311
ENSG00000153832	29.14707394	-0.771252554	0.340167571	-2.267272429	0.023373589	0.065547774
ENSG00000153879	323.988413	-0.359155918	0.111784197	-3.212939983	0.001313837	0.005694376
ENSG00000153885	290.2583482	-0.446696617	0.113709792	-3.928391813	8.55E-05	0.000500924
ENSG00000153896	16.69266277	0.264880015	0.417706621	0.634129318	0.525996431	0.679230728
ENSG00000153898	100.3608989	-0.119517536	0.18974678	-0.629879129	0.528773669	0.681438874
ENSG00000153902	18.59682087	-0.805516231	0.432898452	-1.86075101	0.062779347	0.145931074
ENSG00000153904	112.2366106	0.498681679	0.170726353	2.920941429	0.003489754	0.013166956
ENSG00000153914	1735.214984	0.060357588	0.075603781	0.798340868	0.424672708	0.591862043
ENSG00000153922	1078.423022	-0.092897611	0.072430351	-1.282578506	0.199639784	0.351641386
ENSG00000153933	269.1571649	-0.198358859	0.116619521	-1.700906137	0.088960614	0.191549229
ENSG00000153936	310.4481682	-0.343541335	0.111741178	-3.0744381	0.002108995	0.008527648
ENSG00000153944	727.3475504	0.087344477	0.083315765	1.048354748	0.294475194	0.464722284

ENSG00000153956	1025.77334	-0.079609528	0.076489348	-1.040792343	0.297971934	0.468661042
ENSG00000153975	62.92542153	-0.550982276	0.224663747	-2.452475239	0.014187714	0.043381546
ENSG00000153982	73.34471112	-0.018018624	0.20511624	-0.087845914	0.929999145	0.962051549
ENSG00000153989	72.31332921	0.158381024	0.233262815	0.67898102	0.497149888	0.655478175
ENSG00000153993	36.87379583	-6.67623033	1.022936113	-6.526536941	6.73E-11	1.10E-09
ENSG00000154001	281.0907474	-0.174584314	0.113281585	-1.541153524	0.123279426	0.247068925
ENSG00000154027	9.867035778	1.309533822	0.571866632	2.289928714	0.022025449	0.062427661
ENSG00000154040	129.2207205	-0.444128894	0.159184039	-2.79003408	0.005270249	0.01871838
ENSG00000154059	149.2429849	0.081753253	0.146800348	0.556900948	0.577595107	0.721236186
ENSG00000154079	35.09178103	0.188681793	0.304011252	0.620640818	0.534835976	0.685853657
ENSG00000154096	461.2375237	0.375941839	0.092785793	4.051717691	5.08E-05	0.000312283
ENSG00000154102	23.04028638	0.111264643	0.369686149	0.300970549	0.763436953	0.857280464
ENSG00000154114	103.9984885	-0.077063492	0.180517714	-0.426902658	0.66945023	0.788840481
ENSG00000154122	70.08481213	3.902398136	0.319225189	12.2245934	2.30E-34	1.45E-32
ENSG00000154124	236.9508032	0.111420204	0.122430334	0.91007025	0.362785463	0.533729304
ENSG00000154127	69.63871954	0.738729566	0.222733578	3.316651105	0.000911033	0.004138836
ENSG00000154134	60.6523935	-0.163911014	0.228245088	-0.718135997	0.472673437	0.633981332
ENSG00000154144	195.1415708	-0.263438375	0.131668833	-2.000764872	0.045417735	0.11307744
ENSG00000154146	368.6515208	0.754089288	0.109153307	6.90853356	4.90E-12	8.97E-11
ENSG00000154174	346.7388804	0.276127655	0.102787255	2.68639973	0.007222662	0.024506458
ENSG00000154188	17.30763997	-0.801295905	0.423575553	-1.891742568	0.058525289	0.137913895
ENSG00000154217	175.9526395	-0.179755704	0.143777214	-1.25023777	0.211212703	0.366284549
ENSG00000154222	186.7141179	0.369354878	0.142303253	2.595547688	0.00944403	0.030743982
ENSG00000154229	335.7383557	0.301453281	0.107415813	2.806414361	0.005009623	0.017937846
ENSG00000154237	118.1461317	-3.103967217	0.223167595	-13.90868245	5.61E-44	4.86E-42
ENSG00000154240	235.9875479	-0.363260699	0.128079126	-2.836220928	0.004565086	0.016574246
ENSG00000154262	13.27260348	-2.901010152	0.622861862	-4.657549817	3.20E-06	2.55E-05
ENSG00000154263	23.54282172	-2.437613114	0.435749567	-5.594068928	2.22E-08	2.60E-07
ENSG00000154265	113.1696655	-1.021714646	0.184331762	-5.54280302	2.98E-08	3.42E-07
ENSG00000154277	258.3980099	1.39828758	0.127879982	10.93437422	7.89E-28	3.95E-26
ENSG00000154305	1656.189047	0.373787169	0.06639635	5.629634318	1.81E-08	2.14E-07
ENSG00000154309	96.52776868	0.000127887	0.197764745	0.000646661	0.999484039	0.999854605
ENSG00000154310	96.17843423	0.714513843	0.180503132	3.958456754	7.54E-05	0.000446542
ENSG00000154319	8.725252831	-1.959265798	0.669820043	-2.92506296	0.003443865	0.01301129
ENSG00000154328	147.1255448	0.273353714	0.14984887	1.824196032	0.068122441	0.155824122
ENSG00000154358	2096.23241	1.533665204	0.138842663	11.04606586	2.29E-28	1.17E-26
ENSG00000154359	181.3544463	-0.07770483	0.139322328	-0.557734222	0.577025885	0.720935926
ENSG00000154370	474.3567099	0.028693227	0.09876636	0.290516197	0.771421361	0.863328623
ENSG00000154380	2044.426561	-0.085715848	0.061659185	-1.390155373	0.164481702	0.305456698
ENSG00000154429	282.5382209	-0.230340997	0.116356459	-1.97961505	0.047746801	0.117645906
ENSG00000154447	62.45073345	0.485602583	0.234317159	2.0724158	0.038226681	0.098202302
ENSG00000154473	772.1463462	0.197232197	0.087716364	2.248522255	0.024542908	0.068228028
ENSG00000154511	34.36024414	-0.745491653	0.306435718	-2.432783152	0.014983271	0.045467706
ENSG00000154518	516.6178359	0.286677463	0.101344592	2.828739634	0.00467317	0.016901138
ENSG00000154529	23.17139901	0.053962038	0.356527471	0.151354501	0.879696079	0.934004754
ENSG00000154548	44.78357483	-0.06264454	0.261665978	-0.239406515	0.810790378	0.888548987
ENSG00000154556	459.2674275	-2.098504894	0.106064924	-19.78509782	4.00E-87	8.52E-85
ENSG00000154582	457.381564	0.148392829	0.101660664	1.459687787	0.1443759	0.277272848
ENSG00000154608	11.9904205	0.317725652	0.498026233	0.637969711	0.523493397	0.677001436
ENSG00000154639	34.77096918	-2.542596548	0.371205695	-6.849562329	7.41E-12	1.32E-10

ENSG00000154640	229.2355653	0.341526448	0.124366806	2.746122208	0.00603043	0.020931417
ENSG00000154642	213.2005805	0.013089018	0.127246992	0.102863084	0.918071637	0.955355281
ENSG00000154645	42.2567958	1.305558817	0.282025736	4.629218725	3.67E-06	2.90E-05
ENSG00000154646	3615.779027	0.312842432	0.06220283	5.029392293	4.92E-07	4.65E-06
ENSG00000154654	188.9870979	1.100207964	0.139423053	7.891148123	2.99E-15	7.39E-14
ENSG00000154710	19.88321666	0.041721724	0.412045773	0.10125507	0.919347982	0.955678317
ENSG00000154719	121.3918639	-0.213005175	0.171810675	-1.239766828	0.215061651	0.371172258
ENSG00000154721	201.6852213	-0.150320246	0.129002531	-1.165250361	0.243917666	0.407118756
ENSG00000154723	1560.093011	0.263338168	0.07300703	3.607024788	0.000309728	0.001582091
ENSG00000154727	258.9651096	0.129028558	0.115734653	1.114865379	0.264908115	0.431659069
ENSG00000154734	1205.46051	-1.570066072	0.071780917	-21.87302897	4.69E-106	1.32E-103
ENSG00000154743	303.5568932	0.091084566	0.110480767	0.824438209	0.409690612	0.578151901
ENSG00000154760	109.7363758	-2.08754427	0.199872086	-10.44440125	1.55E-25	7.14E-24
ENSG00000154767	566.4653019	-0.081997	0.088059817	-0.931151151	0.351775382	0.523452203
ENSG00000154781	285.3484733	0.295329859	0.111810772	2.641336365	0.008257969	0.027501111
ENSG00000154783	19.34113613	-4.703738759	0.790722946	-5.94865595	2.70E-09	3.55E-08
ENSG00000154803	84.75961717	0.496315744	0.209727411	2.366480093	0.01795814	0.052884275
ENSG00000154813	103.823525	0.143869263	0.176187414	0.81656947	0.414174508	0.582431606
ENSG00000154814	217.4623182	-0.066767298	0.127239928	-0.524735422	0.599767129	0.738316437
ENSG00000154822	266.1808789	0.948571089	0.116727263	8.126388528	4.42E-16	1.16E-14
ENSG00000154832	291.6846499	0.437671477	0.115291654	3.796211291	0.000146924	0.000813178
ENSG00000154839	152.867193	0.123921474	0.159907784	0.774955858	0.438365744	0.603816788
ENSG00000154845	296.3862497	-0.166568291	0.110799251	-1.503334085	0.132752915	0.260917895
ENSG00000154889	43.98669331	-0.034850868	0.25929123	-0.134408202	0.893079796	0.941935367
ENSG00000154914	41.64271801	-0.107970823	0.271517079	-0.397657574	0.690882615	0.805446767
ENSG00000154917	161.757764	-0.881792032	0.159658129	-5.523001149	3.33E-08	3.80E-07
ENSG00000154920	128.852936	-0.463908686	0.163879106	-2.830798241	0.0046432	0.016830556
ENSG00000154930	178.4575881	1.350473527	0.147422405	9.160571807	5.16E-20	1.75E-18
ENSG00000154945	851.2343164	-0.14285888	0.077395252	-1.845835186	0.06491614	0.149979773
ENSG00000154957	40.8157605	0.075180769	0.279437714	0.269043029	0.787896571	0.873121205
ENSG00000154978	109.0946377	0.113087965	0.172046867	0.657309065	0.510982209	0.667402909
ENSG00000155008	197.6236139	-0.361710336	0.146437248	-2.470070563	0.01350864	0.041631389
ENSG00000155011	14.70971797	-1.704429584	0.499553096	-3.411908759	0.000645097	0.003035738
ENSG00000155016	38.95799726	-0.060898871	0.276889408	-0.219939331	0.825918404	0.898324082
ENSG00000155034	188.4006306	0.26667352	0.161364845	1.652612247	0.098409804	0.207126538
ENSG00000155090	901.1648922	-0.108240996	0.07847821	-1.379249046	0.167817981	0.310059773
ENSG00000155096	899.1712758	-0.342321926	0.076246082	-4.489698608	7.13E-06	5.28E-05
ENSG00000155097	496.4468421	0.353505381	0.092874235	3.806280399	0.000141072	0.000784499
ENSG00000155099	221.1638619	0.329478622	0.125942929	2.616094637	0.00889419	0.029224957
ENSG00000155100	236.6611783	0.376555699	0.120500751	3.124924081	0.00177851	0.007389152
ENSG00000155111	172.407328	0.083559836	0.138615695	0.60281656	0.546630729	0.695077564
ENSG00000155115	296.1356379	0.415670396	0.112927288	3.680867597	0.000232442	0.001222887
ENSG00000155158	89.28272933	-0.723967172	0.189787246	-3.814625004	0.00013639	0.000761474
ENSG00000155189	370.6167205	-0.357376119	0.101808717	-3.510270332	0.000447651	0.00219482
ENSG00000155229	375.2037508	0.095626475	0.101795267	0.93940002	0.347525403	0.519343415
ENSG00000155252	178.0401171	-0.087357578	0.141834572	-0.615911739	0.537952774	0.688718794
ENSG00000155254	474.5571152	0.078503781	0.094772196	0.828341901	0.4074769	0.575837828
ENSG00000155256	123.4616073	-0.00667178	0.159196376	-0.041909119	0.966571147	0.98273197
ENSG00000155275	134.9493887	-0.041738295	0.162351774	-0.257085548	0.797112734	0.878938874
ENSG00000155287	76.01010514	0.10830516	0.200875748	0.539164937	0.589773052	0.731165495

ENSG00000155304	306.9202842	-0.184079475	0.116465701	-1.580546659	0.11398173	0.232077311
ENSG00000155307	35.45947031	-1.095607667	0.313852245	-3.490839035	0.000481506	0.002342819
ENSG00000155313	424.941762	0.140025376	0.098388683	1.423185792	0.154682303	0.291967524
ENSG00000155329	205.6315078	-0.148015336	0.129322864	-1.144541121	0.252399295	0.417013984
ENSG00000155330	121.2990778	-0.284787655	0.164891565	-1.727120818	0.084145968	0.183654196
ENSG00000155363	972.1081813	0.089735952	0.074258892	1.208420285	0.226885641	0.385361548
ENSG00000155366	413.9593883	-0.201483807	0.097187579	-2.073143589	0.038158918	0.098046154
ENSG00000155367	18.09867398	0.452792513	0.433571726	1.044331274	0.296332149	0.466656692
ENSG00000155368	1307.985858	0.171376707	0.069555894	2.463870374	0.013744581	0.042228812
ENSG00000155380	712.9334821	-0.004253248	0.081540326	-0.052161284	0.958400182	0.977701483
ENSG00000155393	178.5919966	0.146687091	0.138537528	1.058825672	0.28967918	0.459891889
ENSG00000155438	298.5365636	0.200204897	0.109859853	1.822366333	0.068399419	0.156228723
ENSG00000155463	1473.531109	-0.126824389	0.069023384	-1.837411933	0.066149107	0.152202539
ENSG00000155506	4284.089127	0.160889943	0.053601524	3.001592728	0.002685712	0.01050379
ENSG00000155508	271.8991758	-0.033199702	0.12255871	-0.270888148	0.78647706	0.87222827
ENSG00000155542	26.17434976	0.659511385	0.344134649	1.916434126	0.055309861	0.132109779
ENSG00000155545	218.7190818	-0.154503399	0.13390822	-1.153800707	0.248581884	0.412749377
ENSG00000155561	1296.305515	-0.094916546	0.077623645	-1.222778785	0.22141326	0.379199228
ENSG00000155592	198.4155716	-0.356264887	0.132278605	-2.69329183	0.007075031	0.024046204
ENSG00000155621	76.41104938	-0.842220203	0.210856119	-3.994288644	6.49E-05	0.00038986
ENSG00000155629	51.91310254	2.575848881	1.215989297	2.118315423	0.034148363	0.089643442
ENSG00000155636	42.36490577	0.104004085	0.269307481	0.386190849	0.69935533	0.811284526
ENSG00000155657	12.27255121	0.074310868	0.504635626	0.147256483	0.882929579	0.935529895
ENSG00000155660	2919.13742	1.035857301	0.085821839	12.06985674	1.52E-33	9.39E-32
ENSG00000155666	36.67714799	0.470626825	0.300251425	1.567442437	0.117011311	0.236872444
ENSG00000155729	97.22591831	-0.09740146	0.184502602	-0.527913749	0.597559201	0.736825325
ENSG00000155744	109.1973941	-0.395377384	0.170414986	-2.320085776	0.020336238	0.058573535
ENSG00000155755	204.6842244	-0.086903402	0.133804033	-0.649482678	0.516026439	0.671168832
ENSG00000155760	227.9867956	-0.613611191	0.127297041	-4.820309918	1.43E-06	1.23E-05
ENSG00000155792	15.7220831	0.443839441	0.450720176	0.984733908	0.324754793	0.496769092
ENSG00000155827	462.2578591	-0.137140707	0.092113964	-1.488815603	0.136535932	0.266119085
ENSG00000155846	456.2618478	-0.066627734	0.09264999	-0.719133741	0.47205852	0.633818202
ENSG00000155850	193.8478568	0.064682957	0.142410541	0.454200625	0.649684433	0.774449867
ENSG00000155858	59.21839009	0.09874422	0.226442656	0.436067221	0.662787959	0.784604829
ENSG00000155868	24.47077154	-0.080673807	0.351354406	-0.229608071	0.818396334	0.892848047
ENSG00000155876	119.8473029	0.025556943	0.169956598	0.150373351	0.880470065	0.934116144
ENSG00000155893	42.12363706	-0.310224054	0.277364538	-1.118470503	0.263366098	0.429874922
ENSG00000155903	134.3540292	0.232331487	0.15710301	1.478848096	0.139180918	0.269814292
ENSG00000155906	155.0272986	0.198269663	0.166287205	1.19233265	0.233130837	0.393279993
ENSG00000155957	14.09588504	0.203566002	0.47371605	0.429721564	0.667398195	0.787413177
ENSG00000155959	233.8324939	0.101721346	0.12438315	0.817806481	0.413467697	0.581859088
ENSG00000155961	47.30106439	-0.104249431	0.269036807	-0.387491334	0.698392499	0.810770201
ENSG00000155970	36.65757226	0.183677434	0.288488173	0.636689649	0.524327017	0.677697977
ENSG00000155975	213.1966823	0.096086605	0.129080855	0.74439083	0.456640065	0.620271199
ENSG00000155980	123.7759067	-0.444780696	0.169248284	-2.62797758	0.008589418	0.028429677
ENSG00000156011	197.6343743	-0.392150997	0.132482341	-2.960024663	0.003076144	0.011786851
ENSG00000156017	318.1523013	0.302394391	0.106840885	2.830324664	0.004650079	0.016834964
ENSG00000156026	99.33972657	-0.141862726	0.180947051	-0.784001319	0.433039336	0.599401284
ENSG00000156030	1022.505551	0.046736976	0.074245939	0.629488651	0.529029196	0.681643104
ENSG00000156042	9.145912326	-0.146858509	0.568809931	-0.258185557	0.796263704	0.878366283

ENSG00000156050	20.68011802	-0.033312967	0.395441785	-0.084242404	0.932863705	0.963270344
ENSG00000156052	406.2003858	-0.181760059	0.09850821	-1.845125998	0.065019212	0.150168509
ENSG00000156076	19.04146636	-5.12937356	0.896999818	-5.718366332	1.08E-08	1.31E-07
ENSG00000156103	60.19014916	-6.39045084	0.746397165	-8.561729786	1.11E-17	3.22E-16
ENSG00000156110	129.4618407	0.030551706	0.175667249	0.173918054	0.861929864	0.921617562
ENSG00000156136	104.1232571	-0.011474462	0.184605825	-0.062156552	0.950438162	0.972594511
ENSG00000156140	111.038815	-1.59182116	0.18381389	-8.659961231	4.72E-18	1.42E-16
ENSG00000156162	175.1346733	-0.053713328	0.1427879	-0.376175629	0.706786332	0.815929291
ENSG00000156170	89.54628383	0.288264202	0.188622837	1.52825717	0.1264487	0.251483064
ENSG00000156171	184.9075295	0.008508473	0.139646294	0.06092874	0.951415961	0.973382375
ENSG00000156172	71.21922997	-0.278515117	0.20962343	-1.328644975	0.183965127	0.331387296
ENSG00000156232	118.3197175	0.015437842	0.163631115	0.094345395	0.924834791	0.958816254
ENSG00000156239	67.88608711	0.121582881	0.211348676	0.575271551	0.565107659	0.71091414
ENSG00000156253	80.76876216	-0.441690722	0.199675564	-2.212041937	0.026963767	0.073917331
ENSG00000156256	337.7153021	-0.155161593	0.104150481	-1.489782777	0.136281361	0.265807314
ENSG00000156261	1959.398914	-0.353521887	0.062943759	-5.616472427	1.95E-08	2.29E-07
ENSG00000156265	20.77744572	-1.750169314	0.425363592	-4.114525428	3.88E-05	0.00024472
ENSG00000156273	242.847699	0.01976444	0.126572303	0.156151381	0.875913699	0.931201069
ENSG00000156299	150.2566612	-0.740558114	0.150319449	-4.926562205	8.37E-07	7.54E-06
ENSG00000156304	946.7414271	-0.06397915	0.073302963	-0.872804418	0.382769691	0.552299228
ENSG00000156313	93.49326121	-0.218676673	0.18459854	-1.184606731	0.236172975	0.397355584
ENSG00000156345	64.38551322	0.58158398	0.227132072	2.560554194	0.010450536	0.033462203
ENSG00000156374	110.6361362	0.109662423	0.181710901	0.603499417	0.546176503	0.694729455
ENSG00000156381	148.953503	0.067429957	0.15083373	0.447048264	0.654840213	0.77867946
ENSG00000156384	21.22110864	0.295266923	0.38543608	0.766059376	0.443640973	0.60851721
ENSG00000156398	140.7594793	-0.178072895	0.155223619	-1.147202314	0.251298015	0.415585498
ENSG00000156411	1086.120294	0.513353312	0.07235261	7.095159543	1.29E-12	2.55E-11
ENSG00000156453	18.55267577	-4.338159763	0.729830941	-5.944061181	2.78E-09	3.65E-08
ENSG00000156463	114.403769	-1.318281384	0.189122268	-6.970524409	3.16E-12	5.93E-11
ENSG00000156467	3598.364346	0.22889147	0.065848388	3.47603757	0.000508881	0.002458133
ENSG00000156469	109.5043449	0.260469093	0.173090126	1.504817749	0.132370939	0.260385742
ENSG00000156471	767.7310639	0.211488879	0.082646991	2.558942279	0.010499117	0.033579485
ENSG00000156482	8199.538328	0.214376062	0.065869467	3.254558934	0.001135686	0.005024561
ENSG00000156500	15.98640695	-0.366632485	0.437940852	-0.83717352	0.402495039	0.57074983
ENSG00000156502	230.2027632	-0.072974308	0.122578066	-0.59532925	0.551623408	0.699273893
ENSG00000156504	280.1741083	-0.044490288	0.123071329	-0.361500019	0.717725691	0.824088015
ENSG00000156508	5112.583413	0.021694395	0.066208535	0.327667651	0.743162965	0.842922019
ENSG00000156509	74.94389791	-0.595316779	0.209486387	-2.841792189	0.004486072	0.016329582
ENSG00000156515	963.2618447	0.210850201	0.071584265	2.945482503	0.003224514	0.012283054
ENSG00000156521	202.4009396	-0.157928806	0.141703477	-1.114501985	0.265063893	0.431693045
ENSG00000156531	290.9098636	-0.135641661	0.11205325	-1.210510724	0.226082978	0.384477917
ENSG00000156587	97.3412094	0.515316068	0.18484312	2.787856365	0.005305806	0.018804464
ENSG00000156599	627.9751415	0.02619203	0.085488494	0.30638076	0.759314756	0.854860493
ENSG00000156603	100.501246	0.177599887	0.182224519	0.97462124	0.329748174	0.502217902
ENSG00000156639	445.5234459	0.029715626	0.103532869	0.287016353	0.774099799	0.864738597
ENSG00000156642	466.0519445	0.33768747	0.092624263	3.645777695	0.000266584	0.001382322
ENSG00000156650	1304.061813	-0.257738475	0.070031955	-3.680298149	0.000232961	0.001225162
ENSG00000156671	333.1093348	-0.217254	0.104385354	-2.081268992	0.037409292	0.096455272
ENSG00000156675	373.3300921	-0.01051155	0.102928447	-0.102124825	0.918657596	0.955355281
ENSG00000156697	316.3310443	0.415960728	0.10902604	3.815242009	0.00013605	0.000759876

ENSG00000156709	608.7531821	-0.209959862	0.086247053	-2.434400419	0.014916484	0.04531398
ENSG00000156735	309.7765155	-0.16012068	0.110443699	-1.449794612	0.147115803	0.281126231
ENSG00000156787	130.9341569	-0.215245447	0.15747758	-1.366832327	0.171677868	0.314824216
ENSG00000156795	48.81556448	0.059367912	0.251894818	0.235685325	0.813676858	0.890577
ENSG00000156802	1372.932368	-0.046575388	0.065834991	-0.707456427	0.479282881	0.639970212
ENSG00000156831	108.5704268	-0.23738817	0.178236783	-1.331869698	0.18290301	0.329996851
ENSG00000156853	181.4250708	-0.149094374	0.144374969	-1.032688527	0.301749684	0.47217935
ENSG00000156858	604.7071313	0.395513443	0.088313609	4.47851069	7.52E-06	5.54E-05
ENSG00000156860	922.3328033	0.26287048	0.104373015	2.518567469	0.011783331	0.037070648
ENSG00000156869	94.08749577	-0.4054223	0.183375962	-2.210880285	0.027044128	0.074066849
ENSG00000156873	146.2429717	-0.14479533	0.154028625	-0.940054683	0.347189512	0.519047582
ENSG00000156875	58.46500841	0.019125433	0.236453043	0.080884699	0.935533648	0.964731953
ENSG00000156876	184.905512	-0.101501191	0.13687982	-0.741535099	0.458369058	0.622157712
ENSG00000156928	155.4865228	0.079076166	0.152612222	0.518150938	0.604352962	0.741751513
ENSG00000156931	95.24616054	-0.330041234	0.185846107	-1.775884564	0.075751946	0.168804037
ENSG00000156958	224.3202849	-0.719977485	0.133235303	-5.403804166	6.52E-08	7.09E-07
ENSG00000156970	537.5368863	0.11278253	0.088033976	1.281125028	0.200149754	0.352230468
ENSG00000156973	56.268814	-0.515660981	0.235884851	-2.186070779	0.028810427	0.077946856
ENSG00000156976	1166.57994	0.006779703	0.070361677	0.096355047	0.923238593	0.957667444
ENSG00000156983	574.669552	0.075054759	0.089799481	0.835803923	0.403265219	0.571553479
ENSG00000156990	351.9664085	0.174870064	0.109311467	1.599741265	0.109655993	0.225426275
ENSG00000157014	215.5866529	0.416505849	0.136556338	3.050066029	0.002287911	0.009171884
ENSG00000157020	745.2574234	0.149336632	0.083515939	1.78812133	0.073756435	0.16528155
ENSG00000157036	131.5061981	0.259191477	0.157084032	1.650017979	0.098939259	0.207960462
ENSG00000157045	96.60668355	0.034695791	0.178339091	0.194549557	0.845745596	0.910778012
ENSG00000157064	16.83135986	-1.838970145	0.475842516	-3.86466128	0.000111243	0.000634702
ENSG00000157077	219.7120247	0.119146227	0.135799998	0.877365455	0.38028817	0.549805262
ENSG00000157106	1058.253053	0.007873589	0.076171395	0.103366737	0.91767191	0.955355281
ENSG00000157107	141.9580252	0.140515683	0.150419616	0.934157969	0.350222413	0.522247796
ENSG00000157110	140.4981636	-9.451583696	1.185001443	-7.976010287	1.51E-15	3.80E-14
ENSG00000157111	26.45248893	1.264628118	0.344605495	3.669785124	0.000242754	0.001271429
ENSG00000157152	170.3707053	0.37559553	0.144893187	2.592223542	0.009535779	0.031013912
ENSG00000157181	278.7524277	0.049216913	0.11424935	0.430785056	0.666624667	0.786764972
ENSG00000157184	271.6335525	-0.198597033	0.114082117	-1.740825272	0.081714211	0.179686837
ENSG00000157191	108.0232687	0.036751552	0.177883125	0.206605048	0.836318318	0.904331203
ENSG00000157193	486.189678	0.35235409	0.094766938	3.718112027	0.000200717	0.001074086
ENSG00000157212	522.3782474	0.478878313	0.088268399	5.425252078	5.79E-08	6.34E-07
ENSG00000157214	177.8997588	0.150520704	0.146938493	1.024378982	0.305656305	0.47668118
ENSG00000157216	464.6340571	-0.028401035	0.092896637	-0.305727266	0.759812311	0.855077995
ENSG00000157224	164.948932	-0.174116757	0.156013094	-1.116039383	0.264405277	0.43107021
ENSG00000157240	120.8075097	1.009208771	0.171633224	5.880031539	4.10E-09	5.27E-08
ENSG00000157259	333.0664171	0.114920412	0.107705729	1.066985137	0.285978548	0.455664775
ENSG00000157306	20.98474873	1.265087418	0.386166035	3.276019388	0.001052813	0.004708331
ENSG00000157326	135.4667423	0.111445644	0.153916733	0.724064511	0.469026131	0.631013319
ENSG00000157343	14.59136847	-0.084490729	0.455326264	-0.18556085	0.852789157	0.915625567
ENSG00000157349	44.50860685	0.092595932	0.26046669	0.355500089	0.722214978	0.827735087
ENSG00000157350	256.9816981	-0.118987991	0.117901309	-1.009216875	0.312870635	0.484076461
ENSG00000157353	57.46384328	-0.188445045	0.231389402	-0.814406553	0.415412086	0.583296038
ENSG00000157379	13.04851813	0.298850673	0.488333282	0.611980964	0.540550352	0.690408328
ENSG00000157426	83.44052267	-0.209724948	0.198229675	-1.057989666	0.290060154	0.460340637

ENSG00000157429	10.92784568	-0.371540424	0.514776294	-0.721751232	0.470447438	0.632319889
ENSG00000157450	404.9368909	0.193056887	0.107939434	1.788566787	0.073684611	0.16521113
ENSG00000157456	334.956363	-0.101488576	0.107331625	-0.945560786	0.344372653	0.516209867
ENSG00000157483	157.3359976	-0.241115621	0.14785441	-1.630763809	0.102940167	0.214551686
ENSG00000157500	811.2665905	0.200147335	0.076120568	2.629346308	0.008554919	0.028342205
ENSG00000157502	34.60082088	0.801630986	0.308439153	2.598992306	0.009349787	0.030514976
ENSG00000157514	98.34501492	-0.315170704	0.179651639	-1.754343609	0.079371648	0.175414338
ENSG00000157538	318.9636063	0.025647988	0.109326607	0.234599686	0.814519453	0.891086819
ENSG00000157540	830.880423	0.071827906	0.082846175	0.867003289	0.385940207	0.554779174
ENSG00000157557	13.42972995	-0.3884539	0.493499078	-0.787142098	0.431198688	0.597556742
ENSG00000157570	9.600004386	-3.34419176	0.794095977	-4.211319357	2.54E-05	0.000167197
ENSG00000157578	16.93977955	-1.091893115	0.438892231	-2.487838788	0.012852198	0.039932497
ENSG00000157593	326.0903838	0.201783332	0.112419885	1.794907836	0.072668385	0.163532972
ENSG00000157600	142.2223811	0.071924566	0.158152715	0.454779202	0.649268094	0.774150659
ENSG00000157613	231.8098018	0.129360855	0.130247107	0.993195608	0.320614638	0.492258588
ENSG00000157617	510.5268727	0.554121177	0.090200325	6.143228162	8.09E-10	1.14E-08
ENSG00000157625	191.7960491	-0.065790617	0.145184632	-0.453151386	0.650439735	0.775087119
ENSG00000157637	567.1652968	-0.077158838	0.100533665	-0.767492538	0.442788728	0.607763657
ENSG00000157653	9.177783899	0.49983478	0.564339805	0.885698254	0.37578015	0.545716162
ENSG00000157657	514.8618703	-0.212130475	0.09189729	-2.30834308	0.020980062	0.060070716
ENSG00000157693	184.432852	-0.371161712	0.142746887	-2.600138752	0.009318607	0.030441507
ENSG00000157734	38.73180444	0.16376561	0.275655562	0.594095069	0.552448529	0.699814635
ENSG00000157741	875.1520401	1.33783593	0.083799831	15.96466139	2.25E-57	2.80E-55
ENSG00000157764	728.5446225	0.303273725	0.081630581	3.715197414	0.000203045	0.00108489
ENSG00000157778	341.9929248	0.361841951	0.118941706	3.042178923	0.002348722	0.009367548
ENSG00000157782	23.46867559	-2.098027474	0.414972836	-5.055818815	4.29E-07	4.09E-06
ENSG00000157796	116.7942276	0.069672938	0.175364968	0.397302487	0.691144414	0.805563918
ENSG00000157800	383.6490937	0.834548766	0.104895194	7.95602482	1.78E-15	4.44E-14
ENSG00000157823	174.9001436	0.096883176	0.142642772	0.67920144	0.497010235	0.655435794
ENSG00000157827	463.3831731	0.164572698	0.098012482	1.679099381	0.093132677	0.198548295
ENSG00000157833	234.6882492	-0.005209092	0.130148581	-0.040024191	0.96807384	0.983121257
ENSG00000157837	281.4985709	0.186027397	0.117159626	1.587811464	0.112328978	0.229711044
ENSG00000157869	127.5063854	1.215495674	0.165186825	7.358308836	1.86E-13	4.00E-12
ENSG00000157870	79.01777201	-0.344212936	0.205445703	-1.675444804	0.093846992	0.199616702
ENSG00000157873	21.92526974	0.782428995	0.407844457	1.918449502	0.055054039	0.131610635
ENSG00000157881	238.2548581	-0.0053567	0.124873312	-0.042897074	0.965783581	0.982286725
ENSG00000157890	13.3383716	0.433274892	0.483317653	0.896459894	0.370007191	0.541314309
ENSG00000157895	122.8332135	-0.001709587	0.167791059	-0.010188786	0.991870665	0.9958407
ENSG00000157911	181.3182315	-0.101974119	0.137854573	-0.739722424	0.459468439	0.622687232
ENSG00000157916	453.6626582	0.221790143	0.096941011	2.287887647	0.022144065	0.06262486
ENSG00000157927	83.21654599	-0.796480284	0.199147785	-3.999443351	6.35E-05	0.00038212
ENSG00000157933	647.349492	0.1228108	0.086642119	1.417449186	0.156351641	0.294012249
ENSG00000157954	312.0934054	0.080090352	0.110642753	0.723864418	0.469148977	0.631118145
ENSG00000157978	94.61922137	-0.069957075	0.196390041	-0.356214982	0.721679573	0.82741046
ENSG00000157985	601.731489	-0.083727827	0.08619882	-0.971333785	0.33138209	0.503834204
ENSG00000158006	87.33070517	0.118304059	0.190059365	0.622458457	0.533640452	0.68507105
ENSG00000158008	24.29514813	-1.327044648	0.377612148	-3.514306032	0.000440905	0.002167035
ENSG00000158019	188.9584035	0.176685516	0.138411746	1.276521111	0.201771369	0.354241023
ENSG00000158023	35.35572111	0.32842754	0.308367916	1.065050945	0.286852876	0.456643513
ENSG00000158042	376.3459738	0.564648255	0.103780616	5.440787275	5.30E-08	5.85E-07

ENSG00000158050	478.6287742	2.057122678	0.102255048	20.11756592	5.18E-90	1.15E-87
ENSG00000158062	96.17504516	-0.556130311	0.185358297	-3.000298989	0.002697147	0.01053365
ENSG00000158077	12.53272208	-0.947609274	0.506229162	-1.871897837	0.061220739	0.143160932
ENSG00000158079	162.8564686	-0.818694596	0.156846874	-5.21970616	1.79E-07	1.81E-06
ENSG00000158089	296.2167624	2.899379533	0.135237093	21.43923285	5.76E-102	1.55E-99
ENSG00000158092	159.1831322	0.206057705	0.142368214	1.447357516	0.147796818	0.281934703
ENSG00000158106	196.6073268	0.277790719	0.143584957	1.934678432	0.053029769	0.127618496
ENSG00000158109	49.66871106	0.42447467	0.246272265	1.723599165	0.084780229	0.184722799
ENSG00000158122	119.1165368	0.605369174	0.165993916	3.646935913	0.000265386	0.001377125
ENSG00000158158	338.0764022	0.207933533	0.105886008	1.963748907	0.049559218	0.121028918
ENSG00000158161	222.8436597	0.00583303	0.128345455	0.045447892	0.963750308	0.981000042
ENSG00000158163	148.4737505	-0.371584049	0.166516493	-2.23151498	0.025647036	0.070820507
ENSG00000158164	14.1997563	-0.791448456	0.473284868	-1.672245426	0.094475936	0.200681004
ENSG00000158169	209.8318654	-1.134824534	0.134991174	-8.40665726	4.22E-17	1.17E-15
ENSG00000158186	400.7960216	0.075126706	0.10212704	0.735620131	0.461961908	0.624920929
ENSG00000158195	1254.73618	0.312221041	0.069524388	4.490813255	7.10E-06	5.26E-05
ENSG00000158201	79.76540273	-0.245500258	0.197541041	-1.242781029	0.213948535	0.369568996
ENSG00000158234	88.15750341	0.056709687	0.190009952	0.298456402	0.765354846	0.858571124
ENSG00000158246	12.56010305	0.311254309	0.507048616	0.613854963	0.539311174	0.689580557
ENSG00000158270	6.62525523	-6.00868758	1.295354237	-4.638644326	3.51E-06	2.78E-05
ENSG00000158286	368.2047998	0.276132904	0.106376958	2.595796221	0.009437202	0.030728876
ENSG00000158290	557.6640364	0.304449137	0.093769517	3.246781538	0.001167179	0.005136394
ENSG00000158292	376.2052705	0.161716417	0.102139014	1.583297217	0.113353737	0.231301561
ENSG00000158296	34.83931426	-1.251432644	0.328539023	-3.80908372	0.000139483	0.000776581
ENSG00000158301	71.58759176	-0.406592777	0.218507424	-1.860773287	0.062776199	0.145931074
ENSG00000158321	11.40855184	1.182652773	0.526468183	2.246389833	0.024679049	0.068538276
ENSG00000158373	776.5386969	-0.642034189	0.168870475	-3.801932744	0.000143572	0.000797766
ENSG00000158402	101.6243799	-0.54671154	0.182714397	-2.992164537	0.002770069	0.010781702
ENSG00000158406	560.6459742	-0.215317563	0.107789259	-1.997579024	0.045762319	0.113794176
ENSG00000158411	169.749672	0.186128928	0.142565864	1.305564477	0.191700707	0.341945376
ENSG00000158417	3722.197207	0.324886409	0.057713618	5.629285114	1.81E-08	2.14E-07
ENSG00000158423	27.95666445	-0.174392448	0.335358772	-0.520017554	0.60305134	0.741060465
ENSG00000158435	279.7294867	-0.000245792	0.121263032	-0.002026935	0.998382741	0.999236364
ENSG00000158457	52.17186021	0.505999101	0.244688147	2.067934661	0.038646168	0.09908062
ENSG00000158458	108.4332887	0.611756389	0.173749525	3.520909706	0.000430069	0.002118226
ENSG00000158467	204.388198	0.436587275	0.136382375	3.20120011	0.001368565	0.005906171
ENSG00000158470	712.0902099	-0.18485161	0.087464897	-2.113437681	0.034563324	0.090479129
ENSG00000158480	136.9806107	0.125829699	0.155423188	0.809594123	0.418173487	0.586118836
ENSG00000158526	195.6717306	0.077998724	0.129702518	0.601366304	0.547596036	0.695834778
ENSG00000158528	327.4302702	0.531318279	0.114547328	4.638417046	3.51E-06	2.78E-05
ENSG00000158545	649.6916594	-0.656318506	0.08331327	-7.877718684	3.33E-15	8.13E-14
ENSG00000158552	60.33613548	0.370222715	0.227721722	1.625768117	0.103998993	0.216073637
ENSG00000158604	405.2462844	0.083275374	0.099071921	0.840554754	0.400597415	0.568747917
ENSG00000158615	1643.615563	0.00456455	0.07051607	0.064730635	0.948388471	0.971358017
ENSG00000158623	140.6215849	0.484823367	0.169855557	2.854327382	0.004312806	0.015788895
ENSG00000158636	350.500741	-0.111433195	0.104004359	-1.071428122	0.283976974	0.453633078
ENSG00000158669	349.6104978	-0.231683258	0.110622369	-2.094361751	0.036227767	0.09409975
ENSG00000158710	180.2747505	-0.382376462	0.137290424	-2.78516485	0.005350052	0.018934788
ENSG00000158711	1631.806112	0.251953529	0.073360549	3.434455338	0.000593746	0.002826279
ENSG00000158715	46.41679691	1.007357225	0.261961983	3.845432896	0.00012034	0.000681069

ENSG00000158717	69.75545924	-0.632214239	0.21673266	-2.917023398	0.003533893	0.013319182
ENSG00000158747	158.0093903	0.112526757	0.14741467	0.763334865	0.445263714	0.610325858
ENSG00000158748	13.52945478	-1.370973008	0.517294779	-2.650274204	0.008042647	0.026870075
ENSG00000158769	24.39805547	-0.533522929	0.35524096	-1.501862084	0.13313273	0.261408368
ENSG00000158773	183.9622736	-0.232398354	0.138295193	-1.680451424	0.092869518	0.19819783
ENSG00000158792	195.8175408	0.108963839	0.15316059	0.711435228	0.476814575	0.63753861
ENSG00000158793	113.4159511	-0.312120547	0.171836206	-1.816384066	0.069311478	0.157798757
ENSG00000158796	430.1996311	0.160120775	0.103479507	1.54736701	0.121774772	0.244751784
ENSG00000158805	386.028486	-0.077325027	0.108239724	-0.714386774	0.474988048	0.635988668
ENSG00000158813	19.71751748	-0.529398179	0.418402025	-1.265285892	0.205768862	0.359372772
ENSG00000158828	127.8465339	-0.441732946	0.158785673	-2.781944604	0.005403426	0.019090022
ENSG00000158850	382.3751682	0.636476097	0.103685471	6.138527299	8.33E-10	1.17E-08
ENSG00000158856	26.58098429	-0.693480531	0.342365185	-2.025557974	0.042810102	0.108021107
ENSG00000158863	196.3300161	-0.01151126	0.131313268	-0.087662584	0.930144858	0.962131431
ENSG00000158864	875.0861676	0.112217254	0.077590572	1.446274358	0.148100265	0.28231299
ENSG00000158882	283.8594063	0.000292037	0.116249127	0.002512165	0.997995584	0.999084016
ENSG00000158941	693.807195	-0.039565488	0.081984651	-0.482596279	0.629382422	0.759693543
ENSG00000158966	230.9334182	0.259454685	0.123225764	2.105523041	0.035245804	0.091923098
ENSG00000158985	148.4617011	-0.382232703	0.164712502	-2.320605285	0.020308157	0.058528658
ENSG00000158987	128.3121842	-0.052164828	0.156926281	-0.332416135	0.739575057	0.840729783
ENSG00000159023	586.0239213	-0.5401069	0.092414999	-5.84436408	5.09E-09	6.49E-08
ENSG00000159055	99.20645973	0.03081141	0.197989197	0.155621673	0.876331241	0.931503968
ENSG00000159063	197.6855113	-0.088191045	0.1357283	-0.649761655	0.515846191	0.671096242
ENSG00000159069	441.7947898	0.512349431	0.102292908	5.008650566	5.48E-07	5.15E-06
ENSG00000159079	44.89168559	0.135223288	0.260864804	0.518365399	0.604203352	0.741697499
ENSG00000159082	137.8298539	0.287333525	0.168090545	1.709397305	0.08737738	0.188858294
ENSG00000159086	392.9018466	-0.066860484	0.103532374	-0.645793013	0.518413414	0.673338255
ENSG00000159110	108.4381572	0.16294205	0.172064	0.946985135	0.343646354	0.515506229
ENSG00000159111	361.9872459	0.220572914	0.116992835	1.885354042	0.059382054	0.139604974
ENSG00000159128	229.9605482	0.139245541	0.124135868	1.121718838	0.261982004	0.428312525
ENSG00000159131	1521.956218	-0.021098747	0.08440856	-0.249959797	0.802618439	0.88267292
ENSG00000159140	3200.596651	-0.046261	0.061472379	-0.752549366	0.451720754	0.616629899
ENSG00000159147	168.5002183	0.017605023	0.14570382	0.120827462	0.903827698	0.948177575
ENSG00000159164	301.8513482	0.104895096	0.112123563	0.935531236	0.349514593	0.521524484
ENSG00000159176	9.487662706	-0.945481681	0.579577015	-1.631330534	0.102820594	0.214354539
ENSG00000159182	35.08893554	0.19487126	0.304318567	0.640352843	0.521943233	0.676047635
ENSG00000159184	465.8354229	-0.641778144	0.097034205	-6.613937275	3.74E-11	6.24E-10
ENSG00000159199	816.0130808	0.365199524	0.086262809	4.233568628	2.30E-05	0.000152502
ENSG00000159200	34.68164859	0.084231464	0.304484595	0.27663621	0.782059458	0.869594649
ENSG00000159202	1139.0576	0.013069864	0.069452993	0.18818287	0.8507333	0.914466687
ENSG00000159208	141.0551941	0.375038749	0.163194364	2.29811092	0.021555477	0.061380455
ENSG00000159210	539.9171705	0.230045409	0.091663124	2.50968328	0.012083949	0.037889114
ENSG00000159217	3186.449935	-0.199279296	0.056003217	-3.558354441	0.000373186	0.001870188
ENSG00000159231	9.644724011	0.480754485	0.581393619	0.826900174	0.408293645	0.576817945
ENSG00000159256	215.1767721	-0.082626409	0.131461541	-0.62852153	0.529662347	0.682186526
ENSG00000159259	243.7952259	-0.286606054	0.130643208	-2.193807535	0.028249249	0.07672413
ENSG00000159267	220.5069791	-0.498571818	0.133191996	-3.743256586	0.000181651	0.000982914
ENSG00000159307	12.60391901	0.532802857	0.487978769	1.091856636	0.27489612	0.443286167
ENSG00000159314	174.622873	0.086542711	0.144322224	0.599649238	0.548740025	0.697002544
ENSG00000159322	362.9410562	0.325176721	0.106745526	3.04627961	0.002316923	0.009259122

ENSG00000159335	2595.373071	0.133508951	0.143530564	0.930177848	0.352279009	0.523715899
ENSG00000159346	1240.635907	0.018871434	0.076706835	0.246020235	0.805666569	0.885189466
ENSG00000159348	67.82841656	0.954569909	0.214773535	4.444541598	8.81E-06	6.39E-05
ENSG00000159352	1153.583728	0.440605229	0.08610598	5.117010769	3.10E-07	3.02E-06
ENSG00000159363	185.3185848	-0.061830306	0.14944147	-0.413742627	0.679062605	0.796492352
ENSG00000159377	1226.095983	0.534100758	0.077165667	6.921481752	4.47E-12	8.24E-11
ENSG00000159388	240.4963724	-0.207678136	0.128963726	-1.610360861	0.1073191	0.221758075
ENSG00000159399	410.9129227	0.425338272	0.096386902	4.412822298	1.02E-05	7.31E-05
ENSG00000159403	36.3841266	0.556516944	0.304195239	1.829472891	0.067328797	0.154360635
ENSG00000159423	47.94507971	-0.683150076	0.259062653	-2.637007185	0.008364106	0.027808425
ENSG00000159433	314.0272782	-0.33485224	0.127611537	-2.623996604	0.008690465	0.028667817
ENSG00000159459	317.0857105	0.046087132	0.112385076	0.410082317	0.681745564	0.79850579
ENSG00000159461	393.1325314	0.244948045	0.102887937	2.380726563	0.017278532	0.051280698
ENSG00000159479	170.5176862	0.176718133	0.147579037	1.19744739	0.231132222	0.390558682
ENSG00000159556	171.6728791	0.595068075	0.137855375	4.31661134	1.58E-05	0.000108674
ENSG00000159579	226.5004839	-0.188449471	0.122718333	-1.535626065	0.124630118	0.248869878
ENSG00000159588	25.55895838	-0.081083458	0.351270043	-0.230829412	0.81744733	0.892507031
ENSG00000159592	554.8200888	-0.014671729	0.097561379	-0.150384595	0.880461195	0.934116144
ENSG00000159593	365.7400628	0.041909483	0.1078199	0.388698959	0.697498853	0.810201471
ENSG00000159596	320.607695	0.047485266	0.115466505	0.411247104	0.680891352	0.797904464
ENSG00000159640	126.2667212	0.573093578	0.161655757	3.545147957	0.000392393	0.001952402
ENSG00000159658	494.8621198	-0.262451797	0.100316236	-2.61624447	0.008890288	0.029224957
ENSG00000159674	8.595464721	-0.026905361	0.595282287	-0.045197651	0.963949766	0.981105184
ENSG00000159685	156.4437208	-0.06505	0.153095483	-0.42489823	0.670910865	0.79003143
ENSG00000159692	735.3026755	-0.041926832	0.080440357	-0.521216384	0.602216042	0.740357846
ENSG00000159714	62.14486896	-0.147989935	0.225802462	-0.655395575	0.512213099	0.668188838
ENSG00000159720	288.5537395	0.210836119	0.113000119	1.865804388	0.062068738	0.144686287
ENSG00000159733	28.73079086	0.088798163	0.32483518	0.273363749	0.784573616	0.871011269
ENSG00000159753	59.75435141	-0.949285333	0.250805696	-3.784943277	0.000153744	0.000847249
ENSG00000159761	12.65657004	0.105740254	0.527547339	0.20043747	0.841138457	0.907697202
ENSG00000159788	314.3682253	0.303039181	0.11771864	2.57426675	0.010045279	0.032393489
ENSG00000159792	130.1708059	-0.748246479	0.167110449	-4.477556507	7.55E-06	5.56E-05
ENSG00000159840	833.2378688	0.387306851	0.07837497	4.941716081	7.74E-07	7.04E-06
ENSG00000159842	773.0533325	0.117916765	0.079175535	1.489308087	0.136406259	0.265918808
ENSG00000159873	325.0616409	0.150012566	0.10684249	1.404053448	0.160302934	0.299330957
ENSG00000159882	17.84706016	0.417129022	0.413267646	1.009343524	0.312809913	0.484076461
ENSG00000159884	64.24468477	-0.13327717	0.229471327	-0.580800975	0.561374598	0.707739093
ENSG00000159899	49.98102737	-0.215252788	0.249252492	-0.863593323	0.387811333	0.556525506
ENSG00000159905	32.53445087	0.822720683	0.332647575	2.473250201	0.013389037	0.041380814
ENSG00000159915	10.93966804	-0.631661684	0.527483852	-1.197499567	0.231111896	0.390558682
ENSG00000159917	56.54383144	0.139393099	0.240955114	0.578502349	0.562925012	0.709057357
ENSG00000159921	539.3966085	-0.091713613	0.096214777	-0.953217534	0.340479864	0.512233121
ENSG00000159958	69.60992888	0.398773216	0.220882123	1.805366637	0.071017328	0.16084818
ENSG00000160007	1078.282877	-0.232857801	0.071915028	-3.237957454	0.001203888	0.005270379
ENSG00000160014	2020.028826	-0.281602132	0.062159418	-4.530321274	5.89E-06	4.45E-05
ENSG00000160049	1037.650094	-0.062980165	0.075441774	-0.834818184	0.403820086	0.571993621
ENSG00000160050	243.6885773	-0.34471634	0.123861871	-2.783070674	0.005384708	0.019038255
ENSG00000160051	31.24668516	-0.120330403	0.315332739	-0.381598191	0.702759426	0.814023391
ENSG00000160055	36.47947462	-0.38506375	0.297584042	-1.293966396	0.195677028	0.346923541
ENSG00000160058	259.4872889	0.130801392	0.122227445	1.070147479	0.284552931	0.454166006

ENSG00000160062	80.48331931	0.004600198	0.204873801	0.022453815	0.982085953	0.990903705
ENSG00000160072	258.1375986	0.637809649	0.125913941	5.065441067	4.07E-07	3.91E-06
ENSG00000160075	534.293498	-0.096555477	0.09244554	-1.044457918	0.296273579	0.466640787
ENSG00000160087	235.1209198	0.379530162	0.137946428	2.75128663	0.005936168	0.020675762
ENSG00000160094	360.9262746	-0.535728022	0.1070773	-5.003189501	5.64E-07	5.27E-06
ENSG00000160097	38.17372562	-1.358152214	0.31636449	-4.292998281	1.76E-05	0.000119795
ENSG00000160113	1048.493523	0.0334558	0.076059826	0.439861642	0.660037319	0.782606924
ENSG00000160117	215.5596985	0.07399652	0.124390187	0.594874256	0.551927527	0.699430612
ENSG00000160124	254.0710027	0.409819971	0.123765537	3.311260803	0.000928766	0.004209867
ENSG00000160131	401.178373	-0.292320874	0.102608861	-2.848885301	0.004387269	0.016028081
ENSG00000160161	288.0638906	0.068642491	0.122145293	0.561974098	0.574133667	0.718419883
ENSG00000160179	132.1372467	0.745894291	0.159540536	4.675265055	2.94E-06	2.36E-05
ENSG00000160188	21.98552515	-0.228229109	0.368354785	-0.619590456	0.535527452	0.686364427
ENSG00000160190	50.43312609	-0.753445682	0.255576809	-2.94802054	0.003198158	0.012201121
ENSG00000160191	7.806189479	-3.306254076	0.875756506	-3.775312032	0.000159807	0.000877224
ENSG00000160193	230.9941038	0.295092383	0.124617065	2.36799336	0.017884857	0.052690559
ENSG00000160194	793.245318	0.072314352	0.07959084	0.908576307	0.363573818	0.534581748
ENSG00000160199	114.7563025	0.134852719	0.168442134	0.800587807	0.423370312	0.590574257
ENSG00000160200	27.98446057	-0.133146322	0.332503482	-0.400435872	0.688835508	0.804194845
ENSG00000160201	40.22233647	-0.156589234	0.280319334	-0.558610181	0.57642779	0.720573211
ENSG00000160207	7.869197413	0.368712016	0.625395454	0.589566192	0.555481523	0.702516337
ENSG00000160208	1228.815245	0.112130381	0.0677883	1.654125882	0.098101938	0.206633367
ENSG00000160209	530.0076409	0.354111357	0.094876791	3.732328564	0.000189718	0.001021451
ENSG00000160211	647.5342451	0.284478434	0.08248048	3.4490395	0.000562584	0.002687622
ENSG00000160213	1686.321059	0.581492565	0.064580278	9.004181814	2.17E-19	7.13E-18
ENSG00000160214	471.7513676	0.352424599	0.09617734	3.664320501	0.000247996	0.001296465
ENSG00000160216	364.2901847	0.503198327	0.102125929	4.927233782	8.34E-07	7.52E-06
ENSG00000160218	219.5871198	-0.058483237	0.135394842	-0.431945824	0.665780789	0.786165328
ENSG00000160221	36.95462801	-0.052995746	0.308660743	-0.171695775	0.863676707	0.922941553
ENSG00000160226	35.28342534	-0.000337855	0.314270596	-0.001075045	0.999142238	0.999711591
ENSG00000160233	135.7683535	0.489485587	0.165220226	2.962625091	0.003050278	0.011716506
ENSG00000160256	214.3760924	0.675874341	0.137305132	4.922425907	8.55E-07	7.68E-06
ENSG00000160271	70.370763	0.359616826	0.211912801	1.697003794	0.089695943	0.192820763
ENSG00000160285	507.0524764	-0.431697459	0.09067282	-4.761045935	1.93E-06	1.61E-05
ENSG00000160293	209.2937076	0.235879434	0.131458908	1.794320662	0.072762002	0.163664987
ENSG00000160294	867.0375925	-0.104922639	0.076134494	-1.378122238	0.168165551	0.310450978
ENSG00000160298	94.43127437	-0.400941184	0.192373483	-2.084181132	0.037143694	0.095946575
ENSG00000160299	1094.070664	0.053496548	0.074852858	0.714689455	0.474800955	0.63590048
ENSG00000160305	347.5620441	0.28946205	0.109965561	2.632297315	0.008480961	0.028137
ENSG00000160310	180.245565	0.205490783	0.134283201	1.530279154	0.125947646	0.250907557
ENSG00000160318	17.57101808	0.033245562	0.419598271	0.079231886	0.936848183	0.965368723
ENSG00000160323	46.2060633	-0.096955062	0.258018932	-0.375767239	0.707089945	0.816145642
ENSG00000160325	9.267973741	-1.019639154	0.605038264	-1.685247389	0.091940852	0.196574299
ENSG00000160326	75.02298991	0.187523733	0.215463658	0.870326509	0.384121996	0.553241226
ENSG00000160336	106.381381	0.301161596	0.172006973	1.750868526	0.079968559	0.176455915
ENSG00000160345	31.43883392	0.24479451	0.311061111	0.786965973	0.431301785	0.597602701
ENSG00000160360	439.3403618	0.419158035	0.10112401	4.14499023	3.40E-05	0.000217475
ENSG00000160392	75.15481845	0.098961971	0.219080762	0.451714566	0.651474624	0.776056991
ENSG00000160404	89.06919406	0.421487112	0.200917842	2.097808278	0.035922085	0.093461294
ENSG00000160408	13.28130668	0.759431421	0.483851966	1.569553239	0.116519085	0.235990463

ENSG00000160410	526.2646071	-0.139351081	0.089578536	-1.555630261	0.119796039	0.241543861
ENSG00000160439	224.7208701	0.093859188	0.126556165	0.741640585	0.458305127	0.622131051
ENSG00000160445	283.2215103	-0.17384814	0.114103349	-1.523602435	0.127608055	0.252943916
ENSG00000160446	107.6771815	-0.215802868	0.189587907	-1.138273382	0.255006345	0.420283249
ENSG00000160447	243.2223904	0.145640966	0.124237751	1.17227626	0.24108616	0.403558929
ENSG00000160460	259.4776387	0.477305473	0.117855091	4.049935127	5.12E-05	0.000314396
ENSG00000160469	137.7985575	-0.187965811	0.161384962	-1.16470462	0.244138578	0.407293897
ENSG00000160539	114.0956399	-0.192377256	0.174019793	-1.105490661	0.268947039	0.436196635
ENSG00000160551	1463.286868	-0.227730789	0.067867871	-3.355502168	0.00079221	0.003644133
ENSG00000160563	68.8520867	0.173222035	0.210940824	0.821187816	0.411539295	0.579886898
ENSG00000160570	136.6871323	-0.008829059	0.155838146	-0.056655315	0.954819771	0.975656748
ENSG00000160584	603.9374173	-0.145948241	0.088885876	-1.641973367	0.100595524	0.210590957
ENSG00000160602	33.89839514	-0.252592761	0.301138764	-0.838791917	0.401586087	0.569921173
ENSG00000160606	110.6680213	-0.282613629	0.179213041	-1.576970223	0.114802377	0.233511293
ENSG00000160613	371.8303289	0.339676721	0.106638275	3.185317088	0.001445955	0.006192478
ENSG00000160633	1433.994602	0.185191242	0.071966009	2.573315433	0.010072936	0.032475219
ENSG00000160678	124.212649	-1.320923169	0.173837454	-7.598610879	2.99E-14	6.87E-13
ENSG00000160679	830.5752933	0.004883383	0.087341074	0.055911649	0.955412191	0.976091797
ENSG00000160685	351.9107709	0.177862661	0.118666379	1.498846277	0.133913516	0.262427896
ENSG00000160688	436.939407	0.032808845	0.098888214	0.331777105	0.740057573	0.840999008
ENSG00000160691	1889.647315	-0.075196942	0.06252895	-1.202594013	0.229133465	0.387901845
ENSG00000160695	269.1427456	-0.02689018	0.117585638	-0.228685919	0.81911304	0.893395096
ENSG00000160703	48.03414216	-0.370125541	0.256035062	-1.445604902	0.148288051	0.282483689
ENSG00000160710	3136.933881	0.196100145	0.059913319	3.273064292	0.001063883	0.004750274
ENSG00000160712	17.3699051	0.971051699	0.429271086	2.262094352	0.023691575	0.066254339
ENSG00000160714	769.4853736	0.086514948	0.083895126	1.031227339	0.302434223	0.473083912
ENSG00000160741	638.5615383	0.19331047	0.085661544	2.256677394	0.02402824	0.067062327
ENSG00000160746	129.0325632	-0.219257664	0.160322444	-1.3676043	0.17143597	0.314504514
ENSG00000160752	1764.090943	0.134770191	0.067521244	1.99596724	0.045937489	0.114048057
ENSG00000160753	275.1649288	-0.131929552	0.115949459	-1.137819474	0.255195871	0.42044762
ENSG00000160767	944.0307698	0.357295876	0.183561341	1.946465815	0.05159881	0.124854611
ENSG00000160781	82.93480248	-0.197101327	0.215602619	-0.914188	0.360618068	0.531933425
ENSG00000160783	223.0848335	-0.214922185	0.12957529	-1.658666443	0.097183025	0.204955405
ENSG00000160785	551.0173251	0.553636331	0.088806588	6.234180866	4.54E-10	6.62E-09
ENSG00000160789	883.9027101	0.367279627	0.084729246	4.33474443	1.46E-05	0.000100979
ENSG00000160791	33.96399742	0.101372542	0.297428986	0.340829399	0.733232017	0.835693316
ENSG00000160796	765.7825744	-0.070547852	0.081923006	-0.861148231	0.389156408	0.557687326
ENSG00000160799	527.1387098	0.19798817	0.087795347	2.25511006	0.024126421	0.06726952
ENSG00000160801	39.16638568	-1.540122334	0.306810979	-5.019775826	5.17E-07	4.88E-06
ENSG00000160803	724.4820169	0.189990254	0.085073868	2.233238695	0.025533208	0.070572793
ENSG00000160813	51.36783507	-0.316226486	0.262412003	-1.205076298	0.228173849	0.386956182
ENSG00000160818	1177.685835	-0.068002537	0.075935299	-0.895532617	0.370502438	0.541536387
ENSG00000160867	87.85869012	-0.070539369	0.199400073	-0.353757989	0.72352026	0.828641589
ENSG00000160877	1022.882287	-0.055242193	0.078998113	-0.699284966	0.484373961	0.644880533
ENSG00000160888	420.2911296	-0.105608072	0.114368353	-0.923402929	0.355797266	0.527227053
ENSG00000160908	160.3271472	0.264058874	0.147462631	1.790683318	0.073344128	0.164605363
ENSG00000160917	214.0480431	-0.000963468	0.137187833	-0.007022985	0.994396514	0.997521091
ENSG00000160932	1675.389027	1.188574359	0.068802152	17.27524964	7.23E-67	1.14E-64
ENSG00000160948	468.5471319	0.721039747	0.103196596	6.987049704	2.81E-12	5.29E-11
ENSG00000160949	348.9863147	0.127400555	0.116170307	1.096670558	0.272785431	0.440844104

ENSG00000160951	14.73672888	-1.245027178	0.475348388	-2.619188808	0.008813915	0.029022284
ENSG00000160953	478.7631684	0.433952656	0.091665216	4.734103923	2.20E-06	1.82E-05
ENSG00000160957	380.0407491	0.556820404	0.105728128	5.266530427	1.39E-07	1.43E-06
ENSG00000160959	106.1223267	0.365551613	0.180327212	2.027157245	0.042646334	0.107701599
ENSG00000160961	116.0032724	0.55674951	0.170763279	3.260358511	0.001112715	0.004936925
ENSG00000160963	103.2865831	-4.119381075	0.312922778	-13.16420973	1.41E-39	1.08E-37
ENSG00000160972	453.6165701	0.411982477	0.094839123	4.344014011	1.40E-05	9.73E-05
ENSG00000160991	276.421708	-0.027041045	0.116787417	-0.231540742	0.816894738	0.89236491
ENSG00000160993	99.73884769	0.014682122	0.176987851	0.082955537	0.933886894	0.963943655
ENSG00000160999	62.74998505	0.639624772	0.223354157	2.863724499	0.00418692	0.015404312
ENSG00000161010	164.5968257	-0.340872738	0.151782786	-2.245793129	0.024717262	0.068617268
ENSG00000161011	1328.606357	0.433955382	0.067040602	6.473023316	9.61E-11	1.52E-09
ENSG00000161013	427.1649542	0.248566759	0.104298718	2.383219697	0.017161949	0.051042535
ENSG00000161016	16163.49516	0.370188868	0.054506458	6.791651472	1.11E-11	1.95E-10
ENSG00000161021	704.7534289	-0.192859616	0.081795186	-2.357835775	0.018381823	0.053894691
ENSG00000161036	227.9758225	0.120493747	0.12268446	0.982143517	0.326029149	0.498175945
ENSG00000161048	40.60509204	-0.291212682	0.272863941	-1.06724502	0.285861208	0.455529483
ENSG00000161057	537.1640098	1.045706516	0.094779178	11.03308274	2.65E-28	1.35E-26
ENSG00000161082	8.097311742	-1.95342872	0.70225807	-2.781639404	0.00540851	0.019098375
ENSG00000161091	359.483163	-0.085189774	0.106299737	-0.801410951	0.422893778	0.59010551
ENSG00000161179	188.5665071	0.373633922	0.166062393	2.249961087	0.024451416	0.06802454
ENSG00000161180	30.60738571	1.225894584	0.324120121	3.782223028	0.000155434	0.000855222
ENSG00000161202	702.2962168	-0.314924924	0.080187982	-3.927333207	8.59E-05	0.000502515
ENSG00000161203	757.1701032	-0.121122117	0.100029582	-1.210862969	0.225947927	0.384346812
ENSG00000161204	118.0832434	-0.112373441	0.174238661	-0.644939767	0.51896622	0.673862508
ENSG00000161217	270.7342727	0.066626914	0.13093763	0.508844663	0.61086111	0.746967107
ENSG00000161243	201.5381909	-1.571410219	0.139820575	-11.23876242	2.63E-29	1.39E-27
ENSG00000161249	183.9709404	-2.895538702	0.173456599	-16.69315967	1.47E-62	2.11E-60
ENSG00000161265	25.49858265	-0.129420219	0.357968714	-0.361540587	0.71769537	0.824088015
ENSG00000161277	50.89465114	0.018375857	0.24673742	0.074475355	0.94063215	0.967262249
ENSG00000161298	129.0178324	-0.251628996	0.160080261	-1.571892712	0.115975435	0.235114293
ENSG00000161328	83.46821523	-0.110257448	0.208678016	-0.52836159	0.597248392	0.736571392
ENSG00000161381	21.49494548	-3.115415434	0.516556084	-6.031127174	1.63E-09	2.21E-08
ENSG00000161395	30.88413745	0.14164639	0.314347863	0.450603956	0.652275015	0.776549465
ENSG00000161405	29.34281756	-0.044345839	0.323524503	-0.137071038	0.890974645	0.94027635
ENSG00000161509	28.82816594	-1.463097937	0.360043205	-4.063673237	4.83E-05	0.000298794
ENSG00000161513	212.5091049	-0.552586116	0.128877081	-4.287698896	1.81E-05	0.000122215
ENSG00000161526	509.5892969	0.017814739	0.093554286	0.190421413	0.848978919	0.913349688
ENSG00000161533	742.1103813	-0.511814229	0.079955832	-6.401211966	1.54E-10	2.38E-09
ENSG00000161542	373.7355144	0.156054806	0.104828327	1.488670191	0.136574238	0.266121799
ENSG00000161547	1389.198622	0.026680542	0.070379849	0.37909348	0.704618448	0.814832908
ENSG00000161558	91.30292298	-0.149283932	0.189535791	-0.787629245	0.430913603	0.597477385
ENSG00000161618	107.2413533	-0.162846845	0.173713587	-0.937444495	0.348529962	0.520494777
ENSG00000161638	435.3407223	0.086755815	0.096949012	0.894860231	0.370861808	0.541865594
ENSG00000161642	397.6081019	-0.342741384	0.098571729	-3.477075919	0.000506914	0.002451162
ENSG00000161647	38.45231648	0.228079478	0.292409897	0.779999171	0.435391363	0.601607562
ENSG00000161653	15.49734244	-1.099398989	0.454345909	-2.419740045	0.015531606	0.046914605
ENSG00000161654	211.9167578	0.169397547	0.137568718	1.231366766	0.218185721	0.375181151
ENSG00000161671	266.2222859	0.435643361	0.128687085	3.38529201	0.000711026	0.003317097
ENSG00000161677	63.31053671	0.923355121	0.239336913	3.857972046	0.000114332	0.000651

ENSG00000161681	140.343071	-0.147264867	0.155230111	-0.948687509	0.342779569	0.514590639
ENSG00000161682	30.43328477	0.252678827	0.317846175	0.794972056	0.426629757	0.593882489
ENSG00000161692	273.3539138	-0.070541406	0.113999062	-0.618789354	0.536055136	0.686852732
ENSG00000161714	92.59339135	-0.235272224	0.193141368	-1.218134814	0.223172746	0.381561291
ENSG00000161791	173.6574687	-0.005621581	0.138805578	-0.04049968	0.967694762	0.982949477
ENSG00000161800	443.1394821	0.08465844	0.097856897	0.865124919	0.386970229	0.555798629
ENSG00000161813	796.4341311	-0.186582646	0.077398412	-2.410677951	0.015922902	0.047925648
ENSG00000161835	50.60101014	1.203401182	0.252363405	4.76852491	1.86E-06	1.55E-05
ENSG00000161847	398.2686344	0.206967364	0.111286826	1.859765178	0.062918756	0.146182561
ENSG00000161888	183.7851403	-0.17132015	0.144921756	-1.182156182	0.237143735	0.39851155
ENSG00000161904	223.067539	0.05258236	0.124419161	0.422622684	0.672570574	0.791282788
ENSG00000161914	38.93100416	0.158817604	0.281933223	0.563316387	0.573219462	0.717878752
ENSG00000161920	64.59360232	-0.195715536	0.233725365	-0.837373967	0.402382394	0.570658715
ENSG00000161921	524.6348691	0.648354943	0.091618626	7.076671794	1.48E-12	2.89E-11
ENSG00000161940	25.82132296	1.552849032	0.373616807	4.156261186	3.23E-05	0.000207876
ENSG00000161956	60.19107659	0.369929963	0.235413279	1.571406524	0.116088251	0.235309043
ENSG00000161958	10.34724396	0.409741302	0.540211147	0.758483612	0.44816152	0.613280552
ENSG00000161960	112.3799232	0.619661926	0.174197677	3.557234142	0.00037478	0.001875503
ENSG00000161970	221.7993748	0.416385567	0.150277342	2.770780754	0.005592207	0.019672859
ENSG00000161980	126.1412232	0.12224515	0.162683676	0.751428493	0.45239482	0.617149659
ENSG00000161981	139.0211648	0.106531495	0.154428863	0.689841865	0.490293638	0.649854192
ENSG00000161996	18.31630742	-0.056900364	0.412222215	-0.138033232	0.890214154	0.939860069
ENSG00000161999	118.6848484	2.630490428	0.206504534	12.73817277	3.63E-37	2.55E-35
ENSG00000162004	30.57969326	-0.602493124	0.315603214	-1.909020877	0.056259399	0.133763672
ENSG00000162032	78.11823347	0.469314093	0.202076135	2.322461739	0.020208086	0.05826416
ENSG00000162039	52.69880842	0.11066615	0.245251685	0.451235023	0.65182017	0.776271126
ENSG00000162062	115.497645	0.21346283	0.166124259	1.284958811	0.198806678	0.350564914
ENSG00000162063	377.991931	-0.087190837	0.101186697	-0.861682805	0.38886209	0.557325353
ENSG00000162065	119.7986351	0.017370907	0.1634646	0.10626709	0.915370442	0.954297804
ENSG00000162066	53.06151784	0.613304649	0.270024365	2.271293736	0.023129202	0.064979181
ENSG00000162068	18.36605926	1.147670267	0.448981485	2.55616391	0.010583325	0.033802631
ENSG00000162073	193.3862417	-0.10551619	0.14251301	-0.740396896	0.459059202	0.62242959
ENSG00000162076	227.6995671	-0.429539112	0.137773975	-3.117708649	0.001822629	0.007547897
ENSG00000162086	71.52595104	-0.035578208	0.220896696	-0.161062654	0.872044054	0.928844619
ENSG00000162104	339.3957898	0.048898975	0.107569733	0.454579307	0.649411924	0.774190724
ENSG00000162129	615.9659678	0.501298592	0.088475193	5.665979059	1.46E-08	1.76E-07
ENSG00000162139	93.06606089	-0.545386373	0.188702554	-2.890190738	0.003850081	0.014315596
ENSG00000162144	54.65711623	0.32204738	0.254223219	1.266789798	0.205230461	0.358682061
ENSG00000162174	54.67863218	-1.559028399	0.258999789	-6.019419576	1.75E-09	2.37E-08
ENSG00000162191	725.7208299	-0.057105077	0.090343817	-0.632086174	0.527330569	0.680265635
ENSG00000162194	61.51450308	0.672014971	0.223278827	3.009756811	0.002614569	0.010270374
ENSG00000162222	162.4538487	0.018892287	0.142491739	0.132585136	0.894521488	0.942843412
ENSG00000162227	125.8413225	-0.06798194	0.176737233	-0.384649789	0.700496901	0.812005278
ENSG00000162231	402.9851479	0.301790094	0.108759517	2.774838502	0.005522913	0.019448572
ENSG00000162236	122.128827	-0.070199287	0.162197368	-0.432801642	0.665158881	0.785780162
ENSG00000162241	15.41440358	-0.655775794	0.474284754	-1.382662605	0.166768343	0.308601622
ENSG00000162244	3366.092478	0.264811436	0.074564435	3.551444272	0.000383123	0.001913844
ENSG00000162298	545.2677759	0.437703585	0.093989296	4.656951402	3.21E-06	2.56E-05
ENSG00000162300	158.7891781	0.445152522	0.14859372	2.995769406	0.002737534	0.010669849
ENSG00000162302	216.9813274	0.511491152	0.129369117	3.95373458	7.69E-05	0.000454685

ENSG00000162337	1165.213176	0.415341356	0.076406186	5.435965032	5.45E-08	6.00E-07
ENSG00000162341	48.60951037	0.339448451	0.25927989	1.309196987	0.190467633	0.340134609
ENSG00000162368	335.6203741	0.074721735	0.120730754	0.618912182	0.535974213	0.686811692
ENSG00000162377	552.8301446	-0.009979932	0.08620139	-0.115774608	0.907831175	0.950649754
ENSG00000162378	238.7570943	-0.325595736	0.130449178	-2.495958514	0.012561729	0.039168615
ENSG00000162384	126.3262709	-0.1244985	0.159483743	-0.780634424	0.435017539	0.601231376
ENSG00000162385	372.1774463	0.182836498	0.108251637	1.688995231	0.091220344	0.195509944
ENSG00000162390	42.93462861	-0.751349685	0.268967394	-2.793460108	0.005214746	0.018554088
ENSG00000162396	65.89883192	-0.118831695	0.242371938	-0.490286526	0.62393116	0.755922074
ENSG00000162402	882.2962182	-0.606204713	0.075477829	-8.031559964	9.62E-16	2.48E-14
ENSG00000162408	678.0003149	0.321137289	0.087490321	3.670546486	0.000242032	0.001268593
ENSG00000162413	223.6937836	-1.830729258	0.148506922	-12.32756852	6.44E-35	4.17E-33
ENSG00000162415	95.780211	0.117619404	0.18862079	0.623576034	0.532906054	0.684378437
ENSG00000162419	269.0075899	0.302881823	0.114207991	2.652019535	0.008001191	0.026772924
ENSG00000162426	11.22098825	-0.194400753	0.508875894	-0.382019969	0.702446553	0.813795194
ENSG00000162430	2153.776816	0.104587975	0.06872734	1.521781203	0.128063913	0.253618185
ENSG00000162433	152.4239637	0.409905427	0.149614187	2.73974973	0.006148598	0.021278483
ENSG00000162434	460.7986922	0.725999695	0.097524341	7.444292275	9.75E-14	2.13E-12
ENSG00000162437	350.9531709	-0.109559673	0.103467444	-1.058880642	0.289654141	0.459891889
ENSG00000162441	210.43068	0.300249587	0.128416924	2.338084238	0.019382879	0.05628929
ENSG00000162458	50.56601733	-0.947235419	0.253423414	-3.737758111	0.000185668	0.001001184
ENSG00000162461	7.679841807	0.290202749	0.619284638	0.468609636	0.639348688	0.767141968
ENSG00000162496	12.93188816	-1.323482482	0.520339731	-2.54349688	0.010974904	0.034894629
ENSG00000162512	460.2949807	0.049233516	0.095525548	0.515396325	0.6062761	0.743202756
ENSG00000162517	170.376343	-0.03817711	0.140094912	-0.272508898	0.785230751	0.871396457
ENSG00000162520	13.9925647	0.397815908	0.47293316	0.841167297	0.400254219	0.568390171
ENSG00000162521	988.1170317	0.572031599	0.07753791	7.377444089	1.61E-13	3.49E-12
ENSG00000162522	144.4211505	0.275622957	0.149535048	1.843199715	0.065299856	0.150715902
ENSG00000162526	10.33909999	0.451985379	0.541064606	0.835363049	0.403513328	0.571732068
ENSG00000162542	119.4798136	-0.522223457	0.180799294	-2.888415356	0.003871882	0.014380838
ENSG00000162545	8.274956806	-6.32802683	1.274459672	-4.96526251	6.86E-07	6.31E-06
ENSG00000162551	62.95447126	-0.719595954	0.234627195	-3.066975909	0.002162363	0.008720847
ENSG00000162572	55.93325431	-0.045717698	0.233324516	-0.195940397	0.844656818	0.909999778
ENSG00000162576	608.8915747	-0.006982281	0.100321962	-0.069598723	0.944513053	0.969143525
ENSG00000162585	373.5287812	0.510847811	0.126442426	4.040161422	5.34E-05	0.000325939
ENSG00000162591	9.950705484	-1.800185391	0.623677395	-2.886404743	0.003896707	0.01446157
ENSG00000162599	19.76916812	-0.726257044	0.415516407	-1.747842037	0.080491384	0.177414478
ENSG00000162600	65.7478107	0.056519664	0.221236442	0.255471764	0.798358749	0.87991725
ENSG00000162601	462.7324606	-0.167843951	0.105435859	-1.591905746	0.111405884	0.228188751
ENSG00000162604	190.702177	0.129739089	0.137337245	0.944675195	0.344824724	0.516556778
ENSG00000162607	476.9861627	-0.234052213	0.092330213	-2.534947171	0.011246429	0.035621935
ENSG00000162613	1524.382322	0.058957326	0.073048984	0.807093042	0.419612881	0.587374403
ENSG00000162614	21.94900061	0.142527084	0.383856513	0.371303023	0.710411847	0.818634554
ENSG00000162616	161.4789967	0.838402234	0.145821818	5.749497878	8.95E-09	1.10E-07
ENSG00000162623	307.0761773	0.077542394	0.115984598	0.668557677	0.503777678	0.661546699
ENSG00000162627	250.3054219	1.148741065	0.12319387	9.324660906	1.11E-20	3.93E-19
ENSG00000162636	55.52761173	-1.587527182	0.265640645	-5.976220922	2.28E-09	3.05E-08
ENSG00000162642	231.4283433	-0.484512874	0.124078758	-3.904881708	9.43E-05	0.000546075
ENSG00000162643	31.90746521	-1.210793947	0.329063754	-3.679511744	0.000233681	0.001228487
ENSG00000162650	254.255553	0.465763824	0.117319818	3.970035352	7.19E-05	0.000426876

ENSG00000162664	496.9591299	-0.174571844	0.090055244	-1.938497263	0.052562584	0.126841887
ENSG00000162669	10.18821123	-2.239507031	0.640070979	-3.498841699	0.000467284	0.002279145
ENSG00000162670	192.5295097	-2.824013035	0.169651364	-16.64597899	3.24E-62	4.50E-60
ENSG00000162687	62.45854912	-1.769334874	0.255458027	-6.926127537	4.33E-12	8.03E-11
ENSG00000162688	186.6892098	-0.023143774	0.138506761	-0.167094904	0.867295376	0.925611468
ENSG00000162694	177.4093592	-0.058517688	0.141755594	-0.412806903	0.679748093	0.796830632
ENSG00000162695	300.9059637	0.007599259	0.109090091	0.069660395	0.944463965	0.969143525
ENSG00000162702	377.3694585	0.053310788	0.101416849	0.525660068	0.599124413	0.7377192
ENSG00000162704	402.7472131	-0.103267289	0.103553084	-0.997240109	0.318647977	0.490203497
ENSG00000162714	590.7297091	-0.221817921	0.085770219	-2.586188109	0.009704395	0.031474865
ENSG00000162729	198.7927346	0.357798685	0.136306458	2.624957688	0.008665974	0.028617476
ENSG00000162733	2346.518556	-0.409178194	0.071010165	-5.762248182	8.30E-09	1.03E-07
ENSG00000162734	555.6886733	0.432021296	0.088538759	4.879459587	1.06E-06	9.37E-06
ENSG00000162735	258.0083568	-0.328806144	0.146771659	-2.240256367	0.025074284	0.069443705
ENSG00000162736	1686.1918	0.31727906	0.068891667	4.605478081	4.12E-06	3.20E-05
ENSG00000162738	199.0118501	-1.04073042	0.147633038	-7.049441219	1.80E-12	3.50E-11
ENSG00000162745	39.92751425	-3.613370482	0.426049478	-8.481105285	2.23E-17	6.32E-16
ENSG00000162746	11.77182337	-0.877743315	0.555526084	-1.580021783	0.114101878	0.232254612
ENSG00000162755	7.814608948	-1.999921346	0.710803453	-2.813606683	0.004898913	0.017588094
ENSG00000162769	470.9154386	0.101438789	0.093904385	1.080234844	0.280037616	0.448897454
ENSG00000162772	27.48687151	-0.545098646	0.367201668	-1.484466692	0.137685153	0.267579323
ENSG00000162775	721.3164234	-0.127673587	0.081052454	-1.575197062	0.115210965	0.234105081
ENSG00000162783	155.007522	0.264450579	0.152274241	1.736673105	0.082444888	0.180784161
ENSG00000162804	33.61579592	-0.060625477	0.30380464	-0.199554152	0.841829288	0.908092152
ENSG00000162813	320.6813708	0.542938345	0.109705724	4.949043012	7.46E-07	6.79E-06
ENSG00000162814	14.16188468	-1.928934749	0.521696702	-3.697425617	0.000217797	0.00115536
ENSG00000162819	213.6750918	0.045731709	0.126433513	0.361705592	0.717572048	0.824088015
ENSG00000162825	45.9056501	-0.520448072	0.258220966	-2.015514384	0.04385078	0.110191756
ENSG00000162836	253.4948045	-0.429716278	0.123952983	-3.466768355	0.000526756	0.002538366
ENSG00000162849	136.2422114	-0.451153562	0.159949725	-2.820596051	0.004793452	0.017269459
ENSG00000162851	249.111783	0.132621438	0.117668864	1.127073329	0.259711479	0.425938954
ENSG00000162852	490.3931905	0.177634162	0.091228663	1.947131045	0.051519026	0.124730741
ENSG00000162869	116.3037438	-0.272050172	0.167623564	-1.622982867	0.104593068	0.217211535
ENSG00000162878	59.5550794	-0.115408119	0.228474789	-0.505124088	0.613471684	0.748612122
ENSG00000162885	515.1676462	-0.087920244	0.08919576	-0.985699813	0.324280441	0.496196952
ENSG00000162889	435.7597566	-0.332938948	0.098088384	-3.394274995	0.000688106	0.003220866
ENSG00000162909	1335.075323	0.255864891	0.068899432	3.713599427	0.000204332	0.001090108
ENSG00000162910	300.4532639	0.52968132	0.111896082	4.733689616	2.20E-06	1.82E-05
ENSG00000162913	40.30870472	1.02229188	0.278891444	3.665554839	0.000246803	0.001290708
ENSG00000162923	1033.735351	0.151619542	0.071986929	2.106209329	0.035186173	0.091818721
ENSG00000162924	267.6330347	-0.061980336	0.115185572	-0.538091141	0.590514128	0.731670065
ENSG00000162927	33.08627939	0.112944362	0.297137976	0.380107463	0.703865646	0.814364144
ENSG00000162928	205.0406293	0.017054464	0.129828237	0.131361748	0.895489147	0.943302291
ENSG00000162929	78.04258139	-0.207821229	0.200810203	-1.034913695	0.300709218	0.471067221
ENSG00000162944	36.19678453	1.682487421	0.311113136	5.407960083	6.37E-08	6.94E-07
ENSG00000162959	48.78162602	0.166357753	0.260649749	0.638242521	0.523315822	0.676910316
ENSG00000162961	290.2353975	0.149294022	0.112174429	1.330909568	0.183218769	0.330466621
ENSG00000162971	125.9486741	-0.272098375	0.162650777	-1.672899326	0.094347117	0.200498328
ENSG00000162972	34.48885584	0.438263862	0.302294995	1.449788678	0.147117458	0.281126231
ENSG00000162976	22.35315735	-0.191262326	0.367028893	-0.521109728	0.602290335	0.740384382

ENSG00000162980	222.3183323	0.32094924	0.125720986	2.552869258	0.010683959	0.0340853
ENSG00000162989	77.55193395	-2.431486098	0.241449349	-10.07037754	7.47E-24	3.10E-22
ENSG00000162994	66.20129465	-0.504103737	0.220911804	-2.281923048	0.022493884	0.06344811
ENSG00000162998	15.49520958	-4.032899574	0.737236163	-5.470295379	4.49E-08	5.00E-07
ENSG00000162999	48.47359953	0.333005429	0.250930029	1.327084807	0.18448063	0.331975329
ENSG00000163001	245.0920129	0.299258165	0.122393885	2.445041801	0.014483535	0.044180287
ENSG00000163002	236.5178218	0.015692017	0.138931247	0.112948075	0.910071722	0.951381168
ENSG00000163006	179.6605696	0.012071846	0.147091581	0.082070271	0.934590834	0.964528502
ENSG00000163013	280.6601497	0.489605658	0.118571685	4.12919543	3.64E-05	0.000230966
ENSG00000163026	152.1590112	0.082662268	0.149928153	0.55134587	0.5813966	0.723809106
ENSG00000163029	1173.163909	-0.011281401	0.071168447	-0.158516886	0.874049516	0.93020483
ENSG00000163040	113.2088832	0.332361595	0.166483492	1.996363669	0.045894352	0.113991285
ENSG00000163041	144.5591943	0.482378534	0.158133737	3.050446685	0.002285012	0.00916288
ENSG00000163050	16.68271848	-1.021409853	0.434234794	-2.352206384	0.018662417	0.054557955
ENSG00000163053	60.34787143	-0.426502799	0.225834293	-1.888565254	0.058950107	0.138752035
ENSG00000163069	144.9069309	-0.388844572	0.150077243	-2.590962929	0.00957078	0.031111821
ENSG00000163071	50.29548806	-0.77292563	0.268434115	-2.879386732	0.003984494	0.014767859
ENSG00000163093	17.16860615	-0.148945993	0.419350496	-0.355182584	0.722452811	0.827824018
ENSG00000163104	311.0944976	-0.525894968	0.108776704	-4.834628628	1.33E-06	1.15E-05
ENSG00000163110	328.1444865	0.31400651	0.11367346	2.762355523	0.005738595	0.020072223
ENSG00000163125	1560.159398	-0.064198744	0.072394281	-0.886793031	0.37519034	0.545128125
ENSG00000163132	371.3802929	-6.42503743	0.310099438	-20.7192811	2.32E-95	5.43E-93
ENSG00000163138	75.9671842	0.481163655	0.209764797	2.293824614	0.021800575	0.061937868
ENSG00000163141	12.99468117	0.357717537	0.503743907	0.710117844	0.477631062	0.638134946
ENSG00000163155	78.44036659	-0.400543871	0.199995064	-2.00276878	0.045202112	0.112600474
ENSG00000163156	161.3516855	-0.192877102	0.146728779	-1.314514456	0.188673136	0.337616758
ENSG00000163159	624.933306	0.07552127	0.083633944	0.902997832	0.366527045	0.537825698
ENSG00000163161	402.250343	0.066822443	0.100812604	0.662838185	0.507434195	0.664432322
ENSG00000163162	229.0039024	0.343624075	0.134090201	2.562633749	0.010388157	0.033351859
ENSG00000163166	815.4552708	0.050552302	0.0764248	0.661464631	0.50831439	0.665159127
ENSG00000163170	262.0437802	-0.045635357	0.117516023	-0.388333061	0.697769574	0.81024793
ENSG00000163171	38.8139117	-2.771878896	0.35848466	-7.732210614	1.06E-14	2.49E-13
ENSG00000163191	424.8889766	-0.615180375	0.108781683	-5.655183461	1.56E-08	1.86E-07
ENSG00000163214	321.8965865	-0.2395011	0.106700696	-2.244606729	0.02479339	0.068760662
ENSG00000163249	102.575918	-0.598370559	0.177333475	-3.374267377	0.000740124	0.003431197
ENSG00000163251	46.53964558	-0.356139093	0.289030468	-1.232185299	0.217879874	0.374930612
ENSG00000163257	312.0629937	-0.015112129	0.108745237	-0.138968196	0.889475281	0.939574317
ENSG00000163273	19.17063272	1.006058613	0.40182527	2.503721611	0.012289469	0.038456264
ENSG00000163281	361.5281982	2.429713247	0.123853505	19.6176381	1.09E-85	2.26E-83
ENSG00000163288	20.63571063	-6.664935681	1.217520771	-5.474186427	4.40E-08	4.91E-07
ENSG00000163291	54.56583602	0.309264083	0.250612288	1.234033998	0.217190236	0.37397294
ENSG00000163297	10.16286685	-4.701425564	1.085255959	-4.33208915	1.48E-05	0.00010203
ENSG00000163312	57.6176383	-0.092539176	0.238291269	-0.388344804	0.697760885	0.81024793
ENSG00000163319	181.0157829	0.087823585	0.135541991	0.647943743	0.517021337	0.671928822
ENSG00000163320	509.8604939	-0.183470046	0.102850125	-1.783858276	0.074446697	0.166441629
ENSG00000163322	125.5654688	0.123240886	0.159809655	0.77117297	0.440604414	0.60586547
ENSG00000163328	19.79339812	0.104830245	0.385038987	0.272258779	0.785423049	0.871541004
ENSG00000163344	181.88632	-0.486556515	0.140891625	-3.453409782	0.000553547	0.002651102
ENSG00000163346	321.9634846	-0.201264897	0.115734246	-1.739026298	0.082030137	0.180127769
ENSG00000163348	401.9401697	-0.115047403	0.110130951	-1.044641871	0.29618852	0.466587433

ENSG00000163349	805.2160429	-0.213178809	0.082770817	-2.575531043	0.010008629	0.032297953
ENSG00000163359	13.29765238	2.939113535	0.626186163	4.693673715	2.68E-06	2.18E-05
ENSG00000163362	79.55416369	-0.659539812	0.19971104	-3.302470469	0.000958372	0.004325915
ENSG00000163374	483.5216985	0.178071387	0.090460273	1.968503741	0.049010106	0.120063649
ENSG00000163376	21.48530647	-2.745180846	0.479281264	-5.727703234	1.02E-08	1.25E-07
ENSG00000163378	223.8251697	0.135455105	0.124484108	1.088131708	0.276536968	0.445050666
ENSG00000163382	686.1738306	0.156556852	0.082067791	1.907652814	0.056436104	0.134093024
ENSG00000163389	144.4395387	-0.15676109	0.151264446	-1.036337974	0.300044496	0.470406446
ENSG00000163393	14.45313289	-0.880228936	0.463279972	-1.8999935	0.057433973	0.135866456
ENSG00000163399	1898.249449	-0.018667924	0.061781612	-0.302159866	0.762530198	0.856780379
ENSG00000163412	12.02988532	0.50679015	0.508348909	0.996933681	0.318796702	0.490350877
ENSG00000163428	880.2198975	0.206693162	0.083225602	2.4835286	0.013008789	0.040380758
ENSG00000163430	477.1387681	-0.876690037	0.101649817	-8.624610128	6.43E-18	1.90E-16
ENSG00000163431	38.20322655	-2.537916033	0.351708923	-7.215955763	5.36E-13	1.09E-11
ENSG00000163444	151.1271916	-0.369122178	0.159094363	-2.320146177	0.020332971	0.058573535
ENSG00000163449	12.08494566	-2.524322823	0.628585641	-4.015877328	5.92E-05	0.000357667
ENSG00000163462	75.63242747	0.678808649	0.220529517	3.078085226	0.002083353	0.008455609
ENSG00000163463	74.94788802	0.756887569	0.212193499	3.566968703	0.000361135	0.001813678
ENSG00000163466	1201.616237	0.272542185	0.068980518	3.951002282	7.78E-05	0.000459329
ENSG00000163468	3966.265992	0.4151155	0.058002339	7.156875212	8.25E-13	1.65E-11
ENSG00000163472	20.60415021	0.203435659	0.400578023	0.507855267	0.611554848	0.747517508
ENSG00000163479	1368.538824	0.449487899	0.069684791	6.450301326	1.12E-10	1.76E-09
ENSG00000163481	112.6202192	0.259139787	0.182821072	1.41745032	0.15635131	0.294012249
ENSG00000163482	153.1024288	-0.224497267	0.155682946	-1.442015798	0.149297917	0.283979396
ENSG00000163485	23.0283319	-0.59492249	0.364315519	-1.632986956	0.10247174	0.213754163
ENSG00000163491	16.36011232	-0.137570497	0.437117399	-0.314722081	0.752972678	0.850788868
ENSG00000163507	262.8707993	-0.276863117	0.120141935	-2.304466942	0.021196446	0.060591469
ENSG00000163510	487.2795443	0.025681788	0.094888679	0.270651758	0.786658884	0.87229218
ENSG00000163512	294.4771652	0.263245311	0.110067488	2.391671832	0.016771831	0.050019939
ENSG00000163513	151.8452099	0.062261275	0.152157793	0.409188864	0.682401066	0.799073672
ENSG00000163516	325.0261279	0.036779345	0.115811073	0.317580559	0.750803123	0.848952062
ENSG00000163517	44.04844772	-0.241655661	0.262357782	-0.921092026	0.357002383	0.528264244
ENSG00000163520	36.32741701	-2.160681367	0.340317329	-6.34901952	2.17E-10	3.29E-09
ENSG00000163521	19.08028678	-0.665541477	0.400037851	-1.663696261	0.096173144	0.20348609
ENSG00000163527	1155.538474	0.261380391	0.074185501	3.523335254	0.000426152	0.002100406
ENSG00000163528	192.4754332	-0.047406905	0.139118488	-0.34076639	0.733279455	0.835693316
ENSG00000163531	76.06370805	0.121044797	0.20864694	0.580141734	0.561819042	0.708172298
ENSG00000163535	374.778362	-0.148433933	0.104951398	-1.414311165	0.15727056	0.295273461
ENSG00000163536	19.46020244	-0.307492463	0.395991922	-0.776511958	0.437446763	0.603314879
ENSG00000163539	466.5214444	-0.192633152	0.103762721	-1.856477459	0.063385533	0.147169683
ENSG00000163541	498.6700709	0.008693703	0.110133129	0.078938131	0.937081833	0.965538657
ENSG00000163545	22.58704541	-1.510182776	0.39384804	-3.834430094	0.000125856	0.000709998
ENSG00000163558	330.1567737	-0.030284678	0.107499916	-0.281718158	0.77815963	0.867249153
ENSG00000163577	144.3809046	0.217105293	0.148854861	1.458503218	0.144701889	0.277681343
ENSG00000163584	1668.145061	0.455911545	0.080368298	5.672778372	1.40E-08	1.69E-07
ENSG00000163590	77.2653227	-0.325324917	0.205349789	-1.584247635	0.113137377	0.230994293
ENSG00000163596	109.7078776	-0.182432799	0.175548542	-1.039215688	0.298704436	0.469182736
ENSG00000163597	1256.599853	0.007598548	0.073855745	0.102883642	0.918055321	0.955355281
ENSG00000163602	224.1498072	0.023053399	0.124841177	0.184661819	0.853494295	0.916032577
ENSG00000163605	253.8705783	0.08037145	0.125813928	0.63881202	0.522945228	0.676628438

ENSG00000163607	105.7220804	-0.267270569	0.178706141	-1.495586933	0.13476133	0.26350117
ENSG00000163608	212.1831312	0.063546468	0.128928381	0.492881918	0.622096024	0.754883986
ENSG00000163611	26.87738046	-0.212046063	0.335518401	-0.631995332	0.527389927	0.680279734
ENSG00000163617	28.98308464	-0.628407399	0.335531583	-1.872871081	0.061086189	0.142893872
ENSG00000163624	14.71112862	2.077008513	0.511543674	4.060276016	4.90E-05	0.000302642
ENSG00000163625	896.9797308	0.002291722	0.080430775	0.028493104	0.977268868	0.988629033
ENSG00000163626	147.6802113	-0.265192825	0.160347762	-1.653860468	0.098155866	0.20671596
ENSG00000163629	683.9602387	-0.08939206	0.092252377	-0.968994653	0.332547857	0.504950789
ENSG00000163634	291.2497318	0.18114266	0.118377836	1.53020756	0.125965361	0.250907557
ENSG00000163635	736.0635488	0.124342456	0.082043916	1.515559751	0.129630711	0.256179318
ENSG00000163636	498.4813057	0.473703701	0.093744706	5.053124842	4.35E-07	4.15E-06
ENSG00000163644	103.1547464	0.041593572	0.179366484	0.231891551	0.816622247	0.892216525
ENSG00000163655	673.6093373	-0.003741297	0.085536568	-0.04373915	0.965112332	0.981675085
ENSG00000163659	135.2637283	-0.008380187	0.159422028	-0.052566054	0.958077665	0.977492516
ENSG00000163660	744.5610353	0.081834661	0.084767458	0.965401849	0.334343576	0.506145512
ENSG00000163681	793.7367251	-0.070252964	0.084591782	-0.83049396	0.406259561	0.57446427
ENSG00000163682	1787.638287	0.220563761	0.079158872	2.786342909	0.005330645	0.018880375
ENSG00000163683	31.29336125	-0.097199278	0.324669805	-0.299378865	0.764650987	0.858254288
ENSG00000163684	72.63433039	-0.254331747	0.20509055	-1.240094905	0.214940293	0.371008393
ENSG00000163686	70.36044395	-0.337259513	0.212935011	-1.58386125	0.113225297	0.231106619
ENSG00000163689	8.703986327	-1.900538214	0.655972459	-2.897283548	0.003764094	0.014024994
ENSG00000163694	63.32804209	-1.277007223	0.253418641	-5.039121116	4.68E-07	4.43E-06
ENSG00000163697	169.5405648	-0.73865601	0.14315639	-5.159783726	2.47E-07	2.45E-06
ENSG00000163702	42.54514374	0.49702984	0.270334949	1.838570415	0.065978396	0.151934185
ENSG00000163703	37.18328734	0.333150527	0.288662913	1.154116141	0.248452557	0.412677435
ENSG00000163704	126.1781437	-0.819615063	0.170560449	-4.805422743	1.54E-06	1.31E-05
ENSG00000163710	173.1699914	1.042643182	0.146477897	7.11809223	1.09E-12	2.17E-11
ENSG00000163714	1505.439267	0.025757434	0.066214738	0.38899851	0.697277248	0.810027539
ENSG00000163719	279.8409919	0.183736753	0.116308348	1.579738309	0.114166809	0.232353109
ENSG00000163728	90.37937834	-0.229787994	0.193474895	-1.187688946	0.23495598	0.395972004
ENSG00000163738	42.20946097	-0.438249552	0.266808036	-1.642565039	0.100472959	0.210428457
ENSG00000163743	56.6327272	0.322283545	0.232604539	1.385542801	0.165886554	0.307537077
ENSG00000163754	125.4156706	0.282255294	0.167317233	1.68694694	0.091613555	0.196083439
ENSG00000163755	156.2782459	-0.10026316	0.151027013	-0.663875674	0.506769887	0.664065744
ENSG00000163781	732.0657897	0.118299637	0.079915851	1.480302532	0.138792528	0.269358751
ENSG00000163785	563.737916	-0.096658409	0.093850146	-1.029922837	0.30304623	0.47377745
ENSG00000163788	268.5902369	-0.252984037	0.114370276	-2.211973647	0.026968486	0.073917331
ENSG00000163794	22.96294452	0.226665493	0.3694199	0.613571421	0.539498574	0.689580557
ENSG00000163795	56.43891231	0.625031326	0.252180553	2.478507241	0.013193343	0.040847894
ENSG00000163798	507.1726301	-0.00891627	0.096836677	-0.092075341	0.92663818	0.959877081
ENSG00000163807	348.4323235	0.343655034	0.114066642	3.012756642	0.002588864	0.010186492
ENSG00000163808	578.3037579	0.036708289	0.086864671	0.422591696	0.672593187	0.791282788
ENSG00000163811	1455.377828	-0.050479043	0.074458681	-0.67794705	0.497805266	0.65591132
ENSG00000163812	564.665839	-0.253135955	0.089225304	-2.837042236	0.004553359	0.01654022
ENSG00000163818	108.0772873	0.045003542	0.174197158	0.258348314	0.796138102	0.878365687
ENSG00000163820	1746.683111	0.550282069	0.069812415	7.882295286	3.21E-15	7.87E-14
ENSG00000163832	233.5384255	-0.073556497	0.131580602	-0.559022347	0.576146469	0.720349751
ENSG00000163840	204.5440011	0.631630176	0.13162781	4.798607352	1.60E-06	1.35E-05
ENSG00000163848	424.7985116	-0.155977185	0.102190993	-1.526330066	0.126927684	0.252078776
ENSG00000163864	21.43124979	-0.079263468	0.375209365	-0.211251305	0.832691178	0.902134873

ENSG00000163866	114.0992737	0.047724264	0.166293752	0.286987719	0.774121724	0.864738597
ENSG00000163867	55.41604718	-0.006144596	0.243294544	-0.02525579	0.979850937	0.989786143
ENSG00000163870	113.4359478	-0.064968444	0.171377156	-0.379096289	0.704616362	0.814832908
ENSG00000163872	439.2300502	-0.140348233	0.097054315	-1.446079269	0.14815497	0.282378949
ENSG00000163874	48.40162373	0.745736021	0.254974225	2.92475062	0.003447324	0.013020854
ENSG00000163875	233.1552324	0.260202405	0.127571857	2.039653663	0.041384833	0.105009529
ENSG00000163877	150.580919	-0.19228289	0.148249437	-1.297022734	0.194623365	0.345841165
ENSG00000163879	12.5891458	0.332784231	0.480187405	0.693029902	0.488290786	0.648178102
ENSG00000163882	265.9146062	0.019713555	0.121528804	0.162213028	0.871138101	0.928186766
ENSG00000163884	9.660041887	-1.493185206	0.644039016	-2.318470106	0.020423786	0.058801582
ENSG00000163888	14.75336696	-1.540468827	0.498275402	-3.091601193	0.001990801	0.008138761
ENSG00000163900	69.65046908	-0.256870952	0.214176445	-1.199342681	0.230394726	0.389640586
ENSG00000163902	1259.111059	0.413171147	0.070320239	5.875565149	4.21E-09	5.41E-08
ENSG00000163904	157.7566923	-0.439616737	0.146292035	-3.005062695	0.002655261	0.010409839
ENSG00000163909	100.4990927	-2.111527255	0.20711958	-10.19472546	2.09E-24	8.91E-23
ENSG00000163913	232.0299741	-0.1788489	0.126106221	-1.418240097	0.156120679	0.293735223
ENSG00000163918	235.8527489	-0.130794595	0.122299304	-1.069463119	0.284861039	0.454408643
ENSG00000163923	111.1383802	-0.335857716	0.171154102	-1.962311809	0.049726193	0.121331992
ENSG00000163930	650.9005558	0.009248013	0.092378093	0.100110453	0.920256636	0.956339792
ENSG00000163931	8545.770192	0.290256203	0.050282799	5.772475051	7.81E-09	9.75E-08
ENSG00000163932	287.842579	0.220351592	0.11442393	1.925747462	0.054135914	0.129771349
ENSG00000163933	216.3323323	-0.165794524	0.125510179	-1.320964758	0.186513115	0.334722864
ENSG00000163935	128.8065509	0.473642389	0.159681733	2.966165144	0.003015385	0.011588814
ENSG00000163938	2041.804843	0.051200294	0.068008152	0.752855248	0.451536903	0.616629899
ENSG00000163939	1128.238702	0.0385946	0.069334949	0.556639919	0.577773474	0.721335669
ENSG00000163945	182.4300547	0.003978876	0.14299164	0.027825935	0.977800981	0.988629033
ENSG00000163946	843.4234565	-0.10323972	0.08141264	-1.268104307	0.204760704	0.358155148
ENSG00000163947	40.79555147	-0.559569731	0.296756648	-1.885618185	0.059346425	0.139567928
ENSG00000163950	235.9021785	-0.008706809	0.122553718	-0.071044837	0.943362071	0.968637121
ENSG00000163956	1350.192455	0.745865085	0.075190313	9.919696503	3.42E-23	1.36E-21
ENSG00000163960	655.6727973	-0.110491278	0.08321084	-1.327847172	0.1842286	0.331691764
ENSG00000163961	328.838419	-0.063133981	0.104989074	-0.601338589	0.547614492	0.695834778
ENSG00000163964	188.3704285	-0.138044759	0.134557558	-1.025916057	0.304931153	0.475876892
ENSG00000163975	128.0876387	-0.429106901	0.173357386	-2.475273253	0.013313427	0.041165245
ENSG00000164002	28.99289504	-0.316534045	0.329369812	-0.961029316	0.336537428	0.508425602
ENSG00000164008	19.45835304	-0.846795249	0.405891592	-2.086259648	0.03695511	0.095582476
ENSG00000164010	52.84617166	-1.455770581	0.258017422	-5.642140628	1.68E-08	2.00E-07
ENSG00000164011	44.29831216	-1.229236317	0.276561036	-4.444719821	8.80E-06	6.39E-05
ENSG00000164022	445.6815411	0.194458049	0.101188635	1.921738035	0.054638729	0.13079588
ENSG00000164023	25.86327436	0.390271189	0.34307787	1.137558622	0.255304833	0.42057781
ENSG00000164024	401.4281468	-0.001952963	0.101787519	-0.019186666	0.984692194	0.992036954
ENSG00000164031	253.4842551	-0.049029256	0.122182989	-0.401277263	0.688216002	0.803671974
ENSG00000164032	1690.442354	-0.117481051	0.077297627	-1.519853273	0.128547857	0.254361423
ENSG00000164037	16.74588955	-0.415546436	0.438935977	-0.946713092	0.343784998	0.515604085
ENSG00000164038	322.6680884	0.187197562	0.111461923	1.679475431	0.093059423	0.198482496
ENSG00000164039	77.10224991	0.015899055	0.206540836	0.07697778	0.938641222	0.966112632
ENSG00000164040	363.2140311	0.116101129	0.11403624	1.018107311	0.308626961	0.479885203
ENSG00000164045	478.7347207	0.021486331	0.092757177	0.231640631	0.816817146	0.892349545
ENSG00000164048	595.0182093	-0.122289242	0.100712362	-1.214242614	0.224655092	0.382809194
ENSG00000164050	73.59129486	-0.243319263	0.216371488	-1.124544022	0.260782306	0.427046987

ENSG00000164051	102.4278673	-0.098182421	0.174330779	-0.563196136	0.573301334	0.717878752
ENSG00000164053	136.2335942	0.142551949	0.15525498	0.918179561	0.358524867	0.529791585
ENSG00000164054	1492.026782	0.175024408	0.076180619	2.297492613	0.021590684	0.061468248
ENSG00000164056	882.6266556	-4.984387917	0.142312433	-35.02426179	9.61E-269	1.23E-265
ENSG00000164061	59.41199166	0.930399069	0.233673225	3.981624635	6.84E-05	0.000409305
ENSG00000164062	1187.540076	-0.197527684	0.076429513	-2.584442537	0.009753656	0.031597846
ENSG00000164066	147.175116	-0.067714285	0.15187679	-0.445850119	0.655705516	0.779180728
ENSG00000164068	538.2023885	0.489832959	0.091738139	5.339469076	9.32E-08	9.86E-07
ENSG00000164070	772.0905259	0.525876678	0.078158893	6.728302542	1.72E-11	2.95E-10
ENSG00000164073	71.5687017	-0.288841232	0.208751681	-1.38365943	0.166462762	0.308279817
ENSG00000164074	79.40508011	-0.203903809	0.196949899	-1.035308019	0.300525086	0.4708514
ENSG00000164076	24.48038041	-1.195657525	0.366659534	-3.260947594	0.001110406	0.004928236
ENSG00000164077	170.1724378	0.303261208	0.153602484	1.974324897	0.048344812	0.118901179
ENSG00000164080	854.5303536	-0.062007337	0.076670394	-0.808752033	0.418657792	0.586578219
ENSG00000164081	401.2516263	0.177606815	0.098605917	1.801178067	0.071674818	0.16189358
ENSG00000164082	14.31209843	-0.419199037	0.468896778	-0.894011341	0.371315823	0.54217406
ENSG00000164086	696.9802252	0.397879028	0.080129159	4.965471187	6.85E-07	6.30E-06
ENSG00000164087	192.7415907	-0.102485765	0.136681586	-0.749813988	0.453366743	0.617396281
ENSG00000164088	32.87097097	-0.386906132	0.309344496	-1.250729001	0.211033366	0.366274791
ENSG00000164091	1106.289527	-0.027290226	0.072078901	-0.378616016	0.704973027	0.815025133
ENSG00000164096	335.9396005	0.300604818	0.106244857	2.82935877	0.004664138	0.016877163
ENSG00000164104	1880.323072	-0.141959099	0.067270212	-2.110281717	0.034834097	0.091086107
ENSG00000164105	133.7985985	0.53535713	0.165053939	3.243528351	0.001180591	0.005184044
ENSG00000164107	629.3026879	4.993172648	0.23324803	21.40713745	1.15E-101	2.98E-99
ENSG00000164109	310.8800705	-0.661023483	0.11559175	-5.718604323	1.07E-08	1.31E-07
ENSG00000164111	1400.310988	0.278912075	0.065648564	4.248563237	2.15E-05	0.00014357
ENSG00000164114	785.2171182	-1.865744116	0.087536772	-21.31383277	8.45E-101	2.12E-98
ENSG00000164116	33.89056448	2.405009091	0.347969383	6.911553736	4.79E-12	8.79E-11
ENSG00000164117	207.531874	1.378608881	0.132876855	10.37508666	3.22E-25	1.46E-23
ENSG00000164118	553.3910129	1.300774809	0.107784903	12.0682468	1.55E-33	9.53E-32
ENSG00000164124	81.17798949	0.535288651	0.197151285	2.715116217	0.006625252	0.022698758
ENSG00000164125	151.7835096	1.89961345	0.161423355	11.76789722	5.71E-32	3.33E-30
ENSG00000164134	884.3689465	0.147795275	0.076546243	1.930797244	0.053508131	0.128461583
ENSG00000164142	78.3723917	-1.208637242	0.22338086	-5.410657131	6.28E-08	6.85E-07
ENSG00000164144	142.3398902	0.080778925	0.149435136	0.54056179	0.58880966	0.730202993
ENSG00000164151	474.8368212	0.064360463	0.092680179	0.694436113	0.487408753	0.64779673
ENSG00000164161	28.0910961	-4.078089476	0.557313912	-7.317401175	2.53E-13	5.33E-12
ENSG00000164163	430.1335911	0.037156793	0.110099058	0.337485108	0.735751236	0.83739245
ENSG00000164164	554.1526306	-0.206436664	0.091328796	-2.26036775	0.023798436	0.066513457
ENSG00000164167	279.4243635	0.176429355	0.115338529	1.529665378	0.126099578	0.2511087
ENSG00000164168	228.4157056	0.037366295	0.12956782	0.288391782	0.773046857	0.864018874
ENSG00000164169	64.91618161	-0.169501845	0.224027316	-0.756612401	0.44928211	0.614298178
ENSG00000164171	74.94400053	0.078412847	0.20261341	0.387007191	0.698750884	0.810918182
ENSG00000164172	126.6680603	0.480340269	0.165050151	2.910268579	0.003611183	0.013563178
ENSG00000164180	129.9450819	-0.317635999	0.157324477	-2.018986527	0.04348862	0.109361608
ENSG00000164182	415.52026	0.26558363	0.101403987	2.61906498	0.008817115	0.029024793
ENSG00000164187	73.48934537	0.227475898	0.208632792	1.090317084	0.275573492	0.443919822
ENSG00000164190	1348.856702	-0.117537484	0.068900197	-1.705909276	0.088024972	0.189907355
ENSG00000164199	12.80699583	0.001674352	0.485459141	0.003449006	0.997248097	0.998813347
ENSG00000164209	159.819493	-0.480008108	0.151756522	-3.163014684	0.001561445	0.0066085

ENSG00000164211	557.1588626	0.107919427	0.086569893	1.246616155	0.212538275	0.367720798
ENSG00000164219	409.9234228	-0.331800917	0.102199628	-3.246596141	0.00116794	0.005138131
ENSG00000164221	174.2714717	0.247114796	0.146750063	1.683916113	0.092197882	0.197033873
ENSG00000164237	287.566282	0.193804279	0.113207738	1.71193491	0.086908664	0.188192695
ENSG00000164244	695.5245784	0.251556689	0.080460133	3.126476192	0.001769148	0.007358965
ENSG00000164252	111.5977576	0.277078202	0.182415561	1.518939506	0.128777724	0.254677743
ENSG00000164253	161.1946562	-0.19042971	0.149896105	-1.270411328	0.20393815	0.357063342
ENSG00000164258	243.2091818	-0.054399219	0.125825021	-0.432340239	0.665494145	0.786025079
ENSG00000164284	347.5549234	-0.347496936	0.105938387	-3.280179612	0.00103741	0.004642402
ENSG00000164291	43.39150031	0.246335982	0.27894955	0.883084351	0.377190699	0.546670343
ENSG00000164292	691.6502984	0.06050269	0.086669466	0.69808542	0.485123776	0.64556022
ENSG00000164294	203.3543434	-0.397251887	0.136297332	-2.914597669	0.003561473	0.01340515
ENSG00000164296	67.72365339	-0.111892698	0.218699921	-0.511626604	0.608912363	0.745198811
ENSG00000164300	155.1559901	-0.269846761	0.152961841	-1.764144305	0.077707687	0.172578637
ENSG00000164303	29.36875678	0.78313889	0.329235774	2.378656727	0.017375849	0.051525975
ENSG00000164305	147.1220756	-0.117552648	0.154183787	-0.762418995	0.445809973	0.61077662
ENSG00000164306	71.88051372	-0.265695267	0.227866759	-1.166011525	0.243609786	0.406701528
ENSG00000164307	385.2396336	0.015113637	0.100307703	0.150672749	0.880233871	0.93402668
ENSG00000164308	134.3222242	0.646910869	0.162011704	3.992988487	6.52E-05	0.00039167
ENSG00000164309	16.98198874	0.44546542	0.433254347	1.028184537	0.303863031	0.474638867
ENSG00000164323	698.5695928	0.234949268	0.086254482	2.723907918	0.006451449	0.022206201
ENSG00000164327	179.5051726	-0.244432183	0.135814398	-1.799751622	0.071899866	0.162219309
ENSG00000164329	280.0249181	-0.215934136	0.113911315	-1.895633774	0.058008488	0.136948778
ENSG00000164331	38.41521531	0.046863184	0.283884076	0.165078594	0.868882122	0.926961915
ENSG00000164332	198.1151695	-0.11752855	0.143912475	-0.816666869	0.41411883	0.582431606
ENSG00000164338	157.1867805	-0.192383041	0.151336339	-1.271228327	0.203647432	0.356643246
ENSG00000164342	20.22824272	1.07867701	0.43806071	2.462391596	0.013801389	0.042375542
ENSG00000164346	1027.579208	0.158957246	0.077424716	2.053055596	0.040067186	0.101958163
ENSG00000164347	337.7094313	-0.137362928	0.105408562	-1.303147729	0.192524331	0.343022486
ENSG00000164362	67.0012125	0.06794774	0.219541482	0.309498411	0.75694242	0.853146929
ENSG00000164366	85.0011746	-0.010302832	0.190796586	-0.053999036	0.956935932	0.976895279
ENSG00000164385	10.34560722	-0.023653032	0.530566033	-0.04458075	0.964441487	0.981348031
ENSG00000164402	212.2097019	-0.032299322	0.131949965	-0.244784622	0.806623201	0.885690308
ENSG00000164403	199.3854987	0.433728522	0.137482091	3.154800146	0.001606081	0.006779032
ENSG00000164404	9.017001113	0.686844565	0.582484393	1.179163894	0.23833292	0.399840264
ENSG00000164405	1051.229185	0.77695412	0.086107022	9.023121487	1.83E-19	6.04E-18
ENSG00000164414	18.65892343	0.293811564	0.417033188	0.704528014	0.48110401	0.641548133
ENSG00000164418	572.7204923	1.676281088	0.092528242	18.11642649	2.36E-73	3.91E-71
ENSG00000164438	99.8796176	-5.097037279	0.393140045	-12.96494049	1.93E-38	1.44E-36
ENSG00000164442	334.1887194	-2.011141487	0.138940876	-14.47480066	1.75E-47	1.71E-45
ENSG00000164463	90.57829417	0.074136896	0.190094357	0.390000508	0.696536171	0.809484868
ENSG00000164465	109.494317	0.54240014	0.173578851	3.12480545	0.001779227	0.007389947
ENSG00000164466	819.5002262	0.312196272	0.076482232	4.081945081	4.47E-05	0.000277709
ENSG00000164494	72.44190859	-0.146818369	0.232571676	-0.631282239	0.527855998	0.680568444
ENSG00000164506	693.3061766	-0.760806344	0.088676944	-8.579528243	9.53E-18	2.78E-16
ENSG00000164532	34.51844996	-0.014256575	0.299145844	-0.047657606	0.961989121	0.979825424
ENSG00000164535	83.16804959	0.133565816	0.195612473	0.682808281	0.494727997	0.653757683
ENSG00000164542	18.02433118	-0.38519839	0.430761479	-0.894226641	0.371200641	0.54217406
ENSG00000164543	49.66101941	0.129768223	0.246394478	0.526668553	0.598423777	0.737337313
ENSG00000164548	944.3914216	-0.018670902	0.076508977	-0.24403544	0.807203373	0.885981073

ENSG00000164574	112.0812917	0.104919093	0.170951361	0.613736515	0.539389454	0.689580557
ENSG00000164576	39.62268053	-0.354267282	0.277068952	-1.278624979	0.20102915	0.353410009
ENSG00000164587	1918.764806	0.459225404	0.085464474	5.373289984	7.73E-08	8.26E-07
ENSG00000164588	10.48135962	-3.660796221	0.842830075	-4.343457037	1.40E-05	9.75E-05
ENSG00000164597	220.3058045	0.47756149	0.129578646	3.685495281	0.000228258	0.001203131
ENSG00000164603	31.49540711	0.389306197	0.30733787	1.266704284	0.205261048	0.358682061
ENSG00000164609	279.9949214	0.17062036	0.11486966	1.485338773	0.137454106	0.267278215
ENSG00000164610	117.7811049	0.123160577	0.164937332	0.74671134	0.455237823	0.618924284
ENSG00000164611	710.7430012	0.24765897	0.085119842	2.909532775	0.003619694	0.01358788
ENSG00000164615	283.2908586	0.127989642	0.117180813	1.092240609	0.274727358	0.443115764
ENSG00000164620	56.22086363	0.661724677	0.236071031	2.803074458	0.005061798	0.018087784
ENSG00000164626	52.33727198	-0.270492196	0.249923809	-1.082298629	0.279119855	0.447809167
ENSG00000164631	254.3283893	-0.107079115	0.121953251	-0.878034113	0.379925206	0.549450157
ENSG00000164638	52.75121967	-1.379674821	0.259794914	-5.310630595	1.09E-07	1.14E-06
ENSG00000164647	58.87493627	0.743081906	0.233979525	3.175841587	0.001494025	0.006371151
ENSG00000164654	163.1891962	-0.332665058	0.147679626	-2.252613083	0.024283555	0.067653928
ENSG00000164663	36.1404778	-0.295451364	0.29436148	-1.003702534	0.315522015	0.486941084
ENSG00000164683	16.93469264	-0.997736238	0.434411392	-2.296754315	0.021632789	0.061550696
ENSG00000164684	303.5515487	0.077393416	0.110506654	0.700350732	0.483708297	0.644164813
ENSG00000164687	689.7373449	0.396956806	0.087261046	4.549072291	5.39E-06	4.10E-05
ENSG00000164694	21.2675939	-3.3010878	0.534898068	-6.171433398	6.77E-10	9.66E-09
ENSG00000164695	67.06606757	0.437660449	0.222365545	1.968202624	0.049044728	0.120127515
ENSG00000164713	423.795757	0.36976417	0.119937272	3.082979665	0.002049392	0.008334628
ENSG00000164715	541.1836175	-0.069534962	0.094926252	-0.73251561	0.463853918	0.626876659
ENSG00000164733	1595.323326	0.207885178	0.070451257	2.950766045	0.003169869	0.012109642
ENSG00000164742	234.0009074	-0.883653676	0.123561744	-7.151515094	8.58E-13	1.71E-11
ENSG00000164749	63.43811378	0.106794547	0.225576434	0.473429538	0.635906761	0.764972362
ENSG00000164751	131.3022718	-0.20228573	0.186048593	-1.087273637	0.276915896	0.445469261
ENSG00000164754	2428.383317	0.065884611	0.06548158	1.006154868	0.314341079	0.485598717
ENSG00000164758	62.18846561	-0.127454704	0.226173031	-0.563527417	0.573075796	0.717878752
ENSG00000164808	313.5413432	-0.062866645	0.108857319	-0.577514177	0.563592164	0.70970678
ENSG00000164815	142.7565188	0.491746545	0.15871384	3.098321765	0.0019462	0.007993646
ENSG00000164818	603.3676389	0.079653425	0.086177858	0.924291082	0.355334785	0.526906758
ENSG00000164823	263.028683	0.268037388	0.12055964	2.223276269	0.026197177	0.072155243
ENSG00000164828	767.1881484	-0.00089168	0.077907911	-0.011445313	0.990868161	0.995236126
ENSG00000164830	185.7464252	0.564853745	0.135946343	4.154975646	3.25E-05	0.000208857
ENSG00000164849	14.3047452	0.650186311	0.464591445	1.39947973	0.161669173	0.301334207
ENSG00000164850	85.94751696	0.433824997	0.19638037	2.20910571	0.027167289	0.074317217
ENSG00000164855	45.21913936	1.261499029	0.272942953	4.621841355	3.80E-06	2.99E-05
ENSG00000164867	659.8922464	0.706811361	0.084551339	8.359552558	6.30E-17	1.74E-15
ENSG00000164877	52.22885396	0.085577772	0.245136381	0.349102697	0.727012212	0.831311943
ENSG00000164880	1178.219	0.173433506	0.076578448	2.26478219	0.023526049	0.065870123
ENSG00000164885	89.85659339	0.619530195	0.185604654	3.337902271	0.000844134	0.00385737
ENSG00000164889	1253.459777	1.121857494	0.071701542	15.64621144	3.53E-55	4.20E-53
ENSG00000164896	229.9583841	0.672382372	0.130933928	5.135279931	2.82E-07	2.76E-06
ENSG00000164897	329.3733595	0.755665627	0.125869073	6.003584569	1.93E-09	2.60E-08
ENSG00000164902	461.4507957	0.128906546	0.092800013	1.389078959	0.164808741	0.305942697
ENSG00000164904	635.2191614	-0.630364446	0.085436075	-7.378199928	1.60E-13	3.47E-12
ENSG00000164916	1284.715951	0.184941164	0.087988753	2.101872764	0.035564423	0.092650862
ENSG00000164919	751.02579	0.299945586	0.098863321	3.033942052	0.002413808	0.009594442

ENSG00000164920	99.33284981	-0.068048894	0.195768924	-0.34759804	0.728142079	0.831967771
ENSG00000164924	1804.74905	0.013073201	0.063105363	0.207164664	0.835881263	0.904036349
ENSG00000164930	122.6662909	-1.253456427	0.169376364	-7.400421163	1.36E-13	2.95E-12
ENSG00000164933	72.19940306	0.119823159	0.246784256	0.485538099	0.627294694	0.758183494
ENSG00000164934	389.9678577	0.310796141	0.100938381	3.079068037	0.002076493	0.0084302
ENSG00000164941	528.433759	0.279558581	0.093142306	3.001413554	0.002687293	0.010506098
ENSG00000164944	641.4427089	0.244072559	0.0831184	2.936444391	0.003319983	0.012597463
ENSG00000164946	1006.199414	-0.000423602	0.076743823	-0.00551969	0.995595947	0.997940364
ENSG00000164951	123.7073237	0.39965778	0.163779942	2.440211997	0.014678645	0.044668745
ENSG00000164953	106.8937119	-0.281381629	0.174271385	-1.614617505	0.106393552	0.22020189
ENSG00000164961	522.300623	0.307284732	0.089950117	3.416168241	0.00063509	0.002997684
ENSG00000164967	50.96056285	0.070257253	0.246469892	0.285054098	0.775602697	0.86552026
ENSG00000164970	177.4392499	0.825742634	0.142624108	5.789642756	7.05E-09	8.86E-08
ENSG00000164975	285.1216207	0.236275598	0.117840158	2.005051605	0.04495753	0.112170236
ENSG00000164976	86.2478215	-0.452440267	0.205056307	-2.206419664	0.027354629	0.074727826
ENSG00000164978	99.37373616	0.186747426	0.17798642	1.049222895	0.294075544	0.464404628
ENSG00000164983	75.00633564	-0.281723868	0.202261477	-1.392869631	0.163659217	0.304203511
ENSG00000164985	5028.732892	-0.047864372	0.059965105	-0.798203752	0.424752261	0.591914187
ENSG00000164989	91.33698015	-0.188920513	0.186199867	-1.014611429	0.310291089	0.481672995
ENSG00000165006	320.1385241	0.149499112	0.111085709	1.345799691	0.178367125	0.324045914
ENSG00000165028	17.71733442	0.105686415	0.410073266	0.257725689	0.796618618	0.878481844
ENSG00000165029	454.3683957	-1.225164814	0.098047921	-12.49557162	7.89E-36	5.25E-34
ENSG00000165030	22.72561941	-0.723077931	0.369699784	-1.955851644	0.050482633	0.122771736
ENSG00000165046	14.04892745	1.221009731	0.469286892	2.601840687	0.009272492	0.030304953
ENSG00000165055	479.3215949	0.843475328	0.092213193	9.147013578	5.85E-20	1.98E-18
ENSG00000165060	163.6490336	-0.078935664	0.147060871	-0.536755042	0.59143683	0.732133279
ENSG00000165097	244.4964309	0.452900113	0.123355616	3.67149974	0.000241131	0.001264342
ENSG00000165102	457.2994416	-0.477448035	0.102044689	-4.678813183	2.89E-06	2.32E-05
ENSG00000165113	58.12271189	-0.097622325	0.231604119	-0.421505132	0.673386264	0.791884207
ENSG00000165115	46.80527211	-0.611674281	0.260294535	-2.349931321	0.018776875	0.054830888
ENSG00000165119	4330.941608	0.215503614	0.055257804	3.899967027	9.62E-05	0.000555904
ENSG00000165138	537.4089965	-0.008801591	0.088479825	-0.099475677	0.920760599	0.956651193
ENSG00000165152	310.7246888	0.165036868	0.118351523	1.394463406	0.163177713	0.303516596
ENSG00000165156	479.4430227	0.059706908	0.092623362	0.644620395	0.519173215	0.67395122
ENSG00000165164	28.64430497	0.33604125	0.319732415	1.051007761	0.293255025	0.46389459
ENSG00000165171	22.86128385	-0.860457284	0.383313433	-2.244787711	0.024781764	0.068745301
ENSG00000165175	581.6416308	-0.106991053	0.091447767	-1.169968998	0.242013445	0.404709865
ENSG00000165185	264.7783288	-0.287197352	0.121576312	-2.362280514	0.018162893	0.05342005
ENSG00000165194	7.662758732	-2.053435756	0.728518133	-2.818647421	0.004822646	0.017352384
ENSG00000165195	38.76107932	0.154256411	0.277157072	0.556566752	0.577823475	0.721335669
ENSG00000165209	250.1979294	-0.051613987	0.118787979	-0.434505136	0.663921666	0.785091981
ENSG00000165219	736.9079074	0.052041088	0.081634416	0.637489559	0.523806009	0.677336188
ENSG00000165233	255.3300803	0.632799969	0.123021758	5.143805287	2.69E-07	2.65E-06
ENSG00000165238	824.4328432	0.454552709	0.080242238	5.664756117	1.47E-08	1.77E-07
ENSG00000165240	87.71030582	-0.212214156	0.190755973	-1.112490227	0.26592743	0.432748217
ENSG00000165244	181.5903307	0.116411187	0.135472772	0.859295823	0.390177328	0.558526539
ENSG00000165259	27.39709141	-1.806594634	0.383200464	-4.714489682	2.42E-06	1.98E-05
ENSG00000165264	97.04906624	0.182239861	0.184135602	0.989704643	0.322318505	0.494235491
ENSG00000165271	309.6742514	0.45351377	0.112367456	4.035988594	5.44E-05	0.000330925
ENSG00000165272	7.698261875	-0.555249321	0.63267605	-0.877620262	0.38014983	0.549661828

ENSG00000165275	57.58471117	0.281506065	0.234986404	1.197967459	0.230929686	0.390451287
ENSG00000165280	1853.467636	0.23609339	0.063266325	3.731738625	0.000190163	0.001023454
ENSG00000165282	248.191722	0.147127182	0.125149158	1.175614637	0.239748904	0.401879815
ENSG00000165283	1141.721832	0.359602152	0.095218391	3.776603945	0.000158981	0.000873029
ENSG00000165288	1040.674841	-0.337809461	0.072052812	-4.688359122	2.75E-06	2.23E-05
ENSG00000165300	524.254856	1.440258216	0.095736001	15.04406077	3.78E-51	4.21E-49
ENSG00000165304	313.1386314	-0.298403712	0.113308987	-2.633539657	0.008449997	0.028054149
ENSG00000165322	195.4933616	-0.34876252	0.131600891	-2.650153181	0.008045528	0.026870075
ENSG00000165338	108.6726463	0.22592981	0.173680111	1.300838705	0.193313671	0.344079718
ENSG00000165349	17.85770147	-2.306998374	0.498259189	-4.630117068	3.65E-06	2.89E-05
ENSG00000165355	63.02918213	0.286212399	0.226985093	1.260930375	0.207333933	0.361342402
ENSG00000165359	208.4138402	-0.312343957	0.138693307	-2.252047796	0.024319252	0.067713088
ENSG00000165379	406.31115	5.371701094	0.199016454	26.99124116	1.87E-160	1.05E-157
ENSG00000165389	228.0142931	-0.321067	0.129844616	-2.472701678	0.013409603	0.041403627
ENSG00000165392	260.6889693	-0.098491556	0.121900921	-0.807964006	0.419111303	0.586964754
ENSG00000165406	69.70258361	0.242406927	0.214539091	1.129896311	0.258519919	0.424331538
ENSG00000165410	179.0307322	-0.055708638	0.143363157	-0.388584061	0.697583859	0.810233212
ENSG00000165416	732.894465	0.064287336	0.081186021	0.791852289	0.428446809	0.595563472
ENSG00000165417	661.4912489	0.11995665	0.082033424	1.46228993	0.143661777	0.276053455
ENSG00000165424	53.97557479	-1.268827151	0.254512167	-4.985330032	6.19E-07	5.74E-06
ENSG00000165434	8.862595691	-0.065694919	0.582388749	-0.112802521	0.910187119	0.951381168
ENSG00000165449	38.92886896	0.043971259	0.291743128	0.150719092	0.880197312	0.93402668
ENSG00000165458	840.5771559	0.286911135	0.080176159	3.578509347	0.00034556	0.001744188
ENSG00000165471	10.17424477	-4.791236265	1.094885976	-4.376013913	1.21E-05	8.51E-05
ENSG00000165475	35.08341467	-0.688605463	0.305045912	-2.257383024	0.023984152	0.066965887
ENSG00000165476	764.4091196	-1.122470702	0.086856739	-12.92324251	3.33E-38	2.45E-36
ENSG00000165480	256.2985513	0.017781723	0.118693665	0.149811892	0.880913027	0.934253325
ENSG00000165487	311.2498046	0.178349314	0.130596018	1.365656608	0.17204677	0.315295626
ENSG00000165490	82.63153766	0.132849874	0.195237385	0.680453051	0.49621764	0.654863696
ENSG00000165494	518.4043398	-0.177301848	0.090032506	-1.969309258	0.048917589	0.119878815
ENSG00000165495	10.44439138	-2.641168832	0.675480161	-3.910061291	9.23E-05	0.00053538
ENSG00000165501	83.13646607	0.15732149	0.206938512	0.760233021	0.44711531	0.612141365
ENSG00000165502	1787.150661	0.073430235	0.074493358	0.985728616	0.324266303	0.496196952
ENSG00000165506	210.3316986	0.324583415	0.135242097	2.400017607	0.016394283	0.049078207
ENSG00000165516	184.1738327	-0.327382722	0.134925975	-2.426387666	0.015249971	0.046147423
ENSG00000165521	386.6136712	-3.00731758	0.131953506	-22.79073643	5.67E-115	1.81E-112
ENSG00000165525	853.0569043	-0.068262928	0.0761035	-0.896974878	0.369732322	0.541059478
ENSG00000165526	87.20282915	-0.172931567	0.192769646	-0.897089195	0.369671324	0.541059478
ENSG00000165527	634.1435538	-0.088603278	0.089953975	-0.984984583	0.324631644	0.496634797
ENSG00000165533	290.0629342	-0.591624272	0.113040677	-5.233729044	1.66E-07	1.69E-06
ENSG00000165548	10.9545692	-1.204821045	0.55443827	-2.17304813	0.029776695	0.079968486
ENSG00000165568	16.92596346	5.222353208	0.911143072	5.731650021	9.95E-09	1.22E-07
ENSG00000165572	268.9933003	0.324847701	0.123800754	2.623955769	0.008691507	0.028667817
ENSG00000165609	880.7987086	0.305112566	0.085543587	3.56674975	0.000361436	0.001814545
ENSG00000165626	98.98650462	-0.102384721	0.181516363	-0.564052295	0.572718543	0.717660783
ENSG00000165629	610.0260532	0.01541027	0.091180634	0.169008151	0.86579023	0.924545891
ENSG00000165630	150.8369558	-0.184501743	0.148296874	-1.244137781	0.213448858	0.368932707
ENSG00000165632	241.6393624	-0.180321899	0.119746172	-1.50586775	0.132101127	0.259927795
ENSG00000165633	22.54712264	-0.067902202	0.364180206	-0.186452205	0.852090157	0.915154861
ENSG00000165637	301.2453383	0.057110827	0.117777383	0.484904872	0.627743826	0.758494295

ENSG00000165644	90.62501035	0.132396476	0.187609728	0.70570155	0.480373757	0.6408825
ENSG00000165650	537.8213131	-0.065584065	0.09173196	-0.71495328	0.474637912	0.635762254
ENSG00000165660	142.1904238	0.131111798	0.150948612	0.86858565	0.385073806	0.554120434
ENSG00000165661	527.2290652	0.300911434	0.088047634	3.417598182	0.000631763	0.002983986
ENSG00000165669	171.6839165	-0.073130657	0.138101126	-0.529544246	0.59642796	0.73589786
ENSG00000165671	1718.136182	-0.218068139	0.066543136	-3.277094401	0.001048813	0.004691935
ENSG00000165672	584.4272924	0.019319673	0.097545075	0.19805893	0.842998957	0.90893509
ENSG00000165675	42.97562039	0.253362659	0.267335073	0.947734449	0.343264659	0.515098671
ENSG00000165678	1694.098435	-0.067449991	0.076828325	-0.87793129	0.379981007	0.549474286
ENSG00000165684	449.2779691	0.690956447	0.104937602	6.584450518	4.57E-11	7.57E-10
ENSG00000165688	657.2944423	0.153957862	0.083809053	1.837007534	0.066208783	0.152289959
ENSG00000165689	426.085562	0.095119319	0.096150615	0.989274158	0.322529024	0.494364363
ENSG00000165698	23.46740922	-0.996513225	0.366099345	-2.721974889	0.006489307	0.022303719
ENSG00000165699	334.6675105	0.075848465	0.106570403	0.711721671	0.476637145	0.63753861
ENSG00000165704	214.2703545	0.255489621	0.132305576	1.931057095	0.053475992	0.128428323
ENSG00000165714	125.7301442	-0.160066222	0.164991806	-0.970146488	0.331973479	0.504440134
ENSG00000165716	340.6221812	-0.276352894	0.105782604	-2.612460688	0.008989303	0.029489196
ENSG00000165724	319.1211566	0.07652405	0.113173836	0.676163788	0.498936661	0.656970685
ENSG00000165730	57.31616286	-0.228462698	0.246567077	-0.926574223	0.354147643	0.525590274
ENSG00000165732	2981.886492	0.018782318	0.063195444	0.297210006	0.766306183	0.859082439
ENSG00000165733	1420.810498	0.120460865	0.06869438	1.753576714	0.079503064	0.175566663
ENSG00000165752	184.4395046	0.06119086	0.136466022	0.448396306	0.653867208	0.777851682
ENSG00000165757	15.56734504	-0.962967729	0.458657599	-2.099535104	0.035769757	0.093099458
ENSG00000165775	118.1013628	0.449389879	0.172981977	2.597900005	0.00937958	0.03059089
ENSG00000165782	139.579828	0.165225983	0.17588819	0.939380772	0.347535281	0.519343415
ENSG00000165792	177.1954374	-0.179182105	0.140118156	-1.278792914	0.20096999	0.353364056
ENSG00000165795	35.09331357	-0.559293666	0.297483042	-1.880085877	0.060096375	0.141024687
ENSG00000165801	567.8527322	-0.487493101	0.090347591	-5.395750952	6.82E-08	7.38E-07
ENSG00000165802	632.1922875	0.476314111	0.092590251	5.144322505	2.68E-07	2.64E-06
ENSG00000165804	239.9155556	-0.288189319	0.125949831	-2.288127869	0.022130076	0.062610508
ENSG00000165806	173.3622906	0.105605653	0.140339738	0.7525	0.45175043	0.616629899
ENSG00000165810	20.47072989	-2.122717669	0.455208502	-4.663176672	3.11E-06	2.49E-05
ENSG00000165813	136.1454209	0.142964967	0.164324924	0.870013893	0.384292812	0.553430504
ENSG00000165819	306.644719	0.150496689	0.111654917	1.347873363	0.177699117	0.323250388
ENSG00000165821	350.6414572	-0.326547679	0.103658376	-3.150229541	0.001631422	0.006869481
ENSG00000165832	176.3988198	0.005756045	0.139321906	0.041314718	0.967045	0.982799821
ENSG00000165861	74.23375025	-0.112348603	0.203079939	-0.553223543	0.580110348	0.723117407
ENSG00000165868	25.18266241	-1.394975205	0.367843091	-3.792310468	0.000149252	0.000824762
ENSG00000165879	37.01852838	0.157404297	0.29327052	0.536720491	0.591460699	0.732133279
ENSG00000165886	86.34144738	-0.180531351	0.194478226	-0.928285675	0.353259403	0.52460864
ENSG00000165891	144.6307284	-0.724144431	0.158074824	-4.581023157	4.63E-06	3.56E-05
ENSG00000165898	75.0393213	0.048808768	0.211999173	0.230230934	0.817912326	0.892592996
ENSG00000165905	91.9267616	-3.283762014	0.258815869	-12.68763782	6.92E-37	4.82E-35
ENSG00000165912	467.9814782	-0.031591615	0.101120799	-0.312414608	0.75472545	0.851673232
ENSG00000165914	236.8509749	0.142296929	0.121528366	1.17089477	0.241641078	0.404279683
ENSG00000165915	93.80069621	0.564099697	0.185279052	3.044595115	0.002329937	0.009305838
ENSG00000165916	794.9104827	0.595049955	0.085643129	6.948017457	3.70E-12	6.92E-11
ENSG00000165923	9.161822677	0.277653644	0.606177469	0.458040192	0.646923566	0.772538913
ENSG00000165934	1265.841972	0.012959874	0.071609972	0.180978617	0.85638436	0.917757792
ENSG00000165943	118.1573469	0.073356541	0.163293058	0.449232455	0.653263978	0.77746328

ENSG00000165948	40.17373605	0.316749032	0.283089677	1.118899975	0.263182814	0.429725559
ENSG00000165949	34.1242194	3.211028496	0.415692434	7.724529556	1.12E-14	2.64E-13
ENSG00000165959	25.52682918	0.226237585	0.354096644	0.638914797	0.522878361	0.676628438
ENSG00000165983	161.7356922	-0.105749388	0.162743966	-0.649789917	0.515827933	0.671096242
ENSG00000165995	21.77994667	1.692346011	0.394302315	4.292001209	1.77E-05	0.000120218
ENSG00000165996	26.44436238	-0.082805961	0.350460186	-0.236277797	0.813217114	0.890235667
ENSG00000165997	445.8438778	-0.082912985	0.094832685	-0.874308106	0.381950486	0.551414026
ENSG00000166004	686.7351116	-0.063319351	0.081551609	-0.776432885	0.437493434	0.603314879
ENSG00000166012	701.8517117	-0.054198533	0.084847685	-0.638774453	0.52296967	0.676628438
ENSG00000166016	66.74478654	0.0730881	0.231523838	0.315682827	0.752243262	0.850101457
ENSG00000166024	89.37421101	-0.332408928	0.196495701	-1.691685502	0.090705954	0.194585605
ENSG00000166025	693.057299	-0.20773302	0.081704073	-2.542505084	0.0110061	0.034981948
ENSG00000166033	85.66112946	1.353497719	0.214157809	6.320095089	2.61E-10	3.91E-09
ENSG00000166037	371.6114909	-0.202395163	0.100800898	-2.007870622	0.044657042	0.111539379
ENSG00000166046	17.42982789	0.381883642	0.4107207	0.92978913	0.352480276	0.523890639
ENSG00000166068	359.4832617	-0.080555587	0.101445652	-0.794076292	0.427151019	0.594372497
ENSG00000166073	131.9098669	0.8484923	0.170324289	4.981628304	6.31E-07	5.83E-06
ENSG00000166086	572.4276383	0.409967638	0.108988117	3.761581087	0.000168843	0.000918564
ENSG00000166105	13.9024594	0.802603698	0.48275937	1.662533653	0.096405822	0.203824891
ENSG00000166123	286.8558265	-0.113212128	0.131600807	-0.860269256	0.389640636	0.558099522
ENSG00000166128	135.4851044	-0.086849912	0.160204518	-0.542118994	0.587736535	0.729570925
ENSG00000166130	111.7763903	-0.164024194	0.17963375	-0.913103435	0.361188144	0.532159624
ENSG00000166133	84.15220685	-0.282064455	0.19772006	-1.426584915	0.153699575	0.290346682
ENSG00000166135	483.1446785	-0.238617154	0.104772375	-2.277481576	0.022757484	0.064088687
ENSG00000166136	31.6613166	0.083061567	0.319588907	0.259901283	0.794939922	0.87776105
ENSG00000166140	203.8227781	-0.587319812	0.137579003	-4.268964012	1.96E-05	0.000132053
ENSG00000166145	11.13643587	-0.799300715	0.540573688	-1.478615651	0.139243067	0.269823061
ENSG00000166147	747.0016146	-0.459871862	0.082268281	-5.589904852	2.27E-08	2.65E-07
ENSG00000166148	28.22079323	-0.085244943	0.326954188	-0.260724426	0.794305028	0.877449453
ENSG00000166153	15.82711321	-0.471569631	0.441052939	-1.069190544	0.284983819	0.454543852
ENSG00000166164	956.1335576	0.226749405	0.077443107	2.927948196	0.003412069	0.012908519
ENSG00000166165	378.8719013	-1.598429222	0.113692906	-14.05918175	6.77E-45	6.02E-43
ENSG00000166166	42.96924332	0.193512836	0.267884004	0.722375479	0.470063658	0.631985472
ENSG00000166167	171.4822327	-0.685032117	0.144453546	-4.742231241	2.11E-06	1.75E-05
ENSG00000166169	139.9398058	-0.021055964	0.151431785	-0.139045869	0.889413903	0.939574317
ENSG00000166170	448.4307193	0.148493317	0.097089949	1.529440666	0.126155238	0.25112853
ENSG00000166171	98.11460318	-0.026169289	0.184215038	-0.142058378	0.887033894	0.938194798
ENSG00000166173	120.7672813	0.144550831	0.167751436	0.861696533	0.388854534	0.557325353
ENSG00000166181	606.0722151	-0.104965409	0.085230746	-1.231543954	0.218119488	0.375127741
ENSG00000166188	100.1118798	0.220204477	0.201754576	1.09144725	0.275076131	0.443440701
ENSG00000166189	68.39100439	-0.057569449	0.212685662	-0.270678562	0.786638267	0.87229218
ENSG00000166192	19.67032701	-0.102527782	0.396519171	-0.258569547	0.795967383	0.878339214
ENSG00000166197	2987.356751	0.056178234	0.057735568	0.973026425	0.330540169	0.502826267
ENSG00000166199	42.49285871	-0.007395018	0.266635885	-0.027734518	0.977873893	0.988629033
ENSG00000166200	659.6773934	0.104328967	0.092200577	1.131543536	0.25782639	0.423501457
ENSG00000166224	168.3913463	-0.194421726	0.14239034	-1.365413737	0.172123049	0.315394269
ENSG00000166225	181.7339677	-0.09049308	0.137748746	-0.65694304	0.511217542	0.667447979
ENSG00000166226	1201.730628	0.327483475	0.072597234	4.510963534	6.45E-06	4.82E-05
ENSG00000166228	1089.707982	0.620956926	0.071495749	8.685228603	3.78E-18	1.15E-16
ENSG00000166233	456.4575145	-0.268172319	0.096166146	-2.788635409	0.005293062	0.018770925

ENSG00000166250	71.65183831	0.888141192	0.21345511	4.160786746	3.17E-05	0.000204266
ENSG00000166260	289.5068975	-0.015325737	0.120718779	-0.126954041	0.898976774	0.945345616
ENSG00000166261	71.22364536	0.27086108	0.21675752	1.249604077	0.211444213	0.366375183
ENSG00000166262	18.78548396	-0.415773738	0.400185714	-1.038951976	0.298827072	0.469270417
ENSG00000166263	203.9369403	-0.163741533	0.137986644	-1.186647697	0.235366614	0.396426238
ENSG00000166266	367.6380736	-0.237156072	0.105562065	-2.246603197	0.024665398	0.068517043
ENSG00000166272	138.3743628	-0.299717085	0.161789591	-1.852511548	0.063952401	0.148191913
ENSG00000166275	73.74626113	0.107231419	0.214128253	0.500781273	0.616525066	0.750779831
ENSG00000166278	14.77082762	-0.026564346	0.445480946	-0.059630711	0.952449758	0.973872598
ENSG00000166289	29.57534813	-0.060043882	0.321048958	-0.187024067	0.851641763	0.914883133
ENSG00000166295	280.058658	-0.4332984	0.120083766	-3.608301234	0.000308209	0.001576048
ENSG00000166311	130.5862748	0.444592373	0.159413728	2.788921497	0.005288388	0.018763827
ENSG00000166313	75.49940114	0.133781159	0.215251709	0.621510322	0.534263905	0.685370327
ENSG00000166326	927.9775438	-0.268847783	0.087451432	-3.074252478	0.002110308	0.008530501
ENSG00000166333	386.0917945	-0.12629088	0.105115985	-1.201443151	0.229579346	0.388542298
ENSG00000166337	40.35353636	-0.093434562	0.315560008	-0.296091265	0.767160385	0.859834192
ENSG00000166340	474.9747982	0.227597025	0.104245595	2.183277144	0.029015407	0.078395733
ENSG00000166341	29.81169913	-0.604386383	0.335682263	-1.800471605	0.071786203	0.162035604
ENSG00000166343	14.94148521	-0.1344745	0.45035959	-0.298593619	0.765250134	0.858571124
ENSG00000166347	26.41475488	-0.018504621	0.337314665	-0.054858632	0.956251089	0.976480012
ENSG00000166348	217.4647965	-0.273769382	0.130847456	-2.092278984	0.036413567	0.094529915
ENSG00000166352	36.02017075	-0.889432676	0.296105193	-3.003772636	0.002666545	0.010445332
ENSG00000166377	162.2773323	-0.134553721	0.146389821	-0.919146701	0.358018845	0.529316947
ENSG00000166387	35.6622008	-1.503956679	0.316283788	-4.755086203	1.98E-06	1.65E-05
ENSG00000166394	11.20361769	-5.78540161	1.251262787	-4.623650341	3.77E-06	2.97E-05
ENSG00000166398	165.4396606	-0.41140348	0.141526928	-2.906891893	0.003650393	0.013666598
ENSG00000166401	35.59348337	0.992880354	0.293502074	3.382873389	0.000717317	0.003343116
ENSG00000166402	338.8145368	0.870481019	0.104447252	8.334168677	7.80E-17	2.13E-15
ENSG00000166405	17.70710489	-1.827850349	0.45324683	-4.032792351	5.51E-05	0.00033488
ENSG00000166411	621.1985086	-0.010220266	0.089188852	-0.114591294	0.90876908	0.951016631
ENSG00000166426	478.0454932	1.982071988	0.108335474	18.29568756	8.96E-75	1.50E-72
ENSG00000166432	100.9611251	-0.407495704	0.17723137	-2.299230116	0.021491876	0.061211756
ENSG00000166435	143.3427615	-0.209688609	0.153134802	-1.369307346	0.170903223	0.313964485
ENSG00000166436	70.56719588	0.007376197	0.218956247	0.033687995	0.973125952	0.986326593
ENSG00000166439	489.1405532	-0.057660684	0.092131819	-0.625849834	0.531413442	0.683156819
ENSG00000166441	4873.072197	0.058721646	0.056320183	1.042639474	0.2971153	0.467627856
ENSG00000166444	267.3286939	-0.365044563	0.118827856	-3.072045354	0.002125974	0.008581483
ENSG00000166448	22.09226841	0.903326842	0.382782856	2.359893675	0.018280174	0.053675083
ENSG00000166450	201.4659436	-0.186859059	0.138410774	-1.350032612	0.177005522	0.322155555
ENSG00000166451	221.612592	-0.058645805	0.135230795	-0.433671967	0.664526666	0.785491334
ENSG00000166452	105.1366093	0.397608134	0.182417699	2.17965765	0.029282848	0.078905844
ENSG00000166454	249.9962459	-0.362166229	0.126564756	-2.861509311	0.004216291	0.015496139
ENSG00000166471	618.5530562	-0.055362714	0.093461276	-0.592359916	0.553609602	0.700906181
ENSG00000166477	684.7656648	-0.079831151	0.080718473	-0.989007201	0.322659619	0.494426426
ENSG00000166478	143.1334154	0.128141987	0.155186485	0.82572904	0.408957815	0.577621114
ENSG00000166479	288.3028632	0.054944389	0.111939585	0.490839669	0.623539851	0.755725995
ENSG00000166483	209.3931338	-0.278883137	0.130130884	-2.143097226	0.032105292	0.08500496
ENSG00000166484	135.0138921	0.020891724	0.156463678	0.133524435	0.893778641	0.942419199
ENSG00000166503	1129.665002	-0.307222239	0.07370266	-4.168400934	3.07E-05	0.000197834
ENSG00000166508	1694.996235	0.24390147	0.072464097	3.365825025	0.000763151	0.003528631

ENSG00000166526	168.1882961	-0.517433695	0.14205749	-3.642424592	0.000270082	0.00139891
ENSG00000166529	49.48034635	-0.358548169	0.247159408	-1.450675788	0.14687016	0.280754828
ENSG00000166532	227.2422542	-0.817060261	0.135405074	-6.034192367	1.60E-09	2.17E-08
ENSG00000166548	143.8970965	0.149610005	0.14852245	1.007322495	0.313779823	0.484998369
ENSG00000166557	508.1415334	0.609742645	0.091031909	6.698119912	2.11E-11	3.59E-10
ENSG00000166562	123.7856726	0.519007547	0.16357727	3.172858589	0.00150946	0.006423323
ENSG00000166575	293.5822905	-0.446191001	0.119357168	-3.738284085	0.000185281	0.000999476
ENSG00000166578	14.17844204	-1.70257636	0.498503213	-3.415376903	0.000636938	0.003005398
ENSG00000166579	271.8862915	0.302994153	0.118503606	2.556834877	0.010562934	0.033745176
ENSG00000166582	384.635715	0.148117209	0.106332615	1.392961221	0.163631518	0.304199567
ENSG00000166595	386.5377933	0.387127341	0.115618742	3.348309576	0.000813061	0.003734818
ENSG00000166598	4593.727996	0.573214859	0.05738633	9.988700483	1.71E-23	6.91E-22
ENSG00000166619	173.7677661	0.085988342	0.14850116	0.579041548	0.562561139	0.708853276
ENSG00000166669	115.4803491	-0.148965514	0.178636489	-0.833903057	0.404335615	0.572415298
ENSG00000166670	28.68219734	-1.458414019	0.365414293	-3.991124718	6.58E-05	0.000394255
ENSG00000166681	1264.803057	0.548493756	0.075486259	7.266140366	3.70E-13	7.68E-12
ENSG00000166685	502.9248335	0.346538357	0.100136577	3.460657123	0.000538859	0.002589582
ENSG00000166689	24.14379186	-0.790225846	0.379034	-2.084841589	0.037083682	0.0958268
ENSG00000166704	33.37729126	-0.446152992	0.298793056	-1.493183937	0.135389046	0.264434083
ENSG00000166710	1937.20014	1.036558541	0.080481592	12.87944884	5.88E-38	4.30E-36
ENSG00000166716	891.7853258	0.26272465	0.07423322	3.539178953	0.000401374	0.00199015
ENSG00000166734	700.2599898	0.089347487	0.082533737	1.082557143	0.279005039	0.447804363
ENSG00000166747	771.9599159	0.253050124	0.080677239	3.136573928	0.001709343	0.007156824
ENSG00000166750	21.29047578	-1.936782771	0.434793881	-4.45448488	8.41E-06	6.13E-05
ENSG00000166762	28.70404722	-0.379680662	0.343481578	-1.105388719	0.26899119	0.436217876
ENSG00000166770	44.62844551	-0.422179817	0.286179822	-1.475225661	0.140151885	0.271135315
ENSG00000166780	46.02781378	-0.477575243	0.262852598	-1.816893756	0.069233383	0.157734668
ENSG00000166783	324.1003553	0.022003579	0.112029867	0.196408152	0.844290716	0.90976923
ENSG00000166788	149.6681006	0.118719193	0.148078557	0.80173116	0.422708487	0.590003496
ENSG00000166794	2793.070845	0.836852134	0.068033212	12.30064132	8.99E-35	5.76E-33
ENSG00000166797	207.02609	-0.123070294	0.136412293	-0.902193575	0.366954047	0.538227368
ENSG00000166801	340.5553384	0.036249317	0.11183002	0.32414657	0.745827071	0.845033294
ENSG00000166803	45.54850496	-0.449956342	0.269567963	-1.669175881	0.095082529	0.201816905
ENSG00000166813	211.2498165	0.241088411	0.130251981	1.850938539	0.064178396	0.148642032
ENSG00000166816	21.60395737	0.398094365	0.38282015	1.039899194	0.298386737	0.468998377
ENSG00000166821	17.46929289	1.082324158	0.423855916	2.553519054	0.010664044	0.034029494
ENSG00000166822	167.7020589	-0.131970279	0.14734357	-0.895663645	0.370432432	0.541536387
ENSG00000166823	49.056999	0.273623832	0.262752741	1.041373843	0.297702077	0.468341481
ENSG00000166831	979.0657027	0.183319715	0.089225706	2.054561668	0.039921361	0.101681843
ENSG00000166839	23.0196221	0.23078008	0.371497475	0.621215742	0.534457683	0.685493706
ENSG00000166845	109.3753539	-0.153105578	0.176862299	-0.86567674	0.386667459	0.555483514
ENSG00000166847	334.2590362	0.243118705	0.106003569	2.29349547	0.021819496	0.061967519
ENSG00000166848	686.4984089	0.33212735	0.080153858	4.143622782	3.42E-05	0.000218577
ENSG00000166851	824.6070574	0.199130978	0.091549583	2.175116158	0.029621413	0.07968058
ENSG00000166855	510.32851	0.164735407	0.093519875	1.761501581	0.078153546	0.173384329
ENSG00000166860	273.8685517	-0.158231899	0.118399157	-1.336427576	0.181409558	0.328045837
ENSG00000166881	410.2702831	-0.272120557	0.106672882	-2.550981585	0.010742	0.034239364
ENSG00000166886	392.9587408	0.250098617	0.126528111	1.976624918	0.048084043	0.118352297
ENSG00000166887	283.6727074	-0.266123296	0.115125851	-2.311585922	0.020800513	0.059653901
ENSG00000166888	650.5773197	0.182833824	0.082824335	2.207489178	0.027279902	0.07456719

ENSG00000166889	547.9665134	0.069218809	0.103155547	0.671013932	0.502211654	0.660354498
ENSG00000166896	33.53597936	-0.055586219	0.301569483	-0.184323091	0.85376	0.916177748
ENSG00000166897	5.611204109	-2.852808913	0.940083701	-3.034632886	0.002408286	0.009575205
ENSG00000166900	150.9161873	-0.329855411	0.150346661	-2.193965658	0.028237879	0.07670808
ENSG00000166902	271.7666283	-0.375373269	0.115512548	-3.24963196	0.001155545	0.00509317
ENSG00000166908	163.5742862	-0.161236419	0.145936509	-1.104839499	0.269229142	0.436452597
ENSG00000166912	222.487803	-0.389560897	0.127670536	-3.051298357	0.00227854	0.009139536
ENSG00000166913	1469.657192	-0.007197012	0.072974946	-0.098623047	0.92143757	0.956727767
ENSG00000166924	21.41827627	-1.007987367	0.396257251	-2.54377015	0.010966322	0.034875238
ENSG00000166925	225.5208523	0.319798603	0.134744454	2.373371173	0.017626541	0.05212632
ENSG00000166938	237.8411589	-0.145658355	0.119930468	-1.214523366	0.224547933	0.382778461
ENSG00000166946	51.55831413	-0.245181702	0.240679067	-1.018708047	0.308341591	0.479653857
ENSG00000166949	322.2898712	-0.227845594	0.109252598	-2.085493594	0.037024519	0.095691521
ENSG00000166963	1221.633039	-0.067323078	0.075808875	-0.888063285	0.37450671	0.54453973
ENSG00000166965	220.5087811	0.484579709	0.134422072	3.604911779	0.000312259	0.001592702
ENSG00000166971	43.87635171	0.217233661	0.265635198	0.817789443	0.413477427	0.581859088
ENSG00000166974	308.8072313	0.09387802	0.122269872	0.767793553	0.442609845	0.607577494
ENSG00000166986	909.2685597	-0.480174499	0.074692068	-6.428721467	1.29E-10	2.01E-09
ENSG00000166987	1160.681719	0.268915416	0.092012561	2.922594635	0.003471281	0.013104296
ENSG00000166997	68.95664793	-0.120154847	0.21347069	-0.562863438	0.573527878	0.718098414
ENSG00000167004	1984.455572	0.341375665	0.067100497	5.087528077	3.63E-07	3.51E-06
ENSG00000167005	261.8241237	0.071100357	0.116434384	0.610647424	0.541433019	0.691142874
ENSG00000167037	6.482527006	-2.107341906	0.814298695	-2.587922491	0.00965567	0.031353029
ENSG00000167065	15.03547585	-0.531866379	0.445374656	-1.19419992	0.232399774	0.392322993
ENSG00000167074	48.51017627	-0.611932471	0.257721093	-2.374398095	0.017577588	0.052014403
ENSG00000167081	1516.480324	0.298898873	0.064558716	4.62987634	3.66E-06	2.89E-05
ENSG00000167085	1846.238031	0.253087079	0.06704488	3.774890446	0.000160078	0.000877638
ENSG00000167088	2347.951009	0.123047328	0.068241472	1.803116557	0.071369911	0.16145361
ENSG00000167100	48.78750782	-0.074424946	0.260610981	-0.285578705	0.775200816	0.865324687
ENSG00000167103	16.49453934	-0.362146764	0.435453811	-0.831653678	0.405604454	0.573801237
ENSG00000167106	161.0209841	0.26432872	0.141793437	1.864181626	0.062296203	0.145120192
ENSG00000167107	148.9881972	-1.384823164	0.165451843	-8.369947037	5.76E-17	1.60E-15
ENSG00000167110	768.890614	0.446793482	0.095381837	4.684261654	2.81E-06	2.27E-05
ENSG00000167112	291.2884225	0.140761699	0.116394006	1.209355224	0.226526403	0.384905816
ENSG00000167113	189.2019564	-0.00168751	0.132951441	-0.012692679	0.989872979	0.994759318
ENSG00000167114	180.0100481	0.197140256	0.142662015	1.38186928	0.167011842	0.308930122
ENSG00000167118	176.2414445	0.197607238	0.137031619	1.442055776	0.149286639	0.283979396
ENSG00000167123	390.707458	0.352393699	0.110235352	3.19673946	0.001389904	0.00598528
ENSG00000167130	103.3570747	0.059906864	0.195866076	0.305856251	0.759714098	0.855077995
ENSG00000167136	154.928105	0.115357783	0.145575798	0.792424182	0.428113384	0.595302146
ENSG00000167157	311.0108067	4.887567047	0.198832706	24.58130324	2.00E-133	8.27E-131
ENSG00000167173	441.8716843	0.33696517	0.104654521	3.21978609	0.001282863	0.005573943
ENSG00000167182	391.0138842	0.168319136	0.10105661	1.66559255	0.095794596	0.203021323
ENSG00000167186	115.902305	0.152591615	0.167143285	0.912938951	0.36127465	0.532231254
ENSG00000167191	81.04921819	0.578991794	0.218229895	2.653127768	0.007974968	0.026696053
ENSG00000167193	458.673251	0.089517529	0.10144803	0.882397901	0.37756167	0.547045774
ENSG00000167196	463.8828358	-0.036190804	0.101759351	-0.355650894	0.722102024	0.827735087
ENSG00000167202	203.5146315	-0.013181296	0.138828179	-0.094946831	0.924357058	0.958686031
ENSG00000167216	79.00859006	-0.88661662	0.20575048	-4.309183715	1.64E-05	0.00011206
ENSG00000167220	82.3246509	-0.232773811	0.201917919	-1.152814035	0.248986722	0.413125028

ENSG00000167232	90.46890245	-2.671389211	0.236229394	-11.3084539	1.19E-29	6.41E-28
ENSG00000167257	311.0552694	0.323919587	0.107667038	3.008530675	0.002625143	0.010303264
ENSG00000167258	1214.937348	-0.074020017	0.067653596	-1.09410322	0.27390971	0.442203161
ENSG00000167264	91.11262407	0.424153263	0.193371433	2.193463923	0.028273971	0.076761591
ENSG00000167272	121.4302847	0.040504006	0.162752208	0.248869162	0.80346199	0.883393126
ENSG00000167280	110.8710116	-0.150439063	0.181114887	-0.830627816	0.406183916	0.57446427
ENSG00000167283	54.00178852	0.037105062	0.250948326	0.147859371	0.882453753	0.935251839
ENSG00000167291	963.4076667	-0.226991048	0.079198078	-2.866118133	0.004155391	0.015311554
ENSG00000167302	100.4774783	0.156757379	0.207543064	0.755300498	0.4500687	0.615051564
ENSG00000167311	38.59195118	-3.179886255	0.392228859	-8.107221535	5.18E-16	1.34E-14
ENSG00000167315	118.3822836	-0.315212175	0.164570852	-1.915358467	0.055446804	0.132279425
ENSG00000167323	206.3182182	-0.392638755	0.133980836	-2.930559084	0.003383526	0.012817798
ENSG00000167325	645.592112	0.07303048	0.090623379	0.80586799	0.42031897	0.587952656
ENSG00000167333	31.78853976	0.004435672	0.304940714	0.014546015	0.988394368	0.994126857
ENSG00000167363	249.1120172	-0.354656365	0.123874047	-2.863040115	0.004195974	0.015433159
ENSG00000167371	257.6705152	-0.336411154	0.127009451	-2.648709605	0.008079973	0.026972285
ENSG00000167377	10.07203768	0.018226789	0.55578396	0.032794738	0.973838275	0.986408007
ENSG00000167378	285.9132042	-0.1030168	0.112086685	-0.919081512	0.358052939	0.529316947
ENSG00000167380	104.5160344	0.210855455	0.178425068	1.181759137	0.237301286	0.398728608
ENSG00000167384	89.10524281	-0.089671437	0.195236905	-0.459295527	0.646021959	0.772115244
ENSG00000167393	143.6520883	0.536867238	0.158078523	3.396206056	0.000683269	0.00320036
ENSG00000167394	153.9945547	0.465914932	0.148753511	3.132127305	0.001735446	0.007246674
ENSG00000167395	452.3177813	-0.331706268	0.109213344	-3.037232032	0.002387616	0.009501088
ENSG00000167397	75.97027938	0.548428449	0.219521414	2.498291348	0.012479358	0.038946355
ENSG00000167447	188.2776476	-0.005508401	0.134325059	-0.041007993	0.967289524	0.982880628
ENSG00000167460	3039.129365	-0.074030098	0.060205375	-1.229626071	0.218837162	0.375933181
ENSG00000167461	654.0909508	0.053040235	0.084966435	0.624249265	0.532463897	0.683998204
ENSG00000167468	1664.285877	-0.086441934	0.090552217	-0.954608691	0.33977562	0.511611978
ENSG00000167470	696.5368198	-0.080725141	0.096392108	-0.837466294	0.402330516	0.570658715
ENSG00000167487	41.92791685	0.163119314	0.280081652	0.582399141	0.56029786	0.706952666
ENSG00000167491	981.6409646	0.013057362	0.071728124	0.182039647	0.855551612	0.917119467
ENSG00000167508	251.7274974	-0.14400666	0.139099116	-1.035280913	0.300537741	0.4708514
ENSG00000167513	544.0827564	-0.690874402	0.09192091	-7.515965661	5.65E-14	1.26E-12
ENSG00000167515	138.9883774	-0.708672703	0.16865524	-4.201901475	2.65E-05	0.000173032
ENSG00000167522	2570.100368	-1.054859812	0.063380312	-16.64333585	3.38E-62	4.66E-60
ENSG00000167523	98.36511846	-0.227497749	0.183858082	-1.23735517	0.215955254	0.372276561
ENSG00000167524	66.13821083	0.287745039	0.213496946	1.34777122	0.177731977	0.3232683
ENSG00000167525	47.97471857	-0.700120495	0.277405844	-2.523813072	0.011608964	0.036612285
ENSG00000167526	5069.225728	0.169439042	0.058405243	2.901092979	0.003718635	0.013874016
ENSG00000167528	247.3567375	-0.659764421	0.130629385	-5.05065854	4.40E-07	4.19E-06
ENSG00000167535	80.14855618	0.260723312	0.197752022	1.318435629	0.18735786	0.335647987
ENSG00000167536	60.92587924	0.240146491	0.226566726	1.059937157	0.28917319	0.459348163
ENSG00000167543	105.9045757	0.173867273	0.365150388	0.476152507	0.633965732	0.763356475
ENSG00000167548	3328.496006	0.031611678	0.061772689	0.511741982	0.6088316	0.745198811
ENSG00000167549	29.00617162	-0.569739862	0.330741256	-1.722615042	0.084958163	0.18505308
ENSG00000167550	17.67094096	-0.049816939	0.427409496	-0.116555526	0.907212282	0.950448309
ENSG00000167552	725.217753	0.951435404	0.08198801	11.60456759	3.91E-31	2.22E-29
ENSG00000167553	790.4164535	0.069475529	0.076817059	0.904428384	0.365768293	0.537105081
ENSG00000167565	199.9549062	-0.306871707	0.131619082	-2.331513806	0.019726283	0.057111426
ENSG00000167566	208.5724824	-0.313624177	0.136380649	-2.299623736	0.021469546	0.061163822

ENSG00000167595	249.907934	-0.108794794	0.124697629	-0.872468822	0.38295267	0.55240667
ENSG00000167600	11.05088503	0.11186408	0.516411378	0.216618154	0.828505937	0.899731186
ENSG00000167601	316.4393186	-0.282498385	0.113828956	-2.48177965	0.013072809	0.040546201
ENSG00000167604	52.7029426	0.091917611	0.244056354	0.376624535	0.706452652	0.815678152
ENSG00000167613	217.4874498	-0.024271484	0.128721602	-0.188557967	0.850439279	0.914361666
ENSG00000167615	1258.402894	0.27358694	0.073955052	3.699367838	0.000216137	0.00114829
ENSG00000167617	87.05324122	-0.644642072	0.20191668	-3.192614256	0.001409912	0.006056583
ENSG00000167619	52.48833915	0.18072821	0.253574204	0.71272317	0.476017076	0.637002178
ENSG00000167625	101.7039352	-0.070170545	0.177312183	-0.395745759	0.692292595	0.806274442
ENSG00000167632	564.3570229	-0.162167908	0.08731469	-1.857280922	0.063271197	0.146952795
ENSG00000167635	1251.005728	0.127758804	0.074313437	1.719188468	0.08558006	0.186090263
ENSG00000167637	78.83139579	0.197048704	0.203244147	0.96951724	0.332287183	0.504664078
ENSG00000167641	97.28529518	-0.145783531	0.186132901	-0.783222796	0.43349629	0.599755973
ENSG00000167645	140.3276318	-0.347047394	0.158900926	-2.184048914	0.028958654	0.078272506
ENSG00000167646	122.567005	0.71413209	0.172194675	4.147236789	3.37E-05	0.000215549
ENSG00000167654	15.17974811	-3.934117313	0.735179985	-5.351230167	8.74E-08	9.30E-07
ENSG00000167657	268.9316813	0.089106227	0.131516032	0.677531288	0.498068926	0.6560741
ENSG00000167658	27401.2865	0.071597173	0.057586458	1.24329877	0.213757757	0.369330285
ENSG00000167670	1282.825092	-0.208298534	0.07048205	-2.955341608	0.00312323	0.011950971
ENSG00000167671	303.6516395	0.36362156	0.116209776	3.129010088	0.001753963	0.007313124
ENSG00000167674	1135.384359	0.025149487	0.070880586	0.354814882	0.722728278	0.828072115
ENSG00000167676	16.47451803	-0.403809795	0.456796437	-0.884003821	0.37669415	0.546352372
ENSG00000167680	166.1367743	-3.079693318	0.191486311	-16.08309907	3.35E-58	4.24E-56
ENSG00000167685	150.7572774	0.316159154	0.14677025	2.154109249	0.031231605	0.08322857
ENSG00000167693	335.2382022	0.145612364	0.105841805	1.375754738	0.168897579	0.311407528
ENSG00000167695	207.2990296	0.188374661	0.131547795	1.431986453	0.152147698	0.288402768
ENSG00000167699	347.2336457	0.199321868	0.107929598	1.846776713	0.064779508	0.149721576
ENSG00000167700	65.8243547	0.4994491	0.234516848	2.129693897	0.033196893	0.087489073
ENSG00000167702	155.5046149	-0.050701127	0.175197445	-0.289394213	0.772279723	0.863504996
ENSG00000167703	82.26980441	-0.399902874	0.197384447	-2.026010054	0.042763754	0.107923546
ENSG00000167705	90.45204073	-0.195647474	0.188600923	-1.037362228	0.299567075	0.470012141
ENSG00000167716	298.4401369	0.57710896	0.11752974	4.910322777	9.09E-07	8.13E-06
ENSG00000167720	44.27659539	-0.472515907	0.264733588	-1.784873275	0.074281874	0.166231877
ENSG00000167721	933.0516699	0.227195435	0.075450609	3.011180934	0.002602337	0.010230908
ENSG00000167723	21.66699893	0.509072006	0.376862973	1.350814601	0.17675483	0.32182438
ENSG00000167733	37.53710617	0.331058911	0.287487145	1.151560748	0.249501619	0.413686171
ENSG00000167740	112.0263915	-0.106611621	0.173757809	-0.613564484	0.539503159	0.689580557
ENSG00000167747	278.0591437	-0.098238667	0.11271116	-0.871596627	0.383428471	0.552582306
ENSG00000167748	30.01886695	-0.310700479	0.332035902	-0.935743626	0.349405203	0.521472045
ENSG00000167766	158.868428	0.223205416	0.151456155	1.473729579	0.140554415	0.271652157
ENSG00000167770	562.2278441	-0.022671632	0.087638022	-0.258696298	0.795869576	0.878339214
ENSG00000167771	526.8601252	-0.387280136	0.092191248	-4.200834086	2.66E-05	0.000173667
ENSG00000167772	32.92408385	0.563508943	0.314377694	1.792458412	0.073059565	0.164179315
ENSG00000167775	254.4677354	0.585592904	0.122402514	4.784157486	1.72E-06	1.45E-05
ENSG00000167778	118.4081824	0.730873785	0.168654102	4.333566617	1.47E-05	0.000101471
ENSG00000167779	72.7921162	-1.527207481	0.23637942	-6.460830989	1.04E-10	1.64E-09
ENSG00000167785	25.86128534	-0.30904046	0.353139292	-0.875123406	0.381506763	0.550886667
ENSG00000167792	965.6706134	-0.022563686	0.073800934	-0.305737141	0.759804792	0.855077995
ENSG00000167797	151.1160543	-0.068765078	0.153203597	-0.448847675	0.653541544	0.777595974
ENSG00000167799	34.16586442	0.44096159	0.303004306	1.455298095	0.145586754	0.278876767

ENSG00000167815	1329.279736	0.269017901	0.074430346	3.614357771	0.000301093	0.001543596
ENSG00000167840	55.36449444	-0.065306072	0.234796172	-0.278139423	0.780905334	0.868723943
ENSG00000167842	87.88338513	-0.415083255	0.188523064	-2.201763781	0.027681999	0.075460709
ENSG00000167861	49.82935712	0.417221807	0.273496727	1.525509322	0.12713211	0.252311531
ENSG00000167862	254.7179929	0.058323395	0.117905444	0.494662447	0.620838414	0.753970589
ENSG00000167863	1115.057399	0.11027765	0.075250253	1.465478807	0.14279033	0.274914442
ENSG00000167874	220.0465777	0.011884201	0.126606619	0.093867139	0.925214697	0.959079836
ENSG00000167880	17.86003639	5.871728335	1.077452709	5.449639031	5.05E-08	5.58E-07
ENSG00000167881	1132.095379	0.264846401	0.071605254	3.698700698	0.000216706	0.001150877
ENSG00000167889	15.1888246	-1.832664524	0.489660821	-3.742722401	0.000182037	0.000984628
ENSG00000167900	389.1913276	0.406049045	0.114580731	3.543781239	0.000394432	0.001959191
ENSG00000167904	64.47486569	-0.06560813	0.242955249	-0.270042038	0.787127913	0.872399068
ENSG00000167920	22.13508547	-0.095315083	0.365285523	-0.260933097	0.794144101	0.877406181
ENSG00000167925	83.69197148	0.064891886	0.198757882	0.326487108	0.744055841	0.843563552
ENSG00000167930	504.020546	-0.156576679	0.093996244	-1.665775907	0.095758057	0.202997185
ENSG00000167962	647.4002263	0.423044271	0.085412409	4.952960319	7.31E-07	6.67E-06
ENSG00000167964	37.88842873	-0.308014384	0.295281347	-1.043121712	0.296891926	0.467381025
ENSG00000167965	508.0846076	-0.248513579	0.090109068	-2.757919773	0.005817047	0.020316276
ENSG00000167967	313.7282373	0.496035564	0.109321183	4.537414914	5.69E-06	4.32E-05
ENSG00000167969	172.0299217	-0.190422345	0.148940781	-1.278510456	0.201069501	0.353410009
ENSG00000167971	163.9970624	0.754193663	0.150863329	4.999184797	5.76E-07	5.37E-06
ENSG00000167972	1141.182187	0.298480199	0.083819124	3.56100358	0.00036944	0.001852079
ENSG00000167977	234.9546908	-0.260248476	0.131239585	-1.983002895	0.047367106	0.116812806
ENSG00000167978	14458.15586	0.119566867	0.048913341	2.44446332	0.014506783	0.044241594
ENSG00000167981	42.07538829	-0.16162669	0.266394278	-0.606719827	0.544036862	0.692782685
ENSG00000167985	51.71057787	0.121918536	0.254152856	0.479705551	0.63143678	0.761420933
ENSG00000167986	1642.265985	0.180073959	0.068887358	2.614034922	0.008947989	0.029367384
ENSG00000167987	214.2731758	0.094671117	0.142838342	0.662785044	0.507468234	0.664432322
ENSG00000167992	67.12306435	-0.413017013	0.216298648	-1.909475701	0.056200753	0.133653282
ENSG00000167994	20.00979468	-0.225399119	0.416272967	-0.541469509	0.588184008	0.72968478
ENSG00000167996	1400.227684	-0.231654455	0.071221952	-3.252571005	0.00114366	0.005054761
ENSG00000168000	76.1036723	0.042825181	0.201630206	0.212394667	0.831799146	0.901565015
ENSG00000168002	161.4477972	0.112610937	0.143670129	0.78381594	0.433148119	0.599418388
ENSG00000168003	459.199714	-0.382880571	0.094611679	-4.046863714	5.19E-05	0.000317625
ENSG00000168005	219.1820068	-0.34324431	0.133203637	-2.57683887	0.009970842	0.032212607
ENSG00000168010	215.9055651	0.51476482	0.133844021	3.846005336	0.000120059	0.000680029
ENSG00000168014	246.1849092	-0.070581692	0.127874242	-0.551961765	0.580974549	0.723616731
ENSG00000168016	18.79358165	2.167095001	0.449821053	4.817682471	1.45E-06	1.24E-05
ENSG00000168026	32.51724072	0.133521357	0.322558964	0.41394403	0.678915097	0.796385835
ENSG00000168028	3693.613912	-0.059844506	0.061909628	-0.966642957	0.333722552	0.505859576
ENSG00000168036	1750.849265	0.053272217	0.074876071	0.711471858	0.476791884	0.63753861
ENSG00000168038	96.30388746	-0.36261233	0.181892201	-1.993556224	0.046200573	0.114580006
ENSG00000168040	188.1585437	0.495226444	0.149192397	3.319381253	0.000902172	0.004101231
ENSG00000168056	676.7088603	0.085192718	0.089711719	0.94962753	0.342301545	0.514366687
ENSG00000168061	127.406088	-0.134791108	0.163725529	-0.823274837	0.410351719	0.578821309
ENSG00000168062	23.95858467	-0.880469485	0.367175368	-2.39795357	0.016486955	0.04930642
ENSG00000168066	2498.493079	0.013587811	0.058979509	0.23038189	0.817795033	0.892584835
ENSG00000168067	147.6850856	0.216158477	0.157781539	1.369985857	0.170691317	0.313791373
ENSG00000168071	97.56444994	-0.483854393	0.194304957	-2.490180382	0.012767828	0.039731873
ENSG00000168077	190.2741039	-0.048241721	0.138200027	-0.349071719	0.727035468	0.831311943

ENSG00000168078	199.36062	-0.423529906	0.133498413	-3.172546365	0.001511084	0.006428286
ENSG00000168090	1125.962236	0.177191775	0.073611592	2.407117828	0.016078981	0.048302277
ENSG00000168092	1023.202733	0.09320684	0.07658031	1.217112328	0.223561481	0.381946986
ENSG00000168096	84.14823736	0.16982801	0.196972026	0.862193545	0.388581021	0.557149904
ENSG00000168101	61.8358833	0.738627144	0.237780723	3.106337363	0.001894205	0.00782125
ENSG00000168116	126.4033302	-0.203974876	0.161149235	-1.265751436	0.205602087	0.359126152
ENSG00000168118	312.868037	-0.154582168	0.116359043	-1.328492954	0.18401531	0.331393173
ENSG00000168124	13.29901289	-0.068858534	0.467822584	-0.147189419	0.882982512	0.935529895
ENSG00000168135	22.99989074	-2.048041488	0.45237807	-4.527278449	5.97E-06	4.51E-05
ENSG00000168137	3039.921595	-0.091965799	0.063726721	-1.443127754	0.148984486	0.283575214
ENSG00000168140	13.056674	-0.09313685	0.500497014	-0.186088723	0.852375185	0.915251049
ENSG00000168148	6.070021872	-0.472049608	0.746616695	-0.63225161	0.527222478	0.680224298
ENSG00000168152	26.39797081	-0.225290843	0.341478964	-0.659750284	0.509414092	0.665836024
ENSG00000168159	559.8233747	0.030087674	0.090260822	0.33334146	0.738876546	0.840132657
ENSG00000168172	523.4969324	-0.282174487	0.088677524	-3.182029397	0.00146247	0.006257482
ENSG00000168175	1588.597131	0.338401806	0.068840566	4.91573246	8.85E-07	7.91E-06
ENSG00000168209	410.3736648	0.135834563	0.102692231	1.322734567	0.185923664	0.334008612
ENSG00000168214	815.4913638	0.224968004	0.08705637	2.584164768	0.009761515	0.031608355
ENSG00000168216	25.27219025	1.140842392	0.359552926	3.17294704	0.001509	0.006423312
ENSG00000168228	52.20995987	0.171318735	0.252200717	0.679295194	0.496950841	0.655435794
ENSG00000168234	109.5962522	0.417447452	0.170291154	2.451374856	0.014231166	0.043495473
ENSG00000168237	60.9889204	0.261672777	0.22735999	1.150918317	0.249765842	0.413789489
ENSG00000168243	24.74663598	-1.730324316	0.385157363	-4.492512627	7.04E-06	5.22E-05
ENSG00000168246	180.2547372	-0.216321813	0.150825221	-1.434254904	0.151499534	0.287449798
ENSG00000168256	209.9509702	-0.173064003	0.134657451	-1.285216682	0.198716576	0.350564914
ENSG00000168259	947.1339508	0.141816122	0.08121962	1.746082059	0.080796694	0.177919918
ENSG00000168264	889.1488738	0.266165458	0.086890563	3.063226316	0.002189644	0.008818213
ENSG00000168268	1384.791727	-0.282353181	0.071077239	-3.972483834	7.11E-05	0.000422998
ENSG00000168273	84.6855896	-0.096169977	0.197429583	-0.487110265	0.626180192	0.757381067
ENSG00000168275	243.6420955	0.484370437	0.125884596	3.847733983	0.000119215	0.000676067
ENSG00000168280	22.17755013	-0.84235646	0.377635612	-2.230606526	0.025707204	0.070958753
ENSG00000168282	361.5122654	0.127604246	0.109265319	1.167838498	0.242871916	0.405759015
ENSG00000168283	190.3211835	0.651083794	0.150933348	4.313717308	1.61E-05	0.00011
ENSG00000168286	239.0612999	-0.013386977	0.128204925	-0.104418585	0.916837174	0.955047222
ENSG00000168288	121.6333651	-0.20711146	0.162491572	-1.274598168	0.202451506	0.355080074
ENSG00000168291	616.5909811	0.218887457	0.089810788	2.437206738	0.014801214	0.044993001
ENSG00000168297	131.2150112	0.406164533	0.166622396	2.437634693	0.014783705	0.044959236
ENSG00000168298	5177.642526	0.057458729	0.074219522	0.774172713	0.438828663	0.604227552
ENSG00000168300	188.0825183	0.083007777	0.143008512	0.580439413	0.561618333	0.707982834
ENSG00000168301	46.02560701	-0.025813535	0.262092112	-0.098490317	0.92154296	0.956727767
ENSG00000168303	80.84996806	-0.470064907	0.206444989	-2.276949951	0.022789215	0.064152324
ENSG00000168310	193.5539517	0.089481934	0.138906462	0.64418842	0.519453259	0.674158727
ENSG00000168374	608.1035025	0.256911552	0.088743173	2.895000761	0.003791576	0.01411616
ENSG00000168385	1858.653561	-0.192736508	0.06586597	-2.926192504	0.003431385	0.012974608
ENSG00000168386	37.70640522	-3.094484458	0.395095319	-7.832247833	4.79E-15	1.15E-13
ENSG00000168389	59.17141884	0.355310209	0.230962127	1.53839166	0.12395288	0.248040784
ENSG00000168393	366.3306871	0.229627938	0.100401249	2.287102401	0.022189847	0.062691226
ENSG00000168394	89.27748754	0.550696126	0.196397691	2.803984724	0.00504753	0.018045978
ENSG00000168395	296.2973364	-0.202920123	0.110601584	-1.834694545	0.066550961	0.152876754
ENSG00000168397	218.8587157	-0.238123323	0.125794608	-1.892953339	0.058364075	0.137626349

ENSG00000168404	65.64069838	0.613113598	0.225938289	2.713633001	0.006654985	0.022778404
ENSG00000168405	23.52250334	0.718165941	0.368148305	1.950751727	0.051086587	0.123851999
ENSG00000168411	624.6584889	-0.23318791	0.086274386	-2.702863747	0.006874492	0.023432659
ENSG00000168434	336.8186231	0.209467372	0.109523149	1.912539713	0.055807004	0.132958104
ENSG00000168438	202.5073398	-0.010365179	0.133721369	-0.07751326	0.938215243	0.966102042
ENSG00000168439	2610.256019	0.303469765	0.059794775	5.075188683	3.87E-07	3.73E-06
ENSG00000168453	8.715915438	0.845512293	0.58998599	1.433105713	0.151827629	0.28797228
ENSG00000168461	210.5065813	0.173218413	0.141082834	1.227778094	0.219530281	0.376893407
ENSG00000168476	190.6947833	-0.085866379	0.135992228	-0.631406517	0.527774756	0.680568444
ENSG00000168477	40.67989096	-0.477623763	0.292721674	-1.631665179	0.102750039	0.214239246
ENSG00000168487	260.1544357	0.320104996	0.123972239	2.582069977	0.009820967	0.031757625
ENSG00000168488	3463.982775	0.119106952	0.057508943	2.07110315	0.038349158	0.098426935
ENSG00000168490	20.59808432	0.121197417	0.406579778	0.298090125	0.765634379	0.858740507
ENSG00000168495	356.2827196	0.113802314	0.104269638	1.091423318	0.275086657	0.443440701
ENSG00000168496	605.7322544	-0.028816592	0.091201686	-0.315965566	0.752028644	0.849927297
ENSG00000168502	130.1122099	0.179838695	0.156684645	1.147774852	0.251061521	0.41534109
ENSG00000168517	41.87502608	-0.098330307	0.278775903	-0.352721689	0.724297098	0.829328442
ENSG00000168522	113.9263294	-0.005855016	0.166020007	-0.035266932	0.971866891	0.985302874
ENSG00000168528	11.06873087	-1.034973596	0.536411692	-1.929438921	0.053676396	0.12879951
ENSG00000168538	150.4485589	-0.168724572	0.152675477	-1.105119011	0.269108024	0.436341904
ENSG00000168542	8176.034926	-0.226234284	0.058547598	-3.864108759	0.000111496	0.000635653
ENSG00000168556	271.7956561	0.312244455	0.116671195	2.676277154	0.007444505	0.025143777
ENSG00000168564	90.69512966	-0.147334369	0.200024987	-0.736579823	0.461377909	0.624585702
ENSG00000168566	126.814762	-0.440623691	0.160794453	-2.740291624	0.006138469	0.021253901
ENSG00000168569	197.814175	0.002272621	0.133474324	0.017026651	0.986415354	0.992847269
ENSG00000168575	200.4563781	-0.076156887	0.135794744	-0.560823524	0.574917849	0.719261758
ENSG00000168591	109.6264482	-0.207884878	0.179815974	-1.156097947	0.247641096	0.411914088
ENSG00000168610	265.2676853	0.151095138	0.116314261	1.299025047	0.193935334	0.34476587
ENSG00000168612	149.6480665	0.130439675	0.145647469	0.895584908	0.370474499	0.541536387
ENSG00000168615	653.6939069	0.129177861	0.083129363	1.553937824	0.120199248	0.242208986
ENSG00000168631	9.855922697	-0.912814578	0.608421681	-1.500299226	0.13353691	0.2619091
ENSG00000168646	61.34271914	-0.263442174	0.2246521	-1.17266731	0.240929246	0.40337701
ENSG00000168653	696.3652227	0.193326746	0.170480565	1.134010471	0.256790156	0.422535821
ENSG00000168661	33.43285163	0.140694262	0.3258933	0.431718794	0.665945806	0.786253443
ENSG00000168671	24.7741066	-2.335784709	0.43315023	-5.392551007	6.95E-08	7.48E-07
ENSG00000168679	25.13089823	-2.721770401	0.44222021	-6.154785189	7.52E-10	1.06E-08
ENSG00000168701	155.6555747	0.522124505	0.149049184	3.50303499	0.000459989	0.002247817
ENSG00000168702	1037.496008	-0.06470204	0.078078994	-0.828674099	0.407288846	0.575687907
ENSG00000168710	900.4434863	-0.031023967	0.073452891	-0.422365498	0.672758257	0.791410706
ENSG00000168724	488.8807918	0.105853554	0.095218483	1.111691245	0.266270925	0.433106495
ENSG00000168734	103.1647262	-0.815859572	0.179110808	-4.555054938	5.24E-06	4.00E-05
ENSG00000168743	11.76707423	-2.850465671	0.657997428	-4.332031629	1.48E-05	0.00010203
ENSG00000168758	357.8578347	0.139917174	0.110590101	1.265187141	0.205804251	0.359373328
ENSG00000168763	205.4650241	-0.020571732	0.129061631	-0.159394641	0.873357961	0.929849389
ENSG00000168765	232.0142908	-0.030983107	0.12249254	-0.25293873	0.80031556	0.880924836
ENSG00000168769	1809.254664	-0.067113998	0.069462886	-0.966184991	0.333951622	0.505990311
ENSG00000168772	2311.549137	-0.437243582	0.063362529	-6.900664951	5.18E-12	9.44E-11
ENSG00000168778	53.10295806	-0.29797284	0.247088847	-1.205933995	0.227842942	0.386581689
ENSG00000168779	4824.88483	0.887623877	0.064679435	13.7234328	7.35E-43	6.26E-41
ENSG00000168781	81.04308958	-0.20539341	0.210676293	-0.974924173	0.329597875	0.502149212

ENSG00000168785	19.36980629	1.238755455	0.401801743	3.083001698	0.00204924	0.008334628
ENSG00000168792	134.5229143	-0.150779552	0.164984107	-0.913903491	0.360767559	0.532042195
ENSG00000168795	171.4812809	-0.435649821	0.153675939	-2.834860319	0.004584573	0.016640695
ENSG00000168802	15.91883977	0.216062858	0.42815585	0.504636006	0.613814516	0.748716754
ENSG00000168807	299.8032439	0.033302998	0.114964061	0.289681812	0.772059672	0.86337203
ENSG00000168811	12.09408928	0.414600514	0.538778438	0.769519501	0.441584973	0.606704236
ENSG00000168813	437.5023837	-0.20480007	0.101264663	-2.022423844	0.043132584	0.108756669
ENSG00000168818	319.6261374	-0.6894714	0.114444155	-6.024522605	1.70E-09	2.30E-08
ENSG00000168826	37.2453504	0.502066019	0.286686925	1.751269328	0.079899528	0.176331292
ENSG00000168827	434.7622777	-0.023038295	0.095194599	-0.242012632	0.808770375	0.887007922
ENSG00000168843	8.255013966	-3.044130359	0.814310479	-3.738292014	0.000185275	0.000999476
ENSG00000168872	350.3341684	0.055593923	0.102003594	0.545019259	0.585740272	0.72755271
ENSG00000168876	59.90929191	0.161549131	0.225516864	0.716350555	0.473774918	0.634909013
ENSG00000168883	433.0987275	0.136017906	0.10211418	1.332017812	0.182854336	0.329996851
ENSG00000168884	301.9372153	0.486407363	0.110851772	4.387907888	1.14E-05	8.10E-05
ENSG00000168887	175.8867919	-0.161250192	0.144538964	-1.11561746	0.264585915	0.431314651
ENSG00000168890	108.9391558	0.175565087	0.176996592	0.991912248	0.321240334	0.492989334
ENSG00000168894	408.8011041	0.309332019	0.108621389	2.847800267	0.004402254	0.016074465
ENSG00000168904	83.18449151	-0.343896472	0.196481053	-1.750278036	0.080070349	0.176597298
ENSG00000168906	971.030699	-0.07307441	0.072889259	-1.002540167	0.316082777	0.487699338
ENSG00000168916	317.2258067	-2.135223421	0.124944696	-17.0893483	1.78E-65	2.66E-63
ENSG00000168917	76.40870439	-0.156403778	0.204113082	-0.76626043	0.443521358	0.608412551
ENSG00000168918	14.31593969	-2.025350133	0.531493042	-3.81068043	0.000138585	0.000772807
ENSG00000168924	956.2389934	0.140505496	0.072717035	1.932222575	0.05333204	0.128202439
ENSG00000168936	188.4971623	0.262492098	0.144207336	1.820240946	0.06872232	0.15676233
ENSG00000168938	108.3421343	0.262540931	0.173803292	1.510563626	0.130899667	0.257997422
ENSG00000168939	16.26113901	-0.409455435	0.435436798	-0.940332642	0.347046961	0.518998461
ENSG00000168944	333.1530498	-0.279976175	0.115707244	-2.41969444	0.015533554	0.046914605
ENSG00000168958	356.344529	0.113516411	0.104327449	1.088078087	0.276560637	0.445050666
ENSG00000168970	8.322821698	-0.141920488	0.605938222	-0.234216101	0.814817215	0.891273744
ENSG00000168994	19.05625052	-0.463870638	0.401574052	-1.155131006	0.248036783	0.412279338
ENSG00000169016	142.9758642	0.194924105	0.154640847	1.260495585	0.207490639	0.361480964
ENSG00000169018	456.4296919	-0.13140432	0.098221213	-1.337840538	0.180948424	0.327507088
ENSG00000169019	30.53642851	0.170465337	0.318792627	0.534721704	0.592842311	0.733089931
ENSG00000169020	1089.059939	0.351432401	0.077644633	4.526164761	6.01E-06	4.53E-05
ENSG00000169021	185.7745115	-0.020388701	0.141765105	-0.14382031	0.885642365	0.937428852
ENSG00000169032	247.4925062	-0.057481834	0.120308227	-0.477788058	0.632801061	0.762346184
ENSG00000169045	6872.967359	-0.029153823	0.058105825	-0.501736663	0.615852771	0.750358564
ENSG00000169047	90.00219568	-1.262695136	0.204516783	-6.174041642	6.66E-10	9.51E-09
ENSG00000169057	424.5422428	-0.114363987	0.097776759	-1.169643868	0.242144316	0.404832326
ENSG00000169062	301.1338042	0.163375684	0.108649312	1.503697366	0.132659308	0.260806899
ENSG00000169071	61.98831744	-0.214454189	0.245948692	-0.871946858	0.383237369	0.552575821
ENSG00000169084	81.37009021	0.254835506	0.197915467	1.287597734	0.197886029	0.349415723
ENSG00000169087	64.37689249	0.125062232	0.219963421	0.568559225	0.569655303	0.714810407
ENSG00000169093	309.0084947	0.234541623	0.110383125	2.124796003	0.033603638	0.088362095
ENSG00000169100	7274.639309	0.021802825	0.057845447	0.376915143	0.706236668	0.815661201
ENSG00000169105	110.2937554	-0.19119582	0.172425219	-1.108862271	0.267489602	0.43438456
ENSG00000169118	293.9255657	-0.032671895	0.114003694	-0.286586284	0.774429118	0.864944408
ENSG00000169131	70.91034747	0.020996862	0.208588582	0.100661607	0.91981909	0.956026545
ENSG00000169136	380.0879958	0.182394311	0.100679787	1.811627902	0.070043711	0.159104787

ENSG00000169139	435.0099271	-0.142931408	0.096151075	-1.486529484	0.137139124	0.266850433
ENSG00000169155	197.1976084	-0.303093267	0.143515348	-2.111922324	0.034693112	0.090768141
ENSG00000169169	689.4300131	0.135085036	0.081322085	1.661111326	0.09669109	0.204212862
ENSG00000169180	1385.007613	0.370749936	0.068901531	5.380866472	7.41E-08	7.93E-07
ENSG00000169188	161.6726739	-0.021029555	0.147202872	-0.142861034	0.886399933	0.938018673
ENSG00000169189	155.3450224	0.295386971	0.150374137	1.964346906	0.049489876	0.120901614
ENSG00000169193	20.74766001	-0.311408954	0.40582359	-0.767350546	0.442873123	0.607820104
ENSG00000169213	79.2492331	0.350583173	0.2060859	1.7011507	0.088914693	0.191532692
ENSG00000169217	301.2381375	-0.012218511	0.111154714	-0.109923458	0.912470078	0.953030514
ENSG00000169220	425.7006254	0.481703755	0.0957901	5.028742571	4.94E-07	4.66E-06
ENSG00000169221	324.1070503	0.156939916	0.11942812	1.314095172	0.188814179	0.337783083
ENSG00000169223	1251.15911	0.297422883	0.077338888	3.845709315	0.000120204	0.000680576
ENSG00000169228	35.17089085	0.58056886	0.304258916	1.908140824	0.056373017	0.133965789
ENSG00000169230	683.4636157	0.410678631	0.092561212	4.436832891	9.13E-06	6.60E-05
ENSG00000169231	528.3167027	0.341242882	0.092496066	3.689269129	0.000224899	0.001188101
ENSG00000169239	22.54168521	-0.02746835	0.387602247	-0.070867364	0.943503317	0.968637121
ENSG00000169241	281.9430496	-0.113264149	0.122194522	-0.926916745	0.353969762	0.525381789
ENSG00000169242	12.36693085	-1.458726262	0.529700699	-2.753868863	0.005889536	0.020553444
ENSG00000169246	25.21808406	0.958577286	0.381260936	2.514228956	0.011929296	0.037462737
ENSG00000169248	24.0992232	-2.38866343	0.433791921	-5.506472836	3.66E-08	4.15E-07
ENSG00000169249	146.7786215	0.477320441	0.152232098	3.135478308	0.001715741	0.007177194
ENSG00000169251	321.0189569	0.386009947	0.107157323	3.602273153	0.000315447	0.001607304
ENSG00000169258	54.85315983	0.09465102	0.236709107	0.399862182	0.689258028	0.804480669
ENSG00000169288	133.2719543	-0.135016856	0.160162866	-0.842997256	0.399229979	0.56732256
ENSG00000169299	328.7313408	0.35109954	0.113524938	3.092708487	0.001983389	0.008117908
ENSG00000169330	16.64322051	-0.013923611	0.423505344	-0.032877061	0.973772626	0.986408007
ENSG00000169359	45.68948866	-0.191223566	0.261672824	-0.730773504	0.46491751	0.627529189
ENSG00000169371	97.12536904	0.164894041	0.184421838	0.894113424	0.371261207	0.54217406
ENSG00000169372	25.77419834	-0.124048407	0.357932956	-0.346568834	0.728915263	0.83264802
ENSG00000169375	1079.633377	-0.352742308	0.074459027	-4.737401522	2.16E-06	1.79E-05
ENSG00000169379	324.0516241	-1.111611206	0.116615978	-9.532237587	1.54E-21	5.63E-20
ENSG00000169398	1417.748936	-0.103112901	0.074196689	-1.38972374	0.164612783	0.305659717
ENSG00000169410	277.7087996	0.069800969	0.129683288	0.538241817	0.590410114	0.731606463
ENSG00000169418	29.01098007	2.475100975	0.386087417	6.410726859	1.45E-10	2.24E-09
ENSG00000169432	46.84828443	-3.717668244	0.398724407	-9.323904377	1.12E-20	3.95E-19
ENSG00000169439	41.50784736	-1.5351922	0.298122934	-5.149527349	2.61E-07	2.58E-06
ENSG00000169446	144.180825	-0.226927201	0.155119575	-1.462917882	0.14348985	0.275859083
ENSG00000169490	89.75314311	-0.607582044	0.192610202	-3.154464494	0.001607929	0.006784053
ENSG00000169499	66.40638716	0.268386694	0.228290014	1.175639218	0.239739077	0.401879815
ENSG00000169504	790.9903744	-0.331542621	0.078712916	-4.212048523	2.53E-05	0.000166736
ENSG00000169515	886.3026859	-0.468598597	0.085911239	-5.454450451	4.91E-08	5.44E-07
ENSG00000169519	101.7565523	-0.207313491	0.183983976	-1.126801883	0.259826254	0.425974404
ENSG00000169548	11.97306042	-0.316389681	0.494156252	-0.640262427	0.522002003	0.676061418
ENSG00000169550	96.80545425	-0.187809183	0.184060037	-1.020369151	0.307553419	0.478639804
ENSG00000169554	977.8621991	0.19664243	0.071589805	2.746793748	0.006018097	0.020893773
ENSG00000169564	2133.014798	0.307687419	0.065344302	4.708710798	2.49E-06	2.04E-05
ENSG00000169567	1089.577805	0.368830887	0.074312493	4.963242022	6.93E-07	6.36E-06
ENSG00000169570	37.22201255	0.133911395	0.287340578	0.46603719	0.641188881	0.768299941
ENSG00000169592	365.3101952	0.447296425	0.108410654	4.125945265	3.69E-05	0.000234042
ENSG00000169598	60.66185074	0.623755784	0.229490733	2.717999881	0.006567786	0.022529351

ENSG00000169599	130.4543298	0.325897582	0.161747559	2.01485317	0.043920036	0.110304062
ENSG00000169604	985.4056473	-0.275284249	0.073784792	-3.730907697	0.000190791	0.001025015
ENSG00000169607	225.0186941	-0.29837711	0.126524195	-2.358261284	0.018360765	0.053864066
ENSG00000169609	84.29201015	0.016962343	0.193713463	0.087564088	0.930223146	0.962133707
ENSG00000169612	71.09252861	0.005603301	0.207877328	0.026954844	0.97849575	0.988843871
ENSG00000169621	13.88988097	-1.28414612	0.48905699	-2.625759669	0.008645584	0.028561739
ENSG00000169635	212.621299	-0.506149677	0.126777074	-3.99243853	6.54E-05	0.000392412
ENSG00000169641	504.0395266	-0.326307738	0.090798392	-3.593761182	0.000325939	0.001654068
ENSG00000169660	114.9415603	-0.675091064	0.175612929	-3.844199092	0.000120947	0.000683953
ENSG00000169679	606.56372	-0.142818109	0.091108849	-1.567554747	0.11698508	0.236853476
ENSG00000169683	194.4246141	-0.059224739	0.137094648	-0.431998913	0.665742203	0.786165328
ENSG00000169684	121.7897937	0.452372425	0.16208559	2.790947821	0.005255394	0.018675063
ENSG00000169689	591.2930863	0.30595088	0.086877816	3.521622581	0.000428914	0.002113279
ENSG00000169692	141.0079985	0.284598032	0.17433089	1.632516371	0.102570753	0.213928933
ENSG00000169696	230.1588523	0.443826927	0.135967014	3.264225004	0.00109764	0.00488083
ENSG00000169710	19351.38174	0.015435112	0.066128001	0.233412658	0.815440985	0.891608898
ENSG00000169714	1840.02046	-0.074075327	0.064047018	-1.156577302	0.2474451	0.411685577
ENSG00000169718	569.9596269	0.128612795	0.088409846	1.454733844	0.145742959	0.279069159
ENSG00000169727	796.2350966	0.345744071	0.07654778	4.516709349	6.28E-06	4.71E-05
ENSG00000169733	140.2614285	0.571871212	0.166704518	3.430448199	0.000602585	0.002862534
ENSG00000169738	275.2627601	0.058258813	0.120275204	0.484379248	0.628116743	0.758785336
ENSG00000169740	36.48582035	-0.457874931	0.294783421	-1.553258762	0.120361328	0.242431681
ENSG00000169750	311.8272147	-0.021249478	0.108343906	-0.196129882	0.844508508	0.909934111
ENSG00000169756	256.540841	-0.009812103	0.120076906	-0.081715156	0.934873226	0.964624614
ENSG00000169758	13.50954857	-0.146849039	0.479942809	-0.305971955	0.759625999	0.855073837
ENSG00000169760	199.0515031	-2.677487429	0.16615239	-16.1146489	2.01E-58	2.57E-56
ENSG00000169762	82.49675095	-0.169287072	0.193949343	-0.872841691	0.382749372	0.552299228
ENSG00000169764	228.0303822	-0.211648666	0.131453897	-1.610060032	0.107384751	0.221861098
ENSG00000169783	55.90054034	-1.547394346	0.270077709	-5.729441175	1.01E-08	1.24E-07
ENSG00000169813	2401.045025	-0.188279765	0.057501828	-3.274326584	0.001059141	0.004730606
ENSG00000169814	167.0422994	0.073848966	0.146569908	0.503848075	0.614368142	0.749042347
ENSG00000169826	193.3151877	-0.103969279	0.133290558	-0.780019838	0.435379199	0.601607562
ENSG00000169851	912.5669723	-2.073682811	0.080864442	-25.6439388	4.94E-145	2.48E-142
ENSG00000169855	878.5547552	-0.051792298	0.076768938	-0.674651742	0.499897052	0.658050219
ENSG00000169857	90.60948785	0.199163474	0.185491535	1.073706542	0.282954228	0.452333908
ENSG00000169871	303.3470004	-0.080511326	0.127521728	-0.631353787	0.527809225	0.680568444
ENSG00000169884	37.51906191	0.811632174	0.284222558	2.855621951	0.004295262	0.015741078
ENSG00000169894	22.26270729	-3.887481929	0.59460963	-6.537872464	6.24E-11	1.02E-09
ENSG00000169895	693.5623877	0.208712451	0.082731357	2.522773191	0.011643347	0.03671248
ENSG00000169902	64.25833061	-0.057835459	0.216084876	-0.267651581	0.788967523	0.873955272
ENSG00000169905	1045.748501	0.237404528	0.071687703	3.311649252	0.000927478	0.004206741
ENSG00000169908	22.61501806	-1.29345978	0.383758554	-3.370504106	0.000750308	0.003474967
ENSG00000169914	281.8986941	-0.156235413	0.117055163	-1.334716112	0.181969278	0.32876173
ENSG00000169919	323.8462109	0.111657785	0.105957558	1.053797262	0.291975747	0.462535239
ENSG00000169925	478.9993961	-0.415237777	0.092497705	-4.489168435	7.15E-06	5.29E-05
ENSG00000169926	376.2677523	0.310333784	0.102679091	3.022365909	0.002508072	0.009918605
ENSG00000169951	65.93914193	0.314248663	0.222623623	1.411569263	0.158076828	0.296319858
ENSG00000169957	363.0961321	-0.222992758	0.107541317	-2.073554284	0.038120725	0.097974443
ENSG00000169964	26.01564799	-0.748142723	0.371325806	-2.014787848	0.043926883	0.110304062
ENSG00000169967	671.2736278	0.046028616	0.081700799	0.563380237	0.573175991	0.717878752

ENSG00000169972	56.20946374	0.023328361	0.240506658	0.096996735	0.922728987	0.957350918
ENSG00000169976	289.2891878	0.322747693	0.129058496	2.500786098	0.0123918	0.038707496
ENSG00000169981	152.2798669	0.018196846	0.146782255	0.123971701	0.901337682	0.946765155
ENSG00000169991	139.6673945	-0.321443893	0.153984863	-2.087503184	0.036842674	0.095344332
ENSG00000169992	754.4418721	-0.111408533	0.088518103	-1.258596031	0.208176281	0.362540568
ENSG00000170004	2755.863504	0.259794722	0.061748588	4.207298187	2.58E-05	0.000169721
ENSG00000170017	1109.201781	2.296169997	0.083159343	27.61169008	8.05E-168	4.92E-165
ENSG00000170027	2544.443709	-0.063968486	0.064444831	-0.992608482	0.32090079	0.492590252
ENSG00000170035	117.1822419	0.588963312	0.171164021	3.440929403	0.00057972	0.002764197
ENSG00000170037	216.4656032	0.125779748	0.12859711	0.978091565	0.328029039	0.500415282
ENSG00000170043	310.9253196	-0.061221671	0.124457157	-0.491909607	0.622783246	0.755134766
ENSG00000170044	7.821755511	-4.345596611	1.108661752	-3.919677578	8.87E-05	0.000516596
ENSG00000170049	55.25677564	0.466957545	0.2408221	1.939014503	0.052499572	0.126733372
ENSG00000170085	302.2493615	-0.335878256	0.115971625	-2.896210655	0.003776988	0.014069304
ENSG00000170088	254.6663633	0.038425523	0.12849249	0.299048784	0.764902823	0.858399773
ENSG00000170100	149.709989	-0.93369507	0.156623683	-5.961391365	2.50E-09	3.31E-08
ENSG00000170113	426.0532897	-0.142955039	0.097634882	-1.464179984	0.143144778	0.275370404
ENSG00000170142	609.448861	0.445058438	0.088820077	5.010786435	5.42E-07	5.10E-06
ENSG00000170144	1210.711327	-0.022699089	0.079062946	-0.28710149	0.774034612	0.864738597
ENSG00000170145	346.3661908	-0.390332913	0.107720942	-3.623556445	0.00029058	0.0014968
ENSG00000170153	37.68223267	-0.240170172	0.294801005	-0.814685729	0.415252224	0.583209876
ENSG00000170160	6.653108198	-1.918230992	0.748880716	-2.561463997	0.010423204	0.033420395
ENSG00000170161	10.92082498	0.03963585	0.513126241	0.077243857	0.938429553	0.966102042
ENSG00000170175	29.28397938	-0.599245873	0.347655257	-1.723678442	0.084765909	0.184720248
ENSG00000170185	157.8226449	-0.185658736	0.144695678	-1.283098002	0.199457744	0.351452951
ENSG00000170190	118.5523604	-0.330051195	0.168195314	-1.962309093	0.049726509	0.121331992
ENSG00000170191	149.4392161	0.269572763	0.156895919	1.718163005	0.085766887	0.186323456
ENSG00000170222	33.28436294	0.04857102	0.307097307	0.158161661	0.874329414	0.930361736
ENSG00000170234	155.3231176	-0.164905091	0.145826433	-1.130831271	0.258126115	0.423744186
ENSG00000170242	690.9470575	-0.151066239	0.081459546	-1.854493982	0.06366852	0.147607147
ENSG00000170248	1600.958141	0.096113712	0.064186125	1.497421942	0.134283503	0.262904084
ENSG00000170260	78.66080841	0.483453996	0.199785667	2.41986326	0.015526344	0.046913005
ENSG00000170264	132.5662058	-0.141753674	0.161242712	-0.879132283	0.379329555	0.548871267
ENSG00000170265	459.8500131	0.518673176	0.094390297	5.494984047	3.91E-08	4.41E-07
ENSG00000170266	935.0424477	0.472203876	0.089240679	5.291352361	1.21E-07	1.26E-06
ENSG00000170270	27.27921413	-0.272966423	0.346030866	-0.788849925	0.430199726	0.59695896
ENSG00000170271	402.4322519	-0.047875746	0.107587626	-0.444993051	0.656324776	0.77965278
ENSG00000170275	2200.243409	0.24446094	0.05929067	4.123092863	3.74E-05	0.00023664
ENSG00000170291	164.44302	0.130945832	0.148566059	0.881398034	0.378102422	0.547434005
ENSG00000170293	181.5018305	0.111826382	0.135156196	0.827386282	0.408018154	0.576486723
ENSG00000170296	112.8086354	-0.046670639	0.177692359	-0.262648542	0.792821482	0.876423602
ENSG00000170310	136.6595151	0.122927395	0.154802327	0.794092685	0.427141476	0.594372497
ENSG00000170312	365.5059553	-0.28124657	0.112115382	-2.508545791	0.012122925	0.037977416
ENSG00000170315	2822.524506	0.487297158	0.072854146	6.688667454	2.25E-11	3.82E-10
ENSG00000170322	193.8078401	-0.294834362	0.132075306	-2.23232012	0.025593813	0.070715242
ENSG00000170325	150.8307637	0.419349556	0.148146298	2.830644863	0.004645427	0.016830556
ENSG00000170340	82.32807825	-0.190416363	0.197118413	-0.96599988	0.334044241	0.505990311
ENSG00000170345	1058.449492	1.025047341	0.091698463	11.17845711	5.20E-29	2.68E-27
ENSG00000170348	807.7532834	0.11045898	0.077719699	1.421248181	0.155244622	0.292709201
ENSG00000170364	17.4951369	-0.287654988	0.424774143	-0.677195146	0.498282147	0.65629342

ENSG00000170365	153.4112301	-0.241952792	0.148721298	-1.626887305	0.103761035	0.215867235
ENSG00000170382	26.37592088	-1.632255152	0.38577631	-4.231092243	2.33E-05	0.00015402
ENSG00000170385	235.1991217	0.02377328	0.136806735	0.17377273	0.862044077	0.921617562
ENSG00000170390	72.3040406	0.33229888	0.213946664	1.55318561	0.120378798	0.242431681
ENSG00000170396	1060.236484	4.802047513	0.11477707	41.83803876	0	0
ENSG00000170412	8.560567378	-1.880659174	0.672941924	-2.794682732	0.005195067	0.018494733
ENSG00000170417	31.91364419	-0.141948442	0.3071954	-0.462078669	0.644024907	0.770422279
ENSG00000170421	56.03097593	1.137433924	0.244789879	4.646572513	3.37E-06	2.69E-05
ENSG00000170425	99.28807041	0.434781452	0.181879869	2.390486937	0.016826048	0.050149691
ENSG00000170430	145.9440859	0.442254279	0.151396194	2.921171714	0.003487176	0.013160762
ENSG00000170439	120.0415392	0.453332869	0.167763308	2.702217042	0.006887878	0.0234669
ENSG00000170445	672.5319789	0.140394304	0.088938414	1.578556418	0.114437838	0.23283724
ENSG00000170448	215.1582801	0.284849191	0.130564895	2.181667516	0.02913408	0.078625633
ENSG00000170456	272.0946525	-0.088783348	0.113332409	-0.783388872	0.433398789	0.599680141
ENSG00000170464	39.00554462	-0.097749003	0.28044017	-0.348555639	0.727422936	0.831595187
ENSG00000170468	39.93989646	-0.594087777	0.293624892	-2.023288192	0.043043444	0.108551392
ENSG00000170469	21.81971222	-0.10558792	0.384977584	-0.274270306	0.783876903	0.871011269
ENSG00000170471	540.5660672	-0.163432045	0.08778101	-1.861815487	0.062629103	0.145777036
ENSG00000170473	149.3222348	-0.132106042	0.153038589	-0.86322047	0.388016261	0.556567387
ENSG00000170485	63.80511065	-1.306064791	0.23684739	-5.514372747	3.50E-08	3.98E-07
ENSG00000170500	62.85080354	0.522320727	0.232495363	2.246585563	0.024666526	0.068517043
ENSG00000170502	225.6119214	0.198144262	0.130642478	1.516690936	0.129344734	0.255722094
ENSG00000170515	1568.044276	0.155507569	0.072868383	2.13408839	0.032835546	0.086666839
ENSG00000170522	977.1481946	-0.204883342	0.074222207	-2.760404865	0.005772977	0.02017239
ENSG00000170525	354.0676259	0.265584635	0.103273005	2.571675291	0.010120777	0.032614487
ENSG00000170537	25.78166751	0.125053766	0.342572241	0.365043488	0.715078944	0.822477747
ENSG00000170540	211.5799274	-0.230080653	0.132218483	-1.740154994	0.081831806	0.179784224
ENSG00000170542	134.0458746	0.127682797	0.158067275	0.807775025	0.419220105	0.587000081
ENSG00000170545	16.20530525	-1.430882693	0.465291227	-3.075241077	0.002103325	0.008514292
ENSG00000170558	75.58720737	0.625629537	0.206861237	3.024392321	0.002491331	0.009857953
ENSG00000170571	448.7314896	0.109325204	0.101216624	1.080111147	0.280092689	0.448934504
ENSG00000170577	329.9959403	0.026913791	0.133365719	0.201804415	0.84006963	0.906960118
ENSG00000170581	164.1711944	-0.147799919	0.140811878	-1.049626786	0.293889737	0.464320002
ENSG00000170584	220.0809756	-0.255320066	0.126174096	-2.023553758	0.043016087	0.108519846
ENSG00000170604	267.4783735	0.132809698	0.12580784	1.055655186	0.291125778	0.461562506
ENSG00000170606	1913.982106	0.190115331	0.067401494	2.820639693	0.0047928	0.017269459
ENSG00000170608	11.92172568	-3.078275253	0.682662806	-4.509217768	6.51E-06	4.85E-05
ENSG00000170619	129.2467343	0.282650587	0.162397224	1.740489036	0.081773184	0.179760236
ENSG00000170631	73.61813881	0.189682014	0.207898512	0.912377929	0.361569804	0.532498536
ENSG00000170632	74.71245401	0.152889165	0.206304369	0.741085448	0.458641632	0.622312879
ENSG00000170633	198.5338572	-0.186700549	0.144107608	-1.295563445	0.19512593	0.346334948
ENSG00000170634	68.94417238	-0.278002888	0.214991666	-1.293086815	0.195981035	0.347103126
ENSG00000170638	402.7376781	0.222601965	0.113966511	1.953222605	0.050793224	0.123313587
ENSG00000170653	386.4347341	0.079225741	0.101167664	0.783113282	0.433560592	0.599785862
ENSG00000170677	174.0555555	-0.118731417	0.141000254	-0.842065268	0.399751421	0.567948641
ENSG00000170684	54.535247	-0.205749478	0.234887042	-0.875950742	0.381056813	0.550538001
ENSG00000170689	413.6938799	-1.151317666	0.106860548	-10.77401981	4.57E-27	2.20E-25
ENSG00000170703	12.22297075	-2.595475678	0.984617238	-2.636025024	0.008388355	0.02787585
ENSG00000170734	304.6479767	0.223515998	0.125357965	1.783021908	0.074582737	0.166666196
ENSG00000170759	2162.648005	-0.088115879	0.064124139	-1.374145208	0.169396607	0.311985595

ENSG00000170776	865.3963568	-0.140441012	0.077635686	-1.808974965	0.070454893	0.159832022
ENSG00000170779	326.9514946	-0.168487305	0.110322274	-1.527228347	0.12670424	0.251884299
ENSG00000170791	104.4298166	-0.065569051	0.176406393	-0.371693165	0.710121315	0.818499776
ENSG00000170802	381.584901	0.134585094	0.099021778	1.35914641	0.174100199	0.318352707
ENSG00000170832	995.9575931	0.090497991	0.075194148	1.203524381	0.228773462	0.387645455
ENSG00000170836	402.233047	-0.127433282	0.098293632	-1.296455115	0.194818734	0.346015632
ENSG00000170837	25.29113734	0.873191297	0.349050059	2.501621972	0.012362585	0.03864505
ENSG00000170846	135.1544303	0.094363267	0.154571899	0.610481385	0.541542971	0.691142874
ENSG00000170852	271.9615854	-0.141502626	0.115742924	-1.222559628	0.221496069	0.379248572
ENSG00000170854	266.1960036	0.388056229	0.116253417	3.338019976	0.000843777	0.003856991
ENSG00000170855	74.22340189	-0.581944981	0.212003782	-2.744974525	0.00605156	0.020989199
ENSG00000170860	712.5055456	0.433945752	0.08176186	5.307434916	1.11E-07	1.16E-06
ENSG00000170871	537.9828727	-0.304192589	0.094525064	-3.218115668	0.001290358	0.005601195
ENSG00000170873	33.32011095	0.253709294	0.310818563	0.816261716	0.414350465	0.582504102
ENSG00000170876	302.9774679	0.280175806	0.108902897	2.572712139	0.01009051	0.032524412
ENSG00000170881	226.2233608	0.056722517	0.130312035	0.435282263	0.663357558	0.784755231
ENSG00000170889	3948.730814	0.128857452	0.05972112	2.157652984	0.030954822	0.082587347
ENSG00000170892	131.8641453	-0.238006086	0.156506974	-1.520737888	0.128325627	0.253993248
ENSG00000170899	166.7783048	-0.011728999	0.143876346	-0.081521385	0.935027319	0.964624614
ENSG00000170903	161.2195566	-0.144973164	0.14462988	-1.002373533	0.31616322	0.487716314
ENSG00000170906	1303.431195	0.114788577	0.070065231	1.638310127	0.101357016	0.211912997
ENSG00000170915	15.76045067	-0.157085814	0.439077969	-0.357762914	0.720520746	0.826419116
ENSG00000170917	40.12213752	-0.531854823	0.276356863	-1.924521857	0.054289204	0.130092195
ENSG00000170919	93.9402169	-0.163698694	0.190108606	-0.861079874	0.389194053	0.557687326
ENSG00000170921	586.8590601	-1.068978518	0.095273375	-11.22011811	3.25E-29	1.70E-27
ENSG00000170946	108.8622615	-0.247881693	0.169832751	-1.459563551	0.144410063	0.277272848
ENSG00000170949	252.7220091	0.339433721	0.124084063	2.735514245	0.006228289	0.021517161
ENSG00000170959	53.72474595	-6.631606557	0.856076346	-7.746513016	9.45E-15	2.23E-13
ENSG00000171004	214.0376903	-0.270705627	0.13133644	-2.061161591	0.039287627	0.100340598
ENSG00000171016	238.2486707	-0.224454505	0.125143125	-1.793582392	0.072879849	0.163903817
ENSG00000171044	38.49502661	-0.605076819	0.283626872	-2.133355048	0.032895612	0.086809066
ENSG00000171045	50.90631372	0.266176356	0.262872031	1.01257009	0.311265552	0.482652303
ENSG00000171055	321.3345147	-0.01189358	0.119273693	-0.099716708	0.920569236	0.956523119
ENSG00000171056	65.56950788	0.727519448	0.225640572	3.224240401	0.001263073	0.005501516
ENSG00000171067	473.0493982	0.089007538	0.093507051	0.951880495	0.341157593	0.51308787
ENSG00000171100	54.43386225	-0.937764499	0.24902676	-3.76571778	0.000166071	0.000905591
ENSG00000171103	76.92785246	-0.010234484	0.210772419	-0.048557036	0.96127231	0.979613475
ENSG00000171105	363.6717683	-1.011367787	0.105616046	-9.575891367	1.01E-21	3.74E-20
ENSG00000171109	184.1716897	0.316181037	0.137674345	2.296586458	0.021642372	0.061565492
ENSG00000171119	82.87628976	0.502090285	0.197825748	2.53804315	0.011147424	0.035376774
ENSG00000171126	90.05573383	0.634738929	0.194491472	3.263582311	0.001100132	0.004887273
ENSG00000171130	227.4637974	0.622078862	0.127365375	4.884207064	1.04E-06	9.17E-06
ENSG00000171132	211.1260812	0.106025655	0.128436361	0.825511198	0.409081428	0.577637398
ENSG00000171135	250.9136624	-0.073952814	0.123751465	-0.597591421	0.55011259	0.698177769
ENSG00000171148	930.3676676	0.234408588	0.076329298	3.071017217	0.002133308	0.008608614
ENSG00000171150	236.6146599	0.143068283	0.125056369	1.14403036	0.252611046	0.417265683
ENSG00000171155	85.90689901	0.223783188	0.189501458	1.180904834	0.237640531	0.398964567
ENSG00000171159	443.3622833	0.439833485	0.104152275	4.222984903	2.41E-05	0.000159144
ENSG00000171160	42.24693152	-0.495517546	0.277080009	-1.788355457	0.073718678	0.165234764
ENSG00000171161	389.0305754	-0.089868146	0.103092566	-0.871722855	0.383359589	0.552575821

ENSG00000171163	510.3481375	0.619853047	0.094709874	6.544756323	5.96E-11	9.78E-10
ENSG00000171169	96.29331316	0.450044544	0.182388235	2.467508627	0.013605694	0.041866197
ENSG00000171174	10.75186069	-2.035427324	0.607032887	-3.353075864	0.000799188	0.003673493
ENSG00000171202	35.00588597	0.313458256	0.289329069	1.083397038	0.27863223	0.447410486
ENSG00000171204	72.85360923	0.26455785	0.219057645	1.20770882	0.227159284	0.385691383
ENSG00000171206	439.123004	0.422154668	0.100813876	4.187465891	2.82E-05	0.000183533
ENSG00000171208	151.6812469	-0.625607003	0.148854379	-4.202812228	2.64E-05	0.000172476
ENSG00000171219	100.0459049	0.422441228	0.178655099	2.364562958	0.018051359	0.05314765
ENSG00000171222	177.5804191	0.537917523	0.140268805	3.83490488	0.000125613	0.000708913
ENSG00000171223	1200.241854	0.318194049	0.098166668	3.241365487	0.001189586	0.005218647
ENSG00000171224	21.53252847	-0.003604019	0.36795814	-0.009794645	0.992185129	0.996085228
ENSG00000171227	10.81098862	-3.858965787	0.84799647	-4.550686144	5.35E-06	4.07E-05
ENSG00000171241	193.9191433	0.27579069	0.141527545	1.948671483	0.051334669	0.124370145
ENSG00000171243	28.70866939	-0.669875793	0.341806455	-1.959810246	0.050017973	0.121894946
ENSG00000171246	20.99758576	-0.152035465	0.372534107	-0.408111532	0.683191794	0.799666288
ENSG00000171262	280.9392932	-0.129028629	0.1218917	-1.058551396	0.289804132	0.460038269
ENSG00000171295	73.84540788	-0.169323271	0.209588337	-0.807884991	0.419156792	0.586969939
ENSG00000171298	618.9391786	-0.136074343	0.10500034	-1.295941933	0.194995491	0.346195862
ENSG00000171302	395.1623191	0.09597333	0.09933829	0.966126255	0.333981008	0.505990311
ENSG00000171307	191.0248635	-0.131692472	0.141111116	-0.933253707	0.350688991	0.522500564
ENSG00000171310	84.68290662	-0.559217651	0.196847597	-2.840866037	0.004499121	0.016360121
ENSG00000171311	161.1691938	0.15837538	0.146144138	1.083693005	0.278500938	0.447353079
ENSG00000171314	220.8756254	0.29406353	0.133092815	2.209462088	0.027142517	0.074270709
ENSG00000171316	1318.628661	-0.051367413	0.069014617	-0.744297584	0.456696463	0.620271199
ENSG00000171320	165.1112202	-0.223195421	0.142715633	-1.563917106	0.117837037	0.238269304
ENSG00000171357	32.29228973	0.738820295	0.320979535	2.301767606	0.021348281	0.060901565
ENSG00000171365	252.0866261	-0.12633629	0.1289207	-0.97995349	0.327109077	0.499337232
ENSG00000171368	16.39496453	0.864522487	0.429980828	2.010607056	0.044366978	0.11099251
ENSG00000171421	121.9024224	0.070142499	0.166198815	0.422039708	0.672996034	0.791624124
ENSG00000171425	171.8671236	0.126623021	0.141996526	0.891733231	0.372535937	0.543068941
ENSG00000171428	11.48897504	-0.220353638	0.514563344	-0.428234231	0.668480597	0.788094582
ENSG00000171435	16.94680598	-3.33662202	0.615716932	-5.419084396	5.99E-08	6.55E-07
ENSG00000171443	38.70828922	0.217259891	0.275927172	0.787381283	0.4310587	0.597552486
ENSG00000171444	223.6445822	-0.051217022	0.131007537	-0.390947141	0.695836308	0.809006175
ENSG00000171448	52.42568526	0.148903561	0.253665427	0.587007707	0.557198531	0.704307366
ENSG00000171453	172.4845878	0.196817189	0.14216447	1.384433041	0.166225898	0.308003586
ENSG00000171456	873.994308	-0.192617479	0.075710047	-2.544146873	0.010954501	0.034861323
ENSG00000171462	44.63978478	-3.26000724	0.377278865	-8.640842473	5.58E-18	1.66E-16
ENSG00000171466	281.3132496	0.161314486	0.112893517	1.428908323	0.153030585	0.28947221
ENSG00000171467	465.6824249	-0.305059131	0.097813461	-3.11878477	0.001815986	0.007522604
ENSG00000171469	156.1853457	0.353438357	0.143878274	2.456509563	0.014029407	0.042943046
ENSG00000171475	575.548832	0.118519025	0.086754691	1.366139672	0.171895128	0.315099944
ENSG00000171488	33.60552744	-1.37708333	0.320329905	-4.298953382	1.72E-05	0.000116847
ENSG00000171490	2339.952983	0.254933464	0.067303646	3.78781059	0.000151981	0.000838191
ENSG00000171492	125.3928687	-0.671324851	0.165965495	-4.0449664	5.23E-05	0.000320021
ENSG00000171497	427.3679514	0.414337605	0.099874842	4.14856831	3.35E-05	0.000214397
ENSG00000171503	208.6963449	0.466368269	0.143194397	3.25688908	0.001126404	0.004991357
ENSG00000171517	76.96393769	-1.867923713	0.229385631	-8.143159223	3.85E-16	1.01E-14
ENSG00000171530	902.2259419	0.378671429	0.076651472	4.940171635	7.81E-07	7.08E-06
ENSG00000171540	28.39357681	-2.134579259	0.395349687	-5.39921828	6.69E-08	7.25E-07

ENSG00000171551	20.1388827	-1.238184713	0.451861482	-2.740186458	0.006140434	0.021255464
ENSG00000171552	213.7538116	0.332732675	0.133141798	2.499085036	0.012451443	0.03887651
ENSG00000171566	196.7939408	0.044059861	0.134109017	0.328537647	0.742505184	0.842621612
ENSG00000171574	86.87280785	0.002666627	0.19224462	0.013871009	0.988932891	0.994454887
ENSG00000171603	1493.074026	0.123916043	0.069804981	1.775174787	0.075869031	0.169002899
ENSG00000171604	19.8327304	1.25663672	0.940086306	1.336724843	0.18131247	0.327968244
ENSG00000171606	167.912292	0.157672328	0.141058808	1.117777262	0.263662135	0.430208156
ENSG00000171608	49.60817481	-0.776689505	0.266863431	-2.91043813	0.003609224	0.013559447
ENSG00000171612	81.08649463	0.034620616	0.194760177	0.17776024	0.858911274	0.919492083
ENSG00000171617	64.11203777	0.308168407	0.238350698	1.292920096	0.196038696	0.347103126
ENSG00000171621	30.56890568	-0.208146539	0.31075935	-0.669799765	0.502985443	0.660882428
ENSG00000171634	2357.304382	-0.11578323	0.064638317	-1.791247597	0.073253571	0.164435711
ENSG00000171680	106.0465504	-0.396283026	0.176982688	-2.239106151	0.02514901	0.069623206
ENSG00000171681	1183.746335	0.312466328	0.085944997	3.635654655	0.000277276	0.001435111
ENSG00000171700	104.6506738	-0.016457393	0.175834279	-0.093596047	0.92543005	0.959161508
ENSG00000171703	212.186924	-0.511190731	0.130707824	-3.910942096	9.19E-05	0.000533651
ENSG00000171720	223.1858252	0.09377216	0.124570065	0.752766407	0.451590297	0.616629899
ENSG00000171723	339.7216176	-0.065995996	0.104389469	-0.632209333	0.5272501	0.680224298
ENSG00000171729	30.50544776	-0.020391191	0.32016924	-0.063688788	0.949218033	0.971841524
ENSG00000171735	111.0995426	-0.069945449	0.1753722	-0.398840003	0.6900111	0.804902039
ENSG00000171763	70.74979391	0.20789654	0.21237326	0.978920508	0.327619258	0.499953039
ENSG00000171766	246.145589	-0.698423793	0.136145487	-5.129981213	2.90E-07	2.83E-06
ENSG00000171791	68.53752544	-0.191884799	0.210389937	-0.91204362	0.361745756	0.532534336
ENSG00000171792	196.9041472	-0.173854757	0.133961444	-1.29779698	0.194357108	0.345411727
ENSG00000171793	943.2831822	0.006719295	0.079038942	0.085012463	0.932251482	0.96324653
ENSG00000171806	33.69704365	0.204873506	0.294925669	0.694661494	0.487267465	0.647676578
ENSG00000171813	132.9812438	0.722724948	0.156391649	4.621250241	3.81E-06	3.00E-05
ENSG00000171823	207.5602022	0.257505185	0.126878073	2.029548359	0.042402469	0.107242974
ENSG00000171824	781.7390734	-0.335251902	0.092259284	-3.633801254	0.000279276	0.0014444
ENSG00000171827	30.35320255	-0.126436935	0.322135643	-0.39249595	0.694691803	0.808344579
ENSG00000171848	1516.542344	-0.142586054	0.071536014	-1.993206562	0.046238833	0.11461764
ENSG00000171853	354.022336	0.028795398	0.108202043	0.266126194	0.790142022	0.874665498
ENSG00000171858	6311.874117	0.467028913	0.07156958	6.525522599	6.78E-11	1.10E-09
ENSG00000171861	116.2646611	-0.196021249	0.177448936	-1.104662861	0.269305702	0.436526332
ENSG00000171862	514.9628595	-0.609344007	0.089808783	-6.784904368	1.16E-11	2.03E-10
ENSG00000171863	2631.966894	0.218009806	0.066719395	3.267562713	0.001084778	0.004826945
ENSG00000171865	239.4152587	-0.017853807	0.126566545	-0.141062606	0.887820484	0.938532085
ENSG00000171867	649.8511123	-0.207060945	0.084725891	-2.443892217	0.014529767	0.044286565
ENSG00000171914	243.5088197	-0.535737454	0.121252308	-4.418369133	9.94E-06	7.13E-05
ENSG00000171928	56.04933232	0.462878457	0.240715073	1.922930919	0.054488726	0.130481271
ENSG00000171940	3993.059386	-0.701519144	0.058744058	-11.94195921	7.15E-33	4.27E-31
ENSG00000171943	79.91559709	0.043250307	0.199975256	0.216278295	0.828770827	0.899949274
ENSG00000171951	36.246783	-0.103976676	0.305366018	-0.340498517	0.733481139	0.835693316
ENSG00000171953	90.12876571	-0.237318938	0.191327981	-1.240377578	0.21483577	0.370873549
ENSG00000171960	432.3770326	-0.737859402	0.102031282	-7.231697821	4.77E-13	9.80E-12
ENSG00000171970	74.39929754	0.025185942	0.206370625	0.12204228	0.902865534	0.947590568
ENSG00000171984	15.2466572	0.762845909	0.449784187	1.696026518	0.089880858	0.193119793
ENSG00000171988	1422.668466	-0.859774036	0.078300667	-10.98041775	4.75E-28	2.41E-26
ENSG00000171992	18.04046244	0.694226428	0.412699076	1.682161333	0.09253756	0.197579435
ENSG00000172006	25.63435553	-0.452683733	0.354072853	-1.278504492	0.201071602	0.353410009

ENSG00000172007	63.48356777	-0.201377224	0.244886761	-0.822327934	0.410890283	0.579262926
ENSG00000172009	654.8973629	0.254602964	0.089823746	2.834472793	0.004590137	0.016656587
ENSG00000172031	28.04789383	1.10422524	0.342254197	3.226330754	0.001253884	0.00546997
ENSG00000172037	1346.326857	-0.213886329	0.067462658	-3.170440288	0.001522081	0.00646919
ENSG00000172046	512.1412604	0.056095012	0.089561688	0.626328217	0.531099682	0.683094573
ENSG00000172053	1079.931871	-0.179713343	0.074513745	-2.411814661	0.015873348	0.047817483
ENSG00000172057	112.257104	0.320784459	0.17179298	1.867273382	0.061863421	0.144351407
ENSG00000172059	145.0492428	-0.177929921	0.158475897	-1.122756993	0.261540715	0.42784004
ENSG00000172061	10.89496704	0.417859033	0.527950689	0.791473601	0.428667675	0.595656824
ENSG00000172062	14.58286649	0.121727115	0.457464147	0.26609105	0.790169088	0.874665498
ENSG00000172071	150.6021538	-0.054047659	0.16031568	-0.337132707	0.736016862	0.837626903
ENSG00000172081	346.109272	0.105953864	0.106118478	0.998448776	0.318061792	0.48988389
ENSG00000172086	133.6657507	-0.741746885	0.165419242	-4.484042333	7.32E-06	5.41E-05
ENSG00000172113	280.4924135	0.416508564	0.116681999	3.569604286	0.000357521	0.001796814
ENSG00000172115	906.6011005	0.338053743	0.096653569	3.497581594	0.000469497	0.002289145
ENSG00000172167	132.6745834	0.056335384	0.154250583	0.365219912	0.714947256	0.822444034
ENSG00000172171	52.66598252	-0.133766936	0.242645605	-0.551285221	0.581438168	0.723809106
ENSG00000172172	230.6798006	0.52315511	0.138600125	3.774564488	0.000160287	0.000878143
ENSG00000172175	224.2635994	-0.177106014	0.129123753	-1.371599035	0.170188294	0.313115255
ENSG00000172183	61.35008244	-1.40E-05	0.248157968	-5.65E-05	0.999954923	0.999954923
ENSG00000172197	27.33571859	0.293395928	0.330564212	0.887561079	0.374776897	0.544696439
ENSG00000172201	366.019793	0.651825464	0.11685198	5.578214965	2.43E-08	2.83E-07
ENSG00000172216	143.5525998	-0.32378012	0.162698417	-1.99006313	0.046583982	0.115408323
ENSG00000172232	7.515100823	-1.213134807	0.653424167	-1.856580867	0.063370808	0.147159818
ENSG00000172239	191.8297699	-0.041946196	0.136384114	-0.307559253	0.75841773	0.854466943
ENSG00000172244	51.92793891	-0.080054921	0.243136128	-0.32925967	0.741959425	0.842206387
ENSG00000172260	88.81609322	0.538593348	0.192280315	2.801084177	0.005093123	0.018176601
ENSG00000172262	381.5236293	0.051593137	0.100290264	0.514438139	0.6069457	0.743700634
ENSG00000172269	227.9041197	0.490559533	0.131109458	3.741602939	0.00018285	0.000988264
ENSG00000172270	2837.562677	0.155632063	0.060651276	2.56601464	0.010287449	0.03309844
ENSG00000172273	68.51213769	0.111120753	0.210839461	0.52703964	0.598166062	0.737250037
ENSG00000172292	529.5400213	0.386151626	0.099688126	3.873597022	0.000107241	0.00061436
ENSG00000172301	287.7009535	0.339644266	0.111831533	3.037106396	0.002388611	0.009502357
ENSG00000172315	51.34644495	0.065401065	0.243394851	0.268703567	0.788157807	0.873316329
ENSG00000172331	70.72347154	-1.058506609	0.241921318	-4.375416839	1.21E-05	8.53E-05
ENSG00000172336	264.3397391	0.106159172	0.1141481	0.930012604	0.352364559	0.52377407
ENSG00000172339	25.18082338	0.399080833	0.353270158	1.129676038	0.258612758	0.424384744
ENSG00000172340	575.1074332	-0.104448808	0.085396112	-1.223109638	0.221288289	0.379077633
ENSG00000172346	13.89338631	-2.927051263	0.611898575	-4.783556268	1.72E-06	1.45E-05
ENSG00000172350	6.949870169	-1.790821079	0.719124685	-2.490278967	0.012764286	0.039729654
ENSG00000172354	962.4187303	0.164539083	0.072729743	2.262335544	0.02367668	0.066235707
ENSG00000172361	10.87754009	-0.153405159	0.531320796	-0.288724176	0.77279246	0.863872011
ENSG00000172366	208.5003192	0.286133092	0.350113439	0.817258239	0.413780867	0.582182384
ENSG00000172375	81.33510843	-0.138207021	0.202208289	-0.683488405	0.494298274	0.653493035
ENSG00000172379	133.2321667	-1.008393732	0.164825662	-6.117941329	9.48E-10	1.32E-08
ENSG00000172380	518.1722965	0.467582318	0.094451876	4.95048205	7.40E-07	6.75E-06
ENSG00000172382	6.931731045	-2.292843285	0.771324869	-2.972603861	0.002952853	0.011378779
ENSG00000172403	158.0717627	-2.341900714	0.172767667	-13.5552025	7.38E-42	6.10E-40
ENSG00000172409	54.27322682	-0.18634089	0.242550649	-0.768255582	0.442335358	0.607378766
ENSG00000172426	16.27519059	0.323725137	0.438001334	0.739096236	0.459848562	0.62308216

ENSG00000172428	148.1206889	0.500870342	0.154719512	3.237279745	0.001206751	0.005280755
ENSG00000172432	333.4482529	-0.132876341	0.115108614	-1.154356185	0.248354171	0.412635231
ENSG00000172456	20.39335627	-0.453793127	0.3842134	-1.181096564	0.237564365	0.398884369
ENSG00000172458	14.02757414	0.409603137	0.460987557	0.888534042	0.374253553	0.544273699
ENSG00000172465	21.55756655	0.4016038	0.374199276	1.073235107	0.283165643	0.452620367
ENSG00000172466	832.6472496	-0.179631601	0.082601001	-2.174690379	0.029653326	0.079751154
ENSG00000172469	92.57602656	0.168689859	0.183716926	0.918205322	0.358511383	0.529791585
ENSG00000172493	523.7967311	-0.136298007	0.090083992	-1.51301028	0.130277048	0.257058813
ENSG00000172500	273.1781332	0.336346051	0.112930585	2.978343313	0.002898112	0.011199388
ENSG00000172530	167.5808589	0.141016838	0.142171489	0.991878465	0.321256815	0.492989334
ENSG00000172531	723.7978227	0.159356567	0.087023933	1.831180935	0.067073541	0.153875884
ENSG00000172534	4512.607072	0.433098921	0.069030438	6.274028273	3.52E-10	5.21E-09
ENSG00000172586	474.4635372	0.14488254	0.102082885	1.419263768	0.155822131	0.293315264
ENSG00000172590	182.0100427	0.36663321	0.138914369	2.639274925	0.008308357	0.027636158
ENSG00000172594	7.875816468	-2.205305944	0.740739493	-2.97716804	0.002909245	0.011239319
ENSG00000172613	106.3137724	-0.000841516	0.173201311	-0.004858602	0.996123411	0.998255373
ENSG00000172638	274.7771937	0.254627752	0.119219452	2.135790325	0.032696508	0.086397263
ENSG00000172661	217.1839205	-0.016697449	0.127588147	-0.130869906	0.895878221	0.943561301
ENSG00000172663	175.0231535	0.05648087	0.1376374	0.410359903	0.681541956	0.798400454
ENSG00000172667	243.6590128	-0.270035372	0.125853626	-2.145630454	0.031902474	0.084585513
ENSG00000172671	118.3220012	0.002847138	0.163025423	0.017464379	0.98606615	0.992634039
ENSG00000172716	254.7202578	0.121092834	0.124834231	0.970029078	0.332031998	0.504440134
ENSG00000172725	589.9276971	0.09193213	0.090068842	1.020687375	0.307402578	0.478511083
ENSG00000172728	135.9778408	0.013348889	0.158178323	0.084391394	0.93274525	0.963270344
ENSG00000172731	73.9921642	-0.184642958	0.207278874	-0.890794873	0.373039226	0.54340148
ENSG00000172732	227.0920912	0.069429748	0.12946285	0.536290899	0.59175752	0.732260534
ENSG00000172748	15.54791576	0.231641786	0.449325951	0.515531733	0.606181501	0.743151644
ENSG00000172757	2327.829675	0.216157827	0.064323774	3.360465542	0.000778112	0.003590718
ENSG00000172765	220.388153	0.08388817	0.123009199	0.681966637	0.495260047	0.654135261
ENSG00000172766	275.1033225	0.040022202	0.113410237	0.352897609	0.724165203	0.829245016
ENSG00000172775	459.7071596	0.075335894	0.100161066	0.752147483	0.451962372	0.616859253
ENSG00000172780	7.575651785	0.217283979	0.686121639	0.316684342	0.751483131	0.849515817
ENSG00000172785	21.64384753	0.134009364	0.384654145	0.348389237	0.727547885	0.831595187
ENSG00000172789	14.45546589	-1.139803192	0.473579352	-2.406783968	0.016093687	0.048325784
ENSG00000172795	368.3450043	-0.575549027	0.103245466	-5.574569489	2.48E-08	2.88E-07
ENSG00000172799	10.81000767	1.126313685	0.548122864	2.054856235	0.039892893	0.101627759
ENSG00000172809	9302.434467	0.248343939	0.060526312	4.103074027	4.08E-05	0.000255894
ENSG00000172819	284.9944674	0.260274175	0.110955821	2.345746022	0.018989042	0.055340057
ENSG00000172828	36.81260714	-2.639958214	0.373543863	-7.067331239	1.58E-12	3.09E-11
ENSG00000172830	141.8309558	2.922148996	0.188823205	15.47558204	5.07E-54	5.94E-52
ENSG00000172831	278.6921643	0.159440989	0.11637126	1.370106235	0.170653742	0.313766114
ENSG00000172840	362.694497	0.261136757	0.106217025	2.458520717	0.013951073	0.042760357
ENSG00000172845	635.3663714	-0.079037785	0.088171998	-0.896404602	0.37003671	0.541314309
ENSG00000172869	312.8867033	-0.026259376	0.10955659	-0.239687778	0.81057231	0.888429248
ENSG00000172878	573.0536785	-0.800414342	0.091766434	-8.722299768	2.73E-18	8.37E-17
ENSG00000172888	320.9648128	0.124372904	0.107107565	1.16119626	0.245562095	0.40905007
ENSG00000172889	358.7520339	0.83918138	0.121241238	6.921583706	4.47E-12	8.24E-11
ENSG00000172890	215.9090538	0.507730435	0.129592982	3.917885257	8.93E-05	0.00052002
ENSG00000172893	1095.704972	1.005191245	0.087416043	11.49893328	1.34E-30	7.47E-29
ENSG00000172915	239.5479492	0.099018107	0.122127301	0.810777818	0.417493275	0.585515978

ENSG00000172922	343.9504951	0.5406974	0.109166842	4.952945336	7.31E-07	6.67E-06
ENSG00000172932	422.1962494	0.287931706	0.107985869	2.666383187	0.007667225	0.025802948
ENSG00000172939	596.0939544	0.158063027	0.083876606	1.88447094	0.059501302	0.139836798
ENSG00000172943	472.3879603	0.00407686	0.091899934	0.044361949	0.964615892	0.981383315
ENSG00000172954	416.0374223	-0.290536045	0.098782224	-2.941177404	0.003269672	0.012430064
ENSG00000172965	195.704517	-0.212448444	0.144922136	-1.465948885	0.142662213	0.274743091
ENSG00000172974	13.3567661	-0.653621646	0.497884685	-1.31279725	0.189251277	0.338478825
ENSG00000172977	280.5867891	0.156499422	0.118100536	1.325137272	0.185125626	0.332965386
ENSG00000172985	111.832773	1.046790922	0.323545478	3.235374908	0.001214831	0.00531446
ENSG00000172986	86.94568425	-0.548977771	0.192453517	-2.852521377	0.004337389	0.015866487
ENSG00000172987	30.44979375	-8.209335504	1.207450818	-6.798898456	1.05E-11	1.86E-10
ENSG00000172992	309.1764957	-0.303228013	0.111702157	-2.714611983	0.006635346	0.0227278
ENSG00000173011	227.3256117	-0.056032167	0.123043568	-0.455384773	0.648832448	0.773893979
ENSG00000173013	26.68816029	-0.96731757	0.346873221	-2.788677565	0.005292373	0.018770925
ENSG00000173020	482.2165372	0.21744863	0.108869092	1.997340354	0.045788222	0.113838436
ENSG00000173039	707.4136973	0.421454562	0.080580963	5.230200135	1.69E-07	1.72E-06
ENSG00000173064	836.0489063	-0.171507947	0.077625555	-2.209426342	0.027145	0.074270709
ENSG00000173065	491.7087343	-0.096657447	0.09055121	-1.067434069	0.285775871	0.455496841
ENSG00000173083	20.03275945	1.349485814	0.41248836	3.271573081	0.001069509	0.004770848
ENSG00000173085	116.682787	-0.093074237	0.179164698	-0.519489821	0.60341921	0.741253138
ENSG00000173113	481.4466525	0.490922493	0.095667662	5.131540627	2.87E-07	2.81E-06
ENSG00000173114	39.26308664	2.389351775	0.334771242	7.137267119	9.52E-13	1.89E-11
ENSG00000173120	1041.948032	0.124370128	0.070696823	1.759203919	0.078542879	0.174048244
ENSG00000173137	34.03152349	-0.605060082	0.323343333	-1.871261966	0.06130878	0.143295246
ENSG00000173141	572.782839	0.17249732	0.09004942	1.915585027	0.055417937	0.132255481
ENSG00000173145	191.3938667	-0.000234508	0.131465122	-0.001783805	0.998576731	0.999359314
ENSG00000173153	230.8318163	0.356067225	0.130361001	2.731393765	0.006306707	0.021750629
ENSG00000173157	97.59113057	1.264584135	0.20438957	6.187126551	6.13E-10	8.79E-09
ENSG00000173163	42.39752266	0.019727931	0.264220864	0.074664548	0.940481616	0.967262249
ENSG00000173166	285.5149162	-0.317168945	0.117207819	-2.706039134	0.006809102	0.023243611
ENSG00000173171	106.7509358	0.053336629	0.173801377	0.306882662	0.758932686	0.854772887
ENSG00000173193	694.1855215	0.602946471	0.085663454	7.038549587	1.94E-12	3.77E-11
ENSG00000173207	327.6582293	-0.27272624	0.110751314	-2.462510205	0.013796825	0.042370791
ENSG00000173208	41.58710435	-1.083418414	0.282656499	-3.832986037	0.000126597	0.000713894
ENSG00000173209	231.3235437	0.019141588	0.153275603	0.124883461	0.900615812	0.946219171
ENSG00000173213	12.48662594	-0.876298064	0.520223096	-1.684465896	0.092091665	0.196836826
ENSG00000173214	139.8319549	-0.348999442	0.164130957	-2.126347443	0.03347434	0.088121076
ENSG00000173218	167.2165771	-0.544692183	0.142686905	-3.817394349	0.000134869	0.000754479
ENSG00000173221	267.7704243	-0.856415672	0.126733473	-6.757612255	1.40E-11	2.44E-10
ENSG00000173226	148.9711778	-0.085670573	0.148990425	-0.575007238	0.565286402	0.710947988
ENSG00000173227	10.6141189	-1.149876894	0.55702858	-2.064305021	0.038988805	0.099795144
ENSG00000173230	1712.057126	-0.250530588	0.06963378	-3.597831212	0.000320882	0.001631352
ENSG00000173258	40.03537453	0.157975306	0.274232292	0.576063835	0.564572035	0.710622166
ENSG00000173264	192.7056741	0.115513071	0.136166821	0.848320241	0.396259648	0.564071674
ENSG00000173267	24.69337456	-0.710817934	0.395035867	-1.799375685	0.071959273	0.16227515
ENSG00000173272	503.5023099	0.436845431	0.09205767	4.745345287	2.08E-06	1.73E-05
ENSG00000173273	643.2019366	0.00898284	0.083717327	0.107299648	0.914551264	0.953830969
ENSG00000173275	65.4123386	0.134423436	0.229205197	0.586476389	0.557555423	0.704695072
ENSG00000173276	285.5864017	-0.325689602	0.129906185	-2.507113901	0.012172148	0.038114615
ENSG00000173281	70.16727763	-0.473316917	0.220861352	-2.143049983	0.032109085	0.08500496

ENSG00000173320	374.0043253	0.05335353	0.106142005	0.502661787	0.615202081	0.74963078
ENSG00000173327	235.0217146	0.040430491	0.148053369	0.273080518	0.784791322	0.871115267
ENSG00000173334	238.8307552	0.640884401	0.125622197	5.10168122	3.37E-07	3.27E-06
ENSG00000173349	47.31947827	-0.569435736	0.265058014	-2.148343779	0.031686455	0.084155727
ENSG00000173376	223.07462	-3.047167756	0.163038677	-18.68984593	5.99E-78	1.05E-75
ENSG00000173402	4118.110921	-0.049481731	0.056036844	-0.883021381	0.37722472	0.546670343
ENSG00000173409	89.12423897	-0.435019544	0.188362004	-2.309486707	0.020916588	0.059913401
ENSG00000173418	605.1244162	1.47348051	0.089693878	16.42788283	1.21E-60	1.60E-58
ENSG00000173436	32.41139235	-0.562642049	0.313229754	-1.796259911	0.07245319	0.163170556
ENSG00000173442	916.3920323	-0.332747813	0.090090858	-3.693469267	0.000221215	0.001170841
ENSG00000173451	30.0360403	-0.605082859	0.322533045	-1.876033692	0.060650654	0.142135424
ENSG00000173456	406.4877589	-0.218331914	0.099326863	-2.198115466	0.027940879	0.076033615
ENSG00000173457	165.2285138	0.07223969	0.145511488	0.496453521	0.619574472	0.753046428
ENSG00000173465	122.4918621	0.253213754	0.165865514	1.526620859	0.126855317	0.252037117
ENSG00000173473	2547.063525	0.255268071	0.058500395	4.363527324	1.28E-05	8.95E-05
ENSG00000173480	28.10879748	-0.285294618	0.332225158	-0.858738753	0.390484665	0.558795649
ENSG00000173486	18.32206716	-0.165762922	0.402250731	-0.412088554	0.680274518	0.797381188
ENSG00000173511	251.4101276	-0.181190705	0.126334507	-1.434213892	0.151511234	0.287449798
ENSG00000173517	585.4417782	-0.274759834	0.086067092	-3.192391289	0.001411	0.006059408
ENSG00000173530	188.904591	-0.650344192	0.138841194	-4.684086719	2.81E-06	2.27E-05
ENSG00000173531	10.74827658	0.081056991	0.537272652	0.150867517	0.880080225	0.934004754
ENSG00000173540	137.9569071	0.066285947	0.158616654	0.417900297	0.676020015	0.794117173
ENSG00000173542	308.0767859	-0.502603035	0.111919653	-4.490748675	7.10E-06	5.26E-05
ENSG00000173545	176.3084417	0.166190859	0.136831605	1.214564856	0.2245321	0.382778461
ENSG00000173546	134.9752464	-2.144120487	0.187472722	-11.43697316	2.73E-30	1.50E-28
ENSG00000173548	168.3811026	0.070883608	0.145748207	0.486342912	0.626724059	0.757754592
ENSG00000173575	2661.752103	-0.187399741	0.058968784	-3.177948219	0.001483212	0.006330807
ENSG00000173581	240.2589408	-0.172875888	0.128429278	-1.346078491	0.178277204	0.323966351
ENSG00000173588	123.5888995	-0.063272708	0.166494025	-0.380029904	0.703923217	0.814364144
ENSG00000173598	173.2960983	0.276342088	0.14283019	1.93475965	0.053019797	0.127616361
ENSG00000173599	35.57381297	0.136386803	0.314840018	0.433193988	0.664873846	0.785659558
ENSG00000173611	116.0246214	-0.360294401	0.173101916	-2.081400423	0.03739727	0.096455272
ENSG00000173614	32.03012322	-0.406888565	0.307223853	-1.32440421	0.185368837	0.333274806
ENSG00000173621	167.3778086	0.807673374	0.286916059	2.815016265	0.004877477	0.01752274
ENSG00000173626	8.433192247	-6.354898806	1.274591838	-4.985830458	6.17E-07	5.73E-06
ENSG00000173638	164.3653927	0.176940829	0.154779443	1.143180422	0.252963689	0.417651733
ENSG00000173641	17.27046132	-0.775009837	0.431240565	-1.797163581	0.072309654	0.162960647
ENSG00000173653	85.3615883	0.236850779	0.193023573	1.227056239	0.219801454	0.377312847
ENSG00000173660	2403.631967	0.510246125	0.136224597	3.745624026	0.000179946	0.000974817
ENSG00000173674	528.1086661	0.39591219	0.090743843	4.36296479	1.28E-05	8.97E-05
ENSG00000173681	48.97516292	-0.39245811	0.25207958	-1.556881803	0.119498551	0.241142959
ENSG00000173692	1286.96513	0.202284395	0.07131869	2.8363448	0.004563315	0.016572101
ENSG00000173705	7.505617346	-1.15181081	0.668735077	-1.722372355	0.085002088	0.185120052
ENSG00000173706	265.4055922	0.806581954	0.118633409	6.798944399	1.05E-11	1.86E-10
ENSG00000173715	35.46905881	0.223376177	0.288555622	0.774118263	0.438860858	0.604227552
ENSG00000173726	1635.168348	-0.064080748	0.066283598	-0.966766289	0.33366088	0.50583001
ENSG00000173744	455.0093171	-0.146767786	0.095282986	-1.540335716	0.123478542	0.247326833
ENSG00000173757	93.86970856	-0.940838179	0.192848097	-4.878649023	1.07E-06	9.39E-06
ENSG00000173762	169.2200451	2.100185778	0.170823059	12.29450984	9.69E-35	6.19E-33
ENSG00000173786	992.8694968	0.336409823	0.072986737	4.609191178	4.04E-06	3.15E-05

ENSG00000173801	422.8129797	-0.351879733	0.098419137	-3.57531822	0.000349802	0.001762436
ENSG00000173805	21.21306338	-0.488591641	0.387972986	-1.259344488	0.207905931	0.362114645
ENSG00000173812	1991.900358	-0.091912492	0.184407803	-0.498419756	0.618188215	0.75188641
ENSG00000173818	70.01312685	-0.474294847	0.219713456	-2.158697313	0.030873657	0.0824491
ENSG00000173821	2262.888619	0.143610468	0.06094705	2.356315338	0.018457242	0.054092376
ENSG00000173846	51.45344947	-0.000290643	0.244939508	-0.00118659	0.999053239	0.999693749
ENSG00000173848	305.7670237	-0.117683499	0.112177918	-1.049079013	0.294141754	0.464428366
ENSG00000173852	115.9805303	-0.251239027	0.183248073	-1.371032298	0.170364888	0.313317044
ENSG00000173875	286.5155586	0.283166451	0.110850212	2.554496253	0.010634157	0.033941834
ENSG00000173889	1340.025366	0.037358578	0.072885806	0.51256315	0.608256935	0.744723395
ENSG00000173894	386.4452436	-0.002081405	0.100692916	-0.020670822	0.983508245	0.991961125
ENSG00000173898	1293.899227	-0.098540599	0.068893168	-1.430339205	0.152619688	0.288928404
ENSG00000173905	1094.743144	0.400243466	0.070941004	5.641919972	1.68E-08	2.00E-07
ENSG00000173914	69.3594512	-0.064703417	0.212428502	-0.30458915	0.760679085	0.855436643
ENSG00000173915	1439.613361	0.706233652	0.091184489	7.745107344	9.55E-15	2.25E-13
ENSG00000173917	631.095781	-0.252373086	0.087015765	-2.900314511	0.003727884	0.013901138
ENSG00000173918	19.55704898	0.643292035	0.392489016	1.639006464	0.101211914	0.21170349
ENSG00000173928	40.42661549	0.607889889	0.27543913	2.206984497	0.027315142	0.07464899
ENSG00000173933	113.7874646	-0.079129601	0.171588252	-0.461159784	0.644683972	0.771040933
ENSG00000173947	12.13779317	-1.027541106	0.565203051	-1.818003465	0.069063603	0.157464118
ENSG00000173950	216.1503184	-0.375801341	0.129342034	-2.905485022	0.003666844	0.013717219
ENSG00000173960	317.5510875	-0.080411238	0.117684242	-0.68327957	0.494430201	0.653548606
ENSG00000173992	83.43523421	0.010345475	0.191569071	0.054003889	0.956932066	0.976895279
ENSG00000174004	43.19609222	0.602400518	0.269947616	2.231545985	0.025644985	0.070820507
ENSG00000174007	31.37904992	-0.459103852	0.314954285	-1.457684097	0.144927637	0.277924713
ENSG00000174010	250.2366711	-0.519191642	0.129025137	-4.023957297	5.72E-05	0.000346802
ENSG00000174013	232.7592594	0.255018903	0.130186816	1.958868892	0.050128143	0.122099883
ENSG00000174021	111.4218269	-0.172829853	0.172817094	-1.000073832	0.317274779	0.489001406
ENSG00000174028	8.906260499	0.620686991	0.597146036	1.039422443	0.298608311	0.469182736
ENSG00000174032	100.1156896	-0.623575138	0.18056103	-3.453542216	0.000553276	0.002650704
ENSG00000174059	41.48787186	-5.242713593	0.630681399	-8.312776634	9.35E-17	2.55E-15
ENSG00000174080	127.1829939	0.216780402	0.173704821	1.247981495	0.212037834	0.367126273
ENSG00000174099	386.225821	-1.413734886	0.107852134	-13.10808446	2.96E-39	2.25E-37
ENSG00000174106	331.4213161	-0.789026924	0.120789443	-6.532250689	6.48E-11	1.06E-09
ENSG00000174109	112.0479953	0.002524213	0.195839551	0.012889191	0.989716199	0.994744093
ENSG00000174130	67.00306173	3.045814242	0.277933152	10.95880148	6.03E-28	3.04E-26
ENSG00000174132	95.91392152	-0.203595114	0.205887988	-0.988863487	0.322729937	0.494479432
ENSG00000174136	175.1785847	-0.281136677	0.139205833	-2.019575402	0.043427449	0.109264799
ENSG00000174137	16.39556452	0.211945936	0.432087727	0.490515983	0.623768822	0.755807871
ENSG00000174151	27.57793581	0.131352126	0.344932812	0.380804962	0.703347977	0.814235127
ENSG00000174165	88.47305627	-0.061308755	0.192682683	-0.318185082	0.750344548	0.84850184
ENSG00000174173	272.9939466	0.120856291	0.113456622	1.065220246	0.286776274	0.45657331
ENSG00000174177	36.17974795	-0.442861621	0.287877501	-1.538368296	0.123958589	0.248040784
ENSG00000174197	1042.863132	-0.200742361	0.072399279	-2.772712159	0.005559127	0.019566288
ENSG00000174206	51.42690671	0.013478975	0.241799304	0.055744474	0.95554537	0.97611416
ENSG00000174227	170.6584436	-0.085862862	0.145397567	-0.590538509	0.554829676	0.701881526
ENSG00000174231	8795.942195	0.275411525	0.052582608	5.237692369	1.63E-07	1.66E-06
ENSG00000174233	330.1917678	-0.009117645	0.108245082	-0.084231493	0.932872379	0.963270344
ENSG00000174238	409.4175563	-0.275765795	0.105949841	-2.602795737	0.009246703	0.030227701
ENSG00000174243	1362.898393	-0.001556871	0.074804567	-0.020812509	0.983395219	0.991939727

ENSG00000174276	38.37135812	0.761649844	0.287295942	2.651098508	0.008023044	0.026826874
ENSG00000174279	6.051546192	-4.878585408	1.304400872	-3.740096709	0.000183949	0.00099344
ENSG00000174282	454.7926638	-0.010189353	0.094076999	-0.10830865	0.913750862	0.953578158
ENSG00000174306	371.8539805	0.026791234	0.100367493	0.266931387	0.789521989	0.874348005
ENSG00000174307	170.5493831	-0.072941231	0.148802399	-0.49018854	0.62400049	0.755922074
ENSG00000174327	61.65998864	-0.257049803	0.226976876	-1.132493353	0.257427077	0.423279662
ENSG00000174332	153.2898854	-0.177655925	0.153873693	-1.15455684	0.24827195	0.4126214
ENSG00000174365	38.086155	0.388127165	0.283261034	1.370210226	0.170621287	0.313747509
ENSG00000174371	318.8546225	-0.237933613	0.106602101	-2.231978653	0.025616374	0.070763659
ENSG00000174373	200.3407927	-0.22709571	0.135441205	-1.676710637	0.09359908	0.199270426
ENSG00000174405	70.38343698	-0.045246879	0.209771088	-0.215696448	0.82922437	0.900163426
ENSG00000174437	1724.897645	-0.282494925	0.066173695	-4.268991251	1.96E-05	0.000132053
ENSG00000174442	225.9268527	0.029491289	0.126437715	0.233247565	0.815569173	0.891619754
ENSG00000174444	6928.53777	0.116772287	0.053594063	2.17882878	0.02934439	0.079026198
ENSG00000174446	118.5718978	-0.109690792	0.16532398	-0.663489905	0.507016843	0.664185917
ENSG00000174456	16.54536054	-0.312918584	0.422497356	-0.740640338	0.458911543	0.62242959
ENSG00000174460	58.05327339	0.344645768	0.244981057	1.406826197	0.159478933	0.298195324
ENSG00000174469	7.707263827	0.276480115	0.64605736	0.427949796	0.668687672	0.788272552
ENSG00000174482	28.83942756	-3.308048226	0.473105731	-6.992196481	2.71E-12	5.14E-11
ENSG00000174483	10.92660853	0.151405187	0.51402654	0.294547411	0.76833964	0.860743833
ENSG00000174485	169.0543987	0.514382037	0.148664045	3.460029865	0.000540115	0.002594734
ENSG00000174498	36.36706161	-0.631171212	0.292950212	-2.154534065	0.031198313	0.083173792
ENSG00000174501	289.1565581	-0.258258751	0.122400459	-2.109949199	0.034862732	0.091144015
ENSG00000174514	30.03888927	-0.757208626	0.32829535	-2.306485993	0.021083492	0.06031768
ENSG00000174516	80.93449556	0.193525645	0.204741755	0.945218259	0.344547459	0.51630661
ENSG00000174529	19.9050828	-0.854457026	0.401918656	-2.125945173	0.033507825	0.088192696
ENSG00000174547	441.0280624	-0.03682784	0.098660328	-0.373279119	0.708940716	0.817673446
ENSG00000174564	8.265186296	-0.431805836	0.601209693	-0.718228333	0.472616511	0.633981332
ENSG00000174574	382.9677871	-0.068585589	0.101053854	-0.67870335	0.497325843	0.655493567
ENSG00000174579	403.4240133	-0.075722874	0.104540499	-0.724340091	0.46885697	0.630937837
ENSG00000174586	8.85877532	-0.157941507	0.577520295	-0.273482175	0.784482593	0.871011269
ENSG00000174606	404.0258802	-0.087813786	0.098723432	-0.889492838	0.373738271	0.54380583
ENSG00000174628	40.46206865	-0.078769393	0.282409243	-0.278919316	0.780306745	0.868470711
ENSG00000174652	68.67823872	-0.274850868	0.220163957	-1.248391752	0.211887628	0.366956665
ENSG00000174669	561.5167006	0.356368067	0.086492345	4.120226695	3.78E-05	0.000239387
ENSG00000174672	29.55986618	-0.972256019	0.340032937	-2.859299536	0.004245777	0.015571913
ENSG00000174684	275.1156142	0.381205517	0.129940287	2.93369766	0.003349503	0.012699182
ENSG00000174695	422.9397195	-0.11989692	0.098757368	-1.214055437	0.224726555	0.382809194
ENSG00000174705	485.7859801	-0.506929898	0.092906191	-5.45636294	4.86E-08	5.39E-07
ENSG00000174718	341.3670632	-0.30670208	0.111596891	-2.748303101	0.00599046	0.020810514
ENSG00000174720	403.3160112	0.127546754	0.10038384	1.270590508	0.203874365	0.356996162
ENSG00000174721	474.0781486	-0.895466336	0.121815807	-7.350986386	1.97E-13	4.20E-12
ENSG00000174738	91.19613431	0.148295929	0.185301066	0.800297227	0.423538609	0.590750357
ENSG00000174740	20.49702937	-0.566952502	0.387189889	-1.464275073	0.143118805	0.275358151
ENSG00000174744	157.5398996	0.204333085	0.151243359	1.35102186	0.17668843	0.321786903
ENSG00000174748	3597.881235	0.221366894	0.063627033	3.479132744	0.000503039	0.002434939
ENSG00000174749	8.838360786	1.65525657	0.647114796	2.557902523	0.010530561	0.033657061
ENSG00000174775	109.1336539	0.0906504	0.17187758	0.527412589	0.597907103	0.736995531
ENSG00000174780	1258.73888	0.212831279	0.080306841	2.650226012	0.008043794	0.026870075
ENSG00000174791	529.6687258	0.315055889	0.105047616	2.999172186	0.002707143	0.01056509

ENSG00000174796	113.2586237	0.236714823	0.168330141	1.406253341	0.15964891	0.298375231
ENSG00000174799	187.5870629	0.051467912	0.138278251	0.372205402	0.709739925	0.818195874
ENSG00000174804	72.55583069	-0.553048366	0.20909398	-2.644975081	0.008169693	0.027220038
ENSG00000174807	391.1989329	-1.54686665	0.10678872	-14.48530007	1.50E-47	1.47E-45
ENSG00000174839	340.7730359	-0.195747082	0.109635974	-1.78542749	0.074192003	0.166083676
ENSG00000174840	690.7971133	0.446139772	0.081172955	5.496162776	3.88E-08	4.38E-07
ENSG00000174842	82.02144053	-0.120779018	0.194163675	-0.622047445	0.533910671	0.685234734
ENSG00000174851	306.3653459	0.456736124	0.117878359	3.874639306	0.000106783	0.000612236
ENSG00000174871	14.64898598	-0.565520704	0.464358537	-1.217853575	0.223279621	0.381655725
ENSG00000174886	61.72905006	0.039117103	0.223539818	0.17498942	0.861087947	0.921015943
ENSG00000174891	553.4597174	-0.215815037	0.090974056	-2.372270161	0.017679159	0.052236112
ENSG00000174903	720.6681232	0.067986323	0.082505562	0.824021081	0.40992758	0.578312013
ENSG00000174915	249.6804708	0.17872841	0.122754062	1.455987739	0.145396009	0.278594699
ENSG00000174917	186.4167049	0.690508311	0.262538304	2.630124067	0.008535371	0.028290788
ENSG00000174928	13.78539229	0.280767191	0.465352906	0.603342511	0.546280858	0.694729455
ENSG00000174938	1152.05091	-0.600375724	0.083818236	-7.162829401	7.90E-13	1.59E-11
ENSG00000174939	511.9687003	0.246934372	0.120965327	2.041364894	0.041214571	0.104615302
ENSG00000174943	497.607612	0.106476796	0.094417145	1.127727348	0.259435084	0.425585032
ENSG00000174953	431.8133085	-0.073190385	0.10043739	-0.728716524	0.466175083	0.628864054
ENSG00000174989	150.229425	-0.444177726	0.150048286	-2.96023192	0.003074075	0.011785354
ENSG00000174996	365.2826223	0.54652508	0.102479859	5.332999937	9.66E-08	1.02E-06
ENSG00000175003	13.81041866	-0.362653848	0.483510533	-0.750043325	0.453228612	0.617396281
ENSG00000175029	565.8491343	-0.193281547	0.092866984	-2.081272997	0.037408925	0.096455272
ENSG00000175040	71.75331947	-0.157031061	0.20837377	-0.753602824	0.451087748	0.616204376
ENSG00000175048	57.03984437	-0.432714035	0.238922428	-1.811106801	0.070124321	0.159217258
ENSG00000175054	373.3944949	0.118067469	0.106471996	1.10890631	0.267470602	0.43438456
ENSG00000175061	1148.298299	0.27843386	0.085637014	3.251326116	0.00114868	0.00507406
ENSG00000175063	419.1364173	-0.095528524	0.102014039	-0.936425273	0.349054271	0.521003649
ENSG00000175066	199.2737721	-0.142028369	0.133487904	-1.063979313	0.287338071	0.457312246
ENSG00000175073	393.7953769	-0.026497685	0.101672142	-0.260618932	0.794386389	0.877466824
ENSG00000175077	14.89298951	0.310350113	0.442984307	0.700589408	0.48355929	0.644088503
ENSG00000175087	43.58484366	-0.779182457	0.274051508	-2.843197119	0.004466344	0.016270418
ENSG00000175104	121.0641878	-0.285146204	0.165847488	-1.719327848	0.085554692	0.186063904
ENSG00000175105	90.65688928	-0.61698285	0.189789354	-3.250882294	0.001150475	0.00508039
ENSG00000175106	50.23638623	-0.338619593	0.2849637	-1.188290275	0.234719068	0.395667676
ENSG00000175110	287.3647927	-0.024370358	0.111943258	-0.217702777	0.8276607	0.898882778
ENSG00000175115	544.8914729	0.084176306	0.097478038	0.863541241	0.387839955	0.556525506
ENSG00000175130	781.4925907	-0.057985421	0.08154131	-0.711117115	0.477011665	0.637664908
ENSG00000175137	132.3080073	-0.112796577	0.155732199	-0.724298369	0.468882578	0.630937837
ENSG00000175155	73.42398162	0.268898337	0.21149875	1.27139445	0.203588356	0.356584244
ENSG00000175161	8.705095561	-3.850872486	0.953177942	-4.040035251	5.34E-05	0.000325973
ENSG00000175166	956.3242328	0.062036616	0.095638151	0.648659718	0.516558347	0.671487608
ENSG00000175175	99.5965352	0.042247266	0.188886732	0.223664551	0.823018332	0.896348169
ENSG00000175182	72.03828547	0.370834472	0.218144859	1.699945964	0.08914109	0.19187326
ENSG00000175183	20.961567	-0.773762769	0.387301782	-1.997829099	0.045735191	0.113746855
ENSG00000175193	11.50965317	-0.56774336	0.54783785	-1.036334674	0.300046035	0.470406446
ENSG00000175197	121.6849365	-0.597514857	0.17443379	-3.425453622	0.000613774	0.002908799
ENSG00000175198	130.8986087	-0.307273874	0.175531626	-1.750532828	0.080026415	0.176555842
ENSG00000175203	410.4652243	0.328765899	0.097809207	3.361298055	0.000775771	0.003581088
ENSG00000175213	79.89105167	0.298961731	0.211838049	1.411274943	0.15816356	0.296348343

ENSG00000175215	1102.212043	0.064687707	0.074936684	0.863231513	0.388010191	0.556567387
ENSG00000175216	2086.645217	-0.069390791	0.066602887	-1.041858596	0.297477242	0.468106358
ENSG00000175220	400.6933428	0.091540238	0.10284808	0.890052957	0.373437451	0.54353703
ENSG00000175221	276.9628945	0.060192271	0.112027507	0.537299027	0.591061077	0.731961117
ENSG00000175224	267.0612763	-0.171209188	0.119174722	-1.436623344	0.150825047	0.286263942
ENSG00000175265	239.313673	0.199741102	0.125504157	1.59150985	0.11149488	0.228304459
ENSG00000175274	83.23651865	-1.616321071	0.219870194	-7.351251386	1.96E-13	4.20E-12
ENSG00000175283	146.4023102	-0.078994094	0.148783895	-0.530931751	0.595466074	0.735147824
ENSG00000175287	401.742645	-0.216562155	0.114959143	-1.883818458	0.059589536	0.139973162
ENSG00000175305	50.4395541	0.230215537	0.257255195	0.894891691	0.370844989	0.541865594
ENSG00000175309	241.5342112	-0.006356352	0.131466861	-0.048349462	0.961437736	0.979684997
ENSG00000175322	100.4611634	-0.73754668	0.182230272	-4.047333477	5.18E-05	0.000317217
ENSG00000175324	208.3776777	-0.14499948	0.141803999	-1.022534489	0.306527998	0.47752037
ENSG00000175334	529.9987034	0.08830917	0.103904555	0.849906633	0.395376998	0.563217718
ENSG00000175344	19.68265909	-0.150828084	0.389659322	-0.387076801	0.698699352	0.810918182
ENSG00000175348	52.90023024	0.257067161	0.236586447	1.086567568	0.277227965	0.445853623
ENSG00000175352	66.15455942	0.845031349	0.222311121	3.801120459	0.000144043	0.000799571
ENSG00000175354	289.5275373	0.043792355	0.113153072	0.387018702	0.698742363	0.810918182
ENSG00000175376	245.9895614	0.350942674	0.120760832	2.906096856	0.003659681	0.013697721
ENSG00000175387	577.8610261	-0.211840245	0.085660505	-2.473021198	0.01339762	0.041389803
ENSG00000175390	671.755137	-0.272986707	0.081563633	-3.346916947	0.000817157	0.003751177
ENSG00000175395	155.4209969	0.514244445	0.148805604	3.455813711	0.000548634	0.002634758
ENSG00000175414	302.780096	0.392949922	0.112683517	3.487199667	0.000488107	0.002368372
ENSG00000175416	391.6533672	0.645005977	0.109878495	5.87017484	4.35E-09	5.58E-08
ENSG00000175455	505.5039748	-0.068708225	0.089485902	-0.767810609	0.442599711	0.607577494
ENSG00000175467	809.2102419	0.301355457	0.089636832	3.361960156	0.000773913	0.003573687
ENSG00000175470	410.8811541	0.120404238	0.097217753	1.238500524	0.215530528	0.371844427
ENSG00000175471	50.10482736	2.962940431	0.316805157	9.352563771	8.55E-21	3.03E-19
ENSG00000175482	10.72917759	-0.711448239	0.561282836	-1.267539631	0.204962402	0.35845454
ENSG00000175505	28.83322569	0.097921873	0.323661952	0.302543663	0.762237654	0.856640717
ENSG00000175536	15.85802661	-0.661688659	0.455673502	-1.452111338	0.14647065	0.280118886
ENSG00000175548	44.41238991	-0.657911994	0.263568599	-2.496169862	0.012554247	0.039153975
ENSG00000175550	1169.672194	0.484286671	0.078625884	6.159379703	7.30E-10	1.04E-08
ENSG00000175556	99.05320052	2.052875938	0.199051516	10.31328963	6.14E-25	2.73E-23
ENSG00000175564	8.968217818	-0.030140908	0.604530018	-0.049858414	0.960235217	0.978911684
ENSG00000175567	239.2793821	-0.935868862	0.137485214	-6.807050982	9.96E-12	1.76E-10
ENSG00000175573	63.64952926	0.161302911	0.224221028	0.719392431	0.471899159	0.633818202
ENSG00000175575	153.0564678	0.066497385	0.147358532	0.451262535	0.651800344	0.776271126
ENSG00000175581	269.8795834	0.789740099	0.130178929	6.066573952	1.31E-09	1.79E-08
ENSG00000175582	578.7749849	0.659719674	0.096144316	6.861764674	6.80E-12	1.22E-10
ENSG00000175591	10.09213426	1.139575712	1.098060309	1.037807944	0.299359478	0.469797808
ENSG00000175592	185.9474512	0.852776585	0.150754241	5.656733629	1.54E-08	1.84E-07
ENSG00000175595	182.2393195	0.233571242	0.136354365	1.712972245	0.086717646	0.187894921
ENSG00000175600	82.71447028	-0.791037897	0.200474323	-3.945831487	7.95E-05	0.000468372
ENSG00000175602	391.8356164	0.135960976	0.106495275	1.276685529	0.201713292	0.354212163
ENSG00000175606	91.59078322	0.086559425	0.201344744	0.429906555	0.667263617	0.787386763
ENSG00000175611	44.97947596	-0.292147498	0.264598085	-1.10411796	0.269541971	0.436758112
ENSG00000175634	205.9180322	0.209223246	0.130981326	1.597351714	0.110187329	0.226386049
ENSG00000175643	54.29814039	-0.768185563	0.244994626	-3.13552006	0.001715497	0.007177194
ENSG00000175662	128.2972536	-0.048183176	0.15720424	-0.306500484	0.759223612	0.854860493

ENSG00000175691	23.73453633	-0.045818349	0.386124387	-0.118662149	0.905543026	0.949407589
ENSG00000175711	52.71813847	-0.615495011	0.242966429	-2.533251255	0.011300992	0.035777559
ENSG00000175727	694.3302122	0.12044173	0.087983142	1.368918267	0.171024825	0.314052555
ENSG00000175745	3613.992442	0.413711041	0.070295196	5.885338795	3.97E-09	5.12E-08
ENSG00000175756	758.8030045	0.082505893	0.097064121	0.850014323	0.395317123	0.563217718
ENSG00000175764	51.09670507	-0.129795818	0.242124561	-0.536070433	0.591909874	0.732366599
ENSG00000175768	401.3155101	0.314137717	0.116266432	2.701878029	0.006894905	0.023485143
ENSG00000175782	112.4436238	0.029781167	0.180829922	0.164691586	0.86918674	0.927066525
ENSG00000175787	32.03113466	0.372717845	0.303882634	1.226519068	0.220003404	0.377521111
ENSG00000175792	790.9430883	0.005069475	0.077238009	0.065634459	0.947668854	0.970963121
ENSG00000175806	81.23536303	0.58491119	0.202617861	2.886770134	0.003892185	0.014448604
ENSG00000175820	8.91705389	-0.094254696	0.58698153	-0.160575233	0.872427963	0.929183077
ENSG00000175826	626.7163536	0.279649202	0.085677119	3.263989324	0.001098553	0.004883346
ENSG00000175832	772.3413146	-0.092979404	0.080337113	-1.15736551	0.247123059	0.4112472
ENSG00000175854	43.72756706	-0.044215614	0.282217292	-0.156672234	0.875503171	0.931046487
ENSG00000175866	374.4231579	0.549268942	0.108320784	5.070762274	3.96E-07	3.81E-06
ENSG00000175893	160.4178391	0.151097768	0.144107483	1.048507441	0.294404876	0.464663516
ENSG00000175895	68.78009327	0.586109385	0.214300758	2.734985124	0.00623831	0.02154648
ENSG00000175906	10.91924538	-0.602182672	0.538648355	-1.117951381	0.263587759	0.430136762
ENSG00000175931	850.018837	0.067401836	0.075514416	0.892569123	0.37208796	0.542757597
ENSG00000175938	38.621648	-0.84437866	0.286287227	-2.949410876	0.003183804	0.01215296
ENSG00000175970	214.0999767	-0.12991154	0.129355081	-1.004301795	0.315233169	0.486655712
ENSG00000175984	52.68498834	-0.000398606	0.242277566	-0.001645246	0.998687284	0.999398752
ENSG00000176014	851.7430958	0.213663539	0.074997454	2.848943922	0.004386461	0.016028081
ENSG00000176018	87.15825531	-0.010011831	0.189545911	-0.052820083	0.95787526	0.977427995
ENSG00000176022	128.1911678	0.163776699	0.159536454	1.026578532	0.304618967	0.475495347
ENSG00000176024	11.77951136	0.919433236	0.535928989	1.715587802	0.086237513	0.187143263
ENSG00000176043	31.25357577	0.262281801	0.332717699	0.788301319	0.430520476	0.597227052
ENSG00000176046	9.13662738	-0.225587903	0.570089489	-0.395706126	0.692321836	0.806274442
ENSG00000176055	142.1287691	-0.088626109	0.160466579	-0.552302605	0.580741046	0.723518357
ENSG00000176058	88.57989508	0.020640661	0.208479099	0.099005902	0.921133584	0.956727767
ENSG00000176087	492.7348904	0.2320952	0.090628221	2.560959456	0.010438354	0.033461332
ENSG00000176092	14.76917683	-0.205365626	0.458642725	-0.447768196	0.654320498	0.778193213
ENSG00000176095	456.6156025	0.235188772	0.10593098	2.22020765	0.026404675	0.072598644
ENSG00000176101	162.4498564	0.423174743	0.150058675	2.820061831	0.00480144	0.017293801
ENSG00000176102	463.8518972	-0.087264057	0.095660438	-0.912227231	0.361649112	0.532503677
ENSG00000176105	1146.986344	-0.400933706	0.071846733	-5.580402721	2.40E-08	2.79E-07
ENSG00000176108	106.5691847	0.080811071	0.174761573	0.462407547	0.643789088	0.770232972
ENSG00000176124	14.01547206	-0.47729954	0.471833153	-1.011585424	0.311736318	0.483222254
ENSG00000176125	13.35765029	-0.035036954	0.527158418	-0.066463804	0.947008575	0.970640602
ENSG00000176142	230.8476462	0.160172362	0.121376038	1.319637421	0.186956103	0.335227518
ENSG00000176148	145.0681901	0.072719519	0.153988135	0.472241055	0.636754739	0.76559906
ENSG00000176155	668.3242278	-0.100776184	0.101603284	-0.991859514	0.321266061	0.492989334
ENSG00000176170	373.856237	0.795768441	0.110351114	7.211240688	5.54E-13	1.13E-11
ENSG00000176171	573.3995064	0.956799583	0.094642172	10.10965366	5.01E-24	2.09E-22
ENSG00000176182	178.8368893	0.289200356	0.147006262	1.967265553	0.049152604	0.120286869
ENSG00000176204	16.81522523	-6.364551517	1.226090431	-5.190931562	2.09E-07	2.10E-06
ENSG00000176208	292.8277077	-0.483327682	0.111113734	-4.349846446	1.36E-05	9.50E-05
ENSG00000176209	55.38867264	-0.208938066	0.242121921	-0.862945681	0.388167333	0.556670396
ENSG00000176225	193.1384899	-0.715183417	0.138373359	-5.168505143	2.36E-07	2.35E-06

ENSG00000176244	58.92300285	-1.127832578	0.244371816	-4.615231814	3.93E-06	3.08E-05
ENSG00000176248	90.84204186	0.015330242	0.188410464	0.081366189	0.935150737	0.96468109
ENSG00000176261	327.3104206	0.584266363	0.113626167	5.142005395	2.72E-07	2.67E-06
ENSG00000176273	109.317161	0.076075253	0.170601036	0.44592492	0.655651481	0.779180728
ENSG00000176340	167.2589445	-0.064698687	0.142063502	-0.455420894	0.648806466	0.773893979
ENSG00000176371	59.79890593	0.047749297	0.229780721	0.207803756	0.8353822	0.903705334
ENSG00000176383	9.295804981	0.283377945	0.560451316	0.50562455	0.613120244	0.748392428
ENSG00000176386	90.11892627	0.366985464	0.19204704	1.91091445	0.056015576	0.133341941
ENSG00000176387	17.71675055	0.244733303	0.414349365	0.590644813	0.554758432	0.701854606
ENSG00000176390	95.21025601	0.249777744	0.181597457	1.375447363	0.168992794	0.311407528
ENSG00000176393	598.9048122	0.053927459	0.084135414	0.640960286	0.521548486	0.675847931
ENSG00000176396	70.29128079	-0.532539189	0.213894764	-2.489725221	0.012784189	0.039773977
ENSG00000176401	14.79705321	-0.144155325	0.454837813	-0.316937864	0.751290751	0.8494267
ENSG00000176406	265.5074846	-0.456584989	0.12124113	-3.765924885	0.000165934	0.000905342
ENSG00000176407	361.349132	0.005632713	0.104909229	0.053691303	0.957181112	0.977003566
ENSG00000176410	82.56113838	-0.017379669	0.202895997	-0.085658019	0.931738275	0.96307046
ENSG00000176422	92.39203376	0.236983303	0.195318784	1.213315477	0.225009228	0.383162156
ENSG00000176428	12.92681042	0.179521477	0.491176869	0.365492531	0.714743781	0.822277305
ENSG00000176438	31.70610592	-0.381704594	0.317355552	-1.202766396	0.229066732	0.387861666
ENSG00000176444	726.5426734	0.164213186	0.079585304	2.063360657	0.039078375	0.099961736
ENSG00000176454	243.6432118	0.161967075	0.123950672	1.306705906	0.191312612	0.341469919
ENSG00000176472	29.93264369	1.295959935	0.338294253	3.83086595	0.000127693	0.000718344
ENSG00000176473	23.53924192	-1.414909267	0.387898355	-3.647628942	0.000264671	0.001373925
ENSG00000176476	62.2649418	-0.103649235	0.222849593	-0.465108479	0.641853775	0.768834312
ENSG00000176485	89.96543238	0.006122873	0.204822814	0.029893509	0.976151982	0.987958767
ENSG00000176490	171.0439466	0.772089092	0.149201092	5.174822007	2.28E-07	2.27E-06
ENSG00000176531	127.7792885	0.338387899	0.172541484	1.961197338	0.049856007	0.121584607
ENSG00000176533	19.15790697	-1.082470951	0.430109526	-2.516733264	0.011844846	0.037222496
ENSG00000176542	1052.979285	-0.051703627	0.08507775	-0.607722076	0.543371819	0.692312375
ENSG00000176571	10.62434378	-6.689122008	1.252477214	-5.340713533	9.26E-08	9.81E-07
ENSG00000176593	30.77305071	0.380799751	0.340922542	1.11696853	0.264007781	0.430572077
ENSG00000176595	29.97462534	1.201792222	0.338513471	3.550205017	0.000384931	0.001919464
ENSG00000176597	42.34278232	0.507391619	0.287885777	1.762475465	0.077988998	0.173148642
ENSG00000176619	2078.586281	0.259867816	0.079036308	3.287954916	0.00100918	0.004531954
ENSG00000176623	181.6031236	0.186886269	0.135587843	1.378340896	0.168098062	0.310367177
ENSG00000176624	362.6292443	0.090186794	0.10225844	0.881949641	0.377804041	0.547235502
ENSG00000176714	60.8429063	-0.440183742	0.223793755	-1.966917	0.04919278	0.120322303
ENSG00000176715	213.1364042	-1.441168387	0.139102253	-10.36049638	3.75E-25	1.69E-23
ENSG00000176720	129.3896927	0.153655989	0.167615641	0.916716298	0.359291324	0.530567328
ENSG00000176723	7.997288098	-1.719178241	0.706388545	-2.433757249	0.014943013	0.045374946
ENSG00000176731	349.879044	0.412036005	0.112640812	3.657963733	0.000254227	0.001326078
ENSG00000176749	37.97456045	-0.151036576	0.279300495	-0.540767307	0.58866798	0.730091746
ENSG00000176783	181.8011042	0.045171747	0.146025866	0.309340721	0.757062358	0.853213651
ENSG00000176788	37.43045625	2.637938264	0.343456149	7.680567863	1.58E-14	3.69E-13
ENSG00000176809	42.96208159	-0.246391896	0.291923046	-0.8440303	0.398652474	0.566731232
ENSG00000176834	148.9819955	-0.143271212	0.150032544	-0.954934231	0.339610957	0.511418859
ENSG00000176840	75.95164835	-0.29554962	0.219310153	-1.347633092	0.177776421	0.323295139
ENSG00000176845	56.69451865	1.127159542	0.250953412	4.49150913	7.07E-06	5.25E-05
ENSG00000176853	406.7664133	0.092205347	0.108005828	0.853707146	0.393267274	0.561290936
ENSG00000176871	319.3461898	-0.095560659	0.108468059	-0.881002755	0.378316329	0.547630819

ENSG00000176884	40.10718891	-1.020613652	0.2834183	-3.601085923	0.000316891	0.001613398
ENSG00000176890	1156.145752	0.117726014	0.068493238	1.718797598	0.085651233	0.18614828
ENSG00000176894	12.41738834	-0.432814987	0.505300713	-0.856549329	0.391694	0.56006979
ENSG00000176903	771.4944438	0.01987092	0.083677273	0.237470937	0.812291463	0.889524775
ENSG00000176907	53.35968882	-9.017917239	1.194045195	-7.552408634	4.27E-14	9.74E-13
ENSG00000176909	119.7396496	0.136841313	0.187654598	0.729219076	0.465867664	0.628509708
ENSG00000176912	33.06173876	-0.65404081	0.306011086	-2.137310836	0.032572718	0.086102554
ENSG00000176915	635.5394434	0.010530227	0.093273238	0.112896557	0.910112566	0.951381168
ENSG00000176919	9.777221089	0.365775237	0.562408692	0.65037266	0.515451533	0.671096242
ENSG00000176945	17.24122717	-1.401866734	0.467104119	-3.001186835	0.002689295	0.010510998
ENSG00000176946	543.2143576	0.360436646	0.097519665	3.696040668	0.000218988	0.001160363
ENSG00000176953	391.8379039	0.233049835	0.113022653	2.061974554	0.039210159	0.100160956
ENSG00000176971	12.77178597	2.933944036	0.609830021	4.811084951	1.50E-06	1.28E-05
ENSG00000176973	59.23046982	-0.695489742	0.2488711	-2.794578171	0.005196748	0.018494733
ENSG00000176974	211.8837112	-0.497800377	0.138030397	-3.60645471	0.000310409	0.001584993
ENSG00000176978	187.9087616	0.299735376	0.136742931	2.191962491	0.028382215	0.077025691
ENSG00000176986	562.9736116	0.212505066	0.088140162	2.410990188	0.015909276	0.047894901
ENSG00000176994	588.7716903	-0.015937363	0.086568982	-0.184100155	0.853934885	0.91622543
ENSG00000177000	320.334854	0.113409757	0.109821138	1.032676937	0.30175511	0.47217935
ENSG00000177030	347.0194975	0.478959365	0.108049414	4.432780786	9.30E-06	6.70E-05
ENSG00000177034	291.2265254	-0.18188582	0.113979346	-1.595778761	0.110538197	0.226791078
ENSG00000177042	127.8333239	0.483876487	0.163378322	2.961693326	0.003059524	0.011742384
ENSG00000177045	327.3352804	0.021425501	0.111425545	0.192285363	0.847518683	0.912267812
ENSG00000177051	226.0590039	0.023964737	0.133385467	0.179665281	0.857415352	0.918346431
ENSG00000177054	23.72639844	-0.455092643	0.356730175	-1.275733523	0.202049734	0.354594883
ENSG00000177058	52.49137156	-0.029537157	0.248646997	-0.11879153	0.90544052	0.949370977
ENSG00000177076	23.50644747	-0.564697551	0.359606517	-1.570320682	0.116340525	0.235786373
ENSG00000177082	285.8337135	0.034096699	0.114459982	0.297891876	0.765785689	0.858773078
ENSG00000177084	1095.433185	-0.138749525	0.072617617	-1.910686843	0.056044837	0.133366393
ENSG00000177098	9.715669434	-0.485814813	0.565308917	-0.859379357	0.390131255	0.558517503
ENSG00000177105	140.5231156	0.453256555	0.156559977	2.895098492	0.003790396	0.014115506
ENSG00000177106	193.1967945	1.739587619	0.146598122	11.86637042	1.77E-32	1.05E-30
ENSG00000177119	552.1939468	-0.053618233	0.08993228	-0.596206759	0.551037111	0.698621617
ENSG00000177125	143.3383954	-0.808302473	0.163580137	-4.941324084	7.76E-07	7.05E-06
ENSG00000177150	144.4424985	0.14174705	0.148261857	0.956058776	0.33904254	0.511001134
ENSG00000177156	763.0870137	0.428281325	0.08558247	5.004311339	5.61E-07	5.25E-06
ENSG00000177169	319.8260705	0.108519015	0.113451047	0.956527227	0.338805936	0.510699322
ENSG00000177181	29.24256007	0.49440936	0.320786976	1.541238884	0.123258657	0.247062551
ENSG00000177189	384.8994112	0.226837057	0.100995418	2.246013356	0.024703152	0.068591655
ENSG00000177192	321.2315579	0.424928296	0.112784144	3.76762443	0.000164808	0.000899753
ENSG00000177200	1058.602525	-0.102842392	0.071378718	-1.440799101	0.149641448	0.284555762
ENSG00000177225	251.7260754	0.447771488	0.128966827	3.471989659	0.000516616	0.002492926
ENSG00000177239	270.3353777	0.386289817	0.113569836	3.401341691	0.00067056	0.003145025
ENSG00000177283	47.71797655	-5.224333962	0.589225168	-8.8664474	7.55E-19	2.42E-17
ENSG00000177302	344.0402441	0.035111564	0.104025306	0.337529059	0.73571811	0.83739245
ENSG00000177303	206.2268754	-0.321459181	0.130770366	-2.458195926	0.013963697	0.042780382
ENSG00000177311	396.0927907	0.253216689	0.101708266	2.48963727	0.012787353	0.03977501
ENSG00000177335	48.89813204	0.180619718	0.251145779	0.719182773	0.472028312	0.633818202
ENSG00000177337	49.45127839	-0.128259223	0.264522343	-0.484871038	0.627767828	0.758494295
ENSG00000177352	252.8511053	0.001109139	0.121779538	0.00910776	0.992733159	0.996284349

ENSG00000177370	353.6010822	0.197131007	0.11122548	1.772354744	0.076335689	0.170015447
ENSG00000177374	359.6447956	0.165889862	0.117044548	1.417322423	0.156388682	0.29404254
ENSG00000177380	239.1196314	0.029283847	0.122029823	0.239972873	0.810351286	0.888256303
ENSG00000177383	190.0948068	-0.50560106	0.138493012	-3.65073338	0.000261493	0.001359432
ENSG00000177410	276.0592794	0.124816724	0.121387573	1.028249604	0.30383243	0.474638867
ENSG00000177425	325.9307528	-0.67754086	0.10682241	-6.342684662	2.26E-10	3.42E-09
ENSG00000177426	164.2577163	0.017026338	0.145506424	0.117014337	0.906848692	0.950209129
ENSG00000177427	33.67146289	-0.008188848	0.296026058	-0.027662591	0.97793126	0.988629033
ENSG00000177432	44.45683513	1.235488084	0.277044994	4.459521421	8.21E-06	6.00E-05
ENSG00000177463	604.2143645	0.185470732	0.088475844	2.096286669	0.036056769	0.093707572
ENSG00000177469	608.3583924	-0.497357896	0.08589391	-5.790374405	7.02E-09	8.84E-08
ENSG00000177479	1199.17298	0.024761479	0.071562059	0.34601406	0.729332148	0.832773591
ENSG00000177485	352.9668376	0.37717452	0.116091562	3.248939995	0.001158359	0.005103974
ENSG00000177542	251.72216	0.172675373	0.125055995	1.380784445	0.167345248	0.309465336
ENSG00000177548	135.262958	0.449337463	0.155470527	2.890177789	0.00385024	0.014315596
ENSG00000177556	197.2027913	0.408117851	0.146236963	2.79079818	0.005257825	0.018678974
ENSG00000177565	631.9088529	-0.107718084	0.085403528	-1.261283766	0.207206628	0.361165344
ENSG00000177576	15.10810501	0.149376874	0.4598846	0.32481382	0.745321984	0.844658162
ENSG00000177595	206.4448591	-0.00240619	0.138439708	-0.017380782	0.986132841	0.992634039
ENSG00000177600	5172.543972	0.378090187	0.060024601	6.298920475	3.00E-10	4.46E-09
ENSG00000177602	136.0566054	-0.133467978	0.155365509	-0.859057964	0.390308537	0.558600512
ENSG00000177606	10.66204261	0.149351185	0.528717799	0.282478073	0.777576957	0.866874883
ENSG00000177613	242.3502873	-0.24493922	0.123185475	-1.988377448	0.046769961	0.115787389
ENSG00000177628	285.6428445	0.35386692	0.114453236	3.091803547	0.001989445	0.008135583
ENSG00000177640	18.56656992	-1.216627683	0.42428971	-2.867445646	0.004137999	0.015260296
ENSG00000177646	439.9374956	0.035919088	0.096175963	0.373472612	0.708796725	0.817578223
ENSG00000177663	281.4242941	-0.001568398	0.128777538	-0.012179128	0.990282702	0.994957522
ENSG00000177666	624.4144609	0.096043249	0.097883483	0.98119975	0.326494247	0.498669641
ENSG00000177674	199.7824809	-0.019293144	0.131660629	-0.146536925	0.883497543	0.93565214
ENSG00000177679	35.48115265	0.038517811	0.295777691	0.130225546	0.896387984	0.943824452
ENSG00000177683	181.1698551	0.714059145	0.143672568	4.970045109	6.69E-07	6.17E-06
ENSG00000177685	342.9445674	0.049715993	0.107015226	0.464569346	0.642239888	0.768968949
ENSG00000177688	8.989427497	-0.725645591	0.577923972	-1.255607358	0.209258362	0.363973776
ENSG00000177697	376.2094788	0.747101909	0.132495089	5.638713971	1.71E-08	2.03E-07
ENSG00000177700	1446.88331	0.059895303	0.070501956	0.849555201	0.395572428	0.563436006
ENSG00000177706	177.8110803	0.060777465	0.136491777	0.445282981	0.656115265	0.779535747
ENSG00000177707	13.9276758	-0.968513971	0.48134334	-2.012106308	0.044208732	0.110714933
ENSG00000177721	42.95864155	-0.361367277	0.2655536	-1.360807301	0.173574594	0.317515604
ENSG00000177728	832.4127201	-0.177102068	0.082834367	-2.138026444	0.032514597	0.085981277
ENSG00000177731	742.0851292	-0.0118696	0.081922852	-0.144887538	0.884799666	0.936819244
ENSG00000177732	616.9172089	-0.06539083	0.087064915	-0.751058341	0.452617546	0.617273657
ENSG00000177733	778.8185882	-0.050074673	0.193694406	-0.258524108	0.796002446	0.878339214
ENSG00000177738	88.59717528	-0.493102831	0.194166505	-2.53958751	0.011098328	0.035239197
ENSG00000177788	79.83152435	0.056359891	0.207159111	0.272060885	0.785575204	0.871640987
ENSG00000177830	363.4113242	0.552487579	0.10588222	5.217944781	1.81E-07	1.83E-06
ENSG00000177842	39.85114885	-0.762708624	0.278264233	-2.740950987	0.006126165	0.021221759
ENSG00000177853	347.9058532	-0.764030666	0.108461833	-7.044235213	1.86E-12	3.63E-11
ENSG00000177854	13.02598853	0.310790427	0.493499734	0.629768174	0.528846271	0.681469917
ENSG00000177855	8.133479861	-0.133173351	0.595408419	-0.223667229	0.823016247	0.896348169
ENSG00000177868	49.72546831	-1.264053265	0.262348989	-4.818212832	1.45E-06	1.24E-05

ENSG00000177873	44.36587891	-0.310067508	0.268220781	-1.156015976	0.247674622	0.411921077
ENSG00000177875	25.13707411	0.334811552	0.345132977	0.970094351	0.331999464	0.504440134
ENSG00000177879	253.5850417	0.172673584	0.116731722	1.479234438	0.139077668	0.269667102
ENSG00000177885	1155.306467	0.069816225	0.071660481	0.974263974	0.329925487	0.502255933
ENSG00000177888	326.2193677	-0.284620315	0.10682032	-2.664477265	0.007710808	0.025924777
ENSG00000177889	355.5510045	-0.116688439	0.105999158	-1.100843083	0.270964957	0.438557985
ENSG00000177917	176.0709605	-0.364046661	0.141332543	-2.575816254	0.010000377	0.032297953
ENSG00000177943	44.8057964	0.190249191	0.274192597	0.693852399	0.487774778	0.647887711
ENSG00000177946	42.25232753	0.228259691	0.268750047	0.849338237	0.39569311	0.563477628
ENSG00000177951	257.8693559	0.170360507	0.119735163	1.422811002	0.154790951	0.292054868
ENSG00000177954	323.4579348	0.213018204	0.124542626	1.710403984	0.087191195	0.188571935
ENSG00000177963	331.0527704	0.241614308	0.109300026	2.2105604	0.027066294	0.07409866
ENSG00000177971	267.3986013	0.024734137	0.11634326	0.212596218	0.831641921	0.901477895
ENSG00000177981	68.46288692	0.468379208	0.217449318	2.153969542	0.031242561	0.08322857
ENSG00000177990	108.3067386	0.386813949	0.188304872	2.054189802	0.039957325	0.101754994
ENSG00000178026	76.28858057	0.114440087	0.210235643	0.544341984	0.586206161	0.728067015
ENSG00000178028	153.3182384	0.21088029	0.146398429	1.440454598	0.149738826	0.284663864
ENSG00000178033	31.02920329	-5.081448845	0.697368	-7.286610289	3.18E-13	6.63E-12
ENSG00000178035	32.99246283	0.119398486	0.314149695	0.380068764	0.703894372	0.814364144
ENSG00000178038	29.39945378	-0.783181642	0.33817106	-2.315933371	0.020561907	0.059126532
ENSG00000178053	189.4333532	0.65634417	0.132286405	4.961539082	6.99E-07	6.41E-06
ENSG00000178057	2130.776561	0.362590404	0.079581426	4.556218979	5.21E-06	3.98E-05
ENSG00000178074	171.1381193	-0.003273262	0.14491871	-0.022586883	0.981979807	0.990867779
ENSG00000178075	20.51619942	0.483143705	0.38641503	1.250323273	0.21118148	0.366275621
ENSG00000178078	41.62922548	0.647275575	0.277489469	2.332613119	0.01966846	0.057022202
ENSG00000178093	11.70060646	-0.088489443	0.501691285	-0.17638226	0.859993641	0.920195816
ENSG00000178096	79.57623935	0.402350556	0.204724323	1.965328548	0.049376223	0.120665938
ENSG00000178104	156.6865427	0.090573704	0.14421477	0.628047351	0.529972921	0.682240213
ENSG00000178105	725.0073762	-1.040494389	0.082115375	-12.67112758	8.55E-37	5.91E-35
ENSG00000178127	39.16522428	-0.118339022	0.283980727	-0.416714979	0.676886897	0.794803163
ENSG00000178149	92.45628072	0.054245306	0.195396612	0.277616412	0.781306832	0.868895343
ENSG00000178150	136.2395498	0.12746509	0.166158029	0.767131696	0.443003218	0.607879868
ENSG00000178163	259.6910972	-0.201735668	0.126372972	-1.596351382	0.110410364	0.226645729
ENSG00000178177	259.513941	-0.189478885	0.117465033	-1.613066285	0.106730108	0.220746286
ENSG00000178184	37.5239994	-0.045441281	0.295768755	-0.153637867	0.877895278	0.932742982
ENSG00000178188	253.7975129	0.015157152	0.12638589	0.119927568	0.904540526	0.94878142
ENSG00000178202	785.029584	0.03937423	0.081594758	0.48255833	0.629409372	0.759693543
ENSG00000178209	4975.216123	0.161895689	0.082227411	1.968877369	0.048967175	0.119979401
ENSG00000178229	78.40791737	0.24847287	0.19774604	1.25652514	0.208925637	0.363485066
ENSG00000178234	313.6642161	0.346210472	0.112362864	3.081182344	0.002061803	0.008375406
ENSG00000178235	6.332109092	-2.06029936	0.821159592	-2.509012108	0.012106933	0.037944242
ENSG00000178252	1545.889648	-0.097971967	0.066558036	-1.47197803	0.14102681	0.272302901
ENSG00000178295	350.8563909	-0.159585986	0.107333408	-1.486824918	0.137061059	0.266735479
ENSG00000178307	237.0532465	0.04725182	0.120509073	0.392101763	0.694983025	0.808537739
ENSG00000178338	118.3785454	0.150752966	0.164675275	0.915455981	0.359952301	0.531230298
ENSG00000178343	10.92995859	0.63158345	0.515062249	1.226227414	0.220113108	0.377663225
ENSG00000178381	33.72249311	0.304663789	0.308558627	0.987377316	0.3234577	0.495216383
ENSG00000178385	114.3427921	0.078694766	0.167056409	0.471067024	0.637592873	0.76614774
ENSG00000178394	7.208321976	-6.130694863	1.286884847	-4.763980924	1.90E-06	1.59E-05
ENSG00000178401	35.28313283	-2.357886722	0.361816479	-6.516803027	7.18E-11	1.16E-09

ENSG00000178404	20.47607937	0.419254503	0.390299938	1.074185419	0.282739586	0.452173323
ENSG00000178409	230.4026424	0.087801828	0.126736295	0.692791499	0.488440408	0.648303739
ENSG00000178425	146.4171653	-1.65807599	0.171186062	-9.685811837	3.46E-22	1.31E-20
ENSG00000178445	567.2249633	0.468728776	0.089357068	5.245570229	1.56E-07	1.59E-06
ENSG00000178449	37.82622755	-0.070817161	0.284245992	-0.249140404	0.803252177	0.883231571
ENSG00000178464	9.785319921	0.116416217	0.5453893	0.213455264	0.830971879	0.901433469
ENSG00000178498	265.8555095	-0.236407177	0.116466985	-2.029821391	0.042374698	0.107192037
ENSG00000178502	291.9487492	0.193425473	0.112391893	1.720991324	0.085252398	0.185521369
ENSG00000178522	42.05558665	-8.674064011	1.199275783	-7.232751747	4.73E-13	9.75E-12
ENSG00000178531	250.5070308	0.315811303	0.266012002	1.187206973	0.235145991	0.396149644
ENSG00000178537	52.48629514	0.420246218	0.258327277	1.626797691	0.103780072	0.215867235
ENSG00000178538	7.960095128	-3.838111999	0.976136217	-3.931943036	8.43E-05	0.000495242
ENSG00000178568	530.3020765	-0.562170381	0.08828035	-6.368012593	1.91E-10	2.91E-09
ENSG00000178585	58.13936366	-0.701476816	0.231339688	-3.032237231	0.002427483	0.009635168
ENSG00000178605	205.4831665	0.266776783	0.127480259	2.092690929	0.036376753	0.094451803
ENSG00000178607	145.8184242	-0.104969886	0.153174115	-0.685297813	0.493156012	0.652452163
ENSG00000178662	10.46525671	-1.828176373	0.619386158	-2.951593849	0.003161385	0.012083801
ENSG00000178665	35.68449684	-0.395602129	0.291338362	-1.357878607	0.174502207	0.318907337
ENSG00000178685	39.65571597	1.989162547	0.306940663	6.480609394	9.14E-11	1.45E-09
ENSG00000178691	653.0151358	0.027435655	0.084745601	0.323741346	0.746133865	0.845169132
ENSG00000178694	171.7810403	-0.654877551	0.142929603	-4.581818874	4.61E-06	3.55E-05
ENSG00000178700	42.85232558	0.333249181	0.270677916	1.231165017	0.218261152	0.375264921
ENSG00000178715	97.63302377	0.134616524	0.179260693	0.750953943	0.452680375	0.61729941
ENSG00000178718	319.0604201	0.143530026	0.116475274	1.232278931	0.217844907	0.37491637
ENSG00000178719	1359.187402	0.531446613	0.078864465	6.73873351	1.60E-11	2.76E-10
ENSG00000178741	1483.609453	0.325906984	0.072240516	4.511415531	6.44E-06	4.81E-05
ENSG00000178752	51.7147349	0.106534689	0.250172889	0.425844261	0.670221333	0.789550408
ENSG00000178761	204.7194576	0.114980685	0.133720065	0.859861121	0.389865602	0.558214797
ENSG00000178773	346.7597757	0.508269218	0.115297243	4.40833802	1.04E-05	7.44E-05
ENSG00000178802	331.6415869	0.366865583	0.106293549	3.45143789	0.000557608	0.00266782
ENSG00000178814	286.15941	1.365444455	0.125748231	10.85855793	1.82E-27	8.98E-26
ENSG00000178821	86.90751313	0.491704448	0.199086232	2.469806384	0.01351862	0.041643871
ENSG00000178878	17.5532305	-1.039507558	0.457745373	-2.27092969	0.023151235	0.065015072
ENSG00000178882	11.28008322	0.457315853	0.52209158	0.875930336	0.381067907	0.550538001
ENSG00000178904	183.7509666	0.181679002	0.139596814	1.301455218	0.193102683	0.343834883
ENSG00000178913	472.6617913	-0.000539948	0.095301112	-0.005665702	0.995479448	0.997940364
ENSG00000178917	96.73539004	-0.05229192	0.179878535	-0.290706836	0.771275543	0.863328623
ENSG00000178919	172.2305736	-1.416159739	0.165122548	-8.576416463	9.79E-18	2.85E-16
ENSG00000178921	481.4836654	0.110110527	0.092600805	1.189088229	0.23440495	0.39518559
ENSG00000178927	265.0199145	-0.262732587	0.117141731	-2.242860711	0.024905798	0.06903152
ENSG00000178935	21.74715027	0.582705256	0.367982578	1.583513165	0.113304549	0.23123478
ENSG00000178947	99.03387221	1.088639506	0.187356437	5.810526305	6.23E-09	7.89E-08
ENSG00000178950	583.6884212	0.048983774	0.091607451	0.534713867	0.592847731	0.733089931
ENSG00000178951	928.7003372	0.329837566	0.073531562	4.485659726	7.27E-06	5.37E-05
ENSG00000178952	3549.012632	0.178868646	0.059389379	3.011795168	0.002597078	0.010213088
ENSG00000178966	66.00056253	-0.769134775	0.224547165	-3.425270474	0.000614188	0.002909779
ENSG00000178971	157.975818	0.134690011	0.144076462	0.934850908	0.34986514	0.521881238
ENSG00000178974	232.729146	0.043877644	0.129432132	0.339001173	0.734608847	0.83646822
ENSG00000178980	518.3078271	0.133232757	0.095495331	1.395175622	0.162962887	0.303277645
ENSG00000178982	1111.956806	0.159403372	0.089160323	1.787828557	0.073803672	0.165346121

ENSG00000178988	212.0539705	-0.345309087	0.134953018	-2.558735573	0.010505362	0.033591519
ENSG00000178996	238.2746192	-0.0985821	0.124266503	-0.793311935	0.427596105	0.594815061
ENSG00000178999	356.6509091	-0.264115255	0.102748931	-2.570491518	0.010155431	0.032711154
ENSG00000179010	1526.980451	-0.019394558	0.065242787	-0.297267457	0.766262325	0.859082439
ENSG00000179021	113.4272335	-0.108546147	0.176373155	-0.615434629	0.538267728	0.688933648
ENSG00000179029	93.88159439	0.114270926	0.182475694	0.62622546	0.53116707	0.683094573
ENSG00000179041	390.3079331	0.086220352	0.107480919	0.802192169	0.422441806	0.589928798
ENSG00000179046	7.239609575	-1.991839146	0.736778604	-2.703443255	0.006862516	0.023403197
ENSG00000179051	1177.913389	-0.202439593	0.072944351	-2.775260732	0.005515747	0.019433082
ENSG00000179071	11.80741267	-0.827932158	0.520657018	-1.590168055	0.111796929	0.228777697
ENSG00000179082	20.3254309	-0.969684436	0.404424346	-2.397690557	0.016498797	0.049320834
ENSG00000179083	24.50955071	-3.48499165	0.520557733	-6.694726505	2.16E-11	3.67E-10
ENSG00000179085	408.7248682	0.572509853	0.127999472	4.472751677	7.72E-06	5.68E-05
ENSG00000179091	1345.291284	0.341242209	0.070976951	4.807789075	1.53E-06	1.30E-05
ENSG00000179094	759.2168257	0.055215546	0.080767437	0.683636223	0.494204906	0.65345363
ENSG00000179097	14.26991445	-7.115913797	1.235550936	-5.759304284	8.45E-09	1.05E-07
ENSG00000179104	243.1278	0.494784363	0.120235981	4.115110609	3.87E-05	0.000244319
ENSG00000179111	78.61845046	-0.948365213	0.210432951	-4.506733415	6.58E-06	4.90E-05
ENSG00000179115	778.669962	0.302567897	0.084752557	3.570014956	0.000356961	0.001795284
ENSG00000179119	173.9260565	0.087499348	0.150879525	0.579928575	0.561962785	0.70822638
ENSG00000179134	1221.620679	0.362776039	0.06920215	5.242265434	1.59E-07	1.62E-06
ENSG00000179151	237.1087651	-0.100113687	0.120493747	-0.830862096	0.406051538	0.574343567
ENSG00000179152	336.9706419	0.075350756	0.1068118	0.705453478	0.480528074	0.640992373
ENSG00000179163	89.66328334	0.350551463	0.188837153	1.856369134	0.063400961	0.147181177
ENSG00000179168	17.60648376	0.350267899	0.412000487	0.850163798	0.395234026	0.563217718
ENSG00000179195	1312.265649	0.712406194	0.069564471	10.24094891	1.30E-24	5.63E-23
ENSG00000179218	5994.565454	-0.51749917	0.058043257	-8.915750087	4.85E-19	1.56E-17
ENSG00000179222	133.6015919	0.109838334	0.154725775	0.709890345	0.477772138	0.638134946
ENSG00000179240	31.33682152	-0.837355467	0.332006253	-2.522107516	0.011665405	0.036773775
ENSG00000179241	288.357528	0.254632955	0.110473089	2.304931979	0.021170383	0.060529284
ENSG00000179262	1142.350149	0.305806128	0.072879507	4.196051018	2.72E-05	0.000177208
ENSG00000179271	1746.733878	0.421941985	0.07045196	5.989073761	2.11E-09	2.83E-08
ENSG00000179295	1285.217816	0.064862971	0.0697999	0.92927026	0.352749044	0.524165716
ENSG00000179299	47.62671912	-3.544928608	0.378301245	-9.370650131	7.21E-21	2.56E-19
ENSG00000179331	56.03937149	-0.253543987	0.241095364	-1.051633607	0.292967683	0.463541004
ENSG00000179335	267.5192519	0.540374122	0.117393587	4.603097471	4.16E-06	3.23E-05
ENSG00000179348	628.5342286	0.329016901	0.106011308	3.103601942	0.001911804	0.007872914
ENSG00000179361	412.0855778	-0.172875026	0.098852533	-1.748817371	0.080322593	0.177070224
ENSG00000179364	291.9788248	-0.153542586	0.116097953	-1.322526214	0.185992986	0.334012206
ENSG00000179387	134.1595126	-0.354092719	0.159812397	-2.215677426	0.026713602	0.073333197
ENSG00000179388	652.5898005	0.21925878	0.0933491	2.348804432	0.018833796	0.054956031
ENSG00000179399	12.83256612	-5.136688916	1.06998045	-4.800731559	1.58E-06	1.34E-05
ENSG00000179403	463.5586496	0.01335015	0.092525604	0.144286012	0.885274623	0.937110221
ENSG00000179406	73.76231281	0.096973526	0.218113509	0.44460119	0.656607987	0.779779681
ENSG00000179409	484.9678746	0.226197943	0.093544628	2.418075174	0.015602853	0.047086843
ENSG00000179431	131.8754038	0.397825145	0.167581142	2.373925485	0.017600102	0.052070057
ENSG00000179454	81.33855136	0.166834476	0.194586301	0.857380379	0.391234699	0.559526962
ENSG00000179455	90.11490151	-1.158863898	0.195573397	-5.925467949	3.11E-09	4.07E-08
ENSG00000179476	18.18472299	0.504157874	0.407138402	1.238296048	0.215606309	0.371929488
ENSG00000179477	14.01880247	-0.262485398	0.456423627	-0.57509161	0.565229342	0.710947988

ENSG00000179523	43.48455817	-0.597199964	0.272656342	-2.19030285	0.02850228	0.077247064
ENSG00000179526	143.688229	-0.061742253	0.153301486	-0.402750518	0.687131766	0.802739514
ENSG00000179532	238.2806363	-0.083983269	0.132780687	-0.632496113	0.527062747	0.68011559
ENSG00000179542	18.56914143	-0.067638302	0.399732818	-0.169208779	0.865632425	0.924539133
ENSG00000179562	164.6673844	0.728623374	0.146714134	4.96627935	6.82E-07	6.28E-06
ENSG00000179588	68.62994623	-0.500576819	0.21891023	-2.286676231	0.022214728	0.062736286
ENSG00000179598	109.8906167	0.184421065	0.180568381	1.021336428	0.307095073	0.478138383
ENSG00000179604	283.0572306	0.158395399	0.114514623	1.383189282	0.166606835	0.30849621
ENSG00000179627	82.51142471	-0.115790649	0.209363731	-0.553059731	0.58022251	0.723159339
ENSG00000179630	34.56225317	0.847806317	0.306800546	2.763379425	0.005720622	0.020018593
ENSG00000179632	513.753259	0.197809482	0.08938974	2.212887978	0.02690537	0.073773127
ENSG00000179698	9.494008775	-0.993433669	0.59291675	-1.675502789	0.093835624	0.199616702
ENSG00000179743	13.85860778	-0.56561236	0.470865489	-1.20121855	0.229666435	0.388596051
ENSG00000179750	330.8502566	-0.094464968	0.121734592	-0.775991169	0.4377542	0.603314879
ENSG00000179761	9.01350524	-1.186289852	0.601904871	-1.970892593	0.048736163	0.11958024
ENSG00000179818	158.0451049	-0.206864413	0.145785906	-1.4189603	0.155910591	0.293418551
ENSG00000179820	922.2303057	-0.309843007	0.076323004	-4.05962803	4.92E-05	0.000303349
ENSG00000179832	221.8876979	-0.167391127	0.125965383	-1.328866101	0.18389215	0.331298323
ENSG00000179833	587.3988796	-0.006566252	0.092078509	-0.071311454	0.943149879	0.968637121
ENSG00000179841	23.64806206	-0.965988857	0.377251201	-2.560598496	0.010449204	0.033462203
ENSG00000179855	29.76730596	-2.398031594	0.391018099	-6.132789245	8.64E-10	1.21E-08
ENSG00000179859	20.96253511	0.317846609	0.398688511	0.797230422	0.425317221	0.592583912
ENSG00000179862	43.62516425	0.929333443	0.2852154	3.258356463	0.001120595	0.004967184
ENSG00000179886	110.11086	0.197316931	0.179981653	1.096316917	0.272940109	0.440962434
ENSG00000179889	536.6588418	-0.124069686	0.090626241	-1.369026073	0.170991125	0.314052555
ENSG00000179899	59.86841401	-0.328725455	0.238978672	-1.375543063	0.168963145	0.311407528
ENSG00000179912	349.5003925	0.075998216	0.106270584	0.715138781	0.474523292	0.635669339
ENSG00000179918	191.8536207	-0.209981241	0.14790431	-1.419710088	0.1556921	0.293203771
ENSG00000179922	46.56258241	0.032576064	0.265825339	0.122546872	0.902465927	0.947258155
ENSG00000179933	119.2715165	0.014035194	0.183975323	0.076288459	0.939189607	0.96636364
ENSG00000179941	53.13781394	-0.875463875	0.246376199	-3.553362206	0.00038034	0.00190062
ENSG00000179943	150.3598115	0.00925738	0.148850834	0.062192328	0.950409672	0.972594511
ENSG00000179950	825.5974966	0.212048395	0.079021452	2.683428231	0.007287161	0.024689522
ENSG00000179954	58.07492392	0.137476792	0.240038879	0.572727187	0.566829426	0.712314631
ENSG00000179958	312.2053714	-0.187834902	0.128822086	-1.458095484	0.144814226	0.277783072
ENSG00000179965	127.5764534	0.099618187	0.162982553	0.611219948	0.541053979	0.690800331
ENSG00000179981	759.8041368	1.164270756	0.083060312	14.01717288	1.22E-44	1.07E-42
ENSG00000179988	108.647529	-0.177112192	0.175499898	-1.009186867	0.312885024	0.484076461
ENSG00000180008	215.5465394	-0.026405269	0.129595124	-0.203752026	0.838547284	0.905873545
ENSG00000180011	398.3766591	0.487814167	0.098546211	4.950105744	7.42E-07	6.76E-06
ENSG00000180035	112.6177423	-0.036343779	0.18126316	-0.200502845	0.841087333	0.907697202
ENSG00000180066	16.07896188	1.063425692	0.441807551	2.406988492	0.016084677	0.048309056
ENSG00000180071	259.6147362	-0.382476867	0.123934339	-3.086125041	0.002027835	0.008268506
ENSG00000180096	31.2251686	-0.250050906	0.335831465	-0.744572596	0.45653014	0.620201052
ENSG00000180098	70.62325773	0.225187006	0.208338615	1.080870224	0.279754845	0.448534962
ENSG00000180104	228.7926949	0.351729834	0.123708571	2.843213132	0.004466119	0.016270418
ENSG00000180139	10.19749673	0.073096574	0.54703469	0.133623288	0.893700468	0.942407513
ENSG00000180182	767.9291487	0.218130885	0.07893679	2.763361485	0.005720937	0.020018593
ENSG00000180185	101.8935849	-0.370198799	0.174950714	-2.116017652	0.034343306	0.090037406
ENSG00000180190	127.4144675	0.132992967	0.161138279	0.825334414	0.409181759	0.577637398

ENSG00000180198	642.8524036	0.365930789	0.086968791	4.207610372	2.58E-05	0.000169566
ENSG00000180228	393.6485896	0.11357744	0.102442135	1.108698484	0.267560276	0.434425455
ENSG00000180233	35.53179053	-1.494941887	0.312630705	-4.781814016	1.74E-06	1.46E-05
ENSG00000180257	20.7500853	0.620039115	0.38948289	1.591954696	0.111394884	0.228188751
ENSG00000180263	85.62092533	-1.082781261	0.19935952	-5.431299504	5.59E-08	6.15E-07
ENSG00000180304	362.3779861	0.113249479	0.107023953	1.058169464	0.28997819	0.460262558
ENSG00000180318	16.97430422	-7.364998175	1.227290268	-6.001023857	1.96E-09	2.64E-08
ENSG00000180329	359.5258281	-0.243258252	0.102598702	-2.370968121	0.017741561	0.052378249
ENSG00000180340	221.5723797	-0.287163499	0.132593286	-2.165746904	0.030330533	0.081214829
ENSG00000180346	59.23006507	0.203235086	0.233423569	0.8706708	0.383933926	0.553140499
ENSG00000180354	37.17697236	0.409961012	0.292099717	1.403496778	0.160468752	0.299530429
ENSG00000180357	1233.3444	-0.144860091	0.068723147	-2.107879195	0.03504144	0.091543074
ENSG00000180370	777.3100293	0.554036918	0.078933911	7.0189974	2.23E-12	4.29E-11
ENSG00000180376	301.0791546	-0.186378917	0.122659467	-1.519482524	0.128641084	0.254474201
ENSG00000180398	654.4816198	0.044535214	0.082864688	0.537445014	0.590960257	0.731900787
ENSG00000180422	7.055584835	-3.144804728	0.892444877	-3.52380837	0.000425392	0.002097395
ENSG00000180423	9.578529503	-0.156911748	0.553811809	-0.283330448	0.776923539	0.86628393
ENSG00000180425	13.71889869	-0.663306315	0.469900796	-1.411587978	0.158071314	0.296319858
ENSG00000180448	131.1303331	0.44167503	0.161576311	2.733538275	0.006265784	0.021632248
ENSG00000180479	33.08555177	0.064269219	0.311754909	0.206153029	0.836671377	0.904612287
ENSG00000180488	150.1551034	0.723841705	0.158950655	4.553876843	5.27E-06	4.02E-05
ENSG00000180525	13.74625018	0.191951621	0.471541773	0.407072357	0.683954845	0.800159383
ENSG00000180530	306.1100201	-0.428490839	0.111459192	-3.84437417	0.00012086	0.00068374
ENSG00000180535	18.45789529	0.808838339	0.408708546	1.979010099	0.047814869	0.117792962
ENSG00000180573	789.9398995	-0.175864362	0.081398026	-2.160548251	0.030730253	0.082144216
ENSG00000180574	12.84822933	0.3162677	0.484739117	0.652449305	0.514111366	0.669918586
ENSG00000180592	288.288647	-0.137529654	0.117840263	-1.167085436	0.243175869	0.406039673
ENSG00000180596	1005.964025	-0.472059766	0.094439127	-4.998561296	5.78E-07	5.38E-06
ENSG00000180628	23.69299623	0.399526633	0.359321039	1.111893237	0.266184057	0.43306549
ENSG00000180660	35.55196809	-0.084536093	0.303509554	-0.278528606	0.780606609	0.868601276
ENSG00000180667	217.1898513	0.190172317	0.131791845	1.442974835	0.14902756	0.28361877
ENSG00000180694	136.1491211	0.420810426	0.17086519	2.462821278	0.013784861	0.042343307
ENSG00000180720	16.98352323	-0.274206468	0.426494876	-0.642930276	0.520269343	0.674688281
ENSG00000180730	6.364615773	-3.050784374	0.921906395	-3.309212726	0.000935587	0.004236684
ENSG00000180739	52.54000937	-0.52432739	0.26142348	-2.005662961	0.04489222	0.11205875
ENSG00000180758	116.6398184	-0.053215136	0.169162363	-0.314580235	0.753080388	0.850814585
ENSG00000180769	26.38938614	0.182922334	0.352579266	0.518811943	0.603891889	0.74150956
ENSG00000180776	321.3578752	-0.122929856	0.108006645	-1.138169372	0.255049765	0.420305497
ENSG00000180806	234.0366368	-0.135036303	0.130668805	-1.033424189	0.301405432	0.471851946
ENSG00000180815	6.929887912	-1.99218132	0.737933327	-2.699676578	0.006940691	0.02361819
ENSG00000180817	1126.453433	0.651453803	0.0811902	8.023798488	1.03E-15	2.63E-14
ENSG00000180818	330.3964391	0.167890957	0.111785459	1.501903364	0.133122068	0.261408368
ENSG00000180822	108.71603	0.076997542	0.176652754	0.435869465	0.662931441	0.784604829
ENSG00000180867	66.38928982	0.098114254	0.22592549	0.434277043	0.664087273	0.78515562
ENSG00000180879	629.1572021	0.410224799	0.084790474	4.838100076	1.31E-06	1.13E-05
ENSG00000180884	36.33227709	-0.06309086	0.293757769	-0.214771716	0.829945306	0.90073721
ENSG00000180891	69.69495196	-0.190580375	0.21916582	-0.869571613	0.384534556	0.55366512
ENSG00000180900	739.1407852	0.280435904	0.095683911	2.930857469	0.003380278	0.012808948
ENSG00000180901	183.1148905	0.080444588	0.135673574	0.592927461	0.553229699	0.700488334
ENSG00000180902	72.38839713	-0.499662693	0.212605462	-2.350187468	0.018763958	0.054809174

ENSG00000180917	191.1833332	-0.039366845	0.137523066	-0.286256307	0.77468182	0.865083113
ENSG00000180921	295.6232386	0.351497015	0.139151105	2.526009523	0.011536635	0.036425066
ENSG00000180957	572.9486358	0.148422801	0.086442086	1.717020125	0.085975495	0.186690026
ENSG00000180964	133.4962264	-8.54112584	1.005972343	-8.490418155	2.06E-17	5.84E-16
ENSG00000180979	103.8782855	0.108815795	0.181436622	0.599745485	0.54867587	0.696984078
ENSG00000180992	210.8747422	0.234707153	0.132050351	1.777406501	0.075501382	0.168344113
ENSG00000180998	19.66993166	-0.574311038	0.395234229	-1.453090334	0.146198674	0.279712991
ENSG00000181004	6.510926582	-1.404496653	0.7144753	-1.965773559	0.049324773	0.12058216
ENSG00000181007	159.0920433	0.365853627	0.144844373	2.525839423	0.011542222	0.036434517
ENSG00000181019	801.2166053	-0.520654726	0.099164474	-5.250415846	1.52E-07	1.56E-06
ENSG00000181026	584.5129575	0.038580973	0.088605115	0.435426023	0.663253225	0.784755231
ENSG00000181027	105.3124186	-0.329266366	0.203071646	-1.621429546	0.104925548	0.217773224
ENSG00000181031	18.25613842	-0.302374993	0.415239344	-0.728194467	0.466494552	0.628880558
ENSG00000181035	81.10256069	0.347067423	0.221108747	1.569668444	0.116492266	0.235990463
ENSG00000181038	60.29665773	0.196597675	0.241897651	0.812730813	0.416372411	0.584177312
ENSG00000181045	92.1762046	-0.095308542	0.185385722	-0.514109399	0.607175506	0.743915773
ENSG00000181061	110.2676	-0.244906984	0.172392501	-1.420635946	0.155422623	0.292931918
ENSG00000181085	76.05024203	0.177569097	0.216137488	0.821556215	0.411329517	0.579707608
ENSG00000181090	681.3094148	0.13874296	0.090209185	1.538013677	0.124045271	0.248072881
ENSG00000181097	9.880750257	-0.316672973	0.548714264	-0.577118172	0.563859629	0.70997994
ENSG00000181104	21.60381007	0.106227409	0.391793797	0.27113091	0.786290349	0.872090054
ENSG00000181135	83.38631664	-0.188138953	0.204053688	-0.922007119	0.356524862	0.52783566
ENSG00000181163	5608.829963	0.093374589	0.051933014	1.797981309	0.07217997	0.162694486
ENSG00000181191	61.38355501	0.143749823	0.229555129	0.62621046	0.531176907	0.683094573
ENSG00000181192	179.8650866	-0.710016479	0.138697704	-5.11916535	3.07E-07	2.99E-06
ENSG00000181218	311.7103532	-0.020325516	0.120225795	-0.169061187	0.865748514	0.924545891
ENSG00000181220	524.4120925	0.798057207	0.096563382	8.264594606	1.40E-16	3.77E-15
ENSG00000181222	6485.679859	0.098201013	0.056440854	1.739892389	0.081877915	0.179849738
ENSG00000181264	49.85537961	-0.439091126	0.24972318	-1.758311446	0.078694531	0.17424686
ENSG00000181274	107.1271436	-0.346664026	0.172909617	-2.004885749	0.044975263	0.112174629
ENSG00000181284	28.70438173	0.259148874	0.330304853	0.784574831	0.432702891	0.599012271
ENSG00000181315	17.42621242	0.256525965	0.425132377	0.603402562	0.546240918	0.694729455
ENSG00000181350	45.82128422	1.025015996	0.264507501	3.875186869	0.000106543	0.00061111
ENSG00000181381	81.00036219	-1.011238732	0.205670367	-4.916793534	8.80E-07	7.88E-06
ENSG00000181392	20.76514334	-3.037426795	0.513066754	-5.92013957	3.22E-09	4.19E-08
ENSG00000181396	408.7749375	0.322539096	0.104812314	3.077301539	0.002088839	0.008468086
ENSG00000181409	112.7533794	0.28857519	0.190710094	1.513161591	0.130238618	0.257019088
ENSG00000181418	48.52844071	1.377923851	0.279958615	4.921884092	8.57E-07	7.69E-06
ENSG00000181444	680.2755581	0.10149826	0.082526803	1.229882367	0.218741158	0.375814218
ENSG00000181450	154.3636211	-0.169649062	0.147450356	-1.150550367	0.249917262	0.413838003
ENSG00000181458	70.82773228	0.920715492	0.214161591	4.299162543	1.71E-05	0.000116847
ENSG00000181467	280.4700255	0.179960594	0.121876642	1.476579848	0.139788297	0.27068438
ENSG00000181472	95.93291129	-0.277239466	0.185839978	-1.491818227	0.135746804	0.264985458
ENSG00000181481	77.11450921	-0.017873573	0.20601053	-0.086760484	0.930861898	0.962535046
ENSG00000181513	36.94395965	-0.756757714	0.305367642	-2.478185671	0.013205241	0.04087572
ENSG00000181523	165.0561217	0.223464998	0.147556558	1.514436229	0.129915236	0.256489013
ENSG00000181524	34.67861898	0.532340871	0.314876297	1.690634943	0.090906547	0.194867123
ENSG00000181544	91.19018672	-0.429869039	0.191464677	-2.24516107	0.024757794	0.068702635
ENSG00000181555	1584.820119	-0.204637192	0.070467488	-2.903994406	0.003684347	0.013768031
ENSG00000181577	26.79468644	1.820590704	0.397122869	4.58445193	4.55E-06	3.52E-05

ENSG00000181588	192.7978473	0.095973985	0.149993045	0.639856233	0.52226607	0.676216378
ENSG00000181610	369.3303674	0.192574229	0.102418103	1.880275302	0.060070568	0.14101099
ENSG00000181619	97.49448926	0.137688153	0.17725459	0.776781881	0.437287469	0.603159571
ENSG00000181638	68.33989955	-0.439463623	0.235458617	-1.866415541	0.061983251	0.144558977
ENSG00000181649	53.82173863	-1.081460703	0.251879028	-4.293571837	1.76E-05	0.000119543
ENSG00000181652	23.21193849	1.141660662	0.367815859	3.103891893	0.001909931	0.007869992
ENSG00000181666	94.96462541	-0.343804581	0.189229994	-1.816860924	0.069238411	0.157734668
ENSG00000181690	186.5894318	-0.058556882	0.141178368	-0.41477234	0.67830857	0.795757928
ENSG00000181704	354.9361859	-0.115969174	0.114615628	-1.011809434	0.311629178	0.483109487
ENSG00000181722	364.4132226	1.514508222	0.112736585	13.43404385	3.82E-41	3.10E-39
ENSG00000181744	135.665774	-0.246966336	0.159432306	-1.549035709	0.121373136	0.244084244
ENSG00000181751	69.76357976	-0.2734248	0.234169073	-1.16763839	0.242952658	0.40584564
ENSG00000181754	18.74684566	-0.055254091	0.394608761	-0.140022463	0.888642243	0.939047437
ENSG00000181773	66.41661952	0.037743241	0.217130077	0.17382779	0.862000804	0.921617562
ENSG00000181788	115.0446569	0.023058692	0.168479438	0.136863539	0.891138659	0.94027635
ENSG00000181789	1219.021763	0.251160466	0.071628112	3.506450997	0.000454125	0.002221907
ENSG00000181790	393.1831268	0.578365379	0.10479496	5.51901903	3.41E-08	3.88E-07
ENSG00000181798	16.98535061	-0.298483953	0.437750082	-0.681859274	0.49532794	0.654135261
ENSG00000181804	14.19781867	0.409624158	0.481832291	0.850138452	0.395248116	0.563217718
ENSG00000181817	198.6373369	0.334569731	0.138907073	2.408586719	0.016014421	0.048170144
ENSG00000181826	59.53141211	-0.488217985	0.227567337	-2.145378117	0.031922627	0.084622975
ENSG00000181827	1072.288338	-0.031152509	0.089995156	-0.346157619	0.729224263	0.832773591
ENSG00000181830	172.2560322	0.094859506	0.1484322	0.639076332	0.522773276	0.676623625
ENSG00000181852	213.8621481	-0.29474894	0.130680742	-2.255488719	0.02410267	0.067229984
ENSG00000181856	17.23846558	-1.397333853	0.443321156	-3.151967448	0.001621744	0.006836924
ENSG00000181873	169.4319047	-0.528945061	0.145713789	-3.630027491	0.000283391	0.001462989
ENSG00000181885	11.94436344	-3.637329485	0.76337646	-4.764791257	1.89E-06	1.58E-05
ENSG00000181894	34.73681731	0.275642947	0.303217133	0.909061253	0.363317794	0.534400529
ENSG00000181896	39.98118977	-0.078699337	0.277139169	-0.283970457	0.776433021	0.865943204
ENSG00000181904	389.8426692	0.165015396	0.11047493	1.493690895	0.135256429	0.264248548
ENSG00000181915	252.2730173	-0.181535951	0.142089828	-1.277613979	0.201385569	0.353805345
ENSG00000181924	424.8220355	0.68413032	0.104067768	6.573892529	4.90E-11	8.09E-10
ENSG00000181929	21.01871041	-1.194546548	0.405303989	-2.947285445	0.003205771	0.012223526
ENSG00000181938	116.5383063	0.026228835	0.169670555	0.154586839	0.877147046	0.932155367
ENSG00000181982	133.6517863	0.011598365	0.160226942	0.07238711	0.942293843	0.968180144
ENSG00000181991	379.5801142	0.464356915	0.100689272	4.611781452	3.99E-06	3.11E-05
ENSG00000182004	376.7071234	0.255685537	0.110425164	2.315464397	0.020587531	0.05916388
ENSG00000182010	36.3558661	-2.047623896	0.349189091	-5.863940045	4.52E-09	5.79E-08
ENSG00000182013	400.9548587	0.271673807	0.109513171	2.48074095	0.013110962	0.040628652
ENSG00000182022	14.31264281	-1.82379197	0.515202811	-3.539949569	0.000400203	0.001985049
ENSG00000182050	8.997069631	-3.904942759	0.948183719	-4.118339812	3.82E-05	0.00024103
ENSG00000182054	540.0374596	-0.74802035	0.088241913	-8.47692805	2.31E-17	6.54E-16
ENSG00000182057	15.73401193	-1.076287489	0.478012586	-2.251588182	0.024348309	0.067780557
ENSG00000182087	772.5422009	0.010824268	0.086083074	0.125742112	0.899936066	0.945967201
ENSG00000182093	59.5089769	-0.321919768	0.226828625	-1.419220206	0.155834827	0.293315264
ENSG00000182095	510.0568844	-0.135880245	0.095167986	-1.427793641	0.153351264	0.289883623
ENSG00000182107	980.6217807	-5.340053882	0.144159734	-37.04261736	2.36E-300	4.15E-297
ENSG00000182108	144.2888611	0.201858422	0.166802027	1.21016768	0.226214557	0.384569454
ENSG00000182117	528.8130972	0.116174533	0.089792777	1.293807111	0.195732056	0.346977308
ENSG00000182118	72.93315631	-0.383070178	0.212645264	-1.801451729	0.071631709	0.161877671

ENSG00000182134	86.35552075	-0.147550021	0.192311336	-0.767245572	0.442935522	0.607846354
ENSG00000182141	95.15790535	-0.417518783	0.182392812	-2.289118625	0.022072461	0.062538262
ENSG00000182149	477.0050947	0.381575175	0.09292124	4.106436528	4.02E-05	0.000252764
ENSG00000182150	615.8776913	-0.311560765	0.092010548	-3.386141823	0.000708827	0.003308062
ENSG00000182154	373.3985671	0.341791693	0.106175635	3.219116068	0.001285864	0.005583473
ENSG00000182158	566.7290932	0.377008897	0.093161572	4.046828437	5.19E-05	0.000317625
ENSG00000182165	18.48397725	-0.235598156	0.398473262	-0.591252108	0.554351514	0.701402964
ENSG00000182168	181.2704217	-0.685670624	0.140652094	-4.874940753	1.09E-06	9.55E-06
ENSG00000182173	156.9684432	0.30086135	0.157873842	1.90570741	0.056688177	0.134614313
ENSG00000182175	18.16260404	-1.614637882	0.456998181	-3.533138532	0.000410657	0.002028306
ENSG00000182179	20.3666627	1.132432442	0.408160278	2.774479787	0.005529007	0.019465153
ENSG00000182180	409.8635505	-0.083989555	0.099759547	-0.841919972	0.39983275	0.56800674
ENSG00000182185	88.91533851	-0.807629327	0.213943354	-3.774968048	0.000160028	0.000877638
ENSG00000182195	373.342626	-0.592626531	0.10566571	-5.608503743	2.04E-08	2.40E-07
ENSG00000182197	414.6818034	-0.272681404	0.103994065	-2.622086209	0.008739332	0.028810467
ENSG00000182199	1197.261351	0.091638732	0.071143629	1.288080643	0.197717893	0.34921957
ENSG00000182208	53.78363798	0.953388213	0.242615653	3.929623667	8.51E-05	0.000498999
ENSG00000182220	270.4121437	0.07923042	0.120886245	0.655413032	0.512201863	0.668188838
ENSG00000182224	39.62314509	0.484773326	0.274338034	1.767065688	0.077217228	0.171652224
ENSG00000182247	169.1301142	-0.057255359	0.155025707	-0.369328156	0.711883142	0.819996249
ENSG00000182253	2202.12861	1.455885446	0.074284415	19.5988007	1.58E-85	3.22E-83
ENSG00000182263	59.08221395	-8.199552815	1.192604583	-6.875332301	6.18E-12	1.11E-10
ENSG00000182272	233.2079286	-0.133723337	0.128035373	-1.044424942	0.296288829	0.466640787
ENSG00000182307	342.3274782	0.232070989	0.112912747	2.055312572	0.039848824	0.101533907
ENSG00000182308	10.32423415	-0.174243505	0.528699487	-0.329570029	0.741724872	0.842008184
ENSG00000182310	43.31759419	0.142364189	0.289722816	0.491380662	0.623157239	0.755392625
ENSG00000182318	61.49056873	0.137929179	0.225651752	0.611247983	0.541035422	0.690800331
ENSG00000182324	29.35979818	-0.099401054	0.323498703	-0.307268787	0.758638792	0.854578918
ENSG00000182325	59.6747857	0.562157261	0.249317235	2.254787007	0.024146701	0.067312703
ENSG00000182326	43.59330703	-0.124885809	0.28997172	-0.430682717	0.666699087	0.786786699
ENSG00000182359	13.74105483	-0.285065976	0.470828575	-0.605455979	0.544876066	0.693599827
ENSG00000182362	120.7457993	-0.110031179	0.16326646	-0.673936208	0.500351871	0.658525507
ENSG00000182372	35.15827288	0.201315902	0.290327133	0.693410569	0.488051931	0.647922265
ENSG00000182378	822.6169331	-0.522842658	0.077694211	-6.72949314	1.70E-11	2.93E-10
ENSG00000182379	33.20280479	-1.717566584	0.329927883	-5.2058849	1.93E-07	1.94E-06
ENSG00000182389	201.9301107	0.369795572	0.132749925	2.785655593	0.00534196	0.018915681
ENSG00000182400	266.0094098	-0.049452541	0.128471985	-0.384928596	0.700290319	0.811966827
ENSG00000182405	16.84777243	0.146199437	0.430673639	0.339466881	0.734258042	0.836235019
ENSG00000182446	1606.040286	0.337280785	0.065101007	5.180884296	2.21E-07	2.21E-06
ENSG00000182459	16.69786178	0.025210441	0.429694086	0.058670672	0.953214419	0.974299858
ENSG00000182472	24.16165247	0.810314366	0.372787701	2.173661748	0.029730547	0.079912935
ENSG00000182473	1305.850307	0.02026232	0.067157968	0.301711327	0.762872133	0.856873889
ENSG00000182481	2519.552253	0.197198941	0.066732323	2.955073814	0.003125943	0.011958092
ENSG00000182484	36.88842948	0.207437635	0.292634622	0.708862243	0.478409968	0.638865369
ENSG00000182504	188.6060855	-0.63156587	0.136108888	-4.640151558	3.48E-06	2.77E-05
ENSG00000182511	38.8572671	-0.892090649	0.294896476	-3.025097687	0.002485528	0.009843309
ENSG00000182512	176.8892665	-0.285022741	0.156382651	-1.822598215	0.068364266	0.156199227
ENSG00000182518	69.07413429	-0.026545209	0.211327453	-0.125611738	0.900039271	0.945967201
ENSG00000182534	501.0186457	0.173818365	0.095485631	1.820361488	0.068703973	0.156757575
ENSG00000182541	106.9324865	-0.603026782	0.177464526	-3.398013089	0.000678772	0.003180356

ENSG00000182544	76.70143296	-0.087758889	0.202042968	-0.43435755	0.664028818	0.785152594
ENSG00000182551	345.3120125	0.086477305	0.111444146	0.775969919	0.437766747	0.603314879
ENSG00000182552	22.73679533	0.186194668	0.367958631	0.506020655	0.61284215	0.748183007
ENSG00000182568	120.6748089	-7.393480032	0.735434353	-10.05321549	8.89E-24	3.66E-22
ENSG00000182580	13.62974926	-1.232620417	0.499585071	-2.467288333	0.013614068	0.04188279
ENSG00000182600	21.31343315	-0.353891179	0.38666107	-0.915249056	0.360060897	0.531334743
ENSG00000182606	1107.853508	0.196273578	0.070506941	2.783748325	0.005373471	0.019008096
ENSG00000182612	17.1362862	0.285773587	0.411688802	0.694149528	0.487588441	0.64779673
ENSG00000182621	386.7365281	8.356265503	0.518550103	16.1146733	2.01E-58	2.57E-56
ENSG00000182628	408.8441497	-0.18418853	0.105704718	-1.742481631	0.081424203	0.179105195
ENSG00000182636	28.04369443	-2.179706025	0.385552847	-5.653455926	1.57E-08	1.88E-07
ENSG00000182648	31.64813916	0.163387888	0.335830152	0.486519412	0.626598945	0.757668536
ENSG00000182670	1426.597272	0.130796386	0.071221282	1.836478974	0.066286849	0.152419605
ENSG00000182685	18.54578916	-1.13951037	0.426605051	-2.671113168	0.007560015	0.025503251
ENSG00000182700	18.61363004	-0.427145997	0.406634382	-1.050442403	0.293514759	0.463987641
ENSG00000182704	172.0210338	0.071722751	0.141930698	0.505336424	0.613322564	0.748574338
ENSG00000182718	207.573219	0.452214294	0.137017849	3.300404271	0.000965456	0.0043523
ENSG00000182742	463.0150987	0.540574987	0.110809883	4.878400489	1.07E-06	9.40E-06
ENSG00000182747	35.68461787	4.553028148	0.518222088	8.785862774	1.55E-18	4.90E-17
ENSG00000182749	192.815896	-0.083482839	0.135907995	-0.614259954	0.539043561	0.689580557
ENSG00000182759	172.1283874	1.622163719	0.156560796	10.36123829	3.72E-25	1.69E-23
ENSG00000182768	263.5987284	-0.055398339	0.126278439	-0.438699899	0.660879	0.783076162
ENSG00000182774	130.3294076	0.464589889	0.162375059	2.861214599	0.004220213	0.015498387
ENSG00000182782	13.12421392	-1.09405652	0.499893741	-2.188578153	0.028627515	0.077526645
ENSG00000182796	104.7616894	-0.185933441	0.176117878	-1.055732916	0.291090255	0.46155828
ENSG00000182809	178.6200845	0.948644043	0.153193315	6.192463721	5.92E-10	8.52E-09
ENSG00000182810	183.8619023	0.160319935	0.135052756	1.187091177	0.235191658	0.396179065
ENSG00000182827	877.2271388	0.059915366	0.074764683	0.801385946	0.422908249	0.59010551
ENSG00000182831	426.3191781	0.172894022	0.098084587	1.762703268	0.077950549	0.173090636
ENSG00000182853	131.6597104	-7.940931385	0.843741953	-9.411563993	4.89E-21	1.76E-19
ENSG00000182858	121.3010428	0.106623562	0.166905509	0.6388259	0.522936197	0.676628438
ENSG00000182871	595.5671793	0.498591257	0.110753782	4.501798924	6.74E-06	5.01E-05
ENSG00000182872	855.4220702	0.269671113	0.080141624	3.364931971	0.000765625	0.003538907
ENSG00000182873	25.04898895	-0.322087226	0.357458647	-0.901047516	0.367563056	0.539008064
ENSG00000182890	23.4376543	-0.862261115	0.366236585	-2.354382793	0.018553494	0.05430734
ENSG00000182899	1924.601438	0.535708245	0.071066709	7.538104062	4.77E-14	1.08E-12
ENSG00000182902	11.10056104	-0.667167826	0.555338703	-1.201371022	0.229607312	0.388542815
ENSG00000182903	458.9375751	-0.142719225	0.103781171	-1.375193813	0.169071366	0.311509113
ENSG00000182912	71.47079937	0.408751731	0.209125592	1.954575368	0.050633211	0.123052719
ENSG00000182919	42.9760404	-0.461470498	0.281668139	-1.638348235	0.101349071	0.211912997
ENSG00000182923	117.489634	-0.484367578	0.169183847	-2.862965851	0.004196958	0.015433159
ENSG00000182934	431.6832394	-0.01027499	0.097140713	-0.105774293	0.915761433	0.954435272
ENSG00000182944	2375.35027	0.027219583	0.061697258	0.441179787	0.659082845	0.781805162
ENSG00000182952	196.4024811	0.175311676	0.132844319	1.319677632	0.186942671	0.335227518
ENSG00000182957	86.11473074	0.885623831	0.206482414	4.289100527	1.79E-05	0.000121563
ENSG00000182963	422.0403215	-0.557677198	0.102340247	-5.449246173	5.06E-08	5.59E-07
ENSG00000182973	447.2712584	-0.114406378	0.095085479	-1.203195049	0.22890085	0.387795886
ENSG00000182979	1341.093029	0.095836107	0.072531774	1.321298262	0.186401933	0.334618269
ENSG00000183010	12.37841597	7.194853674	1.258520024	5.716916327	1.08E-08	1.32E-07
ENSG00000183011	1036.15049	0.271299363	0.080767568	3.359013633	0.000782212	0.003607266

ENSG00000183020	460.5586643	-0.031768014	0.098980613	-0.320951885	0.748246854	0.846879668
ENSG00000183023	16.36945552	-2.354859406	0.52742819	-4.464796256	8.01E-06	5.88E-05
ENSG00000183032	37.8369169	-0.288104093	0.28673528	-1.004773786	0.315005789	0.486465075
ENSG00000183044	71.70271021	-0.454599393	0.211293205	-2.151509761	0.031435986	0.083601154
ENSG00000183048	100.6012096	0.191323645	0.189515378	1.009541529	0.312714995	0.484076461
ENSG00000183049	174.0189208	0.780417247	0.140916194	5.538165807	3.06E-08	3.50E-07
ENSG00000183060	40.49583073	-0.216568801	0.273524743	-0.791770422	0.428494552	0.595563472
ENSG00000183072	162.1791278	0.249731462	0.161742041	1.544010821	0.122585711	0.246029645
ENSG00000183077	210.1125533	0.634282399	0.129486967	4.898426557	9.66E-07	8.58E-06
ENSG00000183087	96.57662865	0.537633905	0.182935238	2.93893025	0.003293472	0.012517153
ENSG00000183091	29.35468945	0.455912201	0.331465652	1.375443271	0.168994062	0.311407528
ENSG00000183092	59.68098946	0.507591315	0.24438128	2.077046636	0.037797255	0.097277032
ENSG00000183098	203.1296161	0.171626272	0.132769857	1.29265992	0.196128706	0.347154729
ENSG00000183114	117.21676	0.233810497	0.166286145	1.406073229	0.159702381	0.298435459
ENSG00000183137	131.5324961	0.003727317	0.155003655	0.024046638	0.980815408	0.990179984
ENSG00000183145	16.53283565	-1.268519937	0.457836522	-2.770683152	0.005593883	0.01967383
ENSG00000183150	12.0872708	-2.286364984	0.590636827	-3.87101664	0.000108382	0.000619637
ENSG00000183155	131.4446458	0.200406591	0.15697984	1.276639033	0.201729715	0.354212163
ENSG00000183161	30.25543492	-0.075078661	0.311269453	-0.241201507	0.809398943	0.887420149
ENSG00000183172	211.7346159	0.177146312	0.13412433	1.320761949	0.186580751	0.334768145
ENSG00000183199	25.69230381	0.108452494	0.339216385	0.319714785	0.749184551	0.847736055
ENSG00000183207	1120.903915	0.348551492	0.069663999	5.003323062	5.64E-07	5.27E-06
ENSG00000183208	22.62824359	-0.096163077	0.361999281	-0.265644387	0.790513098	0.874908406
ENSG00000183230	11.5279061	-5.826101515	1.248594471	-4.666127914	3.07E-06	2.45E-05
ENSG00000183248	399.5162972	-1.395883274	0.202112661	-6.906461309	4.97E-12	9.08E-11
ENSG00000183250	31.50088137	-0.893596257	0.340101609	-2.627439077	0.008603025	0.028447902
ENSG00000183255	896.2546758	0.301257231	0.074148638	4.062882853	4.85E-05	0.000299676
ENSG00000183258	866.8756029	0.388866279	0.081386053	4.778045698	1.77E-06	1.48E-05
ENSG00000183283	826.5305721	0.322216896	0.084241926	3.824899436	0.000130825	0.000733615
ENSG00000183291	228.5428812	0.138424437	0.124200026	1.114528247	0.265052633	0.431693045
ENSG00000183307	9.597260658	-0.483655951	0.57221578	-0.845233508	0.397980477	0.566119672
ENSG00000183309	196.4690705	-0.14145698	0.14000974	-1.010336705	0.312334001	0.483935111
ENSG00000183317	6.756625467	-2.432864356	0.803820529	-3.026626302	0.002472995	0.00980473
ENSG00000183323	104.8337676	0.391165141	0.17926584	2.182039487	0.029106619	0.078581717
ENSG00000183337	845.9814833	-0.300037595	0.078756255	-3.809698612	0.000139136	0.000775574
ENSG00000183340	44.21999897	0.053470431	0.260689796	0.205111331	0.837485144	0.905074151
ENSG00000183346	9.757238933	0.28576874	0.565080195	0.505713601	0.613057719	0.74838114
ENSG00000183354	417.2131731	-0.055253719	0.097861839	-0.564609451	0.572339435	0.717377715
ENSG00000183386	340.5965013	0.188592193	0.112762494	1.672472707	0.094431145	0.200616198
ENSG00000183401	17.97638292	0.630072078	0.419296279	1.50268941	0.132919155	0.261171544
ENSG00000183426	8.111082116	-0.020043673	0.606465725	-0.033049968	0.973634741	0.986408007
ENSG00000183431	814.5354625	-0.076607646	0.078538445	-0.975415867	0.329354016	0.501837061
ENSG00000183474	51.9977308	0.19853245	0.253877526	0.782000884	0.434214054	0.600453319
ENSG00000183475	112.5184628	-0.146912305	0.167847184	-0.875274176	0.381424742	0.550886667
ENSG00000183495	2219.81654	0.006548881	0.061179159	0.107044315	0.914753824	0.953830969
ENSG00000183496	137.4335607	0.372356242	0.16159664	2.304232572	0.021209592	0.060616711
ENSG00000183506	29.66307107	-0.483520501	0.322331812	-1.500070684	0.133596094	0.261988598
ENSG00000183513	106.104569	-0.609020014	0.189907324	-3.206932744	0.001341584	0.005800315
ENSG00000183520	550.3595051	0.224285209	0.094163049	2.381881332	0.017224447	0.051152602
ENSG00000183527	269.3777557	0.285372222	0.128369512	2.223052939	0.02621223	0.072168405

ENSG00000183530	1098.223363	-0.164618818	0.072543808	-2.269233212	0.023254148	0.065264937
ENSG00000183569	12.68786908	-0.128756234	0.482457372	-0.266875877	0.789564731	0.874348005
ENSG00000183571	202.608786	-0.206271232	0.149554424	-1.379238588	0.167821204	0.310059773
ENSG00000183576	254.3459854	-0.337186865	0.120871346	-2.789634409	0.005276759	0.018736762
ENSG00000183579	247.6138616	-0.041867396	0.12008726	-0.348641447	0.727358508	0.831595187
ENSG00000183580	48.52639645	-2.621915784	0.31112832	-8.427120301	3.54E-17	9.89E-16
ENSG00000183597	109.7592687	0.412881927	0.16939943	2.43732772	0.014796263	0.044987685
ENSG00000183598	76.31116725	-0.417804749	0.39017255	-1.070820459	0.284250166	0.453798699
ENSG00000183605	192.1829661	0.207526742	0.134140726	1.547082292	0.121843403	0.244854691
ENSG00000183617	87.24726326	0.194517734	0.199766167	0.973727119	0.330192049	0.50245994
ENSG00000183621	28.27942472	0.197540744	0.341599862	0.578281099	0.563074353	0.709181874
ENSG00000183624	239.1759496	-0.047450051	0.126968882	-0.373714018	0.708617095	0.817505283
ENSG00000183647	20.70173664	0.344555493	0.380357917	0.905871751	0.365003738	0.5362066
ENSG00000183648	443.8044984	0.079371195	0.098009695	0.809830034	0.418037868	0.58598722
ENSG00000183655	81.67863722	-0.050628101	0.201122464	-0.25172773	0.801251521	0.881445698
ENSG00000183665	88.64093468	-0.194331916	0.195599633	-0.993518819	0.320457184	0.49212442
ENSG00000183684	1136.140667	0.244015834	0.075268369	3.241943964	0.001187174	0.005210842
ENSG00000183688	320.3773796	0.323744414	0.107581998	3.00928056	0.002618672	0.010280738
ENSG00000183696	104.1088607	-0.791113418	0.177183477	-4.464939006	8.01E-06	5.87E-05
ENSG00000183718	49.41037876	-0.384824407	0.252025743	-1.526925	0.126779662	0.251998573
ENSG00000183722	14.80035207	0.10257487	0.467250916	0.219528451	0.82623842	0.898391554
ENSG00000183723	423.5039292	-0.407725809	0.097404155	-4.185918031	2.84E-05	0.000184531
ENSG00000183726	228.1258731	0.178930728	0.123539596	1.448367435	0.147514317	0.281752149
ENSG00000183735	323.1012472	0.300626925	0.108524062	2.770140738	0.005603208	0.019681985
ENSG00000183741	1666.138192	0.617825948	0.08823157	7.002322968	2.52E-12	4.81E-11
ENSG00000183751	255.4096054	0.115403326	0.126449792	0.912641486	0.361431128	0.532405941
ENSG00000183762	96.12919064	-0.081869663	0.185280966	-0.441867639	0.658584989	0.781412564
ENSG00000183763	118.32182	-0.065420862	0.163094357	-0.401122781	0.688329729	0.803737964
ENSG00000183765	262.8369958	0.124371251	0.122254407	1.017315068	0.309003571	0.480151898
ENSG00000183770	185.3959618	0.309597904	0.134875461	2.295435367	0.021708186	0.061727712
ENSG00000183779	168.4341631	0.633295052	0.149540236	4.234947518	2.29E-05	0.000151687
ENSG00000183793	19.3245321	0.240120767	0.39561262	0.606959321	0.543877908	0.692768678
ENSG00000183801	41.06463286	-0.944200109	0.286475084	-3.29592402	0.000980985	0.00441522
ENSG00000183808	465.9299021	0.075874987	0.099312625	0.764001423	0.444866395	0.609959809
ENSG00000183814	286.7972989	-0.002241513	0.110327182	-0.020316956	0.983790529	0.991961125
ENSG00000183826	176.9243758	0.17790955	0.137411398	1.294721926	0.195416174	0.346545487
ENSG00000183828	219.5838642	0.638200133	0.126299863	5.053054834	4.35E-07	4.15E-06
ENSG00000183837	102.246551	0.013896184	0.179826058	0.077275697	0.938404224	0.966102042
ENSG00000183853	553.0893679	0.345511466	0.088622066	3.898706968	9.67E-05	0.000558248
ENSG00000183856	349.6869213	-0.488367478	0.114541726	-4.263664399	2.01E-05	0.000135097
ENSG00000183864	588.7617381	-0.091318293	0.090848942	-1.005166278	0.31481679	0.486226658
ENSG00000183873	41.06099882	0.430858388	0.272656041	1.58022682	0.114054932	0.2321927
ENSG00000183891	28.63178769	-0.549011465	0.329726457	-1.6650513	0.095902522	0.203097048
ENSG00000183935	31.15283612	-0.278156754	0.311785891	-0.892140287	0.372317744	0.54287242
ENSG00000183943	342.9978605	0.270913461	0.108562206	2.495467531	0.012579127	0.039205457
ENSG00000183955	417.2884444	0.671315978	0.098685014	6.802613158	1.03E-11	1.82E-10
ENSG00000183963	220.7614841	-0.062857803	0.138692844	-0.453215908	0.650393279	0.775087119
ENSG00000183971	312.6188937	6.06781425	0.282441696	21.48342239	2.22E-102	6.13E-100
ENSG00000183978	153.0490748	0.156914485	0.154808495	1.013603841	0.310771824	0.48215284
ENSG00000184007	1193.692711	-0.350272222	0.068064357	-5.146191584	2.66E-07	2.62E-06

ENSG00000184009	10623.58918	0.030915787	0.058100543	0.5321084	0.59465092	0.734702534
ENSG00000184014	777.8567482	0.075551416	0.0833327	0.906623881	0.364605729	0.535733962
ENSG00000184047	53.04596514	0.139274206	0.245156492	0.568103276	0.569964843	0.715039399
ENSG00000184058	764.2179306	0.394626563	0.09036169	4.367188807	1.26E-05	8.82E-05
ENSG00000184060	12.07734475	-1.310199288	0.540899196	-2.422261479	0.015424248	0.046634612
ENSG00000184076	417.3006115	0.197148037	0.112808443	1.747635486	0.080527167	0.177465502
ENSG00000184083	54.44573404	-0.17452297	0.238852703	-0.730671948	0.464979554	0.627552632
ENSG00000184117	968.6443555	-0.383830145	0.0812629	-4.723313411	2.32E-06	1.91E-05
ENSG00000184160	46.61228	1.905688456	0.287782586	6.621972785	3.54E-11	5.93E-10
ENSG00000184162	163.9174029	0.304602622	0.145172896	2.098205861	0.035886964	0.093387215
ENSG00000184163	42.78946236	2.149691677	0.303738495	7.077442305	1.47E-12	2.88E-11
ENSG00000184164	380.075937	0.96218631	0.103410102	9.304567827	1.35E-20	4.68E-19
ENSG00000184178	55.97812161	0.307600267	0.23631796	1.301637279	0.19304041	0.343767576
ENSG00000184182	37.32209437	-0.632496763	0.291998104	-2.166098868	0.030303633	0.08115827
ENSG00000184185	99.40664527	-0.132479329	0.184900231	-0.71649088	0.473688297	0.634892679
ENSG00000184203	324.3144138	-0.375649905	0.112518293	-3.338567412	0.000842116	0.003851906
ENSG00000184205	631.3975705	-0.214325808	0.086736334	-2.471003759	0.01347344	0.04153202
ENSG00000184207	541.3796524	0.275625511	0.088631617	3.109787685	0.001872219	0.007737292
ENSG00000184208	191.0967674	0.045537989	0.135191072	0.336841691	0.736236243	0.837783014
ENSG00000184209	255.1558344	0.355791225	0.118186888	3.010412001	0.002608935	0.010251109
ENSG00000184216	1106.085267	1.146716754	0.081549405	14.06162007	6.54E-45	5.85E-43
ENSG00000184220	282.9421535	0.109434661	0.112716731	0.970882139	0.331606974	0.504073644
ENSG00000184226	290.6384994	-0.731566197	0.115929153	-6.310459279	2.78E-10	4.15E-09
ENSG00000184232	316.3820798	-0.093279463	0.110749604	-0.842255496	0.399644956	0.567854815
ENSG00000184254	96.63422294	-0.767341602	0.198005475	-3.875355478	0.000106469	0.000610936
ENSG00000184260	1087.653233	-0.167568947	0.210485734	-0.796105961	0.425970449	0.593199852
ENSG00000184270	893.7095467	-0.220135528	0.089277657	-2.46574044	0.013673037	0.042036585
ENSG00000184271	100.5646224	0.751659682	0.181424278	4.143104176	3.43E-05	0.000218859
ENSG00000184277	123.9248531	0.411282154	0.160454336	2.563234901	0.010370186	0.033311229
ENSG00000184281	162.4264009	0.36982908	0.161708817	2.287006278	0.022195457	0.062694466
ENSG00000184302	15.81737738	-2.160309489	0.507896544	-4.253443962	2.11E-05	0.000140743
ENSG00000184304	83.21369138	-0.569204424	0.199946313	-2.846786304	0.0044163	0.01612156
ENSG00000184305	374.4271992	-2.090915922	0.12332105	-16.955061	1.77E-64	2.56E-62
ENSG00000184307	136.1482556	0.946689686	0.165754939	5.711381475	1.12E-08	1.37E-07
ENSG00000184349	607.4742693	0.263387962	0.091167665	2.889050222	0.003864074	0.014355631
ENSG00000184363	17.41969096	-0.639933912	0.426407534	-1.500756579	0.133418533	0.261750019
ENSG00000184368	510.0102441	0.217656903	0.0995801	2.185746989	0.028834121	0.077995937
ENSG00000184371	62.17675533	0.019364727	0.221342869	0.087487467	0.930284047	0.962133707
ENSG00000184378	24.20192725	-0.095704631	0.349735209	-0.273648831	0.784354504	0.871011269
ENSG00000184381	62.87456609	0.332593296	0.241116667	1.379387414	0.167775338	0.310056594
ENSG00000184384	187.0802172	2.051912995	0.154588844	13.27335749	3.30E-40	2.61E-38
ENSG00000184402	171.7189631	-0.160761015	0.139129496	-1.155477597	0.247894902	0.412129074
ENSG00000184408	16.08338095	-4.128999192	0.7415429	-5.568119109	2.58E-08	2.98E-07
ENSG00000184428	1067.623542	0.3007527	0.075645357	3.975824987	7.01E-05	0.000418342
ENSG00000184432	1080.469411	0.040410602	0.072897045	0.554351711	0.579338167	0.722635356
ENSG00000184436	175.2075136	-0.089458299	0.14671459	-0.609743713	0.542031593	0.691497292
ENSG00000184441	26.39777256	-0.269233533	0.349128042	-0.771159863	0.440612181	0.60586547
ENSG00000184445	555.6780552	-0.743708401	0.089049772	-8.351603679	6.73E-17	1.85E-15
ENSG00000184451	80.69750802	-0.672873476	0.211152661	-3.186668232	0.001439218	0.006167386
ENSG00000184465	213.3990867	-0.749421963	0.145588316	-5.147541953	2.64E-07	2.61E-06

ENSG00000184470	281.1496428	-0.199106671	0.112100874	-1.776138437	0.075710102	0.168782702
ENSG00000184481	31.86514631	0.584074253	0.31169692	1.873853141	0.06095067	0.142648127
ENSG00000184489	220.8642657	0.040953442	0.140787303	0.290888746	0.771136409	0.863277474
ENSG00000184497	14.36219592	1.850590101	0.497739798	3.717987002	0.000200817	0.001074208
ENSG00000184500	190.492666	-0.666208801	0.142303283	-4.681612301	2.85E-06	2.29E-05
ENSG00000184508	145.5021187	0.489363261	0.158443444	3.088567421	0.002011241	0.008215148
ENSG00000184517	111.8505862	0.126655389	0.170921701	0.741014093	0.458684895	0.622312879
ENSG00000184524	64.13369563	0.013228952	0.21835206	0.060585425	0.951689382	0.973475269
ENSG00000184557	60.49831407	-0.834369341	0.237816482	-3.508458855	0.000450711	0.002208279
ENSG00000184564	14.49463196	0.502809813	0.469506855	1.070931782	0.284200104	0.453798699
ENSG00000184575	2112.120272	0.169474739	0.060130371	2.818454922	0.004825539	0.017358346
ENSG00000184602	106.0984644	-0.093088597	0.176426068	-0.527635164	0.597752582	0.736869725
ENSG00000184613	96.73437812	0.063465029	0.185130757	0.342811916	0.731739961	0.834585192
ENSG00000184619	38.39713098	-0.845636654	0.285540629	-2.961528303	0.003061164	0.011742715
ENSG00000184634	560.2319641	0.215943402	0.090159703	2.395121039	0.016614879	0.049636157
ENSG00000184640	2363.845935	0.053723903	0.059630717	0.900943438	0.367618394	0.539032941
ENSG00000184661	261.1229047	-0.233256134	0.116428286	-2.003431839	0.045130957	0.11246311
ENSG00000184675	165.9256589	0.02278196	0.143801529	0.158426413	0.874120802	0.93021022
ENSG00000184677	577.4453696	0.113456394	0.084477552	1.343036002	0.179260317	0.325121971
ENSG00000184678	4974.483919	-0.161826272	0.066507221	-2.433213567	0.014965471	0.045423503
ENSG00000184697	25.86892883	-4.538970163	0.655501891	-6.924419632	4.38E-12	8.10E-11
ENSG00000184702	21.46069866	0.193780522	0.40141376	0.482745091	0.629276742	0.759664008
ENSG00000184708	306.1071137	-0.042853859	0.11012821	-0.389126989	0.69718221	0.810027539
ENSG00000184719	6.771799967	-4.122257393	1.126270531	-3.660095224	0.000252122	0.001316073
ENSG00000184743	431.9599026	0.356261244	0.098098452	3.63167039	0.000281593	0.00145424
ENSG00000184752	479.2840698	0.033644024	0.100928	0.333346782	0.73887253	0.840132657
ENSG00000184786	11.85715028	0.287482749	0.506656451	0.567411602	0.570434569	0.715500883
ENSG00000184787	845.2775354	0.068391338	0.079933073	0.855607509	0.392214915	0.560529343
ENSG00000184792	84.6860715	0.212567867	0.193183834	1.100339825	0.271184086	0.438811526
ENSG00000184831	78.16622783	0.2240902	0.202937541	1.104232365	0.269492354	0.436728091
ENSG00000184838	9.284513228	-5.51052749	1.261784791	-4.367248306	1.26E-05	8.82E-05
ENSG00000184840	542.1039614	0.499783799	0.103108539	4.847162056	1.25E-06	1.09E-05
ENSG00000184857	42.82986434	-0.097848625	0.263507991	-0.371330771	0.710391181	0.818634554
ENSG00000184863	1651.738345	0.50206969	0.062974258	7.972617806	1.55E-15	3.90E-14
ENSG00000184867	215.4233427	0.136577029	0.140674401	0.970873364	0.331611344	0.504073644
ENSG00000184897	508.9260014	0.775891026	0.242901887	3.194256893	0.001401913	0.006027754
ENSG00000184898	62.08076322	-0.457425371	0.245246798	-1.865163477	0.062158493	0.144847438
ENSG00000184900	299.3960306	0.340236045	0.111241468	3.058536094	0.002224213	0.008942049
ENSG00000184903	86.31610422	0.880751981	0.192752882	4.569332348	4.89E-06	3.75E-05
ENSG00000184916	339.0697306	0.779670127	0.11087097	7.032229661	2.03E-12	3.93E-11
ENSG00000184922	41.48544425	-0.586013723	0.272985592	-2.146683707	0.031818471	0.084394648
ENSG00000184924	291.9415067	0.455617046	0.113213181	4.024416946	5.71E-05	0.000346288
ENSG00000184925	9.595753425	-1.360791768	0.624055924	-2.180560612	0.029215931	0.078781864
ENSG00000184933	8.429773516	-1.489207617	0.652970636	-2.280665523	0.022568247	0.063619539
ENSG00000184939	107.9456433	-0.169137252	0.169490745	-0.99791438	0.318320878	0.48997802
ENSG00000184949	52.13008125	-0.312949679	0.249743844	-1.253082656	0.210175627	0.365252634
ENSG00000184967	122.6350758	0.863660135	0.168337445	5.130528941	2.89E-07	2.83E-06
ENSG00000184979	43.62947979	0.989213577	0.278766148	3.548542684	0.000387369	0.00193025
ENSG00000184983	750.339863	0.104635956	0.092845667	1.12698803	0.259747542	0.425948368
ENSG00000184986	59.39396034	0.302230086	0.225891586	1.337943083	0.180914991	0.327507088

ENSG00000184990	530.4991889	0.241396723	0.091206588	2.646702689	0.008128078	0.027108334
ENSG00000184992	729.1988877	-0.013568607	0.086504165	-0.156854952	0.875359163	0.931034312
ENSG00000185000	112.9530405	0.339820518	0.176933002	1.920616928	0.054780019	0.131067098
ENSG00000185008	191.9347312	5.315643924	0.280643571	18.94090754	5.25E-80	9.70E-78
ENSG00000185009	525.3497936	0.442711605	0.09400081	4.709657318	2.48E-06	2.03E-05
ENSG00000185010	18.29048477	-0.680933633	0.409499024	-1.66284556	0.096343354	0.203737267
ENSG00000185015	18.97655628	-1.599177885	0.468119755	-3.416172607	0.00063508	0.002997684
ENSG00000185019	30.87088445	-0.022956901	0.312907516	-0.073366409	0.941514547	0.967676806
ENSG00000185022	101.2721687	0.357962258	0.194230125	1.842980111	0.065331914	0.150717261
ENSG00000185024	272.7568325	-0.048032406	0.130596559	-0.367792277	0.713028128	0.820852564
ENSG00000185033	312.5507481	-0.591812327	0.112786252	-5.247202723	1.54E-07	1.58E-06
ENSG00000185043	295.0588055	0.126482255	0.112111229	1.128185424	0.259241618	0.425317334
ENSG00000185046	45.31822203	3.150122558	0.338493065	9.306313432	1.32E-20	4.61E-19
ENSG00000185049	471.4968643	0.41304637	0.092551932	4.462860579	8.09E-06	5.92E-05
ENSG00000185053	34.90733946	-5.981126113	0.868546527	-6.886362361	5.72E-12	1.04E-10
ENSG00000185070	37.74293144	-3.325588368	0.405186113	-8.207557613	2.26E-16	6.01E-15
ENSG00000185085	99.26291176	-0.092055427	0.186825642	-0.492734437	0.622200242	0.754883986
ENSG00000185088	481.2530756	0.142128334	0.09192362	1.546156847	0.122066692	0.245233242
ENSG00000185090	202.7114669	0.102887351	0.137247211	0.749649845	0.453465622	0.617471073
ENSG00000185100	45.75891047	-0.563676889	0.267580297	-2.106570985	0.035154784	0.091753856
ENSG00000185104	280.7462922	-0.487015907	0.122959484	-3.960783586	7.47E-05	0.0004426
ENSG00000185112	36.88381594	-0.226719666	0.293054881	-0.773642346	0.439142323	0.604471554
ENSG00000185115	189.571591	0.062880585	0.137803253	0.456306971	0.648169252	0.773694211
ENSG00000185122	688.3864487	0.35997388	0.086876423	4.143516341	3.42E-05	0.000218579
ENSG00000185127	226.3760649	0.152379995	0.125477095	1.21440487	0.224593157	0.382778461
ENSG00000185129	375.8953476	-0.253580376	0.105657664	-2.400018753	0.016394232	0.049078207
ENSG00000185130	641.2661827	-0.149119887	0.085331413	-1.747538008	0.080544058	0.177474883
ENSG00000185133	31.09926428	-1.642811541	0.343202128	-4.786717231	1.70E-06	1.43E-05
ENSG00000185158	91.00736885	0.243261349	0.183823417	1.323342546	0.185721489	0.333706249
ENSG00000185163	303.5369115	0.362072402	0.12291562	2.945698873	0.003222259	0.012279728
ENSG00000185164	151.2996274	0.319089286	0.146287687	2.181245005	0.0291653	0.078694768
ENSG00000185187	341.7014605	-0.67003519	0.10920044	-6.135828675	8.47E-10	1.19E-08
ENSG00000185189	174.1479311	0.240988306	0.144674924	1.665722702	0.095768658	0.202997185
ENSG00000185201	60.7372433	-0.186858586	0.2319975	-0.805433621	0.420569497	0.588244521
ENSG00000185215	77.54508985	0.935902398	0.21214584	4.411599114	1.03E-05	7.34E-05
ENSG00000185219	782.8151975	-0.187090374	0.078531552	-2.382359328	0.017202103	0.051111459
ENSG00000185220	56.45089512	0.021680508	0.254888427	0.085058816	0.932214631	0.96324653
ENSG00000185222	376.235151	0.409984504	0.107907663	3.799401199	0.000145046	0.00080405
ENSG00000185236	730.5431408	-0.189635069	0.096795729	-1.959126405	0.050097985	0.122047589
ENSG00000185238	260.4124416	0.225538174	0.116147685	1.941822374	0.052158607	0.126083627
ENSG00000185246	324.7173708	0.314329612	0.115431519	2.723083032	0.00646758	0.022241781
ENSG00000185250	42.28915597	-1.188226543	0.28568305	-4.159247613	3.19E-05	0.000205459
ENSG00000185252	182.8096435	0.331699083	0.146846277	2.258818466	0.023894679	0.066742605
ENSG00000185262	346.1378274	-0.270236071	0.10439832	-2.588509769	0.009639221	0.031314094
ENSG00000185272	10.65359598	0.515844802	0.55893564	0.922905546	0.35605643	0.527308874
ENSG00000185278	380.4656518	-0.109662157	0.100845596	-1.087426332	0.276848439	0.445411754
ENSG00000185298	664.2029469	0.35361274	0.08434771	4.192321769	2.76E-05	0.000179813
ENSG00000185305	50.65657312	-0.201521798	0.247528959	-0.814134227	0.41556806	0.583340116
ENSG00000185324	440.186045	0.34611993	0.100262793	3.452127371	0.000556185	0.002662646
ENSG00000185332	11.28960649	0.706991181	0.580525703	1.217846474	0.22328232	0.381655725

ENSG00000185339	35.39780306	0.671490587	0.303275534	2.214127131	0.026820035	0.073567863
ENSG00000185340	596.4267118	0.602261554	0.097445939	6.180468446	6.39E-10	9.15E-09
ENSG00000185344	287.2457025	0.383417493	0.116632289	3.287404336	0.001011155	0.004539373
ENSG00000185345	25.30499389	-0.706566063	0.358095902	-1.973119658	0.048481931	0.119099371
ENSG00000185347	75.8798797	0.027596885	0.223727299	0.123350549	0.901829514	0.947140126
ENSG00000185359	1174.711244	0.510489485	0.084935524	6.010317739	1.85E-09	2.50E-08
ENSG00000185361	245.7635461	-0.060580154	0.119545667	-0.506753237	0.612327972	0.747774297
ENSG00000185379	155.9820951	0.275253234	0.150830474	1.824917909	0.068013418	0.155625425
ENSG00000185386	23.48638019	-1.290532675	0.37562025	-3.435737757	0.000590943	0.002813889
ENSG00000185414	264.3204578	-0.091366097	0.11550393	-0.791021545	0.428931419	0.595858127
ENSG00000185418	111.2456875	-0.41473968	0.176210079	-2.353666045	0.018589303	0.054376477
ENSG00000185420	190.0227811	0.042708851	0.140417618	0.304155931	0.761009097	0.855602272
ENSG00000185432	108.9276092	0.573933785	0.173497814	3.308017391	0.00093959	0.004253438
ENSG00000185436	24.64210011	-0.419374944	0.349611593	-1.199545301	0.230315981	0.389554315
ENSG00000185437	47.43865823	-1.796575838	0.287060364	-6.258529777	3.89E-10	5.72E-09
ENSG00000185442	16.36527398	-1.184197018	0.445119244	-2.660404003	0.007804697	0.026177789
ENSG00000185453	274.6542061	0.734211529	0.266747762	2.752456187	0.005915006	0.020612277
ENSG00000185475	221.1238247	0.379794022	0.134284564	2.828277589	0.004679921	0.016921196
ENSG00000185477	32.84646126	-0.629995996	0.328201129	-1.919542442	0.05491572	0.131369401
ENSG00000185480	120.0948879	0.000942586	0.163848313	0.005752796	0.995409958	0.997940364
ENSG00000185482	9.898799011	-0.171793511	0.549816005	-0.312456366	0.754693719	0.851673232
ENSG00000185483	8.541572608	-1.548754651	0.639878199	-2.420389776	0.015503879	0.046865286
ENSG00000185499	38.55105337	-0.046643738	0.308902678	-0.150998167	0.879977162	0.934004754
ENSG00000185504	352.9818405	0.154363655	0.109259732	1.412813782	0.157710485	0.295854591
ENSG00000185513	78.90595518	0.092844359	0.202504369	0.458480769	0.646607076	0.77242024
ENSG00000185515	228.6676538	0.099185711	0.124520461	0.796541464	0.425717385	0.592965008
ENSG00000185532	1380.364084	-1.714868651	0.070364106	-24.37135572	3.44E-131	1.34E-128
ENSG00000185551	11.08927967	-0.241343021	0.509617236	-0.473577038	0.635801553	0.764911306
ENSG00000185559	22.78827809	-4.371313501	0.669491407	-6.529304867	6.61E-11	1.08E-09
ENSG00000185561	98.06571453	-0.409725986	0.197757638	-2.071859221	0.038278572	0.0982925
ENSG00000185567	214.2866934	-0.72635653	0.130305437	-5.574261122	2.49E-08	2.89E-07
ENSG00000185585	19.01246528	-1.876432917	0.449019004	-4.178961022	2.93E-05	0.000189566
ENSG00000185591	1565.226374	-0.040528016	0.079960538	-0.506850216	0.612259919	0.747774297
ENSG00000185608	74.90057181	0.136023368	0.208210567	0.653297138	0.513564737	0.669454701
ENSG00000185615	17.95545475	1.762348268	0.457533903	3.851841925	0.000117233	0.00066563
ENSG00000185619	309.9366571	-0.15072207	0.114151846	-1.320364721	0.186713277	0.334920367
ENSG00000185621	109.556299	0.07356336	0.176866159	0.415926711	0.677463633	0.795306274
ENSG00000185624	6695.786513	0.610678522	0.059148822	10.32444103	5.46E-25	2.44E-23
ENSG00000185627	235.7300742	-0.343229778	0.121258039	-2.830573383	0.004646465	0.016830556
ENSG00000185630	430.0678113	-0.870895228	0.099252443	-8.774547055	1.72E-18	5.40E-17
ENSG00000185633	8.111013013	-1.425646807	0.643208082	-2.216462832	0.026659821	0.073199863
ENSG00000185634	15.78156416	-0.121211779	0.434474217	-0.278984977	0.780256354	0.868470711
ENSG00000185650	915.5505115	-0.486899342	0.078000847	-6.242231497	4.31E-10	6.31E-09
ENSG00000185651	415.1741137	-0.001953588	0.09665299	-0.020212395	0.98387394	0.991961125
ENSG00000185658	799.9970429	-0.112118859	0.08349323	-1.342849704	0.179320645	0.325189402
ENSG00000185670	36.67771558	0.054428323	0.290178163	0.187568637	0.851214814	0.914699033
ENSG00000185686	1203.999997	0.752300956	0.075675772	9.941107109	2.76E-23	1.10E-21
ENSG00000185697	86.26196504	-1.057786481	0.209428063	-5.050834486	4.40E-07	4.19E-06
ENSG00000185716	66.63526901	-0.207045174	0.213544993	-0.969562296	0.332264714	0.504664078
ENSG00000185721	283.2394019	0.157387113	0.129130787	1.218819444	0.22291273	0.381163131

ENSG00000185722	570.3432605	0.067558413	0.086176902	0.783950355	0.433069241	0.599401284
ENSG00000185728	683.5097147	0.043899447	0.08630737	0.508640767	0.611004048	0.746977708
ENSG00000185730	65.42384398	-0.343377956	0.221075259	-1.553217477	0.120371187	0.242431681
ENSG00000185742	93.12956921	-8.014493233	1.007259187	-7.956733818	1.77E-15	4.42E-14
ENSG00000185745	98.89314578	0.481694895	0.204260315	2.358240243	0.018361805	0.053864066
ENSG00000185753	85.30742539	0.411665028	0.209410084	1.965831921	0.049318029	0.12058216
ENSG00000185760	307.679479	1.148109559	0.117337723	9.784658632	1.31E-22	5.04E-21
ENSG00000185761	62.48562002	-0.729815944	0.245546145	-2.972215031	0.002956595	0.011387797
ENSG00000185787	1022.963896	0.150343055	0.083460929	1.801358524	0.071646389	0.161877671
ENSG00000185798	53.06129911	0.050333347	0.246136807	0.204493378	0.837967966	0.905456617
ENSG00000185800	210.9217377	-0.102650211	0.14005107	-0.732948418	0.463589891	0.626640415
ENSG00000185803	110.4245403	0.335803347	0.170666409	1.967600709	0.049113998	0.120234286
ENSG00000185808	103.5208301	-0.042163471	0.178955612	-0.235608546	0.813736442	0.890577
ENSG00000185813	366.1891578	0.107767114	0.121758632	0.885088085	0.376109125	0.546004956
ENSG00000185818	333.3130748	0.132343664	0.108401269	1.220868215	0.222135921	0.380205103
ENSG00000185825	555.5115982	0.88245419	0.09589329	9.20246024	3.50E-20	1.19E-18
ENSG00000185829	7.508849423	-0.193789476	0.649497989	-0.298368093	0.765422239	0.858571124
ENSG00000185838	33.65601083	0.132746506	0.296348854	0.447940002	0.654196498	0.778174175
ENSG00000185842	897.3067084	-0.083018234	0.075124949	-1.105068758	0.269129797	0.436341904
ENSG00000185864	38.89643709	-0.027216675	0.291979635	-0.093214292	0.925733321	0.959334816
ENSG00000185875	56.13859989	-0.229416297	0.262133623	-0.875188365	0.381471423	0.550886667
ENSG00000185880	80.42682609	-0.180448778	0.201216765	-0.896787986	0.36983206	0.541149056
ENSG00000185885	558.7139675	0.764989555	0.11754539	6.508035379	7.61E-11	1.23E-09
ENSG00000185896	1608.727772	0.706238639	0.065739708	10.74295371	6.40E-27	3.07E-25
ENSG00000185900	797.2299332	-0.279569609	0.077173471	-3.622612848	0.000291642	0.001500622
ENSG00000185904	220.3116545	0.252330589	0.133420592	1.891241716	0.058592085	0.138001848
ENSG00000185909	15.47464586	-0.267359948	0.446063468	-0.599376472	0.548921863	0.697107442
ENSG00000185917	59.12062264	0.063531314	0.23833329	0.266565004	0.789804104	0.874468215
ENSG00000185920	782.3685638	-0.3690859	0.080828004	-4.56631218	4.96E-06	3.80E-05
ENSG00000185924	163.5101624	0.798337582	0.174337908	4.579254115	4.67E-06	3.59E-05
ENSG00000185946	105.7136832	-0.025295049	0.178664692	-0.141578331	0.887413084	0.93824263
ENSG00000185947	191.5938456	-0.258234817	0.135009685	-1.912713282	0.055784768	0.132927674
ENSG00000185963	595.2191272	0.119647769	0.084251196	1.420131393	0.155569432	0.29309064
ENSG00000185973	97.48742454	-0.122387796	0.18829211	-0.649988978	0.515699341	0.671096242
ENSG00000185989	227.2986266	0.850427986	0.124385862	6.837014853	8.09E-12	1.44E-10
ENSG00000186001	427.4937524	-0.026934767	0.096108872	-0.280252663	0.779283665	0.868088632
ENSG00000186010	11.99361531	0.588185914	0.498415706	1.180111114	0.237956025	0.399351049
ENSG00000186017	54.64041307	0.081121086	0.235403645	0.344604204	0.730391945	0.83331836
ENSG00000186020	102.3154152	0.227136117	0.176766039	1.284953372	0.198808579	0.350564914
ENSG00000186026	28.72401213	1.143087918	0.338493434	3.376986971	0.000732845	0.003403067
ENSG00000186056	21.85878943	-0.259496086	0.371722317	-0.698091221	0.485120148	0.64556022
ENSG00000186063	79.55897488	0.318125931	0.196470099	1.619207872	0.105402545	0.218472709
ENSG00000186073	260.0050097	0.387128263	0.119186773	3.248080743	0.001161863	0.005117808
ENSG00000186088	21.37023903	0.228613095	0.381665247	0.598988504	0.549180551	0.69718384
ENSG00000186104	55.37733594	-0.070180935	0.233942448	-0.299992309	0.764183022	0.857934696
ENSG00000186106	61.01967293	0.094133804	0.224800655	0.418743458	0.675403632	0.793548715
ENSG00000186111	551.7066908	0.101260759	0.086918918	1.165002532	0.244017968	0.407189403
ENSG00000186130	101.1304547	0.052276396	0.179400473	0.291394974	0.770749258	0.862998381
ENSG00000186132	54.20503848	0.818383575	0.24109594	3.394431173	0.000687713	0.003220103
ENSG00000186141	203.1319897	-0.047610259	0.133254978	-0.35728691	0.720877028	0.82669276

ENSG00000186153	89.34324957	-1.512498102	0.211630847	-7.146869782	8.88E-13	1.77E-11
ENSG00000186166	148.4483339	0.291136206	0.157848333	1.844404694	0.065124184	0.150361507
ENSG00000186174	1427.376385	-0.168706281	0.07544428	-2.236170594	0.025340599	0.070098344
ENSG00000186184	638.5109176	0.075063688	0.084556584	0.887733214	0.374684275	0.544618172
ENSG00000186185	603.3033598	-0.09636515	0.092092927	-1.046390355	0.295380844	0.465817428
ENSG00000186187	164.5231095	0.318127927	0.147101825	2.162637531	0.030569069	0.081794572
ENSG00000186193	1067.622068	0.445243758	0.07037731	6.326524212	2.51E-10	3.76E-09
ENSG00000186197	154.8594361	-6.757327566	0.525715648	-12.85357892	8.21E-38	5.95E-36
ENSG00000186222	47.27212792	0.401962513	0.261348346	1.538033506	0.124040423	0.248072881
ENSG00000186230	94.54967434	0.14895643	0.185497509	0.803010406	0.42196872	0.589614505
ENSG00000186235	8.891343525	0.801045578	0.581384609	1.377823846	0.168257682	0.310553665
ENSG00000186260	557.0818869	-0.057654071	0.093962717	-0.613584545	0.539489899	0.689580557
ENSG00000186272	20.73294735	0.268662485	0.388094001	0.69226137	0.488773205	0.648634597
ENSG00000186280	24.30921383	-0.476565277	0.385233623	-1.237081212	0.216056934	0.372341032
ENSG00000186283	582.8673387	0.185048051	0.085974055	2.15237087	0.031368156	0.083468174
ENSG00000186297	14.59574305	-6.170784183	1.234322817	-4.999327646	5.75E-07	5.37E-06
ENSG00000186298	984.8434321	-0.175609134	0.074806339	-2.347516774	0.018899022	0.055112013
ENSG00000186300	39.16548733	-0.298511044	0.288000441	-1.036495095	0.299971226	0.470406446
ENSG00000186310	148.2735192	-3.272535127	0.208733479	-15.67805577	2.14E-55	2.57E-53
ENSG00000186314	110.3280214	0.138204295	0.171426669	0.80620067	0.420127152	0.587797573
ENSG00000186318	172.5339506	-0.618406831	0.140045793	-4.41574729	1.01E-05	7.22E-05
ENSG00000186326	10.67140613	-0.473805416	0.533672941	-0.887819823	0.374637678	0.544606796
ENSG00000186340	78.56612143	3.332725365	0.269323539	12.3744303	3.59E-35	2.37E-33
ENSG00000186350	304.355094	0.085286011	0.111006891	0.768294745	0.442312096	0.607378766
ENSG00000186352	16.23498823	0.737122777	0.43117988	1.709548176	0.087349456	0.188826994
ENSG00000186364	33.11257981	-0.114032612	0.301482791	-0.378239206	0.705252903	0.815029825
ENSG00000186376	74.70217688	-0.1548288	0.205751252	-0.752504778	0.451747558	0.616629899
ENSG00000186395	155.4230562	0.256273762	0.1499054	1.70956992	0.087345432	0.188826994
ENSG00000186409	11.91362496	-0.802355494	0.550834535	-1.456617994	0.145221858	0.278336941
ENSG00000186416	229.0045184	0.216598481	0.134565791	1.609610281	0.107482962	0.221998702
ENSG00000186432	731.9032487	0.015996386	0.08166151	0.195886482	0.844699018	0.909999778
ENSG00000186448	75.35127323	-0.326232524	0.20740144	-1.572952068	0.115729917	0.234692844
ENSG00000186468	8884.396053	0.205345199	0.080192452	2.560654948	0.010447506	0.033462203
ENSG00000186470	131.7802592	0.321567391	0.163451302	1.967359002	0.049141837	0.120281476
ENSG00000186472	10.21834409	-1.326454982	0.579145181	-2.290366949	0.022000054	0.062371399
ENSG00000186480	828.7691547	1.162581968	0.082378304	14.11272038	3.17E-45	2.87E-43
ENSG00000186501	87.26451657	0.232805662	0.198646111	1.171961843	0.241212376	0.403706689
ENSG00000186510	5.446130866	-5.726044647	1.320470833	-4.336365867	1.45E-05	0.000100336
ENSG00000186522	250.258545	-0.454817548	0.11869637	-3.831773016	0.000127223	0.000716274
ENSG00000186523	13.3116834	0.154725571	0.466317534	0.331803032	0.740037995	0.840999008
ENSG00000186532	125.6827297	-0.23468333	0.164237297	-1.428928354	0.153024827	0.28947221
ENSG00000186566	1257.805914	-0.181969928	0.069251788	-2.627656749	0.008597522	0.028447185
ENSG00000186567	122.8236368	0.294331782	0.172028345	1.710949335	0.087090466	0.188441124
ENSG00000186575	762.4533483	-0.149920707	0.083063975	-1.804882398	0.071093087	0.160967858
ENSG00000186577	120.8220473	0.680644675	0.16479378	4.130281334	3.62E-05	0.000230086
ENSG00000186591	631.2241794	0.461560408	0.082136064	5.619460968	1.92E-08	2.26E-07
ENSG00000186594	27.92178705	-0.237940338	0.326897713	-0.727873976	0.466690735	0.628895314
ENSG00000186615	38.67097085	0.224429092	0.281870156	0.796214455	0.425907397	0.593170852
ENSG00000186625	120.1537257	0.630472775	0.173597357	3.631810909	0.000281439	0.001453982
ENSG00000186635	604.6169582	0.780497856	0.090708724	8.604440947	7.67E-18	2.25E-16

ENSG00000186638	146.5229895	-0.497997837	0.152359281	-3.268575661	0.001080903	0.004817081
ENSG00000186648	17.70719385	-0.826969342	0.41913144	-1.973054903	0.048489307	0.119099371
ENSG00000186654	80.6445544	-0.522989727	0.200016544	-2.614732345	0.00892974	0.029328048
ENSG00000186660	449.5829316	-0.067496405	0.098204496	-0.687304634	0.491890779	0.651295106
ENSG00000186665	107.4358827	0.023687682	0.17214143	0.137605931	0.890551868	0.940145956
ENSG00000186666	23.60197023	-0.079726563	0.362164578	-0.220139042	0.82576287	0.898324082
ENSG00000186684	58.96820963	-0.657585809	0.234335875	-2.806167898	0.005013456	0.017946998
ENSG00000186687	139.1743644	0.356472319	0.157789607	2.259162223	0.023873296	0.066696139
ENSG00000186716	618.5262995	0.184602102	0.086213282	2.14122578	0.032255835	0.085361288
ENSG00000186765	26.63388962	0.956441291	0.3649325	2.62087178	0.008770524	0.028899731
ENSG00000186767	78.04260348	0.13985068	0.202242984	0.6914983	0.489252449	0.649021541
ENSG00000186787	21.62532672	0.11282814	0.388469158	0.290442981	0.771477366	0.863328623
ENSG00000186792	41.94569206	-0.11735212	0.265493571	-0.442014922	0.658478408	0.781352103
ENSG00000186806	82.36569516	-0.360269537	0.201881467	-1.784559733	0.074332758	0.166292761
ENSG00000186812	201.9556408	0.146707102	0.137955931	1.06343454	0.287584936	0.45760145
ENSG00000186814	135.877904	-0.51662946	0.159993612	-3.229063053	0.001241965	0.005424916
ENSG00000186815	206.8149231	-0.471843723	0.131600379	-3.585428295	0.000336525	0.001701646
ENSG00000186834	531.6386698	-0.18828213	0.088948962	-2.116743408	0.034281631	0.089909273
ENSG00000186862	32.49350068	-0.457906895	0.307449656	-1.489371952	0.13638945	0.265918808
ENSG00000186866	141.8334413	0.434335589	0.160140183	2.712221134	0.006683399	0.022870087
ENSG00000186868	45.8246197	-0.001820808	0.272617902	-0.006678975	0.994670988	0.99758811
ENSG00000186871	163.6317186	-0.259061147	0.142758304	-1.814683559	0.069572553	0.158264883
ENSG00000186897	70.25050402	-0.849865282	0.213824951	-3.97458425	7.05E-05	0.00042011
ENSG00000186907	13.06028553	0.105327859	0.48167803	0.218668597	0.826908213	0.898668717
ENSG00000186908	156.9301038	-0.615780837	0.146832768	-4.193756244	2.74E-05	0.000178845
ENSG00000186918	260.4931808	0.258059082	0.117526044	2.195760812	0.028109068	0.076417278
ENSG00000186951	76.81545588	-0.751853954	0.217861804	-3.451059073	0.000558391	0.002670658
ENSG00000186998	51.32094855	0.775588376	0.247913121	3.128468446	0.001757199	0.00732227
ENSG00000187013	31.87653885	0.002119938	0.352787253	0.006009113	0.995205451	0.997940364
ENSG00000187017	105.623365	-0.841648091	0.177119244	-4.751872652	2.02E-06	1.68E-05
ENSG00000187024	46.89192438	0.385822522	0.259355185	1.487622164	0.136850565	0.26648561
ENSG00000187049	37.89838631	0.278978325	0.298179722	0.93560462	0.349476795	0.521523482
ENSG00000187051	558.691944	0.216040259	0.097009563	2.2269996	0.025947301	0.071551184
ENSG00000187079	1188.970902	0.107172127	0.07027838	1.524965806	0.127267627	0.252485707
ENSG00000187091	46.44231617	-0.09653465	0.269738136	-0.357882841	0.720430992	0.826383648
ENSG00000187097	148.286404	0.043866912	0.148303259	0.295791959	0.767388964	0.860009596
ENSG00000187109	3004.818271	-0.118088803	0.059624491	-1.980541911	0.047642669	0.117409924
ENSG00000187118	212.3590385	0.006153972	0.125637076	0.048982131	0.960933536	0.979339286
ENSG00000187147	442.5952051	0.082284838	0.094781811	0.868150089	0.385312173	0.554220656
ENSG00000187164	78.34378093	0.015444671	0.215522182	0.071661631	0.942871191	0.968520046
ENSG00000187187	49.69671356	-0.006023592	0.262111512	-0.022981026	0.981665408	0.990764045
ENSG00000187189	56.13316311	-0.509979537	0.234101918	-2.178450912	0.029372483	0.079078779
ENSG00000187239	522.5455717	-0.075059554	0.08836037	-0.849470801	0.395619371	0.563445737
ENSG00000187240	1106.12369	-0.031635138	0.079152286	-0.399674347	0.689396389	0.804503503
ENSG00000187244	276.2414484	-1.600928125	0.123526439	-12.96020622	2.06E-38	1.52E-36
ENSG00000187257	332.7549049	0.063901178	0.110991916	0.575728217	0.5647989	0.710778833
ENSG00000187260	9.540231138	-1.916475273	0.641580745	-2.987114699	0.00281624	0.010940191
ENSG00000187266	84.04647375	-0.451297007	0.195878077	-2.303968952	0.021224386	0.060634321
ENSG00000187325	75.16340434	0.388550269	0.20802929	1.867767126	0.061794537	0.144238596
ENSG00000187446	506.7818654	-0.051248158	0.100822754	-0.508299526	0.611243303	0.747205175

ENSG00000187475	17.16063588	1.961629914	0.461085063	4.25437749	2.10E-05	0.00014029
ENSG00000187486	20.5474551	0.052369869	0.377906173	0.138579025	0.889782819	0.939631412
ENSG00000187514	6502.2208	0.233182257	0.050744314	4.595239096	4.32E-06	3.35E-05
ENSG00000187522	61.30450645	0.271897767	0.229895724	1.182700412	0.236927901	0.398196485
ENSG00000187531	261.8851498	0.539190619	0.136250871	3.957337049	7.58E-05	0.000448451
ENSG00000187535	303.9437113	-0.493888733	0.109403805	-4.514365235	6.35E-06	4.75E-05
ENSG00000187546	395.8214975	-0.592544949	0.105317307	-5.626282748	1.84E-08	2.18E-07
ENSG00000187555	1260.492482	0.177274109	0.070203735	2.525137869	0.011565291	0.036490934
ENSG00000187583	51.05433913	-0.577803995	0.260414823	-2.218783047	0.026501488	0.072822066
ENSG00000187595	11.81680389	-0.682768183	0.527081019	-1.295376153	0.1951905	0.346334948
ENSG00000187601	80.23220231	-0.853560786	0.204316408	-4.177641886	2.95E-05	0.000190405
ENSG00000187605	368.946498	-0.676952287	0.102431604	-6.608822497	3.87E-11	6.45E-10
ENSG00000187607	37.93939422	0.087783365	0.311220379	0.282061751	0.777896161	0.86702431
ENSG00000187608	329.2618512	1.783034362	0.125672326	14.18796345	1.09E-45	1.01E-43
ENSG00000187609	45.38026257	-0.159635221	0.273610608	-0.583439445	0.559597511	0.70625932
ENSG00000187624	100.9466912	0.245771201	0.182430631	1.34720359	0.177914672	0.323391227
ENSG00000187626	22.36657927	0.158812395	0.378518518	0.419563079	0.674804665	0.793044913
ENSG00000187630	14.45535387	-0.259717269	0.461094369	-0.56326272	0.573256	0.717878752
ENSG00000187634	115.0181949	-0.335391393	0.173162821	-1.936855681	0.052762987	0.127172561
ENSG00000187672	17.24542073	-5.570565425	1.05256232	-5.292385373	1.21E-07	1.26E-06
ENSG00000187676	107.780716	0.070341761	0.171180611	0.410921307	0.681130238	0.798112265
ENSG00000187678	2453.993576	-0.340933392	0.062437946	-5.460355633	4.75E-08	5.28E-07
ENSG00000187699	12.42024647	0.630049292	0.493851874	1.275785969	0.202031189	0.354594883
ENSG00000187713	85.59891401	-0.100855515	0.190036638	-0.530716161	0.595615485	0.735203051
ENSG00000187720	54.35154789	-1.030771816	0.245312476	-4.201872785	2.65E-05	0.000173032
ENSG00000187735	132.903377	0.124514517	0.159722848	0.779566094	0.43564632	0.601782265
ENSG00000187736	106.5270712	-0.467076616	0.174017855	-2.684072943	0.007273124	0.024653853
ENSG00000187741	543.0143138	0.081011129	0.102458952	0.790669109	0.429137107	0.59601433
ENSG00000187742	323.988911	-0.192903718	0.107381978	-1.796425441	0.072426881	0.163146311
ENSG00000187764	92.76529153	0.108164034	0.185291315	0.583751237	0.55938769	0.706121395
ENSG00000187772	533.1290754	-0.471038613	0.090353392	-5.213291997	1.86E-07	1.87E-06
ENSG00000187778	339.666056	0.11720779	0.107078105	1.094600901	0.273691521	0.4419017
ENSG00000187790	135.877201	-0.062497034	0.15959301	-0.39160258	0.695351881	0.808571942
ENSG00000187792	234.4699618	0.198151091	0.123316442	1.606850533	0.10808716	0.222886133
ENSG00000187796	13.3782535	1.14311532	0.510290646	2.240125953	0.025082747	0.069453449
ENSG00000187801	148.3016458	-0.047483477	0.149201223	-0.318251262	0.750294351	0.84850184
ENSG00000187808	16.23784843	1.21588725	0.437282755	2.780551571	0.005426664	0.019157665
ENSG00000187824	61.05587187	-0.363829089	0.226732682	-1.604660985	0.108568432	0.223648742
ENSG00000187837	3122.065661	-0.015859694	0.070817641	-0.223951181	0.822795291	0.896348169
ENSG00000187840	323.7952737	-0.178699393	0.119770144	-1.492019517	0.135694029	0.264919253
ENSG00000187860	19.32442957	-1.213195334	0.408113152	-2.972693551	0.00295199	0.011378779
ENSG00000187866	49.38716621	-0.127186578	0.247749361	-0.513367934	0.607693972	0.744291326
ENSG00000187867	139.5667778	-1.238454818	0.168718661	-7.34035469	2.13E-13	4.51E-12
ENSG00000187902	17.02543321	-0.183577002	0.421006232	-0.436043432	0.662805218	0.784604829
ENSG00000187944	21.96879652	0.538545973	0.403176755	1.335756505	0.181628874	0.328354098
ENSG00000187954	93.51634091	-0.292103606	0.190974949	-1.52953886	0.126130913	0.25112853
ENSG00000187961	151.0498856	-0.037088037	0.156982758	-0.236255478	0.813234433	0.890235667
ENSG00000188010	28.18212739	0.097687446	0.323207967	0.302243311	0.76246659	0.856780379
ENSG00000188021	413.0323485	0.182081325	0.097368034	1.870031856	0.061479394	0.143598446
ENSG00000188026	457.9964439	0.497162739	0.096358847	5.159492408	2.48E-07	2.45E-06

ENSG00000188033	29.30452368	-0.017122574	0.329708758	-0.051932421	0.958582541	0.977723566
ENSG00000188070	446.3519057	0.525813594	0.098754226	5.324466743	1.01E-07	1.06E-06
ENSG00000188092	39.39241478	0.275836634	0.278810647	0.989333218	0.322500137	0.494364363
ENSG00000188130	357.8515758	-0.141348365	0.102746555	-1.375699304	0.168914748	0.311407528
ENSG00000188153	226.5768851	-0.385156923	0.130747156	-2.945814923	0.003221051	0.012278453
ENSG00000188157	478.0955986	-0.266423594	0.106762975	-2.495468063	0.012579108	0.039205457
ENSG00000188167	104.5293964	-0.251061641	0.180065422	-1.394280135	0.163233027	0.30356937
ENSG00000188177	123.5894515	-0.728925301	0.165251768	-4.410998508	1.03E-05	7.36E-05
ENSG00000188185	54.65104502	-0.116949851	0.238646474	-0.490054805	0.624095118	0.75594241
ENSG00000188186	481.676512	0.188421485	0.109039286	1.728014666	0.083985595	0.183532304
ENSG00000188191	162.4031734	0.37418357	0.141759015	2.639575115	0.008301003	0.027624777
ENSG00000188211	737.2148359	0.142782925	0.080799218	1.7671325	0.077206041	0.171652224
ENSG00000188215	68.39756058	0.018298866	0.217613626	0.084088789	0.932985838	0.963270344
ENSG00000188219	197.1470546	0.273259337	0.145053596	1.883850827	0.059585156	0.139973162
ENSG00000188229	1909.304634	0.005566767	0.064642752	0.086115869	0.931374309	0.962835952
ENSG00000188234	10.46903802	-0.212242672	0.530740664	-0.399899021	0.689230894	0.804480669
ENSG00000188243	485.6362649	0.178974943	0.098052145	1.825303694	0.067955213	0.155542916
ENSG00000188263	8.548186231	-1.553983116	0.63223694	-2.457912561	0.013974719	0.042804816
ENSG00000188266	88.26477339	-0.724792468	0.196178395	-3.694558044	0.00022027	0.001166275
ENSG00000188277	18.18804938	-0.563491346	0.420092289	-1.34135132	0.179806415	0.325817945
ENSG00000188283	33.15002852	0.170659556	0.306213075	0.55732289	0.577306839	0.721094538
ENSG00000188290	128.5767241	-1.179179091	0.183045201	-6.442010429	1.18E-10	1.85E-09
ENSG00000188295	62.71852769	-0.026287253	0.222403594	-0.11819617	0.905912225	0.949652912
ENSG00000188312	97.05883189	-0.036079349	0.182426088	-0.19777516	0.843220981	0.909104699
ENSG00000188313	83.98461851	1.09537314	0.20401983	5.368954272	7.92E-08	8.45E-07
ENSG00000188315	87.04394554	-0.121555937	0.198520743	-0.612308493	0.540333672	0.690319881
ENSG00000188322	62.77292531	-0.548125125	0.262808273	-2.08564639	0.037010666	0.095673321
ENSG00000188338	47.22414289	-0.606253071	0.261918835	-2.31466008	0.020631542	0.059278231
ENSG00000188342	174.0726883	-0.125118126	0.139412427	-0.897467527	0.369469494	0.540900258
ENSG00000188343	244.1750326	-0.01023393	0.125956375	-0.081249798	0.935243297	0.964705728
ENSG00000188352	253.2857634	0.229691715	0.120111161	1.912326166	0.055834372	0.132980141
ENSG00000188368	12.25161275	0.750182727	0.512589161	1.463516565	0.143326084	0.275605956
ENSG00000188372	56.73916369	0.234240624	0.230611905	1.015735177	0.309755512	0.481094524
ENSG00000188385	104.6189668	1.953413796	0.196087313	9.961959135	2.24E-23	9.03E-22
ENSG00000188394	10.74440135	0.017212302	0.539256622	0.031918573	0.974536987	0.9869734
ENSG00000188419	285.5565711	-0.08732791	0.115338545	-0.757144197	0.448963478	0.613959304
ENSG00000188428	42.01810023	-0.463009309	0.274533414	-1.686531715	0.091693432	0.196187994
ENSG00000188483	374.8637212	0.047511724	0.110583773	0.429644629	0.667454168	0.787413177
ENSG00000188486	885.9238993	-0.153748736	0.100760268	-1.525886527	0.127038127	0.252210352
ENSG00000188493	136.155316	-0.054825862	0.15554166	-0.352483457	0.724475722	0.829465358
ENSG00000188517	10.19454212	0.60456776	0.557664205	1.084107166	0.278317286	0.447160347
ENSG00000188529	1135.445326	-0.066587214	0.070235811	-0.948052186	0.343102888	0.515020973
ENSG00000188542	36.57023703	-0.954502469	0.321448635	-2.969377888	0.002984034	0.011484034
ENSG00000188554	669.7586253	0.341281623	0.089785663	3.80107036	0.000144072	0.000799571
ENSG00000188559	146.6029401	-0.004291089	0.154581337	-0.027759427	0.977854026	0.988629033
ENSG00000188566	78.88110167	-0.181201234	0.21368658	-0.847976667	0.396450965	0.564286828
ENSG00000188582	14.03057782	-0.111372493	0.455814665	-0.24433723	0.806969652	0.8858602
ENSG00000188603	10.81274988	0.660474621	0.529364083	1.247675547	0.2121499	0.367255658
ENSG00000188610	15.49313316	-0.368531241	0.471663245	-0.781343988	0.434600205	0.600869004
ENSG00000188612	1459.418475	0.157121184	0.075193394	2.089560993	0.036657254	0.095001498

ENSG00000188613	8.560982508	-1.331978201	0.617361208	-2.157534654	0.03096403	0.082596227
ENSG00000188636	379.7801095	-0.064469602	0.099848442	-0.64567459	0.51849012	0.673375621
ENSG00000188641	75.34611613	0.51557698	0.208558659	2.47209578	0.013432352	0.041441742
ENSG00000188647	455.4363614	0.018838904	0.096694264	0.194829595	0.845526352	0.910611721
ENSG00000188659	33.17950858	-0.281606455	0.300651097	-0.936655337	0.348935877	0.520882281
ENSG00000188677	274.6511536	-0.817584502	0.116119042	-7.040916718	1.91E-12	3.71E-11
ENSG00000188690	501.6204558	0.099962593	0.093713788	1.066679681	0.286116505	0.455781192
ENSG00000188706	327.9461477	0.565486536	0.111791263	5.058414422	4.23E-07	4.04E-06
ENSG00000188725	241.294113	0.184533671	0.129192166	1.428365798	0.153186598	0.289728308
ENSG00000188729	158.684688	-2.567056119	0.177890208	-14.43056452	3.32E-47	3.18E-45
ENSG00000188735	231.9548079	0.235562575	0.12412488	1.897786927	0.057724156	0.136346262
ENSG00000188739	11.81311959	0.055404324	0.499001012	0.111030484	0.911592174	0.952621281
ENSG00000188747	25.56838334	0.302222828	0.342422727	0.882601544	0.377451594	0.546942701
ENSG00000188760	22.18154766	-0.403174686	0.379242614	-1.063104912	0.287734377	0.457787382
ENSG00000188763	15.03166598	0.26168006	0.473738024	0.552372929	0.580692874	0.723518357
ENSG00000188766	109.2759795	-0.067187304	0.181703384	-0.369763638	0.71155861	0.819754269
ENSG00000188785	52.24182937	-0.067776859	0.251927059	-0.26903366	0.78790378	0.873121205
ENSG00000188786	164.1469585	0.184586996	0.152438683	1.210893405	0.22593626	0.384346812
ENSG00000188807	259.7133094	0.040866473	0.121037218	0.337635595	0.735637815	0.83739245
ENSG00000188811	161.4653816	0.112037442	0.154970067	0.722961825	0.469703338	0.631654585
ENSG00000188818	87.42805443	-0.035897647	0.199185707	-0.180222003	0.856978286	0.918060409
ENSG00000188827	268.2220501	0.163055925	0.115929177	1.406513263	0.159571769	0.298270744
ENSG00000188846	2344.088793	0.114559445	0.059355849	1.930044758	0.053601292	0.128663252
ENSG00000188848	28.87460213	-3.635701051	0.492431261	-7.383164594	1.55E-13	3.35E-12
ENSG00000188878	41.34029837	-0.185941542	0.267621135	-0.694793937	0.487184448	0.647676578
ENSG00000188883	11.45822057	-1.512581459	0.575667169	-2.627527745	0.008600783	0.028447185
ENSG00000188895	641.6480491	0.226674635	0.085830182	2.640966486	0.00826699	0.027524628
ENSG00000188897	77.15158417	0.361212487	0.207577261	1.740135143	0.081835291	0.179784224
ENSG00000188906	182.1679953	0.500495391	0.147595635	3.39099047	0.000696405	0.003258476
ENSG00000188916	11.56305945	0.561599173	0.508681576	1.104028923	0.269580591	0.436770308
ENSG00000188917	132.8195731	0.220554136	0.15742118	1.40104487	0.161200656	0.300555563
ENSG00000188938	342.1174246	-0.097094205	0.114648065	-0.846889175	0.397056897	0.56492031
ENSG00000188958	27.67807268	-0.075086834	0.335596887	-0.223741152	0.822958723	0.896348169
ENSG00000188976	952.6493824	-0.012710281	0.084558765	-0.150312995	0.880517681	0.934116144
ENSG00000188986	355.8879544	0.20031046	0.106823307	1.875156889	0.060771143	0.142346548
ENSG00000188994	873.9432502	-0.173127854	0.077147961	-2.244101478	0.024825873	0.068837157
ENSG00000188997	44.90180661	0.155536373	0.261861813	0.593963553	0.552536491	0.69986274
ENSG00000189007	143.5924922	0.320637808	0.150969844	2.123853334	0.03368241	0.088519515
ENSG00000189042	116.1159841	0.544837507	0.169448432	3.21535879	0.001302816	0.005651838
ENSG00000189043	1397.912768	0.412117371	0.075977597	5.424195899	5.82E-08	6.37E-07
ENSG00000189046	127.3800185	-0.006143496	0.164555958	-0.037333782	0.97021887	0.984516685
ENSG00000189050	86.88527364	0.024914841	0.190485995	0.130796182	0.895936544	0.943561301
ENSG00000189057	61.88453932	-1.721483868	0.246363205	-6.987585132	2.80E-12	5.28E-11
ENSG00000189060	2194.732149	-0.011463238	0.062699831	-0.182827249	0.854933567	0.916666806
ENSG00000189067	590.4642569	0.097916936	0.088144861	1.110863809	0.266626976	0.433380991
ENSG00000189077	120.7708312	0.316039082	0.173414183	1.822452334	0.06838638	0.156224342
ENSG00000189079	1023.05562	-0.18725674	0.093004512	-2.013415649	0.044070921	0.110507715
ENSG00000189091	1540.30625	0.180701767	0.064397548	2.806034887	0.005015526	0.017949833
ENSG00000189114	49.51606906	0.327325444	0.256591969	1.275665194	0.202073897	0.354594883
ENSG00000189127	12.86170792	-0.075032451	0.496495474	-0.151124139	0.879877791	0.934004754

ENSG00000189129	40.15720283	1.8284466	0.298024181	6.135228999	8.50E-10	1.19E-08
ENSG00000189144	26.74754702	0.355906193	0.349490819	1.018356344	0.308508641	0.479754332
ENSG00000189149	8.960712991	-1.251023878	0.617131081	-2.027160705	0.04264598	0.107701599
ENSG00000189159	924.1372054	0.492762245	0.076065443	6.478135463	9.29E-11	1.48E-09
ENSG00000189164	22.75733394	0.198248982	0.361798829	0.547953631	0.583723742	0.725753886
ENSG00000189171	102.2829884	-0.760622201	0.353709562	-2.150414586	0.031522436	0.083799329
ENSG00000189180	431.3480626	-0.118140566	0.098624154	-1.197886738	0.230961113	0.390457427
ENSG00000189184	1950.730381	1.497712044	0.075611001	19.80812358	2.53E-87	5.48E-85
ENSG00000189195	35.87976204	-0.140778374	0.297099917	-0.473841849	0.635612689	0.764749588
ENSG00000189221	8.551805542	-4.541938928	1.107492927	-4.101099715	4.11E-05	0.000257627
ENSG00000189227	36.48837815	0.230514422	0.29460937	0.782440906	0.433955502	0.600174999
ENSG00000189241	1299.199174	-0.224571073	0.069907156	-3.212418962	0.001316223	0.005701197
ENSG00000189266	403.0910075	-0.290814646	0.106954219	-2.719057266	0.006546827	0.022479414
ENSG00000189283	70.51724613	-0.274740817	0.21050645	-1.305142038	0.191844487	0.342028113
ENSG00000189298	72.80849829	-0.621127202	0.224334751	-2.768751602	0.005627152	0.019756212
ENSG00000189306	703.4049073	0.584614996	0.080815957	7.233905523	4.69E-13	9.68E-12
ENSG00000189308	287.1176796	-0.202949072	0.116387223	-1.743740137	0.081204413	0.178649709
ENSG00000189319	255.240617	0.100257092	0.136193857	0.736135198	0.461648424	0.624677335
ENSG00000189337	45.40417592	-1.234879426	0.274060251	-4.505868398	6.61E-06	4.92E-05
ENSG00000189339	191.1057346	0.104583942	0.144500983	0.723759384	0.469213468	0.631144459
ENSG00000189343	42.53220992	0.134499195	0.2789006	0.482247778	0.62962994	0.759894472
ENSG00000189362	53.17894141	-0.546554787	0.246379746	-2.218343009	0.026531454	0.072875896
ENSG00000189369	104.655133	-0.267067327	0.176278131	-1.515033801	0.129763844	0.256334232
ENSG00000189376	38.65688227	0.325609502	0.290775458	1.119797056	0.262800254	0.429250601
ENSG00000189403	1228.379701	0.181646393	0.079545891	2.28354212	0.022398455	0.063191624
ENSG00000189410	30.76435994	-0.167155581	0.339496964	-0.49236252	0.62246309	0.75505595
ENSG00000196072	88.11880163	0.100149213	0.189496426	0.528501859	0.597151057	0.736571392
ENSG00000196074	298.5597911	-2.664724575	0.135005992	-19.7378245	1.02E-86	2.14E-84
ENSG00000196081	69.65892805	-0.325700088	0.215169817	-1.513688547	0.130104851	0.256827269
ENSG00000196110	59.43501664	0.318981971	0.225981327	1.411541279	0.158085073	0.296319858
ENSG00000196116	30.7009789	0.755995579	0.320946732	2.355517301	0.018496936	0.054175658
ENSG00000196118	54.38425118	0.085455775	0.243437103	0.351038417	0.725559527	0.830232541
ENSG00000196123	130.0344452	-0.526706509	0.162040556	-3.250461008	0.001152181	0.005084132
ENSG00000196132	8.872385761	-2.750791809	0.762218932	-3.608926116	0.000307467	0.001573196
ENSG00000196141	54.49414945	0.491872524	0.242565951	2.027788822	0.042581806	0.107599681
ENSG00000196150	92.77395258	-0.495611937	0.20732534	-2.390503435	0.016825292	0.050149691
ENSG00000196151	32.78468194	-0.000736232	0.307275977	-0.002395995	0.998088275	0.999084016
ENSG00000196152	44.35541704	-0.188922652	0.2621535	-0.720656608	0.471120816	0.633103807
ENSG00000196154	241.0372936	-0.57311561	0.129248151	-4.434226761	9.24E-06	6.66E-05
ENSG00000196155	14.93794923	5.018029581	0.911104873	5.50763115	3.64E-08	4.13E-07
ENSG00000196159	606.9553486	-1.773950879	0.095150561	-18.64361968	1.42E-77	2.47E-75
ENSG00000196169	6.351186355	-2.22866935	0.820765384	-2.715354954	0.006620477	0.022687934
ENSG00000196177	101.4075633	-0.329753297	0.180230965	-1.829615115	0.067307512	0.154337026
ENSG00000196182	406.2219594	0.076659693	0.097460613	0.786571017	0.431533031	0.597805177
ENSG00000196187	283.2732734	0.241494627	0.116736255	2.06872002	0.038572367	0.098945589
ENSG00000196189	16.61156741	-0.501260776	0.459681162	-1.090453161	0.275513575	0.443874205
ENSG00000196199	728.1275221	0.015265764	0.088065735	0.173345109	0.862380168	0.921766415
ENSG00000196204	111.532366	0.184563478	0.177887825	1.037527315	0.299490173	0.469943974
ENSG00000196205	28.56523955	0.251859401	0.345130807	0.729750564	0.465542667	0.628131577
ENSG00000196208	155.2220616	-1.144989743	0.1518676	-7.539394488	4.72E-14	1.07E-12

ENSG00000196214	127.7917913	-0.07033223	0.16237344	-0.433151074	0.66490502	0.785659558
ENSG00000196220	90.96314285	-0.860590395	0.207720836	-4.143014311	3.43E-05	0.000218859
ENSG00000196227	319.4792849	-0.361302315	0.115477796	-3.128760045	0.001755456	0.007317178
ENSG00000196230	5056.231132	0.06081054	0.062971176	0.965688491	0.33420008	0.505990311
ENSG00000196233	980.5253304	-0.169432848	0.071952772	-2.354778604	0.018533744	0.054272151
ENSG00000196235	1132.80359	0.197908607	0.070671019	2.800421022	0.005103599	0.018200117
ENSG00000196236	378.5972256	0.00393368	0.100098945	0.039297914	0.96865287	0.983424201
ENSG00000196247	535.3580738	0.063082059	0.090415444	0.697691193	0.485370337	0.645704813
ENSG00000196262	1320.684114	0.191779662	0.077819279	2.464423519	0.013723385	0.042172913
ENSG00000196263	19.09372818	0.083028509	0.401656108	0.206715414	0.836232118	0.904331203
ENSG00000196284	40.51321235	-0.343564934	0.276973673	-1.240424516	0.214818418	0.370873549
ENSG00000196290	171.727788	0.162384967	0.142305451	1.141101526	0.253827676	0.418674985
ENSG00000196295	246.9179556	-0.067646691	0.128027775	-0.528375117	0.597239005	0.736571392
ENSG00000196296	24.40205444	-1.120049467	0.367751755	-3.045667221	0.002321646	0.00927536
ENSG00000196305	1901.186669	0.001251058	0.062746595	0.019938254	0.984092629	0.991961125
ENSG00000196313	758.5458777	0.151012012	0.0792731	1.904959076	0.056785391	0.134740606
ENSG00000196323	153.1375109	-0.571293767	0.165333428	-3.455403877	0.000549469	0.002636963
ENSG00000196337	10.89289963	-1.034721784	0.581126962	-1.780543412	0.074987071	0.167436559
ENSG00000196338	56.78336626	-0.655208277	0.253527627	-2.584366386	0.00975581	0.031597846
ENSG00000196352	199.2509484	0.663516376	0.133115356	4.984521652	6.21E-07	5.76E-06
ENSG00000196357	14.44015989	0.460605648	0.458556971	1.00446766	0.315153252	0.486585813
ENSG00000196358	16.55145909	1.274011363	0.44148699	2.885727985	0.003905095	0.014488872
ENSG00000196361	15.5754462	1.376958783	0.460439282	2.990532818	0.002784912	0.01083047
ENSG00000196363	340.5031178	0.110003474	0.113996524	0.964972177	0.334558748	0.506275374
ENSG00000196365	2341.486625	0.419132544	0.071280122	5.880076164	4.10E-09	5.27E-08
ENSG00000196367	2320.700201	-0.004463738	0.058284619	-0.076585178	0.93895355	0.966262309
ENSG00000196368	80.89538401	-0.142255412	0.194415151	-0.731709495	0.4643459	0.627179505
ENSG00000196369	17.5719994	-0.429402349	0.410265387	-1.046645323	0.29526319	0.465756545
ENSG00000196372	294.6141307	-0.127789585	0.118093705	-1.082103269	0.279206643	0.44787207
ENSG00000196378	54.17408672	-0.090103449	0.235978262	-0.381829446	0.702587876	0.813891794
ENSG00000196387	46.89689003	-0.032040182	0.255832823	-0.125238747	0.900334543	0.946135956
ENSG00000196391	23.62337691	0.017466862	0.362334666	0.048206433	0.961551723	0.979684997
ENSG00000196396	164.9367396	-0.220518188	0.143366181	-1.538146491	0.124012801	0.248072881
ENSG00000196405	353.8191171	0.195630749	0.126884884	1.54179712	0.123122902	0.246860891
ENSG00000196411	248.6837049	-0.025799324	0.125618927	-0.205377681	0.837277057	0.905074151
ENSG00000196417	60.23090354	0.331220348	0.230363242	1.437817708	0.150485784	0.285851765
ENSG00000196418	59.1157867	-0.091929296	0.246323703	-0.373205237	0.708995698	0.817673446
ENSG00000196419	2610.276269	0.309915343	0.059268853	5.228974884	1.70E-07	1.73E-06
ENSG00000196421	13.14275431	-0.630533039	0.477957456	-1.319224191	0.187094173	0.335389465
ENSG00000196422	231.7024117	-0.220159264	0.122836436	-1.792296095	0.073085548	0.164182424
ENSG00000196428	378.1534405	0.036982729	0.098984056	0.373623089	0.708684753	0.817516197
ENSG00000196437	75.47588376	0.100198827	0.20708075	0.483863551	0.628482709	0.759031607
ENSG00000196440	308.8532081	0.236391766	0.111806254	2.114298242	0.034489802	0.090337172
ENSG00000196449	163.3012191	0.150396902	0.146583696	1.026013851	0.304885055	0.475857819
ENSG00000196453	348.517477	0.338574012	0.105004957	3.224362182	0.001262536	0.005500882
ENSG00000196455	222.0897009	-0.008898506	0.130975037	-0.067940475	0.945833019	0.969848951
ENSG00000196456	66.34534773	0.335536521	0.220056718	1.524772903	0.127315751	0.252492278
ENSG00000196458	89.06431017	-0.108655962	0.192692912	-0.563881471	0.572834801	0.717717124
ENSG00000196459	70.47049513	0.529699281	0.209261148	2.531283452	0.011364597	0.035954615
ENSG00000196465	209.4328591	-0.212549666	0.126623986	-1.678589286	0.093232116	0.198699975

ENSG00000196470	79.59972472	-0.361021599	0.19973371	-1.80751461	0.070682079	0.160241062
ENSG00000196476	93.10266622	-0.344376953	0.189434719	-1.817918883	0.069076532	0.157468036
ENSG00000196482	125.2218068	-2.819361613	0.20966656	-13.44688257	3.21E-41	2.62E-39
ENSG00000196497	9.865021192	-0.272011949	0.550115366	-0.494463463	0.620978904	0.753988302
ENSG00000196498	3275.833025	0.364114638	0.061835429	5.888446829	3.90E-09	5.03E-08
ENSG00000196504	1801.704459	-0.002965913	0.064464351	-0.04600858	0.963303411	0.980758355
ENSG00000196505	161.5319959	-0.298923913	0.148192284	-2.017135472	0.043681378	0.109785706
ENSG00000196507	161.9605926	0.639978418	0.148865813	4.299028799	1.72E-05	0.000116847
ENSG00000196510	328.0660601	-0.135347908	0.108247949	-1.250350782	0.211171435	0.366275621
ENSG00000196511	23.38260753	-0.026868768	0.357165157	-0.075227853	0.940033424	0.966948558
ENSG00000196517	186.27983	-1.294674357	0.140938462	-9.186096809	4.07E-20	1.38E-18
ENSG00000196526	114.7507108	-1.497412579	0.182977522	-8.18358758	2.76E-16	7.29E-15
ENSG00000196531	1035.603017	0.088440301	0.088741322	0.99660788	0.318954879	0.490460776
ENSG00000196535	493.9390455	-0.23115226	0.09437267	-2.449355939	0.014311195	0.043711535
ENSG00000196544	78.35719489	-0.00585098	0.20534857	-0.02849292	0.977269015	0.988629033
ENSG00000196547	629.0284818	0.515480118	0.089396035	5.766252562	8.11E-09	1.01E-07
ENSG00000196550	14.76990493	0.050473513	0.448890404	0.112440615	0.910474053	0.951594421
ENSG00000196557	14.24851999	0.817967751	0.47355028	1.727309191	0.084112149	0.183637451
ENSG00000196562	7.81448497	-2.272714827	0.726286147	-3.129227836	0.001752664	0.007309877
ENSG00000196576	1393.876842	0.258882177	0.075445714	3.431370214	0.00060054	0.002853786
ENSG00000196584	326.7370964	0.380468593	0.106747247	3.564200522	0.000364967	0.001831615
ENSG00000196586	12.38620872	-2.459866869	0.599670155	-4.102033172	4.10E-05	0.000256934
ENSG00000196588	351.1544028	-0.365627076	0.104426133	-3.501298642	0.000462997	0.002260589
ENSG00000196591	1062.30649	-0.012025709	0.072853192	-0.165067707	0.868890691	0.926961915
ENSG00000196597	133.104027	-0.456517874	0.158627279	-2.877927912	0.004002966	0.014812872
ENSG00000196604	99.66228174	-0.013329769	0.196053226	-0.067990564	0.945793145	0.969848951
ENSG00000196605	82.92675213	0.388794001	0.204371257	1.902390809	0.057120082	0.135260585
ENSG00000196628	590.1086403	-1.075157254	0.089328098	-12.03604778	2.30E-33	1.40E-31
ENSG00000196632	136.5676014	0.029889598	0.156688074	0.190758603	0.848714723	0.91320534
ENSG00000196636	58.40445677	0.048634578	0.228782497	0.212579977	0.831654591	0.901477895
ENSG00000196642	1634.490991	0.058253274	0.067248995	0.866232622	0.386362607	0.555102336
ENSG00000196646	22.90228117	-0.093264602	0.367709524	-0.253636624	0.7997763	0.880907053
ENSG00000196652	192.6093299	-0.013715396	0.133795261	-0.102510329	0.918351615	0.955355281
ENSG00000196655	117.6910653	-0.092618767	0.167748884	-0.552127471	0.580861022	0.723539485
ENSG00000196663	129.9754545	-0.53586798	0.162118502	-3.305409147	0.000948378	0.00428356
ENSG00000196668	20.04520072	-0.692503731	0.399158406	-1.734909551	0.082756827	0.18129837
ENSG00000196670	279.690128	-0.506397667	0.119272891	-4.24570632	2.18E-05	0.000145274
ENSG00000196678	89.01900265	0.043909234	0.19027221	0.230770611	0.817493014	0.892507031
ENSG00000196683	891.0061818	0.355007701	0.077478628	4.582008083	4.61E-06	3.55E-05
ENSG00000196689	29.94126128	-0.281714047	0.328274263	-0.858166718	0.390800411	0.559133568
ENSG00000196693	86.23530865	-0.049051855	0.187934616	-0.261004896	0.794088732	0.877406181
ENSG00000196700	226.404722	0.390207144	0.132317557	2.949020179	0.003187832	0.012165029
ENSG00000196704	610.0725835	0.069426833	0.09279177	0.748200327	0.454339334	0.618301165
ENSG00000196705	85.57041546	-0.417163271	0.193330545	-2.157772185	0.030945548	0.082587347
ENSG00000196712	951.1812806	-0.174629424	0.076922434	-2.270201491	0.023195361	0.065112956
ENSG00000196715	349.088175	-0.049682336	0.105639746	-0.470299654	0.638140945	0.766295498
ENSG00000196739	442.7235811	0.910887049	0.102442565	8.891685301	6.02E-19	1.93E-17
ENSG00000196743	314.9439564	-0.227884971	0.118932277	-1.916090199	0.055353616	0.132161437
ENSG00000196747	465.3608247	-0.129708184	0.545022974	-0.237986636	0.811891459	0.889400967
ENSG00000196756	621.2778141	0.26599103	0.09154776	2.905489217	0.003666795	0.013717219

ENSG00000196757	38.44887343	0.09550637	0.280989736	0.339892736	0.733937307	0.836005299
ENSG00000196776	202.8826591	0.498320999	0.130167571	3.828303745	0.000129029	0.000724122
ENSG00000196781	32.13068863	-3.934677078	0.502701793	-7.827059965	4.99E-15	1.20E-13
ENSG00000196782	366.3300162	0.455796035	0.104817731	4.348463098	1.37E-05	9.55E-05
ENSG00000196787	1865.401816	-0.545892691	0.076280764	-7.15636113	8.28E-13	1.65E-11
ENSG00000196792	404.0559174	-0.004306686	0.097914077	-0.043984342	0.964916885	0.981618445
ENSG00000196810	94.061197	-0.041025587	0.183877662	-0.223113491	0.823447178	0.896524765
ENSG00000196811	44.09164881	-0.419269594	0.273531852	-1.532799894	0.125325167	0.249920871
ENSG00000196814	127.2143463	-0.045436704	0.169406352	-0.268211338	0.788536648	0.873546869
ENSG00000196821	391.9660007	-0.068831961	0.102714043	-0.670131948	0.50277368	0.660722415
ENSG00000196839	95.74053785	-0.44614164	0.185493432	-2.405161381	0.016165325	0.04850979
ENSG00000196843	62.737025	0.134934379	0.219730901	0.614089228	0.539156366	0.689580557
ENSG00000196850	276.0330115	0.107013089	0.115893833	0.923371736	0.355813516	0.527227053
ENSG00000196865	538.2415981	-0.408685126	0.089218557	-4.58071886	4.63E-06	3.56E-05
ENSG00000196867	53.96799257	0.2172671	0.23959542	0.906808234	0.364508215	0.535646709
ENSG00000196872	12.49733326	-1.022429324	0.520956932	-1.962598559	0.049692838	0.121291972
ENSG00000196876	34.7901425	-0.933518755	0.30243446	-3.086681176	0.002024046	0.008257847
ENSG00000196890	155.7870173	-0.231604679	0.150886974	-1.534954758	0.124794941	0.249111062
ENSG00000196911	99.82381249	-0.349879191	0.187179525	-1.869217217	0.0615926	0.143791134
ENSG00000196912	300.0902409	-0.285344147	0.122546126	-2.328463224	0.01988752	0.057528829
ENSG00000196914	1135.874359	-0.027894566	0.071215841	-0.39169047	0.695286932	0.80856822
ENSG00000196923	679.8354562	-0.432111891	0.091473024	-4.72392703	2.31E-06	1.90E-05
ENSG00000196924	3162.47148	0.241206294	0.068862985	3.502698765	0.00046057	0.002249523
ENSG00000196935	130.661062	0.288310377	0.157431634	1.83133701	0.067050257	0.153847592
ENSG00000196937	135.2815863	1.151361754	0.190925121	6.030436162	1.64E-09	2.22E-08
ENSG00000196943	273.5377413	-0.029609648	0.116804573	-0.253497332	0.799883923	0.880907053
ENSG00000196950	539.5781755	0.278604321	0.098169853	2.837982463	0.004539968	0.016495842
ENSG00000196951	14.21264302	0.030855927	0.454648453	0.067867661	0.945890982	0.969848951
ENSG00000196961	924.573679	-0.001613077	0.088695712	-0.018186641	0.98548996	0.992555924
ENSG00000196967	12.12998791	0.568345845	0.541910813	1.048781148	0.294278856	0.46451681
ENSG00000196968	215.692974	0.523645113	0.129481036	4.044183843	5.25E-05	0.000320952
ENSG00000196975	100.2342507	-0.298817661	0.186068342	-1.60595649	0.10828347	0.223225404
ENSG00000196976	284.3247588	-0.011671283	0.117960568	-0.098942243	0.921184128	0.956727767
ENSG00000196981	16.01025085	0.235910133	0.431122412	0.547199883	0.584241416	0.726268953
ENSG00000196998	77.51069047	-0.077938841	0.204420763	-0.381266753	0.703005321	0.814114448
ENSG00000197006	520.1402665	-0.075735686	0.092563225	-0.818204919	0.413240187	0.581641774
ENSG00000197008	63.45891058	-0.747570268	0.227864869	-3.280761401	0.001035273	0.00463547
ENSG00000197016	30.50126932	0.204666349	0.319781493	0.640019368	0.522160008	0.676154371
ENSG00000197019	149.5459343	0.237607864	0.148298546	1.602226528	0.109105526	0.224590464
ENSG00000197021	59.32661808	0.611623598	0.237585215	2.574333587	0.010043339	0.032393489
ENSG00000197024	944.9564565	0.529202295	0.073852131	7.165701099	7.74E-13	1.56E-11
ENSG00000197037	126.0901379	-0.192251601	0.163943853	-1.172667333	0.240929236	0.40337701
ENSG00000197043	716.940957	-0.407094267	0.080569024	-5.052739198	4.36E-07	4.15E-06
ENSG00000197044	35.54364913	-0.723466564	0.300381486	-2.408492525	0.016018554	0.048172261
ENSG00000197045	217.4081933	0.21332797	0.12728037	1.676047686	0.093728853	0.199516471
ENSG00000197050	33.43904424	0.710435663	0.304031919	2.336714071	0.019454056	0.056460977
ENSG00000197056	202.3684419	-0.291612726	0.136023463	-2.143841351	0.032045601	0.084916913
ENSG00000197061	785.9118655	-0.124055978	0.1770536	-0.700669054	0.483509573	0.644083354
ENSG00000197062	48.74570919	-0.029372613	0.253718467	-0.115768525	0.907835996	0.950649754
ENSG00000197063	255.2195526	0.069954772	0.117277593	0.596488814	0.550848724	0.698621617

ENSG00000197070	112.0418877	0.006858036	0.173244615	0.039585853	0.968423306	0.983405059
ENSG00000197077	446.6993976	-0.263334997	0.098903467	-2.662545663	0.007755205	0.026055336
ENSG00000197081	2370.442142	-0.323494935	0.058358701	-5.543216868	2.97E-08	3.41E-07
ENSG00000197102	7122.785602	0.434208955	0.052790346	8.225158366	1.95E-16	5.20E-15
ENSG00000197111	745.6297893	0.042884442	0.085114645	0.503843282	0.61437151	0.749042347
ENSG00000197114	39.88576776	0.091711375	0.306482858	0.299238189	0.764758313	0.85830617
ENSG00000197119	72.22918805	-0.942215369	0.215084644	-4.380672418	1.18E-05	8.35E-05
ENSG00000197121	287.1942148	0.188667121	0.122446424	1.540813653	0.123362146	0.247199438
ENSG00000197122	242.2282453	0.163895717	0.118611951	1.381780808	0.167039014	0.3089397
ENSG00000197128	13.27904906	5.445768992	1.093968816	4.977992893	6.42E-07	5.94E-06
ENSG00000197136	511.3931334	0.044566871	0.115676558	0.385271418	0.700036336	0.811739343
ENSG00000197147	317.2007614	0.061291528	0.110389106	0.555231675	0.578736206	0.722173535
ENSG00000197150	239.9606497	0.83626224	0.12350586	6.7710329	1.28E-11	2.23E-10
ENSG00000197153	11.68046867	-1.279296279	0.53447769	-2.393544767	0.016686445	0.049807584
ENSG00000197157	1817.735204	0.546959158	0.061384451	8.910386129	5.09E-19	1.64E-17
ENSG00000197168	16.98179405	-0.238400331	0.417689	-0.570760377	0.568162085	0.713670107
ENSG00000197170	831.8372368	0.15980614	0.077348995	2.066040297	0.038824675	0.099483803
ENSG00000197182	129.2299925	-0.761124398	0.161306868	-4.718487233	2.38E-06	1.95E-05
ENSG00000197183	449.2369284	0.233088071	0.094491055	2.466773933	0.01363364	0.041924637
ENSG00000197217	438.5357863	-0.104599635	0.100203072	-1.04387653	0.296542518	0.46693563
ENSG00000197223	60.18009904	-0.497600344	0.231722372	-2.147398796	0.031761546	0.08427549
ENSG00000197226	1036.13538	0.364578954	0.074900793	4.867491226	1.13E-06	9.89E-06
ENSG00000197256	584.9329676	-0.361609355	0.091001239	-3.973675086	7.08E-05	0.000421244
ENSG00000197258	22.54840325	-0.070529564	0.363545737	-0.194004651	0.84617224	0.911097766
ENSG00000197265	365.0558948	-0.121896269	0.102406969	-1.190312236	0.233923693	0.394468922
ENSG00000197275	79.42300574	-0.475129634	0.20550095	-2.312055664	0.020774616	0.059603743
ENSG00000197279	19.92315674	-0.809215064	0.401909387	-2.013426634	0.044069766	0.110507715
ENSG00000197283	605.6679177	0.131441445	0.087352769	1.504719846	0.132396118	0.260398807
ENSG00000197296	49.78398179	-0.392663241	0.250884746	-1.565114052	0.117556169	0.237838328
ENSG00000197299	407.0238134	-0.175636252	0.096636042	-1.817502545	0.069140199	0.157587599
ENSG00000197301	18.44197017	-5.642920531	1.045762725	-5.39598553	6.81E-08	7.38E-07
ENSG00000197302	152.447694	-0.679226114	0.150153606	-4.523541807	6.08E-06	4.58E-05
ENSG00000197312	554.8852411	-0.310306208	0.094418846	-3.286485924	0.001014458	0.004551293
ENSG00000197321	407.1615614	-1.831511064	0.107051188	-17.10874113	1.28E-65	1.93E-63
ENSG00000197323	818.0789076	0.026919833	0.084347022	0.319155705	0.749608444	0.848021916
ENSG00000197324	424.1547636	-0.301743536	0.101213772	-2.981249773	0.002870745	0.01112117
ENSG00000197329	114.0919647	-0.058975263	0.170435833	-0.346026198	0.729323026	0.832773591
ENSG00000197332	6.945788451	-0.850219013	0.694886193	-1.223537066	0.221126913	0.378906994
ENSG00000197343	343.8098059	0.113030517	0.110452653	1.023339091	0.306147547	0.477244989
ENSG00000197345	391.7572124	0.867992348	0.125583462	6.911677158	4.79E-12	8.79E-11
ENSG00000197355	192.9957676	0.591750171	0.153104809	3.865000548	0.000111089	0.000634078
ENSG00000197362	350.0451994	0.621938247	0.104415805	5.956361172	2.58E-09	3.41E-08
ENSG00000197363	41.87109728	0.087299409	0.278331204	0.313652972	0.753784623	0.851208619
ENSG00000197372	100.6153322	-0.219682848	0.176453409	-1.244990673	0.213135179	0.36852657
ENSG00000197375	156.7384247	0.264586773	0.146392752	1.807376181	0.070703645	0.160241062
ENSG00000197380	85.55160249	-0.241966424	0.216535935	-1.117442351	0.263805236	0.430338785
ENSG00000197381	326.0321938	-0.431606143	0.106492588	-4.052921906	5.06E-05	0.000310951
ENSG00000197386	1148.41682	-0.443945708	0.070477275	-6.299132664	2.99E-10	4.46E-09
ENSG00000197405	43.03597725	-1.655621187	0.294412232	-5.623479615	1.87E-08	2.21E-07
ENSG00000197410	76.21593426	0.703873129	0.20183512	3.487366968	0.000487802	0.002367708

ENSG00000197415	7.947936486	-3.055251069	0.829606659	-3.682770668	0.000230713	0.001215612
ENSG00000197417	19.03329372	-0.276317554	0.397941783	-0.69436678	0.487452221	0.64779673
ENSG00000197429	58.32614464	-0.155587328	0.226861027	-0.685826607	0.492822456	0.65209844
ENSG00000197442	121.2266389	2.013653334	0.185633173	10.8474865	2.05E-27	1.01E-25
ENSG00000197444	227.2790541	-0.208362533	0.124484189	-1.673807203	0.094168496	0.200179308
ENSG00000197448	309.6260188	0.524733842	0.109784187	4.779685083	1.76E-06	1.48E-05
ENSG00000197451	4955.918779	0.270859538	0.07914055	3.42251269	0.000620452	0.002936486
ENSG00000197457	75.17355798	-0.824341547	0.20747412	-3.973225897	7.09E-05	0.000421861
ENSG00000197467	26.99934806	-1.983100953	0.410698582	-4.828604332	1.37E-06	1.18E-05
ENSG00000197472	78.10384338	-0.302264695	0.207866005	-1.454132413	0.145909599	0.279312093
ENSG00000197479	8.294062066	-0.482998294	0.613140299	-0.787745146	0.430845793	0.597477385
ENSG00000197483	133.0156063	0.067522076	0.166705559	0.405037941	0.685449622	0.801641066
ENSG00000197496	119.9830461	-0.238043249	0.174165826	-1.366762095	0.171699887	0.314824216
ENSG00000197498	348.9561767	0.250751733	0.110173284	2.275975835	0.022847456	0.064276395
ENSG00000197530	104.7699001	-0.236100551	0.178157393	-1.325235778	0.185092962	0.332949269
ENSG00000197535	322.807228	0.189761316	0.110523284	1.716935195	0.085991013	0.186694863
ENSG00000197536	19.03044726	-0.112881532	0.396776192	-0.284496738	0.776029733	0.865699624
ENSG00000197548	97.12699708	-0.133251216	0.188896905	-0.705417682	0.480550344	0.640992373
ENSG00000197555	421.0914463	-0.122245781	0.101460956	-1.204855406	0.228259126	0.38696065
ENSG00000197558	175.3057388	0.661906667	0.141310719	4.684051357	2.81E-06	2.27E-05
ENSG00000197562	101.2533628	0.501514754	0.186270448	2.692401069	0.007093958	0.02409887
ENSG00000197563	167.4037512	-0.118587526	0.149599967	-0.792697542	0.427954063	0.595195121
ENSG00000197565	75.38600762	0.126741757	0.217803397	0.581908997	0.56062798	0.707146494
ENSG00000197566	64.85882526	0.041555896	0.234609156	0.177128195	0.859407696	0.919849124
ENSG00000197568	49.74792504	-0.554727249	0.270376985	-2.051680725	0.040200701	0.102189513
ENSG00000197579	423.390874	-0.235764313	0.101086974	-2.332291734	0.019685349	0.057049742
ENSG00000197580	6.349913707	-0.487870127	0.732351782	-0.666169099	0.505303022	0.662868093
ENSG00000197586	191.6698623	0.180041715	0.133497061	1.348656764	0.177447239	0.322834007
ENSG00000197594	43.35246644	0.666561691	0.290597509	2.293762574	0.02180414	0.061937868
ENSG00000197599	10.9102261	-0.324440084	0.523332437	-0.619950268	0.535290529	0.686182213
ENSG00000197601	283.8857243	-0.190827624	0.118640128	-1.608457679	0.107734978	0.222413047
ENSG00000197603	371.6978797	-0.518755399	0.105115209	-4.935112637	8.01E-07	7.26E-06
ENSG00000197619	29.77430255	0.673078312	0.334866767	2.009988384	0.044432418	0.11112039
ENSG00000197620	15.95513179	0.585534407	0.435710314	1.343861708	0.17899311	0.324901173
ENSG00000197622	442.0316551	0.009725729	0.095159032	0.102204998	0.91859396	0.955355281
ENSG00000197653	57.81585398	1.193576702	0.26043878	4.582945365	4.58E-06	3.54E-05
ENSG00000197694	3597.335621	-0.08240774	0.057985202	-1.421185707	0.155262779	0.292709201
ENSG00000197696	115.3199132	0.920999333	0.177587737	5.18616515	2.15E-07	2.15E-06
ENSG00000197702	68.5164626	-0.292348112	0.217654813	-1.343173198	0.179215898	0.325114685
ENSG00000197712	341.8993962	1.504979281	0.11997643	12.54395786	4.29E-36	2.90E-34
ENSG00000197713	191.745086	0.027685162	0.138195624	0.200333131	0.841220053	0.907713787
ENSG00000197714	401.5772979	-0.155661602	0.102319347	-1.521331065	0.128176778	0.253805921
ENSG00000197724	514.5710134	0.195381289	0.097074693	2.012690264	0.044147224	0.110600332
ENSG00000197728	118.834188	0.60877054	0.165359125	3.681505581	0.000231861	0.001220745
ENSG00000197744	18.30324583	0.029298973	0.398336401	0.073553342	0.941365798	0.96761052
ENSG00000197746	2300.38773	0.531809824	0.062512783	8.507217213	1.78E-17	5.10E-16
ENSG00000197756	7287.062314	-0.207407004	0.06362735	-3.259714659	0.001115244	0.004946582
ENSG00000197757	362.6133345	-0.324662465	0.102799343	-3.158215377	0.001587382	0.006702122
ENSG00000197763	99.18086221	0.002547131	0.181577358	0.014027801	0.988807801	0.994400285
ENSG00000197766	61.84742431	-0.597802682	0.224843639	-2.658748478	0.007843149	0.026294204

ENSG00000197771	671.7397938	0.064733133	0.081481398	0.794452903	0.426931817	0.594244077
ENSG00000197774	230.1207912	0.024214927	0.124889895	0.193890202	0.846261856	0.911124418
ENSG00000197779	91.94331312	-0.202417693	0.189780196	-1.066590179	0.286156937	0.455793911
ENSG00000197780	85.73841787	0.62736026	0.20214463	3.103521769	0.001912322	0.007872914
ENSG00000197782	46.22610511	0.074437222	0.260103696	0.286182871	0.774738062	0.865083113
ENSG00000197785	332.99781	0.292392165	0.111853515	2.614063268	0.008947247	0.029367384
ENSG00000197798	36.97749764	-0.042552411	0.306671033	-0.138755886	0.889643055	0.939610225
ENSG00000197808	25.67172512	1.001078703	0.363914944	2.75085901	0.005943922	0.020697638
ENSG00000197813	21.50442417	-0.143294691	0.384906183	-0.37228472	0.709680874	0.818195874
ENSG00000197816	13.55718152	-1.00832419	0.503040258	-2.004460228	0.045020783	0.11226823
ENSG00000197818	44.27350412	0.316557051	0.281263156	1.125483535	0.260384191	0.426544224
ENSG00000197822	118.5581869	-0.160422311	0.168177726	-0.953885599	0.340141554	0.511833787
ENSG00000197837	136.627238	-0.564511028	0.180638557	-3.125086014	0.001777531	0.007387271
ENSG00000197841	27.3271063	-0.093717443	0.328893323	-0.284947845	0.7756841	0.86552026
ENSG00000197852	12.91693533	-0.583591574	0.499986891	-1.16721375	0.24312406	0.40603539
ENSG00000197857	11.22484327	-2.802147668	0.677877343	-4.13370899	3.57E-05	0.00022709
ENSG00000197858	195.4114056	0.536654218	0.13768937	3.897571892	9.72E-05	0.000560277
ENSG00000197860	81.32150484	-0.126730526	0.204312386	-0.620278235	0.535074622	0.685971818
ENSG00000197863	11.95954606	-0.544684466	0.504975766	-1.078634863	0.280750534	0.449518669
ENSG00000197879	1289.620094	-0.325977658	0.070607816	-4.616736197	3.90E-06	3.06E-05
ENSG00000197885	77.84044213	0.020070598	0.205376086	0.097726069	0.922149815	0.95696206
ENSG00000197892	67.22159974	0.235908953	0.220164774	1.071510891	0.283939776	0.453633078
ENSG00000197894	281.5879558	0.251985593	0.111973512	2.250403588	0.024423337	0.06797595
ENSG00000197903	3013.214755	-0.170492485	0.069295573	-2.460366178	0.013879532	0.042559657
ENSG00000197905	239.9545457	0.403167639	0.120626573	3.342278813	0.000830936	0.003808206
ENSG00000197912	611.9136233	-0.017115079	0.100776677	-0.169831748	0.865142458	0.924195157
ENSG00000197930	1171.441191	0.353514259	0.074162945	4.766723604	1.87E-06	1.57E-05
ENSG00000197933	9.460638028	-1.736063168	0.624899486	-2.778147858	0.005466973	0.019285429
ENSG00000197937	24.18029394	-1.673356105	0.386286987	-4.33189872	1.48E-05	0.000102042
ENSG00000197948	78.8464637	0.39970817	0.200339736	1.995151726	0.046026334	0.114241729
ENSG00000197951	64.73847063	-0.051230368	0.226475406	-0.2262072	0.821040271	0.895083638
ENSG00000197958	3784.227855	0.236753496	0.129794639	1.824062206	0.068142668	0.155845012
ENSG00000197959	213.0914475	1.34441144	0.134244884	10.01461952	1.31E-23	5.37E-22
ENSG00000197961	966.0804567	0.131300128	0.078592471	1.670645123	0.094791794	0.201317943
ENSG00000197965	1594.902669	0.231441632	0.065442585	3.536560078	0.000405374	0.002006446
ENSG00000197969	709.7799995	-0.17388306	0.07904333	-2.199844808	0.027817908	0.075787073
ENSG00000197971	33.4528233	-7.376030141	1.205650676	-6.117883301	9.48E-10	1.32E-08
ENSG00000197976	451.6123348	0.267520968	0.094689268	2.825251201	0.004724356	0.017059905
ENSG00000197977	22.57974357	-0.243039814	0.362528085	-0.670402719	0.502601102	0.660644616
ENSG00000197978	28.68033272	-0.200726733	0.342715274	-0.585695322	0.558080278	0.705122829
ENSG00000197982	200.7012808	-0.078882673	0.129980717	-0.606879807	0.54393068	0.692773077
ENSG00000197989	147.8532195	0.139753818	0.152264597	0.917835271	0.358705114	0.529946438
ENSG00000198000	323.3121454	0.250195747	0.105574896	2.369841277	0.017795723	0.052505045
ENSG00000198001	112.1842297	0.175119458	0.18154573	0.964602461	0.334743967	0.506369642
ENSG00000198003	72.77031129	-1.074838766	0.229315076	-4.687170088	2.77E-06	2.24E-05
ENSG00000198015	530.5937637	-0.151174516	0.092810603	-1.628849622	0.103344857	0.215160102
ENSG00000198018	104.14092	-0.542546196	0.177122001	-3.063121421	0.002190412	0.008818281
ENSG00000198026	386.6858296	0.501290806	0.100313046	4.997264329	5.81E-07	5.41E-06
ENSG00000198034	6933.554283	0.126720157	0.063346253	2.000436502	0.04545315	0.113145561
ENSG00000198039	46.24866387	-0.64010081	0.267307631	-2.394622284	0.016637495	0.04969315

ENSG00000198040	308.3655167	0.130179641	0.108395814	1.200965577	0.229764554	0.388715247
ENSG00000198042	488.7621018	0.101046686	0.090944831	1.111076726	0.266535325	0.433380991
ENSG00000198046	121.7470691	0.485680248	0.160850689	3.019447734	0.00253236	0.009994959
ENSG00000198053	537.9328025	0.280105195	0.092115642	3.040799458	0.002359509	0.009394565
ENSG00000198055	538.6422023	0.116894785	0.088577014	1.319696613	0.186936331	0.335227518
ENSG00000198056	134.1536343	-0.476099561	0.161175897	-2.953912897	0.003137726	0.011999899
ENSG00000198060	185.9451676	-0.296954057	0.135620684	-2.189592685	0.02855379	0.077356815
ENSG00000198064	13.03400458	0.18382767	0.478675359	0.38403412	0.700953159	0.812392722
ENSG00000198081	89.14911083	-0.279934174	0.188088525	-1.488310752	0.136668961	0.266230605
ENSG00000198087	307.8350008	-0.202620099	0.110031194	-1.841478693	0.06555144	0.151174041
ENSG00000198089	194.5789285	-0.205886963	0.135472621	-1.519768069	0.128569277	0.254367977
ENSG00000198105	109.7097974	-0.376329617	0.168996715	-2.226845753	0.025957584	0.071565493
ENSG00000198113	14.90969249	-0.342067254	0.444881088	-0.768895921	0.441955099	0.607051742
ENSG00000198130	317.1760984	-0.100115882	0.109070833	-0.91789784	0.358672353	0.529946438
ENSG00000198133	13.53447492	-0.29478578	0.474215421	-0.621628415	0.534186232	0.685370327
ENSG00000198134	22.25613198	-1.825635295	0.414225678	-4.407344573	1.05E-05	7.46E-05
ENSG00000198142	402.2844712	0.146160398	0.104260706	1.401874248	0.160952802	0.300212987
ENSG00000198146	1272.90748	0.271698182	0.07060704	3.848032468	0.00011907	0.000675517
ENSG00000198157	549.2211	0.46183483	0.100954434	4.57468594	4.77E-06	3.66E-05
ENSG00000198160	389.3178243	-0.022449844	0.103892234	-0.216087796	0.828919312	0.89997137
ENSG00000198162	521.1727131	0.007756403	0.08942063	0.086740645	0.930877668	0.962535046
ENSG00000198169	169.1127798	0.401504976	0.139626224	2.875569958	0.004032988	0.014912181
ENSG00000198171	519.1063575	0.451929971	0.095843797	4.715276172	2.41E-06	1.98E-05
ENSG00000198176	703.7849032	0.308084601	0.080621799	3.821356046	0.00013272	0.000743644
ENSG00000198182	20.84197462	0.133405763	0.391853379	0.340448163	0.733519053	0.835693316
ENSG00000198189	87.29636062	-0.363232837	0.191011001	-1.901633073	0.057219141	0.135426668
ENSG00000198198	477.2915114	-0.159758416	0.099846769	-1.600035906	0.109590618	0.225351448
ENSG00000198205	48.37370232	0.531555162	0.259893229	2.045282843	0.040826981	0.103687688
ENSG00000198208	31.75747654	-0.521050103	0.315509662	-1.651455302	0.098645641	0.20749855
ENSG00000198218	63.17528329	0.223818306	0.224258986	0.998034952	0.318262411	0.48997802
ENSG00000198231	2100.013735	0.011550756	0.063086771	0.183093153	0.854724928	0.916518175
ENSG00000198242	539.8755131	0.602814477	0.092635917	6.507351516	7.65E-11	1.23E-09
ENSG00000198246	16.82304122	0.014079709	0.423315683	0.033260542	0.97346682	0.986330341
ENSG00000198252	385.5638761	0.244680524	0.104381136	2.344106748	0.019072712	0.055527683
ENSG00000198258	386.3656688	0.309549082	0.119248257	2.595837369	0.009436072	0.030728876
ENSG00000198265	2347.352088	0.117043555	0.065161341	1.796211565	0.072460876	0.163170556
ENSG00000198270	25.72587823	-0.784325806	0.355573011	-2.205808036	0.027397443	0.074786609
ENSG00000198276	233.577806	0.139550825	0.131524629	1.061024287	0.288678864	0.458770449
ENSG00000198301	284.077568	-0.090317411	0.111413243	-0.810652384	0.417565325	0.585558562
ENSG00000198315	485.8096222	-0.036415736	0.091972195	-0.395942886	0.692147163	0.806274442
ENSG00000198324	48.18981956	-0.265061553	0.280951424	-0.943442641	0.345454541	0.517169343
ENSG00000198331	37.31259759	-0.475080547	0.292169359	-1.626045072	0.103940068	0.215983155
ENSG00000198353	89.56940488	-1.743283727	0.205207531	-8.495222954	1.98E-17	5.62E-16
ENSG00000198355	325.5219978	0.042297808	0.12180698	0.347252745	0.728401449	0.832196434
ENSG00000198356	340.9123246	0.42841605	0.106530407	4.021537703	5.78E-05	0.000350149
ENSG00000198363	7631.282507	1.406918659	0.055418842	25.38700948	3.51E-142	1.70E-139
ENSG00000198369	1320.379871	-0.34626181	0.07398399	-4.680226229	2.87E-06	2.31E-05
ENSG00000198373	228.6308346	0.054567095	0.127888705	0.426676422	0.669615026	0.788968485
ENSG00000198380	631.0444896	-0.093340837	0.083049404	-1.123919408	0.261047217	0.427271747
ENSG00000198382	216.0983963	-0.151861597	0.131957602	-1.150836291	0.249799592	0.413789489

ENSG00000198393	176.0700502	-0.057104513	0.141466207	-0.403661861	0.686461389	0.802156487
ENSG00000198399	548.6894044	0.202253019	0.086596545	2.335578391	0.019513226	0.056609311
ENSG00000198408	904.2016419	-0.216085493	0.078002492	-2.770238336	0.005601529	0.019681009
ENSG00000198414	10.78027376	-3.480506441	0.784348501	-4.437448962	9.10E-06	6.59E-05
ENSG00000198417	13.58594508	0.695517743	0.493133637	1.41040418	0.158420372	0.296710795
ENSG00000198420	183.889948	0.315929315	0.136251303	2.318725086	0.020409947	0.058773787
ENSG00000198431	793.0113126	-0.187032678	0.080871132	-2.312724852	0.020737772	0.059533212
ENSG00000198435	338.5925771	-0.340924956	0.111649548	-3.053527404	0.002261681	0.00907562
ENSG00000198440	21.70371171	0.18451741	0.365334211	0.505064689	0.613513401	0.748612122
ENSG00000198455	77.0701151	0.063991928	0.205685386	0.311115579	0.755712758	0.852102835
ENSG00000198464	208.3312282	-0.077099609	0.127467581	-0.604856614	0.545274273	0.693980947
ENSG00000198466	97.9126811	-0.176396342	0.189601518	-0.930353008	0.35218834	0.523715899
ENSG00000198467	3256.828871	0.227228047	0.060378196	3.76341232	0.000167611	0.000912568
ENSG00000198468	63.59621344	-0.236510844	0.224370309	-1.054109362	0.291832851	0.462422566
ENSG00000198478	184.6645283	-0.717247067	0.138175771	-5.190830935	2.09E-07	2.10E-06
ENSG00000198482	41.78984616	0.27172319	0.29453725	0.9225427	0.356245568	0.527477758
ENSG00000198492	516.2718037	0.047093573	0.100130283	0.470322984	0.638124279	0.766295498
ENSG00000198496	135.2801437	-0.323117019	0.154694766	-2.088739185	0.036731207	0.095140248
ENSG00000198498	206.2163291	-0.206558274	0.137420266	-1.503113624	0.132809746	0.260993075
ENSG00000198517	96.96129098	0.299257133	0.200455583	1.492884999	0.135467292	0.264550126
ENSG00000198522	143.3903609	-0.100106479	0.157298489	-0.636410934	0.524508616	0.677870322
ENSG00000198538	48.59969598	-0.817541391	0.280436538	-2.915245628	0.003554087	0.013380933
ENSG00000198546	109.2740901	0.069088245	0.171728835	0.402310101	0.687455821	0.802917762
ENSG00000198551	33.87563489	0.33106277	0.298026081	1.110851669	0.266632202	0.433380991
ENSG00000198554	263.12829	-0.224783152	0.120093897	-1.871728359	0.061244194	0.143191943
ENSG00000198556	54.35459286	-0.936139858	0.248097507	-3.77327395	0.000161119	0.000882011
ENSG00000198561	182.3801406	-0.32143148	0.136885805	-2.348172476	0.018865783	0.055026506
ENSG00000198563	464.6164602	0.024281702	0.108482243	0.223831124	0.822888711	0.896348169
ENSG00000198576	558.3465679	3.743609697	0.132070444	28.34555246	9.50E-177	6.67E-174
ENSG00000198585	279.9282826	0.145169748	0.1117646	1.298888453	0.193982213	0.344789086
ENSG00000198586	508.2843385	-0.277071074	0.090174598	-3.072606717	0.002121979	0.008572748
ENSG00000198589	1480.181337	-0.206085087	0.064624907	-3.18894211	0.001427945	0.006122814
ENSG00000198590	105.4001514	-0.295497988	0.182631337	-1.618002653	0.105662026	0.218849083
ENSG00000198598	119.3429765	0.100646088	0.187263898	0.537455908	0.590952734	0.731900787
ENSG00000198604	562.8754531	0.34710713	0.092756586	3.742129206	0.000182468	0.000986575
ENSG00000198612	203.9878966	0.060768987	0.138853242	0.437649032	0.661640722	0.783714244
ENSG00000198618	34.23564163	0.472054012	0.309802977	1.523723295	0.127577848	0.252940865
ENSG00000198624	67.51185198	-0.514387501	0.21930789	-2.345503855	0.019001382	0.055364533
ENSG00000198625	1359.823666	0.125245132	0.069524972	1.801440956	0.071633406	0.161877671
ENSG00000198642	104.0043675	-0.311837828	0.1843671	-1.691396285	0.090761141	0.19461483
ENSG00000198646	1247.023435	0.13749186	0.071102783	1.933705751	0.053149316	0.127884284
ENSG00000198648	343.8914242	0.254391123	0.108069814	2.353951704	0.018575024	0.054359035
ENSG00000198663	609.3444777	0.305184889	0.088171911	3.461248447	0.000537676	0.002584784
ENSG00000198668	534.0711277	-0.337694672	0.088569968	-3.812744654	0.000137432	0.000766683
ENSG00000198673	112.7060619	1.14343689	0.174028746	6.570390903	5.02E-11	8.27E-10
ENSG00000198677	733.819254	0.276073314	0.08183963	3.373345094	0.000742608	0.003441576
ENSG00000198680	36.36143048	0.416016454	0.303679578	1.369919098	0.170712157	0.313791373
ENSG00000198682	69.80388731	-1.434886739	0.22975246	-6.245359642	4.23E-10	6.19E-09
ENSG00000198689	113.2170963	0.426513223	0.166694019	2.558659432	0.010507663	0.033591519
ENSG00000198690	249.4694852	0.002339128	0.118903374	0.019672514	0.984304617	0.992000554

ENSG00000198695	11169.88834	0.614292875	0.073520352	8.355412555	6.52E-17	1.80E-15
ENSG00000198700	1973.893675	-0.172880396	0.064478738	-2.681200041	0.007335865	0.02481264
ENSG00000198707	539.5751131	0.058060455	0.092317382	0.628922249	0.529399959	0.682058262
ENSG00000198712	203273.4616	-0.290610817	0.056041571	-5.185629358	2.15E-07	2.15E-06
ENSG00000198718	141.1674746	-0.780882947	0.158306492	-4.932728518	8.11E-07	7.34E-06
ENSG00000198720	358.4978333	0.280048747	0.103355355	2.709571733	0.006737014	0.023042325
ENSG00000198721	493.7030423	0.133423547	0.092132264	1.448173972	0.147568402	0.281757964
ENSG00000198722	461.8598203	-0.092255906	0.093367524	-0.988094162	0.323106535	0.494894504
ENSG00000198723	12.35993563	-0.33607623	0.503188078	-0.667893864	0.504201344	0.661979277
ENSG00000198727	122138.1511	0.076720496	0.056166635	1.365944327	0.171956438	0.315171201
ENSG00000198728	635.6572249	-0.043002165	0.084537299	-0.508676831	0.610978765	0.746977708
ENSG00000198729	28.21725958	2.231921179	0.377970437	5.905015211	3.53E-09	4.56E-08
ENSG00000198730	631.8810909	0.124746863	0.08348584	1.494227794	0.13511609	0.264011089
ENSG00000198736	109.1609364	0.300395299	0.169154675	1.775861648	0.075755724	0.168804037
ENSG00000198740	888.2064682	-0.173348284	0.076951641	-2.252691201	0.024278626	0.067653613
ENSG00000198742	425.8424466	0.012150469	0.098970463	0.122768643	0.902290306	0.947258155
ENSG00000198743	574.3919168	0.006818123	0.087087499	0.078290488	0.937596984	0.965856911
ENSG00000198744	251.6131737	-0.13910745	0.130734189	-1.064047983	0.287306963	0.457312246
ENSG00000198746	84.83769323	-0.037675295	0.205308626	-0.183505662	0.854401277	0.916445841
ENSG00000198752	1359.554679	0.082191325	0.066361075	1.238547227	0.215513222	0.371844427
ENSG00000198755	1650.307951	0.363596159	0.067819907	5.361201088	8.27E-08	8.81E-07
ENSG00000198756	162.2916562	-0.525245056	0.148827154	-3.529228648	0.000416773	0.002057066
ENSG00000198763	84071.71791	-0.261779407	0.06060339	-4.319550526	1.56E-05	0.000107289
ENSG00000198780	185.362489	-0.69242747	0.140546279	-4.926686618	8.36E-07	7.54E-06
ENSG00000198783	179.1020284	-0.036555962	0.140083131	-0.260959059	0.79412408	0.877406181
ENSG00000198785	21.02862753	1.31858873	0.407091666	3.239046238	0.001199301	0.005254705
ENSG00000198786	278977.6144	-0.4080417	0.060747989	-6.716958201	1.86E-11	3.18E-10
ENSG00000198791	409.293469	-0.044592651	0.099259182	-0.449254667	0.653247956	0.77746328
ENSG00000198792	341.5046315	-0.322525859	0.110776093	-2.911511419	0.003596848	0.013520182
ENSG00000198793	925.97782	-0.137937582	0.080835514	-1.706398275	0.087933951	0.189769276
ENSG00000198794	121.5810928	0.336385685	0.162044086	2.075889924	0.037904133	0.097513442
ENSG00000198799	249.3012621	-0.246902963	0.117760436	-2.096654632	0.036024159	0.093640149
ENSG00000198804	182555.3325	-0.146661529	0.064577713	-2.271085846	0.023141782	0.065001521
ENSG00000198805	445.1565997	0.319926185	0.106065252	3.016314757	0.002558675	0.010084654
ENSG00000198807	104.0141607	-3.238745422	0.245181123	-13.20960349	7.72E-40	5.99E-38
ENSG00000198814	24.43021902	-0.086867303	0.37655097	-0.230692016	0.817554076	0.892507031
ENSG00000198815	727.1514116	-0.046619747	0.081072695	-0.575036357	0.565266709	0.710947988
ENSG00000198816	208.2911255	-0.032606261	0.146001028	-0.223328985	0.82327947	0.896481141
ENSG00000198818	90.21618633	0.267410926	0.219266926	1.219567997	0.222628684	0.380873325
ENSG00000198824	622.3367848	0.208872286	0.090688223	2.30319086	0.021268107	0.060746868
ENSG00000198825	194.4489046	-0.369065738	0.138010414	-2.674187605	0.007491053	0.025288831
ENSG00000198826	335.3258576	-0.151806987	0.104203435	-1.456832856	0.145162524	0.278261186
ENSG00000198830	656.9425337	0.338820156	0.092642554	3.657284261	0.000254902	0.001329103
ENSG00000198832	248.1972878	0.643992584	0.119083559	5.407905098	6.38E-08	6.94E-07
ENSG00000198833	596.7294414	0.106642149	0.088679095	1.202562439	0.22914569	0.387901845
ENSG00000198836	966.3998383	0.037594284	0.081565035	0.460911758	0.644861915	0.771188092
ENSG00000198837	592.4217973	-0.04303201	0.085798563	-0.501546981	0.615986223	0.750390987
ENSG00000198839	52.04249509	0.089188089	0.24985174	0.356964051	0.721118718	0.826834923
ENSG00000198840	36439.2528	-0.138386333	0.075945494	-1.822179652	0.068427731	0.156267979
ENSG00000198841	31.55969517	0.334997367	0.31234201	1.072533812	0.283480336	0.453071825

ENSG00000198843	216.492737	0.52052495	0.130101784	4.000905552	6.31E-05	0.000379929
ENSG00000198846	52.75950218	0.06580372	0.248442485	0.264865005	0.791113458	0.875349719
ENSG00000198853	322.7283999	-0.129447635	0.113413919	-1.141373439	0.253714553	0.418654548
ENSG00000198855	16.13984474	0.031576513	0.443641493	0.071175742	0.943257887	0.968637121
ENSG00000198856	160.6168509	0.093350422	0.143667839	0.649765619	0.51584363	0.671096242
ENSG00000198858	122.8967924	0.155472312	0.175539801	0.885681259	0.37578931	0.545716162
ENSG00000198860	274.1317489	-0.369595991	0.12219136	-3.024730967	0.002488544	0.009849697
ENSG00000198862	275.9330425	-1.117575805	0.117105022	-9.543363583	1.38E-21	5.08E-20
ENSG00000198863	191.3544859	0.215723903	0.135077228	1.597041239	0.110256515	0.226395741
ENSG00000198868	171.8585198	-0.161337922	0.158538798	-1.017655764	0.308841576	0.480085296
ENSG00000198870	9.173841618	-0.411046471	0.567564164	-0.724229078	0.46892511	0.630937837
ENSG00000198873	57.36060337	0.255422554	0.23318025	1.095386741	0.27334724	0.441498067
ENSG00000198874	215.2058461	-0.217133686	0.141540907	-1.534070195	0.125012385	0.249438774
ENSG00000198876	310.1188909	-0.060797666	0.114029303	-0.533175814	0.593911882	0.73406777
ENSG00000198885	271.7042751	0.224414406	0.1144449	1.960894767	0.0498913	0.121649556
ENSG00000198886	291546.1275	-0.359772441	0.059918697	-6.004343565	1.92E-09	2.59E-08
ENSG00000198887	829.2781923	-0.166598782	0.08498246	-1.960390197	0.049950201	0.121750906
ENSG00000198888	258981.7606	-0.586962507	0.051925211	-11.3039985	1.25E-29	6.72E-28
ENSG00000198890	142.1777695	-0.027055478	0.15482274	-0.17475132	0.86127504	0.921145918
ENSG00000198892	33.84920234	-0.345126109	0.310855664	-1.110245524	0.266893241	0.43356648
ENSG00000198894	142.1250744	0.201636227	0.151558836	1.330415519	0.183381404	0.330675043
ENSG00000198898	404.1341308	0.567272877	0.102351363	5.542406658	2.98E-08	3.42E-07
ENSG00000198899	96612.45348	-0.370474834	0.062389233	-5.938121327	2.88E-09	3.77E-08
ENSG00000198900	2343.996776	0.020119077	0.061030739	0.329654819	0.741660796	0.842008184
ENSG00000198901	126.2695597	0.104567907	0.17065966	0.612727737	0.540056379	0.690091145
ENSG00000198909	141.6234385	-0.521461084	0.164644093	-3.167201893	0.001539134	0.006527844
ENSG00000198911	2771.588812	0.330736875	0.059724254	5.537731399	3.06E-08	3.51E-07
ENSG00000198912	198.8218514	0.113622651	0.141584667	0.802506751	0.422259885	0.589889605
ENSG00000198915	104.4061906	-0.148739603	0.17215584	-0.863982329	0.387597598	0.5563077
ENSG00000198918	38.51707139	0.689526964	0.287179354	2.401032508	0.016348884	0.048976919
ENSG00000198919	165.7776833	-1.772609034	0.158655474	-11.17269379	5.55E-29	2.85E-27
ENSG00000198920	287.8950462	0.093880424	0.112560856	0.834041488	0.404257605	0.57241129
ENSG00000198924	141.9778502	0.149791854	0.167474112	0.894417965	0.371098304	0.542154745
ENSG00000198925	190.7578865	0.274456111	0.137271316	1.999369702	0.045568368	0.113372097
ENSG00000198929	10.04310112	0.45405843	0.545945136	0.831692419	0.405582581	0.573801237
ENSG00000198931	262.4512946	-0.627615963	0.118582212	-5.292665323	1.21E-07	1.25E-06
ENSG00000198932	37.90528332	-0.434235377	0.290742892	-1.49353738	0.135296577	0.264290227
ENSG00000198933	313.4163275	-0.076118942	0.118717129	-0.6411791	0.521406327	0.675726051
ENSG00000198934	10.98140732	-0.060143005	0.585290659	-0.1027575	0.918155437	0.955355281
ENSG00000198937	115.2309231	0.703718309	0.171405172	4.105583874	4.03E-05	0.000253585
ENSG00000198938	119452.0989	-0.245421891	0.057940177	-4.235780822	2.28E-05	0.000151197
ENSG00000198939	9.019766747	-0.659377911	0.583021407	-1.130966896	0.258069025	0.423739987
ENSG00000198945	114.6790706	0.16825668	0.164925781	1.020196347	0.30763535	0.478661249
ENSG00000198947	884.9843508	-4.496077001	0.129792102	-34.64060553	6.19E-263	6.69E-260
ENSG00000198948	159.712827	-0.251960904	0.143262883	-1.758731212	0.078623173	0.174142921
ENSG00000198951	261.069432	-0.30602131	0.128739718	-2.377054368	0.017451516	0.051728517
ENSG00000198952	725.0907049	0.322900071	0.081743628	3.950155866	7.81E-05	0.000460762
ENSG00000198954	166.3655124	-0.220044692	0.140792116	-1.56290493	0.118074958	0.238647329
ENSG00000198960	66.77414727	0.171831737	0.22183419	0.774595371	0.438578794	0.604051016
ENSG00000198961	589.1800866	0.12312781	0.087043433	1.414555989	0.15719872	0.295249422

ENSG00000198964	85.03635711	-0.104981172	0.189746486	-0.553270706	0.580078057	0.723117407
ENSG00000199377	112.3509841	0.513945094	0.199538746	2.575665649	0.010004734	0.032297953
ENSG00000199568	20.07390202	0.447222289	0.391034913	1.1436889	0.252752679	0.417437186
ENSG00000199719	9.136448328	-0.808931087	0.582216741	-1.389398535	0.164711595	0.305802773
ENSG00000199753	44.65306667	0.365210798	0.258227711	1.414297466	0.157274581	0.295273461
ENSG00000199879	89.40415519	0.620852406	0.194322446	3.19495981	0.001398503	0.006016776
ENSG00000199883	131.2536736	-0.272785537	0.181329335	-1.504365177	0.132487366	0.260541792
ENSG00000200087	240.6251644	-0.086203659	0.317386557	-0.271604633	0.785926037	0.871892516
ENSG00000200091	15.7720745	-0.231884159	0.435315297	-0.532680935	0.594254466	0.734327425
ENSG00000200169	154.2460162	0.73284183	0.154796312	4.734233147	2.20E-06	1.82E-05
ENSG00000200312	67.56715178	-0.377721935	0.246996533	-1.529260073	0.126199983	0.251166218
ENSG00000200488	57.85074358	-0.236000212	0.237297195	-0.994534351	0.31996279	0.49159168
ENSG00000200492	24.1722258	0.233167564	0.372800364	0.625448862	0.531676501	0.683361681
ENSG00000200794	10.72252053	-0.848956006	0.574726516	-1.477147795	0.139636024	0.270509893
ENSG00000200795	44.7197502	-0.199281059	0.267937908	-0.743758358	0.457022677	0.62063008
ENSG00000200879	11.00965808	0.652506071	0.579055119	1.126846217	0.259807506	0.425974404
ENSG00000200959	14.79419928	0.177798182	0.447705071	0.397132382	0.691269842	0.805563918
ENSG00000200972	34.44059254	0.827276	0.308329204	2.683093231	0.007294465	0.024708308
ENSG00000200997	34.99597914	-0.948243565	0.319266347	-2.970070519	0.002977314	0.011461312
ENSG00000201098	75.20689172	-0.923287661	0.61696687	-1.496494717	0.134524784	0.263178099
ENSG00000201302	20.46947073	-0.705623028	0.40653943	-1.73568165	0.08262014	0.181055399
ENSG00000201592	129.1278316	-0.488801072	0.174845149	-2.795622722	0.005179983	0.018449093
ENSG00000201772	25.29206436	-0.089049676	0.681404495	-0.130685483	0.896024117	0.943582785
ENSG00000201782	9.85693022	-0.438242954	0.566950663	-0.772982522	0.439532724	0.604795228
ENSG00000201793	16.3153824	-0.960947116	0.477132077	-2.014006524	0.044008849	0.110430921
ENSG00000201801	37.13133285	0.543699737	0.289202867	1.879994286	0.060108857	0.141030418
ENSG00000201998	27.55965006	-0.439220766	0.345112506	-1.27268864	0.20312855	0.356089698
ENSG00000202058	8.265050698	-0.3631392	0.593323027	-0.612042991	0.540509314	0.690408328
ENSG00000202233	14.33051267	0.297260533	0.4812361	0.617702067	0.536771749	0.687520084
ENSG00000202360	8.107021748	-1.173728863	0.643078924	-1.825170782	0.067975261	0.155563457
ENSG00000202512	36.39643874	-0.165619894	0.299322309	-0.553316239	0.580046883	0.723117407
ENSG00000202515	83.282225	-0.360656113	0.22641445	-1.592902365	0.111182096	0.227896527
ENSG00000202538	26.81041732	0.190330117	0.375459168	0.506926274	0.61220655	0.747774297
ENSG00000203279	13.78591755	-0.047059521	0.476731368	-0.098712868	0.921366251	0.956727767
ENSG00000203392	9.294685639	-0.192342697	0.557490573	-0.345015156	0.730082976	0.833168866
ENSG00000203485	190.1733387	0.612486563	0.151561364	4.041178751	5.32E-05	0.000324669
ENSG00000203499	35.04788641	0.758011972	0.294090992	2.577474299	0.009952528	0.032160838
ENSG00000203644	30.67327055	-0.257385073	0.332988924	-0.772953858	0.439549689	0.604795228
ENSG00000203645	13.70287417	-1.295002935	0.493002421	-2.626767903	0.008620011	0.028490657
ENSG00000203647	6.907462451	-1.622038491	0.735835419	-2.204349571	0.027499769	0.07502219
ENSG00000203666	263.4844812	-1.046818579	0.125458466	-8.343945339	7.19E-17	1.96E-15
ENSG00000203667	434.4764726	0.053838686	0.096448901	0.558209436	0.576701378	0.720722799
ENSG00000203668	180.4362171	0.075858024	0.146501951	0.517795316	0.604601087	0.741943439
ENSG00000203705	82.96793199	-0.017450535	0.198003644	-0.088132392	0.929771451	0.961886845
ENSG00000203709	46.98664768	-0.028621423	0.277528267	-0.103129759	0.917859987	0.955355281
ENSG00000203737	9.883293763	-0.392497522	0.550260955	-0.713293426	0.475664205	0.636590614
ENSG00000203739	47.58446163	-0.014539307	0.264153809	-0.055041065	0.956105748	0.976402577
ENSG00000203760	72.14578309	-0.004196413	0.214817221	-0.019534808	0.98441447	0.992000554
ENSG00000203761	10.06234584	0.148745204	0.564684389	0.263412991	0.792232279	0.876120833
ENSG00000203778	13.26717127	1.333292316	0.50028696	2.665055104	0.007697571	0.025886468

ENSG00000203791	83.52605401	-0.228913855	0.206050717	-1.11095879	0.266586088	0.433380991
ENSG00000203799	10.42882151	-0.642107335	0.558831939	-1.149016888	0.250549017	0.414688588
ENSG00000203804	11.49217359	-0.05745452	0.509423589	-0.112783392	0.910202286	0.951381168
ENSG00000203808	13.58470739	-2.361481544	0.564593434	-4.182623109	2.88E-05	0.000186968
ENSG00000203814	3901.332145	-0.180448755	0.06268477	-2.878669833	0.003993562	0.014785863
ENSG00000203865	39.07248465	-0.457554993	0.289332947	-1.581413379	0.11378355	0.231839783
ENSG00000203867	498.1692851	0.452528409	0.096924647	4.668868268	3.03E-06	2.43E-05
ENSG00000203872	24.85781626	-0.045875556	0.369619816	-0.124115521	0.90122381	0.946716337
ENSG00000203875	63.58296324	0.198397589	0.223610341	0.887246931	0.37494597	0.54488579
ENSG00000203877	53.57887601	0.911406453	0.251639037	3.621880231	0.00029247	0.001504067
ENSG00000203879	752.6052744	0.139409529	0.082209412	1.695785489	0.089926512	0.193179035
ENSG00000203880	114.9518685	-0.044970366	0.164630479	-0.273159421	0.784730672	0.871115267
ENSG00000203883	260.8835014	0.786989589	0.126440434	6.224192417	4.84E-10	7.02E-09
ENSG00000203930	3328.164959	-0.257314452	0.081913386	-3.141299197	0.001682001	0.007052856
ENSG00000203950	356.9743974	0.001010215	0.115268925	0.008763985	0.993007441	0.996412282
ENSG00000203965	29.29532452	-0.167123304	0.322524302	-0.518172749	0.604337746	0.741751513
ENSG00000203993	53.66184169	0.129662201	0.249032275	0.520664243	0.602600688	0.740701073
ENSG00000203995	124.3303432	0.059934432	0.160948935	0.372381665	0.709608703	0.818195874
ENSG00000204019	16.42423136	0.300419603	0.424766046	0.707258986	0.479405549	0.640073162
ENSG00000204052	42.64059468	0.896993269	0.294908779	3.04159568	0.002353278	0.009380389
ENSG00000204054	380.2225688	-0.106892699	0.102290641	-1.04499002	0.296027582	0.466490851
ENSG00000204060	50.51997055	-0.571850121	0.255189262	-2.240886303	0.025033441	0.06937162
ENSG00000204070	96.2121162	0.054730016	0.199391181	0.274485642	0.783711436	0.870948935
ENSG00000204084	143.4578431	-0.055388713	0.157216041	-0.352309551	0.724606126	0.829479444
ENSG00000204104	416.056059	-0.227555975	0.097808884	-2.326536858	0.019989929	0.057736276
ENSG00000204116	90.53637325	0.335915539	0.190756229	1.760967605	0.078243886	0.173549956
ENSG00000204120	1871.858292	0.125487631	0.063220045	1.984934206	0.047151792	0.116424894
ENSG00000204128	8.561206707	-2.312926297	0.734157719	-3.150448789	0.001630198	0.006866386
ENSG00000204130	178.7846254	-0.25587306	0.144422322	-1.771700218	0.076444334	0.170176474
ENSG00000204131	87.3872833	-0.958119418	0.201442441	-4.756293736	1.97E-06	1.64E-05
ENSG00000204138	802.4732447	-0.388379042	0.085015997	-4.568305439	4.92E-06	3.77E-05
ENSG00000204147	51.71024066	-0.260271413	0.251874087	-1.033339383	0.301445103	0.471851946
ENSG00000204160	299.3279403	0.61368764	0.116686172	5.259300473	1.45E-07	1.49E-06
ENSG00000204172	18.19336254	-0.027589398	0.401812825	-0.068662314	0.945258417	0.969665557
ENSG00000204178	110.3362512	-0.420822444	0.171473046	-2.45416089	0.014121378	0.043197518
ENSG00000204209	221.9122722	-0.101679934	0.128645972	-0.79038568	0.429302563	0.596126245
ENSG00000204217	624.5910625	-0.324986777	0.090530934	-3.589787077	0.000330948	0.001675858
ENSG00000204219	15.81275108	-0.354565877	0.446739954	-0.793673979	0.427385252	0.594580632
ENSG00000204220	482.7335378	-0.045375491	0.092937179	-0.488238301	0.625381058	0.756912608
ENSG00000204227	208.0852301	-0.032150362	0.131307239	-0.244848356	0.80657385	0.885690308
ENSG00000204228	44.75898468	-1.329602047	0.286340043	-4.643437333	3.43E-06	2.73E-05
ENSG00000204231	186.848363	0.401301405	0.146158098	2.74566657	0.00603881	0.020955328
ENSG00000204237	131.4819491	0.006777756	0.163358082	0.041490178	0.966905123	0.982787985
ENSG00000204248	15.6262836	-0.028400369	0.450742765	-0.06300793	0.949760189	0.972113187
ENSG00000204252	5.017611145	-5.60678661	1.331563883	-4.210677898	2.55E-05	0.000167515
ENSG00000204253	20.37877078	0.747125363	0.397766121	1.878303166	0.060339704	0.141477519
ENSG00000204256	2073.029739	-0.164474979	0.061992354	-2.653149423	0.007974456	0.026696053
ENSG00000204257	7.38916989	-1.317764969	0.682442291	-1.930954437	0.053488687	0.128436853
ENSG00000204262	3469.277549	-2.061405763	0.058384474	-35.30743048	4.52E-273	6.34E-270
ENSG00000204267	114.3718411	0.219014595	0.18073463	1.211802053	0.225588162	0.383961822

ENSG00000204271	77.9830638	-0.363724663	0.20750923	-1.752811971	0.079634288	0.175828802
ENSG00000204272	372.9500579	0.212245491	0.102055742	2.079701613	0.03755291	0.096772285
ENSG00000204301	11.32774922	-1.230553274	0.538633016	-2.284585679	0.022337133	0.06303128
ENSG00000204304	561.6302773	0.085289234	0.088376954	0.965061934	0.334513791	0.506275374
ENSG00000204305	30.20758424	0.14708776	0.319758445	0.459996481	0.645518746	0.771726296
ENSG00000204308	190.3773311	-0.061756694	0.14513187	-0.425521248	0.670456736	0.789629057
ENSG00000204310	584.2409686	-0.371742942	0.087438504	-4.251478763	2.12E-05	0.000141781
ENSG00000204311	13.9146102	-0.540013247	0.463896384	-1.164081605	0.244390943	0.407618092
ENSG00000204314	123.2327201	0.131331845	0.165878715	0.791734163	0.428515698	0.595563472
ENSG00000204315	44.88793014	-0.237452089	0.259796551	-0.91399246	0.360720808	0.532029104
ENSG00000204316	26.53687193	0.127778534	0.3527803	0.362204279	0.717199382	0.823876127
ENSG00000204344	47.49592744	0.100026877	0.259969329	0.384764147	0.700412165	0.811974059
ENSG00000204348	69.902546	0.824948425	0.220507103	3.741142193	0.000183186	0.000989697
ENSG00000204351	513.5127085	0.410114991	0.092436675	4.436712929	9.13E-06	6.60E-05
ENSG00000204356	348.9973972	0.410972639	0.104418183	3.935834039	8.29E-05	0.000487694
ENSG00000204366	73.07833702	-0.245539093	0.209276163	-1.17327788	0.24068439	0.403207349
ENSG00000204370	41.31568553	-0.018965727	0.26987909	-0.070274902	0.943974858	0.968874385
ENSG00000204371	763.1196751	0.310958717	0.078688283	3.951779162	7.76E-05	0.000458032
ENSG00000204386	798.9223642	0.195335806	0.081967917	2.383076376	0.017168632	0.051051603
ENSG00000204387	201.3637141	0.103076961	0.131262721	0.785272167	0.432294011	0.598623223
ENSG00000204388	320.9636266	0.112956604	0.109285968	1.03358744	0.301329074	0.471828057
ENSG00000204392	275.7077321	0.146064765	0.139619416	1.046163703	0.295485458	0.465897882
ENSG00000204394	1349.535345	0.361608162	0.06601445	5.477712247	4.31E-08	4.82E-07
ENSG00000204396	10.08310909	1.264986124	0.595601957	2.123878388	0.033680314	0.088519515
ENSG00000204406	447.5814005	-0.215611355	0.097743072	-2.205899112	0.027391064	0.074783728
ENSG00000204410	58.42103707	0.296916362	0.234144499	1.268090273	0.204765715	0.358155148
ENSG00000204427	12.5031523	-0.049098019	0.500716887	-0.098055449	0.921888264	0.956832011
ENSG00000204435	15.03980349	-0.636239282	0.448212222	-1.419504536	0.155751975	0.293237903
ENSG00000204438	133.4252909	0.040604564	0.158165267	0.256722382	0.797393092	0.879045586
ENSG00000204439	86.82738477	-0.508218857	0.19618314	-2.590532785	0.009582749	0.031137839
ENSG00000204444	10.06155444	-0.780418528	0.572709467	-1.36267789	0.172984049	0.316650919
ENSG00000204463	2093.280472	0.26381216	0.070063099	3.765351012	0.000166315	0.00090657
ENSG00000204469	5435.269181	0.091665061	0.075920369	1.207384298	0.22728418	0.385773426
ENSG00000204498	118.7199723	0.6952909	0.169268526	4.10762069	4.00E-05	0.000251697
ENSG00000204514	107.4601069	-0.186951411	0.181276494	-1.031305312	0.302397668	0.473079412
ENSG00000204516	494.2715552	-0.106682533	0.097075135	-1.098968679	0.271781728	0.439474839
ENSG00000204519	113.5886083	-0.120846284	0.175928454	-0.68690585	0.49214206	0.651504997
ENSG00000204520	236.3507721	0.49812948	0.126865911	3.926424978	8.62E-05	0.000503995
ENSG00000204524	137.0319587	0.319014034	0.153978119	2.071814069	0.038282784	0.0982925
ENSG00000204525	1437.115173	0.881429387	0.076871294	11.46630094	1.95E-30	1.08E-28
ENSG00000204536	229.9309452	0.163585658	0.129625361	1.261988057	0.206953084	0.3608377
ENSG00000204560	455.3048145	0.027104956	0.096044749	0.282211739	0.777781158	0.86696492
ENSG00000204564	206.5903836	0.489183627	0.136227332	3.590935976	0.000329493	0.001669088
ENSG00000204568	226.6408356	0.092710834	0.125218238	0.740394016	0.459060949	0.62242959
ENSG00000204569	628.9176175	-0.256791682	0.082927847	-3.096567569	0.001957752	0.008030158
ENSG00000204574	1299.026486	-0.048445681	0.079394091	-0.61019252	0.541734284	0.69122913
ENSG00000204576	242.2676645	-0.454378619	0.124015512	-3.663885363	0.000248418	0.001297705
ENSG00000204580	241.292136	-0.238100903	0.122944823	-1.936648469	0.052788329	0.127211813
ENSG00000204584	12.5415799	-0.942475424	0.511852134	-1.841304081	0.06557701	0.151208185
ENSG00000204590	532.111271	0.087632479	0.102181558	0.857615406	0.391104865	0.559455198

ENSG00000204592	227.8925859	-0.274313953	0.124727006	-2.199314822	0.027855544	0.075860185
ENSG00000204599	41.796551	0.144853342	0.27040229	0.535695692	0.592168881	0.732558017
ENSG00000204604	14.1919177	0.03944707	0.489861639	0.080526963	0.935818153	0.964731953
ENSG00000204611	75.6558739	-0.164675301	0.200792021	-0.820128709	0.412142738	0.580465208
ENSG00000204619	195.7066034	0.280901135	0.146473483	1.917761018	0.055141321	0.131782491
ENSG00000204623	92.76783195	-0.361360399	0.200370162	-1.803464123	0.071315355	0.161367073
ENSG00000204628	12615.51588	0.193067006	0.130338259	1.481276548	0.138532895	0.268929184
ENSG00000204634	99.62727115	-0.280548912	0.181510035	-1.545638575	0.122191879	0.24544964
ENSG00000204650	71.9179454	-0.656583485	0.211437989	-3.10532411	0.001900706	0.007834912
ENSG00000204673	920.1188797	0.557667956	0.081105525	6.875831924	6.16E-12	1.11E-10
ENSG00000204681	506.6544646	0.379183924	0.102001847	3.717422143	0.000201266	0.001076202
ENSG00000204682	166.9913779	-0.874498951	0.147884821	-5.913378711	3.35E-09	4.35E-08
ENSG00000204685	34.57672527	0.341928111	0.320971072	1.06529261	0.286743536	0.456572937
ENSG00000204706	6.904214733	-1.621260263	0.734843324	-2.206266574	0.02736534	0.074730083
ENSG00000204713	611.165802	0.065105639	0.086780189	0.750236188	0.453112467	0.617396281
ENSG00000204745	10.3817789	0.238387294	0.546496903	0.43620978	0.662684533	0.784604829
ENSG00000204758	10.20180846	-0.473120445	0.564711314	-0.837809396	0.402137761	0.570588801
ENSG00000204764	150.5884785	-0.463013381	0.147000583	-3.149738394	0.001634167	0.006876916
ENSG00000204815	24.0664236	-0.583139422	0.363622268	-1.603695573	0.108781172	0.223988438
ENSG00000204839	144.157521	0.26763225	0.159208912	1.68101299	0.092760391	0.198025109
ENSG00000204842	2181.358102	0.053406259	0.061113441	0.873887292	0.382179636	0.551631458
ENSG00000204843	848.3646447	0.262448653	0.078215	3.355477249	0.000792282	0.003644133
ENSG00000204851	7.634034676	-6.214211535	1.287131322	-4.827954558	1.38E-06	1.19E-05
ENSG00000204852	156.8700694	-0.27462469	0.144355346	-1.902421327	0.057116095	0.135260585
ENSG00000204856	205.4705257	0.208878253	0.130102992	1.605483849	0.108387365	0.223374019
ENSG00000204859	74.13679246	-0.450438524	0.207552902	-2.170234772	0.029989065	0.080435267
ENSG00000204860	17.59826694	0.542785965	0.431672014	1.25740365	0.208607508	0.363066493
ENSG00000204882	52.91205858	0.630785306	0.269496129	2.340609895	0.019252272	0.055967852
ENSG00000204899	171.1487106	0.237696223	0.16222797	1.465198779	0.142866693	0.275023768
ENSG00000204920	57.28748835	-0.020827109	0.231682867	-0.089894905	0.92837073	0.96093314
ENSG00000204922	152.0939429	-0.042296303	0.150661602	-0.280737112	0.778912042	0.867881133
ENSG00000204923	15.94059015	-0.858069929	0.442745343	-1.938066527	0.052615107	0.126925022
ENSG00000204929	22.25830932	-1.476374022	0.392726476	-3.759293334	0.000170394	0.000926286
ENSG00000204934	47.76759703	0.182362184	0.27090072	0.673169799	0.500839272	0.659105233
ENSG00000204946	109.2629714	0.348935646	0.176786426	1.97376945	0.048407964	0.118982795
ENSG00000204947	34.43253242	-0.685507396	0.299605553	-2.28803301	0.022135599	0.062613523
ENSG00000204954	57.47345906	0.288528749	0.233850701	1.233816052	0.217271457	0.374066939
ENSG00000204977	153.4525919	-0.091296571	0.14738132	-0.619458224	0.535614535	0.686413409
ENSG00000204991	143.4611824	0.205368718	0.155082305	1.324256295	0.18541794	0.333320426
ENSG00000205002	125.0887046	-3.251103623	0.222421115	-14.61688394	2.19E-48	2.25E-46
ENSG00000205041	15.80101123	-0.681686612	0.435924172	-1.563773369	0.117870801	0.238303272
ENSG00000205060	435.1305199	0.257724276	0.094516672	2.726759963	0.006395955	0.022025981
ENSG00000205084	82.20681227	-0.072917526	0.198859768	-0.366678123	0.713859127	0.821528813
ENSG00000205085	25.85188428	-0.568927651	0.346069081	-1.643971343	0.100182122	0.210007203
ENSG00000205116	12.74832161	0.99877056	0.501609373	1.991132171	0.046466358	0.115137226
ENSG00000205133	149.6596661	0.374388109	0.153282059	2.442478333	0.014586805	0.044427765
ENSG00000205138	46.31904643	0.050565162	0.267037597	0.189355966	0.849813834	0.914037896
ENSG00000205155	10.03435689	-0.296415773	0.571485477	-0.518675951	0.603986735	0.741561198
ENSG00000205189	393.7743169	-0.301455501	0.098540026	-3.05921882	0.00221915	0.008926804
ENSG00000205208	164.3623227	-0.580995436	0.152258262	-3.815854902	0.000135712	0.000758595

ENSG00000205213	401.7233848	0.765425489	0.109445862	6.993644881	2.68E-12	5.09E-11
ENSG00000205250	481.5437483	0.406992391	0.092724971	4.389242594	1.14E-05	8.06E-05
ENSG00000205268	135.5849218	-0.480477653	0.160073626	-3.001604104	0.002685612	0.01050379
ENSG00000205269	62.70638224	0.619304965	0.224939443	2.753207516	0.005901448	0.020575238
ENSG00000205302	605.6425906	0.102474262	0.090619629	1.130817496	0.258131914	0.423744186
ENSG00000205309	12.3930586	-0.354560691	0.502426398	-0.705696779	0.480376724	0.6408825
ENSG00000205323	11.13711265	-0.927988657	0.563486063	-1.646870646	0.099584637	0.209066715
ENSG00000205334	10.94370668	-0.708450025	0.555021246	-1.27643767	0.201800847	0.354248501
ENSG00000205336	22.58544918	1.535148527	0.38589006	3.978201787	6.94E-05	0.000414888
ENSG00000205339	2108.464983	-0.31939674	0.063782805	-5.007568119	5.51E-07	5.18E-06
ENSG00000205352	171.7448815	0.044803495	0.139300359	0.321632303	0.747731269	0.846500736
ENSG00000205356	106.2377484	-0.10403802	0.17636407	-0.589904846	0.555254445	0.702355623
ENSG00000205363	40.39910952	-1.144732376	0.291172881	-3.931452586	8.44E-05	0.000496046
ENSG00000205423	44.3968119	-0.471100592	0.274817292	-1.714231986	0.086486129	0.187566875
ENSG00000205464	22.30991348	-1.210050618	0.385346478	-3.140162651	0.001688541	0.007078165
ENSG00000205476	461.4020227	-0.147940783	0.094854197	-1.559665118	0.118839039	0.239979424
ENSG00000205517	130.8508782	-2.350031976	0.191955721	-12.24257324	1.84E-34	1.17E-32
ENSG00000205531	745.3567109	-0.055443905	0.082957585	-0.6683404	0.50391633	0.661666918
ENSG00000205542	17.59323249	-2.389664448	0.513682843	-4.652023095	3.29E-06	2.62E-05
ENSG00000205544	16.80422326	-0.47440755	0.451385526	-1.051003016	0.293257205	0.46389459
ENSG00000205559	22.33447967	-0.200803092	0.381163214	-0.526816558	0.598320983	0.737311593
ENSG00000205581	1551.509584	0.028663232	0.07973258	0.359492094	0.719226991	0.82519996
ENSG00000205592	16.64861009	-1.712820982	0.466784661	-3.66940289	0.000243118	0.001272856
ENSG00000205593	13.83827117	-1.236429863	0.490900745	-2.518696245	0.011779022	0.037065396
ENSG00000205611	124.6132008	-0.944894906	0.19427475	-4.863704141	1.15E-06	1.01E-05
ENSG00000205629	165.307924	0.255461204	0.144990176	1.761920785	0.078082682	0.173274477
ENSG00000205643	42.79733244	0.179470139	0.302194595	0.593889309	0.55258615	0.69986274
ENSG00000205659	72.92478918	-0.594404729	0.216089	-2.750740341	0.005946076	0.020700006
ENSG00000205683	7.538649287	-2.894929721	0.827162545	-3.499831731	0.000465552	0.002271486
ENSG00000205702	10.60462736	-0.547662777	0.550984988	-0.993970414	0.320237271	0.491948048
ENSG00000205707	73.87399669	0.32329034	0.21341889	1.514815953	0.129819019	0.256407166
ENSG00000205726	776.739323	-0.108501262	0.082159094	-1.320623884	0.186626805	0.334808012
ENSG00000205730	292.6189811	0.144988137	0.11422638	1.26930519	0.204332236	0.357575049
ENSG00000205740	14.68111359	0.868976107	0.469929704	1.849161906	0.064434437	0.149087555
ENSG00000205746	15.5178565	0.163648457	0.481695655	0.339734135	0.734056753	0.836073559
ENSG00000205758	88.25068991	-0.057142157	0.189319008	-0.301830002	0.762781658	0.856873889
ENSG00000205765	160.1802206	-0.003713464	0.149938081	-0.024766653	0.98024109	0.989966683
ENSG00000205795	20.99211837	-2.128533705	0.442410352	-4.811220386	1.50E-06	1.28E-05
ENSG00000205808	40.71275649	-0.17498766	0.272392634	-0.642409662	0.520607228	0.674960491
ENSG00000205885	59.15707433	-0.214243911	0.229365532	-0.934071956	0.350266777	0.522258509
ENSG00000205903	231.9183718	0.341745955	0.12614497	2.709152455	0.006745534	0.023060236
ENSG00000205913	26.36686836	-0.253245317	0.355993573	-0.711376093	0.47685121	0.63753861
ENSG00000205937	1459.230885	0.277372687	0.087541549	3.168469012	0.001532441	0.006503383
ENSG00000205940	10.49987115	0.523797839	0.535715797	0.977753209	0.328196398	0.500616224
ENSG00000205978	92.53500338	-1.100694685	0.210725998	-5.223345446	1.76E-07	1.78E-06
ENSG00000205981	55.80715405	0.05839041	0.241672133	0.241610023	0.809082355	0.887211541
ENSG00000206052	20.60326621	-3.99447044	0.641565586	-6.226129532	4.78E-10	6.95E-09
ENSG00000206053	960.3205412	-0.050308338	0.07915438	-0.63557239	0.52505517	0.678389438
ENSG00000206077	21.46208704	1.36282555	0.385488414	3.53532169	0.000407279	0.002014555
ENSG00000206172	11.51515934	-1.503438062	0.542807412	-2.769744902	0.005610021	0.019700992

ENSG00000206190	226.6511118	1.370574134	0.130255356	10.52220944	6.83E-26	3.20E-24
ENSG00000206195	40.0570513	-0.029018783	0.282956163	-0.102555754	0.918315561	0.955355281
ENSG00000206262	76.51779368	-0.271327682	0.208719392	-1.299963936	0.19361333	0.344438506
ENSG00000206341	46.96892468	1.693602528	0.280497757	6.03784696	1.56E-09	2.13E-08
ENSG00000206417	16.60243595	0.096654788	0.462020769	0.209200095	0.834292039	0.902804119
ENSG00000206418	321.2724803	-0.105110025	0.106510061	-0.986855364	0.323713549	0.495500078
ENSG00000206503	33.08053841	1.589966042	0.332721781	4.778665336	1.76E-06	1.48E-05
ENSG00000206527	313.6073635	-0.055247135	0.112765918	-0.489927595	0.624185136	0.755965861
ENSG00000206530	52.74774999	-0.481321914	0.241472202	-1.99328084	0.046230703	0.11461764
ENSG00000206538	18.25883901	-4.64950252	0.803056381	-5.789758516	7.05E-09	8.86E-08
ENSG00000206557	289.6715751	-3.318326474	0.156043356	-21.26541347	2.37E-100	5.85E-98
ENSG00000206560	1034.80757	1.343195806	0.075866119	17.7048178	3.85E-70	6.21E-68
ENSG00000206561	13.62622621	-0.217398111	0.465043695	-0.467478893	0.64015729	0.767653189
ENSG00000206562	201.9398632	0.160242071	0.131433474	1.219187675	0.222772969	0.380970525
ENSG00000206573	222.8832165	-1.047782051	0.128877742	-8.130046608	4.29E-16	1.12E-14
ENSG00000206603	29.64458383	-0.256777313	0.348811325	-0.736149587	0.461639668	0.624677335
ENSG00000206634	542.8355209	-0.606644817	0.244405612	-2.482123108	0.013060215	0.040516085
ENSG00000206754	11.47301175	0.250495438	0.946601763	0.264626	0.791297589	0.875431774
ENSG00000206828	17.75482059	0.88408434	0.415894582	2.125741418	0.033524796	0.08820431
ENSG00000207119	11.23393426	0.523048567	0.528862819	0.989006126	0.322660144	0.494426426
ENSG00000207175	37.1961561	0.123412662	0.283421213	0.435439046	0.663243774	0.784755231
ENSG00000207181	30.80817842	0.680109546	0.339420483	2.003737485	0.045098189	0.112421341
ENSG00000207205	116.4602067	0.646994135	0.16772032	3.857577523	0.000114516	0.000651787
ENSG00000207217	9.020348852	-1.833475417	0.675521394	-2.714163361	0.006644339	0.022748003
ENSG00000207313	9.715537018	-4.07715458	0.950476357	-4.28959074	1.79E-05	0.000121412
ENSG00000207344	105.5484726	-0.947567272	0.193279652	-4.902571287	9.46E-07	8.43E-06
ENSG00000207445	221.2595962	-0.273578849	0.252501486	-1.083474211	0.278597992	0.447406653
ENSG00000207475	13.82818163	-2.612340062	0.580561398	-4.499679228	6.81E-06	5.06E-05
ENSG00000207697	13.10165097	-0.620701494	0.482368546	-1.28677854	0.198171489	0.349844778
ENSG00000208028	13.29450058	0.22751078	0.479175217	0.474796634	0.634931932	0.764126872
ENSG00000209082	679.8834715	-1.001516795	0.10357746	-9.669254215	4.07E-22	1.53E-20
ENSG00000209482	20.56771706	0.823888371	0.394776022	2.086976732	0.036890238	0.095449838
ENSG00000210049	38.15693383	0.276822292	0.313268477	0.883658308	0.376880693	0.546505362
ENSG00000210082	16976.15724	-0.304584953	0.122744153	-2.481462003	0.013084466	0.040573398
ENSG00000210100	31.38749683	-0.297331469	0.339120339	-0.876772739	0.380610089	0.55021405
ENSG00000210107	49.83558942	-0.012538954	0.24998385	-0.050159056	0.959995639	0.978814678
ENSG00000210112	188.710184	-0.425837361	0.141292605	-3.0138687	0.002579394	0.010160642
ENSG00000210117	11.0863863	0.48075865	0.52064489	0.923390701	0.355803636	0.527227053
ENSG00000210127	29.48366378	0.048136257	0.32271803	0.149158872	0.881428272	0.934729197
ENSG00000210151	28.81429823	0.29643913	0.346851993	0.854655979	0.392741627	0.560996709
ENSG00000210154	258.4606118	-0.14739127	0.127789243	-1.153393409	0.248748946	0.412925601
ENSG00000210164	10.05780526	-1.417834684	0.585116136	-2.423167977	0.015385811	0.046528413
ENSG00000210176	15.23105363	0.009958824	0.43818489	0.022727447	0.981867682	0.990825814
ENSG00000210194	27.61880103	-0.240259847	0.347236717	-0.691919475	0.488987899	0.648858211
ENSG00000210195	93.79907263	-0.303166333	0.214798933	-1.411395899	0.158127911	0.296339531
ENSG00000210196	147.3007101	-0.259852918	0.152774501	-1.700891951	0.088963278	0.191549229
ENSG00000211445	8.6027232	-2.638750721	0.751481232	-3.511399366	0.000445754	0.002188574
ENSG00000211448	33.96540092	-1.664226072	0.326379249	-5.099056011	3.41E-07	3.31E-06
ENSG00000211450	150.6373595	0.424220781	0.181501774	2.337281734	0.01942454	0.056398617
ENSG00000211454	9.167466223	-0.210810138	0.582124349	-0.362139358	0.717247893	0.823876127

ENSG00000211455	102.63623	-0.24294002	0.179310827	-1.354854157	0.175464028	0.320013399
ENSG00000211456	464.0962249	-0.026713199	0.098771156	-0.270455462	0.786809877	0.87232189
ENSG00000211459	8267.412323	-0.121013379	0.078801331	-1.535676835	0.124617659	0.248869878
ENSG00000211460	206.440699	-0.08312112	0.128909957	-0.644799842	0.519056904	0.673862508
ENSG00000211584	77.64242667	0.037470377	0.202330038	0.185194338	0.85307661	0.915794202
ENSG00000212122	10.19762821	-0.848521073	0.547912069	-1.54864461	0.121467176	0.244238394
ENSG00000212123	16.51062733	1.080175728	0.453446559	2.382145608	0.01721209	0.051126714
ENSG00000212127	30.68409812	-0.592961434	0.316438074	-1.873862479	0.060949382	0.142648127
ENSG00000212182	17.53124798	-0.055699271	0.462790738	-0.120355199	0.90420178	0.948496931
ENSG00000212195	53.74466311	0.812634743	0.242973251	3.344544058	0.000824179	0.003779709
ENSG00000212232	101.4128878	0.32835728	0.18459244	1.778823011	0.075268783	0.16793997
ENSG00000212283	6.778476325	-0.961404887	0.767827407	-1.252110667	0.210529541	0.365654348
ENSG00000212402	7.097135036	-0.659027587	0.68048884	-0.968462007	0.332813685	0.505081442
ENSG00000212464	45.52333101	-0.332422549	0.262884149	-1.264521085	0.206043058	0.359535554
ENSG00000212607	32.59557833	-0.294171488	0.31001948	-0.948880657	0.342681313	0.514590639
ENSG00000212694	19.57604992	-0.088961373	0.397632949	-0.223727368	0.822969449	0.896348169
ENSG00000212719	210.453435	0.453018763	0.128141231	3.535308331	0.0004073	0.002014555
ENSG00000212747	80.81232531	-0.531616933	0.207725186	-2.559231952	0.010490372	0.033574449
ENSG00000212802	31.78542614	0.127510018	0.319085865	0.399610362	0.689443523	0.804503503
ENSG00000212864	146.8514787	0.666455371	0.169960887	3.921227885	8.81E-05	0.000513922
ENSG00000212907	133204.3304	-0.268527068	0.069579268	-3.859297134	0.000113714	0.000647743
ENSG00000212993	31.21160328	7.639159528	1.209787303	6.314464957	2.71E-10	4.05E-09
ENSG00000213015	507.3431949	0.142592266	0.097004403	1.469956636	0.141573499	0.273170733
ENSG00000213020	49.27804067	0.131910647	0.255320377	0.516647549	0.605402217	0.742455469
ENSG00000213023	7.26230446	-1.133009003	0.679148419	-1.668278939	0.095260367	0.202125198
ENSG00000213024	1030.446817	0.347986289	0.072091547	4.827005405	1.39E-06	1.19E-05
ENSG00000213057	14.55057604	0.340878104	0.475480357	0.716913115	0.473427709	0.634685916
ENSG00000213058	22.6054823	0.649184874	0.366979456	1.768995138	0.076894686	0.171043492
ENSG00000213062	11.17916694	-0.316389223	0.53069964	-0.596173803	0.551059124	0.698621617
ENSG00000213064	513.1608537	0.099580849	0.094938964	1.048893358	0.294227203	0.464487471
ENSG00000213066	161.3835788	-0.316871225	0.146167527	-2.167863354	0.030169085	0.080890463
ENSG00000213079	722.2126602	-0.327219061	0.081184509	-4.030560311	5.56E-05	0.00033793
ENSG00000213096	74.3609472	-0.648697532	0.210118173	-3.08729856	0.002019846	0.008243108
ENSG00000213160	182.1206785	0.013218805	0.134735112	0.098109576	0.921845283	0.956832011
ENSG00000213169	23.01875092	0.271205167	0.417288323	0.649922732	0.515742134	0.671096242
ENSG00000213178	14.60482972	0.244768684	0.466053896	0.525193945	0.599448374	0.737988721
ENSG00000213190	375.0998226	-0.125557086	0.277020212	-0.453241605	0.650374776	0.775087119
ENSG00000213197	7.396751421	0.088628213	0.638587784	0.13878783	0.889617811	0.939610225
ENSG00000213199	78.5186324	0.570637794	0.208221194	2.740536563	0.006133896	0.021243302
ENSG00000213204	9.285792225	-0.072136284	0.569801764	-0.126598914	0.899257857	0.945499634
ENSG00000213235	9.314410172	0.006657263	0.584609104	0.011387546	0.990914249	0.995236126
ENSG00000213246	58.07585132	-0.111662225	0.254420005	-0.43888933	0.660741729	0.783076162
ENSG00000213281	202.8115429	-0.047677405	0.13926315	-0.342354777	0.732083917	0.834847437
ENSG00000213300	13.28617354	0.175895653	0.47658025	0.369078771	0.712069012	0.820132623
ENSG00000213337	51.80426239	0.130024566	0.246426997	0.527639291	0.597749717	0.736869725
ENSG00000213339	122.870852	0.292633059	0.171916918	1.702177203	0.088722155	0.191147256
ENSG00000213341	126.6723431	0.269603455	0.176608658	1.52655854	0.126870823	0.252037117
ENSG00000213347	37.84564736	-0.416672743	0.295908425	-1.40811382	0.159097369	0.297661261
ENSG00000213366	218.3662641	-0.22405197	0.132852216	-1.686475216	0.091704305	0.196187994
ENSG00000213380	165.8362289	-0.225236311	0.141703556	-1.589489475	0.111949928	0.228969226

ENSG00000213390	180.9609265	-0.274963227	0.140061685	-1.963158062	0.049627811	0.121154303
ENSG00000213397	10.03464411	-0.043118323	0.562907617	-0.076599289	0.938942325	0.966262309
ENSG00000213398	30.39049966	0.200964387	0.317314547	0.63332863	0.526519061	0.679655693
ENSG00000213420	26.50891042	-0.473881508	0.344874127	-1.374070915	0.169419668	0.311987162
ENSG00000213442	40.78851726	0.013646	0.277936126	0.049097613	0.960841506	0.979316546
ENSG00000213445	298.1876754	-0.493589268	0.126759542	-3.893902267	9.86E-05	0.000567428
ENSG00000213453	16.05170863	0.04991927	0.442415047	0.112833571	0.910162502	0.951381168
ENSG00000213462	22.1098528	-1.057439005	0.381060288	-2.774991356	0.005520318	0.019444308
ENSG00000213463	216.0630664	-0.047382919	0.12452281	-0.380515977	0.703562439	0.814282078
ENSG00000213465	358.9612708	0.196737937	0.110004722	1.788449919	0.073703449	0.165226994
ENSG00000213468	62.90394309	-0.823948767	0.231612727	-3.557441676	0.000374484	0.00187469
ENSG00000213516	252.2175102	-0.047736418	0.123468947	-0.386626919	0.699032426	0.8110439
ENSG00000213523	140.9796012	0.055842653	0.157079029	0.355506738	0.722209998	0.827735087
ENSG00000213533	14.02926762	0.456414587	0.462101063	0.987694302	0.323302386	0.495086516
ENSG00000213551	297.847677	-0.012430835	0.11957494	-0.103958532	0.917202257	0.955230368
ENSG00000213553	70.73346103	0.104677727	0.209986495	0.498497426	0.618133483	0.75188641
ENSG00000213563	172.9276485	1.140302232	0.151273492	7.53801752	4.77E-14	1.08E-12
ENSG00000213585	1900.781783	0.083757222	0.064676757	1.295012694	0.195315851	0.346501863
ENSG00000213588	161.1366034	-0.012258961	0.145045538	-0.084518012	0.932644582	0.963270344
ENSG00000213593	244.2030855	-0.357116196	0.120304511	-2.968435623	0.002993198	0.011512993
ENSG00000213598	19.93164334	0.260044618	0.381944508	0.680843978	0.495970219	0.654672813
ENSG00000213613	20.45590553	-0.974103541	0.412743119	-2.360072155	0.018271381	0.053660482
ENSG00000213614	276.3025962	0.339965861	0.116914111	2.907825735	0.003639511	0.01364768
ENSG00000213619	305.195274	0.387732641	0.109373123	3.545045002	0.000392546	0.001952402
ENSG00000213625	23.52891889	1.787633037	0.387605002	4.611996825	3.99E-06	3.11E-05
ENSG00000213638	18.57802107	-0.175389991	0.399794971	-0.438699842	0.660879042	0.783076162
ENSG00000213639	841.6568389	-0.120781847	0.076097135	-1.587206241	0.112465942	0.229860614
ENSG00000213659	34.3696922	-0.812158844	0.306381063	-2.650812804	0.008029833	0.026843186
ENSG00000213672	532.3236632	0.590229103	0.090368759	6.531340123	6.52E-11	1.06E-09
ENSG00000213676	309.3953455	0.294146528	0.108374946	2.714156176	0.006644483	0.022748003
ENSG00000213693	10.3706586	0.252376706	0.537846455	0.469235604	0.638901239	0.76673607
ENSG00000213694	13.39358525	-2.248150911	0.557814856	-4.030281533	5.57E-05	0.000338185
ENSG00000213699	390.3144732	0.11393918	0.104449429	1.090854975	0.275336702	0.443721173
ENSG00000213719	1090.545702	-0.365252094	0.070400843	-5.188177864	2.12E-07	2.13E-06
ENSG00000213722	96.58297014	-1.044217042	0.186785601	-5.590457904	2.26E-08	2.65E-07
ENSG00000213740	9.029344714	0.306251733	0.582438124	0.525809903	0.599020293	0.737672211
ENSG00000213741	6685.839436	0.368875914	0.056107159	6.574489249	4.88E-11	8.08E-10
ENSG00000213742	61.57107762	-0.555134311	0.224845016	-2.468964266	0.013550475	0.0417237
ENSG00000213753	97.82473378	0.1537853	0.177842328	0.864728334	0.387187914	0.555890088
ENSG00000213760	7.412047749	-0.577428457	0.642389783	-0.898875531	0.368718963	0.540082927
ENSG00000213762	158.3035707	0.109485975	0.14783126	0.740614503	0.458927212	0.62242959
ENSG00000213781	5.593301062	-5.764512785	1.315895363	-4.38067718	1.18E-05	8.35E-05
ENSG00000213782	13.03047377	0.663672285	0.485275893	1.367618493	0.171431525	0.314504514
ENSG00000213793	31.24172802	0.396060485	0.314244707	1.260356903	0.207540641	0.361523238
ENSG00000213842	16.49372088	0.217482551	0.466128971	0.466571624	0.640806393	0.768195995
ENSG00000213853	508.1908608	0.395317907	0.089686817	4.407759349	1.04E-05	7.45E-05
ENSG00000213859	27.70508379	-1.26516511	0.35141285	-3.600224377	0.000317943	0.001617581
ENSG00000213866	22.73335809	-0.85846404	0.3683604	-2.330500344	0.019779722	0.057240575
ENSG00000213888	25.74308137	-0.431122661	0.348333809	-1.237671021	0.215838069	0.372146477
ENSG00000213889	73.06232188	-0.965596325	0.211750067	-4.560075662	5.11E-06	3.91E-05

ENSG00000213903	55.69849723	0.086666051	0.238336568	0.363628847	0.716135183	0.823334922
ENSG00000213904	47.07622763	-0.258126318	0.253684034	-1.017511089	0.30891036	0.48011328
ENSG00000213906	13.98952001	-0.106141797	0.511395974	-0.207553055	0.835577963	0.903847501
ENSG00000213918	56.88027459	-1.089817147	0.245673503	-4.436038616	9.16E-06	6.62E-05
ENSG00000213923	285.8668557	0.200455528	0.110905707	1.807441049	0.070693539	0.160241062
ENSG00000213928	28.53649992	1.347311139	0.359439897	3.748362806	0.000177993	0.000964608
ENSG00000213930	26.59378109	-0.106070411	0.372784103	-0.284535768	0.775999827	0.865699624
ENSG00000213942	21.53659043	0.176013237	0.37751743	0.466238702	0.64104465	0.768258186
ENSG00000213949	17.30116232	-0.229141618	0.413378241	-0.554314657	0.579363521	0.722635356
ENSG00000213963	98.30416465	-0.817400349	0.192224564	-4.252319966	2.12E-05	0.000141316
ENSG00000213965	163.3363438	-0.097137263	0.143203312	-0.678317158	0.497570622	0.655663652
ENSG00000213977	22.19969259	-0.054069672	0.381739369	-0.141640281	0.887364148	0.93824263
ENSG00000213983	255.7410674	-1.013425032	0.127035773	-7.977477579	1.49E-15	3.77E-14
ENSG00000213995	148.9184039	0.174485856	0.149927754	1.163799567	0.244505248	0.407760324
ENSG00000214013	128.5573047	-0.010881845	0.160096099	-0.067970706	0.945808954	0.969848951
ENSG00000214021	662.4236025	0.16544238	0.101496438	1.630031394	0.103094862	0.214735102
ENSG00000214022	872.3262332	0.305871239	0.080916932	3.780064701	0.000156788	0.000862332
ENSG00000214026	62.55060684	0.693669334	0.242073767	2.865528727	0.004163135	0.015332868
ENSG00000214029	103.6451432	-0.001617505	0.179445928	-0.009013886	0.992808057	0.996284349
ENSG00000214046	115.4546145	-0.004752393	0.169182141	-0.028090392	0.977590057	0.988629033
ENSG00000214050	35.12010019	-0.300378492	0.301028956	-0.997839195	0.318357341	0.48997802
ENSG00000214063	162.8830382	-0.711328928	0.151917022	-4.68235172	2.84E-06	2.29E-05
ENSG00000214078	147.7424992	-0.123612995	0.155712901	-0.793851979	0.427281608	0.59449532
ENSG00000214087	192.1067852	-0.068638066	0.140822087	-0.487409803	0.625967948	0.757361909
ENSG00000214106	59.37817208	0.93479496	0.237508109	3.935844399	8.29E-05	0.000487694
ENSG00000214113	162.218749	0.164870215	0.144816716	1.13847503	0.25492218	0.420193835
ENSG00000214114	62.00596804	-0.362556823	0.23331217	-1.553955899	0.120194936	0.242208986
ENSG00000214160	563.5912811	0.416503862	0.086015952	4.842169981	1.28E-06	1.11E-05
ENSG00000214182	121.8686595	2.613113642	0.196485928	13.29924067	2.34E-40	1.86E-38
ENSG00000214188	28.47324965	0.675970071	0.348962055	1.937087601	0.052734636	0.12712604
ENSG00000214193	169.8167481	-0.062501436	0.147503455	-0.423728627	0.671763735	0.790770568
ENSG00000214194	57.64610623	0.503478892	0.244893726	2.05590768	0.039791416	0.101442834
ENSG00000214199	20.05359687	-0.120079453	0.3892974	-0.308451722	0.75773863	0.853838805
ENSG00000214212	7.531786196	-0.802254367	0.631737462	-1.269917356	0.20411407	0.357326813
ENSG00000214226	45.73582445	0.126612212	0.261648147	0.483902575	0.628455013	0.759031607
ENSG00000214248	10.22367316	0.062866521	0.554191698	0.113438223	0.909683138	0.951381168
ENSG00000214253	223.6430342	0.381878733	0.127330458	2.999115371	0.002707648	0.01056509
ENSG00000214279	23.17775491	-1.526129533	0.429079653	-3.556751108	0.00037547	0.001878284
ENSG00000214293	29.5672816	0.157663959	0.3393652	0.464584936	0.642228722	0.768968949
ENSG00000214309	23.3687747	-0.16002384	0.358239337	-0.446695334	0.655095051	0.778784714
ENSG00000214357	60.29492445	-0.272599574	0.237947954	-1.145626888	0.251949567	0.416515896
ENSG00000214367	15.71951272	-0.619071966	0.470205851	-1.31659775	0.187973493	0.336611362
ENSG00000214389	17.59867851	0.682700029	0.433565486	1.574618023	0.11534464	0.234266221
ENSG00000214391	12.6996642	-0.392177525	0.482288582	-0.813159465	0.416126636	0.583987529
ENSG00000214413	146.7042703	0.014953343	0.149012816	0.100349377	0.920066958	0.956213418
ENSG00000214425	21.72770123	0.128232403	0.387310345	0.331084372	0.740580756	0.841457403
ENSG00000214435	10.04025835	-1.301986879	0.579041711	-2.248520017	0.02454305	0.068228028
ENSG00000214456	21.14445891	-0.346743976	0.39007378	-0.888918954	0.374046638	0.544085443
ENSG00000214485	13.76412953	0.221806675	0.477631715	0.464388498	0.642369429	0.769058499
ENSG00000214517	307.4107404	0.497310717	0.10804816	4.602676426	4.17E-06	3.24E-05

ENSG00000214530	34.49048941	0.15501618	0.309041668	0.501602847	0.615946916	0.750390987
ENSG00000214575	110.8437001	0.286307031	0.168997011	1.694154411	0.090235945	0.193709482
ENSG00000214595	120.03755	0.201830911	0.167929464	1.20187909	0.229410377	0.388303117
ENSG00000214654	21.84332988	-0.492618283	0.377984716	-1.303275669	0.192480665	0.342988189
ENSG00000214655	408.5617613	0.267830065	0.100272326	2.671026741	0.007561962	0.025503694
ENSG00000214706	408.1931924	0.516441503	0.100345623	5.146627093	2.65E-07	2.62E-06
ENSG00000214717	350.8076468	0.138348292	0.111812428	1.237324814	0.215966519	0.372276561
ENSG00000214719	15.52990258	-0.195822847	0.456179894	-0.42926672	0.667729133	0.787594299
ENSG00000214753	390.901245	-0.135033806	0.109172608	-1.236883576	0.216130308	0.372421791
ENSG00000214756	32.12447399	0.467277903	0.31312055	1.492325892	0.135613732	0.264799291
ENSG00000214826	53.28260261	0.005848936	0.24107367	0.024262029	0.980643601	0.990159622
ENSG00000214900	20.61715728	0.370494273	0.394519771	0.939101917	0.34767842	0.51950205
ENSG00000214922	34.42621443	0.20985043	0.291270039	0.72046693	0.471237554	0.633139543
ENSG00000214941	91.25531143	-0.319986462	0.18734971	-1.707963483	0.087643118	0.189287036
ENSG00000214944	28.64582614	0.875711051	0.338753042	2.585101655	0.009735029	0.031552366
ENSG00000214954	16.24352945	0.343935195	0.430415837	0.799076534	0.424246035	0.591443436
ENSG00000214960	52.53377745	-0.711211147	0.245186554	-2.900693926	0.003723374	0.013888006
ENSG00000214973	13.17730554	0.30693911	0.472385158	0.649764507	0.515844349	0.671096242
ENSG00000215006	18.44156195	-0.095609226	0.402340303	-0.237632733	0.81216596	0.889524775
ENSG00000215012	196.556183	0.250369623	0.13977472	1.791236804	0.073255303	0.164435711
ENSG00000215014	14.56932684	0.035008836	0.472263851	0.074129824	0.940907084	0.967262249
ENSG00000215021	2069.501386	0.123965127	0.061603633	2.012302207	0.04418809	0.110682971
ENSG00000215030	242.9953275	0.292970645	0.147563508	1.98538683	0.04710145	0.116341935
ENSG00000215039	13.06620869	0.836191965	0.486570442	1.718542461	0.085697716	0.186201984
ENSG00000215041	188.9075712	0.309254641	0.135102207	2.28904212	0.022076905	0.062538262
ENSG00000215067	16.04552006	-0.35788923	0.436158156	-0.820549209	0.411903089	0.58028309
ENSG00000215068	57.80495594	-0.533523068	0.23915371	-2.230879328	0.025689123	0.070922782
ENSG00000215105	9.129130493	-0.913624942	0.610501448	-1.496515602	0.134519345	0.263178099
ENSG00000215114	228.4009355	-0.368578051	0.133184824	-2.767417787	0.005650229	0.019817422
ENSG00000215193	279.4915143	0.514316093	0.115134276	4.467097988	7.93E-06	5.83E-05
ENSG00000215251	208.8737081	0.084467752	0.128991551	0.654831662	0.512576143	0.668538262
ENSG00000215252	108.6059959	-0.248196043	0.182964098	-1.356528661	0.174931022	0.319373027
ENSG00000215256	50.95673247	-0.395784177	0.245255078	-1.613765471	0.106578307	0.220486815
ENSG00000215301	2622.39623	-0.023811371	0.058241226	-0.408840486	0.682656727	0.799239793
ENSG00000215305	196.1943422	0.049046627	0.129447588	0.37889178	0.704768228	0.814939027
ENSG00000215374	12.88632853	-0.65216807	0.485562066	-1.343119895	0.179233155	0.325114685
ENSG00000215375	53.52806747	-0.247600774	0.250749178	-0.987444011	0.323425018	0.495216383
ENSG00000215386	197.1259545	-3.541291859	0.20524391	-17.25406546	1.04E-66	1.63E-64
ENSG00000215388	14.92862226	-0.048479827	0.44571471	-0.108768739	0.913385919	0.953417734
ENSG00000215417	72.68477541	-0.120230992	0.212939552	-0.564624986	0.572328867	0.717377715
ENSG00000215421	432.0378131	0.050937149	0.109085646	0.466946393	0.640538232	0.768044434
ENSG00000215424	70.57131488	-0.151406038	0.21556423	-0.702370878	0.482447902	0.643034982
ENSG00000215712	82.32274338	-0.214174373	0.19732419	-1.085393395	0.277747456	0.446500174
ENSG00000215717	114.4695777	0.147520096	0.167263376	0.881962924	0.377796857	0.547235502
ENSG00000215788	20.54817871	-0.67832586	0.385400387	-1.760054954	0.078398489	0.17381054
ENSG00000215808	178.5387642	-2.469142281	0.169464949	-14.57022411	4.34E-48	4.42E-46
ENSG00000215883	56.54623937	0.20386671	0.233844024	0.871806371	0.383314018	0.552575821
ENSG00000215908	10.15418832	0.586066175	0.564324759	1.038526427	0.299025038	0.469468087
ENSG00000215912	12.43041245	-0.920774686	0.500855027	-1.838405601	0.06600266	0.151940244
ENSG00000215915	32.59180141	-0.145553026	0.309407127	-0.470425578	0.638050994	0.766295498

ENSG00000216809	11.54634431	-0.861662637	0.532231239	-1.618962913	0.105455243	0.218501439
ENSG00000216895	12.67735878	-0.10639837	0.48387958	-0.219886052	0.8259599	0.898324082
ENSG00000216937	34.73088018	-0.016246598	0.294676135	-0.055133743	0.956031914	0.97639816
ENSG00000217128	404.9967961	-0.051467132	0.100476111	-0.512232525	0.608488282	0.74493942
ENSG00000217130	9.433230337	0.642022282	0.586339474	1.094966842	0.273531163	0.441744338
ENSG00000217512	42.86703533	-1.152155419	0.280113101	-4.113179338	3.90E-05	0.000245931
ENSG00000217527	9.15159332	0.700839374	0.580331952	1.207652571	0.227180929	0.385691383
ENSG00000217555	43.41205382	-0.757480964	0.279618537	-2.708979787	0.006749045	0.023066627
ENSG00000217930	36.23727563	0.456076764	0.297359551	1.533755222	0.125089883	0.24952252
ENSG00000218208	9.296064657	0.392930559	0.572703173	0.6860981	0.492651249	0.652056166
ENSG00000218283	40.08647882	-0.25614697	0.284763478	-0.899507801	0.368382242	0.539702269
ENSG00000218336	266.5688334	-0.435934558	0.121512451	-3.587571105	0.000333773	0.001688943
ENSG00000218426	150.8332263	0.157695831	0.14583607	1.081322551	0.27955366	0.448275144
ENSG00000218510	16.37444279	-0.181318823	0.432189631	-0.419535339	0.674824934	0.793044913
ENSG00000218596	11.45348162	-1.00655987	0.558987156	-1.800685149	0.07175252	0.162016982
ENSG00000218739	400.3538106	0.076884804	0.100284504	0.766666842	0.443279623	0.608199732
ENSG00000218891	202.5735319	0.05555491	0.132005566	0.420852786	0.673862584	0.792311686
ENSG00000219200	25.56321382	0.614214536	0.342522144	1.793211172	0.072939164	0.163962765
ENSG00000219470	8.577823417	0.45402974	0.583645419	0.777920507	0.436615878	0.602647464
ENSG00000219481	157.483539	-0.090054828	0.146895088	-0.613055406	0.539839705	0.689877032
ENSG00000219545	15.14516568	-0.407756992	0.474567146	-0.859218755	0.390219838	0.558530473
ENSG00000219607	9.329177499	0.100938208	0.570831099	0.17682675	0.859644477	0.920032454
ENSG00000219626	55.99332512	0.127510162	0.241289147	0.528453782	0.597184418	0.736571392
ENSG00000219642	10.9193438	-0.102356105	0.517754669	-0.197692288	0.843285824	0.909104833
ENSG00000219665	21.93727921	-0.358265214	0.381055866	-0.940190784	0.347119708	0.518998461
ENSG00000219747	8.242371452	-0.081064833	0.603344514	-0.134359112	0.893118612	0.941935367
ENSG00000219928	8.988727305	0.195619743	0.576524386	0.339308705	0.734377186	0.836302905
ENSG00000220008	26.44275872	-0.230439961	0.339525012	-0.678712768	0.497319875	0.655493567
ENSG00000220161	9.776672233	-0.694311068	0.559509507	-1.240928099	0.214632312	0.370701814
ENSG00000220205	222.786422	0.151277182	0.12234438	1.236486568	0.216277755	0.372584453
ENSG00000220305	8.419304524	-1.110532717	0.612883734	-1.811979428	0.069989375	0.159007076
ENSG00000220494	22.63211882	-1.136675456	0.402348927	-2.825098761	0.004726604	0.017063637
ENSG00000220515	9.72890836	1.224270746	0.571273831	2.143054137	0.032108752	0.08500496
ENSG00000220848	25.60602067	-0.199347002	0.356973083	-0.558437069	0.576545966	0.720601927
ENSG00000221164	32.05663406	-0.617945803	0.323728291	-1.908840901	0.056282619	0.133773595
ENSG00000221184	11.18420488	-0.142167209	0.548899704	-0.259003983	0.795632167	0.878221362
ENSG00000221303	29.26670273	-1.080710361	0.38178279	-2.830694281	0.004644709	0.016830556
ENSG00000221400	13.90216639	0.462398834	0.465481065	0.993378396	0.320525586	0.492175656
ENSG00000221817	25.52883527	-0.662840848	0.352536966	-1.880202396	0.060080499	0.14101099
ENSG00000221821	12.43054439	-0.082076185	0.489756368	-0.167585743	0.866909188	0.925339922
ENSG00000221823	353.0235089	-0.128722791	0.110078474	-1.169372963	0.242253398	0.404918311
ENSG00000221829	215.3312723	-0.330271896	0.129985166	-2.540842987	0.011058557	0.035128799
ENSG00000221838	94.61492255	0.547053155	0.181573311	3.01285002	0.002588068	0.010186212
ENSG00000221869	278.4721759	0.569709556	0.121890739	4.673936372	2.95E-06	2.37E-05
ENSG00000221883	9.137949553	0.816970601	0.582112524	1.40345821	0.160480245	0.299530429
ENSG00000221886	13.14082849	-0.762838841	0.493310598	-1.546366213	0.122016149	0.245166764
ENSG00000221887	12.07466414	0.949893769	0.515599566	1.842309094	0.06542995	0.150918639
ENSG00000221890	50.57967021	-2.844991996	0.319854474	-8.894644994	5.86E-19	1.88E-17
ENSG00000221909	45.39011575	-0.086071199	0.274579832	-0.313465117	0.753927318	0.851251269
ENSG00000221914	462.9984532	0.14093912	0.09541818	1.47706778	0.139657469	0.27051413

ENSG00000221916	16.05390621	-0.27614431	0.444266905	-0.621572992	0.534222685	0.685370327
ENSG00000221926	27.15016536	0.337947615	0.378307265	0.893315162	0.371688421	0.542396348
ENSG00000221944	39.98835217	-0.369567324	0.278051515	-1.329132566	0.183804238	0.331182418
ENSG00000221947	13.9946676	-0.490228369	0.466136547	-1.051684046	0.292944533	0.463541004
ENSG00000221963	39.44650341	1.993558621	0.312202035	6.385476061	1.71E-10	2.62E-09
ENSG00000221968	90.98640528	0.183932937	0.185934936	0.989232798	0.322549255	0.494364363
ENSG00000221978	932.3523896	0.025410429	0.091289186	0.278350918	0.780742993	0.868632739
ENSG00000221983	2949.683102	0.496373073	0.063388647	7.830630486	4.85E-15	1.17E-13
ENSG00000221988	106.1734109	-0.064066448	0.187611591	-0.341484489	0.73273888	0.835385362
ENSG00000222011	31.04950164	-0.467551946	0.326614941	-1.43150814	0.152284636	0.288410909
ENSG00000222041	113.0704259	-0.418826226	0.173703149	-2.411160811	0.015901835	0.047882761
ENSG00000222257	16.10336009	-0.127105793	0.426883272	-0.297753042	0.765891657	0.858785918
ENSG00000222259	10.7815902	-0.027633876	0.518069695	-0.053340075	0.957460951	0.977076206
ENSG00000222489	22.82364849	-0.47880386	0.369807238	-1.294739016	0.195410276	0.346545487
ENSG00000222664	10.52030141	-0.566916981	0.561219535	-1.010151902	0.312422519	0.484018873
ENSG00000222724	112.020797	1.182958332	0.17802361	6.644951945	3.03E-11	5.10E-10
ENSG00000222898	14.32435399	1.139517373	0.467037049	2.439886462	0.014691879	0.044699333
ENSG00000223118	10.61142808	0.356409547	0.532852682	0.668870702	0.503577961	0.661346262
ENSG00000223145	9.604867477	-0.92044263	0.565073924	-1.628888877	0.103336545	0.215160102
ENSG00000223260	10.73271636	-0.45738272	0.55424256	-0.825239259	0.409235769	0.577637398
ENSG00000223305	11.31287775	-0.256883693	0.52955311	-0.48509524	0.627608788	0.758494295
ENSG00000223482	260.9873548	-0.066904409	0.116172153	-0.57590745	0.56467774	0.710691534
ENSG00000223486	28.05000772	-0.073127212	0.329696289	-0.221801744	0.824468222	0.89742775
ENSG00000223496	479.7463652	-0.140208906	0.093402408	-1.501127315	0.133322635	0.261708085
ENSG00000223501	269.4515393	0.236062348	0.118901312	1.985363706	0.047104021	0.116341935
ENSG00000223508	12.24424565	-0.172901671	0.500903877	-0.345179343	0.729959548	0.833095691
ENSG00000223519	15.20673579	-0.253354534	0.44459321	-0.569856958	0.56877472	0.714184186
ENSG00000223529	12.11258259	-0.250819588	0.497066686	-0.504599472	0.613840181	0.748716754
ENSG00000223546	61.63187972	-0.446075354	0.250474699	-1.780919815	0.074925551	0.16732579
ENSG00000223572	20.5296989	-1.995676942	0.436565966	-4.571306735	4.85E-06	3.72E-05
ENSG00000223599	11.18124784	-0.838991169	0.543114704	-1.544777122	0.122400183	0.245762631
ENSG00000223669	8.119611535	-0.16750001	0.606122856	-0.276346632	0.782281842	0.869773076
ENSG00000223722	73.46018755	0.73502619	0.20698137	3.551170771	0.000383521	0.001915153
ENSG00000223745	66.54885028	-0.515767441	0.215786596	-2.390173677	0.016840407	0.050158465
ENSG00000223749	29.23240011	1.060931044	0.339137472	3.128321498	0.001758078	0.007323759
ENSG00000223760	13.5743291	0.450467265	0.504796516	0.892373957	0.372192525	0.542850006
ENSG00000223764	28.58744477	-0.326247253	0.33203193	-0.982577951	0.325815201	0.497957363
ENSG00000223768	103.4339329	-0.470127297	0.181088454	-2.596119661	0.009428322	0.030714203
ENSG00000223770	7.087531141	-2.866654963	0.837801566	-3.421639538	0.000622448	0.002943947
ENSG00000223802	99.39182664	0.498728966	0.185432184	2.689549111	0.007154862	0.024294016
ENSG00000223812	26.75387731	-2.048723217	0.404875085	-5.060136548	4.19E-07	4.01E-06
ENSG00000223921	7.950922559	-1.875937445	0.703802199	-2.665432768	0.00768893	0.025869798
ENSG00000223959	163.756817	0.074540879	0.144044101	0.517486513	0.604816582	0.741997252
ENSG00000223960	81.27416858	-0.221623007	0.213103394	-1.039978777	0.298349761	0.468992736
ENSG00000224032	343.1052457	-0.225254894	0.116045217	-1.941095882	0.052246648	0.126252997
ENSG00000224043	13.73648906	-0.317330614	0.466661288	-0.680002011	0.496503187	0.655099069
ENSG00000224051	109.7198014	0.045234527	0.170823216	0.264803158	0.791161104	0.875349719
ENSG00000224063	58.46813428	-3.030746845	0.321107672	-9.438413064	3.78E-21	1.37E-19
ENSG00000224078	1421.260977	-0.477387085	0.077372417	-6.169990636	6.83E-10	9.73E-09
ENSG00000224083	13.736898	-0.225814031	0.46682906	-0.483718883	0.62858539	0.759090352

ENSG00000224099	45.06243821	-3.208750864	0.36671579	-8.749966465	2.13E-18	6.63E-17
ENSG00000224141	34.64908611	0.402523082	0.295350563	1.362865465	0.172924915	0.316650919
ENSG00000224152	64.81172299	0.142341779	0.221037995	0.643969734	0.51959506	0.67424959
ENSG00000224165	10.32379345	-0.174081077	0.531308858	-0.327645727	0.743179544	0.842922019
ENSG00000224186	11.04965575	-0.635763929	0.557453494	-1.140478867	0.254086852	0.418964434
ENSG00000224237	17.42832891	-0.25644819	0.408926509	-0.627125375	0.530577055	0.682760708
ENSG00000224322	9.999660234	-6.603047413	1.258323575	-5.247495592	1.54E-07	1.58E-06
ENSG00000224356	14.873353	-1.946254806	0.513243399	-3.792069826	0.000149397	0.000825237
ENSG00000224411	16.69329619	0.622922266	0.418829573	1.487292937	0.136937459	0.266605751
ENSG00000224420	16.38893892	-0.048854105	0.422192811	-0.115715151	0.907878298	0.950649754
ENSG00000224424	9.125396852	-0.701175934	0.578617861	-1.211811768	0.225584443	0.383961822
ENSG00000224470	344.7140925	0.518167782	0.10611773	4.882952014	1.05E-06	9.22E-06
ENSG00000224505	28.46232425	-0.210834812	0.326803706	-0.645142048	0.518835137	0.67376141
ENSG00000224531	240.7683908	0.114506036	0.127134548	0.900668134	0.367764796	0.539135055
ENSG00000224616	26.74000821	-0.90566548	0.345926025	-2.618090037	0.008842347	0.029095444
ENSG00000224631	33.78083377	-0.269855847	0.308709438	-0.874141874	0.382040996	0.551488015
ENSG00000224635	10.74956693	-0.659138156	0.544387109	-1.210789428	0.225976118	0.384346812
ENSG00000224660	336.0491269	0.101914211	0.117839772	0.864854115	0.387118865	0.555847766
ENSG00000224699	33.80549408	-0.354701	0.300212438	-1.181500016	0.237404147	0.398806034
ENSG00000224728	9.324208579	0.103973134	0.570970823	0.182098857	0.855505146	0.917119467
ENSG00000224729	9.858380049	-0.058221933	0.565689533	-0.102922062	0.918024828	0.955355281
ENSG00000224790	14.87296016	-0.033992103	0.44919351	-0.075673629	0.939678757	0.966654522
ENSG00000224817	11.47097545	-0.506956643	0.518178999	-0.978342704	0.327904856	0.500280168
ENSG00000224819	6.933914674	-3.623784307	0.9954231	-3.640446265	0.000272166	0.001409183
ENSG00000224852	12.26850451	-0.559890537	0.496956362	-1.126639239	0.259895042	0.425974404
ENSG00000224861	14.21986647	-0.61681415	0.473116374	-1.303726067	0.192326998	0.342757846
ENSG00000224870	75.64024175	-0.177809183	0.210582535	-0.844368139	0.39846372	0.566577577
ENSG00000224877	285.2268832	0.150771658	0.110721413	1.361720869	0.173285993	0.317070254
ENSG00000224903	66.57858364	0.165199072	0.223462744	0.739268966	0.45974369	0.623000156
ENSG00000224905	17.45254412	-0.166773489	0.423382707	-0.393907182	0.693649568	0.807332462
ENSG00000224914	14.72741256	-0.458232571	0.451190019	-1.015608838	0.309815695	0.481094524
ENSG00000224934	10.52258918	-0.731104696	0.544633129	-1.34238014	0.179472771	0.325381261
ENSG00000224986	14.23217522	-0.27630106	0.490037919	-0.563836081	0.572865694	0.717717124
ENSG00000225022	13.73797239	0.01460197	0.466459823	0.031303811	0.975027251	0.987327552
ENSG00000225031	11.67545862	0.463390427	0.502890599	0.921453747	0.356813579	0.528096127
ENSG00000225077	32.33867973	-0.890210522	0.316934993	-2.808811085	0.004972482	0.017818482
ENSG00000225092	17.06313188	-0.727315755	0.435981643	-1.668225639	0.095270943	0.202125198
ENSG00000225110	9.709815097	-0.048601077	0.56849775	-0.08549036	0.931871559	0.963137355
ENSG00000225131	15.80921466	0.503105531	0.442397629	1.137224746	0.255444344	0.420758291
ENSG00000225138	76.8837498	-0.258902552	0.202661954	-1.277509401	0.201422463	0.353805345
ENSG00000225190	60.89558397	0.148835697	0.251688371	0.591349121	0.554286524	0.701383911
ENSG00000225200	8.116473848	0.220668272	0.611366283	0.360942822	0.718142191	0.824364089
ENSG00000225206	282.3857308	1.39618003	0.118383751	11.79367963	4.21E-32	2.48E-30
ENSG00000225210	8.430883129	-1.043851548	0.609600735	-1.712352836	0.086831665	0.188083948
ENSG00000225215	10.61959949	-0.783614188	0.555011829	-1.411887362	0.15798313	0.296247366
ENSG00000225224	11.25049921	-0.323976168	0.510613357	-0.63448432	0.525764797	0.678994033
ENSG00000225264	25.20830825	-0.004784248	0.366754953	-0.013044809	0.989592044	0.994744093
ENSG00000225285	7.979119666	-1.186268578	0.632533571	-1.875423903	0.060734429	0.142284278
ENSG00000225300	9.895242451	-0.073763173	0.569560096	-0.129509026	0.896954883	0.944134611
ENSG00000225345	10.73194959	0.549691349	0.537689321	1.022321493	0.306628764	0.477595697

ENSG00000225361	9.841614135	-0.307596951	0.571159067	-0.538548662	0.590198319	0.731408539
ENSG00000225377	44.2989656	-0.534866829	0.265726573	-2.012846602	0.04413077	0.11057883
ENSG00000225423	9.451705557	-0.009775628	0.556247773	-0.017574232	0.985978513	0.992629448
ENSG00000225439	63.59883419	0.220153234	0.218440147	1.007842361	0.313530146	0.484772478
ENSG00000225447	7.635274157	-0.362800618	0.656762907	-0.552407291	0.580669336	0.723518357
ENSG00000225465	117.6260082	0.161738863	0.172643092	0.936839471	0.348841139	0.520796203
ENSG00000225470	648.2893641	-0.20732555	0.104565558	-1.982732698	0.047397295	0.116866738
ENSG00000225484	222.538238	-0.291840695	0.129149747	-2.259707824	0.02383939	0.066614664
ENSG00000225518	15.36188297	0.247227062	0.441789086	0.559604276	0.575749388	0.719981452
ENSG00000225530	10.2397609	0.485536986	0.56701752	0.856299795	0.391831975	0.560096036
ENSG00000225614	117.9634646	-1.08317639	0.198412595	-5.459211846	4.78E-08	5.31E-07
ENSG00000225616	43.16180345	1.104530168	0.296941093	3.719694556	0.000199464	0.001067785
ENSG00000225630	37912.39217	-0.324368152	0.07518306	-4.31437815	1.60E-05	0.000109725
ENSG00000225663	124.1767477	0.812263329	0.177399555	4.578722472	4.68E-06	3.59E-05
ENSG00000225697	390.6058418	0.297442689	0.098322368	3.025178251	0.002484866	0.009843309
ENSG00000225733	519.8850881	-0.21431224	0.095143972	-2.252504656	0.024290399	0.067659574
ENSG00000225756	20.31737571	-0.089887825	0.386225813	-0.232733862	0.815968073	0.891907519
ENSG00000225761	10.42675597	-1.744954741	0.600264122	-2.90697824	0.003649386	0.013666468
ENSG00000225783	20.00313079	-0.12224159	0.406103191	-0.301011153	0.76340599	0.857280464
ENSG00000225791	24.35138268	-0.732566954	0.364834824	-2.007941416	0.044649517	0.111539379
ENSG00000225813	22.40686076	-0.609082852	0.386488202	-1.575941641	0.115039253	0.233891502
ENSG00000225828	43.30300078	0.241463119	0.27162083	0.888971285	0.374018512	0.544085443
ENSG00000225830	161.5873791	-0.167455062	0.144119435	-1.16191867	0.245268503	0.408790538
ENSG00000225855	40.53232146	-0.780666112	0.286719584	-2.722751269	0.006474078	0.022256822
ENSG00000225857	13.1491692	0.358396999	0.488682826	0.733393892	0.463318224	0.62643451
ENSG00000225880	13.23520896	0.208591481	0.487077831	0.428250822	0.668468519	0.788094582
ENSG00000225891	9.430511439	-1.107324353	0.609536666	-1.816665698	0.069268317	0.157777209
ENSG00000225921	513.5355718	0.337063191	0.093184119	3.617174207	0.000297837	0.001529135
ENSG00000225950	9.735505885	-1.658063976	0.604756358	-2.741705737	0.006112107	0.021178285
ENSG00000225968	114.046395	-3.830952211	0.269311284	-14.22499701	6.41E-46	5.96E-44
ENSG00000225972	10338.14265	-0.610330255	0.071134474	-8.579950389	9.49E-18	2.77E-16
ENSG00000225973	45.48584335	-0.244530644	0.259875516	-0.940952991	0.34672895	0.518745507
ENSG00000225975	14.93358547	0.439569311	0.453715822	0.968820766	0.332634624	0.505027946
ENSG00000226043	52.03858542	5.431797566	0.54970635	9.881271266	5.02E-23	1.97E-21
ENSG00000226049	8.46452722	-0.157172011	0.598362465	-0.262670238	0.792804758	0.876423602
ENSG00000226053	19.18051541	0.931534988	0.406956884	2.289026248	0.022077827	0.062538262
ENSG00000226121	23.44103612	-0.11313723	0.365254927	-0.309748676	0.756752083	0.853000844
ENSG00000226124	21.62279267	-0.277526268	0.388344266	-0.714639799	0.474831646	0.63590048
ENSG00000226137	503.5038566	0.108380137	0.09196171	1.178535469	0.238583201	0.400164564
ENSG00000226174	11.86131704	-0.581386785	0.558198662	-1.041540988	0.297624541	0.468271945
ENSG00000226179	8.259088203	-1.20507734	0.623915654	-1.931474763	0.053424367	0.128348228
ENSG00000226180	18.92370354	-1.286514054	0.418363786	-3.075108548	0.00210426	0.008514292
ENSG00000226200	25.50456883	-0.408480373	0.351402235	-1.16242964	0.245060989	0.408493143
ENSG00000226210	103.4805925	-0.154564697	0.173393214	-0.891411454	0.372708475	0.543151359
ENSG00000226266	13.88553385	-0.641960363	0.465106106	-1.380244968	0.167511233	0.309718114
ENSG00000226278	7.069585199	-0.931004809	0.692911064	-1.343613715	0.179073332	0.324908693
ENSG00000226310	21.21329206	0.042011071	0.389980998	0.107725944	0.914213089	0.953636867
ENSG00000226328	16.41499371	-0.201994517	0.441931966	-0.457071522	0.647619638	0.773169561
ENSG00000226396	10.48897475	-0.090492231	0.539722468	-0.167664376	0.866847323	0.925339922
ENSG00000226419	129.5742285	-0.094812717	0.168603058	-0.562342809	0.573882477	0.718394287

ENSG00000226445	17.76544299	-0.024702215	0.41322154	-0.059779593	0.952331179	0.973822237
ENSG00000226479	224.5635372	0.326154691	0.126685646	2.574519696	0.010037937	0.032384681
ENSG00000226519	12.10616535	-0.630492836	0.513454157	-1.227943775	0.219468075	0.376832667
ENSG00000226562	93.6834027	-0.047063363	0.185327889	-0.253946471	0.799536913	0.880801115
ENSG00000226608	56.4450004	0.303690077	0.232452618	1.306460126	0.19139613	0.341575587
ENSG00000226666	13.85031416	-0.363189437	0.467287872	-0.777228468	0.437023989	0.603033004
ENSG00000226683	39.60070842	-2.806333991	0.361216727	-7.769114165	7.90E-15	1.87E-13
ENSG00000226688	317.077583	0.056392306	0.110020786	0.51256047	0.608258811	0.744723395
ENSG00000226696	27.16050839	0.328957312	0.356424588	0.922936641	0.356040224	0.527308874
ENSG00000226701	9.002513037	0.065108802	0.56698536	0.114833304	0.908577251	0.95095631
ENSG00000226711	10.34193257	0.061662288	0.527603222	0.116872462	0.90696112	0.95025605
ENSG00000226742	59.2490104	0.193284195	0.250052564	0.772974255	0.439537617	0.604795228
ENSG00000226752	112.9392457	-0.2525831	0.176521423	-1.430892045	0.152461157	0.288706104
ENSG00000226763	19.90891662	-0.341297075	0.394570962	-0.864982748	0.387048258	0.555803197
ENSG00000226800	10.05214213	-0.216760787	0.544724427	-0.397927423	0.690683685	0.805348529
ENSG00000226819	8.279194438	-0.873443777	0.614466832	-1.42146611	0.155181299	0.292709201
ENSG00000226824	31.54010454	-0.333873467	0.340059159	-0.981809952	0.326193485	0.498318638
ENSG00000226833	12.57678984	-0.582602498	0.504179635	-1.155545479	0.24786712	0.412129074
ENSG00000226889	8.415962495	0.404598897	0.585396509	0.691153587	0.489469028	0.6491557
ENSG00000226900	65.92489652	-0.3647936	0.218620739	-1.668613883	0.095193926	0.202022825
ENSG00000226950	154.1420817	0.371952474	0.161260671	2.306529371	0.021081071	0.06031768
ENSG00000227001	8.886543267	-0.935381747	0.619512602	-1.509867182	0.131077318	0.25831132
ENSG00000227057	325.9008748	0.109812668	0.111878043	0.981539055	0.326326985	0.498468373
ENSG00000227081	48.41297189	0.151159162	0.26388045	0.572831983	0.566758462	0.712289175
ENSG00000227097	26.67765037	0.353819233	0.373827017	0.946478495	0.343904586	0.515728378
ENSG00000227124	58.57896693	0.101328387	0.23147704	0.43774703	0.661569673	0.783696171
ENSG00000227128	22.10288064	-5.891919417	1.035698471	-5.688836645	1.28E-08	1.55E-07
ENSG00000227195	70.88237127	1.758830293	0.880929147	1.996562719	0.045872706	0.113967793
ENSG00000227252	14.30442635	-0.903000295	0.483673295	-1.86696331	0.061906712	0.14442843
ENSG00000227268	13.88162087	-1.885452097	0.527222069	-3.576201013	0.000348624	0.001757759
ENSG00000227308	11.96931874	-1.85027563	0.56870223	-3.253505143	0.001139907	0.005041646
ENSG00000227309	21.86029065	-0.213628219	0.377404881	-0.566045194	0.571363059	0.716537531
ENSG00000227331	22.15341132	-0.357849959	0.372075207	-0.961767816	0.336166248	0.508028756
ENSG00000227345	192.9562143	-0.209200817	0.152942416	-1.367840413	0.171362035	0.314504514
ENSG00000227354	19.3842839	-0.080329402	0.396597056	-0.202546643	0.839489398	0.906612407
ENSG00000227355	11.40320104	0.29620518	0.515580617	0.574507982	0.565624101	0.711309019
ENSG00000227372	201.4130498	-0.565154575	0.131765022	-4.289109255	1.79E-05	0.000121563
ENSG00000227398	34.98214609	-0.395272532	0.301980841	-1.308932485	0.190557222	0.34025134
ENSG00000227500	461.9201883	-0.015209583	0.115359879	-0.131844654	0.895107166	0.943073102
ENSG00000227583	10.27744954	-0.650125181	0.56635917	-1.147902629	0.251008763	0.41530272
ENSG00000227615	127.3524419	0.26999111	0.16161977	1.670532697	0.094814016	0.201317943
ENSG00000227627	12.10247534	-0.240022097	0.494070536	-0.485805324	0.627105199	0.758020838
ENSG00000227671	38.151389	-0.338739314	0.283049094	-1.196751097	0.231403584	0.390923169
ENSG00000227704	12.84282721	-0.560496241	0.492592197	-1.137850424	0.255182945	0.42044762
ENSG00000227740	11.52316931	-2.022613229	0.580567908	-3.483852968	0.000494251	0.002397356
ENSG00000227885	15.66649348	-0.921151818	0.444701655	-2.071392827	0.038322101	0.098375466
ENSG00000227946	53.10435674	-0.152736046	0.239653571	-0.637320134	0.523916339	0.677354147
ENSG00000227953	247.0271314	-0.183129423	0.135409453	-1.3524124	0.176243425	0.321143001
ENSG00000228043	8.252645952	-0.295658322	0.594588507	-0.497248633	0.61901373	0.75257797
ENSG00000228109	36.79344375	0.473015757	0.289353868	1.634731067	0.102105437	0.213180005

ENSG00000228166	11.34706087	0.06241864	0.512329066	0.121833104	0.903031196	0.947693628
ENSG00000228172	15.93433455	-0.778454697	0.445111635	-1.748897658	0.080308712	0.17706741
ENSG00000228192	13.82676139	-1.291653606	0.513704094	-2.514392277	0.011923772	0.037453763
ENSG00000228221	10.8261911	-0.0849599	0.52550177	-0.161673861	0.87156269	0.928472706
ENSG00000228223	45.7323333	1.031614686	0.269343139	3.830113104	0.000128084	0.000719968
ENSG00000228232	27.05817565	0.671634218	0.330733499	2.030741426	0.042281232	0.107013417
ENSG00000228253	114209.2909	-0.210130881	0.059795938	-3.51413305	0.000441192	0.002167688
ENSG00000228274	48.06883928	0.079963024	0.257734526	0.310253441	0.756368234	0.852705022
ENSG00000228288	57.32349495	-0.026168417	0.228366079	-0.114589771	0.908770288	0.951016631
ENSG00000228300	228.2023557	0.823273044	0.141470414	5.819400812	5.91E-09	7.49E-08
ENSG00000228315	52.94826364	-0.387030408	0.256023408	-1.511699302	0.130610376	0.257607969
ENSG00000228393	61.76404031	-0.062046081	0.249506419	-0.248675292	0.803611963	0.883419725
ENSG00000228412	97.26718695	0.243805373	0.197876404	1.232109376	0.217908229	0.374933476
ENSG00000228423	9.378828422	-0.445242855	0.658399473	-0.67625032	0.498881729	0.656959937
ENSG00000228436	11.84945281	0.77005813	0.498992258	1.54322661	0.122775803	0.246340767
ENSG00000228474	1453.731729	0.442545302	0.080403283	5.504070081	3.71E-08	4.21E-07
ENSG00000228486	21.86162397	-0.358474374	0.396569246	-0.903938916	0.366027792	0.537374197
ENSG00000228502	22.74889676	-0.021553911	0.35695813	-0.060382181	0.95185125	0.973475269
ENSG00000228506	45.71647983	-0.0829063	0.266103899	-0.311556126	0.755377882	0.852072204
ENSG00000228526	12.96698216	0.339055205	0.476973227	0.710847455	0.47717877	0.637712665
ENSG00000228544	21.98936713	0.068438083	0.378219705	0.180947958	0.856408425	0.917757792
ENSG00000228549	13.20988921	-0.222800019	0.496250766	-0.4489666	0.653455751	0.777559757
ENSG00000228594	199.2442721	-0.339026358	0.135554148	-2.501040086	0.012382916	0.038690492
ENSG00000228630	21.07755597	-1.231466886	0.412774471	-2.983389168	0.002850753	0.011058968
ENSG00000228649	67.31272741	-0.680727614	0.222927949	-3.053576799	0.002261308	0.00907562
ENSG00000228672	13.06816411	0.297876571	0.497675022	0.598536309	0.549482139	0.69750367
ENSG00000228705	27.66673053	0.244049139	0.331445354	0.736317874	0.461537271	0.624677335
ENSG00000228708	91.64354907	-0.259267894	0.191212855	-1.355912471	0.175127019	0.319606241
ENSG00000228709	152.4439501	1.312079728	0.151862803	8.639901955	5.63E-18	1.67E-16
ENSG00000228716	151.4756748	-0.455248095	0.14942904	-3.046583814	0.002314579	0.00925239
ENSG00000228794	127.7961961	0.045579725	0.165711248	0.275055106	0.783273902	0.870531569
ENSG00000228801	29.24324285	-0.214209655	0.34081484	-0.62852209	0.52966198	0.682186526
ENSG00000228808	8.669937114	-0.26238515	0.601613775	-0.436135542	0.662738391	0.784604829
ENSG00000228824	32.21438367	-0.520169085	0.31483535	-1.652194027	0.098495004	0.207274804
ENSG00000228828	15.96576197	-0.466896514	0.439971358	-1.06119752	0.288600146	0.458749151
ENSG00000228878	13.74354523	-0.184326767	0.464085133	-0.397183089	0.691232452	0.805563918
ENSG00000228889	11.64400396	-0.016497903	0.50231856	-0.032843507	0.973799384	0.986408007
ENSG00000228929	12.81464797	-0.315994651	0.503030747	-0.628181583	0.529884994	0.682240213
ENSG00000228933	122.4347084	0.026962431	0.161083548	0.167381656	0.867069758	0.925440991
ENSG00000228998	8.254583937	1.677071488	0.654314947	2.563095183	0.01037436	0.033317019
ENSG00000229043	51.09478131	-0.018366286	0.242551928	-0.075721047	0.939641031	0.966654522
ENSG00000229081	12.52274124	0.160165189	0.511306663	0.313246826	0.754093145	0.851326439
ENSG00000229117	2770.429776	0.311113928	0.069700797	4.463563394	8.06E-06	5.91E-05
ENSG00000229127	30.37547791	-0.365352443	0.325887446	-1.121100084	0.262245263	0.428593276
ENSG00000229152	21.8397515	-0.343151901	0.371378967	-0.923993902	0.355489491	0.527037577
ENSG00000229235	17.13741322	-0.234230173	0.438123814	-0.534620958	0.592911989	0.733089931
ENSG00000229246	56.14650825	-1.20664399	0.251050117	-4.806386878	1.54E-06	1.31E-05
ENSG00000229267	10.99618269	-0.523005718	0.550871524	-0.949415056	0.342409556	0.514366687
ENSG00000229320	12.50264071	-0.68294195	0.512243209	-1.333237686	0.182453816	0.329425289
ENSG00000229344	154.2960669	0.027094642	0.154664786	0.175183004	0.860935838	0.920923372

ENSG00000229358	15.05679779	0.103269577	0.461332151	0.223850813	0.82287339	0.896348169
ENSG00000229419	12.3730312	-0.657051768	0.511305408	-1.285047563	0.198775664	0.350564914
ENSG00000229492	13.84901801	0.093598855	0.470202163	0.199060878	0.842215124	0.908438597
ENSG00000229539	19.20090858	0.037884227	0.391759628	0.096702735	0.922962468	0.957522436
ENSG00000229587	11.36246564	0.301899172	0.527357051	0.572475843	0.566999647	0.712464804
ENSG00000229604	455.5622911	-0.292152488	0.097720738	-2.989667223	0.002792815	0.0108582
ENSG00000229619	22.71337583	-0.903026422	0.369861903	-2.441523211	0.014625448	0.044535805
ENSG00000229622	25.35529582	0.600998287	0.358145012	1.678086438	0.093330226	0.198818575
ENSG00000229638	46.23872262	0.453005738	0.272898989	1.659975873	0.096919306	0.204510364
ENSG00000229676	7.23010803	-4.228845752	1.120260656	-3.774876613	0.000160087	0.000877638
ENSG00000229739	8.414577388	-0.539835677	0.609983136	-0.885000985	0.3761561	0.546004956
ENSG00000229807	6220.823819	0.137259274	0.059929444	2.290347864	0.022001159	0.062371399
ENSG00000229809	36.21196028	0.169387666	0.292749599	0.578609386	0.56285277	0.70902994
ENSG00000229816	17.88894075	0.304282973	0.406872011	0.747859191	0.454545095	0.618431859
ENSG00000229921	73.29631759	0.003454243	0.219009711	0.015772097	0.987416209	0.993427552
ENSG00000229953	77.25012426	-0.524477549	0.21484306	-2.441212434	0.014638041	0.044564492
ENSG00000229954	6.482194468	-0.065901915	0.706998764	-0.093213621	0.925733853	0.959334816
ENSG00000229980	9.278384117	-0.091075471	0.588299517	-0.154811399	0.876970004	0.932041889
ENSG00000229994	15.79034333	-0.210178195	0.443365354	-0.474051914	0.635462887	0.764634845
ENSG00000230002	12.78037234	-0.950454588	0.514482489	-1.847399295	0.064689291	0.149578679
ENSG00000230074	14.3838537	-0.466081363	0.476793638	-0.977532679	0.328305508	0.500640031
ENSG00000230091	8.904995351	-1.140161931	0.661638351	-1.723240392	0.084845062	0.184835389
ENSG00000230124	502.0844053	-0.036598713	0.092189592	-0.396993983	0.691371898	0.805616014
ENSG00000230148	78.05594627	-0.21132796	0.207595122	-1.017981338	0.308686825	0.479925161
ENSG00000230184	8.665227966	-0.014148172	0.609548631	-0.023210899	0.981482044	0.990660448
ENSG00000230196	15.70482901	-0.623370618	0.465329388	-1.33963303	0.18036468	0.326618881
ENSG00000230202	63.94261359	-0.105732746	0.226772096	-0.466251129	0.641035756	0.768258186
ENSG00000230207	16.52032114	0.563099232	0.434916126	1.294730634	0.195413168	0.346545487
ENSG00000230291	18.33023868	-0.088678857	0.405564279	-0.218655493	0.826918422	0.898668717
ENSG00000230325	9.7036638	-0.405334678	0.562967251	-0.719996904	0.471526901	0.633407133
ENSG00000230383	15.85844079	0.197024331	0.462554811	0.425948074	0.670145683	0.789527503
ENSG00000230392	142.3416832	0.823783116	0.169101366	4.871534378	1.11E-06	9.70E-06
ENSG00000230409	10.06324464	0.087497258	0.535492056	0.163395996	0.870206655	0.927872543
ENSG00000230426	33.65523769	-0.28740601	0.298793236	-0.961889278	0.336105225	0.507991187
ENSG00000230450	22.5020066	0.107468735	0.389838306	0.275675153	0.782797581	0.870139877
ENSG00000230453	399.9658385	-0.112921197	0.104319128	-1.082459175	0.279048547	0.447809167
ENSG00000230454	14.5298612	-0.295507459	0.461395742	-0.640464209	0.52187085	0.676016215
ENSG00000230457	10.05701272	0.148279587	0.541525346	0.273818369	0.784224207	0.871011269
ENSG00000230482	11.67238415	0.463310208	0.53327369	0.868803799	0.384954455	0.554099316
ENSG00000230487	38.16049396	-0.452839504	0.295643522	-1.531707855	0.125594543	0.250315912
ENSG00000230513	19.24481127	-0.361695311	0.405025091	-0.893019517	0.371846721	0.542514634
ENSG00000230551	104.4729905	-0.422643656	0.179217643	-2.35827036	0.018360316	0.053864066
ENSG00000230581	13.63354011	-0.149086479	0.466902622	-0.319309577	0.749491771	0.848015377
ENSG00000230590	331.9145137	-0.116023733	0.120441701	-0.963318618	0.335387653	0.507124904
ENSG00000230606	34.10940438	-0.768113384	0.310700978	-2.472194935	0.013428627	0.041441742
ENSG00000230615	11.62543417	-1.241445073	0.558664595	-2.222165293	0.026272137	0.072304998
ENSG00000230629	16.66583046	-0.109352626	0.445923417	-0.245227368	0.806280387	0.885511821
ENSG00000230630	712.3616506	-0.446630412	0.08674266	-5.148913025	2.62E-07	2.59E-06
ENSG00000230699	31.99463794	-0.125853974	0.308484585	-0.407974919	0.683292088	0.799704389
ENSG00000230701	17.99642793	0.097324428	0.410719889	0.236960591	0.812687363	0.889844836

ENSG00000230731	27.30109067	-0.501345477	0.343858411	-1.457999749	0.144840612	0.277795751
ENSG00000230732	23.74779128	-0.291494781	0.388494203	-0.750319514	0.453062293	0.617396281
ENSG00000230777	48.00517628	0.509649569	0.259099329	1.967004591	0.049182682	0.120320505
ENSG00000230797	22.6531247	0.28558339	0.392138542	0.728271669	0.466447301	0.628880558
ENSG00000230844	13.57643024	0.080702559	0.507715064	0.158952461	0.873706328	0.930050984
ENSG00000230897	19.78751081	0.487674921	0.398861516	1.222667271	0.221455393	0.379225151
ENSG00000230922	13.57109124	-0.114510762	0.466438163	-0.245500415	0.806068987	0.885358594
ENSG00000230928	10.63989256	-0.074219003	0.532019066	-0.139504405	0.889051576	0.93933866
ENSG00000230989	340.9525357	-0.020820137	0.108888204	-0.191206546	0.848363775	0.912928344
ENSG00000231028	13.26171982	-0.881155308	0.504917243	-1.745147981	0.080959113	0.1782217
ENSG00000231043	7.952616407	-0.79610663	0.618169588	-1.287845027	0.197799915	0.349320519
ENSG00000231050	12.65862762	0.175774646	0.533184463	0.329669482	0.741649715	0.842008184
ENSG00000231064	24.38926029	-0.633263349	0.359414169	-1.761932064	0.078080776	0.173274477
ENSG00000231074	525.8928656	-0.512071794	0.100478868	-5.09631335	3.46E-07	3.36E-06
ENSG00000231167	24.03083571	-0.747744911	0.380436097	-1.965494118	0.049357076	0.120640133
ENSG00000231177	40.23066174	0.289814202	0.284070101	1.020220714	0.307623796	0.478661249
ENSG00000231181	10.32667335	-0.917862673	0.606035754	-1.514535516	0.129890073	0.256489013
ENSG00000231185	60.8284556	-0.604822898	0.237319605	-2.548558509	0.010816914	0.034462507
ENSG00000231222	34.90685339	-1.157694979	0.311154978	-3.720637822	0.00019872	0.00106421
ENSG00000231274	6.194280686	-2.845103645	0.907028445	-3.136730343	0.001708432	0.00715514
ENSG00000231312	35.88087547	-0.163306875	0.291484504	-0.560259199	0.575302653	0.719550918
ENSG00000231327	8.75474646	0.660634656	0.622469456	1.061312566	0.288547877	0.458717975
ENSG00000231365	99.06435078	0.0690518	0.178407663	0.387045036	0.698722868	0.810918182
ENSG00000231369	7.818344838	0.083311835	0.607384048	0.137165003	0.890900373	0.94027635
ENSG00000231389	14.03172817	-1.077363284	0.488598081	-2.205009244	0.027453446	0.074910364
ENSG00000231416	16.56687934	-0.113905813	0.43128068	-0.264110632	0.791694672	0.875713229
ENSG00000231500	10988.14648	0.286137078	0.05426353	5.273101029	1.34E-07	1.39E-06
ENSG00000231503	14.57566085	-0.935551812	0.465896752	-2.008066827	0.044636191	0.111526961
ENSG00000231533	26.04246122	-1.576632613	0.389468676	-4.048162822	5.16E-05	0.000316371
ENSG00000231563	10.64201699	-0.472384305	0.528477588	-0.89385873	0.371397482	0.54217406
ENSG00000231566	10.29411329	-0.043906772	0.574794999	-0.076386838	0.939111341	0.966353894
ENSG00000231607	107.8116845	0.106875876	0.175284905	0.60972664	0.542042905	0.691497292
ENSG00000231663	14.33672867	-0.019686864	0.489959925	-0.040180559	0.967949177	0.983065728
ENSG00000231689	25.56108283	-0.09807081	0.337981268	-0.290166408	0.771688934	0.86337203
ENSG00000231690	8.562914223	-0.955869312	0.601129739	-1.590121483	0.111807424	0.228777697
ENSG00000231697	9.871970911	-1.098697432	0.583249339	-1.883752556	0.059598454	0.139973162
ENSG00000231721	754.7986824	1.223314195	0.089935156	13.6021802	3.89E-42	3.23E-40
ENSG00000231744	8.2439491	-1.068647705	0.617853664	-1.729612961	0.083699449	0.183049379
ENSG00000231770	12.99112855	-0.593517385	0.478324991	-1.240824535	0.214670576	0.370701814
ENSG00000231784	12.15339674	0.002877368	0.495037323	0.005812427	0.99536238	0.997940364
ENSG00000231789	20.1055014	-1.78769344	0.431802875	-4.140068405	3.47E-05	0.000221391
ENSG00000231806	146.331975	-0.036608884	0.158746962	-0.230611556	0.817616589	0.892507031
ENSG00000231856	9.896711941	0.560229294	0.558151698	1.003722278	0.315512496	0.486941084
ENSG00000231871	8.007981749	-0.156703711	0.63306337	-0.247532424	0.804496201	0.88418419
ENSG00000231887	53.37746986	-0.654903276	0.247024598	-2.651166246	0.008021435	0.026826874
ENSG00000231888	16.32044121	0.078549036	0.441982992	0.177719591	0.858943199	0.919492083
ENSG00000231889	38.77441678	0.171280768	0.279283947	0.613285402	0.539687644	0.689745459
ENSG00000231925	884.0201318	0.064076046	0.086357792	0.741983379	0.458097406	0.621909177
ENSG00000232004	9.513204028	0.125091894	0.585454648	0.213666242	0.830807339	0.901344809
ENSG00000232024	13.27803714	0.064179943	0.496141305	0.129358193	0.897074227	0.944134611

ENSG00000232040	17.34443007	-1.107458483	0.454958221	-2.434198202	0.01492482	0.045329502
ENSG00000232046	12.50839065	-0.624115403	0.504923288	-1.236059847	0.216436319	0.37281189
ENSG00000232093	8.885272869	0.338210804	0.571739801	0.591546719	0.554154161	0.701279595
ENSG00000232098	56.66292151	0.090607672	0.235002445	0.38556055	0.699822155	0.811562424
ENSG00000232104	25.04725389	-0.730717064	0.353872878	-2.064914012	0.038931137	0.099683865
ENSG00000232110	37.90488237	-6.137008166	0.866736937	-7.080589163	1.44E-12	2.82E-11
ENSG00000232112	606.3848936	0.381358347	0.08681294	4.392874454	1.12E-05	7.94E-05
ENSG00000232119	119.0994311	0.265315873	0.170474766	1.556335165	0.119628414	0.241335678
ENSG00000232160	14.8882917	-0.389302541	0.467103497	-0.833439577	0.404596862	0.572631951
ENSG00000232177	3635.960842	-0.494906064	0.316934107	-1.561542455	0.118395815	0.239192581
ENSG00000232282	7.369130609	-0.565159751	0.702134075	-0.804917138	0.420867498	0.588378475
ENSG00000232320	8.882021213	0.385997538	0.570342629	0.676781847	0.498544377	0.656577243
ENSG00000232346	39.95532808	0.264000724	0.27770745	0.95064329	0.341785485	0.513702195
ENSG00000232373	18.30048396	0.150522312	0.413324733	0.364174462	0.715727736	0.823072248
ENSG00000232387	28.9503047	0.171040102	0.327273834	0.522620768	0.601238185	0.739437208
ENSG00000232388	17.71197612	0.179926091	0.40414782	0.445198717	0.656176154	0.779542155
ENSG00000232412	7.415155931	-0.475429276	0.649419194	-0.732083808	0.464117416	0.627026563
ENSG00000232434	180.4291788	0.290583427	0.1464362	1.984368803	0.047214741	0.116539355
ENSG00000232442	45.97180058	0.206864521	0.268284221	0.771064809	0.440668518	0.60586547
ENSG00000232445	97.06502131	-0.143506569	0.182272805	-0.787317497	0.43109603	0.597552486
ENSG00000232447	28.40410729	-0.851758173	0.357484423	-2.382644161	0.0171888	0.051089945
ENSG00000232526	23.72062867	0.059985519	0.394260844	0.152146782	0.879071167	0.933709844
ENSG00000232533	10.17706001	-0.105499646	0.541300177	-0.194900446	0.845470885	0.910611721
ENSG00000232545	7.697671812	-0.59565749	0.621532406	-0.958369159	0.337876641	0.509885824
ENSG00000232573	37.90852738	0.143886802	0.28724178	0.500925741	0.616423386	0.750779831
ENSG00000232586	21.35967863	-0.513872788	0.403841457	-1.272461703	0.203209123	0.356186492
ENSG00000232587	20.45930364	-0.203322679	0.400813967	-0.507274436	0.611962273	0.747707172
ENSG00000232593	87.79317589	-0.200951511	0.192293807	-1.045023311	0.296012196	0.466490851
ENSG00000232648	18.66704212	0.354384138	0.397522124	0.891482803	0.372670213	0.543151359
ENSG00000232656	9.580498403	-0.493478185	0.575207395	-0.857913492	0.390940234	0.559276654
ENSG00000232671	20.69191239	-0.000878957	0.393958508	-0.002231089	0.99821985	0.999144522
ENSG00000232855	35.01706587	-6.004901709	0.867895112	-6.918925603	4.55E-12	8.38E-11
ENSG00000232859	30.50590507	-0.872075286	0.33404401	-2.610659856	0.009036773	0.029624164
ENSG00000232888	78.43601553	-0.145207595	0.202219028	-0.718070878	0.472713585	0.633981332
ENSG00000232931	361.5176151	-0.193246776	0.121093676	-1.595845314	0.110523334	0.226791078
ENSG00000232940	11.83697709	-0.597504411	0.51037724	-1.170711318	0.241714834	0.404354922
ENSG00000232956	204.6751757	0.085901261	0.130909325	0.656189015	0.511702516	0.667791925
ENSG00000232973	17.9317769	-3.755931308	0.645206399	-5.821286515	5.84E-09	7.41E-08
ENSG00000232995	11.54546722	-0.337908983	0.507843829	-0.665379716	0.505807655	0.663390746
ENSG00000233006	14.16046472	-0.0373351	0.456035586	-0.081868831	0.934751021	0.964622949
ENSG00000233008	9.452379723	-3.655701442	0.859394991	-4.253808178	2.10E-05	0.000140581
ENSG00000233016	541.500694	0.543407292	0.103138757	5.268701211	1.37E-07	1.42E-06
ENSG00000233080	14.88687673	-5.326381115	1.059804199	-5.025816204	5.01E-07	4.73E-06
ENSG00000233098	629.4226268	0.175894182	0.091329634	1.925926708	0.054113526	0.129759764
ENSG00000233101	66.57903489	-2.848110933	0.279949797	-10.17364886	2.60E-24	1.10E-22
ENSG00000233175	16.92614168	-0.148162606	0.428117908	-0.346078973	0.729283365	0.832773591
ENSG00000233184	19.60077664	-0.768618159	0.398885976	-1.926911965	0.053990602	0.129509219
ENSG00000233215	54.81078839	1.99012524	0.263654416	7.548234053	4.41E-14	1.00E-12
ENSG00000233223	17.107196	-0.37684827	0.421685323	-0.893671773	0.371497532	0.542230448
ENSG00000233230	7.066803465	-1.023159297	0.674922824	-1.51596488	0.129528233	0.256012817

ENSG00000233237	9.472931053	-0.229150419	0.578973013	-0.395787737	0.692261624	0.806274442
ENSG00000233421	10.33917265	0.479718216	0.5386977	0.890514692	0.373189583	0.54340148
ENSG00000233452	5.764195116	-1.65610656	0.774018818	-2.139620539	0.032385445	0.085672006
ENSG00000233476	12.04698029	-0.485476335	0.513136496	-0.9460959	0.344099675	0.515855724
ENSG00000233491	77.61686052	-0.683760951	0.219854227	-3.110065062	0.001870461	0.007732305
ENSG00000233527	12.69804338	-0.441293951	0.504133039	-0.875352172	0.381382315	0.550886667
ENSG00000233558	23.24495301	0.641664424	0.401726145	1.597268268	0.110205921	0.226391133
ENSG00000233560	8.712277977	0.207371025	0.600051056	0.345588967	0.729651639	0.832888023
ENSG00000233579	13.72274077	-0.201860029	0.464259431	-0.43480006	0.663707561	0.784970962
ENSG00000233593	18.89028015	-0.375722369	0.403548166	-0.93104715	0.351829175	0.523476795
ENSG00000233622	9.133890793	-1.603560745	0.665704817	-2.408816498	0.016004343	0.04815014
ENSG00000233695	87.53192762	2.50619519	0.222680424	11.25467224	2.20E-29	1.16E-27
ENSG00000233723	10.03787422	-1.336223063	0.594457557	-2.247802297	0.024588799	0.068341683
ENSG00000233757	116.9269229	-0.184745387	0.174760687	-1.057133559	0.290450637	0.460752129
ENSG00000233762	17.70496292	-0.177715692	0.4155984	-0.427613996	0.668932175	0.788491923
ENSG00000233822	637.4548896	-0.326382454	0.090838267	-3.593006153	0.000326885	0.001657543
ENSG00000233871	7.418651623	0.444269624	0.64429294	0.689546007	0.490479732	0.650039512
ENSG00000233875	7.403079503	0.321971048	0.657652649	0.489576143	0.624433862	0.756091928
ENSG00000233901	21.43390136	-0.221483086	0.385898463	-0.573941352	0.566007489	0.711600035
ENSG00000233913	448.6724443	0.353340088	0.290772565	1.215176845	0.22429865	0.382628019
ENSG00000233922	11.94426557	-0.549858915	0.509254497	-1.079733058	0.280261068	0.449067691
ENSG00000233927	622.1771158	0.21483809	0.247146257	0.869275113	0.384696672	0.553785012
ENSG00000233937	24.35906692	0.162473299	0.347374404	0.467718109	0.639986189	0.767565619
ENSG00000233954	67.62107659	0.403133642	0.236220078	1.706601933	0.087896066	0.189716662
ENSG00000233966	10.81847622	0.48261019	0.541552694	0.89116017	0.372843249	0.543291402
ENSG00000233967	34.04298567	-0.076753658	0.306681393	-0.250271649	0.80237728	0.882476797
ENSG00000234031	7.216365931	-0.179453453	0.667999032	-0.268643282	0.788204202	0.873316329
ENSG00000234072	24.6868722	-0.51718017	0.355115321	-1.456372452	0.145289687	0.278428953
ENSG00000234127	399.3227445	0.263697326	0.097007376	2.718322448	0.006561386	0.022515788
ENSG00000234129	12.5181102	-0.787586745	0.497740037	-1.582325483	0.113575286	0.231551821
ENSG00000234147	33.02803979	-0.956257636	0.314719254	-3.038446567	0.002378013	0.009465555
ENSG00000234160	9.934835093	0.170762695	0.561154618	0.304305961	0.760894804	0.855550842
ENSG00000234171	163.7277367	-0.65798008	0.145306361	-4.528226259	5.95E-06	4.49E-05
ENSG00000234176	17.27904805	0.059407701	0.419102798	0.141749713	0.887277706	0.93824263
ENSG00000234219	12.72216645	0.182673403	0.48066129	0.380046004	0.703911266	0.814364144
ENSG00000234284	23.2984965	-0.430652929	0.357749348	-1.203783964	0.228673089	0.387522124
ENSG00000234297	16.04057287	0.225114654	0.435016359	0.517485491	0.604817295	0.741997252
ENSG00000234327	49.14208004	-0.01989917	0.248207032	-0.080171662	0.936100728	0.964881635
ENSG00000234329	16.48521928	-0.414717566	0.435989034	-0.951211003	0.341497274	0.513488783
ENSG00000234373	28.85628101	-0.200626659	0.332547399	-0.603302444	0.546307507	0.694729455
ENSG00000234420	883.6848178	0.02192989	0.076904104	0.285158899	0.775522407	0.86552026
ENSG00000234432	12.29551542	-0.150462329	0.492414025	-0.305560608	0.759939217	0.855152302
ENSG00000234473	18.28827586	-0.421680915	0.41248732	-1.02228819	0.306644521	0.477595697
ENSG00000234497	4.423919851	-5.425404171	1.349370511	-4.020692705	5.80E-05	0.000351189
ENSG00000234518	9.975678831	0.974524971	0.571796322	1.704321859	0.088320973	0.190428966
ENSG00000234545	123.4528829	0.185478392	0.16395734	1.131260065	0.257945648	0.42358692
ENSG00000234602	24.83662023	1.673012979	0.374696137	4.464985929	8.01E-06	5.87E-05
ENSG00000234608	41.24234203	0.527487966	0.28649156	1.841198971	0.065592406	0.151218863
ENSG00000234616	234.175805	-0.052723626	0.128071475	-0.41167345	0.680578787	0.797671275
ENSG00000234617	20.41459157	-0.70629043	0.422238426	-1.67272893	0.094380671	0.200539297

ENSG00000234648	17.01023736	0.089540145	0.436073287	0.205332791	0.837312127	0.905074151
ENSG00000234665	14.9017137	-0.027398671	0.453773253	-0.060379651	0.951853265	0.973475269
ENSG00000234684	29.24500676	-1.129138873	0.366169528	-3.083650573	0.002044776	0.008320675
ENSG00000234705	11.97000729	-0.426329913	0.509968151	-0.835993213	0.403158721	0.571460198
ENSG00000234709	14.88704462	-0.076143442	0.453593747	-0.167867045	0.866687876	0.925244308
ENSG00000234737	19.49466678	-0.052245064	0.385909147	-0.13538177	0.892310036	0.941285707
ENSG00000234740	10.15236431	-0.573627742	0.553523675	-1.036320157	0.300052805	0.470406446
ENSG00000234741	2254.429135	0.351242576	0.058685725	5.985145057	2.16E-09	2.90E-08
ENSG00000234745	725.3108767	1.490720996	0.197920194	7.531929756	5.00E-14	1.13E-12
ENSG00000234753	19.20698176	0.989674218	0.407657612	2.427709403	0.015194512	0.046009337
ENSG00000234771	170.3857489	-0.058507777	0.161369878	-0.36256938	0.716926587	0.823776505
ENSG00000234782	13.67468951	0.172050728	0.474779136	0.362380558	0.717067666	0.823803836
ENSG00000234788	10.92924043	-0.336527777	0.522435567	-0.644151735	0.519477045	0.674158727
ENSG00000234851	15.23651044	0.287388648	0.451662131	0.636291218	0.524586628	0.677908773
ENSG00000234886	9.459953755	-0.276706429	0.577528037	-0.479122072	0.631851789	0.761725204
ENSG00000234911	8.239800509	-0.226276463	0.597506155	-0.378701477	0.704909557	0.815025133
ENSG00000234912	26.6697564	0.134259468	0.342697413	0.391772634	0.695226217	0.808564553
ENSG00000234913	6.774019253	1.192747992	0.718464153	1.660135703	0.096887156	0.204501687
ENSG00000234996	10.22322302	-0.313910009	0.557357847	-0.56321089	0.573291288	0.717878752
ENSG00000235034	34.51598952	0.005214385	0.296884953	0.017563657	0.98598695	0.992629448
ENSG00000235072	15.86008634	-0.41563091	0.442298936	-0.939705878	0.347368449	0.519221095
ENSG00000235082	10.22266807	-0.335522444	0.536337209	-0.625581143	0.53158971	0.683312652
ENSG00000235092	32.50141304	1.097412831	0.312554526	3.511108426	0.000446242	0.002190205
ENSG00000235106	214.0572746	-0.452833962	0.132400892	-3.420173052	0.000625813	0.002958868
ENSG00000235109	23.42071721	-0.622278002	0.371623371	-1.674485652	0.094035192	0.199926191
ENSG00000235162	150.0192709	-0.146280389	0.147086581	-0.994518929	0.319970295	0.49159168
ENSG00000235169	86.95449189	-0.489189021	0.190839918	-2.563347466	0.010366824	0.033309877
ENSG00000235173	216.8336272	0.420410122	0.132814618	3.165390438	0.00154875	0.006564661
ENSG00000235194	49.99783821	-0.011145201	0.250747347	-0.044447931	0.964547356	0.981383315
ENSG00000235217	37.12902908	-0.389906913	0.290181603	-1.34366517	0.179056685	0.324908693
ENSG00000235225	15.58680419	-0.394044807	0.452671355	-0.870487612	0.384033986	0.553227915
ENSG00000235244	74.54538769	-0.00779559	0.204286634	-0.038160059	0.969560066	0.984132542
ENSG00000235257	38.17754239	-0.230189796	0.283634306	-0.811572476	0.41703699	0.58499287
ENSG00000235272	21.76001278	-0.20304827	0.403091115	-0.503727971	0.61445255	0.749042347
ENSG00000235279	17.87481602	-0.940489323	0.426431592	-2.205486981	0.02741994	0.074833476
ENSG00000235316	28.07075344	0.196218215	0.329012836	0.596384679	0.550918273	0.698621617
ENSG00000235381	27.44691367	-0.083470028	0.330371237	-0.252655252	0.80053463	0.880976166
ENSG00000235408	15.78667111	-0.131090559	0.452130435	-0.289939692	0.771862375	0.86337203
ENSG00000235411	19.99929761	-0.971116437	0.412146	-2.356243754	0.018460799	0.054092376
ENSG00000235437	402.0570301	-0.143067427	0.102331468	-1.398078514	0.162089491	0.301932248
ENSG00000235453	32.93874332	-0.048862859	0.303280014	-0.161114668	0.872003088	0.928844619
ENSG00000235501	39.23425172	0.034174112	0.278292243	0.122799368	0.902265974	0.947258155
ENSG00000235508	40.15411154	0.068125031	0.279513648	0.243727028	0.80744224	0.886174009
ENSG00000235529	18.98006123	0.713479612	0.412558118	1.729403883	0.083736836	0.183102635
ENSG00000235530	12.54705693	0.237504278	0.509562855	0.466094174	0.641148093	0.768299941
ENSG00000235552	15.70818143	-0.010946127	0.436693473	-0.025065928	0.980002377	0.989867936
ENSG00000235559	18.4017013	0.029747758	0.455726015	0.065275532	0.947954624	0.971185078
ENSG00000235587	11.79134918	0.404098237	0.511586774	0.789891877	0.429590916	0.596349797
ENSG00000235613	26.82366187	-0.279321193	0.338478771	-0.825225145	0.409243781	0.577637398
ENSG00000235616	15.02454379	-6.213911022	1.231228814	-5.046918127	4.49E-07	4.26E-06

ENSG00000235652	10.60384281	-0.477725315	0.539831903	-0.884951986	0.376182528	0.546004956
ENSG00000235655	15.86080647	-0.128669622	0.444849297	-0.289243172	0.772395297	0.863565484
ENSG00000235683	9.291809596	-0.385464772	0.568031146	-0.678597951	0.497392641	0.655493567
ENSG00000235701	8.256904573	-0.708001177	0.614013964	-1.153070156	0.24888159	0.413038861
ENSG00000235703	51.6639064	-0.282247153	0.247358739	-1.141043788	0.253851701	0.418674985
ENSG00000235706	10.32796367	-1.234255692	0.572022098	-2.157706312	0.030950673	0.082587347
ENSG00000235748	24.80380174	0.020954154	0.343790785	0.060950308	0.951398784	0.973382375
ENSG00000235750	11.25109947	0.397947974	0.542821466	0.733110238	0.463491196	0.626567302
ENSG00000235786	13.28839208	-0.185234595	0.489604598	-0.37833508	0.705181688	0.815025133
ENSG00000235823	226.739812	-0.568638941	0.139028543	-4.090087761	4.31E-05	0.000269209
ENSG00000235859	34.98738436	-1.342884042	0.347012734	-3.869840817	0.000108906	0.000621873
ENSG00000235890	59.94507083	0.185797035	0.230134301	0.807341772	0.419469604	0.587278278
ENSG00000235917	19.6770943	0.223244677	0.390710749	0.571380946	0.567741442	0.713205512
ENSG00000235920	26.26991075	-1.472187135	0.367541948	-4.00549418	6.19E-05	0.000372947
ENSG00000235954	38.539838	-0.305860451	0.291202991	-1.05033417	0.2935645	0.463987641
ENSG00000235961	24.97302009	1.238630262	0.356964198	3.469900533	0.000520651	0.002511534
ENSG00000235994	9.715179572	-0.291264843	0.553956018	-0.525790556	0.599033737	0.737672211
ENSG00000236017	20.17188943	0.230204454	0.422053472	0.545439072	0.585451574	0.727328526
ENSG00000236081	25.64326015	6.410967306	1.062884171	6.031670693	1.62E-09	2.20E-08
ENSG00000236088	53.25149807	0.036685688	0.246493773	0.148830078	0.881687716	0.934792614
ENSG00000236104	94.7701326	-0.096135472	0.189519622	-0.507258676	0.611973329	0.747707172
ENSG00000236144	109.8879234	-0.338026717	0.177970014	-1.89934646	0.057518938	0.135998741
ENSG00000236200	9.168095621	-0.502884779	0.568731667	-0.884221521	0.376576643	0.546267833
ENSG00000236254	8.696641054	-0.432307286	0.583425507	-0.740981121	0.458704887	0.622312879
ENSG00000236255	57.1743964	-0.272815329	0.237349355	-1.149425193	0.250380697	0.414458832
ENSG00000236287	242.2681705	-0.202337204	0.121499664	-1.665331378	0.095846662	0.203070597
ENSG00000236333	13.9883545	-3.403133804	0.674578567	-5.044829428	4.54E-07	4.30E-06
ENSG00000236352	16.83060264	-1.534743108	0.452748102	-3.389838858	0.000699337	0.003267994
ENSG00000236397	7.521816877	0.745272572	0.640879474	1.162890377	0.244873982	0.408278318
ENSG00000236423	10.22538874	0.443537905	0.551652908	0.804016254	0.421387588	0.588978253
ENSG00000236432	25.51054829	-0.376545694	0.357763651	-1.052498468	0.292570913	0.46317408
ENSG00000236440	7.070167302	-0.168781328	0.662465112	-0.254777686	0.798894811	0.880369923
ENSG00000236449	13.72369332	1.938907121	0.511474885	3.790815894	0.000150153	0.000828763
ENSG00000236451	8.119685989	-2.732473249	0.76578217	-3.568212159	0.000359425	0.00180574
ENSG00000236456	14.30045239	-0.773561283	0.516188488	-1.498602353	0.133976822	0.262478719
ENSG00000236519	12.96717302	0.358473555	0.485630841	0.738160604	0.46041686	0.623671709
ENSG00000236552	55.18150016	0.039177243	0.240294937	0.163038156	0.870488392	0.927945136
ENSG00000236609	82.60734175	-0.661617413	0.198103711	-3.339752753	0.00083853	0.003839254
ENSG00000236618	45.54055915	-0.38925424	0.268823636	-1.447991128	0.147619532	0.281817282
ENSG00000236651	10.04363613	-0.808831152	0.555805946	-1.455240193	0.145602777	0.278876767
ENSG00000236671	22.13228942	-2.516306865	0.452683222	-5.558648394	2.72E-08	3.13E-07
ENSG00000236682	11.46202125	-0.056036659	0.520195339	-0.107722339	0.914215949	0.953636867
ENSG00000236711	10.65071385	0.095664332	0.519242376	0.184238299	0.853826516	0.916179136
ENSG00000236753	23.7775739	0.080575779	0.351901431	0.228972581	0.818890227	0.893294325
ENSG00000236778	11.01522681	-0.109657058	0.536865025	-0.204254427	0.838154681	0.905588709
ENSG00000236780	14.9141298	-3.076360887	0.607478739	-5.064145772	4.10E-07	3.93E-06
ENSG00000236810	10.65133949	0.762375357	0.557792126	1.36677325	0.17169639	0.314824216
ENSG00000236859	21.69156654	0.258601179	0.369492178	0.699882688	0.48400057	0.644492938
ENSG00000236871	8.276153209	-0.007981742	0.596824834	-0.013373675	0.989329669	0.994711464
ENSG00000236901	33.83068499	-1.03751071	0.318089041	-3.261699014	0.001107467	0.004916746

ENSG00000236914	7.983679726	-0.151985256	0.634902273	-0.239383701	0.810808067	0.888548987
ENSG00000236924	18.77723595	0.184186566	0.391904339	0.469978379	0.638370465	0.766295498
ENSG00000236956	11.35382386	0.080156367	0.507915156	0.157814481	0.874602988	0.930511866
ENSG00000237004	20.7833539	-0.450447142	0.394697029	-1.141247866	0.25376679	0.418674985
ENSG00000237036	12.72496149	-0.151512813	0.478157633	-0.316867916	0.751343828	0.8494267
ENSG00000237037	21.111008	-0.110337046	0.380958671	-0.289629965	0.772099341	0.86337203
ENSG00000237125	97.11351891	5.419865655	0.402898537	13.45218501	2.99E-41	2.46E-39
ENSG00000237172	16.28908673	1.14303372	0.446677793	2.558966974	0.010498371	0.033579485
ENSG00000237187	504.8887839	-0.025274908	0.099745874	-0.253393016	0.799964524	0.880924836
ENSG00000237190	158.9167067	0.013959332	0.148909192	0.093743923	0.925312578	0.959110521
ENSG00000237280	26.74216066	-0.115603294	0.343551272	-0.336495024	0.736497603	0.837902473
ENSG00000237289	25.38940518	-1.840543258	0.384984534	-4.780823893	1.75E-06	1.47E-05
ENSG00000237298	130.4548794	-0.464013425	0.163742482	-2.833799882	0.004599813	0.016687389
ENSG00000237356	19.45383492	-0.238045617	0.395867472	-0.601326541	0.547622515	0.695834778
ENSG00000237361	34.49733893	-0.514641199	0.312176558	-1.64855812	0.099238195	0.208464023
ENSG00000237399	9.886803403	0.125660683	0.594073354	0.21152385	0.832478524	0.902022973
ENSG00000237429	9.047383638	0.357157103	0.577525822	0.618426205	0.536294428	0.687065174
ENSG00000237441	225.9648522	-0.300705241	0.12893959	-2.33214051	0.019693301	0.057061013
ENSG00000237457	7.251422108	-2.895813708	0.85417798	-3.390176023	0.000698478	0.003265063
ENSG00000237461	11.37654431	-1.088249066	0.529820706	-2.053994971	0.039976178	0.101767839
ENSG00000237491	21.69929398	1.339440066	0.397546318	3.369267948	0.000753681	0.003487141
ENSG00000237493	8.702365602	-0.218994111	0.581780546	-0.376420477	0.706604325	0.815786221
ENSG00000237505	4.8632648	-4.51094361	1.334947494	-3.379116878	0.000727191	0.003381281
ENSG00000237523	19.22049172	0.757574147	0.396465502	1.91081984	0.056027737	0.133348293
ENSG00000237638	8.569224895	-0.664667438	0.590390256	-1.125810312	0.260245818	0.426417008
ENSG00000237649	384.9408896	-0.013026112	0.112138855	-0.116160558	0.907525295	0.950634438
ENSG00000237748	11.22071855	-0.486019451	0.516595092	-0.94081314	0.346800626	0.518797486
ENSG00000237765	21.05814862	-0.39328728	0.385354602	-1.020585399	0.30745091	0.478533289
ENSG00000237877	38.16302099	-1.118199967	0.295630482	-3.782424466	0.000155308	0.000854865
ENSG00000237883	6.341352552	-1.757345713	0.749430558	-2.344908003	0.019031775	0.055430094
ENSG00000237886	21.52663104	0.229692325	0.383352765	0.599166997	0.549061528	0.69718384
ENSG00000237973	114.3711812	-0.169841527	0.172979187	-0.98186105	0.326168307	0.498318638
ENSG00000237975	75.37178106	-2.438844578	0.253545262	-9.618971214	6.65E-22	2.49E-20
ENSG00000237976	9.287082741	-0.211372661	0.605104033	-0.349316232	0.726851914	0.831258753
ENSG00000237984	17.96745564	-0.379311207	0.436878282	-0.868230861	0.385267963	0.554220656
ENSG00000238045	8.838545387	-0.974716388	0.614049262	-1.587358617	0.112431446	0.229853664
ENSG00000238058	17.21417552	0.177182348	0.438041108	0.404487947	0.685853939	0.801843308
ENSG00000238083	27.12581999	0.16130538	0.338961349	0.475881336	0.63415892	0.763476761
ENSG00000238113	13.11627324	0.842493672	0.494052766	1.70527063	0.088143962	0.190105672
ENSG00000238227	173.0696283	0.415808718	0.175433758	2.370175065	0.017779664	0.052468686
ENSG00000238554	142.0938779	-0.067872261	0.17954016	-0.378033865	0.705405437	0.81513904
ENSG00000238578	15.48182593	0.214678533	0.456583009	0.470185111	0.63822277	0.766295498
ENSG00000238741	1636.355118	-0.297315825	0.163928156	-1.813695898	0.069724557	0.158584983
ENSG00000238825	23.01705098	0.85859717	0.364141678	2.357865694	0.018380342	0.053894691
ENSG00000238835	8.239029162	-3.110788472	0.82100261	-3.789011674	0.000151248	0.000834477
ENSG00000238961	132.2418217	-0.105314751	0.188592053	-0.558426237	0.576553361	0.720601927
ENSG00000239002	34.04578566	0.106756945	0.35352815	0.301975797	0.762670513	0.856852972
ENSG00000239039	25.83058029	0.10215935	0.372955027	0.273918683	0.784147114	0.871011269
ENSG00000239213	10.48089578	-0.760035799	0.540631079	-1.405830756	0.159774387	0.298530302
ENSG00000239218	12.80874091	-0.040135761	0.494659084	-0.081138226	0.935332026	0.964726409

ENSG00000239264	275.3778772	0.316688077	0.117216839	2.701728512	0.006898006	0.02349001
ENSG00000239291	12.98631498	-0.346336728	0.481742211	-0.718925434	0.472186865	0.633869351
ENSG00000239305	95.2200859	-0.231607273	0.182458946	-1.269366491	0.204310382	0.357575049
ENSG00000239306	667.3888922	0.124404637	0.086892595	1.431705846	0.152228023	0.288402768
ENSG00000239382	16.42886185	0.055486185	0.432535066	0.128281356	0.897926319	0.944497571
ENSG00000239407	15.8116752	-0.2859186	0.431547107	-0.662543196	0.507623161	0.664441509
ENSG00000239415	11.79384548	0.001818242	0.524527971	0.003466435	0.99723419	0.998813347
ENSG00000239523	25.48283232	-0.165221834	0.359418016	-0.459692687	0.645736819	0.771840121
ENSG00000239569	59.14989673	0.774119858	0.255577816	3.028900825	0.002454452	0.009736711
ENSG00000239653	52.20467619	0.087320259	0.246336175	0.35447599	0.722982195	0.828279902
ENSG00000239672	424.1330349	0.455976611	0.121767172	3.744659617	0.000180639	0.000977815
ENSG00000239697	22.76552117	0.385039198	0.366537423	1.050477179	0.293498778	0.463987641
ENSG00000239779	15.83202127	-0.062861782	0.431358742	-0.145729705	0.88413477	0.936256398
ENSG00000239789	29.12469856	0.563228049	0.322777488	1.744942166	0.080994937	0.178272622
ENSG00000239857	87.40100247	0.223471206	0.195026036	1.145853193	0.251855901	0.416459075
ENSG00000239883	24.19485514	-0.497973911	0.351653843	-1.416091195	0.156748806	0.294561936
ENSG00000239887	9.594587267	0.06014829	0.555928424	0.108194305	0.913841562	0.953578158
ENSG00000239899	115.5961375	-0.97916162	0.208334052	-4.699959569	2.60E-06	2.12E-05
ENSG00000239900	114.4348856	0.248592501	0.168978731	1.471146698	0.141251448	0.272683793
ENSG00000240053	16.64385383	-0.426069657	0.42751381	-0.996621973	0.318948036	0.490460776
ENSG00000240057	7.798708609	-0.956966093	0.640181879	-1.494834711	0.134957585	0.263811466
ENSG00000240065	11.72310852	-0.23590393	0.535926918	-0.44017929	0.659807258	0.782521221
ENSG00000240103	10.90357314	-0.043620621	0.524219318	-0.083210632	0.933684059	0.963875944
ENSG00000240137	16.22508148	-0.55406883	0.44075234	-1.257097876	0.208718196	0.363214136
ENSG00000240184	121.4313739	-1.588238354	0.176752005	-8.985687907	2.57E-19	8.40E-18
ENSG00000240204	20.86650112	0.363994041	0.378544966	0.961560909	0.336270216	0.508131209
ENSG00000240230	221.6614443	-0.10049546	0.132201809	-0.760167056	0.447154734	0.612141365
ENSG00000240270	10.05282255	-0.482424194	0.598237225	-0.80640952	0.420006759	0.587769236
ENSG00000240288	84.40785518	0.024542284	0.208987934	0.117433977	0.906516161	0.950002425
ENSG00000240291	25.52792026	0.227338647	0.355980283	0.638627074	0.523065564	0.67669018
ENSG00000240303	36.68292743	0.017185004	0.292147047	0.05882313	0.953092985	0.974299858
ENSG00000240342	226.1369291	0.285180447	0.125106496	2.279501506	0.022637271	0.063797179
ENSG00000240344	176.0985704	-0.065785936	0.139070269	-0.47304098	0.636183943	0.765174745
ENSG00000240356	14.44881546	0.055137893	0.460532251	0.119726453	0.904699844	0.948877676
ENSG00000240370	28.86835332	-0.257494302	0.332040513	-0.775490617	0.438049808	0.603559162
ENSG00000240376	28.01069479	0.333574098	0.342723445	0.973303994	0.330402238	0.50267088
ENSG00000240409	59.86252819	-0.255103392	0.229755174	-1.110327082	0.266858107	0.43356648
ENSG00000240429	29.21925108	-0.264497905	0.319303824	-0.828358087	0.407467736	0.575837828
ENSG00000240445	59.77651259	-0.272762972	0.233433177	-1.168484172	0.24261152	0.405372193
ENSG00000240489	7.527263781	-0.093082819	0.639454779	-0.145565913	0.884264079	0.936322743
ENSG00000240498	7.708888572	-0.171709451	0.615995554	-0.27875112	0.780435828	0.868545562
ENSG00000240616	11.07555892	0.05737682	0.508119929	0.112919838	0.910094108	0.951381168
ENSG00000240682	60.48223222	0.216502423	0.22626947	0.956834443	0.338650825	0.510520298
ENSG00000240694	12.12011102	0.823886748	0.504443694	1.633258099	0.102414725	0.21372934
ENSG00000240695	37.76979416	-0.04477813	0.289811775	-0.154507629	0.877209497	0.932155367
ENSG00000240764	9.950678306	0.132278671	0.572418416	0.231087378	0.817246921	0.892507031
ENSG00000240771	70.69440955	-0.187724643	0.225128443	-0.833855735	0.404362284	0.572415298
ENSG00000240809	79.77618301	2.81207924	0.251864841	11.16503294	6.05E-29	3.10E-27
ENSG00000240849	98.82458529	-0.275031284	0.184054145	-1.494295521	0.135098395	0.264011089
ENSG00000240857	80.27283859	0.015752585	0.197563299	0.079734368	0.936448525	0.965098491

ENSG00000240859	11.37689866	-0.346407853	0.533738547	-0.649021615	0.516324405	0.671307749
ENSG00000240869	10.46003036	-0.129834671	0.533905293	-0.243179216	0.807866567	0.886465967
ENSG00000240875	9.463476706	-0.451266765	0.616449912	-0.732041251	0.46414339	0.627026563
ENSG00000240889	13.45606829	0.546679213	0.48719406	1.122097452	0.261821007	0.428099137
ENSG00000240950	14.29522615	0.25924728	0.462706721	0.560284234	0.57528558	0.719550918
ENSG00000240972	1482.783946	0.436235749	0.075478287	5.779619115	7.49E-09	9.37E-08
ENSG00000240990	37.77614896	-1.807348902	0.322114637	-5.610887232	2.01E-08	2.37E-07
ENSG00000241015	32.33797656	0.4554832	0.327615333	1.390298786	0.164438166	0.305416227
ENSG00000241058	102.19254	0.32229399	0.183659692	1.754843357	0.079286105	0.175252859
ENSG00000241061	11.56607297	0.044063194	0.539511611	0.081672373	0.934907248	0.964624614
ENSG00000241081	12.44749525	0.622602393	0.492159843	1.265041025	0.205856622	0.359391992
ENSG00000241127	61.29158553	-0.089488049	0.221049112	-0.404833333	0.685600025	0.801750213
ENSG00000241157	7.65279944	-0.024875401	0.633547865	-0.039263649	0.968680188	0.983424201
ENSG00000241258	182.8135049	0.291162594	0.134048741	2.172065115	0.029850752	0.080113395
ENSG00000241288	29.38082648	-0.899400165	0.338380119	-2.657958064	0.007861567	0.026349663
ENSG00000241316	20.68004058	-0.852925096	0.394575569	-2.161626728	0.03064696	0.081968363
ENSG00000241343	89.10577498	-0.100690752	0.187245256	-0.537747947	0.590751073	0.731770751
ENSG00000241360	24.01910603	0.288973364	0.358948233	0.80505582	0.420787469	0.588373639
ENSG00000241370	29.74620152	0.674473562	0.340486517	1.980911218	0.047601231	0.117328389
ENSG00000241399	23.50618797	-0.214293189	0.356221394	-0.601573047	0.547458375	0.695834778
ENSG00000241404	10.72088435	0.553266707	0.547022333	1.011415209	0.311817745	0.483295141
ENSG00000241468	139.6538893	0.559857146	0.152800791	3.663967578	0.000248338	0.001297705
ENSG00000241478	9.62614634	0.556134585	0.55958732	0.993829856	0.320305708	0.491999375
ENSG00000241484	32.27599215	-0.36706869	0.356725788	-1.028993984	0.303482502	0.474358887
ENSG00000241506	15.33140991	0.018295444	0.440971442	0.041488955	0.966906098	0.982787985
ENSG00000241529	54.94698968	-1.258176966	0.256290302	-4.909186791	9.15E-07	8.17E-06
ENSG00000241553	168.1170541	0.133690468	0.161929765	0.825607745	0.409026641	0.577621114
ENSG00000241634	18.2049491	-0.259560199	0.406695517	-0.638217508	0.523332102	0.676910316
ENSG00000241680	12.14139738	1.086994524	0.509827256	2.132083978	0.032999944	0.087051682
ENSG00000241685	54.66717424	-0.018869643	0.244259931	-0.077252305	0.938422833	0.966102042
ENSG00000241764	11.9586318	0.828673952	0.50233731	1.64963648	0.099017309	0.208093378
ENSG00000241772	18.75145638	0.025752437	0.403391358	0.063839834	0.94909776	0.971789214
ENSG00000241837	107.3189785	0.182802855	0.197514589	0.925515708	0.354697717	0.526128704
ENSG00000241839	53.3076346	-0.08356251	0.260440023	-0.320851263	0.748323109	0.846897737
ENSG00000241852	58.95683384	0.217759717	0.232280536	0.937485855	0.348508696	0.520494777
ENSG00000241878	77.08630555	-0.20591112	0.203956451	-1.009583757	0.312694755	0.484076461
ENSG00000241945	56.23971646	0.241756506	0.24971148	0.968143339	0.332972788	0.505268311
ENSG00000241973	682.7068635	0.056721232	0.081774174	0.693632587	0.487912652	0.647922265
ENSG00000242021	14.31538521	-0.427223771	0.466199514	-0.91639686	0.359458782	0.530724985
ENSG00000242028	23.44413428	-0.17718745	0.358480641	-0.494273414	0.621113098	0.754086058
ENSG00000242071	26.40443561	0.360557671	0.349446422	1.031796718	0.302167357	0.472771761
ENSG00000242086	102.6868329	-0.304028628	0.190758929	-1.593784514	0.110984307	0.227590739
ENSG00000242114	27.35628892	-0.006776874	0.343347909	-0.01973763	0.984252672	0.992000554
ENSG00000242125	1022.639113	0.429290207	0.086561172	4.959385337	7.07E-07	6.47E-06
ENSG00000242173	26.63324047	1.505315406	0.370642348	4.061369172	4.88E-05	0.00030136
ENSG00000242193	39.2217761	-0.046019775	0.27761737	-0.165766916	0.868340383	0.926585944
ENSG00000242247	144.9450367	0.127211301	0.154323991	0.824313191	0.409761625	0.578173226
ENSG00000242259	152.980144	-0.038116471	0.157221958	-0.242437322	0.808441318	0.886924024
ENSG00000242299	38.60047167	0.123852955	0.283337982	0.437120904	0.662023668	0.784101725
ENSG00000242372	344.6399077	0.37253165	0.114805073	3.244905811	0.001174895	0.005163876

ENSG00000242474	20.26268855	-0.557904498	0.389616459	-1.43193257	0.15216312	0.288402768
ENSG00000242485	430.7935704	0.17866318	0.101256437	1.764462447	0.077654153	0.172487015
ENSG00000242498	299.6367136	-0.317629301	0.112391263	-2.826103138	0.004711809	0.017023062
ENSG00000242588	353.6577117	0.231723173	0.111511085	2.078028143	0.037706767	0.097115321
ENSG00000242612	61.27600997	-0.062898094	0.231787004	-0.27136161	0.786112926	0.872030977
ENSG00000242616	64.64898947	0.148948605	0.227207769	0.655561233	0.512106476	0.66817385
ENSG00000242622	18.73590038	-0.443467146	0.400177983	-1.108174775	0.267786343	0.434665444
ENSG00000242689	9.866516147	-0.020185381	0.54408593	-0.03709962	0.970405575	0.984539698
ENSG00000242732	144.7237007	0.204467977	0.152918534	1.337103952	0.181188707	0.3278575
ENSG00000242802	124.0269122	0.290960168	0.16716312	1.740576324	0.081757871	0.179754705
ENSG00000242861	10.49325538	0.223602159	0.527959536	0.423521395	0.671914892	0.79081596
ENSG00000243056	23.79280607	0.910789545	0.372072809	2.447879886	0.014369955	0.043879177
ENSG00000243107	6.913450594	0.150587193	0.711595239	0.211619169	0.832404153	0.902011968
ENSG00000243147	544.3147351	0.306291454	0.088920357	3.444559441	0.000571991	0.00272827
ENSG00000243155	22.1080668	-0.646180155	0.373396399	-1.730547368	0.083532526	0.182769687
ENSG00000243156	541.1986087	-0.129212464	0.090263194	-1.431507784	0.152284738	0.288410909
ENSG00000243176	19.73180612	-0.751561441	0.416850235	-1.802953142	0.071395574	0.161470557
ENSG00000243199	16.83547968	0.017134053	0.417560055	0.041033744	0.967268994	0.982880628
ENSG00000243232	61.66952246	0.433553019	0.225450147	1.923054942	0.05447315	0.130481271
ENSG00000243244	34.02417626	-0.003762339	0.304155977	-0.012369767	0.990130606	0.994914727
ENSG00000243279	299.0208101	1.1856662	0.124339114	9.535745939	1.49E-21	5.46E-20
ENSG00000243317	165.6667617	0.593096787	0.151028223	3.927059301	8.60E-05	0.000502878
ENSG00000243335	51.16274412	-0.070346901	0.244456737	-0.287768304	0.773524101	0.864345959
ENSG00000243364	31.95008987	0.233683918	0.30354029	0.769861286	0.441382181	0.60654127
ENSG00000243403	10.30989769	-0.391122003	0.543769463	-0.719279088	0.471968978	0.633818202
ENSG00000243449	115.789327	1.196574779	0.185448334	6.452335006	1.10E-10	1.73E-09
ENSG00000243477	35.4462434	0.41793614	0.30559682	1.367606312	0.17143534	0.314504514
ENSG00000243547	15.49109337	0.226453535	0.456577049	0.495980986	0.619907826	0.753273809
ENSG00000243609	10.92994483	0.549833747	0.51754784	1.062382459	0.288062094	0.458124652
ENSG00000243646	21.44656411	-0.924767021	0.403640902	-2.291063704	0.021959729	0.062279087
ENSG00000243660	16.59101252	-0.363476888	0.452485286	-0.803289962	0.421807159	0.589447389
ENSG00000243667	30.14464514	-0.823962567	0.336284534	-2.450194654	0.0142779	0.043619328
ENSG00000243678	129.5310799	0.363908883	0.183418281	1.98403824	0.047251577	0.116568828
ENSG00000243696	11.7801622	0.547733156	0.512330948	1.069100272	0.285024489	0.454557107
ENSG00000243701	26.46451555	-0.030449285	0.34128312	-0.089220016	0.928907058	0.961275781
ENSG00000243716	150.2454466	0.278993269	0.158385542	1.761481915	0.078156871	0.173384329
ENSG00000243725	85.49064675	0.007143339	0.18971494	0.037653013	0.96996434	0.984394442
ENSG00000243766	53.03189944	-2.606340369	0.306491133	-8.503803503	1.83E-17	5.23E-16
ENSG00000243927	132.6447053	0.229187699	0.177214049	1.29328177	0.195913623	0.347068876
ENSG00000243978	7.082013362	-2.255384588	0.75500731	-2.987235433	0.002815128	0.010938896
ENSG00000243989	14.17231882	0.339622673	0.460828364	0.736983006	0.461132684	0.624460697
ENSG00000244005	242.5417405	0.111197791	0.128800598	0.863332876	0.387954474	0.556567387
ENSG00000244038	1163.327743	0.267484167	0.075558969	3.540071707	0.000400018	0.001984831
ENSG00000244041	15.45456472	0.975002412	0.501505757	1.944149988	0.051877367	0.125490162
ENSG00000244045	74.95541452	0.142055671	0.206429097	0.688157206	0.491353789	0.651075057
ENSG00000244055	19.08034007	0.378066726	0.399906064	0.945388829	0.344460403	0.516231226
ENSG00000244119	12.92274498	-0.414643671	0.503793341	-0.823043176	0.410483441	0.578921777
ENSG00000244125	40.03935904	0.490062069	0.297482423	1.647364789	0.099483088	0.208884745
ENSG00000244165	105.1231861	0.034890706	0.186441475	0.18714026	0.851550663	0.914865313
ENSG00000244187	125.356967	-0.081380425	0.169426702	-0.480328212	0.630994032	0.761038109

ENSG00000244274	32.16953777	0.181788001	0.323481839	0.561972819	0.574134539	0.718419883
ENSG00000244300	81.09109422	0.09526882	0.207702213	0.45867985	0.646464086	0.772319358
ENSG00000244313	22.78336878	0.099748719	0.371860464	0.268242335	0.78851279	0.873546869
ENSG00000244398	15.04028145	-0.422197444	0.44679139	-0.944954296	0.34468221	0.51645344
ENSG00000244405	350.1689052	-0.226443233	0.103826238	-2.180982741	0.029184693	0.078731973
ENSG00000244462	821.0013168	-0.134356783	0.084517177	-1.589697959	0.111902904	0.228906377
ENSG00000244486	103.6769252	0.735984202	0.374625146	1.964588361	0.0494619	0.12085429
ENSG00000244491	11.80402363	-0.481604691	0.52822583	-0.911740138	0.361905529	0.532657897
ENSG00000244509	1020.16507	-0.013329142	0.075835092	-0.175764825	0.860478705	0.920644708
ENSG00000244578	50.67123379	-0.631203655	0.256104296	-2.464635171	0.013715282	0.042157236
ENSG00000244625	24.45205143	-0.022217952	0.367252696	-0.060497724	0.951759229	0.973475269
ENSG00000244642	57.49120238	-1.320134389	0.257890106	-5.118980361	3.07E-07	2.99E-06
ENSG00000244649	22.56402408	-1.004440353	0.373865811	-2.686633342	0.007217613	0.024495244
ENSG00000244675	13.84198936	-0.188784371	0.465445487	-0.405599316	0.685037032	0.801225244
ENSG00000244687	53.61323052	-0.253178164	0.24570313	-1.03042303	0.302811467	0.47346312
ENSG00000244694	11.79971502	-2.757194984	0.648358492	-4.252577882	2.11E-05	0.000141221
ENSG00000244754	877.3716627	0.020234104	0.079951932	0.253078366	0.800207656	0.880924836
ENSG00000244879	311.4356447	-0.399705941	0.118436754	-3.374847139	0.000738567	0.003427371
ENSG00000244921	31.50921214	-0.185344337	0.318612907	-0.581722628	0.560753527	0.707146494
ENSG00000245105	14.25665583	-0.445662992	0.504376287	-0.883592278	0.376916349	0.546505362
ENSG00000245146	10.9455677	0.038795219	0.523794731	0.074065691	0.940958115	0.967262249
ENSG00000245149	16.69266482	0.454674506	0.452241209	1.00538053	0.314713651	0.486120812
ENSG00000245205	22.36552531	0.105858266	0.417164098	0.253756894	0.799683377	0.880893381
ENSG00000245213	13.87596177	-0.210297507	0.47422303	-0.443456969	0.657435238	0.78050987
ENSG00000245248	15.35008159	-0.943062361	0.454561281	-2.074664958	0.038017597	0.097736674
ENSG00000245384	73.58633433	-1.776263306	0.225908954	-7.862739708	3.76E-15	9.10E-14
ENSG00000245498	24.18919915	-0.325319491	0.371399934	-0.875927704	0.381069337	0.550538001
ENSG00000245532	2233.612541	-0.033102931	0.155350998	-0.213084764	0.831260848	0.901477895
ENSG00000245534	9.204357951	-0.402486632	0.579969699	-0.693978725	0.487695551	0.647877758
ENSG00000245556	32.22287599	-0.505616604	0.310105668	-1.630465534	0.103003144	0.214639543
ENSG00000245571	9.638519249	-0.335144685	0.566232889	-0.591884879	0.55392768	0.701056142
ENSG00000245573	20.38748657	-0.722058156	0.389104318	-1.855692993	0.06349733	0.147330714
ENSG00000245685	15.62665696	-0.354510404	0.448284108	-0.790816353	0.429051166	0.595953894
ENSG00000245750	9.749744014	-0.901523662	0.572094502	-1.575829969	0.115064994	0.233909981
ENSG00000245848	127.8706078	-0.025168456	0.169387583	-0.148585011	0.881881101	0.934927082
ENSG00000245849	36.44803561	0.005466847	0.290700333	0.018805782	0.984996041	0.992271901
ENSG00000245857	18.99994068	-2.171545003	0.470553527	-4.614873504	3.93E-06	3.08E-05
ENSG00000245910	706.3354597	0.430208648	0.095056959	4.525798573	6.02E-06	4.54E-05
ENSG00000245937	335.924511	-0.257187996	0.105932851	-2.427839829	0.01518905	0.046002712
ENSG00000245970	11.45921602	-0.350223625	0.533329858	-0.656673576	0.511390828	0.667550131
ENSG00000246067	60.67526603	-0.363853976	0.227626261	-1.598470995	0.109938195	0.225973344
ENSG00000246089	30.50859857	0.114721754	0.316109261	0.362918044	0.716666108	0.823611946
ENSG00000246090	12.08440092	-1.168411494	0.520289722	-2.245693978	0.024723616	0.068621347
ENSG00000246095	19.39763405	5.425571658	0.894057142	6.068484219	1.29E-09	1.77E-08
ENSG00000246174	8.226856654	-0.827682561	0.62898101	-1.315910254	0.188204167	0.336949264
ENSG00000246263	32.31796423	-0.026375012	0.319094411	-0.082655826	0.934125211	0.964118797
ENSG00000246273	25.37119065	-0.339391946	0.362065925	-0.937376102	0.348565129	0.520494777
ENSG00000246334	10.76152338	0.304410144	0.526552811	0.578118924	0.563183832	0.709256167
ENSG00000246560	16.38965081	-0.121885872	0.426648584	-0.28568212	0.775121601	0.865324687
ENSG00000246596	10.38259306	-0.261802925	0.556976017	-0.470043443	0.63832398	0.766295498

ENSG00000246695	41.76833385	-0.570886265	0.270897927	-2.107385137	0.035084209	0.091620726
ENSG00000246705	113.9975274	-0.6557383	0.173319589	-3.78340558	0.000154697	0.000852168
ENSG00000246731	9.016111579	-1.371654045	0.61611075	-2.226310845	0.025993368	0.071650085
ENSG00000246877	13.12260311	0.683408646	0.49439741	1.382306283	0.166877678	0.308722605
ENSG00000246889	26.27058659	0.470896864	0.357493656	1.317217402	0.187765763	0.336292958
ENSG00000246922	56.19956913	0.308331011	0.23702022	1.300863745	0.193305098	0.344079718
ENSG00000246985	34.36942996	-2.451097695	0.381737409	-6.420899905	1.35E-10	2.10E-09
ENSG00000247077	361.4262994	0.399202374	0.115641995	3.45205367	0.000556337	0.002662646
ENSG00000247081	27.8299499	-2.233642769	0.393107734	-5.68201176	1.33E-08	1.61E-07
ENSG00000247092	26.98065839	-0.302808811	0.348799795	-0.868145039	0.385314938	0.554220656
ENSG00000247095	11.43679607	1.142610325	0.553573684	2.064061854	0.039011852	0.099835942
ENSG00000247121	12.12607297	-1.082240809	0.510015926	-2.121974539	0.033839876	0.088916712
ENSG00000247134	49.69750064	-2.960922047	0.33168314	-8.926959782	4.38E-19	1.42E-17
ENSG00000247137	27.78926963	-0.238385504	0.343782719	-0.6934191	0.488046579	0.647922265
ENSG00000247151	9.337101037	-0.488273625	0.572103122	-0.853471351	0.393397968	0.561420427
ENSG00000247240	16.32320636	0.162662744	0.459017827	0.3543713	0.72306064	0.828279902
ENSG00000247271	33.92303833	-0.516808383	0.317792425	-1.626245126	0.10389752	0.215926685
ENSG00000247315	336.9644732	-0.266140878	0.111343185	-2.390275416	0.016835742	0.050157301
ENSG00000247317	9.923760018	0.045956154	0.544431143	0.084411325	0.932729403	0.963270344
ENSG00000247373	18.16459746	0.597759372	0.405649098	1.473587334	0.140592734	0.27166143
ENSG00000247400	10.34651971	-0.885600356	0.544402063	-1.62673953	0.103792429	0.215867235
ENSG00000247516	22.10194311	0.756829658	0.381371724	1.984493374	0.047200866	0.116525583
ENSG00000247556	627.0542869	-0.149543221	0.093164278	-1.605156236	0.108459426	0.22348974
ENSG00000247572	39.77840797	-1.058661439	0.290021375	-3.650287641	0.000261947	0.00136129
ENSG00000247595	20.95725144	0.117121502	0.377149766	0.310543747	0.756147497	0.852524592
ENSG00000247596	178.3571694	0.219344785	0.15257352	1.437633373	0.150538107	0.285912491
ENSG00000247626	71.21268836	0.379875282	0.210971701	1.80059828	0.071766221	0.162021874
ENSG00000247627	2269.419751	-0.490529028	0.08935407	-5.48972226	4.03E-08	4.53E-07
ENSG00000247708	114.9832606	-1.763423787	0.187578584	-9.400986753	5.41E-21	1.94E-19
ENSG00000247735	11.78652954	-0.338453146	0.514786695	-0.657462885	0.510883328	0.66738381
ENSG00000247746	39.89706567	-1.53458501	0.302857484	-5.067020264	4.04E-07	3.88E-06
ENSG00000247796	20.67827274	-0.110260107	0.386880517	-0.284997827	0.775645807	0.86552026
ENSG00000247828	294.6316107	-0.00288939	0.110655087	-0.026111675	0.979168265	0.989381141
ENSG00000247934	9.861840428	0.541478677	0.550164009	0.984213194	0.3250107	0.496998182
ENSG00000247950	9.586102893	-0.920809309	0.566137198	-1.626477315	0.103848155	0.215867235
ENSG00000247982	19.95159957	-0.465902093	0.393628457	-1.18360877	0.236567967	0.39772109
ENSG00000248008	112.9546936	0.037780091	0.170410006	0.221701129	0.824546551	0.897443469
ENSG00000248015	19.49014409	-0.905520932	0.411646174	-2.199755492	0.027824247	0.075789646
ENSG00000248019	11.96865	-0.093935841	0.508305078	-0.184802091	0.853384268	0.916032577
ENSG00000248049	83.60135805	0.203549041	0.190590825	1.067989715	0.285525151	0.455148865
ENSG00000248079	12.54317328	-1.078062438	0.515470051	-2.091416243	0.036490767	0.094660353
ENSG00000248092	110.5784312	-0.073815784	0.170362261	-0.433287183	0.664806148	0.785659558
ENSG00000248098	30.09651128	0.480795647	0.318175426	1.51110239	0.130762366	0.257830759
ENSG00000248099	16.97718267	-0.791778345	0.437660196	-1.809116645	0.070432884	0.159807901
ENSG00000248103	72.6527376	0.016426005	0.213488559	0.076940914	0.938670549	0.966112632
ENSG00000248206	16.74571462	0.577943131	0.425668409	1.357730852	0.174549103	0.318924461
ENSG00000248238	20.49683466	0.529656472	0.391766432	1.351970023	0.176384905	0.321359113
ENSG00000248275	47.58594274	-0.197527087	0.256840083	-0.769066437	0.441853871	0.607014311
ENSG00000248283	8.451280415	-0.431197426	0.604361088	-0.713476488	0.475550957	0.636560348
ENSG00000248329	29.87459978	-7.212563153	1.206409783	-5.978535033	2.25E-09	3.01E-08

ENSG00000248333	326.5764694	0.204024981	0.114746273	1.778053223	0.075395114	0.168187259
ENSG00000248370	55.24317826	-0.08117095	0.247774116	-0.327600605	0.743213664	0.842922019
ENSG00000248383	18.93974595	-0.91869574	0.424806615	-2.162621081	0.030570335	0.081794572
ENSG00000248429	43.84688718	2.22019848	0.304346959	7.294958644	2.99E-13	6.25E-12
ENSG00000248449	13.30502061	0.292191132	0.468952288	0.623072196	0.53323708	0.684615781
ENSG00000248483	59.33135761	-0.230371686	0.226705234	-1.016172772	0.30954712	0.480836934
ENSG00000248487	231.1224822	0.692428722	0.129844013	5.33277359	9.67E-08	1.02E-06
ENSG00000248489	20.73065987	-0.280351656	0.401397924	-0.698438231	0.484903174	0.645388942
ENSG00000248508	15.33728766	-0.712815622	0.453089476	-1.573233677	0.11566472	0.23461983
ENSG00000248515	30.73128099	-0.20616789	0.357582124	-0.576560952	0.564236084	0.710326606
ENSG00000248521	9.148412376	-0.8674271	0.579821975	-1.496023153	0.134647622	0.26335215
ENSG00000248527	29415.34893	-0.300433454	0.073903065	-4.065236731	4.80E-05	0.000296928
ENSG00000248538	32.71002423	-1.232320391	0.326737264	-3.771594269	0.000162208	0.000886934
ENSG00000248605	13.32251721	7.31083652	1.253261498	5.833448591	5.43E-09	6.91E-08
ENSG00000248641	10.21647352	0.010475259	0.537260711	0.019497535	0.984444204	0.992000554
ENSG00000248643	10.67873781	0.518960973	0.525919413	0.986769	0.323755895	0.495510902
ENSG00000248746	175.6646959	-2.543236612	0.170301686	-14.93371368	1.99E-50	2.12E-48
ENSG00000248796	15.10180605	0.677766449	0.462462625	1.465559405	0.142768357	0.274909817
ENSG00000248837	42.9362682	2.811205804	0.336678441	8.349824232	6.84E-17	1.87E-15
ENSG00000248866	9.47370194	-0.424073834	0.556287355	-0.762328731	0.44586383	0.610790835
ENSG00000248869	18.5202597	1.885429112	0.438961908	4.295199831	1.75E-05	0.000118784
ENSG00000248923	301.80732	-0.491228544	0.13057019	-3.762179904	0.000168439	0.000916722
ENSG00000248932	38.33458593	-1.392046823	0.306939521	-4.53524792	5.75E-06	4.36E-05
ENSG00000248971	20.53195397	-0.192225431	0.377549392	-0.509139823	0.61065422	0.746810014
ENSG00000249042	35.66833429	-0.317712029	0.293562602	-1.082263297	0.27913555	0.447809167
ENSG00000249087	24.79883235	-0.234338633	0.352766522	-0.664288186	0.506505881	0.663949991
ENSG00000249115	154.1260912	-0.187943264	0.146935841	-1.279083865	0.200867525	0.353228107
ENSG00000249119	87.41182945	0.42074071	0.204965354	2.052740633	0.040097739	0.10198315
ENSG00000249158	18.09090785	-0.241836319	0.425143803	-0.568834163	0.569468688	0.714810407
ENSG00000249242	16.09955125	-0.886784002	0.441805042	-2.007183977	0.044730077	0.111701937
ENSG00000249258	15.9395653	-0.525616936	0.431018821	-1.219475604	0.22266373	0.380876451
ENSG00000249264	16.25627198	-0.473845377	0.445387788	-1.063893961	0.287376739	0.457321973
ENSG00000249348	17.08599428	-0.286077914	0.433608507	-0.659760842	0.509407316	0.665836024
ENSG00000249353	34.99505823	-0.3721387	0.294411675	-1.264007959	0.20622717	0.359725948
ENSG00000249378	42.69670539	-0.889803345	0.272265185	-3.268149562	0.001082531	0.00482064
ENSG00000249464	18.06921527	-4.634395414	0.812980993	-5.700496635	1.19E-08	1.45E-07
ENSG00000249471	43.42077237	0.354807928	0.276275629	1.284253442	0.199053291	0.350872328
ENSG00000249476	31.53162429	0.013065394	0.319678176	0.040870459	0.967399168	0.982880628
ENSG00000249513	10.15201846	1.063628227	0.562293788	1.891588081	0.058545885	0.13793929
ENSG00000249550	57.86614738	-2.36300534	0.277631447	-8.511302899	1.72E-17	4.94E-16
ENSG00000249565	19.30711527	0.039293887	0.404625821	0.097111664	0.922637718	0.957326934
ENSG00000249572	9.762829655	-0.1933348	0.549685301	-0.351719064	0.725048962	0.829715908
ENSG00000249592	100.93984	0.233642875	0.181254269	1.289033778	0.197386344	0.348853293
ENSG00000249661	20.24024735	-0.140986941	0.398965824	-0.353380999	0.723802829	0.82889763
ENSG00000249669	6.481372167	-2.350155819	0.817481475	-2.874873488	0.004041894	0.014941181
ENSG00000249673	86.26479377	-0.141058742	0.193732397	-0.72811127	0.466545475	0.628880558
ENSG00000249776	22.01039337	-1.16068656	0.387117733	-2.998277944	0.002715099	0.010588284
ENSG00000249780	27.08390298	-0.700729258	0.344628915	-2.033286317	0.042023605	0.106476475
ENSG00000249803	10.29557741	6.957149242	1.267010354	5.49099636	4.00E-08	4.50E-07
ENSG00000249850	24.18715188	-0.322228705	0.360789984	-0.893119873	0.371792982	0.542492574

ENSG00000249855	17.12056865	0.195709812	0.41062455	0.476614982	0.633636313	0.763121272
ENSG00000249859	852.1739206	-0.093165064	0.100378635	-0.928136395	0.353336823	0.52460864
ENSG00000249863	11.49018073	-0.506649388	0.510977023	-0.991530667	0.321426525	0.493181693
ENSG00000249898	13.43394345	-1.521586126	0.509743096	-2.985005858	0.002835729	0.011003725
ENSG00000249915	41.29449676	-0.58157906	0.280697261	-2.071908565	0.038273969	0.0982925
ENSG00000249936	8.876792616	0.28224877	0.572049078	0.493399571	0.621730285	0.754639706
ENSG00000249984	73.27629872	-4.455486756	0.380534201	-11.70850541	1.15E-31	6.61E-30
ENSG00000249992	171.7337898	0.11396937	0.142996477	0.797008239	0.425446247	0.592704892
ENSG00000250033	8.521672763	-3.446213957	0.877216874	-3.928576913	8.55E-05	0.000500758
ENSG00000250049	11.48326951	-4.888639155	1.082119349	-4.517652475	6.25E-06	4.69E-05
ENSG00000250067	62.54035195	0.149719547	0.234209517	0.639254751	0.522657219	0.67653575
ENSG00000250069	13.1555327	-0.388028151	0.4968943	-0.780906826	0.434857297	0.601106126
ENSG00000250091	99.68622907	0.107987555	0.17556493	0.615086139	0.538497835	0.689165369
ENSG00000250130	10.66123594	-0.371920773	0.553674495	-0.671731813	0.501754445	0.660124069
ENSG00000250131	11.64266764	-1.959070663	0.583378495	-3.358146865	0.000784669	0.003613851
ENSG00000250132	10.76295766	0.93244243	0.531087143	1.755723976	0.079135551	0.174947605
ENSG00000250133	52.86630526	-1.08382417	0.263093601	-4.11953832	3.80E-05	0.000239887
ENSG00000250159	32.49231849	-0.290026212	0.306483318	-0.946303419	0.34399385	0.515807175
ENSG00000250182	33.68446369	0.410302824	0.299768488	1.368732339	0.171082958	0.314061985
ENSG00000250241	132.9421996	1.036739847	0.161111005	6.434941211	1.24E-10	1.93E-09
ENSG00000250251	13.93862332	0.44380935	0.463465402	0.957588957	0.33827007	0.510265437
ENSG00000250280	123.2171958	-2.262911296	0.202295769	-11.18615236	4.77E-29	2.47E-27
ENSG00000250317	96.10055188	-0.200681175	0.182583513	-1.099119915	0.271715764	0.439469354
ENSG00000250332	12.06520979	-0.196524209	0.499860289	-0.393158276	0.694202584	0.807842242
ENSG00000250337	326.8846073	-0.535375994	0.111805943	-4.788439486	1.68E-06	1.42E-05
ENSG00000250479	1712.839138	0.38036013	0.066956422	5.680711721	1.34E-08	1.62E-07
ENSG00000250508	27.09596841	1.057452183	0.340059454	3.10960972	0.001873347	0.007739678
ENSG00000250510	57.301281	0.685241651	0.238018249	2.878945847	0.003990068	0.014779876
ENSG00000250539	24.80083327	-0.701758281	0.368368122	-1.905046171	0.05677407	0.134740606
ENSG00000250565	175.2842094	-0.223572613	0.146387713	-1.527263512	0.126695499	0.251884299
ENSG00000250568	16.58032859	0.464471056	0.422839672	1.098456666	0.27200513	0.439734844
ENSG00000250571	81.27317636	-0.156757704	0.196726703	-0.796829827	0.42554987	0.592790462
ENSG00000250722	322.9525605	1.051755907	0.11995523	8.767903701	1.82E-18	5.71E-17
ENSG00000250742	38.64649156	0.934383435	0.291236165	3.208335872	0.001335055	0.005773866
ENSG00000250802	90.92782023	-0.620301078	0.194106138	-3.195679878	0.001395018	0.00600362
ENSG00000250838	10.17984166	0.396232329	0.537595326	0.73704571	0.461094552	0.624460697
ENSG00000250899	60.00884668	-0.182512267	0.229433378	-0.795491346	0.426327743	0.593579721
ENSG00000250903	69.50775359	-0.922489482	0.223448727	-4.128416818	3.65E-05	0.000231645
ENSG00000250938	7.491577647	-2.540276085	0.783976259	-3.240246187	0.001194265	0.005235907
ENSG00000250982	27.27404649	0.34048965	0.341548833	0.996898882	0.318813594	0.490350877
ENSG00000250988	13.73401868	0.226821226	0.482595485	0.470002792	0.638353022	0.766295498
ENSG00000251022	126.7639116	-0.261873517	0.164525879	-1.591685866	0.111455306	0.228256696
ENSG00000251023	16.35269617	-0.525757935	0.432265086	-1.216285914	0.223876026	0.382164648
ENSG00000251033	25.94630292	2.199356892	0.395530352	5.560526213	2.69E-08	3.10E-07
ENSG00000251095	442.5851267	-4.108304296	0.1511904	-27.17305002	1.35E-162	7.92E-160
ENSG00000251127	26.68252928	0.58671978	0.341979385	1.715658328	0.086224596	0.187143263
ENSG00000251129	216.3488094	1.753349633	0.138733764	12.63823296	1.30E-36	8.90E-35
ENSG00000251136	124.5886079	-0.494349814	0.170375272	-2.901534993	0.003713393	0.013858138
ENSG00000251141	321.0671589	-4.611126221	0.195462253	-23.59087835	4.78E-123	1.64E-120
ENSG00000251143	9.01184518	0.610335264	0.575584315	1.060375079	0.288973995	0.459187524

ENSG00000251151	33.17511775	-0.40349576	0.303480592	-1.32956034	0.183663173	0.330989821
ENSG00000251161	10.6349964	-0.054829407	0.538100471	-0.101894368	0.91884052	0.955433621
ENSG00000251192	23.62323381	-0.487625079	0.426715258	-1.142741138	0.253146084	0.417903755
ENSG00000251199	6.938179464	-1.355619525	0.692969591	-1.95624677	0.050436091	0.122682436
ENSG00000251244	12.68361975	-2.462757954	0.584839013	-4.211001491	2.54E-05	0.000167354
ENSG00000251279	15.67514911	-0.103651789	0.443545308	-0.233689291	0.815226202	0.891560379
ENSG00000251322	235.3610815	-0.2614926	0.123772945	-2.112679792	0.034628184	0.090632029
ENSG00000251340	9.612370256	-0.156663874	0.553150304	-0.283221166	0.777007304	0.866308565
ENSG00000251348	20.8287314	-0.94465217	0.388948445	-2.428733632	0.01515166	0.045916146
ENSG00000251364	19.56657386	-1.087268936	0.424880817	-2.558997474	0.01049745	0.033579485
ENSG00000251369	98.67019367	0.594990774	0.1782113	3.338681525	0.00084177	0.003851578
ENSG00000251381	354.9776486	0.190461214	0.102873818	1.8514061	0.064111152	0.148510778
ENSG00000251429	8.912991732	0.76039665	0.605148907	1.256544697	0.208918551	0.363485066
ENSG00000251432	27.69024603	-0.313827297	0.336298538	-0.933180676	0.35072669	0.522500564
ENSG00000251485	20.75874491	-0.198182792	0.399350466	-0.496262828	0.619708989	0.753097341
ENSG00000251493	52.07392563	5.099406524	0.498623541	10.22696706	1.50E-24	6.45E-23
ENSG00000251550	9.473636857	-0.639011563	0.570465702	-1.120157724	0.262646556	0.429049444
ENSG00000251562	208519.967	0.10525716	0.069601717	1.512278218	0.1304631	0.257353625
ENSG00000251573	12.39042639	-0.015337355	0.497970885	-0.030799703	0.975429277	0.987592263
ENSG00000251580	12.34191646	-1.179943599	0.531087397	-2.221750331	0.026300183	0.072353833
ENSG00000251602	37.07964348	-0.263717378	0.298174877	-0.884438627	0.376459479	0.54623761
ENSG00000251615	32.04188677	-0.194151964	0.302569952	-0.641676289	0.521083388	0.675369842
ENSG00000251667	10.29951101	-0.839440276	0.55985016	-1.49940169	0.133769455	0.262218747
ENSG00000251733	18.27195599	-0.590896688	0.422441301	-1.398766377	0.161883052	0.301587696
ENSG00000251791	13.56669851	-0.15353932	0.467090716	-0.328714134	0.74237177	0.842538277
ENSG00000251972	9.609455075	0.096104761	0.556558194	0.172676931	0.862905378	0.922223828
ENSG00000252010	164.3318623	-0.35584126	0.151441892	-2.349688428	0.018789131	0.054848489
ENSG00000252213	50.6285101	0.436084872	0.246824013	1.766784626	0.077264304	0.171681177
ENSG00000252498	12.71099061	-0.693853109	0.506898623	-1.368820268	0.171055464	0.314052555
ENSG00000252577	14.90354271	-1.690870105	0.499619117	-3.384318265	0.000713552	0.003327779
ENSG00000252690	11.33384151	-1.226074246	0.559679794	-2.190670912	0.028475615	0.077189689
ENSG00000252835	196.0307013	-0.193446802	0.137951193	-1.40228437	0.160830346	0.300104127
ENSG00000252947	30.5249575	-1.496050785	0.35413712	-4.224495827	2.39E-05	0.000158302
ENSG00000253159	34.83772607	-0.576829094	0.332583482	-1.734388885	0.082849106	0.181472227
ENSG00000253178	11.99560771	-0.588857722	0.510322258	-1.153893864	0.248543685	0.412749377
ENSG00000253187	58.9360073	-1.147056726	0.245629226	-4.669870697	3.01E-06	2.42E-05
ENSG00000253200	25.10628423	-0.255925014	0.343477975	-0.745098764	0.456212017	0.61991199
ENSG00000253210	18.31034126	-0.539196218	0.407677842	-1.322603689	0.185967207	0.334008612
ENSG00000253213	12.81082378	-0.072201498	0.487185606	-0.148201213	0.882183974	0.935107024
ENSG00000253218	15.01397299	-0.22569139	0.461440326	-0.489102009	0.624769479	0.756367911
ENSG00000253276	45.41505919	-0.14498439	0.263090978	-0.551080814	0.581578277	0.723904157
ENSG00000253284	140.4416728	-0.253517893	0.173432963	-1.461763022	0.14380616	0.276264378
ENSG00000253293	254.4090377	-1.508904977	0.128488835	-11.74347153	7.63E-32	4.41E-30
ENSG00000253304	12.27158679	-3.203264544	0.694350188	-4.613327107	3.96E-06	3.10E-05
ENSG00000253305	46.33747522	-0.227172571	0.265499786	-0.855641259	0.392196241	0.560529343
ENSG00000253308	5.582676515	-5.762497859	1.318709961	-4.369799295	1.24E-05	8.73E-05
ENSG00000253320	11.36082283	-1.236541886	0.542704941	-2.278479136	0.022698046	0.06393412
ENSG00000253341	21.43933851	-0.136451375	0.36992945	-0.368857831	0.712233696	0.820165336
ENSG00000253352	788.2990028	-0.266312042	0.084009264	-3.170031842	0.001524222	0.006474373
ENSG00000253368	24.78529478	-0.549593168	0.352055747	-1.561096992	0.118500868	0.239370389

ENSG00000253405	7.221312847	-6.132566806	1.289537313	-4.755633471	1.98E-06	1.65E-05
ENSG00000253438	48.7834488	4.307537337	0.419977105	10.25660038	1.11E-24	4.82E-23
ENSG00000253485	18.64118203	0.410149577	0.400051939	1.025240815	0.305249574	0.476215101
ENSG00000253508	20.78474231	-2.560093897	0.483762697	-5.292044871	1.21E-07	1.26E-06
ENSG00000253537	15.46556938	-0.239996754	0.438235098	-0.547643845	0.583936477	0.725954128
ENSG00000253598	9.477931253	-0.202206095	0.557316154	-0.362821163	0.716738481	0.823627736
ENSG00000253626	21.51679575	1.886147128	0.418504811	4.50687084	6.58E-06	4.90E-05
ENSG00000253636	24.18067011	-0.846647055	0.361715588	-2.340642991	0.019250565	0.055967852
ENSG00000253649	307.2358172	0.215000128	0.110646017	1.943134828	0.05199987	0.125743187
ENSG00000253669	77.92373942	-1.023019696	0.206396126	-4.956583802	7.17E-07	6.55E-06
ENSG00000253671	9.72111811	-2.165402908	0.645894526	-3.352564268	0.000800666	0.003679085
ENSG00000253683	9.311662064	0.327681239	0.575677557	0.569209683	0.56921385	0.714607825
ENSG00000253710	94.46067598	-0.194758101	0.188200755	-1.034842295	0.300742567	0.471067221
ENSG00000253716	61.38552081	0.369854917	0.224837197	1.644989892	0.099971896	0.209660379
ENSG00000253719	287.1052124	-0.365643223	0.113456405	-3.222764064	0.001269601	0.005524809
ENSG00000253729	6503.72068	-0.080004944	0.057136358	-1.400245775	0.161439733	0.30096137
ENSG00000253731	17.99461795	0.517241991	0.413754815	1.250117151	0.211256755	0.366298879
ENSG00000253738	371.0205048	-0.192360246	0.11934633	-1.61178183	0.107009423	0.221248362
ENSG00000253741	44.19546304	-0.596422574	0.273282116	-2.182442758	0.029076872	0.078531593
ENSG00000253767	10.9696078	-0.314296044	0.556441731	-0.564831906	0.572188103	0.717316045
ENSG00000253846	32.24727045	-0.250988311	0.306513925	-0.81884799	0.412873147	0.581183394
ENSG00000253873	25.9125714	0.139755834	0.347334769	0.402366379	0.687414409	0.802917762
ENSG00000253910	22.28810196	-0.50038281	0.370501852	-1.350554138	0.1768383	0.32193463
ENSG00000253948	23.17702101	-0.140697145	0.358975918	-0.391940346	0.695102291	0.808554313
ENSG00000253982	19.86929475	0.813230943	0.420028267	1.936133843	0.052851311	0.127319904
ENSG00000254004	120.7810559	-0.0934018	0.163322812	-0.571884594	0.567400162	0.71284054
ENSG00000254087	329.3604236	0.058063354	0.1091436	0.531990461	0.594732602	0.734702534
ENSG00000254093	82.28738995	0.291364866	0.195793076	1.488126507	0.136717534	0.266288298
ENSG00000254115	13.52211683	-7.038586962	1.239915699	-5.676665736	1.37E-08	1.65E-07
ENSG00000254119	13.874296	1.606738663	0.49887491	3.220724534	0.00127867	0.005559713
ENSG00000254122	102.6728124	0.11674168	0.174411386	0.66934667	0.503274362	0.661009347
ENSG00000254165	25.62419161	0.06088151	0.360352876	0.168949699	0.865836207	0.924545891
ENSG00000254206	40.04031076	0.175441201	0.287348312	0.610552398	0.541495945	0.691142874
ENSG00000254221	26.5955747	0.51738058	0.354560266	1.459217599	0.144505226	0.277379737
ENSG00000254319	7.191201994	-6.128137353	1.292131703	-4.742656912	2.11E-06	1.75E-05
ENSG00000254346	9.054989997	-2.900726971	0.777486687	-3.730902431	0.000190795	0.001025015
ENSG00000254363	7.389116786	-1.026631532	0.648518413	-1.583041454	0.113412016	0.231353266
ENSG00000254389	19.59412406	-0.078513224	0.391618812	-0.20048379	0.841102235	0.907697202
ENSG00000254394	224.2223031	0.190600753	0.149525278	1.274705895	0.202413359	0.3550575
ENSG00000254402	34.78667777	0.749533429	0.324135179	2.312409998	0.0207551	0.059560141
ENSG00000254469	23.84204327	-0.091602652	0.369419477	-0.247963786	0.804162426	0.88388651
ENSG00000254470	286.0571607	0.070924321	0.116839561	0.607023166	0.543835538	0.692768678
ENSG00000254505	22.3086824	0.159916094	0.366912735	0.43584231	0.662951144	0.784604829
ENSG00000254535	147.9300222	-0.928180365	0.157165513	-5.905750874	3.51E-09	4.54E-08
ENSG00000254602	34.4090884	-1.118245375	0.345206684	-3.239350302	0.001198023	0.005250744
ENSG00000254612	11.49224054	0.646312192	0.510002409	1.267272823	0.205057754	0.358576656
ENSG00000254635	106.2898899	-0.572210093	0.183752666	-3.114023354	0.001845549	0.007640561
ENSG00000254665	21.64192537	-0.353096519	0.386603225	-0.913330505	0.361068743	0.532125413
ENSG00000254682	18.18775815	0.174896752	0.423540788	0.412939573	0.679650886	0.796783175
ENSG00000254685	13.71648141	-0.289796173	0.478710942	-0.605367765	0.544934665	0.693611565

ENSG00000254712	13.56660796	-0.887396531	0.48664106	-1.823513476	0.068225658	0.155958636
ENSG00000254726	348.5416415	-0.158049745	0.112028147	-1.410803877	0.158302451	0.296569022
ENSG00000254791	8.726260452	0.294354091	0.573080883	0.513634463	0.60750758	0.744127919
ENSG00000254815	19.02270457	-0.418611279	0.396271877	-1.056373926	0.290797413	0.461146
ENSG00000254819	8.985406823	0.163520348	0.572502832	0.285623649	0.775166388	0.865324687
ENSG00000254837	14.48029317	-0.488917109	0.458779896	-1.065689916	0.286563837	0.456390275
ENSG00000254858	106.7020249	-0.127234775	0.181015233	-0.702895403	0.482120936	0.642721153
ENSG00000254860	16.67279654	0.572943341	0.444383441	1.289299485	0.19729399	0.348733947
ENSG00000254901	74.78026195	-0.608243802	0.206523784	-2.945151354	0.003227967	0.012288145
ENSG00000254936	38.32225009	-0.692653869	0.28836522	-2.402002116	0.016305614	0.048868138
ENSG00000254986	311.5494638	-0.213642321	0.108100188	-1.976336254	0.048116706	0.118411943
ENSG00000254996	22.42103009	-0.187695027	0.38155281	-0.491924111	0.622772993	0.755134766
ENSG00000254999	301.2620216	-0.007550688	0.12799679	-0.058991232	0.952959092	0.974251555
ENSG00000255046	10.60837692	-0.332113123	0.530573728	-0.62595094	0.531347121	0.683156819
ENSG00000255052	8.27085157	-1.465171489	0.638436331	-2.294937518	0.021736705	0.061783792
ENSG00000255062	12.93651837	-0.511328383	0.521437158	-0.980613627	0.326783312	0.498977615
ENSG00000255112	175.4942734	-0.224713617	0.138760334	-1.619436986	0.105353274	0.218402811
ENSG00000255121	36.9669672	-0.371978434	0.30593373	-1.215879119	0.224030974	0.38233059
ENSG00000255135	24.8336017	-0.07092491	0.360818059	-0.196566964	0.844166425	0.90976923
ENSG00000255150	22.26057608	0.401844696	0.367311135	1.094017191	0.273947439	0.442213246
ENSG00000255182	23.57425126	0.202431966	0.368899798	0.548745127	0.583180373	0.725270889
ENSG00000255198	85.17433747	0.300404133	0.194062751	1.547974207	0.121628507	0.244492793
ENSG00000255284	33.34921821	-0.25555093	0.298260819	-0.856803554	0.391553461	0.559925834
ENSG00000255302	657.9009419	-0.100711254	0.088811243	-1.133992172	0.256797832	0.422535821
ENSG00000255310	28.05407727	-0.733474708	0.337070451	-2.176027908	0.029553174	0.079527478
ENSG00000255374	14.16674345	-0.4089968	0.481956874	-0.848617006	0.396094441	0.563893646
ENSG00000255423	33.95849331	-0.181312547	0.31103514	-0.582932678	0.559938621	0.706626342
ENSG00000255491	8.967867791	-1.389672424	0.640350384	-2.170175046	0.029993588	0.080435267
ENSG00000255517	15.20974564	0.047771701	0.441659552	0.108164085	0.913865534	0.953578158
ENSG00000255529	51.3759679	0.190593201	0.251161421	0.758847439	0.447943824	0.613042371
ENSG00000255561	9.462574141	0.763807883	0.572816852	1.333424254	0.182392617	0.329357128
ENSG00000255571	36.96440654	0.443460909	0.288720574	1.535951883	0.124550182	0.248799261
ENSG00000255642	20.07371662	-0.280936976	0.394737434	-0.71170594	0.476646889	0.63753861
ENSG00000255710	11.77642257	-0.564782507	0.513825812	-1.099171146	0.271693422	0.439469354
ENSG00000255717	505.7275502	0.328076805	0.093209092	3.51979402	0.000431882	0.00212492
ENSG00000255837	12.69265324	0.061252071	0.489461318	0.125141801	0.900411291	0.946145827
ENSG00000255856	15.95881975	-0.645763401	0.434479227	-1.486292924	0.137201658	0.266871768
ENSG00000255857	63.53571649	0.321623595	0.217816284	1.476581956	0.139787731	0.27068438
ENSG00000255946	12.91894588	-1.315830762	0.548770387	-2.397780188	0.01649476	0.049319263
ENSG00000255960	17.11919982	1.12075089	0.448117438	2.501020478	0.012383602	0.038690492
ENSG00000256043	99.04814222	-1.785911707	0.205691627	-8.682471586	3.87E-18	1.17E-16
ENSG00000256053	51.04900132	-0.025181966	0.245892993	-0.102410264	0.918431037	0.955355281
ENSG00000256073	8.816928637	0.282165505	0.600080785	0.470212532	0.638203181	0.766295498
ENSG00000256092	9.850765353	0.407782958	0.559693396	0.728582759	0.466256928	0.628880558
ENSG00000256139	15.69222573	-0.484435587	0.461085775	-1.050640928	0.293423536	0.463987641
ENSG00000256188	17.25052617	-0.576881815	0.42044803	-1.372064496	0.170043361	0.313012592
ENSG00000256223	29.77996933	-0.042978147	0.317632692	-0.135307693	0.892368603	0.941285707
ENSG00000256235	79.92461621	-0.222589668	0.203039438	-1.096287844	0.272952827	0.440962434
ENSG00000256238	16.11043371	-0.381509073	0.443162176	-0.86087914	0.389304612	0.557731961
ENSG00000256269	125.915176	-0.148350126	0.167773846	-0.884226766	0.376573812	0.546267833

ENSG00000256294	25.29518368	0.390585612	0.341538165	1.143607512	0.252786445	0.417437186
ENSG00000256341	11.33549604	0.111465186	0.511066247	0.218103205	0.827348701	0.898686295
ENSG00000256436	9.287413228	-0.287518143	0.56360699	-0.51013942	0.60995379	0.74621328
ENSG00000256525	226.8940131	0.021145637	0.128182243	0.164965414	0.868971205	0.926977409
ENSG00000256537	179.5285946	-0.033461174	0.143316396	-0.233477638	0.815390532	0.891608898
ENSG00000256591	12.55208476	-0.570447229	0.498229189	-1.144949437	0.252230104	0.416881547
ENSG00000256616	14.32914404	1.498397517	0.479804392	3.122934144	0.001790578	0.007432698
ENSG00000256628	35.01881766	-0.297289578	0.294156014	-1.010652729	0.312182669	0.483753994
ENSG00000256663	43.71482783	0.113472205	0.277541876	0.40884715	0.682651836	0.799239793
ENSG00000256682	9.703473049	-0.465879811	0.599621534	-0.776956438	0.437184472	0.603138409
ENSG00000256712	33.29639441	-0.598133038	0.306495689	-1.951521863	0.050994998	0.123702343
ENSG00000256751	10.33097531	-1.077371339	0.555535133	-1.939339701	0.052459988	0.126659582
ENSG00000256806	24.5570132	0.244671617	0.359103928	0.681339294	0.495656825	0.654431001
ENSG00000256973	9.621478119	-0.027544865	0.547938443	-0.050269998	0.959907231	0.978814678
ENSG00000256981	24.75256551	-0.176385141	0.348532205	-0.506079891	0.612800567	0.748183007
ENSG00000257027	17.00919406	-0.644019516	0.432615044	-1.488666482	0.136575215	0.266121799
ENSG00000257086	11.04337654	0.136792168	0.517948257	0.264103925	0.791699839	0.875713229
ENSG00000257093	68.38304277	1.107021745	0.234705674	4.716638184	2.40E-06	1.97E-05
ENSG00000257103	913.7659871	-0.046394618	0.075844226	-0.611709288	0.540730116	0.690575137
ENSG00000257135	20.83387955	-0.249949604	0.378816229	-0.65981757	0.509370907	0.665836024
ENSG00000257167	43.50718335	-0.481303747	0.278882331	-1.72583091	0.084377837	0.184103057
ENSG00000257176	8.582719397	-0.785400383	0.595671762	-1.318512027	0.187332302	0.335645005
ENSG00000257178	69.1912535	-1.053355849	0.222648957	-4.731016322	2.23E-06	1.84E-05
ENSG00000257218	162.1783857	0.145210198	0.148331759	0.978955545	0.327601945	0.499953039
ENSG00000257242	14.79910705	-0.887901216	0.464060938	-1.913328928	0.055705957	0.132830007
ENSG00000257261	48.37205543	-1.529951351	0.278248859	-5.498499997	3.83E-08	4.33E-07
ENSG00000257267	117.0524548	0.167430786	0.16917455	0.989692515	0.322324435	0.494235491
ENSG00000257298	15.75252086	-1.496799582	0.476269017	-3.142760768	0.001673626	0.007021929
ENSG00000257315	113.9662094	-0.004676161	0.172665505	-0.027082196	0.978394175	0.988843871
ENSG00000257337	23.1870674	-0.370213599	0.367014402	-1.008716816	0.313110465	0.484283494
ENSG00000257354	65.67697825	-0.670375112	0.219489139	-3.054251872	0.002256226	0.009061931
ENSG00000257365	37.17855582	0.337466635	0.298239996	1.131527092	0.257833307	0.423501457
ENSG00000257475	9.476023037	-0.261442867	0.566252006	-0.461707623	0.644291004	0.770636558
ENSG00000257497	8.5699115	-0.698589815	0.614537544	-1.136773206	0.255633107	0.420970487
ENSG00000257509	8.390413213	-0.267677215	0.609935944	-0.438861191	0.660762119	0.783076162
ENSG00000257511	19.68093198	0.233090428	0.398895397	0.584339728	0.558991769	0.705748461
ENSG00000257556	11.08295917	-0.556221004	0.526485584	-1.056479076	0.290749395	0.46112191
ENSG00000257594	25.74950949	-1.676862949	0.396090808	-4.23353159	2.30E-05	0.000152502
ENSG00000257621	339.5864597	-0.261282867	0.105882536	-2.467667266	0.013599667	0.041856818
ENSG00000257698	14.49853277	-0.369024041	0.455616251	-0.809944862	0.417971866	0.58598722
ENSG00000257702	42.08639513	-1.495321859	0.291109657	-5.136627471	2.80E-07	2.74E-06
ENSG00000257704	75.51878749	0.005884755	0.211464498	0.027828571	0.977798878	0.988629033
ENSG00000257727	87.41603846	0.343184591	0.190744283	1.799186775	0.071989141	0.162316446
ENSG00000257803	9.260959135	-0.165653893	0.570426168	-0.29040374	0.771507383	0.863328623
ENSG00000257815	19.17086236	-0.657896427	0.418088595	-1.573581378	0.115584261	0.234516753
ENSG00000257880	15.55546226	1.666236428	0.473304328	3.520433531	0.000430842	0.002121289
ENSG00000257893	29.69333901	4.125448045	0.511194461	8.070212727	7.02E-16	1.81E-14
ENSG00000257913	129.148076	0.462617597	0.161726174	2.86049924	0.004229746	0.015525908
ENSG00000257923	1521.360163	-0.189146353	0.066930149	-2.826026171	0.004712941	0.017023062
ENSG00000258011	7.094040703	0.581822916	0.673804014	0.863489833	0.387868207	0.556525506

ENSG00000258017	14.87448478	0.210792029	0.475442	0.443360134	0.657505267	0.780527041
ENSG00000258056	40.40581656	-0.141329243	0.27448251	-0.514893437	0.606627487	0.743503736
ENSG00000258057	9.290259684	-0.64102542	0.594182509	-1.078835897	0.280660889	0.449486206
ENSG00000258125	15.02065183	3.739371732	0.680104785	5.498228824	3.84E-08	4.34E-07
ENSG00000258289	163.6045385	-0.476885884	0.159061187	-2.99812853	0.002716431	0.010590537
ENSG00000258301	48.01616061	0.057800358	0.251078073	0.230208707	0.817929597	0.892592996
ENSG00000258365	14.15970686	-1.035635954	0.503636389	-2.056316771	0.039751993	0.101360727
ENSG00000258366	37.99308276	-0.188121857	0.282916976	-0.664936618	0.506091033	0.663653916
ENSG00000258429	14.1835042	0.513272528	0.506067398	1.014237491	0.310469443	0.481844765
ENSG00000258441	374.6790831	-0.636848866	0.104516853	-6.093264825	1.11E-09	1.53E-08
ENSG00000258469	14.93386641	-0.156594275	0.459483355	-0.340805108	0.733250305	0.835693316
ENSG00000258525	33.44802024	-0.337384398	0.308759376	-1.092709806	0.274521233	0.442834148
ENSG00000258561	13.63760011	-0.962596833	0.487642268	-1.973981535	0.048383843	0.118965139
ENSG00000258568	14.80005333	0.074951318	0.442525213	0.169371859	0.865504157	0.924472427
ENSG00000258603	13.73800572	0.255472578	0.464206042	0.550343069	0.582084089	0.724322418
ENSG00000258616	41.95890865	4.392801278	0.459226523	9.565652377	1.11E-21	4.11E-20
ENSG00000258634	128.7776769	0.152893041	0.167599356	0.912253154	0.361635469	0.532503677
ENSG00000258636	26.3487716	4.61424877	0.614648875	7.507129613	6.04E-14	1.34E-12
ENSG00000258655	13.30764494	0.023964767	0.478670538	0.050065264	0.96007038	0.978814678
ENSG00000258700	12.79922568	7.32071814	1.248522991	5.86350287	4.53E-09	5.80E-08
ENSG00000258704	23.33609887	0.826603642	0.362634339	2.279441172	0.022640853	0.063797179
ENSG00000258725	16.03441586	0.482775399	0.439793459	1.097732103	0.272321487	0.440195619
ENSG00000258727	68.16248632	1.041001101	0.221282075	4.70440771	2.55E-06	2.08E-05
ENSG00000258728	11.80784764	0.572071556	0.505423105	1.131866648	0.257690502	0.423403097
ENSG00000258732	114.4032954	0.043278884	0.167748645	0.257998411	0.796408134	0.878387645
ENSG00000258738	11.23550339	-0.462072582	0.543663589	-0.849923724	0.395367495	0.563217718
ENSG00000258743	7.692433106	-2.932354392	0.820236782	-3.575009627	0.000350215	0.001763883
ENSG00000258744	16.20450271	-1.229557375	0.451535926	-2.723055474	0.00646812	0.022241781
ENSG00000258827	7.665530636	-2.259178403	0.739867528	-3.053490413	0.002261959	0.00907562
ENSG00000258839	39.90139626	0.464916466	0.273307035	1.701077564	0.088928423	0.191532898
ENSG00000258881	11.20000286	0.686158019	0.527054707	1.30187248	0.192959981	0.343667917
ENSG00000258890	350.4122554	-0.252539036	0.109943892	-2.296981053	0.021619851	0.061534861
ENSG00000258904	8.977404534	0.439109579	0.588460117	0.746201087	0.455545951	0.619283265
ENSG00000258920	23.19705645	0.199304996	0.358130978	0.556514259	0.57785935	0.721335669
ENSG00000258925	7.095106861	0.24352704	0.671951074	0.362417815	0.717039828	0.823803836
ENSG00000258947	32.68965922	-0.202890697	0.322967169	-0.628208425	0.529867412	0.682240213
ENSG00000259020	11.05772114	0.815281362	0.555257751	1.468293529	0.142024508	0.273777723
ENSG00000259045	28.79238139	-0.033611058	0.339832913	-0.098904658	0.921213971	0.956727767
ENSG00000259098	130.9866241	0.149130502	0.17454972	0.854372626	0.392898558	0.561106755
ENSG00000259144	8.748383064	0.388623342	0.604079488	0.643331466	0.520009043	0.674538277
ENSG00000259153	13.5558613	-1.742680586	0.518958232	-3.358036311	0.000784983	0.003614112
ENSG00000259248	10.75323183	-0.00268577	0.525392079	-0.005111935	0.995921284	0.99812402
ENSG00000259291	10.34134599	-2.151987956	0.619148886	-3.47571966	0.000509485	0.002460203
ENSG00000259326	14.78458919	-0.762149318	0.463822534	-1.643191654	0.100343286	0.210250916
ENSG00000259330	88.08385343	-0.284126264	0.19055102	-1.491077107	0.135941252	0.26532816
ENSG00000259336	28.65789387	-1.082370187	0.355826637	-3.041846994	0.002351314	0.009375221
ENSG00000259363	42.28526171	0.011068369	0.271548207	0.040760237	0.967487039	0.982880628
ENSG00000259366	40.83431278	-0.224808084	0.276096634	-0.814236962	0.415509215	0.583340116
ENSG00000259431	31.1840766	0.532529149	0.30925764	1.721959555	0.085076845	0.185225426
ENSG00000259439	95.93755279	0.031686246	0.182691462	0.173441308	0.862304558	0.921755736

ENSG00000259462	12.01476776	-0.451858694	0.511046083	-0.884183852	0.376596974	0.546267833
ENSG00000259475	28.64509	0.24889386	0.332830121	0.747810502	0.454574466	0.618431859
ENSG00000259488	8.811117657	-0.063158086	0.60263562	-0.104803108	0.916532044	0.954800179
ENSG00000259494	71.84827166	0.102777483	0.207446278	0.495441443	0.620288547	0.753606056
ENSG00000259495	11.02662019	-1.854668881	0.586137324	-3.164222452	0.001554979	0.00658709
ENSG00000259516	18.71524547	0.040975423	0.398439291	0.102839816	0.918090104	0.955355281
ENSG00000259539	11.94819772	-0.027775022	0.512140923	-0.054233162	0.9567494	0.97684684
ENSG00000259562	10.66004187	-0.265250787	0.53134535	-0.499205999	0.617634268	0.751514768
ENSG00000259581	20.65168473	-0.181560929	0.38816474	-0.467741941	0.639969144	0.767565619
ENSG00000259585	807.0291813	-0.826390617	0.079890824	-10.34399922	4.46E-25	1.99E-23
ENSG00000259592	54.21686314	-0.674285953	0.2448567	-2.75379825	0.005890807	0.020553444
ENSG00000259605	14.64289603	0.235625563	0.449326175	0.52439759	0.600002032	0.738540882
ENSG00000259642	26.42550173	0.315966504	0.355827094	0.887977643	0.374552778	0.54453973
ENSG00000259663	29.25853311	-0.762041361	0.334655613	-2.27709123	0.022780778	0.06414143
ENSG00000259673	36.82307386	-0.364469493	0.29587816	-1.231822898	0.218015248	0.375025739
ENSG00000259687	11.18715359	0.04201926	0.552373425	0.076070386	0.939363101	0.96647136
ENSG00000259706	20.71400934	-0.155018955	0.392045955	-0.395410162	0.69254021	0.806442206
ENSG00000259768	79.90984249	-0.13424312	0.198628655	-0.675849714	0.499136067	0.65717165
ENSG00000259781	48.29659336	0.171008573	0.2502248	0.683419759	0.494341638	0.653493035
ENSG00000259790	9.736827778	-0.278798267	0.550605507	-0.50634849	0.612612028	0.74803209
ENSG00000259797	8.266305638	0.095039053	0.604824939	0.157134812	0.875138602	0.9309407
ENSG00000259802	20.83837266	-0.246058404	0.374016244	-0.657881599	0.510614215	0.667094297
ENSG00000259820	20.26485232	-0.257067038	0.383962599	-0.669510621	0.503169808	0.660933822
ENSG00000259865	9.621220314	-0.919465688	0.575694444	-1.597141847	0.110234092	0.226395741
ENSG00000259891	11.24434784	-1.542558932	0.578479149	-2.666576546	0.007662815	0.025794289
ENSG00000259895	9.782084102	-0.383121984	0.577165712	-0.663798934	0.506819008	0.664065744
ENSG00000259915	6.045166876	-1.983984852	0.788242778	-2.516971809	0.01183683	0.037219882
ENSG00000259917	21.7204601	0.699008365	0.36829333	1.897966397	0.057700509	0.136321964
ENSG00000259921	33.05013478	-0.499860412	0.304397154	-1.642132347	0.100562579	0.210553369
ENSG00000259924	12.95818304	0.183101747	0.48105608	0.380624535	0.703481873	0.814282078
ENSG00000259943	12.98664368	-0.14670834	0.473397962	-0.309904883	0.75663329	0.852935384
ENSG00000259946	15.92355519	1.818590438	0.477435164	3.809083569	0.000139483	0.000776581
ENSG00000259956	775.6599493	0.414243726	0.079201357	5.230260465	1.69E-07	1.72E-06
ENSG00000259959	55.15640206	-0.304505323	0.243697134	-1.249523612	0.211473623	0.366375183
ENSG00000259972	45.41269283	-0.254615342	0.27286923	-0.933103896	0.350766328	0.5225042
ENSG00000259994	20.55696235	-0.014983626	0.382128501	-0.039210961	0.968722195	0.983424201
ENSG00000260007	25.16228291	-0.180263395	0.348905596	-0.516653781	0.605397865	0.742455469
ENSG00000260027	298.4586547	0.033418027	0.109652836	0.304762083	0.760547362	0.855365062
ENSG00000260032	1301.192854	-0.147198085	0.072839358	-2.020859186	0.043294344	0.109066651
ENSG00000260035	13.49410053	-0.105379659	0.503384998	-0.209342073	0.83418121	0.902777673
ENSG00000260060	19.78063892	-0.256687398	0.392724903	-0.653606114	0.513365604	0.669319347
ENSG00000260063	16.82714871	-0.558087409	0.430586872	-1.296108743	0.194938023	0.346137582
ENSG00000260083	12.81974876	-0.363613508	0.490851569	-0.740780984	0.458826247	0.622417411
ENSG00000260103	11.07993376	-0.754560117	0.535730442	-1.408469741	0.158992021	0.297536515
ENSG00000260123	10.90659658	-0.368886871	0.523230347	-0.705018113	0.480798964	0.641202227
ENSG00000260136	8.879875049	-0.193352258	0.574363428	-0.336637481	0.736390198	0.837848138
ENSG00000260190	9.861214968	0.810059993	0.559804397	1.447041139	0.147885402	0.282031909
ENSG00000260193	10.35513799	-0.869772306	0.565009613	-1.539393821	0.123708182	0.247716155
ENSG00000260196	99.65752194	0.163565783	0.183577762	0.890989088	0.372935024	0.543368768
ENSG00000260219	22.69317913	-1.062356868	0.375877777	-2.826335937	0.004708386	0.017019736

ENSG00000260236	52.13650164	0.05634045	0.263273729	0.213999515	0.830547436	0.90129605
ENSG00000260257	47.74029196	-0.121073161	0.263272245	-0.459878182	0.645603662	0.771746629
ENSG00000260260	89.58940073	-0.141767309	0.192476467	-0.736543593	0.461399948	0.624585702
ENSG00000260267	18.58060204	-0.706451691	0.416290222	-1.697017257	0.089693398	0.192820763
ENSG00000260274	10.48408852	0.165034481	0.528028725	0.312548301	0.754623861	0.851673232
ENSG00000260290	7.799393342	0.001522795	0.618891797	0.002460519	0.998036792	0.999084016
ENSG00000260293	47.28383987	-0.15900832	0.267200555	-0.595089781	0.551783459	0.699350559
ENSG00000260296	9.583124601	-1.968890132	0.657641141	-2.993867032	0.00275466	0.010730645
ENSG00000260318	53.06538083	-0.051470497	0.246792119	-0.208558106	0.834793218	0.90327687
ENSG00000260361	8.083016668	-0.59633035	0.626352389	-0.952068453	0.341062267	0.512999428
ENSG00000260400	60.8278793	-0.882063018	0.238187633	-3.703227613	0.000212874	0.001132237
ENSG00000260423	9.423604632	-1.241033908	0.631864164	-1.964083388	0.049520422	0.120955203
ENSG00000260428	137.2378574	2.3259246	0.176098265	13.20810626	7.88E-40	6.08E-38
ENSG00000260442	134.4618568	-0.445722607	0.166796319	-2.672256858	0.007534295	0.025422591
ENSG00000260448	18.7388768	0.254643761	0.402607404	0.632486534	0.527069005	0.68011559
ENSG00000260456	22.25732834	3.207772465	0.749557743	4.279553505	1.87E-05	0.000126409
ENSG00000260528	15.49175808	0.097419315	0.464908481	0.209545144	0.834022698	0.902721285
ENSG00000260565	86.30607465	-0.33352877	0.191980724	-1.737303429	0.082333627	0.180624778
ENSG00000260597	140.9178102	-0.73024514	0.160460289	-4.550939955	5.34E-06	4.07E-05
ENSG00000260630	57.54222913	-0.928352034	0.243867256	-3.806792467	0.000140781	0.000783187
ENSG00000260645	10.01821288	0.279351033	0.548075816	0.509694142	0.610265758	0.746529922
ENSG00000260698	13.24978892	-1.274795003	0.510070047	-2.499254779	0.01244548	0.038866531
ENSG00000260708	33.40210577	0.866909627	0.311388497	2.784013008	0.005369088	0.018997375
ENSG00000260751	16.65486221	1.094547254	0.450341086	2.430484999	0.01507863	0.045737318
ENSG00000260793	23.58929345	-1.333130502	0.387506984	-3.440274776	0.000581124	0.002769012
ENSG00000260804	56.80475723	-1.154214624	0.240657619	-4.796085946	1.62E-06	1.37E-05
ENSG00000260852	55.6082332	0.29722935	0.243677205	1.219766738	0.222553314	0.380826702
ENSG00000260855	66.97676056	0.121268895	0.22422525	0.540835144	0.588621217	0.730091746
ENSG00000260912	7.498209058	-0.740545181	0.647233267	-1.144170454	0.252552954	0.417218786
ENSG00000260916	31.71866103	0.380051124	0.310400388	1.224389976	0.220805149	0.378533021
ENSG00000260917	24.11567716	-0.281814427	0.355466927	-0.792800694	0.427893952	0.595170447
ENSG00000260918	14.39019503	-7.125124572	1.245690835	-5.719817767	1.07E-08	1.30E-07
ENSG00000260920	57.03613826	-0.170342555	0.239357358	-0.711666257	0.476671467	0.63753861
ENSG00000260924	9.867110232	-0.441421452	0.578233102	-0.763397064	0.445226631	0.610325858
ENSG00000260947	29.44264999	0.003558624	0.323727503	0.010992653	0.991229308	0.995481382
ENSG00000260948	18.99654188	0.112852699	0.403095159	0.279965405	0.779504047	0.868244866
ENSG00000260949	10.16365767	-1.780604278	0.608602894	-2.925724304	0.003436553	0.012990653
ENSG00000261008	90.26723274	-0.379658276	0.190806473	-1.989755745	0.046617849	0.115451502
ENSG00000261026	136.7340304	-0.583444878	0.170956758	-3.412821372	0.000642941	0.003026605
ENSG00000261054	11.91764637	1.086687052	0.521328053	2.084459193	0.037118418	0.095898919
ENSG00000261056	21.20971134	-1.037922006	0.392143509	-2.646791243	0.00812595	0.027108334
ENSG00000261071	12.85664665	0.429717116	0.473555006	0.907428093	0.364180459	0.535221061
ENSG00000261072	23.9928765	-0.052697358	0.359352262	-0.146645406	0.883411912	0.935631994
ENSG00000261094	10.78387001	-1.021465756	0.53691885	-1.902458362	0.057111257	0.135260585
ENSG00000261098	32.19534396	-0.476799627	0.307100117	-1.552586926	0.120521851	0.2426502
ENSG00000261115	28.54755793	-5.265442014	0.76805925	-6.8555154	7.11E-12	1.27E-10
ENSG00000261167	28.3704516	-0.479689435	0.346198658	-1.385590106	0.165872101	0.307537077
ENSG00000261221	261.6928716	-0.101420208	0.119428298	-0.849214216	0.395762104	0.563477628
ENSG00000261236	615.8057411	0.422474669	0.086709007	4.872327379	1.10E-06	9.66E-06
ENSG00000261315	8.803238985	-0.337210893	0.612767885	-0.550307713	0.582108335	0.724322418

ENSG00000261326	26.29245675	-0.850915167	0.347691754	-2.447326279	0.014392049	0.043929836
ENSG00000261341	11.83522031	-1.164635943	0.527108949	-2.2094786	0.027141369	0.074270709
ENSG00000261342	11.79944793	-0.598134797	0.503713528	-1.187450333	0.235050036	0.396035487
ENSG00000261351	10.64673223	-0.422454118	0.52831823	-0.799620559	0.423930672	0.591201918
ENSG00000261373	415.1015076	0.103328885	0.099318152	1.040382677	0.298162147	0.468750272
ENSG00000261455	13.2905699	0.110370154	0.470790092	0.234436017	0.8146465	0.891156404
ENSG00000261485	9.488528263	-0.384954322	0.56117003	-0.685985177	0.492722456	0.652082093
ENSG00000261514	110.8423376	0.73236547	0.169331387	4.325042636	1.53E-05	0.00010501
ENSG00000261526	12.65178608	-0.009565959	0.495707563	-0.019297586	0.984603709	0.992018958
ENSG00000261560	13.41444532	-0.340446274	0.492380461	-0.691429293	0.489295801	0.649021541
ENSG00000261572	17.92328465	-0.313515601	0.414445129	-0.756470711	0.449367027	0.614331739
ENSG00000261578	24.82726554	-0.109966307	0.351638736	-0.312725236	0.754489421	0.851612125
ENSG00000261609	201.7720225	-0.409900854	0.131077601	-3.127161705	0.001765028	0.007348355
ENSG00000261616	36.84528793	1.283920742	0.293504285	4.374453149	1.22E-05	8.56E-05
ENSG00000261655	59.35601477	1.174842436	0.241669722	4.861355516	1.17E-06	1.02E-05
ENSG00000261659	28.84130082	-0.063465164	0.324502739	-0.195576665	0.844941526	0.910191228
ENSG00000261739	6.352252512	-2.959556515	0.915343875	-3.233272869	0.001223806	0.005352056
ENSG00000261770	80.05918202	-0.896200841	0.208236917	-4.303755802	1.68E-05	0.000114731
ENSG00000261799	26.78129283	-0.253021545	0.344206163	-0.735087203	0.462286388	0.625299652
ENSG00000261801	8.989004506	-0.643225394	0.596202061	-1.078871469	0.280645029	0.449486206
ENSG00000261824	311.3566475	-0.75943098	0.121856835	-6.232157451	4.60E-10	6.70E-09
ENSG00000261840	11.95536414	-0.26591124	0.494398073	-0.537848456	0.590681675	0.731749316
ENSG00000261884	11.99941252	0.759089868	0.524318302	1.447765344	0.147682688	0.281855101
ENSG00000261934	16.97100834	0.788544671	0.430115279	1.833333316	0.066753018	0.153265714
ENSG00000261949	568.9478723	-2.388924312	0.101799378	-23.46698336	8.87E-122	2.97E-119
ENSG00000261970	19.62042224	-1.362350465	0.413449547	-3.295082738	0.000983927	0.004427041
ENSG00000261971	101.0997798	-0.185900934	0.182732003	-1.017341957	0.308990783	0.480151898
ENSG00000262001	54.8396256	-0.518654522	0.255295039	-2.031588717	0.04219531	0.106853708
ENSG00000262049	30.91682738	0.366178774	0.309111416	1.18461744	0.236168738	0.397355584
ENSG00000262050	16.55915789	-0.504669767	0.427461678	-1.180619908	0.237753751	0.399106949
ENSG00000262075	12.36891772	-0.09260586	0.493775974	-0.187546305	0.851232321	0.914699033
ENSG00000262155	14.90683229	-0.838242471	0.462839169	-1.811088015	0.070127228	0.159217258
ENSG00000262202	10.64014248	0.76080898	0.533897763	1.425008744	0.154154673	0.291128085
ENSG00000262209	17.45231817	0.029747475	0.425026684	0.069989665	0.944201886	0.969036596
ENSG00000262223	16.64934023	-0.694759785	0.447692724	-1.551867493	0.120693932	0.242892216
ENSG00000262246	66.81365852	0.506901449	0.214889043	2.358898531	0.018329267	0.053807986
ENSG00000262265	16.19527525	-0.42712778	0.436943045	-0.977536513	0.32830361	0.500640031
ENSG00000262312	12.99091597	-0.323889118	0.490899884	-0.659786503	0.509390846	0.665836024
ENSG00000262454	13.01606417	-0.320329664	0.51150198	-0.626253029	0.53114899	0.683094573
ENSG00000262468	10.23634574	-0.21836247	0.554493017	-0.393805626	0.69372455	0.807352838
ENSG00000262484	10.3256289	-0.1391233	0.530891715	-0.262055888	0.793278355	0.87679448
ENSG00000262528	29.85101503	0.817747835	0.323597924	2.527049073	0.011502543	0.03634193
ENSG00000262533	16.97069922	0.229195808	0.434307044	0.527727587	0.597688423	0.736869725
ENSG00000262576	16.06802617	-0.488490694	0.430784966	-1.133954832	0.256813495	0.422535821
ENSG00000262580	53.18254009	0.713834962	0.242912135	2.938655011	0.003296398	0.012524885
ENSG00000262585	13.5661828	-1.436307435	0.524695916	-2.737409215	0.006192521	0.021409388
ENSG00000262652	9.571839097	-1.137548839	0.580252308	-1.960438285	0.049944585	0.121750906
ENSG00000262691	20.23442682	0.715750414	0.399747428	1.790506617	0.073372504	0.164642743
ENSG00000262712	10.58458854	0.57862973	0.549158367	1.053666419	0.292035668	0.462535239
ENSG00000262766	19.32356053	0.253770438	0.402499143	0.630486904	0.528376068	0.681051443

ENSG00000262814	129.6864382	0.132607699	0.168708667	0.786015927	0.431858156	0.598137598
ENSG00000262874	53.31030331	-0.808000527	0.262808092	-3.074488767	0.002108637	0.008527648
ENSG00000262877	31.39645937	-1.011001063	0.319616171	-3.163172444	0.001560599	0.00660691
ENSG00000262879	191.7415421	-0.395042096	0.134139372	-2.94501227	0.003229419	0.012290339
ENSG00000262902	21.3856276	-0.310098905	0.371374389	-0.835003475	0.403715752	0.571903506
ENSG00000262919	59.3333502	0.14398863	0.239264056	0.601797999	0.547308607	0.695750589
ENSG00000263001	672.0805134	0.264170796	0.091015088	2.902494554	0.003702036	0.013823099
ENSG00000263002	69.85813346	0.535450554	0.212388728	2.521087434	0.011699278	0.03686401
ENSG00000263004	12.52948596	-1.570659302	0.561073865	-2.799380615	0.005120075	0.018254235
ENSG00000263050	8.696342042	-0.183222091	0.582580437	-0.314500933	0.753140608	0.850814585
ENSG00000263069	10.30823932	-0.483549188	0.543380295	-0.889890915	0.373524463	0.543607349
ENSG00000263072	48.45178389	0.182404022	0.25046836	0.728251751	0.466459492	0.628880558
ENSG00000263266	49.52692896	0.033683455	0.2556375	0.131762573	0.895172091	0.943073102
ENSG00000263327	32.66740431	-1.200958558	0.335776376	-3.576661857	0.00034801	0.001755294
ENSG00000263400	12.79815757	0.170825819	0.49119277	0.347777551	0.72800725	0.831881383
ENSG00000263465	243.7763577	0.161894565	0.122969339	1.316544159	0.187991467	0.336611362
ENSG00000263528	85.03684279	-0.168713721	0.198110583	-0.851613872	0.394428438	0.562548103
ENSG00000263603	39.19958501	0.295525521	0.287427273	1.02817495	0.30386754	0.474638867
ENSG00000263606	9.754907628	-1.065146273	0.569519942	-1.870252812	0.061448719	0.143574544
ENSG00000263731	116.4603437	-0.295561747	0.172457924	-1.713819468	0.086561887	0.187644135
ENSG00000263740	40.26785509	-0.838171768	0.309318323	-2.709738501	0.006733627	0.023036353
ENSG00000263786	24.85685939	0.437414142	0.357253407	1.224380602	0.220808683	0.378533021
ENSG00000263843	16.28311789	-0.601906521	0.434859347	-1.384140701	0.166315376	0.308088104
ENSG00000263874	7.259052803	-1.126582598	0.66911457	-1.683691625	0.092241281	0.197035205
ENSG00000263934	11082.31184	0.357793766	0.186716584	1.916239891	0.055334568	0.132146324
ENSG00000263956	76.25433825	0.200194638	0.207071739	0.9667888	0.333649625	0.50583001
ENSG00000264112	787.8040644	-0.059279097	0.106055208	-0.558945649	0.576198814	0.72035108
ENSG00000264229	44.9570723	0.394754171	0.266606483	1.480662308	0.138696583	0.269209741
ENSG00000264247	39.88986198	-1.109868806	0.285038851	-3.893745719	9.87E-05	0.000567562
ENSG00000264316	17.63058474	-0.109649538	0.429521432	-0.255283042	0.798504497	0.88000884
ENSG00000264350	74.36350499	0.191047348	0.206295549	0.926085651	0.35440147	0.525826821
ENSG00000264364	516.3789651	-0.070109062	0.101976046	-0.687505196	0.491764427	0.651295106
ENSG00000264456	10.94399998	-0.210117839	0.527678791	-0.398192693	0.690488152	0.805254219
ENSG00000264522	697.4047071	-0.18985886	0.087019711	-2.181791434	0.029124929	0.078616041
ENSG00000264546	8.075767856	-0.456239642	0.64117555	-0.711567436	0.476732678	0.63753861
ENSG00000264743	9.450928302	0.166192348	0.562119408	0.295653104	0.767495014	0.860009596
ENSG00000264853	13.88550848	-0.257476155	0.475066117	-0.541979624	0.587832543	0.729570925
ENSG00000264895	99.34764995	-0.619231499	0.181323994	-3.415055482	0.00063769	0.003007936
ENSG00000264920	28.32275243	-0.530170329	0.337773324	-1.569603905	0.116507289	0.235990463
ENSG00000265142	9.56733429	-2.134136132	0.642618735	-3.320998929	0.000896959	0.004080175
ENSG00000265145	11.66714526	-0.291857603	0.519355681	-0.561960934	0.574142636	0.718419883
ENSG00000265185	26.36035521	0.603027263	0.352836373	1.709084744	0.087435253	0.188922499
ENSG00000265241	258.088687	-0.108529442	0.118547456	-0.915493639	0.35993254	0.531230298
ENSG00000265354	413.7844925	0.001753419	0.104940733	0.016708661	0.986669037	0.992960307
ENSG00000265458	12.85076854	-1.157327396	0.505118442	-2.291200043	0.021951846	0.062269303
ENSG00000265491	260.8722304	-0.338741688	0.119139887	-2.843226531	0.004465932	0.016270418
ENSG00000265590	11.3407857	-0.408279084	0.517793948	-0.788497212	0.430405929	0.597156056
ENSG00000265666	16.82534866	-0.7599706	0.443237617	-1.714589582	0.0864205	0.187453484
ENSG00000265681	266.8368608	-0.050478652	0.117007276	-0.431414641	0.666166906	0.786348438
ENSG00000265688	85.35858372	0.182082797	0.191552057	0.950565606	0.341824935	0.513706523

ENSG00000265735	175.4316499	-0.687827032	0.168395188	-4.084600274	4.42E-05	0.000275161
ENSG00000265763	8.729846517	-0.283503563	0.580930967	-0.488015925	0.625538561	0.757038009
ENSG00000265778	9.756469817	-0.252453612	0.544440166	-0.463693951	0.642867031	0.769588648
ENSG00000265806	7.57258658	-0.503224643	0.652555305	-0.771160144	0.440612015	0.60586547
ENSG00000265808	222.2438089	0.428899058	0.14964803	2.866052141	0.004156258	0.015311554
ENSG00000265817	144.0677941	-0.251513575	0.164835308	-1.525847692	0.1270478	0.252210352
ENSG00000265972	265.3218317	-0.392616859	0.119674212	-3.280713984	0.001035447	0.00463547
ENSG00000266019	23.54923687	0.315549897	0.358848535	0.879340073	0.379216914	0.548764808
ENSG00000266028	896.3832428	-0.255046074	0.073825524	-3.454714045	0.000550877	0.002641914
ENSG00000266066	11.58400982	0.526605268	0.549953625	0.957544861	0.338292315	0.510265437
ENSG00000266074	953.052227	-0.562393534	0.093496761	-6.015112504	1.80E-09	2.43E-08
ENSG00000266094	86.53447615	0.915743658	0.194779856	4.701428964	2.58E-06	2.11E-05
ENSG00000266126	10.64313849	0.245746881	0.536514133	0.458043629	0.646921097	0.772538913
ENSG00000266155	11.92289258	0.090158685	0.498144293	0.180989096	0.856376135	0.917757792
ENSG00000266173	123.1416706	-0.018835696	0.172071701	-0.109464231	0.912834291	0.953048924
ENSG00000266208	23.60304454	-0.91756614	0.374480792	-2.450235525	0.01427628	0.043619328
ENSG00000266256	50.24834349	-1.46054887	0.266816182	-5.47398909	4.40E-08	4.91E-07
ENSG00000266338	105.295352	0.262419816	0.179968828	1.458140384	0.144801852	0.277783072
ENSG00000266402	264.5747898	-0.08877545	0.133145146	-0.666756937	0.504927404	0.662682319
ENSG00000266412	529.0468404	0.062846733	0.092217397	0.681506263	0.495551205	0.654353053
ENSG00000266472	1025.046039	0.552395113	0.078511722	7.035829838	1.98E-12	3.84E-11
ENSG00000266714	144.7670482	-0.28495619	0.157243733	-1.812194262	0.069956185	0.158957382
ENSG00000266777	15.50925957	0.06782294	0.432708747	0.156740396	0.875449449	0.931046487
ENSG00000266830	60.10949505	2.789923434	0.281092251	9.92529472	3.23E-23	1.29E-21
ENSG00000266844	20.19897264	-0.461840337	0.400167145	-1.154118581	0.248451557	0.412677435
ENSG00000266962	135.6894336	0.190993885	0.159045808	1.200873429	0.229800303	0.388728908
ENSG00000266965	28.50536546	0.291489767	0.322101287	0.904963063	0.365484956	0.53674513
ENSG00000266967	26.15275634	0.428978477	0.342573768	1.252222197	0.21048891	0.365654348
ENSG00000266983	12.41631796	0.008114307	0.483680488	0.016776171	0.98661518	0.992960307
ENSG00000266993	16.79730306	-1.621026986	0.463048898	-3.500768479	0.000463919	0.002264304
ENSG00000267002	47.83697303	-0.015440374	0.260137177	-0.059354737	0.952669564	0.974026448
ENSG00000267040	25.84384884	-0.260358281	0.3386224	-0.768874951	0.441967549	0.607051742
ENSG00000267041	64.0872797	-0.006516136	0.222830499	-0.029242567	0.976671132	0.988350939
ENSG00000267042	28.43235508	-0.132446686	0.346469506	-0.382275161	0.702257277	0.813676721
ENSG00000267060	11.75948904	-2.701197564	0.641695221	-4.209471222	2.56E-05	0.000168333
ENSG00000267080	109.3626311	0.325427306	0.175708816	1.852082971	0.064013909	0.148309976
ENSG00000267100	31.8507238	0.066130526	0.309538492	0.213642334	0.830825983	0.901344809
ENSG00000267106	50.809311	0.291723579	0.241168662	1.209624736	0.226422922	0.384823083
ENSG00000267119	9.588616811	-0.137218277	0.589407743	-0.232807049	0.815911239	0.891907519
ENSG00000267147	28.20096054	-0.528457091	0.32642438	-1.618926536	0.105463071	0.218501439
ENSG00000267199	10.61089683	-0.601353997	0.534873874	-1.124291212	0.260889505	0.427162401
ENSG00000267213	13.59273184	0.685764369	0.475121115	1.443346438	0.148922904	0.283496414
ENSG00000267216	16.97423424	-0.288413619	0.415431314	-0.694251035	0.487524792	0.64779673
ENSG00000267248	7.520974737	-0.876232741	0.643994318	-1.360621851	0.173633223	0.317581495
ENSG00000267265	16.71092354	-0.218189805	0.43579918	-0.500665936	0.61660625	0.750779831
ENSG00000267270	49.94126551	-0.339919133	0.263825919	-1.288422057	0.197599085	0.349141427
ENSG00000267272	20.29069059	-0.201187802	0.415193426	-0.484564037	0.62798563	0.758692194
ENSG00000267278	9.892724514	0.773790339	0.548953346	1.409573955	0.158665522	0.297090721
ENSG00000267280	58.00417855	-0.254455301	0.247827712	-1.02674273	0.304541623	0.475480291
ENSG00000267296	32.93926408	-0.776424203	0.306557444	-2.532720112	0.011318129	0.035823738

ENSG00000267317	35.41370957	-0.236401996	0.326039774	-0.725071034	0.468408454	0.630503404
ENSG00000267321	53.84196144	-0.267501004	0.242492592	-1.103130621	0.269970445	0.437301066
ENSG00000267322	217.1967408	0.562349847	0.338154818	1.662995222	0.096313392	0.20372146
ENSG00000267325	14.44547056	-6.141477618	1.233616782	-4.978432288	6.41E-07	5.93E-06
ENSG00000267327	6.021380085	-5.871750897	1.310204252	-4.481553841	7.41E-06	5.46E-05
ENSG00000267348	13.38812319	-0.191214903	0.485108507	-0.394169345	0.693456016	0.807174069
ENSG00000267374	76.87174127	-0.634370623	0.212970347	-2.978680512	0.002894925	0.01119161
ENSG00000267397	15.65171415	-0.644014752	0.457775741	-1.406834601	0.15947644	0.298195324
ENSG00000267430	8.835759796	0.007842386	0.583382834	0.013442949	0.989274402	0.994711464
ENSG00000267432	18.49423414	0.330573491	0.395720032	0.835372143	0.40350821	0.571732068
ENSG00000267475	12.47467968	-0.055731744	0.520778865	-0.10701614	0.914776176	0.953830969
ENSG00000267493	14.38910402	0.247891221	0.458380241	0.540798225	0.588646666	0.730091746
ENSG00000267508	22.29583716	-0.350281096	0.377121287	-0.928828755	0.35297784	0.524343746
ENSG00000267519	58.38350521	-0.679477133	0.244574774	-2.778197944	0.005466131	0.019285429
ENSG00000267526	15.16811292	-0.208862297	0.443027741	-0.471442932	0.637324464	0.765956258
ENSG00000267534	92.05265159	-0.041283147	0.189286111	-0.218099187	0.827351831	0.898686295
ENSG00000267541	11.06091334	-1.034160692	0.534424671	-1.935091601	0.052979056	0.127540154
ENSG00000267575	179.5464466	0.077578752	0.138416514	0.560473241	0.575156687	0.71949648
ENSG00000267577	8.270779448	2.558157152	0.720429962	3.550875571	0.000383952	0.00191594
ENSG00000267587	11.34382309	0.17227859	0.531497475	0.324138115	0.745833472	0.845033294
ENSG00000267632	14.65695904	0.251961624	0.456665098	0.551742677	0.581124666	0.723664022
ENSG00000267680	75.60230471	0.173122756	0.219743219	0.787841178	0.430789612	0.597477385
ENSG00000267698	9.319221867	0.449882457	0.557278472	0.80728483	0.419502402	0.587278278
ENSG00000267751	146.9669032	-0.232862644	0.151756092	-1.534453348	0.124918162	0.249318239
ENSG00000267787	24.8514318	-0.293891487	0.360381566	-0.815500886	0.414785657	0.582882566
ENSG00000267796	13.63466375	0.645558548	0.496933092	1.299085446	0.193914607	0.34476587
ENSG00000267858	31.73577682	-0.185350563	0.305462286	-0.606787064	0.543992234	0.692782685
ENSG00000267904	21.82888327	0.202397549	0.376806382	0.537139386	0.591171336	0.732033124
ENSG00000268006	12.41274182	-0.424043242	0.515090088	-0.823240929	0.410370998	0.578821309
ENSG00000268043	93.22668331	0.035529577	0.197955476	0.179482666	0.857558727	0.918429967
ENSG00000268049	21.59988335	-0.404803272	0.3757526	-1.077313298	0.281340324	0.450317631
ENSG00000268061	12.62612181	-1.031356677	0.528078371	-1.953037151	0.050815193	0.1233372
ENSG00000268129	8.075238855	0.296457874	0.630320253	0.470328968	0.638120005	0.766295498
ENSG00000268205	144.9649881	0.174819731	0.153195584	1.141153854	0.253805904	0.418674985
ENSG00000268222	9.043216979	0.300744253	0.596138354	0.504487341	0.613918956	0.748716754
ENSG00000268362	167.0452753	-0.414495683	0.144186672	-2.874715646	0.004043915	0.014944719
ENSG00000268364	18.19488897	-0.533615491	0.408834699	-1.305210865	0.191821056	0.342028113
ENSG00000268403	20.06833078	0.342472917	0.381561626	0.897555976	0.369422318	0.540887566
ENSG00000268471	8.163345086	-0.470464494	0.620946708	-0.757656797	0.448656469	0.61365895
ENSG00000268516	94.23610085	0.155394851	0.182235564	0.852714188	0.393817821	0.561848357
ENSG00000268573	16.96484928	0.858945152	0.427341377	2.009974224	0.044433917	0.11112039
ENSG00000268575	14.94571659	-0.397131417	0.446133235	-0.890163265	0.373378226	0.54353703
ENSG00000268858	55.9350406	-0.239590403	0.232373308	-1.031058194	0.302513531	0.47315528
ENSG00000268895	42.32766728	-0.395758182	0.272502324	-1.452311219	0.14641509	0.280050757
ENSG00000268903	24.11460537	0.031391545	0.372739928	0.084218359	0.932882822	0.963270344
ENSG00000268996	10.91396197	0.715875754	0.555147343	1.289523878	0.197216021	0.34864
ENSG00000269044	31.69629626	-0.469828114	0.326634126	-1.438392615	0.150322687	0.285619205
ENSG00000269069	22.81462509	-0.732963079	0.388554214	-1.886385614	0.05924301	0.13934805
ENSG00000269151	10.48921075	-1.108066197	0.548505651	-2.020154567	0.043367357	0.109133154
ENSG00000269176	14.81568023	0.295612308	0.457418149	0.646262743	0.518109212	0.673067613

ENSG00000269190	467.9457109	0.158957317	0.093618184	1.697932077	0.089520582	0.192542585
ENSG00000269293	61.01582083	0.160158998	0.221543857	0.722922314	0.469727614	0.631654585
ENSG00000269313	11.81846638	0.130713063	0.495177433	0.263972173	0.791801361	0.875713229
ENSG00000269335	21.71170407	-0.585436396	0.379352435	-1.543251981	0.12276965	0.246340767
ENSG00000269343	94.73536549	0.259912791	0.18528475	1.402774869	0.160683983	0.299870853
ENSG00000269352	51.87491891	0.214898048	0.240764772	0.892564329	0.372090529	0.542757597
ENSG00000269374	20.61343479	-0.087502585	0.400442269	-0.218514856	0.827027985	0.898668717
ENSG00000269386	34.47668316	0.17112211	0.298039822	0.574158545	0.565860519	0.711478939
ENSG00000269416	10.94175907	-2.880451353	0.69619258	-4.137434719	3.51E-05	0.000223739
ENSG00000269439	15.65477926	-0.295764293	0.441733067	-0.669554342	0.503141928	0.660933822
ENSG00000269473	13.47079405	-0.075076606	0.475387625	-0.157927135	0.874514216	0.930487895
ENSG00000269486	23.10928473	-0.241616517	0.374138468	-0.645794371	0.518412534	0.673338255
ENSG00000269514	11.22222756	-0.433851223	0.520401978	-0.833684807	0.404458622	0.57249398
ENSG00000269556	31.06139114	-0.012627192	0.314112556	-0.040199576	0.967934016	0.983065728
ENSG00000269609	94.01093203	0.186477685	0.195456236	0.954063623	0.340051438	0.511753006
ENSG00000269688	14.88028603	0.328937951	0.465937094	0.705970732	0.480206339	0.640808597
ENSG00000269713	125.3408079	0.189783939	0.16737902	1.133857391	0.256854373	0.422535821
ENSG00000269720	18.1929914	1.840631435	0.436147519	4.220203846	2.44E-05	0.000160968
ENSG00000269743	39.41748189	-0.105696028	0.281736243	-0.375159499	0.707541849	0.816600144
ENSG00000269821	4264.242229	-0.599933478	0.083352508	-7.197545635	6.13E-13	1.24E-11
ENSG00000269825	46.51027747	0.394410888	0.254387461	1.550433682	0.12103746	0.243478905
ENSG00000269837	9.431632569	-0.895544206	0.575605512	-1.555829795	0.119748571	0.241508712
ENSG00000269858	83.75921611	0.30901171	0.191403236	1.61445395	0.106428998	0.2202428
ENSG00000269893	247.7597062	0.158645922	0.122472742	1.295356988	0.195197109	0.346334948
ENSG00000269918	13.25772845	-0.685524328	0.483471179	-1.417921807	0.156213595	0.293831329
ENSG00000269929	119.9357934	-0.592613984	0.189141111	-3.133184429	0.001729208	0.00722922
ENSG00000269937	20.96755561	0.089549055	0.392135039	0.228362799	0.819364207	0.893395096
ENSG00000269951	9.308646485	0.25833554	0.558136794	0.462853449	0.643469417	0.769990566
ENSG00000269958	41.69564624	-0.112291165	0.277973625	-0.403963378	0.686239649	0.801964089
ENSG00000269967	7.26428327	-0.17823613	0.684547378	-0.260370773	0.794577785	0.877609227
ENSG00000269968	20.51274308	0.167271663	0.391442564	0.427321089	0.669145474	0.788596542
ENSG00000269973	31.18822071	-0.599335528	0.314669208	-1.904652611	0.056825243	0.134801789
ENSG00000270006	10.77882618	-1.379199675	0.553461455	-2.491952531	0.012704302	0.039578027
ENSG00000270012	10.91049758	-0.709137187	0.536549423	-1.321662378	0.186280602	0.334443202
ENSG00000270019	9.449337084	-0.330744413	0.56486598	-0.58552723	0.558193262	0.705122829
ENSG00000270020	8.737543706	-0.622701965	0.599138522	-1.039328874	0.298651811	0.469182736
ENSG00000270049	10.90439751	0.536608369	0.53948495	0.994667911	0.319897806	0.491587844
ENSG00000270061	8.586065723	-0.003505278	0.603222577	-0.00581092	0.995363583	0.997940364
ENSG00000270069	57.38541474	-0.529862531	0.238783128	-2.219011602	0.026485935	0.072793569
ENSG00000270072	12.39170933	0.033959333	0.544927998	0.062318937	0.950308849	0.972594511
ENSG00000270140	9.420345097	0.294344691	0.600856312	0.489875342	0.624222113	0.755965861
ENSG00000270170	147.8791565	-0.104034003	0.154976637	-0.671288295	0.50203689	0.66031013
ENSG00000270175	15.69426366	-0.572446766	0.43879521	-1.304587545	0.192033332	0.342321347
ENSG00000270194	19.84018487	0.528115974	0.409046207	1.291091238	0.196672042	0.347928616
ENSG00000270225	29.21324754	0.049186511	0.330337231	0.148897873	0.881634219	0.934792614
ENSG00000270231	75.57069098	0.101354369	0.204042378	0.496731954	0.619378086	0.752890617
ENSG00000270307	35.77678724	-0.214470242	0.290307779	-0.7387685	0.460047582	0.623291704
ENSG00000270344	91.43688988	-1.885213297	0.216129006	-8.722629767	2.72E-18	8.37E-17
ENSG00000270350	189.0353576	-0.411230022	0.15655001	-2.626828463	0.008618477	0.028490657
ENSG00000270362	21.22480335	-0.610424435	0.396210803	-1.54065571	0.123400602	0.247209663

ENSG00000270388	146.8925505	-0.419821387	0.152470874	-2.753453009	0.005897024	0.020570025
ENSG00000270441	8.43076888	-1.051233969	0.627767557	-1.67455925	0.09402074	0.199925725
ENSG00000270558	30.86162947	-0.35031591	0.327537524	-1.069544356	0.284824453	0.454408643
ENSG00000270574	11.80515449	-0.249541564	0.499983058	-0.49910004	0.61770891	0.751514768
ENSG00000270605	9.042510797	-0.367493603	0.5720771	-0.642384746	0.520623401	0.674960491
ENSG00000270629	27.15798062	0.125654771	0.335524909	0.374502066	0.70803082	0.81689601
ENSG00000270647	2522.61742	0.284381084	0.078612729	3.617494125	0.000297469	0.001527806
ENSG00000270722	134.9540592	0.160805326	0.162371305	0.990355568	0.322000355	0.493846379
ENSG00000270804	12.13026323	0.21676493	0.50086681	0.432779585	0.665174906	0.785780162
ENSG00000270885	236.1290421	0.260740394	0.132503976	1.967792985	0.049091862	0.120221998
ENSG00000270906	66.08153073	-0.441483133	0.223559514	-1.974790176	0.048291964	0.118801615
ENSG00000270959	11.54932092	0.022351088	0.499896572	0.044711425	0.964337327	0.981313129
ENSG00000270977	25.79827224	0.248206573	0.34630373	0.716730869	0.473540175	0.634776108
ENSG00000271013	27.88990348	0.139841347	0.326413872	0.428417293	0.668347337	0.788069759
ENSG00000271074	12.6933394	-2.930290075	0.647800932	-4.523442201	6.08E-06	4.58E-05
ENSG00000271122	27.92268977	-0.408190516	0.351470738	-1.161378378	0.245488058	0.409010883
ENSG00000271147	18.17410266	0.342882746	0.4033636	0.85005872	0.395292441	0.563217718
ENSG00000271204	98.46989818	1.208513766	0.202240842	5.975616767	2.29E-09	3.06E-08
ENSG00000271207	7.702493337	0.088988276	0.619531117	0.143638105	0.885786249	0.937510506
ENSG00000271254	41.73374561	-0.191720462	0.273091818	-0.702036637	0.482656316	0.643251733
ENSG00000271270	53.50354101	-0.223091837	0.251545738	-0.886883788	0.375141471	0.545113504
ENSG00000271303	33.11839377	0.482536808	0.329478985	1.464545025	0.14304509	0.275291736
ENSG00000271380	9.315248111	-0.617348087	0.60898802	-1.013727802	0.310712654	0.482114288
ENSG00000271425	15.49988809	-0.044265174	0.432330849	-0.102387266	0.918449291	0.955355281
ENSG00000271533	35.33982688	0.005321515	0.300373463	0.017716328	0.985865155	0.992629448
ENSG00000271601	431.9000662	-0.037634549	0.098015659	-0.383964661	0.70100464	0.812392722
ENSG00000271615	9.139573757	-5.463604634	1.263764677	-4.323276899	1.54E-05	0.000105751
ENSG00000271643	43.8278811	-0.24109561	0.27132229	-0.888594925	0.374220819	0.544273699
ENSG00000271687	13.59829851	-0.379520172	0.487325523	-0.778781642	0.436108353	0.602183627
ENSG00000271754	8.968054424	-0.90299358	0.606075729	-1.489902229	0.136249945	0.265805225
ENSG00000271780	26.15661414	0.821638728	0.353492999	2.324342292	0.020107156	0.058032714
ENSG00000271781	28.01898924	-1.14256755	0.348861584	-3.275131463	0.001056128	0.004718649
ENSG00000271811	7.921879635	-6.267855097	1.28492389	-4.877997169	1.07E-06	9.41E-06
ENSG00000271852	9.746891949	0.178494336	0.548252974	0.325569298	0.744750244	0.844210781
ENSG00000271862	9.919144901	-0.705082676	0.564412467	-1.249232994	0.211579868	0.366514046
ENSG00000271918	17.60841744	0.854559893	0.431118681	1.98219175	0.047457784	0.116995347
ENSG00000271936	51.18130186	-0.332286549	0.249186956	-1.333482916	0.182373378	0.329357128
ENSG00000271943	16.07397855	-0.822647955	0.436556349	-1.884402682	0.059510527	0.139836798
ENSG00000271976	29.49597546	-0.631906169	0.329335847	-1.918728781	0.055018667	0.131570857
ENSG00000271984	7.195240915	0.033837213	0.653248605	0.05179837	0.958689354	0.977761516
ENSG00000272031	213.6726117	0.494610028	0.131136112	3.771730147	0.00016212	0.000886796
ENSG00000272033	20.39117685	-0.424283041	0.380578412	-1.114837384	0.264920113	0.431659069
ENSG00000272046	50.22353377	0.115555598	0.256918185	0.44977586	0.652872066	0.777194399
ENSG00000272047	208.1765323	0.065671404	0.127184652	0.516346928	0.605612126	0.74264806
ENSG00000272068	34.2855805	-1.044280682	0.336972328	-3.099010205	0.001941683	0.007979762
ENSG00000272077	56.80147502	-0.209918272	0.23224484	-0.903866251	0.366066327	0.537374197
ENSG00000272086	9.279038885	-0.958579885	0.586923103	-1.633229089	0.102420824	0.21372934
ENSG00000272137	31.852531	-1.758567359	0.356299368	-4.935645459	7.99E-07	7.24E-06
ENSG00000272142	40.26117751	-0.18399797	0.28355076	-0.648906634	0.516398726	0.67134224
ENSG00000272145	21.41660608	-0.353972594	0.372265294	-0.950861119	0.341674882	0.513645876

ENSG00000272153	7.423724685	-1.035711517	0.67842311	-1.526645395	0.126849212	0.252037117
ENSG00000272195	12.71034155	-0.655433333	0.491862197	-1.332554803	0.182677946	0.329787573
ENSG00000272219	10.55512038	-0.582721585	0.58366272	-0.998387535	0.318091476	0.48988389
ENSG00000272273	34.54916884	0.197423351	0.303345422	0.650820275	0.515162511	0.670852674
ENSG00000272293	16.41742117	0.763514172	0.443446136	1.721774326	0.085110407	0.18526978
ENSG00000272316	9.915147826	0.274325507	0.541264211	0.506823657	0.612278556	0.747774297
ENSG00000272325	484.0695224	0.0254928	0.094380232	0.270107407	0.787077624	0.872399068
ENSG00000272333	747.2224334	0.119811346	0.081362613	1.472560205	0.140869662	0.272074267
ENSG00000272335	32.92663685	-0.966376209	0.314260776	-3.075077395	0.002104479	0.008514292
ENSG00000272338	23.66245395	-0.348256846	0.370610461	-0.939684339	0.347379501	0.519221095
ENSG00000272374	19.1487239	-0.618717244	0.418176652	-1.479559513	0.138990838	0.269606605
ENSG00000272391	595.8044559	0.068178292	0.087925292	0.775411607	0.438096479	0.603564264
ENSG00000272405	155.4043661	-0.676063057	0.149947315	-4.508670634	6.52E-06	4.86E-05
ENSG00000272434	8.588839496	0.6608495	0.599471219	1.102387369	0.270293301	0.43772308
ENSG00000272462	9.432364679	0.057196081	0.566472923	0.100968782	0.919575242	0.955843823
ENSG00000272476	9.73847031	0.321971067	0.57801292	0.557030917	0.577506305	0.721215423
ENSG00000272491	9.732879868	0.548932403	0.549054073	0.999778402	0.31741776	0.489168105
ENSG00000272512	22.02560785	-0.674513402	0.405287641	-1.664283174	0.096055853	0.203299167
ENSG00000272518	23.95558276	-0.874564127	0.379855034	-2.302362875	0.021314717	0.060842883
ENSG00000272533	26.00746002	-0.700128362	0.348038021	-2.011643328	0.044257549	0.11079768
ENSG00000272599	16.97064209	-0.150116369	0.430909827	-0.348370726	0.727561785	0.831595187
ENSG00000272604	10.04080756	0.490984257	0.539664037	0.909796139	0.362930031	0.533886077
ENSG00000272610	22.1650684	-0.672613647	0.3777839	-1.780419035	0.075007409	0.167455352
ENSG00000272631	23.51601579	0.141435746	0.356927071	0.396259509	0.691913594	0.806113483
ENSG00000272636	44.57540118	-0.729355222	0.26998262	-2.701489533	0.006902965	0.023501201
ENSG00000272674	12.68850127	2.888248028	0.615307338	4.693992497	2.68E-06	2.18E-05
ENSG00000272686	10.52563845	0.285519355	0.545738195	0.523180084	0.600848939	0.739194697
ENSG00000272688	21.5449292	-0.442537214	0.370433612	-1.19464649	0.232225177	0.392122499
ENSG00000272690	13.71034933	-1.643170823	0.504114145	-3.25952136	0.001116004	0.004948392
ENSG00000272701	10.0281585	0.079098283	0.566990444	0.139505495	0.889050714	0.93933866
ENSG00000272711	44.7564361	-0.200631098	0.268644034	-0.746828787	0.455166916	0.618887782
ENSG00000272758	19.1941692	0.326996051	0.397991821	0.821615003	0.411296048	0.579707608
ENSG00000272763	54.5589654	-0.083031824	0.260440619	-0.318812879	0.749868411	0.848168256
ENSG00000272767	50.01730185	-2.707559837	0.315260092	-8.58833677	8.82E-18	2.58E-16
ENSG00000272808	16.67002493	-0.138059446	0.424214096	-0.325447569	0.744842358	0.844210781
ENSG00000272817	11.84432826	-0.247719487	0.500811512	-0.494636169	0.620856966	0.753970589
ENSG00000272831	15.80718424	-0.098064603	0.429139026	-0.228514764	0.81924608	0.893395096
ENSG00000272841	17.4204507	0.720638374	0.411922077	1.749453146	0.080212721	0.176883531
ENSG00000272870	8.741552941	1.241686393	0.621865217	1.996713048	0.045856364	0.113951132
ENSG00000272871	8.555620265	0.426128168	0.582917661	0.731026346	0.464763061	0.627441299
ENSG00000272886	625.4593735	-0.016491772	0.084454993	-0.195272908	0.845179305	0.910377555
ENSG00000272888	204.1604537	-0.024563143	0.130661434	-0.187990769	0.850883887	0.914488597
ENSG00000272899	15.48516637	0.476825607	0.449999968	1.059612536	0.289320908	0.459478891
ENSG00000272918	21.06868858	0.252804679	0.387696993	0.652067679	0.514357513	0.670177163
ENSG00000272983	31.69520445	-0.147299479	0.326714459	-0.450850811	0.652097077	0.776439474
ENSG00000272994	26.81960178	-0.211824319	0.33764205	-0.627363561	0.530420948	0.68262236
ENSG00000273014	14.31267829	-1.271006374	0.484779371	-2.621824384	0.008746049	0.028825843
ENSG00000273015	73.13526967	0.365111398	0.212399348	1.718985499	0.085617012	0.186128115
ENSG00000273032	11.05890894	-0.435472013	0.52208059	-0.834108797	0.404219678	0.57241129
ENSG00000273033	40.8692638	-0.661732878	0.292100318	-2.26543019	0.023486293	0.065785038

ENSG00000273058	26.81445952	-0.252051414	0.357389362	-0.705257182	0.480650201	0.641064696
ENSG00000273061	14.89654635	0.975422187	0.451311014	2.161308184	0.030671541	0.082012793
ENSG00000273066	31.19323439	0.830252367	0.345111579	2.405750541	0.016139281	0.048441983
ENSG00000273080	10.35412906	0.034224484	0.565727072	0.060496457	0.951760237	0.973475269
ENSG00000273084	24.70062449	-1.45574655	0.392822967	-3.70585906	0.000210675	0.001122244
ENSG00000273117	12.96762354	0.459766485	0.481816769	0.954235125	0.339964639	0.511717865
ENSG00000273136	14.59784479	0.871220012	0.468025664	1.861479144	0.062676543	0.145788608
ENSG00000273142	42.6310247	0.040481166	0.30709486	0.131819745	0.895126869	0.943073102
ENSG00000273145	18.99556029	0.004388613	0.412612996	0.010636148	0.991513741	0.995624671
ENSG00000273156	18.98477038	-0.729811174	0.416087411	-1.753985231	0.079433038	0.175467193
ENSG00000273162	11.21681571	-1.68456616	0.566178094	-2.975329099	0.002926744	0.011297601
ENSG00000273173	7.38854845	0.208238304	0.646065556	0.322317607	0.747212096	0.846027998
ENSG00000273175	14.52955525	0.348086232	0.519243075	0.67037241	0.502620418	0.660644616
ENSG00000273199	8.277785914	-1.062752591	0.640450144	-1.659383794	0.09703848	0.204731079
ENSG00000273203	35.53726315	-0.214822817	0.323671143	-0.663707041	0.506877832	0.664065744
ENSG00000273230	12.55139207	-0.78437185	0.494515837	-1.586141012	0.112707325	0.230250116
ENSG00000273247	37.20808877	0.41317205	0.288771637	1.430791659	0.152489934	0.288721675
ENSG00000273270	19.99198333	-0.323941347	0.388347591	-0.8341531	0.404194716	0.57241129
ENSG00000273271	8.282686191	0.038255455	0.593729708	0.064432442	0.948625899	0.971432914
ENSG00000273274	9.893314479	-0.520852036	0.550152441	-0.946741297	0.343770621	0.515604085
ENSG00000273297	20.17833908	-0.727863656	0.420509862	-1.730907458	0.083468271	0.182686009
ENSG00000273306	18.73081141	0.136014872	0.41914699	0.324503993	0.7455565	0.844855772
ENSG00000273311	23.46439776	-0.086734165	0.357928657	-0.242322493	0.808530286	0.886947088
ENSG00000273314	11.10686572	0.429573669	0.560879582	0.765892863	0.443740053	0.608593685
ENSG00000273320	11.38592383	0.672096526	0.504687921	1.331707174	0.182956431	0.330035827
ENSG00000273329	95.99814992	-0.073160252	0.185581022	-0.3942227	0.693416627	0.807174069
ENSG00000273338	64.00431073	-0.057064804	0.245938155	-0.232029079	0.816515428	0.892216525
ENSG00000273344	81.90004919	0.5405219	0.198977274	2.716500678	0.006597606	0.022626116
ENSG00000273373	42.18675448	-0.395404165	0.270379463	-1.462404578	0.143630376	0.276039935
ENSG00000273382	10.74313264	-0.444625345	0.541914129	-0.820471955	0.411947111	0.58028691
ENSG00000273391	13.01322194	0.612575857	0.496375774	1.234097008	0.217166758	0.37397294
ENSG00000273419	11.48564417	-1.02818655	0.539840083	-1.904613204	0.056830369	0.134801789
ENSG00000273442	9.127302817	-0.936854	0.607352197	-1.542521793	0.122946846	0.246578291
ENSG00000273451	8.878944408	-1.030544745	0.59366077	-1.735915185	0.082578832	0.181021358
ENSG00000273559	184.2452399	0.099676133	0.150385691	0.662803304	0.507456537	0.664432322
ENSG00000273576	11.50033704	-0.666871355	0.510934537	-1.305199211	0.191825023	0.342028113
ENSG00000273599	13.47144892	-0.677018822	0.479099367	-1.413107317	0.157624172	0.295771673
ENSG00000273604	254.1066843	-0.394913066	0.126289149	-3.127054602	0.001765671	0.007348855
ENSG00000273611	110.7738426	0.081654573	0.169846442	0.480755277	0.63069044	0.760912875
ENSG00000273619	15.18817188	-0.154824983	0.444981335	-0.347935903	0.727888321	0.83181315
ENSG00000273691	16.12762054	0.097543683	0.443871876	0.219756393	0.826060882	0.898326021
ENSG00000273702	23.11832986	0.401427658	0.37767105	1.062902908	0.287825984	0.457829419
ENSG00000273703	1957.477409	-1.030902164	0.071412061	-14.43596718	3.07E-47	2.96E-45
ENSG00000273727	22.93180833	0.468934298	0.359202052	1.305488916	0.191726419	0.341947817
ENSG00000273729	9.9393239	-0.444266917	0.551815452	-0.805100537	0.420761666	0.588373639
ENSG00000273748	18.6182492	-0.050117072	0.397556715	-0.126062697	0.899682295	0.945875099
ENSG00000273749	1209.217688	0.130977611	0.07532411	1.738853745	0.082060492	0.18016626
ENSG00000273759	15.09437228	0.083123104	0.446493819	0.186168543	0.852312592	0.915251049
ENSG00000273802	432.3413137	-0.116579747	0.103507081	-1.126297312	0.260039695	0.426128978
ENSG00000273805	10.21296032	-0.60427501	0.547063434	-1.104579419	0.269341873	0.43653459

ENSG00000273820	62.13022803	-0.173664817	0.231016671	-0.75174149	0.452206534	0.616965713
ENSG00000273841	412.8571113	0.001336436	0.101968796	0.013106323	0.989542966	0.994744093
ENSG00000273899	29.49684719	-0.056077186	0.324833501	-0.172633628	0.862939417	0.922223828
ENSG00000273983	483.1010258	-0.787526528	0.100346983	-7.848033913	4.23E-15	1.02E-13
ENSG00000274015	8.85452251	-0.670318639	0.59204125	-1.132216107	0.25754359	0.423322585
ENSG00000274020	16.34183358	-0.797749626	0.44160052	-1.806496122	0.070840879	0.160500295
ENSG00000274021	8.829309506	-1.286465537	0.614214391	-2.09448941	0.036216405	0.094087636
ENSG00000274023	11.16169625	-1.200863466	0.566198179	-2.120924282	0.033928175	0.089115384
ENSG00000274070	158.4310783	-1.253592304	0.150888662	-8.308061641	9.73E-17	2.64E-15
ENSG00000274080	13.90885849	-0.058732753	0.460013931	-0.127676032	0.898405358	0.944886199
ENSG00000274124	9.113509405	-0.608627782	0.586170212	-1.03831237	0.299124652	0.469527766
ENSG00000274180	59.80551712	-0.155268009	0.224813547	-0.690652368	0.489784029	0.649485156
ENSG00000274210	169.4518606	1.041249937	0.152369493	6.833716632	8.27E-12	1.47E-10
ENSG00000274211	289.202099	-0.180099396	0.120332507	-1.49668116	0.134476241	0.263163521
ENSG00000274213	10.25290318	0.802596335	0.554656343	1.447015516	0.147892578	0.282031909
ENSG00000274238	7.664993677	-2.858422911	0.818916045	-3.490495673	0.000482125	0.002345019
ENSG00000274253	29.47508805	-2.075303887	0.370928936	-5.594882695	2.21E-08	2.59E-07
ENSG00000274270	8.989144403	-0.037468061	0.569590587	-0.065780688	0.947552432	0.970914655
ENSG00000274276	16.4556424	-3.015105478	0.586590587	-5.140050907	2.75E-07	2.70E-06
ENSG00000274290	580.1388754	-0.058921206	0.092963492	-0.633810162	0.52620472	0.679374789
ENSG00000274292	17.72887359	-0.430654096	0.424609527	-1.014235595	0.310470348	0.481844765
ENSG00000274308	20.73062393	-0.389340839	0.382249373	-1.018551935	0.308415733	0.479662954
ENSG00000274317	6.047975965	-4.879416246	1.307839696	-3.7308978	0.000190799	0.001025015
ENSG00000274349	15.21852526	0.399501605	0.470055591	0.84990289	0.395379079	0.563217718
ENSG00000274383	56.82502408	0.481386743	0.259420569	1.855622875	0.063507331	0.147330714
ENSG00000274422	20.37104495	-0.348240599	0.389611023	-0.893816084	0.371420302	0.54217406
ENSG00000274425	30.70948005	-0.334916773	0.319164399	-1.049355048	0.294014739	0.464360809
ENSG00000274428	224.2656328	0.165255525	0.136440729	1.211189111	0.225822935	0.384268294
ENSG00000274523	449.6434085	-0.054519401	0.096362192	-0.565775851	0.571546164	0.716690513
ENSG00000274565	11.38743059	0.434200177	0.539442992	0.804904657	0.420874701	0.588378475
ENSG00000274605	42.09712144	-0.024584693	0.265587095	-0.092567346	0.926247286	0.959654494
ENSG00000274641	673.2631881	-0.373441386	0.098216677	-3.802219737	0.000143405	0.000797157
ENSG00000274677	7.400469144	-0.630397433	0.633034381	-0.995834432	0.319330597	0.490931039
ENSG00000274750	174.8830306	0.09391555	0.142143023	0.660711645	0.508797254	0.665497779
ENSG00000274828	10.29548639	-0.025938791	0.538032962	-0.048210412	0.961548552	0.979684997
ENSG00000274925	26.25598361	-1.046636454	0.350884908	-2.982848303	0.002855795	0.01107547
ENSG00000274964	10.35155746	-0.834952083	0.553472495	-1.508570146	0.131408668	0.258819064
ENSG00000274997	730.9156848	-0.182266234	0.096966513	-1.879682254	0.060151396	0.141106656
ENSG00000275004	115.9604447	-0.176514326	0.169850848	-1.039231348	0.298697155	0.469182736
ENSG00000275023	908.7277829	0.010120025	0.076824748	0.131728716	0.895198871	0.943073102
ENSG00000275029	29.05709507	-0.353009613	0.325790684	-1.083547291	0.278565572	0.447405738
ENSG00000275038	17.94717401	-5.602194843	1.053891976	-5.31572018	1.06E-07	1.11E-06
ENSG00000275052	1058.317559	0.281448158	0.076461378	3.680919242	0.000232395	0.001222887
ENSG00000275064	6.481773448	-5.976879128	1.297813901	-4.605343742	4.12E-06	3.20E-05
ENSG00000275066	284.1700641	-0.25421816	0.119058768	-2.135232569	0.032742018	0.086501246
ENSG00000275074	15.34325802	0.179052608	0.44300044	0.404181558	0.686079214	0.801843308
ENSG00000275111	38.32914698	0.34559653	0.282478213	1.2234449	0.221161703	0.378906994
ENSG00000275131	14.03209824	0.611439334	0.480643403	1.272126758	0.203328085	0.356350545
ENSG00000275180	13.71152401	-0.442978594	0.469392641	-0.943727181	0.345309081	0.517089495
ENSG00000275183	30.1878444	2.388139293	0.370915088	6.438506731	1.21E-10	1.89E-09

ENSG00000275221	199.9727412	-0.253856949	0.136433651	-1.86066228	0.062791884	0.145936067
ENSG00000275291	27.04803469	-0.319467651	0.336019908	-0.950740249	0.341736251	0.513683158
ENSG00000275318	16.11006014	-0.053767508	0.42316282	-0.127061041	0.898892087	0.94532733
ENSG00000275342	54.95529847	-0.091554237	0.237320424	-0.385783218	0.699657225	0.811562424
ENSG00000275395	18.7150919	-0.950719834	0.414897777	-2.291455598	0.021937077	0.062263317
ENSG00000275401	8.901364522	-0.828250577	0.681105942	-1.21603781	0.223970519	0.382273862
ENSG00000275468	22.43510874	-0.447667422	0.382213642	-1.171249199	0.241498625	0.404089479
ENSG00000275481	24.04499368	-1.097166964	0.371085554	-2.956641539	0.003110095	0.011907195
ENSG00000275494	6.935059484	-0.712869514	0.687302906	-1.037198457	0.299643377	0.470079352
ENSG00000275591	33.13076783	-2.279035897	0.372160919	-6.123791572	9.14E-10	1.28E-08
ENSG00000275632	50.47590014	-0.503555086	0.253520578	-1.986249359	0.047005643	0.116186567
ENSG00000275700	636.8371566	0.348277059	0.091125036	3.821968964	0.00013239	0.000742094
ENSG00000275713	740.5242597	-0.360540978	0.095738751	-3.76588346	0.000165961	0.000905342
ENSG00000275719	11.19504959	-0.606633349	0.530939312	-1.142566271	0.253218716	0.417974537
ENSG00000275734	11.10165698	-0.476425957	0.528943314	-0.900712693	0.367741098	0.539135055
ENSG00000275764	16.01346972	0.326429389	0.44845797	0.727892936	0.466679128	0.628895314
ENSG00000275832	83.47642218	0.214982536	0.192604978	1.116183691	0.264343514	0.431019541
ENSG00000275835	261.7589549	-0.118374664	0.118694149	-0.997308339	0.318614867	0.490203497
ENSG00000275888	9.558571123	1.081788234	0.58288924	1.855907023	0.063466812	0.1473097
ENSG00000275895	11.3587202	0.854782243	0.520113848	1.643452192	0.100289409	0.210198391
ENSG00000276007	8.970283179	-1.559878736	0.630759823	-2.473015368	0.013397838	0.041389803
ENSG00000276023	146.7966494	0.334173165	0.154575988	2.161869834	0.030628211	0.081933818
ENSG00000276043	665.5702175	0.041619066	0.085431488	0.487163067	0.626142776	0.757381067
ENSG00000276045	176.5945402	-0.240247319	0.142770475	-1.682752116	0.092423088	0.197365023
ENSG00000276047	13.93080356	-0.424250169	0.469780861	-0.903080998	0.366482908	0.537817114
ENSG00000276168	85562.07874	-0.801924023	0.17383479	-4.613138851	3.97E-06	3.10E-05
ENSG00000276180	8.28654158	-1.538189723	0.656214882	-2.344033587	0.019076453	0.055527683
ENSG00000276234	102.4052412	-0.05093166	0.18857654	-0.270084814	0.787095005	0.872399068
ENSG00000276248	16.67433881	-0.284452191	0.423864332	-0.671092541	0.502161578	0.660354498
ENSG00000276255	7.996625092	-1.331071457	0.645906319	-2.06078098	0.03932394	0.100415085
ENSG00000276256	11.91435295	-0.78905011	0.520997631	-1.51449846	0.129899464	0.256489013
ENSG00000276293	510.564506	-0.011195694	0.091965785	-0.1217376	0.903106834	0.947702203
ENSG00000276334	22.59723917	-0.335871999	0.367593187	-0.913705725	0.360871495	0.532058728
ENSG00000276368	566.6648242	0.059875797	0.559166742	0.107080398	0.914725198	0.953830969
ENSG00000276399	29.55968421	1.120132259	0.330684181	3.387317332	0.000705797	0.003295988
ENSG00000276434	10.64047474	0.011006602	0.527425823	0.020868531	0.983350529	0.991939727
ENSG00000276449	10.91854706	0.65882003	0.542216521	1.215049717	0.22434713	0.382637705
ENSG00000276517	48.59271584	-0.433737787	0.249040474	-1.741635726	0.081572206	0.179402658
ENSG00000276529	22.39219242	0.132184065	0.377022753	0.350599702	0.72588868	0.830406246
ENSG00000276542	10.03105778	-4.189144961	0.952770523	-4.3968037	1.10E-05	7.81E-05
ENSG00000276545	13.41244066	-0.36704224	0.471547066	-0.778378801	0.436345732	0.602452182
ENSG00000276564	12.80901024	0.417165826	0.501206756	0.832322831	0.405226747	0.573465658
ENSG00000276570	10.54645864	-0.170783433	0.560392888	-0.304756603	0.760551536	0.855365062
ENSG00000276644	9.734643969	-2.781758677	0.716962226	-3.879923623	0.000104489	0.000599821
ENSG00000276649	26.9419389	-0.611504798	0.348046686	-1.756961987	0.078924289	0.174617969
ENSG00000276672	81.83403324	-0.677419707	0.198587996	-3.411181545	0.00064682	0.003042827
ENSG00000276728	17.49546821	0.537195862	0.442209483	1.214799506	0.224442569	0.382754009
ENSG00000276791	9.333432479	-0.098345962	0.560216667	-0.175549869	0.86064759	0.92075527
ENSG00000276966	877.3333323	-0.392902871	0.10264449	-3.827802843	0.000129292	0.000725307
ENSG00000277027	85571.39478	-0.361054414	0.067097498	-5.381041374	7.41E-08	7.93E-07

ENSG00000277075	1583.385323	-0.12630939	0.073664383	-1.714660261	0.086407533	0.187453484
ENSG00000277149	31.40649563	-0.435178612	0.320659336	-1.357136884	0.174737721	0.319144554
ENSG00000277151	25.32657094	-0.316466393	0.350180278	-0.903724203	0.366141662	0.537428624
ENSG00000277157	445.5834223	0.129337218	0.115721256	1.117661725	0.263711496	0.43023872
ENSG00000277161	74.36468647	-0.572107984	0.209563584	-2.729997137	0.006333488	0.021826914
ENSG00000277182	11.95660523	-0.766238148	0.507093408	-1.511039458	0.130778398	0.257830759
ENSG00000277224	1182.57524	-0.376991487	0.080140832	-4.704112465	2.55E-06	2.08E-05
ENSG00000277258	866.2465074	-0.236540182	0.0783959	-3.017251928	0.002550777	0.010057529
ENSG00000277283	13.53150656	-0.629104655	0.488961538	-1.286613785	0.198228936	0.349902221
ENSG00000277287	12.4385348	-0.624372539	0.510294534	-1.223553258	0.221120802	0.378906994
ENSG00000277363	145.1252133	-1.279238173	0.162219385	-7.885852673	3.12E-15	7.67E-14
ENSG00000277443	229.3874166	0.56555133	0.122234396	4.626777317	3.71E-06	2.93E-05
ENSG00000277449	11.09953431	-0.747325263	0.52907512	-1.412512582	0.157799089	0.295981279
ENSG00000277462	14.58201854	-1.119709717	0.468747854	-2.388724997	0.016906952	0.050316093
ENSG00000277476	29.28597738	0.054102032	0.341144033	0.158589998	0.873991909	0.93020483
ENSG00000277534	13.60644473	0.757005831	0.481269965	1.572933874	0.11573413	0.234692844
ENSG00000277589	10.33237633	0.127750487	0.538080237	0.237419028	0.81233173	0.889524775
ENSG00000277610	22.03484745	0.314156752	0.373800235	0.840440221	0.400661606	0.568781564
ENSG00000277687	10.03204563	-0.33031614	0.541174613	-0.610368876	0.541617481	0.691142874
ENSG00000277693	128.7353491	0.223087409	0.167650836	1.330666844	0.183298657	0.330568268
ENSG00000277738	31.37431465	-2.634998708	0.406476722	-6.48253286	9.02E-11	1.44E-09
ENSG00000277775	289.8113453	-0.373284825	0.137820908	-2.708477484	0.00675927	0.023095953
ENSG00000277791	656.0705495	0.620818144	0.092632235	6.701966574	2.06E-11	3.51E-10
ENSG00000277879	15.77348123	0.059712035	0.436850797	0.136687482	0.891277825	0.94027635
ENSG00000277918	340.3667924	-0.466242582	0.124918422	-3.732376482	0.000189682	0.001021451
ENSG00000277938	10.49171699	-0.183126805	0.542231586	-0.337728029	0.73556815	0.837387617
ENSG00000277945	8.718936628	-1.566604638	0.682685193	-2.294768737	0.021746381	0.06179879
ENSG00000277959	11.63826565	-0.248079589	0.503134345	-0.493068285	0.621964339	0.754793354
ENSG00000277969	8.261112538	-0.347301797	0.622610854	-0.557815198	0.576970584	0.720930955
ENSG00000277972	156.1420613	0.055113798	0.1456793	0.378322785	0.705190821	0.815025133
ENSG00000278000	24.88994123	-0.240650076	0.352866406	-0.681986361	0.495247575	0.654135261
ENSG00000278023	11.8076509	-0.683338769	0.505820987	-1.350949816	0.176711509	0.321787213
ENSG00000278053	326.4943386	0.08727504	0.119406929	0.730904318	0.464837598	0.62748162
ENSG00000278099	9.008202188	0.562605167	0.582951969	0.96509695	0.334496255	0.506275374
ENSG00000278112	17.11960878	0.62183709	0.413912657	1.502338912	0.133009604	0.261298813
ENSG00000278129	184.2014706	0.019929411	0.135480808	0.147101358	0.883052018	0.935532973
ENSG00000278133	33.0482699	-0.459320206	0.309455261	-1.484286305	0.137732982	0.267635246
ENSG00000278175	22.9004107	-0.047962218	0.364117879	-0.131721678	0.895204438	0.943073102
ENSG00000278192	8.463783573	0.123483863	0.603824213	0.204502999	0.837960449	0.905456617
ENSG00000278200	11.64879186	0.061189486	0.499299626	0.122550636	0.902462947	0.947258155
ENSG00000278259	706.2083244	-0.039698148	0.082359603	-0.482009946	0.629798881	0.760033065
ENSG00000278291	54.5009027	0.408572866	0.242094734	1.687656972	0.091477096	0.195910774
ENSG00000278311	508.9750553	-0.12740229	0.08998	-1.415895645	0.15680606	0.294630113
ENSG00000278376	9.919713697	0.314030525	0.544647922	0.576575275	0.564226406	0.710326606
ENSG00000278384	9.918722272	-0.015140195	0.553451909	-0.027355936	0.978175843	0.988805129
ENSG00000278390	11.19105785	-1.397886303	0.575469543	-2.429123	0.015135397	0.045889686
ENSG00000278463	289.9811339	0.315998741	0.352777223	0.895745871	0.370388505	0.541536387
ENSG00000278535	154.0442478	0.191088138	0.157096066	1.216377616	0.223841107	0.382164648
ENSG00000278540	2722.667849	-0.345311634	0.057948064	-5.958984876	2.54E-09	3.35E-08
ENSG00000278576	20.62333404	-0.257669607	0.383987013	-0.671037296	0.50219677	0.660354498

ENSG00000278588	482.9005448	-0.391215188	0.096907543	-4.036994189	5.41E-05	0.000329653
ENSG00000278600	30.37253865	0.080316134	0.330302895	0.243159038	0.807882198	0.886465967
ENSG00000278619	70.52123599	0.051475623	0.210697077	0.244311043	0.806989931	0.8858602
ENSG00000278662	24.20905731	0.490231834	0.383715108	1.277593253	0.201392881	0.353805345
ENSG00000278677	203.3307771	-0.503802101	0.145477696	-3.46308826	0.000534013	0.002569813
ENSG00000278705	217.4984294	-0.014738786	0.133115481	-0.110721802	0.911836957	0.952735327
ENSG00000278730	204.5724277	-0.201422095	0.140661279	-1.431965476	0.152153702	0.288402768
ENSG00000278768	105.2816029	-0.310406867	0.177673854	-1.747059906	0.080626947	0.177601808
ENSG00000278771	9407.633928	-1.006794124	0.25275106	-3.983342837	6.80E-05	0.000406702
ENSG00000278784	7.807275574	-1.084301811	0.633823794	-1.710730682	0.087130841	0.188470422
ENSG00000278828	896.929708	-0.416937776	0.090067289	-4.629180948	3.67E-06	2.90E-05
ENSG00000278834	17.15382282	-0.987842182	0.427145906	-2.312657494	0.020741478	0.059533212
ENSG00000278845	223.6700754	0.198559757	0.127777065	1.553954592	0.120195248	0.242208986
ENSG00000278861	7.549815091	0.61199221	0.650280882	0.941119794	0.346643473	0.518672866
ENSG00000278864	15.19434648	-0.624592619	0.45254112	-1.380189757	0.167528227	0.309718114
ENSG00000278873	20.22539126	-1.625520804	0.416430477	-3.903462627	9.48E-05	0.000548609
ENSG00000278896	39.04710993	-1.282582318	0.306832135	-4.180078193	2.91E-05	0.000188724
ENSG00000278905	61.3237504	-0.774429425	0.246233389	-3.145103225	0.001660282	0.006976364
ENSG00000278948	29.3846885	0.152390966	0.315376826	0.483202802	0.628951746	0.759402199
ENSG00000278962	11.4790547	-0.155000355	0.524277119	-0.29564585	0.767500555	0.860009596
ENSG00000278963	43.73448046	-0.254096992	0.291303456	-0.872275926	0.383057867	0.552444955
ENSG00000278972	15.38523065	-0.360649205	0.468078234	-0.77048916	0.441009783	0.606269761
ENSG00000278983	26.12475027	0.63561341	0.343571193	1.850019508	0.064310738	0.148850377
ENSG00000278985	10.45761656	-1.673024595	0.582908249	-2.870133674	0.004102983	0.015143091
ENSG00000278987	19.54619024	-0.744937541	0.412681435	-1.805115223	0.071056653	0.160911302
ENSG00000278991	14.45947315	-0.339363053	0.451432762	-0.751746621	0.452203448	0.616965713
ENSG00000279041	7.819133253	-6.246908409	1.277823219	-4.888710984	1.01E-06	8.98E-06
ENSG00000279059	42.91881301	-0.178173455	0.267337408	-0.666474085	0.505108123	0.662798113
ENSG00000279069	18.70730406	-0.679728487	0.408313471	-1.66472217	0.095968198	0.203174872
ENSG00000279077	9.724967851	-0.434190843	0.582639263	-0.745213841	0.456142457	0.61991199
ENSG00000279078	27.26625284	0.330804752	0.333948215	0.990586975	0.3218873	0.493780814
ENSG00000279088	48.32294958	0.358692822	0.249149417	1.439669522	0.14996092	0.285008936
ENSG00000279089	19.76878078	-0.498939573	0.410064094	-1.216735578	0.223704838	0.382052506
ENSG00000279092	46.2298661	0.409705311	0.257223432	1.592799329	0.111205216	0.22791066
ENSG00000279133	127.9501828	-0.346577015	0.158513931	-2.186413609	0.028785359	0.077894035
ENSG00000279138	15.79927017	-0.080294756	0.437294247	-0.183617226	0.854313749	0.916431867
ENSG00000279145	15.40129609	-0.346136626	0.46326171	-0.747172967	0.45495916	0.618784985
ENSG00000279166	48.56023237	0.012466433	0.264101167	0.047203248	0.962351239	0.98007307
ENSG00000279170	117.4706112	0.168706013	0.167454443	1.007474092	0.313707002	0.484939172
ENSG00000279175	35.65934542	-0.020276508	0.313408787	-0.064696679	0.948415507	0.971358017
ENSG00000279207	33.7402084	-0.170897366	0.299531585	-0.570548731	0.56830558	0.713722708
ENSG00000279233	12.23308995	0.059020118	0.521964353	0.113073082	0.909972616	0.951381168
ENSG00000279236	16.27757659	-0.020508925	0.429998495	-0.047695342	0.961959046	0.979825424
ENSG00000279253	17.40222113	0.003236964	0.411628014	0.007863808	0.993725653	0.996990519
ENSG00000279281	17.98823246	-0.448516632	0.422694192	-1.061090122	0.288648946	0.458770449
ENSG00000279342	53.44237778	-0.596078092	0.240263442	-2.480935455	0.01310381	0.040615449
ENSG00000279347	28.18387782	-0.414560034	0.32606393	-1.27140722	0.203583815	0.356584244
ENSG00000279348	59.6836005	-1.163878424	0.239736102	-4.854831685	1.20E-06	1.05E-05
ENSG00000279356	16.68091307	0.448261582	0.425572113	1.053315214	0.292196548	0.462685708
ENSG00000279392	24.19376172	0.4463811	0.350065871	1.275134588	0.202261609	0.354879942

ENSG00000279407	50.1170604	0.357964419	0.265648983	1.347509087	0.177816329	0.323295139
ENSG00000279425	35.77612272	-0.430218539	0.30559876	-1.407788891	0.159193591	0.297801621
ENSG00000279432	25.40076435	0.035152067	0.346468813	0.101458098	0.919186819	0.955581501
ENSG00000279443	44.24375824	-0.272817903	0.265165021	-1.028860826	0.303545079	0.474358887
ENSG00000279453	7.564185179	-0.200745423	0.637411887	-0.314938311	0.752808492	0.850671779
ENSG00000279481	16.51895917	-1.034799051	0.44549248	-2.322820467	0.020188799	0.058232456
ENSG00000279491	7.965129592	0.142186096	0.616711162	0.230555412	0.81766021	0.892507031
ENSG00000279495	7.637380904	-0.488625577	0.64706374	-0.755142881	0.450163257	0.61512094
ENSG00000279502	13.53179731	-0.528335208	0.503400581	-1.049532377	0.293933162	0.464336384
ENSG00000279516	38.31250064	0.167652806	0.282079019	0.594346953	0.55228008	0.699664348
ENSG00000279520	8.903045831	-0.154864017	0.637844629	-0.242792695	0.808165995	0.886691223
ENSG00000279544	39.05256522	-0.5032433	0.304376665	-1.653357035	0.098258222	0.206838489
ENSG00000279555	30.80432052	0.348605408	0.322980605	1.079338522	0.280436846	0.44922983
ENSG00000279573	12.76818813	-0.474209719	0.509805921	-0.930176954	0.352279472	0.523715899
ENSG00000279583	13.0334755	-0.057792581	0.5030974	-0.114873543	0.908545356	0.95095631
ENSG00000279589	10.79171478	-0.97171531	0.552040415	-1.760224946	0.078369674	0.173774082
ENSG00000279598	40.88438549	-0.670089726	0.278834677	-2.403179307	0.016253216	0.048721494
ENSG00000279602	27.15664938	0.495831175	0.34395745	1.441548002	0.149429927	0.284192009
ENSG00000279608	23.09720034	-0.347753144	0.372143881	-0.934458852	0.350067252	0.522071843
ENSG00000279619	19.31403521	-1.351856269	0.425297131	-3.178616011	0.0014798	0.006320082
ENSG00000279632	11.35125274	-0.806545409	0.575560699	-1.401321198	0.161118045	0.300441415
ENSG00000279655	7.50266237	-6.18840925	1.284275214	-4.818600549	1.45E-06	1.24E-05
ENSG00000279659	72.95716206	-1.876027343	0.249277005	-7.525874044	5.24E-14	1.17E-12
ENSG00000279670	31.06976307	-1.322949756	0.336520262	-3.931263304	8.45E-05	0.000496229
ENSG00000279692	10.00914023	-1.066021773	0.572732433	-1.861291089	0.062703081	0.145813188
ENSG00000279696	12.21518514	-0.724338819	0.50413347	-1.436799701	0.150774915	0.286207465
ENSG00000279738	71.70937383	-0.302639277	0.232844219	-1.299750012	0.193686663	0.344525333
ENSG00000279744	14.1584327	-0.373152524	0.467142674	-0.798797764	0.424407687	0.59161008
ENSG00000279759	31.34124534	-0.323449706	0.308001543	-1.050156122	0.293646339	0.463987641
ENSG00000279766	12.66957667	-0.01996242	0.49231179	-0.040548328	0.967655978	0.982949477
ENSG00000279770	15.10676745	2.370163544	0.533002307	4.446816671	8.72E-06	6.34E-05
ENSG00000279786	20.95480224	-0.434395844	0.37986104	-1.143565142	0.252804025	0.417437186
ENSG00000279789	45.27790631	0.242475206	0.259465821	0.934516944	0.3500373	0.522071843
ENSG00000279792	26.09559656	-0.666610947	0.36708952	-1.815935652	0.069380244	0.157929717
ENSG00000279799	31.95580646	-0.296338836	0.318883243	-0.929302	0.352732599	0.524165716
ENSG00000279801	15.5849574	-1.186778435	0.469407583	-2.52824726	0.011463359	0.036242584
ENSG00000279812	20.07490309	0.210686185	0.382036774	0.551481427	0.581303695	0.723769988
ENSG00000279821	18.41796601	-0.457977444	0.404121234	-1.133267459	0.257101953	0.422868358
ENSG00000279838	28.98777103	-0.566846932	0.335815203	-1.687972809	0.091416449	0.195810743
ENSG00000279852	16.55938432	-0.112097449	0.42067894	-0.266467935	0.789878851	0.874482048
ENSG00000279880	15.16213853	-0.308435965	0.453615422	-0.679950349	0.4965359	0.655099069
ENSG00000279891	18.43250663	-1.38297709	0.430675735	-3.211179497	0.001321913	0.005722316
ENSG00000279917	8.373385473	-1.051650066	0.632774547	-1.661966447	0.096519502	0.203942456
ENSG00000279970	15.02533543	0.469806513	0.468676795	1.002410442	0.316145401	0.487716314
ENSG00000279977	15.20066913	-0.183285255	0.459496322	-0.398882965	0.689979442	0.804902039
ENSG00000280010	11.21164609	-0.867377783	0.530015784	-1.636513119	0.101732242	0.212572927
ENSG00000280028	7.411018858	0.122022171	0.665485073	0.183358238	0.854516941	0.916499922
ENSG00000280046	7.096910658	0.453560589	0.657404793	0.689925892	0.490240792	0.649845466
ENSG00000280063	11.10252236	-0.804116432	0.536963662	-1.49752486	0.134256742	0.262904084
ENSG00000280069	25.30916142	-0.440304085	0.373956371	-1.177421003	0.239027511	0.400861921

ENSG00000280077	8.680717116	0.077327072	0.600988496	0.128666476	0.897621563	0.944486149
ENSG00000280088	40.51411172	-0.467287673	0.293521366	-1.592005646	0.111383436	0.228188751
ENSG00000280099	59.69620329	-0.939592653	0.249647219	-3.763681639	0.00016743	0.000911938
ENSG00000280120	12.08957858	-0.290809885	0.497226504	-0.584864007	0.558639162	0.705620385
ENSG00000280128	101.2332202	-0.562890944	0.186215486	-3.022793412	0.002504532	0.009907394
ENSG00000280138	91.25819754	0.025735201	0.187652287	0.13714302	0.890917749	0.94027635
ENSG00000280152	10.8834848	-0.455109091	0.541056497	-0.841148925	0.40026451	0.568390171
ENSG00000280173	59.86388999	-0.180868184	0.236295982	-0.765430636	0.444015156	0.608911539
ENSG00000280176	10.88201165	1.063073546	0.577616009	1.840450283	0.065702157	0.151397343
ENSG00000280184	13.11542491	-0.335324402	0.474900851	-0.706093497	0.480129996	0.640796774
ENSG00000280195	10.35751631	-0.06243369	0.551409762	-0.113225579	0.909851717	0.951381168
ENSG00000280205	20.96307422	0.032022743	0.392614295	0.081562856	0.93499434	0.964624614
ENSG00000280206	53.57913092	0.188378513	0.243008722	0.775192394	0.438225982	0.603683473
ENSG00000280211	15.30583916	0.236932949	0.467295669	0.507030055	0.612133731	0.747774297
ENSG00000280219	9.446222814	-0.707942804	0.585060728	-1.210033028	0.22626622	0.38460329
ENSG00000280239	30.57893213	-0.813535938	0.319482821	-2.54641528	0.010883563	0.034659126
ENSG00000280287	11.06232187	-0.893909852	0.526905294	-1.696528508	0.089785836	0.192965367
ENSG00000280303	15.61818469	-0.444973463	0.440828714	-1.009402176	0.312781795	0.484076461
ENSG00000280332	9.911427854	-0.781895546	0.555525818	-1.407487321	0.159282934	0.297929078
ENSG00000280347	76.71003117	0.895176081	0.207216368	4.320006621	1.56E-05	0.00010712
ENSG00000280351	14.42877814	0.010906519	0.470146655	0.023198121	0.981492237	0.990660448
ENSG00000280355	21.98248599	-0.185356164	0.36733465	-0.504597549	0.613841532	0.748716754
ENSG00000280383	99.86728918	-0.019749026	0.17993926	-0.109753844	0.912604597	0.953041169
ENSG00000280388	18.2813239	0.07632084	0.402182801	0.189766543	0.849492074	0.913831764
ENSG00000280392	18.06254367	-0.53521486	0.437463683	-1.22344981	0.22115985	0.378906994
ENSG00000280434	62.19686155	-0.168297135	0.229968041	-0.73182836	0.464273337	0.627141798
ENSG00000280543	18.64900143	0.028903085	0.40858985	0.070738627	0.943605778	0.968637121
ENSG00000280734	54.67995103	0.553407249	0.236372005	2.341255469	0.019219011	0.055894296
ENSG00000280739	35.52373934	-0.787974232	0.301123702	-2.616779173	0.008876375	0.02920057
ENSG00000280789	68.58562838	-0.305693728	0.216243977	-1.413651987	0.157464109	0.295550286
ENSG00000280798	80.39799667	-0.007472451	0.196918416	-0.037946937	0.96972999	0.984233899
ENSG00000280997	15.67150479	7.589510462	1.232087296	6.159880463	7.28E-10	1.04E-08
ENSG00000281005	8.951121353	-0.835300756	0.60196944	-1.387613225	0.165254852	0.306568266
ENSG00000281026	69.86696123	-0.459132493	0.231146814	-1.986324124	0.046997346	0.116186504
ENSG00000281128	28.70648265	0.363146515	0.325828279	1.114533448	0.265050403	0.431693045
ENSG00000281183	37.63279988	-0.158167799	0.296305407	-0.533799907	0.593479978	0.733598491
ENSG00000281195	62.87649335	-0.025218014	0.221658565	-0.113769636	0.909420409	0.951381168
ENSG00000281202	31.81093476	4.38647987	0.532560605	8.236583455	1.77E-16	4.73E-15
ENSG00000281207	75.83458837	-0.267616266	0.213513238	-1.253394256	0.210062261	0.365145969
ENSG00000281332	53.67103535	-0.139680321	0.237570501	-0.587953138	0.556563748	0.703568301
ENSG00000281344	420.2376374	-0.826804092	0.109603524	-7.54359043	4.57E-14	1.04E-12
ENSG00000281398	134.0332855	0.364248185	0.153334932	2.375506876	0.017524866	0.051902128
ENSG00000281501	51.64074088	-1.600660203	0.268016894	-5.972236234	2.34E-09	3.12E-08
ENSG00000281649	251.3701554	-0.034971968	0.124242204	-0.281482193	0.778340584	0.867349986
ENSG00000281706	11.08650375	-1.04232178	0.548624921	-1.899880482	0.057448806	0.135878663
ENSG00000281880	9.244686462	-5.482461726	1.268835711	-4.320860201	1.55E-05	0.000106759
ENSG00000282057	10.0055817	-0.331889949	0.54646842	-0.607336009	0.543627946	0.69257589
ENSG00000282164	28.82691287	-0.488330072	0.331534685	-1.472938108	0.140767725	0.271914774
ENSG00000282381	16.21950415	-0.845075985	0.441738772	-1.913067264	0.055739442	0.132856354
ENSG00000282508	11.03258868	0.26872017	0.525200644	0.5116524	0.608894305	0.745198811

ENSG00000282851	28.51786524	1.254283747	0.345581582	3.629486677	0.000283985	0.001464981
ENSG00000282936	121.0977372	0.285981653	0.179147141	1.596350637	0.11041053	0.226645729
ENSG00000282977	16.6689805	0.565275197	0.431563491	1.309830902	0.190253047	0.339881031
ENSG00000282988	9.571001617	1.716317447	0.600020849	2.860429685	0.004230674	0.015525908
ENSG00000283041	40.85007891	0.382480451	0.275049853	1.390585914	0.16435103	0.30533513
ENSG00000283103	18.60244709	0.109783145	0.396000508	0.277229808	0.781603653	0.869156627
ENSG00000283122	18.72749467	0.046888155	0.411623488	0.1139103	0.9093089	0.951381168
ENSG00000283154	5.46000313	-5.728711165	1.319638334	-4.341122122	1.42E-05	9.84E-05
ENSG00000283172	14.00406258	-0.198625492	0.456976139	-0.43465178	0.663815204	0.785032175
ENSG00000283199	16.48680666	0.692349167	0.444670787	1.556992693	0.11947222	0.241124465
ENSG00000283355	10.81796865	0.618110498	0.520957739	1.186488753	0.23542934	0.396484348
ENSG00000283415	43.73700691	-0.721362237	0.279007885	-2.58545466	0.009725066	0.031532501
ENSG00000283563	10.76005551	-1.729361028	0.58724038	-2.944894605	0.003230647	0.012291684
ENSG00000283674	24.5856158	-0.384110263	0.359387313	-1.068791938	0.285163433	0.454727068
ENSG00000283761	14.89206691	0.134991696	0.459637963	0.293691356	0.76899376	0.861270559
ENSG00000284024	232.9523248	0.156718196	0.126621435	1.237690886	0.2158307	0.372146477
ENSG00000284052	13.98629149	-0.399206691	0.472728634	-0.84447326	0.398404999	0.56655143
ENSG00000284194	49.14364551	-0.074551929	0.266420397	-0.279828159	0.779609347	0.868244866
ENSG00000284428	22.95513262	-0.492627146	0.378757559	-1.300639773	0.193381786	0.344113751
ENSG00000284591	11.04135632	0.331492708	0.516563385	0.64172707	0.52105041	0.675369842
ENSG00000284602	9.492602122	-0.484125582	0.566784476	-0.854161682	0.393015411	0.561159532
ENSG00000284624	20.43432229	0.168914123	0.389527746	0.433638231	0.664551168	0.785491334
ENSG00000284634	14.16764863	-0.447946814	0.493346055	-0.907976884	0.363890435	0.534850783
ENSG00000284691	18.3140985	0.208618482	0.396608403	0.526006206	0.598883895	0.737617016
ENSG00000284753	27.4652355	-0.199805071	0.334481437	-0.597357728	0.55026857	0.698254257
ENSG00000284770	77.30602925	-0.170271565	0.209876902	-0.811292539	0.417197693	0.58515986
ENSG00000284946	41.75557128	-0.271772615	0.268337682	-1.012800783	0.311155327	0.482641219
ENSG00000284948	14.788052	0.538851674	0.459671692	1.172253335	0.241095361	0.403558929
ENSG00000285043	8.555816926	-0.42937394	0.585319069	-0.733572444	0.46320936	0.626367143
ENSG00000285053	44.35506558	0.080282946	0.265124371	0.302812395	0.762032837	0.856479057
ENSG00000285077	19.98179357	-0.595193371	0.419170752	-1.419930583	0.155627891	0.293122149
ENSG00000285184	21.84021963	-0.883637304	0.375075191	-2.355893764	0.018478201	0.054132074
ENSG00000285210	10.7226564	-1.218291608	0.563334343	-2.162643946	0.030568575	0.081794572
ENSG00000285219	12.52592743	-0.899250885	0.508980446	-1.766769024	0.077266918	0.171681177
ENSG00000285230	13.69369598	0.474765419	0.467449758	1.015650155	0.309796012	0.481094524
ENSG00000285280	17.3506561	-1.239794186	0.444185219	-2.791164888	0.005251871	0.018667265
ENSG00000285410	82.09210416	-0.166006959	0.203402531	-0.816149919	0.414414396	0.582535677
ENSG00000285437	7.414998605	0.097563242	0.649873286	0.150126562	0.880664764	0.934201626
ENSG00000285533	17.72200274	0.418951129	0.412786889	1.014933225	0.310137659	0.48148803
ENSG00000285589	51.90169289	0.275181729	0.252576927	1.089496704	0.275934908	0.444400097
ENSG00000285646	18.87913295	1.149032243	0.414768691	2.770296474	0.005600529	0.019681009
ENSG00000285650	8.465674001	-0.973212356	0.638020547	-1.525362092	0.127168809	0.252343587
ENSG00000285667	9.259993097	-0.66084212	0.602677363	-1.096510604	0.272855385	0.44090643
ENSG00000285669	16.98833857	-0.908485588	0.425700737	-2.134094468	0.032835049	0.086666839
ENSG00000285679	11.33164636	0.515029137	0.5381944	0.956957445	0.338588735	0.510520298
ENSG00000285693	16.94985355	0.061791162	0.421299435	0.146668039	0.883394047	0.935631994
ENSG00000285730	11.2043659	0.290970746	0.514340977	0.56571566	0.571587088	0.716690513
ENSG00000285756	82.07973725	-0.044600102	0.193198747	-0.230850886	0.817430647	0.892507031
ENSG00000285793	31.4295296	0.065974474	0.30970095	0.213026385	0.831306383	0.901477895
ENSG00000285796	30.35278461	-0.261525919	0.323255739	-0.809037203	0.418493748	0.586450686

ENSG00000285816	10.49403049	-0.238644165	0.534952655	-0.446103338	0.655522602	0.779161109
ENSG00000285922	15.27869227	1.8839128	0.483770214	3.894230656	9.85E-05	0.000566892
ENSG00000285967	86.3818654	-0.823209698	0.198513135	-4.146877724	3.37E-05	0.000215789
ENSG00000285999	9.443803387	-0.487621345	0.562055589	-0.867567825	0.385630967	0.554504882
ENSG00000286019	7.078405669	-0.677615322	0.662682352	-1.022534129	0.306528168	0.47752037
ENSG00000286058	21.98573192	-0.033866547	0.364263955	-0.092972546	0.925925372	0.959462504
ENSG00000286121	8.715887441	-1.789342129	0.654593793	-2.73351527	0.006266222	0.021632248
ENSG00000286129	16.05639436	-0.36736302	0.446539992	-0.822687836	0.410685534	0.579077887
ENSG00000286162	8.579306476	-0.204026202	0.594661129	-0.343096584	0.731525801	0.834408683
ENSG00000286172	63.42404008	0.426914577	0.229280732	1.861973199	0.062606868	0.14577137
ENSG00000286177	26.79799235	0.259730759	0.333794769	0.778115126	0.436501146	0.602607528
ENSG00000286207	45.11941823	-0.732547426	0.26554682	-2.75863754	0.005804287	0.020276753
ENSG00000286214	33.38903131	-1.471143928	0.33094649	-4.445262216	8.78E-06	6.38E-05
ENSG00000286219	18.59099246	0.179521154	0.394577306	0.454970804	0.649130243	0.774121217
ENSG00000286264	31.26043719	0.146877641	0.338757115	0.433578024	0.664594896	0.785491334
ENSG00000286314	20.77186547	-0.489010121	0.383028386	-1.276694206	0.201710228	0.354212163
ENSG00000286340	20.26561358	-1.011386787	0.399721353	-2.530229569	0.011398792	0.036054679
ENSG00000286379	79.98034013	-1.232397714	0.217051241	-5.677911395	1.36E-08	1.65E-07
ENSG00000286385	25.96988624	0.819831457	0.344388537	2.380542238	0.017287179	0.051295523
ENSG00000286388	36.7199778	-0.512621435	0.300807056	-1.704153623	0.088352391	0.19046747
ENSG00000286389	19.16608615	-0.554698979	0.3956412	-1.402025317	0.160907687	0.300168695
ENSG00000286402	11.24710812	0.954094755	0.519343846	1.837115741	0.066192811	0.152278155
ENSG00000286432	17.42449318	1.391904727	0.436125171	3.191525779	0.001415235	0.006075735
ENSG00000286452	20.19117041	-5.77795274	1.041231642	-5.549152086	2.87E-08	3.30E-07
ENSG00000286522	1091.553119	-0.169761253	0.080940568	-2.097356818	0.035962001	0.093530499
ENSG00000286634	24.53260048	-0.200139665	0.347672854	-0.57565514	0.564848303	0.710778833
ENSG00000286676	15.03889082	-0.637942799	0.45682956	-1.396456917	0.162576946	0.302719825
ENSG00000286681	15.36911184	-0.673982085	0.449433676	-1.499625242	0.133711505	0.262141733
ENSG00000286707	7.847143627	0.36409941	0.621265392	0.586060989	0.557834531	0.704984405
ENSG00000286721	10.36842403	0.232326345	0.547260473	0.424526084	0.671182187	0.790284676
ENSG00000286775	11.79418562	-2.15039507	0.58309785	-3.687880293	0.00022613	0.001193705
ENSG00000286786	65.74166001	-0.935762325	0.228148528	-4.101548812	4.10E-05	0.000257357
ENSG00000286787	11.95022769	-0.880514289	0.509674169	-1.727602343	0.084059542	0.183551126
ENSG00000286813	35.36984364	-0.527868297	0.311067608	-1.696956814	0.089704826	0.192820763
ENSG00000286825	13.4034095	-0.470686391	0.485994796	-0.968500888	0.332794276	0.505081442
ENSG00000286835	12.08009511	-0.224392374	0.493620042	-0.454585218	0.649407671	0.774190724
ENSG00000286860	6.182422547	-5.908940585	1.305667237	-4.525609909	6.02E-06	4.54E-05
ENSG00000286872	10.78334437	-1.650729256	0.591158501	-2.792363221	0.005232458	0.018604436
ENSG00000286881	10.44239495	0.425944225	0.554665149	0.767930391	0.442528541	0.607577494
ENSG00000286952	9.60972036	-0.57194286	0.603339551	-0.947961821	0.343148891	0.51503499
ENSG00000286977	34.18796112	-0.532307162	0.30809735	-1.72772392	0.084037733	0.183533579
ENSG00000286980	26.02909833	5.076603384	0.706888104	7.181622316	6.89E-13	1.39E-11
ENSG00000286997	53.96828358	-1.9616635	0.274086581	-7.157094286	8.24E-13	1.65E-11
ENSG00000287001	18.53617017	0.192308404	0.409107401	0.47006826	0.63830625	0.766295498
ENSG00000287014	27.36052665	1.13662611	0.342718088	3.316504584	0.000911511	0.004139668
ENSG00000287022	9.626903377	-0.710231449	0.570248237	-1.245477677	0.212956216	0.36831023
ENSG00000287038	12.11546997	-2.572191133	0.621704614	-4.137320322	3.51E-05	0.000223749
ENSG00000287064	338.2254964	0.510187253	0.108541267	4.700398919	2.60E-06	2.12E-05
ENSG00000287110	12.79659576	0.374991167	0.49403794	0.759033137	0.447832735	0.612950056
ENSG00000287126	12.41382372	-0.094205957	0.489264878	-0.192545922	0.8473146	0.912187936

ENSG00000287151	18.33220428	-0.042070124	0.423615902	-0.099311956	0.920890585	0.956715484
ENSG00000287168	29.57397659	-0.740884281	0.327752255	-2.26050094	0.023790178	0.066503608
ENSG00000287176	9.124765862	5.828519766	1.276432213	4.566258753	4.97E-06	3.80E-05
ENSG00000287200	17.62237212	0.395686048	0.44206388	0.89508794	0.37074008	0.541800448
ENSG00000287222	20.77994838	-0.175685605	0.388789452	-0.451878527	0.651356494	0.775982078
ENSG00000287260	8.729842497	-1.369855475	0.623626709	-2.196595262	0.028049365	0.076284497
ENSG00000287263	9.591612813	-0.686465368	0.576273656	-1.191214211	0.233569502	0.393918933
ENSG00000287264	12.24123196	-0.550339706	0.514335773	-1.070000834	0.284618933	0.454184517
ENSG00000287356	20.89873126	0.383765915	0.399376296	0.960913101	0.336595863	0.508459199
ENSG00000287364	10.03168941	-1.906225369	0.605703702	-3.147125176	0.001648844	0.006934523
ENSG00000287376	17.46634437	0.799279463	0.428484807	1.865362436	0.062130619	0.144806505
ENSG00000287431	40.82103639	-0.084010781	0.27535778	-0.305096814	0.760292418	0.855275694
ENSG00000287553	33.48260633	0.149196311	0.314147961	0.474923697	0.63484136	0.764083333
ENSG00000287562	18.28870644	-0.327052407	0.404400559	-0.808733816	0.418668273	0.586578219
ENSG00000287665	11.059281	0.336341772	0.518197183	0.649061366	0.516298712	0.671307749
ENSG00000287697	22.28869425	0.049566047	0.362460497	0.136748825	0.891229336	0.94027635
ENSG00000287707	10.31084781	-0.328356656	0.544195089	-0.603380409	0.546255652	0.694729455
ENSG00000287730	36.79695587	-2.960755722	0.387240008	-7.645789839	2.08E-14	4.81E-13
ENSG00000287763	11.24288462	0.075159756	0.53083218	0.141588546	0.887405015	0.93824263
ENSG00000287787	11.06277444	-0.482116609	0.533666822	-0.903403752	0.36631165	0.537621956
ENSG00000287804	93.73761159	-0.017590683	0.185811186	-0.094669667	0.924577212	0.958810774
ENSG00000287811	144.9896593	-1.983699509	0.175163374	-11.32485328	9.88E-30	5.34E-28
ENSG00000287837	40.5333494	0.249934642	0.287396304	0.869651554	0.384490855	0.553658948
ENSG00000287853	16.40428497	1.599019527	0.460991114	3.468655855	0.000523069	0.002521466
ENSG00000287859	10.8705606	-0.712431408	0.547715437	-1.300732754	0.193349947	0.344100684
ENSG00000287925	23.38527833	-1.76318387	0.401667773	-4.389657292	1.14E-05	8.05E-05
ENSG00000287978	38.49587455	0.458416009	0.284445558	1.611612472	0.107046295	0.221259462
ENSG00000288009	11.28597633	-0.841955131	0.542935313	-1.550746673	0.120962405	0.243397636
ENSG00000288032	18.95660473	-0.367299216	0.416635814	-0.881583396	0.378002138	0.547401652
ENSG00000288035	71.02998264	-1.352261433	0.238838496	-5.661823595	1.50E-08	1.79E-07
ENSG00000288044	20.04503797	-0.234595027	0.418926041	-0.559991512	0.575485227	0.719715188
ENSG00000288061	18.76843318	0.154148642	0.39590375	0.389358883	0.697010684	0.809969314
ENSG00000288398	24.82296018	0.611878645	0.361184099	1.694090764	0.090248037	0.193709482
ENSG00000288558	27.33323378	0.675814925	0.391633945	1.725629083	0.084414164	0.184153713
ENSG00000288559	25.8451845	0.228534091	0.335773598	0.680619597	0.496112223	0.654786094
ENSG00000288596	13.27712467	0.042402903	0.467379212	0.090724837	0.927711235	0.960604431
ENSG00000288612	19.37714716	0.301261242	0.401443225	0.750445451	0.452986465	0.617396281
ENSG00000288670	25.61083245	-0.18788494	0.348817919	-0.53863328	0.59013992	0.731400693
ENSG00000288701	3397.938826	-0.022644088	0.057795992	-0.391793391	0.695210879	0.808564553
ENSG00000288714	6.31311414	-4.910015678	1.304383666	-3.764241921	0.000167055	0.000910249
ENSG00000288719	11.3056799	-0.614879789	0.550686951	-1.116568656	0.2641788	0.430800975
ENSG00000288741	7.701972296	0.280469542	0.653874785	0.428934634	0.667970795	0.787758018
ENSG00000288744	16.24501222	0.152351419	0.437820473	0.347976919	0.727857518	0.83181315
ENSG00000288776	17.25339507	4.505429475	0.738674347	6.099344714	1.07E-09	1.48E-08
ENSG00000288793	28.95101927	6.626814932	1.058023404	6.26339163	3.77E-10	5.55E-09
ENSG00000288808	11.20862828	-2.526173297	0.637797636	-3.960775572	7.47E-05	0.0004426
ENSG00000288810	9.54074299	1.112316977	0.596135621	1.865879069	0.062058287	0.144685934
ENSG00000288826	22.74152835	0.653966214	0.366598877	1.783874025	0.074444138	0.166441629
ENSG00000288827	59.81624082	0.637245109	0.226823879	2.809426905	0.004962979	0.017799521
ENSG00000288838	16.39095886	-0.828699616	0.431955194	-1.918485128	0.055049526	0.131610635

ENSG00000288839	75.7657109	0.117034422	0.210627799	0.555645659	0.578453112	0.721948544
ENSG00000288863	9.804338289	0.047373981	0.570084337	0.083099951	0.933772065	0.963895958
ENSG00000288876	29.04686151	-1.330035055	0.344815734	-3.857234238	0.000114677	0.000652439
ENSG00000288879	10.41756972	-0.081839792	0.574395352	-0.142479899	0.886700955	0.938065731
ENSG00000288881	9.747977584	-0.751298114	0.55597469	-1.351317114	0.176593872	0.321656396
ENSG00000288899	20.61965574	-0.33714342	0.389971318	-0.864533887	0.387294673	0.555986538
ENSG00000288910	10.50040952	-0.552234699	0.566476682	-0.974858658	0.329630376	0.502149212
ENSG00000288913	11.95086737	0.626680445	0.495557642	1.264596471	0.206016019	0.359535554
ENSG00000288920	27.94313634	-0.163054291	0.325693176	-0.500637726	0.616626106	0.750779831
ENSG00000288924	58.67402149	-0.246399729	0.233496449	-1.055261139	0.291305909	0.461787846
ENSG00000288928	64.57745306	-0.082075211	0.232906971	-0.352394825	0.724542183	0.829473842
ENSG00000288934	8.384509794	-0.019143068	0.616191634	-0.031066745	0.97521631	0.987447813
ENSG00000288940	6.191731091	-3.451156855	1.013108864	-3.406501491	0.000658012	0.003093405
ENSG00000288947	17.62663703	0.409705053	0.423622921	0.967145622	0.333471242	0.505751515
ENSG00000288953	10.93036567	0.215047647	0.52510601	0.409531872	0.682149382	0.798912154
ENSG00000288958	30.29643017	0.293384605	0.314241693	0.93362724	0.350496209	0.522448749
ENSG00000288961	17.32002954	-0.088565827	0.420941457	-0.210399394	0.833355966	0.902386945
ENSG00000288973	65.4893074	0.044226681	0.217619873	0.203229054	0.838956005	0.906175702
ENSG00000288983	16.81961277	-0.089159536	0.422103182	-0.211226874	0.832710241	0.902134873
ENSG00000288993	31.01566483	-1.934541414	0.373751291	-5.176012654	2.27E-07	2.26E-06
ENSG00000288999	11.65968827	0.620237124	0.524044932	1.183557146	0.236588413	0.39772109
ENSG00000289004	13.15737816	-0.468827043	0.490503983	-0.955806801	0.339169852	0.511070291
ENSG00000289007	8.408815574	-1.171686213	0.620533069	-1.888193024	0.059000042	0.138846304
ENSG00000289017	15.46924617	-0.098341756	0.451440356	-0.217839976	0.827553796	0.898836169
ENSG00000289019	13.39861168	-1.823072876	0.531152239	-3.432298203	0.000598489	0.002845
ENSG00000289041	8.982619382	-0.604909456	0.593855346	-1.018614146	0.308386186	0.479662954
ENSG00000289044	11.6827743	0.238029219	0.497525656	0.478426019	0.632347018	0.761995244
ENSG00000289047	63.91691971	-0.397976657	0.22056755	-1.804330044	0.071179583	0.161111762
ENSG00000289049	13.03986706	2.825697448	0.58584087	4.823319083	1.41E-06	1.21E-05
ENSG00000289056	8.439326197	-0.231209043	0.585498898	-0.394892363	0.692922326	0.806697999
ENSG00000289065	8.739146823	-0.631320363	0.617792635	-1.021896876	0.306829711	0.477778179
ENSG00000289089	13.98266394	-0.034539141	0.482980047	-0.071512564	0.942989826	0.968571079
ENSG00000289092	9.87593679	-0.024510487	0.544517484	-0.045013223	0.964096769	0.981139413
ENSG00000289100	10.39421315	-0.223294972	0.546746273	-0.408406939	0.68297494	0.799479082
ENSG00000289106	23.81717797	-0.452289124	0.36099712	-1.252888454	0.210246305	0.365330262
ENSG00000289115	12.0700845	0.825730243	0.510140903	1.618631711	0.105526528	0.218600669
ENSG00000289121	12.11279095	-0.282973858	0.512930413	-0.551680795	0.58116707	0.723664022
ENSG00000289138	94.64189694	-0.165674172	0.191006991	-0.867372292	0.385738059	0.554545339
ENSG00000289154	7.964352238	-1.319096679	0.66310515	-1.98927226	0.04667116	0.115563156
ENSG00000289155	28.29865762	1.537553118	0.344854208	4.458559828	8.25E-06	6.03E-05
ENSG00000289159	14.91723684	0.806007556	0.460497502	1.750297346	0.080067019	0.176597298
ENSG00000289164	7.340750592	-0.565756556	0.667419099	-0.847678104	0.396617264	0.564466333
ENSG00000289179	16.28852992	0.729516344	0.438125179	1.665086554	0.095895489	0.203097048
ENSG00000289182	18.07199344	-1.417124665	0.454217425	-3.119925806	0.001808966	0.007500161
ENSG00000289189	6.203671642	-5.912550956	1.303746693	-4.535045794	5.76E-06	4.36E-05
ENSG00000289194	28.33118971	-0.35011659	0.326808394	-1.071320675	0.284025266	0.453633078
ENSG00000289210	18.94395781	0.821896182	0.397147528	2.069498422	0.038499339	0.09878655
ENSG00000289222	13.9329089	0.484112099	0.501053853	0.966187757	0.333950238	0.505990311
ENSG00000289227	13.70124955	-3.622622486	0.735334684	-4.926494785	8.37E-07	7.54E-06
ENSG00000289234	158.4216552	-1.111903679	0.152715999	-7.280859164	3.32E-13	6.91E-12

ENSG00000289267	11.94391353	0.595441828	0.511332368	1.164490779	0.244225178	0.407389986
ENSG00000289268	50.5959405	-0.336093355	0.254205458	-1.322132723	0.186123959	0.334204685
ENSG00000289269	9.326472748	-0.311472836	0.573157927	-0.543432833	0.586831825	0.728779652
ENSG00000289270	33.30773281	-0.812316472	0.317205078	-2.560855827	0.010441468	0.033462203
ENSG00000289274	7.576530651	-1.79232601	0.744475369	-2.407502093	0.01606207	0.048272122
ENSG00000289283	15.60286625	-0.528570896	0.457083361	-1.156399337	0.247517853	0.411757849
ENSG00000289293	74.75927726	0.573809289	0.472968693	1.213207761	0.225050398	0.38318581
ENSG00000289294	25.06173624	-0.372345506	0.356893414	-1.043296097	0.296811178	0.467306279
ENSG00000289295	10.05389087	-0.035069238	0.54206546	-0.064695578	0.948416384	0.971358017
ENSG00000289318	18.39253882	0.147095997	0.421126945	0.349291344	0.726870596	0.831258753
ENSG00000289326	12.38189643	-2.930365776	0.648835848	-4.516343823	6.29E-06	4.72E-05
ENSG00000289353	34.99892972	-0.69311925	0.299280361	-2.315953001	0.020560835	0.059126532
ENSG00000289357	44.68707828	-0.321371635	0.283033739	-1.135453449	0.256185376	0.421781057
ENSG00000289372	13.46004383	0.055257606	0.476616016	0.115937366	0.907702181	0.950649754
ENSG00000289378	12.70805557	-0.777411448	0.50961126	-1.525498963	0.127134692	0.252311531
ENSG00000289380	8.113468296	0.415745842	0.608158548	0.683614238	0.494218791	0.65345363
ENSG00000289394	8.714602995	-0.0774815	0.584614225	-0.132534408	0.894561609	0.942843412
ENSG00000289396	26.26104953	0.20910245	0.350057932	0.5973367	0.550282606	0.698254257
ENSG00000289411	6.791135676	5.354618978	1.301384973	4.114554179	3.88E-05	0.00024472
ENSG00000289413	204.5285948	0.924436759	0.143808512	6.428247858	1.29E-10	2.01E-09
ENSG00000289417	35.34293378	-3.07030418	0.399572275	-7.683977017	1.54E-14	3.60E-13
ENSG00000289419	31.35984154	0.161602197	0.318420855	0.507511348	0.611796076	0.74762066
ENSG00000289433	16.8034388	0.348849623	0.443589143	0.78642507	0.431618501	0.597864617
ENSG00000289436	61.83502434	4.180148555	0.360475996	11.59619117	4.31E-31	2.44E-29
ENSG00000289463	18.71602441	-0.22748088	0.402965031	-0.564517669	0.572401879	0.717385398
ENSG00000289468	11.80197756	-0.719107871	0.522578585	-1.376076042	0.168798092	0.3113323
ENSG00000289472	38.39941963	-0.340026982	0.303966089	-1.118634593	0.263296059	0.429810546
ENSG00000289483	18.66875555	0.398116264	0.408471651	0.974648458	0.329734668	0.502217902
ENSG00000289486	12.408607	0.186963264	0.490511785	0.381159575	0.703084843	0.814114448
ENSG00000289488	11.34452937	-0.320935125	0.527819449	-0.608039597	0.543161212	0.692169604
ENSG00000289494	8.154764352	-0.472670117	0.609100417	-0.776013451	0.437741044	0.603314879
ENSG00000289496	13.548487	-0.575294506	0.485051535	-1.186048212	0.23560326	0.396663823
ENSG00000289499	9.006392485	1.203329973	0.595540085	2.020569235	0.043324376	0.10908362
ENSG00000289504	44.90445105	-0.519748679	0.284295452	-1.828199062	0.067519682	0.154747751
ENSG00000289507	10.77014532	-0.67447134	0.53931377	-1.25061027	0.211076702	0.366274791
ENSG00000289511	14.21459647	-0.032272614	0.46541399	-0.069341737	0.944717605	0.969282608
ENSG00000289558	43.5658864	-0.276115617	0.276657572	-0.998041061	0.318259448	0.48997802
ENSG00000289562	8.126146406	-0.555672717	0.629178166	-0.883172283	0.377143195	0.546670343
ENSG00000289579	14.15161261	-0.590882026	0.468680478	-1.260735306	0.207404229	0.361420072
ENSG00000289586	31.94761931	-0.555226841	0.321336714	-1.727866182	0.084012219	0.183533579
ENSG00000289605	34.62784849	4.806330295	0.56946539	8.440074468	3.17E-17	8.87E-16
ENSG00000289606	19.34621401	-0.317149885	0.42531219	-0.745687268	0.455856352	0.61958531
ENSG00000289609	94.50087962	2.258510438	0.212044567	10.65111205	1.72E-26	8.20E-25
ENSG00000289727	39.66068558	-0.484783038	0.296590359	-1.634520554	0.102149594	0.213240503
ENSG00000289740	366.8146384	-0.561306213	0.211667871	-2.651825287	0.008005796	0.026781951
ENSG00000289858	14.84773367	-0.615275161	0.493291506	-1.247285133	0.212292967	0.367432147
ENSG00000289871	14.26127079	-6.138053921	1.2362969	-4.964870431	6.87E-07	6.32E-06
ENSG00000289872	9.4729803	0.548915532	0.575521716	0.953770321	0.340199916	0.511866775
ENSG00000289883	13.74037215	0.256640252	0.473065326	0.542504887	0.587470741	0.729379663
ENSG00000289915	25.69583078	-0.064702463	0.342716097	-0.188793185	0.850254914	0.914361666

ENSG00000289926	239.353515	-0.355196147	0.127668346	-2.782178655	0.005399531	0.019081059
ENSG00000289975	9.871465673	0.28891357	0.58201976	0.496398215	0.619613485	0.753046428
ENSG00000289997	24.2588956	0.105788566	0.351660835	0.300825556	0.76354752	0.857289746
ENSG00000290032	10.49621018	0.677548129	0.544625487	1.244062471	0.213476572	0.368935212
ENSG00000290039	49.63728636	0.796241844	0.248927361	3.19869154	0.001380528	0.005955859
ENSG00000290058	10.51771286	0.518777405	0.548712357	0.945445092	0.344431691	0.516231226
ENSG00000290065	11.845371	0.132145681	0.501832941	0.263326039	0.792299292	0.876125966
ENSG00000290082	14.77532661	0.09475516	0.477686611	0.198362603	0.842761374	0.908748678
ENSG00000290085	15.58084778	-0.636549391	0.463994548	-1.371889806	0.170097744	0.313030658
ENSG00000290121	89.27962832	0.337166713	0.188144119	1.792066181	0.073122365	0.164238866
ENSG00000290292	189.4167702	-0.541609985	0.14116554	-3.836701122	0.000124698	0.000704122
ENSG00000290374	13.29720712	0.264578058	0.468245491	0.565041336	0.572045649	0.717201466
ENSG00000290376	204.7620319	-0.563623567	0.141898177	-3.972028241	7.13E-05	0.000423629
ENSG00000290385	16.18959406	-0.173194371	0.436674127	-0.396621555	0.691646555	0.805869207
ENSG00000290387	15.39490928	0.779545237	0.4537322	1.718073429	0.085783223	0.186330127
ENSG00000290399	23.33386564	-0.186790159	0.357231394	-0.522882821	0.6010558	0.739384432
ENSG00000290445	29.81604744	1.074281323	0.33366329	3.219656926	0.001283441	0.005574674
ENSG00000290450	29.96714337	-0.097187206	0.315342916	-0.308195304	0.757933726	0.853990137
ENSG00000290474	14.46236296	-0.154792151	0.449000423	-0.344748341	0.730283573	0.83331836
ENSG00000290535	38.44345794	-0.184551351	0.28747994	-0.641962533	0.52089751	0.675253537
ENSG00000290548	41.78871333	-0.184571065	0.275391922	-0.670212342	0.502722437	0.660716886
ENSG00000290555	9.693497738	0.574227718	0.593524683	0.967487509	0.333300382	0.505601563
ENSG00000290571	252.3699424	-0.280286624	0.121173542	-2.313100854	0.020717095	0.059487537
ENSG00000290576	24.65438527	0.268142047	0.348285024	0.769892553	0.441363632	0.60654127
ENSG00000290588	26.27023372	-0.63437436	0.351966639	-1.802370705	0.071487101	0.161651531
ENSG00000290591	27.46195987	-0.160387105	0.335076917	-0.478657575	0.632182252	0.761927421
ENSG00000290600	32.67739197	0.153176668	0.32848145	0.466317561	0.640988211	0.768258186
ENSG00000290654	29.01443199	-0.464427173	0.329217869	-1.410698555	0.158333517	0.296587667
ENSG00000290682	52.48505855	-0.024201598	0.268037083	-0.090291975	0.928055197	0.960813764
ENSG00000290683	14.42837069	-0.383851615	0.460250767	-0.834005379	0.404277953	0.57241129
ENSG00000290687	8.692565507	0.084580831	0.653923946	0.129343529	0.89708583	0.944134611
ENSG00000290706	15.86907086	-0.884606268	0.468235762	-1.889232605	0.058860668	0.138564739
ENSG00000290707	12.83169173	0.48622409	0.502342041	0.96791439	0.333087127	0.505387219
ENSG00000290712	9.157783854	-0.654271534	0.568320868	-1.15123616	0.249635093	0.413789489
ENSG00000290738	16.39181505	0.620364858	0.425650667	1.457450688	0.144992014	0.278010213
ENSG00000290746	61.4227179	0.664138375	0.231895985	2.863949437	0.004183948	0.015399522
ENSG00000290762	13.53607233	0.26935137	0.486175128	0.554021286	0.579564278	0.722800027
ENSG00000290770	340.9656643	-0.21057458	0.128945531	-1.633050618	0.102458351	0.213754163
ENSG00000290791	61.49469343	-0.021828357	0.221495418	-0.098549925	0.92149563	0.956727767
ENSG00000290792	39.92256848	-0.688982035	0.284688424	-2.420126623	0.015515103	0.046889126
ENSG00000290796	26.93035815	-0.247047214	0.363757754	-0.679153119	0.497040848	0.655435794
ENSG00000290832	25.55746782	0.581478543	0.349695692	1.662813003	0.096349873	0.203737267
ENSG00000290835	27.80236745	0.204396767	0.335671865	0.608918377	0.542578543	0.691866448
ENSG00000290842	34.72129462	0.76381962	0.297912346	2.563907234	0.01035012	0.033269597
ENSG00000290854	7.376039285	0.412894044	0.636869982	0.64831764	0.516779527	0.671712966
ENSG00000290865	34.45035344	0.309722257	0.31529206	0.982334467	0.3259351	0.498086417
ENSG00000290867	10.49523009	-0.821599965	0.549037574	-1.496436681	0.134539897	0.263178099
ENSG00000290872	11.11877105	0.210824808	0.530320988	0.39754189	0.690967902	0.805479346
ENSG00000290873	22.89789258	-0.07724541	0.363064314	-0.212759579	0.831514493	0.901477895
ENSG00000290890	19.79946316	0.098471421	0.414046585	0.237826912	0.812015345	0.889455673

ENSG00000290907	15.85313065	-0.571245547	0.443427645	-1.288249736	0.197659044	0.349203445
ENSG00000290920	15.0601738	0.904630537	0.455301104	1.98688413	0.046935239	0.116091213
ENSG00000290921	38.17089421	0.001907556	0.283237743	0.006734824	0.994626429	0.99758811
ENSG00000290927	26.69394616	0.035136148	0.333452362	0.105370817	0.916081571	0.954614083
ENSG00000290928	41.5308825	-0.025615651	0.287958515	-0.088956048	0.92911684	0.961351227
ENSG00000290931	122.1806726	-0.222750639	0.16431358	-1.355643514	0.17521262	0.319720924
ENSG00000290937	15.37277282	0.059759665	0.437171015	0.136696311	0.891270845	0.94027635
ENSG00000290945	41.44329169	-0.455349896	0.296530683	-1.53559116	0.124638684	0.248869878
ENSG00000290948	16.83191703	-0.089075186	0.414188733	-0.215059413	0.829720997	0.900563348
ENSG00000290989	55.66439783	0.491811482	0.242176969	2.030793777	0.042275919	0.107013417
ENSG00000290993	47.33287357	0.126303393	0.256617819	0.492184814	0.622588699	0.75505595
ENSG00000290994	26.30180865	-0.500336957	0.339385645	-1.474243137	0.140416139	0.271518409
ENSG00000291003	12.5038522	-0.811967658	0.524441106	-1.548253273	0.12156133	0.244392729
ENSG00000291006	39.83404131	-0.160814704	0.276677482	-0.581235244	0.561081917	0.7074971
ENSG00000291015	30.08015021	-0.547609452	0.322078441	-1.700236283	0.08908649	0.191785123
ENSG00000291027	17.12611789	0.660389354	0.431585974	1.530145542	0.125980708	0.250907557
ENSG00000291035	76.29255421	0.182946783	0.208233672	0.878564839	0.379637267	0.549203367
ENSG00000291040	22.11980299	0.590341804	0.371143719	1.590601629	0.111699258	0.228656291
ENSG00000291041	10.97555476	-0.350138994	0.539291925	-0.649256883	0.51617235	0.671234308
ENSG00000291043	20.35362258	0.725737708	0.383005443	1.894849594	0.058112331	0.13717088
ENSG00000291044	24.06805103	0.584982404	0.352042642	1.66168053	0.096576847	0.204032933
ENSG00000291049	24.08036712	-0.006369412	0.350101528	-0.018193044	0.985484852	0.992555924
ENSG00000291050	11.05689492	1.207088825	0.543046615	2.222808856	0.026228692	0.072199575
ENSG00000291054	12.84010878	-0.215345795	0.480031113	-0.448607995	0.653714465	0.777735843
ENSG00000291055	34.42264821	0.364025037	0.30500897	1.193489612	0.232677678	0.392697747
ENSG00000291060	12.89977235	0.471441329	0.486449797	0.969146933	0.332471884	0.504890006
ENSG00000291063	30.69890317	0.99901231	0.328498222	3.041149821	0.002356765	0.009388963
ENSG00000291064	20.57196025	-0.179643182	0.416469797	-0.431347443	0.666215758	0.786348438
ENSG00000291067	15.02889987	0.35905656	0.445848353	0.805333377	0.420627326	0.588266831
ENSG00000291068	48.37156401	0.328660808	0.254200328	1.292920469	0.196038567	0.347103126
ENSG00000291069	14.16614581	-0.309545953	0.458578116	-0.675012484	0.499667834	0.657810127
ENSG00000291071	33.76740417	-0.102848721	0.314412474	-0.327113997	0.743581666	0.843162065
ENSG00000291072	57.31982544	0.397219659	0.243765719	1.629514028	0.103204248	0.21493107
ENSG00000291073	22.16707577	0.362791597	0.391358127	0.927006679	0.353923067	0.525367993
ENSG00000291074	14.48993075	1.026531433	0.494219225	2.077077096	0.037794444	0.097277032
ENSG00000291075	53.05667536	-0.244905231	0.254774736	-0.961261839	0.33642053	0.508303667
ENSG00000291078	11.36038251	-0.239952867	0.518026666	-0.463205627	0.643216983	0.769810766
ENSG00000291079	11.86893707	-0.468922031	0.520870046	-0.900266842	0.367978262	0.539391699
ENSG00000291080	57.985272	-0.246015807	0.245565424	-1.001834064	0.316423742	0.488011013
ENSG00000291081	21.03402563	-0.332817157	0.387071014	-0.859834875	0.389880072	0.558214797
ENSG00000291087	37.85680322	0.329996841	0.297152348	1.110530821	0.266770355	0.433517257
ENSG00000291088	7.694404616	0.252900317	0.615553455	0.410850293	0.681182313	0.798112265
ENSG00000291091	25.39237267	0.697338295	0.346226316	2.014111186	0.043997861	0.110423076
ENSG00000291092	11.09719766	0.534852575	0.56129057	0.952897846	0.340641831	0.512421911
ENSG00000291093	59.39426832	-0.055057494	0.236680586	-0.232623616	0.816053687	0.891910855
ENSG00000291100	29.28268539	-0.269747408	0.327908106	-0.822631107	0.410717804	0.579077887
ENSG00000291102	26.9009456	-0.475894243	0.359017795	-1.325545	0.184990454	0.332807494
ENSG00000291104	13.47174783	-0.824485501	0.487401519	-1.691594031	0.090723405	0.194593323
ENSG00000291106	20.89421569	-0.366422035	0.400683527	-0.914492388	0.360458176	0.531753413
ENSG00000291110	8.106929226	0.031034673	0.626060794	0.04957134	0.960463986	0.979002801

ENSG00000291113	75.83888619	0.670111975	0.211096936	3.174427772	0.001501323	0.006396444
ENSG00000291117	34.0407543	0.222036904	0.305713917	0.726289814	0.467661122	0.629756504
ENSG00000291118	148.4638263	-0.362326013	0.148807376	-2.434865949	0.014897307	0.045265516
ENSG00000291120	10.65492545	0.240813425	0.528157721	0.455949834	0.648426053	0.773796594
ENSG00000291122	31.5958413	-0.873761493	0.3146042	-2.777335755	0.005480653	0.01932398
ENSG00000291129	15.59376643	-0.32553018	0.457883377	-0.710945618	0.477117936	0.637712665
ENSG00000291132	14.59444322	-0.241971002	0.448894956	-0.539037025	0.589861307	0.731184414
ENSG00000291133	66.32370413	0.230510843	0.234557602	0.982747272	0.325731838	0.497884128
ENSG00000291136	125.8114189	0.051585569	0.175213429	0.294415612	0.768440339	0.860787994
ENSG00000291137	13.01365833	0.108462309	0.478302641	0.226765023	0.820606463	0.894680145
ENSG00000291138	9.128831401	-1.833965683	0.634402247	-2.890856222	0.003841938	0.014292296
ENSG00000291140	21.87647259	0.013864883	0.372275112	0.037243646	0.970290738	0.984518492
ENSG00000291141	25.14046099	-0.111752965	0.351056993	-0.318332828	0.750232485	0.84850184
ENSG00000291142	17.49138535	0.411084924	0.445906879	0.921907562	0.356576794	0.527856911
ENSG00000291145	9.750070644	-0.00564572	0.547204035	-0.010317395	0.991768056	0.995808855
ENSG00000291146	12.56163342	0.560372358	0.493914705	1.134552895	0.256562699	0.422352776
ENSG00000291147	8.844678958	0.467696639	0.588804816	0.794315241	0.427011934	0.594296704
ENSG00000291152	48.08773402	0.094107409	0.254014388	0.370480625	0.71102441	0.819206028
ENSG00000291153	44.60673635	-0.213423604	0.2584841	-0.825674011	0.408989039	0.577621114
ENSG00000291154	21.25508743	-0.198819075	0.382683047	-0.519539802	0.603384364	0.741253138
ENSG00000291158	15.80152861	-0.092614599	0.434832486	-0.212989143	0.831335431	0.901477895
ENSG00000291159	93.23597272	0.840751518	0.190677169	4.409293066	1.04E-05	7.41E-05
ENSG00000291171	188.965407	0.120909395	0.133926979	0.902800888	0.36663158	0.537922895
ENSG00000291174	65.35394326	0.148981842	0.218730661	0.681120064	0.495795522	0.654552604
ENSG00000291175	63.39964685	-0.023652585	0.228756301	-0.103396431	0.917648344	0.955355281
ENSG00000291176	10.34718272	-1.130991364	0.557662471	-2.028093018	0.042550755	0.107540565
ENSG00000291177	8.631150476	0.446211194	0.610297279	0.731137446	0.464695204	0.627409989
ENSG00000291178	71.60673765	0.080404564	0.205998018	0.390317175	0.696302024	0.809436951
ENSG00000291182	14.73137431	-0.330705346	0.454293993	-0.727954477	0.466641453	0.628895314
ENSG00000291185	43.12292992	0.465762649	0.272532168	1.709018985	0.087447433	0.188922499
ENSG00000291186	68.55443376	0.424486726	0.210113978	2.02026886	0.043355507	0.109122883
ENSG00000291201	175.3168214	0.219886303	0.142896112	1.538784359	0.123856949	0.247978701
ENSG00000291202	104.4032182	0.044380735	0.175481514	0.252908321	0.800339059	0.880924836
ENSG00000291203	138.6654117	-0.118630016	0.151161743	-0.784788622	0.432577512	0.598956759
ENSG00000291207	81.82307081	-0.075299211	0.199796955	-0.376878674	0.706263771	0.815661201
ENSG00000291208	61.98283508	-0.1930713	0.225450198	-0.856381151	0.391786987	0.560088725
ENSG00000291212	22.36796887	-0.199021403	0.378031383	-0.526467938	0.598563123	0.737415909
ENSG00000291214	89.212706	0.095337206	0.190653766	0.500054146	0.617036952	0.751149845
ENSG00000291215	39.82335704	-0.205937187	0.286398154	-0.719059057	0.472104532	0.633819399
ENSG00000291220	8.720860482	-0.596386271	0.58155081	-1.025510173	0.305122528	0.476122656
ENSG00000291223	61.18420172	0.113333209	0.227680547	0.497772915	0.618644108	0.752323937
ENSG00000291224	170.8120931	-0.296853138	0.147036686	-2.018905261	0.043497067	0.109361608
ENSG00000291228	41.03100765	0.582762378	0.278778194	2.090415931	0.036580453	0.09485797
ENSG00000291237	416.3822987	-0.093071459	0.099735356	-0.933184205	0.350724868	0.522500564
ENSG00000291242	8.638099348	0.669902625	0.701842041	0.954492017	0.339834648	0.511646013
ENSG00000291258	35.9569744	-1.167734621	0.312829675	-3.732812824	0.000189353	0.001020271
ENSG00000291272	27.60309966	0.509891151	0.333597712	1.528461175	0.126398076	0.25141798
ENSG00000291299	45.74573297	-0.092479477	0.257889814	-0.358600734	0.719893797	0.825834885
ENSG00000291317	29.95566666	0.220369525	0.351804157	0.626398298	0.531053725	0.683094573
