# Supplementary material for: A Stem-like Patient-Derived Ovarian Cancer Model of Platinum Resistance Reveals Dissociation of Stemness and Resistance
Source: Int J Mol Sci. 2024 Mar 29;25(7):3843. doi: 10.3390/ijms25073843 (PMC11011340; doi:10.3390/ijms25073843)
Supplement: Supplementary file 1 [file ijms-25-03843-s001.zip › Table S2.pdf]

Table S2. Subset of genes differentially expressed in RNA-seq that are associated with cancer stemness, epithelial-mesenchymal transition, and cisplatin resistance

ENSEMBL ID	Gene Symbol	log2FoldChange	P-Value	Adj. P-Value	Correlation
ENSG00000182621	PLCB1	8.356265503	2.01225E-58	2.57065E-56	Stemness
ENSG00000067066	SP100	8.082933832	5.25858E-15	1.25839E-13	Stemness
ENSG00000071575	TRIB2	6.20964948	2.4397E-70	3.98493E-68	Cisplatin Resistance, Stemness
ENSG00000183971	NPW	6.06781425	2.2249E-102	6.1282E-100	Stemness
ENSG00000084636	COL16A1	5.754984029	1.1499E-198	9.5012E-196	EMT, Stemness
ENSG00000040731	CDH10	5.65704701	6.72638E-39	5.0527E-37	Stemness
ENSG00000024422	EHD2	5.404936271	4.75805E-51	5.2627E-49	Stemness
ENSG00000165379	LRFN5	5.371701094	1.8727E-160	1.0522E-157	Stemness
ENSG00000185008	ROBO2	5.315643924	5.24846E-80	9.70067E-78	Stemness
ENSG00000118785	SPP1	4.939013763	8.0586E-98	1.95171E-95	Cisplatin Resistance, EMT, Stemness
ENSG00000170396	ZNF804A	4.802047513	0	0	Stemness
ENSG00000064763	FAR2	4.4161414	3.60164E-31	2.0566E-29	Stemness
ENSG00000141837	CACNA1A	4.409561452	3.9282E-112	1.1996E-109	Stemness
ENSG00000001084	GCLC	4.21014563	0	0	Cisplatin Resistance, Stemness
ENSG00000154122	ANKH	3.902398136	2.29728E-34	1.44708E-32	Stemness
ENSG00000145147	SLIT2	-5.515043392	1.86557E-54	2.20216E-52	EMT, Stemness
ENSG00000135333	EPHA7	-5.633873415	6.31167E-66	9.63696E-64	Stemness
ENSG00000137872	SEMA6D	-5.736345708	4.07951E-57	5.02674E-55	Stemness
ENSG00000079385	CEACAM1	-6.129294961	1.25868E-61	1.71657E-59	EMT, Stemness
ENSG00000115828	QPCT	-6.228380664	1.30957E-20	4.58742E-19	Stemness
ENSG00000156103	MMP16	-6.39045084	1.11187E-17	3.22028E-16	Stemness
ENSG00000163132	MSX1	-6.42503743	2.32125E-95	5.43444E-93	Cisplatin Resistance, Stemness
ENSG00000186197	EDARADD	-6.757327566	8.21212E-38	5.94616E-36	Stemness
ENSG00000182568	SATB1	-7.393480032	8.89177E-24	3.66284E-22	EMT, Stemness
ENSG00000137203	TFAP2A	-7.528844143	2.62135E-30	1.444E-28	Stemness
ENSG00000134853	PDGFRA	-7.560090284	7.77318E-14	1.71144E-12	EMT, Stemness
ENSG00000007372	PAX6	-7.57531094	1.27013E-59	1.65199E-57	Stemness
ENSG00000122870	BICC1	-7.969495544	2.57203E-15	6.36078E-14	EMT, Stemness
ENSG00000075213	SEMA3A	-8.186816235	7.12969E-12	1.27418E-10	Stemness
ENSG00000182263	FIGN	-8.199552815	6.18455E-12	1.11377E-10	Stemness
ENSG00000176907	TCIM	-9.017917239	4.27281E-14	9.74354E-13	Stemness
ENSG00000157110	RBPMS	-9.451583696	1.51139E-15	3.80476E-14	Stemness
ENSG00000114251	WNT5A	-10.31533138	3.34841E-18	1.02028E-16	EMT, Stemness
