# Supplementary material for: A Stem-like Patient-Derived Ovarian Cancer Model of Platinum Resistance Reveals Dissociation of Stemness and Resistance
Source: Int J Mol Sci. 2024 Mar 29;25(7):3843. doi: 10.3390/ijms25073843 (PMC11011340; doi:10.3390/ijms25073843)
Supplement: Supplementary file 1 [file ijms-25-03843-s001.zip › Table S3.pdf]

Table S3. List of differentially expressed micro-RNAs in PDX4 CR relative to PDX4 SE generated by QIAGEN CLC Genomics Workbench and QIAGEN Ingenuity Pathway Analysis.

Name	Fold change	FDR p-value	P-value	Cisplatin Resistant - Min CPM	Cisplatin Resistant - Mean CPM	Cisplatin Resistant - Max CPM	Cisplatin Sensitive - Min CPM	Cisplatin Sensitive - Mean CPM	Cisplatin Sensitive - Max CPM
hsa-miR-548ap-3p	-3.836717681		0.44908091	0	0	0	0	0.332284867	0.996854601
hsa-miR-548t-3p				0	0	0	0	0	0
hsa-miR-548e-3p	1.409919696	0.43401933	0.22492973	7.878460651	14.07374676	18.44392223	7.974836806	9.697127049	11.36909687
hsa-miR-548az-3p	3.543660986		0.51087004	0	0.175656402	0.526969207	0	0	0
hsa-miR-548f-3p	-3.388438971	0.0996189	0.02399579	0.836781969	1.034263751	1.212070869	3.100662783	3.987268307	4.873723736
hsa-miR-548bc	-1.008195306	0.9772691	0.96649536	15.7569213	17.6399532	19.24598529	13.95596441	17.35161202	19.49489494
hsa-miR-1537-3p	1.223440543		0.81257339	0	0.682950946	1.212070869	0	0.497173959	0.974744747
hsa-miR-553				0	0	0	0	0	0
hsa-miR-7844-5p				0	0	0	0	0	0
hsa-miR-548av-3p	-3.836695655		0.44908316	0	0	0	0	0.324914916	0.974744747
hsa-miR-548g-3p	-3.835816219		0.44917283	0	0	0	0	0.172259043	0.51677713
hsa-miR-6835-3p				0	0	0	0	0	0
hsa-miR-4422	-2.816715966		0.30243633	0	0.175656402	0.526969207	0	0.669433003	1.033554261
hsa-miR-4429	-1.127278468		0.92096409	0	0.175656402	0.526969207	0	0.324914916	0.974744747
hsa-miR-320d	2.204508332	0.06212745	0.011972	10.30260239	17.1344418	26.87542953	3.987418403	7.812004108	11.69693697
hsa-miR-320b	1.655560828	0.09253034	0.0210935	23.63538195	32.05535654	47.42722859	14.95281901	19.50033368	23.39387393
hsa-miR-320a-3p	1.635461861	0.001467	0.0001949	1354.489197	1627.540841	2056.233844	828.3937401	996.2546108	1222.329913
hsa-miR-320c	1.748842554	0.02690705	0.004596	23.63538195	31.88935637	36.88784446	15.94967361	18.16883762	20.46963969
hsa-miR-548an				0	0	0	0	0	0
hsa-miR-4484	-1.611385864	0.54326907	0.32278907	0.606035435	1.534877548	3.161815239	1.993709202	2.672868742	3.100662783
hsa-miR-10526-3p				0	0	0	0	0	0
hsa-miR-548bb-5p	3.543845864		0.51085051	0	0.202011812	0.606035435	0	0	0
hsa-miR-548ak	3.543660986		0.51087004	0	0.175656402	0.526969207	0	0	0
hsa-miR-548w	1.04458591	0.96770494	0.9461822	0.606035435	1.111179262	1.673563938	0	1.009087821	1.993709202
hsa-miR-548y				0	0	0	0	0	0
hsa-miR-548h-5p	-1.868629795		0.3723568	0	0.682950946	1.212070869	0.51677713	1.49402856	2.990563802
hsa-miR-548ar-5p	8.433010632	0.02268279	0.003742	2.634846033	4.899072176	7.878460651	0	0.50454391	0.996854601
hsa-miR-548a-5p	2.586419001	0.23448634	0.0866059	0.836781969	2.492225787	4.215753652	0	1.009087821	1.993709202
hsa-miR-548i	-1.468681138	0.61606818	0.4102144	1.58090762	1.690859287	1.818106304	1.949489494	2.347953826	3.100662783
hsa-miR-548as-5p	-1.178047529	0.90300712	0.78963688	0	1.086974569	2.424141739	0.974744747	1.173976913	1.550331391
hsa-miR-548ap-5p	3.543845864		0.51085051	0	0.202011812	0.606035435	0	0	0
hsa-miR-548j-5p	1.504311319	0.10248718	0.02513554	37.65518861	41.8692417	47.9541978	27.90596504	28.36078204	28.90878342
hsa-miR-548au-5p	-1.036185439	0.95639406	0.91303246	3.347127876	6.025396071	7.878460651	5.848468483	6.514851128	6.977982205
hsa-miR-548c-5p	-1.10363903	0.78927331	0.61669823	18.40920332	20.81275062	22.42331108	17.54540545	21.64836157	29.45629643
hsa-miR-548ab	-9.586756314		0.16940324	0	0	0	0	0.676802954	1.033554261
hsa-miR-548b-5p	1.199976739	0.67661118	0.47510946	8.484496086	9.894963124	11.71494757	7.751656957	8.166398829	8.772702724
hsa-miR-548ad-5p	-1.453501343	0.18981192	0.06123859	15.06207544	17.88776649	21.21124021	18.94023741	24.98329336	33.59051348
hsa-miR-548ay-5p	-1.622133594	0.19947798	0.06756043	4.742722859	7.198346212	8.484496086	10.72219222	11.19115561	11.885874
hsa-miR-548d-5p	-1.798388577	0.006325	0.0008956	31.79771483	34.21667934	38.18023239	54.82700304	59.8785297	69.24813548
hsa-miR-548av-5p				0	0	0	0	0	0
hsa-miR-548k	3.07903166	0.0006418	0.00007402	17.57242135	23.01053688	32.67209081	3.987418403	7.487089193	10.72219222
hsa-miR-548l	-1.072580026	0.94510719	0.87816653	1.673563938	2.042172092	2.634846033	1.550331391	2.481006527	3.898978988
hsa-miR-548ah-5p	1.058549475	0.94510719	0.87050958	3.688784446	4.038314111	4.242248043	2.924234241	3.681956563	4.134217044
hsa-miR-106a-5p	-1.258889763	0.14233405	0.0388264	204.1748005	217.3424939	229.6874298	270.0042949	274.8552646	279.1192882
hsa-miR-548at-5p	4.020033725		0.14423453	0.606035435	1.183564743	2.107876826	0	0.172259043	0.51677713
hsa-miR-3646				0	0	0	0	0	0
hsa-miR-224-3p				0	0	0	0	0	0
hsa-miR-522-3p	33.58040217	0.154705	0.0449435	2.107876826	2.828393959	3.347127876	0	0	0
hsa-miR-3606-3p				0	0	0	0	0	0
hsa-miR-495-3p	-6.710501692		0.25817211	0	0	0	0	0.344518087	1.033554261
hsa-miR-122b-3p				0	0	0	0	0	0
hsa-miR-543				0	0	0	0	0	0
hsa-miR-451a	-4.580437646		0.16260368	0	0.175656402	0.526969207	0	1.329139468	3.987418403
hsa-miR-649				0	0	0	0	0	0
hsa-miR-5583-5p	3.544185779		0.5108146	0	0.278927323	0.836781969	0	0	0
hsa-miR-3129-3p	-6.197224937		0.04718578	0	0.202011812	0.606035435	0.974744747	1.838546647	2.990563802
hsa-miR-606				0	0	0	0	0	0
hsa-miR-618				0	0	0	0	0	0
hsa-miR-641	1.221070162	0.65871246	0.45196329	9.20460166	12.28153868	13.938815	7.797957977	10.36420347	12.95910981
hsa-miR-3617-5p				0	0	0	0	0	0
hsa-miR-3667-5p				0	0	0	0	0	0
hsa-miR-548ao-3p	3.543845864		0.51085051	0	0.202011812	0.606035435	0	0	0
hsa-miR-4432				0	0	0	0	0	0
hsa-miR-4662a-3p				0	0	0	0	0	0
hsa-miR-3156-5p				0	0	0	0	0	0
hsa-miR-4662b				0	0	0	0	0	0
hsa-miR-10527-5p	3.615577372	0.03307497	0.005939	4.242248043	5.753378091	6.694255753	0	1.956859445	4.873723736
hsa-miR-498-3p				0	0	0	0	0	0
hsa-miR-518e-3p				0	0	0	0	0	0
hsa-miR-320e	-1.972268678		0.5371786	0	0.278927323	0.836781969	0	0.657199783	0.996854601
hsa-miR-651-3p	3.450803474		0.21382094	0	1.240805592	2.510345907	0	0.332284867	0.996854601
hsa-miR-3173-3p	6.045864117		0.32988072	0	0.351312804	1.053938413	0	0	0
hsa-miR-541-5p				0	0	0	0	0	0
hsa-miR-3910				0	0	0	0	0	0
hsa-miR-548ba	4.610289413		0.10439595	0	1.282305634	2.634846033	0	0.332284867	0.996854601
hsa-miR-570-5p				0	0	0	0	0	0
hsa-miR-548ag	-1.606871867	0.50388098	0.28393877	0	2.09035288	3.636212608	1.993709202	3.514560788	4.650994174
hsa-miR-3974				0	0	0	0	0	0
hsa-miR-4735-3p				0	0	0	0	0	0
hsa-miR-562				0	0	0	0	0	0
hsa-miR-3184-3p				0	0	0	0	0	0
hsa-miR-519b-3p	3.543845864		0.51085051	0	0.202011812	0.606035435	0	0	0
hsa-miR-519a-3p	11.53922808	0.03480969	0.006454	3.347127876	3.733031379	4.215753652	0	0.172259043	0.51677713
hsa-miR-519c-3p				0	0	0	0	0	0
hsa-miR-372-3p				0	0	0	0	0	0
hsa-miR-520a-3p				0	0	0	0	0	0
hsa-miR-520b-3p				0	0	0	0	0	0
hsa-miR-520c-3p				0	0	0	0	0	0
hsa-miR-520e-3p				0	0	0	0	0	0
hsa-miR-520d-3p				0	0	0	0	0	0
hsa-miR-148a-5p	-1.556733462	0.44717479	0.23370595	1.212070869	3.730880662	5.796661272	4.134217044	6.296015791	8.772702724
hsa-miR-1277-5p	-2.680220376	0.05012282	0.009366	1.818106304	2.145443013	2.510345907	4.984273004	5.830678223	6.82321323
hsa-miR-4517	1.449939054	0.55762999	0.33783423	3.347127876	4.260000586	5.796661272	1.550331391	3.485231079	5.981127605
hsa-miR-216b-5p				0	0	0	0	0	0
hsa-miR-4506				0	0	0	0	0	0
hsa-miR-513b-3p				0	0	0	0	0	0
hsa-miR-944	-3.835816219		0.44917283	0	0	0	0	0.172259043	0.51677713
hsa-miR-4760-3p				0	0	0	0	0	0
hsa-miR-3529-3p				0	0	0	0	0	0
hsa-miR-338-5p	1.219621136	0.89225575	0.76299426	0	1.363751176	2.510345907	0	1.147003791	2.924234241
hsa-miR-362-3p	1.125975824	0.86996003	0.73406846	4.742722859	5.678613297	7.272425217	3.100662783	5.633952739	6.977982205
hsa-miR-329-3p	-3.835816219		0.44917283	0	0	0	0	0.172259043	0.51677713
hsa-miR-6757-3p				0	0	0	0	0	0
hsa-miR-552-3p				0	0	0	0	0	0
hsa-miR-376c-3p	-21.08842941		0.05586631	0	0	0	0.974744747	1.678520823	2.067108522
hsa-miR-499a-3p				0	0	0	0	0	0
hsa-miR-499b-3p				0	0	0	0	0	0
hsa-miR-181c-5p	1.334901267	0.39076896	0.18753308	21.21124021	36.05084466	44.26541335	25.83885652	26.99980618	29.24234241
hsa-miR-181a-5p	-1.434809918	0.20403358	0.0694012	39.39230326	59.41722437	76.93750416	66.78925825	85.71366014	116.9693697
hsa-miR-181b-5p	1.007456192	0.9772691	0.96617782	70.90614586	109.4001539	150.1862239	88.3688893	109.3244949	123.6099705
hsa-miR-181d-5p	1.332996218	0.19034956	0.06224569	97.57170499	118.3919446	149.6592547	84.75144939	89.68023793	93.57549572

hsa-miR-1255b-2-3p				0	0	0	0	0	0
hsa-miR-634				0	0	0	0	0	0
hsa-miR-181c-3p	-1.150818394	0.60445661	0.39532345	53.55404602	67.72107683	76.36046477	76.03009027	79.46313329	81.74207726
hsa-miR-4482-5p	8.549500881		0.23672994	0.526969207	0.656595537	0.836781969	0	0	0
hsa-miR-5195-5p				0	0	0	0	0	0
hsa-miR-100-5p	-2.783760591	0.1193964	0.02997983	0	1.993991303	3.347127876	1.949489494	5.227424685	7.751656957
hsa-miR-99a-5p	-4.510528186	1.279E-15	4.669E-17	96.96566955	170.1910089	252.9452191	641.837196	769.1850131	924.0580203
hsa-miR-6813-3p				0	0	0	0	0	0
hsa-miR-521				0	0	0	0	0	0
hsa-miR-122-3p	3.543845864		0.51085051	0	0.202011812	0.606035435	0	0	0
hsa-miR-548al	6.046179412		0.32986518	0	0.377668214	0.606035435	0	0	0
hsa-miR-4681				0	0	0	0	0	0
hsa-miR-4661-5p	-1.167760937		0.8406365	0.526969207	0.656595537	0.836781969	0	0.822088875	1.949489494
hsa-miR-3923				0	0	0	0	0	0
hsa-miR-4798-3p				0	0	0	0	0	0
hsa-miR-3145-5p	3.543845864		0.51085051	0	0.202011812	0.606035435	0	0	0
hsa-miR-4636				0	0	0	0	0	0
hsa-miR-5684				0	0	0	0	0	0
hsa-miR-4714-5p				0	0	0	0	0	0
hsa-miR-3664-5p				0	0	0	0	0	0
hsa-miR-4724-5p				0	0	0	0	0	0
hsa-miR-1243	1.430120254	0.7628317	0.55681146	0.836781969	1.411931964	1.818106304	0	0.994347918	1.949489494
hsa-miR-193b-3p	2.060426917	0.25882881	0.10144839	0.836781969	6.216197105	10.53938413	1.993709202	3.15043953	4.873723736
hsa-miR-193a-3p	-1.04263835	0.96612968	0.9344061	1.053938413	1.717214697	2.424141739	0.974744747	1.838546647	2.990563802
hsa-miR-452-5p				0	0	0	0	0	0
hsa-miR-5197-3p				0	0	0	0	0	0
hsa-miR-483-5p	1.513623305		0.70103844	0	0.404023623	1.212070869	0	0.332284867	0.996854601
hsa-miR-4499				0	0	0	0	0	0
hsa-miR-3925-5p	-3.836695655		0.44908316	0	0	0	0	0.324914916	0.974744747
hsa-miR-6856-5p				0	0	0	0	0	0
hsa-miR-3916	-1.127226131		0.92099351	0	0.202011812	0.606035435	0	0.332284867	0.996854601
hsa-miR-576-3p	1.61642808	0.08476164	0.01917964	18.78709848	25.20045542	30.03724477	13.64642646	15.21763026	17.0536453
hsa-miR-3942-5p	2.586610977	0.23448634	0.08654268	1.673563938	2.674562936	4.242248043	0.51677713	1.161743693	1.993709202
hsa-miR-6838-5p				0	0	0	0	0	0
hsa-miR-646				0	0	0	0	0	0
hsa-miR-1179	1.047063781	0.94268083	0.86148642	7.878460651	13.86720492	19.49786064	10.72219222	13.36935707	15.94967361
hsa-miR-6779-3p				0	0	0	0	0	0
hsa-miR-129-2-3p	-4.623515005	0.1319728	0.03510811	0	0.454583725	0.836781969	1.550331391	2.488376478	2.990563802
hsa-miR-129-1-3p	3.543845864		0.51085051	0	0.202011812	0.606035435	0	0	0
hsa-miR-6819-3p				0	0	0	0	0	0
hsa-miR-596				0	0	0	0	0	0
hsa-miR-4670-5p				0	0	0	0	0	0
hsa-miR-6854-5p				0	0	0	0	0	0
hsa-miR-22-3p	1.070569157	0.7723693	0.57755542	173.2138676	198.4146023	228.7046356	167.4357903	188.1676717	205.6711416
hsa-miR-208b-5p				0	0	0	0	0	0
hsa-miR-3914				0	0	0	0	0	0
hsa-miR-6888-5p				0	0	0	0	0	0
hsa-miR-28-5p	1.106702442	0.78066984	0.59999424	21.7563312	26.52188178	34.77996763	22.41912918	24.86096116	28.90878342
hsa-miR-708-5p	-1.650875011		0.55359289	0	0.377668214	0.606035435	0	0.669433003	1.033554261
hsa-miR-940	1.107051076		0.8890658	0	0.728981018	1.58090762	0	0.669433003	1.033554261
hsa-miR-4747-3p				0	0	0	0	0	0
hsa-miR-3142				0	0	0	0	0	0
hsa-miR-9500				0	0	0	0	0	0
hsa-miR-7845-5p				0	0	0	0	0	0
hsa-miR-4739				0	0	0	0	0	0
hsa-miR-3158-3p	-1.866902125	0.57236579	0.36420769	0.526969207	0.93552286	1.673563938	0	1.964229397	3.898978988
hsa-miR-6743-5p				0	0	0	0	0	0
hsa-miR-6085				0	0	0	0	0	0
hsa-miR-4716-3p				0	0	0	0	0	0
hsa-miR-4678				0	0	0	0	0	0
hsa-miR-4464				0	0	0	0	0	0
hsa-miR-1244				0	0	0	0	0	0
hsa-miR-5588-3p				0	0	0	0	0	0
hsa-miR-6838-3p				0	0	0	0	0	0
hsa-miR-1245a	1.647787834	0.53977044	0.31834636	1.58090762	3.614615826	5.020691815	0.974744747	1.690754043	3.100662783
hsa-miR-371b-3p				0	0	0	0	0	0
hsa-miR-371a-3p				0	0	0	0	0	0
hsa-miR-519e-3p				0	0	0	0	0	0
hsa-miR-512-3p				0	0	0	0	0	0
hsa-miR-520f-3p	6.046494721		0.32984963	0	0.404023623	1.212070869	0	0	0
hsa-miR-648				0	0	0	0	0	0
hsa-miR-148b-5p	-1.259471868	0.39706008	0.19708091	49.53510541	56.25724164	64.84579151	48.73723736	70.03323079	80.74522266
hsa-miR-4715-5p	-3.836695655		0.44908316	0	0	0	0	0.324914916	0.974744747
hsa-miR-5590-3p				0	0	0	0	0	0
hsa-miR-369-3p	-1.971805422		0.52426684	0	0.278927323	0.836781969	0	0.497173959	0.974744747
hsa-miR-5692b				0	0	0	0	0	0
hsa-miR-5692c				0	0	0	0	0	0
hsa-miR-7705	1.622637366	0.62264757	0.41631035	1.212070869	1.664503878	2.107876826	0.51677713	1.161743693	1.993709202
hsa-miR-1295b-3p				0	0	0	0	0	0
hsa-miR-410-3p				0	0	0	0	0	0
hsa-miR-656-3p				0	0	0	0	0	0
hsa-miR-34c-3p				0	0	0	0	0	0
hsa-miR-5094	1.655551254	0.38453048	0.18075739	3.636212608	5.303324396	7.531037722	0.996854601	2.837757834	3.898978988
hsa-miR-154-3p				0	0	0	0	0	0
hsa-miR-487a-3p	-3.835816219		0.44917283	0	0	0	0	0.172259043	0.51677713
hsa-miR-96-3p	-1.106502398	0.92358062	0.82111037	1.818106304	3.261152305	5.857473784	2.583885652	3.490094348	3.987418403
hsa-miR-382-3p	-6.712040629		0.25810652	0	0	0	0	0.497173959	0.974744747
hsa-miR-5092				0	0	0	0	0	0
hsa-miR-4733-5p	4.907499185		0.10815391	0	1.642678499	3.347127876	0	0.172259043	0.51677713
hsa-miR-500b-5p	6.046179412		0.32986518	0	0.377668214	0.606035435	0	0	0
hsa-miR-362-5p	1.002621259	0.99275461	0.98840677	75.75442934	94.67054678	111.1905026	59.94614713	95.43473487	128.6663066
hsa-miR-501-5p	1.029189394	0.96612968	0.93165713	3.030177174	7.797929497	10.8781656	6.201325565	7.642251747	9.747447471
hsa-miR-5188	3.543660986		0.51087004	0	0.175656402	0.526969207	0	0	0
hsa-miR-487b-3p	-6.710501692		0.25817211	0	0	0	0	0.344518087	1.033554261
hsa-miR-3200-5p	18.55836984		0.09881017	0.836781969	1.411931964	1.818106304	0	0	0
hsa-miR-425-5p	-1.254995195	0.25882881	0.10158552	3292.590517	4827.663643	6551.281176	5058.214553	6059.560741	7125.384101
hsa-miR-4712-3p				0	0	0	0	0	0
hsa-miR-501-3p	1.530518488	0.34014857	0.14984613	8.484496086	12.95350744	19.49786064	5.981127605	8.648832886	11.69693697
hsa-miR-502-3p	1.310647391	0.44847878	0.23569688	24.24141739	40.71880273	56.91267431	20.15430809	31.65484468	41.91402413
hsa-miR-1246	3.289672664	0.00003346	0.00000298	21.81727565	25.44611799	31.09118319	3.987418403	7.506692364	9.747447471
hsa-miR-652-3p	1.08300145	0.83328558	0.67271084	35.15005521	40.85250189	50.58904383	33.07373635	39.00347423	48.84587544
hsa-miR-889-5p				0	0	0	0	0	0
hsa-miR-4495				0	0	0	0	0	0
hsa-miR-3668				0	0	0	0	0	0
hsa-miR-4657				0	0	0	0	0	0
hsa-miR-4491				0	0	0	0	0	0
hsa-miR-511-3p	-3.836717681		0.44908091	0	0	0	0	0.332284867	0.996854601
hsa-miR-4781-3p	-2.146783163		0.3421637	0	0.377668214	0.606035435	0.51677713	1.154373742	1.949489494
hsa-miR-4307				0	0	0	0	0	0
hsa-miR-10399-5p	3.344832903	0.001293	0.0001681	10.30260239	13.51804283	16.86301461	2.924234241	4.334293076	6.977982205
hsa-miR-3938	-12.46353505		0.12248806	0	0	0	0	0.994347918	1.949489494
hsa-miR-4778-5p				0	0	0	0	0	0
hsa-miR-363-3p	-1.608538213	0.001044	0.000125	142.4183272	160.7200714	194.9786064	239.2678114	260.9883082	289.4991899

hsa-miR-367-3p				0	0	0	0	0	0
hsa-miR-4699-3p				0	0	0	0	0	0
hsa-miR-5002-5p				0	0	0	0	0	0
hsa-miR-3613-3p	1.148466016	0.92597494	0.82594261	0.836781969	1.385576555	2.107876826	0	1.471918706	3.898978988
hsa-miR-3973				0	0	0	0	0	0
hsa-miR-520h				0	0	0	0	0	0
hsa-miR-520g-3p				0	0	0	0	0	0
hsa-miR-4709-5p				0	0	0	0	0	0
hsa-miR-4761-5p	3.543845864		0.51085051	0	0.202011812	0.606035435	0	0	0
hsa-miR-216b-3p				0	0	0	0	0	0
hsa-miR-3681-3p				0	0	0	0	0	0
hsa-miR-4717-3p	-1.518066424		0.565876	0	0.553324616	1.053938413	0	0.994347918	1.949489494
hsa-miR-6865-3p				0	0	0	0	0	0
hsa-miR-6760-3p				0	0	0	0	0	0
hsa-miR-6500-3p	-1.415938639	0.53093569	0.3031432	3.030177174	4.64672886	6.694255753	4.650994174	6.800559701	8.772702724
hsa-miR-499b-5p	-3.836717681		0.44908091	0	0	0	0	0.332284867	0.996854601
hsa-miR-6847-5p	-1.174381957		0.81254649	0	0.728981018	1.58090762	0	1.009087821	1.993709202
hsa-miR-10b-3p	3.543660986		0.51087004	0	0.175656402	0.526969207	0	0	0
hsa-miR-383-3p	-29.73171436	0.12328077	0.03200973	0	0	0	1.550331391	2.820661345	3.987418403
hsa-miR-214-3p	1.341697431	0.10144636	0.02458408	1807.803702	2110.0709	2633.792094	1249.567101	1574.127058	1899.777512
hsa-miR-5685				0	0	0	0	0	0
hsa-miR-4433a-3p				0	0	0	0	0	0
hsa-miR-6715b-5p				0	0	0	0	0	0
hsa-miR-4514				0	0	0	0	0	0
hsa-miR-3937				0	0	0	0	0	0
hsa-miR-4441				0	0	0	0	0	0
hsa-miR-3918				0	0	0	0	0	0
hsa-miR-9900				0	0	0	0	0	0
hsa-miR-125a-3p	-1.066858257	0.8421173	0.68844626	42.15753652	49.4695998	59.3914726	50.68672685	52.10156244	54.77837583
hsa-miR-936				0	0	0	0	0	0
hsa-miR-199a-3p	1.229392697	0.14285303	0.03932942	199212.6834	204327.2309	209030.2403	162275.9667	166200.4225	169703.4083
hsa-miR-622				0	0	0	0	0	0
hsa-miR-1-5p				0	0	0	0	0	0
hsa-miR-4793-5p	-1.127020076		0.92111946	0	0.175656402	0.526969207	0	0.172259043	0.51677713
hsa-miR-6785-3p				0	0	0	0	0	0
hsa-miR-4317				0	0	0	0	0	0
hsa-miR-1250-3p				0	0	0	0	0	0
hsa-miR-3691-3p	2.14020261		0.45718989	0.526969207	0.656595537	0.836781969	0	0.332284867	0.996854601
hsa-miR-181a-2-3p	-1.476596748	0.09884543	0.02298462	46.05869304	62.46552167	72.7217505	82.73893186	92.3186135	104.2976879
hsa-miR-6849-3p				0	0	0	0	0	0
hsa-miR-4738-5p				0	0	0	0	0	0
hsa-miR-665				0	0	0	0	0	0
hsa-miR-4645-5p				0	0	0	0	0	0
hsa-miR-6837-5p				0	0	0	0	0	0
hsa-miR-181a-3p	-1.082567259	0.94510719	0.88005677	1.053938413	2.400165643	3.636212608	0	2.333213924	3.898978988
hsa-miR-4726-3p				0	0	0	0	0	0
hsa-miR-4286	1.459551702	0.53977044	0.31420276	3.030177174	8.087471422	13.70119937	5.167771304	5.665789131	5.981127605
hsa-miR-6840-5p				0	0	0	0	0	0
hsa-miR-6795-3p	1.514045233		0.70086641	0	0.454583725	0.836781969	0	0.172259043	0.51677713
hsa-miR-6801-3p				0	0	0	0	0	0
hsa-miR-7108-3p				0	0	0	0	0	0
hsa-miR-1247-5p	-3.836695655		0.44908316	0	0	0	0	0.324914916	0.974744747
hsa-miR-454-5p	-1.426718499	0.08202825	0.01820189	67.87596869	77.64248498	96.4353648	107.4896431	112.7369955	118.6256975
hsa-miR-4655-3p				0	0	0	0	0	0
hsa-miR-4780				0	0	0	0	0	0
hsa-miR-6832-3p	1.581128634		0.57193519	0.526969207	0.858607348	1.212070869	0	0.657199783	0.996854601
hsa-miR-4671-5p				0	0	0	0	0	0
hsa-miR-4485-5p	3.543660986		0.51087004	0	0.175656402	0.526969207	0	0	0
hsa-miR-6827-3p	-3.836695655		0.44908316	0	0	0	0	0.324914916	0.974744747
hsa-miR-1973	15.60867919	0.03078032	0.005438	1.58090762	5.163083464	10.8781656	0	0.172259043	0.51677713
hsa-miR-132-5p	1.134162142	0.55456859	0.33274116	877.5393095	1230.349996	1542.438868	1053.191792	1085.097765	1140.451354
hsa-miR-660-3p	-3.268811027	0.23272127	0.08459503	0	0.579680025	1.212070869	0	2.16095488	3.898978988
hsa-miR-10401-3p	1.244938652	0.81713422	0.6441642	0.836781969	2.342924794	3.161815239	0.996854601	1.996065788	2.924234241
hsa-miR-3140-5p	-5.352857639		0.07147175	0	0.202011812	0.606035435	0.974744747	1.666287603	2.990563802
hsa-miR-544b				0	0	0	0	0	0
hsa-miR-4722-3p				0	0	0	0	0	0
hsa-miR-3690				0	0	0	0	0	0
hsa-miR-8073				0	0	0	0	0	0
hsa-miR-221-5p	-1.009668306	0.9749497	0.95787029	31.5138426	35.95164658	39.52269049	31.52340496	37.73368795	46.78774786
hsa-miR-6742-3p	-3.233053172	0.24540178	0.0924287	0	0.759866458	1.673563938	0.974744747	1.863013087	3.617439913
hsa-miR-4421	-1.457135635	0.56403117	0.35323996	1.673563938	2.621852117	3.161815239	2.990563802	4.152157399	5.848468483
hsa-miR-3667-3p				0	0	0	0	0	0
hsa-miR-1248				0	0	0	0	0	0
hsa-miR-6841-3p				0	0	0	0	0	0
hsa-miR-1915-5p	3.543845864		0.51085051	0	0.202011812	0.606035435	0	0	0
hsa-miR-212-5p	1.24515437	0.3300008	0.14356239	186.6589139	271.3580061	378.8908595	171.4589913	218.219001	273.9032739
hsa-miR-1249-3p	6.046179412		0.32986518	0	0.377668214	0.606035435	0	0	0
hsa-miR-5091				0	0	0	0	0	0
hsa-miR-105-3p	13.75583635		0.14751038	0	1.086974569	2.424141739	0	0	0
hsa-miR-6075				0	0	0	0	0	0
hsa-miR-7112-5p				0	0	0	0	0	0
hsa-miR-6777-5p	-1.127020076		0.92111946	0	0.175656402	0.526969207	0	0.172259043	0.51677713
hsa-miR-137-5p	3.727760571	9.093E-08	6.505E-09	28.48366543	30.45752115	31.79771483	4.650994174	9.119183818	12.95910981
hsa-miR-125b-1-3p				0	0	0	0	0	0
hsa-miR-1250-5p	1.651015809	0.03480969	0.006439	34.30806073	38.29434278	40.5766289	20.93394662	23.19703014	24.36861868
hsa-miR-1306-3p	1.3141439	0.65871246	0.45100341	2.510345907	6.124136962	8.484496086	1.949489494	4.390595908	7.234879826
hsa-miR-5707				0	0	0	0	0	0
hsa-miR-4509				0	0	0	0	0	0
hsa-miR-190b-3p				0	0	0	0	0	0
hsa-miR-616-5p	-2.14529688		0.35430516	0	0.377668214	0.606035435	0	0.689036174	2.067108522
hsa-miR-371b-5p				0	0	0	0	0	0
hsa-miR-373-5p				0	0	0	0	0	0
hsa-miR-371a-5p	-3.835816219		0.44917283	0	0	0	0	0.172259043	0.51677713
hsa-miR-4264				0	0	0	0	0	0
hsa-miR-6758-3p	-1.174589763		0.81233105	0	0.755336428	1.212070869	0.51677713	1.161743693	1.993709202
hsa-miR-5695				0	0	0	0	0	0
hsa-miR-766-3p	1.512355637	0.45614066	0.24118137	4.848283478	6.627130072	10.01241492	1.993709202	4.489305535	6.82321323
hsa-miR-3925-3p	6.047074529		0.32982105	0	0.480939135	0.836781969	0	0	0
hsa-miR-136-5p	-3.835816219		0.44917283	0	0	0	0	0.172259043	0.51677713
hsa-miR-6814-3p				0	0	0	0	0	0
hsa-miR-1307-3p	1.706998086	0.00071	0.00008292	607.2475056	754.0294155	900.0634048	356.8739471	442.0451318	551.7055269
hsa-miR-4638-5p				0	0	0	0	0	0
hsa-miR-1251-5p				0	0	0	0	0	0
hsa-miR-130b-5p	-1.516654028	0.0593457	0.01134372	55.33176669	58.67968986	61.92186571	73.89912965	91.01759121	101.6791693
hsa-miR-5187-3p	-13.23777811		0.11902967	0	0	0	0	1.314399565	1.993709202
hsa-miR-3660				0	0	0	0	0	0
hsa-miR-6751-3p				0	0	0	0	0	0
hsa-miR-3920				0	0	0	0	0	0
hsa-miR-29a-5p	2.317743465	0.00001863	1.577E-06	75.75442934	93.58476456	109.618438	36.88362023	39.28332557	43.92605609
hsa-miR-17-3p	-1.321811872	0.09253034	0.02120768	143.8625934	159.0139833	166.6597445	179.4338281	206.6677754	230.9993773
hsa-miR-20a-3p	-1.477541664	0.08204669	0.01832576	47.27076391	55.35782004	66.10577556	78.95432452	80.265619	81.74207726
hsa-miR-18a-3p	1.211085172	0.57873542	0.37089759	17.57502761	23.76372259	35.30693684	16.94652821	20.12569707	25.34336342
hsa-miR-93-3p	1.21966096	0.30928323	0.12687385	118.1769098	128.4935885	145.9704702	91.71062327	105.8674495	118.9188591

hsa-miR-6128				0	0	0	0	0	0
hsa-miR-378i	1.47380164	0.18930182	0.06054791	30.96093286	37.99537321	48.481167	19.93709201	26.15310077	32.16657665
hsa-miR-422a	1.330899224	0.61493375	0.40486879	3.636212608	4.993511634	6.323630479	1.949489494	3.689326514	4.984273004
hsa-miR-378f	1.014864501	0.9772691	0.96272307	6.323630479	7.167460772	8.484496086	4.873723736	7.346816636	10.96540061
hsa-miR-378b	1.514045233		0.70086641	0	0.454583725	0.836781969	0	0.172259043	0.51677713
hsa-miR-378d	1.410097284	0.40828805	0.20801829	10.90863782	13.5135128	17.91695302	7.974836806	9.982835622	12.67168171
hsa-miR-378c	2.361157976	0.39744107	0.19785023	1.053938413	2.149973043	4.183909846	0.51677713	0.829458826	0.996854601
hsa-miR-378a-3p	1.305352978	0.15610166	0.04580501	1412.668598	1756.663279	2321.826324	1216.162613	1346.541139	1596.631896
hsa-miR-378e	-1.517798015		0.58189746	0	0.526969207	1.58090762	0	0.841692046	1.550331391
hsa-miR-378h	-1.022063801	0.9772691	0.96794805	0	1.308661043	2.107876826	0	1.491521878	2.924234241
hsa-miR-378j				0	0	0	0	0	0
hsa-miR-6868-5p				0	0	0	0	0	0
hsa-miR-4799-3p				0	0	0	0	0	0
hsa-miR-5588-5p				0	0	0	0	0	0
hsa-miR-3176	-1.786135382	0.53093569	0.303143	0.606035435	1.534877548	3.161815239	0.996854601	3.123466407	6.82321323
hsa-miR-664a-5p	-1.194835749	0.70512731	0.50336825	9.090531521	10.2417459	11.59332254	7.974836806	12.46870542	17.54540545
hsa-miR-4646-5p				0	0	0	0	0	0
hsa-miR-6506-5p				0	0	0	0	0	0
hsa-miR-6860				0	0	0	0	0	0
hsa-miR-378g	1.058339689	0.94510719	0.88164014	1.212070869	4.113078906	5.857473784	2.924234241	3.841982386	4.984273004
hsa-miR-4667-5p	-1.127219659		0.9209974	0	0.202011812	0.606035435	0	0.324914916	0.974744747
hsa-miR-637				0	0	0	0	0	0
hsa-miR-6861-5p				0	0	0	0	0	0
hsa-miR-20b-3p	-2.81592411		0.31708658	0	0.202011812	0.606035435	0	0.51677713	1.550331391
hsa-miR-6773-3p				0	0	0	0	0	0
hsa-miR-367-5p				0	0	0	0	0	0
hsa-miR-302a-5p				0	0	0	0	0	0
hsa-miR-600				0	0	0	0	0	0
hsa-miR-412-3p				0	0	0	0	0	0
hsa-miR-635				0	0	0	0	0	0
hsa-miR-6774-5p				0	0	0	0	0	0
hsa-miR-643	1.157524452	0.78649401	0.60623188	5.020691815	8.56818196	11.59332254	6.82321323	7.688828041	8.268434087
hsa-miR-302b-5p				0	0	0	0	0	0
hsa-miR-302d-5p				0	0	0	0	0	0
hsa-miR-3124-3p				0	0	0	0	0	0
hsa-miR-5681a				0	0	0	0	0	0
hsa-miR-6730-5p				0	0	0	0	0	0
hsa-miR-4680-5p				0	0	0	0	0	0
hsa-miR-3185				0	0	0	0	0	0
hsa-miR-10522-5p				0	0	0	0	0	0
hsa-miR-4699-5p				0	0	0	0	0	0
hsa-miR-1252-5p				0	0	0	0	0	0
hsa-miR-605-3p	1.858416833	0.56165999	0.34847517	0.836781969	1.411931964	1.818106304	0	0.982114698	1.949489494
hsa-miR-4493				0	0	0	0	0	0
hsa-miR-7515				0	0	0	0	0	0
hsa-miR-3179	-1.155733934		0.87485783	0	0.454583725	0.836781969	0	0.497173959	0.974744747
hsa-miR-548ao-5p				0	0	0	0	0	0
hsa-miR-548ax				0	0	0	0	0	0
hsa-miR-4795-5p				0	0	0	0	0	0
hsa-miR-10393-5p				0	0	0	0	0	0
hsa-miR-219b-3p	1.357941266		0.66097827	0.526969207	1.214450183	2.510345907	0	0.836828777	1.993709202
hsa-miR-219a-2-3p				0	0	0	0	0	0
hsa-miR-642a-3p	-3.663705919	0.01343303	0.001981	1.212070869	2.367129487	4.215753652	4.650994174	9.746903796	14.62117121
hsa-miR-642b-3p				0	0	0	0	0	0
hsa-miR-3117-5p				0	0	0	0	0	0
hsa-miR-4645-3p	2.721573948		0.37049535	0	0.702625609	2.107876826	0	0.172259043	0.51677713
hsa-miR-3913-3p	2.767096596		0.32484423	0	0.781691837	1.818106304	0	0.172259043	0.51677713
hsa-miR-591				0	0	0	0	0	0
hsa-miR-4725-5p				0	0	0	0	0	0
hsa-miR-504-5p	-6.710501692		0.25817211	0	0	0	0	0.344518087	1.033554261
hsa-miR-631				0	0	0	0	0	0
hsa-miR-6855-3p	-1.650726203		0.55365274	0	0.454583725	0.836781969	0	0.676802954	1.033554261
hsa-miR-4513				0	0	0	0	0	0
hsa-miR-617				0	0	0	0	0	0
hsa-miR-1253				0	0	0	0	0	0
hsa-miR-6088				0	0	0	0	0	0
hsa-miR-4708-5p				0	0	0	0	0	0
hsa-miR-5186				0	0	0	0	0	0
hsa-miR-4468				0	0	0	0	0	0
hsa-miR-3670				0	0	0	0	0	0
hsa-miR-3160-3p	3.543845864		0.51085051	0	0.202011812	0.606035435	0	0	0
hsa-miR-4487				0	0	0	0	0	0
hsa-miR-27b-5p	-1.893899379	0.19063229	0.06261645	3.347127876	4.786969792	7.377568892	5.848468483	8.914244517	12.91942826
hsa-miR-4751				0	0	0	0	0	0
hsa-miR-1185-5p				0	0	0	0	0	0
hsa-miR-6884-5p	3.544185779		0.5108146	0	0.278927323	0.836781969	0	0	0
hsa-miR-485-5p				0	0	0	0	0	0
hsa-miR-3188	1.357818876		0.66107824	0.526969207	1.137534671	1.673563938	0.51677713	0.829458826	0.996854601
hsa-miR-4458	-1.438654489	0.50304972	0.2820016	2.510345907	3.80541686	5.269692065	3.987418403	5.975964144	8.772702724
hsa-let-7d-5p	1.656946787	0.001084	0.0001314	511.2737831	608.5019204	682.3958995	331.952582	367.3056366	385.9989199
hsa-miR-202-3p				0	0	0	0	0	0
hsa-miR-655-5p				0	0	0	0	0	0
hsa-miR-377-5p				0	0	0	0	0	0
hsa-miR-4785				0	0	0	0	0	0
hsa-miR-924				0	0	0	0	0	0
hsa-miR-4424				0	0	0	0	0	0
hsa-miR-219a-1-3p	-1.285301594	0.75962047	0.55026385	0.836781969	2.342924794	3.161815239	2.990563802	3.330068524	3.898978988
hsa-miR-3145-3p	-1.155569477		0.88129843	0	0.557854646	1.673563938	0	0.50454391	0.996854601
hsa-miR-383-5p	-167.9604199	2.221E-18	6.16E-20	0	0.733511048	1.673563938	109.1714117	118.5170531	130.744614
hsa-miR-369-5p	-9.588952533		0.1693546	0	0	0	0.51677713	0.829458826	0.996854601
hsa-miR-3171				0	0	0	0	0	0
hsa-miR-219b-5p	1.044328942	0.96119655	0.92050356	0.836781969	2.89624941	4.215753652	1.949489494	3.01252356	3.987418403
hsa-miR-4445-5p				0	0	0	0	0	0
hsa-miR-1468-3p				0	0	0	0	0	0
hsa-miR-4708-3p				0	0	0	0	0	0
hsa-miR-8064				0	0	0	0	0	0
hsa-miR-298				0	0	0	0	0	0
hsa-miR-4771				0	0	0	0	0	0
hsa-miR-2467-3p				0	0	0	0	0	0
hsa-miR-107	1.074392569	0.76108188	0.55422118	1491.982251	1624.963274	1781.155918	1336.385659	1514.817557	1793.530335
hsa-miR-103a-3p	1.046824526	0.8380233	0.68131512	17545.93791	19546.95214	22229.66901	17241.59717	18673.96734	20800.07816
hsa-miR-6132				0	0	0	0	0	0
hsa-miR-3665				0	0	0	0	0	0
hsa-miR-6499-3p				0	0	0	0	0	0
hsa-miR-5689	3.543660986		0.51087004	0	0.175656402	0.526969207	0	0	0
hsa-miR-6076				0	0	0	0	0	0
hsa-miR-4480	3.543845864		0.51085051	0	0.202011812	0.606035435	0	0	0
hsa-miR-4755-3p	3.543660986		0.51087004	0	0.175656402	0.526969207	0	0	0
hsa-miR-4313				0	0	0	0	0	0
hsa-miR-210-5p	3.543660986		0.51087004	0	0.175656402	0.526969207	0	0	0
hsa-miR-4707-3p				0	0	0	0	0	0
hsa-miR-3648	-3.835816219		0.44917283	0	0	0	0	0.172259043	0.51677713
hsa-miR-6743-3p				0	0	0	0	0	0

hsa-miR-6811-3p				0	0	0	0	0	0
hsa-miR-5581-5p				0	0	0	0	0	0
hsa-miR-381-5p	-6.71207816		0.25810492	0	0	0	0	0.50454391	0.996854601
hsa-miR-2277-5p	1.881331031	0.1682263	0.05108186	7.531037722	16.92314133	26.87542953	4.650994174	9.407248978	15.59591595
hsa-miR-4706				0	0	0	0	0	0
hsa-miR-4746-3p				0	0	0	0	0	0
hsa-miR-6853-5p				0	0	0	0	0	0
hsa-miR-548v				0	0	0	0	0	0
hsa-miR-222-3p	-1.205468984	0.35867223	0.1612716	110.4552199	134.0018052	161.2525772	144.5439171	163.1978241	191.0499704
hsa-miR-221-3p	-1.125065168	0.56627984	0.35714714	4291.942949	5065.18534	6456.953688	4720.242309	5700.390626	6504.471697
hsa-miR-549a-5p				0	0	0	0	0	0
hsa-miR-7106-3p				0	0	0	0	0	0
hsa-miR-423-3p	1.348250298	0.23272127	0.08446669	431.4972295	639.4945254	889.5240206	375.8141845	474.7930271	667.7001518
hsa-miR-6502-5p				0	0	0	0	0	0
hsa-miR-3194-3p				0	0	0	0	0	0
hsa-miR-4663				0	0	0	0	0	0
hsa-miR-133a-5p	-1.602885353	0.34014857	0.15080411	2.510345907	3.480459465	4.242248043	4.984273004	5.678022351	6.201325565
hsa-miR-138-5p	-1.117068891	0.93212443	0.84469266	0.526969207	2.070678218	4.848283478	0.996854601	2.187928003	3.617439913
hsa-miR-1537-5p	-1.971542787		0.52490531	0	0.202011812	0.606035435	0	0.344518087	1.033554261
hsa-miR-4718				0	0	0	0	0	0
hsa-miR-626				0	0	0	0	0	0
hsa-miR-6876-3p				0	0	0	0	0	0
hsa-miR-2115-5p				0	0	0	0	0	0
hsa-miR-103a-2-5p	2.766423252		0.303571	0	0.80589653	1.58090762	0	0.332284867	0.996854601
hsa-miR-3140-3p	-2.486496084	0.18930182	0.06079767	0	1.133004641	1.818106304	2.924234241	3.177412652	3.617439913
hsa-miR-4261				0	0	0	0	0	0
hsa-miR-5093				0	0	0	0	0	0
hsa-miR-6828-5p				0	0	0	0	0	0
hsa-miR-671-5p	2.322758454	6.973E-08	4.784E-09	727.8485571	961.9517756	1266.833973	358.6433285	414.7570419	508.816758
hsa-miR-613				0	0	0	0	0	0
hsa-miR-492				0	0	0	0	0	0
hsa-miR-659-5p	2.587535631	0.23448634	0.08648404	1.212070869	2.64605681	4.215753652	0	0.841692046	1.550331391
hsa-miR-4740-5p				0	0	0	0	0	0
hsa-miR-4515				0	0	0	0	0	0
hsa-miR-4434				0	0	0	0	0	0
hsa-miR-5703				0	0	0	0	0	0
hsa-miR-5192	3.543660986		0.51087004	0	0.175656402	0.526969207	0	0	0
hsa-miR-6081				0	0	0	0	0	0
hsa-miR-4633-3p				0	0	0	0	0	0
hsa-miR-6500-5p				0	0	0	0	0	0
hsa-miR-766-5p	-1.139456557	0.78066984	0.60060293	6.694255753	8.801079211	10.01241492	9.968546008	10.34209361	10.72219222
hsa-miR-7154-3p				0	0	0	0	0	0
hsa-miR-11181-3p				0	0	0	0	0	0
hsa-miR-6894-5p				0	0	0	0	0	0
hsa-miR-650				0	0	0	0	0	0
hsa-miR-3612				0	0	0	0	0	0
hsa-miR-6797-5p				0	0	0	0	0	0
hsa-miR-1249-5p				0	0	0	0	0	0
hsa-miR-6851-5p	8.549500881		0.23672994	0.526969207	0.656595537	0.836781969	0	0	0
hsa-miR-5191				0	0	0	0	0	0
hsa-miR-1255a	1.544931855	0.38453048	0.18064295	7.904538098	8.252284625	8.484496086	2.924234241	5.03556247	6.201325565
hsa-miR-3167				0	0	0	0	0	0
hsa-miR-31-5p	3.543660986		0.51087004	0	0.175656402	0.526969207	0	0	0
hsa-miR-593-5p				0	0	0	0	0	0
hsa-miR-564				0	0	0	0	0	0
hsa-miR-885-3p				0	0	0	0	0	0
hsa-miR-4763-3p				0	0	0	0	0	0
hsa-miR-4736				0	0	0	0	0	0
hsa-miR-34c-5p	-2.155051254	0.01899888	0.002995	5.269692065	6.739232457	9.090531521	12.40265113	15.32135325	18.94023741
hsa-miR-449b-5p	2.14020261		0.45908666	0.526969207	0.656595537	0.836781969	0	0.332284867	0.996854601
hsa-miR-3622b-5p				0	0	0	0	0	0
hsa-miR-1256	8.548235796		0.23676536	0	0.553324616	1.053938413	0	0	0
hsa-miR-4529-5p				0	0	0	0	0	0
hsa-miR-6775-3p				0	0	0	0	0	0
hsa-miR-4757-5p				0	0	0	0	0	0
hsa-miR-6735-3p	3.543845864		0.51085051	0	0.202011812	0.606035435	0	0	0
hsa-miR-6717-5p				0	0	0	0	0	0
hsa-miR-6722-5p				0	0	0	0	0	0
hsa-miR-25-5p	1.077443469	0.83195049	0.66555806	42.67588042	50.75354959	65.34418161	42.86474783	47.41538132	50.64415878
hsa-miR-663a				0	0	0	0	0	0
hsa-miR-6822-3p				0	0	0	0	0	0
hsa-miR-604				0	0	0	0	0	0
hsa-miR-4430				0	0	0	0	0	0
hsa-miR-4505				0	0	0	0	0	0
hsa-miR-7974	1.157869241	0.71525803	0.51164443	16.96899217	21.69126123	26.34846033	11.36909687	19.5993435	28.90878342
hsa-miR-4668-5p	3.543845864		0.51085051	0	0.202011812	0.606035435	0	0	0
hsa-miR-4747-5p	11.05059118		0.18081904	0	0.755336428	1.212070869	0	0	0
hsa-miR-5196-5p				0	0	0	0	0	0
hsa-miR-3649				0	0	0	0	0	0
hsa-miR-92b-5p	1.110124182	0.83107589	0.66198177	14.54485043	19.00310821	27.40239874	7.974836806	17.15473644	24.36861868
hsa-miR-3199	6.045864117		0.32988072	0	0.351312804	1.053938413	0	0	0
hsa-miR-365b-5p	2.201682567	0.36825655	0.16734801	1.212070869	2.501285847	4.183909846	0	1.147003791	2.924234241
hsa-miR-365a-5p	1.432758278	0.67661118	0.47438233	1.212070869	3.173026016	5.796661272	0.996854601	2.320980704	3.898978988
hsa-miR-6878-5p				0	0	0	0	0	0
hsa-miR-149-3p				0	0	0	0	0	0
hsa-miR-6883-5p				0	0	0	0	0	0
hsa-miR-638				0	0	0	0	0	0
hsa-miR-3945				0	0	0	0	0	0
hsa-miR-4253				0	0	0	0	0	0
hsa-miR-4726-5p	4.020003532		0.14412191	0	1.363751176	2.510345907	0	0.332284867	0.996854601
hsa-miR-296-5p	4.64775041		0.10334357	0.526969207	1.541558295	2.424141739	0	0.172259043	0.51677713
hsa-miR-6877-5p	-6.710501692		0.25817211	0	0	0	0	0.344518087	1.033554261
hsa-miR-5001-5p				0	0	0	0	0	0
hsa-miR-27a-5p	-1.183325826	0.56627984	0.35612741	46.66472847	80.70611153	103.7609642	89.71691407	94.89944879	103.3554261
hsa-miR-3144-5p	-2.166383722		0.31254696	0	0.579680025	1.212070869	0	1.63194453	3.898978988
hsa-miR-4652-5p				0	0	0	0	0	0
hsa-miR-185-3p	2.15647491	0.09899549	0.02370111	5.020691815	6.934563521	7.904538098	2.924234241	3.337438476	3.987418403
hsa-miR-625-5p	1.215150649	0.43679937	0.22700814	54.39082799	64.50440006	72.72425216	43.86160243	52.60595625	58.39581574
hsa-miR-5010-5p				0	0	0	0	0	0
hsa-miR-4697-5p				0	0	0	0	0	0
hsa-miR-4488				0	0	0	0	0	0
hsa-miR-6885-5p				0	0	0	0	0	0
hsa-miR-6835-5p				0	0	0	0	0	0
hsa-miR-342-5p	-1.114762977	0.83195049	0.66556039	11.59332254	14.18562055	17.57502761	9.968546008	14.76201923	20.67108522
hsa-miR-608				0	0	0	0	0	0
hsa-miR-6747-5p				0	0	0	0	0	0
hsa-miR-555				0	0	0	0	0	0
hsa-miR-6756-5p				0	0	0	0	0	0
hsa-miR-4455	2.861474213	0.24706924	0.09341742	0.836781969	2.114557573	3.688784446	0	0.676802954	1.033554261
hsa-miR-609				0	0	0	0	0	0
hsa-miR-4650-3p				0	0	0	0	0	0
hsa-miR-3529-5p	-3.836695655		0.44908316	0	0	0	0	0.324914916	0.974744747
hsa-miR-5681b				0	0	0	0	0	0

hsa-miR-6503-5p				0	0	0	0	0	0
hsa-miR-3085-5p				0	0	0	0	0	0
hsa-miR-3907				0	0	0	0	0	0
hsa-miR-3165	11.05116695		0.18108552	0	0.781691837	1.818106304	0	0	0
hsa-miR-6769a-5p	-6.71207816		0.25810492	0	0	0	0	0.50454391	0.996854601
hsa-miR-323a-5p				0	0	0	0	0	0
hsa-miR-3650				0	0	0	0	0	0
hsa-miR-4694-5p				0	0	0	0	0	0
hsa-miR-409-5p	-3.836717681		0.44908091	0	0	0	0	0.332284867	0.996854601
hsa-miR-152-5p	6.047074529		0.32982105	0	0.480939135	0.836781969	0	0	0
hsa-miR-563				0	0	0	0	0	0
hsa-miR-656-5p				0	0	0	0	0	0
hsa-miR-92a-1-5p	-1.370047768	0.64133774	0.43161562	1.673563938	3.833922986	6.666389782	0.996854601	5.32362763	8.772702724
hsa-miR-323b-5p				0	0	0	0	0	0
hsa-miR-494-5p				0	0	0	0	0	0
hsa-miR-410-5p				0	0	0	0	0	0
hsa-miR-6832-5p	3.543660986		0.51087004	0	0.175656402	0.526969207	0	0	0
hsa-miR-567				0	0	0	0	0	0
hsa-miR-630				0	0	0	0	0	0
hsa-miR-616-3p	1.187086458	0.86978632	0.72325725	1.58090762	2.171798422	2.510345907	0.996854601	1.823806744	2.924234241
hsa-miR-1257	2.766318217		0.30359255	0	0.755336428	1.212070869	0	0.324914916	0.974744747
hsa-miR-550a-3-5p	6.046759143		0.32983659	0	0.454583725	0.836781969	0	0	0
hsa-miR-550a-5p	1.237494259	0.86978632	0.73257931	0.606035435	1.183564743	2.107876826	0	0.849061997	1.550331391
hsa-miR-1271-3p	-6.195772183		0.04721837	0	0.202011812	0.606035435	0.974744747	1.678520823	2.067108522
hsa-miR-4800-5p				0	0	0	0	0	0
hsa-miR-3691-5p	-1.03491309	0.96612968	0.9351007	1.673563938	2.999520331	3.688784446	1.949489494	3.184782603	3.987418403
hsa-miR-3688-5p				0	0	0	0	0	0
hsa-miR-891a-3p				0	0	0	0	0	0
hsa-miR-6778-5p				0	0	0	0	0	0
hsa-miR-1233-5p				0	0	0	0	0	0
hsa-miR-491-5p	1.344434001	0.42662204	0.21985048	13.33277956	17.29710429	22.65967588	10.96540061	13.16024827	15.59591595
hsa-miR-6742-5p				0	0	0	0	0	0
hsa-miR-203a-5p				0	0	0	0	0	0
hsa-miR-644a				0	0	0	0	0	0
hsa-miR-569				0	0	0	0	0	0
hsa-miR-1258				0	0	0	0	0	0
hsa-miR-22-5p	1.197903902	0.80405547	0.63150634	3.636212608	5.127667994	7.531037722	2.583885652	4.457469144	7.797957977
hsa-miR-6501-5p	1.237501357	0.86978632	0.73257283	0.606035435	1.183564743	2.107876826	0	0.841692046	1.550331391
hsa-miR-4423-5p	-4.128329166	0.17176543	0.05281793	0	0.351312804	1.053938413	1.550331391	2.163461563	2.990563802
hsa-miR-6740-5p	3.543845864		0.51085051	0	0.202011812	0.606035435	0	0	0
hsa-miR-19b-1-5p	-1.83808758	0.01825809	0.002825	9.485445718	12.90569423	15.89885741	20.46963969	23.04201768	25.91821962
hsa-miR-19b-2-5p	-3.836695655		0.44908316	0	0	0	0	0.324914916	0.974744747
hsa-miR-19a-5p	2.140500396		0.45652905	0	0.579680025	1.212070869	0	0.172259043	0.51677713
hsa-miR-9-3p	-1.219907242	0.25882881	0.10202012	267.7003569	277.8605609	293.9271858	304.0406532	335.1316582	360.1936599
hsa-miR-3143	13.38999402		0.15667779	0	0.983703648	2.424141739	0	0	0
hsa-miR-208b-3p				0	0	0	0	0	0
hsa-miR-208a-3p				0	0	0	0	0	0
hsa-miR-374c-5p	3.543845864		0.51085051	0	0.202011812	0.606035435	0	0	0
hsa-miR-655-3p				0	0	0	0	0	0
hsa-miR-466				0	0	0	0	0	0
hsa-miR-4666a-5p				0	0	0	0	0	0
hsa-miR-5591-3p				0	0	0	0	0	0
hsa-miR-4777-3p				0	0	0	0	0	0
hsa-miR-4693-5p				0	0	0	0	0	0
hsa-miR-4766-3p	1.185085494	0.78884656	0.61265163	4.742722859	5.986275343	8.367819691	3.617439913	5.134272098	7.797957977
hsa-miR-4524b-5p				0	0	0	0	0	0
hsa-miR-4524a-5p				0	0	0	0	0	0
hsa-miR-3117-3p				0	0	0	0	0	0
hsa-miR-4423-3p	-1.650970574		0.55435136	0	0.351312804	1.053938413	0	0.676802954	1.033554261
hsa-miR-4727-3p				0	0	0	0	0	0
hsa-miR-4460	3.543660986		0.51087004	0	0.175656402	0.526969207	0	0	0
hsa-miR-374b-5p	1.191836522	0.39076896	0.1884557	390.8928554	447.1839024	522.2264837	337.4554662	377.1818988	413.2917728
hsa-miR-1185-2-3p				0	0	0	0	0	0
hsa-miR-1185-1-3p	-18.21087286		0.06992402	0	0	0	0.974744747	1.346235957	2.067108522
hsa-miR-3144-3p	-1.271592544	0.39565644	0.19502432	25.94024104	27.17001343	29.51027557	31.00662783	35.97370408	39.87418403
hsa-miR-4633-5p				0	0	0	0	0	0
hsa-miR-3115	-1.117865728	0.81263576	0.63943164	8.484496086	9.864077684	11.06635334	8.772702724	10.553559	12.91942826
hsa-miR-3924				0	0	0	0	0	0
hsa-miR-556-3p	1.608811314	0.68263391	0.48232819	1.053938413	1.313191074	1.673563938	0	0.669433003	1.033554261
hsa-miR-4795-3p				0	0	0	0	0	0
hsa-miR-3671				0	0	0	0	0	0
hsa-miR-421	1.128073095	0.53977044	0.31790056	333.0392237	365.2538782	393.3169971	309.0249262	324.4142041	332.804472
hsa-miR-561-5p	-1.52249024	0.3330132	0.14535905	5.796661272	12.83070084	21.81727565	14.62117121	19.41887063	21.93080122
hsa-miR-377-3p	-1.972560749		0.52403358	0	0.175656402	0.526969207	0	0.657199783	0.996854601
hsa-miR-23b-3p	-1.867438217	0.00001507	1.254E-06	299.5679449	321.8367157	339.8951382	552.4347524	607.8913782	648.2052568
hsa-miR-23a-3p	1.12178896	0.53977044	0.31534488	4757.105494	5227.293633	5785.594919	4170.90822	4663.647896	5053.076769
hsa-miR-23c	-1.415403905	0.47892666	0.26358446	3.347127876	4.260000586	5.796661272	5.848468483	6.182566261	6.718102696
hsa-miR-3127-5p	1.312452306	0.67689605	0.47629766	2.424141739	5.002571694	8.367819691	1.949489494	3.357041647	4.134217044
hsa-miR-539-3p	-2.816585182		0.30244876	0	0.202011812	0.606035435	0	0.676802954	1.033554261
hsa-miR-376a-3p	-1.987204058	0.47081866	0.25568546	0.606035435	0.832251939	1.053938413	1.550331391	1.831176696	1.993709202
hsa-miR-376b-3p	-3.836717681		0.44908091	0	0	0	0	0.332284867	0.996854601
hsa-miR-5007-3p				0	0	0	0	0	0
hsa-miR-433-3p				0	0	0	0	0	0
hsa-miR-6516-3p	21.06168926		0.08424303	1.212070869	1.664503878	2.107876826	0	0	0
hsa-miR-5195-3p				0	0	0	0	0	0
hsa-miR-1260b	1.155148701	0.67661118	0.47372495	31.5138426	43.55602815	54.80479748	26.91507422	36.99403116	47.02671887
hsa-miR-1260a	-1.107512962	0.77631463	0.59311361	45.4526576	73.50456123	92.21961114	65.79240365	81.42775627	99.4239642
hsa-miR-2355-5p				0	0	0	0	0	0
hsa-miR-623				0	0	0	0	0	0
hsa-miR-937-3p	1.84395212	0.24163709	0.08995249	3.636212608	6.388148249	9.20460166	2.583885652	3.490094348	3.987418403
hsa-miR-7975				0	0	0	0	0	0
hsa-miR-7111-3p	3.543845864		0.51085051	0	0.202011812	0.606035435	0	0	0
hsa-miR-6881-3p	-3.836717681		0.44908091	0	0	0	0	0.332284867	0.996854601
hsa-miR-502-5p	-1.025714756	0.96770494	0.94150309	3.347127876	5.674083267	7.878460651	4.134217044	6.635670609	7.974836806
hsa-miR-639				0	0	0	0	0	0
hsa-miR-4790-5p				0	0	0	0	0	0
hsa-miR-425-3p	-1.437899305	0.02386105	0.003997	291.2001252	320.3737664	336.3496663	411.7009501	461.8765447	500.0440553
hsa-miR-3181				0	0	0	0	0	0
hsa-miR-517a-3p	-1.127020076		0.92111946	0	0.175656402	0.526969207	0	0.172259043	0.51677713
hsa-miR-517c-3p				0	0	0	0	0	0
hsa-miR-4799-5p	-2.258229188	0.38005615	0.1762404	0	0.728981018	1.58090762	1.949489494	2.003435739	2.067108522
hsa-miR-4256				0	0	0	0	0	0
hsa-miR-4727-5p	6.046179412		0.32986518	0	0.377668214	0.606035435	0	0	0
hsa-miR-6828-3p				0	0	0	0	0	0
hsa-miR-516b-5p	3.543660986		0.51087004	0	0.175656402	0.526969207	0	0	0
hsa-miR-3686				0	0	0	0	0	0
hsa-miR-6888-3p	-3.836717681		0.44908091	0	0	0	0	0.332284867	0.996854601
hsa-miR-7843-3p				0	0	0	0	0	0
hsa-miR-3616-5p	-3.835816219		0.44917283	0	0	0	0	0.172259043	0.51677713
hsa-miR-215-5p	-1.336927678	0.46005234	0.24446577	9.20460166	10.39319761	11.06635334	13.64642646	14.02405017	14.46975965
hsa-miR-3672				0	0	0	0	0	0
hsa-miR-5708				0	0	0	0	0	0
hsa-miR-640				0	0	0	0	0	0

hsa-miR-500a-3p	-1.070747861	0.83107589	0.66243422	47.87679934	54.02138264	64.8172124	56.84548435	59.34785654	62.38366381
hsa-miR-6811-5p				0	0	0	0	0	0
hsa-miR-6836-3p				0	0	0	0	0	0
hsa-miR-4737				0	0	0	0	0	0
hsa-miR-4754				0	0	0	0	0	0
hsa-miR-5089-3p	-3.836695655		0.44908316	0	0	0	0	0.324914916	0.974744747
hsa-miR-628-5p	-1.35665214	0.31869443	0.13463661	14.22529347	22.20226103	30.56421398	28.9395193	31.66638413	33.89305643
hsa-miR-4531				0	0	0	0	0	0
hsa-miR-620				0	0	0	0	0	0
hsa-miR-6874-5p				0	0	0	0	0	0
hsa-miR-1261	1.634129912	0.61592748	0.40663468	0	2.799887833	4.215753652	0.974744747	1.518495	2.583885652
hsa-miR-4642				0	0	0	0	0	0
hsa-miR-548s	8.550766568		0.23669451	0	0.759866458	1.673563938	0	0	0
hsa-miR-4435	1.901177255	0.55611842	0.33529475	0	1.642678499	3.347127876	0	0.676802954	1.033554261
hsa-miR-6810-5p				0	0	0	0	0	0
hsa-miR-6812-5p				0	0	0	0	0	0
hsa-miR-1262				0	0	0	0	0	0
hsa-miR-4701-3p				0	0	0	0	0	0
hsa-miR-1263				0	0	0	0	0	0
hsa-miR-5587-5p	-3.836695655		0.44908316	0	0	0	0	0.324914916	0.974744747
hsa-miR-6843-3p				0	0	0	0	0	0
hsa-miR-218-1-3p				0	0	0	0	0	0
hsa-miR-135a-2-3p				0	0	0	0	0	0
hsa-miR-135b-3p				0	0	0	0	0	0
hsa-miR-568				0	0	0	0	0	0
hsa-miR-297				0	0	0	0	0	0
hsa-miR-10395-3p	-1.986566449	0.50304972	0.28174384	0	0.728981018	1.58090762	0	1.511125049	2.583885652
hsa-miR-3912-5p	-1.126853197		0.92121401	0	0.278927323	0.836781969	0	0.172259043	0.51677713
hsa-miR-4433b-5p				0	0	0	0	0	0
hsa-miR-8070				0	0	0	0	0	0
hsa-miR-550b-2-5p				0	0	0	0	0	0
hsa-miR-4296				0	0	0	0	0	0
hsa-miR-4477b	-2.011972624	0.18093068	0.05705259	1.673563938	2.999520331	3.688784446	3.617439913	7.127981299	9.968546008
hsa-miR-6509-5p	-3.836695655		0.44908316	0	0	0	0	0.324914916	0.974744747
hsa-miR-384				0	0	0	0	0	0
hsa-miR-4263				0	0	0	0	0	0
hsa-miR-576-5p	1.293369583	0.24163709	0.08973483	104.2380948	126.6896631	167.0492385	88.88566644	98.87100815	115.0198802
hsa-miR-4768-5p				0	0	0	0	0	0
hsa-miR-544a				0	0	0	0	0	0
hsa-miR-6875-3p				0	0	0	0	0	0
hsa-miR-510-3p				0	0	0	0	0	0
hsa-miR-514b-3p				0	0	0	0	0	0
hsa-miR-514a-3p	2.766439134		0.30356933	0	0.80589653	1.58090762	0	0.324914916	0.974744747
hsa-miR-4774-3p				0	0	0	0	0	0
hsa-miR-3152-5p				0	0	0	0	0	0
hsa-miR-4529-3p				0	0	0	0	0	0
hsa-miR-450a-1-3p	1.136639945	0.77631463	0.59309515	14.22529347	17.17846014	19.39313391	8.971691407	15.15380738	20.46963969
hsa-miR-450a-2-3p	1.916754187	0.31869443	0.13581525	3.688784446	4.442337735	5.454318912	1.033554261	2.648402302	3.987418403
hsa-miR-3674				0	0	0	0	0	0
hsa-miR-4646-3p				0	0	0	0	0	0
hsa-miR-2355-3p				0	0	0	0	0	0
hsa-miR-6739-3p	-1.344137402	0.76519354	0.5596525	0.606035435	1.669033908	3.347127876	1.949489494	2.003435739	2.067108522
hsa-miR-4446-5p				0	0	0	0	0	0
hsa-miR-2113				0	0	0	0	0	0
hsa-miR-548d-3p	-1.468078568	0.61606818	0.41076906	1.212070869	1.664503878	2.107876826	0.974744747	2.03527213	4.134217044
hsa-miR-548bb-3p	3.543660986		0.51087004	0	0.175656402	0.526969207	0	0	0
hsa-miR-548h-3p	-1.149497016	0.83425622	0.67593022	4.215753652	6.291558075	9.20460166	4.873723736	6.374428476	8.268434087
hsa-miR-548ac	2.012660726		0.51798923	0	0.526969207	1.58090762	0	0.332284867	0.996854601
hsa-miR-548ae-3p	3.463770543	0.0636499	0.01272998	3.161815239	5.632583225	7.878460651	0.51677713	1.818943476	2.990563802
hsa-miR-548aq-3p	-1.13519356	0.7217083	0.51836567	19.99916935	22.96963301	26.77702301	17.54540545	24.34334016	32.55695922
hsa-miR-548ah-3p	-1.521604556	0.50217329	0.27931098	1.673563938	2.876574747	4.848283478	3.898978988	4.511415389	4.984273004
hsa-miR-548am-3p	-1.167940449		0.84048618	0	0.553324616	1.053938413	0.51677713	0.829458826	0.996854601
hsa-miR-548j-3p	1.107314616		0.88906942	0	0.90916745	1.673563938	0	0.676802954	1.033554261
hsa-miR-548c-3p	-1.553917917	0.61379757	0.40322468	1.053938413	1.313191074	1.673563938	0.996854601	1.996065788	2.924234241
hsa-miR-548at-3p	-3.835816219		0.44917283	0	0	0	0	0.172259043	0.51677713
hsa-miR-548ay-3p	1.525570721	0.37777265	0.17316878	5.857473784	8.873464692	11.06635334	2.990563802	5.682885619	7.234879826
hsa-miR-424-3p	1.264225566	0.58645201	0.37920356	11.71494757	12.38933963	13.33277956	5.981127605	8.899504614	12.91942826
hsa-miR-548a-3p	1.024622651	0.95639406	0.91061613	26.77702301	35.41516012	43.6345513	18.52015019	34.26682259	43.40927896
hsa-miR-548e-5p	1.068676442	0.93212443	0.8463962	4.215753652	6.791943275	10.30260239	5.684548435	6.487878005	7.797957977
hsa-miR-548n	1.44420469	0.55068321	0.32799818	4.742722859	8.179670547	13.938815	1.949489494	5.547476332	7.974836806
hsa-miR-548t-5p	1.144752756	0.9019592	0.78477034	1.053938413	2.323250131	4.242248043	1.550331391	2.156091611	2.924234241
hsa-miR-548az-5p	3.393837036		0.20671826	0.836781969	1.034263751	1.212070869	0	0.172259043	0.51677713
hsa-miR-497-3p	-1.07049503	0.95109507	0.8920203	0.526969207	2.224509241	3.636212608	0.996854601	2.015668959	3.100662783
hsa-miR-6771-3p				0	0	0	0	0	0
hsa-miR-186-5p	1.335255958	0.0636499	0.01261709	2775.019842	3292.464355	4077.406405	2278.953219	2465.467623	2571.483001
hsa-miR-548u	-1.121523057	0.89266359	0.77276619	2.510345907	3.427748647	4.742722859	1.993709202	3.534163959	5.684548435
hsa-miR-583				0	0	0	0	0	0
hsa-miR-518b				0	0	0	0	0	0
hsa-miR-518d-3p				0	0	0	0	0	0
hsa-miR-518c-3p				0	0	0	0	0	0
hsa-miR-6768-3p				0	0	0	0	0	0
hsa-miR-548m	-3.835816219		0.44917283	0	0	0	0	0.172259043	0.51677713
hsa-miR-6507-3p	-29.71778926	0.12328077	0.032035	0	0	0	1.993709202	2.500609698	2.924234241
hsa-miR-3609				0	0	0	0	0	0
hsa-miR-519d-3p				0	0	0	0	0	0
hsa-miR-20b-5p	-1.857612515	3.631E-06	2.862E-07	500.3956175	564.4830293	626.0346041	1021.775966	1051.576138	1074.379654
hsa-miR-93-5p	1.132170508	0.46457826	0.2516183	20491.11686	22302.16136	24468.68068	18068.98649	19698.37153	20621.69987
hsa-miR-17-5p	-1.325208104	0.07289177	0.015004	2235.930343	2535.28886	3038.66167	2925.208986	3358.948194	3725.446333
hsa-miR-561-3p	-1.835149377	0.20861866	0.07126535	2.107876826	4.473223174	5.857473784	6.82321323	7.688828041	8.268434087
hsa-miR-5692a				0	0	0	0	0	0
hsa-miR-1252-3p				0	0	0	0	0	0
hsa-miR-4694-3p				0	0	0	0	0	0
hsa-miR-10a-3p	-1.770076773	0.00001503	1.229E-06	895.3567069	1015.177079	1139.307425	1628.860418	1799.155984	1905.625981
hsa-miR-7-1-3p	1.089780006	0.77631463	0.59057864	55.75525999	61.36902985	70.61387368	54.82700304	56.89131645	58.48468483
hsa-miR-7-2-3p	-3.835816219		0.44917283	0	0	0	0	0.172259043	0.51677713
hsa-miR-196a-1-3p	-1.657035575	0.12255343	0.03130927	10.04138363	12.15644238	13.70119937	14.95281901	20.03671398	22.73819374
hsa-miR-21-3p	-1.8384478	0.0001031	0.00001039	317.7624315	371.5150205	454.3726092	581.922614	680.2675252	855.782928
hsa-miR-6776-3p	-1.651650742		0.55964145	0	0.377668214	0.606035435	0	0.98948465	1.993709202
hsa-miR-652-5p	1.116778778	0.83857163	0.68432342	10.01241492	14.01449418	16.96899217	6.977982205	11.5650034	16.02009104
hsa-miR-3150a-5p	3.012791357	0.31869443	0.13678156	0.836781969	1.587588366	2.107876826	0	0.657199783	0.996854601
hsa-miR-3150b-5p				0	0	0	0	0	0
hsa-miR-490-3p				0	0	0	0	0	0
hsa-miR-191-5p	-1.206573156	0.26297932	0.1044239	14853.71673	16169.93872	17599.19059	17692.89861	19512.70339	22936.71864
hsa-miR-708-3p				0	0	0	0	0	0
hsa-miR-548b-3p	1.355812888	0.24485471	0.0918652	45.84632097	54.94144266	72.11821673	36.06555564	40.43534272	42.86474783
hsa-miR-7109-3p				0	0	0	0	0	0
hsa-miR-99a-3p	-8.039294265	1.794E-14	7.334E-16	3.636212608	4.187615104	4.742722859	29.90563802	33.74321919	37.20795339
hsa-miR-99b-3p	-1.032332818	0.90482321	0.7951877	283.6690875	308.5274148	347.8643395	282.109852	317.5292244	340.0393518
hsa-miR-100-3p	3.544185779		0.5108146	0	0.278927323	0.836781969	0	0	0
hsa-miR-4428				0	0	0	0	0	0
hsa-miR-12118				0	0	0	0	0	0
hsa-miR-4753-5p	1.513474153		0.70111189	0	0.351312804	1.053938413	0	0.324914916	0.974744747
hsa-miR-4475				0	0	0	0	0	0

hsa-miR-1264				0	0	0	0	0	0
hsa-miR-34b-3p	-2.066326359		0.38236999	0	0.480939135	0.836781969	0	0.689036174	2.067108522
hsa-miR-34a-3p	1.631779609	0.56759447	0.3596146	0.836781969	1.99161199	3.030177174	0.996854601	1.326632785	1.949489494
hsa-miR-5197-5p				0	0	0	0	0	0
hsa-miR-8066				0	0	0	0	0	0
hsa-miR-33a-3p	-1.437280361	0.53203037	0.30679124	1.818106304	4.345976157	7.531037722	3.987418403	5.823308272	7.797957977
hsa-miR-5571-5p				0	0	0	0	0	0
hsa-miR-32-3p	-1.427166374	0.21645565	0.07615447	14.75513778	20.93391307	25.45348826	20.46963969	29.60108852	38.87732943
hsa-miR-624-3p	-1.439200165	0.70047356	0.4990235	1.053938413	2.073057531	3.347127876	0	2.505472967	3.898978988
hsa-miR-4731-3p				0	0	0	0	0	0
hsa-miR-8485				0	0	0	0	0	0
hsa-miR-603				0	0	0	0	0	0
hsa-miR-6768-5p	3.543660986		0.51087004	0	0.175656402	0.526969207	0	0	0
hsa-miR-4789-3p				0	0	0	0	0	0
hsa-miR-3120-3p	83.61066753	0.0593457	0.01134932	4.848283478	6.833671914	8.958476511	0	0	0
hsa-miR-4700-3p				0	0	0	0	0	0
hsa-miR-4438				0	0	0	0	0	0
hsa-miR-5580-3p				0	0	0	0	0	0
hsa-miR-942-3p	-1.971994552		0.5242206	0	0.202011812	0.606035435	0	0.497173959	0.974744747
hsa-miR-323a-3p	-8.09241619	0.01630337	0.002475	0	0.377668214	0.606035435	3.100662783	4.312183223	5.848468483
hsa-miR-1911-3p				0	0	0	0	0	0
hsa-miR-6753-5p				0	0	0	0	0	0
hsa-miR-7153-3p				0	0	0	0	0	0
hsa-miR-1295b-5p				0	0	0	0	0	0
hsa-miR-4640-3p	-15.34117865		0.08996754	0	0	0	0.974744747	1.334002737	1.993709202
hsa-miR-941	-1.146518676	0.55616661	0.33613573	99.38981129	101.3396835	102.9241822	95.69804167	113.2648618	127.1271741
hsa-miR-99b-5p	1.045640097	0.83425622	0.67500645	3085.21512	3198.984986	3287.742233	2895.862615	3062.641612	3292.687756
hsa-miR-6510-3p				0	0	0	0	0	0
hsa-miR-4655-5p				0	0	0	0	0	0
hsa-miR-6800-3p	-1.127020076		0.92111946	0	0.175656402	0.526969207	0	0.172259043	0.51677713
hsa-miR-3200-3p	-1.026376382	0.93212443	0.84612561	93.27354956	115.881882	129.691583	113.0703907	117.2572936	125.0600656
hsa-miR-6782-3p	8.486417713		0.23996954	0	0.606035435	1.818106304	0	0	0
hsa-miR-574-3p	3.094281333	5.992E-21	1.312E-22	357.285122	380.5755739	401.1954578	115.7580772	124.3395424	128.6663066
hsa-miR-4445-3p				0	0	0	0	0	0
hsa-miR-28-3p	1.178723552	0.38150312	0.17795955	743.0623886	843.7524919	902.1712816	661.9114549	717.5036817	775.8968187
hsa-miR-512-5p				0	0	0	0	0	0
hsa-miR-4418				0	0	0	0	0	0
hsa-miR-6807-3p	16.05749237		0.11790755	0.606035435	1.286835664	1.673563938	0	0	0
hsa-miR-892b				0	0	0	0	0	0
hsa-miR-1183				0	0	0	0	0	0
hsa-miR-4318				0	0	0	0	0	0
hsa-miR-892a	6.046759143		0.32983659	0	0.454583725	0.836781969	0	0	0
hsa-miR-4676-3p	-2.066326359		0.38249232	0	0.480939135	0.836781969	0	0.689036174	2.067108522
hsa-miR-892c-3p				0	0	0	0	0	0
hsa-miR-374c-3p				0	0	0	0	0	0
hsa-miR-4773				0	0	0	0	0	0
hsa-miR-3154	-3.835816219		0.44917283	0	0	0	0	0.172259043	0.51677713
hsa-miR-3123				0	0	0	0	0	0
hsa-miR-6873-5p				0	0	0	0	0	0
hsa-miR-5586-3p				0	0	0	0	0	0
hsa-miR-5694				0	0	0	0	0	0
hsa-miR-6871-3p	-3.836717681		0.44908091	0	0	0	0	0.332284867	0.996854601
hsa-miR-424-5p	1.300588253	0.20878245	0.07177184	288.7791252	391.0350359	527.8568636	243.6861868	299.9696545	359.1601057
hsa-miR-497-5p	-1.343368383	0.1319728	0.03525697	98.01627242	129.4380872	147.2736266	133.5400304	172.5646611	192.7578697
hsa-miR-6165				0	0	0	0	0	0
hsa-miR-6510-5p				0	0	0	0	0	0
hsa-miR-4274				0	0	0	0	0	0
hsa-miR-4519				0	0	0	0	0	0
hsa-miR-449b-3p	3.543845864		0.51085051	0	0.202011812	0.606035435	0	0	0
hsa-miR-6858-3p				0	0	0	0	0	0
hsa-miR-4323				0	0	0	0	0	0
hsa-miR-3940-3p	3.745533578	0.0938745	0.02165281	2.510345907	3.858127679	4.848283478	0	0.689036174	2.067108522
hsa-miR-4687-5p				0	0	0	0	0	0
hsa-miR-6746-3p				0	0	0	0	0	0
hsa-miR-6877-3p				0	0	0	0	0	0
hsa-miR-4293				0	0	0	0	0	0
hsa-miR-7846-3p				0	0	0	0	0	0
hsa-miR-6876-5p				0	0	0	0	0	0
hsa-miR-4476				0	0	0	0	0	0
hsa-miR-8083				0	0	0	0	0	0
hsa-miR-4695-5p				0	0	0	0	0	0
hsa-miR-4433b-3p				0	0	0	0	0	0
hsa-miR-4661-3p				0	0	0	0	0	0
hsa-miR-10524-5p				0	0	0	0	0	0
hsa-miR-1265				0	0	0	0	0	0
hsa-miR-3622a-5p				0	0	0	0	0	0
hsa-miR-6511a-5p	-1.127020076		0.92111946	0	0.175656402	0.526969207	0	0.172259043	0.51677713
hsa-miR-6808-5p				0	0	0	0	0	0
hsa-miR-6893-5p	3.544185779		0.5108146	0	0.278927323	0.836781969	0	0	0
hsa-miR-2682-5p	4.619032976	6.254E-10	3.613E-11	33.72602922	35.43350884	36.81840664	3.898978988	6.898575557	9.818765478
hsa-miR-15a-3p	1.646988174	0.49258886	0.27254187	3.030177174	3.458634086	4.183909846	0.974744747	1.850779867	2.583885652
hsa-miR-3186-5p				0	0	0	0	0	0
hsa-miR-5584-5p				0	0	0	0	0	0
hsa-miR-6750-5p	-3.836717681		0.44908091	0	0	0	0	0.332284867	0.996854601
hsa-miR-6822-5p				0	0	0	0	0	0
hsa-miR-6760-5p				0	0	0	0	0	0
hsa-miR-4756-5p				0	0	0	0	0	0
hsa-miR-1321				0	0	0	0	0	0
hsa-miR-6735-5p				0	0	0	0	0	0
hsa-miR-4436b-3p				0	0	0	0	0	0
hsa-miR-6879-5p				0	0	0	0	0	0
hsa-miR-6895-5p	3.543660986		0.51087004	0	0.175656402	0.526969207	0	0	0
hsa-miR-7160-3p				0	0	0	0	0	0
hsa-miR-4512	-3.835816219		0.44917283	0	0	0	0	0.172259043	0.51677713
hsa-miR-5088-5p	-9.58450593		0.1798856	0	0	0	0	0.51677713	1.550331391
hsa-miR-4446-3p				0	0	0	0	0	0
hsa-miR-6813-5p				0	0	0	0	0	0
hsa-miR-6794-5p				0	0	0	0	0	0
hsa-miR-1178-5p				0	0	0	0	0	0
hsa-miR-3927-3p				0	0	0	0	0	0
hsa-miR-370-5p	-3.835816219		0.44917283	0	0	0	0	0.172259043	0.51677713
hsa-miR-431-3p				0	0	0	0	0	0
hsa-miR-12114				0	0	0	0	0	0
hsa-miR-802				0	0	0	0	0	0
hsa-miR-8079				0	0	0	0	0	0
hsa-miR-8062				0	0	0	0	0	0
hsa-miR-3666				0	0	0	0	0	0
hsa-miR-301a-3p	-1.41190726	0.0764259	0.01626552	507.9983151	697.8175214	859.9642818	764.1998817	984.2759574	1137.943241
hsa-miR-301b-3p	-1.250008354	0.40645558	0.20530457	36.36087525	43.40078157	50.20691815	42.86474783	51.68265107	61.49647852
hsa-miR-130b-3p	1.009079295	0.96770494	0.94257952	507.9266552	557.8506888	646.0337734	544.1663183	554.0210495	565.2165586
hsa-miR-130a-3p	-1.395259378	0.03331209	0.00603	1605.675172	1928.876008	2436.262448	2480.725381	2691.120375	2829.354789
hsa-miR-4295				0	0	0	0	0	0
hsa-miR-33b-3p	-2.082620051	0.17240127	0.05335865	3.161815239	3.660645898	4.183909846	2.990563802	6.908302095	11.885874

hsa-miR-3677-5p				0	0	0	0	0	0
hsa-miR-140-5p	-1.057452918	0.83857163	0.68394373	211.3146518	279.9004084	336.3496663	228.0902708	294.6966225	366.9117626
hsa-miR-4255				0	0	0	0	0	0
hsa-miR-101-5p	2.611478778	0.31204792	0.12960706	0.606035435	2.092732194	3.161815239	0	0.669433003	1.033554261
hsa-miR-5000-5p	-3.836717681		0.44908091	0	0	0	0	0.332284867	0.996854601
hsa-miR-6874-3p	1.82130972	0.58833609	0.38134485	0	1.361371862	3.030177174	0	0.822088875	1.949489494
hsa-miR-4259				0	0	0	0	0	0
hsa-miR-142-5p	-3.836695655		0.44908316	0	0	0	0	0.324914916	0.974744747
hsa-miR-4666a-3p				0	0	0	0	0	0
hsa-miR-10397-3p				0	0	0	0	0	0
hsa-miR-3651				0	0	0	0	0	0
hsa-miR-2115-3p				0	0	0	0	0	0
hsa-miR-3617-3p				0	0	0	0	0	0
hsa-miR-217-3p				0	0	0	0	0	0
hsa-miR-136-3p	-15.34460457		0.09093486	0	0	0	0.51677713	1.479288657	2.924234241
hsa-miR-188-5p	-1.037244539	0.93841009	0.85617692	17.38998382	21.12924413	24.24141739	13.64642646	21.59220883	28.90878342
hsa-miR-4800-3p	3.543660986		0.51087004	0	0.175656402	0.526969207	0	0	0
hsa-miR-3675-3p	1.947160856	0.19865641	0.06641214	4.848283478	5.397535257	6.323630479	1.949489494	3.00029034	4.984273004
hsa-miR-3126-3p	-2.460729457	0.27739988	0.11136492	0	0.93099283	1.58090762	1.033554261	2.958577315	5.848468483
hsa-miR-1298-3p				0	0	0	0	0	0
hsa-miR-200a-5p	-7.100074952		0.25157641	0	0	0	0	0.649829831	1.949489494
hsa-miR-200b-5p	3.543845864		0.51085051	0	0.202011812	0.606035435	0	0	0
hsa-miR-141-5p	-1.586090396	0.44704443	0.2329852	3.030177174	3.179706763	3.347127876	1.993709202	4.395459177	8.268434087
hsa-miR-4757-3p				0	0	0	0	0	0
hsa-miR-532-5p	-1.016877064	0.95109507	0.89277976	820.8831117	922.9527825	975.7170499	848.3232652	939.314694	999.1133658
hsa-miR-3146	4.087234715	0.16797341	0.0505182	0.836781969	2.140912982	3.161815239	0	0.497173959	0.974744747
hsa-miR-4728-3p	1.513623305		0.70103844	0	0.404023623	1.212070869	0	0.332284867	0.996854601
hsa-miR-6871-5p				0	0	0	0	0	0
hsa-miR-6890-5p				0	0	0	0	0	0
hsa-miR-218-2-3p				0	0	0	0	0	0
hsa-miR-6504-3p				0	0	0	0	0	0
hsa-miR-126-5p	1.096841872	0.62948546	0.42180121	2369.780522	2559.682352	2921.090795	2011.873158	2331.867131	2721.865146
hsa-miR-4272				0	0	0	0	0	0
hsa-miR-25-3p	1.12521431	0.50388098	0.28367566	12001.127	12836.33674	13448.53233	10569.64933	11407.20988	11859.51837
hsa-miR-548o-3p	1.65452708	0.3955824	0.19379328	3.347127876	4.971457658	7.878460651	1.949489494	3.01252356	3.987418403
hsa-miR-6715a-3p				0	0	0	0	0	0
hsa-miR-4762-5p	1.93887859		0.39970694	0	0.957348239	1.818106304	0	0.657199783	0.996854601
hsa-miR-4714-3p	-1.034523965	0.96770494	0.93997214	1.212070869	3.410453297	5.857473784	1.993709202	3.475354445	5.848468483
hsa-miR-6830-5p				0	0	0	0	0	0
hsa-miR-16-2-3p	1.019812596	0.95639406	0.91090214	44.24058673	48.06658891	50.58904383	38.01504514	46.64738879	52.83329384
hsa-miR-195-3p	-1.255215433	0.53203037	0.30551073	9.696566955	15.92964785	21.7563312	14.95281901	18.90931336	23.25497087
hsa-miR-4275				0	0	0	0	0	0
hsa-miR-4733-3p	-3.835816219		0.44917283	0	0	0	0	0.172259043	0.51677713
hsa-miR-181d-3p	2.554021059	0.17240127	0.05360799	1.673563938	4.334536784	6.060354347	0	1.663780921	2.924234241
hsa-miR-1306-5p	1.436053352	0.40479328	0.20387399	5.857473784	10.11189097	13.938815	5.981127605	7.176914179	7.797957977
hsa-miR-6767-3p				0	0	0	0	0	0
hsa-miR-6890-3p				0	0	0	0	0	0
hsa-miR-3614-5p	6.528067129	0.1489464	0.0424008	1.212070869	2.222358524	3.347127876	0	0.172259043	0.51677713
hsa-miR-1324				0	0	0	0	0	0
hsa-miR-4494				0	0	0	0	0	0
hsa-miR-4756-3p				0	0	0	0	0	0
hsa-miR-6501-3p				0	0	0	0	0	0
hsa-miR-4257				0	0	0	0	0	0
hsa-miR-4691-3p	1.513821126		0.70687876	0	0.351312804	1.053938413	0	0.172259043	0.51677713
hsa-miR-4768-3p				0	0	0	0	0	0
hsa-miR-3155b				0	0	0	0	0	0
hsa-miR-3155a	-3.836717681		0.44908091	0	0	0	0	0.332284867	0.996854601
hsa-miR-6754-5p				0	0	0	0	0	0
hsa-miR-6798-5p				0	0	0	0	0	0
hsa-miR-16-1-3p	-1.343318997	0.56165999	0.34710476	7.904538098	9.060331871	10.90863782	5.848468483	11.51036339	21.70463948
hsa-miR-1910-5p	-2.11234523	0.3158808	0.131886	0.606035435	1.390106585	2.510345907	1.949489494	2.52021287	3.617439913
hsa-miR-5190				0	0	0	0	0	0
hsa-miR-194-3p	-2.758180853	0.07078478	0.01426029	0.526969207	2.301424752	3.347127876	3.898978988	5.55720287	8.785211217
hsa-miR-4302				0	0	0	0	0	0
hsa-miR-935	1.068512555	0.77631463	0.59116052	165.4683309	189.3500238	215.1425793	159.1673562	178.4777646	199.8226732
hsa-miR-4328				0	0	0	0	0	0
hsa-miR-8060				0	0	0	0	0	0
hsa-miR-490-5p				0	0	0	0	0	0
hsa-miR-10226				0	0	0	0	0	0
hsa-miR-323b-3p	-1.972097433		0.52419545	0	0.175656402	0.526969207	0	0.497173959	0.974744747
hsa-miR-324-3p	1.217446015	0.29610451	0.11973861	121.2029175	130.4921592	147.2666106	100.6823147	107.9967181	118.9188591
hsa-miR-9902				0	0	0	0	0	0
hsa-miR-488-5p				0	0	0	0	0	0
hsa-miR-4530				0	0	0	0	0	0
hsa-miR-4685-5p				0	0	0	0	0	0
hsa-miR-199a-5p	1.176418474	0.39013864	0.18563834	10862.94322	13498.36923	16623.55197	9780.588792	11473.4567	12395.41625
hsa-miR-199b-5p	-4.810520002	1.668E-32	2.679E-34	214.2161841	240.6359413	276.3521582	1044.926369	1158.386035	1330.701111
hsa-miR-6884-3p	-6.71207816		0.25810492	0	0	0	0	0.50454391	0.996854601
hsa-miR-6872-3p				0	0	0	0	0	0
hsa-miR-1224-3p	-3.836717681		0.44908091	0	0	0	0	0.332284867	0.996854601
hsa-miR-1915-3p	3.544185779		0.5108146	0	0.278927323	0.836781969	0	0	0
hsa-miR-4258				0	0	0	0	0	0
hsa-miR-1247-3p				0	0	0	0	0	0
hsa-miR-4783-3p	1.506976905	0.61606818	0.40996826	1.58090762	2.373810233	3.030177174	0	1.338866005	2.067108522
hsa-miR-4292	-3.835816219		0.44917283	0	0	0	0	0.172259043	0.51677713
hsa-miR-6791-5p				0	0	0	0	0	0
hsa-miR-6886-5p	-1.114069998		0.90713933	0	0.557854646	1.673563938	0	0.344518087	1.033554261
hsa-miR-1203				0	0	0	0	0	0
hsa-miR-6850-3p	2.034976507		0.50409615	0	0.606035435	1.818106304	0	0.332284867	0.996854601
hsa-miR-6891-3p				0	0	0	0	0	0
hsa-miR-4723-3p	-1.126853197		0.92121401	0	0.278927323	0.836781969	0	0.172259043	0.51677713
hsa-miR-6769b-3p	2.655363241	0.3955824	0.19403421	1.053938413	1.515202885	1.818106304	0	0.649829831	1.949489494
hsa-miR-4324	-3.835816219		0.44917283	0	0	0	0	0.172259043	0.51677713
hsa-miR-6893-3p				0	0	0	0	0	0
hsa-miR-939-3p	2.611750286	0.31035511	0.12817726	1.818106304	2.145443013	2.510345907	0	0.669433003	1.033554261
hsa-miR-6852-5p				0	0	0	0	0	0
hsa-miR-1914-5p	3.543845864		0.51085051	0	0.202011812	0.606035435	0	0	0
hsa-miR-1266-3p	3.543660986		0.51087004	0	0.175656402	0.526969207	0	0	0
hsa-miR-6734-3p	6.045864117		0.32988072	0	0.351312804	1.053938413	0	0	0
hsa-miR-3189-3p	-1.971994552		0.5242206	0	0.202011812	0.606035435	0	0.497173959	0.974744747
hsa-miR-106b-3p	1.286220788	0.18473928	0.05852325	478.6392863	521.7381044	585.9897577	365.8456385	407.289681	475.6754366
hsa-miR-6078				0	0	0	0	0	0
hsa-miR-6880-3p	-3.835816219		0.44917283	0	0	0	0	0.172259043	0.51677713
hsa-miR-6812-3p	-1.127278468		0.92096409	0	0.175656402	0.526969207	0	0.324914916	0.974744747
hsa-miR-4315				0	0	0	0	0	0
hsa-miR-4304				0	0	0	0	0	0
hsa-miR-10392-3p				0	0	0	0	0	0
hsa-miR-1908-3p	-6.71207816		0.25810492	0	0	0	0	0.50454391	0.996854601
hsa-miR-6746-5p				0	0	0	0	0	0
hsa-miR-5090				0	0	0	0	0	0
hsa-miR-4746-5p	1.300416989	0.7723693	0.58068641	1.673563938	2.217828494	3.161815239	1.033554261	1.983832568	2.924234241
hsa-miR-6070				0	0	0	0	0	0
hsa-miR-1181				0	0	0	0	0	0

hsa-miR-4735-5p				0	0	0	0	0	0
hsa-miR-8067				0	0	0	0	0	0
hsa-miR-4305				0	0	0	0	0	0
hsa-miR-325				0	0	0	0	0	0
hsa-miR-26a-2-3p	-1.085682042	0.86978632	0.72033376	28.98330636	40.6525072	60.24830177	24.36861868	43.45218588	54.82700304
hsa-miR-26a-1-3p	1.145761315	0.64643168	0.4369312	47.42722859	50.02338161	53.55404602	33.89305643	41.32832424	50.12738165
hsa-miR-372-5p				0	0	0	0	0	0
hsa-miR-6511b-3p	1.058176924	0.94510719	0.87598576	2.510345907	3.80541686	5.269692065	1.949489494	3.689326514	4.984273004
hsa-miR-6511a-3p	-1.193144883	0.77290285	0.58221587	4.183909846	5.118607934	6.323630479	4.984273004	6.480508054	8.772702724
hsa-miR-6862-3p				0	0	0	0	0	0
hsa-miR-4330				0	0	0	0	0	0
hsa-miR-1266-5p	1.22312497		0.82677544	0	0.526969207	1.58090762	0	0.50454391	0.996854601
hsa-miR-519d-5p				0	0	0	0	0	0
hsa-miR-532-3p	1.085882614	0.82459654	0.6512507	38.46875208	41.03520662	43.6345513	32.04018209	39.21297661	49.7119821
hsa-miR-2116-3p	-1.517841793		0.56493154	0.526969207	0.656595537	0.836781969	0	0.994347918	1.949489494
hsa-miR-7113-3p				0	0	0	0	0	0
hsa-miR-3605-3p	-1.07481645	0.92728265	0.82981644	3.030177174	4.264530616	5.020691815	3.617439913	4.831467036	6.977982205
hsa-miR-1976	2.04040424		0.50146558	0	0.606035435	1.818106304	0	0.324914916	0.974744747
hsa-miR-8088				0	0	0	0	0	0
hsa-miR-520f-5p				0	0	0	0	0	0
hsa-miR-520b-5p	8.550320498		0.236707	0	0.733511048	1.673563938	0	0	0
hsa-miR-517-5p				0	0	0	0	0	0
hsa-miR-3059-3p	-18.21524977		0.07073366	0	0	0	0	1.513631731	2.990563802
hsa-miR-6845-3p	-1.250467405		0.82827054	0	0.404023623	1.212070869	0	0.649829831	1.949489494
hsa-miR-146a-3p				0	0	0	0	0	0
hsa-miR-326	1.082432222	0.89225575	0.76981481	8.367819691	11.57223232	14.22816858	5.981127605	10.67673506	13.64642646
hsa-miR-1236-3p	-1.181231608	0.89225575	0.76800466	0.526969207	1.339546483	1.818106304	1.033554261	1.658917652	1.993709202
hsa-miR-6730-3p				0	0	0	0	0	0
hsa-miR-4750-3p				0	0	0	0	0	0
hsa-miR-4251				0	0	0	0	0	0
hsa-miR-4329				0	0	0	0	0	0
hsa-miR-1199-5p				0	0	0	0	0	0
hsa-miR-3151-3p				0	0	0	0	0	0
hsa-miR-4772-3p				0	0	0	0	0	0
hsa-miR-3158-5p				0	0	0	0	0	0
hsa-miR-1184				0	0	0	0	0	0
hsa-miR-6068				0	0	0	0	0	0
hsa-miR-4520-5p				0	0	0	0	0	0
hsa-miR-3692-5p				0	0	0	0	0	0
hsa-miR-875-3p	-15.8682632	8.348E-21	1.95E-22	3.030177174	4.295416055	6.694255753	55.82385764	63.02159928	71.83202113
hsa-miR-4638-3p	-6.710501692		0.25817211	0	0	0	0	0.344518087	1.033554261
hsa-miR-4720-5p				0	0	0	0	0	0
hsa-miR-3187-5p	13.55222419		0.144307	0	0.93099283	1.58090762	0	0	0
hsa-miR-8089				0	0	0	0	0	0
hsa-miR-4456				0	0	0	0	0	0
hsa-miR-3120-5p				0	0	0	0	0	0
hsa-miR-6529-3p				0	0	0	0	0	0
hsa-miR-5008-3p				0	0	0	0	0	0
hsa-miR-134-3p				0	0	0	0	0	0
hsa-miR-26b-3p	-1.100475284	0.83107589	0.65956029	15.7569213	17.39191132	20.08276726	8.772702724	18.15841731	23.771748
hsa-miR-1267	8.548235796		0.23676536	0	0.553324616	1.053938413	0	0	0
hsa-miR-6837-3p	-1.126961281		0.92115277	0	0.202011812	0.606035435	0	0.172259043	0.51677713
hsa-miR-570-3p	1.100757336	0.89225575	0.76508728	3.688784446	8.722609157	13.38851151	5.981127605	7.501829095	8.772702724
hsa-miR-15b-3p	1.027974779	0.92728265	0.82966632	477.9610703	526.6286832	573.9155567	450.5782795	509.3896485	591.1930372
hsa-miR-6790-3p				0	0	0	0	0	0
hsa-miR-2114-3p				0	0	0	0	0	0
hsa-miR-3616-3p				0	0	0	0	0	0
hsa-miR-6738-5p				0	0	0	0	0	0
hsa-miR-6804-3p	3.544185779		0.5108146	0	0.278927323	0.836781969	0	0	0
hsa-miR-3166				0	0	0	0	0	0
hsa-miR-6836-5p				0	0	0	0	0	0
hsa-miR-1909-3p	3.544185779		0.5108146	0	0.278927323	0.836781969	0	0	0
hsa-miR-324-5p	1.027924328	0.93101652	0.8372353	328.8287849	381.7410222	473.3136745	336.9368551	370.5299008	414.9720357
hsa-miR-4749-3p				0	0	0	0	0	0
hsa-miR-6869-3p				0	0	0	0	0	0
hsa-miR-2682-3p	4.176297244	6.508E-11	3.515E-12	63.02768521	87.35044195	121.2029175	17.57042243	21.28994744	24.36861868
hsa-miR-4763-5p				0	0	0	0	0	0
hsa-miR-3195	-6.71207816		0.25810492	0	0	0	0	0.50454391	0.996854601
hsa-miR-6720-3p				0	0	0	0	0	0
hsa-miR-4479	8.550766568		0.23669451	0	0.759866458	1.673563938	0	0	0
hsa-miR-4767				0	0	0	0	0	0
hsa-miR-3621				0	0	0	0	0	0
hsa-miR-1301-5p				0	0	0	0	0	0
hsa-miR-9851-5p				0	0	0	0	0	0
hsa-miR-1251-3p				0	0	0	0	0	0
hsa-miR-1255b-5p	2.201848342	0.36210109	0.16334195	0.836781969	2.193623801	3.636212608	0	0.841692046	1.550331391
hsa-miR-196a-3p	-1.149053209	0.741328	0.53353972	18.97089144	25.05152201	30.12415089	14.62117121	27.77393122	39.79183904
hsa-miR-874-5p	1.351401976	0.57236579	0.36430874	4.848283478	5.604077099	6.694255753	2.990563802	4.304813272	6.82321323
hsa-miR-1538				0	0	0	0	0	0
hsa-miR-6789-3p	3.543845864		0.51085051	0	0.202011812	0.606035435	0	0	0
hsa-miR-4634				0	0	0	0	0	0
hsa-miR-10400-5p				0	0	0	0	0	0
hsa-miR-1908-5p	-1.610299713	0.54326907	0.32196738	0.526969207	1.820485618	2.510345907	0.974744747	2.527582821	3.617439913
hsa-miR-10396b-5p				0	0	0	0	0	0
hsa-miR-760	1.612131932	0.25882881	0.10044877	6.694255753	12.99930892	18.97089144	7.751656957	8.498683696	8.971691407
hsa-miR-3652				0	0	0	0	0	0
hsa-miR-8052				0	0	0	0	0	0
hsa-miR-6726-5p				0	0	0	0	0	0
hsa-miR-6862-5p				0	0	0	0	0	0
hsa-miR-6781-5p				0	0	0	0	0	0
hsa-miR-9899				0	0	0	0	0	0
hsa-miR-1268a	-1.284233779	0.44748829	0.23452306	20.91954923	27.39362203	33.33194891	23.39387393	34.73451675	43.92605609
hsa-miR-1268b	-1.127020076		0.92111946	0	0.175656402	0.526969207	0	0.172259043	0.51677713
hsa-miR-4741				0	0	0	0	0	0
hsa-miR-3175				0	0	0	0	0	0
hsa-miR-486-3p	-6.710501692		0.25817211	0	0	0	0	0.344518087	1.033554261
hsa-miR-6845-5p				0	0	0	0	0	0
hsa-miR-6762-5p				0	0	0	0	0	0
hsa-miR-128-1-5p	1.306570811	0.66505512	0.45825696	3.030177174	4.512572499	6.323630479	2.990563802	3.65498344	4.873723736
hsa-miR-3196				0	0	0	0	0	0
hsa-miR-1237-5p				0	0	0	0	0	0
hsa-miR-7704				0	0	0	0	0	0
hsa-miR-4651				0	0	0	0	0	0
hsa-miR-193b-5p	1.393522615	0.41743101	0.21389531	10.30260239	12.15429166	15.28210699	6.718102696	9.498044979	14.95281901
hsa-miR-197-5p	-3.836695655		0.44908316	0	0	0	0	0.324914916	0.974744747
hsa-miR-363-5p	-3.836717681		0.44908091	0	0	0	0	0.332284867	0.996854601
hsa-miR-6766-5p				0	0	0	0	0	0
hsa-miR-12130				0	0	0	0	0	0
hsa-miR-9901	3.543660986		0.51087004	0	0.175656402	0.526969207	0	0	0
hsa-miR-8071				0	0	0	0	0	0
hsa-miR-505-3p	1.159426034	0.46328363	0.24888814	134.9041169	165.9838726	191.5071974	138.5627895	142.487933	145.2369673
hsa-miR-4433a-5p				0	0	0	0	0	0
hsa-miR-4449				0	0	0	0	0	0

hsa-miR-200c-5p	-2.568806094	0.34014857	0.15083978	0.526969207	0.656595537	0.836781969	0.974744747	1.838546647	2.990563802
hsa-miR-1227-3p	-2.281708627	0.29802817	0.12138666	0.526969207	1.214450183	2.510345907	0.996854601	2.340583875	3.100662783
hsa-miR-7847-3p				0	0	0	0	0	0
hsa-miR-376b-5p				0	0	0	0	0	0
hsa-miR-8056				0	0	0	0	0	0
hsa-miR-223-5p				0	0	0	0	0	0
hsa-miR-4711-3p				0	0	0	0	0	0
hsa-miR-10401-5p	3.543660986		0.51087004	0	0.175656402	0.526969207	0	0	0
hsa-miR-124-5p				0	0	0	0	0	0
hsa-miR-633				0	0	0	0	0	0
hsa-miR-524-5p				0	0	0	0	0	0
hsa-miR-520d-5p				0	0	0	0	0	0
hsa-miR-7151-3p	-113.0158076	0.01641484	0.002516	0	0	0	6.82321323	9.226926212	11.885874
hsa-miR-6798-3p	2.766976765		0.30346474	0.526969207	0.93552286	1.673563938	0	0.332284867	0.996854601
hsa-miR-7848-3p				0	0	0	0	0	0
hsa-miR-3682-5p				0	0	0	0	0	0
hsa-miR-151a-3p	1.253766044	0.14285303	0.03964629	8303.387479	8780.398339	9020.837446	6398.809682	6999.992667	7657.086742
hsa-miR-585-5p				0	0	0	0	0	0
hsa-miR-4266				0	0	0	0	0	0
hsa-miR-4278				0	0	0	0	0	0
hsa-miR-331-5p	1.400117305	0.53093569	0.30383473	5.857473784	7.538448239	9.485445718	3.898978988	5.360477386	6.201325565
hsa-miR-6802-5p				0	0	0	0	0	0
hsa-miR-921				0	0	0	0	0	0
hsa-miR-4473				0	0	0	0	0	0
hsa-let-7b-3p	1.579833276	0.53203037	0.30670239	1.673563938	4.466313831	9.090531521	2.067108522	2.985550437	3.898978988
hsa-miR-98-3p	8.548681411		0.23675288	0	0.579680025	1.212070869	0	0	0
hsa-let-7a-3p	-1.039017819	0.89266359	0.772773	186.5470991	218.6245763	244.2322802	164.7318623	225.6851631	279.0596504
hsa-let-7f-1-3p	8.548235796		0.23676536	0	0.553324616	1.053938413	0	0	0
hsa-let-7f-2-3p	3.133258683	0.05602545	0.01055078	3.161815239	6.440630471	10.30260239	0.974744747	1.850779867	2.583885652
hsa-let-7d-3p	8.385935317	0.01839466	0.002873	3.688784446	4.846361358	6.666389782	0	0.50454391	0.996854601
hsa-let-7e-3p	2.150135411	0.01630337	0.002454	17.38998382	20.30175082	25.94024104	8.268434087	9.335512473	10.96540061
hsa-miR-190a-3p	3.372551252	0.25723756	0.09904122	1.053938413	1.99614202	2.510345907	0	0.497173959	0.974744747
hsa-miR-8074				0	0	0	0	0	0
hsa-miR-4477a	-3.835816219		0.44917283	0	0	0	0	0.172259043	0.51677713
hsa-miR-6715b-3p				0	0	0	0	0	0
hsa-miR-520e-5p				0	0	0	0	0	0
hsa-miR-4465	-13.9344666		0.11657391	0	0	0	0	1.307029614	2.924234241
hsa-miR-6794-3p				0	0	0	0	0	0
hsa-miR-181b-3p				0	0	0	0	0	0
hsa-miR-181b-2-3p	-1.082422575	0.94510719	0.87593984	1.58090762	2.527641256	4.183909846	0.996854601	2.340583875	3.100662783
hsa-miR-222-5p	-1.057492386	0.8800976	0.74519686	55.85873589	62.24136627	67.26993325	47.76249261	65.08178814	78.75151346
hsa-miR-4276				0	0	0	0	0	0
hsa-miR-452-3p				0	0	0	0	0	0
hsa-miR-1912-5p				0	0	0	0	0	0
hsa-miR-5696	-2.918037373	0.25211327	0.09642873	0.526969207	0.656595537	0.836781969	0.974744747	1.850779867	2.583885652
hsa-miR-520a-5p				0	0	0	0	0	0
hsa-miR-525-5p				0	0	0	0	0	0
hsa-miR-188-3p	1.005817868	0.99392903	0.99215983	1.053938413	1.313191074	1.673563938	0	1.01395109	2.067108522
hsa-miR-3156-3p				0	0	0	0	0	0
hsa-miR-6763-3p	-1.12711155		0.92105864	0	0.278927323	0.836781969	0	0.324914916	0.974744747
hsa-miR-6826-3p	-2.817214277		0.32625281	0	0.202011812	0.606035435	0	0.822088875	1.949489494
hsa-miR-4497	3.543660986		0.51087004	0	0.175656402	0.526969207	0	0	0
hsa-miR-1468-5p	-1.182395128	0.56165999	0.34632909	18.18106304	20.86569004	22.65967588	20.46963969	24.08780516	26.87241078
hsa-miR-155-3p				0	0	0	0	0	0
hsa-miR-337-3p				0	0	0	0	0	0
hsa-miR-6792-3p				0	0	0	0	0	0
hsa-miR-6749-3p	-9.589007565		0.1718824	0	0	0	0	0.836828777	1.993709202
hsa-miR-6780a-3p				0	0	0	0	0	0
hsa-miR-378a-5p	1.288685515	0.120468	0.03060063	242.666771	268.5286226	307.8660008	193.3897925	207.0566632	228.9322688
hsa-miR-1200				0	0	0	0	0	0
hsa-miR-1343-3p	-2.146907497		0.34347195	0	0.351312804	1.053938413	0.51677713	1.161743693	1.993709202
hsa-miR-4444				0	0	0	0	0	0
hsa-miR-6726-3p	-1.972097433		0.52419545	0	0.175656402	0.526969207	0	0.497173959	0.974744747
hsa-miR-4665-3p				0	0	0	0	0	0
hsa-miR-1469				0	0	0	0	0	0
hsa-miR-6771-5p				0	0	0	0	0	0
hsa-miR-4750-5p	6.046759143		0.32983659	0	0.454583725	0.836781969	0	0	0
hsa-miR-6727-5p				0	0	0	0	0	0
hsa-miR-3677-3p	3.543660986		0.51087004	0	0.175656402	0.526969207	0	0	0
hsa-miR-518f-5p				0	0	0	0	0	0
hsa-miR-526a-5p				0	0	0	0	0	0
hsa-miR-519c-5p	73.27538778	0.07179599	0.01456882	2.510345907	5.693757929	7.904538098	0	0	0
hsa-miR-1287-3p				0	0	0	0	0	0
hsa-miR-1229-3p	-1.186694728	0.86618737	0.71444651	1.212070869	2.119087603	2.634846033	1.949489494	2.347953826	3.100662783
hsa-miR-6867-3p				0	0	0	0	0	0
hsa-miR-4279				0	0	0	0	0	0
hsa-miR-10399-3p	2.176461424	0.02386105	0.004006	10.90863782	13.19308544	15.28210699	4.650994174	6.483014737	7.974836806
hsa-miR-4684-5p				0	0	0	0	0	0
hsa-miR-3191-5p				0	0	0	0	0	0
hsa-miR-3192-3p	3.543660986		0.51087004	0	0.175656402	0.526969207	0	0	0
hsa-miR-4314				0	0	0	0	0	0
hsa-miR-526b-5p				0	0	0	0	0	0
hsa-miR-7158-3p				0	0	0	0	0	0
hsa-miR-127-5p	-6.710501692		0.25817211	0	0	0	0	0.344518087	1.033554261
hsa-miR-573	1.328684785	0.69613117	0.4949137	2.424141739	3.535549598	5.020691815	0.996854601	2.187928003	3.617439913
hsa-miR-5585-3p	-3.836695655		0.44908316	0	0	0	0	0.324914916	0.974744747
hsa-miR-192-5p	-1.829901447	0.00003027	2.651E-06	635.1175145	698.6258653	807.8452345	1159.946249	1277.114772	1497.103347
hsa-miR-3136-5p	2.366828245	0.46197048	0.24750827	0	2.00067205	4.183909846	0	0.669433003	1.033554261
hsa-miR-943	1.112351363	0.90458342	0.7936564	2.510345907	3.278447654	3.688784446	1.993709202	2.997783658	3.898978988
hsa-miR-3161	8.548235796		0.23676536	0	0.553324616	1.053938413	0	0	0
hsa-miR-518a-5p	4.359132233	0.23272127	0.08442785	0	2.169419109	5.454318912	0	0.344518087	1.033554261
hsa-miR-106a-3p	-2.005146604	0.19581238	0.06517551	1.58090762	3.508965591	7.272425217	4.873723736	7.199024033	8.971691407
hsa-miR-1273h-3p				0	0	0	0	0	0
hsa-miR-3678-3p	-6.710501692		0.25817211	0	0	0	0	0.344518087	1.033554261
hsa-miR-7156-3p				0	0	0	0	0	0
hsa-miR-6511b-5p	-1.127278468		0.92096409	0	0.175656402	0.526969207	0	0.324914916	0.974744747
hsa-miR-192-3p	-1.438115081	0.56165999	0.34405159	2.424141739	3.080965872	4.183909846	2.924234241	4.186500473	4.984273004
hsa-miR-12121				0	0	0	0	0	0
hsa-miR-3972				0	0	0	0	0	0
hsa-miR-4659a-5p	3.543845864		0.51085051	0	0.202011812	0.606035435	0	0	0
hsa-miR-3157-3p				0	0	0	0	0	0
hsa-miR-874-3p	1.150624254	0.65481771	0.44737911	25.94024104	29.59415517	33.33194891	24.28852513	26.51250886	29.90563802
hsa-miR-6514-3p	-2.816585182		0.30244876	0	0.202011812	0.606035435	0	0.676802954	1.033554261
hsa-let-7i-3p	4.612748904	0.10404687	0.02582185	0.836781969	3.475929435	4.848283478	0	0.51677713	1.550331391
hsa-miR-1269b				0	0	0	0	0	0
hsa-miR-1269a	26.07164525	0.19306631	0.06369779	1.212070869	2.222358524	3.347127876	0	0	0
hsa-miR-8058				0	0	0	0	0	0
hsa-miR-1270				0	0	0	0	0	0
hsa-miR-4309				0	0	0	0	0	0
hsa-miR-432-3p				0	0	0	0	0	0
hsa-miR-7150				0	0	0	0	0	0
hsa-miR-328-3p	-1.49698861	0.35637021	0.15971629	4.242248043	7.365171151	9.485445718	6.82321323	10.72832472	12.95910981
hsa-miR-6878-3p				0	0	0	0	0	0

hsa-miR-4730	-1.12728494		0.9209602	0	0.175656402	0.526969207	0	0.332284867	0.996854601
hsa-miR-6770-3p	-3.267456246	0.21351159	0.07449528	0	0.733511048	1.673563938	0.996854601	2.015668959	3.100662783
hsa-miR-4298				0	0	0	0	0	0
hsa-miR-6788-5p				0	0	0	0	0	0
hsa-miR-30c-2-3p	-1.617217177	0.40770922	0.20712819	1.212070869	3.55522426	5.269692065	2.990563802	5.377573875	8.268434087
hsa-miR-30c-1-3p	-1.889098365	0.02019433	0.003243	10.8781656	12.03134608	13.70119937	17.54540545	22.27623164	26.35563365
hsa-miR-6779-5p				0	0	0	0	0	0
hsa-miR-1273h-5p				0	0	0	0	0	0
hsa-miR-30b-3p	-2.075839962	0.25882881	0.10182748	0.606035435	1.917075792	2.634846033	2.924234241	4.174267253	5.981127605
hsa-miR-3689a-3p	10.89231148		0.20392919	0	0.808047246	2.424141739	0	0	0
hsa-miR-3689b-3p				0	0	0	0	0	0
hsa-miR-769-3p	-1.085239976	0.83328558	0.67215942	22.59311317	28.39937965	31.5138426	24.92136502	32.09722853	41.91402413
hsa-miR-6724-5p	2.611500387	0.31035511	0.1282197	1.673563938	2.068527501	2.424141739	0	0.669433003	1.033554261
hsa-miR-10400-3p				0	0	0	0	0	0
hsa-miR-4674				0	0	0	0	0	0
hsa-miR-3150a-3p	3.903040765		0.2189257	0	1.053938413	3.161815239	0	0.172259043	0.51677713
hsa-miR-6763-5p	8.550766568		0.23669451	0	0.759866458	1.673563938	0	0	0
hsa-miR-4665-5p				0	0	0	0	0	0
hsa-miR-7109-5p				0	0	0	0	0	0
hsa-miR-6803-5p				0	0	0	0	0	0
hsa-miR-3170				0	0	0	0	0	0
hsa-miR-6736-5p	-1.12728494		0.9209602	0	0.175656402	0.526969207	0	0.332284867	0.996854601
hsa-miR-4507				0	0	0	0	0	0
hsa-miR-150-3p				0	0	0	0	0	0
hsa-miR-29b-2-5p	-1.06528968	0.92808503	0.83223118	7.377568892	7.874616411	8.367819691	2.990563802	7.996496372	11.69693697
hsa-miR-2054				0	0	0	0	0	0
hsa-let-7c-3p	-2.977556224	0.001171	0.0001487	3.030177174	4.998041664	6.694255753	10.72219222	14.07062647	16.53686817
hsa-let-7a-2-3p	3.543845864		0.51085051	0	0.202011812	0.606035435	0	0	0
hsa-let-7g-3p	4.646201413		0.10282674	1.212070869	1.488847476	1.673563938	0	0.324914916	0.974744747
hsa-miR-675-3p	3.670955264	0.0006399	0.00007286	10.53938413	14.39216239	17.57502761	2.583885652	4.162034033	6.977982205
hsa-miR-676-3p	-1.716919606	0.07597088	0.01585961	10.30260239	12.0095207	13.17423016	19.12075383	21.16426469	22.92765582
hsa-miR-210-3p	-1.180647644	0.46012561	0.24546826	95.14756325	102.9938013	112.1287839	98.68860547	118.8188642	144.6975965
hsa-miR-4322				0	0	0	0	0	0
hsa-miR-4265				0	0	0	0	0	0
hsa-miR-744-3p	1.249712696	0.71985744	0.5159854	4.183909846	5.02201776	6.666389782	2.924234241	3.854215606	4.650994174
hsa-miR-7702				0	0	0	0	0	0
hsa-miR-8061				0	0	0	0	0	0
hsa-miR-374b-3p	-1.208938531	0.49656668	0.27546765	40.5766289	52.77998626	64.43221162	45.81300311	62.19159678	75.96623817
hsa-miR-6083				0	0	0	0	0	0
hsa-miR-374a-3p	-1.173479255	0.38005615	0.17547877	210.0322742	225.06543	241.202103	213.4690996	262.3083968	316.2676038
hsa-miR-491-3p	1.871276781	0.41000418	0.20949118	1.818106304	3.612465109	5.857473784	0.996854601	1.823806744	2.924234241
hsa-miR-3180-5p	-1.300569012	0.75962047	0.54958479	0.836781969	2.89624941	4.215753652	1.949489494	4.341663028	7.974836806
hsa-miR-3679-3p	3.392557361		0.20682336	0	0.93099283	1.58090762	0	0.324914916	0.974744747
hsa-miR-718				0	0	0	0	0	0
hsa-miR-4664-3p	-1.302436699	0.77631463	0.58795567	1.58090762	2.171798422	2.510345907	1.550331391	3.13820631	4.873723736
hsa-miR-1238-3p				0	0	0	0	0	0
hsa-miR-6809-3p				0	0	0	0	0	0
hsa-miR-4762-3p	3.013481984	0.31869443	0.13673508	0.836781969	1.587588366	2.107876826	0	0.50454391	0.996854601
hsa-miR-6738-3p				0	0	0	0	0	0
hsa-miR-578	-3.835816219		0.44917283	0	0	0	0	0.172259043	0.51677713
hsa-miR-8081				0	0	0	0	0	0
hsa-miR-4776-3p				0	0	0	0	0	0
hsa-miR-5004-3p				0	0	0	0	0	0
hsa-miR-12133				0	0	0	0	0	0
hsa-miR-1271-5p	1.934699819	0.00005494	5.133E-06	130.161394	132.2560898	133.8851151	64.5971413	69.13145999	77.00483502
hsa-miR-887-5p	-15.33757961		0.08999788	0	0	0	0.974744747	1.173976913	1.550331391
hsa-miR-4260				0	0	0	0	0	0
hsa-miR-659-3p	-1.127020076		0.92111946	0	0.175656402	0.526969207	0	0.172259043	0.51677713
hsa-miR-30d-3p	-1.611723614	0.08130617	0.01776123	26.34846033	39.27470874	46.0230083	45.81300311	61.99387733	87.33533504
hsa-miR-30e-3p	-1.211784307	0.32474344	0.14032709	434.289842	458.3069643	504.8275171	502.9682895	552.9252604	644.4210817
hsa-miR-30a-3p	-1.158678443	0.58373563	0.37580644	67.97902764	71.99526369	79.39064195	66.2826428	83.72097669	101.6791693
hsa-miR-8055				0	0	0	0	0	0
hsa-miR-6885-3p	1.859090476	0.56165999	0.34827627	0	1.335016453	2.424141739	0	0.676802954	1.033554261
hsa-miR-129-5p	-5.776747381	4.188E-18	1.225E-19	9.20460166	14.3280122	20.60520478	77.75465886	84.63646164	89.40244357
hsa-miR-12136	1.687358708	0.00007317	7.156E-06	290.3633433	300.588008	319.9867095	147.1864568	175.6610043	196.3753096
hsa-miR-548ad-3p				0	0	0	0	0	0
hsa-miR-4668-3p				0	0	0	0	0	0
hsa-miR-3662	-1.085054903	0.88673682	0.75599168	7.272425217	9.501554103	13.70119937	6.82321323	10.07598821	13.43620539
hsa-miR-4311				0	0	0	0	0	0
hsa-miR-518a-3p	3.543845864		0.51085051	0	0.202011812	0.606035435	0	0	0
hsa-miR-526a-3p				0	0	0	0	0	0
hsa-miR-518f-3p	2.140500396		0.45652905	0	0.579680025	1.212070869	0	0.172259043	0.51677713
hsa-miR-548aq-5p	1.045030614	0.96770494	0.94607676	0.526969207	1.06061916	1.818106304	0	0.689036174	2.067108522
hsa-miR-8054				0	0	0	0	0	0
hsa-miR-526b-3p				0	0	0	0	0	0
hsa-miR-551b-5p	-1.97210875		0.52419149	0	0.175656402	0.526969207	0	0.50454391	0.996854601
hsa-miR-614				0	0	0	0	0	0
hsa-miR-523-3p				0	0	0	0	0	0
hsa-miR-337-5p	-12.46653252		0.12042892	0	0	0	0.51677713	1.161743693	1.993709202
hsa-miR-6750-3p	-1.971542787		0.5243785	0	0.202011812	0.606035435	0	0.344518087	1.033554261
hsa-miR-4511	1.514124203		0.70082798	0	0.480939135	0.836781969	0	0.172259043	0.51677713
hsa-miR-6507-5p	-35.46917239	0.09899549	0.02364114	0	0	0	2.924234241	3.005153609	3.100662783
hsa-miR-8087				0	0	0	0	0	0
hsa-miR-4426				0	0	0	0	0	0
hsa-miR-4647				0	0	0	0	0	0
hsa-miR-6796-3p	-3.835816219		0.44917283	0	0	0	0	0.172259043	0.51677713
hsa-miR-8080				0	0	0	0	0	0
hsa-miR-6816-3p				0	0	0	0	0	0
hsa-miR-3714				0	0	0	0	0	0
hsa-miR-525-3p				0	0	0	0	0	0
hsa-miR-524-3p				0	0	0	0	0	0
hsa-miR-373-3p	6.045864117		0.32988072	0	0.351312804	1.053938413	0	0	0
hsa-miR-12117				0	0	0	0	0	0
hsa-miR-595				0	0	0	0	0	0
hsa-miR-495-5p				0	0	0	0	0	0
hsa-miR-382-5p	-8.09241619	0.01630337	0.002475	0	0.377668214	0.606035435	3.100662783	4.312183223	5.848468483
hsa-miR-6082				0	0	0	0	0	0
hsa-miR-8084				0	0	0	0	0	0
hsa-miR-5583-3p	1.063645167	0.95020048	0.88777855	2.107876826	3.03040577	3.636212608	0.974744747	2.527582821	3.617439913
hsa-miR-3610				0	0	0	0	0	0
hsa-miR-409-3p	-14.03172609	0.001123	0.0001386	0	0.480939135	0.836781969	4.984273004	7.149941057	9.747447471
hsa-miR-10523-5p	1.328221008	0.78927331	0.61845538	1.58090762	2.700918345	4.848283478	0	1.710357215	4.134217044
hsa-miR-7849-3p				0	0	0	0	0	0
hsa-miR-4643				0	0	0	0	0	0
hsa-miR-602				0	0	0	0	0	0
hsa-miR-4704-5p				0	0	0	0	0	0
hsa-miR-4797-5p				0	0	0	0	0	0
hsa-miR-4262				0	0	0	0	0	0
hsa-miR-4523	1.161991005	0.91504057	0.80894564	0.526969207	1.464642783	3.030177174	0.996854601	1.326632785	1.949489494
hsa-miR-619-3p				0	0	0	0	0	0
hsa-miR-625-3p	-1.013616599	0.95639406	0.91311199	154.8046643	177.6196536	191.5071974	161.4904453	181.9300152	208.5953759
hsa-miR-3680-5p	1.581257087		0.57190555	0	0.755336428	1.212070869	0	0.50454391	0.996854601
hsa-miR-888-3p				0	0	0	0	0	0

hsa-miR-3654				0	0	0	0	0	0
hsa-miR-12126				0	0	0	0	0	0
hsa-miR-5680	1.581183758		0.57194863	0	0.728981018	1.58090762	0	0.497173959	0.974744747
hsa-miR-4524b-3p				0	0	0	0	0	0
hsa-miR-4463				0	0	0	0	0	0
hsa-miR-6529-5p				0	0	0	0	0	0
hsa-miR-2278	1.652559416	0.39536146	0.19219762	4.183909846	4.617994137	5.454318912	0.996854601	2.970810535	5.848468483
hsa-miR-6859-5p	-3.176649946	0.31204792	0.12983016	0	0.630240127	1.053938413	0	2.294007581	5.848468483
hsa-miR-3908	-2.146073531		0.34237803	0	0.454583725	0.836781969	0	0.994347918	1.949489494
hsa-miR-4690-5p				0	0	0	0	0	0
hsa-miR-4676-5p				0	0	0	0	0	0
hsa-miR-575				0	0	0	0	0	0
hsa-miR-6769a-3p				0	0	0	0	0	0
hsa-miR-590-5p	1.022107384	0.95639406	0.91222156	43.73844414	67.04160794	92.11738608	48.73723736	65.27114367	75.76094966
hsa-miR-4538				0	0	0	0	0	0
hsa-miR-4453				0	0	0	0	0	0
hsa-miR-208a-5p				0	0	0	0	0	0
hsa-miR-4496				0	0	0	0	0	0
hsa-miR-12124				0	0	0	0	0	0
hsa-miR-1910-3p				0	0	0	0	0	0
hsa-miR-4478				0	0	0	0	0	0
hsa-miR-3929				0	0	0	0	0	0
hsa-miR-7843-5p				0	0	0	0	0	0
hsa-miR-4632-5p				0	0	0	0	0	0
hsa-miR-4761-3p	1.581546114		0.57178512	0	0.728981018	1.58090762	0	0.344518087	1.033554261
hsa-miR-3141	3.544185779		0.5108146	0	0.278927323	0.836781969	0	0	0
hsa-miR-1182				0	0	0	0	0	0
hsa-miR-296-3p	-2.146291617		0.34232812	0	0.377668214	0.606035435	0.974744747	1.00171787	1.033554261
hsa-miR-4748	-6.71207816		0.25810492	0	0	0	0	0.50454391	0.996854601
hsa-miR-329-5p				0	0	0	0	0	0
hsa-miR-515-3p				0	0	0	0	0	0
hsa-miR-6849-5p	3.543660986		0.51087004	0	0.175656402	0.526969207	0	0	0
hsa-miR-1296-3p	-1.126961281		0.92115277	0	0.202011812	0.606035435	0	0.172259043	0.51677713
hsa-miR-4280				0	0	0	0	0	0
hsa-miR-3168	-2.149977966	0.37186568	0.16991818	0	1.567913704	3.030177174	1.949489494	3.02475678	4.134217044
hsa-miR-3074-3p	-3.230333731	0.28466839	0.1146985	0.526969207	0.656595537	0.836781969	0.51677713	2.490883161	5.981127605
hsa-miR-6074				0	0	0	0	0	0
hsa-miR-7151-5p	-12.46075309		0.1203013	0	0	0	0	0.849061997	1.550331391
hsa-miR-6866-3p	-1.167933758		0.84330386	0	0.553324616	1.053938413	0	0.822088875	1.949489494
hsa-miR-1285-5p	1.04438303	0.96770494	0.94679772	0	0.981552932	2.107876826	0.974744747	1.00171787	1.033554261
hsa-miR-556-5p	3.543845864		0.51085051	0	0.202011812	0.606035435	0	0	0
hsa-miR-1272	-1.576029626	0.61379757	0.40294834	0.526969207	2.253243964	5.020691815	1.550331391	3.485231079	5.981127605
hsa-miR-1322	-1.44509816	0.76832627	0.56820785	0	0.755336428	1.212070869	0.974744747	1.173976913	1.550331391
hsa-miR-4787-3p	-1.25075081	0.69094308	0.49021655	3.636212608	4.290886025	5.020691815	4.134217044	5.65355591	6.977982205
hsa-miR-758-5p				0	0	0	0	0	0
hsa-miR-12129				0	0	0	0	0	0
hsa-miR-205-3p				0	0	0	0	0	0
hsa-miR-330-3p	-1.867664801	4.382E-06	3.519E-07	99.99584673	106.4591162	117.1494757	183.4558813	199.8211146	210.3363208
hsa-miR-500b-3p	-3.256884767	0.32558507	0.14116608	0	0.682950946	1.212070869	0	2.446663453	6.82321323
hsa-miR-3919				0	0	0	0	0	0
hsa-miR-759				0	0	0	0	0	0
hsa-miR-1288-5p				0	0	0	0	0	0
hsa-miR-922				0	0	0	0	0	0
hsa-miR-761				0	0	0	0	0	0
hsa-miR-4310				0	0	0	0	0	0
hsa-miR-4690-3p	6.339263239		0.32187516	0	0.557854646	1.673563938	0	0	0
hsa-miR-873-5p	-6.641428608	2.962E-19	7.352E-21	10.01241492	14.52416804	18.40920332	80.90381401	92.42010508	111.6238602
hsa-miR-4436a				0	0	0	0	0	0
hsa-miR-4269				0	0	0	0	0	0
hsa-miR-211-3p	6.046494721		0.32984963	0	0.404023623	1.212070869	0	0	0
hsa-miR-764				0	0	0	0	0	0
hsa-miR-3129-5p	-7.590557645	0.02131174	0.003485	0	0.404023623	1.212070869	1.949489494	3.52930069	4.650994174
hsa-miR-455-3p	1.323114225	0.29802817	0.12137086	51.04370011	65.25964687	91.51135064	40.93927938	49.03690554	56.32870722
hsa-miR-5693				0	0	0	0	0	0
hsa-miR-4277				0	0	0	0	0	0
hsa-miR-4289				0	0	0	0	0	0
hsa-miR-186-3p	1.186639509	0.86978632	0.72383041	1.673563938	2.068527501	2.424141739	0.996854601	1.976462617	3.898978988
hsa-miR-6840-3p	3.393039631		0.20676287	0.836781969	1.034263751	1.212070869	0	0.332284867	0.996854601
hsa-miR-4707-5p	-1.126853197		0.92121401	0	0.278927323	0.836781969	0	0.172259043	0.51677713
hsa-miR-5587-3p				0	0	0	0	0	0
hsa-miR-331-3p	1.200483891	0.55089489	0.32973271	51.04370011	70.85429633	90.90531521	46.85216624	58.46527041	74.93268391
hsa-miR-4740-3p				0	0	0	0	0	0
hsa-miR-1471				0	0	0	0	0	0
hsa-miR-10398-3p				0	0	0	0	0	0
hsa-miR-1470				0	0	0	0	0	0
hsa-miR-2276-5p				0	0	0	0	0	0
hsa-miR-345-3p	-1.178108888	0.90300712	0.78878251	0.526969207	1.06061916	1.818106304	0.974744747	1.173976913	1.550331391
hsa-miR-4732-3p				0	0	0	0	0	0
hsa-miR-146b-3p	2.341352474	0.00003879	3.568E-06	53.55404602	64.41865309	70.61387368	23.92451042	27.99901737	32.16657665
hsa-miR-4442				0	0	0	0	0	0
hsa-miR-6784-5p				0	0	0	0	0	0
hsa-miR-3927-5p				0	0	0	0	0	0
hsa-miR-3183	1.513689498		0.70100621	0	0.454583725	0.836781969	0	0.332284867	0.996854601
hsa-miR-370-3p	-4.126394436	0.17176543	0.05290877	0	0.351312804	1.053938413	0.996854601	1.843409916	2.583885652
hsa-miR-4254	-1.624319986		0.49897174	0	0.630240127	1.053938413	0	1.307029614	2.924234241
hsa-miR-320a-5p	-3.835816219		0.44917283	0	0	0	0	0.172259043	0.51677713
hsa-miR-551a	-11.8751348	0.005079	0.000697	0	0.404023623	1.212070869	1.949489494	5.719735375	7.974836806
hsa-miR-551b-3p	1.65310796	0.4035061	0.20224481	3.161815239	4.670704956	6.666389782	0.996854601	2.818154663	4.873723736
hsa-miR-375-5p				0	0	0	0	0	0
hsa-miR-4431				0	0	0	0	0	0
hsa-miR-611				0	0	0	0	0	0
hsa-miR-6825-3p				0	0	0	0	0	0
hsa-miR-10392-5p				0	0	0	0	0	0
hsa-miR-6125				0	0	0	0	0	0
hsa-miR-5739				0	0	0	0	0	0
hsa-miR-4285				0	0	0	0	0	0
hsa-miR-4508				0	0	0	0	0	0
hsa-miR-4787-5p				0	0	0	0	0	0
hsa-miR-598-5p				0	0	0	0	0	0
hsa-miR-6786-5p				0	0	0	0	0	0
hsa-miR-4521	-1.021251781	0.97018991	0.95177755	4.183909846	5.979365999	8.484496086	2.990563802	5.549832918	8.785211217
hsa-miR-138-1-3p				0	0	0	0	0	0
hsa-miR-554				0	0	0	0	0	0
hsa-miR-138-2-3p	3.544185779		0.5108146	0	0.278927323	0.836781969	0	0	0
hsa-miR-4518				0	0	0	0	0	0
hsa-miR-6870-3p				0	0	0	0	0	0
hsa-miR-4469				0	0	0	0	0	0
hsa-miR-3917	-1.127118021		0.92105475	0	0.278927323	0.836781969	0	0.332284867	0.996854601
hsa-miR-301b-5p	-2.083974283	0.11386991	0.02842592	2.107876826	3.790272228	5.020691815	6.718102696	7.821880742	8.772702724
hsa-miR-301a-5p	-1.36585892	0.154705	0.04477747	82.00463297	117.9491715	169.0838863	127.6915619	161.1144699	190.173984
hsa-miR-130a-5p	-1.748757377	0.06332899	0.01229599	10.53938413	13.56466908	19.24598529	15.59591595	22.15541216	28.9395193
hsa-miR-4539				0	0	0	0	0	0
hsa-miR-4526				0	0	0	0	0	0

hsa-miR-345-5p	-1.949181365	0.000279	0.00003054	34.30806073	37.16133813	38.99572128	61.80498525	73.61575216	87.72702724
hsa-miR-4502	-1.971645644		0.52726264	0	0.175656402	0.526969207	0	0.344518087	1.033554261
hsa-miR-3130-3p				0	0	0	0	0	0
hsa-miR-191-3p	-1.444789016	0.02888232	0.005018	85.89598067	102.8513201	112.9655658	124.7673276	146.0002624	174.6706701
hsa-miR-4734				0	0	0	0	0	0
hsa-miR-204-3p				0	0	0	0	0	0
hsa-miR-619-5p	4.020837465		0.14417001	1.053938413	1.313191074	1.673563938	0	0.172259043	0.51677713
hsa-miR-612				0	0	0	0	0	0
hsa-miR-4486				0	0	0	0	0	0
hsa-miR-3663-5p				0	0	0	0	0	0
hsa-miR-4299				0	0	0	0	0	0
hsa-miR-548q	1.597998933	0.39706008	0.19679277	5.269692065	6.565955368	8.367819691	0.996854601	3.221482264	6.718102696
hsa-miR-29b-1-5p	2.863565546	5.909E-08	3.968E-09	94.32748797	112.9342477	131.5096893	28.26759767	38.22947437	47.543496
hsa-miR-3182	1.336278077	0.64513834	0.4351152	5.454318912	9.40125867	15.89885741	0.974744747	6.600633764	10.85231974
hsa-miR-3655				0	0	0	0	0	0
hsa-miR-1180-5p	-3.835816219		0.44917283	0	0	0	0	0.172259043	0.51677713
hsa-miR-539-5p				0	0	0	0	0	0
hsa-miR-873-3p	-5.171722197	1.223E-11	6.429E-13	7.531037722	9.940764599	13.33277956	35.88676563	51.53721505	65.11391844
hsa-miR-3928-3p	3.01349929	0.31869443	0.13673391	0.836781969	1.587588366	2.107876826	0	0.497173959	0.974744747
hsa-miR-6089				0	0	0	0	0	0
hsa-miR-3197				0	0	0	0	0	0
hsa-miR-1914-3p				0	0	0	0	0	0
hsa-miR-6086				0	0	0	0	0	0
hsa-miR-4481				0	0	0	0	0	0
hsa-miR-144-5p	-4.505143786		0.11179036	0	0.202011812	0.606035435	0	1.021321041	2.067108522
hsa-miR-4454	-1.27917348	0.3561974	0.15866172	35.75609065	51.86029382	70.2896854	62.01325565	65.6634755	70.18162179
hsa-miR-4534				0	0	0	0	0	0
hsa-miR-8069				0	0	0	0	0	0
hsa-miR-145-3p	-1.042436207	0.96612968	0.93471504	1.58090762	1.690859287	1.818106304	0	1.663780921	2.924234241
hsa-miR-4722-5p				0	0	0	0	0	0
hsa-miR-657	3.543660986		0.51087004	0	0.175656402	0.526969207	0	0	0
hsa-miR-4252				0	0	0	0	0	0
hsa-miR-3194-5p	-1.972268678		0.53263922	0	0.278927323	0.836781969	0	0.657199783	0.996854601
hsa-miR-10396b-3p	-3.835816219		0.44917283	0	0	0	0	0.172259043	0.51677713
hsa-miR-10396a-3p				0	0	0	0	0	0
hsa-miR-4312				0	0	0	0	0	0
hsa-miR-1273c	1.608680705	0.68321525	0.48373635	0.526969207	1.416461994	2.510345907	0	0.822088875	1.949489494
hsa-miR-4783-5p				0	0	0	0	0	0
hsa-miR-658				0	0	0	0	0	0
hsa-miR-3960				0	0	0	0	0	0
hsa-miR-8072				0	0	0	0	0	0
hsa-miR-10396a-5p				0	0	0	0	0	0
hsa-miR-187-5p				0	0	0	0	0	0
hsa-miR-621				0	0	0	0	0	0
hsa-miR-7158-5p				0	0	0	0	0	0
hsa-miR-6847-3p	-1.445554337	0.76832627	0.5678202	0.526969207	0.858607348	1.212070869	0.51677713	1.486658609	1.993709202
hsa-miR-4268				0	0	0	0	0	0
hsa-miR-4448				0	0	0	0	0	0
hsa-miR-8077				0	0	0	0	0	0
hsa-miR-3135b				0	0	0	0	0	0
hsa-miR-5589-5p				0	0	0	0	0	0
hsa-miR-6131				0	0	0	0	0	0
hsa-miR-103a-1-5p				0	0	0	0	0	0
hsa-miR-4327				0	0	0	0	0	0
hsa-miR-3160-5p				0	0	0	0	0	0
hsa-miR-6124				0	0	0	0	0	0
hsa-miR-6077				0	0	0	0	0	0
hsa-miR-3619-3p				0	0	0	0	0	0
hsa-miR-711				0	0	0	0	0	0
hsa-miR-6503-3p				0	0	0	0	0	0
hsa-miR-4516	6.046179412		0.32986518	0	0.377668214	0.606035435	0	0	0
hsa-miR-505-5p	1.367601974	0.38637578	0.18254699	10.8781656	19.48812018	25.45348826	10.85231974	15.10738118	18.52015019
hsa-miR-3689d				0	0	0	0	0	0
hsa-miR-4294				0	0	0	0	0	0
hsa-miR-504-3p				0	0	0	0	0	0
hsa-miR-3201	3.544185779		0.5108146	0	0.278927323	0.836781969	0	0	0
hsa-miR-1193				0	0	0	0	0	0
hsa-miR-4320				0	0	0	0	0	0
hsa-miR-6744-3p				0	0	0	0	0	0
hsa-miR-6069				0	0	0	0	0	0
hsa-miR-4284	11.05328531		0.18077106	0.526969207	0.93552286	1.673563938	0	0	0
hsa-miR-5787				0	0	0	0	0	0
hsa-miR-3153				0	0	0	0	0	0
hsa-miR-8059				0	0	0	0	0	0
hsa-miR-6090				0	0	0	0	0	0
hsa-miR-920				0	0	0	0	0	0
hsa-miR-6799-5p	-3.835816219		0.44917283	0	0	0	0	0.172259043	0.51677713
hsa-miR-2861				0	0	0	0	0	0
hsa-miR-3178				0	0	0	0	0	0
hsa-miR-4492				0	0	0	0	0	0
hsa-miR-762				0	0	0	0	0	0
hsa-miR-4675				0	0	0	0	0	0
hsa-miR-4271				0	0	0	0	0	0
hsa-miR-128-2-5p				0	0	0	0	0	0
hsa-miR-4525				0	0	0	0	0	0
hsa-miR-328-5p				0	0	0	0	0	0
hsa-miR-6752-5p				0	0	0	0	0	0
hsa-miR-615-5p	3.543660986		0.51087004	0	0.175656402	0.526969207	0	0	0
hsa-miR-503-3p	1.019508595	0.96612968	0.93381631	8.484496086	10.59758873	11.71494757	9.968546008	10.34209361	10.72219222
hsa-miR-4483	6.047074529		0.32982105	0	0.480939135	0.836781969	0	0	0
hsa-miR-23a-5p	1.581931679		0.57156453	0	0.90916745	1.673563938	0	0.344518087	1.033554261
hsa-miR-4281				0	0	0	0	0	0
hsa-miR-4710				0	0	0	0	0	0
hsa-miR-4466				0	0	0	0	0	0
hsa-miR-92a-2-5p	-3.836695655		0.44908316	0	0	0	0	0.324914916	0.974744747
hsa-miR-5087	3.543845864		0.51085051	0	0.202011812	0.606035435	0	0	0
hsa-miR-376a-2-5p	-3.836717681		0.44908091	0	0	0	0	0.332284867	0.996854601
hsa-miR-6073				0	0	0	0	0	0
hsa-miR-3713				0	0	0	0	0	0
hsa-miR-198				0	0	0	0	0	0
hsa-miR-489-5p				0	0	0	0	0	0
hsa-miR-8078				0	0	0	0	0	0
hsa-miR-4316				0	0	0	0	0	0
hsa-miR-143-5p	2.444797175		0.26424265	0	1.519732915	3.347127876	0	0.497173959	0.974744747
hsa-miR-376c-5p				0	0	0	0	0	0
hsa-miR-663b				0	0	0	0	0	0
hsa-miR-4769-5p				0	0	0	0	0	0
hsa-miR-3151-5p				0	0	0	0	0	0
hsa-miR-4447				0	0	0	0	0	0
hsa-miR-4472				0	0	0	0	0	0
hsa-miR-642b-5p	-1.127020076		0.92111946	0	0.175656402	0.526969207	0	0.172259043	0.51677713
hsa-miR-2116-5p	-1.895337792	0.39013864	0.18581321	1.212070869	1.664503878	2.107876826	1.993709202	3.170042701	3.898978988
hsa-miR-3147				0	0	0	0	0	0

hsa-miR-6792-5p				0	0	0	0	0	0
hsa-miR-6892-5p				0	0	0	0	0	0
hsa-miR-4724-3p				0	0	0	0	0	0
hsa-miR-877-5p	-1.079085607	0.91009337	0.80114788	5.269692065	9.240289707	15.7569213	7.797957977	10.1797112	13.95596441
hsa-miR-376a-5p	-3.836717681		0.44908091	0	0	0	0	0.332284867	0.996854601
hsa-miR-5682	-3.835816219		0.44917283	0	0	0	0	0.172259043	0.51677713
hsa-miR-6824-5p				0	0	0	0	0	0
hsa-miR-6789-5p				0	0	0	0	0	0
hsa-miR-6800-5p				0	0	0	0	0	0
hsa-miR-4789-5p				0	0	0	0	0	0
hsa-miR-4420				0	0	0	0	0	0
hsa-miR-485-3p	-3.136179324		0.13141714	0	0.351312804	1.053938413	0	1.353605908	2.067108522
hsa-miR-4436b-5p				0	0	0	0	0	0
hsa-miR-145-5p	-1.301686975	0.38637578	0.18275293	14.75513778	16.63419558	17.57502761	14.95281901	21.46942634	28.26759767
hsa-miR-642a-5p	-5.16219488	7.024E-09	4.512E-10	5.269692065	5.527161587	5.857473784	23.25497087	30.10381952	34.88991103
hsa-miR-670-5p				0	0	0	0	0	0
hsa-miR-572				0	0	0	0	0	0
hsa-miR-5571-3p				0	0	0	0	0	0
hsa-miR-4691-5p				0	0	0	0	0	0
hsa-miR-11181-5p				0	0	0	0	0	0
hsa-miR-203a-3p	11.96932635	0.00235	0.0003156	4.848283478	7.143484676	9.20460166	0	0.50454391	0.996854601
hsa-miR-887-3p	-7.36214747	0.000205	0.00002154	0.526969207	1.214450183	2.510345907	7.974836806	8.338657872	8.772702724
hsa-miR-6126				0	0	0	0	0	0
hsa-miR-183-3p	1.537532429	0.19034956	0.06207092	19.39313391	19.93684793	20.91954923	8.772702724	12.87955307	16.94652821
hsa-miR-489-3p				0	0	0	0	0	0
hsa-miR-4439				0	0	0	0	0	0
hsa-miR-6864-3p				0	0	0	0	0	0
hsa-miR-6807-5p	-1.155905029		0.8749476	0	0.351312804	1.053938413	0	0.497173959	0.974744747
hsa-miR-1224-5p				0	0	0	0	0	0
hsa-miR-6858-5p				0	0	0	0	0	0
hsa-miR-6765-5p				0	0	0	0	0	0
hsa-miR-6834-5p				0	0	0	0	0	0
hsa-miR-1226-5p	6.045864117		0.32988072	0	0.351312804	1.053938413	0	0	0
hsa-miR-6869-5p	1.513979025		0.70089864	0	0.404023623	1.212070869	0	0.172259043	0.51677713
hsa-miR-937-5p	3.543660986		0.51087004	0	0.175656402	0.526969207	0	0	0
hsa-miR-627-5p	-1.261721646	0.6018896	0.39188724	8.367819691	11.65129855	13.938815	6.977982205	14.37578812	18.6039767
hsa-miR-1238-5p				0	0	0	0	0	0
hsa-miR-4758-5p				0	0	0	0	0	0
hsa-miR-4658	3.543845864		0.51085051	0	0.202011812	0.606035435	0	0	0
hsa-miR-6790-5p				0	0	0	0	0	0
hsa-miR-10395-5p				0	0	0	0	0	0
hsa-miR-548aw	6.046179412		0.32986518	0	0.377668214	0.606035435	0	0	0
hsa-miR-5011-3p				0	0	0	0	0	0
hsa-miR-33b-5p	-1.134840385	0.83328558	0.6723213	7.531037722	8.553037327	9.696566955	2.924234241	9.736483487	15.94967361
hsa-miR-33a-5p	1.311940336	0.37906072	0.1743126	35.30693684	50.44699028	66.66389782	27.29285292	38.01565239	43.86160243
hsa-miR-4715-3p				0	0	0	0	0	0
hsa-miR-1202				0	0	0	0	0	0
hsa-miR-6850-5p				0	0	0	0	0	0
hsa-miR-6821-5p				0	0	0	0	0	0
hsa-miR-3977				0	0	0	0	0	0
hsa-miR-3978				0	0	0	0	0	0
hsa-miR-4776-5p				0	0	0	0	0	0
hsa-miR-4535				0	0	0	0	0	0
hsa-miR-3198	8.548681411		0.23675288	0	0.579680025	1.212070869	0	0	0
hsa-miR-8057				0	0	0	0	0	0
hsa-miR-647				0	0	0	0	0	0
hsa-miR-5089-5p				0	0	0	0	0	0
hsa-miR-1228-5p				0	0	0	0	0	0
hsa-miR-3620-5p	3.543660986		0.51087004	0	0.175656402	0.526969207	0	0	0
hsa-miR-1227-5p				0	0	0	0	0	0
hsa-miR-1275	-1.521341633	0.15124013	0.04327455	12.72674413	17.02270699	21.60573747	18.94023741	25.26900193	31.52340496
hsa-miR-1225-5p				0	0	0	0	0	0
hsa-miR-1229-5p				0	0	0	0	0	0
hsa-miR-6741-5p				0	0	0	0	0	0
hsa-miR-3940-5p	3.543660986		0.51087004	0	0.175656402	0.526969207	0	0	0
hsa-miR-6848-3p				0	0	0	0	0	0
hsa-miR-487a-5p				0	0	0	0	0	0
hsa-miR-487b-5p				0	0	0	0	0	0
hsa-miR-632				0	0	0	0	0	0
hsa-miR-1231				0	0	0	0	0	0
hsa-miR-511-5p				0	0	0	0	0	0
hsa-miR-6808-3p	1.513474153		0.70111189	0	0.351312804	1.053938413	0	0.324914916	0.974744747
hsa-miR-147b-3p	1.812678312	0.33928192	0.14859062	3.688784446	4.798180569	5.857473784	1.550331391	2.813291394	3.898978988
hsa-miR-6833-5p				0	0	0	0	0	0
hsa-miR-7108-5p				0	0	0	0	0	0
hsa-miR-147a				0	0	0	0	0	0
hsa-miR-2053				0	0	0	0	0	0
hsa-miR-4273				0	0	0	0	0	0
hsa-miR-653-5p				0	0	0	0	0	0
hsa-miR-2909				0	0	0	0	0	0
hsa-miR-607	-6.712040629		0.25810652	0	0	0	0	0.497173959	0.974744747
hsa-miR-3692-3p				0	0	0	0	0	0
hsa-miR-3074-5p	-1.337656502	0.2998406	0.12256258	14.22529347	15.86979909	17.57502761	19.12075383	22.16111929	25.91821962
hsa-miR-629-3p	1.505594594	0.54326907	0.32202996	2.510345907	4.0864949	6.060354347	1.993709202	2.672868742	3.100662783
hsa-miR-4652-3p				0	0	0	0	0	0
hsa-miR-3122	-1.12728494		0.9209602	0	0.175656402	0.526969207	0	0.332284867	0.996854601
hsa-miR-5572				0	0	0	0	0	0
hsa-miR-599	-12.54871164	5.767E-31	1.01E-32	9.485445718	11.3090458	12.72674413	104.2976879	140.6906809	176.2210015
hsa-miR-552-5p				0	0	0	0	0	0
hsa-miR-557				0	0	0	0	0	0
hsa-miR-7850-5p				0	0	0	0	0	0
hsa-miR-2681-5p				0	0	0	0	0	0
hsa-miR-548x-3p	2.845555639	6.254E-10	3.652E-11	118.5680715	137.0012413	159.3873193	41.91402413	48.25486688	55.82385764
hsa-miR-548aj-3p	18.55933571		0.09914778	0.836781969	1.438287374	2.424141739	0	0	0
hsa-miR-548as-3p	-2.818039139		0.32211266	0	0.175656402	0.526969207	0	0.98948465	1.993709202
hsa-miR-548ar-3p	1.357798994		0.66113055	0	0.957348239	1.818106304	0	0.676802954	1.033554261
hsa-miR-5582-3p	1.549484407	0.36825655	0.16773145	6.694255753	8.397055588	10.01241492	3.898978988	5.520503209	6.977982205
hsa-miR-4282				0	0	0	0	0	0
hsa-miR-3133	1.918601423	0.52446598	0.29783543	0.606035435	2.165117675	4.215753652	0	1.321769516	2.990563802
hsa-miR-7161-5p				0	0	0	0	0	0
hsa-miR-1276	1.365306997	0.59949783	0.3894548	2.424141739	4.971686255	6.694255753	3.100662783	3.662353391	3.987418403
hsa-miR-12135	-3.146277564	7.748E-19	2.036E-20	333.8760057	386.8138636	418.1644499	1130.703907	1219.183725	1356.539967
hsa-miR-559				0	0	0	0	0	0
hsa-miR-106b-5p	-1.232946937	0.25882881	0.1015311	186.5470991	231.341163	271.5038747	229.0650156	284.3434647	316.999763
hsa-miR-20a-5p	-1.351727051	0.03388766	0.006184	7304.320172	7755.242326	8512.373716	10067.16375	10484.87899	11169.7558
hsa-miR-4796-3p	8.549500881		0.23672994	0.526969207	0.656595537	0.836781969	0	0	0
hsa-miR-3121-3p	8.549500881		0.23672994	0.526969207	0.656595537	0.836781969	0	0	0
hsa-miR-605-5p	1.585923451	0.43044836	0.22245069	4.183909846	5.996889945	8.958476511	1.949489494	3.381508087	6.201325565
hsa-miR-513c-3p				0	0	0	0	0	0
hsa-miR-513a-3p				0	0	0	0	0	0
hsa-miR-5688				0	0	0	0	0	0
hsa-miR-141-3p	1.127978569	0.65481771	0.44638227	55.33176669	59.70919498	66.05786238	43.86351362	52.00535949	56.32870722
hsa-miR-200a-3p	1.120222919	0.76832627	0.56786166	18.40920332	21.56808705	23.63538195	11.69693697	18.14854068	24.80530226

hsa-miR-132-3p	1.016546829	0.95639406	0.90254815	8397.833019	12085.44882	15931.86002	10083.35537	11889.46756	14204.9552
hsa-miR-212-3p	1.303372258	0.09884543	0.02318725	318.7746387	339.9827271	367.297537	229.965823	264.1863179	291.4486794
hsa-miR-4803				0	0	0	0	0	0
hsa-miR-6732-3p				0	0	0	0	0	0
hsa-miR-3912-3p	-1.183113672	0.8800976	0.74776175	1.053938413	1.592118397	2.510345907	0.974744747	1.838546647	2.990563802
hsa-miR-4485-3p	6.446480719	0.0008407	0.00009941	7.878460651	10.56061872	15.89885741	0	1.338866005	2.067108522
hsa-miR-582-3p	9.128175937	1.197E-59	8.738E-62	589.9312882	608.7849276	639.3673836	57.50994008	65.21704733	72.34879826
hsa-miR-12120				0	0	0	0	0	0
hsa-miR-6891-5p				0	0	0	0	0	0
hsa-miR-506-3p				0	0	0	0	0	0
hsa-miR-124-3p				0	0	0	0	0	0
hsa-miR-3936				0	0	0	0	0	0
hsa-miR-18a-5p	-1.260527109	0.21209007	0.07338007	1288.43971	1595.517805	1878.103812	1582.985469	2010.169104	2247.46374
hsa-miR-18b-5p	-2.379949184	0.008938	0.001279	6.323630479	8.283170065	10.04138363	10.72219222	19.10383235	25.91821962
hsa-miR-302e				0	0	0	0	0	0
hsa-miR-302c-3p				0	0	0	0	0	0
hsa-miR-302d-3p				0	0	0	0	0	0
hsa-miR-302b-3p	-4.505143786		0.11179036	0	0.202011812	0.606035435	0	1.021321041	2.067108522
hsa-miR-302a-3p	-3.835816219		0.44917283	0	0	0	0	0.172259043	0.51677713
hsa-miR-200c-3p	1.309370685	0.120468	0.03046915	136.9640082	146.0278915	163.8874232	100.7715404	114.0201024	129.6410514
hsa-miR-200b-3p	-1.223587187	0.66977671	0.46346593	6.694255753	8.247754595	9.090531521	4.984273004	10.15258798	14.62117121
hsa-miR-429	-1.327037963	0.67457881	0.4697432	2.510345907	3.884483089	5.454318912	2.924234241	5.663432544	10.96540061
hsa-miR-500a-5p	1.250638174	0.45222259	0.23832461	47.87679934	67.40119628	89.05779591	44.85845703	54.36678399	70.18162179
hsa-miR-216a-5p	2.140705583		0.4564663	0.526969207	0.656595537	0.836781969	0	0.172259043	0.51677713
hsa-miR-580-5p				0	0	0	0	0	0
hsa-miR-5700				0	0	0	0	0	0
hsa-miR-365a-3p	1.126654715	0.55956053	0.34063758	502.0691815	550.7508951	596.3388678	456.5594071	490.691425	530.2611424
hsa-miR-302f				0	0	0	0	0	0
hsa-miR-590-3p	1.121849954	0.53977044	0.31638869	2272.291219	2556.206254	2967.149488	2002.125711	2277.277524	2497.067094
hsa-miR-6882-5p	-1.266799339	0.75962047	0.55024625	2.634846033	3.282977684	4.183909846	1.993709202	3.686819831	5.167771304
hsa-miR-4801				0	0	0	0	0	0
hsa-miR-5683				0	0	0	0	0	0
hsa-miR-6856-3p	6.045864117		0.32988072	0	0.351312804	1.053938413	0	0	0
hsa-miR-101-3p	-1.118417993	0.56627984	0.35795499	2048.856275	2518.620656	2913.81837	2260.433069	2815.207572	3444.836351
hsa-miR-144-3p	-6.710501692		0.25817211	0	0	0	0	0.344518087	1.033554261
hsa-miR-4802-3p				0	0	0	0	0	0
hsa-miR-140-3p	1.255724854	0.20878245	0.07193089	504.2214817	583.1820396	648.6990933	411.7009501	464.4520913	517.5894607
hsa-miR-6512-5p				0	0	0	0	0	0
hsa-miR-1912-3p				0	0	0	0	0	0
hsa-miR-3130-5p	1.107304627		0.89137262	0	0.728981018	1.58090762	0	0.51677713	1.550331391
hsa-miR-660-5p	-1.182786412	0.36696807	0.16607314	697.0393803	719.9064587	757.2547498	750.6315144	849.1807831	924.5142864
hsa-miR-10b-5p	-1.38540027	0.14081714	0.03803091	72.72425216	85.70575171	109.0826258	111.6238602	120.2368616	137.4390093
hsa-miR-10a-5p	-1.691660268	0.0003196	0.00003546	9906.861251	12594.37253	16162.14556	19243.23001	21306.78082	24782.8852
hsa-miR-7851-3p	-1.174021061		0.81288541	0.526969207	0.93552286	1.673563938	0	0.994347918	1.949489494
hsa-miR-3939	1.044113542	0.96770494	0.94736595	0.606035435	1.111179262	1.673563938	0	1.307029614	2.924234241
hsa-miR-433-5p				0	0	0	0	0	0
hsa-miR-1277-3p	1.15906882	0.82772881	0.65493287	4.183909846	5.144963343	5.796661272	1.949489494	4.206103644	5.684548435
hsa-miR-598-3p	-1.155546808	0.4035061	0.20183081	225.9311317	242.2484202	259.9892015	278.1224336	280.7005326	284.2274217
hsa-miR-4637				0	0	0	0	0	0
hsa-miR-888-5p	13.55493457		0.14386816	0.526969207	1.06061916	1.818106304	0	0	0
hsa-miR-510-5p	3.543845864		0.51085051	0	0.202011812	0.606035435	0	0	0
hsa-miR-508-5p				0	0	0	0	0	0
hsa-miR-514a-5p				0	0	0	0	0	0
hsa-miR-509-5p				0	0	0	0	0	0
hsa-miR-509-3-5p				0	0	0	0	0	0
hsa-miR-217-5p	1.00492908	0.99392903	0.99392903	0	1.159360051	2.424141739	0.51677713	1.479288657	2.924234241
hsa-miR-9898				0	0	0	0	0	0
hsa-miR-890				0	0	0	0	0	0
hsa-miR-5003-3p	-3.835816219		0.44917283	0	0	0	0	0.172259043	0.51677713
hsa-miR-6502-3p				0	0	0	0	0	0
hsa-miR-6863				0	0	0	0	0	0
hsa-miR-6758-5p				0	0	0	0	0	0
hsa-miR-3125	3.543660986		0.51087004	0	0.175656402	0.526969207	0	0	0
hsa-miR-12127				0	0	0	0	0	0
hsa-miR-5007-5p				0	0	0	0	0	0
hsa-miR-577				0	0	0	0	0	0
hsa-miR-7161-3p				0	0	0	0	0	0
hsa-miR-548p	-3.835816219		0.44917283	0	0	0	0	0.172259043	0.51677713
hsa-miR-451b	3.543660986		0.51087004	0	0.175656402	0.526969207	0	0	0
hsa-miR-4703-5p				0	0	0	0	0	0
hsa-miR-29a-3p	2.479090979	1.939E-17	6.226E-19	34498.01024	36418.17604	37751.1593	13073.74809	14686.87849	16175.64096
hsa-miR-29b-3p	2.304765837	4.036E-09	2.534E-10	21265.84233	30015.26982	38201.44363	10120.77471	13022.50842	14797.39635
hsa-miR-29c-3p	1.215798284	0.26297932	0.10405114	1089.772319	1179.127547	1322.975354	860.6996117	967.5741801	1107.970168
hsa-miR-195-5p	-1.044838852	0.86787749	0.71710753	328.0185319	359.3994695	376.2560135	364.327877	380.6600352	390.8726436
hsa-miR-15a-5p	1.125555007	0.55502392	0.33382461	903.7245266	965.8406969	1044.199054	845.3327014	858.1160066	879.0378989
hsa-miR-15b-5p	1.094308116	0.6029521	0.39345926	2523.128561	2637.55915	2787.156964	2368.629735	2410.479056	2458.243445
hsa-miR-16-5p	1.105209446	0.53204552	0.30757668	113817.4441	117534.1181	122191.8945	104601.8083	106342.2612	108493.2243
hsa-miR-503-5p	1.642393348	0.001123	0.0001394	216.72653	262.7014875	336.3496663	143.5470625	159.8712313	168.6308412
hsa-miR-3943				0	0	0	0	0	0
hsa-miR-3614-3p	13.55352259		0.14406764	0.606035435	1.007908341	1.58090762	0	0	0
hsa-miR-4781-5p	3.543845864		0.51085051	0	0.202011812	0.606035435	0	0	0
hsa-miR-21-5p	-1.581454429	0.0001945	0.00002016	91507.71443	107976.6161	123268.1098	157101.2812	170762.0284	179327.6901
hsa-miR-1197				0	0	0	0	0	0
hsa-miR-4759				0	0	0	0	0	0
hsa-miR-3169				0	0	0	0	0	0
hsa-miR-3139	4.444202248	0.13756065	0.0369506	0.836781969	2.342924794	3.161815239	0	0.657199783	0.996854601
hsa-miR-4779				0	0	0	0	0	0
hsa-miR-2392				0	0	0	0	0	0
hsa-miR-3159	-1.174296674		0.81260589	0.526969207	0.93552286	1.673563938	0.51677713	1.154373742	1.949489494
hsa-miR-5582-5p				0	0	0	0	0	0
hsa-miR-449c-5p	3.681565715	0.13025557	0.03422774	0.606035435	5.316318312	9.485445718	0	1.471918706	3.898978988
hsa-miR-34b-5p	-1.504803894	0.61606818	0.41101191	0.526969207	1.695389317	3.347127876	0	2.008299008	3.100662783
hsa-miR-4717-5p				0	0	0	0	0	0
hsa-miR-1245b-5p	6.046759143		0.32983659	0	0.454583725	0.836781969	0	0	0
hsa-miR-6757-5p				0	0	0	0	0	0
hsa-miR-6783-5p	-1.021533882	0.9772691	0.96870908	1.053938413	1.515202885	1.818106304	0.974744747	1.346235957	2.067108522
hsa-miR-4688				0	0	0	0	0	0
hsa-miR-6805-5p	-1.126961281		0.92115277	0	0.202011812	0.606035435	0	0.172259043	0.51677713
hsa-miR-6732-5p				0	0	0	0	0	0
hsa-miR-6782-5p				0	0	0	0	0	0
hsa-miR-6841-5p				0	0	0	0	0	0
hsa-miR-6755-5p	-3.836717681		0.44908091	0	0	0	0	0.332284867	0.996854601
hsa-miR-6831-5p	-3.836695655		0.44908316	0	0	0	0	0.324914916	0.974744747
hsa-miR-196a-5p	-1.105060899	0.57355449	0.36590264	21100.29413	22361.86272	24072.3335	23009.39789	24708.34007	27751.96546
hsa-miR-196b-5p	-2.070062228	2.094E-08	1.376E-09	3476.942825	3980.140771	4522.236414	7005.490497	8236.999866	9822.382918
hsa-miR-6865-5p	-3.836695655		0.44908316	0	0	0	0	0.324914916	0.974744747
hsa-miR-6815-5p	-3.835816219		0.44917283	0	0	0	0	0.172259043	0.51677713
hsa-miR-154-5p	-6.710501692		0.25817211	0	0	0	0	0.344518087	1.033554261
hsa-miR-624-5p	-1.051180613	0.94510719	0.88133416	3.688784446	7.828357743	13.938815	6.977982205	8.178632049	8.785211217
hsa-miR-1278	1.761156243	0.23206329	0.08333952	6.323630479	7.321291795	8.367819691	3.617439913	4.166897302	4.984273004
hsa-miR-411-5p	-7.095018679	0.02866143	0.004937	0	0.557854646	1.673563938	1.949489494	3.357041647	4.134217044
hsa-miR-2114-5p	-1.155579957		0.8749822	0	0.377668214	0.606035435	0	0.344518087	1.033554261
hsa-miR-3174	1.217155966	0.78709407	0.61004463	3.347127876	3.935043191	4.242248043	0.996854601	3.16267275	4.873723736

hsa-miR-454-3p	-1.142553653	0.45614066	0.24172126	4470.089279	4775.354913	5110.090785	5301.272767	5459.530338	5658.393257
hsa-miR-12115				0	0	0	0	0	0
hsa-miR-3681-5p	11.05165014		0.18080018	0.606035435	0.832251939	1.053938413	0	0	0
hsa-miR-6718-5p				0	0	0	0	0	0
hsa-miR-203b-5p				0	0	0	0	0	0
hsa-miR-5584-3p				0	0	0	0	0	0
hsa-miR-3163	-3.836717681		0.44908091	0	0	0	0	0.332284867	0.996854601
hsa-miR-381-3p	-3.619936868	0.14285303	0.03983201	0.526969207	0.656595537	0.836781969	1.993709202	2.653265571	3.898978988
hsa-miR-300				0	0	0	0	0	0
hsa-miR-875-5p	-17.56383322	7.944E-43	9.277E-45	6.323630479	10.93805834	13.938815	155.9591595	187.1614911	219.1135033
hsa-miR-3976				0	0	0	0	0	0
hsa-miR-135a-3p				0	0	0	0	0	0
hsa-miR-5011-5p				0	0	0	0	0	0
hsa-miR-8076				0	0	0	0	0	0
hsa-miR-2681-3p				0	0	0	0	0	0
hsa-miR-5586-5p	2.655332848	0.3955824	0.1940375	1.053938413	1.515202885	1.818106304	0	0.664569734	1.993709202
hsa-miR-4686				0	0	0	0	0	0
hsa-miR-586	1.721413712	0.6633392	0.45610622	0	1.332865736	3.161815239	0	0.836828777	1.993709202
hsa-miR-3688-3p	-1.667401684	0.51261592	0.29035763	1.053938413	1.515202885	1.818106304	2.067108522	2.660635522	2.990563802
hsa-miR-1843	1.136686697	0.68073213	0.47999068	21.81727565	25.92097255	30.12415089	21.93080122	22.36270805	22.73819374
hsa-miR-4802-5p	-6.712040629		0.25810652	0	0	0	0	0.497173959	0.974744747
hsa-miR-6514-5p	3.72059519	0.14422374	0.0406114	0.836781969	3.071905812	4.742722859	0	0.974744747	2.924234241
hsa-miR-183-5p	1.025825029	0.92338521	0.81958862	4214.033996	4626.005535	5076.294367	4085.110154	4509.897502	4737.259471
hsa-miR-135b-5p	-1.218986368	0.83328558	0.66911394	1.673563938	2.999520331	3.688784446	0	3.396247989	6.201325565
hsa-miR-135a-5p	-1.651104372		0.55345249	0	0.454583725	0.836781969	0.51677713	0.829458826	0.996854601
hsa-miR-3675-5p	4.868613606	0.0636499	0.01268516	3.347127876	3.908687781	4.742722859	0	0.51677713	1.550331391
hsa-miR-5579-5p				0	0	0	0	0	0
hsa-miR-411-3p	-3.836695655		0.44908316	0	0	0	0	0.324914916	0.974744747
hsa-miR-379-3p	-3.835816219		0.44917283	0	0	0	0	0.172259043	0.51677713
hsa-miR-380-3p				0	0	0	0	0	0
hsa-miR-7852-3p				0	0	0	0	0	0
hsa-miR-6834-3p	3.543660986		0.51087004	0	0.175656402	0.526969207	0	0	0
hsa-miR-654-3p	-13.07307142	0.00007483	7.429E-06	0	0.606035435	1.818106304	8.772702724	9.18771987	9.818765478
hsa-miR-4501				0	0	0	0	0	0
hsa-miR-455-5p	1.257066348	0.23206329	0.08319719	467.4216862	529.3102944	586.6423008	354.8070879	419.6807629	452.5719887
hsa-miR-299-3p	-6.710501692		0.25817211	0	0	0	0	0.344518087	1.033554261
hsa-miR-12132				0	0	0	0	0	0
hsa-miR-892c-5p				0	0	0	0	0	0
hsa-miR-506-5p				0	0	0	0	0	0
hsa-miR-664a-3p	1.744475811	0.10339499	0.02550913	15.15088587	20.77541303	27.92936795	8.772702724	11.88269847	13.95596441
hsa-miR-32-5p	-1.242208539	0.32250008	0.13888689	242.405835	296.1512134	351.5005521	250.5094	367.6654901	468.5216624
hsa-miR-92b-3p	1.254880043	0.4035061	0.20263664	296.3513276	438.2717983	650.2800009	270.7912163	349.866297	471.7764576
hsa-miR-92a-3p	-1.109165439	0.66759236	0.46097983	5074.94073	6960.080512	9548.682023	6116.574116	7720.855688	9678.240594
hsa-miR-1323				0	0	0	0	0	0
hsa-miR-8063				0	0	0	0	0	0
hsa-miR-4698				0	0	0	0	0	0
hsa-miR-7853-5p				0	0	0	0	0	0
hsa-miR-105-5p	4.680160767	0.0001945	0.00001997	10.53938413	13.50719964	15.7569213	0.996854601	2.512842918	3.617439913
hsa-miR-3065-5p	3.239082368	1.219E-21	2.492E-23	2446.750478	2622.102614	2925.939079	767.5780426	808.8377056	829.5077798
hsa-miR-335-5p	-2.625117232	2.89E-15	1.139E-16	677.1554304	730.4351971	829.6625101	1899.008014	1919.013093	1930.969344
hsa-miR-3922-5p				0	0	0	0	0	0
hsa-miR-5697	1.622491367	0.63327654	0.425266	0.836781969	1.587588366	2.107876826	0.51677713	1.154373742	1.949489494
hsa-miR-224-5p				0	0	0	0	0	0
hsa-miR-6513-3p	1.430526728	0.768441	0.57100215	0.526969207	1.618473806	2.510345907	0	1.009087821	1.993709202
hsa-miR-95-5p	-18.21524977		0.07005654	0	0	0	0	1.513631731	2.990563802
hsa-miR-6826-5p	-1.106252009	0.93841009	0.85398131	0	2.520731913	5.454318912	0	2.830387883	4.873723736
hsa-miR-4705				0	0	0	0	0	0
hsa-miR-4719				0	0	0	0	0	0
hsa-miR-5003-5p	3.543660986		0.51087004	0	0.175656402	0.526969207	0	0	0
hsa-miR-4457	-1.127020076		0.92111946	0	0.175656402	0.526969207	0	0.172259043	0.51677713
hsa-miR-125b-2-3p	-5.228366943	1.574E-12	7.811E-14	5.857473784	7.76681546	8.958476511	34.62406774	43.73677531	48.73723736
hsa-miR-1228-3p	-3.836695655		0.44908316	0	0	0	0	0.324914916	0.974744747
hsa-miR-128-3p	1.182728418	0.31633177	0.13253608	4485.988136	4810.132261	5335.535967	3894.710925	4066.953501	4154.362112
hsa-miR-216a-3p				0	0	0	0	0	0
hsa-miR-1226-3p	-1.315163845	0.24779999	0.09441722	50.58904383	55.19447176	60.60354347	70.77667665	72.70733698	76.03009027
hsa-miR-3620-3p	-1.18300133	0.88116134	0.74995191	0.526969207	1.464642783	3.030177174	0.974744747	1.678520823	2.067108522
hsa-miR-3622a-3p				0	0	0	0	0	0
hsa-miR-3622b-3p				0	0	0	0	0	0
hsa-miR-6765-3p	8.549500881		0.23672994	0.526969207	0.656595537	0.836781969	0	0	0
hsa-miR-3186-3p				0	0	0	0	0	0
hsa-miR-11401	-6.712040629		0.25810652	0	0	0	0	0.497173959	0.974744747
hsa-miR-4999-3p				0	0	0	0	0	0
hsa-miR-483-3p	4.020487312		0.1441511	0.526969207	1.416461994	2.510345907	0	0.332284867	0.996854601
hsa-miR-4639-3p	-3.836717681		0.44908091	0	0	0	0	0.332284867	0.996854601
hsa-miR-1208				0	0	0	0	0	0
hsa-miR-589-3p	-1.078916179	0.84399671	0.69121482	25.10345907	31.07179187	34.77996763	33.07373635	34.68377096	35.88676563
hsa-miR-1245b-3p	-3.835816219		0.44917283	0	0	0	0	0.172259043	0.51677713
hsa-miR-545-3p	1.559443359	0.56399273	0.35239254	2.107876826	3.636441205	5.454318912	0	2.350460508	4.984273004
hsa-miR-3065-3p	2.912039353	5.997E-17	2.101E-18	244.340335	254.9895298	262.4133432	77.75465886	87.99906033	99.4239642
hsa-miR-3619-5p				0	0	0	0	0	0
hsa-miR-7157-5p	3.544185779		0.5108146	0	0.278927323	0.836781969	0	0	0
hsa-miR-5690	-1.191813188	0.78927331	0.61695553	3.161815239	5.15164409	7.272425217	2.990563802	5.835541492	7.797957977
hsa-miR-6736-3p	8.550766568		0.23669451	0	0.759866458	1.673563938	0	0	0
hsa-miR-1207-3p				0	0	0	0	0	0
hsa-miR-5000-3p	2.721573948		0.37176032	0	0.702625609	2.107876826	0	0.172259043	0.51677713
hsa-miR-4742-5p	5.271134485	0.21497903	0.07532112	0	1.484317446	2.634846033	0	0.324914916	0.974744747
hsa-miR-4692				0	0	0	0	0	0
hsa-miR-1972				0	0	0	0	0	0
hsa-miR-4650-5p				0	0	0	0	0	0
hsa-miR-484	2.544170677	0.00006667	6.393E-06	61.20957891	92.50922398	125.9456404	27.90596504	36.84541961	48.73723736
hsa-miR-4270				0	0	0	0	0	0
hsa-miR-6823-5p	8.209990129		0.25904058	0	0.526969207	1.58090762	0	0	0
hsa-miR-3934-5p	2.572765652	0.22907983	0.08126482	3.161815239	3.381718575	3.636212608	0	1.166606962	1.949489494
hsa-miR-545-5p	2.225712797	0.16126346	0.04802591	3.161815239	7.633255275	10.04138363	0.996854601	3.315328622	5.848468483
hsa-miR-4704-3p				0	0	0	0	0	0
hsa-miR-148a-3p	-2.556928036	1.36E-13	6.156E-15	4026.571707	4340.372887	4518.600201	9072.924106	11095.26438	13777.79507
hsa-miR-148b-3p	1.015557308	0.95109507	0.89094691	9883.307469	10258.38144	10815.30837	9041.732274	10097.85306	11773.73336
hsa-miR-152-3p	-2.850355964	1.431E-15	5.433E-17	82.00463297	92.19722025	106.0562011	238.812463	261.9210466	299.7307357
hsa-miR-6733-3p	-1.506028673	0.61592748	0.40732138	0.836781969	1.438287374	2.424141739	1.949489494	2.335720606	2.990563802
hsa-miR-584-3p				0	0	0	0	0	0
hsa-miR-103b				0	0	0	0	0	0
hsa-miR-1279				0	0	0	0	0	0
hsa-miR-6729-3p	-3.836717681		0.44908091	0	0	0	0	0.332284867	0.996854601
hsa-miR-5196-3p				0	0	0	0	0	0
hsa-miR-4528				0	0	0	0	0	0
hsa-miR-4729	-3.836717681		0.44908091	0	0	0	0	0.332284867	0.996854601
hsa-miR-153-5p				0	0	0	0	0	0
hsa-miR-6777-3p	-1.155733934		0.87485783	0	0.454583725	0.836781969	0	0.497173959	0.974744747
hsa-miR-338-3p	1.161913729	0.47887536	0.26285713	288.7791252	356.8738562	395.7978714	242.711442	305.3170548	375.1801967
hsa-miR-4267				0	0	0	0	0	0
hsa-miR-4673	13.55552724		0.14386093	0.606035435	1.111179262	1.673563938	0	0	0
hsa-miR-7113-5p	-3.835816219		0.44917283	0	0	0	0	0.172259043	0.51677713
hsa-miR-4712-5p				0	0	0	0	0	0

hsa-miR-770-5p				0	0	0	0	0	0
hsa-miR-1825				0	0	0	0	0	0
hsa-miR-4716-5p				0	0	0	0	0	0
hsa-miR-885-5p				0	0	0	0	0	0
hsa-miR-6814-5p				0	0	0	0	0	0
hsa-miR-662				0	0	0	0	0	0
hsa-miR-4301				0	0	0	0	0	0
hsa-miR-6764-5p	1.107044721		0.89075196	0	0.728981018	1.58090762	0	0.676802954	1.033554261
hsa-miR-6793-3p	-1.12728494		0.9209602	0	0.175656402	0.526969207	0	0.332284867	0.996854601
hsa-miR-361-3p	1.175881676	0.39076896	0.18899474	335.1524154	349.4494217	375.1359341	277.125579	296.3170208	308.9940848
hsa-miR-6887-3p				0	0	0	0	0	0
hsa-miR-6810-3p	3.543660986		0.51087004	0	0.175656402	0.526969207	0	0	0
hsa-miR-6756-3p				0	0	0	0	0	0
hsa-miR-3127-3p	-1.368004705	0.76832627	0.56358534	0.526969207	1.262630971	2.424141739	1.033554261	1.983832568	2.924234241
hsa-miR-3162-3p				0	0	0	0	0	0
hsa-miR-4667-3p	-3.835816219		0.44917283	0	0	0	0	0.172259043	0.51677713
hsa-miR-6803-3p	1.295703199		0.71687864	0.526969207	1.06061916	1.818106304	0	0.974744747	2.924234241
hsa-miR-6892-3p				0	0	0	0	0	0
hsa-miR-125b-5p	-6.69490467	9.28E-68	4.064E-70	597.4623259	633.9552274	663.608801	4109.034664	4255.464185	4362.632535
hsa-miR-125a-5p	-1.115906059	0.53977044	0.31733909	10125.89861	10331.7785	10703.79852	10795.47425	11534.50781	12561.53556
hsa-miR-4319				0	0	0	0	0	0
hsa-miR-6752-3p	6.045864117		0.32988072	0	0.351312804	1.053938413	0	0	0
hsa-miR-4308				0	0	0	0	0	0
hsa-miR-339-5p	1.506140369	0.005917	0.0008292	1146.391298	1270.245715	1436.518057	759.6032058	844.8976973	994.239642
hsa-miR-5088-3p	-7.100074952		0.25151592	0	0	0	0	0.649829831	1.949489494
hsa-miR-6780b-3p	-2.817393593		0.32785328	0	0.175656402	0.526969207	0	0.836828777	1.993709202
hsa-miR-6716-3p	-1.357239544	0.76832627	0.5686736	0.606035435	1.813804871	3.161815239	0.974744747	2.207531174	4.650994174
hsa-miR-615-3p	-1.351141298	0.09884543	0.02337658	218.4000939	267.7821123	303.0177174	320.9871814	363.6100524	430.8371782
hsa-miR-671-3p	2.349603953	0.0002624	0.00002835	21.81727565	26.97491097	30.12415089	9.968546008	11.33644153	12.67168171
hsa-miR-3678-5p				0	0	0	0	0	0
hsa-miR-340-3p	-1.032334752	0.94510719	0.87698618	24.2405835	30.94468384	41.81644499	28.90878342	31.44184166	36.17439913
hsa-miR-5009-3p				0	0	0	0	0	0
hsa-miR-6761-3p	6.047074529		0.32982105	0	0.480939135	0.836781969	0	0	0
hsa-miR-6072				0	0	0	0	0	0
hsa-miR-5193	-3.836695655		0.44908316	0	0	0	0	0.324914916	0.974744747
hsa-miR-877-3p	-1.356829758	0.67195313	0.46693385	1.673563938	2.77115311	4.215753652	2.583885652	4.464839095	6.82321323
hsa-miR-6727-3p				0	0	0	0	0	0
hsa-miR-6747-3p	1.016566297	0.96770494	0.94792703	7.878460651	9.38313855	11.06635334	8.971691407	9.340375742	9.747447471
hsa-miR-1539				0	0	0	0	0	0
hsa-miR-3193	1.765898025	0.56165999	0.34742405	0.606035435	1.813804871	3.161815239	0	1.169113644	2.990563802
hsa-miR-486-5p	-3.867023923	8.396E-08	5.884E-09	11.71494757	17.43066447	28.45633715	57.36226148	69.2601931	92.60075097
hsa-miR-6748-3p	-9.586701267		0.16940445	0	0	0	0	0.669433003	1.033554261
hsa-miR-5699-3p	2.29737607	0.27035609	0.10803941	0.836781969	3.300273033	4.848283478	0.974744747	1.50626178	1.993709202
hsa-miR-205-5p	-1.174252841		0.81266788	0	0.781691837	1.818106304	0.974744747	1.00171787	1.033554261
hsa-miR-1237-3p	11.55183084		0.18650024	0	1.012438371	2.510345907	0	0	0
hsa-miR-10397-5p	1.358129929		0.66086274	0.526969207	1.137534671	1.673563938	0	0.676802954	1.033554261
hsa-miR-3121-5p				0	0	0	0	0	0
hsa-miR-196b-3p	-1.138966364	0.86978632	0.72314445	4.742722859	5.909359831	7.531037722	0.996854601	6.051870146	10.33554261
hsa-miR-1307-5p	1.076465374	0.75962047	0.55114069	1017.577538	1172.581046	1398.123748	907.4873596	1088.237562	1225.795353
hsa-miR-3131	-1.167655692		0.84072394	0	0.733511048	1.673563938	0.51677713	0.829458826	0.996854601
hsa-miR-151b	-3.835816219		0.44917283	0	0	0	0	0.172259043	0.51677713
hsa-miR-151a-5p	1.103517265	0.64702639	0.43827773	876.3497905	1072.465703	1301.764114	832.432014	970.5552926	1131.225139
hsa-miR-6767-5p	-6.710501692		0.25817211	0	0	0	0	0.344518087	1.033554261
hsa-miR-1281				0	0	0	0	0	0
hsa-miR-1292-3p	3.543660986		0.51087004	0	0.175656402	0.526969207	0	0	0
hsa-miR-579-5p	-1.5178505		0.5649257	0.526969207	0.656595537	0.836781969	0.974744747	1.00171787	1.033554261
hsa-miR-127-3p	-14.03960465	0.001172	0.0001506	0	0.404023623	1.212070869	5.684548435	7.469992704	9.747447471
hsa-miR-1234-3p	6.045864117		0.32988072	0	0.351312804	1.053938413	0	0	0
hsa-miR-7107-5p				0	0	0	0	0	0
hsa-miR-6741-3p	-7.100074952		0.25145555	0	0	0	0	0.649829831	1.949489494
hsa-miR-11400				0	0	0	0	0	0
hsa-miR-6749-5p				0	0	0	0	0	0
hsa-miR-6499-5p				0	0	0	0	0	0
hsa-miR-6889-5p				0	0	0	0	0	0
hsa-miR-542-5p	1.23139772	0.57873542	0.37052323	13.70119937	18.58192821	23.63538195	12.67168171	15.18830055	18.94023741
hsa-miR-6775-5p				0	0	0	0	0	0
hsa-miR-101-2-5p	-3.072001274	0.15662579	0.04618746	0	0.755336428	1.212070869	2.067108522	2.985550437	3.898978988
hsa-miR-126-3p	1.066000584	0.77631463	0.59385236	6297.808987	6899.899191	7234.85102	5602.832806	6469.728733	7816.254098
hsa-miR-6506-3p				0	0	0	0	0	0
hsa-miR-4536-3p	-3.836717681		0.44908091	0	0	0	0	0.332284867	0.996854601
hsa-miR-1204				0	0	0	0	0	0
hsa-miR-6774-3p				0	0	0	0	0	0
hsa-miR-187-3p	-2.200227981	0.0194353	0.003093	5.796661272	9.502150278	14.22529347	15.59591595	20.3594224	27.91192882
hsa-miR-1282				0	0	0	0	0	0
hsa-miR-4744	8.549946618		0.23671746	0	0.682950946	1.212070869	0	0	0
hsa-miR-1283	21.13906577	2.589E-13	1.209E-14	36.36087525	39.32252195	41.00231649	0.996854601	1.823806744	2.924234241
hsa-miR-139-5p	-1.955157601	0.15481397	0.04520116	1.673563938	3.93051316	5.269692065	7.234879826	8.658709519	9.968546008
hsa-miR-6508-5p				0	0	0	0	0	0
hsa-miR-520g-5p				0	0	0	0	0	0
hsa-miR-645				0	0	0	0	0	0
hsa-miR-628-3p	-1.464465565	0.18906832	0.06017065	12.12029175	14.34183089	17.57242135	19.93709201	20.51192818	21.44438444
hsa-miR-6080				0	0	0	0	0	0
hsa-miR-1284				0	0	0	0	0	0
hsa-miR-6731-3p				0	0	0	0	0	0
hsa-miR-342-3p	1.255144424	0.16947325	0.0517079	2523.53155	2963.873255	3485.374332	2113.101686	2362.443794	2859.901088
hsa-miR-6784-3p	-3.835816219		0.44917283	0	0	0	0	0.172259043	0.51677713
hsa-miR-1304-3p	1.17162448	0.46450801	0.25090214	98.74027235	120.3830097	133.3232093	102.6760239	104.4373939	106.2471774
hsa-miR-6787-3p	6.046179412		0.32986518	0	0.377668214	0.606035435	0	0	0
hsa-miR-3664-3p				0	0	0	0	0	0
hsa-miR-4797-3p	2.766302339		0.30359422	0	0.755336428	1.212070869	0	0.332284867	0.996854601
hsa-miR-150-5p	-1.345834308	0.67195313	0.46692986	2.107876826	3.232417582	4.242248043	2.924234241	4.666577943	7.974836806
hsa-miR-4685-3p	-2.016252951		0.53608045	0	0.278927323	0.836781969	0	0.649829831	1.949489494
hsa-miR-4287				0	0	0	0	0	0
hsa-miR-6872-5p				0	0	0	0	0	0
hsa-miR-12125				0	0	0	0	0	0
hsa-miR-3615	1.631642854	0.58080845	0.37307404	1.212070869	2.119087603	2.634846033	0	1.471918706	3.898978988
hsa-miR-7110-3p	-3.836695655		0.44908316	0	0	0	0	0.324914916	0.974744747
hsa-miR-6817-3p	1.582096547		0.59025362	0	0.961878269	1.673563938	0	0.344518087	1.033554261
hsa-miR-4653-5p				0	0	0	0	0	0
hsa-miR-3921				0	0	0	0	0	0
hsa-miR-6728-3p	3.544185779		0.5108146	0	0.278927323	0.836781969	0	0	0
hsa-miR-518c-5p				0	0	0	0	0	0
hsa-miR-330-5p	-1.683893595	0.0002407	0.00002565	221.854036	228.8923997	235.1357333	337.2616825	381.4416305	438.2270066
hsa-miR-6824-3p	3.543660986		0.51087004	0	0.175656402	0.526969207	0	0	0
hsa-miR-6764-3p	3.544185779		0.5108146	0	0.278927323	0.836781969	0	0	0
hsa-miR-6515-3p	-1.042348371	0.96612968	0.93484548	1.212070869	1.767774799	2.510345907	0.974744747	1.678520823	2.067108522
hsa-miR-4766-5p	-1.584011566		0.63146851	0	0.404023623	1.212070869	0	0.51677713	1.550331391
hsa-miR-12113				0	0	0	0	0	0
hsa-miR-4427				0	0	0	0	0	0
hsa-miR-4680-3p				0	0	0	0	0	0
hsa-miR-6719-3p				0	0	0	0	0	0
hsa-miR-6761-5p				0	0	0	0	0	0
hsa-miR-4649-3p				0	0	0	0	0	0

hsa-miR-7162-3p				0	0	0	0	0	0
hsa-miR-4682	3.543845864		0.51085051	0	0.202011812	0.606035435	0	0	0
hsa-miR-2276-3p	1.845956163	0.38005615	0.17643482	2.424141739	3.577049639	5.796661272	1.550331391	2.156091611	2.924234241
hsa-miR-4793-3p	-1.126853197		0.92121401	0	0.278927323	0.836781969	0	0.172259043	0.51677713
hsa-miR-1205				0	0	0	0	0	0
hsa-miR-10394-5p	-6.710501692		0.25817211	0	0	0	0	0.344518087	1.033554261
hsa-miR-6817-5p	-3.836695655		0.44908316	0	0	0	0	0.324914916	0.974744747
hsa-miR-4769-3p				0	0	0	0	0	0
hsa-miR-1913				0	0	0	0	0	0
hsa-miR-767-3p	6.046179412		0.32986518	0	0.377668214	0.606035435	0	0	0
hsa-miR-6839-5p	-3.836695655		0.44908316	0	0	0	0	0.324914916	0.974744747
hsa-miR-3128	-1.058896419	0.95639406	0.90469092	1.58090762	1.892871099	2.424141739	0.996854601	1.996065788	2.924234241
hsa-miR-3190-5p				0	0	0	0	0	0
hsa-miR-3922-3p				0	0	0	0	0	0
hsa-miR-4794				0	0	0	0	0	0
hsa-miR-149-5p	1.19000556	0.3561974	0.15911884	185.4931607	189.6087699	197.5675517	132.8117225	162.1384408	188.1257362
hsa-miR-3085-3p				0	0	0	0	0	0
hsa-miR-6504-5p				0	0	0	0	0	0
hsa-miR-3064-5p	-1.405439415	0.50701077	0.28644258	3.688784446	4.798180569	5.857473784	5.848468483	6.674876951	7.974836806
hsa-miR-3192-5p	28.56614587	0.17988145	0.05645914	1.673563938	2.217828494	3.161815239	0	0	0
hsa-miR-1285-3p	-1.071838769	0.94510719	0.87932586	1.673563938	2.042172092	2.634846033	0.996854601	2.015668959	3.100662783
hsa-miR-5189-5p				0	0	0	0	0	0
hsa-miR-4700-5p	3.543660986		0.51087004	0	0.175656402	0.526969207	0	0	0
hsa-miR-7155-5p				0	0	0	0	0	0
hsa-miR-6776-5p				0	0	0	0	0	0
hsa-miR-4490				0	0	0	0	0	0
hsa-miR-4774-5p	-3.835816219		0.44917283	0	0	0	0	0.172259043	0.51677713
hsa-miR-7152-3p				0	0	0	0	0	0
hsa-miR-7978				0	0	0	0	0	0
hsa-miR-3137				0	0	0	0	0	0
hsa-miR-4742-3p	1.47427106	0.47158408	0.25678958	5.020691815	7.786490123	11.06635334	2.990563802	5.491023405	7.797957977
hsa-miR-215-3p				0	0	0	0	0	0
hsa-miR-4677-3p	1.21405957	0.53977044	0.31572191	20.55179906	25.5015757	29.28736892	15.59591595	19.28817452	25.32207939
hsa-miR-4679				0	0	0	0	0	0
hsa-miR-6889-3p				0	0	0	0	0	0
hsa-miR-7157-3p				0	0	0	0	0	0
hsa-miR-6737-3p	3.543845864		0.51085051	0	0.202011812	0.606035435	0	0	0
hsa-miR-7114-5p	-3.836695655		0.44908316	0	0	0	0	0.324914916	0.974744747
hsa-miR-550b-3p				0	0	0	0	0	0
hsa-miR-676-5p	-2.816732127		0.30243324	0	0.175656402	0.526969207	0	0.676802954	1.033554261
hsa-miR-6754-3p	8.209990129		0.26121765	0	0.526969207	1.58090762	0	0	0
hsa-miR-942-5p	-1.037773243	0.89225575	0.76506332	111.2920019	117.6547776	123.6312287	112.0956459	120.3408599	132.2949454
hsa-miR-4778-3p	-1.651358268		0.55607115	0	0.351312804	1.053938413	0	0.836828777	1.993709202
hsa-miR-4635	-3.836695655		0.44908316	0	0	0	0	0.324914916	0.974744747
hsa-miR-432-5p	-44.09103935	0.07618147	0.01601479	0	0	0	2.924234241	3.681956563	4.134217044
hsa-miR-581	1.111123348	0.93212443	0.8418621	1.053938413	1.87104572	3.347127876	0	1.353605908	2.067108522
hsa-miR-9-5p	-1.256741606	0.15774659	0.04674826	5481.75868	5660.123664	5774.001596	6692.881789	7109.256044	7941.314163
hsa-miR-627-3p	-1.399638504	0.78066984	0.5998913	0.526969207	1.137534671	1.673563938	0.51677713	1.804203573	3.898978988
hsa-miR-494-3p	-8.730337374	0.08149347	0.01796425	0	0.278927323	0.836781969	0.974744747	2.515349601	3.987418403
hsa-miR-4738-3p	-1.155579957		0.8749822	0	0.377668214	0.606035435	0	0.344518087	1.033554261
hsa-miR-4786-3p	3.543845864		0.51085051	0	0.202011812	0.606035435	0	0	0
hsa-miR-7706	1.732258016	0.19947798	0.06755142	8.484496086	10.90740149	14.22529347	2.990563802	6.140853236	9.747447471
hsa-miR-3928-5p				0	0	0	0	0	0
hsa-miR-6806-3p	8.549055212		0.23674242	0	0.630240127	1.053938413	0	0	0
hsa-miR-493-3p	-3.662641483		0.18037451	0	0.202011812	0.606035435	0.51677713	1.154373742	1.949489494
hsa-miR-5585-5p				0	0	0	0	0	0
hsa-miR-4670-3p				0	0	0	0	0	0
hsa-miR-4790-3p				0	0	0	0	0	0
hsa-miR-549a-3p				0	0	0	0	0	0
hsa-miR-4462				0	0	0	0	0	0
hsa-miR-2277-3p	2.747747637	0.07909756	0.01708969	3.347127876	5.498426864	7.878460651	1.033554261	2.323487386	3.987418403
hsa-miR-7114-3p				0	0	0	0	0	0
hsa-miR-6859-3p	-1.868288732		0.36818363	0.526969207	0.656595537	0.836781969	0.974744747	1.334002737	1.993709202
hsa-miR-6846-3p	1.513631995		0.70103503	0	0.404023623	1.212070869	0	0.324914916	0.974744747
hsa-miR-29c-5p	-1.102185193	0.78709407	0.61014154	23.42989513	27.00080918	33.33194891	24.36861868	29.27602351	32.55695922
hsa-miR-6821-3p				0	0	0	0	0	0
hsa-miR-3661	-1.155519665		0.87670336	0	0.404023623	1.212070869	0	0.344518087	1.033554261
hsa-miR-6759-3p	-1.971816736		0.52426289	0	0.278927323	0.836781969	0	0.50454391	0.996854601
hsa-miR-6786-3p	-1.058614171	0.95639406	0.90513737	1.212070869	1.943431201	2.510345907	0.974744747	1.850779867	2.583885652
hsa-miR-6831-3p	3.544185779		0.5108146	0	0.278927323	0.836781969	0	0	0
hsa-miR-10525-3p	-3.836717681		0.44908091	0	0	0	0	0.332284867	0.996854601
hsa-miR-4522				0	0	0	0	0	0
hsa-miR-6857-3p	-1.181077564	0.89225575	0.76919202	0.606035435	1.286835664	1.673563938	0.996854601	1.498891829	1.949489494
hsa-miR-5002-3p				0	0	0	0	0	0
hsa-miR-6505-3p	1.125979729	0.86978632	0.72771362	5.454318912	5.702817989	5.857473784	3.100662783	5.619212837	8.772702724
hsa-miR-589-5p	1.249303979	0.38150312	0.17822043	53.55404602	65.9381574	75.75442934	45.85531163	53.51702822	60.43417432
hsa-miR-146b-5p	2.787510116	4.188E-18	1.284E-19	11405.33824	12646.69869	14641.31243	4171.836504	4537.259316	4891.269141
hsa-miR-146a-5p	1.515047806	0.47887536	0.26257683	4.183909846	6.875171956	11.59332254	3.898978988	4.523648609	5.684548435
hsa-miR-7153-5p				0	0	0	0	0	0
hsa-miR-6770-5p				0	0	0	0	0	0
hsa-miR-4524a-3p	-2.818023327		0.3223078	0	0.175656402	0.526969207	0	0.982114698	1.949489494
hsa-miR-4786-5p	8.549500881		0.23672994	0.526969207	0.656595537	0.836781969	0	0	0
hsa-miR-769-5p	-1.922888076	3.365E-06	2.604E-07	194.9786064	207.2376513	224.2331108	336.2869377	396.140946	472.3342972
hsa-miR-4693-3p				0	0	0	0	0	0
hsa-miR-143-3p	1.018993622	0.94510719	0.87830314	538.159466	596.9056095	706.1387368	554.6297611	585.4063884	642.3539731
hsa-miR-4770				0	0	0	0	0	0
hsa-miR-3663-3p				0	0	0	0	0	0
hsa-miR-1225-3p				0	0	0	0	0	0
hsa-miR-1233-3p				0	0	0	0	0	0
hsa-miR-4537				0	0	0	0	0	0
hsa-miR-6823-3p				0	0	0	0	0	0
hsa-miR-339-3p	1.61328017	0.005841	0.0008101	113.3286263	152.0455991	185.4931607	73.76724046	93.92023436	107.2219222
hsa-miR-610	11.10309858		0.18060845	0	0.884962758	1.818106304	0	0	0
hsa-miR-558				0	0	0	0	0	0
hsa-miR-5004-5p	6.045864117		0.32988072	0	0.351312804	1.053938413	0	0	0
hsa-miR-3150b-3p				0	0	0	0	0	0
hsa-miR-4784				0	0	0	0	0	0
hsa-miR-3679-5p	-1.184388978	0.86978632	0.7303435	0.606035435	1.741419389	2.510345907	0.996854601	1.843409916	2.583885652
hsa-miR-3605-5p	1.858853315	0.56165999	0.34833139	0.836781969	1.411931964	1.818106304	0	0.822088875	1.949489494
hsa-miR-1827				0	0	0	0	0	0
hsa-miR-5008-5p	-7.100074952		0.2513953	0	0	0	0	0.649829831	1.949489494
hsa-miR-3975				0	0	0	0	0	0
hsa-miR-2467-5p	-1.173925974		0.81299537	0.606035435	0.832251939	1.053938413	0	0.841692046	1.550331391
hsa-miR-3126-5p	-6.71207816		0.25810492	0	0	0	0	0.50454391	0.996854601
hsa-miR-6875-5p				0	0	0	0	0	0
hsa-miR-6133				0	0	0	0	0	0
hsa-miR-4510	1.166208467	0.86978632	0.72712382	2.510345907	2.90077944	3.161815239	1.949489494	2.347953826	3.100662783
hsa-miR-6130				0	0	0	0	0	0
hsa-miR-6127				0	0	0	0	0	0
hsa-miR-6129				0	0	0	0	0	0
hsa-miR-4721				0	0	0	0	0	0
hsa-miR-423-5p	-1.031442169	0.91672291	0.81233694	444.8300091	507.8947822	585.9897577	466.5279531	524.8699265	606.2912327
hsa-miR-3184-5p				0	0	0	0	0	0

hsa-miR-5194				0	0	0	0	0	0
hsa-miR-6804-5p	-3.835816219		0.44917283	0	0	0	0	0.172259043	0.51677713
hsa-let-7e-5p	2.482981815	3.769E-14	1.595E-15	4454.190421	4778.311284	5122.211494	1752.470388	1923.705179	2044.039735
hsa-miR-98-5p	6.946384372	1.822E-25	3.457E-27	142.808655	147.6205164	156.9631776	19.63753096	21.67617855	24.92136502
hsa-let-7f-5p	5.303966425	2.117E-48	1.855E-50	7155.322618	7752.803295	8094.209266	1380.643622	1458.336222	1575.136694
hsa-let-7a-5p	1.229320754	0.14233405	0.03885616	40951.27279	44359.8265	47442.27194	34612.78545	36087.08885	37782.08114
hsa-let-7c-5p	-1.591382018	0.00117	0.0001468	666.0784474	715.6996298	761.180506	1082.941414	1137.763782	1212.875925
hsa-let-7b-5p	4.196039138	1.019E-37	1.487E-39	4606.237834	4826.956442	5242.20651	1018.785402	1148.558835	1214.531955
hsa-miR-4500				0	0	0	0	0	0
hsa-let-7g-5p	8.077964776	1.918E-62	1.12E-64	2096.283504	2365.252626	2855.638968	282.109852	292.6425842	300.2213821
hsa-let-7i-5p	12.01263494	3.605E-88	1.053E-90	2988.985194	3220.521513	3535.004691	229.0650156	265.7110457	304.898507
hsa-miR-7854-3p				0	0	0	0	0	0
hsa-miR-6134	-3.835816219		0.44917283	0	0	0	0	0.172259043	0.51677713
hsa-miR-1911-5p				0	0	0	0	0	0
hsa-miR-496				0	0	0	0	0	0
hsa-miR-5702				0	0	0	0	0	0
hsa-miR-1236-5p	-1.126853197		0.92121401	0	0.278927323	0.836781969	0	0.172259043	0.51677713
hsa-miR-4765				0	0	0	0	0	0
hsa-miR-1909-5p	3.543845864		0.51085051	0	0.202011812	0.606035435	0	0	0
hsa-miR-4745-5p	3.543845864		0.51085051	0	0.202011812	0.606035435	0	0	0
hsa-miR-574-5p	2.76181732	7.517E-14	3.292E-15	321.9781852	367.1931067	449.0722571	108.1966669	131.8015966	157.6170248
hsa-miR-3659	3.544185779		0.5108146	0	0.278927323	0.836781969	0	0	0
hsa-miR-571				0	0	0	0	0	0
hsa-miR-190a-5p	-1.380690235	0.08476164	0.01908112	275.604895	333.247487	414.5282373	388.9231541	459.3664221	513.6764677
hsa-miR-190b-5p	1.106440825	0.78709407	0.60814995	33.19906001	45.61705552	59.41151981	31.19183191	40.76777769	51.83643924
hsa-miR-4772-5p				0	0	0	0	0	0
hsa-miR-4695-3p				0	0	0	0	0	0
hsa-miR-3682-3p	1.005069367	0.99392903	0.99343806	0.606035435	1.286835664	1.673563938	0.51677713	1.479288657	2.924234241
hsa-miR-8082				0	0	0	0	0	0
hsa-miR-3134	-3.835816219		0.44917283	0	0	0	0	0.172259043	0.51677713
hsa-miR-509-3p	3.012774186	0.31869443	0.13678272	0.836781969	1.587588366	2.107876826	0	0.664569734	1.993709202
hsa-miR-508-3p	3.948336031		0.17216385	0	1.080293822	2.634846033	0	0.172259043	0.51677713
hsa-miR-219a-5p	-1.400436361	0.19889794	0.06678325	27.27159456	28.51408995	29.28736892	37.20795339	40.67698169	44.85845703
hsa-miR-4782-3p				0	0	0	0	0	0
hsa-miR-6766-3p				0	0	0	0	0	0
hsa-miR-548f-5p				0	0	0	0	0	0
hsa-miR-548g-5p	2.443517492	0.02561353	0.004337	8.431507305	16.14241325	22.42331108	4.873723736	6.362195256	7.234879826
hsa-miR-891a-5p	-3.836695655		0.44908316	0	0	0	0	0.324914916	0.974744747
hsa-miR-891b				0	0	0	0	0	0
hsa-miR-4696				0	0	0	0	0	0
hsa-miR-5589-3p				0	0	0	0	0	0
hsa-miR-767-5p	7.91427893	2.602E-07	1.937E-08	14.22529347	18.13580837	21.21124021	0.974744747	2.183064734	2.990563802
hsa-miR-3177-3p				0	0	0	0	0	0
hsa-miR-4660	2.139898218		0.45666179	0	0.553324616	1.053938413	0	0.324914916	0.974744747
hsa-miR-1286	-1.741214597	0.50304972	0.28146606	0.606035435	1.390106585	2.510345907	1.033554261	2.641032351	3.898978988
hsa-miR-6722-3p				0	0	0	0	0	0
hsa-miR-7112-3p	1.318593519	0.78927331	0.61854004	1.212070869	1.664503878	2.107876826	0.996854601	1.326632785	1.949489494
hsa-miR-4711-5p				0	0	0	0	0	0
hsa-miR-6797-3p	-1.972560749		0.52403358	0	0.175656402	0.526969207	0	0.657199783	0.996854601
hsa-miR-5189-3p	3.544185779		0.5108146	0	0.278927323	0.836781969	0	0	0
hsa-miR-4641				0	0	0	0	0	0
hsa-miR-5699-5p	2.454844177	0.01256085	0.001815	12.55172954	15.70713661	20.02482985	6.201325565	6.667507	6.977982205
hsa-miR-4758-3p				0	0	0	0	0	0
hsa-miR-3189-5p	-3.836717681		0.44908091	0	0	0	0	0.332284867	0.996854601
hsa-miR-18b-3p	1.223312988		0.81267236	0	0.630240127	1.053938413	0	0.497173959	0.974744747
hsa-miR-4290				0	0	0	0	0	0
hsa-miR-7976	1.058883698	0.95639406	0.90644477	1.673563938	3.429899363	5.454318912	0.974744747	2.896567348	6.718102696
hsa-miR-6799-3p	8.548235796		0.23676536	0	0.553324616	1.053938413	0	0	0
hsa-miR-3173-5p	1.777330339	0.13133476	0.03470305	12.55172954	15.27675757	20.55179906	8.268434087	9.003227606	9.968546008
hsa-miR-938	-3.836717681		0.44908091	0	0	0	0	0.332284867	0.996854601
hsa-miR-6886-3p				0	0	0	0	0	0
hsa-miR-4632-3p				0	0	0	0	0	0
hsa-miR-24-2-5p	-4.506409906		0.11172227	0	0.175656402	0.526969207	0.974744747	1.173976913	1.550331391
hsa-miR-24-1-5p	-3.836695655		0.44908316	0	0	0	0	0.324914916	0.974744747
hsa-miR-3135a				0	0	0	0	0	0
hsa-miR-6778-3p				0	0	0	0	0	0
hsa-miR-6829-3p				0	0	0	0	0	0
hsa-miR-6791-3p	-1.127020076		0.92111946	0	0.175656402	0.526969207	0	0.172259043	0.51677713
hsa-miR-6781-3p	-6.710501692		0.25817211	0	0	0	0	0.344518087	1.033554261
hsa-miR-3116	3.543845864		0.51085051	0	0.202011812	0.606035435	0	0	0
hsa-miR-661				0	0	0	0	0	0
hsa-miR-214-5p	1.179052912	0.42341199	0.21757813	300.8994169	353.3855219	438.7696547	260.2568475	299.2439436	322.4689294
hsa-miR-4297				0	0	0	0	0	0
hsa-miR-3683				0	0	0	0	0	0
hsa-miR-668-5p				0	0	0	0	0	0
hsa-miR-6820-5p	-3.835816219		0.44917283	0	0	0	0	0.172259043	0.51677713
hsa-miR-1199-3p				0	0	0	0	0	0
hsa-miR-4749-5p				0	0	0	0	0	0
hsa-miR-744-5p	1.266555887	0.15436815	0.04439493	233.4621694	269.2013614	313.5466779	193.7914239	214.6014677	244.6609315
hsa-miR-6854-3p	-1.126853197		0.92121401	0	0.278927323	0.836781969	0	0.172259043	0.51677713
hsa-miR-8086				0	0	0	0	0	0
hsa-miR-31-3p				0	0	0	0	0	0
hsa-miR-3934-3p	-2.101612495		0.36836684	0	0.454583725	0.836781969	0	0.689036174	2.067108522
hsa-miR-7160-5p				0	0	0	0	0	0
hsa-miR-8075				0	0	0	0	0	0
hsa-miR-6882-3p	3.543845864		0.51085051	0	0.202011812	0.606035435	0	0	0
hsa-miR-1287-5p	-2.150824389	0.14422374	0.04084585	1.58090762	2.575822045	3.636212608	2.924234241	5.207821514	6.718102696
hsa-miR-5580-5p				0	0	0	0	0	0
hsa-miR-4731-5p				0	0	0	0	0	0
hsa-miR-4999-5p	-1.185497882	0.86618737	0.71401399	1.212070869	1.943431201	2.510345907	0.974744747	2.02303891	3.100662783
hsa-miR-4804-3p	11.05107429		0.18100615	0	0.80589653	1.58090762	0	0	0
hsa-miR-4720-3p				0	0	0	0	0	0
hsa-miR-516b-3p				0	0	0	0	0	0
hsa-miR-7162-5p				0	0	0	0	0	0
hsa-miR-3148				0	0	0	0	0	0
hsa-miR-147b-5p	2.140817191		0.45643216	0	0.682950946	1.212070869	0	0.172259043	0.51677713
hsa-miR-7-5p	1.035490291	0.8800976	0.74677767	22335.38432	24185.7173	25895.28809	21358.60668	23354.68429	25634.21278
hsa-miR-4533				0	0	0	0	0	0
hsa-miR-3202	-3.836695655		0.44908316	0	0	0	0	0.324914916	0.974744747
hsa-miR-1-3p	-2.35059475	1.312E-10	7.28E-12	415.0438567	459.0139262	507.4713459	1006.823147	1080.996129	1158.097549
hsa-miR-206				0	0	0	0	0	0
hsa-miR-6861-3p				0	0	0	0	0	0
hsa-miR-184	-1.181919704	0.90124123	0.78282998	0	1.106649232	2.107876826	0.51677713	1.818943476	2.990563802
hsa-miR-1288-3p	-7.100074952		0.25304837	0	0	0	0	0.649829831	1.949489494
hsa-miR-139-3p	-1.972097433		0.52419545	0	0.175656402	0.526969207	0	0.497173959	0.974744747
hsa-miR-4306				0	0	0	0	0	0
hsa-miR-185-5p	1.434988864	0.01326534	0.001937	1876.901957	1963.026809	2095.064498	1279.961307	1369.767687	1499.157421
hsa-miR-4644				0	0	0	0	0	0
hsa-miR-4683				0	0	0	0	0	0
hsa-miR-765	1.318391167	0.78927331	0.61874418	0.836781969	1.561232957	2.634846033	0.974744747	1.334002737	1.993709202
hsa-miR-1289	-1.126853197		0.92121401	0	0.278927323	0.836781969	0	0.172259043	0.51677713
hsa-miR-122-5p	284.049571	1.986E-09	1.218E-10	91.51135064	95.53144382	98.01627242	0	0.332284867	0.996854601
hsa-miR-4653-3p	-1.971645644		0.52720881	0	0.175656402	0.526969207	0	0.344518087	1.033554261

hsa-miR-4791	-1.082151279	0.94502314	0.86500658	1.053938413	3.0349358	5.020691815	0	2.849991054	4.650994174
hsa-miR-6744-5p				0	0	0	0	0	0
hsa-miR-4764-5p				0	0	0	0	0	0
hsa-miR-876-5p	-14.46721468	0.00006667	6.423E-06	0	0.606035435	1.818106304	5.848468483	9.898865897	11.96225521
hsa-miR-1290	-1.259551799	0.83328558	0.66956429	0	2.11670829	4.242248043	0	2.677732011	4.134217044
hsa-miR-4470	-1.155905029		0.8749476	0	0.351312804	1.053938413	0	0.497173959	0.974744747
hsa-miR-6809-5p				0	0	0	0	0	0
hsa-miR-9851-3p				0	0	0	0	0	0
hsa-miR-1207-5p				0	0	0	0	0	0
hsa-miR-449a	1.77037322	0.27035609	0.10814244	6.850599685	8.834115367	12.12070869	1.949489494	4.390595908	7.234879826
hsa-miR-34a-5p	1.421736961	0.03078032	0.005482	1053.938413	1197.676537	1403.578067	745.6797315	840.9073059	944.6685944
hsa-miR-548au-3p				0	0	0	0	0	0
hsa-miR-3926				0	0	0	0	0	0
hsa-miR-3136-3p				0	0	0	0	0	0
hsa-miR-7155-3p				0	0	0	0	0	0
hsa-miR-4745-3p	3.543845864		0.51085051	0	0.202011812	0.606035435	0	0	0
hsa-miR-1291	-2.568203907	0.34014857	0.15095644	0.526969207	0.656595537	0.836781969	0.974744747	1.678520823	2.067108522
hsa-miR-6851-3p	1.514045233		0.70086641	0	0.454583725	0.836781969	0	0.172259043	0.51677713
hsa-miR-4743-5p				0	0	0	0	0	0
hsa-miR-8053				0	0	0	0	0	0
hsa-miR-4467	1.055460117	0.94510719	0.8809551	4.848283478	5.046222453	5.269692065	0.996854601	4.8068505	8.772702724
hsa-miR-6787-5p				0	0	0	0	0	0
hsa-miR-24-3p	1.002416527	0.98948735	0.98245606	4587.238755	4896.05367	5444.622345	4736.2624	4886.729979	4999.465808
hsa-miR-10398-5p				0	0	0	0	0	0
hsa-miR-6762-3p				0	0	0	0	0	0
hsa-miR-4687-3p				0	0	0	0	0	0
hsa-miR-6815-3p	-3.836695655		0.44908316	0	0	0	0	0.324914916	0.974744747
hsa-miR-3119				0	0	0	0	0	0
hsa-miR-6733-5p	-1.566761978	0.56177778	0.34936837	1.58090762	1.892871099	2.424141739	1.949489494	2.852497737	3.617439913
hsa-miR-6739-5p	3.543660986		0.51087004	0	0.175656402	0.526969207	0	0	0
hsa-miR-1292-5p	2.647879506	0.08130617	0.01780427	3.347127876	5.243704234	6.323630479	1.550331391	2.163461563	2.990563802
hsa-miR-4471				0	0	0	0	0	0
hsa-miR-6716-5p				0	0	0	0	0	0
hsa-miR-8085				0	0	0	0	0	0
hsa-miR-6731-5p				0	0	0	0	0	0
hsa-miR-6827-5p				0	0	0	0	0	0
hsa-miR-5591-5p				0	0	0	0	0	0
hsa-miR-4300				0	0	0	0	0	0
hsa-miR-7106-5p				0	0	0	0	0	0
hsa-miR-6785-5p	-3.661972688		0.18045693	0	0.175656402	0.526969207	0	0.994347918	1.949489494
hsa-miR-4728-5p	-3.835816219		0.44917283	0	0	0	0	0.172259043	0.51677713
hsa-miR-4713-3p				0	0	0	0	0	0
hsa-miR-5187-5p	-1.935965078	0.17519428	0.05473223	2.107876826	3.03040577	3.636212608	3.898978988	5.692762253	6.977982205
hsa-miR-6721-5p				0	0	0	0	0	0
hsa-miR-4640-5p	-1.127020076		0.92111946	0	0.175656402	0.526969207	0	0.172259043	0.51677713
hsa-miR-6508-3p	-1.127278468		0.92096409	0	0.175656402	0.526969207	0	0.324914916	0.974744747
hsa-miR-6729-5p				0	0	0	0	0	0
hsa-miR-4649-5p				0	0	0	0	0	0
hsa-miR-10394-3p				0	0	0	0	0	0
hsa-miR-585-3p				0	0	0	0	0	0
hsa-miR-664b-5p	3.543660986		0.51087004	0	0.175656402	0.526969207	0	0	0
hsa-miR-4437				0	0	0	0	0	0
hsa-miR-4656	-3.836717681		0.44908091	0	0	0	0	0.332284867	0.996854601
hsa-miR-6829-5p				0	0	0	0	0	0
hsa-miR-4498				0	0	0	0	0	0
hsa-miR-4725-3p	1.513474153		0.70111189	0	0.351312804	1.053938413	0	0.324914916	0.974744747
hsa-miR-6780b-5p				0	0	0	0	0	0
hsa-miR-3191-3p				0	0	0	0	0	0
hsa-miR-1343-5p	3.543845864		0.51085051	0	0.202011812	0.606035435	0	0	0
hsa-miR-939-5p	6.046759143		0.32983659	0	0.454583725	0.836781969	0	0	0
hsa-miR-6825-5p	-1.127020076		0.92111946	0	0.175656402	0.526969207	0	0.172259043	0.51677713
hsa-miR-4450	-1.155844751		0.87477853	0	0.377668214	0.606035435	0	0.497173959	0.974744747
hsa-miR-3180	1.988726782	0.02024997	0.003281	35.75609065	46.04527845	66.39812002	14.46975965	23.85256711	32.16657665
hsa-miR-3180-3p	2.38257922	0.003131	0.000425	14.54485043	24.59226927	31.61815239	6.977982205	10.16732789	12.67168171
hsa-miR-6816-5p	-1.155579957		0.8749822	0	0.377668214	0.606035435	0	0.344518087	1.033554261
hsa-miR-4489				0	0	0	0	0	0
hsa-miR-4283				0	0	0	0	0	0
hsa-miR-6870-5p				0	0	0	0	0	0
hsa-miR-4723-5p				0	0	0	0	0	0
hsa-miR-7111-5p	3.543660986		0.51087004	0	0.175656402	0.526969207	0	0	0
hsa-miR-5698	3.543660986		0.51087004	0	0.175656402	0.526969207	0	0	0
hsa-miR-6846-5p				0	0	0	0	0	0
hsa-miR-6848-5p				0	0	0	0	0	0
hsa-miR-6887-5p				0	0	0	0	0	0
hsa-miR-6795-5p				0	0	0	0	0	0
hsa-miR-6842-5p				0	0	0	0	0	0
hsa-miR-7110-5p				0	0	0	0	0	0
hsa-miR-6881-5p	3.543845864		0.51085051	0	0.202011812	0.606035435	0	0	0
hsa-miR-4664-5p	1.514045233		0.70086641	0	0.454583725	0.836781969	0	0.172259043	0.51677713
hsa-miR-3132	2.140091045		0.4566015	0	0.630240127	1.053938413	0	0.332284867	0.996854601
hsa-miR-193a-5p	1.340358722	0.31869443	0.13447007	17.57242135	25.74211207	30.56421398	17.54540545	20.03686407	22.92765582
hsa-miR-6745				0	0	0	0	0	0
hsa-miR-1293	-1.127219659		0.9209974	0	0.202011812	0.606035435	0	0.324914916	0.974744747
hsa-miR-6772-5p				0	0	0	0	0	0
hsa-miR-23b-5p	-3.620826452	0.14285303	0.03978813	0.526969207	0.656595537	0.836781969	1.550331391	2.828031296	4.984273004
hsa-miR-629-5p	-1.025057431	0.93212443	0.84171772	303.534263	320.6622164	332.1074182	305.0375078	323.5751439	358.6433285
hsa-miR-379-5p	-54.29416887	0.0636499	0.01251244	0	0	0	0.996854601	4.368486054	7.234879826
hsa-miR-4451	-2.842914431		0.17549073	0	0.480939135	0.836781969	0	1.646684432	2.990563802
hsa-miR-6801-5p				0	0	0	0	0	0
hsa-miR-10393-3p				0	0	0	0	0	0
hsa-miR-412-5p				0	0	0	0	0	0
hsa-miR-601				0	0	0	0	0	0
hsa-miR-4527				0	0	0	0	0	0
hsa-miR-6753-3p	2.016438871		0.51157068	0	0.526969207	1.58090762	0	0.324914916	0.974744747
hsa-miR-7107-3p				0	0	0	0	0	0
hsa-miR-675-5p	2.35645485	0.16797341	0.05075985	1.818106304	5.999269258	9.485445718	0.996854601	2.493239747	3.898978988
hsa-miR-6880-5p				0	0	0	0	0	0
hsa-miR-541-3p				0	0	0	0	0	0
hsa-miR-654-5p				0	0	0	0	0	0
hsa-miR-6769b-5p				0	0	0	0	0	0
hsa-miR-876-3p	-6.198930599		0.0471533	0	0.175656402	0.526969207	1.033554261	1.983832568	2.924234241
hsa-miR-182-3p	1.454825423	0.47887536	0.26221892	5.269692065	6.085016233	7.531037722	1.949489494	3.861585557	4.984273004
hsa-miR-597-3p	11.05165014		0.18080018	0.606035435	0.832251939	1.053938413	0	0	0
hsa-miR-380-5p				0	0	0	0	0	0
hsa-miR-299-5p	-12.46353505		0.12246853	0	0	0	0	0.994347918	1.949489494
hsa-miR-30d-5p	-1.002470134	0.98948735	0.98370933	4247.702362	4924.103117	5661.230186	4430.021846	4937.444183	5940.094489
hsa-miR-30b-5p	-1.157526187	0.53977044	0.31382786	383.1066132	473.60336	632.7009938	483.4733946	547.6704691	637.1862018
hsa-miR-30c-5p	-1.254034853	0.16728333	0.05006289	1897.821506	2231.489367	2727.765492	2573.326132	2798.712678	2938.911541
hsa-miR-30a-5p	-1.026391297	0.92808503	0.83324422	866.0246362	884.6185909	920.0882346	838.2804825	903.9641855	1011.332844
hsa-miR-30e-5p	-1.360808057	0.0764259	0.01628932	4840.739131	6116.000246	7096.67494	6354.361006	8321.303677	9973.798617
hsa-miR-194-5p	-1.622629079	0.0004966	0.00005582	143.8625934	155.9559401	177.5683824	226.1407813	253.5037574	280.1161428
hsa-miR-4732-5p				0	0	0	0	0	0
hsa-miR-3935				0	0	0	0	0	0

hsa-miR-8065				0	0	0	0	0	0
hsa-miR-6806-5p				0	0	0	0	0	0
hsa-miR-142-3p	-1.660730917	0.1938181	0.06422877	6.850599685	9.266873713	10.90863782	8.772702724	14.58991028	17.94338281
hsa-miR-4703-3p				0	0	0	0	0	0
hsa-miR-12122				0	0	0	0	0	0
hsa-miR-6879-3p	-1.714508625		0.54046349	0	0.404023623	1.212070869	0	0.982114698	1.949489494
hsa-miR-668-3p				0	0	0	0	0	0
hsa-miR-4697-3p				0	0	0	0	0	0
hsa-miR-223-3p	-12.46360655		0.12046724	0	0	0	0.974744747	1.00171787	1.033554261
hsa-miR-3909	1.344557059	0.77631463	0.58627091	0.836781969	2.544936606	3.636212608	0.974744747	2.330857337	4.984273004
hsa-miR-6852-3p				0	0	0	0	0	0
hsa-miR-4440	1.568443049		0.60488876	0	1.012438371	2.510345907	0	0.649829831	1.949489494
hsa-miR-3618				0	0	0	0	0	0
hsa-miR-934				0	0	0	0	0	0
hsa-miR-4796-5p	13.55352259		0.14406764	0.606035435	1.007908341	1.58090762	0	0	0
hsa-miR-6895-3p	2.361329418	0.39013864	0.18624136	1.673563938	1.86651569	2.107876826	0	0.51677713	1.550331391
hsa-miR-593-3p				0	0	0	0	0	0
hsa-miR-346				0	0	0	0	0	0
hsa-miR-550a-3p	1.138548361	0.87490792	0.73952071	4.848283478	6.48235911	7.904538098	1.949489494	6.003087362	12.95910981
hsa-miR-6740-3p	-1.650884479		0.55358787	0	0.377668214	0.606035435	0	0.676802954	1.033554261
hsa-miR-431-5p	-5.612451283	0.0730799	0.01514941	0	0.351312804	1.053938413	0.974744747	2.687608644	3.987418403
hsa-miR-6830-3p	-3.836717681		0.44908091	0	0	0	0	0.332284867	0.996854601
hsa-miR-4504	-1.911732684	0.40770922	0.2069139	0	1.288986381	3.030177174	0.996854601	2.493239747	3.898978988
hsa-miR-542-3p	-1.054575309	0.86618737	0.71192614	1214.664021	1456.004442	1927.945657	1127.779672	1533.954226	2036.618671
hsa-miR-7973				0	0	0	0	0	0
hsa-miR-3118	1.069323527	0.90350303	0.79138952	6.850599685	10.95988372	15.15088587	9.747447471	10.0171787	10.33554261
hsa-miR-134-5p	-7.043289579	0.12351607	0.03227646	0	0.175656402	0.526969207	0.996854601	2.14872166	3.898978988
hsa-miR-6820-3p	3.543845864		0.51085051	0	0.202011812	0.606035435	0	0	0
hsa-miR-3164	2.655056466	0.39565644	0.19522901	0.606035435	1.462492066	2.107876826	0	0.664569734	1.993709202
hsa-miR-1294	1.237393883	0.86978632	0.73265213	0.606035435	1.286835664	1.673563938	0.974744747	1.00171787	1.033554261
hsa-miR-9986				0	0	0	0	0	0
hsa-miR-3689a-5p	41.23260809	6.974E-10	4.174E-11	30.56421398	34.83177483	38.78626782	0.51677713	0.829458826	0.996854601
hsa-miR-3689f	28.43962929	0.00133	0.0001747	6.694255753	9.152392015	11.06635334	0	0.332284867	0.996854601
hsa-miR-19b-3p	-1.423980037	0.03017653	0.005286	2393.494136	2977.395749	3839.234479	3666.989739	4239.44607	4573.477604
hsa-miR-19a-3p	-1.324860661	0.14422374	0.04068046	723.5287206	963.0942824	1289.643405	1007.886069	1275.523555	1448.009519
hsa-miR-3944-5p	-1.155740566		0.87485273	0	0.454583725	0.836781969	0	0.50454391	0.996854601
hsa-miR-933	1.223369741		0.8126278	0.526969207	0.656595537	0.836781969	0	0.50454391	0.996854601
hsa-miR-636	1.301261172	0.7723693	0.57979024	0.606035435	2.371659517	3.347127876	0.974744747	1.678520823	2.067108522
hsa-miR-3190-3p	2.76736178		0.30344754	0.526969207	0.858607348	1.212070869	0	0.172259043	0.51677713
hsa-miR-3138				0	0	0	0	0	0
hsa-miR-4648				0	0	0	0	0	0
hsa-miR-4654				0	0	0	0	0	0
hsa-miR-6748-5p				0	0	0	0	0	0
hsa-miR-6793-5p				0	0	0	0	0	0
hsa-miR-4536-5p	3.543660986		0.51087004	0	0.175656402	0.526969207	0	0	0
hsa-miR-3177-5p	1.513698188		0.7010028	0	0.454583725	0.836781969	0	0.324914916	0.974744747
hsa-miR-597-5p	1.429850807	0.76832627	0.56757909	0.836781969	1.438287374	2.424141739	0.51677713	1.161743693	1.993709202
hsa-miR-3657	3.19236955	0.21722977	0.07674395	0.526969207	3.667326645	5.454318912	0.51677713	1.161743693	1.993709202
hsa-miR-4669				0	0	0	0	0	0
hsa-miR-3911	-1.12728494		0.9209602	0	0.175656402	0.526969207	0	0.332284867	0.996854601
hsa-miR-6867-5p	-3.835816219		0.44917283	0	0	0	0	0.172259043	0.51677713
hsa-miR-3152-3p				0	0	0	0	0	0
hsa-miR-1206				0	0	0	0	0	0
hsa-miR-6853-3p	6.046179412		0.32986518	0	0.377668214	0.606035435	0	0	0
hsa-miR-4326	1.581270987		0.57269639	0.526969207	0.93552286	1.673563938	0	0.664569734	1.993709202
hsa-miR-2117				0	0	0	0	0	0
hsa-miR-4684-3p				0	0	0	0	0	0
hsa-miR-4425	-3.836717681		0.44908091	0	0	0	0	0.332284867	0.996854601
hsa-miR-3613-5p	1.028745814	0.94510719	0.87610697	76.93750416	86.61103467	104.2380948	52.63621634	83.42871712	105.9393117
hsa-miR-6755-3p	-9.586756314		0.16940324	0	0	0	0	0.676802954	1.033554261
hsa-miR-5705				0	0	0	0	0	0
hsa-miR-8068				0	0	0	0	0	0
hsa-miR-2052				0	0	0	0	0	0
hsa-miR-4764-3p				0	0	0	0	0	0
hsa-miR-499a-5p	-1.514742368	0.46450801	0.25081104	1.212070869	3.937422504	5.857473784	3.898978988	5.372710606	7.234879826
hsa-miR-889-3p	-12.46068196		0.12030223	0	0	0	0	0.841692046	1.550331391
hsa-miR-155-5p	-3.746022533	0.00003385	3.063E-06	4.183909846	4.793650539	5.454318912	17.54540545	18.2031807	19.12075383
hsa-miR-4775	-3.234134227	0.10168479	0.02479031	0	1.260480255	2.107876826	3.100662783	4.637098138	6.82321323
hsa-miR-3941	-1.126853197		0.92121401	0	0.278927323	0.836781969	0	0.172259043	0.51677713
hsa-miR-4672	6.046494721		0.32984963	0	0.404023623	1.212070869	0	0	0
hsa-miR-582-5p	6.089481797	2.532E-17	8.503E-19	114.3523178	142.6756921	173.9321698	13.64642646	22.39134398	33.59051348
hsa-miR-4788				0	0	0	0	0	0
hsa-miR-5687	-1.126961281		0.92115277	0	0.202011812	0.606035435	0	0.172259043	0.51677713
hsa-miR-3684	1.035598288	0.96119655	0.92032801	5.020691815	6.960918931	8.484496086	2.924234241	7.189297495	12.95910981
hsa-miR-6866-5p	1.201535859	0.84573612	0.69387402	2.107876826	2.90530947	4.183909846	0.996854601	2.320980704	3.898978988
hsa-miR-4662a-5p	-1.180622959	0.90300712	0.78933784	0	1.365901892	2.424141739	0.974744747	1.346235957	2.067108522
hsa-miR-4321				0	0	0	0	0	0
hsa-miR-5579-3p				0	0	0	0	0	0
hsa-miR-5704				0	0	0	0	0	0
hsa-miR-1295a				0	0	0	0	0	0
hsa-miR-12116				0	0	0	0	0	0
hsa-miR-3162-5p	-6.712040629		0.25810652	0	0	0	0	0.497173959	0.974744747
hsa-miR-1296-5p	-1.070750777	0.8380233	0.68142917	40.60437413	46.53409068	51.04370011	44.85845703	49.81797517	56.53519533
hsa-miR-4540				0	0	0	0	0	0
hsa-miR-4474-5p				0	0	0	0	0	0
hsa-miR-3606-5p				0	0	0	0	0	0
hsa-miR-4671-3p				0	0	0	0	0	0
hsa-miR-340-5p	1.034754847	0.89225575	0.76711475	1696.993833	1801.393478	1857.566453	1655.116581	1738.532568	1844.894356
hsa-miR-374a-5p	1.212975062	0.25723756	0.09913973	1890.224521	2006.448882	2176.909792	1596.631896	1656.423002	1736.520714
hsa-miR-361-5p	1.040311809	0.86978632	0.72956009	1916.284045	2073.358743	2328.149955	1838.69303	1996.039175	2174.655531
hsa-miR-9903	1.144779497	0.89301664	0.77438231	0.836781969	2.193623801	3.636212608	0.974744747	2.01080569	2.990563802
hsa-miR-584-5p	-3.836695655		0.44908316	0	0	0	0	0.324914916	0.974744747
hsa-miR-12123				0	0	0	0	0	0
hsa-miR-137-3p	4.538332595	1.106E-38	1.454E-40	7599.653843	8219.96755	8936.343805	1741.504988	1808.646361	1942.565233
hsa-miR-5701	2.140214897		0.45656489	0.526969207	0.656595537	0.836781969	0	0.324914916	0.974744747
hsa-miR-7159-5p				0	0	0	0	0	0
hsa-miR-95-3p	-3.532135663	6.258E-12	3.198E-13	31.5138426	36.27207394	42.15753652	102.6760239	128.7246747	149.1359463
hsa-miR-26a-5p	1.204317088	0.23448634	0.08626188	22568.15355	23905.79602	26269.94192	18691.31203	19854.17565	20823.47203
hsa-miR-26b-5p	1.123499357	0.46012561	0.24584814	28565.22608	30904.08739	34374.32986	26720.67775	27505.87211	29042.35795
hsa-miR-1297	1.513979025		0.70089864	0	0.404023623	1.212070869	0	0.172259043	0.51677713
hsa-miR-513b-5p				0	0	0	0	0	0
hsa-miR-513a-5p				0	0	0	0	0	0
hsa-miR-9985	1.301403895	0.7723693	0.57962543	1.818106304	2.424370336	3.347127876	0.996854601	1.671150872	2.067108522
hsa-miR-27a-3p	-1.101855051	0.65481771	0.44583717	2438.286519	3141.927081	3825.295664	2720.512589	3460.792622	4128.015718
hsa-miR-27b-3p	-1.996393197	9.288E-08	6.78E-09	1950.313033	2245.970054	2478.078893	3867.787156	4481.028034	5222.032903
hsa-miR-197-3p	1.033764192	0.93841009	0.85621359	38.49197058	52.31885538	62.70933558	36.88362023	51.0040352	62.38366381
hsa-miR-6802-3p	-3.836717681		0.44908091	0	0	0	0	0.332284867	0.996854601
hsa-miR-653-3p				0	0	0	0	0	0
hsa-miR-5100	8.548681411		0.23675288	0	0.579680025	1.212070869	0	0	0
hsa-miR-4291				0	0	0	0	0	0
hsa-miR-3157-5p	3.543845864		0.51085051	0	0.202011812	0.606035435	0	0	0
hsa-miR-12128				0	0	0	0	0	0
hsa-miR-11399				0	0	0	0	0	0

hsa-miR-7154-5p				0	0	0	0	0	0
hsa-miR-1298-5p				0	0	0	0	0	0
hsa-miR-664b-3p	1.063696793	0.95313168	0.89608292	1.673563938	2.876574747	4.848283478	0.996854601	2.360187046	4.134217044
hsa-miR-579-3p	-1.313164859	0.76108188	0.55442315	0	1.659973848	3.161815239	1.949489494	2.347953826	3.100662783
hsa-miR-6509-3p				0	0	0	0	0	0
hsa-miR-6720-5p				0	0	0	0	0	0
hsa-miR-6512-3p	-3.835816219		0.44917283	0	0	0	0	0.172259043	0.51677713
hsa-miR-5581-3p	-1.44623128	0.7723693	0.57752824	0.606035435	0.832251939	1.053938413	0	1.646684432	2.990563802
hsa-miR-7977	-1.403664294	0.2472901	0.09386194	16.96899217	30.19419565	38.46875208	39.79183904	42.52776155	46.85216624
hsa-miR-6883-3p				0	0	0	0	0	0
hsa-miR-204-5p	1.223089413		0.81282437	0.526969207	0.656595537	0.836781969	0	0.657199783	0.996854601
hsa-miR-211-5p	-3.836695655		0.44908316	0	0	0	0	0.324914916	0.974744747
hsa-miR-6084				0	0	0	0	0	0
hsa-miR-202-5p	1.514124203		0.70082798	0	0.480939135	0.836781969	0	0.172259043	0.51677713
hsa-miR-6783-3p	1.939222112		0.40235547	0	0.93099283	1.58090762	0	0.50454391	0.996854601
hsa-miR-6868-3p	1.389737	0.56232001	0.35052649	3.347127876	7.386767933	10.90863782	4.650994174	5.833184905	7.974836806
hsa-miR-6788-3p				0	0	0	0	0	0
hsa-miR-3124-5p	13.55422855		0.14387678	0.836781969	1.034263751	1.212070869	0	0	0
hsa-miR-3944-3p	18.56014699		0.09879842	1.212070869	1.488847476	1.673563938	0	0	0
hsa-miR-4798-5p				0	0	0	0	0	0
hsa-miR-4777-5p				0	0	0	0	0	0
hsa-miR-514b-5p				0	0	0	0	0	0
hsa-miR-513c-5p				0	0	0	0	0	0
hsa-miR-515-5p	2.766727453		0.30351398	0.526969207	0.858607348	1.212070869	0	0.324914916	0.974744747
hsa-miR-519e-5p				0	0	0	0	0	0
hsa-miR-4713-5p				0	0	0	0	0	0
hsa-miR-516a-5p	45.84435282	8.426E-48	8.611E-50	212.3685902	223.1247457	243.6262448	2.990563802	4.66893453	5.848468483
hsa-miR-6873-3p	1.642320196	0.56627984	0.3577072	1.58090762	3.05676118	4.242248043	0.51677713	2.143858391	2.990563802
hsa-miR-4753-3p	3.393158471		0.21326996	0	0.90463742	2.107876826	0	0.172259043	0.51677713
hsa-miR-4303				0	0	0	0	0	0
hsa-miR-12119				0	0	0	0	0	0
hsa-miR-5001-3p	-1.180788814	0.83107589	0.66215964	4.242248043	6.032305414	7.531037722	2.067108522	7.578578965	11.69693697
hsa-miR-6071				0	0	0	0	0	0
hsa-miR-1299	1.112856479	0.91051708	0.80285009	3.030177174	3.458634086	4.183909846	0	2.867237639	4.984273004
hsa-miR-5706	3.543845864		0.51085051	0	0.202011812	0.606035435	0	0	0
hsa-miR-4782-5p				0	0	0	0	0	0
hsa-miR-6844	-1.033773322	0.95639406	0.91258077	6.323630479	13.20836905	22.42331108	7.797957977	13.57595919	17.94338281
hsa-miR-488-3p				0	0	0	0	0	0
hsa-miR-203b-3p	3.543845864		0.51085051	0	0.202011812	0.606035435	0	0	0
hsa-miR-4709-3p				0	0	0	0	0	0
hsa-miR-6864-5p				0	0	0	0	0	0
hsa-miR-4452	-3.835816219		0.44917283	0	0	0	0	0.172259043	0.51677713
hsa-miR-580-3p	1.044226856	0.96565133	0.92617945	1.818106304	2.145443013	2.510345907	1.033554261	2.316117435	2.990563802
hsa-miR-3915				0	0	0	0	0	0
hsa-miR-4689	-1.155998823		0.88123652	0	0.454583725	0.836781969	0	0.649829831	1.949489494
hsa-miR-6734-5p	-1.126853197		0.92121401	0	0.278927323	0.836781969	0	0.172259043	0.51677713
hsa-miR-7703	2.13988595		0.45666426	0	0.553324616	1.053938413	0	0.332284867	0.996854601
hsa-miR-4325				0	0	0	0	0	0
hsa-miR-1301-3p	1.990914813	4.511E-07	3.424E-08	417.5542026	442.416706	464.259871	199.9927495	225.1679537	263.1810817
hsa-miR-5047				0	0	0	0	0	0
hsa-miR-153-3p	1.473548381	0.58645201	0.3792675	2.107876826	4.703969708	8.367819691	1.949489494	2.692471913	4.134217044
hsa-miR-448				0	0	0	0	0	0
hsa-miR-4666b				0	0	0	0	0	0
hsa-miR-3064-3p	3.543660986		0.51087004	0	0.175656402	0.526969207	0	0	0
hsa-miR-5006-5p				0	0	0	0	0	0
hsa-miR-5590-5p				0	0	0	0	0	0
hsa-miR-4659b-5p				0	0	0	0	0	0
hsa-miR-6894-3p	3.543660986		0.51087004	0	0.175656402	0.526969207	0	0	0
hsa-miR-4639-5p	8.548235796		0.23676536	0	0.553324616	1.053938413	0	0	0
hsa-miR-449c-3p				0	0	0	0	0	0
hsa-miR-1178-3p				0	0	0	0	0	0
hsa-miR-6772-3p	-1.651186054		0.56297267	0	0.404023623	1.212070869	0	0.836828777	1.993709202
hsa-miR-5691				0	0	0	0	0	0
hsa-miR-6805-3p				0	0	0	0	0	0
hsa-miR-9718				0	0	0	0	0	0
hsa-miR-6079				0	0	0	0	0	0
hsa-miR-6505-5p	1.96858743	0.18991213	0.06154817	4.183909846	6.198901756	8.958476511	2.924234241	3.177412652	3.617439913
hsa-miR-4520-3p				0	0	0	0	0	0
hsa-miR-4804-5p	-1.435178574	0.65481771	0.44652909	1.053938413	2.525261943	4.848283478	1.949489494	3.369274867	5.167771304
hsa-miR-5009-5p	1.091692238	0.92597494	0.82565532	3.030177174	3.634290480	4.183909846	1.993709202	3.322698573	4.873723736
hsa-miR-4443	1.300541442	0.7723693	0.58054157	1.212070869	2.294744005	3.161815239	1.033554261	1.983832568	2.924234241
hsa-miR-6515-5p	8.549055212		0.23674242	0	0.630240127	1.053938413	0	0	0
hsa-miR-588				0	0	0	0	0	0
hsa-miR-4701-5p				0	0	0	0	0	0
hsa-miR-3187-3p	-1.604047754	0.39076896	0.18816309	2.510345907	2.90077944	3.161815239	3.898978988	4.523648609	5.684548435
hsa-miR-6842-3p	2.140326479		0.48017263	0	0.682950946	1.212070869	0	0.324914916	0.974744747
hsa-miR-1302	-1.868018776		0.36833066	0	0.579680025	1.212070869	0	1.166606962	1.949489494
hsa-miR-6780a-5p				0	0	0	0	0	0
hsa-miR-450b-3p	-6.71207816		0.25810492	0	0	0	0	0.50454391	0.996854601
hsa-miR-6728-5p	-6.710501692		0.25817211	0	0	0	0	0.344518087	1.033554261
hsa-miR-6773-5p				0	0	0	0	0	0
hsa-miR-1587				0	0	0	0	0	0
hsa-miR-2110	6.046759143		0.32983659	0	0.454583725	0.836781969	0	0	0
hsa-miR-6857-5p				0	0	0	0	0	0
hsa-miR-6751-5p				0	0	0	0	0	0
hsa-miR-6819-5p	3.543845864		0.51085051	0	0.202011812	0.606035435	0	0	0
hsa-miR-6737-5p				0	0	0	0	0	0
hsa-miR-6855-5p	-6.712040629		0.25810652	0	0	0	0	0.497173959	0.974744747
hsa-miR-6839-3p				0	0	0	0	0	0
hsa-miR-7855-5p				0	0	0	0	0	0
hsa-miR-493-5p	-3.630675373	0.23206329	0.08280793	0	0.377668214	0.606035435	0.974744747	1.518495	2.583885652
hsa-miR-6818-3p	3.730128247	0.2130965	0.07403937	1.212070869	2.119087603	2.634846033	0	0.497173959	0.974744747
hsa-miR-4288				0	0	0	0	0	0
hsa-miR-3611	1.369911122	0.55956053	0.340181	5.269692065	8.711169784	13.33277956	4.873723736	6.349962036	7.974836806
hsa-miR-218-5p	-92.93505491	1.026E-149	1.498E-152	15.06207544	22.78876079	36.96816152	1676.560965	2140.688269	2610.241286
hsa-miR-4752				0	0	0	0	0	0
hsa-miR-4474-3p				0	0	0	0	0	0
hsa-miR-6796-5p	-3.835816219		0.44917283	0	0	0	0	0.172259043	0.51677713
hsa-miR-6759-5p	-3.835816219		0.44917283	0	0	0	0	0.172259043	0.51677713
hsa-miR-592	-6.710501692		0.25817211	0	0	0	0	0.344518087	1.033554261
hsa-miR-6818-5p	3.544185779		0.5108146	0	0.278927323	0.836781969	0	0	0
hsa-miR-7156-5p				0	0	0	0	0	0
hsa-miR-4677-5p	1.193638712	0.768441	0.57049181	5.454318912	9.26710231	13.38851151	5.167771304	7.984413248	11.96225521
hsa-miR-302c-5p				0	0	0	0	0	0
hsa-miR-3658				0	0	0	0	0	0
hsa-miR-4503	-1.155579957		0.8749822	0	0.377668214	0.606035435	0	0.344518087	1.033554261
hsa-miR-1303	1.638464312	0.07289177	0.01493786	19.24598529	25.36897388	28.98330636	13.43620539	16.64061608	18.94023741
hsa-miR-4760-5p				0	0	0	0	0	0
hsa-miR-651-5p	1.163616351	0.55089489	0.32908721	93.71958054	102.4256943	119.6220099	69.20687704	86.95856148	105.9393117
hsa-miR-122b-5p				0	0	0	0	0	0
hsa-miR-498-5p				0	0	0	0	0	0
hsa-miR-3942-3p				0	0	0	0	0	0
hsa-miR-587				0	0	0	0	0	0

hsa-miR-4755-5p	-1.263813377		0.81492219	0	0.351312804	1.053938413	0	0.649829831	1.949489494
hsa-miR-5006-3p	3.544185779		0.5108146	0	0.278927323	0.836781969	0	0	0
hsa-miR-1180-3p	1.107557976	0.77631463	0.59343326	59.99750804	87.40397754	114.3523178	58.91259287	79.64970687	105.2724327
hsa-miR-3685				0	0	0	0	0	0
hsa-miR-670-3p	-21.08336624		0.05846224	0	0	0	0	1.511125049	2.583885652
hsa-miR-3059-5p	-2.582244399	0.12328077	0.03199276	0.836781969	1.763244769	2.634846033	2.990563802	4.535881829	6.718102696
hsa-miR-7152-5p				0	0	0	0	0	0
hsa-miR-7159-3p				0	0	0	0	0	0
hsa-miR-4482-3p	-4.322415645	0.07909756	0.01698485	0	0.526969207	1.58090762	2.067108522	3.325205256	4.984273004
hsa-miR-6833-3p	1.22312497		0.82413785	0	0.526969207	1.58090762	0	0.50454391	0.996854601
hsa-miR-4743-3p				0	0	0	0	0	0
hsa-miR-4659a-3p	2.520582841	0.24485471	0.09175107	0.606035435	5.202432789	9.20460166	0.51677713	2.143858391	2.990563802
hsa-miR-4659b-3p				0	0	0	0	0	0
hsa-miR-1304-5p				0	0	0	0	0	0
hsa-miR-6516-5p	1.87153041	0.39076896	0.18939459	2.107876826	3.511344905	4.242248043	0.974744747	1.678520823	2.067108522
hsa-miR-4520-2-3p				0	0	0	0	0	0
hsa-miR-12131				0	0	0	0	0	0
hsa-miR-182-5p	-1.086278134	0.67661118	0.47503544	6076.71066	6682.029109	6998.151063	6480.551759	7257.219668	8013.662962
hsa-miR-96-5p	-1.141318449	0.48161522	0.26576723	1510.293746	1603.745078	1678.58463	1604.429854	1828.210826	1986.731219
hsa-miR-3913-5p	-1.106845333	0.91504057	0.80951035	2.424141739	2.874424031	3.688784446	2.924234241	3.337438476	3.987418403
hsa-miR-6513-5p				0	0	0	0	0	0
hsa-miR-133b	-1.167493477		0.8408778	0.526969207	0.656595537	0.836781969	0	0.669433003	1.033554261
hsa-miR-133a-3p	-1.94689325	0.00002126	1.831E-06	64.43221162	73.47267179	84.84496086	135.9123853	146.4539353	157.908649
hsa-miR-3149	-2.641188145		0.21237513	0	0.377668214	0.606035435	0	1.166606962	1.949489494
hsa-miR-758-3p				0	0	0	0	0	0
hsa-miR-375-3p	-1.561963221	0.09884543	0.02334406	19.39313391	30.34111733	38.99572128	40.8253933	48.07091829	56.53519533
hsa-miR-7856-5p				0	0	0	0	0	0
hsa-miR-1305	-21.55383027		0.06336426	0	0	0	0	1.978969299	3.987418403
hsa-miR-450b-5p	-1.02440294	0.93212443	0.84263552	283.6245834	306.0223249	339.8951382	294.3729136	312.8797996	331.2541406
hsa-miR-507				0	0	0	0	0	0
hsa-miR-3680-3p	10.22210643		0.21458537	0	0.702625609	2.107876826	0	0	0
hsa-miR-450a-5p	-1.00879868	0.9772691	0.96607306	15.8090762	20.14078186	27.87763	15.59591595	20.39612206	24.92136502
hsa-miR-5010-3p	-1.360307279	0.56108715	0.34238603	3.030177174	4.440187018	5.269692065	4.650994174	6.483014737	7.974836806
hsa-miR-335-3p	-2.700116664	2.705E-13	1.303E-14	246.0503865	272.5027828	296.1566941	701.8162179	738.2587816	790.5056984
hsa-miR-9983-3p	-18.21087286		0.06992402	0	0	0	0.974744747	1.346235957	2.067108522
