# Supplementary material for: A Stem-like Patient-Derived Ovarian Cancer Model of Platinum Resistance Reveals Dissociation of Stemness and Resistance
Source: Int J Mol Sci. 2024 Mar 29;25(7):3843. doi: 10.3390/ijms25073843 (PMC11011340; doi:10.3390/ijms25073843)
Supplement: Supplementary file 1 [file ijms-25-03843-s001.zip › Table S4.pdf]

Table S4. Top hits for hallmark pathways in gene set enrichment analysis for PDX4 SE.

NAME	SIZE	ES	NES	NOM p-val	FDR q-val	FWER p-val	RANK AT MAX	LEADING EDGE
HALLMARK_UV_RESPONSE_DN	116	-0.5773815	-1.9607254	0	0	0	1217	tags=26%, list=9%, signal=28%
HALLMARK_EPITHELIAL_MESENCHYMAL_TRANSITION	124	-0.524127	-1.7717289	0	0.00586276	0.017	863	tags=29%, list=7%, signal=31%
HALLMARK_ESTROGEN_RESPONSE_EARLY	145	-0.4339967	-1.5064288	0.0074813	0.11621336	0.403	2172	tags=30%, list=16%, signal=35%
HALLMARK_KRAS_SIGNALING_DN	78	-0.4742461	-1.4985373	0.01628223	0.09456088	0.429	1593	tags=29%, list=12%, signal=33%
HALLMARK_MYOGENESIS	120	-0.4346434	-1.4716043	0.01159794	0.10072976	0.524	1570	tags=22%, list=12%, signal=24%
HALLMARK_IL2_STAT5_SIGNALING	139	-0.4232688	-1.4616113	0.01130653	0.09156377	0.553	1140	tags=22%, list=9%, signal=24%
HALLMARK_IL6_JAK_STAT3_SIGNALING	47	-0.4982558	-1.4508231	0.03188406	0.08779717	0.59	1027	tags=28%, list=8%, signal=30%
HALLMARK_ANGIOGENESIS	22	-0.5228466	-1.3428416	0.13064516	0.20146058	0.915	659	tags=36%, list=5%, signal=38%
HALLMARK_COAGULATION	69	-0.4271852	-1.3334436	0.09190672	0.19305976	0.931	2204	tags=32%, list=17%, signal=38%
HALLMARK_APICAL_JUNCTION	136	-0.3593872	-1.232883	0.1238825	0.35841218	0.997	1722	tags=20%, list=13%, signal=23%
HALLMARK_INFLAMMATORY_RESPONSE	92	-0.364889	-1.1947147	0.19015957	0.4154494	0.999	709	tags=17%, list=5%, signal=18%
HALLMARK_NOTCH_SIGNALING	28	-0.4413919	-1.1797172	0.26971608	0.41892126	1	648	tags=18%, list=5%, signal=19%
HALLMARK_BILE_ACID_METABOLISM	69	-0.3717468	-1.1601362	0.23119777	0.43029636	1	2600	tags=35%, list=20%, signal=43%
HALLMARK_MITOTIC_SPINDLE	191	-0.3102576	-1.0924199	0.2967819	0.57727784	1	3987	tags=35%, list=30%, signal=50%
HALLMARK_HEDGEHOG_SIGNALING	25	-0.3956718	-1.0315069	0.43037975	0.7076481	1	1537	tags=20%, list=12%, signal=23%
HALLMARK_WNT_BETA_CATENIN_SIGNALING	35	-0.3647906	-1.0186746	0.4427481	0.7006348	1	612	tags=11%, list=5%, signal=12%
HALLMARK_UV_RESPONSE_UP	128	-0.2974617	-1.0095041	0.45271122	0.6834069	1	1098	tags=13%, list=8%, signal=14%
HALLMARK_HYPOXIA	151	-0.2891088	-0.9959388	0.45742092	0.6795045	1	1299	tags=15%, list=10%, signal=17%
HALLMARK_XENOBIOTIC_METABOLISM	127	-0.2888062	-0.9849293	0.50128865	0.67036897	1	2367	tags=24%, list=18%, signal=29%
HALLMARK_ESTROGEN_RESPONSE_LATE	131	-0.2817175	-0.9679362	0.5070243	0.6787682	1	1038	tags=15%, list=8%, signal=16%
HALLMARK_COMPLEMENT	118	-0.2863833	-0.9591986	0.5474359	0.6657625	1	2329	tags=24%, list=18%, signal=29%
HALLMARK_HEME_METABOLISM	154	-0.266092	-0.918512	0.6223067	0.7301204	1	2443	tags=22%, list=19%, signal=27%
HALLMARK_ADIPOGENESIS	169	-0.2031782	-0.7101315	0.97146404	1	1	2362	tags=16%, list=18%, signal=19%
HALLMARK_G2M_CHECKPOINT	197	-0.1978788	-0.7030231	0.98062956	1	1	3867	tags=26%, list=29%, signal=36%
HALLMARK_APICAL_SURFACE	25	-0.2733564	-0.698557	0.8569132	0.98734784	1	1415	tags=24%, list=11%, signal=27%
HALLMARK_E2F_TARGETS	198	-0.1837773	-0.6478955	0.9976134	0.97766256	1	3446	tags=22%, list=26%, signal=30%
