# Supplementary material for: A Stem-like Patient-Derived Ovarian Cancer Model of Platinum Resistance Reveals Dissociation of Stemness and Resistance
Source: Int J Mol Sci. 2024 Mar 29;25(7):3843. doi: 10.3390/ijms25073843 (PMC11011340; doi:10.3390/ijms25073843)
Supplement: Supplementary file 1 [file ijms-25-03843-s001.zip › Table S5.pdf]

Table S5. Top hits for hallmark pathways in gene set enrichment analysis for PDX4 CR.

NAME	SIZE	ES	NES	NOM p-val	FDR q-val	FWER p-val	RANK AT MAX	LEADING EDGE
HALLMARK_INTERFERON_ALPHA_RESPONSE	72	0.63786113	2.306093	0	0	0	1090	tags=43%, list=8%, signal=47%
HALLMARK_MYC_TARGETS_V1	200	0.5149191	2.1980624	0	0	0	4457	tags=60%, list=34%, signal=89%
HALLMARK_INTERFERON_GAMMA_RESPONSE	132	0.5243855	2.0870988	0	0	0	1556	tags=39%, list=12%, signal=44%
HALLMARK_MYC_TARGETS_V2	58	0.537346	1.8659847	0	0.00289367	0.006	3958	tags=62%, list=30%, signal=88%
HALLMARK_APOPTOSIS	126	0.39773452	1.5908104	0	0.03245121	0.081	1480	tags=25%, list=11%, signal=27%
HALLMARK_OXIDATIVE_PHOSPHORYLATION	196	0.34320012	1.4486942	0	0.08995173	0.245	3593	tags=39%, list=27%, signal=53%
HALLMARK_KRAS_SIGNALING_UP	94	0.37426615	1.3925596	0.04291846	0.11573479	0.347	877	tags=18%, list=7%, signal=19%
HALLMARK_GLYCOLYSIS	162	0.31592062	1.3067553	0.04188482	0.19214387	0.562	2603	tags=33%, list=20%, signal=40%
HALLMARK_CHOLESTEROL_HOMEOSTASIS	68	0.35949185	1.2871991	0.08185054	0.19339053	0.604	1642	tags=24%, list=12%, signal=27%
HALLMARK_TNFA_SIGNALING_VIA_NFKB	145	0.3169191	1.2808524	0.05913978	0.18159954	0.617	2423	tags=35%, list=18%, signal=43%
HALLMARK_SPERMATOGENESIS	70	0.34449476	1.2599834	0.09747292	0.19064651	0.665	1500	tags=20%, list=11%, signal=22%
HALLMARK_REACTIVE_OXYGEN_SPECIES_PATHWAY	46	0.36971268	1.2343553	0.169279	0.21463448	0.732	2517	tags=35%, list=19%, signal=43%
HALLMARK_PEROXISOME	83	0.32535616	1.1965047	0.16666667	0.259604	0.823	867	tags=13%, list=7%, signal=14%
HALLMARK_P53_PATHWAY	168	0.2819077	1.1662177	0.11827957	0.28872156	0.88	3061	tags=31%, list=23%, signal=40%
HALLMARK_ALLOGRAFT_REJECTION	92	0.30337194	1.1165661	0.21722846	0.363785	0.946	1710	tags=25%, list=13%, signal=29%
HALLMARK_DNA_REPAIR	144	0.27842268	1.1086253	0.2393617	0.35714132	0.951	2180	tags=20%, list=17%, signal=24%
HALLMARK_ANDROGEN_RESPONSE	85	0.30064085	1.1042707	0.2644628	0.34763595	0.959	717	tags=13%, list=5%, signal=14%
HALLMARK_MTORC1_SIGNALING	190	0.2654577	1.1040522	0.18817204	0.3285189	0.959	2320	tags=24%, list=18%, signal=29%
HALLMARK_PI3K_AKT_MTOR_SIGNALING	84	0.29097688	1.0722754	0.31707317	0.37383735	0.977	1480	tags=13%, list=11%, signal=15%
HALLMARK_TGF_BETA_SIGNALING	49	0.3232918	1.0619392	0.3277592	0.37499785	0.983	747	tags=10%, list=6%, signal=11%
HALLMARK_PROTEIN_SECRETION	90	0.28311387	1.0564181	0.33204633	0.36889958	0.984	3828	tags=38%, list=29%, signal=53%
HALLMARK_FATTY_ACID_METABOLISM	121	0.21440676	0.8489165	0.85096157	0.82475466	1	3666	tags=36%, list=28%, signal=49%
HALLMARK_UNFOLDED_PROTEIN_RESPONSE	108	0.20879067	0.8070447	0.8836207	0.86422765	1	1976	tags=20%, list=15%, signal=24%
