# Supplementary material for: A Stem-like Patient-Derived Ovarian Cancer Model of Platinum Resistance Reveals Dissociation of Stemness and Resistance
Source: Int J Mol Sci. 2024 Mar 29;25(7):3843. doi: 10.3390/ijms25073843 (PMC11011340; doi:10.3390/ijms25073843)
Supplement: Supplementary file 1 [file ijms-25-03843-s001.zip › Table S6.pdf]

Table S6. Ingenuity Pathway Analysis of PDX4 RNA-sequencing data for canonical pathways associated with autophagy. The ratio column refers to the ratio of differentially expressed genes within the RNA-seq data to the total number of molecules within the canonical pathway. The z-score refers to the predicted activation (greater than or equal to 2) or inhibition (less than or equal to 2) of a specific pathway. The molecules column lists the differentially expressed genes within the pathway

Ingenuity Canonical Pathways	p-value	Ratio	z-score	Molecules
**Microautophagy Signaling Pathway**	2.00E-14	4.75E-01	3.641	BAG6, CERT1, CHMP4B, COL11A1, COL12A1, COL13A1, COL16A1, COL17A1, COL18A1, COL1A1, COL23A1, COL26A1, COL27A1, COL2A1, COL3A1, COL4A2, COL4A5, COL5A1, COL5A2, COL6A1, COL6A2, COL6A3, COL9A3, HGS, HSPA8, LAMP2, MAP1LC3B, MAP1LC3B2, PRKAB2, PRKAG1, PRKAG2, PSMA4, PSMA5, PSMA7, PSMB1, PSMB2, PSMB3, PSMB4, PSMB5, PSMB6, PSMB7, PSMC1, PSMC2, PSMC3, PSMC4, PSMC5, PSMC6, PSMD1, PSMD11, PSMD13, PSMD14, PSMD3, PSMD4, PSMD5, PSMD6, PSMD7, PSMD8, PSME2, PSMF1, SEC23A, SEC23B, SEC24B, SEC24C, SEC24D, SEC31A, SIDT2, SNF8, SPATA18, SQSTM1, STX17, TP53, VAMP8, VPS18, VPS28, VPS36, VPS39
**Autophagy**	4.47E-06	3.36E-01	0.956	AKT1S1, AMBRA1, ATF4, ATG2A, ATG3, ATG9B, ATM, BIRC6, BNIP3, CALM1 (includes others), CAMKK2, CREB3L2, DDIT3, E2F1, EPG5, FOS, FOXO1, GCG, GSK3A, GSK3B, HGF, HIF1A, INSR, IRS1, LAMP1, LAMP2, MAP1LC3B, MAP1LC3B2, MAPK10, MAPK3, MLST8, MYC, NFKB1, NFKB2, PDGFB, PIK3C2A, PIK3C2G, PIK3CA, PIK3CB, PIK3CD, PIK3R1, PIK3R5, PPP2R2C, PPP2R3B, PPP2R5C, PPP3CA, PPP3CB, PRKAB2, PRKAG1, PRKAG2, PRKAR1A, PRKAR1B, PRKAR2B, PTEN, PTPA, RAF1, RALB, RIPK2, SIRT1, SLC3A2, SLC7A5, SQSTM1, STK11, STX17, TBK1, TGFB1, TGFB2, TICAM1, TP53, VAMP8, VEGFA, VPS18, VPS39
**Chaperone Mediated Autophagy**	4.57E-04	5.45E-01	2.309	ARL13B, HDAC6, HSP90AA1, HSP90AB1, HSPA8, LAMP2, PARK7, RPS27A, UBA52, UBB, UBC, VIM
**Late endosomal microautophagy**	7.24E-02	3.24E-01	2.111	ARL13B, CHMP4B, HDAC6, HSPA8, PARK7, RPS27A, UBA52, UBB, UBC, VIM, VPS28
**Macroautophagy**	3.54E-01	2.31E-01	1.291	AMBRA1, ATG3, ATG9B, CHMP4B, DYNLL1, LAMTOR1, LAMTOR2, LAMTOR5, MAP1LC3B, MLST8, PRKAB2, PRKAG1, PRKAG2, RHEB, RRAGC
**Chaperone Mediated Autophagy Signaling Pathway**	1.00E+00	1.45E-01	0.417	APP, ATP6AP1, ATP6V0A1, ATP6V0A2, ATP6V0B, ATP6V0E2, ATP6V1A, ATP6V1C1, ATP6V1F, ATP6V1G1, BAG1, CCND1, CD247, CHEK1, CIP2A, CTSA, EEF1A2, EIF4A1, EIF4H, EPS8, HDAC6, HIF1A, HK2, HMGB1, HSP90AA1, HSP90AB1, HSP90B1, HSPA4, HSPA5, HSPA8, HSPA9, HSPBP1, HTT, IDH2, IGF1R, IL17RB, KAT2B, LAMP2, LRRK2, MAPK11, MCL1, MEF2D, MLST8, MMP10, MMP15, MMP16, MMP25, MMP3, MYC, NCOR2, NFATC2, NFKB1, NFKB2, NFKBIA, NOS3, PARK7, PEA15, PIK3C2A, PIK3C2G, PIK3CA, PIK3CB, PIK3CD, PIK3R1, PIK3R5, PPP3CA, PPP3CB, PRKAB2, PRKAG1, PRKAG2, PRR5, PSMA4, PSMA5, PSMA7, PSMB1, PSMB2, PSMB3, PSMB4, PSMB5, PSMB6, PSMB7, PSME2, RAB11A, RELA, RND3, SNCA, STIP1, STUB1, TBK1, TCIRG1, TP53, TPD52, UCHL1
