# Supplementary material for: A Stem-like Patient-Derived Ovarian Cancer Model of Platinum Resistance Reveals Dissociation of Stemness and Resistance
Source: Int J Mol Sci. 2024 Mar 29;25(7):3843. doi: 10.3390/ijms25073843 (PMC11011340; doi:10.3390/ijms25073843)
Supplement: Supplementary file 1 [file ijms-25-03843-s001.zip › Table S7.pdf]

Table S7. Primer sequences for RT-qPCR. Amplicon length displayed under gene name.

Gene	Forward Primer Sequence (5' – 3')	Reverse Primer Sequence (5' – 3')
ABCC2 (131 bp)	CCC TGC TGT TCG ATA TAC CAA TC	TCG AGA GAA TCC AGA ATA GGG AC
ACTB (151 bp)	TGA AGT GTG ACG TGG ACA TC	GGA GGA GCA ATG ATC TTG AT
ATP7A (73 bp)	AGC AAT TGC TTC ATC TG	GCA GGC AGT TCA TAA CTC TCG
ATP7B (145 bp)	TCT CTG GTC ATC CTG GTG GTT	GGG CTT CTG AGG TTT TGC TCT
CD44 (213 bp)	TGG CAC CCG CTA TGT CCA	GTA GCA GGG ATT CTG TCT G
CDH1 (291 bp)	GAG AAA CAG GAT GGC TGA AGG	TGA GGA TGG TGT AAG CGA TGG
CDH2 (122 bp)	GAG GAG TCA GTG AAG GAG TCA	GGG AAG TTG ATT GGA GGG ATG
LIN28A (128 bp)	GAG CAT GCA GAA GCG CAG ATC A	TAT GGC TGA TGC TCT GGC AGA A
LIN28B (128 bp)	GCC CCT TGG ATA TTC CAG TC	TGA CTC AAG GCC TTT GGA AG
NANOG (158 bp)	CAA AGG CAA ACA ACC CAC TT	TCT GCT GGA GGC TGA GGT AT
POU5F1 (163 bp)	AAG CGA TCA AGC AGC GAC TAT	GGA AAG GGA CCG AGG AGT ACA
SLC31A1 (167 bp)	GGG GAT GAG CTA TAT GGA CTC C	TCA CCA AAC CGG AAA ACA GTA G
SNAI1 (113 bp)	CAC TAT GCC GCG CTC TTT C	GGT CGT AGG GCT GCT GGA A
SNAI2 (119 bp)	AAG CAT TTC AAC GCC TCC AAA	AGG ATC TCT GGT TGT GGT ATG AC
SOX2 (140 bp)	GCG CCC TGC AGT ACA ACT C	GCT GGC CTC GGA CTT GAC
TWIST1 (201 bp)	GGA GTC CGC AGT CTT ACG AG	TCT GGA GGA CCT GGT AGA GG
VIM (183 bp)	GGT GGA CCA GCT AAC CAA CGA	TCA AGG TCA AGA CGT GCC AGA
ZEB1 (141 bp)	GCA CAA GAA GAG CCA CAA GTA	GCA AGA CAA GTT CAA GGG TTC
ZEB2 (160 bp)	TTC CTG GGC TAC GAC CAT AC	TGT GCT CCA TCA AGC AAT TC
